# Supplementary material for: Transcription start site analysis reveals widespread divergent transcription in D. melanogaster and core promoter-encoded enhancer activities
Source: Nucleic Acids Res. 2018 Apr 6;46(11):5455–69. doi: 10.1093/nar/gky244 (PMC6009668; doi:10.1093/nar/gky244)
Supplement: Supplementary Data [file gky244_supplemental_files.zip › Rennie_et_al_2018_supplement.pdf]

Supplementary Materials and Methods to:

Transcription start site analysis reveals widespread divergent transcription in D. melanogaster and core promoter-encoded enhancer activities

Sarah Rennie [1,*], Maria Dalby [1,*], Marta Lloret-Llinares [2], Stylianos Bakoulis [1], Christian Dalager Vaagensø [1], Torben Heick Jensen [2] and Robin Andersson [1,†]

[1]The Bioinformatics Centre, Department of Biology, University of Copenhagen, Ole Maaloes Vej 5, 2200 Copenhagen N, Denmark and [2]Department of Molecular Biology and Genetics, Aarhus University, C.F. Møllers Allé 3, Building 1130, 8000 Aarhus C, Denmark

SUPPLEMENTARY FILES

1. **Expression and STARR-seq data associated with transcribed DHSs.** For each transcribed DHS, the supplementary file holds the DHS ID (hotspot coordinate), the expression (TPM) per replicate in control and exosome knockdown CAGE libraries, the exosome sensitivity on minus and plus strands, the transcriptional directionality based on control and knockdown CAGE data, the hkCP and dCP STARR-seq signal, and the DHS class.

2. **Core promoter elements of transcribed DHSs.** For each transcribed DHS and strand, the supplementary file holds the DHS ID (hotspot coordinate) and strand, as well as the maximum motif match score and associated p-value for each considered core promoter element (DPE, DRE, E-box, INR, Ohler1, Ohler6, MTE, TATA, Trl).

3. **ChIP-seq signal for transcribed DHSs.** For each transcribed DHS, the supplementary file holds the DHS ID (hotspot coordinate) and aggregate ChIP-seq signals for H3K27ac, H3K4me1, and H3K4me3.

4. **Normalised ChIP-seq signal for transcribed DHSs.** For each transcribed DHS, the supplementary file holds the DHS ID (hotspot coordinate) and the counts-per-million (CPM) normalised aggregate ChIP-seq signals for H3K27ac, H3K4me1, and H3K4me3.

SUPPLEMENTARY METHODS

Estimation of CAGE genomic background noise

For robust assessment of lowly expressed loci, CAGE genomic background noise levels were estimated. First, we calculated the CAGE mappability of the dm3 reference genome, by mapping each 25-sized subsequence of the reference genome back to itself, using the same mapping approach as for real CAGE data. Then, we quantified the number of CAGE 5' ends from each out of the four control CAGE libraries mapping to genomic regions of size 200 bp, that were uniquely mappable (as determined by the mappability track) in at least 50% of its potential TSS positions (unique bps). We discarded regions that were proximal (within 500bp) of FlyBase gene TSSs, FlyBase transcript ends, or midpoints of DHS hotspot regions (as defined above), or proximal (within 100bp) of FlyBase gene exons. Based on the empirical distribution of CAGE expression noise from annotation-distal genomic regions, we extracted the 99th percentile and used the max value across control libraries as a threshold to call regions significantly expressed in subsequent analyses.

CAGE tag clustering

First, individual genomic bps supported by at least two CAGE 5' ends of CAGE reads in a single library that were located within 20 bp from each other on the same strand were merged into tag clusters (TCs). For each TC, the summit position (representing the within-TC bp with the highest frequency of CAGE 5' ends) or the median of multiple summit positions was identified. Second, for each TC, the fraction between the CAGE tag frequency of each TC-covered bp to that of the summit position was calculated.

*These authors contributed equally to this work.
†To whom correspondence should be addressed. Email: robin@binf.ku.dk

All positions within a TC associated with less than 1/10 of the summit signal was discarded. This strategy causes multi-peak TCs to be split into new TCs representing each sub peak. Also, it removes tails from wide TCs with a single peak. Subsequently, TCs split on summit fraction on the same strand were merged if positioned within 20bp from each other. This resulted in a set of 670,681 TCs.

DHSs as focus points for transcription initiation

DHSs were used as focus points for characterising patterns of transcription initiation events at TREs as described elsewhere (1), with minor modifications. Instead of focusing on DHS positions that maximised DNase-seq signal (DHS summits), we defined centre points of DHSs as positions optimising the coverage of proximal CAGE tags within 200 bp immediately flanking windows associated with minus and plus strand expression as illustrated in Supplementary Fig. 5. We required DHSs to be supported by significant expression above inferred noise threshold (see above) on at least one strand and in at least two out of four replicates in either control of exosome knockdown CAGE data. This resulted in 9,471 transcribed DHSs out of the original set of 11,947 DHSs. For these, exosome sensitivity and transcriptional directionality measures were calculated as defined previously (1). For each DHS, we defined the major and minor strand as the strand with the highest and lowest average expression level in exosome knockdown CAGE libraries, respectively.

Annotation-unbiased clustering of DNase I hypersensitive sites

Unsupervised clustering was performed on the basis of five variables: the major and minor strand expression levels of the knockdown, the major and minor strand exosome sensitivity and the transcriptional directionality based on the knockdown samples. A total of 37,884 data vectors (4 replicates across 9,471 DHSs) were clustered. In order to determine the optimal number of clusters, we applied the kmeans++ algorithm and Mclust (2) at varying cluster sizes, with both methods concluding that either 5 or 6 clusters were optimal (based on a scree plot for cluster sizes ranging from 2 to 20 for kmeans++, and the default internal metrics from the Mclust package). We then applied the kmeans++ algorithm, which optimises the centre points of the initial centroids in the kmeans algorithm to achieve stable clusterings at 5 or 6 clusters. Correspondence analysis between pairs of replicates was used to check the overall performance of the clustering in grouping together replicates of the same DHS within a single cluster, suggesting the replicates in general to be of high quality agreement (Supplementary Fig. 6). In order to obtain a set of 'centralised' clusters for the 9,471 DHSs that agreed according to the clusters selected for the individual replicates, we then applied a further clustering step on the replicate cluster assignments. A distance matrix based on the hamming distance between each pair of DHSs was clustered using k-medoids, selecting for either 5 or 6 clusters. Based on biological interpretations of the resulting clusters according to their transcriptional properties, we manually choose the set based on 6 clusters, with the difference between the two clusterings surmounting to whether the *weak bidirectional unstable* and the *intermediate bidirectional unstable* DHSs should be treated as a single class or two separate classes.

Analysis of downstream RNA processing events

To examine the propensity of RNA-processing motifs downstream of TSSs and their association with DHS transcriptional properties, the occurrences of 5' splice sites (SS) and polyadenylation site (AWTAAA) termination motifs were assessed using HOMER (3) using position weight matrices collected from the JASPAR database (4) (ID: SD0001.1, NAME: at_AC_acceptor) and custom-made based on consensus, respectively. The enrichment per bp of motif hits over increasing distances up to 1kb downstream of the CAGE summit, considering the respective strand of each DHS, was calculated by comparison to the predicted motif occurrences from a genomic uniform distribution (enrichment over genomic background).

Transcript assembly from RNA-sequencing data

RNA-seq reads from exosome (Rrp6)-depleted S2 cells (5) were adapter and quality-trimmed using cutadapt (6) and sickle (https://github.com/najoshi/sickle), with standard options for single-end reads. Trimmed reads from exosome knockdown samples were pooled to increase the likelihood of detecting transcripts and assembled into transcripts using Cufflinks (v2.2.1) (7) with non-default parameters –min-frags-per-transfrag 5 and –overlap-radius 100 for the identification of low-abundant transcripts. 5'ends of transcripts were overlapped with windows -100 to +200 around CAGE summit positions associated with each strand window of transcribed DHSs.

Assessment of the relationship between chromatin architecture and type of regulatory element

All pairs of transcribed DHSs within 1 Mbp were annotated according to whether they were supported by a significant interaction or not. The resulting dataset was then distance balanced, by splitting the range of distances between the pairs into 100, and for each split the number of interacting pairs was balanced against the number of non-interacting pairs (1-1 ratio, taking 10 the minimum of interaction or non-interacting). Only pairs at above the 5th split were considered, to remove the bias from difficulties of interaction calling algorithms to detect interactions at short distances. Next, pairs of transcribed DHSs were split according to pairs which overlapped dCP enhancers on both ends, pairs which overlapped hkCP enhancers on both ends or pairs which overlapped a dcP enhancer in one and a hkCP enhancer on the other element. Cases where one side overlapped both were removed from the analysis. For each class and STARR-seq enhancer class at targets, we fit a GLM to predict which of the 5

potential classes the given class interacted with. For interpretation of coefficients we generated a background class according to a random subset based on 1/5th of the dataset. We repeated the distance balancing and random background sampling 100 times, and averaged the final coefficients and p-values.

Enrichment within and between TADs proceeded similar to the GLM approach with interactions, except the response was "within TAD vs between TAD" and there was no minimum distance cut off.

REFERENCES

1. Andersson, R., Refsing Andersen, P., Valen, E., Core, L. J., Bornholdt, J., Boyd, M., Heick Jensen, T., and Sandelin, A. (November, 2014) Nuclear stability and transcriptional directionality separate functionally distinct RNA species. *Nature Communications,* **5**, 5336.
2. Fraley, C. and Raftery, A. E. (2002) Model-Based Clustering, Discriminant Analysis, and Density Estimation. *Journal of the American Statistical Association,* **97**(458), 611–631.
3. Heinz, S., Benner, C., Spann, N., Bertolino, E., Lin, Y. C., Laslo, P., Cheng, J. X., Murre, C., Singh, H., and Glass, C. K. (May, 2010) Simple Combinations of Lineage-Determining Transcription Factors Prime cis-Regulatory Elements Required for Macrophage and B Cell Identities. *Molecular Cell,* **38**(4), 576–589.
4. Mathelier, A., Fornes, O., Arenillas, D. J., Chen, C.-Y., Denay, G., Lee, J., Shi, W., Shyr, C., Tan, G., Worsley-Hunt, R., Zhang, A. W., Parcy, F., Lenhard, B., Sandelin, A., and Wasserman, W. W. (January, 2016) JASPAR 2016: a major expansion and update of the open-access database of transcription factor binding profiles. *Nucleic acids research,* **44**(D1), D110–D115.
5. Lim, S. J., Boyle, P. J., Chinen, M., Dale, R. K., and Lei, E. P. March Genome-wide localization of exosome components to active promoters and chromatin insulators in Drosophila.. *Nucleic acids research,* **41**(5), 2963–2980.
6. Martin, M. (August, 2011) Cutadapt removes adapter sequences from high-throughput sequencing reads. *EMBnet.journal,* **17**(1), 10.
7. Trapnell, C., Roberts, A., Goff, L., Pertea, G., Kim, D., Kelley, D. R., Pimentel, H., Salzberg, S. L., Rinn, J. L., and Pachter, L. (March, 2012) Differential gene and transcript expression analysis of RNA-seq experiments with TopHat and Cufflinks. *Nature protocols,* **7**(3), 562–578.

SUPPLEMENTARY FIGURES

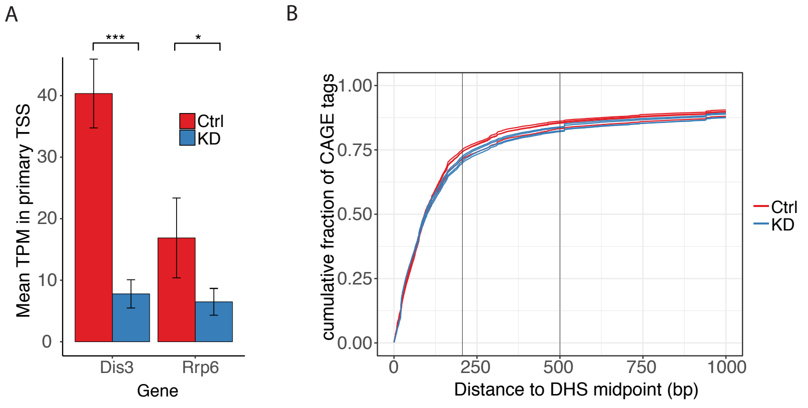

Supplementary Figure 1. Expression of knockdown target genes and assessment of cumulative CAGE tag fractions around DHSs. **(A)** Mean TPM expression of the exosome knockdown targets *Dis3* and *Rrp6* primary TSSs, measured for each control (Ctrl) and knockdown (KD) samples. Unpaired t-test determined significant reduction in knock down samples of both genes. Significance stars interpreted as: $* = P < 0.1$, $*** = P < 0.001$. **(B)** Cumulative CAGE tag fraction (vertical axis) in control samples (four replicates) and knockdown samples (four replicates), as a function of the distance to the midpoints (signal summits) of DNase I hypersensitive sites (DHSs).

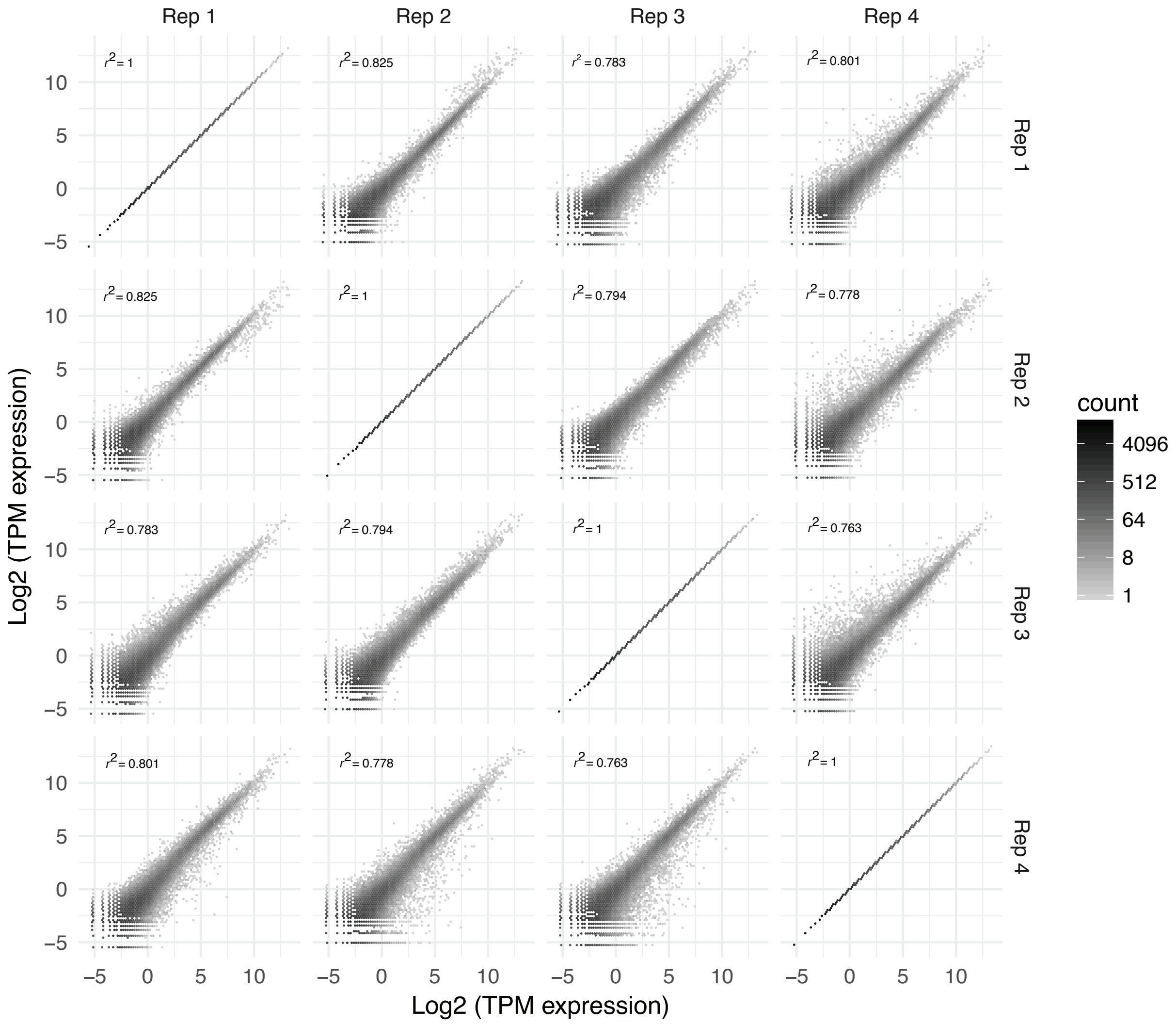

Supplementary Figure 2. Replicate expression in control samples. Log_2 TPM expression of TCs expressed above background noise for each pair of control replicates. Colour scale indicates number of TCs. Expression correlation between each replicate pair is indicated by r^2 in the top left corner, based on a linear model fit.

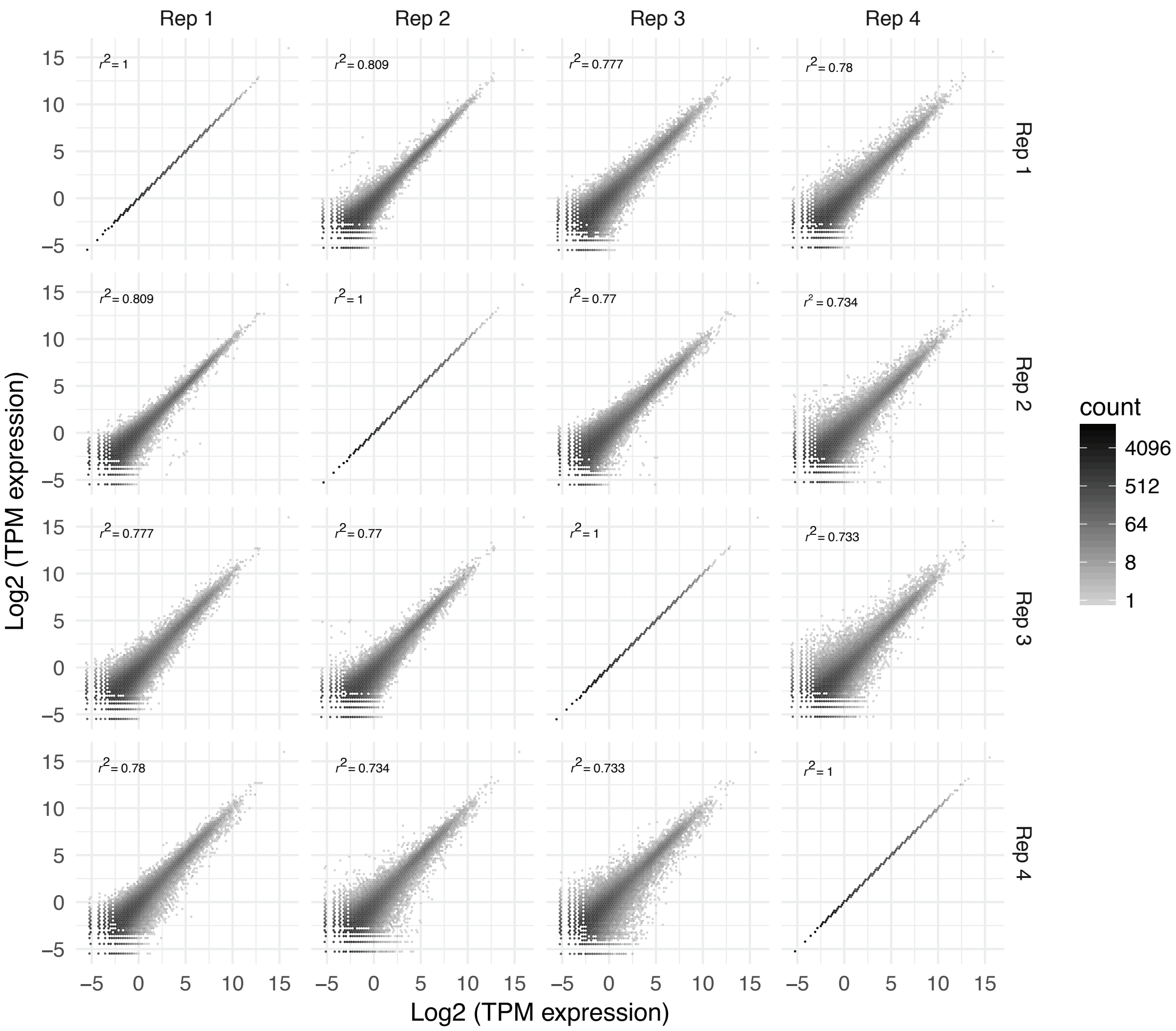

Supplementary Figure 3. Replicate expression in knockdown samples. Log_2 TPM expression of TCs expressed above background noise, for each pair of knockdown replicates. Colour scale indicates the number of TCs. Expression correlation between each replicate pair is indicated by r^2 in the top left corner, based on a linear model fit.

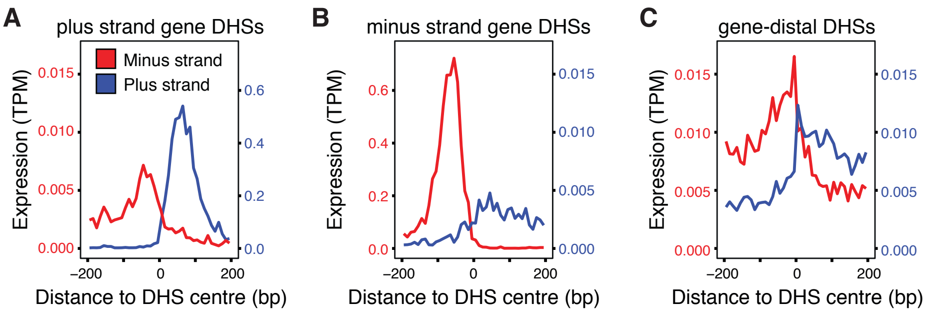

Supplementary Figure 4. CAGE CTSS coverage over DHSs. Exosome KD CAGE CTSS expression (TPM) overlaid on transcribed DHSs were averaged across DHSs proximal to FlyBase plus strand gene TSSs **(A)**, minus strand gene TSSs **(B)**, and distal to gene TSSs **(C)**. Outlier values above the 99th percentile of data were removed prior to averaging. Minus and plus strand expression levels are given on the left and right side of each panel, respectively.

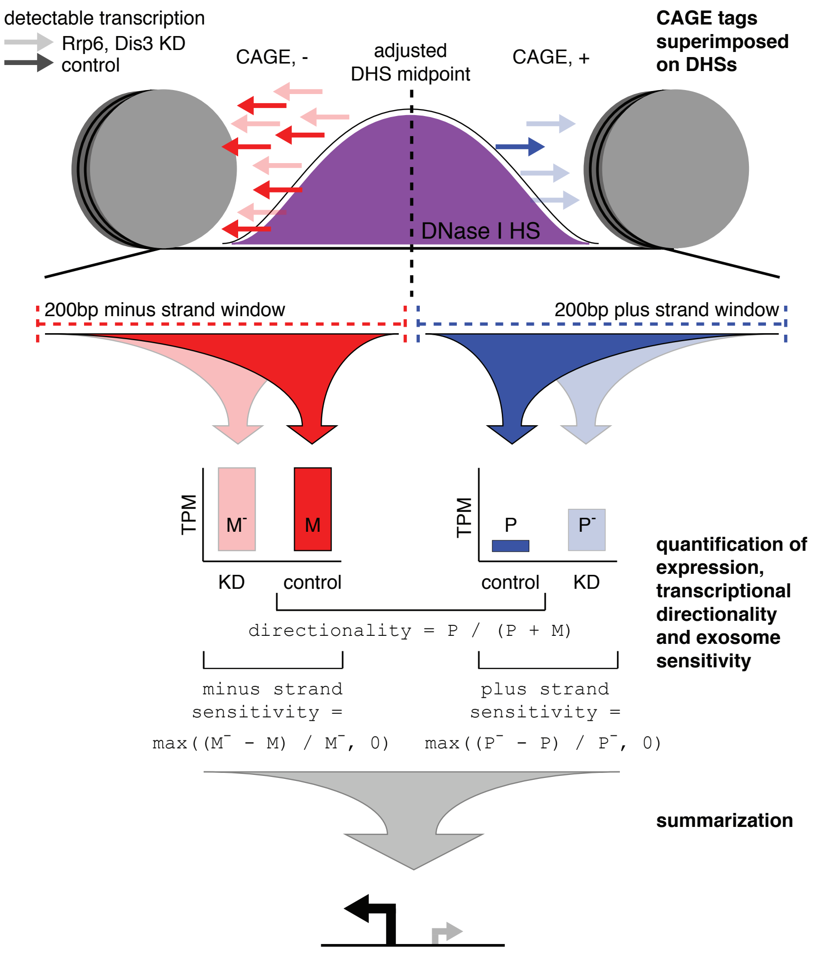

Supplementary Figure 5. Schematic illustration of the utilisation of DNase I hypersensitive sites as focus points for transcription initiation events at regulatory elements. DHS-associated strand-specific expression levels in control and exosome depleted S2 cells were quantified by counting of CAGE tags in genomic windows of 200 bp immediately flanking the DHS centre points that optimised CAGE tag coverage (see Methods). Based on strand-specific expression levels both a directionality score, measuring the strand bias in expression level, and a strand-specific exosome sensitivity score, measuring the relative amount of degraded RNAs by the exosome, were calculated. These measures were used for clustering the DHSs to infer classes of DHSs with similar transcriptional properties.

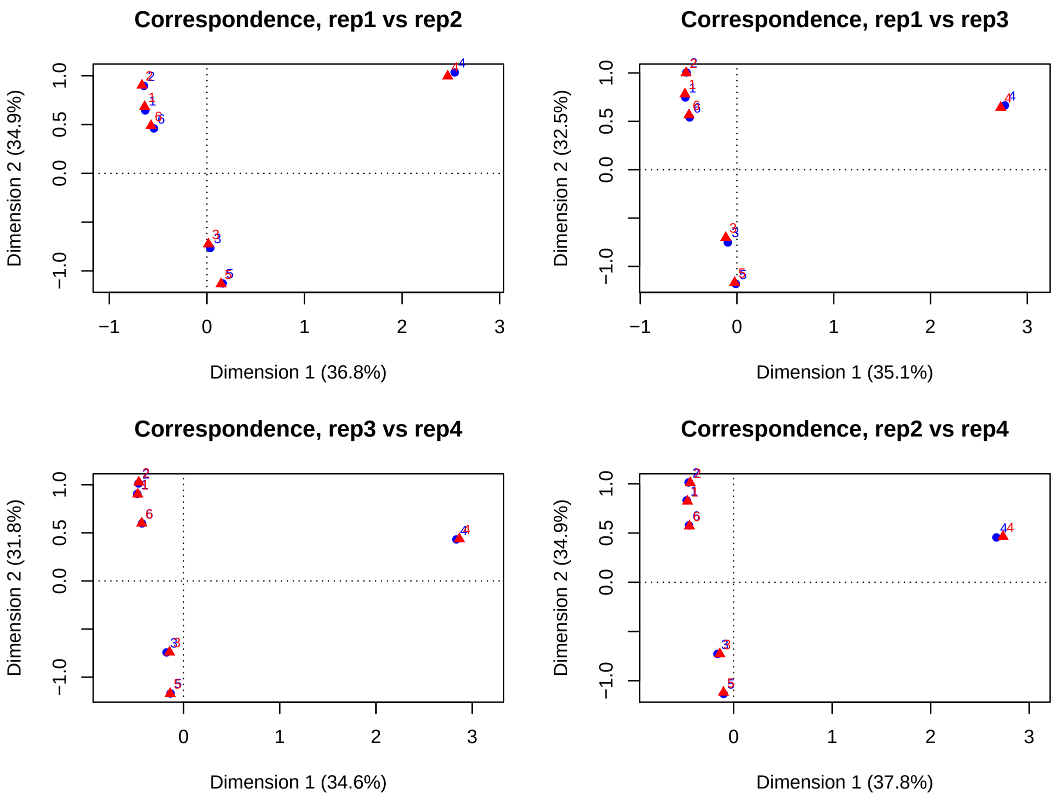

Supplementary Figure 6. Correspondence analysis of CAGE replicate agreement in DHS clustering.

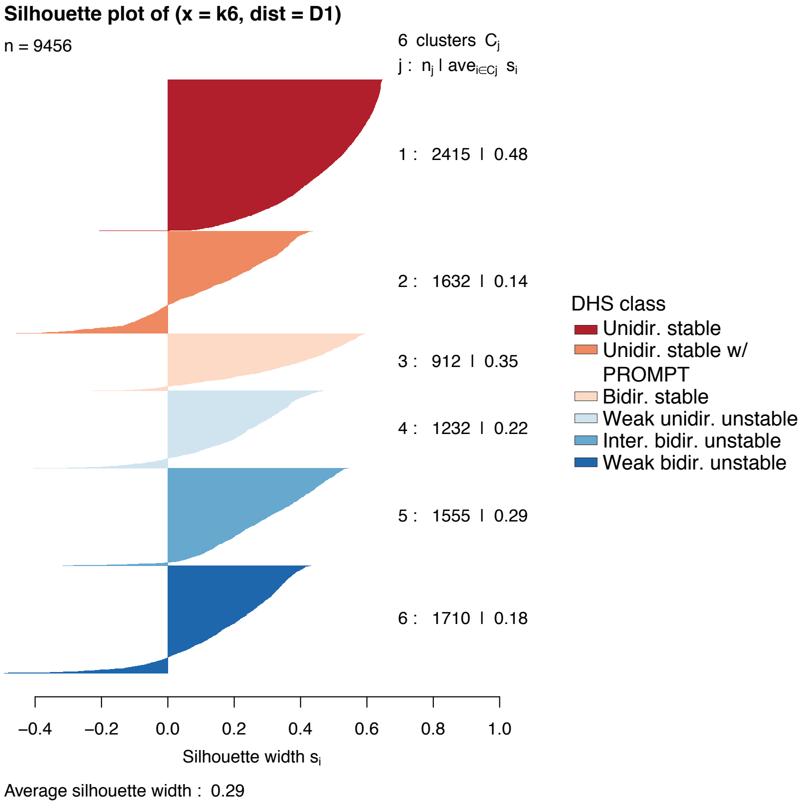

Supplementary Figure 7. Silhouette plot representing cluster purity. Clusters based on results from the two step clustering procedure of 9456 sites. Briefly, large silhouette widths indicate that a DHS fits well within its assigned cluster. The distance measure used is the euclidean distance between scaled data vectors averaged across the four replicates.

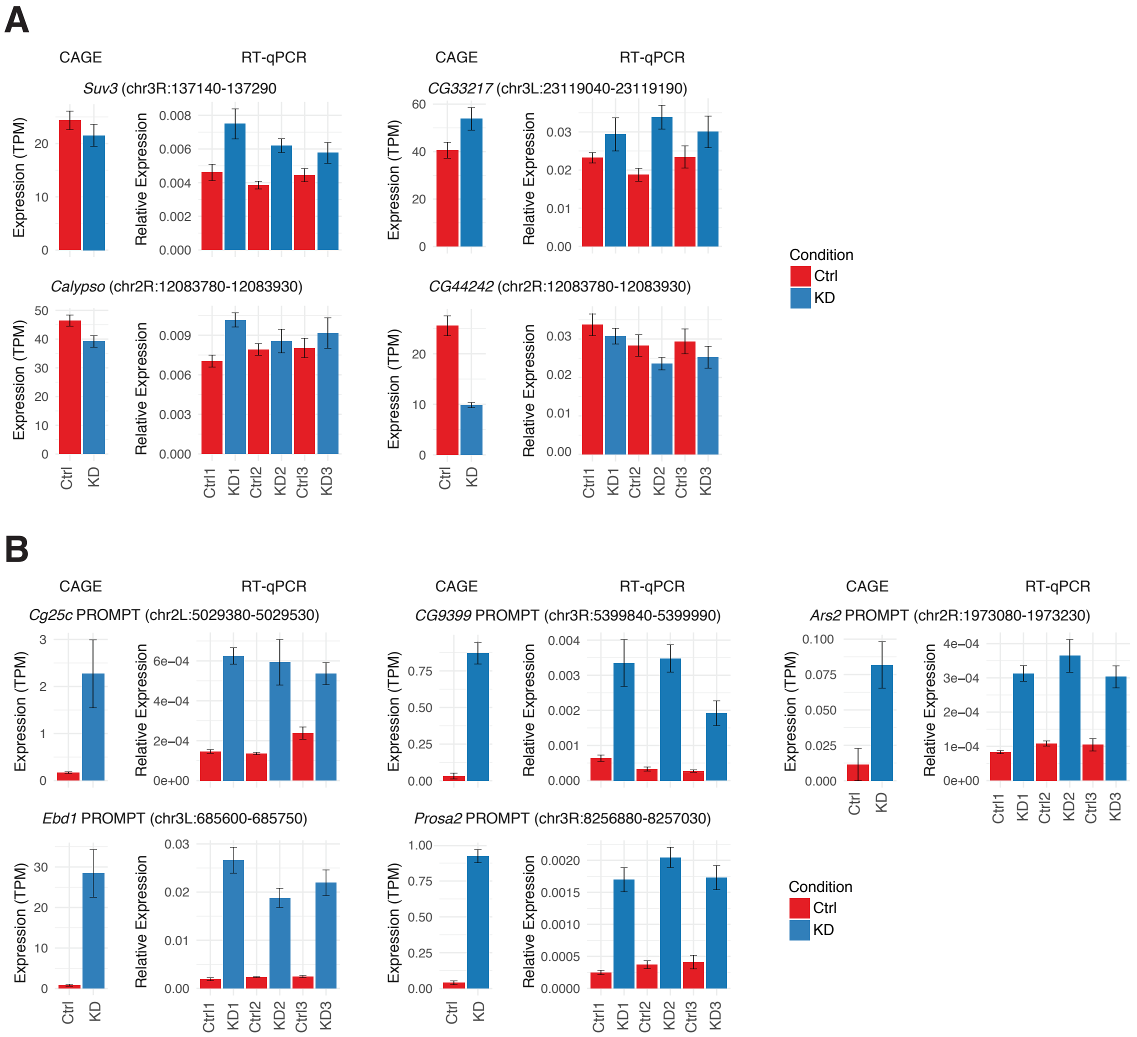

Supplementary Figure 8. CAGE expression and RT-qPCR validation of mRNA and PROMPT tagets. (**A**) CAGE expression and RT-qPCR validation of four randomly selected *unidirectional stable* targets, listed with FlyBase gene ID and DHS coordinates. For each target, left panel shows mean CAGE expression (TPM) and standard deviations of DHS major strand (gene sense direction), within all four replicates per CAGE condition; control (Ctrl) and knock down (KD). Right panel shows RT-qPCR mean relative expression with standard deviations of three technical replicates (normalised to *Act5C*), for three biological replicates per condition; control (Ctrl) and knock down (KD). (**B**) CAGE expression and RT-qPCR validation of four randomly selected PROMPTs of the *unidirectional stable w/ PROMPT* DHS cluster, together with the *Cg25C* PROMPT shown in Figure 1A. FlyBase gene ID and DHS coordinates are listed on top of each PROMPT plot section. For each target, left panel shows mean CAGE expression (TPM) and standard deviations of DHS minor strand (antisense of gene direction), within all four replicates per CAGE condition; control (Ctrl) and knock down (KD). Right panel shows RT-qPCR mean relative expression of PROMPT targets (upstream of and antisense to annotated FlyBase gene TSSs), with standard deviations of three technical replicates (normalised to *Act5C*), for three biological replicates per condition; control (Ctrl) and knock down (KD).

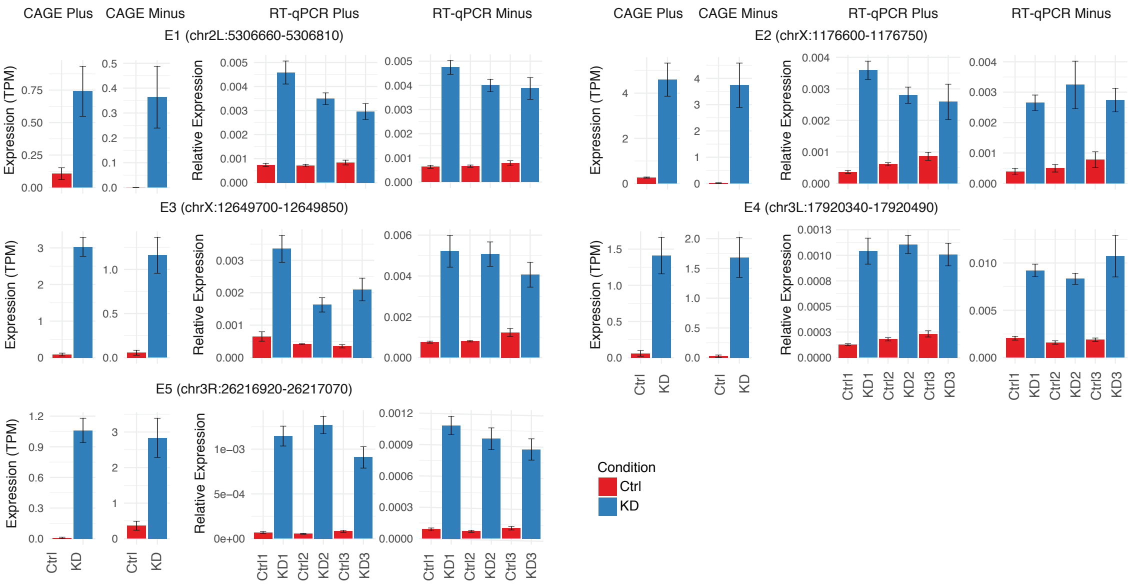

Supplementary Figure 9. CAGE expression and RT-qPCR validation of bidirectional unstable transcription. CAGE expression and RT-qPCR validation of four randomly selected enhancer RNA tagets from the *bidirectional weak unstable* DHS cluster, together with the intragenic dCP STARR-seq enhancer shown in Figure 1B. DHS coordinates are listed above each plot section. For each target, left panel shows DHS plus and minus strand mean CAGE expression (TPM) and standard deviations within all four replicates per CAGE condition; control (Ctrl) and knock down (KD). Right panel shows RT-qPCR mean relative expression with standard deviation of three technical replicates (normalised to *Act5C*) for three biological replicates per condition; control (Ctrl) and knock down (KD). RT-qPCR was performed on both plus and minus TC peak

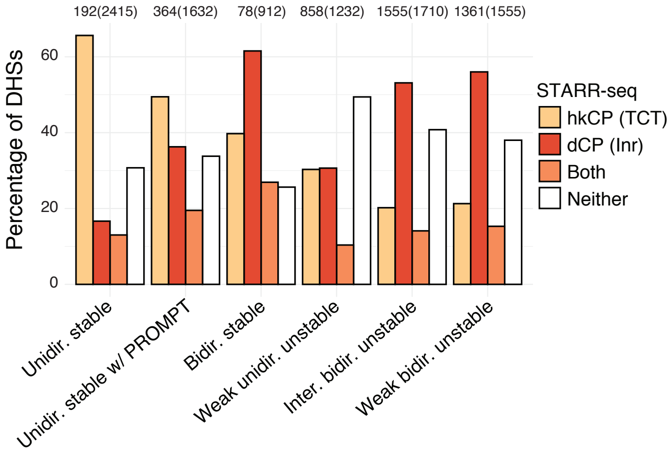

Supplementary Figure 10. The percentage of gene distal DHSs in each cluster that are overlapping with or are distal to called STARR-seq enhancers, broken up by those overlapping with hkCP enhancers, dCP enhancers or both classes. The number of DHSs distal to FlyBase gene TSSs in each DHS class (in parenthesis the total number of DHSs in the class) is given above each DHS class.

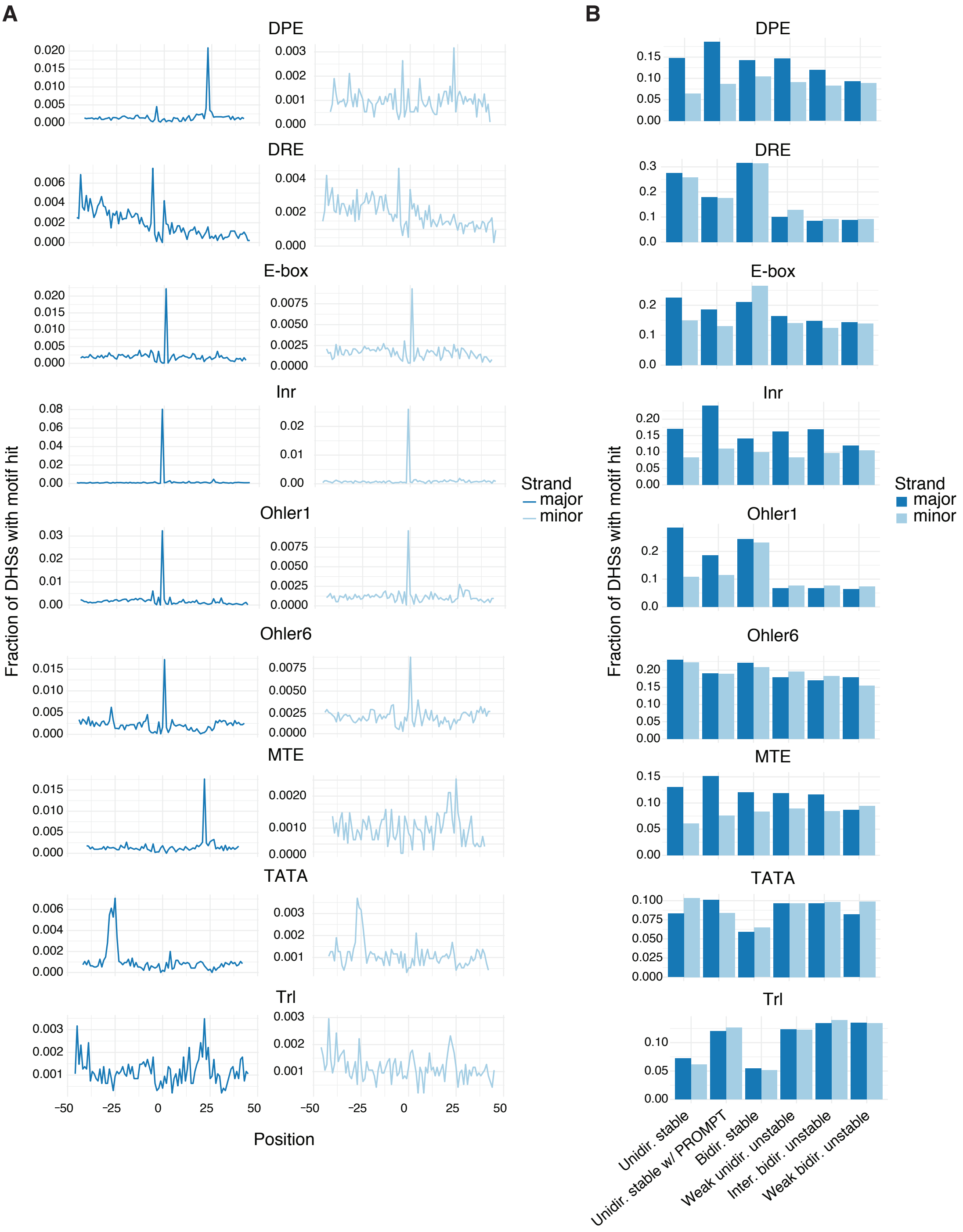

Supplementary Figure 11. Fraction of core promoter elements on minor and major strands of transcribed DHSs. **(A)** Fraction of transcribed DHSs (vertical axis) with an identified core promoter element at a given position relative to the major (left panels) and minor (right panels) strand CAGE summits. **(B)** Fraction of DHSs with a core promoter element in each DHS class broken up by major and minor strand.

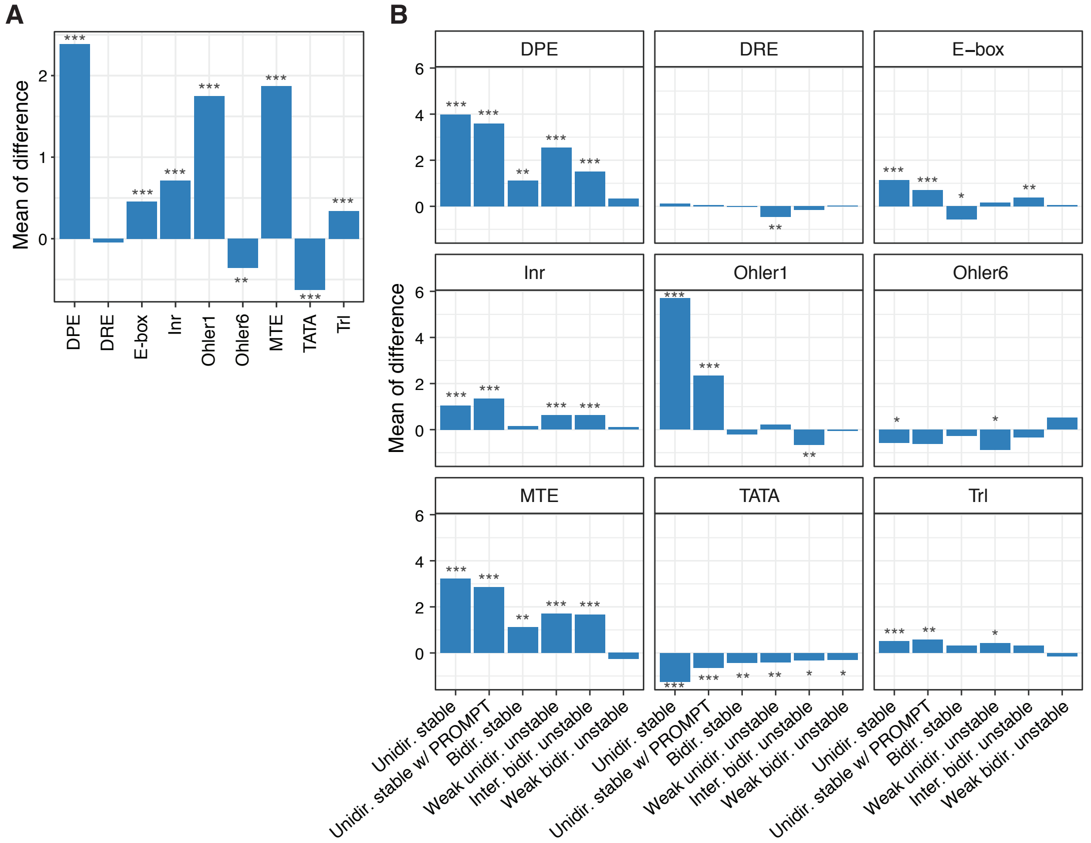

Supplementary Figure 12. Motif score differences between major and minor strand. **(A)** Motif score mean of differences between major and minor strand. **(B)** Motif score mean of differences between major and minor strand, tested within each DHS cluster. Significance tested via paired t-test. Significance stars interpreted as: $* = P < 0.05$, $** = P < 0.01$ or $*** = P < 0.0001$.

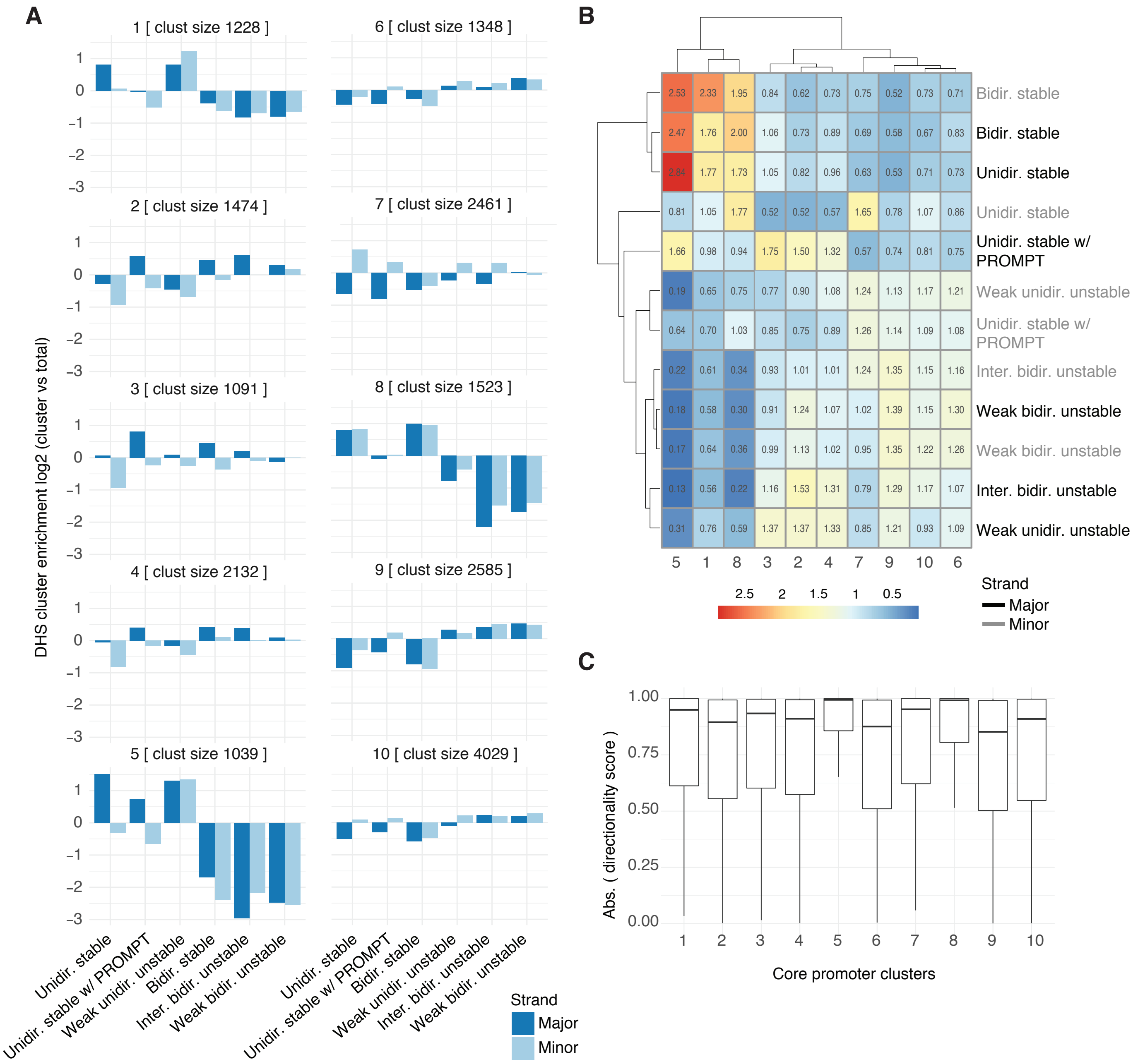

Supplementary Figure 13. DHS enrichment for identified core promoter element clusters. **(A)** DHS class enrichments, calculated as the fraction of DHSs in each DHS class associated with each core promoter element cluster versus the fraction of total transcribed DHSs, displayed in $\log_2$ scale enrichment, broken up by major and minor strand. **(B)** DHS class enrichment, hierarchically clustered with complete linkage, for major and minor strands separately, within each identified core promoter element clusters. **(C)** Absolute directionality score within each identified core promoter cluster.

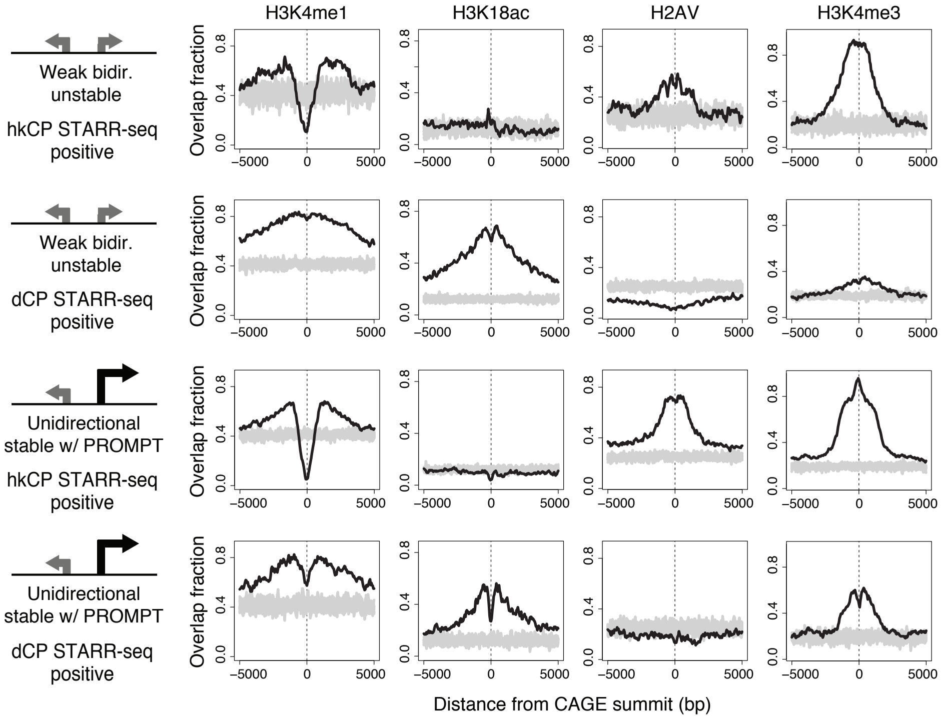

Supplementary Figure 14. Binding enrichments at DHS classes with respect to enhancer potential. Detailed binding enrichments for H3K4me1, H3K18ac, H2Av, and H3K4me3 at *weak bidirectional unstable* and *unidirectional stable w/ PROMPT* DHSs, broken up according to STARR-seq enhancer potential (overlapping either a hkCP or dCP enhancer), based on binding proportions within 5,000 bp from the CAGE summit. Grey represents background distribution based on randomised locations, generated 10 times per plot.

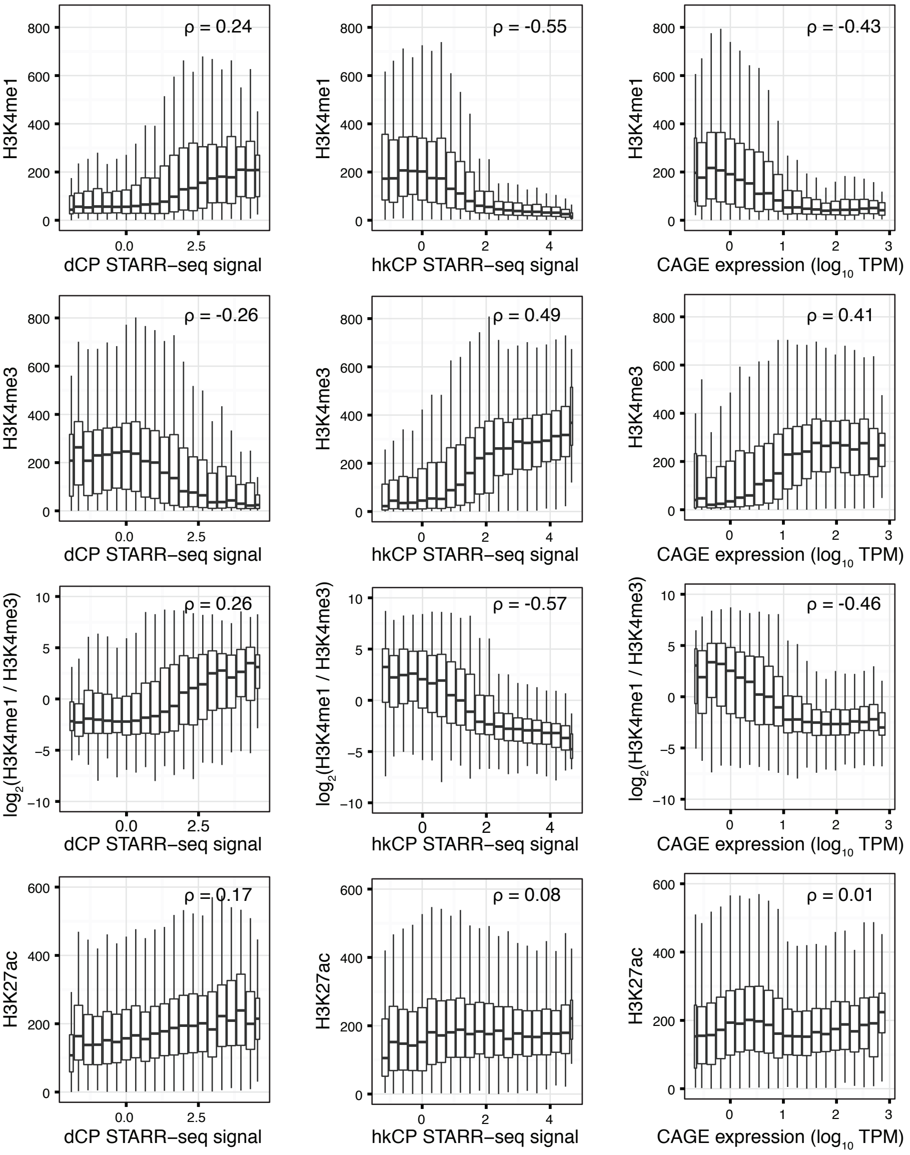

Supplementary Figure 15. Histone modifications versus STARR-seq signal and expression strength. Normalised ChIP-seq data of H3K4me1, H3K4me3, $\log_2$ H3K4me1 over H3K4me3 ratio, and H3K27ac (vertical axis) versus binned dCP (left) and hkCP (middle) STARR-seq signal, and binned TPM scaled $\log_{10}$ CAGE expression (right) (horisontal axes). Spearman's rho statistics calculated on non-binned data are displayed in the top right corners of panels. All associations are significantly different from zero (Spearman rank correlation test, $P < 2.2 \times 10^{-16}$), except H3K27ac versus CAGE expression.

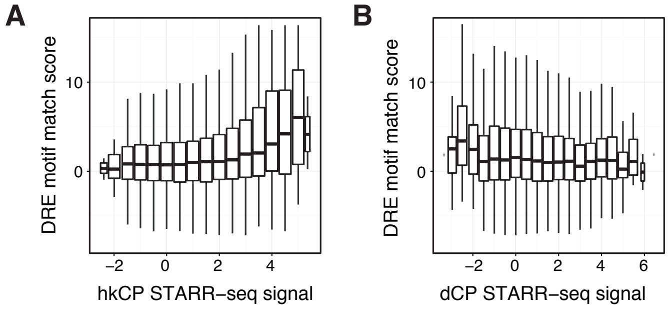

Supplementary Figure 16. DRE motif score versus STARR-seq signal. FIMO DRE motif match score (see Methods) versus STARR-seq $\log_2$ signal fold change versus input for hkCP (A) and dCP (B) enhancer potential. STARR-seq signal is binned in 0.5 sized ranges.

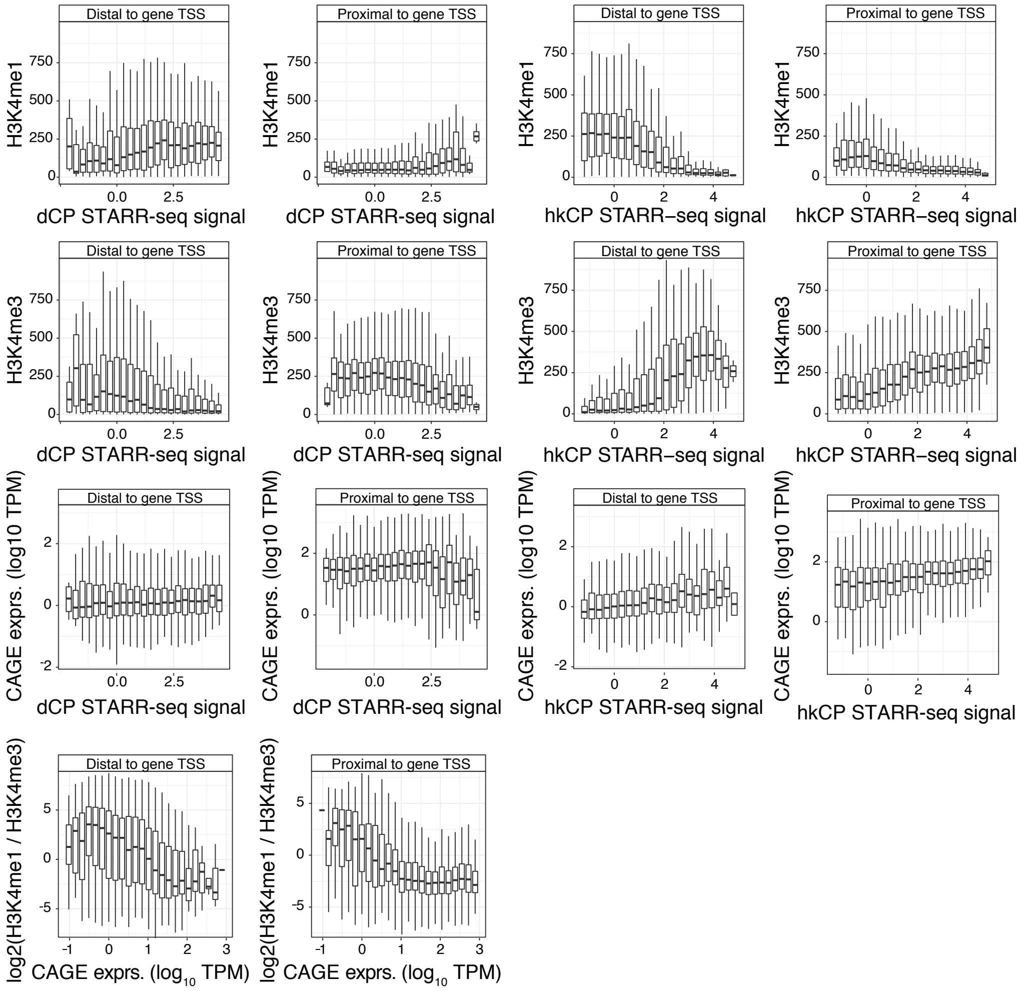

Supplementary Figure 17. Histone modifications versus STARR-seq signal and expression strength of gene proximal and distal DHSs. Normalised ChIP-seq data of H3K4me1, H3K4me3, $\log_2$ H3K4me1 over H3K4me3 ratio, and TPM scaled expression (vertical axis) versus binned dCP (left) and hkCP (right) STARR-seq signal, and binned TPM scaled $\log_{10}$ CAGE expression (bottom) (horisontal axes).

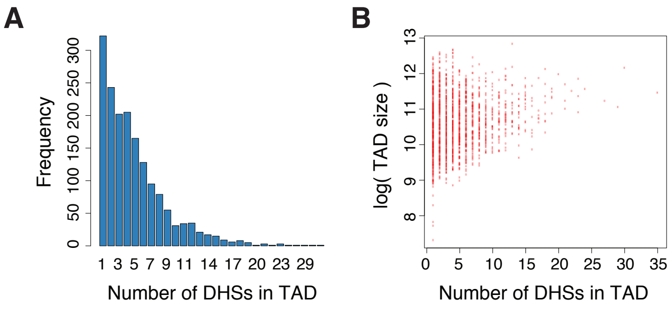

Supplementary Figure 18. DHSs linked to TAD information. **(A)** The frequency of transcribed DHSs within Kc167 TADs. **(B)** Number of DHSs within a TAD versus the size of the TAD in which they were contained.

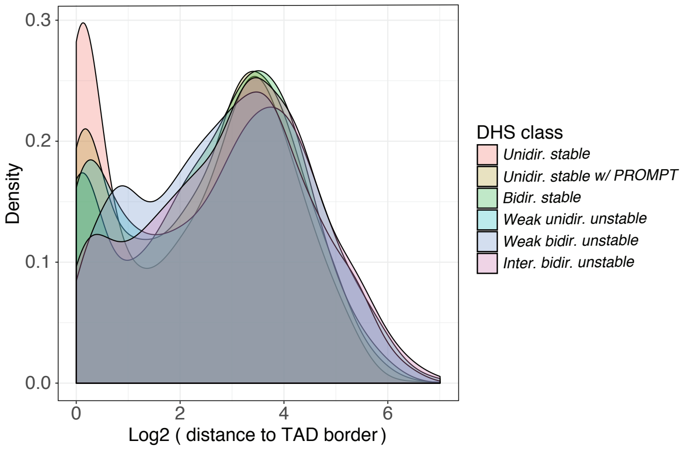

Supplementary Figure 19. DHS distance to TAD borders. Smoothed density estimates of $\log_2$ scaled kb distances per DHS class, measured between DHS windows within Kc167 TADs and TAD borders.

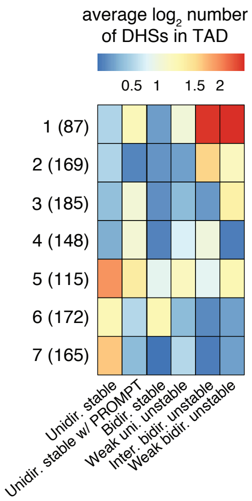

Supplementary Figure 20. Heatmap representing clusters of TADs generated according to the membership of TREs defined by DHS classes. Colours represent the average $\log_2$(number of elements in TAD) for the given DHS class and TAD membership cluster. The number of TADs represented in each TAD cluster is given in parentheses.

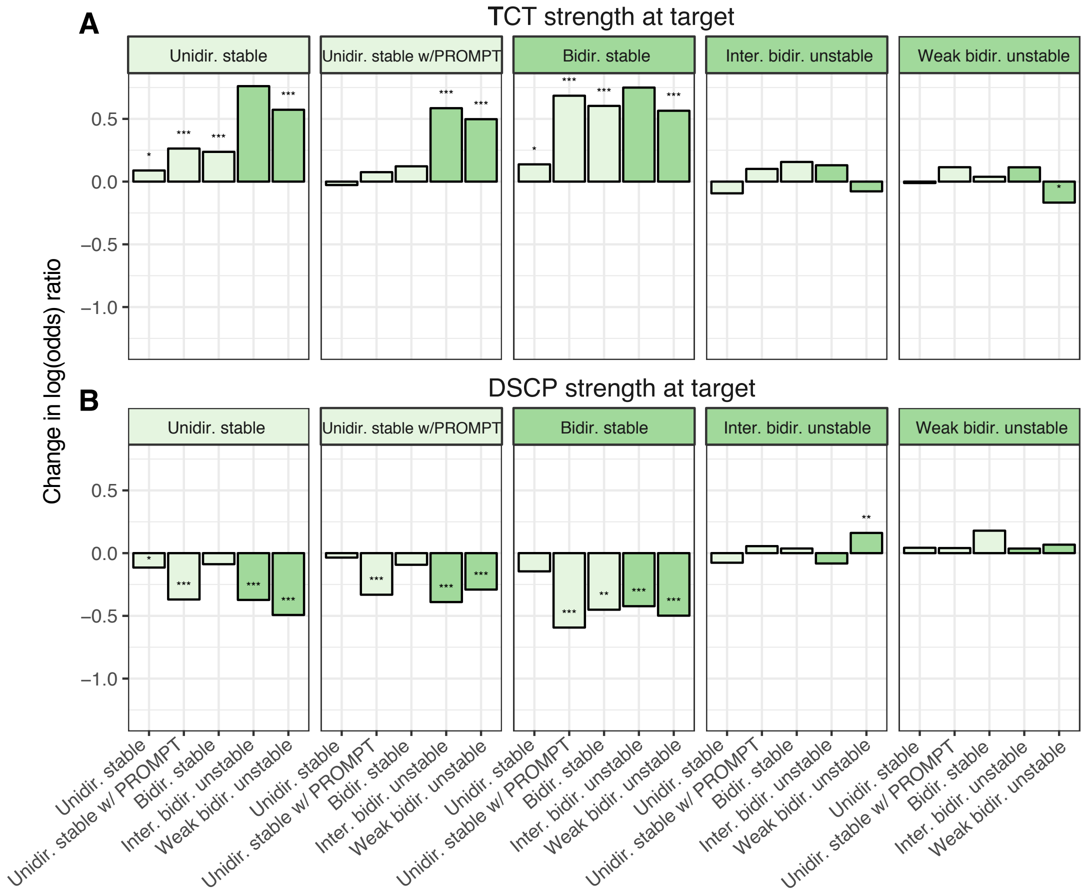

Supplementary Figure 21. Class chromatin-interaction preferences based on modelled interactions with STARR-seq strength overlap (see Methods). Significance stars interpreted as: $* = P < 0.1$, $** = P < 0.01$ or $*** = P < 0.001$. A single model was fitted per bait class, with all covariates together which were scaled prior to model fitting. **(A)** Modelled interaction between target class and hkCP enhancer strength ($\log_2$(signal over input)) at the target. **(B)** Modelled interaction between target class and dCP enhancer strength ($\log_2$(signal over input)) at the target.

SUPPLEMENTARY TABLES

Supplementary Table 1. Cluster agreement statistics from k-medoids clustering of replicate cluster assignments based on the hamming distance between each pair of DHSs (max_diss: max dissimilarity, av_diss: average dissimilarity).

DHS class	size	max_diss	av_diss	diameter	separation
Weak bidir. unstable	1723	3	0.85	4	1
Unidirectional stable w/ PROMPT	1633	3	1.00	4	1
Weak unidir. unstable	1232	2	0.82	4	1
Bidirectional stable	912	2	0.14	4	1
Intermediate bidir. unstable	1556	3	0.88	4	1
Unidirectional stable	2415	2	0.40	4	1

Supplementary Table 2. RT-qPCR relative mean expression. Mean relative expression of three technical replicates (normalised to *Act5C*) for all validated target sites in three biological replicates, listed by DHS coordinates and FlyBase gene name, where P indicates PROMPT target upstream and antisense of gene, or enhancer ID (E) including TC strand information.

DHS ID	Name	Ctrl 1	KD 1	Ctrl 2	KD 2	Ctrl 3	KD 3
3R:137140-137290	*Suv3*	4.605e-03	7.49e-03	3.854e-03	6.201e-03	4.445e-03	5.764e-03
3L:23119040-23119190	*CG33217*	0.023219	0.029347	0.018738	0.033844	0.02343	0.030002
2R:12083780-12083930	*Calypso*	7.038e-03	0.010163	7.921e-03	8.561e-03	8.036e-03	9.162e-03
2R:12083780-12083930	*CG44242*	0.033783	0.03073	0.028301	0.023585	0.02938	0.02526
2L:5029380-5029530	$Cg25c_P$	1.46e-04	6.25e-04	1.35e-04	5.93e-04	2.38e-04	5.36e-04
3R:5399840-5399990	$CG9399_P$	6.36e-04	3.35e-03	3.29e-04	3.479e-03	2.72e-04	1.919e-03
2R:1973080-1973230	$Ars2_P$	8.3e-05	3.13e-04	1.08e-04	3.64e-04	1.04e-04	3.03e-04
3L:685600-685750	$Ebd1_P$	1.913e-03	0.026606	2.35e-03	0.018791	2.468e-03	0.021944
3R:8256880-8257030	$Prosa2_P$	2.49e-04	1.697e-03	3.71e-04	2.045e-03	4.12e-04	1.73e-03
2L:5306660-5306810	$E1_{plus}$	7.4e-04	4.581e-03	7.12e-04	3.491e-03	8.37e-04	2.96e-03
2L:5306660-5306810	$E1_{minus}$	6.28e-04	4.747e-03	6.57e-04	3.997e-03	7.78e-04	3.875e-03
X:1176600-1176750	$E2_{plus}$	3.64e-04	3.588e-03	6.08e-04	2.796e-03	8.61e-04	2.587e-03
X:1176600-1176750	$E2_{minus}$	3.9e-04	2.648e-03	4.98e-04	3.24e-03	7.81e-04	2.742e-03
X:12649700-12649850	$E3_{plus}$	6.52e-04	3.354e-03	4.15e-04	1.626e-03	3.49e-04	2.101e-03
X:12649700-12649850	$E3_{minus}$	7.59e-04	5.212e-03	8.05e-04	5.071e-03	1.233e-03	4.064e-03
3L:17920340-17920490	$E4_{plus}$	1.28e-04	1.041e-03	1.79e-04	1.107e-03	2.32e-04	1.008e-03
3L:17920340-17920490	$E4_{minus}$	2.052e-03	9.212e-03	1.599e-03	8.322e-03	1.889e-03	0.010724
3R:26216920-26217070	$E5_{plus}$	6.8e-05	1.147e-03	5.3e-05	1.27e-03	8e-05	9.08e-04
3R:26216920-26217070	$E5_{minus}$	8.8e-05	1.082e-03	7e-05	9.55e-04	9.9e-05	8.52e-04

Supplementary Table 3. Oligos for PCR templates for making dsRNA.

Promoter	strand	sequence
GFP-T7	forward	TAATACGACTCACTATAGGGAGAATGGTGAGCAAGGGCGAGGAGCTG
GFP-T7	reverse	TAATACGACTCACTATAGGGAGAGCGGTCACGAACTCCAGCAG
Rrp6-T7	forward	TAATACGACTCACTATAGGGAGATCTGTTGATAGACACTGCCC
Rrp6-T7	reverse	TAATACGACTCACTATAGGGAGAATTCTCTTAAACCGCTGGCC
Dis3-T7	forward	TAATACGACTCACTATAGGGAGAGACGGAAATGGCGACAGTAT
Dis3-T7	reverse	TAATACGACTCACTATAGGGAGATGATGACGCTCTTGTGGAAG

Supplementary Table 4. Oligos for qPCR.

Target	forward sequence	reverse sequence
Suv3	TACTCCATATGAGGTGGCTG	TTTTTGCAGGAGGTCCAATG
CG33217	TATTTTCCGCTATGGCACTC	TAAGGCAGCAATAGTTGAGC
Calypso	AGGATTTTGGATGTCACGATG	AGCCATATGGACTCTCGATG
CG44242	CCAGAAGGCAAGTTCCCAG	ATTAATGTAAGCCGCCTCTTC
$Cg25c_{PROMPT}$	GAAACTTGGCGATAAGTCCG	TGATGTGCTATTGAGCAACTT
$CG9399_{PROMPT}$	CGAGCTGGAACCACTTTCATA	TTACCCATCATCTACATGGGC
$Ars2_{PROMPT}$	TTGTTACCTATCCACCTTCCA	TCTCAATTGGATTATGCCGAG
$Ebd1_{PROMPT}$	ATTATCGGCAGGAACCATGT	GAGGGTTACGAGAAGACCTA
$Prosa2_{PROMPT}$	GGGCACACTACCGATTTCTAA	CTATGCGGCACTAAATCGGA
$\mathrm{E1}_{plus}$	CAGACAAGATCGCATTAGACA	AATCAGGCTGAGCATTTGATA
$\mathrm{E1}_{minus}$	TGCTCAAAGAAGCACATAGTT	ACTACAAGCAGGATAATCCCA
$\mathrm{E2}_{minus}$	TTGATCGTCCCTTCATCTCA	GCTACCACGATAGTTCCGTA
$\mathrm{E2}_{plus}$	TCTAATCGGGCTATTCGGG	GATTGATAGGCGACACTAGG
$\mathrm{E3}_{minus}$	CTGTTTTGTTACAAGCGCGAA	AGCGAGATGTGAAACAGTAAAA
$\mathrm{E3}_{plus}$	TTCTGAAACGCAATATCCCAA	AAAAAGACAAGAGCTTGGTTCA
$\mathrm{E4}_{minus}$	AAAGTACTCACTCGCCCTAA	GGCATCTCGAATACCCCTTA
$\mathrm{E4}_{plus}$	CTTACCTTACAGTTCATCGGT	ATAGGGTTTTTATGCGCCAG
$\mathrm{E5}_{plus}$	CATCGCAGTTTCCCTATCTT	TCACAAATAGGGATTCACAACA
$\mathrm{E5}_{minus}$	CACTGCGAGAGATAGGTTTC	CAGAGACTGGAATCACTTAGG
