# Supplementary material for: Transcription start site analysis reveals widespread divergent transcription in D. melanogaster and core promoter-encoded enhancer activities
Source: Nucleic Acids Res. 2018 Apr 6;46(11):5455–69. doi: 10.1093/nar/gky244 (PMC6009668; doi:10.1093/nar/gky244)
Supplement: Supplementary Data [file gky244_supplemental_files.zip › Supplementary_File_2.pdf]

DHS.strand score.DPE score.DRE score.E-box score.INR score.Ohler1 score.Ohler6 score.MTE score.TATA score.Trl p.value.DPE p.value.DRE p.value.E-box p.value.INR p.value.Ohler1 p.value.Ohler6 p.value.MTE p.value.TATA p.value.Trl
2L:10002460-10002610:minus -31 2.45872 -1.19266 11.2936 -18.4898 0.204082 -17.7982 -0.694444 -2.87156 0.0105 0.00424 0.0169 0.0000139 0.0116 0.000296 0.0145 0.0206 0.043
2L:10002460-10002610:plus -22.8878 -1.72477 1.16514 4.15596 -18.9796 -9.37755 -15.2569 -1.66667 4.14679 0.00441 0.0179 0.0078 0.00539 0.0155 0.00265 0.00944 0.0272 0.00406
2L:10003680-10003830:minus -24.1531 1.90826 -0.623853 6.29358 -27.2347 -9.53061 -20.8991 3.25 -4.76147 0.00744 0.00519 0.0142 0.00202 0.0283 0.0031 0.0241 0.00542 0.0686
2L:10003680-10003830:plus -13.449 5.66972 0.311927 6.21101 -18.3776 -27.8265 -6.85321 -0.513889 3.46789 0.000914 0.00123 0.0104 0.00213 0.00926 0.0498 0.00163 0.0196 0.00582
2L:10004100-10004250:minus -23.9286 1.22936 -2.04587 2.21101 -18.1939 -9.7551 -6.29358 4.09722 2.15596 0.00701 0.00666 0.0219 0.0129 0.0086 0.00358 0.00142 0.00386 0.00881
2L:10004100-10004250:plus -34.1429 3.09174 -3.66972 4.12844 -17.8878 -26.4898 -18.1835 8.09722 3 0.0313 0.00337 0.0347 0.00549 0.00704 0.0225 0.0154 0.000552 0.00702
2L:10004640-10004790:minus -31.3265 2.10092 3.98165 3.36697 -27.2653 -36.8878 -11.5413 -2.86111 4.13761 0.0114 0.00484 0.0027 0.00764 0.0302 0.123 0.00418 0.0373 0.00408
2L:10004640-10004790:plus -22.4388 0.807339 7.13761 2.6422 -18.1531 -26.6327 -17.7982 -4.29167 8.19266 0.00348 0.00776 0.00068 0.0107 0.00829 0.0251 0.0145 0.0527 0.000548
2L:10009360-10009510:minus -23.7857 7.94495 1.87156 4.79817 -8.93878 -8.72449 -17 4.79167 1.36697 0.00679 0.000394 0.00606 0.00453 0.00216 0.00169 0.0128 0.00288 0.0124
2L:10009360-10009510:plus -14.0714 12.844 1.01835 1.78899 -18.5306 -9.30612 -7.11927 2.52778 1.22936 0.00117 0.0000255 0.00821 0.0158 0.0118 0.00257 0.00174 0.00712 0.0132
2L:10009940-10010090:minus -22 -1.89908 14.3578 7.45872 -17.9286 -8.5 -11.8073 -0.305556 7.62385 0.00291 0.0189 4.55e-06 0.000894 0.0071 0.00126 0.00442 0.0184 0.000941
2L:10009940-10010090:plus -31.7041 -3.34862 12.3119 7.29358 -17.1122 -9.42857 -23.2569 -0.194444 3.6422 0.0146 0.0284 0.0000343 0.00104 0.00341 0.0027 0.0366 0.0178 0.00516
2L:10056840-10056990:minus -13.6735 4.51376 7.87156 4.02752 -17.6837 -8.7551 -13.2844 -2.41667 -1.33945 0.00103 0.00198 0.000469 0.00581 0.0058 0.00172 0.00617 0.0333 0.0285
2L:10056840-10056990:plus -32.398 8.54128 0.504587 2.14679 -17.449 -26.8265 -23.0275 0.472222 1.21101 0.019 0.000285 0.0098 0.0133 0.00484 0.0285 0.0352 0.0144 0.0134
2L:10057400-10057550:minus -3.56122 -0.798165 8.65138 4.33945 -27.3061 -28.3367 -12.7431 -1.70833 9.55046 0.0000803 0.0134 0.000306 0.00509 0.0315 0.0606 0.00545 0.0275 0.000321
2L:10057400-10057550:plus -40.4082 -2.0367 -0.238532 9.59633 -18.9796 -18.5714 -22.2385 -1.47222 -0.642202 0.0399 0.0196 0.0125 0.000275 0.0155 0.0136 0.0308 0.0258 0.0234
2L:10060480-10060630:minus -14.4082 0.0366972 -0.146789 5.48624 -18.1531 -27.7857 2.46789 -3.94444 1.44037 0.00136 0.0102 0.0122 0.00357 0.00829 0.0466 0.000142 0.0486 0.012
2L:10060480-10060630:plus -32.2143 4.05505 0.614679 4.31193 -9.27551 -27.602 -10.5505 7.16667 0.348624 0.0182 0.00235 0.00943 0.00516 0.00284 0.0416 0.00342 0.000922 0.0167
2L:10143400-10143550:minus -39.9898 -0.495413 -2.42202 2.44037 -17.5612 -18.5714 -16.156 4.72222 7.25688 0.0351 0.0122 0.0244 0.0116 0.00544 0.0136 0.0111 0.00296 0.00115
2L:10143400-10143550:plus -13.1429 0.12844 12.4862 7.29358 -18.4184 -15.9796 -21.3394 5.45833 1.02752 0.00065 0.00987 0.0000304 0.00104 0.0101 0.00405 0.0262 0.00214 0.0141
2L:10201000-10201150:minus -22.0714 8.05505 2.05505 2.70642 20.4694 -17.051 -6.3945 -3.51389 4.13761 0.00299 0.000371 0.00566 0.0103 1.65e-07 0.00479 0.00145 0.0439 0.00408
2L:10201000-10201150:plus -21.6633 -1.95413 -0.715596 2.92661 -17.0816 -9.16327 -20.789 0.777778 -0.284404 0.00223 0.0192 0.0146 0.00931 0.00334 0.00223 0.0237 0.0131 0.0207
2L:10204280-10204430:minus -40.2551 0.798165 -2.16514 2.78899 20.4694 9.53061 -15.5046 5.54167 8.11009 0.0382 0.00779 0.0227 0.00992 1.65e-07 0.0000316 0.0099 0.00205 0.000597
2L:10204280-10204430:plus -21.8878 3.2844 0.66055 6.21101 -18.449 -9.86735 2.99083 2.23611 -0.926606 0.00258 0.00313 0.00929 0.00213 0.0107 0.00391 0.000119 0.00792 0.0256
2L:10204760-10204910:minus -31.8878 -0.229358 -0.963303 5.30275 -9.20408 -17.2755 -14.3303 3.70833 5.53211 0.0164 0.0111 0.0158 0.00396 0.00262 0.00544 0.0078 0.00452 0.00245

2L:10204760-10204910:plus -14.7041 -1.26606 0.504587 7.46789 -17.4184 0.94898 -18.4587
1.56944 -4.76147 0.00154 0.0156 0.0098 0.000868 0.00457 0.00014 0.016 0.01 0.0686
2L:10206860-10207010:minus -23.2653 -1.78899 6.15596 3.62385 -7.67347 -26.6735 -11.211
0.694444 7.42202 0.00593 0.0183 0.00108 0.00673 0.00055 0.0256 0.0039 0.0134 0.00106
2L:10206860-10207010:plus -23.449 -0.348624 0.834862 4.9633 -27.4592 -8.79592 -19.578
7.13889 -0.165138 0.00631 0.0116 0.00875 0.0044 0.0329 0.0019 0.0192 0.000935 0.0195
2L:10209000-10209150:minus -23.3776 -2.0367 -0.477064 2.99083 -8.93878 -9.02041 -17.367
5.75 1.69725 0.00619 0.0196 0.0135 0.00899 0.00216 0.00213 0.0136 0.00187 0.0108
2L:10209000-10209150:plus -23.3367 -1.22018 2.09174 6.68807 -9.20408 -8.23469 -0.559633
4.18056 2.93578 0.00614 0.0154 0.00558 0.00157 0.00262 0.000875 0.000322 0.00373 0.00717
2L:10211120-10211270:minus -32.2245 0.302752 2.6422 6.84404 -27.2245 0.826531 -27.3578
0.708333 1.2844 0.0183 0.00929 0.00455 0.0014 0.0272 0.000153 0.0633 0.0134 0.0129
2L:10211120-10211270:plus -32.3265 1.66055 3.99083 1.44954 -8.60204 -9.42857 -19.8991
6.06944 -3.70642 0.0187 0.00569 0.00269 0.0179 0.00125 0.0027 0.0202 0.0016 0.0517
2L:10220080-10220230:minus -39.4796 3.20183 2.80734 8.77982 -18.6735 -0.244898 -23.789 7.61111
1.48624 0.0334 0.00323 0.00427 0.000533 0.0123 0.000496 0.0398 0.000726 0.0118
2L:10220080-10220230:plus -22.8571 -1.66055 7.66055 11.0183 -17.3776 -9.57143 -22.9541
2.66667 -0.0825688 0.00427 0.0176 0.000523 0.000052 0.00419 0.00326 0.0348 0.00677 0.0189
2L:10220880-10221030:minus -23.0408 -3.3211 8.05505 9.08257 -17.602 -7.79592 -8.87156 0.722222
3.99083 0.00512 0.0282 0.000425 0.000444 0.00547 0.000723 0.0025 0.0133 0.00433
2L:10220880-10221030:plus -31.6224 2.19266 -0.788991 5.69725 -27.3061 -9.7551 -6.02752
0.333333 3.66972 0.0137 0.00468 0.0149 0.00314 0.0315 0.00358 0.00132 0.0151 0.00514
2L:10221220-10221370:minus -50.0714 0.0183486 0.0275229 6.83486 -18.4184 -8.60204
-24.7064 2.48611 1.88073 0.122 0.0102 0.0115 0.00142 0.0101 0.00151 0.0454 0.00723 0.00987
2L:10221220-10221370:plus -31.3673 -1.19266 4.57798 2.31193 -26.6327 -16.9796 -9.66055
1.47222 -2.81651 0.0116 0.0152 0.00212 0.0123 0.0188 0.0044 0.00289 0.0104 0.0424
2L:10222580-10222730:minus -30.9286 2.97248 0.311927 9.59633 -18.7143 -8.45918 -16.2202
0.277778 -6.27523 0.0104 0.00351 0.0104 0.000275 0.0136 0.00111 0.0113 0.0153 0.0952
2L:10222580-10222730:plus -32.2653 3.86239 2.22936 6.27523 20.4694 -7.72449 -23.0092 2.36111
-9.04587 0.0184 0.00253 0.00531 0.00206 1.65e-07 0.00068 0.0351 0.00757 0.167
2L:10226440-10226590:minus -32.3367 4.87156 12.6697 5.74312 -18.7551 -19.5714 -13.7706
6.93056 -2.83486 0.0187 0.00172 0.0000269 0.00302 0.0144 0.0202 0.00689 0.00104 0.0427
2L:10226440-10226590:plus -40.551 5.05505 2.54128 3.76147 -17.1531 -8.5 -16.6055 4.44444 -1.6422
0.0418 0.00159 0.00473 0.00645 0.00364 0.00126 0.012 0.00334 0.0304
2L:10226720-10226870:minus -31.9694 3.06422 -1.05505 1.53211 -17.1531 -8.5 -16.0826
4.30556 -1.05505 0.0168 0.0034 0.0162 0.0174 0.00364 0.00126 0.011 0.00354 0.0264
2L:10226720-10226870:plus -33.0102 0.174312 -4.79817 0.733945 -28.7959 -8.02041
-24.5413 5.29167 1.74312 0.0259 0.00971 0.0469 0.0222 0.0641 0.000761 0.0443 0.0023 0.0105
2L:10227480-10227630:minus -21.9286 3.2844 -1.69725 5.89908 -27.5306 -8.23469 -15.8899
4.25 -2.0367 0.0027 0.00313 0.0197 0.00272 0.0351 0.000875 0.0106 0.00362 0.0336
2L:10227480-10227630:plus -33.2245 3.76147 2.81651 1.76147 -18.7449 -17.2755 -20.4587
0.277778 -4.37615 0.0276 0.00263 0.00426 0.0159 0.0138 0.00544 0.0223 0.0153 0.0617
2L:102280-102430:minus -4.22449 8.46789 3.22018 6.16514 -9.16327 -18.0102 -7.27523 -1.88889
-0.376147 0.00013 0.000296 0.00365 0.00223 0.00249 0.00812 0.0018 0.0289 0.0211
2L:102280-102430:plus -32.3061 14.5046 -0.366972 4.81651 -18.5306 -8.38776 -26.1835
4.23611 1.54128 0.0186 4.87e-06 0.0131 0.00451 0.0118 0.00101 0.0547 0.00364 0.0115
2L:10230720-10230870:minus -31.8163 -1.40367 -0.146789 3.01835 -18.4184 -7.23469
-22.1284 5.44444 -3.42202 0.016 0.0163 0.0122 0.0089 0.0101 0.000557 0.0302 0.00215 0.0488
2L:10230720-10230870:plus -4.04082 7.74312 -1.26606 -0.880734 -7.82653 -19.051 -9.62385
-1.375 -0.0275229 0.000123 0.000438 0.0173 0.0353 0.00059 0.0165 0.00287 0.0251 0.0186
2L:10231680-10231830:minus -22.1122 8.45872 6.31193 9.44037 -8.16327 -0.0204082 -20.6422
0.527778 1.22936 0.00304 0.000297 0.001 0.000339 0.000873 0.000411 0.0231 0.0142 0.0132
2L:10231680-10231830:plus -14.7857 -2.10092 -0.862385 6.06422 -18.9796 -18.2755
-23.7798 1.41667 3.15596 0.00158 0.02 0.0153 0.00239 0.0155 0.0112 0.0397 0.0106 0.00659

2L:10232340-10232490:minus -32.9592 0.605505 5.92661 5.90826 -17.3776 -18.0102 -12.5138
2.40278 -0.238532 0.0252 0.00834 0.00119 0.00271 0.00419 0.00812 0.00517 0.00746 0.0203
2L:10232340-10232490:plus -23.2245 14.6147 3.56881 6.21101 -18.7143 -8.72449 -26.789 0.180556
2.74312 0.00576 4.19e-06 0.00318 0.00213 0.0136 0.00169 0.0589 0.0158 0.00751
2L:10239300-10239450:minus -33.0306 8.54128 1.12844 4.02752 -18.4184 -17.8571 -17.0183
5.375 -3.55963 0.026 0.000285 0.00791 0.00581 0.0101 0.00719 0.0129 0.00222 0.0503
2L:10239300-10239450:plus -31.551 7.97248 0.137615 3.9633 -27.0408 -18.1224 -14.7706
2.80556 1.01835 0.0134 0.000387 0.0111 0.0059 0.0242 0.00988 0.00856 0.00642 0.0142
2L:10239720-10239870:minus -22.4898 0.623853 -0.0183486 4.86239 -18.449 9.67347 -23.9908
-1.31944 0.12844 0.00353 0.00829 0.0117 0.00449 0.0107 0.0000217 0.041 0.0247 0.0179
2L:10239720-10239870:plus -32.7041 2.06422 -0.201835 -0.100917 -17.6429 -10.0204
-18.8716 8.98611 3.98165 0.0222 0.0049 0.0124 0.0277 0.00562 0.00399 0.0171 0.000323 0.00439
2L:10246680-10246830:minus -39.9898 -2.9633 0.889908 2.45872 -18.7551 -17.1224 -24.2752
4.41667 -0.642202 0.0351 0.0255 0.00859 0.0116 0.0144 0.00499 0.0427 0.00338 0.0234
2L:10246680-10246830:plus -30.6633 -2.83486 0.733945 3.19266 -26.898 -0.244898 -14.055
1.18056 -2.3945 0.00976 0.0246 0.00906 0.00829 0.0203 0.000496 0.00734 0.0114 0.0375
2L:10249880-10250030:minus -29.1429 13.5306 -1.83486 6.09174 -17.4184 1.38776 -12.8257
2.56944 1.12844 0.00834 0.0000206 0.0206 0.00235 0.00457 0.0000559 0.00555 0.00701 0.0137
2L:10249880-10250030:plus -21.6633 7.36697 6.10092 7.20183 -26.5306 -9.5 -15.7339 2.94444
-3.87156 0.00223 0.000535 0.0011 0.00112 0.0185 0.00294 0.0103 0.00609 0.0537
2L:10250680-10250830:minus -31.7347 9.29358 0.192661 3.05505 0.908163 0.755102 -16.8532
1.47222 -1.01835 0.0149 0.000192 0.0109 0.00878 0.000153 0.000175 0.0125 0.0104 0.0263
2L:10250680-10250830:plus -23.0714 6.55963 1.98165 0.137615 -18.7143 -9.20408 -18.1468
5.51389 4.19266 0.0052 0.00081 0.00582 0.0259 0.0136 0.00225 0.0153 0.00208 0.00401
2L:10255820-10255970:minus -31.8878 -2.78899 1.66972 3.17431 -7.93878 -9.30612 -8.3945
2.625 -3.53211 0.0164 0.0243 0.00652 0.00842 0.000767 0.00257 0.00228 0.00687 0.0501
2L:10255820-10255970:plus -41.3673 -4.33028 6.48624 1.57798 -7.86735 -7.79592 -26.1651
-0.111111 5.70642 0.0551 0.037 0.000925 0.0171 0.000617 0.000723 0.0546 0.0173 0.00222
2L:10260900-10261050:minus -14.5204 6.33945 -2.84404 1.86239 -8.60204 -17.1224 -14.5138
-2.66667 9.9633 0.00145 0.0009 0.0276 0.0153 0.00125 0.00499 0.00811 0.0355 0.000171
2L:10260900-10261050:plus -12.6633 8.38532 -2.06422 2.15596 -18.449 -18.4184 -12.1927
-3.19444 4.02752 0.000458 0.000309 0.022 0.0133 0.0107 0.0127 0.00481 0.0406 0.00428
2L:10263520-10263670:minus -23.3367 6.18349 4.63303 1.92661 -18.3776 0.755102 -10.0367
0.0416667 -0.348624 0.00614 0.000972 0.00207 0.0148 0.00926 0.000175 0.0031 0.0165 0.0209
2L:10263520-10263670:plus -40.2653 0.0733945 1.13761 -1.91743 -26.8571 0.530612
-22.4862 3.625 5.33028 0.0383 0.0101 0.00788 0.0499 0.0198 0.000203 0.0321 0.00468 0.00273
2L:10264200-10264350:minus -34 -2.26606 4.25688 5.55046 -9.23469 -18.3469 -16.3303
5.06944 2.0367 0.0307 0.021 0.00242 0.00345 0.00277 0.0124 0.0115 0.00255 0.00923
2L:10264200-10264350:plus -33.0714 -0.834862 3.42202 -0.0733945 -28.4898 -9.72449
-27.2018 7.20833 3.72477 0.0267 0.0136 0.00337 0.0274 0.0581 0.00352 0.062 0.000902 0.00497
2L:10265220-10265370:minus -23.4898 0.908257 4.81651 3.14679 -18.4184 -8.72449 -18.6789
4.58333 -7.51376 0.00639 0.00748 0.00192 0.00847 0.0101 0.00169 0.0166 0.00315 0.124
2L:10265220-10265370:plus -22.2245 6.84404 0.33945 2.86239 -18.7143 -18.0102 -15.4128
8.40278 1.49541 0.00325 0.000705 0.0104 0.00954 0.0136 0.00812 0.00973 0.000462 0.0118
2L:10266560-10266710:minus -22.8163 -5.30275 -0.330275 2.26606 -19.0204 -17.3469
-18.211 0.305556 -3.24771 0.00418 0.0478 0.0129 0.0126 0.0158 0.0058 0.0154 0.0152 0.0471
2L:10266560-10266710:plus -34.2653 2.0367 -1.11009 5.29358 1.17347 -9.82653 -12.9083
3.88889 4.14679 0.0316 0.00495 0.0165 0.00398 0.000101 0.00381 0.00566 0.00421 0.00406
2L:10268800-10268950:minus -34.3367 2.99083 0.155963 3.88991 -17.7143 -0.173469 -22.2477
8.01389 9.92661 0.0318 0.00349 0.011 0.00608 0.00627 0.000456 0.0308 0.000579 0.000193
2L:10268800-10268950:plus -33.0306 -3.2844 1.46789 2.09174 10.3571 -17.1224 -11.6239 1.16667
-8.00917 0.026 0.0279 0.007 0.0137 0.0000208 0.00499 0.00425 0.0115 0.136
2L:10269940-10270090:minus -31.8878 5.04587 3.81651 9.49541 -26.5306 -8.45918 -12.3303
0.638889 -1.02752 0.0164 0.0016 0.00289 0.000308 0.0185 0.00111 0.00496 0.0137 0.0263

2L:10269940-10270090:plus -14.8163 2.61468 11.2661 7.78899 -8.7449 -27.3367 -1.16514 -1.44444
-3.66055 0.00159 0.004 0.0000678 0.000642 0.00161 0.0347 0.000368 0.0256 0.0513
2L:10277060-10277210:minus -21.4082 -1.61468 -2.81651 2.25688 -18.4184 -9.57143
-22.0183 5.875 -2.26606 0.00198 0.0173 0.0274 0.0127 0.0101 0.00326 0.0296 0.00176 0.0359
2L:10277060-10277210:plus -33.0408 -0.642202 -0.761468 -3.50459 -17.1837 1.82653
-15.9541 3.23611 1.95413 0.0264 0.0128 0.0148 0.0761 0.00366 0.0000498 0.0108 0.00545 0.00964
2L:10277500-10277650:minus -32.7347 5.19266 -1.51376 -1.07339 -17.449 -17.5714 -14.2202
1.52778 -4.34862 0.0225 0.0015 0.0187 0.0377 0.00484 0.00616 0.00761 0.0102 0.0612
2L:10277500-10277650:plus -31.7041 11.4404 3.27523 0.00917431 -8.86735 -27.1939 -1.52294
4.86111 5.44037 0.0146 0.0000634 0.00357 0.0269 0.00173 0.0327 0.000398 0.00279 0.00259
2L:10278780-10278930:minus -44.1429 1.50459 -2.40367 2.12844 -26.2653 -7.86735 -26.7431
6.29167 1.69725 0.0949 0.00602 0.0243 0.0134 0.018 0.00073 0.0586 0.00144 0.0108
2L:10278780-10278930:plus -23.2959 13.3878 0.678899 4.22018 -17.3776 -18.051 -13.6055
-0.333333 -2.37615 0.00605 0.0000869 0.00923 0.00531 0.00419 0.00886 0.00664 0.0185 0.0373
2L:10291600-10291750:minus -31.7755 -1.18349 2.68807 2.58716 -27.1939 -17.3469 -13.8899
5.08333 -3.22018 0.0157 0.0152 0.00447 0.011 0.0267 0.0058 0.00708 0.00253 0.0469
2L:10291600-10291750:plus -23.5612 5.11927 0.963303 5.49541 -17.6735 -8.72449 -23.3761
2.05556 -6.54128 0.00653 0.00155 0.00837 0.00355 0.00572 0.00169 0.0373 0.00845 0.102
2L:10292240-10292390:minus -23.4898 12.8624 1.76147 5.74312 -27 -17.2755 -9.29358 0.0138889
-2.6055 0.00639 0.0000248 0.00631 0.00302 0.0233 0.00544 0.0027 0.0167 0.04
2L:10292240-10292390:plus -31.7347 12.3761 -2.92661 3.61468 -8.93878 -9.30612 -16.7248
1.77778 3.14679 0.0149 0.0000356 0.0283 0.0068 0.00216 0.00257 0.0123 0.00932 0.00666
2L:10299480-10299630:minus -32.0816 4.90826 2.9633 6.76147 -26.8571 -18.2755 -20.7615
-0.680556 -1.56881 0.0176 0.00169 0.00403 0.00148 0.0198 0.0112 0.0236 0.0206 0.0298
2L:10299480-10299630:plus -32.7347 4.05505 3.94495 1.62385 -27.2245 -8.57143 -18.8624
4.90278 4.45872 0.0225 0.00235 0.00274 0.0168 0.0272 0.00149 0.0171 0.00274 0.00366
2L:10303500-10303650:minus -13.6327 3.6422 6.61468 10.5872 -18.1224 -19.0102 -6.84404
0.527778 -2.83486 0.00101 0.00275 0.000869 0.000115 0.00782 0.0159 0.00163 0.0142 0.0427
2L:10303500-10303650:plus -30.1735 0.33945 1.46789 6.18349 -27.7041 -9.79592 -9.99083
1.05556 1.53211 0.00869 0.00917 0.007 0.00217 0.038 0.00377 0.00307 0.0119 0.0116
2L:10303880-10304030:minus -32.2143 -1.70642 5.72477 11.2936 20.2041 -17.4898 -10.5505 2.05556
0.0825688 0.0182 0.0178 0.0013 0.0000139 3.64e-07 0.0059 0.00342 0.00845 0.0181
2L:10303880-10304030:plus -33.0714 11.4679 4.51376 6.29358 -18.3776 -8.79592 -21.6789 -0.638889
-4.43119 0.0267 0.0000627 0.00218 0.00202 0.00926 0.0019 0.0278 0.0203 0.0627
2L:10306740-10306890:minus -22.1531 10.2385 6.66972 7 10.9184 -18.2041 -11.6881 -4.01389 -4.41284
0.0031 0.000119 0.000847 0.00132 4.19e-06 0.0103 0.00431 0.0494 0.0624
2L:10306740-10306890:plus -32.5918 6.3945 4.40367 3.94495 -26.5306 -16.7143 -12 1.40278 3.41284 0.021
0.000878 0.00228 0.00595 0.0185 0.00419 0.00461 0.0106 0.006
2L:10311280-10311430:minus -40.7755 0 0.00917431 9.49541 -27.2653 -9.79592 -16.7523 9.375
-2.78899 0.0433 0.0103 0.0116 0.000308 0.0302 0.00377 0.0123 0.000251 0.0422
2L:10311280-10311430:plus -30.4796 -1.12844 1.77982 2.19266 -18.0204 -9.20408 -11.844 2.625 -2.44954
0.00941 0.0149 0.00627 0.0131 0.00733 0.00225 0.00446 0.00687 0.0381
2L:10311840-10311990:minus -24.5204 2.57798 6.49541 3.31193 -16.7143 -9.79592 -15.6606 7.52778
-2.31193 0.00813 0.00406 0.000921 0.00786 0.00319 0.00377 0.0102 0.00076 0.0363
2L:10311840-10311990:plus -33 0.66055 5.88991 6.00917 -18.6429 -9.82653 -12.8532 5.94444 -1.00917
0.0258 0.00818 0.00121 0.00255 0.0121 0.00381 0.00559 0.0017 0.0262
2L:10314360-10314510:minus -32.9694 5.77064 5.2844 2.54128 -27.4592 -8.16327 -21.9633 6.5 5.3945
0.0254 0.00117 0.00158 0.0112 0.0329 0.000783 0.0293 0.0013 0.00263
2L:10314360-10314510:plus -23.0714 1.62385 0.0825688 5.73394 -27.9694 -19.6429 -12.9633 5.56944
3.31193 0.0052 0.00577 0.0113 0.00307 0.0437 0.0204 0.00573 0.00203 0.00618
2L:10314700-10314850:minus -42.7041 -2.52294 1.33028 1.61468 -27.2347 -9.09184 -24.2844 5.31944
-0.0275229 0.0842 0.0226 0.00736 0.0169 0.0283 0.00219 0.0428 0.00227 0.0186
2L:10314700-10314850:plus -33.2959 12.1927 14.8257 5.57798 -17.449 -9.5 -21.3853 6.22222 -4.24771
0.0279 0.000041 2.55e-06 0.0034 0.00484 0.00294 0.0264 0.00149 0.0591

2L:10327880-10328030:minus -41.5918 5.2844 0.284404 5.86239 10.3571 -17.9796 -24.5596 2.97222
-4.78899 0.0623 0.00145 0.0105 0.00282 0.0000208 0.00764 0.0445 0.00603 0.0691
2L:10327880-10328030:plus -14.3673 -2.16514 3.15596 2.84404 -17.3776 -18.5 -12.2477 1 -1.43119
0.00132 0.0204 0.00374 0.00961 0.00419 0.0131 0.00487 0.0121 0.029
2L:10331420-10331570:minus -43.0408 -0.504587 3.44954 9.49541 -19.3163 -8.72449 -33.0917 6.45833
-4.84404 0.0892 0.0122 0.00334 0.000308 0.0165 0.00169 0.133 0.00133 0.0701
2L:10331420-10331570:plus -42.5918 0.944954 -1.26606 7.51376 1.61224 10.5714 -25.9174 8.08333 -0.440367
0.0819 0.00739 0.0173 0.000836 0.0000631 4.67e-06 0.0529 0.000556 0.0216
2L:10333700-10333850:minus -14.0408 6.30275 -0.0275229 2.3945 -9.23469 -9.72449 -6.31193 3.79167
1.77064 0.00115 0.000917 0.0117 0.0119 0.00277 0.00352 0.00142 0.00437 0.0103
2L:10333700-10333850:plus -31.4796 -0.449541 5.37615 2.84404 -9.20408 -9.02041 -15.3486 0.0138889
5.38532 0.0128 0.012 0.00152 0.00961 0.00262 0.00213 0.00961 0.0167 0.00267
2L:10335100-10335250:minus -31.8061 6.80734 6.73394 1.9633 -27.9694 -26.8265 -15.8899 11.2361
3.51376 0.0158 0.000716 0.000821 0.0146 0.0437 0.0285 0.0106 0.0000623 0.00566
2L:10335100-10335250:plus -14.8571 7.15596 -1.17431 4.0367 -18.2653 -17.2041 -20.633 3.83333 2
0.0016 0.000599 0.0169 0.00578 0.00882 0.00507 0.023 0.0043 0.00941
2L:10339380-10339530:minus -24 9.7156 2.78899 3.10092 -8.97959 -9.53061 -16.3119 2.97222 -0.284404
0.00719 0.000154 0.0043 0.00865 0.0023 0.0031 0.0114 0.00603 0.0207
2L:10339380-10339530:plus -14.4082 4.99083 1.99083 5.2844 -18.7143 -18.051 -12.5688 -2.20833 3.47706
0.00136 0.00164 0.0058 0.004 0.0136 0.00886 0.00524 0.0315 0.00575
2L:10341500-10341650:minus -31.7041 -0.715596 6.43119 6.20183 -8.82653 0.0510204 -27.2661
4.59722 1.90826 0.0146 0.0131 0.000949 0.00214 0.00163 0.000349 0.0625 0.00313 0.00974
2L:10341500-10341650:plus -22.2653 0.752294 3.48624 3.9633 -26.2653 -18.0816 -17.6972 5.86111 1.95413
0.00331 0.00791 0.00329 0.0059 0.018 0.00934 0.0143 0.00177 0.00964
2L:10342540-10342690:minus -32.8163 14.4128 13.3945 9.33028 -27.2245 -18.0102 -20.1376 5.27778
2.83486 0.024 5.49e-06 0.0000143 0.000374 0.0272 0.00812 0.0211 0.00232 0.00739
2L:10342540-10342690:plus -24.4796 -0.577982 13.6606 7.45872 -8.67347 -18.0102 -7.12844 3.91667
-2.04587 0.00804 0.0125 0.0000112 0.000894 0.00154 0.00812 0.00174 0.00416 0.0336
2L:10356720-10356870:minus -39.0306 -0.752294 -0.568807 2.31193 -27.0102 -17.7857 -13.5963
-2.43056 10.3486 0.0326 0.0132 0.0139 0.0123 0.0237 0.0068 0.00663 0.0334 0.0000988
2L:10356720-10356870:plus -30.4082 -3.6055 15.6239 3.76147 -27.5306 -7.5 -19.2294 2.90278 -2.83486
0.00908 0.0304 5.44e-07 0.00645 0.0351 0.000614 0.0181 0.00619 0.0427
2L:10362720-10362870:minus -33.3061 2.86239 -0.651376 3.07339 -7.60204 -8.20408 -19.4037 5.43056
1.78899 0.0279 0.00366 0.0143 0.00873 0.000466 0.000796 0.0186 0.00216 0.0103
2L:10362720-10362870:plus -13.5918 3.82569 5.61468 6.69725 -17.7143 -25.7551 -21.1101 6.38889 -0.761468
0.00099 0.00257 0.00137 0.00156 0.00627 0.0208 0.0251 0.00137 0.0244
2L:10366640-10366790:minus -41.3673 -0.311927 -2.07339 2 -8.93878 -10.0918 -20.4312 7.86111
1.38532 0.0551 0.0115 0.0221 0.0143 0.00216 0.00404 0.0222 0.000631 0.0123
2L:10366640-10366790:plus -31.9592 0.880734 -0.706422 1.19266 -18.4184 -17.7857 -10.0826 1.63889
1.48624 0.0167 0.00756 0.0146 0.0195 0.0101 0.0068 0.00313 0.00978 0.0118
2L:10375720-10375870:minus -31.4388 0.110092 1.75229 2.51376 10.9898 -9.45918 -16.5596 3.25 4.99083
0.0122 0.00993 0.00633 0.0113 3.28e-06 0.00279 0.0119 0.00542 0.00311
2L:10375720-10375870:plus -21.6939 -5.26606 -0.0825688 7.99083 -25.9286 -7.72449 -14.7615 5.16667
2.88073 0.00226 0.0473 0.0119 0.00059 0.0168 0.00068 0.00855 0.00244 0.00727
2L:10385740-10385890:minus -34.1837 0.458716 2.05505 1.57798 -17.9388 -17.051 -26.2752 4.47222
-1.57798 0.0314 0.00879 0.00566 0.0171 0.00715 0.00479 0.0553 0.0033 0.0299
2L:10385740-10385890:plus -41.7755 -2.30275 1.95413 2.3945 -18.7449 -18.1531 -25.0642 6.5 -4.76147
0.0652 0.0212 0.00588 0.0119 0.0138 0.00997 0.0476 0.0013 0.0686
2L:10386600-10386750:minus -32.1122 10.1743 0.522936 -1.18349 -27.4898 0.755102 -24.8165 5.77778
1.73394 0.0178 0.000123 0.00974 0.0389 0.0336 0.000175 0.046 0.00184 0.0106
2L:10386600-10386750:plus -31.7755 6.81651 2.66055 0.513761 -17.449 -8.23469 -30.0183 7.20833 -5.88991
0.0157 0.000713 0.00452 0.0235 0.00484 0.000875 0.0912 0.000902 0.0868
2L:10388240-10388390:minus -30.9286 3.24771 1.62385 6.41284 -26.9694 -8.68367 -18.6606 0.847222
-4.40367 0.0104 0.00317 0.00663 0.00189 0.023 0.00157 0.0165 0.0128 0.0623

2L:10388240-10388390:plus -33.7755 2.87156 1.27523 2.19266 -27.5306 -9.79592 -9.09174 1.43056 -0.440367
0.0297 0.00365 0.0075 0.0131 0.0351 0.00377 0.0026 0.0105 0.0216
2L:10396720-10396870:minus -42.7041 4.3578 -3.34862 5.06422 -9.5 -0.397959 -37.9174 3.79167
-14.2202 0.0842 0.0021 0.0318 0.00433 0.00293 0.000525 0.216 0.00437 0.361
2L:10396720-10396870:plus -23.4898 14.633 2.40367 3.41284 -18.4184 -16.9796 -16.8716 1.23611 -1.22018
0.00639 4.09e-06 0.00498 0.00756 0.0101 0.0044 0.0126 0.0112 0.0278
2L:10397100-10397250:minus -33.1122 13.4592 5.00917 2.50459 -27.3061 -19.5714 -15.1927 11.8333
5.44037 0.0271 0.000064 0.00177 0.0114 0.0315 0.0202 0.00932 0.0000364 0.00259
2L:10397100-10397250:plus -32.8163 -1.94495 2.06422 -0.807339 -18.4898 -8.72449 -20.5138 7.08333
-5.9633 0.024 0.0191 0.00564 0.0345 0.0116 0.00169 0.0225 0.000963 0.0882
2L:10403900-10404050:minus -33.2653 13.4592 0.577982 1.99083 -18.3469 -17.4286 -22.844 4.48611
7.68807 0.0277 0.000064 0.00955 0.0144 0.00894 0.00586 0.0342 0.00328 0.000841
2L:10403900-10404050:plus -22.1531 0.220183 3.22936 1.69725 -18.4592 1.16327 -19.6239 7.11111 9.61468
0.0031 0.00956 0.00363 0.0163 0.0109 0.0000831 0.0193 0.000949 0.00029
2L:10405020-10405170:minus -31.6327 3.68367 -3.69725 2.04587 -27.5306 -8.5 -21.3394 1.09722 0.183486
0.0138 0.00144 0.035 0.014 0.0351 0.00126 0.0262 0.0118 0.0176
2L:10405020-10405170:plus -31.6633 6.57798 -3.82569 3.44037 -19.0102 -17.2755 -27.7706 4.77778 -5.22018
0.0141 0.000803 0.0362 0.00746 0.0157 0.00544 0.0667 0.00289 0.0762
2L:10408220-10408370:minus -33.0714 1.88073 -2.70642 -1.3578 -27.2347 -17.3469 -5.23853 3.77778
0.66055 0.0267 0.00524 0.0265 0.0415 0.0283 0.0058 0.00106 0.0044 0.0156
2L:10408220-10408370:plus -31.8571 -2.68807 0.183486 5.90826 1.21429 -9.42857 -20.6514 -4.75 -0.486239
0.0163 0.0236 0.0109 0.00271 0.0000864 0.0027 0.0231 0.0584 0.0219
2L:10413380-10413530:minus -24.0306 6.10092 5.25688 1.61468 -26.1939 -8.72449 -16.0734 -2.08333
-1.20183 0.00723 0.00101 0.0016 0.0169 0.0175 0.00169 0.011 0.0305 0.0276
2L:10413380-10413530:plus -22.1531 9.13761 -0.293578 2.05505 -17.7857 -17.3061 -19.6881 11.8889
-1.06422 0.0031 0.000208 0.0128 0.0139 0.00676 0.00548 0.0195 0.0000344 0.0266
2L:10413880-10414030:minus -31.6633 0.87156 9.3945 6.73394 -18.449 -0.244898 -25.1376 4.40278
-1.9633 0.0141 0.00758 0.000202 0.00152 0.0107 0.000496 0.048 0.0034 0.0329
2L:10413880-10414030:plus -30.7041 0.522936 -0.917431 4.37615 -27.4592 -8.23469 -26.4404 -0.208333
-0.697248 0.00993 0.00859 0.0156 0.00504 0.0329 0.000875 0.0565 0.0178 0.0239
2L:10414500-10414650:minus -34.2959 0.0366972 0.220183 1.10092 -18.6429 -18.0102 -13.1743 8.25
-2.65138 0.0316 0.0102 0.0108 0.02 0.0121 0.00812 0.00602 0.000505 0.0406
2L:10414500-10414650:plus -33.0408 0.678899 -0.477064 2.23853 -27.2347 -8.79592 -29.422 1.19444
-1.57798 0.0264 0.00813 0.0135 0.0128 0.0283 0.0019 0.0838 0.0114 0.0299
2L:10419120-10419270:minus -41.6224 2.90826 2.6055 4.02752 1.83673 0.826531 -29.3211 3.33333
-2.53211 0.0628 0.0036 0.00462 0.00581 0.0000532 0.000153 0.0826 0.00525 0.0392
2L:10419120-10419270:plus -31 14.1284 -3.84404 3.3578 -26.9694 -18.0102 -24.6422 -1.47222 -4.66055
0.0105 7.2e-06 0.0364 0.00767 0.023 0.00812 0.045 0.0258 0.0667
2L:10422760-10422910:minus -23.2653 2.76147 -0.0642202 1.15596 -27.7551 0.0510204 -13.2294
5.44444 3.09174 0.00593 0.0038 0.0118 0.0197 0.0385 0.000349 0.00609 0.00215 0.00678
2L:10422760-10422910:plus -40.551 -1.11009 -1.11009 6.12844 -18.9388 -9.09184 -21.422 7.18056 2.16514
0.0418 0.0148 0.0165 0.00231 0.0151 0.00219 0.0266 0.000915 0.00879
2L:10423240-10423390:minus -23.2959 5.48624 0.0366972 3.19266 -18.449 -18.5714 -22.156 6.27778
1.68807 0.00605 0.00133 0.0115 0.00829 0.0107 0.0136 0.0303 0.00145 0.0109
2L:10423240-10423390:plus -41.8163 3.64286 2.84404 7.61468 -27.1531 19.051 -32.0917 0.5 -4.29358
0.0662 0.00159 0.00421 0.000779 0.0249 1.23e-06 0.119 0.0143 0.06
2L:10424020-10424310:minus -33.3367 0.422018 7.18349 10.7615 -17.7143 -18.2755 -19.3211 4.40278
-0.229358 0.0281 0.0089 0.000665 0.0000826 0.00627 0.0112 0.0184 0.0034 0.0202
2L:10424020-10424310:plus -31.8163 1.97248 -0.348624 0.495413 -8.60204 -27.602 -17.2661 -1.93056
2.11009 0.016 0.00507 0.013 0.0236 0.00125 0.0416 0.0134 0.0293 0.00898
2L:10426580-10426730:minus -23.1429 9.62385 3.97248 2.66972 -18.1837 -7.45918 -8.6789 3.97222
1.07339 0.00533 0.000161 0.00271 0.0106 0.00835 0.000582 0.00241 0.00407 0.014
2L:10426580-10426730:plus -24.2245 14.4128 0.495413 5.94495 -17.4184 -9.45918 -24.0183 1.125 -0.605505
0.00763 5.49e-06 0.00983 0.00261 0.00457 0.00279 0.0412 0.0117 0.023

2L:10426820-10426970:minus -21.9286 7.95413 3.97248 3.93578 -18.1837 -18.0102 -12.9725 3.18056
1.74312 0.0027 0.000392 0.00271 0.00597 0.00835 0.00812 0.00574 0.00557 0.0105
2L:10426820-10426970:plus -23.1837 -1.22936 -1.37615 5.94495 -18.1531 -9.45918 -18.4312 -1.26389 3.30275
0.00553 0.0154 0.0179 0.00261 0.00829 0.00279 0.016 0.0243 0.00619
2L:10428700-10428850:minus -34.2245 0.743119 1.55963 -1.83486 -17.4898 -10.0918 -18.4404 4.58333
-3.97248 0.0315 0.00794 0.00678 0.0484 0.0053 0.00404 0.016 0.00315 0.0553
2L:10428700-10428850:plus -41.8163 -0.0642202 -0.40367 -0.284404 1.14286 -8.42857 -20.4495 1.58333
1.0367 0.0662 0.0105 0.0132 0.0291 0.000113 0.00104 0.0223 0.00997 0.0141
2L:10430940-10431090:minus -32.7755 1.51376 1.80734 -0.40367 -8.64286 -27.051 -15.2294 -1.90278
0.0183486 0.0234 0.006 0.00621 0.0302 0.00139 0.0315 0.00939 0.029 0.0183
2L:10430940-10431090:plus -31.9694 2.25688 4.26606 -1.48624 -18.5306 -9.02041 -24.1284 1.59722 3.46789
0.0168 0.00457 0.00241 0.0431 0.0118 0.00213 0.0418 0.00993 0.00582
2L:10435740-10435890:minus -30.102 -0.229358 3.33028 4.11927 -17.4184 -18.2755 -15.2385 -2.20833
2.26606 0.00859 0.0111 0.00349 0.00551 0.00457 0.0112 0.00941 0.0315 0.00847
2L:10435740-10435890:plus -22.3776 15.7706 0.587156 5.59633 -18.6429 -9.72449 -27.6422 3.25 1.2844
0.00342 4.56e-07 0.00952 0.00331 0.0121 0.00352 0.0656 0.00542 0.0129
2L:10437500-10437650:minus -13.3673 7.69725 4.78899 4.65138 0.581633 -8.57143 -9.76147 1.61111
-4.25688 0.000813 0.00045 0.00194 0.00471 0.000292 0.00149 0.00294 0.00988 0.0593
2L:10437500-10437650:plus -31.7041 10.211 1.52294 4.37615 -8.93878 -17.2755 -11.367 4.36111 -4.70642
0.0146 0.000121 0.00687 0.00504 0.00216 0.00544 0.00403 0.00346 0.0676
2L:10439260-10439410:minus -23.0714 13.4592 4.05505 0.522936 0.357143 -8.94898 -14.9358 -2.86111
5.17431 0.0052 0.000064 0.00262 0.0234 0.000324 0.00203 0.00886 0.0373 0.00296
2L:10439260-10439410:plus -41.7857 14.7523 9.76147 5.31193 -18.7551 -18.051 -24.3394 1.51389 -2.22936
0.0653 3.48e-06 0.000163 0.00393 0.0144 0.00886 0.0431 0.0102 0.0356
2L:10442480-10442630:minus -32 1.83486 5.34862 1.6789 -36.0102 -17.0816 -25.1284 8.47222 3.09174
0.0171 0.00533 0.00154 0.0165 0.072 0.00487 0.048 0.000443 0.00678
2L:10442480-10442630:plus -23.1122 0.174312 0.651376 9.7156 -18.4184 -18.0102 -16.156 2.83333 2.88073
0.0053 0.00971 0.00932 0.000242 0.0101 0.00812 0.0111 0.00635 0.00727
2L:10457520-10457670:minus -31.898 3.97959 1.33028 1.20183 -18.7551 -8.79592 -28.9083 3.94444
-0.816514 0.0164 0.000669 0.00736 0.0195 0.0144 0.0019 0.0779 0.00411 0.0249
2L:10457520-10457670:plus 6.14286 3.47706 1.16514 7.34862 -26.9286 -9.27551 -9.21101 -4.54167 5.58716
5.85e-06 0.00292 0.0078 0.000998 0.0214 0.00244 0.00266 0.0557 0.00237
2L:10468560-10468710:minus -31.1429 1.87156 4.92661 6.31193 -17.7143 -19.3061 -22.5229 0.180556
3.88073 0.0108 0.00526 0.00183 0.00199 0.00627 0.0187 0.0323 0.0158 0.00475
2L:10468560-10468710:plus -32.1122 1.10092 0.46789 5.33028 -17.602 -8.79592 -8.61468 4.16667 1.34862
0.0178 0.00698 0.00992 0.00389 0.00547 0.0019 0.00238 0.00375 0.0125
2L:10483240-10483390:minus -40.2857 -1.54128 -0.706422 0.559633 -19.051 -18.8571 -22.7064 4.48611
-1.98165 0.0385 0.0169 0.0146 0.0232 0.0162 0.0151 0.0334 0.00328 0.033
2L:10483240-10483390:plus -31.398 13.6422 6.00917 3.29358 -26.9286 -9.20408 -13.0275 5.01389 2.41284
0.0117 0.0000127 0.00115 0.00791 0.0214 0.00225 0.00582 0.00261 0.0082
2L:10488700-10488850:minus -22.8878 13.4592 2.22018 6.12844 -27.2653 -9.53061 -11.9358 -0.527778
-0.192661 0.00441 0.000064 0.00532 0.00231 0.0302 0.0031 0.00455 0.0196 0.0198
2L:10488700-10488850:plus -33.6327 5.36697 14.3119 2.66972 -7.33673 -8.86735 -4.76147 6.29167 -1.56881
0.029 0.0014 4.82e-06 0.0106 0.000388 0.00198 0.000927 0.00144 0.0298
2L:10499320-10499470:minus -21.5816 0.183486 4.48624 7.80734 -18.6429 -18.0816 -3.07339 10.75
1.90826 0.00205 0.00968 0.0022 0.00063 0.0121 0.00934 0.000579 0.0000934 0.00974
2L:10499320-10499470:plus -23.7041 -0.394495 5.25688 6.29358 -27.6327 -9.45918 -26.9174 5.66667
1.00917 0.00669 0.0118 0.0016 0.00202 0.0369 0.00279 0.0599 0.00194 0.0143
2L:10500420-10500570:minus -23.0306 1.7156 2.74312 2.68807 -28.0102 -19.5714 -12.5138 2.76389
5.95413 0.00505 0.00557 0.00438 0.0104 0.0449 0.0202 0.00517 0.00652 0.00192
2L:10500420-10500570:plus -23.449 4.12844 -0.697248 4.0367 -17.9796 -17.3469 -22.0917 1.375
-6.89908 0.00631 0.00229 0.0145 0.00578 0.00726 0.0058 0.03 0.0107 0.111
2L:10503780-10503930:minus -22.6633 -2.12844 9.11009 4.07339 -18.1122 0.122449 0.0642202 -1.02778
1.95413 0.00384 0.0202 0.000238 0.00562 0.00771 0.000312 0.000279 0.0227 0.00964

2L:10503780-10503930:plus -23.1837 -0.577982 1.68807 9.59633 -18.4898 1.05102 -13.9817 -0.180556
-4.06422 0.00553 0.0125 0.00648 0.000275 0.0116 0.000107 0.00722 0.0177 0.0565
2L:10506760-10506910:minus 5.46939 1.63303 1.42202 6.29358 -8.26531 -18.0102 -19.2661 0.0694444
2.11009 9.74e-06 0.00575 0.00712 0.00202 0.000934 0.00812 0.0182 0.0164 0.00898
2L:10506760-10506910:plus -21.6939 4.05102 15.4495 6.27523 20.4694 0.0510204 -7.27523 -1.40278
-3.82569 0.00226 0.000335 9.17e-07 0.00206 1.65e-07 0.000349 0.0018 0.0253 0.0531
2L:10516560-10516710:minus -2.07143 5.3578 5.6055 6.3578 -17.0816 0.387755 0.944954 -1.875
4.09174 0.0000251 0.0014 0.00137 0.00195 0.00334 0.000217 0.000224 0.0288 0.00416
2L:10516560-10516710:plus -12.2245 0.926606 6.54128 7.37615 -9.16327 -8.57143 -16.7064 1.56944 8.88073
0.000376 0.00743 0.000901 0.000951 0.00249 0.00149 0.0122 0.01 0.000468
2L:10517120-10517270:minus -32.0714 5.09174 5.22018 3.61468 -27.3061 -27.3367 -8.58716 2.09722
5.49541 0.0176 0.00157 0.00162 0.0068 0.0315 0.0347 0.00236 0.00832 0.0025
2L:10517120-10517270:plus -31.7347 9.6789 13.6789 7.29358 -17.4184 -0.173469 -15.4954 1.31944
-0.651376 0.0149 0.000157 0.0000109 0.00104 0.00457 0.000456 0.00989 0.0109 0.0235
2L:10529220-10529370:minus -21.7653 -2.56881 3.6055 1.25688 -17.4184 -18.051 -12.2936 -0.666667
-2.6055 0.00237 0.0229 0.00314 0.0192 0.00457 0.00886 0.00492 0.0205 0.04
2L:10529220-10529370:plus -13.1429 0.743119 6.3211 9.24771 -8.96939 -16.7551 10.7431 -3.33333 -0.87156
0.00065 0.00794 0.000999 0.000409 0.00226 0.0042 9.79e-06 0.042 0.0252
2L:10728880-10729030:minus -31.3571 10.1193 4.86239 2.2844 -27.5612 -18.5714 -8.58716 -0.638889
-0.100917 0.0114 0.000126 0.00188 0.0124 0.0354 0.0136 0.00236 0.0203 0.019
2L:10728880-10729030:plus -41.2551 0.550459 12.9725 7.90826 -18.4592 0.122449 -19.5138 8.63889 0.183486
0.0513 0.00851 0.0000215 0.000611 0.0109 0.000312 0.019 0.000401 0.0176
2L:10732380-10732530:minus -31.8469 7.05505 6.44037 6.72477 -7.89796 -27.602 -16.0642 6.01389
-0.605505 0.0162 0.00063 0.000945 0.00154 0.000678 0.0416 0.011 0.00165 0.023
2L:10732380-10732530:plus -13.7755 14.5046 -0.348624 1.78899 -16.4184 0.244898 -12.7798 7.15278
-3.06422 0.00107 4.87e-06 0.013 0.0158 0.00304 0.000285 0.0055 0.000928 0.0453
2L:10737240-10737390:minus -40.102 1.3945 2.98165 2.29358 -19.3163 -9.57143 -15.5046 4.69444
-3.81651 0.036 0.00627 0.004 0.0124 0.0165 0.00326 0.0099 0.003 0.0529
2L:10737240-10737390:plus -23.2245 0.761468 0.394495 3.84404 -18.1531 -8.94898 -10.1193 1.04167 0.0733945
0.00576 0.00789 0.0102 0.00624 0.00829 0.00203 0.00315 0.012 0.0181
2L:10743900-10744050:minus -32.8061 1.25688 14.3119 3.29358 -17.7551 -18.0816 -1.57798 -2.875
-0.302752 0.0237 0.00659 4.82e-06 0.00791 0.00658 0.00934 0.000403 0.0375 0.0208
2L:10743900-10744050:plus -21.1531 7.65138 3.40367 3.7156 -18.6735 -16.9796 -16.633 4.70833 -8.47706
0.00188 0.00046 0.0034 0.00655 0.0123 0.0044 0.0121 0.00298 0.15
2L:10746220-10746370:minus -22.0306 0.293578 -1.87156 8.98165 -9.0102 -18.051 -15.422 0.0416667
4.14679 0.00293 0.00932 0.0208 0.000461 0.00236 0.00886 0.00975 0.0165 0.00406
2L:10746220-10746370:plus -31.7347 0.678899 2.43119 3.17431 -18.4898 -17.9796 -17.0275 1.86111 1.86239
0.0149 0.00813 0.00493 0.00842 0.0116 0.00764 0.0129 0.00905 0.00994
2L:10767880-10768030:minus -23 6.6789 3.44037 2.89908 -27.3061 -27.7551 -6.01835 4.33333 3.24771
0.00498 0.000764 0.00335 0.00938 0.0315 0.0458 0.00132 0.0035 0.00636
2L:10767880-10768030:plus -31.4796 13.6239 0.93578 3.05505 -18.1939 -18.2041 -13.2294 5.68056 5.22936
0.0128 0.000013 0.00845 0.00878 0.0086 0.0103 0.00609 0.00193 0.00288
2L:10769920-10770070:minus -30.3673 3.00917 -6.22936 3.77064 -17.3776 9.37755 -23.0367 -0.930556
-5.59633 0.00897 0.00347 0.0669 0.00637 0.00419 0.0000363 0.0353 0.0221 0.0819
2L:10769920-10770070:plus -22.1837 8.51376 0.247706 -0.284404 -18.3469 -18.2041 -17.0459 4.98611
5.13761 0.00315 0.000289 0.0107 0.0291 0.00894 0.0103 0.0129 0.00264 0.00298
2L:1077180-1077330:minus -31.7755 0.752294 2.11009 4.13761 -27.3367 -27.898 -18.4862 2.61111 1.57798
0.0157 0.00791 0.00554 0.00546 0.0317 0.0537 0.0161 0.00691 0.0114
2L:1077180-1077330:plus -24.2653 -0.192661 5.97248 3.6422 -27 -9.42857 -14.0367 2.76389 6.17431
0.00773 0.011 0.00117 0.00669 0.0233 0.0027 0.00731 0.00652 0.00171
2L:1077680-1077830:minus -30.7347 -4.99083 -0.174312 4.50459 11.6939 0.204082 3.97248 -1.58333
1.69725 0.01 0.044 0.0123 0.00484 1.17e-06 0.000296 0.0000833 0.0266 0.0108
2L:1077680-1077830:plus -24.2959 13.3878 0.788991 1.78899 1.14286 -19.5 -12.8624 2.52778 -3.77064
0.00781 0.0000869 0.00889 0.0158 0.000113 0.0199 0.0056 0.00712 0.0524

2L:107960-108110:minus -31.6633 4.77982 3.00917 5.08257 -8.86735 -18.7857 -22.0459 -1.22222 -0.880734
0.0141 0.00178 0.00396 0.00429 0.00173 0.0146 0.0297 0.024 0.0252
2L:107960-108110:plus -30.6327 2.66972 1.74312 3.56881 -26.5918 -9.38776 -11.0183 6.93056 2.83486
0.00968 0.00393 0.00635 0.00699 0.0186 0.00266 0.00375 0.00104 0.00739
2L:1080900-1081050:minus -30.8776 5.42202 -4.6789 2.27523 -9.20408 -0.397959 -23.2385 2.25 -0.40367
0.0103 0.00137 0.0454 0.0125 0.00262 0.000525 0.0365 0.00788 0.0213
2L:1080900-1081050:plus -31.6633 0.862385 6.33945 10.5963 10.3571 9.86735 -22.0642 0.305556 3.46789
0.0141 0.00761 0.000991 0.0001 0.0000208 0.0000132 0.0298 0.0152 0.00582
2L:10842480-10842630:minus -32.2653 10.9266 8.46789 3.97248 -18.4184 -16.9388 -19.2202 6.55556
0.238532 0.0184 0.0000837 0.00034 0.00587 0.0101 0.00433 0.0181 0.00126 0.0173
2L:10842480-10842630:plus -13.8469 10.5321 -2.68807 2.51376 -17.4184 -17.5714 -16.7615 8.95833 4.04587
0.0011 0.000102 0.0264 0.0113 0.00457 0.00616 0.0123 0.000329 0.00421
2L:10850320-10850470:minus -13.5204 8.31193 0.0275229 5.86239 -27.2653 -18.0102 -11.3578 -2.31944
6.37615 0.00097 0.000323 0.0115 0.00282 0.0302 0.00812 0.00402 0.0325 0.00159
2L:10850320-10850470:plus -22.4388 -2.51376 9.55963 3.89908 -18.6837 -25.602 -12.3394 -2.77778 -0.293578
0.00348 0.0225 0.000184 0.00604 0.0126 0.0207 0.00497 0.0365 0.0207
2L:10850760-10850910:minus -30.6735 -5.19266 -3.94495 1.7156 10.0918 -9.5 -24.0917 -0.402778
-1.6422 0.00978 0.0464 0.0374 0.0162 0.0000251 0.00294 0.0416 0.0189 0.0304
2L:10850760-10850910:plus -32.9898 -2.9633 0.0550459 1.74312 0.908163 -19.0102 -16.3761 2.52778
-3.91743 0.0254 0.0255 0.0114 0.016 0.000153 0.0159 0.0116 0.00712 0.0544
2L:10858040-10858190:minus -21.9286 4.11009 0.697248 4.21101 20.1735 -27.6327 2.42202 3.68056
3.81651 0.0027 0.00231 0.00917 0.00532 5.68e-07 0.043 0.000144 0.00457 0.00482
2L:10858040-10858190:plus -24.2959 3.90816 -0.954128 6.27523 -8.93878 0.0204082 -15.5688 4.125
1.44037 0.00781 0.00102 0.0157 0.00206 0.00216 0.000377 0.01 0.00382 0.012
2L:10858420-10858570:minus -23.4898 0.40367 4.88073 2.17431 -9.19388 -10.0918 -16.844 1.30556
2.80734 0.00639 0.00896 0.00187 0.0132 0.00253 0.00404 0.0125 0.011 0.00742
2L:10858420-10858570:plus -32.398 2.22018 -0.990826 5.30275 1.87755 0.316327 -15.3028 -1.41667
3.45872 0.019 0.00463 0.0159 0.00396 0.0000492 0.000243 0.00953 0.0254 0.00591
2L:109260-109410:minus -21.9286 4.84404 1.25688 5.89908 -17.449 -18.0816 -7.57798 6.69444 5.49541
0.0027 0.00174 0.00755 0.00272 0.00484 0.00934 0.00192 0.00118 0.0025
2L:109260-109410:plus -22.9286 -3.3945 2.46789 6.84404 -18.4184 -18.3469 -7.44037 -6.68056 -0.577982
0.0046 0.0287 0.00486 0.0014 0.0101 0.0124 0.00187 0.0871 0.0227
2L:10967880-10968030:minus -24.3367 1.61468 4.98165 3.97248 -27.6327 -17.7857 -17.8991 -0.416667
8.05505 0.00789 0.00578 0.00179 0.00587 0.0369 0.0068 0.0147 0.019 0.00064
2L:10967880-10968030:plus -30.7347 -3.2844 1.9633 3.08257 -26.5306 -18.3469 -20.789 -1.84722 1.02752
0.01 0.0279 0.00586 0.0087 0.0185 0.0124 0.0237 0.0286 0.0141
2L:10970240-10970390:minus -12.5204 5.27523 1.59633 4.40367 1.87755 -9.0102 -14.8991 2.16667
5.23853 0.000441 0.00145 0.00669 0.00497 0.0000492 0.00208 0.00879 0.00812 0.00285
2L:10970240-10970390:plus -40.2143 0.899083 1.41284 -1.52294 20.2041 -9.02041 -12.2936 4.15278 0.917431
0.0372 0.00751 0.00714 0.0437 3.64e-07 0.00213 0.00492 0.00377 0.0146
2L:10972000-10972150:minus -23.2245 5.44954 0.66055 3.33945 -27.1224 -10.0204 -23.9541 1.84722
2.19266 0.00576 0.00135 0.00929 0.00774 0.0245 0.00399 0.0408 0.0091 0.0087
2L:10972000-10972150:plus -31.3265 -1.77982 -1.65138 6.54128 -18.3776 -8.79592 -20.7431 7.04167 3.51376
0.0114 0.0182 0.0195 0.00173 0.00926 0.0019 0.0235 0.000984 0.00566
2L:10972380-10972530:minus -32.5918 -1.95413 3.25688 7.27523 -16.1531 -19.3469 -16.3486 1.875
1.33028 0.021 0.0192 0.0036 0.00109 0.00297 0.0194 0.0115 0.00901 0.0127
2L:10972380-10972530:plus -22.8061 -3.06422 -2.04587 3.27523 -17.7143 -26.602 -13.5138 -0.638889
5.17431 0.00414 0.0262 0.0219 0.00796 0.00627 0.0248 0.0065 0.0203 0.00296
2L:10975120-10975270:minus -21.5816 -4.36697 2.83486 6.54128 -18.4592 -27.3367 -19.5229 0.680556
0.440367 0.00205 0.0373 0.00423 0.00173 0.0109 0.0347 0.019 0.0135 0.0164
2L:10975120-10975270:plus -21.7041 0.146789 3.81651 3.04587 -17.1939 -27.3061 -18.6055 -3.38889 -1.05505
0.00231 0.0098 0.00289 0.00883 0.00377 0.0336 0.0164 0.0426 0.0264
2L:10976320-10976470:minus -34.0714 9.6789 0.431193 2.98165 -8.64286 -8.53061 -15.3945 0.597222
-4.3578 0.031 0.000157 0.01 0.00905 0.00139 0.00136 0.0097 0.0139 0.0614

2L:10976320-10976470:plus -23 10.9817 -1.07339 4.20183 -19.051 -8.53061 -21.7156 6.81944 0.816514
0.00498 0.0000816 0.0163 0.00533 0.0162 0.00136 0.028 0.0011 0.015
2L:10977100-10977250:minus -31.4796 0.394495 3.76147 2.80734 -26.6633 -18.9796 -17.2569 -1.41667
0.394495 0.0128 0.00899 0.00295 0.00985 0.0189 0.0155 0.0133 0.0254 0.0166
2L:10977100-10977250:plus -21.551 -1.7156 2.55046 3.09174 -17.4184 -7.57143 -18.7156 0.708333 2.73394
0.00205 0.0179 0.00471 0.00867 0.00457 0.000644 0.0167 0.0134 0.00756
2L:10986200-10986350:minus -31.7347 1.11927 0.550459 6.12844 -26.9286 -18.3061 -22.0917 3.06944
5.6055 0.0149 0.00693 0.00965 0.00231 0.0214 0.0114 0.03 0.00581 0.00232
2L:10986200-10986350:plus -22.9286 0.633028 2.27523 3.79817 -27.2653 -17.3469 -23.6239 2.125 11.8532
0.0046 0.00826 0.00522 0.00629 0.0302 0.0058 0.0388 0.00824 0.0000428
2L:10987720-10987870:minus -23.0306 4.58716 -3.11927 0.385321 -27.5306 -17.3469 -12.9174 5.65278
5.3945 0.00505 0.00192 0.0298 0.0243 0.0351 0.0058 0.00567 0.00195 0.00263
2L:10987720-10987870:plus -34.0714 -2.61468 -0.0917431 -1.98165 -8.33673 -8.5 -21.6422 4.15278 -0.486239
0.031 0.0232 0.0119 0.051 0.00101 0.00126 0.0276 0.00377 0.0219
2L:10989320-10989470:minus -22.2959 0.908257 -3.75229 4.75229 -8.89796 -18.2755 -18.4128 3.61111
3.55963 0.00335 0.00748 0.0355 0.00461 0.00189 0.0112 0.0159 0.0047 0.00545
2L:10989320-10989470:plus -24.2959 2.80734 6.6789 5.93578 0.94898 -0.173469 -5.77982 4.5 -2.05505
0.00781 0.00373 0.000844 0.00264 0.000134 0.000456 0.00124 0.00326 0.0338
2L:11004060-11004210:minus -22.8878 -3.3578 -2.81651 3.22936 -8.96939 -8.53061 -15 1.18056 -2.98165
0.00441 0.0284 0.0274 0.00816 0.00226 0.00136 0.00897 0.0114 0.0445
2L:11004060-11004210:plus -12.8163 1.09174 2.0367 3.70642 -18.051 -8.38776 -20.7706 -1.80556 -2.69725
0.000489 0.007 0.0057 0.0066 0.00746 0.00101 0.0236 0.0283 0.0411
2L:11004740-11004890:minus -24.4184 8.19266 -4.11927 4.27523 -17.9388 19.0816 -27.4037 4.875
-0.651376 0.00797 0.000343 0.0392 0.00518 0.00715 1.12e-06 0.0636 0.00277 0.0235
2L:11004740-11004890:plus -31.5918 0.587156 1.66055 11.0275 1.61224 -8.34694 -21.5046 -1.625 7.01835
0.0136 0.0084 0.00654 0.0000401 0.0000631 0.000993 0.027 0.0269 0.00134
2L:11011080-11011230:minus -32.9286 0.651376 0.486239 2.85321 -27.2245 -18.2755 -23.6147 3.77778
-8.04587 0.025 0.00821 0.00986 0.00955 0.0272 0.0112 0.0387 0.0044 0.137
2L:11011080-11011230:plus -23.1122 -1.76147 -0.944954 5.98165 -8.93878 9.53061 -21.7615 1.25 1.17431
0.0053 0.0181 0.0157 0.00258 0.00216 0.0000316 0.0282 0.0112 0.0134
2L:11057380-11057530:minus -4.36735 -0.724771 -1.55046 7.73394 -18.3776 -8.53061 -3.6422 -0.472222
8.95413 0.000143 0.0131 0.0189 0.000669 0.00926 0.00136 0.000675 0.0193 0.000458
2L:11057380-11057530:plus -30.7041 -2.12844 -1.3211 0.917431 -27.8571 -36.449 1.69725 0.75 10.3394
0.00993 0.0202 0.0176 0.021 0.039 0.109 0.000181 0.0132 0.000112
2L:11066620-11066770:minus -32.551 -3.18349 7.00917 2.26606 -26.1531 -26.7551 -16.3853 3.51389
7.18349 0.0207 0.0271 0.000725 0.0126 0.0172 0.0263 0.0116 0.00489 0.00121
2L:11066620-11066770:plus -21.5918 7.51376 -0.293578 7.90826 -8.89796 -17.7143 -9.43119 0.236111
-3.20183 0.00209 0.000495 0.0128 0.000611 0.00189 0.00634 0.00277 0.0155 0.0467
2L:11067000-11067150:minus -12.8163 4.80734 2.21101 4.46789 -19.0918 -17.2755 -14.0826 2.76389
2.11009 0.000489 0.00176 0.00534 0.0049 0.0163 0.00544 0.00739 0.00652 0.00898
2L:11067000-11067150:plus -22.7755 1.91743 5.13761 6.29358 -27.5714 -0.244898 -12.8532 -0.111111
10.3486 0.00412 0.00518 0.00168 0.00202 0.0357 0.000496 0.00559 0.0173 0.0000988
2L:1108180-1108330:minus -22.6224 2.11927 10.2477 3.14679 -17.1837 -17.051 -8.45872 1.05556 -0.119266
0.00371 0.00481 0.000123 0.00847 0.00366 0.00479 0.00231 0.0119 0.0192
2L:1108180-1108330:plus -3.07143 2.66055 10.0183 7.21101 -27 -17.9796 -5.75229 3.94444 -1.21101
0.0000441 0.00394 0.00014 0.0011 0.0233 0.00764 0.00123 0.00411 0.0277
2L:11091320-11091470:minus -41.4796 13.4592 1.6055 2.05505 -17.7143 -18.0816 -13.3028 7 1.85321
0.0577 0.000064 0.00667 0.0139 0.00627 0.00934 0.0062 0.00101 0.01
2L:11091320-11091470:plus -22.9286 3.82569 3.78899 9.59633 -18.4898 -8.45918 -20.6972 0.472222 -2.51376
0.0046 0.00257 0.00292 0.000275 0.0116 0.00111 0.0233 0.0144 0.039
2L:11094140-11094290:minus -21.6939 0.899083 1.01835 1.48624 -9.23469 -27.2653 -13.1009 4.19444
-1.59633 0.00226 0.00751 0.00821 0.0177 0.00277 0.0333 0.00592 0.00371 0.0301
2L:11094140-11094290:plus -21.6224 0.302752 2.9633 2 -17.449 -27.4082 -6.01835 -2.16667 -1.21101
0.00213 0.00929 0.00403 0.0143 0.00484 0.0356 0.00132 0.0312 0.0277

2L:11100740-11100890:minus	-23.8878	4.97959	1.10092	3.94495	-18.3776	-17.3469	-21.5229	0.375
0.568807	0.00692	0.000156	0.00798	0.00595	0.00926	0.0058	0.027	0.0149	0.016
2L:11100740-11100890:plus	-23.2653	11.4954	0.412844	4.04587	-17.9286	-27.3367	-11.4312	-2.88889	6.02752
0.00593	0.0000618	0.0101	0.00572	0.0071	0.0347	0.00408	0.0376	0.00183
2L:11106660-11106810:minus	-30.1735	15.3119	11.7615	3.42202	-8.40816	-28.9286	-7.24771	-0.319444
5.70642	0.00869	1.47e-06	0.0000506	0.00751	0.00108	0.0717	0.00179	0.0184	0.00222
2L:11106660-11106810:plus	-14.2653	1.90826	0.266055	2	-8.60204	-9.72449	-24.3486	-8.16667	-2.00917
0.00128	0.00519	0.0106	0.0143	0.00125	0.00352	0.0432	0.114	0.0333
2L:11107040-11107190:minus	-32.0714	3.01835	-1.2844	3.87156	-26.5306	-16.7143	-3.88991	6.01389
1.69725	0.0176	0.00346	0.0174	0.00618	0.0185	0.00419	0.000723	0.00165	0.0108
2L:11107040-11107190:plus	-21.7347	0.348624	7.83486	2.73394	-7.97959	-18.7857	-1.36697	-0.513889
3.92661	0.00235	0.00914	0.000478	0.0101	0.000799	0.0146	0.000385	0.0196	0.00461
2L:11120280-11120430:minus	-12.8469	14.9908	13.3761	2.84404	-17.9388	-0.204082	-15.5046	1.93056
1.22936	0.000502	2.1e-06	0.0000145	0.00961	0.00715	0.000463	0.0099	0.00883	0.0132
2L:11120280-11120430:plus	-31.0408	6.43119	-0.247706	5.57798	-17.7449	-8.72449	-21.6881	1.77778
-3.98165	0.0106	0.000862	0.0126	0.0034	0.0063	0.00169	0.0279	0.00932	0.0555
2L:11123560-11123710:minus	-22.8878	4.91743	3.84404	2.98165	20.2041	-9.57143	-21.9541	3.55556
-0.12844	0.00441	0.00169	0.00286	0.00905	3.64e-07	0.00326	0.0292	0.00481	0.0193
2L:11123560-11123710:plus	-30.949	-1.29358	4.0367	1.77982	-18.1531	-9.53061	-17.055	-1.30556	0.733945
0.0104	0.0157	0.00264	0.0158	0.00829	0.0031	0.0129	0.0246	0.0153
2L:11130640-11130790:minus	-24.5204	3.97959	3.97248	5.57798	-18.5204	-8.5	-24.4495	5.02778	3.98165
0.00813	0.000669	0.00271	0.0034	0.0117	0.00126	0.0438	0.00259	0.00439
2L:11130640-11130790:plus	-31.6735	2.6789	5.36697	9.33028	-18.4592	1.09184	-23	6.19444	3	0.0141
0.00391	0.00152	0.000374	0.0109	0.000091	0.0351	0.00151	0.00702
2L:11155720-11155870:minus	-22.9592	1.44954	-1.38532	9.44037	-27.2347	-8.68367	-13.9725	-4.44444
-1.07339	0.00475	0.00615	0.018	0.000339	0.0283	0.00157	0.00721	0.0545	0.0267
2L:11155720-11155870:plus	-30.7755	-4.29358	2.6422	0.651376	-27.9286	-8.65306	-7.89908	-1.45833	11.8073
0.0102	0.0366	0.00455	0.0226	0.0412	0.00156	0.00206	0.0257	0.0000538
2L:1117320-1117470:minus	-31.6224	-0.541284	1	5.3945	-8.96939	-19.051	-26.7523	-2.80556	-2.75229
0.0137	0.0124	0.00827	0.00379	0.00226	0.0165	0.0587	0.0368	0.0418
2L:1117320-1117470:plus	-32.1429	-0.623853	5.20183	3.31193	-18.4592	-8.30612	-23.156	0.125
4.18349	0.0179	0.0127	0.00163	0.00786	0.0109	0.000973	0.036	0.0161	0.00404
2L:11178920-11179070:minus	-32.9592	-0.568807	-2.74312	3.05505	-19.3163	-7.94898	-17.6422	5.56944
-3.47706	0.0252	0.0125	0.0268	0.00878	0.0165	0.00074	0.0142	0.00203	0.0493
2L:11178920-11179070:plus	-12.1122	-2.16514	0.293578	4.11927	1.58163	-17.051	-13.9266	4.88889	1.54128
0.000336	0.0204	0.0105	0.00551	0.0000662	0.00479	0.00714	0.00276	0.0115
2L:1118280-1118430:minus	-22.4184	2.74312	1.18349	6.27523	-9.23469	9.60204	-11.4495	-0.0277778
4.21101	0.00345	0.00382	0.00775	0.00206	0.00277	0.0000248	0.00409	0.0169	0.00396
2L:1118280-1118430:plus	-32.1837	0.477064	4.76147	10.8624	-18.7551	-17.7143	-14.8716	-1.05556	5.18349
0.0181	0.00873	0.00196	0.0000687	0.0144	0.00634	0.00874	0.0229	0.00295
2L:1119020-1119170:minus	-11.8469	-3.16514	1.41284	7.15596	-18.1531	-19.3061	-7.70642	14.2083	3.6055
0.000279	0.027	0.00714	0.00117	0.00829	0.0187	0.00198	1.9e-06	0.00532
2L:1119020-1119170:plus	-31.8776	-1.12844	-1.40367	2.6422	-26.9592	-44.6633	-6.87156	0.875	-0.972477
0.0163	0.0149	0.0181	0.0107	0.0215	0.202	0.00164	0.0127	0.0259
2L:11260-11410:minus	-31.9184	3.2844	-2.51376	0.0366972	-7.89796	-27.9286	-12.1927	4.47222	3.78899
0.0164	0.00313	0.0251	0.0266	0.000678	0.054	0.00481	0.0033	0.00487
2L:11260-11410:plus	-31.9286	6.50459	1.02752	2.3578	-27.1224	-8.42857	-11.6697	0.861111	0.394495
0.0166	0.000833	0.00819	0.0121	0.0245	0.00104	0.00429	0.0127	0.0166
2L:11262760-11262910:minus	-23.8163	-6.68807	4.85321	-0.724771	-17.2245	-26.6735	-14.6789	-0.597222
1.08257	0.00682	0.0677	0.00189	0.0338	0.00384	0.0256	0.0084	0.0201	0.0139
2L:11262760-11262910:plus	-23.2959	-5.55046	10.8899	5.73394	-18.3776	-9.02041	-18.6147	-2.91667	1.07339
0.00605	0.0509	0.0000856	0.00307	0.00926	0.00213	0.0164	0.0378	0.014
2L:11268960-11269110:minus	-24.0306	6.10092	4.27523	3.18349	-18.9796	-17.7857	-14.1835	2.98611
2.88073	0.00723	0.00101	0.0024	0.00837	0.0155	0.0068	0.00755	0.006	0.00727

2L:11268960-11269110:plus -22.9592 9.77982 -0.431193 6.21101 -27 -9.86735 -8.62385 5.29167 3.14679
0.00475 0.000149 0.0133 0.00213 0.0233 0.00391 0.00238 0.0023 0.00666
2L:11269600-11269750:minus -41.4796 5.61468 -0.00917431 -2.57798 -18.5204 -8.02041 -25.5321 3.41667
1.90826 0.0577 0.00126 0.0116 0.0614 0.0117 0.000761 0.0505 0.00508 0.00974
2L:11269600-11269750:plus -14.5612 4.15596 -1.07339 3.20183 0.581633 -8.23469 -10.6881 1.875 7.68807
0.00147 0.00227 0.0163 0.00824 0.000292 0.000875 0.00351 0.00901 0.000841
2L:11277940-11278090:minus -13.4082 13.5306 -1.88073 4.3211 -27.5612 -17.2755 -17.7615 0.888889
2.38532 0.000867 0.0000206 0.0209 0.00514 0.0354 0.00544 0.0144 0.0126 0.00822
2L:11277940-11278090:plus -39.1837 -0.954128 2.44037 2.54128 -18.1939 -18.1224 -7.22936 -1.52778
-1.94495 0.0328 0.0141 0.00491 0.0112 0.0086 0.00988 0.00178 0.0262 0.0327
2L:11280220-11280370:minus -31.6327 0.733945 4.27523 4.22018 -17.7551 -9.82653 -19.8349 4.66667
-0.623853 0.0138 0.00797 0.0024 0.00531 0.00658 0.00381 0.02 0.00304 0.0232
2L:11280220-11280370:plus -31.6224 3.68367 5.66972 3.44954 -18.5612 -9.53061 -9.9633 2.91667 9.88073
0.0137 0.00144 0.00134 0.00738 0.0119 0.0031 0.00306 0.00616 0.000206
2L:11282060-11282210:minus -30.4388 0.183486 1.76147 3.95413 -17.6429 -0.204082 -26.1743 4.97222
-7.22018 0.00923 0.00968 0.00631 0.00593 0.00562 0.000463 0.0546 0.00266 0.118
2L:11282060-11282210:plus -42.0408 13.3878 -0.192661 3.30275 -18.1224 -17.7857 -28.055 3.30556
3.14679 0.069 0.0000869 0.0123 0.00789 0.00782 0.0068 0.0693 0.0053 0.00666
2L:1128680-1128830:minus -41.4796 6.37615 2.20183 1.46789 -17.4082 -25.8265 -24.4862 1 2.11927
0.0577 0.000885 0.00536 0.0179 0.00421 0.0212 0.044 0.0121 0.00889
2L:1128680-1128830:plus -13.5612 0.504587 -0.238532 3.88991 -8.86735 -7.72449 -23.633 -0.222222
-4.79817 0.000984 0.00865 0.0125 0.00608 0.00173 0.00068 0.0388 0.0179 0.0693
2L:1129560-1129710:minus -31.6633 2.11009 0.963303 2.43119 -18.3469 -10.0204 -5.69725 0.291667 -3.54128
0.0141 0.00482 0.00837 0.0117 0.00894 0.00399 0.00121 0.0153 0.0501
2L:1129560-1129710:plus -20.4388 -0.0917431 4.58716 0.0275229 -8.37755 -18.8571 -13.6606 -2.61111
-1.83486 0.00167 0.0106 0.00211 0.0267 0.00106 0.0151 0.00672 0.035 0.0318
2L:1142980-1143130:minus -21.5918 2.69725 -2.61468 3.33028 -17.7143 -16.051 -13.4771 -0.0555556
-0.633028 0.00209 0.00389 0.0259 0.00778 0.00627 0.00408 0.00645 0.017 0.0232
2L:1142980-1143130:plus -21.8163 5.47706 5.53211 10.8624 -26.8878 -17.1224 12.0183 2.06944 1.70642
0.00244 0.00133 0.00142 0.0000687 0.0199 0.00499 5.59e-06 0.00841 0.0108
2L:114540-114690:minus -42 2.00917 -2.51376 2.93578 -27.4898 -9.02041 -37.422 8.81944 -4.84404
0.0684 0.005 0.0251 0.00927 0.0336 0.00213 0.205 0.000359 0.0701
2L:114540-114690:plus -23.449 1.63303 5.23853 2.72477 -16.7143 -17.2755 -14.4771 -1.79167 -1.20183
0.00631 0.00575 0.00161 0.0102 0.00319 0.00544 0.00805 0.0282 0.0276
2L:1147740-1147890:minus -32.6633 13.8624 5.92661 2.41284 -26.6327 -16.9388 -23.3945 4.05556 1.59633
0.0216 9.75e-06 0.00119 0.0118 0.0188 0.00433 0.0374 0.00393 0.0113
2L:1147740-1147890:plus -13.3776 -1.95413 5.74312 9.33028 -18.1939 -18.8571 -5.74312 -1.61111 3.20183
0.000831 0.0192 0.00129 0.000374 0.0086 0.0151 0.00122 0.0268 0.0065
2L:11499020-11499170:minus -21.6939 2.3578 14.9633 7.29358 -8.93878 -8.72449 1.82569 -1.26389
-0.642202 0.00226 0.0044 1.77e-06 0.00104 0.00216 0.00169 0.000174 0.0243 0.0234
2L:11499020-11499170:plus -41.8163 0.266055 5.79817 5.92661 -26.9694 -18.3469 -12.945 3.55556 2.11009
0.0662 0.0094 0.00126 0.00266 0.023 0.0124 0.00571 0.00481 0.00898
2L:1150640-1150790:minus -5.19388 13.578 0.715596 3.77064 -8.93878 -17.051 -6.23853 1.22222 0.348624
0.000229 0.0000133 0.00912 0.00637 0.00216 0.00479 0.0014 0.0113 0.0167
2L:1150640-1150790:plus -22.5918 8.49541 4.65138 3.86239 -8.67347 -18.051 -13.1101 3.125 3.93578
0.00365 0.000292 0.00206 0.00622 0.00154 0.00886 0.00593 0.00569 0.00456
2L:11517060-11517210:minus -33.2245 3.36697 2.3578 6.00917 20.4694 0.122449 -19.0092 3.02778
6.07339 0.0276 0.00304 0.00506 0.00255 1.65e-07 0.000312 0.0175 0.0059 0.0018
2L:11517060-11517210:plus -31.5918 0.449541 0.733945 4.22018 -8.89796 -17.0816 -17.3028 2.79167 0.385321
0.0136 0.00882 0.00906 0.00531 0.00189 0.00487 0.0134 0.00646 0.0166
2L:11518480-11518630:minus -32.6633 5.33945 4.89908 6.59633 -9.0102 -27.1224 -17.4404 6.875
-1.09174 0.0216 0.00142 0.00185 0.00164 0.00236 0.0325 0.0137 0.00107 0.0268
2L:11518480-11518630:plus -23.3265 1.04587 4.54128 6.02752 -8.71429 -18.7551 -8.72477 -1.63889 1.79817
0.00607 0.00712 0.00215 0.00246 0.00159 0.0141 0.00243 0.027 0.0102

2L:11526760-11526910:minus -21.2857 3.24771 4.69725 6.33028 -19.0102 -18.3469 -10.7615 -2.01389
-4.90826 0.00191 0.00317 0.00202 0.00198 0.0157 0.0124 0.00356 0.0299 0.0714
2L:11526760-11526910:plus -31.6633 9.21101 2.69725 2.83486 -19.051 -10.0204 -8.08257 -0.333333
-2.88991 0.0141 0.000201 0.00446 0.0097 0.0162 0.00399 0.00214 0.0185 0.0433
2L:11527840-11527990:minus -31.9286 6.51376 1.43119 7.70642 -19.0204 -18.051 -12.9541 10.2083
1.74312 0.0166 0.000829 0.0071 0.000708 0.0158 0.00886 0.00572 0.000141 0.0105
2L:11527840-11527990:plus -14.5918 -0.972477 4.57798 5.16514 -18.7449 -0.316327 -1.50459 3.94444
-0.183486 0.00148 0.0142 0.00212 0.00424 0.0138 0.000524 0.000397 0.00411 0.0197
2L:11528140-11528290:minus 5.07143 0.0458716 10.4587 10.7615 -27.0408 -18.898 -1.9633 1.01389
5.18349 0.0000161 0.0101 0.000108 0.0000826 0.0242 0.0152 0.00044 0.0121 0.00295
2L:11528140-11528290:plus -23.0306 -1.68807 0.394495 7.45872 -18.7143 -8.65306 10.0367 -4.52778 7.63303
0.00505 0.0177 0.0102 0.000894 0.0136 0.00156 0.0000128 0.0556 0.000928
2L:1153640-1153790:minus -30.4796 -2.13761 4.56881 1.61468 -17.8571 -27.9694 -17.844 -0.166667
-1.3578 0.00941 0.0202 0.00213 0.0169 0.00698 0.0547 0.0146 0.0176 0.0286
2L:1153640-1153790:plus -13.1122 2.44037 1.95413 7.78899 -18.0816 -27.602 3.2844 -3.36111 2.19266
0.000632 0.00427 0.00588 0.000642 0.00759 0.0416 0.000107 0.0423 0.0087
2L:1157800-1157950:minus -31.2551 -1.61468 13.578 -0.642202 -17.449 -8.20408 -12.3211 0.875
10.3486 0.0113 0.0173 0.0000123 0.0328 0.00484 0.000796 0.00495 0.0127 0.0000988
2L:1157800-1157950:plus -33.4388 0.981651 0.100917 7.99083 -17.449 -36.1122 -21.1284 -1.27778 8.11009
0.0284 0.00729 0.0112 0.00059 0.00484 0.0878 0.0252 0.0244 0.000597
2L:1164320-1164470:minus -31.0714 0.706422 -0.449541 0.642202 -27.602 -9.79592 -7.6422 4.625
2.36697 0.0106 0.00805 0.0134 0.0227 0.0364 0.00377 0.00195 0.00309 0.00828
2L:1164320-1164470:plus -29.9898 -0.87156 5.33945 7.55963 -28.2347 -17.6429 -12.9541 7.69444 1.70642
0.00856 0.0138 0.00154 0.000807 0.051 0.00619 0.00572 0.000693 0.0108
2L:1166460-1166610:minus -31.4796 -1.91743 3.9633 1.7156 -27.1939 -27.4898 -2.30275 -6.15278 7.15596
0.0128 0.019 0.00272 0.0162 0.0267 0.0363 0.000477 0.0785 0.00124
2L:1166460-1166610:plus -31.4082 11.4128 -2.91743 5.44954 -9.5 -17.7857 -27.578 6.27778 -3.88073
0.0119 0.0000647 0.0282 0.00365 0.00293 0.0068 0.0651 0.00145 0.0539
2L:1170000-1170150:minus -4.95918 4.0367 -0.110092 6.6422 -18.3776 -9.72449 17.7248 -3.26389
-0.284404 0.000213 0.00237 0.012 0.00161 0.00926 0.00352 5.36e-07 0.0413 0.0207
2L:1170000-1170150:plus -33.0408 -2.3945 2.26606 -1.34862 -17.9796 -8.72449 -14.9266 8.44444 1.54128
0.0264 0.0218 0.00524 0.0413 0.00726 0.00169 0.00884 0.000451 0.0115
2L:1172760-1172910:minus -23.0306 6.38532 3.79817 0.605505 -8.37755 -9.34694 -21.1101 2.79167 2.94495
0.00505 0.000882 0.00291 0.023 0.00106 0.00261 0.0251 0.00646 0.00714
2L:1172760-1172910:plus -23.1531 3.07339 -4.81651 -0.889908 -26.898 -8.79592 -16.578 11.8472
3.6055 0.00542 0.00339 0.0471 0.0355 0.0203 0.0019 0.012 0.0000359 0.00532
2L:1177600-1177750:minus -40.7755 0.330275 2.62385 2.30275 11.6939 -26.898 -15.2752 5.01389 -1.43119
0.0433 0.00919 0.00458 0.0124 1.17e-06 0.0302 0.00948 0.00261 0.029
2L:1177600-1177750:plus -22 14.945 0.155963 8.80734 -8.57143 -17.3469 -14.7798 2.56944 3.56881
0.00291 2.29e-06 0.011 0.000518 0.00113 0.0058 0.00858 0.00701 0.00543
2L:11806960-11807110:minus -23.0408 1 1.10092 6.12844 -26.5918 -8.45918 -21.4128 -0.138889
-1.69725 0.00512 0.00724 0.00798 0.00231 0.0186 0.00111 0.0265 0.0175 0.0308
2L:11806960-11807110:plus -31.7041 3.3578 -3.2844 0.633028 -16.7143 -9.79592 -21.1651 -1.52778 5.90826
0.0146 0.00305 0.0312 0.0229 0.00319 0.00377 0.0253 0.0262 0.00198
2L:11807760-11807910:minus -23.8469 0.238532 -1.07339 5.73394 -26.0408 -8.5 -18.578 2.08333 11.4771
0.00685 0.0095 0.0163 0.00307 0.0171 0.00126 0.0163 0.00837 0.0000679
2L:11807760-11807910:plus -22.1531 -1.68807 -0.40367 6.47706 2.14286 -9.37755 -17 2.06944 -1.3578
0.0031 0.0177 0.0132 0.00179 0.0000316 0.00265 0.0128 0.00841 0.0286
2L:11807980-11808130:minus -31.4286 -1.46789 -1.83486 0.899083 -18.7143 -8.86735 -8.7156 4.125
5.38532 0.0119 0.0166 0.0206 0.0212 0.0136 0.00198 0.00242 0.00382 0.00267
2L:11807980-11808130:plus -30.8469 1.99083 2.75229 1.91743 -27.2653 -27.8265 -6.42202 0.361111 4.56881
0.0103 0.00504 0.00436 0.015 0.0302 0.0498 0.00146 0.0149 0.00356
2L:11808820-11808970:minus -22.9286 -1.20183 -0.522936 -0.522936 -17.0408 -16.0102 -14.7982
-2.86111 -0.963303 0.0046 0.0153 0.0137 0.0313 0.00329 0.00406 0.00861 0.0373 0.0258

2L:11808820-11808970:plus -32.7347 6.61468 -2.16514 1.99083 -18.449 -18.0816 -4.78899 1.51389 1.93578 0.0225 0.000789 0.0227 0.0144 0.0107 0.00934 0.000935 0.0102 0.00969
2L:11813260-11813410:minus -32.9592 -1.79817 -1.33028 -1.02752 -18.449 -27.602 -17.789 2.41667 2.9633 0.0252 0.0183 0.0177 0.037 0.0107 0.0416 0.0145 0.00742 0.00709
2L:11813260-11813410:plus -29.6939 -0.825688 12.578 5.75229 -18.4184 -18.051 -11.7339 -0.847222 0.12844 0.00847 0.0136 0.0000285 0.00296 0.0101 0.00886 0.00435 0.0216 0.0179
2L:11813480-11813630:minus -30.4388 -0.513761 11.1651 7.29358 -17.4898 -19.3061 -21.6881 3.55556 3.54128 0.00923 0.0123 0.000072 0.00104 0.0053 0.0187 0.0279 0.00481 0.0055
2L:11813480-11813630:plus -22.0612 -1.91743 3.70642 3.38532 -17.8571 -8.23469 -16.7431 -1.97222 1.23853 0.00297 0.019 0.00302 0.00761 0.00698 0.000875 0.0123 0.0296 0.0132
2L:11813880-11814030:minus -31.4796 14.6789 0.376147 5.57798 1.36735 0.346939 -14.1101 6.36111 -2.87156 0.0128 3.91e-06 0.0102 0.0034 0.0000781 0.000221 0.00743 0.00139 0.043
2L:11813880-11814030:plus -40.4796 0.862385 -2.2844 1.55046 -28.3061 -9.02041 -23.9174 11.5694 -6.11009 0.0407 0.00761 0.0235 0.0173 0.0553 0.00213 0.0405 0.0000463 0.0912
2L:11829400-11829550:minus -21.9592 -1.11927 -4.05505 7.27523 1.80612 -7.5 -19.844 1.65278 -3.78899 0.00279 0.0149 0.0385 0.00109 0.0000557 0.000614 0.02 0.00974 0.0526
2L:11829400-11829550:plus -4.33673 -2.15596 5.04587 4.07339 -7.86735 -9.34694 -10.055 1.11111 2.89908 0.00014 0.0203 0.00174 0.00562 0.000617 0.00261 0.00311 0.0117 0.00722
2L:11849920-11850070:minus -23.1837 3.09174 2.33945 3.01835 -7.93878 0.755102 -3.88991 5.375 1.94495 0.00553 0.00337 0.0051 0.0089 0.000767 0.000175 0.000723 0.00222 0.00968
2L:11849920-11850070:plus -15.0102 4.59633 15.7798 5.48624 -27.9286 -27.3367 -17.6881 -0.805556 0.495413 0.00162 0.00191 1.81e-07 0.00357 0.0412 0.0347 0.0143 0.0213 0.0162
2L:1186000-1186150:minus -13.4082 -0.587156 2.16514 10.1468 -18.0816 -16.8265 -9.80734 0.138889 10.3486 0.000867 0.0126 0.00544 0.000128 0.00759 0.0043 0.00297 0.016 0.0000988
2L:1186000-1186150:plus -21.5918 5.02752 3.93578 4.49541 -18.3878 -17.9388 -19.9174 -4.70833 -3.53211 0.00209 0.00161 0.00275 0.00487 0.00931 0.00738 0.0203 0.0579 0.0501
2L:11961040-11961190:minus -22.5918 5 2.2844 10.5963 -9.20408 -19.0816 -8.74312 5.77778 -1.07339 0.00365 0.00163 0.0052 0.0001 0.00262 0.017 0.00244 0.00184 0.0267
2L:11961040-11961190:plus -23.1531 -2.84404 3.12844 2.3945 -28.2347 1.34694 -23.2202 3.45833 -0.431193 0.00542 0.0247 0.00378 0.0119 0.051 0.0000568 0.0364 0.005 0.0214
2L:11961360-11961510:minus -33.0714 4.97248 3.11009 6.85321 -18.7959 -28.6735 -8.6055 0.555556 9.61468 0.0267 0.00165 0.00381 0.00137 0.0148 0.0667 0.00237 0.014 0.00029
2L:11961360-11961510:plus -22.551 -0.733945 1.49541 9.7156 -9.16327 0.244898 -14.5229 0.694444 -7.73394 0.00359 0.0132 0.00694 0.000242 0.00249 0.000285 0.00813 0.0134 0.129
2L:11988120-11988270:minus -31.1735 -3.45872 5.72477 2.88991 10.9184 0.979592 -13.3761 3.68056 -0.706422 0.0109 0.0292 0.0013 0.00942 4.19e-06 0.000136 0.0063 0.00457 0.024
2L:11988120-11988270:plus -32.5102 0.0642202 1.22018 1.3578 -26.9286 -7.94898 -9.90826 3.94444 -3.93578 0.0202 0.0101 0.00765 0.0186 0.0214 0.00074 0.00303 0.00411 0.0547
2L:11989800-11989950:minus -32.6224 7.65138 -3.69725 -0.651376 0.877551 -0.316327 -14.5321 8.38889 6.95413 0.0211 0.00046 0.035 0.0329 0.00019 0.000524 0.00814 0.000466 0.00136
2L:11989800-11989950:plus -22 9.10092 3.31193 8.77982 20.4694 -17.0816 -5.46789 0.819444 -1.31193 0.00291 0.000212 0.00352 0.000533 1.65e-07 0.00487 0.00113 0.0129 0.0284
2L:11990820-11990970:minus -21.2551 -2.59633 3.05505 5.3945 -27.1939 -9.5 -12.2661 0.972222 5.79817 0.00191 0.023 0.00389 0.00379 0.0267 0.00294 0.00489 0.0123 0.00208
2L:11990820-11990970:plus -33.3673 -0.238532 1.25688 4.22936 10.6939 -9.53061 -14.6239 0.25 1.95413 0.0282 0.0112 0.00755 0.00525 7.35e-06 0.0031 0.0083 0.0155 0.00964
2L:12002240-12002390:minus -23.9592 0.12844 3.65138 2.88991 10.6531 19.1531 -12.6697 0.361111 5.44037 0.00708 0.00987 0.00308 0.00942 0.0000102 7.95e-07 0.00536 0.0149 0.00259
2L:12002240-12002390:plus -41.9592 1.47706 -4.54128 -2.9633 -8.70408 -9.02041 -16.5963 6.22222 5.33028 0.0679 0.00608 0.0438 0.0683 0.00155 0.00213 0.012 0.00149 0.00273
2L:12020880-12021030:minus -32.9286 0.40367 14.9266 2.3578 20.2041 -0.204082 -15.7615 -1.04167 -2.34862 0.025 0.00896 1.9e-06 0.0121 3.64e-07 0.000463 0.0104 0.0228 0.0368
2L:12020880-12021030:plus -30.551 -5.11009 -0.559633 9.59633 -18.4184 -8.5 -23.3394 3.88889 0.137615 0.00959 0.0454 0.0139 0.000275 0.0101 0.00126 0.0371 0.00421 0.0178

2L:12027360-12027510:minus -23.9184 6.77064 4.13761 1.82569 -8.16327 -8.53061 -19.2569
4.81944 3.36697 0.00695 0.000731 0.00254 0.0156 0.000873 0.00136 0.0182 0.00284 0.00607
2L:12027360-12027510:plus -1.77551 0.247706 0.174312 6.74312 -27.1939 -17.2755
-13.0275 7.11111 -0.495413 0.0000235 0.00947 0.0109 0.0015 0.0267 0.00544 0.00582 0.000949
0.022
2L:12038420-12038570:minus -23.4796 13.2844 6.21101 5.47706 -27.1939 -17.9388 -19.3945
-2.875 -0.229358 0.00634 0.0000175 0.00105 0.0036 0.0267 0.00738 0.0186 0.0375 0.0202
2L:12038420-12038570:plus -29.7755 -1.36697 0.834862 2.0367 -28.1939 -17.1224
-19.6239 6.41667 7.88991 0.0085 0.0161 0.00875 0.0141 0.0482 0.00499 0.0193 0.00135 0.000688
2L:12040640-12040790:minus -23.8878 0.908257 6.41284 3.19266 -8.70408 0.867347 -18.4128
1.16667 -0.440367 0.00692 0.00748 0.000959 0.00829 0.00155 0.000144 0.0159 0.0115 0.0216
2L:12040640-12040790:plus -32.1429 3.05505 7.30275 7.80734 -17.3776 -17.051 -14.7339 0.5 0.614679
0.0179 0.00341 0.000626 0.00063 0.00419 0.00479 0.0085 0.0143 0.0158
2L:12044720-12044870:minus -22.8878 4.70642 6.69725 2.75229 -18.6837 -9.5 -21.0917 5.61111
-0.0183486 0.00441 0.00183 0.000836 0.0101 0.0126 0.00294 0.025 0.00199 0.0185
2L:12044720-12044870:plus -32.5816 7.3945 -0.559633 3.23853 -18.4898 0.826531 -17.4771 11.0694
3.51376 0.0208 0.000526 0.0139 0.00815 0.0116 0.000153 0.0138 0.000072 0.00566
2L:12045080-12045230:minus -31.8469 4.06422 4.12844 7.90826 -18.7551 -9.16327 -13.8532 -2.31944
7.68807 0.0162 0.00235 0.00254 0.000611 0.0144 0.00223 0.00702 0.0325 0.000841
2L:12045080-12045230:plus -24 2 0.605505 2.19266 -26.8163 -27.6327 -11.4771 10.6528 -0.0733945
0.00719 0.00502 0.00946 0.0131 0.0195 0.043 0.00412 0.000101 0.0188
2L:12046180-12046330:minus -24.0306 1.12844 5 6.12844 -26.9286 -27.7857 -10.2294 14.9722 8.11009
0.00723 0.00691 0.00178 0.00231 0.0214 0.0466 0.00321 4.28e-07 0.000597
2L:12046180-12046330:plus -39.3265 -2.25688 0.266055 1.59633 -17.7143 -17.2755 -25.0275 1.45833 3.93578
0.0332 0.0209 0.0106 0.017 0.00627 0.00544 0.0473 0.0104 0.00456
2L:12048900-12049050:minus -23.7449 0.743119 15.6606 7.33945 20.2041 9.79592 -13.9083 4.29167
-0.834862 0.00674 0.00794 3.94e-07 0.00102 3.64e-07 0.0000183 0.00711 0.00356 0.0249
2L:12048900-12049050:plus -40.2143 0.963303 1.91743 1.51376 -18.7143 0.755102 -19.6881 8.11111 1.69725
0.0372 0.00734 0.00596 0.0175 0.0136 0.000175 0.0195 0.000547 0.0108
2L:12054800-12054950:minus -24.1837 7.88073 0.330275 0.66055 -28.4592 -17.3469 -7.44954 3.13889
0.0275229 0.00751 0.000407 0.0104 0.0225 0.0568 0.0058 0.00187 0.00566 0.0183
2L:12054800-12054950:plus -24 5.16514 7.43119 1.06422 -17.7551 -17.051 -22.5963 9.41667 4.18349
0.00719 0.00152 0.000587 0.0203 0.00658 0.00479 0.0328 0.000245 0.00404
2L:12054960-12055110:minus -30.5918 -1.52294 -1.41284 0.990826 -8.60204 -18.0102 -20.9174 2.52778
3.24771 0.00963 0.0169 0.0181 0.0206 0.00125 0.00812 0.0242 0.00712 0.00636
2L:12054960-12055110:plus -21.551 1.55963 2 2.98165 -18.1531 -27.3367 -5.33945 1.76389 1.34862
0.00205 0.0059 0.00578 0.00905 0.00829 0.0347 0.00109 0.00937 0.0125
2L:12055420-12055570:minus -23.6939 0.899083 4.72477 4.16514 -17.9796 -9.79592 -13.1101 -2.84722
-5.40367 0.00667 0.00751 0.00199 0.00538 0.00726 0.00377 0.00593 0.0372 0.0791
2L:12055420-12055570:plus -30.102 0.990826 4.74312 2.11927 -18.0816 -18.0102 -11.789 -0.916667
-0.59633 0.00859 0.00726 0.00198 0.0135 0.00759 0.00812 0.0044 0.022 0.0229
2L:12055940-12056090:minus -23.4898 3.12844 0.577982 6.02752 -8.30612 -9.45918 3.65138 7.98611
-4.62385 0.00639 0.00332 0.00955 0.00246 0.00097 0.00279 0.0000937 0.000588 0.066
2L:12055940-12056090:plus -32.8469 -3.50459 5.14679 2.83486 -27.2347 -36.0408 -16.7156 4.04167 0.614679
0.0243 0.0296 0.00167 0.0097 0.0283 0.0837 0.0122 0.00395 0.0158
2L:12063120-12063270:minus -13.7857 3.31193 13.9266 7.29358 -27.2653 -17.3469 -11 3.31944 1.53211
0.00108 0.0031 7.79e-06 0.00104 0.0302 0.0058 0.00373 0.00528 0.0116
2L:12063120-12063270:plus -30.8469 13.5306 5.09174 2.55963 -17.4898 -8.34694 -13.2661 2.01389 -4.51376
0.0103 0.0000206 0.00171 0.0111 0.0053 0.000993 0.00614 0.00858 0.064
2L:120820-120970:minus -33.9592 5.75229 6.44954 6.3578 -18.6837 -8.16327 -15.4862 0.638889 -3.14679
0.0304 0.00118 0.000941 0.00195 0.0126 0.000783 0.00987 0.0137 0.0463
2L:120820-120970:plus -23.8878 1.90826 1.08257 9.33028 -8.40816 -17.0102 -24.1651 4.15278 -4.31193
0.00692 0.00519 0.00803 0.000374 0.00108 0.00453 0.042 0.00377 0.0604
2L:12093700-12093850:minus -24.7857 0.981651 -4.19266 1.6789 -28.7959 -0.316327 -22.6239 3.36111

-2.36697 0.00829 0.00729 0.04 0.0165 0.0641 0.000524 0.0329 0.00519 0.0371
2L:12093700-12093850:plus -32 -0.321101 6.98165 9.75229 10.9898 1.09184 -11.0826 -2.125 3.42202
0.0171 0.0115 0.000733 0.000226 3.28e-06 0.000091 0.0038 0.0308 0.00597
2L:12096480-12096630:minus -4.32653 1.38532 8.99083 2.09174 -17.3776 -8.5 -8 6.51389 0.614679
0.000136 0.00629 0.000256 0.0137 0.00419 0.00126 0.0021 0.00129 0.0158
2L:12096480-12096630:plus -22.2245 -4.31193 9.33028 1.86239 -18.1531 -17.6429 -13.7431 0.194444 -0.743119
0.00325 0.0368 0.000209 0.0153 0.00829 0.00619 0.00685 0.0157 0.0243
2L:12097800-12097950:minus -30.398 6.61468 5.52294 7.6422 -19.0102 0.316327 -19.8349 0.430556
-6.00917 0.00901 0.000789 0.00142 0.000721 0.0157 0.000243 0.02 0.0146 0.089
2L:12097800-12097950:plus -23.6327 1.57798 4.7156 7 20.2041 -19.3469 9.73394 4.83333 3.46789
0.00661 0.00586 0.002 0.00132 3.64e-07 0.0194 0.0000142 0.00283 0.00582
2L:12104060-12104210:minus -21.4694 -1.14679 3.54128 3.44954 -8.37755 -27.602 -18.7798 -1.73611
-1.26606 0.00201 0.015 0.00322 0.00738 0.00106 0.0416 0.0169 0.0277 0.0281
2L:12104060-12104210:plus -29.2143 -0.944954 3.17431 2.66972 -18.7143 1.38776 -11.4404 -0.75
-6.33945 0.00836 0.0141 0.00371 0.0106 0.0136 0.0000559 0.00409 0.021 0.0966
2L:12109000-12109150:minus -23.4184 2.93578 8.0367 4.61468 10.6531 -8.23469 -19.2569 0.194444
3.15596 0.00624 0.00356 0.00043 0.00475 0.0000102 0.000875 0.0182 0.0157 0.00659
2L:12109000-12109150:plus -21.9184 14.3394 7.13761 10.5872 -8.63265 -17.7857 -20.578 1.59722 1.48624
0.00261 5.96e-06 0.00068 0.000115 0.0013 0.0068 0.0228 0.00993 0.0118
2L:12172900-12173050:minus -24.0714 0.513761 4.06422 3.86239 -18.4898 -7.79592 -12.156 5.01389
5.70642 0.00731 0.00862 0.00261 0.00622 0.0116 0.000723 0.00477 0.00261 0.00222
2L:12172900-12173050:plus -31.4694 -0.944954 1.37615 3.41284 20.4694 -17.3469 1.6789 3.06944
3.15596 0.0124 0.0141 0.00724 0.00756 1.65e-07 0.0058 0.000182 0.00581 0.00659
2L:12207660-12207810:minus -31.7449 -1.68807 3.29358 1.9633 -27.1939 -10.0204 -19.9541 3.05556
10.1284 0.0152 0.0177 0.00354 0.0146 0.0267 0.00399 0.0204 0.00584 0.000129
2L:12207660-12207810:plus -12.1531 0.972477 15.4495 -0.633028 -18.0816 -8.20408 -12.3578 -4.18056
7.11009 0.000352 0.00731 9.17e-07 0.0327 0.00759 0.000796 0.00499 0.0513 0.00128
2L:122320-122470:minus -23.1122 2.99083 3.78899 0.954128 10.1327 -10.0204 -24.8073 3.18056 0.275229
0.0053 0.00349 0.00292 0.0208 0.0000225 0.00399 0.046 0.00557 0.0171
2L:122320-122470:plus -12.1429 4.36697 5.21101 3.0367 -27.2245 -18.4184 -7.21101 6.31944 1.27523
0.000343 0.00209 0.00163 0.00886 0.0272 0.0127 0.00177 0.00142 0.013
2L:12366060-12366210:minus -21.9286 13.4592 8.33028 5.94495 -18.0816 -8.53061 -16.633 -0.736111
0.752294 0.0027 0.000064 0.000367 0.00261 0.00759 0.00136 0.0121 0.0209 0.0152
2L:12366060-12366210:plus -22.9592 2.3211 4.6422 11.2936 -17.5306 -19.0816 -6.21101 -2.68056 3.6422
0.00475 0.00446 0.00206 0.0000139 0.0054 0.017 0.00139 0.0356 0.00516
2L:12404600-12404750:minus -22.8571 4.55963 8.22936 5.3945 -7.85714 -0.244898 -12.6147 -1.875
-2.19266 0.00427 0.00194 0.000388 0.00379 0.000594 0.000496 0.00529 0.0288 0.0352
2L:12404600-12404750:plus -31.4082 -3.99083 5.45872 3.31193 -17.5306 -7.72449 -14.2018 5.625 7.04587
0.0119 0.0337 0.00147 0.00786 0.0054 0.00068 0.00758 0.00198 0.00132
2L:12405220-12405370:minus -24.3367 5.38532 1.44037 2.78899 -18.4898 -9.79592 -22.0183 7.04167
-2.6055 0.00789 0.00139 0.00707 0.00992 0.0116 0.00377 0.0296 0.000984 0.04
2L:12405220-12405370:plus -13.6735 9.11009 -0.66055 7.34862 -27.1939 10.5 -12.1284 2.47222 -4.41284
0.00103 0.000212 0.0143 0.000998 0.0267 5.88e-06 0.00474 0.00727 0.0624
2L:12421360-12421510:minus -31.6633 4.93578 -0.00917431 2.86239 -17.3776 -19.0102 -23.1009 8.375
-0.504587 0.0141 0.00167 0.0116 0.00954 0.00419 0.0159 0.0357 0.00047 0.0221
2L:12421360-12421510:plus -30.2143 -4.27523 0.816514 5.57798 -26.9694 9.67347 -7.34862 0.541667 0.0183486
0.0088 0.0364 0.0088 0.0034 0.023 0.0000217 0.00183 0.0141 0.0183
2L:12421860-12422010:minus -31.9694 0.174312 3.3211 7.88991 -26.6327 -17.0102 -8.09174 3.16667
2.99083 0.0168 0.00971 0.00351 0.00062 0.0188 0.00453 0.00214 0.0056 0.00706
2L:12421860-12422010:plus -32.1837 0.642202 6.80734 3.69725 -9.27551 -9.86735 -14.2936 5.73611 9.88073
0.0181 0.00823 0.000794 0.00665 0.00284 0.00391 0.00774 0.00188 0.000206
2L:12423260-12423410:minus -14.8163 3.90816 0.449541 4.13761 0.540816 -8.0102 -15.8532 -0.0833333
1.01835 0.00159 0.00102 0.00998 0.00546 0.000321 0.000754 0.0106 0.0172 0.0142
2L:12423260-12423410:plus -22.9592 5.07339 0.238532 3.01835 -17.4898 -18.3061 -12.8257 -0.708333

4.66055 0.00475 0.00158 0.0107 0.0089 0.0053 0.0114 0.00555 0.0207 0.0035
2L:12424700-12424850:minus -44.0714 -1.6789 -2.55963 6.55963 -28.1939 -9.68367 -23.2018 3.33333
-4.25688 0.0948 0.0177 0.0254 0.00172 0.0482 0.00342 0.0363 0.00525 0.0593
2L:12424700-12424850:plus -29.7041 3.3945 4.24771 2.85321 -17.6837 -9.7551 -24.6972 4.05556 2.14679
0.00848 0.00301 0.00243 0.00955 0.0058 0.00358 0.0453 0.00393 0.00883
2L:12427540-12427690:minus -40.1429 -2.41284 0.678899 4.50459 -27.3061 -26.5612 -12.7615 -0.597222
4.19266 0.0363 0.0219 0.00923 0.00484 0.0315 0.0237 0.00547 0.0201 0.00401
2L:12427540-12427690:plus -31.6735 10.1284 -2.21101 5.73394 -17.7143 -18.2755 -33.8899 1.68056 4.30275
0.0141 0.000125 0.023 0.00307 0.00627 0.0112 0.144 0.00964 0.00388
2L:12433360-12433510:minus -23.8878 10.6147 8 10.5963 -27.4184 -9.72449 -21.6789 -2.75 -8.09174
0.00692 0.0000977 0.000438 0.0001 0.0323 0.00352 0.0278 0.0363 0.138
2L:12433360-12433510:plus -14.449 0.724771 2.97248 -0.311927 -17.6735 -9.7551 -9.3578 2.43056
8.11009 0.0014 0.008 0.00401 0.0294 0.00572 0.00358 0.00273 0.00738 0.000597
2L:12433940-12434090:minus -31.6735 10.5138 4.07339 4.31193 -19.2755 -17.7857 -13.633 -0.791667
-3.07339 0.0141 0.000103 0.0026 0.00516 0.0164 0.0068 0.00668 0.0212 0.0454
2L:12433940-12434090:plus -24.1122 7.95413 -1 3.77064 -17.449 -17.5714 -7.68807 2.54167 -5.57798
0.00738 0.000392 0.016 0.00637 0.00484 0.00616 0.00197 0.00709 0.0816
2L:12434380-12434530:minus -21.6633 9.61468 1.49541 1.46789 0.877551 -8.72449 -28.7706 2.61111
-3.65138 0.00223 0.000163 0.00694 0.0179 0.00019 0.00169 0.0764 0.00691 0.0512
2L:12434380-12434530:plus -31.398 10.6972 -0.330275 0.293578 -8.64286 -18.9286 -14.7248 2.48611
-0.284404 0.0117 0.0000939 0.0129 0.0248 0.00139 0.0153 0.00848 0.00723 0.0207
2L:12434720-12434870:minus -21.8469 -2.92661 0.137615 3.17431 -16.4898 -9.65306 -20.6789 -4.11111
3.40367 0.00247 0.0253 0.0111 0.00842 0.00312 0.0034 0.0232 0.0505 0.00603
2L:12434720-12434870:plus -24.3776 1.30275 8.3211 5.94495 -27.3469 -26.4898 -18.6972 0.888889 8.10092
0.00793 0.00649 0.000368 0.00261 0.0318 0.0225 0.0166 0.0126 0.000623
2L:12435540-12435690:minus -22.1531 11.2661 2.92661 0.605505 -18.3469 -9.72449 -14.4037 -3.52778
-0.807339 0.0031 0.0000708 0.00408 0.023 0.00894 0.00352 0.00792 0.044 0.0247
2L:12435540-12435690:plus -22.8878 4.57798 5.16514 3.48624 -18.6429 -17.8265 -13.2844 1.15278 -1.66055
0.00441 0.00193 0.00166 0.0072 0.0121 0.00699 0.00617 0.0115 0.0305
2L:12436380-12436530:minus -31.4796 3.94495 3.41284 2.6789 -17.7551 -18.7857 -21.3853 1.22222
0.743119 0.0128 0.00245 0.00338 0.0105 0.00658 0.0146 0.0264 0.0113 0.0153
2L:12436380-12436530:plus -11.8163 1.04587 -0.33945 3.9633 -18.4592 -18.051 -9.49541 -5.36111 2.58716
0.000272 0.00712 0.0129 0.0059 0.0109 0.00886 0.0028 0.0667 0.00779
2L:12437100-12437250:minus -11.6224 4.26606 3.09174 3.87156 -8.89796 -27.4898 -5.17431 -6.81944
2.36697 0.000259 0.00217 0.00383 0.00618 0.00189 0.0363 0.00104 0.0894 0.00828
2L:12437100-12437250:plus -21.7755 -1.43119 0.211009 4.11927 -16.4898 -26.7143 -12.6514 8.20833 0.59633
0.00241 0.0164 0.0108 0.00551 0.00312 0.0257 0.00534 0.000518 0.016
2L:12437380-12437530:minus -33 6.50459 -2.51376 1.58716 -18.0102 -7.72449 -21.9817 1.13889 3.45872
0.0258 0.000833 0.0251 0.017 0.00732 0.00068 0.0294 0.0116 0.00591
2L:12437380-12437530:plus -33.0408 13.3878 -2.21101 1.78899 -17.7143 -18.5714 -25.1376 5.22222 1.69725
0.0264 0.0000869 0.023 0.0158 0.00627 0.0136 0.048 0.00238 0.0108
2L:12456280-12456430:minus -31.9592 -0.247706 -0.981651 0.458716 -17.7449 -17.5 -16.8532
3.59722 -0.119266 0.0167 0.0112 0.0159 0.024 0.0063 0.00599 0.0125 0.00473 0.0192
2L:12456280-12456430:plus -32.2245 1.99083 4.25688 1.69725 -27.898 0.122449 -23.0092 0.25 -1.93578
0.0183 0.00504 0.00242 0.0163 0.04 0.000312 0.0351 0.0155 0.0326
2L:12457320-12457470:minus -12.8878 -1.77064 6.48624 0.93578 -18.7143 -17.2347 -20.5321 7.04167
7.84404 0.000523 0.0182 0.000925 0.0209 0.0136 0.00511 0.0226 0.000984 0.000701
2L:12457320-12457470:plus -31.8061 5.52294 4.17431 2.19266 -18.3878 -7.23469 -21.8624 2.04167
-0.0275229 0.0158 0.00131 0.0025 0.0131 0.00931 0.000557 0.0288 0.00849 0.0186
2L:12461340-12461490:minus -31.6939 0.614679 1.54128 11.2936 -26.9286 -18.9286 -8.56881 10.25
-2.87156 0.0142 0.00832 0.00683 0.0000139 0.0214 0.0153 0.00236 0.000136 0.043
2L:12461340-12461490:plus -32.4796 1.86239 3.29358 4.81651 -26.9286 -15.9388 -16.6422 -2.68056 -0.623853
0.02 0.00528 0.00354 0.00451 0.0214 0.00405 0.0121 0.0356 0.0232
2L:12467700-12467850:minus -33.4082 2.93578 1.3578 1.46789 -27.1939 -9.57143 -23.5413 -1.48611

3.46789 0.0283 0.00356 0.00728 0.0179 0.0267 0.00326 0.0383 0.0259 0.00582
2L:12467700-12467850:plus -14.4796 -0.458716 1.45872 9.7156 -9.23469 0.0918367 -16.4954 7.68056
-0.559633 0.00142 0.012 0.00703 0.000242 0.00277 0.000323 0.0118 0.000698 0.0226
2L:12474560-12474710:minus -31.8878 0.990826 8.33028 3.05505 -17.6735 -18.0102 -16.6055 2.52778
6.07339 0.0164 0.00726 0.000367 0.00878 0.00572 0.00812 0.012 0.00712 0.0018
2L:12474560-12474710:plus -32.7347 -1.52294 5.16514 6.45872 -27.2653 -9.7551 -17.6514 1.81944 -4.3578
0.0225 0.0169 0.00166 0.00182 0.0302 0.00358 0.0142 0.00919 0.0614
2L:12475360-12475510:minus -41.8061 -0.825688 3.63303 1.99083 -16.449 -18.4184 -12.6055 5.43056
1.48624 0.0654 0.0136 0.00311 0.0144 0.00307 0.0127 0.00528 0.00216 0.0118
2L:12475360-12475510:plus -33.3061 -2.6055 1.97248 0.853211 -19.0918 -27.6735 -12.9083 7.86111 2.99083
0.0279 0.0231 0.00584 0.0214 0.0163 0.0443 0.00566 0.000631 0.00706
2L:12483080-12483230:minus -23.1224 4.05102 -0.330275 5.75229 -8.96939 0.163265 -21.4587 6.34722
3.98165 0.00531 0.000335 0.0129 0.00296 0.00226 0.000307 0.0267 0.0014 0.00439
2L:12483080-12483230:plus -30.7041 2.83486 6.66972 6.11009 -17.9796 -8.72449 -25.1927 -4.58333 -1.6055
0.00993 0.0037 0.000847 0.00233 0.00726 0.00169 0.0484 0.0563 0.0302
2L:12483640-12483790:minus -23.4082 0.394495 1.94495 9.08257 -18.4184 -9.53061 -13.9725 -2.40278
5.13761 0.00623 0.00899 0.0059 0.000444 0.0101 0.0031 0.00721 0.0332 0.00298
2L:12483640-12483790:plus -33.1429 2.98165 -0.137615 -0.40367 -27.3367 -27.449 -16.6055 3.94444
7.42202 0.0272 0.0035 0.0121 0.0302 0.0317 0.0357 0.012 0.00411 0.00106
2L:12484300-12484450:minus -23.4898 6.54128 -1.21101 2.19266 -18.0816 1.05102 -27.5229 5.375
-4.89908 0.00639 0.000818 0.017 0.0131 0.00759 0.000107 0.0646 0.00222 0.0712
2L:12484300-12484450:plus -22.3367 1.86239 1.97248 2.19266 -28.2347 -25.602 -8.59633 0.361111 9.88073
0.00339 0.00528 0.00584 0.0131 0.051 0.0207 0.00237 0.0149 0.000206
2L:12492320-12492470:minus -30.398 0.229358 -0.862385 6.47706 -19.0102 -9.72449 -17.1835 0.805556
5.6055 0.00901 0.00953 0.0153 0.00179 0.0157 0.00352 0.0132 0.013 0.00232
2L:12492320-12492470:plus -22.6633 3.47706 0.366972 1.9633 -18.1837 -9.72449 -11.789 -0.222222
7.02752 0.00384 0.00292 0.0103 0.0146 0.00835 0.00352 0.0044 0.0179 0.00133
2L:12493700-12493850:minus -23.3776 0.394495 2.10092 9.59633 -27.1633 -8.79592 -13.578 -0.638889
1.22018 0.00619 0.00899 0.00556 0.000275 0.025 0.0019 0.0066 0.0203 0.0133
2L:12493700-12493850:plus -48.7755 2.58716 -0.522936 1.58716 -27.3061 -0.132653 -18.5872 1.20833
4.11927 0.0981 0.00404 0.0137 0.017 0.0315 0.000444 0.0164 0.0113 0.00411
2L:12495380-12495530:minus -32.6224 7.77064 2.10092 3.95413 -27.3367 -10.0918 5.07339 6.06944
2.75229 0.0211 0.000432 0.00556 0.00593 0.0317 0.00404 0.0000562 0.0016 0.00748
2L:12495380-12495530:plus -34.449 0 3.50459 1.18349 -17.9796 -9.72449 -21.8349 3.26389 5.44037
0.0319 0.0103 0.00326 0.0196 0.00726 0.00352 0.0286 0.00539 0.00259
2L:12504720-12504870:minus -23.0714 -0.348624 -2.0367 9.41284 -8.89796 -9.30612 -11.5596 5.625
-0.889908 0.0052 0.0116 0.0219 0.000354 0.00189 0.00257 0.00419 0.00198 0.0253
2L:12504720-12504870:plus -22.9592 -3.57798 3.62385 5.57798 -27 -18.1224 -1.83486 0.75 11.8532 0.00475
0.0302 0.00312 0.0034 0.0233 0.00988 0.000428 0.0132 0.0000428
2L:12507740-12507890:minus -23.3776 4.08257 0.614679 7.27523 -18.3878 -8.72449 -21.2385 1.79167
-4.18349 0.00619 0.00233 0.00943 0.00109 0.00931 0.00169 0.0257 0.00928 0.0581
2L:12507740-12507890:plus -22 0.972477 4.55963 3.53211 -17.4592 -15.9796 -6.69725 -1.625 -1.6422
0.00291 0.00731 0.00214 0.00704 0.00491 0.00405 0.00157 0.0269 0.0304
2L:12514100-12514250:minus -30.4796 3.81651 -0.990826 2.66972 -27.5918 -17.7857 -15.3945 7.75
2.16514 0.00941 0.00258 0.0159 0.0106 0.0358 0.0068 0.0097 0.000672 0.00879
2L:12514100-12514250:plus -23.4388 -1.07339 5.88991 2.6422 -26.2653 -19.1224 -15.1468 11.8889 -1.41284
0.00625 0.0147 0.00121 0.0107 0.018 0.0175 0.00924 0.0000344 0.0289
2L:12515020-12515170:minus -32.551 8.01835 0.238532 1.77982 -18.1531 -8.79592 -15.1376 5.58333
3.41284 0.0207 0.000378 0.0107 0.0158 0.00829 0.0019 0.00922 0.00202 0.006
2L:12515020-12515170:plus -31.7041 3.89908 -0.174312 9.88073 -18.1531 -8.5 -18.1009 1.34722 -0.642202
0.0146 0.0025 0.0123 0.000183 0.00829 0.00126 0.0152 0.0108 0.0234
2L:12521240-12521390:minus -40.4388 5.13761 -0.844037 5.90826 -8.86735 -8.42857 -35.9725 -3.30556
-10.4771 0.0401 0.00154 0.0152 0.00271 0.00173 0.00104 0.176 0.0417 0.212
2L:12521240-12521390:plus -23.0816 1.42202 3.31193 3.3578 -17.6429 -8.65306 -31.9358 3.68056 0.816514

0.00522 0.00621 0.00352 0.00767 0.00562 0.00156 0.117 0.00457 0.015
2L:12529680-12529830:minus -31.449 3.36697 1.20183 4.6422 -26.9694 -17.9796 -19.9541 3.83333
-4.63303 0.0123 0.00304 0.0077 0.00473 0.023 0.00764 0.0204 0.0043 0.0661
2L:12529680-12529830:plus -21.0408 -2.81651 0.559633 3.76147 -27 0.122449 -9.62385 1.68056 11.3853
0.00186 0.0245 0.00961 0.00645 0.0233 0.000312 0.00287 0.00964 0.0000815
2L:12531280-12531430:minus -32.9286 -4.17431 9.3578 6.43119 -17.7857 -18.8265 -11.5413 3.61111
5.3945 0.025 0.0354 0.000206 0.00187 0.00676 0.0148 0.00418 0.0047 0.00263
2L:12531280-12531430:plus -21.8163 2.70642 5.6422 6.17431 -17.4898 -18.2347 -21.8716 1.77778 0.816514
0.00244 0.00387 0.00135 0.0022 0.0053 0.0104 0.0288 0.00932 0.015
2L:12534480-12534630:minus -31.7755 -4.40367 0.834862 3.45872 -17.602 -17.5 -15.4771 4.86111
1.45872 0.0157 0.0377 0.00875 0.00731 0.00547 0.00599 0.00985 0.00279 0.0119
2L:12534480-12534630:plus -33.2245 -1.29358 4.36697 2.85321 -17.3061 -7.45918 -20.8991 5.88889 -9.07339
0.0276 0.0157 0.00231 0.00955 0.00389 0.000582 0.0241 0.00175 0.168
2L:12535460-12535610:minus -24.1429 2.99083 -1.86239 5.57798 -27.7041 -9.72449 -18.2569 2.27778
7.42202 0.0074 0.00349 0.0208 0.0034 0.038 0.00352 0.0155 0.0078 0.00106
2L:12535460-12535610:plus -30.9184 -1.23853 -3.91743 2.76147 -17.6429 -9.27551 -12.8165 1.01389 9.92661
0.0103 0.0154 0.0371 0.01 0.00562 0.00244 0.00554 0.0121 0.000193
2L:12540420-12540570:minus -29.9592 -3.62385 13.6422 -0.633028 -27.2653 -16.9796 -19.6881 -3.40278
5.58716 0.00855 0.0306 0.0000116 0.0327 0.0302 0.0044 0.0195 0.0427 0.00237
2L:12540420-12540570:plus -31.8061 4.99083 13.4862 9.59633 -17.4082 -17.9388 -10.3211 -1.83333 -2.94495
0.0158 0.00164 0.0000132 0.000275 0.00421 0.00738 0.00327 0.0285 0.0441
2L:12543280-12543430:minus -22.7449 -0.0458716 1.86239 4.23853 -17.3061 -26.4898 -22.3853 -0.111111
4.36697 0.00404 0.0105 0.00608 0.00522 0.00389 0.0225 0.0316 0.0173 0.00379
2L:12543280-12543430:plus -13.3367 5.44037 11.1101 3.76147 -7.56122 -18.3061 -3.74312 -0.833333
11.8073 0.0008 0.00135 0.0000741 0.00645 0.000419 0.0114 0.000694 0.0215 0.0000538
2L:12544060-12544210:minus -31.5204 -1.12844 -0.908257 7.52294 -27.2347 -17.7857 -17.633 0.972222
7.11009 0.0131 0.0149 0.0155 0.000819 0.0283 0.0068 0.0141 0.0123 0.00128
2L:12544060-12544210:plus -31.2959 2.36697 -0.577982 3.27523 -19.0102 -26.1224 -17.2569 1.79167
11.8532 0.0113 0.00439 0.014 0.00796 0.0157 0.0217 0.0133 0.00928 0.0000428
2L:12545780-12545930:minus -32.2551 -1.25688 2.89908 4.78899 -18.7551 -26.4898 -10.2936 -5.93056
0.495413 0.0184 0.0155 0.00413 0.00456 0.0144 0.0225 0.00325 0.075 0.0162
2L:12545780-12545930:plus -32.8469 0.522936 -0.706422 -3.53211 -26.9694 -27.898 -13.5505 1.47222
11.8532 0.0243 0.00859 0.0146 0.0765 0.023 0.0537 0.00656 0.0104 0.0000428
2L:125460-125610:minus -40.4796 0.761468 -3.77064 -1.66055 -18.449 -26.602 -21.2936 6.27778 -4.08257
0.0407 0.00789 0.0357 0.0456 0.0107 0.0248 0.0259 0.00145 0.0567
2L:125460-125610:plus -40.551 -0.779817 4.56881 7.6422 -8.97959 -18.3469 -15.633 5.43056 0.816514
0.0418 0.0134 0.00213 0.000721 0.0023 0.0124 0.0101 0.00216 0.015
2L:12546200-12546350:minus -31.1429 -2.73394 4.59633 0.93578 -27.1939 -9.34694 -5.94495 5.94444
0.587156 0.0108 0.0239 0.0021 0.0209 0.0267 0.00261 0.00129 0.0017 0.016
2L:12546200-12546350:plus -30.9082 4.50459 4.72477 2.77064 -6.89796 -18.0102 -14.844 -3.83333 -0.293578
0.0103 0.00198 0.00199 0.01 0.000364 0.00812 0.00869 0.0473 0.0207
2L:12546580-12546730:minus -13.5204 0.440367 3.6422 7.61468 -19.0204 9.89796 -2.46789 4.55556
0.495413 0.00097 0.00885 0.00309 0.000779 0.0158 0.0000111 0.000497 0.00318 0.0162
2L:12546580-12546730:plus -31.8469 13.5306 3.31193 2.62385 20.1735 9.30612 -20.2661 5.15278 -2.91743
0.0162 0.0000206 0.00352 0.0108 5.68e-07 0.0000398 0.0216 0.00245 0.0436
2L:125860-126010:minus -22.5918 -3.16514 5.52294 -0.00917431 -18.7551 1.09184 -19.9541 2.09722
-0.486239 0.00365 0.027 0.00142 0.027 0.0144 0.000091 0.0204 0.00832 0.0219
2L:125860-126010:plus -22.5918 -3.45872 6.50459 5.31193 -16.7551 -19.0102 -20 1.22222 -4.55963 0.00365
0.0292 0.000917 0.00393 0.00322 0.0159 0.0206 0.0113 0.0648
2L:12618040-12618190:minus -3.37755 -3.42202 -0.247706 2.54128 -18.6837 -9.30612 -12.367 3.66667
-1.89908 0.0000645 0.0289 0.0126 0.0112 0.0126 0.00257 0.005 0.0046 0.0323
2L:12618040-12618190:plus -31.2143 4.25688 4.00917 7.90826 -27.2347 -17.9388 -3.19266 -1.36111 1.37615
0.0111 0.00218 0.00267 0.000611 0.0283 0.00738 0.000598 0.025 0.0123
2L:12692420-12692570:minus -30.9184 -2.7156 0.504587 1.63303 -27.2653 0.755102 -17.9817 2.27778

9.88073 0.0103 0.0238 0.0098 0.0167 0.0302 0.000175 0.0149 0.0078 0.000206
2L:12692420-12692570:plus -21.9592 2.12844 5.22936 5.29358 -19.0102 -26.9796 -20.7706 -2.19444 1.44037
0.00279 0.00479 0.00162 0.00398 0.0157 0.0306 0.0236 0.0314 0.012
2L:12692880-12693030:minus -13.4082 3.30275 2.80734 2.24771 2.14286 10.0204 -8.47706 3.94444
2.11009 0.000867 0.00311 0.00427 0.0127 0.0000316 0.0000106 0.00232 0.00411 0.00898
2L:12692880-12693030:plus -23.8878 9.98165 3.90826 4.22018 -18.4184 -8.23469 -18.3761 -0.277778
-2.46789 0.00692 0.000134 0.00278 0.00531 0.0101 0.000875 0.0158 0.0182 0.0384
2L:12694960-12695110:minus -22.8878 2.30275 3.63303 1 -8.64286 -8.86735 -14.7339 -0.916667
-2.46789 0.00441 0.00449 0.00311 0.0206 0.00139 0.00198 0.0085 0.022 0.0384
2L:12694960-12695110:plus -12.449 3.62385 8.15596 8.81651 -9.27551 -9.45918 -18.2477 -1 -0.816514
0.000428 0.00276 0.000403 0.000501 0.00284 0.00279 0.0155 0.0226 0.0249
2L:12704480-12704630:minus -12.4388 0.40367 -2.70642 5.90826 -18.4184 -19.0102 -5.07339 0.833333
-1.41284 0.000417 0.00896 0.0265 0.00271 0.0101 0.0159 0.00101 0.0128 0.0289
2L:12704480-12704630:plus -22.7347 -2.09174 6.62385 5.76147 -18.1837 0.540816 -12.3853 0.708333 7.15596
0.00403 0.0199 0.000865 0.00295 0.00835 0.0002 0.00503 0.0134 0.00124
2L:12707720-12707870:minus -14.4082 14.9908 -1.30275 6.54128 -18.7143 -8.72449 -19.2844 1.90278
3.42202 0.00136 2.1e-06 0.0175 0.00173 0.0136 0.00169 0.0183 0.00892 0.00597
2L:12707720-12707870:plus -24 0.302752 9.59633 7.55963 -18.4592 -9.79592 -20.9358 2.36111 -0.174312
0.00719 0.00929 0.00018 0.000807 0.0109 0.00377 0.0243 0.00757 0.0196
2L:12712460-12712610:minus -32.551 -2.9633 2.72477 4.33945 -18.4184 -8.57143 -21.2477 -0.625
5.44037 0.0207 0.0255 0.00441 0.00509 0.0101 0.00149 0.0257 0.0202 0.00259
2L:12712460-12712610:plus -40.7041 3.22936 0.284404 0.486239 -28.5 19.3776 -24.789 3.54167 1.48624
0.0428 0.0032 0.0105 0.0237 0.0585 3.24e-07 0.0459 0.00483 0.0118
2L:12719300-12719450:minus -4.77551 4.27523 2.38532 7.3578 -27.0102 -18.0102 -1.51376 2 3.20183
0.000192 0.00217 0.00501 0.000965 0.0237 0.00812 0.000398 0.00862 0.0065
2L:12719300-12719450:plus -14.6735 2.16514 3.48624 0.651376 -27.2347 -7.5 -11.8532 -0.611111 4.80734
0.00152 0.00473 0.00329 0.0226 0.0283 0.000614 0.00447 0.0201 0.00334
2L:12722180-12722330:minus -30.7041 4.37615 -2.53211 3.18349 -19.0204 -8.72449 -25.789 3.34722
-0.0733945 0.00993 0.00208 0.0252 0.00837 0.0158 0.00169 0.0521 0.00522 0.0188
2L:12722180-12722330:plus -22 2.0367 15.7798 3.97248 -17.3776 -9.5 -15.9358 -2.40278 2.58716 0.00291
0.00495 1.81e-07 0.00587 0.00419 0.00294 0.0107 0.0332 0.00779
2L:12822760-12822910:minus -12.1531 4.49541 -0.40367 9.7156 -8.26531 -18.0816 -2.74312 -3.01389
3.25688 0.000352 0.00199 0.0132 0.000242 0.000934 0.00934 0.000532 0.0388 0.00632
2L:12822760-12822910:plus -21.4388 0.0733945 1.6789 7.62385 -17.0816 -17.7143 -9.02752 2 9.61468
0.002 0.0101 0.0065 0.000759 0.00334 0.00634 0.00257 0.00862 0.00029
2L:12823480-12823630:minus -23.9694 -5.48624 -1 2.68807 -18.7143 -19.1224 -23.5046 -0.805556
-0.0183486 0.0071 0.0501 0.016 0.0104 0.0136 0.0175 0.0381 0.0213 0.0185
2L:12823480-12823630:plus -24.3673 0.266055 1.19266 5.65138 -18.051 -18.3469 -16.8165 3.04167 4.92661
0.00791 0.0094 0.00773 0.00322 0.00746 0.0124 0.0124 0.00587 0.00318
2L:12823940-12824090:minus -23.2551 -1.05505 1.65138 7.17431 -27.1939 -9.16327 -3.51376 -1.01389
5.6055 0.00583 0.0146 0.00656 0.00115 0.0267 0.00223 0.000652 0.0227 0.00232
2L:12823940-12824090:plus -21.9184 0.477064 -1.3211 6.09174 -8.67347 -18.1224 -3.85321 2.95833 5.90826
0.00261 0.00873 0.0176 0.00235 0.00154 0.00988 0.000716 0.00606 0.00198
2L:12837640-12837790:minus -32.8776 6.06422 12.5596 1.72477 -26.9286 1.12245 -11.0917 -0.541667
3.88991 0.0244 0.00103 0.000029 0.0161 0.0214 0.000085 0.0038 0.0197 0.00467
2L:12837640-12837790:plus -32.6224 -0.385321 5.42202 5.22018 -25.7041 -18.2347 -5.48624 -1.55556
1.83486 0.0211 0.0117 0.00149 0.00418 0.0165 0.0104 0.00114 0.0264 0.0101
2L:128600-128750:minus -31.4388 2.49541 1.7156 3.29358 -26.8571 -18.7857 -20.7156 -2.88889 -0.651376
0.0122 0.00418 0.00642 0.00791 0.0198 0.0146 0.0234 0.0376 0.0235
2L:128600-128750:plus -20.6327 -0.972477 6.43119 2.61468 -17.0816 -27.3061 -19.2018 -0.125 1.36697
0.00171 0.0142 0.000949 0.0109 0.00334 0.0336 0.018 0.0174 0.0124
2L:129240-129390:minus -2.84694 -1.3578 2.73394 10.8624 -8.86735 -8.53061 -2.31193 2.29167 7.83486
0.0000385 0.016 0.00439 0.0000687 0.00173 0.00136 0.000478 0.00776 0.000712
2L:129240-129390:plus -31.2143 2.06422 1.52294 4.36697 -26.7041 -18.3469 -16.9725 -1 1.49541 0.0111

0.0049 0.00687 0.00505 0.0193 0.0124 0.0128 0.0226 0.0118
2L:12974200-12974350:minus -14.7041 -4.38532 1.58716 1.97248 11.6531 -8.45918 -16.5321 3.125
9.45872 0.00154 0.0375 0.00672 0.0146 1.55e-06 0.00111 0.0119 0.00569 0.000337
2L:12974200-12974350:plus -20.551 2.21101 2.58716 11.1927 -17.4082 -8.93878 -17.7615 1.26389 3.45872
0.00168 0.00464 0.00465 0.0000254 0.00421 0.00201 0.0144 0.0111 0.00591
2L:12974960-12975110:minus -21.8878 -2.68807 11.2385 7.2844 -9.23469 -17.3469 -19.0183 0.888889
2.25688 0.00258 0.0236 0.0000688 0.00105 0.00277 0.0058 0.0175 0.0126 0.00853
2L:12974960-12975110:plus -24.4796 -2.53211 -1.56881 9.08257 -8.86735 -8.42857 -25.2752 6.02778 -4.90826
0.00804 0.0226 0.019 0.000444 0.00173 0.00104 0.0489 0.00164 0.0714
2L:13000600-13000750:minus -31.8878 9.83486 0.954128 1.99083 -17.4184 -9.57143 -16.6147 -2.01389
2.74312 0.0164 0.000145 0.0084 0.0144 0.00457 0.00326 0.012 0.0299 0.00751
2L:13000600-13000750:plus -24.8163 13.3878 0.321101 2.50459 -27.1939 -8.45918 -9.89908 1.59722 -2.87156
0.0083 0.0000869 0.0104 0.0114 0.0267 0.00111 0.00302 0.00993 0.043
2L:13019120-13019270:minus -32.5204 -0.192661 0.587156 7.27523 -8.67347 1.31633 -12.9083
-0.0833333 -0.834862 0.0204 0.011 0.00952 0.00109 0.00154 0.000066 0.00566 0.0172 0.0249
2L:13019120-13019270:plus -33.4694 6.55046 3.63303 6.57798 -18.9796 -17.5 -21.367 2.29167 -1.9633
0.0285 0.000814 0.00311 0.00167 0.0155 0.00599 0.0263 0.00776 0.0329
2L:13041260-13041410:minus -32.7041 -0.449541 1.93578 4.7156 -17.6735 -18.3061 -17 3.56944
-2.92661 0.0222 0.012 0.00592 0.00464 0.00572 0.0114 0.0128 0.00478 0.0437
2L:13041260-13041410:plus -24.0714 6.20183 1.93578 3.19266 -18.3878 1.05102 -20.156 1.77778 4.04587
0.00731 0.000962 0.00592 0.00829 0.00931 0.000107 0.0212 0.00932 0.00421
2L:13060220-13060370:minus -14.0714 3.61468 5.88073 4.17431 -8.26531 -18.6429 -4.97248 -2.23611
2.58716 0.00117 0.00277 0.00122 0.00537 0.000934 0.0139 0.000985 0.0317 0.00779
2L:13060220-13060370:plus -21.5918 -0.174312 5.62385 4.13761 0.877551 -18.7551 -19.1743 -1.86111
6.7156 0.00209 0.0109 0.00136 0.00546 0.00019 0.0141 0.0179 0.0287 0.00144
2L:13066940-13067090:minus -20.551 -1.09174 -0.550459 5.43119 -8.53061 -8.0102 -12.8532 3.75
-3.33945 0.00168 0.0148 0.0139 0.00371 0.0011 0.000754 0.00559 0.00445 0.0479
2L:13066940-13067090:plus -23.6633 -2.81651 5.81651 6.33028 -18.3469 -19.2755 -16.3578 3.22222 1.2844
0.00665 0.0245 0.00125 0.00198 0.00894 0.0185 0.0115 0.00548 0.0129
2L:13068420-13068570:minus -31.3673 -1.98165 3.15596 4.12844 -7.86735 -18.3469 -12.6697 9.875
1.54128 0.0116 0.0193 0.00374 0.00549 0.000617 0.0124 0.00536 0.000179 0.0115
2L:13068420-13068570:plus -31.5102 5.74312 1.26606 0.798165 -9.16327 -17.2347 -16.8257 2.06944 3.87156
0.013 0.00119 0.00753 0.0218 0.00249 0.00511 0.0125 0.00841 0.00477
2L:13069060-13069210:minus -13.6327 -2.43119 2 1.68807 -8.85714 -17.2347 -16.6881 3.69444 -1.80734
0.00101 0.022 0.00578 0.0164 0.00165 0.00511 0.0122 0.00455 0.0316
2L:13069060-13069210:plus -32.551 2.56881 6.05505 5.19266 -18.2245 -17.7551 -24.422 4.11111 5.44954
0.0207 0.00407 0.00113 0.00422 0.00877 0.00643 0.0436 0.00384 0.00253
2L:13071800-13071950:minus -33.0408 -0.844037 0.559633 2.12844 -27.1939 -17.0102 -16.4037 10.6806
3.01835 0.0264 0.0136 0.00961 0.0134 0.0267 0.00453 0.0116 0.0000985 0.00697
2L:13071800-13071950:plus -31.0714 -3.33945 3.50459 6.06422 -26.2653 -8.72449 -20.211 -1.875 4.88073
0.0106 0.0283 0.00326 0.00239 0.018 0.00169 0.0214 0.0288 0.00324
2L:13073200-13073350:minus -13.4082 4.05102 -0.211009 6.21101 -18.1224 -17.7857 -10.5413 -0.444444
-2.37615 0.000867 0.000335 0.0124 0.00213 0.00782 0.0068 0.00341 0.0192 0.0373
2L:13073200-13073350:plus -32.898 3.83673 -0.513761 3.47706 -25.9694 -19.2755 -7.63303 4.36111
2.09174 0.0247 0.00115 0.0137 0.00724 0.017 0.0185 0.00195 0.00346 0.00904
2L:13074160-13074310:minus -31.1429 0.678899 3.43119 11.1927 -7.37755 -9.5 -16.8624 -1.19444 8.11009
0.0108 0.00813 0.00336 0.0000254 0.000398 0.00294 0.0125 0.0239 0.000597
2L:13074160-13074310:plus -23.8571 4.30275 1.55963 6.00917 -17.1122 -0.204082 -7.86239 0.180556
9.66055 0.00687 0.00214 0.00678 0.00255 0.00341 0.000463 0.00204 0.0158 0.000268
2L:13074820-13074970:minus -23.102 0.761468 7.57798 8.98165 -19.0102 -9.42857 -7.94495 -2.59722
-5.17431 0.00522 0.00789 0.000545 0.000461 0.0157 0.0027 0.00208 0.0349 0.0755
2L:13074820-13074970:plus -13.4388 9.86239 1.62385 2.93578 -8.16327 -19.0816 -20.1835 3.59722 -3.81651
0.000879 0.000143 0.00663 0.00927 0.000873 0.017 0.0213 0.00473 0.0529
2L:13079440-13079590:minus -32.6633 -0.0458716 11.2018 7.45872 -27.2653 -28.1939 -19.3578 5.51389

-2.06422 0.0216 0.0105 0.0000701 0.000894 0.0302 0.0591 0.0185 0.00208 0.0339
2L:13079440-13079590:plus -31.3673 -3.99083 11.4954 9.49541 -27.2347 -18.0102 -17.4587 0.569444 -3.30275
0.0116 0.0337 0.0000592 0.000308 0.0283 0.00812 0.0138 0.014 0.0475
2L:13082940-13083090:minus -41.5612 4.02752 1.42202 5.43119 -17.4898 -8.30612 -18.7798 -1.51389
-2.68807 0.0609 0.00238 0.00712 0.00371 0.0053 0.000973 0.0169 0.0261 0.041
2L:13082940-13083090:plus -23.9286 3.97959 -2.33028 6.09174 -8.64286 -16.7143 -7.14679 3.16667 -0.458716
0.00701 0.000669 0.0238 0.00235 0.00139 0.00419 0.00175 0.0056 0.0217
2L:13083560-13083710:minus -22.0816 4.06422 3.19266 3.6055 -18.6837 -16.7857 -22.9908 -2.40278
-6.47706 0.003 0.00235 0.00369 0.00686 0.0126 0.00426 0.035 0.0332 0.1
2L:13083560-13083710:plus -31.7041 5.79817 1.61468 6.00917 -26.2653 -18.6429 -20.7064 -0.694444
2.16514 0.0146 0.00116 0.00665 0.00255 0.018 0.0139 0.0233 0.0206 0.00879
2L:13085240-13085390:minus -3.67347 -2.13761 0.422018 2.33945 -18.6837 -19.2041 -17.5229 1.52778
5.44037 0.0000972 0.0202 0.0101 0.0122 0.0126 0.0179 0.0139 0.0102 0.00259
2L:13085240-13085390:plus -24.7449 -5.47706 1.77982 7.88991 -17.6429 -17.2755 -19.7706 2.94444 5.33028
0.00827 0.05 0.00627 0.00062 0.00562 0.00544 0.0198 0.00609 0.00273
2L:13105020-13105170:minus -31.7041 -5.77982 3.09174 0.211009 -27.3061 -8.87755 -19.3761 1.91667
1.21101 0.0146 0.054 0.00383 0.0254 0.0315 0.00198 0.0185 0.00888 0.0134
2L:13105020-13105170:plus -23.7449 2.08257 -1.21101 6.21101 -18.7857 -18.6327 -5.73394 3.48611 7.48624
0.00674 0.00487 0.017 0.00213 0.0148 0.0137 0.00122 0.00494 0.00101
2L:13113700-13113850:minus -30.4796 -0.66055 0.825688 2.82569 -18.3776 0.602041 -17.2844 2.27778
-2.17431 0.00941 0.0129 0.00878 0.00976 0.00926 0.000188 0.0134 0.0078 0.035
2L:13113700-13113850:plus -31.7347 -1.27523 -0.110092 5.43119 -17.7857 0.826531 -12.7615 -2 3.88991
0.0149 0.0156 0.012 0.00371 0.00676 0.000153 0.00547 0.0298 0.00467
2L:13165460-13165610:minus -22.1122 -1.6789 -1.45872 1.25688 -18.7551 18.8571 -34.1468 -0.833333
-0.46789 0.00304 0.0177 0.0184 0.0192 0.0144 1.47e-06 0.148 0.0215 0.0218
2L:13165460-13165610:plus -12.8878 -6.56881 0.385321 2.70642 -16.1531 -18.2041 -15.8899 -2.66667 0.348624
0.000523 0.0658 0.0102 0.0103 0.00297 0.0103 0.0106 0.0355 0.0167
2L:13165740-13165890:minus -20.551 1.92661 3.66055 6.01835 -17.5204 -17.7143 -18.5229 -2.54167
-10.2936 0.00168 0.00516 0.00307 0.00248 0.00532 0.00634 0.0162 0.0344 0.205
2L:13165740-13165890:plus -29.1837 -2.07339 -1.87156 4.37615 -18.4184 -16.7143 -15.4771 -2.76389 5.7156
0.00835 0.0198 0.0208 0.00504 0.0101 0.00419 0.00985 0.0364 0.00217
2L:13176880-13177030:minus -13.4796 14.4862 2.22936 3.9633 -8.56122 -9.7551 -9.11009 1.09722
2.93578 0.000935 5.19e-06 0.00531 0.0059 0.00112 0.00358 0.00261 0.0118 0.00717
2L:13176880-13177030:plus -32.6735 1.30275 1.46789 3.23853 -8.96939 -8.42857 -16.2385 -1.11111 4.51376
0.0216 0.00649 0.007 0.00815 0.00226 0.00104 0.0113 0.0233 0.0036
2L:13179120-13179270:minus -42.1122 1.04587 14.8532 1.26606 -19.3163 -9.02041 -26.211 8.06944
-2.18349 0.0698 0.00712 2.28e-06 0.0191 0.0165 0.00213 0.0549 0.000561 0.0351
2L:13179120-13179270:plus -31.7041 3.64286 13.0459 7.27523 -27.7041 -9.0102 -20.7982 -2.83333 -3.82569
0.0146 0.00159 0.0000199 0.00109 0.038 0.00208 0.0237 0.0371 0.0531
2L:13182580-13182730:minus -31.5204 -5.70642 14.4404 7.2844 -27.5306 -16.9796 -18.8073 -2.15278
5.66055 0.0131 0.053 4.12e-06 0.00105 0.0351 0.0044 0.0169 0.0311 0.00227
2L:13182580-13182730:plus -31.551 8.80734 -2.0367 -1.66055 -18.7143 -9.5 -17.4587 3.31944 -4.31193
0.0134 0.000248 0.0219 0.0456 0.0136 0.00294 0.0138 0.00528 0.0604
2L:131900-132050:minus -30.8061 -1.6055 0.284404 4.9633 -17.449 -25.051 -18.1101 1.05556 7.68807
0.0102 0.0173 0.0105 0.0044 0.00484 0.0204 0.0152 0.0119 0.000841
2L:131900-132050:plus -13.2245 5.42202 2.59633 3.10092 -8.56122 -27.9286 -12.2936 0.277778 -0.0733945
0.000738 0.00137 0.00463 0.00865 0.00112 0.054 0.00492 0.0153 0.0188
2L:13190620-13190770:minus -32.4388 -1.72477 -2.94495 9.23853 -8.63265 -8.7551 -21.7798 7.34722
-2.61468 0.0195 0.0179 0.0284 0.000424 0.0013 0.00172 0.0283 0.000838 0.0401
2L:13190620-13190770:plus -34.0408 -0.201835 1.30275 9.61468 -8.64286 -18.5612 -12.0826 5.18056
5.49541 0.0309 0.011 0.00743 0.000255 0.00139 0.0132 0.0047 0.00242 0.0025
2L:13191940-13192090:minus -22.7755 -2.93578 2.81651 2.99083 10.9184 -7.86735 -11.3486 5.73611
1.54128 0.00412 0.0253 0.00426 0.00899 4.19e-06 0.00073 0.00401 0.00188 0.0115
2L:13191940-13192090:plus -34.1122 -0.66055 12.4037 2.54128 20.2041 -8.68367 -18.1927 2.29167 3.93578

0.0312	0.0129	0.0000324	0.0112	3.64e-07	0.00157	0.0154	0.00776	0.00456
2L:13203800-13203950:minus	-29.9184	-0.220183	1.16514	5.86239	-7.89796	-26.602	1.31193	0.875
7.15596	0.00854	0.0111	0.0078	0.00282	0.000678	0.0248	0.000202	0.0127	0.00124
2L:13203800-13203950:plus	-14.1837	4.15596	2.22936	3.46789	-27.602	-18.3469	-10.3945	8.26389	1.07339
0.00124	0.00227	0.00531	0.00726	0.0364	0.0124	0.00332	0.000502	0.014
2L:13205340-13205490:minus	-21.9592	-4.53211	4.44954	4.91743	-18.4898	-26.6327	-2.69725	1.72222
9.88073	0.00279	0.039	0.00224	0.00447	0.0116	0.0251	0.000526	0.0095	0.000206
2L:13205340-13205490:plus	-23.1837	-1.44954	1.68807	0.357798	-27.2347	-36.3061	-14.5872	-8.25	-3.93578
0.00553	0.0165	0.00648	0.0245	0.0283	0.0959	0.00824	0.116	0.0547
2L:13206260-13206410:minus	-22	-2.11927	1.93578	6.84404	-18.6429	-9.72449	-0.0825688	-3.30556
5.44037	0.00291	0.0201	0.00592	0.0014	0.0121	0.00352	0.000289	0.0417	0.00259
2L:13206260-13206410:plus	-30.4388	-1.49541	-2.43119	6.2844	-26.2653	-9.57143	-22.0642	3.52778	4.55963
0.00923	0.0167	0.0245	0.00205	0.018	0.00326	0.0298	0.00486	0.00357
2L:13207740-13207890:minus	-30.3673	-3.16514	7.12844	7.45872	-18.4592	-19.3469	-11.3303	0.402778
-0.46789	0.00897	0.027	0.000683	0.000894	0.0109	0.0194	0.00399	0.0147	0.0218
2L:13207740-13207890:plus	-21.6224	3.80734	-0.284404	6.6422	-16.8571	-8.79592	-12.6972	2.30556
-1.98165	0.00213	0.00259	0.0127	0.00161	0.00324	0.0019	0.00539	0.00772	0.033
2L:13211820-13211970:minus	-24.0714	5.68807	-0.724771	2.61468	-26.2653	-27.898	-4.44037	-4.84722
7.73394	0.00731	0.00122	0.0146	0.0109	0.018	0.0537	0.000846	0.0597	0.000784
2L:13211820-13211970:plus	-31.5102	1.75229	4.20183	10.8624	-18.1939	-9.02041	-6.18349	-0.291667
-1.10092	0.013	0.0055	0.00247	0.0000687	0.0086	0.00213	0.00138	0.0183	0.0268
2L:13219840-13219990:minus	-13.2959	14.4128	-1.3578	5.85321	-18.3776	-27.1224	-13.9174	0.902778
-0.541284	0.000775	5.49e-06	0.0178	0.00287	0.00926	0.0325	0.00712	0.0125	0.0223
2L:13219840-13219990:plus	-22.1531	8.47706	1.49541	9.59633	-17.3469	0.0918367	-19.8716	1.93056
-5.40367	0.0031	0.000294	0.00694	0.000275	0.00394	0.000323	0.0201	0.00883	0.0791
2L:13222480-13222630:minus	-32.5408	3.61468	5.16514	4.10092	-8.63265	19.6429	-15.9633	11.1944
3.09174	0.0204	0.00277	0.00166	0.00554	0.0013	7.32e-08	0.0108	0.0000647	0.00678
2L:13222480-13222630:plus	-33	13.3878	3.78899	1.99083	0.612245	0.0204082	-27.8624	7.90278	-0.12844
0.0258	0.0000869	0.00292	0.0144	0.000269	0.000377	0.0676	0.000616	0.0193
2L:13286620-13286770:minus	-38.9592	-4.68807	-0.440367	-0.66055	-28.3061	-0.0204082	-27.0642
0.444444	7.37615	0.0324	0.0406	0.0134	0.033	0.0553	0.000411	0.061	0.0145	0.00107
2L:13286620-13286770:plus	-24.5918	-1.73394	16.2202	5.31193	-27.1939	0.0918367	-15.0183	-1.34722
0.394495	0.00821	0.018	1.24e-07	0.00393	0.0267	0.000323	0.00901	0.0249	0.0166
2L:13289940-13290090:minus	-31.5102	-5.55963	12.0734	7.29358	-18.4184	-9.57143	3.19266	-2.97222
2.93578	0.013	0.051	0.0000408	0.00104	0.0101	0.00326	0.00011	0.0384	0.00717
2L:13289940-13290090:plus	4.66327	1.74312	12.1743	3.42202	-8.93878	-18.3469	-8.09174	2.84722	-0.807339
0.0000214	0.00552	0.0000382	0.00751	0.00216	0.0124	0.00214	0.00632	0.0247
2L:13290700-13290850:minus	-13.3776	-0.0366972	3.33028	6.11009	-18.9796	-17.8571	-16.7982	6.18056
9.92661	0.000831	0.0104	0.00349	0.00233	0.0155	0.00719	0.0124	0.00152	0.000193
2L:13290700-13290850:plus	-23.1837	1.70642	6.29358	2.77982	-17.4184	-19.0102	-9.02752	3.48611	5.00917
0.00553	0.00559	0.00101	0.00997	0.00457	0.0159	0.00257	0.00494	0.0031
2L:13296360-13296510:minus	-31.1837	-3.34862	5.25688	5.22018	-8.89796	-16.7551	-13.9174	-0.0277778
4.6055	0.0109	0.0284	0.0016	0.00418	0.00189	0.0042	0.00712	0.0169	0.00352
2L:13296360-13296510:plus	-32.2245	4.6055	1.97248	6.11009	-17.3776	-18.2755	-12.633	6.05556	3.77064
0.0183	0.00191	0.00584	0.00233	0.00419	0.0112	0.00532	0.00161	0.00491
2L:13296720-13296870:minus	-23.5204	6.63303	7.78899	7.21101	-9.45918	0.0918367	-7.15596	-0.902778
2.58716	0.00645	0.000782	0.00049	0.0011	0.00287	0.000323	0.00175	0.0219	0.00779
2L:13296720-13296870:plus	-31.9694	-0.899083	9.86239	6.02752	-27.2653	-8.53061	-19.7615	10.4167
-1.57798	0.0168	0.0139	0.000155	0.00246	0.0302	0.00136	0.0198	0.00012	0.0299
2L:13366220-13366370:minus	5.40816	1.66972	6.79817	4.66972	-17.2245	-19.3776	-10.8716	1.20833
1.70642	0.0000106	0.00567	0.000798	0.00466	0.00384	0.0195	0.00364	0.0113	0.0108
2L:13366220-13366370:plus	-34.2347	0.0366972	12.7156	6.78899	-17.4184	0.755102	-20.2844	-1.45833
1.16514	0.0315	0.0102	0.000026	0.00146	0.00457	0.000175	0.0216	0.0257	0.0135
2L:13367500-13367650:minus	-33.1122	-1.91743	1.10092	0.706422	-18.6837	-17.9796	-20.3853	4.15278

-4.94495 0.0271 0.019 0.00798 0.0223 0.0126 0.00764 0.022 0.00377 0.0719
2L:13367500-13367650:plus -32.9286 -0.779817 15.6972 5.57798 -18.3878 0.0510204 -24.6147 0.819444
-4.24771 0.025 0.0134 3.11e-07 0.0034 0.00931 0.000349 0.0448 0.0129 0.0591
2L:13369200-13369350:minus -32.9694 -2.95413 3.00917 0.0550459 -27.5612 -8.65306 -23.4679 -2.11111
8.10092 0.0254 0.0255 0.00396 0.0265 0.0354 0.00156 0.0378 0.0307 0.000623
2L:13369200-13369350:plus -40.551 3.08257 1.56881 5.21101 -18.7857 -18.0816 -14.2661 1.41667 7.68807
0.0418 0.00338 0.00676 0.0042 0.0148 0.00934 0.00769 0.0106 0.000841
2L:13370960-13371110:minus -12.8163 0.0825688 14 4.22018 -26.9694 -9.42857 -4.68807 0.444444
0.348624 0.000489 0.01 7.16e-06 0.00531 0.023 0.0027 0.000908 0.0145 0.0167
2L:13370960-13371110:plus -31.8469 0.155963 -0.990826 -0.834862 -8.37755 -26.898 -5.21101 0.333333
5.6055 0.0162 0.00977 0.0159 0.0349 0.00106 0.0302 0.00105 0.0151 0.00232
2L:13374120-13374270:minus -40.2551 -4.47706 5.70642 2.62385 1.65306 -27.7857 -15.5596 4.20833
1.82569 0.0382 0.0384 0.00131 0.0108 0.0000586 0.0466 0.01 0.00369 0.0101
2L:13374120-13374270:plus -32.5816 -3.68807 5.61468 1.92661 -18.4898 -18.9796 -12.1651 1.52778 2.16514
0.0208 0.0311 0.00137 0.0148 0.0116 0.0155 0.00478 0.0102 0.00879
2L:13378500-13378650:minus -12.6735 15.3303 1.20183 2.97248 -26.898 -8.65306 -20.6514 1.59722
-4.29358 0.000461 1.39e-06 0.0077 0.00909 0.0203 0.00156 0.0231 0.00993 0.06
2L:13378500-13378650:plus -23.7347 2.04587 5.55046 -0.146789 -17.6429 -25.602 -12.2477 0.958333
3.58716 0.00673 0.00494 0.00141 0.028 0.00562 0.0207 0.00487 0.0123 0.00534
2L:13382500-13382650:minus -32.6633 4.90826 -0.211009 -0.146789 -19.051 -7.79592 -25.4037
6.43056 3.52294 0.0216 0.00169 0.0124 0.028 0.0162 0.000723 0.0497 0.00134 0.00558
2L:13382500-13382650:plus -24.449 2.23853 -2.88073 6.83486 -28.2653 1.09184 -24.9174 2.68056 -2.62385
0.00802 0.0046 0.0279 0.00142 0.0537 0.000091 0.0466 0.00673 0.0402
2L:13387300-13387450:minus -13.5918 4.41284 11.6606 2.55046 -17.4082 -18.051 -7.2844 11.3333
7.42202 0.00099 0.00205 0.0000539 0.0112 0.00421 0.00886 0.0018 0.0000573 0.00106
2L:13387300-13387450:plus -41.5204 1.50459 -0.522936 5.47706 -8.93878 -9.86735 -24.6606 1.56944
-4.33028 0.0592 0.00602 0.0137 0.0036 0.00216 0.00391 0.0451 0.01 0.0607
2L:13395080-13395230:minus -30.9286 0.688073 8.31193 6.19266 0.846939 -8.38776 -29.8073 -3.22222
-2.51376 0.0104 0.0081 0.00037 0.00216 0.000197 0.00101 0.0885 0.0408 0.039
2L:13395080-13395230:plus -12.449 -1.74312 0.798165 -2.20183 -18.1531 10.3776 -4.23853 1.01389 1.94495
0.000428 0.018 0.00886 0.0549 0.00829 6.32e-06 0.000798 0.0121 0.00968
2L:13398860-13399010:minus -33.5306 4.36697 -0.220183 4.6422 -27.5306 -7.79592 -30.2569 5
-0.981651 0.0287 0.00209 0.0125 0.00473 0.0351 0.000723 0.0943 0.00263 0.026
2L:13398860-13399010:plus -31.4388 3.53211 2.07339 2.88991 1.10204 -19.2347 2.44037 -3.01389 4.66055
0.0122 0.00286 0.00562 0.00942 0.000124 0.018 0.000143 0.0388 0.0035
2L:13445640-13445790:minus -14.3673 1.98165 -0.486239 6.3578 10.6531 -17.2755 -14.4679 4.44444
0.495413 0.00132 0.00505 0.0136 0.00195 0.0000102 0.00544 0.00803 0.00334 0.0162
2L:13445640-13445790:plus -21.5918 -1.07339 -0.688073 0.321101 -27.1939 -9.27551 -8.75229 0.680556
3.41284 0.00209 0.0147 0.0145 0.0247 0.0267 0.00244 0.00244 0.0135 0.006
2L:134720-134870:minus -40.5918 -2.12844 13.3486 3.89908 -8.67347 -9.72449 -15.0092 -0.875 5.38532
0.0422 0.0202 0.0000145 0.00604 0.00154 0.00352 0.00899 0.0218 0.00267
2L:134720-134870:plus -30.4796 -1.13761 9.69725 3.69725 -17.5306 -17.3469 -12.8349 -0.0972222 3.34862
0.00941 0.015 0.00017 0.00665 0.0054 0.0058 0.00557 0.0172 0.00609
2L:13507800-13507950:minus -21.898 -0.220183 2.74312 6.06422 -17.4592 -8.93878 -25.7339 -7.81944
1.02752 0.00259 0.0111 0.00438 0.00239 0.00491 0.00201 0.0518 0.108 0.0141
2L:13507800-13507950:plus -22.1939 -0.93578 -0.146789 5.48624 -8.63265 -26.8265 -12.5963 -2.19444
5.69725 0.00317 0.014 0.0122 0.00357 0.0013 0.0285 0.00527 0.0314 0.00225
2L:13510000-13510150:minus -30.449 0.972477 1.66055 3.49541 -18.3776 -16.9796 -15.0459 2.59722
-3.61468 0.00925 0.00731 0.00654 0.00716 0.00926 0.0044 0.00906 0.00694 0.0509
2L:13510000-13510150:plus -13.5204 -1.2844 4.33945 9.51376 -17.2245 -17.898 -11.7523 1.59722 2.99083
0.00097 0.0157 0.00234 0.000292 0.00384 0.00726 0.00437 0.00993 0.00706
2L:13511460-13511610:minus -21.7755 -3.79817 4.09174 2.00917 -27.1939 -10.0918 -14.6422 3.125
2.16514 0.00241 0.032 0.00258 0.0142 0.0267 0.00404 0.00834 0.00569 0.00879
2L:13511460-13511610:plus -40.102 -5.31193 2.45872 0.0642202 -17.7143 -9.5 -8.47706 7.02778 4.82569

0.036 0.0479 0.00488 0.0264 0.00627 0.00294 0.00232 0.000991 0.00331
2L:13512740-13512890:minus -41.4388 -0.908257 1.58716 5.55046 -18.4592 -26.8571 -12.9174 3.55556
4.40367 0.0562 0.0139 0.00672 0.00345 0.0109 0.0288 0.00567 0.00481 0.00373
2L:13512740-13512890:plus -21.1837 0.697248 7.14679 4.17431 -18.0816 -28.2653 -9.83486 -6.93056 -0.954128
0.00189 0.00807 0.000677 0.00537 0.00759 0.0595 0.00298 0.0913 0.0258
2L:13513420-13513570:minus -4.36735 2.26606 8.75229 10.7615 -18.4184 -9.34694 -12.1927 -2.16667
8.10092 0.000143 0.00455 0.000289 0.0000826 0.0101 0.00261 0.00481 0.0312 0.000623
2L:13513420-13513570:plus -21.0612 -1.21101 8.04587 6.33945 -17.3776 -26.3776 -3.47706 4.93056 3.17431
0.00187 0.0153 0.000428 0.00196 0.00419 0.0222 0.000645 0.00271 0.00653
2L:13514320-13514470:minus -12.7755 3.83673 1.97248 4.3945 -18.1531 -35.3061 -12.1193 -6.66667
5.24771 0.000477 0.00115 0.00584 0.00499 0.00829 0.0762 0.00473 0.0868 0.00282
2L:13514320-13514470:plus -23.7347 -3.54128 0.981651 5.57798 -27.2245 -27.3367 -19.2844 1.51389 4.92661
0.00673 0.0299 0.00832 0.0034 0.0272 0.0347 0.0183 0.0102 0.00318
2L:13516400-13516550:minus -30.7041 -2.36697 7.47706 6.73394 -18.3776 -8.79592 -7.57798 3.80556
3.46789 0.00993 0.0216 0.000573 0.00152 0.00926 0.0019 0.00192 0.00435 0.00582
2L:13516400-13516550:plus -30.2857 1.11927 12.156 7.2844 -7.82653 -18.7143 -7.85321 -2.95833 -1.37615
0.00887 0.00693 0.0000384 0.00105 0.00059 0.014 0.00204 0.0382 0.0287
2L:13549520-13549670:minus -14.1531 3.98165 7.40367 6.42202 -7.93878 -26.4898 -20.9908 -0.222222
-2.88991 0.00121 0.00242 0.000595 0.00188 0.000767 0.0225 0.0245 0.0179 0.0433
2L:13549520-13549670:plus -24.3265 0.366972 8.98165 3.59633 -27.5306 -9.79592 -18.5413 3.73611 -2.73394
0.00784 0.00908 0.000257 0.00693 0.0351 0.00377 0.0162 0.00447 0.0416
2L:13550060-13550210:minus -32.5918 1.07339 2.43119 3.08257 -18.449 -0.316327 -29.3119 2.58333
2.56881 0.021 0.00705 0.00493 0.0087 0.0107 0.000524 0.0825 0.00698 0.00783
2L:13550060-13550210:plus -22.8061 -0.247706 -0.614679 1.74312 -18.7143 -7.79592 -0.238532
7.31944 5.90826 0.00414 0.0112 0.0141 0.016 0.0136 0.000723 0.000299 0.00085 0.00198
2L:13553740-13553890:minus -21.8878 -3.44954 6.34862 3.27523 -18.2245 -25.8265 -10.0459 4.02778
1.22018 0.00258 0.0292 0.000987 0.00796 0.00877 0.0212 0.0031 0.00397 0.0133
2L:13553740-13553890:plus -24.6327 2.43119 -0.963303 3.23853 -27.2653 -16.3469 -13.8165 3.94444
5.75229 0.00824 0.00428 0.0158 0.00815 0.0302 0.00416 0.00696 0.00411 0.00213
2L:13554780-13554930:minus -31.7041 -0.990826 -0.651376 5.57798 -17.1122 1.12245 -28.3578
-4.88889 -4.7156 0.0146 0.0143 0.0143 0.0034 0.00341 0.000085 0.0722 0.0602 0.0678
2L:13554780-13554930:plus -30.8571 1.52294 5.07339 6.84404 -26.4898 -26.9694 -20.633 1.54167 5.9633
0.0103 0.00598 0.00172 0.0014 0.0183 0.0304 0.023 0.0101 0.00188
2L:13570100-13570250:minus -12.3061 2.62385 2.6055 5.49541 -19.0204 -8.57143 -14.9817 1.55556
-2.88073 0.000385 0.00399 0.00462 0.00355 0.0158 0.00149 0.00894 0.0101 0.0432
2L:13570100-13570250:plus -31.8163 3.64286 -2.3211 6.44037 -8.60204 -17.0102 -20.4954 1.86111 -7.92661
0.016 0.00159 0.0237 0.00185 0.00125 0.00453 0.0225 0.00905 0.133
2L:13571360-13571510:minus -24.2245 3.13761 10.1193 3.90826 -18.7143 -18.8571 -7.36697 5.61111
4.45872 0.00763 0.00331 0.000133 0.00603 0.0136 0.0151 0.00184 0.00199 0.00366
2L:13571360-13571510:plus -23.8878 4.68807 3.72477 5.57798 -27.5306 -7.79592 -19.5505 3.40278 0.33945
0.00692 0.00184 0.003 0.0034 0.0351 0.000723 0.0191 0.00511 0.0168
2L:13571960-13572110:minus -34.1122 -0.311927 0.559633 2.53211 -9.0102 -18.2755 -13.5688 6.30556
9.04587 0.0312 0.0115 0.00961 0.0113 0.00236 0.0112 0.00658 0.00143 0.000423
2L:13571960-13572110:plus -33.0306 -0.633028 -1.91743 2.62385 -18.8265 -8.34694 -11.4587 4.01389
5.3211 0.026 0.0127 0.0211 0.0108 0.0149 0.000993 0.0041 0.004 0.00275
2L:13572580-13572730:minus -34 2.07339 1.61468 7.15596 -18.1531 -18.8571 -13.3486 -3.47222 0.394495
0.0307 0.00488 0.00665 0.00117 0.00829 0.0151 0.00626 0.0434 0.0166
2L:13572580-13572730:plus -24.449 5.93578 5.08257 2.06422 -19.051 -8.45918 -5.06422 3.68056 1.11927
0.00802 0.00109 0.00172 0.0139 0.0162 0.00111 0.00101 0.00457 0.0137
2L:13573780-13573930:minus -32.102 -1.25688 0.348624 -0.834862 -17.7143 -9.5 -15.7706 6.31944
0.422018 0.0177 0.0155 0.0103 0.0349 0.00627 0.00294 0.0104 0.00142 0.0164
2L:13573780-13573930:plus -29.1429 -2.22018 3.05505 5.57798 -18.1939 9.37755 -14.422 -3.75 1.11927
0.00834 0.0207 0.00389 0.0034 0.0086 0.0000363 0.00796 0.0464 0.0137
2L:13576420-13576570:minus -32.6224 2.08257 6 8.88073 -18.3776 -9.72449 -12.3119 -3.44444 1.70642

0.0211 0.00487 0.00115 0.000479 0.00926 0.00352 0.00494 0.0431 0.0108
2L:13576420-13576570:plus -24.2653 5.22936 2.15596 1.6789 -19.3163 0.683673 -19.1743 5.80556 6.11927
0.00773 0.00148 0.00545 0.0165 0.0165 0.000184 0.0179 0.00182 0.00178
2L:13615600-13615750:minus -23.449 2.89908 2.54128 7.99083 -17.7143 -18.3061 -6.05505 -5.88889
6.49541 0.00631 0.00361 0.00473 0.00059 0.00627 0.0114 0.00133 0.0744 0.00154
2L:13615600-13615750:plus -21.5204 3.22018 1.38532 2.57798 1.61224 -27.3367 -0.146789 -7.01389
4.13761 0.00202 0.00321 0.00721 0.011 0.0000631 0.0347 0.000293 0.0928 0.00408
2L:1361700-1361850:minus -23.0714 0.284404 -0.284404 9.61468 -26.9286 -19.0816 -5.66972 8.54167
5.40367 0.0052 0.00935 0.0127 0.000255 0.0214 0.017 0.0012 0.000425 0.0026
2L:1361700-1361850:plus -14.4082 9.92661 1.88073 2.11927 -18.6735 -18.0816 -12.4495 0.944444 0.807339
0.00136 0.000138 0.00604 0.0135 0.0123 0.00934 0.0051 0.0124 0.015
2L:136360-136510:minus -3.47959 -1.12844 7.45872 5.11927 -18.3776 -8.45918 4.16514 0.472222 5.59633
0.0000728 0.0149 0.000579 0.00427 0.00926 0.00111 0.0000777 0.0144 0.00234
2L:136360-136510:plus -23.449 1.48624 4.22018 10.1468 1.94898 -17.3469 -13.6881 1.625 -2.65138
0.00631 0.00606 0.00245 0.000128 0.0000358 0.0058 0.00676 0.00983 0.0406
2L:13640600-13640750:minus -32.7041 1.30275 15.7339 7.29358 -17.7143 -18.0102 -7.84404 6.72222
-4.51376 0.0222 0.00649 2.45e-07 0.00104 0.00627 0.00812 0.00204 0.00116 0.064
2L:13640600-13640750:plus -31.4796 -0.908257 0.889908 -0.752294 -27.602 -18.0102 -19.8073 8.63889
9.3945 0.0128 0.0139 0.00859 0.034 0.0364 0.00812 0.0199 0.000401 0.000358
2L:13641200-13641350:minus -32.8061 2.79817 2.85321 9.75229 -26.1939 9.60204 -15.7156 0.375
4.91743 0.0237 0.00374 0.0042 0.000226 0.0175 0.0000248 0.0103 0.0149 0.00321
2L:13641200-13641350:plus -33.2551 1.34862 -4.20183 5.47706 -26.9286 -9.02041 -24.1284 2.97222 -5.44037
0.0276 0.00637 0.0401 0.0036 0.0214 0.00213 0.0418 0.00603 0.0796
2L:1368100-1368250:minus -31.6327 1.55046 2.44037 3.88991 -8.60204 -27.3061 -18.367 -2.69444 0.697248
0.0138 0.00592 0.00491 0.00608 0.00125 0.0336 0.0158 0.0358 0.0154
2L:1368100-1368250:plus -31.7755 -0.633028 -2.13761 -0.917431 -18.9796 -18.3776 -10.0459 4.33333
0.293578 0.0157 0.0127 0.0225 0.0358 0.0155 0.0125 0.0031 0.0035 0.017
2L:137220-137370:minus -21.7653 -2.20183 0.559633 6.09174 -18.4184 -26.2653 -0.165138 -3.05556
1.06422 0.00237 0.0206 0.00961 0.00235 0.0101 0.0218 0.000294 0.0392 0.014
2L:137220-137370:plus -31.3673 0.816514 1.83486 5.57798 -26 -9.7551 -17.5596 -2.47222 0.587156 0.0116
0.00773 0.00614 0.0034 0.0171 0.00358 0.014 0.0338 0.016
2L:13770840-13770990:minus -41.7041 -1.55963 -1.12844 -4.20183 -28.4592 -0.540816 -25.945 2.45833
0.183486 0.0641 0.017 0.0166 0.0882 0.0568 0.00054 0.0531 0.00731 0.0176
2L:13770840-13770990:plus -40.551 -2.74312 4.11009 1.44037 -18.7857 -8.30612 -26.3211 -1.77778 4.83486
0.0418 0.024 0.00256 0.018 0.0148 0.000973 0.0556 0.0281 0.00329
2L:13780020-13780170:minus -33.0714 3.59633 -1.29358 7.70642 -19.051 -9.60204 -13.7431 2.98611
-1.68807 0.0267 0.00279 0.0175 0.000708 0.0162 0.00334 0.00685 0.006 0.0308
2L:13780020-13780170:plus -14.5204 0.0825688 0.0642202 4.22018 -17.8878 -8.72449 -4.04587 1.95833
6.6422 0.00145 0.01 0.0113 0.00531 0.00704 0.00169 0.000756 0.00875 0.00148
2L:13780560-13780710:minus -41.5204 3.93578 0.522936 4.02752 -17.6735 -0.204082 -28.8165 1.43056
-0.623853 0.0592 0.00246 0.00974 0.00581 0.00572 0.000463 0.0769 0.0105 0.0232
2L:13780560-13780710:plus -31.8469 14.8899 13.5872 3.90826 -17.1531 -18.7857 -6.82569 -1.13889 5.6055
0.0162 2.44e-06 0.0000122 0.00603 0.00364 0.0146 0.00162 0.0235 0.00232
2L:13783440-13783590:minus -4.70408 14.2477 -0.972477 2.00917 -18.6429 -8.23469 -8.56881 5.11111
3.08257 0.000186 6.21e-06 0.0158 0.0142 0.0121 0.000875 0.00236 0.0025 0.00681
2L:13783440-13783590:plus -14.2245 6.51376 -2.36697 -2.40367 -7.86735 -7.72449 -10.8624 6.86111 -4.29358
0.00126 0.000829 0.0241 0.0583 0.000617 0.00068 0.00363 0.00108 0.06
2L:13783940-13784090:minus -39.949 0.706422 -1.70642 4.13761 -17.4184 -18.3061 -19.789 5.13889
0.587156 0.0345 0.00805 0.0198 0.00546 0.00457 0.0114 0.0199 0.00247 0.016
2L:13783940-13784090:plus -3.33673 8.31193 -2.13761 3.09174 -18.449 -17.3469 -22.7156 2.01389 9.3945
0.0000599 0.000323 0.0225 0.00867 0.0107 0.0058 0.0334 0.00858 0.000358
2L:13784240-13784390:minus -23.1531 -4.95413 4.84404 1.26606 -26.5714 -27.4082 -19.4495 2.875
4.36697 0.00542 0.0436 0.0019 0.0191 0.0185 0.0356 0.0188 0.00626 0.00379
2L:13784240-13784390:plus -32.6633 0.366972 2.55046 1.91743 -18.3776 -18.6429 -7.33945 -1.13889 3.97248

0.0216	0.00908	0.00471	0.015	0.00926	0.0139	0.00182	0.0235	0.00444
2L:13790760-13790910:minus	-30.8163	-1.88991	-4.74312	5.65138	-28.8265	-8.5	-20.0917	3.41667	5.24771
0.0103	0.0188	0.0462	0.00322	0.0648	0.00126	0.0209	0.00508	0.00282
2L:13790760-13790910:plus	-24.1531	1.94495	-0.229358	5.57798	10.3571	-9.23469	-16.055	-1.34722
-4.14679	0.00744	0.00512	0.0125	0.0034	0.0000208	0.00238	0.011	0.0249	0.0576
2L:13793440-13793590:minus	-40.5918	1.83486	-0.275229	0.633028	-18.7245	19.1531	-28.0275
-0.0277778	1.95413	0.0422	0.00533	0.0127	0.0229	0.0136	7.95e-07	0.069	0.0169	0.00964
2L:13793440-13793590:plus	-33.1939	3.55046	3.86239	7	11.6939	1.02041	-12.8716	1.20833	4.40367
0.0274	0.00284	0.00283	0.00132	1.17e-06	0.000118	0.00561	0.0113	0.00373
2L:13811440-13811590:minus	-4.92857	13.3878	2.2844	6.21101	-19.051	-8.57143	3.41284	4.90278
-2.52294	0.000212	0.0000869	0.0052	0.00213	0.0162	0.00149	0.000102	0.00274	0.0391
2L:13811440-13811590:plus	-32.6735	-1.89908	-0.715596	-3.26606	10.7245	-8.72449	-24.3303	0.555556
0.908257	0.0216	0.0189	0.0146	0.0727	5.2e-06	0.00169	0.0431	0.014	0.0147
2L:13813740-13813890:minus	-24.0408	-4.3211	0.201835	3.62385	-27.8673	-10.0918	-20.0183	5.94444
9.14679	0.00726	0.0369	0.0108	0.00673	0.0391	0.00404	0.0207	0.0017	0.000414
2L:13813740-13813890:plus	-13.1122	0.256881	-4.23853	5.48624	10.6531	-10.0204	-24.9633	2.84722	-0.146789
0.000632	0.00944	0.0405	0.00357	0.0000102	0.00399	0.0469	0.00632	0.0194
2L:13827840-13827990:minus	-22.7857	-5.91743	4.15596	10.8624	-17.4184	-18.0102	-5.86239	3.19444
3.20183	0.00412	0.0559	0.00251	0.0000687	0.00457	0.00812	0.00126	0.00554	0.0065
2L:13827840-13827990:plus	-22.449	-0.66055	1.6055	5.84404	-18.1122	0.316327	-12.3394	3.40278	5.81651
0.0035	0.0129	0.00667	0.00289	0.00771	0.000243	0.00497	0.00511	0.00204
2L:13829740-13829890:minus	-14.5612	11.0459	-0.651376	2.55046	-27.1224	-8.79592	-22.7431	-2.33333
1.78899	0.00147	0.0000789	0.0143	0.0112	0.0245	0.0019	0.0336	0.0326	0.0103
2L:13829740-13829890:plus	-34.3367	0.477064	2.18349	1.56881	19.9082	-17.898	-14.789	4.11111	5.90826
0.0318	0.00873	0.0054	0.0172	8.13e-07	0.00726	0.00859	0.00384	0.00198
2L:13833140-13833290:minus	-32.7449	-4.0367	3.22018	9.61468	-9.2449	-9.53061	-21.3486	-1.61111
2.17431	0.0228	0.0342	0.00365	0.000255	0.00278	0.0031	0.0262	0.0268	0.00872
2L:13833140-13833290:plus	-13.2551	13.4587	4.90826	0.183486	-16.449	-9.72449	-8.75229	0.0833333
7.24771	0.000751	0.0000146	0.00185	0.0256	0.00307	0.00352	0.00244	0.0163	0.00116
2L:13835040-13835190:minus	-13.6327	13.6697	4.27523	1.78899	-18.6837	-17.8265	-16.156	1.19444
-0.605505	0.00101	0.0000123	0.0024	0.0158	0.0126	0.00699	0.0111	0.0114	0.023
2L:13835040-13835190:plus	-22.7755	-2.66055	-1.26606	-1.6789	-18.7143	9.60204	-15.2294	3.31944	-8.29358
0.00412	0.0235	0.0173	0.0459	0.0136	0.0000248	0.00939	0.00528	0.144
2L:13840340-13840490:minus	-24.1837	4.54128	0.238532	7.00917	-9.27551	-17.3469	-22.2569	7.80556
4.91743	0.00751	0.00195	0.0107	0.00126	0.00284	0.0058	0.0309	0.000651	0.00321
2L:13840340-13840490:plus	-33.7347	-3.91743	-2.09174	1.92661	-26.2245	-18.3469	-9.66972	5.70833	3.98165
0.0294	0.0331	0.0222	0.0148	0.0175	0.0124	0.0029	0.0019	0.00439
2L:13841620-13841770:minus	-22.1531	9.00917	5.73394	2.77064	-8.89796	-8.65306	-21.6972	3.01389
2.95413	0.0031	0.000222	0.0013	0.01	0.00189	0.00156	0.0279	0.00593	0.0071
2L:13841620-13841770:plus	-34.3673	5.20183	4.05505	5.77982	-27.5306	-18.1939	-16.6881	0.638889	9.56881
0.0318	0.0015	0.00262	0.00294	0.0351	0.0101	0.0122	0.0137	0.000303
2L:13878960-13879110:minus	-20.8571	-2.97248	0.981651	0.46789	-18.3776	-9.5	-17.8532	2.72222	-3
0.00178	0.0256	0.00832	0.0239	0.00926	0.00294	0.0146	0.00663	0.0447
2L:13878960-13879110:plus	-22.2245	2.69725	6.31193	11.0183	-18.4184	-17.4898	-9.87156	11.5556	1.73394
0.00325	0.00389	0.001	0.000052	0.0101	0.0059	0.00301	0.0000469	0.0106
2L:13882040-13882190:minus	-24.2653	3.81651	-2.84404	-1.88991	-27.3367	-17.2755	-21.2202	7.63889
3.81651	0.00773	0.00258	0.0276	0.0493	0.0317	0.00544	0.0256	0.000715	0.00482
2L:13882040-13882190:plus	-33.0714	-3.37615	1.95413	3.78899	-26.0408	-18.3469	-13.8807	2.72222	3.46789
0.0267	0.0286	0.00588	0.00632	0.0171	0.0124	0.00706	0.00663	0.00582
2L:13887300-13887450:minus	-22.1837	2.90826	6.73394	3.50459	-27.9286	-18.051	-14.4954	-5.23611
4.78899	0.00315	0.0036	0.000821	0.00713	0.0412	0.00886	0.00808	0.0649	0.00336
2L:13887300-13887450:plus	-32.7041	1.20183	1.66972	7.27523	-26.2653	-17.0102	-20.9174	3.98611
-3.15596	0.0222	0.00673	0.00652	0.00109	0.018	0.00453	0.0242	0.00404	0.0464
2L:13904080-13904230:minus	-32.8163	3.2844	9.23853	5.30275	-27.2653	-9.45918	-18.2477

3.34722 0.963303 0.024 0.00313 0.00022 0.00396 0.0302 0.00279 0.0155 0.00522 0.0145
2L:13904080-13904230:plus -32.898 8.06422 2.70642 7.00917 -17.449 0.0510204 -24.4771 -4.56944
-6.52294 0.0247 0.000369 0.00444 0.00126 0.00484 0.000349 0.0439 0.0561 0.101
2L:13904520-13904670:minus -33.3367 -1.41284 0.926606 6.54128 -17.5204 -27.5612
-14.3119 1.94444 9.88073 0.0281 0.0163 0.00848 0.00173 0.00532 0.0393 0.00777 0.00879 0.000206
2L:13904520-13904670:plus -22.2245 5.0367 7.56881 9.92661 -8.30612 -8.0102 -11.4862 -1.90278
0.816514 0.00325 0.0016 0.000547 0.000169 0.00097 0.000754 0.00413 0.029 0.015
2L:139820-139970:minus -32.7449 0.779817 5.6422 4.08257 -18.4898 -17.898 -21.5138 -3.51389
2.59633 0.0228 0.00784 0.00135 0.00558 0.0116 0.00726 0.027 0.0439 0.00772
2L:139820-139970:plus -32.9286 -1.66972 -3.54128 1 -17.9796 0.530612 -23.7431
-2.76389 -2.01835 0.025 0.0176 0.0335 0.0206 0.00726 0.000203 0.0395 0.0364 0.0334
2L:1406620-1406770:minus -32.4796 0.990826 1.54128 9.59633 -27.4592 -18.2755 -25.789
-4.18056 1.07339 0.02 0.00726 0.00683 0.000275 0.0329 0.0112 0.0521 0.0513 0.014
2L:1406620-1406770:plus -32.7347 -3.40367 2.61468 3.6422 -17.9796 -26.8265 -13.2202
3.44444 4.45872 0.0225 0.0288 0.0046 0.00669 0.00726 0.0285 0.00608 0.00502 0.00366
2L:1420480-1420630:minus -22.8878 8.11927 4.34862 11.1927 -17.3776 -26.5612 -4.52294
-6.79167 3.62385 0.00441 0.000356 0.00233 0.0000254 0.00419 0.0237 0.000866 0.089 0.00523
2L:1420480-1420630:plus -11.7755 4.05102 0.394495 5.85321 -17.3061 -8.79592 -12.2018
-7.76389 5.08257 0.000266 0.000335 0.0102 0.00287 0.00389 0.0019 0.00482 0.106 0.00303
2L:14219660-14219810:minus -33.2959 -3.12844 -3.18349 10.0459 -8.71429 -26.898 -20.9633
-0.805556 2.36697 0.0279 0.0267 0.0304 0.000139 0.00159 0.0302 0.0244 0.0213 0.00828
2L:14219660-14219810:plus -31.551 -1.70642 1.0367 -0.733945 -28.1531 -9.86735 -17.1376
7.40278 5.44954 0.0134 0.0178 0.00816 0.0339 0.0459 0.00391 0.0131 0.000813 0.00253
2L:14220020-14220170:minus -33.9286 -3.34862 2.33945 6.56881 -8.33673 -7.86735 -17.9174
7.20833 -4.76147 0.0303 0.0284 0.0051 0.0017 0.00101 0.00073 0.0148 0.000902 0.0686
2L:14220020-14220170:plus -40.0306 1.04587 1.13761 5.73394 -18.4184 -18.0102 -21.6239
1.13889 -2 0.0357 0.00712 0.00788 0.00307 0.0101 0.00812 0.0275 0.0116 0.0332
2L:14234060-14234210:minus -12.2245 2.23853 7.58716 6.47706 -18.051 -8.16327 -0.733945
0.291667 7.15596 0.000376 0.0046 0.000543 0.00179 0.00746 0.000783 0.000335 0.0153
0.00124
2L:14234060-14234210:plus -22.4694 1.3211 6.3945 3.90826 -27.9388 -37.8878 -1.43119
-1.66667 4.92661 0.0035 0.00644 0.000966 0.00603 0.0415 0.179 0.00039 0.0272 0.00318
2L:14234820-14234970:minus -31.5102 0.954128 6.70642 2.76147 -17.7551 -19.3469 -12.2936
-1.11111 1.9633 0.013 0.00736 0.000832 0.01 0.00658 0.0194 0.00492 0.0233 0.00951
2L:14234820-14234970:plus -31.551 2.90826 10.6147 6.00917 -27.5306 -10.0204 -11.7339 2.27778
-2.57798 0.0134 0.0036 0.0000988 0.00255 0.0351 0.00399 0.00435 0.0078 0.0397
2L:1424860-1425010:minus -32.2653 1.84404 1.37615 0.990826 -17.7551 -18.3469 -23.789
2.36111 -0.743119 0.0184 0.00532 0.00724 0.0206 0.00658 0.0124 0.0398 0.00757 0.0243
2L:1424860-1425010:plus -32.1122 8.00917 0.513761 7.15596 -17.9286 -27.4082 -6.49541
1.72222 7.46789 0.0178 0.00038 0.00977 0.00117 0.0071 0.0356 0.00149 0.0095 0.00102
2L:14262980-14263130:minus -33.0816 0.862385 -1.59633 4.22018 -18.7143 -16.3469
-27.5688 4.52778 -4.47706 0.0268 0.00761 0.0192 0.00531 0.0136 0.00416 0.065 0.00322 0.0636
2L:14262980-14263130:plus -12.3367 0.0917431 0.706422 9.44954 -16.449 -17.9796 4.17431
-5.09722 2.07339 0.000393 0.00999 0.00914 0.000326 0.00307 0.00764 0.0000774 0.063 0.00906
2L:1426560-1426710:minus -31.7347 3.90816 9.38532 -0.46789 0.877551 -7.65306 -17.3761
3.54167 2.9633 0.0149 0.00102 0.000203 0.0309 0.00019 0.000647 0.0136 0.00483 0.00709
2L:1426560-1426710:plus -30.898 7.11927 5.22018 7.00917 -16.4898 -17.2041 -15.9266 9.34722 2.29358
0.0103 0.000611 0.00162 0.00126 0.00312 0.00507 0.0107 0.000256 0.0084
2L:142700-142850:minus -31.6939 -2.05505 -3.65138 11.2936 -18.1531 -18.0102 -10.8349
5.43056 5.0367 0.0142 0.0197 0.0345 0.0000139 0.00829 0.00812 0.00361 0.00216 0.00306
2L:142700-142850:plus -32.7347 3.98165 0.394495 7.63303 -17.6429 -27.602 -15.2202 -0.0138889
-2.48624 0.0225 0.00242 0.0102 0.000745 0.00562 0.0416 0.00937 0.0168 0.0387
2L:1427040-1427190:minus -14.1531 2.04587 1.86239 1.43119 -17.7857 -25.8265 -13.1376
1.48611 7.25688 0.00121 0.00494 0.00608 0.0181 0.00676 0.0212 0.00597 0.0103 0.00115

2L:1427040-1427190:plus -21.8163 5.80734 -0.422018 9.08257 -26.1939 -9.23469 -21.0092
3.02778 -6.06422 0.00244 0.00116 0.0133 0.000444 0.0175 0.00238 0.0246 0.0059 0.0902
2L:143160-143310:minus -22.5918 7.41284 4.3211 3.93578 -18.0816 -18.2347 -19.0092 0.0277778
-2.43119 0.00365 0.000521 0.00235 0.00597 0.00759 0.0104 0.0175 0.0166 0.038
2L:143160-143310:plus -29.6633 5.17431 0.761468 5.69725 -16.449 -17.051 -19.2202 6.47222
-0.201835 0.00847 0.00151 0.00897 0.00314 0.00307 0.00479 0.0181 0.00132 0.0199
2L:14334080-14334230:minus -43.1122 -0.238532 8.0367 -1.84404 -27.9286 -17.0816
-32.789 4.29167 -0.642202 0.0897 0.0112 0.00043 0.0485 0.0412 0.00487 0.129 0.00356 0.0234
2L:14334080-14334230:plus -13.7449 6.34862 1.68807 6.17431 1.10204 0.122449 -16.7339 -3.48611
-2.10092 0.00107 0.000897 0.00648 0.0022 0.000124 0.000312 0.0123 0.0436 0.0342
2L:1434040-1434190:minus -13.6531 6.22018 4.19266 4.31193 -18.2653 -19.0816 -5.83486
-4.09722 4.09174 0.00101 0.000954 0.00248 0.00516 0.00882 0.017 0.00125 0.0504 0.00416
2L:1434040-1434190:plus -13.0408 5.82569 5.15596 6.69725 -26.8571 -25.8265 -3.99083 -2.56944
-2.25688 0.000576 0.00114 0.00167 0.00156 0.0198 0.0212 0.000744 0.0346 0.0357
2L:14348740-14348890:minus -22.1531 -0.119266 14.9633 10.8624 -17.3776 -16.9388 -27.4404
2.80556 -6.37615 0.0031 0.0107 1.77e-06 0.0000687 0.00419 0.00433 0.0639 0.00642 0.0975
2L:14348740-14348890:plus -23.0408 4.3578 2 3.88991 -18.7449 -9.82653 -28.9908 1.55556
-2.11009 0.00512 0.0021 0.00578 0.00608 0.0138 0.00381 0.0788 0.0101 0.0343
2L:1435040-1435190:minus -32.3673 2.77064 -0.724771 10.8624 -18.449 -28.1224 -13.8991
9.36111 12.2752 0.0189 0.00378 0.0146 0.0000687 0.0107 0.0583 0.00709 0.000254 0.0000141
2L:1435040-1435190:plus -23.5612 1.6422 0.59633 11.0183 -17.1122 0.755102 -15.7064 4.61111 -4.00917
0.00653 0.00573 0.00949 0.000052 0.00341 0.000175 0.0103 0.00311 0.0558
2L:14355080-14355230:minus -22.8878 14.6972 5.69725 3.6055 -25.7041 -8.5 -15.9541 0.555556 7.6789
0.00441 3.63e-06 0.00132 0.00686 0.0165 0.00126 0.0108 0.014 0.000866
2L:14355080-14355230:plus -31.4796 -4.08257 2.89908 3.13761 -18.6837 -36.1122 -9.87156 -2.18056 6.17431
0.0128 0.0346 0.00413 0.00854 0.0126 0.0878 0.00301 0.0313 0.00171
2L:14355440-14355590:minus -11.9592 6.31193 -3.16514 4.0367 -8.37755 -7.5 -8.89908 -1.40278 8.97248
0.000301 0.000913 0.0302 0.00578 0.00106 0.000614 0.00251 0.0253 0.000449
2L:14355440-14355590:plus -39.0714 0.513761 8.51376 4.17431 -17.1939 0.163265 -13.0917 0.861111 2.0367
0.0326 0.00862 0.00033 0.00537 0.00377 0.000307 0.0059 0.0127 0.00923
2L:14356320-14356470:minus -32.7755 5.74312 2.80734 3.81651 -28.5306 -19.3469 -14.7064 2.91667
2.75229 0.0234 0.00119 0.00427 0.00626 0.061 0.0194 0.00845 0.00616 0.00748
2L:14356320-14356470:plus -40.2245 -2.15596 1.15596 1.42202 -17.9796 -19.1224 -16.1927 4.125 3.56881
0.0374 0.0203 0.00783 0.0182 0.00726 0.0175 0.0112 0.00382 0.00543
2L:14409600-14409750:minus -22.6633 4.82569 -3.48624 2.9633 -27.2347 -8.79592 -8.0367 3.63889
9.45872 0.00384 0.00175 0.033 0.00914 0.0283 0.0019 0.00212 0.00465 0.000337
2L:14409600-14409750:plus -12.0408 -1.25688 4.6789 8.77982 -17.1122 -8.53061 -8.88073 -5.11111 -4.80734
0.000311 0.0155 0.00203 0.000533 0.00341 0.00136 0.0025 0.0632 0.0694
2L:14490540-14490690:minus -22.0408 -4.3211 9.91743 5.21101 -7.67347 -9.42857 -3.85321 -7.29167
9.66055 0.00296 0.0369 0.00015 0.0042 0.00055 0.0027 0.000716 0.0977 0.000268
2L:14490540-14490690:plus -12.8878 -0.0642202 -0.0642202 7.80734 -17.3776 -17.2755 -11.5413 -5.01389
3.92661 0.000523 0.0105 0.0118 0.00063 0.00419 0.00544 0.00418 0.0619 0.00461
2L:15007340-15007490:minus -24.5612 0.761468 4.82569 2.18349 1.17347 18.8571 -14.2294 8.13889
1.2844 0.00819 0.00789 0.00191 0.0131 0.000101 1.47e-06 0.00763 0.000539 0.0129
2L:15007340-15007490:plus -13.551 -0.321101 1.74312 -0.440367 -18.9796 -8.86735 -16.9083 1.73611
3.45872 0.000974 0.0115 0.00635 0.0306 0.0155 0.00198 0.0126 0.00946 0.00591
2L:15009720-15009870:minus -24.1429 1.11927 15.1193 7.29358 -17.4184 -17.5 -6.43119 6.98611
-2.98165 0.0074 0.00693 1.13e-06 0.00104 0.00457 0.00599 0.00147 0.00101 0.0445
2L:15009720-15009870:plus -13.1122 7.58716 4.90826 6.02752 -8.64286 -27.7041 -11.578 -1.33333 1.90826
0.000632 0.000476 0.00185 0.00246 0.00139 0.0445 0.00421 0.0248 0.00974
2L:1502820-1502970:minus -32.4388 -3.40367 14.4128 6.74312 -8.64286 -17.2755 -10.5413 -1.30556 4.66055
0.0195 0.0288 4.28e-06 0.0015 0.00139 0.00544 0.00341 0.0246 0.0035
2L:1502820-1502970:plus -22.9286 -2.79817 15.0183 1.41284 -18.1531 -19.051 -16.6881 1.36111 2.75229
0.0046 0.0244 1.42e-06 0.0183 0.00829 0.0165 0.0122 0.0108 0.00748

2L:15038820-15038970:minus -13.0408 13.3878 -2.65138 6.21101 -18.1531 -9.23469 -13.2385 1.04167
-3.07339 0.000576 0.0000869 0.0261 0.00213 0.00829 0.00238 0.00611 0.012 0.0454
2L:15038820-15038970:plus -31.8878 0.33945 -0.174312 2.33945 -17.7143 -8.57143 -27.6514 2.19444
-8.68807 0.0164 0.00917 0.0123 0.0122 0.00627 0.00149 0.0657 0.00804 0.156
2L:15039400-15039550:minus -23.8571 13.5306 2.04587 2.77064 -17.449 -9.02041 -15.6239 7.44444
2.99083 0.00687 0.0000206 0.00568 0.01 0.00484 0.00213 0.0101 0.000795 0.00706
2L:15039400-15039550:plus -32.7347 13.5138 -0.587156 11.1927 -18.6735 -9.16327 -14.5321 7.5 2.75229
0.0225 0.0000141 0.014 0.0000254 0.0123 0.00223 0.00814 0.000771 0.00748
2L:15057880-15058030:minus -22.449 6.2844 6.05505 9.33028 1.94898 9.60204 -23.1743 -3.5 3.88991
0.0035 0.000926 0.00113 0.000374 0.0000358 0.0000248 0.0361 0.0437 0.00467
2L:15057880-15058030:plus -21.9592 10.5229 2.41284 6.49541 -19.051 -18.0816 -7.26606 6.61111 1.22936
0.00279 0.000103 0.00496 0.00176 0.0162 0.00934 0.00179 0.00123 0.0132
2L:15060680-15060830:minus -4.67347 0.33945 0.440367 2.66972 10.3571 -17.8265 -19.3853 0.805556
2.73394 0.000182 0.00917 0.01 0.0106 0.0000208 0.00699 0.0186 0.013 0.00756
2L:15060680-15060830:plus -24.1939 -1.3211 4.13761 4.7156 -27.3061 -8.45918 -24.7248 1.66667 5.69725
0.00753 0.0159 0.00254 0.00464 0.0315 0.00111 0.0455 0.00969 0.00225
2L:15061520-15061670:minus -40.8469 1.41284 -3.91743 3.6055 -28.8265 -7.68367 -23.5505 3.33333
-3.89908 0.0437 0.00623 0.0371 0.00686 0.0648 0.000649 0.0383 0.00525 0.0541
2L:15061520-15061670:plus -14.2551 0.981651 1 6.3578 -18.449 9.60204 -7 -1.52778 1.22936 0.00127
0.00729 0.00827 0.00195 0.0107 0.0000248 0.00169 0.0262 0.0132
2L:15067980-15068130:minus -13.9286 -1.88991 -2.70642 -0.844037 -8.27551 -17.7857 -23.0917 -1.83333
3.87156 0.00112 0.0188 0.0265 0.035 0.000951 0.0068 0.0356 0.0285 0.00477
2L:15067980-15068130:plus -14.1837 13.4592 2.33945 2.62385 -27 -8.57143 -16.9266 2.33333 5.48624
0.00124 0.000064 0.0051 0.0108 0.0233 0.00149 0.0127 0.00765 0.00251
2L:15113340-15113490:minus -21.8163 -3.47706 2.6789 6.84404 -18.7143 -8.5 -14.8165 3.81944 -5.00917
0.00244 0.0294 0.00449 0.0014 0.0136 0.00126 0.00864 0.00433 0.0728
2L:15113340-15113490:plus -30.4082 13.5963 3.97248 3.80734 -18.4592 -9.57143 -21.9908 0.805556 -1.22018
0.00908 0.0000131 0.00271 0.00627 0.0109 0.00326 0.0294 0.013 0.0278
2L:15256500-15256650:minus -40.4796 -2.10092 1.92661 3.94495 -9.23469 -0.204082 -23.9908 2.59722
-6.86239 0.0407 0.02 0.00594 0.00595 0.00277 0.000463 0.041 0.00694 0.11
2L:15256500-15256650:plus -32.8878 14.7523 1.56881 0.788991 -27.2653 -18.6429 -20.0642 1.01389 3.62385
0.0247 3.48e-06 0.00676 0.0218 0.0302 0.0139 0.0208 0.0121 0.00523
2L:15264580-15264730:minus -2.29592 1.0367 -1.86239 9.61468 -18.1224 -8.16327 11.4771 -1.875
9.77064 0.000028 0.00714 0.0208 0.000255 0.00782 0.000783 7.17e-06 0.0288 0.000228
2L:15264580-15264730:plus -30.7041 5.24771 -0.00917431 2.97248 -8.40816 -16.051 -20.6881 1.63889
-4.40367 0.00993 0.00147 0.0116 0.00909 0.00108 0.00408 0.0232 0.00978 0.0623
2L:15271580-15271730:minus -24.2653 5.94495 -4.49541 0.174312 -27.3061 -26.4898 -16.9908 3.02778
4.92661 0.00773 0.00108 0.0433 0.0257 0.0315 0.0225 0.0128 0.0059 0.00318
2L:15271580-15271730:plus -30.2245 -1.59633 2.05505 1.58716 -8.33673 -6.5 -12.1468 2.22222 1.9633
0.00881 0.0172 0.00566 0.017 0.00101 0.000542 0.00476 0.00796 0.00951
2L:15271880-15272030:minus -33.6939 7.00917 -3.01835 3.76147 -26.8571 -18.2755 -12.3486 8.38889
-4.33945 0.0291 0.000646 0.029 0.00645 0.0198 0.0112 0.00498 0.000466 0.0609
2L:15271880-15272030:plus -31.398 -3.38532 7.54128 6.73394 -26.8571 -18.1531 -6.34862 3.47222 5.44037
0.0117 0.0287 0.000555 0.00152 0.0198 0.00997 0.00144 0.00497 0.00259
2L:15275040-15275190:minus -13.7041 6.81651 6.3578 9.44954 -19.051 -18.0816 -13.0275 -0.736111
0.697248 0.00104 0.000713 0.000982 0.000326 0.0162 0.00934 0.00582 0.0209 0.0154
2L:15275040-15275190:plus -30.1735 -0.366972 14.1927 7.29358 -18.2245 -18.0816 -16.3394 -3.77778
-3.51376 0.00869 0.0117 5.79e-06 0.00104 0.00877 0.00934 0.0115 0.0467 0.0498
2L:15333640-15333790:minus -23.2959 4.53211 -0.522936 3.57798 -27.5306 0.244898 3.15596 4.02778
-0.0825688 0.00605 0.00196 0.0137 0.00696 0.0351 0.000285 0.000112 0.00397 0.0189
2L:15333640-15333790:plus -22.1837 3.90816 1.44954 8.98165 -17.2245 -18.9286 -9.57798 15.2639 1.16514
0.00315 0.00102 0.00705 0.000461 0.00384 0.0153 0.00285 2.12e-07 0.0135
2L:15498980-15499130:minus -32.551 -1.04587 7.24771 2.55963 -19.2755 9.23469 -22.2018 3.04167
7.68807 0.0207 0.0145 0.000644 0.0111 0.0164 0.0000427 0.0306 0.00587 0.000841

2L:15498980-15499130:plus -22.9184 6.33945 13.3303 7.61468 0.806122 -7.45918 0.559633 6.79167 5.3945
0.00447 0.0009 0.000015 0.000779 0.000229 0.000582 0.000247 0.00112 0.00263
2L:15499500-15499650:minus -21.8163 0.889908 3.22018 2.45872 -26.9694 -8.45918 -8.90826 2.5 9.9633
0.00244 0.00753 0.00365 0.0116 0.023 0.00111 0.00251 0.0072 0.000171
2L:15499500-15499650:plus -22.7755 6.44954 7.7156 7.2844 -27.5714 -18.5 -20.1651 6.06944 12.2752
0.00412 0.000854 0.000509 0.00105 0.0357 0.0131 0.0212 0.0016 0.0000141
2L:15500240-15500390:minus -31.6327 4.11009 -2.54128 5.65138 -16.4184 -17.1224 -19.3578 1.26389
6.18349 0.0138 0.00231 0.0253 0.00322 0.00304 0.00499 0.0185 0.0111 0.00167
2L:15500240-15500390:plus -13.1122 -0.357798 -1.9633 5.89908 -17.7143 -18.0102 -10.789 0.333333
-0.59633 0.000632 0.0116 0.0214 0.00272 0.00627 0.00812 0.00358 0.0151 0.0229
2L:15501020-15501170:minus -33.0714 3.90816 4.80734 8.6055 -26.9694 -8.94898 -11.3853 -0.222222
-0.770642 0.0267 0.00102 0.00193 0.000567 0.023 0.00203 0.00404 0.0179 0.0245
2L:15501020-15501170:plus -30.3673 -1.26606 0.844037 -0.779817 -8.89796 -17.7857 -20.4128 3.375
-0.944954 0.00897 0.0156 0.00872 0.0343 0.00189 0.0068 0.0221 0.00516 0.0257
2L:155300-155450:minus -31.2143 -3.93578 -1.33945 -2.18349 -19.0102 0.204082 2.22936 7.47222 1.10092
0.0111 0.0333 0.0177 0.0545 0.0157 0.000296 0.000153 0.000783 0.0138
2L:155300-155450:plus -32.8061 10.3945 -2.69725 2.66972 -17.7143 -9.23469 -2.52294 4.93056 -1.14679
0.0237 0.000109 0.0265 0.0106 0.00627 0.00238 0.000503 0.00271 0.0272
2L:156040-156190:minus -33.9592 -3.19266 0.174312 5.47706 -8.37755 -8.7551 -10.211 -0.375 1.75229
0.0304 0.0272 0.0109 0.0036 0.00106 0.00172 0.0032 0.0188 0.0104
2L:156040-156190:plus -22.5102 6.36697 0.12844 3.58716 10.6531 0.540816 -12.2385 1.65278 5.00917
0.00354 0.000889 0.0111 0.00694 0.0000102 0.0002 0.00486 0.00974 0.0031
2L:15745040-15745190:minus -21.449 0.486239 -3.54128 0.816514 -17.7143 0.755102 -10.1284 -4.83333
1.84404 0.002 0.00871 0.0335 0.0216 0.00627 0.000175 0.00315 0.0595 0.01
2L:15745040-15745190:plus -31.9592 6.08257 -2.16514 6.36697 -17.1122 -9.30612 -7.34862 -0.875 5.6055
0.0167 0.00102 0.0227 0.00193 0.00341 0.00257 0.00183 0.0218 0.00232
2L:15745620-15745770:minus -33.0408 5.91743 3.50459 11.0275 -18.449 -18.3469 -15.4862 -1.20833
9.14679 0.0264 0.0011 0.00326 0.0000401 0.0107 0.0124 0.00987 0.0239 0.000414
2L:15745620-15745770:plus -23 -3.22018 -2.11009 5.93578 -27.3061 -18.051 -12.6697 9.45833 4.31193
0.00498 0.0274 0.0223 0.00264 0.0315 0.00886 0.00536 0.000238 0.00386
2L:15746260-15746410:minus -21.8163 -1.08257 2.7156 2.19266 -8.60204 -7.72449 -28.2477 5.36111
-2.36697 0.00244 0.0147 0.00442 0.0131 0.00125 0.00068 0.0711 0.00223 0.0371
2L:15746260-15746410:plus -30.1327 -0.192661 2.59633 -1.3578 -17.4082 -9.53061 -19.789 -0.111111
-4.85321 0.0086 0.011 0.00463 0.0415 0.00421 0.0031 0.0199 0.0173 0.0703
2L:15746680-15746830:minus -4.22449 0.623853 5 6.47706 -8.60204 -18.2347 -7.47706 -5.375 -0.247706
0.00013 0.00829 0.00178 0.00179 0.00125 0.0104 0.00188 0.0669 0.0204
2L:15746680-15746830:plus -13.2143 -5.3211 1.22936 -0.954128 -27.0102 -19.2755 -2.88073 0.916667
9.04587 0.000714 0.048 0.00763 0.0362 0.0237 0.0185 0.000551 0.0125 0.000423
2L:15747480-15747630:minus -31.3673 13.4592 2.47706 7.27523 -18.4592 -17.2755 -16.7706 3.33333
3.02752 0.0116 0.000064 0.00484 0.00109 0.0109 0.00544 0.0124 0.00525 0.00695
2L:15747480-15747630:plus -22.551 11.6881 -2.53211 0.733945 -17.6429 -9.7551 -21.1743 1.55556 7.63303
0.00359 0.0000552 0.0252 0.0222 0.00562 0.00358 0.0254 0.0101 0.000928
2L:15748060-15748210:minus -23.4184 5.83486 -1.91743 3.20183 0.612245 -17.9388 -25.7339 -5.51389
0.550459 0.00624 0.00114 0.0211 0.00824 0.000269 0.00738 0.0518 0.0688 0.0161
2L:15748060-15748210:plus -24.1837 5.13761 6.76147 3.27523 1.10204 -18.4898 -18.1468 2.83333 11.8532
0.00751 0.00154 0.000811 0.00796 0.000124 0.0129 0.0153 0.00635 0.0000428
2L:15751040-15751190:minus -22.0408 0.697248 15.4495 6.11009 20.4694 9.53061 -20.3761 6.30556
3.62385 0.00296 0.00807 9.17e-07 0.00233 1.65e-07 0.0000316 0.022 0.00143 0.00523
2L:15751040-15751190:plus -32.7755 1.18349 1.06422 1.22936 -17.3776 9.82653 -17.5688 2.58333 1.14679
0.0234 0.00677 0.00808 0.0193 0.00419 0.0000148 0.014 0.00698 0.0136
2L:15752120-15752270:minus -32.3367 -1.15596 3.61468 2.63303 -9.27551 -9.02041 -10.8716 0.777778 3
0.0187 0.0151 0.00313 0.0107 0.00284 0.00213 0.00364 0.0131 0.00702
2L:15752120-15752270:plus -30.1837 13.3878 1.58716 2.06422 -18.4184 -9.57143 -21.8807 -0.583333
-0.53211 0.00873 0.0000869 0.00672 0.0139 0.0101 0.00326 0.0289 0.02 0.0222

2L:15761640-15761790:minus -32.9286 2.53211 1.62385 5.73394 -18.3776 -7.57143 -8.63303 6.31944
-2.87156 0.025 0.00413 0.00663 0.00307 0.00926 0.000644 0.00239 0.00142 0.043
2L:15761640-15761790:plus -31.8061 3.79817 1.26606 2.44037 -8.60204 -18.2041 -2.81651 2.02778 5.13761
0.0158 0.00259 0.00753 0.0116 0.00125 0.0103 0.000542 0.00853 0.00298
2L:15762640-15762790:minus -13.449 4.29358 0.908257 9.44037 -26.898 -16.9388 4.3211 -1.31944
6.11927 0.000914 0.00215 0.00853 0.000339 0.0203 0.00433 0.0000734 0.0247 0.00178
2L:15762640-15762790:plus -29.102 0.954128 11.0367 7.2844 -26.5918 -18.4184 -10.6972 3 3.21101
0.00833 0.00736 0.0000778 0.00105 0.0186 0.0127 0.00352 0.00597 0.00644
2L:15768200-15768350:minus -23.3367 0.293578 11.4312 1.63303 -7.71429 -18.7857 -8.84404 1.88889
3.92661 0.00614 0.00932 0.0000613 0.0167 0.000572 0.0146 0.00248 0.00896 0.00461
2L:15768200-15768350:plus -14.7449 14.5688 11.7798 7.29358 -18.1531 -18.3469 -12.7798 3.76389 7.15596
0.00157 4.39e-06 0.0000501 0.00104 0.00829 0.0124 0.0055 0.00442 0.00124
2L:158940-159090:minus -32.2245 -1.58716 5.22936 9.33028 -27.9592 -17.5 -14.1927 1.80556 2.99083
0.0183 0.0172 0.00162 0.000374 0.0416 0.00599 0.00757 0.00923 0.00706
2L:158940-159090:plus -21.398 -1.01835 -3.57798 6 10.6531 -8.79592 -4.38532 -2.26389 5.70642 0.00197
0.0144 0.0339 0.00256 0.0000102 0.0019 0.000832 0.032 0.00222
2L:159540-159690:minus -32.7347 6.83486 2.36697 5.74312 -18.3061 -0.469388 -21.1101 -2.75
-10.367 0.0225 0.000707 0.00505 0.00302 0.00884 0.000533 0.0251 0.0363 0.208
2L:159540-159690:plus -12.4082 1.12844 -0.688073 9.3211 -8.23469 -9.82653 -1.85321 3.69444 1.6422
0.000414 0.00691 0.0145 0.000391 0.000927 0.00381 0.000429 0.00455 0.0111
2L:160140-160290:minus -33.1939 3.64286 -1.63303 0.752294 -9.45918 -18.051 -20.0275 4.43056 -4.46789
0.0274 0.00159 0.0194 0.0221 0.00287 0.00886 0.0207 0.00336 0.0635
2L:160140-160290:plus -23.3367 -0.495413 8.12844 7.21101 -18.1531 -18.898 -18.2477 -2.375 3.24771
0.00614 0.0122 0.000408 0.0011 0.00829 0.0152 0.0155 0.0329 0.00636
2L:160740-160890:minus -32 2.7156 4.61468 6.44954 -8.60204 0.755102 -19.156 3.51389 1.02752
0.0171 0.00386 0.00209 0.00183 0.00125 0.000175 0.0179 0.00489 0.0141
2L:160740-160890:plus -21.7347 0.458716 -1.05505 2.55046 -27.0102 -26.898 -19.9266 9.65278 6.29358
0.00235 0.00879 0.0162 0.0112 0.0237 0.0302 0.0203 0.000209 0.00161
2L:1611160-1611310:minus -13.3673 0.844037 13.3945 7.29358 -8.64286 -7.72449 -8.87156 -1.09722 -4.9633
0.000813 0.00766 0.0000143 0.00104 0.00139 0.00068 0.0025 0.0232 0.0722
2L:1611160-1611310:plus -32.2653 0.486239 10.5229 1.91743 -26.9694 -10.0918 -11.7706 -1.77778 -3.73394
0.0184 0.00871 0.000104 0.015 0.023 0.00404 0.00439 0.0281 0.052
2L:1614060-1614210:minus -30.449 1.6055 0.990826 7.02752 20.4694 -9.23469 -8.3945 -1.29167 3.34862
0.00925 0.0058 0.00829 0.00122 1.65e-07 0.00238 0.00228 0.0245 0.00609
2L:1614060-1614210:plus -22.9286 3.54128 0.614679 2.06422 -17.3776 -8.42857 -15.7431 0.805556 2.17431
0.0046 0.00285 0.00943 0.0139 0.00419 0.00104 0.0104 0.013 0.00872
2L:161600-161750:minus -12.8571 4.46789 0.33945 11.2936 -17.4184 0.0204082 -13.3945 -0.861111
1.19266 0.000505 0.00201 0.0104 0.0000139 0.00457 0.000377 0.00633 0.0217 0.0134
2L:161600-161750:plus -32.8776 -0.522936 1.51376 0.12844 -28.2653 -18.4184 -15.3945 5.25 2.38532
0.0244 0.0123 0.00689 0.0259 0.0537 0.0127 0.0097 0.00235 0.00822
2L:16250840-16250990:minus -31.8878 0.798165 0.972477 5.86239 -26.1939 9.08163 -16.7339 5.54167
-0.642202 0.0164 0.00779 0.00835 0.00282 0.0175 0.0000431 0.0123 0.00205 0.0234
2L:16250840-16250990:plus -33.0714 -0.119266 -2.43119 1.7156 -17.3776 -8.79592 -22.367 7.5 1.22018
0.0267 0.0107 0.0245 0.0162 0.00419 0.0019 0.0315 0.000771 0.0133
2L:16251720-16251870:minus -30.5204 7.6055 2.43119 7.15596 -18.3776 -28.4082 -11.6972 3.90278
10.1284 0.00951 0.000472 0.00493 0.00117 0.00926 0.0615 0.00432 0.00418 0.000129
2L:16251720-16251870:plus -29.9184 0.00917431 -2.12844 3.44037 -17.5918 -17.8571 -11.5596 0.388889
11.8532 0.00854 0.0103 0.0224 0.00746 0.00545 0.00719 0.00419 0.0148 0.0000428
2L:16255340-16255490:minus -31.9592 0.651376 0.788991 5.30275 -17.4592 -9.60204 -15.9633 -2.40278
1.48624 0.0167 0.00821 0.00889 0.00396 0.00491 0.00334 0.0108 0.0332 0.0118
2L:16255340-16255490:plus -21.8878 8.30275 -2.6055 3.95413 -18.7143 -16.0102 -6.90826 0.791667 3.29358
0.00258 0.000324 0.0258 0.00593 0.0136 0.00406 0.00165 0.013 0.00623
2L:16257900-16258050:minus -33.1531 3.42202 -4.44954 5.29358 -17.5612 -8.57143 -23.9358 2.97222
2.33028 0.0273 0.00298 0.0428 0.00398 0.00544 0.00149 0.0407 0.00603 0.00836

2L:16257900-16258050:plus -31.6224 4.21101 2.88991 5.02752 -18.9796 -18.2755 -17.9817 -1.65278 2.3211 0.0137 0.00222 0.00414 0.00435 0.0155 0.0112 0.0149 0.0271 0.00839
2L:16261660-16261810:minus -32.7653 7.74312 1.42202 4.04587 -8.60204 -19.1531 -10.8073 3.97222 5.18349 0.0228 0.000438 0.00712 0.00572 0.00125 0.0177 0.00359 0.00407 0.00295
2L:16261660-16261810:plus -22.9286 4.33028 4.66055 5.86239 -17.1531 -18.051 -6.6789 8.38889 1.53211 0.0046 0.00212 0.00205 0.00282 0.00364 0.00886 0.00156 0.000466 0.0116
2L:16261960-16262110:minus -21.2245 4.33028 5.47706 5.37615 -18.0102 -17.7551 -13.8165 11.0833 1.97248 0.0019 0.00212 0.00145 0.00384 0.00732 0.00643 0.00696 0.0000712 0.00948
2L:16261960-16262110:plus -22.2245 0.192661 5.18349 7.47706 20.2041 0.826531 -21.5963 1.52778 -6.22018 0.00325 0.00965 0.00165 0.00085 3.64e-07 0.000153 0.0274 0.0102 0.0938
2L:16269660-16269810:minus -24.1837 0.0917431 12.0092 7.2844 -18.4898 -27.3776 -14.6972 4.48611 11.8532 0.00751 0.00999 0.0000428 0.00105 0.0116 0.0351 0.00843 0.00328 0.0000428
2L:16269660-16269810:plus -21.5918 4.69725 12.6606 1.42202 -17.449 -9.08163 -21.3303 14.1944 -0.651376 0.00209 0.00184 0.000027 0.0182 0.00484 0.00216 0.0261 1.94e-06 0.0235
2L:16274220-16274370:minus -21.6633 2.14679 -0.807339 3.6422 -18.6837 -8.16327 -8.68807 0.652778 1.84404 0.00223 0.00476 0.015 0.00669 0.0126 0.000783 0.00241 0.0136 0.01
2L:16274220-16274370:plus -22.8878 14.3394 2.66972 2.62385 -18.4592 -18.9796 -9.74312 -3.16667 1.79817 0.00441 5.96e-06 0.0045 0.0108 0.0109 0.0155 0.00293 0.0403 0.0102
2L:16279100-16279250:minus -14.1122 0.541284 4.83486 6.13761 -18.7551 -26.6735 -5.94495 1.16667 8.19266 0.00119 0.00854 0.00191 0.00226 0.0144 0.0256 0.00129 0.0115 0.000548
2L:16279100-16279250:plus -31.7041 5.90826 -0.513761 1.7156 -9.20408 0.755102 -24.6881 3.41667 -3.78899 0.0146 0.0011 0.0137 0.0162 0.00262 0.000175 0.0452 0.00508 0.0526
2L:16279600-16279750:minus -32.4388 -1.52294 -3.20183 2.18349 -18.051 -17.051 -18.8899 0.222222 3.94495 0.0195 0.0169 0.0305 0.0131 0.00746 0.00479 0.0171 0.0156 0.00452
2L:16279600-16279750:plus -23.8163 -4.53211 5.01835 2.68807 -9.16327 -17.7143 -12.4495 7.15278 5.29358 0.00682 0.039 0.00176 0.0104 0.00249 0.00634 0.0051 0.000928 0.00278
2L:16280060-16280210:minus -32.9694 1.00917 -0.192661 2.19266 -18.7143 -17.4184 -21.1376 0.888889 -5.14679 0.0254 0.00722 0.0123 0.0131 0.0136 0.00585 0.0252 0.0126 0.0751
2L:16280060-16280210:plus -20.102 1.43119 0.66055 6.3578 -18.1224 -8.16327 -17.6789 -5.77778 -8.95413 0.00164 0.00619 0.00929 0.00195 0.00782 0.000783 0.0142 0.0727 0.165
2L:16284720-16284870:minus -32.9286 -1.08257 4.06422 -1.22936 -17.1837 -26.6327 -10.8716 2.73611 -3.12844 0.025 0.0147 0.00261 0.0395 0.00366 0.0251 0.00364 0.00659 0.046
2L:16284720-16284870:plus -22.6735 -0.605505 3.19266 7.43119 -17.5204 -8.72449 -5.6055 1.18056 2.20183 0.00385 0.0126 0.00369 0.000939 0.00532 0.00169 0.00118 0.0114 0.00869
2L:16285440-16285590:minus -31.7755 -2.51376 2.95413 6.29358 -18.7551 -17.3469 -17.3119 6.69444 -4.70642 0.0157 0.0225 0.00404 0.00202 0.0144 0.0058 0.0135 0.00118 0.0676
2L:16285440-16285590:plus -32.6633 0.816514 7.45872 4.3211 -27.3061 -8.86735 -8.82569 7.59722 1.49541 0.0216 0.00773 0.000579 0.00514 0.0315 0.00198 0.00248 0.000731 0.0118
2L:16286060-16286210:minus -32.9286 -0.192661 10.9083 7.29358 -17.7143 -17.5 -16.6422 -0.458333 -3.87156 0.025 0.011 0.0000846 0.00104 0.00627 0.00599 0.0121 0.0192 0.0537
2L:16286060-16286210:plus -32.6633 -2.93578 4.22936 1.92661 -28.602 -19.3061 -9.49541 1.56944 11.8532 0.0216 0.0253 0.00244 0.0148 0.0631 0.0187 0.0028 0.01 0.0000428
2L:16286700-16286850:minus -40.8878 -0.623853 8.42202 5.73394 -17.5306 -8.72449 -24.1284 2.13889 -1.33945 0.0439 0.0127 0.000348 0.00307 0.0054 0.00169 0.0418 0.0082 0.0285
2L:16286700-16286850:plus -40.6327 -2.83486 0.486239 1.14679 -17.4592 0.346939 -24.8624 -1.09722 3.99083 0.0424 0.0246 0.00986 0.0198 0.00491 0.000221 0.0463 0.0232 0.00433
2L:16287440-16287590:minus -23 -1.45872 5.40367 1.62385 -18.4082 -18.3469 -5.70642 0.708333 7.73394 0.00498 0.0165 0.0015 0.0168 0.00937 0.0124 0.00121 0.0134 0.000784
2L:16287440-16287590:plus -22.551 1.77064 8.55963 6.12844 -27.9286 -26.602 -12.4954 3.375 1.44037 0.00359 0.00546 0.000322 0.00231 0.0412 0.0248 0.00515 0.00516 0.012
2L:16288100-16288250:minus -31.9286 6.23853 0.137615 3.49541 -18.0102 -9.82653 -22.4862 3.33333 1.33028 0.0166 0.000946 0.0111 0.00716 0.00732 0.00381 0.0321 0.00525 0.0127
2L:16288100-16288250:plus -31.5918 2.50459 -3.07339 1.44954 1.21429 -0.27551 -15.6881 2.43056 -2.22018 0.0136 0.00417 0.0294 0.0179 0.0000864 0.000502 0.0102 0.00738 0.0355

2L:16299740-16299890:minus -40.4082 1.34862 10.1193 2.72477 -19.3163 -8.42857 -21.5505 0.652778
-0.926606 0.0399 0.00637 0.000133 0.0102 0.0165 0.00104 0.0272 0.0136 0.0256
2L:16299740-16299890:plus -33.4388 0.633028 1.88073 4.91743 -18.1939 -19.0102 -13.6881 3.45833 -2.04587
0.0284 0.00826 0.00604 0.00447 0.0086 0.0159 0.00676 0.005 0.0336
2L:16302800-16302950:minus -30.6327 -0.623853 0.0366972 3.34862 10.9898 -17.5 -22.0826
-0.416667 -1.6422 0.00968 0.0127 0.0115 0.0077 3.28e-06 0.00599 0.0299 0.019 0.0304
2L:16302800-16302950:plus -32.9388 -2.62385 -0.211009 0.330275 -18.449 -8.65306 -21.7156 -1.70833
1.43119 0.025 0.0232 0.0124 0.0246 0.0107 0.00156 0.028 0.0275 0.0121
2L:16303120-16303270:minus -30.6327 -0.623853 0.0366972 3.34862 10.9898 -17.5 -22.0826
-0.416667 -1.6422 0.00968 0.0127 0.0115 0.0077 3.28e-06 0.00599 0.0299 0.019 0.0304
2L:16303120-16303270:plus -22.6633 0.66055 -1.78899 -0.926606 -18.3776 -9.72449 -21.4587 -0.0138889
9.14679 0.00384 0.00818 0.0203 0.036 0.00926 0.00352 0.0267 0.0168 0.000414
2L:16307140-16307290:minus -23.2551 14.2294 -4.0367 6.56881 -18.3469 0.0510204 -7.55963 0.708333
-2.43119 0.00583 6.48e-06 0.0384 0.0017 0.00894 0.000349 0.00192 0.0134 0.038
2L:16307140-16307290:plus -24.0816 -0.972477 4.2844 3.94495 -28.5714 -9.79592 -26.4862 -5.52778
3.45872 0.00732 0.0142 0.00239 0.00595 0.0623 0.00377 0.0568 0.069 0.00591
2L:16310700-16310850:minus -29.1429 2.21101 12.2661 4.49541 -17.8878 -19.6429 -9.77064 2.54167
0.651376 0.00834 0.00464 0.0000357 0.00487 0.00704 0.0204 0.00295 0.00709 0.0156
2L:16310700-16310850:plus -31.8776 2.69725 2.20183 2.88991 20.1735 -18.3776 -5.15596 1.79167 -1.22018
0.0163 0.00389 0.00536 0.00942 5.68e-07 0.0125 0.00104 0.00928 0.0278
2L:16315080-16315230:minus -23.5612 1.81651 4.29358 5.49541 -19.0918 -9.45918 -15.5872 3.43056
-2.26606 0.00653 0.00537 0.00238 0.00355 0.0163 0.00279 0.0101 0.00505 0.0359
2L:16315080-16315230:plus -14.5918 -3.14679 1.53211 -0.0366972 -8.67347 -17.1224 -12.5505 3.59722
3.05505 0.00148 0.0268 0.00685 0.0271 0.00154 0.00499 0.00522 0.00473 0.00692
2L:16325080-16325230:minus -31.2143 3.12844 3.19266 4.17431 -8.63265 -17.8571 -3.69725 1.16667
3.40367 0.0111 0.00332 0.00369 0.00537 0.0013 0.00719 0.000685 0.0115 0.00603
2L:16325080-16325230:plus -24.5918 -0.642202 -0.0917431 2.34862 -27.602 -8.79592 -6.36697 5.09722
1.90826 0.00821 0.0128 0.0119 0.0121 0.0364 0.0019 0.00144 0.00251 0.00974
2L:16325900-16326050:minus -24.4082 5.41284 2.06422 6.78899 -7.53061 0.387755 -13.4954 5.66667
-5.97248 0.00797 0.00137 0.00564 0.00146 0.000407 0.000217 0.00648 0.00194 0.0883
2L:16325900-16326050:plus -22.1531 -1.80734 1.77982 1.38532 -26.9286 -16.2755 -9.12844 1.55556 5.59633
0.0031 0.0184 0.00627 0.0185 0.0214 0.00413 0.00262 0.0101 0.00234
2L:16329100-16329250:minus -23.6327 15.3303 0.0733945 1.78899 -18.9796 -18.3061 -7.22936 2.5
-0.229358 0.00661 1.39e-06 0.0113 0.0158 0.0155 0.0114 0.00178 0.0072 0.0202
2L:16329100-16329250:plus -24.7857 13.5306 -1.87156 1.99083 -8.96939 -26.6327 -19.3303 -1.08333 -4.54128
0.00829 0.0000206 0.0208 0.0144 0.00226 0.0251 0.0184 0.0231 0.0645
2L:16350260-16350410:minus -21.6224 0.12844 11.2294 2.6789 -18.4184 -17.9796 -12.1927 -8.20833
1.02752 0.00213 0.00987 0.000069 0.0105 0.0101 0.00764 0.00481 0.115 0.0141
2L:16350260-16350410:plus -3.67347 -1.40367 7.09174 0.293578 -7.71429 -8.45918 -15.2385 0.0972222
-0.275229 0.0000972 0.0163 0.000696 0.0248 0.000572 0.00111 0.00941 0.0162 0.0206
2L:16350840-16350990:minus -30.398 1.74312 1.69725 2.33945 -27.3776 -9.53061 3.2844 9.26389
7.42202 0.00901 0.00552 0.00646 0.0122 0.032 0.0031 0.000107 0.00027 0.00106
2L:16350840-16350990:plus -31.2143 0.541284 0.486239 5.22936 -8.93878 -35.8878 -19.4495 0.347222 5.70642
0.0111 0.00854 0.00986 0.0041 0.00216 0.0822 0.0188 0.015 0.00222
2L:16351300-16351450:minus -23.3367 7.00917 3.81651 2.62385 -19.3163 -9.79592 -14.5596 4.52778
3.49541 0.00614 0.000646 0.00289 0.0108 0.0165 0.00377 0.00819 0.00322 0.0057
2L:16351300-16351450:plus -31.7347 -3.56881 1.6789 1.91743 -8.0102 -25.4898 -6.48624 6.51389 3.77982
0.0149 0.0301 0.0065 0.015 0.000826 0.0205 0.00149 0.00129 0.00489
2L:16351720-16351870:minus -23.102 0.137615 -0.284404 6.00917 -27.3367 -8.68367 -2.20183 3.69444
7.22018 0.00522 0.00984 0.0127 0.00255 0.0317 0.00157 0.000466 0.00455 0.00119
2L:16351720-16351870:plus -32.0714 3.3578 2.44037 4.79817 -26.5306 -19.0816 -18.1284 0.111111 1.66972
0.0176 0.00305 0.00491 0.00453 0.0185 0.017 0.0152 0.0162 0.011
2L:16352080-16352230:minus -39.8776 -1.18349 4.27523 7.72477 -16.7143 -18.0816 -16.6789 3.09722
-2.72477 0.0341 0.0152 0.0024 0.000681 0.00319 0.00934 0.0122 0.00575 0.0414

2L:16352080-16352230:plus -38.9184 3.62385 -1.25688 4.22018 -18.9388 -8.5 -18.8073 -4.13889 -4.66055 0.0323 0.00276 0.0173 0.00531 0.0151 0.00126 0.0169 0.0508 0.0667
2L:16352760-16352910:minus -23.5204 14.945 0.0917431 9.41284 -8.33673 -19.1531 -21.633 3.65278 1.37615 0.00645 2.29e-06 0.0112 0.000354 0.00101 0.0177 0.0276 0.00462 0.0123
2L:16352760-16352910:plus -2.70408 13.4592 -1.09174 2.05505 -27.1224 -18.2041 -12.4771 -1.83333 4.09174 0.0000372 0.000064 0.0164 0.0139 0.0245 0.0103 0.00513 0.0285 0.00416
2L:16356480-16356630:minus -12.1837 0.944954 1.88073 4.14679 -19.051 -9.27551 -7.89908 -1.11111 3.87156 0.000365 0.00739 0.00604 0.00542 0.0162 0.00244 0.00206 0.0233 0.00477
2L:16356480-16356630:plus -29.9184 12.9174 -5.47706 4.14679 -26.9694 -9.7551 -9.7156 3.88889 5.70642 0.00854 0.0000238 0.0557 0.00542 0.023 0.00358 0.00292 0.00421 0.00222
2L:16356780-16356930:minus -31.551 3.90816 -5.06422 1.26606 -18.3776 -9.94898 -15.0183 8.76389 5.29358 0.0134 0.00102 0.0502 0.0191 0.00926 0.00393 0.00901 0.000371 0.00278
2L:16356780-16356930:plus -31.1429 12.9174 6.07339 4.14679 -18.7551 -9.7551 -25.5046 1.125 -0.605505 0.0108 0.0000238 0.00112 0.00542 0.0144 0.00358 0.0503 0.0117 0.023
2L:16442100-16442250:minus -23 -1.48624 1.04587 0.0733945 -26.9694 -9.42857 -2.44954 2.125 3.24771 0.00498 0.0167 0.00813 0.0263 0.023 0.0027 0.000494 0.00824 0.00636
2L:16442100-16442250:plus -30.4388 5.65138 -1.26606 2.98165 -18.4184 -7.45918 -19.7156 0.180556 0.816514 0.00923 0.00124 0.0173 0.00905 0.0101 0.000582 0.0196 0.0158 0.015
2L:16442540-16442690:minus -24.4082 4.15596 6.97248 0.633028 -18.7551 -18.5714 -3.63303 3.05556 5.18349 0.00797 0.00227 0.000737 0.0229 0.0144 0.0136 0.000673 0.00584 0.00295
2L:16442540-16442690:plus -33.3265 -0.201835 1.59633 5.57798 -26.898 -18.5 -18.8532 1.93056 -6.08257 0.0279 0.011 0.00669 0.0034 0.0203 0.0131 0.017 0.00883 0.0907
2L:16445060-16445210:minus -31.8469 -0.293578 -3.69725 0.220183 -27.4592 0.826531 -7.9633 0.347222 -0.366972 0.0162 0.0114 0.035 0.0253 0.0329 0.000153 0.00209 0.015 0.021
2L:16445060-16445210:plus -31.9694 -0.66055 4.57798 10.8624 -26.898 -9.5 -23.1101 6.26389 3.40367 0.0168 0.0129 0.00212 0.0000687 0.0203 0.00294 0.0357 0.00146 0.00603
2L:16448160-16448310:minus -31.9694 3.97959 -1.23853 7.27523 -27.2653 -17.3469 -24.5046 0.486111 -1.05505 0.0168 0.000669 0.0172 0.00109 0.0302 0.0058 0.0441 0.0144 0.0264
2L:16448160-16448310:plus -22.9184 2.53211 -3.17431 3.78899 -17.1122 -8.45918 2.58716 3.11111 6.49541 0.00447 0.00413 0.0303 0.00632 0.00341 0.00111 0.000136 0.00572 0.00154
2L:16450980-16451130:minus -32.3673 -2.48624 1.57798 6.11009 0.836735 -19.0816 -12.4037 -2.29167 1.38532 0.0189 0.0223 0.00674 0.00233 0.000215 0.017 0.00505 0.0322 0.0123
2L:16450980-16451130:plus -22.2245 2.20183 -1.56881 9.59633 -26.898 -8.45918 -10.3945 2.54167 2.84404 0.00325 0.00466 0.019 0.000275 0.0203 0.00111 0.00332 0.00709 0.00736
2L:16491540-16491690:minus -33.0408 3.82569 10.6055 6.57798 -17.3776 -26.6735 -19.3394 0.75 2.25688 0.0264 0.00257 0.000099 0.00167 0.00419 0.0256 0.0184 0.0132 0.00853
2L:16491540-16491690:plus -30.6633 13.7798 -1.31193 1.34862 -25.9286 1.02041 -10.5505 0.722222 -6.44037 0.00976 0.0000109 0.0176 0.0187 0.0168 0.000118 0.00342 0.0133 0.099
2L:16492000-16492150:minus -12 10.0917 0.587156 3.3945 -18.3776 -9.23469 -25.6881 -4.05556 -4.26606 0.000305 0.000128 0.00952 0.0076 0.00926 0.00238 0.0515 0.0499 0.0595
2L:16492000-16492150:plus -31.8061 11.2477 0.577982 3.26606 -18.3776 -18.1939 -18.5321 7.16667 3.66972 0.0158 0.0000718 0.00955 0.00802 0.00926 0.0101 0.0162 0.000922 0.00514
2L:16511400-16511550:minus -23.0714 5.84404 4.43119 7.45872 -27 -18.8571 -19.1284 -0.361111 3.25688 0.0052 0.00113 0.00225 0.000894 0.0233 0.0151 0.0178 0.0187 0.00632
2L:16511400-16511550:plus -39.5204 3.90816 1.01835 2.49541 -18.4898 -19.0102 -10.5138 8.36111 5.33028 0.0334 0.00102 0.00821 0.0114 0.0116 0.0159 0.00339 0.000474 0.00273
2L:1651200-1651350:minus -22.5102 -1.3578 1.55963 5.15596 -9.20408 -19.5714 -13.3578 2.33333 11.8532 0.00354 0.016 0.00678 0.00426 0.00262 0.0202 0.00628 0.00765 0.0000428
2L:1651200-1651350:plus -24.1837 5.62385 1.24771 5.85321 -18.4898 -27.3776 -6.84404 -2.31944 3.46789 0.00751 0.00125 0.00758 0.00287 0.0116 0.0351 0.00163 0.0325 0.00582
2L:165160-165310:minus -21.7755 0.53211 5.97248 9.7156 -18.9388 -18.0102 -17.8624 2.15278 7.63303 0.00241 0.00856 0.00117 0.000242 0.0151 0.00812 0.0146 0.00816 0.000928
2L:165160-165310:plus -32.2449 0.284404 4.74312 3.9633 -26.9286 -9.82653 -11.5505 2.26389 1.94495 0.0183 0.00935 0.00198 0.0059 0.0214 0.00381 0.00418 0.00784 0.00968

2L:16520240-16520390:minus -22.551 -0.669725 -2.0367 2.11009 -18.5204 -9.45918 -8.7156 0.0833333
-1.88073 0.00359 0.0129 0.0219 0.0136 0.0117 0.00279 0.00242 0.0163 0.0321
2L:16520240-16520390:plus -13.449 8.79817 -3.83486 2.98165 -18.4184 -8.23469 -13.7615 4.25 5.9633
0.000914 0.000249 0.0363 0.00905 0.0101 0.000875 0.00688 0.00362 0.00188
2L:16520720-16520870:minus -40.4796 -1.18349 -1.47706 6.6422 -17.7143 -16.2041 -22.5413 3.625
0.614679 0.0407 0.0152 0.0185 0.00161 0.00627 0.00408 0.0324 0.00468 0.0158
2L:16520720-16520870:plus -30.6633 -3.88991 -5.3211 3.79817 -17.4184 -18.051 -13.8991 2.84722 11.8991
0.00976 0.0328 0.0535 0.00629 0.00457 0.00886 0.00709 0.00632 0.0000255
2L:16521440-16521590:minus -31.9286 7.33028 6.23853 2.77064 -26.2347 -9.72449 -17.7523 0.722222
4.77982 0.0166 0.000546 0.00104 0.01 0.0177 0.00352 0.0144 0.0133 0.00339
2L:16521440-16521590:plus -39.1837 -0.348624 3.81651 2.69725 -17.7449 -26.2653 -16.367 6.33333
-7.36697 0.0328 0.0116 0.00289 0.0104 0.0063 0.0218 0.0116 0.00141 0.121
2L:16526260-16526410:minus -24.2347 -1.91743 0.0275229 2.95413 -7.60204 -8.72449 -18.7982 1.83333
-2.94495 0.00763 0.019 0.0115 0.00917 0.000466 0.00169 0.0169 0.00914 0.0441
2L:16526260-16526410:plus -22.2245 -0.137615 3.15596 2.59633 -7.53061 -17.7143 -11.6147 -4.45833
-2.3211 0.00325 0.0108 0.00374 0.0109 0.000407 0.00634 0.00424 0.0547 0.0365
2L:16526960-16527110:minus -40.8878 -2.7156 2.7156 -0.678899 -17.4898 -7.5 -15.8165 7.41667
-1.22936 0.0439 0.0238 0.00442 0.0332 0.0053 0.000614 0.0105 0.000807 0.0279
2L:16526960-16527110:plus -41.6327 1.33945 3.89908 1.72477 -6.67347 -18.3469 -27.6239 3.05556 -2.22018
0.0631 0.0064 0.00279 0.0161 0.000359 0.0124 0.0655 0.00584 0.0355
2L:16528600-16528750:minus -21.551 4.66055 -2.12844 -1.24771 -27.2653 -8.79592 -16.7615 7.72222
-0.761468 0.00205 0.00187 0.0224 0.0398 0.0302 0.0019 0.0123 0.000682 0.0244
2L:16528600-16528750:plus -23.0306 6.33028 -0.761468 5.69725 -9.20408 0.244898 -11.1101 -3.875
-3.20183 0.00505 0.000904 0.0148 0.00314 0.00262 0.000285 0.00382 0.0478 0.0467
2L:16529760-16529910:minus -23.1837 7.49541 2.83486 4.19266 -8.86735 -9.38776 -9.90826 10.25
2.25688 0.00553 0.0005 0.00423 0.00536 0.00173 0.00266 0.00303 0.000136 0.00853
2L:16529760-16529910:plus -32.4796 0.40367 3.81651 1.77982 -27.3061 -9.60204 -14.2294 7.16667 1.25688
0.02 0.00896 0.00289 0.0158 0.0315 0.00334 0.00763 0.000922 0.0131
2L:16530200-16530350:minus -3.11224 7.57798 6.20183 7.21101 -18.3469 -8.45918 -14.4587 -2.66667
4.52294 0.0000458 0.000479 0.00106 0.0011 0.00894 0.00111 0.00802 0.0355 0.00358
2L:16530200-16530350:plus -21.6633 -0.46789 2.04587 3.76147 -18.4898 -9.79592 -10.5688 6.94444 5.12844
0.00223 0.0121 0.00568 0.00645 0.0116 0.00377 0.00343 0.00103 0.00301
2L:16530800-16530950:minus -31.7449 -0.844037 0.357798 9.08257 -28.2959 -8.86735 -27.3028 5.02778
-2.43119 0.0152 0.0136 0.0103 0.000444 0.0541 0.00198 0.0628 0.00259 0.038
2L:16530800-16530950:plus -20.3265 0.284404 10.7339 1.94495 -17.6735 -26.898 -6.59633 -2.45833 1.29358
0.00165 0.00935 0.0000931 0.0147 0.00572 0.0302 0.00153 0.0337 0.0128
2L:16531260-16531410:minus -32.2551 -3.43119 -0.587156 -1.72477 -19.0102 -27.6327 -21.6881 4.41667
1.48624 0.0184 0.029 0.014 0.0465 0.0157 0.043 0.0279 0.00338 0.0118
2L:16531260-16531410:plus -33.0102 -0.550459 -1.54128 3.40367 -17.7143 -8.42857 -34.2018 0.263889
-3.29358 0.0259 0.0124 0.0188 0.00759 0.00627 0.00104 0.148 0.0154 0.0474
2L:16532940-16533090:minus 6.43878 8.43119 1.3945 3.97248 -8.64286 -18.0816 -16.8073 2.77778
2.09174 3.32e-06 0.000302 0.00719 0.00587 0.00139 0.00934 0.0124 0.00649 0.00904
2L:16532940-16533090:plus -23.0714 -4.20183 1.47706 1.68807 -18.2245 -18.3469 -16.0275 0.333333 12.2752
0.0052 0.0357 0.00698 0.0164 0.00877 0.0124 0.0109 0.0151 0.0000141
2L:1653300-1653450:minus -32.0408 -1.07339 -1.16514 2.97248 -28.1939 -8.79592 -13.9541 1.08333 -2.22018
0.0174 0.0147 0.0168 0.00909 0.0482 0.0019 0.00718 0.0118 0.0355
2L:1653300-1653450:plus -32.8061 3.75229 1.77982 -1.62385 -28.4898 -8.86735 -25.8716 6.59722 -0.862385
0.0237 0.00264 0.00627 0.0451 0.0581 0.00198 0.0527 0.00124 0.0251
2L:16534300-16534450:minus -41.2959 -0.0825688 -1.75229 -0.963303 -18.5204 -18.2755 -10.633
3.59722 7.68807 0.0529 0.0106 0.0201 0.0362 0.0117 0.0112 0.00347 0.00473 0.000841
2L:16534300-16534450:plus -34.5612 -1.50459 -3.76147 6.57798 -9.16327 -9.5 -0.201835 6 -0.926606
0.032 0.0168 0.0356 0.00167 0.00249 0.00294 0.000297 0.00166 0.0256
2L:16544800-16544950:minus -21.6633 1.88991 -0.697248 3.59633 -18.6837 -9.7551 -14.3761 -2.72222
-7.77982 0.00223 0.00523 0.0145 0.00693 0.0126 0.00358 0.00788 0.036 0.13

2L:16544800-16544950:plus -11.6224 3.61224 -2.59633 2.66972 -18.1531 -17.3469 -1.33028 11.0833 10.0367
0.000259 0.00167 0.0257 0.0106 0.00829 0.0058 0.000382 0.0000712 0.000146
2L:165500-165650:minus -41.8163 0.807339 8.45872 3.45872 -18.9388 -18.3776 -11.9541 5.65278 -0.697248
0.0662 0.00776 0.000341 0.00731 0.0151 0.0125 0.00456 0.00195 0.0239
2L:165500-165650:plus -23.2551 -5.51376 1.52294 1.06422 -18.4898 -27.7857 -17.2385 -2.66667 2.20183
0.00583 0.0504 0.00687 0.0203 0.0116 0.0466 0.0133 0.0355 0.00869
2L:1656700-1656850:minus -22.1531 2.16514 3.99083 5.90826 -8.60204 -8.45918 -32.0826 0.638889 -4.92661
0.0031 0.00473 0.00269 0.00271 0.00125 0.00111 0.119 0.0137 0.0717
2L:1656700-1656850:plus -23.2551 2.46789 -1.94495 1.33028 -7.63265 -36.449 -13.8807 8.54167 7.15596
0.00583 0.00422 0.0213 0.0188 0.000479 0.109 0.00706 0.000425 0.00124
2L:1657100-1657250:minus -14.7857 -1.22936 -0.449541 7 -18.3776 -9.72449 -2.14679 5.56944 3.29358
0.00158 0.0154 0.0134 0.00132 0.00926 0.00352 0.00046 0.00203 0.00623
2L:1657100-1657250:plus -21.1837 -1.06422 -0.605505 5.59633 -26.4898 -26.4898 -11.5596 6.25 0.0825688
0.00189 0.0146 0.0141 0.00331 0.0183 0.0225 0.00419 0.00147 0.0181
2L:16588480-16588630:minus -23.1531 1.69725 0.12844 3.51376 -28.5306 -16.9388 -17.5872 1.52778
-0.440367 0.00542 0.00561 0.0111 0.00709 0.061 0.00433 0.014 0.0102 0.0216
2L:16588480-16588630:plus -23.1837 6.79817 5.00917 6.13761 -7.89796 -28.7041 -1.88991 -1 -2.50459
0.00553 0.00072 0.00177 0.00226 0.000678 0.067 0.000433 0.0226 0.0388
2L:16601660-16601810:minus -33.6735 -1.89908 4.44037 6.56881 -27.0102 -18.051 -15.2844 -2.84722
4.77064 0.0291 0.0189 0.00224 0.0017 0.0237 0.00886 0.00949 0.0372 0.0034
2L:16601660-16601810:plus -12.7449 0.798165 5.06422 7.34862 -8.89796 -17.7143 -1.77982 5.625 1.16514
0.000471 0.00779 0.00173 0.000998 0.00189 0.00634 0.000422 0.00198 0.0135
2L:16602460-16602610:minus -23.6327 0.880734 0.972477 -1.05505 -27.8265 10.3061 -20.2294 5.97222
5.18349 0.00661 0.00756 0.00835 0.0374 0.0388 7.25e-06 0.0214 0.00168 0.00295
2L:16602460-16602610:plus -22.9286 1.14679 -0.605505 7.27523 -27.1939 -19.2347 -15.5963 1.27778
3.40367 0.0046 0.00686 0.0141 0.00109 0.0267 0.018 0.0101 0.0111 0.00603
2L:16612220-16612370:minus -31.1122 -2.17431 0.853211 1.92661 -27.4592 0.0204082 -14.1927 5.02778
7.22018 0.0107 0.0204 0.0087 0.0148 0.0329 0.000377 0.00757 0.00259 0.00119
2L:16612220-16612370:plus -41.4796 1.95413 -3.97248 2.55046 -27.7041 -8.79592 -13.3761 4.02778 3.88073
0.0577 0.0051 0.0377 0.0112 0.038 0.0019 0.0063 0.00397 0.00475
2L:16613500-16613650:minus -23.3776 -0.53211 2.98165 6.74312 -7.40816 -0.244898 -12.6789 -2.29167
4.61468 0.00619 0.0123 0.004 0.0015 0.000401 0.000496 0.00537 0.0322 0.00351
2L:16613500-16613650:plus -33.0714 -2.31193 5.56881 6.72477 -27.9694 -27.3367 -21.5046 5.29167 1.49541
0.0267 0.0213 0.0014 0.00154 0.0437 0.0347 0.027 0.0023 0.0118
2L:16637460-16637610:minus -21.8878 3.08257 5.31193 3.12844 -19.051 -18.3469 -13.2844 2.97222
-1.79817 0.00258 0.00338 0.00156 0.00857 0.0162 0.0124 0.00617 0.00603 0.0315
2L:16637460-16637610:plus -24.2245 -1.7156 2.75229 3.80734 -18.7551 -18.8571 -10.6514 -0.0694444
5.90826 0.00763 0.0179 0.00436 0.00627 0.0144 0.0151 0.00348 0.0171 0.00198
2L:16639500-16639650:minus -22.7041 -1.91743 5.44037 5.85321 -17.9796 -8.30612 -18.9908 -2.13889
-3.88991 0.00395 0.019 0.00148 0.00287 0.00726 0.000973 0.0174 0.0309 0.054
2L:16639500-16639650:plus -21.6633 4.55046 2.3945 6.33028 -17.7551 -18.0102 -11.4771 -0.180556
1.2844 0.00223 0.00195 0.00499 0.00198 0.00658 0.00812 0.00412 0.0177 0.0129
2L:16641240-16641390:minus -3.92857 -1.0367 -1.12844 2.34862 -18.051 -7.94898 -17.4404 -0.958333
0.53211 0.000116 0.0145 0.0166 0.0121 0.00746 0.00074 0.0137 0.0223 0.0161
2L:16641240-16641390:plus -23.0714 -0.458716 -0.330275 5.16514 -27.0408 -28.7041 -7.19266 7.44444
0.348624 0.0052 0.012 0.0129 0.00424 0.0242 0.067 0.00176 0.000795 0.0167
2L:1665440-1665590:minus -13.3673 -3.29358 -1.90826 5.49541 -18.7449 -8.7551 -7.92661 10.5833 7.51376
0.000813 0.028 0.021 0.00355 0.0138 0.00172 0.00207 0.000106 0.000984
2L:1665440-1665590:plus -24.1122 -3.22936 -1.12844 3.86239 -17.1837 -16.3469 -22.6972 -1.79167 1.79817
0.00738 0.0275 0.0166 0.00622 0.00366 0.00416 0.0333 0.0282 0.0102
2L:16656800-16656950:minus -23.3776 -3.91743 5.29358 1.92661 -17.4898 -0.0204082 -25.9817 3.58333
-3.88991 0.00619 0.0331 0.00157 0.0148 0.0053 0.000411 0.0534 0.00475 0.054
2L:16656800-16656950:plus -21.8163 -2.82569 1.63303 3.3578 -8.33673 9.82653 -8.16514 3.83333 0.697248
0.00244 0.0246 0.00661 0.00767 0.00101 0.0000148 0.00218 0.0043 0.0154

2L:166580-166730:minus -4.89796 5.42202 -1.49541 -0.889908 -18.4898 -16.051 -14.4495 0.666667
-1.44954 0.000208 0.00137 0.0186 0.0355 0.0116 0.00408 0.008 0.0135 0.0291
2L:166580-166730:plus -13.2551 0.59633 8.37615 3.42202 -17.4082 -8.87755 -7.56881 2.125 1.11927
0.000751 0.00837 0.000358 0.00751 0.00421 0.00198 0.00192 0.00824 0.0137
2L:1667000-1667150:minus -32.8469 0.53211 1.55963 3.19266 -17.4184 -17.8571 -11.633 3.19444 5.07339
0.0243 0.00856 0.00678 0.00829 0.00457 0.00719 0.00426 0.00554 0.00305
2L:1667000-1667150:plus -23.5612 -0.495413 4.24771 2.95413 -27.1939 1.05102 -19.6055 2.23611
3.98165 0.00653 0.0122 0.00243 0.00917 0.0267 0.000107 0.0193 0.00792 0.00439
2L:1667580-1667730:minus -30.7449 -0.449541 1.24771 3.3578 -17.6837 0.755102 -16.7431 5.19444
10.0367 0.0101 0.012 0.00758 0.00767 0.0058 0.000175 0.0123 0.00241 0.000146
2L:1667580-1667730:plus -22.4082 -0.87156 -3.11927 9.44037 -8.93878 -17.0102 -28.2752 1.23611 3.97248
0.00345 0.0138 0.0298 0.000339 0.00216 0.00453 0.0714 0.0112 0.00444
2L:16677100-16677250:minus -22.8061 -1.88991 2.94495 1.33028 -27.7041 -8.16327 -12.5413 5.19444
5.75229 0.00414 0.0188 0.00405 0.0188 0.038 0.000783 0.00521 0.00241 0.00213
2L:16677100-16677250:plus -32.9286 13.3878 0.541284 0.522936 -27.1939 -18.2755 -13.9083 0.722222 -4.55963
0.025 0.0000869 0.00968 0.0234 0.0267 0.0112 0.00711 0.0133 0.0648
2L:16679940-16680090:minus -32.7755 9.77982 2.44954 7.61468 -18.9796 0.0204082 -24.4128 2.77778
-4.6789 0.0234 0.000149 0.00489 0.000779 0.0155 0.000377 0.0436 0.00649 0.067
2L:16679940-16680090:plus -15.0816 13.3878 -0.12844 5.73394 -17.4184 0.683673 -9.09174 2.84722 -0.899083
0.00163 0.0000869 0.0121 0.00307 0.00457 0.000184 0.0026 0.00632 0.0254
2L:16680420-16680570:minus -31.7755 -4.30275 -0.229358 0.788991 -8.90816 -8.57143 -18.3211 4.44444
-2.26606 0.0157 0.0367 0.0125 0.0218 0.00192 0.00149 0.0157 0.00334 0.0359
2L:16680420-16680570:plus -34.3367 -1.49541 4.14679 10.0275 10.7245 -9.68367 -20.1376 2.80556 -1.2844
0.0318 0.0167 0.00252 0.000156 5.2e-06 0.00342 0.0211 0.00642 0.0283
2L:1668700-1668850:minus -31.9286 1.00917 11.3303 6.78899 -17.0816 1.38776 -16.9266 2.98611 5.23853
0.0166 0.00722 0.0000653 0.00146 0.00334 0.0000559 0.0127 0.006 0.00285
2L:1668700-1668850:plus -40.4898 0.275229 -4.65138 1.46789 -18.0816 -7.72449 -20.6972 -0.486111
9.92661 0.0407 0.00938 0.0451 0.0179 0.00759 0.00068 0.0233 0.0194 0.000193
2L:1669180-1669330:minus -31.9694 -0.0275229 1.59633 5.57798 -27.1633 -9.68367 -16.0826 1.58333
1.74312 0.0168 0.0104 0.00669 0.0034 0.025 0.00342 0.011 0.00997 0.0105
2L:1669180-1669330:plus -40.9898 -1.21101 3.82569 4.81651 -17.1837 -27.6735 -17.8532 8.59722 4.98165
0.0455 0.0153 0.00288 0.00451 0.00366 0.0443 0.0146 0.000411 0.00313
2L:16703200-16703350:minus -32.8163 -3.27523 5.6422 -0.201835 -27.2245 -18.3469 -12.4771 6.68056
2.16514 0.024 0.0278 0.00135 0.0285 0.0272 0.0124 0.00513 0.00119 0.00879
2L:16703200-16703350:plus -32.0714 4.66972 -4.66055 7.10092 -8.82653 -18.0816 -23.1376 0 -2.83486
0.0176 0.00186 0.0452 0.00121 0.00163 0.00934 0.0359 0.0167 0.0427
2L:16719840-16719990:minus -14.1122 -0.513761 0.724771 -0.40367 -18.1837 -7.79592 -15.7982 6.06944
1.46789 0.00119 0.0123 0.00909 0.0302 0.00835 0.000723 0.0105 0.0016 0.0119
2L:16719840-16719990:plus -13.1837 10.7982 -3.29358 7 19.9082 -19.0816 -14.0826 4.06944 -0.825688
0.000705 0.0000889 0.0313 0.00132 8.13e-07 0.017 0.00739 0.00391 0.0249
2L:16720180-16720330:minus -32.6735 0.256881 0.633028 -1.91743 -16.6735 0.0510204 -24.156 8.58333
-5.31193 0.0216 0.00944 0.00938 0.0499 0.00313 0.000349 0.042 0.000415 0.0776
2L:16720180-16720330:plus -30.1837 -1.10092 3.2844 6.29358 -27.9388 -8.42857 -7.17431 -0.430556
5.22936 0.00873 0.0148 0.00356 0.00202 0.0415 0.00104 0.00176 0.0191 0.00288
2L:16721180-16721330:minus -31.5918 -0.577982 1.92661 7.15596 -8.12245 -17.5714 -9.7156 3.5
0.440367 0.0136 0.0125 0.00594 0.00117 0.000843 0.00616 0.00292 0.00491 0.0164
2L:16721180-16721330:plus -23.3367 -3.61468 0.440367 0.192661 -18.6837 -16.7857 -13.8349 2.76389 7.42202
0.00614 0.0305 0.01 0.0256 0.0126 0.00426 0.00699 0.00652 0.00106
2L:16727760-16727910:minus -32.4082 7.15596 9.22936 7.2844 -17.7143 -18.3469 -17.7431 4.18056
-2.52294 0.0191 0.000599 0.000221 0.00105 0.00627 0.0124 0.0144 0.00373 0.0391
2L:16727760-16727910:plus -22 8.88073 0.715596 5.37615 -27.6735 -0.0204082 -18.4771 0.902778 1.74312
0.00291 0.000238 0.00912 0.00384 0.0375 0.000411 0.0161 0.0125 0.0105
2L:16729620-16729770:minus -33.2959 -2.44954 1.78899 5.51376 19.9082 -0.0918367 -21.9174 5.51389
-1.41284 0.0279 0.0221 0.00625 0.00348 8.13e-07 0.000442 0.0291 0.00208 0.0289

2L:16729620-16729770:plus -22.2245 -1.55046 1.49541 6.21101 -18.4184 19.1531 -14.4587 3.05556 7.16514
0.00325 0.017 0.00694 0.00213 0.0101 7.95e-07 0.00802 0.00584 0.00122
2L:16732080-16732230:minus -13.0714 2.69725 -0.385321 3.78899 1.43878 1.31633 -19.2936 4.08333
3.47706 0.000594 0.00389 0.0131 0.00632 0.0000769 0.000066 0.0183 0.00388 0.00575
2L:16732080-16732230:plus -40.2959 3.22936 0.743119 -1.11927 -26.2653 -8.94898 -24.2844 5.66667 0.229358
0.0389 0.0032 0.00903 0.0381 0.018 0.00203 0.0428 0.00194 0.0173
2L:16738400-16738550:minus -22.449 5.36697 8.85321 3.47706 20.4694 0.244898 -16.945 0.0277778
5.23853 0.0035 0.0014 0.000275 0.00724 1.65e-07 0.000285 0.0127 0.0166 0.00285
2L:16738400-16738550:plus -41.7755 -0.779817 -0.449541 2.09174 -18.0102 -7.79592 -20.5138 5.31944
-7.02752 0.0652 0.0134 0.0134 0.0137 0.00732 0.000723 0.0225 0.00227 0.114
2L:16743340-16743490:minus -22.8061 13.5306 3.12844 1.99083 -27.8265 -8.94898 -11.9266 5.19444
6.95413 0.00414 0.0000206 0.00378 0.0144 0.0388 0.00203 0.00454 0.00241 0.00136
2L:16743340-16743490:plus -24.2653 14.6881 2.82569 5.06422 10.7245 -17.5 -25.9174 -1.02778 4.55963
0.00773 3.75e-06 0.00424 0.00433 5.2e-06 0.00599 0.0529 0.0227 0.00357
2L:16790900-16791050:minus -22 1.68807 0.733945 9.08257 -26.898 -8.30612 0.678899 5.81944 -0.247706
0.00291 0.00563 0.00906 0.000444 0.0203 0.000973 0.00024 0.00181 0.0204
2L:16790900-16791050:plus -21.9286 0.862385 0.522936 6.02752 -18.1224 -27.3367 -10.2018 -0.527778
3.41284 0.0027 0.00761 0.00974 0.00246 0.00782 0.0347 0.0032 0.0196 0.006
2L:16791440-16791590:minus -22.9592 -0.798165 0.568807 1.6055 -16.3776 -26.6327 -16.8716 3.48611
6.17431 0.00475 0.0134 0.00958 0.0169 0.003 0.0251 0.0126 0.00494 0.00171
2L:16791440-16791590:plus -13.7449 -2.45872 4.38532 10.8624 -8.64286 -18.051 -6.79817 5.43056 3.20183
0.00107 0.0222 0.0023 0.0000687 0.00139 0.00886 0.00161 0.00216 0.0065
2L:16816620-16816770:minus -30.7041 10.6514 -1.24771 4.15596 -18.4184 -17.2755 -22.2569 -0.375
-2.3211 0.00993 0.0000959 0.0172 0.00539 0.0101 0.00544 0.0309 0.0188 0.0365
2L:16816620-16816770:plus 6.10204 1.08257 6.88991 10.5872 -18.8265 -7.72449 -10.1101 6.20833 5.70642
6.19e-06 0.00703 0.000765 0.000115 0.0149 0.00068 0.00314 0.0015 0.00222
2L:16825100-16825250:minus -31.4796 13.3878 -2.52294 5.69725 -26.8571 -19.3061 -18.6422 -3.02778
1.78899 0.0128 0.0000869 0.0252 0.00314 0.0198 0.0187 0.0165 0.0389 0.0103
2L:16825100-16825250:plus -41.7041 4.23853 6.29358 5.86239 -27.4592 -8.38776 -13.9083 1.70833 -2.49541
0.0641 0.0022 0.00101 0.00282 0.0329 0.00101 0.00711 0.00955 0.0387
2L:16827200-16827350:minus -22 0.275229 -1.55963 1.80734 -8.71429 -25.8265 -16.055 3.59722 3.6789
0.00291 0.00938 0.0189 0.0156 0.00159 0.0212 0.011 0.00473 0.00506
2L:16827200-16827350:plus -31.1735 -0.00917431 -0.293578 3.02752 -18.4592 -7.57143 -20.6055 10.3056
-4.90826 0.0109 0.0103 0.0128 0.00887 0.0109 0.000644 0.0229 0.000131 0.0714
2L:168320-168470:minus -23.2245 0.550459 2.3211 1.43119 -18.7143 -17.051 -13.0917 3.16667 -0.697248
0.00576 0.00851 0.00513 0.0181 0.0136 0.00479 0.0059 0.0056 0.0239
2L:168320-168470:plus -30.6327 1.87156 2.75229 3.92661 -9.16327 -8.94898 -0.559633 -3.09722 -2.3945
0.00968 0.00526 0.00436 0.00601 0.00249 0.00203 0.000322 0.0396 0.0375
2L:16879340-16879490:minus -40.0612 -3.58716 0.0733945 2.41284 -27.4592 -0.173469 -12.5596 1.75
-2.43119 0.0358 0.0303 0.0113 0.0118 0.0329 0.000456 0.00523 0.00941 0.038
2L:16879340-16879490:plus -41.2245 0.376147 14.8991 2.33945 -18.7857 -18.3061 -21.6514 7.58333 1.02752
0.0497 0.00905 1.97e-06 0.0122 0.0148 0.0114 0.0277 0.000737 0.0141
2L:16881060-16881210:minus -12.5204 -0.40367 9.55963 7.55963 -18.3878 -28.3776 -6.81651 -2.15278
4.31193 0.000441 0.0118 0.000184 0.000807 0.00931 0.0609 0.00161 0.0311 0.00386
2L:16881060-16881210:plus -21.551 -3.10092 5.29358 6.73394 -17.1531 -19.1224 -10.4587 -6.95833 -0.550459
0.00205 0.0265 0.00157 0.00152 0.00364 0.0175 0.00336 0.0918 0.0225
2L:16886520-16886670:minus -22.0714 -2.44954 2.68807 8.80734 -9.20408 -9.23469 -11.4954 -0.458333
6.95413 0.00299 0.0221 0.00447 0.000518 0.00262 0.00238 0.00413 0.0192 0.00136
2L:16886520-16886670:plus -23.0306 -0.605505 0.293578 6.14679 -8.90816 -7.79592 -10.3211 6.47222
1.38532 0.00505 0.0126 0.0105 0.00224 0.00192 0.000723 0.00327 0.00132 0.0123
2L:16887940-16888090:minus -12.9184 8.05505 6.61468 1.76147 -8.56122 -8.5 -6.66055 -2.26389 -0.440367
0.000532 0.000371 0.000869 0.0159 0.00112 0.00126 0.00155 0.032 0.0216
2L:16887940-16888090:plus -32.4796 2.78899 -1.55963 7.17431 -18.449 -8.5 -23.5963 -0.375 5.44954 0.02
0.00376 0.0189 0.00115 0.0107 0.00126 0.0386 0.0188 0.00253

2L:16888360-16888510:minus -33.2959 3.97959 -3.87156 3.33028 -27.602 -8.7551 -20.4128 0.958333
1.95413 0.0279 0.000669 0.0367 0.00778 0.0364 0.00172 0.0221 0.0123 0.00964
2L:16888360-16888510:plus -21.9592 4.26606 15.5596 8.80734 -27.1939 -18.051 -20.1009 -2.875 2.75229
0.00279 0.00217 6.69e-07 0.000518 0.0267 0.00886 0.021 0.0375 0.00748
2L:16888860-16889010:minus -32.6633 4.86239 -4.77064 2.25688 -8.93878 -10.0204 -24.5321 4.38889
0.697248 0.0216 0.00172 0.0465 0.0127 0.00216 0.00399 0.0443 0.00342 0.0154
2L:16888860-16889010:plus -31.2551 4.59633 -1.74312 -1.47706 -18.1837 -28.3776 -16.6422 -2.36111 3.7156
0.0113 0.00191 0.02 0.0429 0.00835 0.0609 0.0121 0.0328 0.00502
2L:16889160-16889310:minus -24.4082 0.247706 2.33028 7.63303 -18.3469 -9.0102 -22.6881 6.93056
1.72477 0.00797 0.00947 0.00511 0.000745 0.00894 0.00208 0.0333 0.00104 0.0106
2L:16889160-16889310:plus -22.9592 2.70642 2.13761 5.66055 -8.60204 -18.9796 -14.9908 -0.5 1.34862
0.00475 0.00387 0.00549 0.00317 0.00125 0.0155 0.00896 0.0195 0.0125
2L:16902180-16902330:minus -33.3776 7.3211 0.642202 -1.6055 2.14286 1.05102 -26.9083 2.18056
6.11927 0.0282 0.000548 0.00935 0.0448 0.0000316 0.000107 0.0598 0.00808 0.00178
2L:16902180-16902330:plus -32.0714 0.853211 -0.908257 -0.137615 -9.23469 -17.9388 -22.2385 4.55556
-2.13761 0.0176 0.00763 0.0155 0.0279 0.00277 0.00738 0.0308 0.00318 0.0345
2L:1692720-1692870:minus -30.6633 -0.0183486 -0.0275229 5.06422 -27.2347 9.53061 -8.01835 -0.569444
-8.9633 0.00976 0.0104 0.0117 0.00433 0.0283 0.0000316 0.00211 0.0199 0.165
2L:1692720-1692870:plus -14.5612 1.43119 2.70642 1.69725 -18.5612 -9.57143 -23.2844 4.72222 2.88991
0.00147 0.00619 0.00444 0.0163 0.0119 0.00326 0.0368 0.00296 0.00725
2L:1704820-1704970:minus -23.0714 0.954128 -1.89908 6.45872 -18.1837 -9.60204 -15.945 7 -0.348624
0.0052 0.00736 0.021 0.00182 0.00835 0.00334 0.0107 0.00101 0.0209
2L:1704820-1704970:plus -23.102 10.0459 -2.21101 4.05505 -18.4796 -26.2653 -14.0734 5.40278 7.83486
0.00522 0.00013 0.023 0.00566 0.011 0.0218 0.00737 0.00219 0.000712
2L:1705180-1705330:minus -31.9694 8.16514 2.44037 2.05505 -17.7143 0.0918367 -32.8899 -1.27778
-10.8807 0.0168 0.000349 0.00491 0.0139 0.00627 0.000323 0.13 0.0244 0.228
2L:1705180-1705330:plus -31.1327 -2.36697 7.11009 11.1927 -17.3469 -26.5306 -11.945 -5.15278 -1.01835
0.0107 0.0216 0.000691 0.0000254 0.00394 0.023 0.00456 0.0638 0.0263
2L:1708300-1708450:minus -30.449 1.18349 3.95413 2.23853 -28.1633 -18.051 -20.2569 4.83333 4.19266
0.00925 0.00677 0.00273 0.0128 0.0462 0.00886 0.0215 0.00283 0.00401
2L:1708300-1708450:plus -21.7041 -2.31193 2.01835 11.1927 -27.1939 -18.051 -7.20183 -0.847222
7.83486 0.00231 0.0213 0.00574 0.0000254 0.0267 0.00886 0.00177 0.0216 0.000712
2L:1710340-1710490:minus -22.7041 7.01835 1.55046 4.6422 20.2041 -26.3367 -12.4404 2.65278 1.55046
0.00395 0.000643 0.0068 0.00473 3.64e-07 0.0221 0.00509 0.0068 0.0115
2L:1710340-1710490:plus -22.9592 -1.56881 12.6789 7.29358 -8.71429 -27.602 -19.2569 3.98611 1.44037
0.00475 0.0171 0.0000266 0.00104 0.00159 0.0416 0.0182 0.00404 0.012
2L:1715800-1715950:minus -24.2245 -0.431193 1.19266 -0.834862 -26.8878 -0.469388 -11.2752
-1.54167 -2.44037 0.00763 0.0119 0.00773 0.0349 0.0199 0.000533 0.00395 0.0263 0.0381
2L:1715800-1715950:plus -32.7755 -1.37615 -1.61468 2.93578 -17.7143 -10.0204 -17.7339 2.52778 -4.84404
0.0234 0.0161 0.0193 0.00927 0.00627 0.00399 0.0144 0.00712 0.0701
2L:1719740-1719890:minus -23.8163 7.22018 1.27523 1.22936 -9.23469 -0.27551 -24.4128 5.56944 2.74312
0.00682 0.000579 0.0075 0.0193 0.00277 0.000502 0.0436 0.00203 0.00751
2L:1719740-1719890:plus -31.8163 6.63303 4.48624 4.61468 -27.3061 -18.8571 -17.5505 -1.23611 5.74312
0.016 0.000782 0.0022 0.00475 0.0315 0.0151 0.014 0.0241 0.00215
2L:1720100-1720250:minus -23.1837 3.84404 1.49541 7.27523 -18.0816 -9.30612 -15.844 1.97222 3.66055
0.00553 0.00255 0.00694 0.00109 0.00759 0.00257 0.0105 0.0087 0.00515
2L:1720100-1720250:plus -24.2959 -2.14679 1.08257 5.94495 -8.7449 -18.1224 1.44037 4.56944 2.51376
0.00781 0.0203 0.00803 0.00261 0.00161 0.00988 0.000195 0.00317 0.00792
2L:1721000-1721150:minus -22.2551 -1.05505 2.0367 2.33945 -27.1531 -7.42857 -5.09174 4.65278 -0.908257
0.00327 0.0146 0.0057 0.0122 0.0249 0.000567 0.00102 0.00305 0.0255
2L:1721000-1721150:plus -30.7755 1.08257 4.86239 6.13761 -17.8571 -27.6735 -21.844 0.680556 4.02752
0.0102 0.00703 0.00188 0.00226 0.00698 0.0443 0.0287 0.0135 0.00428
2L:1723220-1723470:minus -23.0306 4.90816 0.862385 5.46789 -8.96939 -18.6327 -4.90826 -2.20833 1.43119
0.00505 0.00023 0.00867 0.00362 0.00226 0.0137 0.000967 0.0315 0.0121

2L:1723220-1723470:plus -21.6633 -0.110092 1.54128 0.917431 -27.3061 -16.2755 -7.6422 2.88889
-0.385321 0.00223 0.0107 0.00683 0.021 0.0315 0.00413 0.00195 0.00622 0.0211
2L:1724380-1724530:minus -30.2551 2.50459 0.00917431 3.26606 2.21429 -8.0102 -12.8624 1.15278
1.12844 0.00885 0.00417 0.0116 0.00802 0.0000277 0.000754 0.0056 0.0115 0.0137
2L:1724380-1724530:plus -33.1837 1.99083 4.05505 6.00917 -19.0102 -9.68367 -22.8073 2.52778 5.34862
0.0274 0.00504 0.00262 0.00255 0.0157 0.00342 0.034 0.00712 0.00271
2L:17260780-17260930:minus -12.9184 0.449541 -4.3578 1.69725 -18.4898 9.30612 -4.82569 1.16667
7.58716 0.000532 0.00882 0.0418 0.0163 0.0116 0.0000398 0.000945 0.0115 0.000954
2L:17260780-17260930:plus -21.9286 -1.79817 -1.38532 9.92661 -18.4184 -17.2755 -14.9633 -1.79167 1.85321
0.0027 0.0183 0.018 0.000169 0.0101 0.00544 0.00891 0.0282 0.01
2L:1727660-1727810:minus -29.4082 0.944954 8.49541 5.93578 -18.1531 -26.5612 -15.7615 5.72222 -1.36697
0.0084 0.00739 0.000334 0.00264 0.00829 0.0237 0.0104 0.00189 0.0286
2L:1727660-1727810:plus -24.0306 -0.0733945 -0.0183486 3.6422 -27.0102 -18.051 -13.7339 1.95833
9.42202 0.00723 0.0106 0.0117 0.00669 0.0237 0.00886 0.00684 0.00875 0.000349
2L:1728400-1728550:minus -22.8878 -3.34862 12.9817 7.29358 -27.0102 -9.72449 -21.0367 1.66667 0.229358
0.00441 0.0284 0.0000211 0.00104 0.0237 0.00352 0.0248 0.00969 0.0173
2L:1728400-1728550:plus -32.5102 0.642202 6.59633 -0.633028 -17.7551 -17.0102 -14.422 4.66667
1.74312 0.0202 0.00823 0.000876 0.0327 0.00658 0.00453 0.00796 0.00304 0.0105
2L:1729780-1729930:minus -31.398 -4 2.70642 2.29358 -8.63265 0.0510204 -17.211 -0.569444
-0.761468 0.0117 0.0338 0.00444 0.0124 0.0013 0.000349 0.0132 0.0199 0.0244
2L:1729780-1729930:plus -12.3367 -3.41284 2.66055 5.7156 -8.60204 -18.0102 -8.25688 -2.08333 3.76147
0.000393 0.0289 0.00452 0.00309 0.00125 0.00812 0.00222 0.0305 0.00494
2L:1732320-1732470:minus -31.2551 -1.61468 12.8716 2 -17.9796 -8.65306 -15.6972 2.625 2.84404
0.0113 0.0173 0.0000231 0.0143 0.00726 0.00156 0.0103 0.00687 0.00736
2L:1732320-1732470:plus -31.0408 1.78899 8.52294 10.5872 -27.4592 -8.23469 -6.48624 5.04167 3.6789
0.0106 0.00542 0.000329 0.000115 0.0329 0.000875 0.00149 0.00258 0.00506
2L:1737420-1737570:minus -22.3776 1.66055 -1.33945 3.23853 -17.0408 -19.1531 -9.50459 4.41667 -4.23853
0.00342 0.00569 0.0177 0.00815 0.00329 0.0177 0.00281 0.00338 0.0589
2L:1737420-1737570:plus -12.6633 -1.58716 -0.678899 2.53211 -26.6633 -17.1224 1.98165 8.58333
11.8073 0.000458 0.0172 0.0144 0.0113 0.0189 0.00499 0.000166 0.000415 0.0000538
2L:17384020-17384170:minus -22.6224 7.01835 7.81651 10.8624 -26.5918 0.0918367 -2.45872 0.0972222
-2.0367 0.00371 0.000643 0.000481 0.0000687 0.0186 0.000323 0.000496 0.0162 0.0336
2L:17384020-17384170:plus -21.7755 14.8807 -0.733945 2.3578 -18.4592 -18.3469 -12.9908 8.94444
3.17431 0.00241 2.63e-06 0.0147 0.0121 0.0109 0.0124 0.00577 0.000332 0.00653
2L:17384700-17384850:minus -41.2551 0.825688 7.86239 5.43119 -17.5204 -17.9796 -12.7706 1.02778
-3.21101 0.0513 0.00771 0.000471 0.00371 0.00532 0.00764 0.00549 0.012 0.0468
2L:17384700-17384850:plus -31.2143 13.5306 1.43119 3.77064 -18.0816 -36.1122 2.04587 -0.0416667
-2.26606 0.0111 0.0000206 0.0071 0.00637 0.00759 0.0878 0.000163 0.017 0.0359
2L:17410480-17410630:minus -11.8878 1.3578 4.79817 0.908257 -17.4898 -9.79592 -28.0092
2.77778 0.733945 0.00029 0.00635 0.00193 0.0211 0.0053 0.00377 0.0689 0.00649 0.0153
2L:17410480-17410630:plus -13.3776 3.46789 7.6055 4.33028 -18.4184 -9.5 -11.0826 -5.04167
-3.13761 0.000831 0.00293 0.000539 0.00511 0.0101 0.00294 0.0038 0.0622 0.0461
2L:17428140-17428290:minus -12.4082 -3.3211 0.385321 9.49541 -27.1939 -26.5612 -5.15596
-3.02778 5.6055 0.000414 0.0282 0.0102 0.000308 0.0267 0.0237 0.00104 0.0389 0.00232
2L:17428140-17428290:plus -12.3367 6.88073 14.1835 7.45872 -17.3061 -17.7143 -11.8532
-5.44444 8.41284 0.000393 0.000691 5.88e-06 0.000894 0.00389 0.00634 0.00447 0.0678
0.000519
2L:17431000-17431150:minus -23.3367 0.633028 2.18349 3.46789 -26.9694 -9.23469 -9.65138
-4.13889 -0.440367 0.00614 0.00826 0.0054 0.00726 0.023 0.00238 0.00289 0.0508 0.0216
2L:17431000-17431150:plus -22.2959 -0.0917431 3.14679 6.18349 -8.56122 -19.0816 -21.6514
-0.347222 0.229358 0.00335 0.0106 0.00375 0.00217 0.00112 0.017 0.0277 0.0186 0.0173
2L:17449740-17449890:minus -33.0714 6.27523 1.02752 -0.40367 -18.7143 -27.8571 -10.7523
7.40278 -1.22018 0.0267 0.00093 0.00819 0.0302 0.0136 0.0508 0.00355 0.000813 0.0278
2L:17449740-17449890:plus -21.9694 6.18349 -2.80734 9.59633 -9.2449 -8.72449 -11.5872

-0.347222 -8.6055 0.00282 0.000972 0.0273 0.000275 0.00278 0.00169 0.00422 0.0186 0.154
2L:17450460-17450610:minus 14.9898 1.3211 2.30275 7.3578 -18.7551 -18.7857 -13.8257 5.08333
1.55963 5.75e-07 0.00644 0.00517 0.000965 0.0144 0.0146 0.00698 0.00253 0.0114
2L:17450460-17450610:plus -13.0408 1.01835 6.94495 4.04587 -27.2347 -18.051 -17.8991 2.65278
3.62385 0.000576 0.00719 0.000746 0.00572 0.0283 0.00886 0.0147 0.0068 0.00523
2L:17450980-17451130:minus -20.8878 -1.77064 4.65138 7.63303 -8.86735 -26.4898 -5.61468
5.23611 1.74312 0.0018 0.0182 0.00206 0.000745 0.00173 0.0225 0.00118 0.00236 0.0105
2L:17450980-17451130:plus -30.2245 7.08257 4.87156 6.29358 -25.9286 -9.45918 -13.8532
5.97222 1.70642 0.00881 0.000622 0.00188 0.00202 0.0168 0.00279 0.00702 0.00168 0.0108
2L:17472640-17472790:minus -31.6633 2.79817 2.20183 2 -17.3776 -10.0204 -16.1376
1.80556 10.3486 0.0141 0.00374 0.00536 0.0143 0.00419 0.00399 0.0111 0.00923 0.0000988
2L:17472640-17472790:plus -21.6633 0.633028 3.22936 5.49541 -17.7449 -19.051 0.779817
-1.41667 3.88991 0.00223 0.00826 0.00363 0.00355 0.0063 0.0165 0.000234 0.0254 0.00467
2L:17477800-17477950:minus -22.8878 -0.93578 1.34862 6.74312 -7.60204 -18.3061 -18.1743
-2.45833 -2.36697 0.00441 0.014 0.00731 0.0015 0.000466 0.0114 0.0154 0.0337 0.0371
2L:17477800-17477950:plus -31.551 13.9083 2.98165 3.44954 -19.051 -18.1224 -18.1927 2.27778
2.11009 0.0134 9.27e-06 0.004 0.00738 0.0162 0.00988 0.0154 0.0078 0.00898
2L:17481100-17481250:minus -24.8265 13.5306 4.3211 6.55963 -18.5306 -17.0102 -7.07339
-0.597222 -2.52294 0.0083 0.0000206 0.00235 0.00172 0.0118 0.00453 0.00172 0.0201 0.0391
2L:17481100-17481250:plus -23.449 14.4862 12.0367 3.89908 -8.86735 -17.2347 -16.8991 1.58333
1.70642 0.00631 5.19e-06 0.000042 0.00604 0.00173 0.00511 0.0126 0.00997 0.0108
2L:17484320-17484470:minus -23.2245 13.4592 1.45872 1.91743 -27.1224 -17.7857 -14.1468
3.30556 -2.95413 0.00576 0.000064 0.00703 0.015 0.0245 0.0068 0.00749 0.0053 0.0441
2L:17484320-17484470:plus -31.4286 -0.504587 11.4587 7.29358 -8.64286 -9.72449 -17.3394
1.44444 -0.449541 0.0119 0.0122 0.0000603 0.00104 0.00139 0.00352 0.0135 0.0105 0.0216
2L:17501040-17501190:minus -23.7449 0.357798 15.4495 9.59633 -17.9796 -9.53061 -18.4312
-3.69444 -2.21101 0.00674 0.00911 9.17e-07 0.000275 0.00726 0.0031 0.016 0.0458 0.0354
2L:17501040-17501190:plus -4.36735 15.6697 15.0826 1.78899 -8.93878 -9.79592 -16.5046 -5
3.6789 0.000143 6.35e-07 1.18e-06 0.0158 0.00216 0.00377 0.0118 0.0617 0.00506
2L:17589940-17590090:minus -31.449 -2.66972 0.807339 2.14679 -17.1224 -9.02041 -21.5872
4.90278 0.311927 0.0123 0.0235 0.00884 0.0133 0.00343 0.00213 0.0274 0.00274 0.0169
2L:17589940-17590090:plus -34.4898 -1.44037 -2.55963 3.13761 -26.2653 -0.173469
-18.9266 -1.44444 -3.94495 0.0319 0.0164 0.0254 0.00854 0.018 0.000456 0.0172 0.0256 0.0549
2L:17963260-17963410:minus -22.8469 13.3878 4.9633 6.09174 -27.1939 0.979592 -9.46789
2.43056 2.53211 0.00424 0.0000869 0.00181 0.00235 0.0267 0.000136 0.00279 0.00738 0.00791
2L:17963260-17963410:plus -31.4388 14.4312 1.87156 5.65138 -18.1939 -27.602 -12.0367 4.52778
5.23853 0.0122 5.39e-06 0.00606 0.00322 0.0086 0.0416 0.00465 0.00322 0.00285
2L:17963600-17963750:minus -30.3673 -4.6422 3.33028 1.61468 -18.8265 -18.2041 -28.4495
5.98611 -4.77064 0.00897 0.0401 0.00349 0.0169 0.0149 0.0103 0.0731 0.00167 0.0687
2L:17963600-17963750:plus -34.5612 4.65138 2.33945 9.76147 -18.7143 0.0510204 -14.5596
3.72222 1.33028 0.032 0.00187 0.0051 0.000212 0.0136 0.000349 0.00819 0.0045 0.0127
2L:18119740-18119890:minus -33.3367 -4.22018 1.12844 0.0917431 -26.5306 -16.9796
-10.2844 -0.263889 1.38532 0.0281 0.0359 0.00791 0.0262 0.0185 0.0044 0.00325 0.0181 0.0123
2L:18119740-18119890:plus -32.2041 -1.11009 2.82569 6.45872 -18.5204 -18.051 -7.18349
-0.819444 7.22018 0.0181 0.0148 0.00424 0.00182 0.0117 0.00886 0.00176 0.0214 0.00119
2L:18138000-18138150:minus -30.6633 -2.93578 -0.981651 6.25688 20.2041 -7.16327 -20.1009
-3.76389 -1.18349 0.00976 0.0253 0.0159 0.00208 3.64e-07 0.000547 0.021 0.0466 0.0276
2L:18138000-18138150:plus -30.7449 12.7615 0.0733945 5.58716 -7.60204 -17.051 -13.3486
0.361111 -0.605505 0.0101 0.0000265 0.0113 0.00334 0.000466 0.00479 0.00626 0.0149 0.023
2L:18141920-18142070:minus -13.1837 0.816514 2.53211 5.37615 -18.2245 -18.8571 -7.25688
-0.180556 2.84404 0.000705 0.00773 0.00475 0.00384 0.00877 0.0151 0.00179 0.0177 0.00736
2L:18141920-18142070:plus -20.5816 0.678899 5.34862 7.47706 -26.9286 -26.8265 -9.37615
2.93056 0.66055 0.00168 0.00813 0.00154 0.00085 0.0214 0.0285 0.00274 0.00612 0.0156
2L:18143340-18143490:minus -23.8571 0.174312 1.26606 9.23853 -8.93878 -18.8571 -17.2018

3.18056 2.16514 0.00687 0.00971 0.00753 0.000424 0.00216 0.0151 0.0132 0.00557 0.00879
2L:18143340-18143490:plus -14.7449 -2.80734 8.38532 3.44037 0.27551 -9.57143 -16.3303
2.90278 -0.972477 0.00157 0.0244 0.000356 0.00746 0.00035 0.00326 0.0115 0.00619 0.0259
2L:18145260-18145410:minus -40.3571 -2.61468 4.40367 4.55046 -16.3776 0.0918367 -21.1009
0.430556 1.44037 0.0394 0.0232 0.00228 0.00482 0.003 0.000323 0.025 0.0146 0.012
2L:18145260-18145410:plus -31.8878 1.6055 1.66972 1.3945 -17.4898 -18.3061 -17.578 2.18056
8.0367 0.0164 0.0058 0.00652 0.0184 0.0053 0.0114 0.014 0.00808 0.000654
2L:18147020-18147170:minus -22.8469 3.04587 1.55963 1.34862 -18.9796 -9.94898 -17.633 3.30556
-2.42202 0.00424 0.00342 0.00678 0.0187 0.0155 0.00393 0.0141 0.0053 0.0378
2L:18147020-18147170:plus -32.5102 -1.84404 -1.46789 3.44954 -26.9286 -27.7041
-5.92661 4.55556 4.80734 0.0202 0.0186 0.0184 0.00738 0.0214 0.0445 0.00129 0.00318 0.00334
2L:18151560-18151710:minus -33.1429 -2.88991 7.16514 1.91743 -16.8878 -37.2551 -21.3853
2.75 3.29358 0.0272 0.025 0.000671 0.015 0.00326 0.142 0.0264 0.00656 0.00623
2L:18151560-18151710:plus -20.7755 0.844037 -2.46789 7.33945 -18.9796 9.72449 -25.3028
7.75 -5.38532 0.00176 0.00766 0.0248 0.00102 0.0155 0.0000214 0.049 0.000672 0.0788
2L:18152400-18152550:minus -20.6939 5.33028 -1.80734 5.85321 10.6939 0.0510204 10.4404
3.97222 5.22936 0.00174 0.00142 0.0204 0.00287 7.35e-06 0.000349 0.000011 0.00407 0.00288
2L:18152400-18152550:plus -22.9184 3.22936 -2.7156 1.77982 -17.7143 -26.4898 -10.1284
2.65278 -0.275229 0.00447 0.0032 0.0266 0.0158 0.00627 0.0225 0.00315 0.0068 0.0206
2L:18156020-18156170:minus -40.2551 3.89908 9.66972 6.02752 -17.9796 -10.0204 -26.7064
3.375 -4.59633 0.0382 0.0025 0.000173 0.00246 0.00726 0.00399 0.0583 0.00516 0.0653
2L:18156020-18156170:plus -22.0408 0.954128 6.51376 7.95413 -8.33673 -26.602 -10.1376
-3.22222 4.02752 0.00296 0.00736 0.000912 0.000599 0.00101 0.0248 0.00316 0.0408 0.00428
2L:18158740-18158890:minus -32.1122 7.3211 4.00917 2.07339 10.6531 9.60204 -17.9083 4.55556
-2.06422 0.0178 0.000548 0.00267 0.0138 0.0000102 0.0000248 0.0147 0.00318 0.0339
2L:18158740-18158890:plus -31.8878 0.431193 -4.23853 5.06422 -27.2755 19.1531 -20.9725
5.69444 -0.229358 0.0164 0.00887 0.0405 0.00433 0.0302 7.95e-07 0.0245 0.00191 0.0202
2L:182960-183110:minus -32.7041 -5.37615 5.7156 0.220183 -8.96939 -0.0204082 -18.2477
5.52778 7.6422 0.0222 0.0487 0.00131 0.0253 0.00226 0.000411 0.0155 0.00207 0.000893
2L:182960-183110:plus -24.6735-3.40367 1.43119 2.61468 -28.0408 -7.5 -11.2385 3.13889 2.54128 0.00825
0.0288 0.0071 0.0109 0.0455 0.000614 0.00392 0.00566 0.00787
2L:18320020-18320170:minus -21.6327 5.58716 0.477064 2.66972 -18.6429 -18.0102 -16.3578 -3.58333
-0.477064 0.00215 0.00127 0.00989 0.0106 0.0121 0.00812 0.0115 0.0446 0.0218
2L:18320020-18320170:plus -31.7041 -0.33945 4.72477 11.2936 -17.0408 -8.27551 -6.08257 9.22222 7.15596
0.0146 0.0116 0.00199 0.0000139 0.00329 0.000919 0.00134 0.000278 0.00124
2L:18443300-18443450:minus -30.9184 2.97248 -4.59633 2.45872 0.846939 -17.4286 -14.5413 -4.08333
2.15596 0.0103 0.00351 0.0445 0.0116 0.000197 0.00586 0.00816 0.0502 0.00881
2L:18443300-18443450:plus -23.0306 6.47706 1.57798 -0.614679 -18.4898 -18.5 -21.633 7.33333
-2.77982 0.00505 0.000843 0.00674 0.0324 0.0116 0.0131 0.0276 0.000844 0.042
2L:18448960-18449110:minus -12.0408 13.4592 3.89908 5.86239 -17.449 -9.42857 -14.6697 2.04167
-4.76147 0.000311 0.000064 0.00279 0.00282 0.00484 0.0027 0.00838 0.00849 0.0686
2L:18448960-18449110:plus -24.449 3.45872 0.330275 6.14679 -18.6429 -0.540816 -28.5872 -0.625
-2.42202 0.00802 0.00294 0.0104 0.00224 0.0121 0.00054 0.0745 0.0202 0.0378
2L:18454520-18454670:minus -15.0408 13.4592 2.21101 7.99083 -17.1122 -19.0816 -9.69725 0.277778
6.18349 0.00162 0.000064 0.00534 0.00059 0.00341 0.017 0.00291 0.0153 0.00167
2L:18454520-18454670:plus -22.9898 4.61468 1.11009 4.08257 -18.1531 -18.3061 -8.17431 1.65278 5.24771
0.00482 0.0019 0.00796 0.00558 0.00829 0.0114 0.00218 0.00974 0.00282
2L:18484500-18484650:minus -31.1429 3.40367 8.21101 5.22018 -17.7857 -19.2041 -25.9725 -4.375
3.08257 0.0108 0.003 0.000392 0.00418 0.00676 0.0179 0.0533 0.0537 0.00681
2L:18484500-18484650:plus -31.2857 2.78899 0.715596 -2.21101 -17.7857 -28.4082 -6.44037 3.02778 11.8532
0.0113 0.00376 0.00912 0.055 0.00676 0.0615 0.00147 0.0059 0.0000428
2L:18501140-18501290:minus -24.3673 -0.926606 0.706422 11.0183 -18.1531 -27.3367 -11.633 0.791667
-0.46789 0.00791 0.014 0.00914 0.000052 0.00829 0.0347 0.00426 0.013 0.0218
2L:18501140-18501290:plus -23 0.816514 1.6422 6.00917 -17.449 -18.2347 -17.9541 -0.847222 -3.21101

0.00498 0.00773 0.00659 0.00255 0.00484 0.0104 0.0148 0.0216 0.0468
2L:18561720-18561870:minus -23.0816 -2.69725 3.10092 1.74312 0.94898 -18.1224 -13.9266 -0.0416667
-0.385321 0.00522 0.0237 0.00382 0.016 0.000134 0.00988 0.00714 0.017 0.0211
2L:18561720-18561870:plus -22.9898 -0.0366972 5.77982 -1.0367 -17.7551 -17.051 -8.68807 6.5 5.25688
0.00482 0.0104 0.00127 0.0371 0.00658 0.00479 0.00241 0.0013 0.0028
2L:18563220-18563370:minus 24.2143 3.06422 1.20183 6.47706 -7.56122 -17.3469 0.66055 -1.47222
7.68807 3.4e-08 0.0034 0.0077 0.00179 0.000419 0.0058 0.000241 0.0258 0.000841
2L:18563220-18563370:plus -20.8469 3.01835 1.06422 3.61468 -27.1531 -9.65306 -12.6972 -1.73611 3.25688
0.00178 0.00346 0.00808 0.0068 0.0249 0.0034 0.00539 0.0277 0.00632
2L:18564180-18564330:minus -32.8163 1.69725 7.0367 1.69725 -28.5306 -8.38776 -28.0092 1.33333
-2.21101 0.024 0.00561 0.000715 0.0163 0.061 0.00101 0.0689 0.0109 0.0354
2L:18564180-18564330:plus -39.9592 -1.01835 1.10092 6.12844 -17.4898 -17.2755 -17.0183 -0.513889
-2.26606 0.0348 0.0144 0.00798 0.00231 0.0053 0.00544 0.0129 0.0196 0.0359
2L:18565680-18565830:minus -21.3673 2 -1.46789 5.22018 -18.4184 -8.72449 -27.4679 4.11111 -4.41284
0.00195 0.00502 0.0184 0.00418 0.0101 0.00169 0.0642 0.00384 0.0624
2L:18565680-18565830:plus -40.5204 -0.651376 2.12844 5.30275 -18.7857 -18.4184 -23.6697 5.25 1.16514
0.0413 0.0128 0.00551 0.00396 0.0148 0.0127 0.0391 0.00235 0.0135
2L:18571840-18571990:minus -21.5918 1.63303 8.51376 2.43119 -28.3367 -18.8571 -13.8073 -0.583333
6.11927 0.00209 0.00575 0.00033 0.0117 0.0557 0.0151 0.00695 0.02 0.00178
2L:18571840-18571990:plus -4.7449 4.51376 3.11009 7.37615 -27.1939 -18.2347 -4.81651 2.93056 5.23853
0.000191 0.00198 0.00381 0.000951 0.0267 0.0104 0.000942 0.00612 0.00285
2L:18572220-18572370:minus -24.6327 -3.33945 6.10092 3.07339 -18.4592 -27.5306 -15.9541 -1.34722
3.88991 0.00824 0.0283 0.0011 0.00873 0.0109 0.0374 0.0108 0.0249 0.00467
2L:18572220-18572370:plus -20.7347 2.66055 11.2385 7.2844 -18.6735 -36.1122 11.2385 -1.88889 7.15596
0.00176 0.00394 0.0000688 0.00105 0.0123 0.0878 7.95e-06 0.0289 0.00124
2L:18576340-18576490:minus -23.3673 -1.88073 -0.0366972 1.45872 -8.86735 -18.1939 0.816514 1.47222
2.16514 0.00617 0.0188 0.0117 0.0179 0.00173 0.0101 0.000231 0.0104 0.00879
2L:18576340-18576490:plus -31.8163 0.275229 1.00917 6.12844 -8.86735 -0.316327 0.770642 3.51389
-0.284404 0.016 0.00938 0.00824 0.00231 0.00173 0.000524 0.000234 0.00489 0.0207
2L:18604760-18604910:minus -22.6633 -2.40367 -0.486239 2.55046 -26.5918 -27.3367 -14.7615 1.70833
1.69725 0.00384 0.0218 0.0136 0.0112 0.0186 0.0347 0.00855 0.00955 0.0108
2L:18604760-18604910:plus -31.2551 5.58716 -3.54128 3.69725 0.806122 -35.6735 -13.7982 -0.361111
1.90826 0.0113 0.00127 0.0335 0.00665 0.000229 0.0809 0.00693 0.0187 0.00974
2L:18605060-18605210:minus -30.7041 -1.63303 8.19266 0.376147 -18.3776 -8.79592 -16.1927 0.0972222
-3.83486 0.00993 0.0174 0.000395 0.0244 0.00926 0.0019 0.0112 0.0162 0.0533
2L:18605060-18605210:plus -40.6633 -3.9633 4.27523 -0.275229 -17.6735 -9.86735 -21.1651 2.52778
3.26606 0.0426 0.0335 0.0024 0.029 0.00572 0.00391 0.0253 0.00712 0.00627
2L:18606720-18606870:minus -23.7143 2.11009 0.174312 9.59633 -28.8265 -8.5 -28.0642 4.08333 -4.93578
0.0067 0.00482 0.0109 0.000275 0.0648 0.00126 0.0694 0.00388 0.0718
2L:18606720-18606870:plus -23.2653 13.4592 15 7.29358 -8.93878 -8.5 -20.367 7.41667 -3.16514 0.00593
0.000064 1.62e-06 0.00104 0.00216 0.00126 0.022 0.000807 0.0464
2L:18612200-18612350:minus -24.0306 -0.100917 -0.229358 2.62385 -7.67347 -8.94898 -2.41284
5.56944 1.69725 0.00723 0.0107 0.0125 0.0108 0.00055 0.00203 0.00049 0.00203 0.0108
2L:18612200-18612350:plus -41.3265 0.816514 1.36697 4.0367 -18.9796 -8.94898 -20.4862 5.93056 -6.22018
0.0541 0.00773 0.00726 0.00578 0.0155 0.00203 0.0224 0.00171 0.0938
2L:18617200-18617350:minus -13 -0.155963 1.92661 2.66055 -17.6429 -9.37755 -18.422 3.80556
2.42202 0.000563 0.0109 0.00594 0.0106 0.00562 0.00265 0.0159 0.00435 0.00816
2L:18617200-18617350:plus -13.5204 0.0825688 -0.853211 1.83486 -7.89796 -17.2755 -10.9083 7.90278
1.48624 0.00097 0.01 0.0152 0.0155 0.000678 0.00544 0.00366 0.000616 0.0118
2L:18622080-18622230:minus -50.6633 -0.853211 -0.394495 5.49541 -17.9796 -17.5714 -18.055
4.61111 1.68807 0.136 0.0137 0.0132 0.00355 0.00726 0.00616 0.0151 0.00311 0.0109
2L:18622080-18622230:plus -21.9286 2.97248 9.61468 10.5872 -17.6429 -0.469388 -19.6697 -3.41667
-1.80734 0.0027 0.00351 0.000178 0.000115 0.00562 0.000533 0.0195 0.0428 0.0316
2L:18625880-18626030:minus -22.8469 -0.477064 2.33028 7.33945 -9.20408 0.530612 -16.6606 -1.97222

-8.36697 0.00424 0.0121 0.00511 0.00102 0.00262 0.000203 0.0121 0.0296 0.146
2L:18625880-18626030:plus -22.9592 2.99083 0.110092 6.45872 -16.449 -18.051 -13.0459 7.30556 -2.90826
0.00475 0.00349 0.0112 0.00182 0.00307 0.00886 0.00584 0.000857 0.0435
2L:18627020-18627170:minus -23.898 1.74312 9.86239 7.21101 -27.2653 -17.2755 -20.7615 -0.722222
-1.38532 0.00693 0.00552 0.000155 0.0011 0.0302 0.00544 0.0236 0.0208 0.0288
2L:18627020-18627170:plus -30.6633 -0.669725 -3.90826 5.22018 -8.37755 -9.82653 -9.70642 3.13889
4.80734 0.00976 0.0129 0.037 0.00418 0.00106 0.00381 0.00291 0.00566 0.00334
2L:18629280-18629430:minus -30.2857 -0.311927 5.94495 -1.08257 -8.97959 -26.3776 -14.9908 -0.902778
9.55046 0.00887 0.0115 0.00118 0.0378 0.0023 0.0222 0.00896 0.0219 0.000321
2L:18629280-18629430:plus -30.6224 2.29358 0.284404 5.92661 -9.20408 -9.02041 -12.8991 0.0972222
-0.541284 0.00964 0.00451 0.0105 0.00266 0.00262 0.00213 0.00565 0.0162 0.0223
2L:18630840-18630990:minus -23.3367 -0.00917431 7.82569 6.56881 -17.1939 -36.0816 -9.36697 7.45833
0.788991 0.00614 0.0103 0.00048 0.0017 0.00377 0.0857 0.00274 0.000789 0.0151
2L:18630840-18630990:plus -23.5204 4.05505 -0.541284 5.24771 -26.2653 -19.3469 -14.633 -1.63889
3.66972 0.00645 0.00235 0.0138 0.00403 0.018 0.0194 0.00832 0.027 0.00514
2L:18631580-18631730:minus -24.4796 -1.38532 4.82569 5.55046 -18.3367 0.979592 -10.7431 -1.16667
-2.6789 0.00804 0.0162 0.00191 0.00345 0.00885 0.000136 0.00355 0.0237 0.0408
2L:18631580-18631730:plus -30.7755 1.85321 7.7156 11.2936 -17.3776 -18.0816 -10.4037 1.31944 -1.22018
0.0102 0.0053 0.000509 0.0000139 0.00419 0.00934 0.00332 0.0109 0.0278
2L:18650760-18650910:minus -22.8571 2.12844 3.75229 2.42202 -27.4592 -18.051 -21.5138 1.33333
-0.284404 0.00427 0.00479 0.00296 0.0118 0.0329 0.00886 0.027 0.0109 0.0207
2L:18650760-18650910:plus -12.9898 -0.87156 -3.26606 1.91743 -27 0.0510204 -1.3945 1.125 5.6055
0.000553 0.0138 0.0311 0.015 0.0233 0.000349 0.000387 0.0117 0.00232
2L:18653240-18653390:minus -31.551 1.86239 8.31193 7.20183 0.540816 0.387755 -18.8899 -3.19444
9.88073 0.0134 0.00528 0.00037 0.00112 0.000321 0.000217 0.0171 0.0406 0.000206
2L:18653240-18653390:plus -22.0408 -3.23853 -1.6055 9.08257 0.806122 -18.2041 -17.1376 -0.5 3.08257
0.00296 0.0275 0.0192 0.000444 0.000229 0.0103 0.0131 0.0195 0.00681
2L:18655940-18656090:minus -23.3673 -3.36697 4.55046 3.19266 -27.1224 -0.244898 -16.3486 11.8889
7.42202 0.00617 0.0285 0.00214 0.00829 0.0245 0.000496 0.0115 0.0000344 0.00106
2L:18655940-18656090:plus -22.551 -3.6055 7.84404 5.57798 -27.1224 0.530612 -11.2752 4.38889 4.6055
0.00359 0.0304 0.000476 0.0034 0.0245 0.000203 0.00395 0.00342 0.00352
2L:18665200-18665350:minus -41.3367 3.64286 3.7156 -0.926606 0.612245 -0.469388 -17.945
5.26389 2.33028 0.0542 0.00159 0.00301 0.036 0.000269 0.000533 0.0148 0.00233 0.00836
2L:18665200-18665350:plus -32.3776 2.59633 4.89908 2.63303 -27.602 -17.8265 -22.9725 1.27778
-0.0733945 0.0189 0.00403 0.00185 0.0107 0.0364 0.00699 0.0349 0.0111 0.0188
2L:18666040-18666190:minus -21.9286 6.16514 9.09174 7.21101 -19.2755 -18.0816 -16.1927 4.98611
1.76147 0.0027 0.00098 0.000241 0.0011 0.0164 0.00934 0.0112 0.00264 0.0104
2L:18666040-18666190:plus -31.4388 -0.651376 8.09174 3.70642 -18.1939 -17.7857 -5.04587 3.36111
0.825688 0.0122 0.0128 0.000416 0.0066 0.0086 0.0068 0.00101 0.00519 0.0149
2L:18688820-18688970:minus -23.7245 -3.38532 1.87156 2.55963 -18.4898 -18.1939 -15.6514 2.63889
5.49541 0.0067 0.0287 0.00606 0.0111 0.0116 0.0101 0.0102 0.00684 0.0025
2L:18688820-18688970:plus -23.2245 0.110092 5.58716 2.93578 -17.7857 -18.3469 -20.6881 0.263889 1.89908
0.00576 0.00993 0.00139 0.00927 0.00676 0.0124 0.0232 0.0154 0.0098
2L:18689600-18689750:minus -24.4796 1.75229 8.18349 1.46789 -17.4898 -9.42857 -10.6881 0.680556
1.87156 0.00804 0.0055 0.000397 0.0179 0.0053 0.0027 0.00351 0.0135 0.00989
2L:18689600-18689750:plus -32.6633 -1.38532 -0.899083 2.97248 -19.0204 0.0510204 -20.5229 5.76389
5.44954 0.0216 0.0162 0.0155 0.00909 0.0158 0.000349 0.0226 0.00185 0.00253
2L:18694120-18694270:minus -12.8878 0.192661 12.0734 4.07339 -26.9286 -0.173469 -4.33945 -2.51389
2.74312 0.000523 0.00965 0.0000408 0.00562 0.0214 0.000456 0.000822 0.0341 0.00751
2L:18694120-18694270:plus -24.1939 -0.779817 5.19266 2.16514 -17.3367 -8.30612 -26.3578 3.97222
5.70642 0.00753 0.0134 0.00164 0.0132 0.00391 0.000973 0.0559 0.00407 0.00222
2L:18694720-18694870:minus -23 -1.11009 0.0458716 11.0275 -9.23469 -0.204082 -5.19266
-0.0972222 -4.62385 0.00498 0.0148 0.0114 0.0000401 0.00277 0.000463 0.00105 0.0172 0.066
2L:18694720-18694870:plus -33 0.192661 4.36697 3.86239 -18.2245 -0.173469 -15.5505 6.40278 -3.49541

0.0258 0.00965 0.00231 0.00622 0.00877 0.000456 0.00999 0.00136 0.0495
2L:18695360-18695510:minus -33.9286 0.284404 1.94495 1.88991 -27.5612 -19.1224 -8.16514 9.88889
1.48624 0.0303 0.00935 0.0059 0.0151 0.0354 0.0175 0.00218 0.000178 0.0118
2L:18695360-18695510:plus -23.2653 -2.97248 10.0367 7.55963 -17.6837 -17.7551 -12.4587 3 3.87156
0.00593 0.0256 0.000139 0.000807 0.0058 0.00643 0.00511 0.00597 0.00477
2L:18702940-18703090:minus -31.4796 3.86239 0.0458716 0.59633 -8.67347 -18.3469 -12.5688 -1.59722
7.15596 0.0128 0.00253 0.0114 0.023 0.00154 0.0124 0.00524 0.0267 0.00124
2L:18702940-18703090:plus -23.0306 9.55046 1.89908 0.174312 -18.4184 0.0510204 4.46789 -2.76389
-2.19266 0.00505 0.000168 0.006 0.0257 0.0101 0.000349 0.0000696 0.0364 0.0352
2L:18703280-18703430:minus -24.1122 8.33028 -1.20183 3.59633 -27.3061 -9.37755 -16.7064 -0.236111
-0.394495 0.00738 0.000319 0.017 0.00693 0.0315 0.00265 0.0122 0.018 0.0212
2L:18703280-18703430:plus -22.0306 1.77982 12.9083 3.42202 -7.40816 -8.65306 -11.9908 -2.08333 3.21101
0.00293 0.00544 0.0000224 0.00751 0.000401 0.00156 0.0046 0.0305 0.00644
2L:18705720-18705870:minus -22.6633 -0.302752 5.41284 5.86239 -26.9286 -19.0102 -13.8991 -1.02778
0.853211 0.00384 0.0114 0.00149 0.00282 0.0214 0.0159 0.00709 0.0227 0.0148
2L:18705720-18705870:plus -34.0408 -0.247706 5.74312 11.0275 -17.2245 -9.94898 -19.2385 0.597222
3.7156 0.0309 0.0112 0.00129 0.0000401 0.00384 0.00393 0.0181 0.0139 0.00502
2L:18708060-18708210:minus -32.5918 1.52294 6.26606 5.85321 -17.1531 -27.6327 -15.3486 -6.05556
3.56881 0.021 0.00598 0.00103 0.00287 0.00364 0.043 0.00961 0.077 0.00543
2L:18708060-18708210:plus -11.8878 -4.5 0.770642 -0.550459 -8.26531 -9.42857 -14.9633 -3.40278 -1.85321
0.00029 0.00216 0.00895 0.0317 0.000934 0.0027 0.00891 0.0427 0.032
2L:18708640-18708790:minus -14.7755 -2.59633 3.89908 5.22018 20.1735 -27.3367 -3.97248 -0.222222
7.6422 0.00157 0.023 0.00279 0.00418 5.68e-07 0.0347 0.00074 0.0179 0.000893
2L:18708640-18708790:plus -40.0306 -3.6055 2.66972 9.88073 -7.67347 0.0510204 -12.9817 4.43056
3.49541 0.0357 0.0304 0.0045 0.000183 0.00055 0.000349 0.00576 0.00336 0.0057
2L:18713640-18713790:minus -30.9898 -0.146789 2.65138 2.31193 -8.67347 -18.7857 -15.1101 2.41667
-1.69725 0.0105 0.0108 0.00453 0.0123 0.00154 0.0146 0.00917 0.00742 0.0308
2L:18713640-18713790:plus -22.0408 -0.614679 6.69725 3.97248 -18.8265 -18.1224 -15.4404 2.97222
3.88073 0.00296 0.0127 0.000836 0.00587 0.0149 0.00988 0.00978 0.00603 0.00475
2L:18831080-18831230:minus -32.0714 2.41284 4.19266 4.77064 -8.57143 -18.0102 -23.3945 0.0833333
-0.458716 0.0176 0.00431 0.00248 0.00457 0.00113 0.00812 0.0374 0.0163 0.0217
2L:18831080-18831230:plus -13.4082 13.4592 -3.33945 11.2936 10.7245 -16.7857 -11.2661 2 -0.697248
0.000867 0.000064 0.0317 0.0000139 5.2e-06 0.00426 0.00394 0.00862 0.0239
2L:18839060-18839210:minus -32.7347 -0.275229 4.90826 6.12844 -7.67347 -28.4082 -8.24771 2.65278
-0.0733945 0.0225 0.0113 0.00185 0.00231 0.00055 0.0615 0.00221 0.0068 0.0188
2L:18839060-18839210:plus -30.1429 0.761468 -0.605505 3.18349 0.908163 -9.42857 -9.22936 -5.55556
-3.85321 0.00866 0.00789 0.0141 0.00837 0.000153 0.0027 0.00267 0.0694 0.0534
2L:18839620-18839770:minus -33.0816 0.504587 3.77064 -2.38532 -27.2653 -26.5612 -8.00917 2.72222
1.73394 0.0268 0.00865 0.00294 0.058 0.0302 0.0237 0.00211 0.00663 0.0106
2L:18839620-18839770:plus -21.6939 0.302752 -1.46789 3.23853 0.581633 -27.5306 -12.9083 -0.166667
3.66972 0.00226 0.00929 0.0184 0.00815 0.000292 0.0374 0.00566 0.0176 0.00514
2L:1884640-1884790:minus -32.8163 0.779817 -4.54128 -0.0183486 -28.2653 -19.0102 -16.8899 0.180556
3.66055 0.024 0.00784 0.0438 0.027 0.0537 0.0159 0.0126 0.0158 0.00515
2L:1884640-1884790:plus -22.5918 -3.33028 -0.376147 3.61468 -26.8571 -8.72449 -18.6422 -0.722222
2.78899 0.00365 0.0282 0.0131 0.0068 0.0198 0.00169 0.0165 0.0208 0.00745
2L:18857640-18857790:minus -24.0714 6.70642 5.6789 6.74312 -27.2347 -18.8571 -18.1468 1.93056
-3.62385 0.00731 0.000754 0.00133 0.0015 0.0283 0.0151 0.0153 0.00883 0.051
2L:18857640-18857790:plus -39.9184 1.11927 -3.26606 4.22936 -18.7857 -18.2755 -8.82569 1.22222 -6.37615
0.0344 0.00693 0.0311 0.00525 0.0148 0.0112 0.00248 0.0113 0.0975
2L:18859220-18859370:minus -32.2245 7.04587 9.56881 3.42202 20.2041 -8.45918 -21.0367 -3.26389
-3.72477 0.0183 0.000633 0.000183 0.00751 3.64e-07 0.00111 0.0248 0.0413 0.0519
2L:18859220-18859370:plus -33.1429 7.3945 -1.99083 2 -18.7551 -19.3469 -17.2202 2.61111 1.26606
0.0272 0.000526 0.0216 0.0143 0.0144 0.0194 0.0133 0.00691 0.013
2L:18936200-18936350:minus -21.9592 -4.57798 3.66055 2.53211 19.9082 -9.02041 -17.9174 3.98611

-4.11927 0.00279 0.0395 0.00307 0.0113 8.13e-07 0.00213 0.0148 0.00404 0.0572
2L:18936200-18936350:plus -22.7347 -3.53211 -0.614679 1.48624 -17.3776 -8.20408 -3.12844 0.361111
6.02752 0.00403 0.0298 0.0141 0.0177 0.00419 0.000796 0.000587 0.0149 0.00183
2L:18943360-18943510:minus -22.0408 1.48624 3.11009 5.91743 -18.7857 -18.2041 -17.9908 -0.902778
7.31193 0.00296 0.00606 0.00381 0.00267 0.0148 0.0103 0.0149 0.0219 0.00112
2L:18943360-18943510:plus -22.1531 -2.93578 11.6697 4.17431 -8.89796 -9.42857 -2.80734 -5.31944 -6.69725
0.0031 0.0253 0.0000536 0.00537 0.00189 0.0027 0.000541 0.0661 0.106
2L:18948840-18948990:minus -33.3776 -2.58716 -1.44954 5.47706 -18.7143 -7.45918 -23.8073 4.06944
-4.12844 0.0282 0.023 0.0183 0.0036 0.0136 0.000582 0.0399 0.00391 0.0573
2L:18948840-18948990:plus -40.9898 4.75229 10.8349 3.59633 -27.1531 -8.72449 -14.8073 1.98611 -3.95413
0.0455 0.0018 0.0000885 0.00693 0.0249 0.00169 0.00863 0.00866 0.055
2L:18951800-18951950:minus -31.7347 15.5321 11.5321 7.15596 -18.449 0.316327 -18.3028 2.16667
7.68807 0.0149 9.74e-07 0.0000579 0.00117 0.0107 0.000243 0.0157 0.00812 0.000841
2L:18951800-18951950:plus -33.0408 -4.19266 -4.23853 1.84404 -18.7143 -7.79592 -19.9083 2.5 4.83486
0.0264 0.0356 0.0405 0.0154 0.0136 0.000723 0.0203 0.0072 0.00329
2L:18954460-18954610:minus -23.2143 0.0642202 -1.61468 1.26606 -27.5306 -17.7857 -13.6422 14.5972
-0.651376 0.00559 0.0101 0.0193 0.0191 0.0351 0.0068 0.00669 1e-06 0.0235
2L:18954460-18954610:plus -31.5204 1.53211 3.11927 2.99083 -18.1939 -27.602 -19.6422 -1.13889 2.78899
0.0131 0.00596 0.00379 0.00899 0.0086 0.0416 0.0194 0.0235 0.00745
2L:18957100-18957250:minus -31.8061 1.33945 2.51376 2.70642 -17.4898 -18.5612 -3.57798 0.569444
3.66972 0.0158 0.0064 0.00478 0.0103 0.0053 0.0132 0.000663 0.014 0.00514
2L:18957100-18957250:plus -31.4796 -2.93578 13.4128 7.45872 -27.2245 -18.0102 -11.5963 -1.94444 1.2844
0.0128 0.0253 0.0000141 0.000894 0.0272 0.00812 0.00422 0.0294 0.0129
2L:18965640-18965790:minus -21.3265 -1.20183 3.94495 6.05505 -18.3878 -8.42857 -11.4954 2.95833
0.66055 0.00193 0.0153 0.00274 0.0024 0.00931 0.00104 0.00413 0.00606 0.0156
2L:18965640-18965790:plus -34.0306 -0.816514 -1.41284 -0.568807 -18.4184 -8.79592 -22.9358 6.55556
-1.11927 0.0307 0.0135 0.0181 0.032 0.0101 0.0019 0.0347 0.00126 0.027
2L:18966060-18966210:minus -24.5204 0.0183486 0.807339 3.01835 -8.71429 -10.0204 -22.6239 0.958333
-3.05505 0.00813 0.0102 0.00884 0.0089 0.00159 0.00399 0.0329 0.0123 0.0452
2L:18966060-18966210:plus -31.5918 -0.688073 2.19266 0.678899 -19.0102 -28.1837 -21.3853 6.375
3.88073 0.0136 0.013 0.00538 0.0225 0.0157 0.0584 0.0264 0.00138 0.00475
2L:18968820-18968970:minus -12.2143 1.92661 5.85321 3.86239 20.2041 -35.3776 -6.6789 -4.20833
7.63303 0.000367 0.00516 0.00123 0.00622 3.64e-07 0.0783 0.00156 0.0517 0.000928
2L:18968820-18968970:plus -23.9184 13.5306 4.87156 3.88991 -18.4184 -9.30612 -18.9083 -3.875 -4.77982
0.00695 0.0000206 0.00188 0.00608 0.0101 0.00257 0.0172 0.0478 0.069
2L:18974260-18974410:minus -13.2551 5.44037 1.76147 10.0275 -18.1531 -17.7857 -5.66972 -0.319444
6.95413 0.000751 0.00135 0.00631 0.000156 0.00829 0.0068 0.0012 0.0184 0.00136
2L:18974260-18974410:plus -42.8163 -0.266055 -0.53211 5.57798 -8.60204 -9.02041 -17.5963 2.09722
-2.83486 0.0865 0.0113 0.0138 0.0034 0.00125 0.00213 0.0141 0.00832 0.0427
2L:18982060-18982210:minus -32.6327 3.52294 -0.504587 2.7156 -17.7551 -8.27551 -8.9633 0.638889
3.88991 0.0212 0.00287 0.0137 0.0102 0.00658 0.000919 0.00254 0.0137 0.00467
2L:18982060-18982210:plus -40.9898 3.24771 4.38532 7.17431 -7.63265 -9.72449 -21.578 -1.30556 -0.908257
0.0455 0.00317 0.0023 0.00115 0.000479 0.00352 0.0273 0.0246 0.0255
2L:18983620-18983770:minus -33.0408 -0.183486 -2.29358 7.45872 -28.2653 0.244898 -18.2018 5.18056
2.73394 0.0264 0.011 0.0236 0.000894 0.0537 0.000285 0.0154 0.00242 0.00756
2L:18983620-18983770:plus -24.2959 3.68367 2.88991 7.00917 -28.5306 -8.42857 -19.1284 4.375 -5.43119
0.00781 0.00144 0.00414 0.00126 0.061 0.00104 0.0178 0.00344 0.0794
2L:18984120-18984270:minus -24 -0.779817 -0.688073 2.33945 -17.6735 -18.7857 -13.2752 3.93056
1.61468 0.00719 0.0134 0.0145 0.0122 0.00572 0.0146 0.00616 0.00413 0.0112
2L:18984120-18984270:plus -14.5612 -2.24771 0.59633 5.45872 -26.9286 -19.4184 -12.4495 3.76389 1.05505
0.00147 0.0209 0.00949 0.00363 0.0214 0.0198 0.0051 0.00442 0.014
2L:18987260-18987410:minus -21.6633 1.84404 3.69725 7.27523 2.21429 9.7551 -7.00917 4.22222
5.22936 0.00223 0.00532 0.00303 0.00109 0.0000277 0.0000198 0.00169 0.00367 0.00288
2L:18987260-18987410:plus -21.2143 2.01835 9.22936 7.2844 -8.96939 0.540816 -12.2385 2.56944 4.51376

0.00189 0.00499 0.000221 0.00105 0.00226 0.0002 0.00486 0.00701 0.0036
2L:19003260-19003410:minus -22.0714 0.66055 -2.3945 6.72477 -8.93878 -6.5 -22.2569 3.125 6.95413
0.00299 0.00818 0.0242 0.00154 0.00216 0.000542 0.0309 0.00569 0.00136
2L:19003260-19003410:plus -11.551 6.74312 2.58716 3.61468 -18.4184 -18.0102 -3.62385 -1.44444 7.04587
0.000255 0.00074 0.00465 0.0068 0.0101 0.00812 0.000671 0.0256 0.00132
2L:19009300-19009450:minus -22.4796 -1.78899 3.44954 7.7156 10.6224 -8.20408 -20.3853 -2.22222
-1.06422 0.00352 0.0183 0.00334 0.000691 0.0000113 0.000796 0.022 0.0316 0.0266
2L:19009300-19009450:plus -32.4796 0.422018 -3.2844 1.36697 -17.7143 -17.9796 -19.2018 4.80556 3.14679
0.02 0.0089 0.0312 0.0186 0.00627 0.00764 0.018 0.00286 0.00666
2L:19033460-19033610:minus -21.8163 13.4592 13.0183 6.33028 -27.1939 -9.42857 -18.1468 0.611111
3.93578 0.00244 0.000064 0.0000203 0.00198 0.0267 0.0027 0.0153 0.0138 0.00456
2L:19033460-19033610:plus -24.2245 13.8991 -3.88991 1.78899 -19.051 -0.469388 -16.8807 -0.0694444
1.69725 0.00763 9.34e-06 0.0369 0.0158 0.0162 0.000533 0.0126 0.0171 0.0108
2L:19034540-19034690:minus -23.5612 9.94495 4.87156 2.05505 -17.449 -0.244898 -16.0459 8.63889
-0.431193 0.00653 0.000137 0.00188 0.0139 0.00484 0.000496 0.0109 0.000401 0.0214
2L:19034540-19034690:plus -31.551 9.48624 1.38532 2.42202 -17.3776 -8.5 -17.7156 0.75 5.3945 0.0134
0.000174 0.00721 0.0118 0.00419 0.00126 0.0143 0.0132 0.00263
2L:19035120-19035270:minus -39.9898 0.963303 -0.0733945 5.22018 -8.64286 -26.5306 -9.24771 1.73611
1.63303 0.0351 0.00734 0.0119 0.00418 0.00139 0.023 0.00268 0.00946 0.0112
2L:19035120-19035270:plus -23.1837 -0.302752 0.12844 3.18349 -18.4796 -19.051 -7.36697 3.29167
1.05505 0.00553 0.0114 0.0111 0.00837 0.011 0.0165 0.00184 0.00533 0.014
2L:19045180-19045330:minus -14.449 3.44037 0.587156 2.11927 -27.0102 -18.2755 -16.8899 7.06944
1.16514 0.0014 0.00296 0.00952 0.0135 0.0237 0.0112 0.0126 0.00097 0.0135
2L:19045180-19045330:plus -22.9592 15.5321 3.73394 1.78899 -26.8571 -17.0816 -13.1284 4.88889 8.10092
0.00475 9.74e-07 0.00299 0.0158 0.0198 0.00487 0.00595 0.00276 0.000623
2L:19049020-19049170:minus -41.2143 -1.11009 -0.798165 1.84404 -16.7143 -18.0816 -30.0642 6.52778
0.798165 0.0494 0.0148 0.015 0.0154 0.00319 0.00934 0.0917 0.00128 0.0151
2L:19049020-19049170:plus -23.0306 12.9725 -0.0733945 5.01835 -19.2755 -18.2755 -20.211 -0.0277778
-3 0.00505 0.0000227 0.0119 0.00438 0.0164 0.0112 0.0214 0.0169 0.0447
2L:19051800-19051950:minus -31.6633 13.4592 -5.6422 1.26606 -18.4898 -9.79592 -4.10092 4 -4.84404
0.0141 0.000064 0.058 0.0191 0.0116 0.00377 0.000768 0.00402 0.0701
2L:19051800-19051950:plus -39.949 1.66972 5.6055 1.91743 -27.602 -7.5 -12.1743 2.16667 3.6789
0.0345 0.00567 0.00137 0.015 0.0364 0.000614 0.00479 0.00812 0.00506
2L:19054000-19054150:minus -23.5612 0.889908 -2.2844 2.66055 -8.93878 -18.9286 -16.0183 1.68056
-0.697248 0.00653 0.00753 0.0235 0.0106 0.00216 0.0153 0.0109 0.00964 0.0239
2L:19054000-19054150:plus -32.5408 -1.65138 5.98165 3.95413 20.4694 -9.0102 -17.1743 1.45833 1.44037
0.0204 0.0175 0.00116 0.00593 1.65e-07 0.00208 0.0132 0.0104 0.012
2L:19054320-19054470:minus -13.1429 -0.385321 5.07339 4.75229 -8.96939 -18.7143 -15.1284 2.91667
3.33028 0.00065 0.0117 0.00172 0.00461 0.00226 0.014 0.00921 0.00616 0.00612
2L:19054320-19054470:plus -22 -2.54128 4.70642 4.33945 -18.1122 -26.8265 -16.211 0.638889 3.66972
0.00291 0.0227 0.00201 0.00509 0.00771 0.0285 0.0113 0.0137 0.00514
2L:19063220-19063370:minus -31.102 -2.42202 -1.93578 3.65138 -17.1531 -8.53061 -18.1468 5.30556
1.74312 0.0106 0.0219 0.0212 0.00668 0.00364 0.00136 0.0153 0.00229 0.0105
2L:19063220-19063370:plus -31.4082 13.5306 5.22018 3.61468 -18.1531 -7.94898 -22.7706 -4.29167 -0.605505
0.0119 0.0000206 0.00162 0.0068 0.00829 0.00074 0.0338 0.0527 0.023
2L:19064800-19064950:minus -31.4796 14.2294 -1.01835 0.908257 -9.23469 -8.27551 -21.5872 0.5 3.80734
0.0128 6.48e-06 0.0161 0.0211 0.00277 0.000919 0.0274 0.0143 0.00484
2L:19064800-19064950:plus -34.0714 13.3878 -0.110092 0.587156 -28.3061 -8.57143 -14.5688 2.80556
1.95413 0.031 0.0000869 0.012 0.0231 0.0553 0.00149 0.00821 0.00642 0.00964
2L:19077080-19077230:minus -39.0306 4.97248 -1.93578 3.80734 -18.1939 -19.5 -22.2018 2.20833
1.62385 0.0326 0.00165 0.0212 0.00627 0.0086 0.0199 0.0306 0.008 0.0112
2L:19077080-19077230:plus -33.2959 1.14679 1.88073 0.486239 -27.3061 -18.5714 -19.0734 8.51389 3.24771
0.0279 0.00686 0.00604 0.0237 0.0315 0.0136 0.0177 0.000433 0.00636
2L:1909840-1909990:minus -22.9286 0.311927 2.36697 3.61468 -26.2347 -8.72449 -20.1101 1.52778 0.559633

0.0046 0.00925 0.00505 0.0068 0.0177 0.00169 0.021 0.0102 0.016
2L:1909840-1909990:plus -30.949 1.82569 4.90826 3.17431 -17.4898 -9.94898 -8.70642 6.63889 4.02752
0.0104 0.00535 0.00185 0.00842 0.0053 0.00393 0.00242 0.00121 0.00428
2L:19114320-19114470:minus -34.1531 3.42202 5.12844 1.58716 -27.1939 -0.204082 -7.29358 4.48611
3.97248 0.0314 0.00298 0.00169 0.017 0.0267 0.000463 0.00181 0.00328 0.00444
2L:19114320-19114470:plus -32.1429 6.33028 15.6972 5.57798 -18.1224 -27.3367 -19.2202 3.125 -4.24771
0.0179 0.000904 3.11e-07 0.0034 0.00782 0.0347 0.0181 0.00569 0.0591
2L:1911560-1911710:minus -32.102 2.49541 12.9083 -0.633028 -18.5204 -19.1224 -4 0.111111 2.75229
0.0177 0.00418 0.0000224 0.0327 0.0117 0.0175 0.000746 0.0162 0.00748
2L:1911560-1911710:plus -21.8163 -0.412844 0.568807 9.08257 -17.7143 -17.9388 -8.23853 -5.68056
5.13761 0.00244 0.0119 0.00958 0.000444 0.00627 0.00738 0.00221 0.0713 0.00298
2L:19123740-19123890:minus -12.1531 13.9817 6.93578 2.66055 -8.11224 -7.79592 -6.48624 9.77778
-2.11927 0.000352 8.73e-06 0.00075 0.0106 0.000838 0.000723 0.00149 0.000192 0.0344
2L:19123740-19123890:plus -33.3367 13.5306 2.31193 5.45872 -18.0102 -19.6429 -22.3578 5.20833 3.46789
0.0281 0.0000206 0.00515 0.00363 0.00732 0.0204 0.0314 0.00239 0.00582
2L:19126960-19127110:minus -22.9592 10.8165 -2.83486 2.77982 -8.64286 -7.93878 -16.7982 4.90278
1.90826 0.00475 0.0000878 0.0275 0.00997 0.00139 0.000736 0.0124 0.00274 0.00974
2L:19126960-19127110:plus -33.551 0.541284 15.6606 9.49541 -17.9796 -8.68367 -20.0826 1.77778 9.14679
0.0288 0.00854 3.94e-07 0.000308 0.00726 0.00157 0.0209 0.00932 0.000414
2L:19131840-19131990:minus -20.8878 8.68807 9.11927 10.5963 -8.93878 -7.57143 -6.23853 4.29167
-0.908257 0.0018 0.000264 0.000237 0.0001 0.00216 0.000644 0.0014 0.00356 0.0255
2L:19131840-19131990:plus -31.6327 -0.302752 0.366972 0.201835 -19.0102 -18.6327 -19.2018 7.18056
0.275229 0.0138 0.0114 0.0103 0.0255 0.0157 0.0137 0.018 0.000915 0.0171
2L:19132260-19132410:minus -32.6327 -0.0917431 -2.91743 0.788991 -18.0918 -18.7143 -21.7431 9.23611
-0.247706 0.0212 0.0106 0.0282 0.0218 0.00761 0.014 0.0282 0.000275 0.0204
2L:19132260-19132410:plus -40.7755 13.9817 -1.77064 6.74312 -26.8878 -18.0816 -15.5413 6.94444 0.238532
0.0433 8.73e-06 0.0202 0.0015 0.0199 0.00934 0.00997 0.00103 0.0173
2L:19133500-19133650:minus -23.6633 10.3578 -0.93578 3.77064 -8.89796 -19.2347 -11.7523 1.86111
4.56881 0.00665 0.000111 0.0156 0.00637 0.00189 0.018 0.00437 0.00905 0.00356
2L:19133500-19133650:plus -13.2245 13.4592 0.458716 5.85321 -18.7551 -26.9694 -10 11.3194 3.46789
0.000738 0.000064 0.00995 0.00287 0.0144 0.0304 0.00308 0.000058 0.00582
2L:19134400-19134550:minus -21.8571 0.174312 -1.42202 6.12844 -28.0102 -16.9796 -20.8716 0.555556 3
0.00249 0.00971 0.0182 0.00231 0.0449 0.0044 0.024 0.014 0.00702
2L:19134400-19134550:plus -24.1122 -3.16514 0.825688 5.58716 -18.1531 -19.0102 -14.8073 2.93056 1.95413
0.00738 0.027 0.00878 0.00334 0.00829 0.0159 0.00863 0.00612 0.00964
2L:19135040-19135190:minus -13.4796 -4.20183 0.715596 1.9633 -18.8265 2.05102 -16.1101 -0.875
12.2752 0.000935 0.0357 0.00912 0.0146 0.0149 0.0000468 0.0111 0.0218 0.0000141
2L:19135040-19135190:plus -20.398 2.46789 -2.44954 3.6055 -17.1122 -8.72449 -8.95413 6.54167 1.01835
0.00166 0.00422 0.0246 0.00686 0.00341 0.00169 0.00254 0.00127 0.0142
2L:19140760-19140910:minus -32.9184 0.174312 -1.55963 -2.07339 -18.5204 -27.6327 -11.4037 3.98611
1.48624 0.0248 0.00971 0.0189 0.0525 0.0117 0.043 0.00406 0.00404 0.0118
2L:19140760-19140910:plus -13.4898 -2.33028 6.09174 7.55963 -17.7551 -28.602 -13.055 1.97222 3.92661
0.000946 0.0214 0.00111 0.000807 0.00658 0.0648 0.00585 0.0087 0.00461
2L:19141380-19141530:minus -30.8571 4.34862 3.73394 1.62385 -17.3776 0.826531 -34.7248 -1.93056
-2.83486 0.0103 0.0021 0.00299 0.0168 0.00419 0.000153 0.156 0.0293 0.0427
2L:19141380-19141530:plus -20.8571 2.89908 0.155963 3.29358 0.94898 -9.02041 -20.3945 0.180556 0.0275229
0.00178 0.00361 0.011 0.00791 0.000134 0.00213 0.0221 0.0158 0.0183
2L:19142020-19142170:minus -33.0408 3.33945 1.46789 -0.807339 -8.86735 -0.316327 -16.5046
7.69444 -0.59633 0.0264 0.00307 0.007 0.0345 0.00173 0.000524 0.0118 0.000693 0.0229
2L:19142020-19142170:plus -22.5918 -3.34862 -0.559633 3.45872 -27.5306 -8.64286 -16.1835 3.11111
-2.66972 0.00365 0.0284 0.0139 0.00731 0.0351 0.00154 0.0112 0.00572 0.0407
2L:19142400-19142550:minus 5.2551 3.6789 5.93578 10.5963 -28.0408 -27.3367 -14.5229 -2.72222
4.09174 0.0000122 0.00271 0.00119 0.0001 0.0455 0.0347 0.00813 0.036 0.00416
2L:19142400-19142550:plus -23.0714 2.26606 7.25688 6.12844 -27.1939 -18.0102 -19.945 -3.84722 -0.40367

0.0052 0.00455 0.000641 0.00231 0.0267 0.00812 0.0204 0.0475 0.0213
2L:19146100-19146250:minus -22.4388 -5.58716 1.68807 6.86239 -26.8163 -28.6327 -9.48624 3.91667
7.73394 0.00348 0.0514 0.00648 0.00136 0.0195 0.066 0.0028 0.00416 0.000784
2L:19146100-19146250:plus -32.7755 -1.61468 1.11009 -0.376147 -18.6837 -0.244898 -11.0183 1.875
1.85321 0.0234 0.0173 0.00796 0.0299 0.0126 0.000496 0.00375 0.00901 0.01
2L:19146980-19147130:minus -31.6633 2.0367 2.29358 -2.75229 -8.97959 -18.8571 -14.2569 0.416667
9.92661 0.0141 0.00495 0.00518 0.0649 0.0023 0.0151 0.00768 0.0147 0.000193
2L:19146980-19147130:plus -13.2653 -0.211009 8.22018 2.66055 -27.3061 -18.051 -15.789 -1.27778
6.04587 0.000759 0.0111 0.00039 0.0106 0.0315 0.00886 0.0104 0.0244 0.00181
2L:19155060-19155210:minus -33.0408 2.37615 -1.59633 3.51376 -17.449 -0.204082 -14.8257 8.26389
3.88991 0.0264 0.00437 0.0192 0.00709 0.00484 0.000463 0.00866 0.000502 0.00467
2L:19155060-19155210:plus -13.449 1.01835 8.77982 7.77982 -17.8571 -8.93878 -13.5321 4.19444 3.15596
0.000914 0.00719 0.000286 0.000652 0.00698 0.00201 0.00653 0.00371 0.00659
2L:19156080-19156230:minus -24.4796 -1.18349 1.98165 3.54128 -18.2245 -27.8265 -18.7706 -0.458333
1.86239 0.00804 0.0152 0.00582 0.00702 0.00877 0.0498 0.0168 0.0192 0.00994
2L:19156080-19156230:plus -31.4388 2.50459 0.908257 3.76147 -19.051 0.826531 -11.8073 1.31944 2.27523
0.0122 0.00417 0.00853 0.00645 0.0162 0.000153 0.00442 0.0109 0.00846
2L:19156860-19157010:minus -13.1429 1.57798 0.431193 3.13761 -18.2245 -9.23469 -6.50459 5.125
1.58716 0.00065 0.00586 0.01 0.00854 0.00877 0.00238 0.00149 0.00248 0.0113
2L:19156860-19157010:plus -21.7755 4.65138 -2.26606 0.66055 -18.4184 -17.7143 1.88991 2.5 -0.46789
0.00241 0.00187 0.0234 0.0225 0.0101 0.00634 0.000171 0.0072 0.0218
2L:19158120-19158270:minus -30.2143 0.770642 1.7156 2.09174 -18.1531 -26.602 -9.29358 6.47222
9.45872 0.0088 0.00786 0.00642 0.0137 0.00829 0.0248 0.0027 0.00132 0.000337
2L:19158120-19158270:plus -31.3673 -6 0.0733945 3.02752 -17.3367 -17.7143 -10.1193 2.66667 1.80734
0.0116 0.0571 0.0113 0.00887 0.00391 0.00634 0.00315 0.00677 0.0102
2L:19160020-19160170:minus -22.9592 1.07339 2.05505 9.76147 -8.60204 0.979592 -9.50459 6.125
-0.174312 0.00475 0.00705 0.00566 0.000212 0.00125 0.000136 0.00281 0.00156 0.0196
2L:19160020-19160170:plus -24.3367 -0.568807 8.20183 1.37615 -17.4184 -8.53061 -17.1468 5.48611
7.46789 0.00789 0.0125 0.000393 0.0185 0.00457 0.00136 0.0131 0.00211 0.00102
2L:19160840-19160990:minus -2.63265 -2.41284 3 2.83486 -18.7449 -19.4184 -10.4495 1.79167 9.97248
0.0000358 0.0219 0.00397 0.0097 0.0138 0.0198 0.00335 0.00928 0.00016
2L:19160840-19160990:plus -23.2959 3.97248 2.36697 3.41284 -17.7143 -27.3776 -1.6789 -0.930556
3.7156 0.00605 0.00243 0.00505 0.00756 0.00627 0.0351 0.000413 0.0221 0.00502
2L:19161720-19161870:minus -40.1735 -3.95413 0.541284 3.16514 -7.85714 -25.4898 -13.1651 0.652778 6
0.0363 0.0334 0.00968 0.00843 0.000594 0.0205 0.006 0.0136 0.00184
2L:19161720-19161870:plus -24.0306 1.46789 -3.50459 4.05505 -18.7143 -27.7041 -12.9083 3.125 2.46789
0.00723 0.0061 0.0332 0.00566 0.0136 0.0445 0.00566 0.00569 0.00805
2L:19163480-19163630:minus -5.04082 1.78899 4.22018 4.61468 -27.602 -18.3469 -13.5963 6.41667
8.11009 0.000223 0.00542 0.00245 0.00475 0.0364 0.0124 0.00663 0.00135 0.000597
2L:19163480-19163630:plus -23.1837 -1.94495 0.981651 6.20183 -17.6429 -8.93878 -22.3578 1.63889 -2.94495
0.00553 0.0191 0.00832 0.00214 0.00562 0.00201 0.0314 0.00978 0.0441
2L:19166120-19166270:minus -40.449 -1.62385 3.06422 4.6422 -18.7857 0.0510204 -30.9908 1.80556
-4.55963 0.0402 0.0174 0.00387 0.00473 0.0148 0.000349 0.104 0.00923 0.0648
2L:19166120-19166270:plus -23.7347 1.27523 4.34862 2.52294 -18.2245 -19.6429 -15.4128 3.97222 9.34862
0.00673 0.00655 0.00233 0.0113 0.00877 0.0204 0.00973 0.00407 0.000371
2L:19181640-19181790:minus -32.0408 0.587156 2.56881 1.69725 20.2041 -27.8265 -18.8349 0.791667
3.46789 0.0174 0.0084 0.00468 0.0163 3.64e-07 0.0498 0.017 0.013 0.00582
2L:19181640-19181790:plus -31.5918 1.73394 2.77064 2.44037 -17.6429 -17.7857 -20.6422 -2.38889 -3.99083
0.0136 0.00553 0.00433 0.0116 0.00562 0.0068 0.0231 0.0331 0.0556
2L:19185920-19186070:minus -23.449 13.4592 3.70642 7 -27.9694 -9.79592 -23.4037 5.09722 -10.3578
0.00631 0.000064 0.00302 0.00132 0.0437 0.00377 0.0375 0.00251 0.207
2L:19185920-19186070:plus -30.7041 1.21101 2.59633 2.25688 1.14286 0.316327 -12.3853 2.375 7.37615
0.00993 0.00671 0.00463 0.0127 0.000113 0.000243 0.00503 0.00753 0.00107
2L:1928100-1928250:minus -31.3673 2.13761 -2.56881 2.9633 -18.3776 10.3776 -21.6881 1.77778 -3.55963

0.0116 0.00477 0.0255 0.00914 0.00926 6.32e-06 0.0279 0.00932 0.0503
2L:1928100-1928250:plus -23.3265 -0.12844 0.247706 -1.3211 -27.2347 -17.2755 -8.34862 2.75 5.70642
0.00607 0.0108 0.0107 0.0407 0.0283 0.00544 0.00226 0.00656 0.00222
2L:19361500-19361650:minus -22.898 -2.7156 2.93578 7.47706 -18.7143 -9.60204 -12.6881 -2.56944
1.68807 0.00442 0.0238 0.00407 0.00085 0.0136 0.00334 0.00538 0.0346 0.0109
2L:19361500-19361650:plus -24.2959 6.06422 7.34862 2.77064 -26.3061 -27.9694 -14.9817 4.01389 4.10092
0.00781 0.00103 0.000611 0.01 0.0181 0.0547 0.00894 0.004 0.00413
2L:19362000-19362150:minus -31.6735 -2.51376 13.7523 7.29358 -26.4898 -18.051 -12.7064 -0.666667
4.3945 0.0141 0.0225 9.94e-06 0.00104 0.0183 0.00886 0.00541 0.0205 0.00375
2L:19362000-19362150:plus -33.0408 -0.256881 6.95413 5.86239 -17.9388 -8.53061 -25.2752 3.98611
4.70642 0.0264 0.0113 0.000743 0.00282 0.00715 0.00136 0.0489 0.00404 0.00346
2L:19362860-19363010:minus -39.9898 -4.04587 2.6055 0.477064 -18.2245 -17.8265 -5.78899 7.02778
4.7156 0.0351 0.0342 0.00462 0.0238 0.00877 0.00699 0.00124 0.000991 0.00345
2L:19362860-19363010:plus -21.8878 0.577982 -3.00917 3.08257 -18.7551 -0.204082 -16.4587 4.15278
4.07339 0.00258 0.00843 0.0289 0.0087 0.0144 0.000463 0.0117 0.00377 0.00417
2L:19363120-19363270:minus -32.7755 -2.21101 9.89908 3.90826 -18.2245 -19.1224 -11.9541 6.41667
-0.220183 0.0234 0.0206 0.000151 0.00603 0.00877 0.0175 0.00456 0.00135 0.0201
2L:19363120-19363270:plus -32.1429 -2.78899 10.8716 3.89908 -18.449 -0.244898 1.36697 0.5 -0.798165
0.0179 0.0243 0.0000865 0.00604 0.0107 0.000496 0.000199 0.0143 0.0246
2L:1938180-1938330:minus -23.2551 6.48624 7.94495 3.18349 -17.449 -0.244898 -14.4404 0.291667
-0.59633 0.00583 0.00084 0.000451 0.00837 0.00484 0.000496 0.00799 0.0153 0.0229
2L:1938180-1938330:plus -32.4082 1.83486 2.82569 6.62385 -17.0408 -19.3061 -18.8349 0.208333 2.57798
0.0191 0.00533 0.00424 0.00163 0.00329 0.0187 0.017 0.0157 0.0078
2L:19397000-19397150:minus -24.7857 2.55963 -0.669725 -0.0458716 -26.9694 -26.898 -24.7339
2.90278 1.90826 0.00829 0.00409 0.0144 0.0271 0.023 0.0302 0.0455 0.00619 0.00974
2L:19397000-19397150:plus -23.5204 2.73394 13.3303 0.779817 -26.9694 -18.051 -11.8991 0.791667 -0.990826
0.00645 0.00383 0.000015 0.0219 0.023 0.00886 0.00451 0.013 0.026
2L:19398400-19398550:minus -30.6327 -0.963303 8.94495 10.5963 -18.3061 -7.93878 -23.4587 -1.33333
-0.954128 0.00968 0.0142 0.000262 0.0001 0.00884 0.000736 0.0378 0.0248 0.0258
2L:19398400-19398550:plus -31.7755 1.21101 -0.724771 1.16514 -8.93878 -18.7143 -3.84404 0.930556
9.34862 0.0157 0.00671 0.0146 0.0197 0.00216 0.014 0.000714 0.0124 0.000371
2L:19414080-19414230:minus -23.6327 2.0367 13.6422 7 -17.3367 -18.3469 -20.9083 -3.56944 1.6422
0.00661 0.00495 0.0000116 0.00132 0.00391 0.0124 0.0242 0.0445 0.0111
2L:19414080-19414230:plus -12.8163 -0.266055 8.74312 0.0733945 -27.1224 -9.42857 -16.0275 6.875
11.3853 0.000489 0.0113 0.000291 0.0263 0.0245 0.0027 0.0109 0.00107 0.0000815
2L:19414460-19414610:minus -13.2653 3.95413 7.04587 10.8624 -27.1224 0.0204082 -12.0183 -2.02778
5.59633 0.000759 0.00244 0.000711 0.0000687 0.0245 0.000377 0.00463 0.03 0.00234
2L:19414460-19414610:plus -21.4388 -0.394495 3.51376 5.48624 -27.0816 0.826531 -4.69725 -1.15278
-2.12844 0.002 0.0118 0.00325 0.00357 0.0243 0.000153 0.00091 0.0236 0.0345
2L:19423480-19423630:minus -12.1122 5.19266 -0.155963 11.2936 -18.4184 -9.53061 13.6972 -2.73611
5.12844 0.000336 0.0015 0.0122 0.0000139 0.0101 0.0031 2.47e-06 0.0362 0.00301
2L:19423480-19423630:plus -14.2245 -0.87156 9.59633 4.07339 -18.4184 -35.0408 -0.412844 -5.04167
11.8073 0.00126 0.0138 0.00018 0.00562 0.0101 0.0742 0.000311 0.0622 0.0000538
2L:19424020-19424170:minus -33 -4.66055 -1.89908 -1.99083 -18.6429 -16.7551 -0.311927 1.70833
1.49541 0.0258 0.0403 0.021 0.0512 0.0121 0.0042 0.000304 0.00955 0.0118
2L:19424020-19424170:plus -21.4082 1.52294 0.247706 5.57798 -26.7449 -26.8265 -9.79817 -2.31944 6.73394
0.00198 0.00598 0.0107 0.0034 0.0195 0.0285 0.00296 0.0325 0.00143
2L:19425120-19425270:minus -31 0.59633 3.30275 7.72477 -17.8878 -17.7857 -21.3761 4.5 -0.513761
0.0105 0.00837 0.00353 0.000681 0.00704 0.0068 0.0263 0.00326 0.0221
2L:19425120-19425270:plus -32.9592 -1 4.6055 5.88073 -18.6837 -27.8265 -11.4862 -1.09722 -3.30275
0.0252 0.0143 0.0021 0.00275 0.0126 0.0498 0.00413 0.0232 0.0475
2L:19426540-19426690:minus -30.3673 4.82569 4.26606 10.8624 -26.6327 -26.4898 -6.22018 2.25 1.95413
0.00897 0.00175 0.00241 0.0000687 0.0188 0.0225 0.00139 0.00788 0.00964
2L:19426540-19426690:plus -14.449 16.4954 14.8899 7.21101 -18.1122 -18.1224 -14.7156 -2.11111 12.2752

0.0014 7.12e-08 2.06e-06 0.0011 0.00771 0.00988 0.00847 0.0307 0.0000141
2L:19430460-19430610:minus -30.2551 3.83673 4.57798 3.14679 -17.6735 -27.5612 -9 2.88889 1.01835
0.00885 0.00115 0.00212 0.00847 0.00572 0.0393 0.00256 0.00622 0.0142
2L:19430460-19430610:plus -21.9592 5.65138 3.97248 2.93578 -18.3469 -18.1224 -13.8624 8.25 -2.40367
0.00279 0.00124 0.00271 0.00927 0.00894 0.00988 0.00704 0.000505 0.0376
2L:19432460-19432610:minus -23.0306 1.11009 6.34862 3.88991 -9.45918 -26.5612 -7.48624 0.861111
2.06422 0.00505 0.00695 0.000987 0.00608 0.00287 0.0237 0.00189 0.0127 0.00912
2L:19432460-19432610:plus -22.3776 -1.40367 12.7706 7.2844 -18.1224 -18.3469 -5.37615 -2.375 0.59633
0.00342 0.0163 0.0000251 0.00105 0.00782 0.0124 0.0011 0.0329 0.016
2L:19444800-19444950:minus -30.4796 1.68807 10.5321 3.90826 -18.4184 -18.0102 -22.1009 1.22222
-2.93578 0.00941 0.00563 0.000103 0.00603 0.0101 0.00812 0.03 0.0113 0.0439
2L:19444800-19444950:plus -32.9592 -2.09174 -0.743119 3.79817 -17.3776 -8.68367 -17.4862 0.638889
-7.93578 0.0252 0.0199 0.0147 0.00629 0.00419 0.00157 0.0138 0.0137 0.134
2L:19453480-19453630:minus -30.6735 10.4771 5.44037 7.34862 -27.2245 -9.57143 -14.7523 1.29167
-4.63303 0.00978 0.000105 0.00148 0.000998 0.0272 0.00326 0.00853 0.011 0.0661
2L:19453480-19453630:plus -29.4796 -1.29358 2.69725 5.82569 -8.37755 -17.7143 -23.1468 -4.40278 3.51376
0.00844 0.0157 0.00446 0.00291 0.00106 0.00634 0.0359 0.054 0.00566
2L:19490120-19490270:minus -24.3673 13.5046 12.6789 7.29358 -18.0816 -18.7143 -18.1835 4.58333
1.70642 0.00791 0.0000142 0.0000266 0.00104 0.00759 0.014 0.0154 0.00315 0.0108
2L:19490120-19490270:plus -32.8469 0.0458716 0.825688 0.788991 -27.2347 -18.2755 -13.5413 -0.444444
0.513761 0.0243 0.0101 0.00878 0.0218 0.0283 0.0112 0.00654 0.0192 0.0162
2L:19493160-19493310:minus -22 13.5413 1.77064 3.95413 -18.7551 -19.4184 -15.0275 1.68056 -0.697248
0.00291 0.0000137 0.00629 0.00593 0.0144 0.0198 0.00902 0.00964 0.0239
2L:19493160-19493310:plus -32.8061 15.0642 -3.55046 3.23853 -27.5306 -18.0816 -20.422 4.08333 1.48624
0.0237 1.87e-06 0.0336 0.00815 0.0351 0.00934 0.0222 0.00388 0.0118
2L:19498100-19498250:minus -23.449 0.688073 1 2.11009 -18.3776 -0.540816 -25.1193 7.84722
-6.56881 0.00631 0.0081 0.00827 0.0136 0.00926 0.00054 0.0479 0.000636 0.103
2L:19498100-19498250:plus -31.7755 10.3945 2.87156 2.6789 -18.9388 -9.53061 -14.6147 5.08333 -0.220183
0.0157 0.000109 0.00417 0.0105 0.0151 0.0031 0.00829 0.00253 0.0201
2L:19508320-19508470:minus -31.7755 5.13761 1.66055 3.24771 -18.7857 -25.898 -12.4128 6.43056 3
0.0157 0.00154 0.00654 0.00808 0.0148 0.0214 0.00506 0.00134 0.00702
2L:19508320-19508470:plus -22.2653 3.83673 9.15596 5.47706 -27.4082 -19.0102 -13.0367 1.63889 4.77064
0.00331 0.00115 0.000232 0.0036 0.0321 0.0159 0.00583 0.00978 0.0034
2L:19508700-19508850:minus -14.6327 11.2936 6.55046 2.44954 20.2041 -18.0816 -6.80734 4.40278
-1.26606 0.0015 0.0000697 0.000896 0.0116 3.64e-07 0.00934 0.00161 0.0034 0.0281
2L:19508700-19508850:plus -30.398 3.66055 -0.93578 2 -16.449 -18.6327 -9.48624 2.38889 3.88991
0.00901 0.00273 0.0156 0.0143 0.00307 0.0137 0.0028 0.00749 0.00467
2L:19526460-19526610:minus -31.8061 0.550459 12.9083 3.42202 -18.7143 0.826531 -22.9908 0.930556
-0.908257 0.0158 0.00851 0.0000224 0.00751 0.0136 0.000153 0.035 0.0124 0.0255
2L:19526460-19526610:plus -21.4388 -4.74312 1.3211 2.88991 10.9184 -18.3061 -6.23853 4.11111 1.73394
0.002 0.0412 0.00738 0.00942 4.19e-06 0.0114 0.0014 0.00384 0.0106
2L:19528500-19528650:minus -40.8163 3.68367 2.2844 8.81651 -19.051 -18.3469 -20.4771 10.375
-0.59633 0.0436 0.00144 0.0052 0.000501 0.0162 0.0124 0.0224 0.000124 0.0229
2L:19528500-19528650:plus -22.7755 2.38532 -2.02752 2.19266 -17.7551 -26.5306 -12.0275 3.98611 8.20183
0.00412 0.00436 0.0218 0.0131 0.00658 0.023 0.00464 0.00404 0.000539
2L:19530280-19530430:minus -23.5612 1.36697 14.6881 3.23853 -9.19388 -18.3469 -5.57798 -0.888889
-0.550459 0.00653 0.00633 3.06e-06 0.00815 0.00253 0.0124 0.00117 0.0218 0.0225
2L:19530280-19530430:plus -22 0.522936 14.4771 1.16514 -9.23469 -7.5 -13.0917 0.888889 -0.174312
0.00291 0.00859 3.9e-06 0.0197 0.00277 0.000614 0.0059 0.0126 0.0196
2L:19533320-19533470:minus -14.7449 -1.3211 6.51376 7.47706 -17.449 -9.34694 -23.422 5.09722
-0.550459 0.00157 0.0159 0.000912 0.00085 0.00484 0.00261 0.0376 0.00251 0.0225
2L:19533320-19533470:plus -31.5102 -1.82569 4.47706 5.57798 -18.3878 -9.23469 -4.65138 0.902778 2.55046
0.013 0.0185 0.00221 0.0034 0.00931 0.00238 0.000899 0.0125 0.00785
2L:19537300-19537450:minus -4.7449 3.64286 4.52294 6.3578 -8.23469 -9.23469 -21.9358 4.18056

0.53211 0.000191 0.00159 0.00217 0.00195 0.000927 0.00238 0.0291 0.00373 0.0161
2L:19537300-19537450:plus -34.3776 -4.79817 9.86239 6.02752 -28.1939 -8.86735 -22.3394 4.15278 1.78899
0.0319 0.0418 0.000155 0.00246 0.0482 0.00198 0.0313 0.00377 0.0103
2L:19542380-19542530:minus -33.0714 13.789 1.91743 9.3211 -26.2245 1.09184 -10.8899 4 -4.83486
0.0267 0.0000107 0.00596 0.000391 0.0175 0.000091 0.00365 0.00402 0.0699
2L:19542380-19542530:plus -33.5102 -0.0917431 5.69725 3.45872 -26.7449 -18.1224 -16.0183 -2.625
1.11009 0.0286 0.0106 0.00132 0.00731 0.0195 0.00988 0.0109 0.0351 0.0138
2L:19543140-19543290:minus -28.8776 -1.42202 0.266055 4.15596 -9.27551 -17.9796 -7.49541 3.27778
-1.85321 0.00832 0.0164 0.0106 0.00539 0.00284 0.00764 0.00189 0.00536 0.032
2L:19543140-19543290:plus -31.6633 0.412844 5.11009 -0.201835 -9.23469 -9.57143 -14.156 -1.34722
0.917431 0.0141 0.00893 0.0017 0.0285 0.00277 0.00326 0.00751 0.0249 0.0146
2L:19546840-19546990:minus -22 -1.74312 6.70642 4.21101 -18.3776 -17.2755 -15.0642 2.25 -3.44037
0.00291 0.018 0.000832 0.00532 0.00926 0.00544 0.00909 0.00788 0.049
2L:19546840-19546990:plus -21.6327 -1.27523 9.20183 3.9633 -26.9694 -18.1224 -8.59633 1.76389 2.47706
0.00215 0.0156 0.000225 0.0059 0.023 0.00988 0.00237 0.00937 0.00801
2L:19547380-19547530:minus -31.9286 8.52294 -0.449541 5.22018 -18.6429 -18.051 -21.4037 2.33333
-3.41284 0.0166 0.000288 0.0134 0.00418 0.0121 0.00886 0.0265 0.00765 0.0487
2L:19547380-19547530:plus -31.8469 0.954128 1.66972 -0.541284 -19.0204 -19.1224 -15.6422 2.91667
5.6055 0.0162 0.00736 0.00652 0.0316 0.0158 0.0175 0.0102 0.00616 0.00232
2L:19547740-19547890:minus -32.5102 4.06422 0.229358 1.9633 -18.9388 -0.244898 -25.156 0.972222
-5.22018 0.0202 0.00235 0.0107 0.0146 0.0151 0.000496 0.0481 0.0123 0.0762
2L:19547740-19547890:plus -24.7449 1.3945 3.90826 6.29358 -27.4592 -9.57143 -22.3486 3.55556 -6.44954
0.00827 0.00627 0.00278 0.00202 0.0329 0.00326 0.0314 0.00481 0.0993
2L:19548340-19548490:minus -3.22449 -0.183486 5.63303 6.47706 -27.1224 -9.65306 6.18349 6.875
0.275229 0.0000495 0.011 0.00136 0.00179 0.0245 0.0034 0.0000392 0.00107 0.0171
2L:19548340-19548490:plus -31.7041 10.4587 8.34862 10.5872 0.877551 0.0204082 -16.789 7.375
9.14679 0.0146 0.000106 0.000363 0.000115 0.00019 0.000377 0.0124 0.000825 0.000414
2L:19549540-19549690:minus -32.4694 6.53211 -1.88991 7.27523 -9.0102 -18.051 -24.211 6.38889
-1.43119 0.0196 0.000822 0.0209 0.00109 0.00236 0.00886 0.0423 0.00137 0.029
2L:19549540-19549690:plus -22.8878 1.58716 0.642202 11.1927 -7.60204 -8.23469 -3.22018 1.97222 -0.174312
0.00441 0.00584 0.00935 0.0000254 0.000466 0.000875 0.000602 0.0087 0.0196
2L:19570420-19570570:minus -34.0408 3.83673 0.366972 5.73394 -18.9796 -8.42857 -15.6606 2.77778
3.3211 0.0309 0.00115 0.0103 0.00307 0.0155 0.00104 0.0102 0.00649 0.00616
2L:19570420-19570570:plus -22.9592 2.22936 -0.0642202 1.99083 -18.5204 -26.8265 -10.2936 -0.75
9.55046 0.00475 0.00461 0.0118 0.0144 0.0117 0.0285 0.00325 0.021 0.000321
2L:19571180-19571330:minus -30.9184 1.38532 6.20183 3.62385 -18.9796 -19.0816 -13.1009 3.18056
11.4771 0.0103 0.00629 0.00106 0.00673 0.0155 0.017 0.00592 0.00557 0.0000679
2L:19571180-19571330:plus -23.1939 1.52294 2.83486 1.09174 -26.2347 -27.3776 -6.33945 11.0139 1.88073
0.00558 0.00598 0.00423 0.02 0.0177 0.0351 0.00143 0.0000754 0.00987
2L:19571940-19572090:minus -22.0714 2.0367 4.34862 1.91743 -27.2347 1.05102 -12.6697 9.5 11.3853
0.00299 0.00495 0.00233 0.015 0.0283 0.000107 0.00536 0.000231 0.0000815
2L:19571940-19572090:plus -32.7449 2.22018 1.55963 -0.743119 -26 -16.3469 -6.76147 2.73611 6.44954
0.0228 0.00463 0.00678 0.0339 0.0171 0.00416 0.00159 0.00659 0.00154
2L:19572620-19572770:minus -29.6327 1.62385 -1.15596 6.3578 -27.9388 -7.72449 -8.11009 0.0833333
-0.706422 0.00846 0.00577 0.0168 0.00195 0.0415 0.00068 0.00215 0.0163 0.024
2L:19572620-19572770:plus -31.8163 -2.66055 -1.7156 1.22936 -18.5306 -17.3469 -19.5138 4.30556
-0.0825688 0.016 0.0235 0.0198 0.0193 0.0118 0.0058 0.019 0.00354 0.0189
2L:19575320-19575470:minus -3.4898 -1.88991 3.61468 3.97248 -8.44898 -27.9694 6.78899 5.77778
8.95413 0.0000735 0.0188 0.00313 0.00587 0.00109 0.0547 0.0000329 0.00184 0.000458
2L:19575320-19575470:plus -23.0306 -2.68807 3.33945 2.72477 -26.9694 -7.94898 -10.5321 1.38889 5.70642
0.00505 0.0236 0.00348 0.0102 0.023 0.00074 0.0034 0.0107 0.00222
2L:19576080-19576230:minus -22.7755 1.84404 6.46789 7.17431 -17.0408 -26.2653 -5.38532 1.73611
7.37615 0.00412 0.00532 0.000933 0.00115 0.00329 0.0218 0.00111 0.00946 0.00107
2L:19576080-19576230:plus -31.6633 0.669725 -1.85321 2.78899 -8.67347 -17.051 -13.7156 5.15278 7.6422

0.0141 0.00815 0.0207 0.00992 0.00154 0.00479 0.00681 0.00245 0.000893
2L:19576520-19576670:minus -22.6327 0.642202 0.706422 5.59633 -26.2653 -26.5306 -17.9266 -0.638889
-0.229358 0.00373 0.00823 0.00914 0.00331 0.018 0.023 0.0148 0.0203 0.0202
2L:19576520-19576670:plus -23.4184 0.321101 1.88073 7.73394 -17.6735 0.387755 -14.8349 -0.541667
0.908257 0.00624 0.00922 0.00604 0.000669 0.00572 0.000217 0.00868 0.0197 0.0147
2L:19577280-19577430:minus -13.0714 4.41284 5.74312 9.59633 -7.93878 -26.7551 -15.2385 3.77778
-0.119266 0.000594 0.00205 0.00129 0.000275 0.000767 0.0263 0.00941 0.0044 0.0192
2L:19577280-19577430:plus -23.4898 3.97959 -0.46789 2.98165 -19.0204 -9.5 -21 2.76389 3.66972 0.00639
0.000669 0.0135 0.00905 0.0158 0.00294 0.0246 0.00652 0.00514
2L:19580320-19580470:minus -23.2245 12.6514 9.55963 3.69725 -18.0816 -27.3061 -9.00917 3.55556
0.238532 0.00576 0.000029 0.000184 0.00665 0.00759 0.0336 0.00256 0.00481 0.0173
2L:19580320-19580470:plus -21.8061 11.7156 -1.92661 6.41284 -18.2245 -17.7143 -7.72477 -4.22222 7.45872
0.00241 0.0000546 0.0212 0.00189 0.00877 0.00634 0.00199 0.0518 0.00103
2L:19582400-19582550:minus -39.6531 1.20183 12.9083 2.37615 10.4286 -27.449 -1.47706 1.5 -0.59633
0.0336 0.00673 0.0000224 0.0119 0.0000139 0.0357 0.000394 0.0103 0.0229
2L:19582400-19582550:plus -31.8469 1.89908 0.293578 9.3211 -8.37755 -0.204082 -20.0275 4.69444
-2.92661 0.0162 0.00521 0.0105 0.000391 0.00106 0.000463 0.0207 0.003 0.0437
2L:19584000-19584150:minus -23.3776 5.3945 14.4404 6.49541 -18.1531 -7.65306 -11.1284 2.93056
6.02752 0.00619 0.00138 4.12e-06 0.00176 0.00829 0.000647 0.00383 0.00612 0.00183
2L:19584000-19584150:plus -30.4388 -1.46789 1.25688 5.85321 -8.67347 -8.7551 -12.0917 -1.80556 -2.6055
0.00923 0.0166 0.00755 0.00287 0.00154 0.00172 0.00471 0.0283 0.04
2L:19587240-19587390:minus -32.8469 -0.201835 13.2385 2.53211 -17.7143 -17.2755 -20.9817 2.27778
-0.59633 0.0243 0.011 0.000016 0.0113 0.00627 0.00544 0.0245 0.0078 0.0229
2L:19587240-19587390:plus -14.2959 3.75229 0.211009 9.44954 -26.9694 -8.72449 1.63303 -4.16667 3.88073
0.00129 0.00264 0.0108 0.000326 0.023 0.00169 0.000184 0.0512 0.00475
2L:19700-19850:minus -40.5918 -0.66055 -3.25688 4.78899 -27.2347 10.5306 -12.1927 3.63889 -4.65138
0.0422 0.0129 0.031 0.00456 0.0283 5.67e-06 0.00481 0.00465 0.0664
2L:19700-19850:plus -23.5612 -0.0825688 4.14679 4.15596 -17.6837 -9.42857 -23.211 0.0138889
-5.77064 0.00653 0.0106 0.00252 0.00539 0.0058 0.0027 0.0363 0.0167 0.0846
2L:19735860-19736010:minus -32.8163 6.74312 4.9633 3.30275 -18.7551 -8.23469 -4.84404 7.29167
2.51376 0.024 0.00074 0.00181 0.00789 0.0144 0.000875 0.00095 0.000863 0.00792
2L:19735860-19736010:plus -22.6633 13.5306 0.0458716 6.44954 -7.60204 -19.1224 4.30275 0.666667
1.48624 0.00384 0.0000206 0.0114 0.00183 0.000466 0.0175 0.0000739 0.0135 0.0118
2L:1974220-1974370:minus -3.18367 2.42202 -0.201835 5.93578 -17.1531 -36.8878 -12.0367 -5.18056
7.46789 0.0000481 0.0043 0.0124 0.00264 0.00364 0.123 0.00465 0.0641 0.00102
2L:1974220-1974370:plus -22.7755 3.00917 1.08257 4.48624 -27.1224 -17.7143 -4.11927 -5.16667 -3.11009
0.00412 0.00347 0.00803 0.00487 0.0245 0.00634 0.000772 0.0639 0.0459
2L:19756900-19757050:minus -21.3673 0.238532 5.09174 4.0367 0.908163 -17.9796 -15.0917 -4.44444
-0.0275229 0.00195 0.0095 0.00171 0.00578 0.000153 0.00764 0.00914 0.0545 0.0186
2L:19756900-19757050:plus -24.3367 6.30275 1.2844 6.12844 -26.4898 -17.7551 -10.3853 -3.68056 -0.33945
0.00789 0.000917 0.00748 0.00231 0.0183 0.00643 0.00331 0.0457 0.0209
2L:19758620-19758770:minus -23.1837 6.11009 0.972477 9.24771 10.3878 -17.0102 -15.2385 5.15278
-4.04587 0.00553 0.00101 0.00835 0.000409 0.0000174 0.00453 0.00941 0.00245 0.0563
2L:19758620-19758770:plus 5.18367 3.64286 -2.0367 2.12844 -27.5612 -9.23469 -12.8716 1.18056 0.357798
0.0000136 0.00159 0.0219 0.0134 0.0354 0.00238 0.00561 0.0114 0.0166
2L:19764060-19764210:minus -3 -0.46789 5.22936 7.72477 -18.6429 -7.72449 -17.8532 -0.513889
1.58716 0.000041 0.0121 0.00162 0.000681 0.0121 0.00068 0.0146 0.0196 0.0113
2L:19764060-19764210:plus -22.1531 1.68807 6.11009 10.8624 -16.8878 -17.898 -7.56881 3.73611 3.85321
0.0031 0.00563 0.0011 0.0000687 0.00326 0.00726 0.00192 0.00447 0.00479
2L:19791200-19791350:minus -32.7653 -2.05505 3.48624 3.41284 0.94898 -16.7143 -16.4495 0.861111
-3.13761 0.0228 0.0197 0.00329 0.00756 0.000134 0.00419 0.0117 0.0127 0.0461
2L:19791200-19791350:plus -32.1837 -2.15596 10.1468 6.19266 -18.7143 -17.2041 -10.578 -3.47222 11.4771
0.0181 0.0203 0.000131 0.00216 0.0136 0.00507 0.00343 0.0434 0.0000679
2L:19791600-19791750:minus -21.6327 -2.19266 -0.816514 0.990826 -36.449 -25.5306 -9.30275 -2.90278

-0.275229 0.00215 0.0205 0.0151 0.0206 0.0783 0.0205 0.00271 0.0377 0.0206
2L:19791600-19791750:plus -31.2959 0.614679 2.18349 3.78899 -17.1837 -18.2755 -13.2294 -1.31944 2
0.0113 0.00832 0.0054 0.00632 0.00366 0.0112 0.00609 0.0247 0.00941
2L:19791820-19791970:minus -14.5204 5.13761 4.73394 4.94495 -8.16327 0.27551 -8.78899 7.34722
0.752294 0.00145 0.00154 0.00199 0.00444 0.000873 0.000257 0.00246 0.000838 0.0152
2L:19791820-19791970:plus -31.8061 1.80734 1.46789 5.86239 -8.67347 0.27551 -5.78899 2.98611
-0.0183486 0.0158 0.00539 0.007 0.00282 0.00154 0.000257 0.00124 0.006 0.0185
2L:1980540-1980690:minus -23.4796 -1.75229 -1.59633 0.0550459 -18.1531 -17.5612 -14.6881 -0.402778
2.69725 0.00634 0.0181 0.0192 0.0265 0.00829 0.00604 0.00842 0.0189 0.0076
2L:1980540-1980690:plus -23.0408 6.91743 2 2.88991 20.4694 -36.8163 -12.3119 1.16667 3.63303
0.00512 0.000678 0.00578 0.00942 1.65e-07 0.12 0.00494 0.0115 0.00518
2L:1981020-1981170:minus -23.5612 3.85321 1.75229 0.46789 -17.4184 -19.1224 -19.0275 4.66667 -0.899083
0.00653 0.00254 0.00633 0.0239 0.00457 0.0175 0.0175 0.00304 0.0254
2L:1981020-1981170:plus -31.4796 13.3878 -3.52294 0.00917431 -18.4898 -8.57143 -7.66055
9.66667 -0.119266 0.0128 0.0000869 0.0334 0.0269 0.0116 0.00149 0.00196 0.000207 0.0192
2L:1982100-1982250:minus -31.7041 4.05102 -2.38532 7.3578 -18.7551 -9.27551 -16.8624
6.52778 1.42202 0.0146 0.000335 0.0242 0.000965 0.0144 0.00244 0.0125 0.00128 0.0121
2L:1982100-1982250:plus -22.6939 13.5306 5.40367 7.21101 -18.7143 -17.2755 -10.7798 3.625
2.49541 0.00388 0.0000206 0.0015 0.0011 0.0136 0.00544 0.00357 0.00468 0.00794
2L:19865760-19865910:minus -30.6735 2.37615 2.54128 7.27523 -8.93878 -7.72449 -10.5046
0.763889 2.20183 0.00978 0.00437 0.00473 0.00109 0.00216 0.00068 0.00339 0.0131 0.00869
2L:19865760-19865910:plus -21.9184 7.19266 9.70642 10.7615 -26.9694 -18.0102 -14.8807
-8.90278 -3 0.00261 0.000587 0.000169 0.0000826 0.023 0.00812 0.00876 0.129 0.0447
2L:1986660-1986810:minus -29.449 5.85321 4.62385 5.2844 -27.3061 -7.93878 -11.633 1.125 1.95413
0.00842 0.00113 0.00208 0.004 0.0315 0.000736 0.00426 0.0117 0.00964
2L:1986660-1986810:plus -41.7755 1.31193 9.75229 2.83486 -17.9388 -8.65306 -34.7064 1.09722
-2.93578 0.0652 0.00646 0.000164 0.0097 0.00715 0.00156 0.156 0.0118 0.0439
2L:1987720-1987870:minus -24.4796 -1.68807 0.798165 6.41284 -27.2347 -0.244898
-17.7982 -1.94444 -2.27523 0.00804 0.0177 0.00886 0.00189 0.0283 0.000496 0.0145 0.0294 0.036
2L:1987720-1987870:plus -22.2551 8.24771 1.93578 3.88991 -26.898 0.122449 5.73394 7.54167 4.6789
0.00327 0.000333 0.00592 0.00608 0.0203 0.000312 0.0000451 0.000754 0.00347
2L:1988180-1988330:minus -31.6633 -1.16514 -1.0367 -0.137615 -27.7551 -8.86735
-25.1284 3.70833 2.89908 0.0141 0.0151 0.0161 0.0279 0.0385 0.00198 0.048 0.00452 0.00722
2L:1988180-1988330:plus -15.0102 0.0183486 1.83486 3.11009 -8.96939 -0.316327 -12.7523
2.63889 7.63303 0.00162 0.0102 0.00614 0.00862 0.00226 0.000524 0.00546 0.00684 0.000928
2L:1989560-1989710:minus -13.3367 5.42202 4.55963 -0.688073 -18.7143 -18.3061 -18.9266
0.902778 8.10092 0.0008 0.00137 0.00214 0.0333 0.0136 0.0114 0.0172 0.0125 0.000623
2L:1989560-1989710:plus -21.8163 6.9633 10.2936 7.6055 -18.4184 0.316327 -5.95413 0.388889
-0.605505 0.00244 0.000662 0.00012 0.000794 0.0101 0.000243 0.00129 0.0148 0.023
2L:19935160-19935310:minus -23.5204 3.64286 -0.559633 7.61468 -26.9694 10.5714 -20.7982
4.83333 1.57798 0.00645 0.00159 0.0139 0.000779 0.023 4.67e-06 0.0237 0.00283 0.0114
2L:19935160-19935310:plus -11.7755 1.30275 7.59633 9.61468 -17.1531 -27.8265 10.0275 0.388889
5.12844 0.000266 0.00649 0.000541 0.000255 0.00364 0.0498 0.0000128 0.0148 0.00301
2L:20060940-20061090:minus -40.1837 1.6422 -0.495413 3.33028 -19.051 -27.2653 -3.68807
2.18056 3.66972 0.0367 0.00573 0.0136 0.00778 0.0162 0.0333 0.000683 0.00808 0.00514
2L:20060940-20061090:plus -31.6633 4.22018 2.51376 2.41284 -27.4898 -19.3061 -24.6422
7.84722 -0.440367 0.0141 0.00221 0.00478 0.0118 0.0336 0.0187 0.045 0.000636 0.0216
2L:20061420-20061570:minus -32 4.72477 0.284404 4.43119 -26.7041 -9.57143 -19.1009
1.15278 -1.11927 0.0171 0.00182 0.0105 0.00495 0.0193 0.00326 0.0177 0.0115 0.027
2L:20061420-20061570:plus -30.102 -0.165138 2.31193 6.22936 -8.89796 0.316327 -9.77982
-1.73611 3.88073 0.00859 0.0109 0.00515 0.00209 0.00189 0.000243 0.00295 0.0277 0.00475
2L:20065680-20065830:minus -23.2245 13.6514 -5.48624 4.0367 -17.8571 -8.45918 -13.1468
3.52778 -3.79817 0.00576 0.0000126 0.0558 0.00578 0.00698 0.00111 0.00598 0.00486 0.0527
2L:20065680-20065830:plus -22.7041 14.4954 0.53211 3.57798 -8.64286 -8.45918 -14.9725

0.958333 1.30275 0.00395 5.09e-06 0.00971 0.00696 0.00139 0.00111 0.00892 0.0123 0.0128
2L:20066340-20066490:minus -23.2959 0.513761 1.72477 7.47706 -17.7143 -18.3469 -20.7615
2.76389 1.69725 0.00605 0.00862 0.0064 0.00085 0.00627 0.0124 0.0236 0.00652 0.0108
2L:20066340-20066490:plus -13.602 -0.238532 9.72477 2.83486 10.9184 9.60204 -13.1101 0.555556
1.69725 0.000995 0.0112 0.000167 0.0097 4.19e-06 0.0000248 0.00593 0.014 0.0108
2L:20075400-20075550:minus -14.2245 4.30275 13.0092 6.31193 -28.2653 -8.79592 -4.06422
-1.34722 4.77982 0.00126 0.00214 0.0000207 0.00199 0.0537 0.0019 0.00076 0.0249 0.00339
2L:20075400-20075550:plus -21.8878 11.0367 0.0917431 4.20183 -8.89796 -18.2755 -5.61468
1.02778 -2.47706 0.00258 0.0000797 0.0112 0.00533 0.00189 0.0112 0.00118 0.012 0.0385
2L:20085020-20085170:minus -22.1939 4.44954 -2.19266 9.59633 -18.449 -9.53061 -12.6972
6.77778 6.17431 0.00317 0.00202 0.0229 0.000275 0.0107 0.0031 0.00539 0.00113 0.00171
2L:20085020-20085170:plus -32.3673 -2.68807 -1.21101 3.09174 -25.6327 -8.7551 -28.945 2.5
-0.743119 0.0189 0.0236 0.017 0.00867 0.0165 0.00172 0.0783 0.0072 0.0243
2L:20091160-20091310:minus -33 3.94495 2.52294 0.889908 -18.7857 -8.5 -13.9817 1.90278
-0.761468 0.0258 0.00245 0.00476 0.0213 0.0148 0.00126 0.00722 0.00892 0.0244
2L:20091160-20091310:plus -31.7755 -2.61468 13.5872 3.90826 -18.4898 -9.60204 -17.156
4.43056 -0.917431 0.0157 0.0232 0.0000122 0.00603 0.0116 0.00334 0.0131 0.00336 0.0256
2L:20091520-20091670:minus -40.4796 -1.30275 2.49541 6.12844 -26.5306 -9.30612 -17.8991
6.54167 -0.743119 0.0407 0.0158 0.00481 0.00231 0.0185 0.00257 0.0147 0.00127 0.0243
2L:20091520-20091670:plus -31.7041 0.678899 14.8257 9.59633 -7.64286 -9.45918 -20.156
1.45833 -2.46789 0.0146 0.00813 2.55e-06 0.000275 0.000499 0.00279 0.0212 0.0104 0.0384
2L:20093800-20093950:minus -14.1429 13.5306 5.66972 5.3945 -8.93878 -18.7143 -13.6514
2.94444 -5.92661 0.0012 0.0000206 0.00134 0.00379 0.00216 0.014 0.00671 0.00609 0.0876
2L:20093800-20093950:plus -4.89796 7.85321 4.73394 2.21101 0.908163 -9.7551 -2.36697 1.29167
-4.86239 0.000208 0.000413 0.00199 0.0129 0.000153 0.00358 0.000485 0.011 0.0705
2L:2009740-2009890:minus -34.5306 1.74312 -3.22936 1.65138 -17.1939 -7.79592 -24.1376
9.84722 -3.84404 0.0319 0.00552 0.0307 0.0166 0.00377 0.000723 0.0419 0.000183 0.0533
2L:2009740-2009890:plus -32.6327 5.22018 3.91743 5.16514 0.571429 -17.2755 -15.4587 0.0138889
1.16514 0.0212 0.00149 0.00277 0.00424 0.0003 0.00544 0.00982 0.0167 0.0135
2L:2010460-2010610:minus -23.2245 3.29358 1.00917 8.88073 -27.5306 -18.0816 -10.4679
-1.47222 7.25688 0.00576 0.00312 0.00824 0.000479 0.0351 0.00934 0.00336 0.0258 0.00115
2L:2010460-2010610:plus -32.6735 -0.266055 0.440367 8.05505 -18.1531 -8.72449 -9.22018
1.02778 -1.42202 0.0216 0.0113 0.01 0.000578 0.00829 0.00169 0.00266 0.012 0.0289
2L:20104600-20104750:minus -32.4388 1.16514 -2.6055 -0.66055 -8.93878 -8.7551 -22.2202
6.97222 1.69725 0.0195 0.00682 0.0258 0.033 0.00216 0.00172 0.0307 0.00102 0.0108
2L:20104600-20104750:plus -30.4796 1.6789 5.23853 9.7156 -18.4898 -18.8571 -8.9633 13.125
4.80734 0.00941 0.00565 0.00161 0.000242 0.0116 0.0151 0.00254 8.94e-06 0.00334
2L:20148480-20148630:minus -43 -0.844037 5.05505 3.59633 -27.7551 0.72449 -32.1651 2.52778
-13.0459 0.0889 0.0136 0.00174 0.00693 0.0385 0.000175 0.12 0.00712 0.313
2L:20148480-20148630:plus -13.2959 1.00917 3.65138 7.33028 -18.7551 0.540816 -15.1193
14.375 6.95413 0.000775 0.00722 0.00308 0.00103 0.0144 0.0002 0.00919 1.47e-06 0.00136
2L:20311000-20311150:minus -24.7857 -3.7156 13.9174 1.89908 -18.6837 -8.5 -16.6881 0.166667
2.11927 0.00829 0.0313 8.08e-06 0.0151 0.0126 0.00126 0.0122 0.0159 0.00889
2L:20311000-20311150:plus -20.2143 0.0366972 -1.02752 4.78899 0.571429 -8.27551
6.12844 4.79167 1.52294 0.00165 0.0102 0.0161 0.00456 0.0003 0.000919 0.0000398 0.00288 0.0117
2L:20349960-20350110:minus -23.1122 5.24771 1.74312 9.7156 -17.7143 -9.45918 -19.8257
-4.72222 4.91743 0.0053 0.00147 0.00635 0.000242 0.00627 0.00279 0.02 0.058 0.00321
2L:20349960-20350110:plus -32.9286 -3.18349 -1.13761 -0.449541 -18.1939 -8.72449
-17.055 5.08333 -1.12844 0.025 0.0271 0.0167 0.0307 0.0086 0.00169 0.0129 0.00253 0.0271
2L:203700-203850:minus -30.398 10.5596 1.43119 5.84404 -7.93878 0.755102 -14.7982 2.52778
-0.697248 0.00901 0.000101 0.0071 0.00289 0.000767 0.000175 0.00861 0.00712 0.0239
2L:203700-203850:plus -24.5612 15.1376 -0.724771 2.07339 -18.7551 -9.86735 -22.4862 2.375
4.83486 0.00819 1.81e-06 0.0146 0.0138 0.0144 0.00391 0.0321 0.00753 0.00329
2L:2037620-2037770:minus -34.0714 -2.49541 3.17431 1.65138 -8.30612 -9.57143 -13.3303

1.125 -4.18349 0.031 0.0224 0.00371 0.0166 0.00097 0.00326 0.00624 0.0117 0.0581
2L:2037620-2037770:plus -23.4796 13.5306 7.3578 10.5963 -9.0102 -27.5612 -11.1835 2.63889
-2.17431 0.00634 0.0000206 0.000608 0.0001 0.00236 0.0393 0.00387 0.00684 0.035
2L:2038420-2038570:minus -22.8469 0.633028 -0.926606 -1.05505 -17.3367 -8.57143
-17.9633 7.08333 5.3945 0.00424 0.00826 0.0156 0.0374 0.00391 0.00149 0.0149 0.000963 0.00263
2L:2038420-2038570:plus -23.7755 13.4592 3.88991 6.55963 0.806122 -28.6327 -7.44954 -2.06944
-2.47706 0.00678 0.000064 0.0028 0.00172 0.000229 0.066 0.00187 0.0304 0.0385
2L:2038880-2039030:minus -24.5612 5.87156 3.56881 7.27523 -17.4184 -17.7143 -19.0734
-0.888889 3.46789 0.00819 0.00112 0.00318 0.00109 0.00457 0.00634 0.0177 0.0218 0.00582
2L:2038880-2039030:plus -32.7347 1.88073 3.59633 4.91743 -18.4898 -8.53061 -17.7156 6.25
1.70642 0.0225 0.00524 0.00315 0.00447 0.0116 0.00136 0.0143 0.00147 0.0108
2L:20417000-20417150:minus -24 6.7156 7.88991 2.3945 -7.71429 -18.1531 -19.4954 -2.29167
0.12844 0.00719 0.00075 0.000464 0.0119 0.000572 0.00997 0.0189 0.0322 0.0179
2L:20417000-20417150:plus -12.8469 3.21101 -2.44037 0.504587 -17.8163 -0.244898
-13.1101 2.40278 1.43119 0.000502 0.00322 0.0246 0.0235 0.00682 0.000496 0.00593 0.00746 0.0121
2L:20417380-20417530:minus -20.3571 3.19266 -2.19266 6.52294 -17.1531 -18.8571 -12.9908
-1.44444 3.40367 0.00165 0.00324 0.0229 0.00174 0.00364 0.0151 0.00577 0.0256 0.00603
2L:20417380-20417530:plus -12.7755 -4.89908 1.79817 5.21101 -17.3776 -36.0408 -2.23853
2.76389 9.45872 0.000477 0.043 0.00623 0.0042 0.00419 0.0837 0.00047 0.00652 0.000337
2L:20428840-20428990:minus -14.1429 2.25688 -0.798165 5.49541 -28.2347 9.86735 -4.3945 1.25
-0.284404 0.0012 0.00457 0.015 0.00355 0.051 0.0000132 0.000835 0.0112 0.0207
2L:20428840-20428990:plus -12.1531 -1.92661 1.01835 11.0275 -18.4184 -18.8571 -20.4954
4.83333 -2.00917 0.000352 0.019 0.00821 0.0000401 0.0101 0.0151 0.0225 0.00283 0.0333
2L:2045400-2045550:minus -20.6633 7.10092 12.6147 7.29358 -8.64286 -27.4082 -11.8624
1.625 7.73394 0.00173 0.000615 0.0000278 0.00104 0.00139 0.0356 0.00447 0.00983 0.000784
2L:2045400-2045550:plus -22.7041 -2.3945 5.88073 3.19266 -8.86735 -9.5 -23.6881 -3.81944
1.02752 0.00395 0.0218 0.00122 0.00829 0.00173 0.00294 0.0392 0.0472 0.0141
2L:20463520-20463670:minus -31.4388 2.65138 4.14679 2.40367 -17.8265 -19.0816 -16.9817
9.20833 5.0367 0.0122 0.00395 0.00252 0.0119 0.00691 0.017 0.0128 0.00028 0.00306
2L:20463520-20463670:plus -24.4082 2.0367 -4.04587 6.74312 -19.2755 -19.4184 -26.0734
3.13889 -0.623853 0.00797 0.00495 0.0384 0.0015 0.0164 0.0198 0.054 0.00566 0.0232
2L:20638500-20638650:minus -13 -5.3211 5.0367 4.50459 10.6224 -17.7551 -4.3945 -2.84722 2.21101
0.000563 0.048 0.00175 0.00484 0.0000113 0.00643 0.000835 0.0372 0.00862
2L:20638500-20638650:plus -22.8571 -4.53211 5.80734 4.04587 -18.7143 -27.4082 -11.5963
-5.51389 1.27523 0.00427 0.039 0.00126 0.00572 0.0136 0.0356 0.00422 0.0688 0.013
2L:20645660-20645810:minus -31.7347 1.36697 15.6606 4.37615 -8.16327 9.86735 -12.6606 2.29167
-1.26606 0.0149 0.00633 3.94e-07 0.00504 0.000873 0.0000132 0.00535 0.00776 0.0281
2L:20645660-20645810:plus -31.551 1.36697 0.0917431 7.61468 -17.4184 19.1224 -5.10092 1.01389
1.54128 0.0134 0.00633 0.0112 0.000779 0.00457 9e-07 0.00102 0.0121 0.0115
2L:20647620-20647770:minus -29.4082 -2.6422 -2.80734 -0.0733945 -27.3061 10.6429 -21.0642 -0.597222
5.3945 0.0084 0.0233 0.0273 0.0274 0.0315 3.35e-06 0.0249 0.0201 0.00263
2L:20647620-20647770:plus -23 -0.422018 4.77982 1.05505 -18.7551 -27.602 -15.3945 -0.25 3.52294
0.00498 0.0119 0.00195 0.0204 0.0144 0.0416 0.0097 0.0181 0.00558
2L:20656220-20656370:minus -12.551 2.89908 -1.12844 6.16514 -8.60204 -18.0102 -7.68807 -5.56944
0.0183486 0.000444 0.00361 0.0166 0.00223 0.00125 0.00812 0.00197 0.0696 0.0183
2L:20656220-20656370:plus -32.2245 13.4592 7.22936 0.642202 10.9184 -8.45918 -19.8991 -2.47222 2.25688
0.0183 0.000064 0.000649 0.0227 4.19e-06 0.00111 0.0202 0.0338 0.00853
2L:20675980-20676130:minus -23.6633 5.95413 4.10092 5.73394 -17.7143 0.0510204 -18.2569 -0.75
-0.495413 0.00665 0.00108 0.00257 0.00307 0.00627 0.000349 0.0155 0.021 0.022
2L:20675980-20676130:plus -31.4082 14.633 -0.100917 1.78899 -8.16327 -8.45918 -20.4128 -3.375
3.45872 0.0119 4.09e-06 0.012 0.0158 0.000873 0.00111 0.0221 0.0424 0.00591
2L:20681880-20682030:minus -23.0816 -0.0458716 -3.66972 6.47706 -18.4184 -9.16327 -19.578 3.125
-0.899083 0.00522 0.0105 0.0347 0.00179 0.0101 0.00223 0.0192 0.00569 0.0254
2L:20681880-20682030:plus -32.7755 0.697248 -0.807339 7.46789 -27.2347 -8.09184 -22.0092 1.94444

3.66972 0.0234 0.00807 0.015 0.000868 0.0283 0.000762 0.0295 0.00879 0.00514
2L:20714660-20714810:minus -3.40816 -1.93578 1.58716 11.2936 -26.2653 -18.3469 -21.3394 4.95833
-0.486239 0.0000685 0.0191 0.00672 0.0000139 0.018 0.0124 0.0262 0.00267 0.0219
2L:20714660-20714810:plus -24.1122 3.08257 -1.59633 6.76147 -26.9694 -27.3367 -13.6147 6.93056 5.22936
0.00738 0.00338 0.0192 0.00148 0.023 0.0347 0.00665 0.00104 0.00288
2L:20716100-20716250:minus -21.898 0.293578 1.18349 1.91743 -17.3367 -18.0816 -6.77982 0.569444
3.20183 0.00259 0.00932 0.00775 0.015 0.00391 0.00934 0.0016 0.014 0.0065
2L:20716100-20716250:plus -23.6633 2.20183 0.678899 7.27523 -26.3673 -17.5612 -20.633 -2.97222 -2.46789
0.00665 0.00466 0.00923 0.00109 0.0182 0.00604 0.023 0.0384 0.0384
2L:207300-207450:minus -31.9694 3.55963 3.2844 7 -17.7143 -18.2755 -15.0275 2.40278 1.63303
0.0168 0.00283 0.00356 0.00132 0.00627 0.0112 0.00902 0.00746 0.0112
2L:207300-207450:plus -33.0714 -1.08257 7.25688 -0.926606 -27.2653 -17.5714 -7.6055 4.63889 -0.908257
0.0267 0.0147 0.000641 0.036 0.0302 0.00616 0.00194 0.00307 0.0255
2L:20733680-20733830:minus -31.1429 -1.33945 4.37615 3.61468 -8.37755 -17.3469 -17.8807 5.48611
2.99083 0.0108 0.0159 0.0023 0.0068 0.00106 0.0058 0.0147 0.00211 0.00706
2L:20733680-20733830:plus -30.9592 -3.66972 -2.77982 0.733945 -18.6837 -9.72449 -15.422 3.36111 0.321101
0.0104 0.031 0.0271 0.0222 0.0126 0.00352 0.00975 0.00519 0.0169
2L:20734840-20734990:minus -42.0408 15.7248 2.30275 2.7156 -19.051 -8.45918 -17.7706 4.44444
-4.24771 0.069 5.64e-07 0.00517 0.0102 0.0162 0.00111 0.0144 0.00334 0.0591
2L:20734840-20734990:plus -32.8878 7.7156 -0.623853 6.41284 -8.7449 -18.2041 -15.2569 5.70833
-0.119266 0.0247 0.000445 0.0142 0.00189 0.00161 0.0103 0.00944 0.0019 0.0192
2L:20735300-20735450:minus -12.8469 0.477064 -0.192661 3.02752 -27.0816 -7.38776 -26.0826 0.472222
-2.22018 0.000502 0.00873 0.0123 0.00887 0.0243 0.000563 0.054 0.0144 0.0355
2L:20735300-20735450:plus -24.5204 4.48624 0.155963 0.633028 -18.1837 -27.9694 -6.20183 4.22222 9.45872
0.00813 0.00199 0.011 0.0229 0.00835 0.0547 0.00138 0.00367 0.000337
2L:20757960-20758110:minus -31.4082 -1.41284 -0.651376 6.48624 -18.1531 -8.5 -19.5413 2.97222
1.85321 0.0119 0.0163 0.0143 0.00177 0.00829 0.00126 0.0191 0.00603 0.01
2L:20757960-20758110:plus -21.9592 4.69725 0.798165 2.88991 20.1735 -19.0816 -5.6055 0.805556 -0.302752
0.00279 0.00184 0.00886 0.00942 5.68e-07 0.017 0.00118 0.013 0.0208
2L:20758380-20758530:minus -31.4082 -2.68807 2.62385 2.97248 -26.7041 -18.9796 -16.7523 -0.625
-8.27523 0.0119 0.0236 0.00458 0.00909 0.0193 0.0155 0.0123 0.0202 0.143
2L:20758380-20758530:plus -24.1837 2.24771 0.266055 2.52294 -17.2653 0.0204082 -11.2294 -1.19444
4.91743 0.00751 0.00458 0.0106 0.0113 0.00387 0.000377 0.00391 0.0239 0.00321
2L:20787560-20787710:minus -23.8878 7.73394 -0.33945 2.77064 -27.2653 -17.2755 -15.844 8.52778
-1.58716 0.00692 0.00044 0.0129 0.01 0.0302 0.00544 0.0105 0.000429 0.03
2L:20787560-20787710:plus -22.8878 6.07339 0.954128 5.22018 -18.7143 -17.7857 -20.7523 -1.31944 -2.83486
0.00441 0.00102 0.0084 0.00418 0.0136 0.0068 0.0235 0.0247 0.0427
2L:20794160-20794310:minus -21.6633 -1.25688 0.981651 8.80734 -16.7143 -17.7857 -13.5321 -5.55556
-0.688073 0.00223 0.0155 0.00832 0.000518 0.00319 0.0068 0.00653 0.0694 0.0238
2L:20794160-20794310:plus -22.4388 -2.23853 0.568807 9.61468 -28.2347 -19.051 -13.2661 -0.125 4.49541
0.00348 0.0208 0.00958 0.000255 0.051 0.0165 0.00614 0.0174 0.00363
2L:20797620-20797770:minus -30.9694 3.64286 -2.3211 2.3578 -28.7959 -18.3469 -25.0275 7 -4.58716
0.0104 0.00159 0.0237 0.0121 0.0641 0.0124 0.0473 0.00101 0.0652
2L:20797620-20797770:plus -20.3265 0.0366972 5.78899 6.12844 -27.4592 19.4184 -11.5413 0.680556
1.24771 0.00165 0.0102 0.00127 0.00231 0.0329 2.36e-07 0.00418 0.0135 0.0131
2L:20798420-20798570:minus -23.4898 1.09174 1.34862 2.62385 -28.2347 0.0510204 -8.9633 0.805556
-4.11009 0.00639 0.007 0.00731 0.0108 0.051 0.000349 0.00254 0.013 0.0571
2L:20798420-20798570:plus -24.449 4.41284 -3.27523 -0.284404 -17.2245 -26.9796 -15.055 -0.694444
11.8532 0.00802 0.00205 0.0311 0.0291 0.00384 0.0306 0.00907 0.0206 0.0000428
2L:20799700-20799850:minus -31.5918 0.238532 -0.614679 9.33028 -27.2959 -8.79592 -20.4771 0.263889
1.90826 0.0136 0.0095 0.0141 0.000374 0.0303 0.0019 0.0224 0.0154 0.00974
2L:20799700-20799850:plus -23.8469 -3.50459 3.19266 6.25688 -17.7143 -17.7857 -20.8349 1.86111 -0.93578
0.00685 0.0296 0.00369 0.00208 0.00627 0.0068 0.0239 0.00905 0.0257
2L:20800620-20800770:minus -31.3673 4.59633 5.78899 6.33028 -17.8878 -18.3061 -16.7982 -2.61111

-0.247706 0.0116 0.00191 0.00127 0.00198 0.00704 0.0114 0.0124 0.035 0.0204
2L:20800620-20800770:plus -13.2653 -4.92661 -1.54128 3.18349 -7.56122 -17.9388 -13.9358 -1.06944 -2.62385
0.000759 0.0433 0.0188 0.00837 0.000419 0.00738 0.00715 0.023 0.0402
2L:20801280-20801430:minus -14.7143 -0.201835 5.49541 5.75229 -26.9694 -9.53061 -22.6147 -1.59722
-0.449541 0.00154 0.011 0.00144 0.00296 0.023 0.0031 0.0329 0.0267 0.0216
2L:20801280-20801430:plus -31.8571 0.697248 0.0275229 6.84404 -27.0408 -9.45918 -20.8165 -0.291667
-4.68807 0.0163 0.00807 0.0115 0.0014 0.0242 0.00279 0.0238 0.0183 0.0672
2L:20804000-20804150:minus -21.4796 3.36697 -2.3578 -0.40367 -18.6429 -27.8265 -11.8899 -0.166667
3.40367 0.00202 0.00304 0.024 0.0302 0.0121 0.0498 0.0045 0.0176 0.00603
2L:20804000-20804150:plus -22.5918 1.59633 -1.11927 -0.651376 -18.8265 -26.6735 -20.9083 2.95833
4.19266 0.00365 0.00582 0.0166 0.0329 0.0149 0.0256 0.0242 0.00606 0.00401
2L:20805680-20805830:minus -24.1429 1.22018 0.981651 9.3211 -27.3061 -9.30612 -16.2385 3.98611
0.146789 0.0074 0.00668 0.00832 0.000391 0.0315 0.00257 0.0113 0.00404 0.0178
2L:20805680-20805830:plus -40.7041 2.55046 -1.06422 3.2844 -18.7143 -0.540816 -28.0642 3.79167
3.88073 0.0428 0.0041 0.0163 0.00793 0.0136 0.00054 0.0694 0.00437 0.00475
2L:20806220-20806370:minus -31.8878 0.0366972 4.56881 5.47706 -26.6633 -18.5714 -10.1009 8.98611
4.14679 0.0164 0.0102 0.00213 0.0036 0.0189 0.0136 0.00314 0.000323 0.00406
2L:20806220-20806370:plus -23.2959 -1.2844 5.40367 5.38532 -17.9286 -7.23469 -12.6055 8.44444 1.68807
0.00605 0.0157 0.0015 0.00382 0.0071 0.000557 0.00528 0.000451 0.0109
2L:20822800-20822950:minus -14.2959 3.51376 8.56881 3.53211 -18.5612 -36.1531 -16.2936 -0.305556
8.11009 0.00129 0.00288 0.00032 0.00704 0.0119 0.0908 0.0114 0.0184 0.000597
2L:20822800-20822950:plus -21.5102 1.33028 8.18349 10.5963 -18.4184 -26.2653 -15.7523 -5.45833 -1
0.00202 0.00642 0.000397 0.0001 0.0101 0.0218 0.0104 0.068 0.0261
2L:20823640-20823790:minus -43.1122 0.541284 2.12844 1.06422 -27.2347 -17.6429 -25.6422 2.70833
-4.22936 0.0897 0.00854 0.00551 0.0203 0.0283 0.00619 0.0512 0.00666 0.0588
2L:20823640-20823790:plus 15.9286 -1.78899 8.13761 -0.669725 -8.63265 -28.6327 -14.5046 -0.5 5.23853
1.53e-07 0.0183 0.000407 0.0331 0.0013 0.066 0.0081 0.0195 0.00285
2L:20824480-20824630:minus -31.3673 1.20183 4.45872 6.29358 -18.449 -9.72449 -22.5413 -1.11111
-3.18349 0.0116 0.00673 0.00223 0.00202 0.0107 0.00352 0.0324 0.0233 0.0465
2L:20824480-20824630:plus 5.5102 -0.908257 -0.513761 1.92661 -27.3061 -8.79592 -10.7248 -0.805556
3.22018 9.3e-06 0.0139 0.0137 0.0148 0.0315 0.0019 0.00353 0.0213 0.0064
2L:20825320-20825470:minus -24.2245 -3.86239 12.2018 9.92661 -18.1531 -18.7857 -18.2202 6.125
-2.3578 0.00763 0.0326 0.0000374 0.000169 0.00829 0.0146 0.0155 0.00156 0.0369
2L:20825320-20825470:plus -22.8878 2.43119 3.76147 11.2936 -8.89796 -18.0816 -3.33945 3.52778 1.34862
0.00441 0.00428 0.00295 0.0000139 0.00189 0.00934 0.000621 0.00486 0.0125
2L:20829480-20829630:minus -24.0714 -2.73394 -0.376147 2.29358 -18.0102 -18.3469 -20.4679 4.91667
-4.3211 0.00731 0.0239 0.0131 0.0124 0.00732 0.0124 0.0223 0.00272 0.0605
2L:20829480-20829630:plus -21.6633 3.91743 0.302752 6.84404 -17.5612 -8.79592 -25.578 1.94444 -0.697248
0.00223 0.00248 0.0105 0.0014 0.00544 0.0019 0.0508 0.00879 0.0239
2L:20830060-20830210:minus -21.7041 7.97248 -0.513761 2.70642 -18.1939 -17.0102 -3.2844 3.69444
1.55963 0.00231 0.000387 0.0137 0.0103 0.0086 0.00453 0.000612 0.00455 0.0114
2L:20830060-20830210:plus -23.2245 1.2844 2.58716 3.45872 -26.9694 -9.57143 -12.5963 4.88889 2.11009
0.00576 0.00653 0.00465 0.00731 0.023 0.00326 0.00527 0.00276 0.00898
2L:20831200-20831350:minus -32 -2.29358 7.21101 1.42202 -17.3776 -8.72449 -7.55963 4.58333 1.95413
0.0171 0.0211 0.000655 0.0182 0.00419 0.00169 0.00192 0.00315 0.00964
2L:20831200-20831350:plus -12.7755 -0.155963 2.29358 11.0183 -18.2653 -9.23469 -6.30275 14.0139
0.917431 0.000477 0.0109 0.00518 0.000052 0.00882 0.00238 0.00142 2.52e-06 0.0146
2L:20864660-20864810:minus -32.9286 -0.00917431 5.72477 6.57798 -8.96939 -17.7857 6.53211 13.2639
1.68807 0.025 0.0103 0.0013 0.00167 0.00226 0.0068 0.0000354 7.59e-06 0.0109
2L:20864660-20864810:plus -14.0408 0.477064 5.90826 3.50459 -27.602 -17.7857 -14.8532 2.98611 10.3394
0.00115 0.00873 0.0012 0.00713 0.0364 0.0068 0.00871 0.006 0.000112
2L:20865060-20865210:minus -23.5306 -2.51376 2.53211 -0.275229 -18.6837 -10.0204 -5.53211 0.5
2.88991 0.00646 0.0225 0.00475 0.029 0.0126 0.00399 0.00115 0.0143 0.00725
2L:20865060-20865210:plus -13.6327 2.99083 4.9633 6.12844 -26.8571 0.755102 -6.83486 -0.972222

5.22936	0.00101	0.00349	0.00181	0.00231	0.0198	0.000175	0.00162	0.0224	0.00288
2L:20879900-20880050:minus	-32.0714	-2.14679	-0.0733945	5.43119	-16.8878	-18.4184	-6.99083	0.708333
-3.36697	0.0176	0.0203	0.0119	0.00371	0.00326	0.0127	0.00168	0.0134	0.0482
2L:20879900-20880050:plus	-31.551	4.91743	7.34862	3.3578	-18.5204	-26.602	-16.8165	3.44444	6.7156
0.0134	0.00169	0.000611	0.00767	0.0117	0.0248	0.0124	0.00502	0.00144
2L:20884600-20884750:minus	-31.7041	4.2844	1.83486	3.43119	-18.7857	-18.2755	-15.7156	5.47222
-2.27523	0.0146	0.00216	0.00614	0.00749	0.0148	0.0112	0.0103	0.00212	0.036
2L:20884600-20884750:plus	-23.8163	5.25688	5.44037	0.0183486	-27.8673	-8.72449	-12.9174	3.27778
4.37615	0.00682	0.00146	0.00148	0.0268	0.0391	0.00169	0.00567	0.00536	0.00376
2L:20886040-20886190:minus	-31.551	-1.82569	-1.21101	0.788991	-26.7041	-18.6327	4.58716	2.34722
1.42202	0.0134	0.0185	0.017	0.0218	0.0193	0.0137	0.0000667	0.00761	0.0121
2L:20886040-20886190:plus	-14.2959	5.78899	0.688073	1.69725	-18.6429	-18.2755	-13.0826	6.08333	9.87156
0.00129	0.00117	0.0092	0.0163	0.0121	0.0112	0.00589	0.00159	0.000217
2L:20891320-20891470:minus	-23.8878	4.74312	5.11009	3.33945	-16.7143	-19.1224	-23.2569	-5.41667
-4.34862	0.00692	0.00181	0.0017	0.00774	0.00319	0.0175	0.0366	0.0674	0.0612
2L:20891320-20891470:plus	-22.8878	-5.76147	0.486239	5.47706	-8.67347	-18.2347	-15.156	0.777778	-0.642202
0.00441	0.0538	0.00986	0.0036	0.00154	0.0104	0.00926	0.0131	0.0234
2L:20911360-20911510:minus	-33	9.77064	2.17431	1.7156	-26.9694	-27.9286	-14.2294	-0.333333
-0.238532	0.0258	0.00015	0.00542	0.0162	0.023	0.054	0.00763	0.0185	0.0203
2L:20911360-20911510:plus	-3.44898	1.77982	-1.10092	9.7156	-26.2347	-19.051	-12.7248	-2.30556	5.70642
0.0000713	0.00544	0.0165	0.000242	0.0177	0.0165	0.00543	0.0323	0.00222
2L:20912900-20913050:minus	-3.22449	3.90816	3.12844	3.09174	-27.4592	-17.9388	-7.26606	1.18056
3.20183	0.0000495	0.00102	0.00378	0.00867	0.0329	0.00738	0.00179	0.0114	0.0065
2L:20912900-20913050:plus	-20.1327	-2.10092	-0.0458716	1.26606	-18.1531	-17.4184	-14.578	2.61111
2.75229	0.00164	0.02	0.0118	0.0191	0.00829	0.00585	0.00822	0.00691	0.00748
2L:20913260-20913410:minus	-24.5918	-3.98165	0.229358	5.01835	-26.2653	-18.7857	-10.0183	5.52778
4.37615	0.00821	0.0337	0.0107	0.00438	0.018	0.0146	0.00309	0.00207	0.00376
2L:20913260-20913410:plus	-22.7347	0.477064	4.33028	3.97248	-8.60204	-17.8265	-15.4037	2.97222	3.97248
0.00403	0.00873	0.00235	0.00587	0.00125	0.00699	0.00972	0.00603	0.00444
2L:20914040-20914190:minus	-21.8878	2.41284	-0.963303	7.95413	-17.8571	-8.5	-13.1835	1.875
-0.0733945	0.00258	0.00431	0.0158	0.000599	0.00698	0.00126	0.00603	0.00901	0.0188
2L:20914040-20914190:plus	-12.8061	5.25688	-2.93578	6.00917	-18.7143	-8.79592	-12.633	4.04167	0.733945
0.000481	0.00146	0.0283	0.00255	0.0136	0.0019	0.00532	0.00395	0.0153
2L:20916560-20916710:minus	-22.1531	-1.06422	-3.11009	1.77982	-27.1939	0.346939	-29.5596	4.16667
7.42202	0.0031	0.0146	0.0297	0.0158	0.0267	0.000221	0.0854	0.00375	0.00106
2L:20916560-20916710:plus	-30.7857	3.29358	-0.0733945	8.81651	-18.3367	-19.0816	-20.8624	2.94444
1.16514	0.0102	0.00312	0.0119	0.000501	0.00885	0.017	0.024	0.00609	0.0135
2L:20917420-20917570:minus	-30.4082	1.94495	-0.577982	6.47706	-17.8163	-8.57143	-8.42202	8.65278
1.58716	0.00908	0.00512	0.014	0.00179	0.00682	0.00149	0.00229	0.000397	0.0113
2L:20917420-20917570:plus	-23.2653	4.08257	-1.06422	7.62385	-17.3061	-18.6429	-20.3119	4.72222	-0.642202
0.00593	0.00233	0.0163	0.000759	0.00389	0.0139	0.0217	0.00296	0.0234
2L:20917960-20918110:minus	-24.2347	-2.86239	1.46789	6.84404	-9.5	-8.72449	-23.1101	7.83333	-2.57798
0.00763	0.0248	0.007	0.0014	0.00293	0.00169	0.0357	0.000641	0.0397
2L:20917960-20918110:plus	-23.8469	1.74312	6.83486	5.55046	-17.6429	-8.79592	-26.7248	0.0416667
-5.83486	0.00685	0.00552	0.000785	0.00345	0.00562	0.0019	0.0585	0.0165	0.0858
2L:20918480-20918630:minus	-13.1224	-3.85321	0.642202	2.9633	-27.5306	-8.45918	-20.5872	4.11111
-5.22936	0.000633	0.0325	0.00935	0.00914	0.0351	0.00111	0.0228	0.00384	0.0764
2L:20918480-20918630:plus	-30.9286	-0.495413	3.07339	3.95413	0.540816	-0.316327	-12.2661	11.1667
2.46789	0.0104	0.0122	0.00386	0.00593	0.000321	0.000524	0.00489	0.0000662	0.00805
2L:20922880-20923030:minus	-30.6327	-1.47706	11.1468	7.45872	-26.5306	-17.8571	-16.6789	2.98611
-2.44037	0.00968	0.0166	0.0000727	0.000894	0.0185	0.00719	0.0122	0.006	0.0381
2L:20922880-20923030:plus	-43.3367	-0.642202	-1.3211	1.97248	-9.45918	-0.469388	-24.2752	3.93056
-2.11927	0.0909	0.0128	0.0176	0.0146	0.00287	0.000533	0.0427	0.00413	0.0344
2L:20926120-20926270:minus	-22.8776	1.11927	3.3945	6.14679	10.6224	-18.9388	-18.1743	-1.18056

-2.6055 0.00428 0.00693 0.00341 0.00224 0.0000113 0.0154 0.0154 0.0238 0.04
2L:20926120-20926270:plus -41.7041 2.65138 1.53211 9.08257 -18.4898 9.34694 -26.1284 2.44444 3.46789
0.0641 0.00395 0.00685 0.000444 0.0116 0.0000366 0.0543 0.00734 0.00582
2L:20927800-20927950:minus -5.26531 13.3878 -2.23853 1.78899 -27.2347 -26.5612 -9.38532 -1.56944
5.13761 0.000231 0.0000869 0.0232 0.0158 0.0283 0.0237 0.00275 0.0265 0.00298
2L:20927800-20927950:plus -31.4796 5.02752 1.20183 2.82569 2.14286 -0.0204082 -25.1927 0.680556
3.11009 0.0128 0.00161 0.0077 0.00976 0.0000316 0.000411 0.0484 0.0135 0.00669
2L:20936180-20936330:minus -30.4796 4.73394 2.38532 2.77064 -17.4184 -17.7857 -13.211 2.26389
6.80734 0.00941 0.00181 0.00501 0.01 0.00457 0.0068 0.00607 0.00784 0.0014
2L:20936180-20936330:plus -22.4796 1.63303 6.58716 5.31193 -18.1531 -19.0816 -1.29358 5.15278 1.89908
0.00352 0.00575 0.00088 0.00393 0.00829 0.017 0.000379 0.00245 0.0098
2L:21051340-21051490:minus -31.5204 -5.2844 3.18349 4.65138 -8.71429 -16.9796 -9.69725 -0.625
2.88073 0.0131 0.0475 0.0037 0.00471 0.00159 0.0044 0.00291 0.0202 0.00727
2L:21051340-21051490:plus -40.6224 -2.91743 0.146789 -0.541284 -18.4796 -37.5204 -6.24771 2.41667
2.78899 0.0424 0.0252 0.011 0.0316 0.011 0.166 0.0014 0.00742 0.00745
2L:21052040-21052190:minus -13.2959 -1.90826 2.36697 7.15596 -17.1939 -18.8571 -2.02752 5.23611
1.54128 0.000775 0.0189 0.00505 0.00117 0.00377 0.0151 0.000447 0.00236 0.0115
2L:21052040-21052190:plus -38.9592 -2.12844 -1.10092 1.55963 0.877551 -17.3469 -12.6881 4.61111 7.73394
0.0324 0.0202 0.0165 0.0173 0.00019 0.0058 0.00538 0.00311 0.000784
2L:21096720-21096870:minus -23.6327 -3.08257 -1.88991 5.6422 -18.4184 -7.94898 -18.3853 -1.63889
0.33945 0.00661 0.0264 0.0209 0.00325 0.0101 0.00074 0.0159 0.027 0.0168
2L:21096720-21096870:plus -30.551 2.53211 3.95413 0.743119 -27.9694 -18.1939 -14.8532 11.7361 5.81651
0.00959 0.00413 0.00273 0.0221 0.0437 0.0101 0.00871 0.0000398 0.00204
2L:21102440-21102590:minus -29.102 1.41284 4.46789 7.61468 10.9184 9.5 -10.0826 2.90278 1.01835
0.00833 0.00623 0.00222 0.000779 4.19e-06 0.0000337 0.00313 0.00619 0.0142
2L:21102440-21102590:plus -21.9592 -2.55046 1.73394 6.06422 -17.3776 -17.2755 -16.9358 11.4306 3
0.00279 0.0227 0.00638 0.00239 0.00419 0.00544 0.0127 0.0000525 0.00702
2L:21108080-21108230:minus -30.3673 2.16514 1.82569 3.74312 -7.89796 -17.9388 -19.945 4.18056
-7.25688 0.00897 0.00473 0.00616 0.00651 0.000678 0.00738 0.0204 0.00373 0.119
2L:21108080-21108230:plus -30.551 -2.45872 12.1193 1.36697 -27.3469 -18.5612 -9.94495 0.736111 11.8073
0.00959 0.0222 0.0000394 0.0186 0.0318 0.0132 0.00305 0.0132 0.0000538
2L:21108520-21108670:minus -24.2959 2.55963 1.41284 2.51376 -18.0816 -26.6327 -10.422 -1.36111
0.238532 0.00781 0.00409 0.00714 0.0113 0.00759 0.0251 0.00333 0.025 0.0173
2L:21108520-21108670:plus -13.449 0.834862 0.944954 11.1927 -26.8571 0.755102 -19.2477 -1.26389 5.95413
0.000914 0.00769 0.00842 0.0000254 0.0198 0.000175 0.0182 0.0243 0.00192
2L:21109200-21109350:minus -33.8878 -3.61468 2.40367 1.41284 1.54082 0.602041 -26.211 0.569444
2.51376 0.0302 0.0305 0.00498 0.0183 0.0000712 0.000188 0.0549 0.014 0.00792
2L:21109200-21109350:plus -24.4796 0.853211 2.38532 5.16514 -18.6429 -17.2755 -17.1927 7.26389 -4.41284
0.00804 0.00763 0.00501 0.00424 0.0121 0.00544 0.0132 0.000876 0.0624
2L:21142280-21142430:minus -14.1122 5.23853 -4.37615 3.77064 2.21429 -16.7857 -2.40367 2.73611
1.74312 0.00119 0.00148 0.042 0.00637 0.0000277 0.00426 0.000489 0.00659 0.0105
2L:21142280-21142430:plus -23.5918 6.12844 -2.91743 -0.66055 -26.2347 -17.9388 -6.0367 3.73611 6.69725
0.00656 0.000997 0.0282 0.033 0.0177 0.00738 0.00132 0.00447 0.00145
2L:21143120-21143270:minus -30.6633 3.61468 1.22936 9.08257 -27.5306 -0.0204082 -31.4954 1.80556
-2.18349 0.00976 0.00277 0.00763 0.000444 0.0351 0.000411 0.111 0.00923 0.0351
2L:21143120-21143270:plus -23.1837 13.4592 -0.706422 0.00917431 -27.9694 -17.2755 -15.6972 2.16667
-6.15596 0.00553 0.000064 0.0146 0.0269 0.0437 0.00544 0.0103 0.00812 0.0923
2L:21154000-21154150:minus -32.7347 8.38532 -1.9633 1 -8.86735 -17.4184 -23.789 4.34722 -2.6789
0.0225 0.000309 0.0214 0.0206 0.00173 0.00585 0.0398 0.00348 0.0408
2L:21154000-21154150:plus -33.6531 -3.0367 14.8624 7.29358 -18.3367 -28.8571 -12.211 1.125 -4.6789
0.029 0.026 2.2e-06 0.00104 0.00885 0.0699 0.00483 0.0117 0.067
2L:21156220-21156370:minus -31.5102 0.431193 -0.431193 -0.550459 0.877551 -28.898 -3.2844
8.91667 8.14679 0.013 0.00887 0.0133 0.0317 0.00019 0.0713 0.000612 0.000338 0.000562
2L:21156220-21156370:plus -31.2143 13.4592 -3.89908 1.99083 -17.7143 -0.469388 -15.0367 2.09722

-4.15596 0.0111 0.000064 0.037 0.0144 0.00627 0.000533 0.00904 0.00832 0.0578
2L:21156760-21156910:minus -21.3673 2.30275 3.92661 6.72477 -8.89796 -19.0816 -4.34862 4.33333
3.42202 0.00195 0.00449 0.00276 0.00154 0.00189 0.017 0.000824 0.0035 0.00597
2L:21156760-21156910:plus -30.449 -1.12844 3.02752 6.17431 1.17347 -17.7143 -29.0367 3.02778 -4.44037
0.00925 0.0149 0.00393 0.0022 0.000101 0.00634 0.0793 0.0059 0.063
2L:21158620-21158850:minus -13.8163 0.0733945 13.9541 7.2844 -26.9286 -8.42857 -16.7706 0.833333
0.93578 0.00109 0.0101 7.63e-06 0.00105 0.0214 0.00104 0.0124 0.0128 0.0145
2L:21158620-21158850:plus -31.6633 1.45872 6.24771 9.7156 -18.7143 -9.5 -8.40367 -2.69444 0.238532
0.0141 0.00613 0.00103 0.000242 0.0136 0.00294 0.00228 0.0358 0.0173
2L:21163040-21163190:minus -23.1122 13.3878 1.38532 7 -18.1531 -18.0102 -23.1009 -1.125 -2.87156
0.0053 0.0000869 0.00721 0.00132 0.00829 0.00812 0.0357 0.0234 0.043
2L:21163040-21163190:plus -33.3673 13.5306 3.24771 5.30275 -27.2347 -9.72449 -17.2936 2.59722 -0.908257
0.0282 0.0000206 0.00361 0.00396 0.0283 0.00352 0.0134 0.00694 0.0255
2L:21166600-21166750:minus -32.8061 -2.11927 -2.24771 1.74312 -27.0816 -18.6429 -7.62385 1.15278
-0.40367 0.0237 0.0201 0.0232 0.016 0.0243 0.0139 0.00194 0.0115 0.0213
2L:21166600-21166750:plus -13.4388 5.98165 10.367 3.42202 -17.4898 -18.1939 -14.055 11.1806 -2.43119
0.000879 0.00107 0.000114 0.00751 0.0053 0.0101 0.00734 0.0000655 0.038
2L:21168500-21168650:minus -31.1429 -0.366972 0.917431 3.6422 -26.9286 -0.173469 -12.1743 -0.5
1.33945 0.0108 0.0117 0.00851 0.00669 0.0214 0.000456 0.00479 0.0195 0.0125
2L:21168500-21168650:plus -23.2959 -2.3945 1.91743 1.85321 -7.93878 -8.72449 -22.055 1.01389 9.75229
0.00605 0.0218 0.00596 0.0153 0.000767 0.00169 0.0298 0.0121 0.000241
2L:21179660-21179810:minus -33 -0.385321 1.88073 3.69725 -17.4184 -17.0102 -21.6789 1.18056
-4.44954 0.0258 0.0117 0.00604 0.00665 0.00457 0.00453 0.0278 0.0114 0.0632
2L:21179660-21179810:plus -32.0714 -1.92661 1.56881 2.31193 20.4694 -18.1224 -17.578 -0.180556
1.68807 0.0176 0.019 0.00676 0.0123 1.65e-07 0.00988 0.014 0.0177 0.0109
2L:21214920-21215070:minus -32.7857 8.12844 2.50459 2.34862 -27.3061 -0.0204082 -28.5229 1.70833
9.9633 0.0235 0.000356 0.0048 0.0121 0.0315 0.000411 0.0739 0.00955 0.000171
2L:21214920-21215070:plus -20.7041 -1.01835 -1.74312 1.13761 -9.23469 -9.0102 -19.3211 2.90278 -1.77982
0.00175 0.0144 0.02 0.0198 0.00277 0.00208 0.0184 0.00619 0.0314
2L:21218640-21218790:minus -31.9694 6.36697 4.78899 6.21101 1.87755 -26.2653 -15.8899 2.01389
9.77064 0.0168 0.000889 0.00194 0.00213 0.0000492 0.0218 0.0106 0.00858 0.000228
2L:21218640-21218790:plus -22.1531 1.89908 9.86239 10.7615 -17.6429 -8.45918 -16.789 0.972222 3.46789
0.0031 0.00521 0.000155 0.0000826 0.00562 0.00111 0.0124 0.0123 0.00582
2L:21219960-21220110:minus -21.2959 3.08257 -0.0183486 4.3945 -8.93878 -9.79592 -20.8807 -2.72222
8.19266 0.00192 0.00338 0.0117 0.00499 0.00216 0.00377 0.0241 0.036 0.000548
2L:21219960-21220110:plus -24.1531 6.6789 6.9633 7.17431 -17.3776 -9.16327 -29.9908 1.61111 -12.211
0.00744 0.000764 0.000739 0.00115 0.00419 0.00223 0.0908 0.00988 0.274
2L:21220480-21220630:minus -32.0306 2.38532 -0.724771 3.10092 -18.449 -17.8265 -5.29358 2.81944
5.12844 0.0172 0.00436 0.0146 0.00865 0.0107 0.00699 0.00108 0.00639 0.00301
2L:21220480-21220630:plus -33.7347 2.01835 4.13761 3.86239 -18.7143 -26.8571 -17.5138 7.94444 -3.13761
0.0294 0.00499 0.00254 0.00622 0.0136 0.0288 0.0139 0.000602 0.0461
2L:21221120-21221270:minus -30.4082 3.90816 1.06422 5.33028 -18.4898 -9.23469 -24.3853 2.76389
-0.0275229 0.00908 0.00102 0.00808 0.00389 0.0116 0.00238 0.0434 0.00652 0.0186
2L:21221120-21221270:plus -32.9286 3.46789 1.15596 4.22936 10.9184 -9.53061 -23.3578 -1.01389 0.394495
0.025 0.00293 0.00783 0.00525 4.19e-06 0.0031 0.0372 0.0227 0.0166
2L:21237680-21237830:minus -31.2143 0.761468 -4.54128 6.00917 -17.1122 -8.45918 -10.8991 -3.16667
-0.623853 0.0111 0.00789 0.0438 0.00255 0.00341 0.00111 0.00366 0.0403 0.0232
2L:21237680-21237830:plus -12.551 2.65138 -0.798165 7.80734 -18.7551 -19.1531 -2.42202 5.375
7.94495 0.000444 0.00395 0.015 0.00063 0.0144 0.0177 0.000491 0.00222 0.000667
2L:2126040-2126190:minus -40.449 0.486239 2.49541 5.49541 -27.1939 -8.5 -14.3119 3.36111 -4.66055
0.0402 0.00871 0.00481 0.00355 0.0267 0.00126 0.00777 0.00519 0.0667
2L:2126040-2126190:plus -24.8571 -0.954128 1.63303 6.12844 -9.27551 -18.3469 0.302752 0.0138889
4.25688 0.00831 0.0141 0.00661 0.00231 0.00284 0.0124 0.000263 0.0167 0.00391
2L:21261000-21261150:minus -31.6735 2.2844 4.3211 3.31193 -17.4184 -7.42857 -14.2569 3.77778

-3.55963	0.0141	0.00452	0.00235	0.00786	0.00457	0.000567	0.00768	0.0044	0.0503
2L:21261000-21261150:plus	-33.0816	0.688073	10.3211	2.72477	1.14286	-18.2755	-14.578	-0.527778
1.53211	0.0268	0.0081	0.000118	0.0102	0.000113	0.0112	0.00822	0.0196	0.0116
2L:21262880-21263030:minus	-40.2857	-1.22018	0.0917431	0.486239	-9.19388	0.530612	-24.5963	-0.430556
1.06422	0.0385	0.0154	0.0112	0.0237	0.00253	0.000203	0.0447	0.0191	0.014
2L:21262880-21263030:plus	-23.1224	3.69725	0.440367	3.88991	20.4694	-9.27551	-13.2018	1.51389	-1.15596
0.00531	0.00269	0.01	0.00608	1.65e-07	0.00244	0.00605	0.0102	0.0273
2L:21263560-21263710:minus	-31.7653	1.78899	7.21101	7.47706	-8.93878	-19.0102	-5.54128	-1.51389
6.17431	0.0152	0.00542	0.000655	0.00085	0.00216	0.0159	0.00116	0.0261	0.00171
2L:21263560-21263710:plus	-31.4796	-3.44954	15.7339	7.29358	-19.0102	-9.02041	-11.3945	10.2639	1.16514
0.0128	0.0292	2.45e-07	0.00104	0.0157	0.00213	0.00405	0.000135	0.0135
2L:2127700-2127850:minus	-13.2245	1.20183	4.08257	2.55963	-17.5612	-17.051	-6.41284	0.361111	9.45872
0.000738	0.00673	0.00259	0.0111	0.00544	0.00479	0.00146	0.0149	0.000337
2L:2127700-2127850:plus	-32.4796	-2.66972	-2	-1.6789	-7.93878	-28.6327	-7.22936	-1.22222	5.6055	0.02
0.0235	0.0216	0.0459	0.000767	0.066	0.00178	0.024	0.00232
2L:2129220-2129370:minus	-22.2245	5.26606	2.20183	5.57798	-26.6633	0.244898	-9.44954	-4.38889	1.29358
0.00325	0.00146	0.00536	0.0034	0.0189	0.000285	0.00278	0.0538	0.0128
2L:2129220-2129370:plus	-20.9184	3.55963	-0.715596	6.6422	-19.3163	0.0204082	-5.88991	-1.84722
-0.513761	0.00181	0.00283	0.0146	0.00161	0.0165	0.000377	0.00127	0.0286	0.0221
2L:2130280-2130430:minus	-32.3367	3.87156	0.513761	2.31193	-26.4898	-17.2755	-20.6514	3.375	0.275229
0.0187	0.00252	0.00977	0.0123	0.0183	0.00544	0.0231	0.00516	0.0171
2L:2130280-2130430:plus	-23.2551	-4.48624	1.00917	-1.19266	-18.4082	-27.5612	-13.6239	4.70833	1.90826
0.00583	0.0385	0.00824	0.0391	0.00937	0.0393	0.00667	0.00298	0.00974
2L:2130720-2130870:minus	-11.8469	0.00917431	1.44954	1.48624	-17.7143	0.244898	-13.9358	-1.72222
3.9633	0.000279	0.0103	0.00705	0.0177	0.00627	0.000285	0.00715	0.0276	0.00446
2L:2130720-2130870:plus	-20.4796	-0.568807	1.44037	1.87156	-27.4592	-17.0102	-7.3945	7.15278
2.16514	0.00167	0.0125	0.00707	0.0152	0.0329	0.00453	0.00185	0.000928	0.00879
2L:21309420-21309570:minus	-13.2551	0.165138	0.284404	5.2844	-17.4898	-8.7551	-26.7064	-6.76389
-3.69725	0.000751	0.00974	0.0105	0.004	0.0053	0.00172	0.0583	0.0885	0.0517
2L:21309420-21309570:plus	-2.92857	3.13761	3.65138	6.12844	-17.4184	-16.7143	1.51376	-1.20833	4.19266
0.0000395	0.00331	0.00308	0.00231	0.00457	0.00419	0.000191	0.0239	0.00401
2L:21310440-21310590:minus	-32.2653	13.7982	-2.53211	1.78899	-18.7143	-8.45918	-24.4128	4.58333
3.98165	0.0184	0.0000105	0.0252	0.0158	0.0136	0.00111	0.0436	0.00315	0.00439
2L:21310440-21310590:plus	-32.9286	15.7248	0.688073	2.27523	-9.16327	-19.3469	-21.2844	-0.958333
-6.22018	0.025	5.64e-07	0.0092	0.0125	0.00249	0.0194	0.0259	0.0223	0.0938
2L:21310980-21311130:minus	-22.1429	3.17431	-0.0183486	3.23853	-27.1531	-9.65306	-19.7064	3.19444
1.83486	0.00305	0.00326	0.0117	0.00815	0.0249	0.0034	0.0196	0.00554	0.0101
2L:21310980-21311130:plus	-31.1429	3.71429	2.78899	7.27523	-8.90816	-17.051	-20.4679	-2.73611	2.74312
0.0108	0.00129	0.0043	0.00109	0.00192	0.00479	0.0223	0.0362	0.00751
2L:21313220-21313370:minus	-12.3367	3.09174	5.48624	4.55046	-17.3061	-17.2755	-13.633	1.04167
-2.99083	0.000393	0.00337	0.00145	0.00482	0.00389	0.00544	0.00668	0.012	0.0445
2L:21313220-21313370:plus	-14.6633	5.0367	12.1101	5.29358	-18.6429	-17.051	-13.6514	3.31944
-0.0183486	0.0015	0.0016	0.0000401	0.00398	0.0121	0.00479	0.00671	0.00528	0.0185
2L:21314240-21314390:minus	-23	-2.42202	11.6606	7.29358	-7.89796	9.82653	-6.27523	2.70833	11.3853
0.00498	0.0219	0.0000539	0.00104	0.000678	0.0000148	0.00141	0.00666	0.0000815
2L:21314240-21314390:plus	-4.18367	0.825688	-2.63303	7.63303	-18.4184	-8.60204	-3.92661	-1.06944	2.09174
0.000128	0.00771	0.026	0.000745	0.0101	0.00151	0.000731	0.023	0.00904
2L:21315800-21315950:minus	-30.4388	-3.25688	-2.06422	-0.100917	-18.1531	-16.7143	-14.4495	3.34722
2.84404	0.00923	0.0277	0.022	0.0277	0.00829	0.00419	0.008	0.00522	0.00736
2L:21315800-21315950:plus	-31.5918	6.33028	0.669725	2.06422	-8.97959	9.37755	-21.3394	4.90278	-2.88991
0.0136	0.000904	0.00926	0.0139	0.0023	0.0000363	0.0262	0.00274	0.0433
2L:21344440-21344590:minus	-23.1837	10.3578	-2.02752	1.78899	-9.27551	-8.72449	-23.2936	-3.25
-2.15596	0.00553	0.000111	0.0218	0.0158	0.00284	0.00169	0.0368	0.0411	0.0348
2L:21344440-21344590:plus	-21.7449	7.97248	6.09174	2.94495	-16.3776	-9.27551	-19.633	1.23611	8.88073

0.00236 0.000387 0.00111 0.0092 0.003 0.00244 0.0193 0.0112 0.000468
2L:21397300-21397450:minus -21.5918 -3.6789 14.0734 3.07339 -17.1531 -26.5612 -13.0917 0.902778
-0.550459 0.00209 0.031 6.63e-06 0.00873 0.00364 0.0237 0.0059 0.0125 0.0225
2L:21397300-21397450:plus -23.2959 13.5306 -0.724771 5.65138 -8.89796 -7.79592 -18.0275 1.16667
1.61468 0.00605 0.0000206 0.0146 0.00322 0.00189 0.000723 0.015 0.0115 0.0112
2L:2147360-2147510:minus -32.2143 2.14679 14.1835 4.79817 -8.97959 -18.5714 -8.83486 -0.569444
5.70642 0.0182 0.00476 5.88e-06 0.00453 0.0023 0.0136 0.00248 0.0199 0.00222
2L:2147360-2147510:plus -31.1837 3.64286 7.3211 3.44037 -17.7857 -18.1531 -7.80734 -1.55556 -0.550459
0.0109 0.00159 0.00062 0.00746 0.00676 0.00997 0.00202 0.0264 0.0225
2L:2151860-2152010:minus -23.2653 0.394495 5.08257 0.201835 -8.0102 -8.42857 -11.1101 3.05556 3.88073
0.00593 0.00899 0.00172 0.0255 0.000826 0.00104 0.00382 0.00584 0.00475
2L:2151860-2152010:plus -13.7755 -1.70642 3.57798 1.53211 -8.71429 -16.9388 -11.8349 -2.11111 0.440367
0.00107 0.0178 0.00317 0.0174 0.00159 0.00433 0.00445 0.0307 0.0164
2L:21553480-21553630:minus -22.7041 -3.37615 4.68807 6.37615 -18.4898 -18.2041 -15.7156 2.625
11.8532 0.00395 0.0286 0.00203 0.00192 0.0116 0.0103 0.0103 0.00687 0.0000428
2L:21553480-21553630:plus -32.2959 -1.90826 -1.85321 2.6422 -17.7551 -9.30612 -8.82569 -1.23611 3.90826
0.0186 0.0189 0.0207 0.0107 0.00658 0.00257 0.00248 0.0241 0.00462
2L:21573340-21573490:minus -40.551 8.08257 -1.48624 7.44037 -18.7143 -9.79592 -16.8073 7.04167
3.88073 0.0418 0.000365 0.0185 0.000912 0.0136 0.00377 0.0124 0.000984 0.00475
2L:21573340-21573490:plus -32.7755 5.53211 0.908257 11.1927 20.1735 -0.173469 -19.5413 4.90278
1.6422 0.0234 0.0013 0.00853 0.0000254 5.68e-07 0.000456 0.0191 0.00274 0.0111
2L:21573560-21573710:minus -14.3367 1.6422 1.33028 2.21101 -9.16327 -18.2041 -11.7248 11.0556
3.46789 0.00131 0.00573 0.00736 0.0129 0.00249 0.0103 0.00434 0.0000728 0.00582
2L:21573560-21573710:plus -22.8163 1.15596 -0.0275229 1.89908 -18.1531 -8.5 -22.3486 -1.25 -4.2844
0.00418 0.00684 0.0117 0.0151 0.00829 0.00126 0.0314 0.0242 0.0597
2L:2161300-2161450:minus -20.9286 -1.56881 -2 4.11009 -7.71429 -7.23469 -21.0459 0.902778 9.3945
0.00183 0.0171 0.0216 0.00553 0.000572 0.000557 0.0248 0.0125 0.000358
2L:2161300-2161450:plus -31.2959 -0.00917431 3.99083 5.24771 -18.449 -18.0102 -13.0459 2.13889
1.90826 0.0113 0.0103 0.00269 0.00403 0.0107 0.00812 0.00584 0.0082 0.00974
2L:21623080-21623230:minus -24.1837 -3.29358 6.50459 10.0275 -9.20408 -17.5714 -17.7523 -1.30556
-4.24771 0.00751 0.028 0.000917 0.000156 0.00262 0.00616 0.0144 0.0246 0.0591
2L:21623080-21623230:plus -31.5102 -0.155963 -1.70642 5.89908 -8.60204 -17.5 -19.5688 6.29167
-5.08257 0.013 0.0109 0.0198 0.00272 0.00125 0.00599 0.0191 0.00144 0.0741
2L:21623620-21623770:minus -23.0714 0 -2.85321 3.22936 -27.2653 -25.5612 -12.8257 0.125 1.01835
0.0052 0.0103 0.0277 0.00816 0.0302 0.0206 0.00555 0.0161 0.0142
2L:21623620-21623770:plus -31.9592 4.76147 0.394495 3.17431 -17.449 -9.72449 -23.633 4.01389 -0.715596
0.0167 0.00179 0.0102 0.00842 0.00484 0.00352 0.0388 0.004 0.0241
2L:21624040-21624190:minus -24.5204 0.880734 3.07339 6.76147 -8.89796 -17.1224 -10.4771 3.80556 -1
0.00813 0.00756 0.00386 0.00148 0.00189 0.00499 0.00337 0.00435 0.0261
2L:21624040-21624190:plus -21.5102 2.14679 4.66055 5.6789 -18.4184 -18.2755 -12.0734 2.98611 5.53211
0.00202 0.00476 0.00205 0.00315 0.0101 0.0112 0.00469 0.006 0.00245
2L:21624520-21624670:minus -33.0408 5.02752 0.981651 2.66055 -17.8265 -18.3469 -24.7798 -2.90278
-1.85321 0.0264 0.00161 0.00832 0.0106 0.00691 0.0124 0.0458 0.0377 0.032
2L:21624520-21624670:plus -14.1837 8.77064 1.9633 2.25688 -9.27551 -18.0102 -11.6422 2.84722 1.38532
0.00124 0.000253 0.00586 0.0127 0.00284 0.00812 0.00427 0.00632 0.0123
2L:2162460-2162610:minus -21.3571 3.55046 -0.770642 4.65138 -17.0408 -28.6327 -8.83486 -4.58333
6.17431 0.00194 0.00284 0.0149 0.00471 0.00329 0.066 0.00248 0.0563 0.00171
2L:2162460-2162610:plus -22.5918 1.93578 1.05505 11.1927 -26.1939 -18.5612 -13.3119 5.11111 10.0367
0.00365 0.00514 0.00811 0.0000254 0.0175 0.0132 0.00621 0.0025 0.000146
2L:21628720-21628870:minus -23.2653 0.522936 1.12844 5.57798 -16.0816 -8.72449 -21.7615 5.05556
1.10092 0.00593 0.00859 0.00791 0.0034 0.00294 0.00169 0.0282 0.00256 0.0138
2L:21628720-21628870:plus -24.3673 0.366972 4.54128 1.66055 -17.3367 -18.1224 -19.578 3.36111 9.92661
0.00791 0.00908 0.00215 0.0166 0.00391 0.00988 0.0192 0.00519 0.000193
2L:2162900-2163050:minus -11.8469 -2.15596 -0.385321 7.63303 -27.2347 -17.051 -0.862385 -2.18056

11.8073 0.000279 0.0203 0.0131 0.000745 0.0283 0.00479 0.000344 0.0313 0.0000538
2L:2162900-2163050:plus -23.4796 3.10092 3.41284 5.90826 -18.1122 -27.7857 -17.7248 2.875 9.87156
0.00634 0.00335 0.00338 0.00271 0.00771 0.0466 0.0143 0.00626 0.000217
2L:21629300-21629450:minus -31.6327 -5.44037 5.95413 5.69725 -26.8571 -18.2041 -15.9817 -1.52778
-6.9633 0.0138 0.0495 0.00118 0.00314 0.0198 0.0103 0.0108 0.0262 0.112
2L:21629300-21629450:plus -30.7755 0.651376 1.68807 2.70642 -8.40816 -17.3469 -12.7064 9.52778 5.55046
0.0102 0.00821 0.00648 0.0103 0.00108 0.0058 0.00541 0.000227 0.00241
2L:21629920-21630070:minus -21.3571 -4.55046 0.146789 7.17431 -17.1531 0.387755 -6.07339 11.375
1.95413 0.00194 0.0392 0.011 0.00115 0.00364 0.000217 0.00134 0.0000552 0.00964
2L:21629920-21630070:plus -31.6633 8.49541 0.798165 0.220183 -26.8571 -8.72449 -24.0183 6.05556 -2.98165
0.0141 0.000292 0.00886 0.0253 0.0198 0.00169 0.0412 0.00161 0.0445
2L:2163220-2163370:minus -5 1.3945 3.53211 7.3578 -17.7143 -18.0816 2.87156 5.55556 1.70642
0.000219 0.00627 0.00323 0.000965 0.00627 0.00934 0.000124 0.00204 0.0108
2L:2163220-2163370:plus -32.2653 -1.14679 4.73394 4.22018 -17.7143 -19.3776 -14.0917 3.09722
-0.0733945 0.0184 0.015 0.00199 0.00531 0.00627 0.0195 0.0074 0.00575 0.0188
2L:2164600-2164750:minus -21.6633 6.94495 6.2844 3.69725 -7.71429 -26.8265 -5.73394 4.33333 3.55963
0.00223 0.000669 0.00102 0.00665 0.000572 0.0285 0.00122 0.0035 0.00545
2L:2164600-2164750:plus -32.6224 0.889908 4.89908 2.55963 -27.2653 -28.4082 -15.055 3.84722 10.3486
0.0211 0.00753 0.00185 0.0111 0.0302 0.0615 0.00907 0.00428 0.0000988
2L:21657740-21657890:minus -30.8878 8.00917 3.10092 2.86239 -17.9796 -8.93878 -18.9083 -0.375
3.42202 0.0103 0.00038 0.00382 0.00954 0.00726 0.00201 0.0172 0.0188 0.00597
2L:21657740-21657890:plus -24.0816 0.587156 0.541284 7.34862 -7.97959 -8.60204 -21.7339 -0.777778
-0.0733945 0.00732 0.0084 0.00968 0.000998 0.000799 0.00151 0.0281 0.0211 0.0188
2L:21658640-21658790:minus -13.6735 1.61468 2.14679 -0.816514 -18.1531 -9.65306 -3.25688 -2.69444
-7.12844 0.00103 0.00578 0.00547 0.0346 0.00829 0.0034 0.000608 0.0358 0.116
2L:21658640-21658790:plus -23.7857 15.2202 -3.55046 1.78899 -18.5204 -9.45918 -25.2385 1.90278 -3.99083
0.00679 1.59e-06 0.0336 0.0158 0.0117 0.00279 0.0486 0.00892 0.0556
2L:2166180-2166330:minus -21.7041 -1.61468 0.834862 -1.68807 -27.0102 -9.42857 -15.6422 0.0694444
5.84404 0.00231 0.0173 0.00875 0.0461 0.0237 0.0027 0.0102 0.0164 0.00202
2L:2166180-2166330:plus -13.449 -2.30275 4.88991 9.3211 -18.6837 -9.23469 -15.3211 2.23611 7.6422
0.000914 0.0212 0.00186 0.000391 0.0126 0.00238 0.00956 0.00792 0.000893
2L:2166820-2166970:minus -40.3673 -1.36697 3.75229 1.41284 -17.4592 -19.4184 2.11009 -0.458333
7.68807 0.0397 0.0161 0.00296 0.0183 0.00491 0.0198 0.000159 0.0192 0.000841
2L:2166820-2166970:plus -31.4082 -1.51376 9.88073 2.95413 -26.2653 0.387755 -13.5872 -0.833333
-1.11927 0.0119 0.0168 0.000153 0.00917 0.018 0.000217 0.00661 0.0215 0.027
2L:2167300-2167450:minus -22 1.69725 1.85321 5.55046 -18.3878 -16.9796 -21.3486 3.125 3.97248
0.00291 0.00561 0.0061 0.00345 0.00931 0.0044 0.0262 0.00569 0.00444
2L:2167300-2167450:plus -32.7449 -0.412844 0.266055 5.86239 -18.5204 -8.53061 -21.0734 -2.41667
3.55046 0.0228 0.0119 0.0106 0.00282 0.0117 0.00136 0.0249 0.0333 0.00548
2L:21676700-21676850:minus -30.4082 2.31193 5.26606 2.50459 -27.4592 -9.5 -20.8991 10.7083 -2.89908
0.00908 0.00448 0.00159 0.0114 0.0329 0.00294 0.0241 0.0000964 0.0434
2L:21676700-21676850:plus -24.2245 0.0642202 7.16514 7 -18.7551 -9.5 -11.8807 3.65278 -0.0733945
0.00763 0.0101 0.000671 0.00132 0.0144 0.00294 0.00449 0.00462 0.0188
2L:21677540-21677830:minus -30.7041 14.6881 -2.44037 1.51376 -18.6429 9.53061 -20.0275 3.91667
-1.80734 0.00993 3.75e-06 0.0246 0.0175 0.0121 0.0000316 0.0207 0.00416 0.0316
2L:21677540-21677830:plus -21.102 1.44037 7.23853 3.25688 -8.96939 -9.7551 -10.0917 2.65278 1.00917
0.00187 0.00617 0.000646 0.00804 0.00226 0.00358 0.00313 0.0068 0.0143
2L:21681740-21681890:minus -22.7041 10.3211 0.807339 1.38532 -17.7143 -9.65306 -18.2018 4.36111
2.57798 0.00395 0.000114 0.00884 0.0185 0.00627 0.0034 0.0154 0.00346 0.0078
2L:21681740-21681890:plus -34.5204 10.7798 -1.38532 3.18349 -16.7143 -8.5 -18.1376 5.68056 1.53211
0.0319 0.0000896 0.018 0.00837 0.00319 0.00126 0.0153 0.00193 0.0116
2L:21682000-21682150:minus -24.1531 2.11927 -2.83486 2.62385 -18.7959 -9.42857 -10.8716 -1.23611
-2.05505 0.00744 0.00481 0.0275 0.0108 0.0148 0.0027 0.00364 0.0241 0.0338
2L:21682000-21682150:plus -23.1122 4.42202 1.21101 -0.743119 -27.2653 -18.898 -8.75229 2.15278

9.45872	0.0053	0.00204	0.00768	0.0339	0.0302	0.0152	0.00244	0.00816	0.000337
2L:2168320-2168470:minus	-32.5918	0.669725	2.84404	-0.568807	-18.4184	-19.3776	-1.57798	4.18056
5.40367	0.021	0.00815	0.00421	0.032	0.0101	0.0195	0.000403	0.00373	0.0026
2L:2168320-2168470:plus	-31.7755	4.3945	2.44954	3.61468	-9.20408	-18.051	-11.2936	-4.40278	1.49541
0.0157	0.00207	0.00489	0.0068	0.00262	0.00886	0.00396	0.054	0.0118
2L:21684800-21684950:minus	-32.0408	6.49541	0.431193	3.78899	-26.9286	-16.3469	-19.211	4.54167
-2.00917	0.0174	0.000837	0.01	0.00632	0.0214	0.00416	0.018	0.0032	0.0333
2L:21684800-21684950:plus	-31.7347	-1.3578	0.944954	1.40367	-18.4184	-8.72449	-15.8073	0.541667	7.62385
0.0149	0.016	0.00842	0.0184	0.0101	0.00169	0.0105	0.0141	0.000941
2L:2169180-2169330:minus	-12.1122	-3.20183	2.19266	9.7156	-26.6327	-8.45918	-8.50459	-3.05556	-3.33028
0.000336	0.0273	0.00538	0.000242	0.0188	0.00111	0.00233	0.0392	0.0478
2L:2169180-2169330:plus	-22	3.26606	-0.504587	0.816514	-27.602	-19.3469	-13.4404	2.80556	1.01835
0.00291	0.00315	0.0137	0.0216	0.0364	0.0194	0.00639	0.00642	0.0142
2L:2170160-2170310:minus	-41.5816	8.56881	-0.963303	7.90826	-17.6429	-9.08163	-11.3211	3.04167
1.07339	0.061	0.000281	0.0158	0.000611	0.00562	0.00216	0.00399	0.00587	0.014
2L:2170160-2170310:plus	-21.6939	0.440367	-0.247706	1.09174	-17.3776	-27.3367	-8.74312	5.44444
-0.12844	0.00226	0.00885	0.0126	0.02	0.00419	0.0347	0.00244	0.00215	0.0193
2L:21753000-21753150:minus	-23	1.66055	2.17431	5.90826	-9.20408	-8.65306	-11.6789	-2.72222	-5.00917
0.00498	0.00569	0.00542	0.00271	0.00262	0.00156	0.0043	0.036	0.0728
2L:21753000-21753150:plus	-31.8469	0.963303	1.36697	6.36697	-26.9694	-7.57143	-24.2936	3.19444	1.74312
0.0162	0.00734	0.00726	0.00193	0.023	0.000644	0.0428	0.00554	0.0105
2L:21753700-21753850:minus	-21.8878	-4.00917	4.37615	9.92661	-18.3776	-26.5612	-13.7798	-4.5	5.49541
0.00258	0.0339	0.0023	0.000169	0.00926	0.0237	0.00691	0.0552	0.0025
2L:21753700-21753850:plus	-23.5612	-1.77982	2.50459	-1.54128	-8.96939	-26.7551	-9.37615	9.22222	7.22018
0.00653	0.0182	0.0048	0.0439	0.00226	0.0263	0.00274	0.000278	0.00119
2L:21756900-21757050:minus	-13.102	-0.0917431	5.09174	-1.72477	-27.9694	-16.051	-7.19266	-0.375
4.30275	0.000602	0.0106	0.00171	0.0465	0.0437	0.00408	0.00176	0.0188	0.00388
2L:21756900-21757050:plus	-40.1735	-1.11009	4.05505	-2.22936	-18.4796	-27.6327	-7.84404	3.30556	2.42202
0.0363	0.0148	0.00262	0.0552	0.011	0.043	0.00204	0.0053	0.00816
2L:21757380-21757530:minus	-32.0408	4.54128	3.11009	4.43119	-28.6429	-0.0204082	-13.9633	0.75
3.88073	0.0174	0.00195	0.00381	0.00495	0.0631	0.000411	0.0072	0.0132	0.00475
2L:21757380-21757530:plus	-41.3265	-0.93578	1.08257	8.61468	-8.86735	-8.57143	-5.14679	3.61111	-3.08257
0.0541	0.014	0.00803	0.000552	0.00173	0.00149	0.00104	0.0047	0.0455
2L:21757940-21758090:minus	-32.4796	1.77982	0	2.30275	-28.2653	-9.23469	-17.1009	5	1.95413	0.02
0.00544	0.0116	0.0124	0.0537	0.00238	0.013	0.00263	0.00964
2L:21757940-21758090:plus	-34.1122	10.6422	-1.06422	0.00917431	-9.27551	-8.45918	-17.844	2.18056
-0.642202	0.0312	0.0000961	0.0163	0.0269	0.00284	0.00111	0.0146	0.00808	0.0234
2L:2178000-2178150:minus	-31.8469	3.22936	-1.33028	3.3945	-27.3061	-26.6327	-7	2.20833	7.16514
0.0162	0.0032	0.0177	0.0076	0.0315	0.0251	0.00169	0.008	0.00122
2L:2178000-2178150:plus	-31.8469	-0.688073	3.3211	5.47706	-26.9592	-18.2755	-12.0183	4.83333
4.80734	0.0162	0.013	0.00351	0.0036	0.0215	0.0112	0.00463	0.00283	0.00334
2L:2178740-2178890:minus	-30.3265	3.90816	1.07339	5.85321	-18.4898	-27.7551	-5.34862	0.652778	3.46789
0.0089	0.00102	0.00806	0.00287	0.0116	0.0458	0.0011	0.0136	0.00582
2L:2178740-2178890:plus	-22.5918	-2.73394	-3.75229	2.47706	-25.8571	-17.3469	-23.3394	2.68056	-1.59633
0.00365	0.0239	0.0355	0.0115	0.0166	0.0058	0.0371	0.00673	0.0301
2L:21794740-21794890:minus	-41.5918	5.02752	0.0917431	3.44037	-27.1224	-9.23469	-22.5046	-1.43056
5.0367	0.0623	0.00161	0.0112	0.00746	0.0245	0.00238	0.0322	0.0255	0.00306
2L:21794740-21794890:plus	-3.37755	5.3578	3.45872	3.45872	-27.1224	-18.2755	-8.41284	-2.16667	6.49541
0.0000645	0.0014	0.00332	0.00731	0.0245	0.0112	0.00229	0.0312	0.00154
2L:21795440-21795590:minus	-12.551	3.90816	3.29358	10.7615	-18.449	-18.2755	-13.422	-4.94444
5.0367	0.000444	0.00102	0.00354	0.0000826	0.0107	0.0112	0.00637	0.0609	0.00306
2L:21795440-21795590:plus	-22.1122	0.944954	0.972477	1.36697	-27.4592	-8.45918	-23.8716	1.65278	-1.85321
0.00304	0.00739	0.00835	0.0186	0.0329	0.00111	0.0403	0.00974	0.032
2L:2180780-2180930:minus	-30.102	1.74312	0.302752	2.34862	-18.7143	-9.53061	-8.86239	-0.722222

-0.192661 0.00859 0.00552 0.0105 0.0121 0.0136 0.0031 0.00249 0.0208 0.0198
2L:2180780-2180930:plus -22.5918 1.68807 -3.52294 3.26606 -17.4184 -9.57143 -18.4128 10.4861 -1.63303
0.00365 0.00563 0.0334 0.00802 0.00457 0.00326 0.0159 0.000114 0.0304
2L:21828240-21828390:minus -21.8163 -0.0825688 1.88991 2.90826 -18.6837 -17.2041 -7.55963 -3.40278
7.83486 0.00244 0.0106 0.00602 0.00936 0.0126 0.00507 0.00192 0.0427 0.000712
2L:21828240-21828390:plus -12.551 -3.20183 7.3945 1.92661 -17.449 -8.45918 -9.9633 7.36111 7.42202
0.000444 0.0273 0.000597 0.0148 0.00484 0.00111 0.00306 0.000832 0.00106
2L:21899240-21899390:minus -19.5918 -3.41284 -0.862385 4.22018 -18.4592 -17.8265 -11.7706 -4.20833
3.14679 0.00163 0.0289 0.0153 0.00531 0.0109 0.00699 0.00439 0.0517 0.00666
2L:21899240-21899390:plus -22.0714 -6.16514 11.1009 4.07339 -26.898 -36.0816 -12.367 4.38889 3.81651
0.00299 0.0595 0.000075 0.00562 0.0203 0.0857 0.005 0.00342 0.00482
2L:2192360-2192510:minus -22.8878 7.41284 -0.137615 -1.15596 1.87755 -8.53061 -14.1009 5.51389
2.11927 0.00441 0.000521 0.0121 0.0386 0.0000492 0.00136 0.00742 0.00208 0.00889
2L:2192360-2192510:plus -30.551 5.68807 4.37615 3.30275 -18.1224 -8.16327 -19.211 3.27778 -2.21101
0.00959 0.00122 0.0023 0.00789 0.00782 0.000783 0.018 0.00536 0.0354
2L:220580-220730:minus -32.4388 -0.321101 -0.954128 4.08257 -17.2653 -36.0816 -12.3211 1.93056
9.04587 0.0195 0.0115 0.0157 0.00558 0.00387 0.0857 0.00495 0.00883 0.000423
2L:220580-220730:plus -4.40816 2.54128 6.06422 1.98165 -27.3061 -17.3469 -11.2844 2.69444 1.48624
0.000148 0.00411 0.00112 0.0145 0.0315 0.0058 0.00396 0.00669 0.0118
2L:22114080-22114230:minus -5.12245 5.88073 -0.614679 6.57798 -9.5 -7.45918 -22.2844 4.13889
7.68807 0.000225 0.00112 0.0141 0.00167 0.00293 0.000582 0.031 0.0038 0.000841
2L:22114080-22114230:plus -50.398 0.614679 -3.59633 2.6422 -27.7959 -18.3469 -34.9725 3.875 -1.9633
0.132 0.00832 0.034 0.0107 0.0386 0.0124 0.16 0.00423 0.0329
2L:22114780-22114930:minus -22.9184 -1.15596 10.9358 7.45872 -26.9286 -17.5714 -17.0459 4.77778
-3.94495 0.00447 0.0151 0.0000828 0.000894 0.0214 0.00616 0.0129 0.00289 0.0549
2L:22114780-22114930:plus -22.7347 6.3945 3.23853 7.00917 -27.4592 -9.5 -12.3028 6.86111 -2.68807
0.00403 0.000878 0.00362 0.00126 0.0329 0.00294 0.00493 0.00108 0.041
2L:22121040-22121190:minus -23.1531 13.4592 4.59633 5.01835 -8.86735 -17.051 -21.1468 0.555556
0.963303 0.00542 0.000064 0.0021 0.00438 0.00173 0.00479 0.0253 0.014 0.0145
2L:22121040-22121190:plus -23.1429 13.4592 -2.63303 5.59633 -19.051 -10.0918 -16.2752 0.513889 1.3211
0.00533 0.000064 0.026 0.00331 0.0162 0.00404 0.0114 0.0142 0.0127
2L:22121740-22121890:minus -23.0714 1.77982 -2.05505 0.981651 -26.898 -0.244898 -9.97248 8.68056
7.73394 0.0052 0.00544 0.022 0.0207 0.0203 0.000496 0.00306 0.000391 0.000784
2L:22121740-22121890:plus -31.7755 3.92661 5.26606 -0.0550459 10.3878 0.612245 -5.84404 2.51389
1.94495 0.0157 0.00247 0.00159 0.0272 0.0000174 0.000187 0.00126 0.00716 0.00968
2L:22129840-22129990:minus -33.0714 7.24771 4.47706 2.90826 10.3878 -18.2755 -19.0826 6.58333
3.72477 0.0267 0.00057 0.00221 0.00936 0.0000174 0.0112 0.0177 0.00124 0.00497
2L:22129840-22129990:plus -33.6327 8.81651 3.6055 9.33028 -27.1939 -8.94898 -20.0642 2.76389 -0.220183
0.029 0.000246 0.00314 0.000374 0.0267 0.00203 0.0208 0.00652 0.0201
2L:22130060-22130210:minus -33.6327 -2.2844 -1.25688 1.43119 -17.9796 -8.86735 -13.6514 2.30556
-1.01835 0.029 0.0211 0.0173 0.0181 0.00726 0.00198 0.00671 0.00772 0.0263
2L:22130060-22130210:plus -23.4898 0.862385 -1.68807 2.52294 -27.5612 -17.2041 -5.17431 -1.11111 -0.40367
0.00639 0.00761 0.0197 0.0113 0.0354 0.00507 0.00104 0.0233 0.0213
2L:22131300-22131450:minus -30.7755 0.568807 -2.65138 7.80734 -26.4898 -19.4184 -24.5505 3.61111
1.2844 0.0102 0.00845 0.0261 0.00063 0.0183 0.0198 0.0444 0.0047 0.0129
2L:22131300-22131450:plus -13.7143 0.816514 -4.21101 2.45872 -17.449 -17.3469 -8.44037 0.305556 -1.89908
0.00105 0.00773 0.0402 0.0116 0.00484 0.0058 0.0023 0.0152 0.0323
2L:22133100-22133250:minus -32.6633 1.30275 5.38532 2.63303 -19.051 -17.7857 -19.5138 2.65278
0.40367 0.0216 0.00649 0.00151 0.0107 0.0162 0.0068 0.019 0.0068 0.0165
2L:22133100-22133250:plus -22.8163 -0.440367 -1 -0.669725 -17.6429 -18.0816 -19.578 2.90278
-5.78899 0.00418 0.012 0.016 0.0331 0.00562 0.00934 0.0192 0.00619 0.0848
2L:22134400-22134550:minus -23.8469 -2.22936 3.83486 4.81651 -18.4898 -35.8878 -0.458716 -3.01389
5.44954 0.00685 0.0208 0.00287 0.00451 0.0116 0.0822 0.000315 0.0388 0.00253
2L:22134400-22134550:plus -30.9286 4.25688 3.22018 2.43119 -9.23469 0.0204082 -13.3853 6.51389

5.3945 0.0104 0.00218 0.00365 0.0117 0.00277 0.000377 0.00631 0.00129 0.00263
2L:22134740-22134890:minus -13.1531 2.40367 2.93578 8.88073 -18.4592 -18.8265 -20.5872 5.86111
7.63303 0.00067 0.00433 0.00407 0.000479 0.0109 0.0148 0.0228 0.00177 0.000928
2L:22134740-22134890:plus -22.0714 -0.0825688 2.14679 -0.706422 -8.16327 -17.051 -19.8165 1.08333
-0.761468 0.00299 0.0106 0.00547 0.0335 0.000873 0.00479 0.02 0.0118 0.0244
2L:2214100-2214250:minus -32.3367 13.3878 -0.816514 2.93578 -7.89796 -7.45918 -20.2018 2.04167
-1.3578 0.0187 0.0000869 0.0151 0.00927 0.000678 0.000582 0.0213 0.00849 0.0286
2L:2214100-2214250:plus -31.8878 0.779817 0.256881 0.733945 -9.23469 -18.2755 -22.156 1.72222 7.31193
0.0164 0.00784 0.0106 0.0222 0.00277 0.0112 0.0303 0.0095 0.00112
2L:22148800-22148950:minus -21.6327 7.55046 0.110092 3.61468 -18.7551 -8.16327 -22.7248 5.15278
0.293578 0.00215 0.000485 0.0112 0.0068 0.0144 0.000783 0.0335 0.00245 0.017
2L:22148800-22148950:plus -31.7041 -0.266055 1.95413 -0.110092 -27.2653 -10.0918 -22.0092 1.34722
-3.7156 0.0146 0.0113 0.00588 0.0277 0.0302 0.00404 0.0295 0.0108 0.0518
2L:22149200-22149350:minus -33.1122 0.366972 -2.3945 2.74312 -9.54082 0.204082 -25.0826 2.33333
-0.541284 0.0271 0.00908 0.0242 0.0101 0.00294 0.000296 0.0477 0.00765 0.0223
2L:22149200-22149350:plus -24.2959 -0.825688 4.38532 -1 -8.71429 -8.42857 -19.6055 -0.0833333
0.587156 0.00781 0.0136 0.0023 0.0367 0.00159 0.00104 0.0193 0.0172 0.016
2L:2216280-2216430:minus -22.1837 2.79817 -0.93578 3.78899 -18.6429 -18.051 -13.2752 0.430556 2.99083
0.00315 0.00374 0.0156 0.00632 0.0121 0.00886 0.00616 0.0146 0.00706
2L:2216280-2216430:plus -30.1837 -1.42202 4.43119 -0.633028 -17.6837 -19.0816 -19.0917 3.63889
0.715596 0.00873 0.0164 0.00225 0.0327 0.0058 0.017 0.0177 0.00465 0.0154
2L:22168640-22168790:minus -13.5918 -3.97248 0.449541 3.7156 -17.7143 -18.4184 -9.14679 7.65278
6.95413 0.00099 0.0336 0.00998 0.00655 0.00627 0.0127 0.00263 0.000709 0.00136
2L:22168640-22168790:plus -32.4082 -1.81651 4.01835 2.76147 -9.0102 -27.6327 -12.8899 5.04167 0.247706
0.0191 0.0184 0.00266 0.01 0.00236 0.043 0.00564 0.00258 0.0172
2L:22169100-22169250:minus -31.7041 2.99083 9.14679 4.05505 -26.8163 -17.9796 -23.7156 2.94444
-3.50459 0.0146 0.00349 0.000233 0.00566 0.0195 0.00764 0.0393 0.00609 0.0497
2L:22169100-22169250:plus -39.4796 5.41284 3.46789 2.83486 -9.19388 -8.02041 -19.7431 2.86111 -0.504587
0.0334 0.00137 0.00331 0.0097 0.00253 0.000761 0.0197 0.00629 0.0221
2L:2217240-2217390:minus -32.7755 13.4592 7.53211 5.75229 -26.9286 -8.20408 -26.6881 -1.45833 -0.12844
0.0234 0.000064 0.000558 0.00296 0.0214 0.000796 0.0582 0.0257 0.0193
2L:2217240-2217390:plus -31.7041 -1.44954 2.00917 2.52294 -18.7143 -8.65306 -13.9817 0.333333 -2.48624
0.0146 0.0165 0.00576 0.0113 0.0136 0.00156 0.00722 0.0151 0.0387
2L:22180140-22180290:minus -23.2245 2.88073 3.42202 2.24771 -18.1224 -8.42857 -4.18349 6.79167
5.13761 0.00576 0.00363 0.00337 0.0127 0.00782 0.00104 0.000786 0.00112 0.00298
2L:22180140-22180290:plus -31.1122 0.183486 -0.0550459 0.605505 -27.2653 -25.8265 -3.97248 6.43056
4.87156 0.0107 0.00968 0.0118 0.023 0.0302 0.0212 0.00074 0.00134 0.00325
2L:22182220-22182370:minus -24.2347 6.14679 -1.13761 1.16514 -27.2347 -8.5 -20.3028
2.59722 -2.55046 0.00763 0.00099 0.0167 0.0197 0.0283 0.00126 0.0217 0.00694 0.0394
2L:22182220-22182370:plus -32.0408 2.36697 0.211009 6.51376 -17.3469 -8.79592 -13.0734
0.972222 -1.3578 0.0174 0.00439 0.0108 0.00175 0.00394 0.0019 0.00588 0.0123 0.0286
2L:22182540-22182690:minus -14.7857 4.14679 -0.678899 3.88991 -18.9796 -19.3469 -18.9725
2.5 4.0367 0.00158 0.00228 0.0144 0.00608 0.0155 0.0194 0.0174 0.0072 0.00424
2L:22182540-22182690:plus -32.0408 0.504587 0.211009 6.51376 -17.3469 0.612245
-22.3303 -0.333333 -3.29358 0.0174 0.00865 0.0108 0.00175 0.00394 0.000187 0.0313 0.0185
0.0474
2L:22203560-22203710:minus -32.5816 4.09174 7.65138 1.40367 -18.9388 -18.051 -15.2844 2.72222
7.58716 0.0208 0.00232 0.000526 0.0184 0.0151 0.00886 0.00949 0.00663 0.000954
2L:22203560-22203710:plus -23 0.752294 4.07339 7.17431 -27.0816 -9.60204 -22.8532
0.777778 -6.33945 0.00498 0.00791 0.0026 0.00115 0.0243 0.00334 0.0342 0.0131 0.0966
2L:2220760-2220910:minus -32.0714 6.92661 6.58716 2.70642 -17.3776 -9.79592 -7.22018
0.361111 1.95413 0.0176 0.000674 0.00088 0.0103 0.00419 0.00377 0.00178 0.0149 0.00964
2L:2220760-2220910:plus -30.4388 -3.40367 -2.70642 -0.449541 10.9184 9.30612 -12.9541
6.06944 -0.697248 0.00923 0.0288 0.0265 0.0307 4.19e-06 0.0000398 0.00572 0.0016 0.0239

2L:2221640-2221790:minus -4.85714 0.972477 -2.83486 2.7156 -8.86735 -16.9796 -8.19266 6.90278 9.88073 0.000202 0.00731 0.0275 0.0102 0.00173 0.0044 0.00219 0.00106 0.000206
2L:2221640-2221790:plus -31.8776 2.25688 4.73394 5.58716 -18.0816 -9.45918 -8.97248 -1.45833 11.8532 0.0163 0.00457 0.00199 0.00334 0.00759 0.00279 0.00254 0.0257 0.0000428
2L:2221940-2222090:minus -32.1122 2.57798 5.30275 5.21101 -18.0408 -9.0102 -19.8073 1.08333 3.05505 0.0178 0.00406 0.00157 0.0042 0.00735 0.00208 0.0199 0.0118 0.00692
2L:2221940-2222090:plus -22.8163 1.06422 -0.165138 8.98165 -26.8571 -17.2347 -19.6147 -1.51389 2.75229 0.00418 0.00707 0.0122 0.000461 0.0198 0.00511 0.0193 0.0261 0.00748
2L:222200-222350:minus -31.5918 1.92661 -0.504587 9.49541 -7.60204 -17.4898 -14.7982 -5.5 -3 0.0136 0.00516 0.0137 0.000308 0.000466 0.0059 0.00861 0.0686 0.0447
2L:222200-222350:plus -13.2551 -0.0550459 4.77064 -1.15596 -26.9694 -36.1837 -7.3578 2.68056 9.45872 0.000751 0.0105 0.00196 0.0386 0.023 0.0914 0.00183 0.00673 0.000337
2L:22239880-22240030:minus -3.7449 3.97959 1.98165 5.3945 -19.051 9.30612 -13.8257 5.13889 7.58716 0.000104 0.000669 0.00582 0.00379 0.0162 0.0000398 0.00698 0.00247 0.000954
2L:22239880-22240030:plus -24.0306 6.6055 1.05505 3.95413 -9.20408 -17.2755 -21.8073 1.22222 0.633028 0.00723 0.000793 0.00811 0.00593 0.00262 0.00544 0.0285 0.0113 0.0158
2L:22240680-22240830:minus -21.8878 -0.541284 0.541284 4.37615 -18.1837 -18.7143 -4.66055 0.638889 8.11009 0.00258 0.0124 0.00968 0.00504 0.00835 0.014 0.000901 0.0137 0.000597
2L:22240680-22240830:plus -31.8163 0 6.36697 2.45872 -18.3776 -17.9796 -13.6239 1.625 7.6789 0.016 0.0103 0.000979 0.0116 0.00926 0.00764 0.00667 0.00983 0.000866
2L:22246680-22246830:minus -24.1837 0.688073 -1.66055 2.93578 -17.3776 -16.9796 -14.0642 -1.41667 -8.16514 0.00751 0.0081 0.0195 0.00927 0.00419 0.0044 0.00736 0.0254 0.14
2L:22246680-22246830:plus -32.7755 -2.69725 4.22936 2.3578 -18.7245 -17.2755 -17.4862 2.04167 0.12844 0.0234 0.0237 0.00244 0.0121 0.0136 0.00544 0.0138 0.00849 0.0179
2L:22246980-22247130:minus -31.4082 -2.23853 3.44037 7.37615 -18.6735 -19.1224 -16.9174 3.625 -0.12844 0.0119 0.0208 0.00335 0.000951 0.0123 0.0175 0.0126 0.00468 0.0193
2L:22246980-22247130:plus 5.88776 4.55963 4.23853 6.21101 -18.3469 -19.2347 -7.59633 -1.11111 1.73394 7.39e-06 0.00194 0.00243 0.00213 0.00894 0.018 0.00193 0.0233 0.0106
2L:22247800-22247950:minus -23.2245 1.29358 0.678899 3.81651 -8.93878 -17.051 -14.1468 -1.95833 1.58716 0.00576 0.00651 0.00923 0.00626 0.00216 0.00479 0.00749 0.0295 0.0113
2L:22247800-22247950:plus -31.4796 0.568807 2.12844 1.84404 0.846939 -27.1224 -28.1835 -0.402778 5.3945 0.0128 0.00845 0.00551 0.0154 0.000197 0.0325 0.0705 0.0189 0.00263
2L:22249240-22249390:minus -42.551 5.6789 -2.33945 -0.568807 -19.3163 0.530612 -7.01835 2.80556 0.816514 0.0803 0.00122 0.0239 0.032 0.0165 0.000203 0.00169 0.00642 0.015
2L:22249240-22249390:plus -23.9286 1.16514 -2.13761 1.05505 0.571429 0.244898 -17.6422 3.31944 -3.99083 0.00701 0.00682 0.0225 0.0204 0.0003 0.000285 0.0142 0.00528 0.0556
2L:22249760-22249910:minus -32.6224 -0.899083 -3.77064 1.42202 -17.1122 -8.68367 -25.3211 9.125 -2.94495 0.0211 0.0139 0.0357 0.0182 0.00341 0.00157 0.0492 0.000296 0.0441
2L:22249760-22249910:plus -30.5204 4.31193 -0.155963 0.990826 10.6531 0.244898 -14.9817 8.58333 1.6789 0.00951 0.00213 0.0122 0.0206 0.0000102 0.000285 0.00894 0.000415 0.011
2L:22250040-22250190:minus -23.0816 4.3945 -2.20183 4.33945 -17.1122 -18.3469 -25.3211 -0.0277778 -4.20183 0.00522 0.00207 0.0229 0.00509 0.00341 0.0124 0.0492 0.0169 0.0584
2L:22250040-22250190:plus -31.9592 3.44037 -3.75229 2.77064 -26.4082 -27.5306 -14.2844 -0.986111 7.68807 0.0167 0.00296 0.0355 0.01 0.0182 0.0374 0.00772 0.0225 0.000841
2L:22261160-22261310:minus -31.2245 11.156 4.6422 5.36697 -8.96939 -18.5612 -14.2936 -2.45833 2.93578 0.0111 0.0000747 0.00206 0.00386 0.00226 0.0132 0.00774 0.0337 0.00717
2L:22261160-22261310:plus -13 0.926606 -1.82569 3.75229 -26.9286 9.57143 -20.2294 -4.43056 -1.3211 0.000563 0.00743 0.0205 0.00649 0.0214 0.0000274 0.0214 0.0544 0.0284
2L:2226360-2226510:minus -4.96939 4.51376 -1.22018 2.29358 -8.7449 -9.72449 2.81651 2.61111 -3.52294 0.000218 0.00198 0.0171 0.0124 0.00161 0.00352 0.000126 0.00691 0.0499
2L:2226360-2226510:plus -31.7347 1.51376 -2.16514 3.59633 -26.9694 -17.8571 -13.7064 -1.08333 1.69725 0.0149 0.006 0.0227 0.00693 0.023 0.00719 0.00679 0.0231 0.0108
2L:2226680-2226830:minus -30.7041 -0.495413 1.47706 7.73394 -19.051 -8.45918 -12.8899 0.555556 0.963303 0.00993 0.0122 0.00698 0.000669 0.0162 0.00111 0.00564 0.014 0.0145

2L:2226680-2226830:plus -24.0408 4.02752 0.192661 6.11009 -28.0408 -8.23469 -13.5505
7.55556 5.84404 0.00726 0.00238 0.0109 0.00233 0.0455 0.000875 0.00656 0.000748 0.00202
2L:2228240-2228390:minus -21.9184 3.11009 -1.55046 2.55963 -18.6429 -18.051 -17.3578
10.7639 -0.59633 0.00261 0.00334 0.0189 0.0111 0.0121 0.00886 0.0136 0.0000924 0.0229
2L:2228240-2228390:plus -30.8163 -4.16514 0.743119 -1.0367 -8.97959 -27.2653 -13.7248
4.73611 7.82569 0.0103 0.0354 0.00903 0.0371 0.0023 0.0333 0.00682 0.00295 0.000728
2L:22282660-22282810:minus -14.7449 9.18349 11.367 7.55963 -27.898 -0.0204082 -12.6422 1.09722
-3.27523 0.00157 0.000203 0.0000636 0.000807 0.04 0.000411 0.00533 0.0118 0.0473
2L:22282660-22282810:plus -14.898 -2 3.11927 5.51376 -26.9694 -8.79592 -14.0917 8
5.44037 0.0016 0.0194 0.00379 0.00348 0.023 0.0019 0.0074 0.000583 0.00259
2L:2228720-2228870:minus -31.7755 4.25688 -2.89908 -0.00917431 -26.9694 -7.79592
-16.7064 2.48611 0.779817 0.0157 0.00218 0.028 0.027 0.023 0.000723 0.0122 0.00723 0.0151
2L:2228720-2228870:plus -24.449 -0.0366972 3.3945 5.06422 -19.0102 -7.72449 -14.2752 3.97222
-3.55963 0.00802 0.0104 0.00341 0.00433 0.0157 0.00068 0.00771 0.00407 0.0503
2L:2229180-2229330:minus -33.1122 -0.550459 3.21101 2.61468 -26.898 -9.7551 -15.0826 1.27778
-0.293578 0.0271 0.0124 0.00366 0.0109 0.0203 0.00358 0.00912 0.0111 0.0207
2L:2229180-2229330:plus -13.4796 2.68807 -1.6422 2.62385 -18.4592 -27.4082 -6.70642 4.34722
1.38532 0.000935 0.0039 0.0194 0.0108 0.0109 0.0356 0.00157 0.00348 0.0123
2L:22354140-22354290:minus -24.449 -1.43119 3.26606 3.6055 -18.4184 -9.5 -23.6606 -0.402778
-5.22936 0.00802 0.0164 0.00358 0.00686 0.0101 0.00294 0.039 0.0189 0.0764
2L:22354140-22354290:plus -22.9694 5.37615 7.26606 2.93578 -27.2347 -18.8571 -20.6697
6.01389 0.110092 0.0048 0.00139 0.000638 0.00927 0.0283 0.0151 0.0232 0.00165 0.018
2L:22354820-22354970:minus -32 2.69725 -0.458716 1.61468 -19.0102 -9.82653 -18.3394
-0.0972222 3.46789 0.0171 0.00389 0.0135 0.0169 0.0157 0.00381 0.0157 0.0172 0.00582
2L:22354820-22354970:plus -22.2245 4.55046 -3.99083 2.93578 -26 -25.051 -9.22018 3.61111
3.09174 0.00325 0.00195 0.0379 0.00927 0.0171 0.0204 0.00266 0.0047 0.00678
2L:2236820-2236970:minus -32.2653 8.72477 0.944954 1.82569 -27.1939 -18.051 -17.6147
0.0972222 7.22018 0.0184 0.000259 0.00842 0.0156 0.0267 0.00886 0.0141 0.0162 0.00119
2L:2236820-2236970:plus -22 0.0825688 11.3119 2.80734 -18.0918 -18.2755 -20.8257 4.40278
-0.229358 0.00291 0.01 0.0000659 0.00985 0.00761 0.0112 0.0238 0.0034 0.0202
2L:2242260-2242410:minus -22.398 7.6789 0.889908 3.98165 -18.2245 -26.5612 -3.47706
-10.9167 11.8073 0.00344 0.000454 0.00859 0.00582 0.00877 0.0237 0.000645 0.176 0.0000538
2L:2242260-2242410:plus -29.5102 6.86239 9.29358 10.5872 -17.4898 -17.7857 -2.50459 -7.16667
3.88073 0.00845 0.000697 0.000214 0.000115 0.0053 0.0068 0.000501 0.0955 0.00475
2L:22426340-22426490:minus -23.8163 -2.3578 2.59633 -0.165138 -8.67347 -18.2041 -7.48624
-0.125 -6.42202 0.00682 0.0215 0.00463 0.0282 0.00154 0.0103 0.00189 0.0174 0.0985
2L:22426340-22426490:plus -33.5612 -0.376147 0.981651 0.752294 -27.3367 -0.244898
-10.1468 3.70833 4.19266 0.0288 0.0117 0.00832 0.0221 0.0317 0.000496 0.00316 0.00452 0.00401
2L:22429460-22429610:minus -39.9592 1.6055 3.37615 2.3578 -7.67347 9.23469 -20.211 0.444444
-0.917431 0.0348 0.0058 0.00343 0.0121 0.00055 0.0000427 0.0214 0.0145 0.0256
2L:22429460-22429610:plus -32 -1.84404 4.09174 2.14679 -26.2653 -0.244898 -15.9083
7.80556 5.6055 0.0171 0.0186 0.00258 0.0133 0.018 0.000496 0.0107 0.000651 0.00232
2L:22430900-22431050:minus -22 2.58716 -3.95413 7.78899 -17.7143 -8.82653 -11.055 4.61111
3.61468 0.00291 0.00404 0.0375 0.000642 0.00627 0.00191 0.00377 0.00311 0.00526
2L:22430900-22431050:plus -40.551 0.0917431 -4.29358 0.486239 -6.60204 -26.8571
-20.0092 5.54167 -2.61468 0.0418 0.00999 0.0411 0.0237 0.000355 0.0288 0.0206 0.00205 0.0401
2L:224380-224530:minus -4.18367 7.92661 0.93578 5.22936 -18.3878 -8.42857 -10.5596 0.847222
-0.229358 0.000128 0.000398 0.00845 0.0041 0.00931 0.00104 0.00342 0.0128 0.0202
2L:224380-224530:plus -31.6939 4.22936 6.3945 6.73394 -17.8163 -7.72449 -22.1009 -1.45833
-4.61468 0.0142 0.0022 0.000966 0.00152 0.00682 0.00068 0.03 0.0257 0.0658
2L:22441400-22441550:minus -40.4796 3.19266 9.78899 5.08257 -17.1531 -8.7551 -22.1284 2.23611
4.80734 0.0407 0.00324 0.000161 0.00429 0.00364 0.00172 0.0302 0.00792 0.00334
2L:22441400-22441550:plus -14.6327 -0.87156 15.0183 5.49541 -9.23469 -19.3469 -10.367
2.375 0.963303 0.0015 0.0138 1.42e-06 0.00355 0.00277 0.0194 0.0033 0.00753 0.0145

2L:22442980-22443130:minus -40.7449 -2.11927 1.93578 3.44954 1.65306 -8.86735 -11.9174 4.5
1.48624 0.0431 0.0201 0.00592 0.00738 0.0000586 0.00198 0.00453 0.00326 0.0118
2L:22442980-22443130:plus -31.7755 0.321101 -1.77064 -1.24771 -18.449 -18.9796
-7.51376 0.486111 3.56881 0.0157 0.00922 0.0202 0.0398 0.0107 0.0155 0.0019 0.0144 0.00543
2L:22470020-22470170:minus -31.4796 13.4592 0.779817 2.55046 -26.8571 -8.68367 -17.6422
-0.444444 0.229358 0.0128 0.000064 0.00892 0.0112 0.0198 0.00157 0.0142 0.0192 0.0173
2L:22470020-22470170:plus -14.5204 -1.33945 -2.30275 3.49541 -8.27551 -19.6429
-11.3578 2.02778 4.31193 0.00145 0.0159 0.0236 0.00716 0.000951 0.0204 0.00402 0.00853 0.00386
2L:22471740-22471890:minus -41.6633 3.81651 2.75229 5.46789 -27.5306 -17.2755 -14.422 5.98611
1.69725 0.0636 0.00258 0.00436 0.00362 0.0351 0.00544 0.00796 0.00167 0.0108
2L:22471740-22471890:plus -23.2959 -1.16514 8.09174 -0.917431 -17.9796 -16.9796
-12.3303 4.52778 0.779817 0.00605 0.0151 0.000416 0.0358 0.00726 0.0044 0.00496 0.00322 0.0151
2L:225020-225170:minus -31.551 5.0367 4.38532 7 -8.56122 -28.4082 -11.2018 0.958333
3.6789 0.0134 0.0016 0.0023 0.00132 0.00112 0.0615 0.00389 0.0123 0.00506
2L:225020-225170:plus -12.0714 0.522936 3.6422 6.31193 -17.7551 -7.5 -19.6239 5.98611
-4.61468 0.000318 0.00859 0.00309 0.00199 0.00658 0.000614 0.0193 0.00167 0.0658
2L:22690860-22691010:minus -30.7755 -1.27523 3.78899 3.31193 -18.4184 -18.1224 -21.8991
0.194444 -0.623853 0.0102 0.0156 0.00292 0.00786 0.0101 0.00988 0.029 0.0157 0.0232
2L:22690860-22691010:plus -33.8571 -0.917431 2.26606 4.91743 -16.7143 -18.9796 -18.0734
5.63889 1.06422 0.0301 0.014 0.00524 0.00447 0.00319 0.0155 0.0151 0.00196 0.014
2L:22736260-22736410:minus -33.8163 4.53211 -3.54128 0.247706 -17.4592 -17.8571
-23.211 2.20833 5.25688 0.0299 0.00196 0.0335 0.0251 0.00491 0.00719 0.0363 0.008 0.0028
2L:22736260-22736410:plus -22.0408 6.97248 0.0458716 2.33945 -27.602 0.163265 -14.7339
4.76389 7.72477 0.00296 0.000659 0.0114 0.0122 0.0364 0.000307 0.0085 0.00291 0.000798
2L:22736940-22737090:minus -42.7755 5.44954 0.981651 -0.623853 -28.4592 1.09184 -24.6422
5.47222 -0.559633 0.0854 0.00135 0.00832 0.0326 0.0568 0.000091 0.045 0.00212 0.0226
2L:22736940-22737090:plus -33.2245 -0.862385 2.36697 3.97248 -18.7449 -8.65306 -19.6697
3.22222 1.00917 0.0276 0.0137 0.00505 0.00587 0.0138 0.00156 0.0195 0.00548 0.0143
2L:22813820-22813970:minus -21.9592 3.90816 2.55963 9.76147 -26.1939 -18.0102 -13.2018
-1.18056 2.47706 0.00279 0.00102 0.0047 0.000212 0.0175 0.00812 0.00605 0.0238 0.00801
2L:22813820-22813970:plus -30.898 0.559633 0.862385 1.24771 -18.1224 -8.5 -19.6972
5.11111 -1.15596 0.0103 0.00848 0.00867 0.0192 0.00782 0.00126 0.0196 0.0025 0.0273
2L:22817580-22817730:minus -23.449 -0.192661 2.79817 4.9633 -27.7449 -18.2347 -20.6606
3.77778 -0.550459 0.00631 0.011 0.00429 0.0044 0.0383 0.0104 0.0231 0.0044 0.0225
2L:22817580-22817730:plus -33.0408 -0.614679 3.83486 2.58716 -18.1122 -19.0816 -7.97248
4.56944 -2.36697 0.0264 0.0127 0.00287 0.011 0.00771 0.017 0.00209 0.00317 0.0371
2L:22961080-22961230:minus -33.0408 6.16514 -2.9633 3.76147 -19.0204 0.755102 -23.5046 0.5
0.0458716 0.0264 0.00098 0.0285 0.00645 0.0158 0.000175 0.0381 0.0143 0.0182
2L:22961080-22961230:plus -22.1122 3.05505 11.8899 3.70642 -17.449 -18.0816 -18.7798 0.0416667
5.01835 0.00304 0.00341 0.0000462 0.0066 0.00484 0.00934 0.0169 0.0165 0.00309
2L:22961720-22961870:minus -13.7449 7.12844 5.72477 2.84404 -8.60204 -19.051 -9.10092 6.375
-0.0183486 0.00107 0.000607 0.0013 0.00961 0.00125 0.0165 0.00261 0.00138 0.0185
2L:22961720-22961870:plus -32.7041 5.82569 1.3211 3.93578 -18.7143 -19.3469 -13.9174 3.25
1.79817 0.0222 0.00114 0.00738 0.00597 0.0136 0.0194 0.00712 0.00542 0.0102
2L:22967860-22968010:minus -15 13.4592 -3.92661 3.3211 -18.7143 -0.0510204 0.87156 1.73611
1.95413 0.00162 0.000064 0.0372 0.00782 0.0136 0.000429 0.000228 0.00946 0.00964
2L:22967860-22968010:plus -21.898 7.30275 -0.46789 6.21101 -17.7857 -8.42857 -20.0459 6.86111
0.963303 0.00259 0.000554 0.0135 0.00213 0.00676 0.00104 0.0208 0.00108 0.0145
2L:22968240-22968390:minus -32 8.2844 -2.68807 2.98165 -27.4592 -9.45918 -26.211 -4.70833 -7.81651
0.0171 0.000327 0.0264 0.00905 0.0329 0.00279 0.0549 0.0579 0.131
2L:22968240-22968390:plus -41.6224 1.49541 2.40367 -0.238532 -17.9796 -0.540816 -30.4771 3.40278
-0.0183486 0.0628 0.00604 0.00498 0.0288 0.00726 0.00054 0.0972 0.00511 0.0185
2L:2357360-2357510:minus -22.3265 5.10092 3.85321 5.11927 -27.5306 -8.45918 -8.54128 1.30556 2.89908
0.00337 0.00156 0.00284 0.00427 0.0351 0.00111 0.00234 0.011 0.00722

2L:2357360-2357510:plus -13.6327 3.16514 14.8257 7.29358 -27.2653 -17.051 -24.3486 6.86111 0.229358
0.00101 0.00327 2.55e-06 0.00104 0.0302 0.00479 0.0432 0.00108 0.0173
2L:2362460-2362610:minus -31.5918 7.37615 3.88073 7.00917 -26.6327 -8.53061 -12.6514 2.31944 0.743119
0.0136 0.000532 0.00281 0.00126 0.0188 0.00136 0.00534 0.00769 0.0153
2L:2362460-2362610:plus -32.0408 4.86239 0.275229 2.84404 -17.1531 1.02041 -15.5229 -0.347222
-6.52294 0.0174 0.00172 0.0106 0.00961 0.00364 0.000118 0.00994 0.0186 0.101
2L:2362780-2362930:minus -34.0408 -0.825688 -0.880734 6 -9.0102 -9.0102 -2.26606 6.25 3.42202
0.0309 0.0136 0.0154 0.00256 0.00236 0.00208 0.000473 0.00147 0.00597
2L:2362780-2362930:plus -22.8163 2.31193 4.27523 0.522936 -18.6837 -18.2755 -11.1651 4.72222 10.0367
0.00418 0.00448 0.0024 0.0234 0.0126 0.0112 0.00386 0.00296 0.000146
2L:2363440-2363590:minus -32.0306 -0.422018 3.01835 3.6422 -18.6837 -8.94898 -12.3211 1.76389
4.51376 0.0172 0.0119 0.00394 0.00669 0.0126 0.00203 0.00495 0.00937 0.0036
2L:2363440-2363590:plus -13.6735 0.587156 -2.19266 1.24771 -18.2245 -9.09184 -16.4404 7.04167 1.55046
0.00103 0.0084 0.0229 0.0192 0.00877 0.00219 0.0117 0.000984 0.0115
2L:2364020-2364170:minus -23.0408 2.11927 1.36697 3.3578 -9.16327 -9.37755 -13.4679 3.01389 -1.11009
0.00512 0.00481 0.00726 0.00767 0.00249 0.00265 0.00644 0.00593 0.0269
2L:2364020-2364170:plus -22.7653 2.61468 -3.11009 -0.550459 -17.4082 -28.4082 -3.29358 7.59722
2.93578 0.00405 0.004 0.0297 0.0317 0.00421 0.0615 0.000614 0.000731 0.00717
2L:2364320-2364470:minus -30.2143 -3.92661 2.11009 2.40367 -8.67347 -28.1939 -0.770642 -5.95833
9.34862 0.0088 0.0332 0.00554 0.0119 0.00154 0.0591 0.000337 0.0755 0.000371
2L:2364320-2364470:plus -21.8878 0.504587 3.52294 0.229358 -8.85714 -27.898 -13.9908 -4.08333 4.6789
0.00258 0.00865 0.00324 0.0252 0.00165 0.0537 0.00724 0.0502 0.00347
2L:2365200-2365350:minus -31.449 3.57143 -0.917431 4.7156 -17.449 -7.5 -23.8624 5.34722 3.93578
0.0123 0.00175 0.0156 0.00464 0.00484 0.000614 0.0402 0.00225 0.00456
2L:2365200-2365350:plus -39.9898 0.559633 4.31193 -0.183486 10.9184 -18.051 -12.211 -0.0694444
3.88991 0.0351 0.00848 0.00236 0.0283 4.19e-06 0.00886 0.00483 0.0171 0.00467
2L:2373800-2373950:minus -24.8571 2.21101 0.59633 7.17431 -26.9286 -28.3367 -6.59633 1.77778 -4.82569
0.00831 0.00464 0.00949 0.00115 0.0214 0.0606 0.00153 0.00932 0.0698
2L:2373800-2373950:plus -23.449 2.46789 3.87156 5.93578 -17.7143 -9.5 -15.633 2.22222 -2.65138
0.00631 0.00422 0.00282 0.00264 0.00627 0.00294 0.0101 0.00796 0.0406
2L:2374480-2374630:minus -23.3776 5.83486 4.31193 1.55963 -26.5918 -18.051 3.2844 9.36111 1.2844
0.00619 0.00114 0.00236 0.0173 0.0186 0.00886 0.000107 0.000254 0.0129
2L:2374480-2374630:plus -14.5612 2.21101 14.8257 7.29358 -17.4184 -9.68367 -16.8165 3.44444 5.58716
0.00147 0.00464 2.55e-06 0.00104 0.00457 0.00342 0.0124 0.00502 0.00237
2L:2375780-2375930:minus -23.5612 -0.40367 5.54128 10.5872 -8.60204 0.459184 -6.24771 3.54167 -4.92661
0.00653 0.0118 0.00141 0.000115 0.00125 0.000213 0.0014 0.00483 0.0717
2L:2375780-2375930:plus -32.551 3.91743 3.00917 4.17431 -19.2755 -19.3776 -9.70642 2.34722 0.302752
0.0207 0.00248 0.00396 0.00537 0.0164 0.0195 0.00291 0.00761 0.0169
2L:2376220-2376370:minus -12.4796 -0.33945 -0.12844 2.72477 -8.63265 -37.1837 -2.45872 -2.36111 -0.972477
0.000433 0.0116 0.0121 0.0102 0.0013 0.14 0.000496 0.0328 0.0259
2L:2376220-2376370:plus -4.71429 4.18349 3.58716 0.770642 -25.9694 -19.6429 -4.2844 -1.95833 6.54128
0.000187 0.00224 0.00316 0.0219 0.017 0.0204 0.000809 0.0295 0.00151
2L:2376800-2376950:minus -34.1122 6.19266 4.88073 5.31193 -18.3776 -27.1939 -21.3486 2.69444 -2.45872
0.0312 0.000967 0.00187 0.00393 0.00926 0.0327 0.0262 0.00669 0.0383
2L:2376800-2376950:plus -21.6327 1.81651 -0.550459 2.0367 -28.0102 -16.3469 -20.8257 5.45833
-0.440367 0.00215 0.00537 0.0139 0.0141 0.0449 0.00416 0.0238 0.00214 0.0216
2L:2383060-2383210:minus -20.8571 0.715596 3.73394 2.77982 -17.9388 -17.8265 -20.7982 2.06944 5.81651
0.00178 0.00802 0.00299 0.00997 0.00715 0.00699 0.0237 0.00841 0.00204
2L:2383060-2383210:plus -32.2245 0.981651 -0.550459 4.40367 -17.9796 -8.72449 -24 6.40278 -4.41284
0.0183 0.00729 0.0139 0.00497 0.00726 0.00169 0.041 0.00136 0.0624
2L:244460-244610:minus -30.1429 -5.87156 7.19266 6.69725 -18.7143 -0.204082 -12.3486 2.48611
12.2752 0.00866 0.0553 0.000661 0.00156 0.0136 0.000463 0.00498 0.00723 0.0000141
2L:244460-244610:plus -39.8776 -5.05505 -0.0366972 2.22936 -17.1122 -8.16327 -16.4404 -0.486111
-9.49541 0.0341 0.0448 0.0117 0.0128 0.00341 0.000783 0.0117 0.0194 0.179

2L:2450940-2451090:minus -31.7857 1.29358 1.88073 4.95413 -17.4082 -8.42857 -17.3394 -4.08333 11.8532
0.0157 0.00651 0.00604 0.00442 0.00421 0.00104 0.0135 0.0502 0.0000428
2L:2450940-2451090:plus -32.4796 3.61224 0.614679 2.68807 -28.1939 -19.051 -20.5138 4.20833 3.83486
0.02 0.00167 0.00943 0.0104 0.0482 0.0165 0.0225 0.00369 0.00481
2L:246780-246930:minus -21.7347 3.7551 -3.18349 3.41284 -17.0816 -9.57143 -19.9541 -3.04167 0.229358
0.00235 0.00122 0.0304 0.00756 0.00334 0.00326 0.0204 0.0391 0.0173
2L:246780-246930:plus -12.4082 -0.798165 5.6055 3.46789 -17.2653 -26.6735 1.72477 6.40278 7.22018
0.000414 0.0134 0.00137 0.00726 0.00387 0.0256 0.000179 0.00136 0.00119
2L:247580-247730:minus -14.1429 -2.80734 -2.10092 3.13761 -18.6429 -8.72449 -6.19266 0.0972222
-4.19266 0.0012 0.0244 0.0223 0.00854 0.0121 0.00169 0.00138 0.0162 0.0583
2L:247580-247730:plus -24.0816 -0.963303 1.90826 6.08257 -17.3469 0.387755 -24.211 1.73611 0.0825688
0.00732 0.0142 0.00598 0.00236 0.00394 0.000217 0.0423 0.00946 0.0181
2L:248260-248410:minus -12.3367 0.385321 8.30275 6.05505 -17.9796 -9.42857 -20.5138 -0.347222
6.91743 0.000393 0.00902 0.000372 0.0024 0.00726 0.0027 0.0225 0.0186 0.00136
2L:248260-248410:plus -31.6633 1.44037 -1.12844 1.92661 -7.56122 -17.5714 -12.7615 2.01389 9.66055
0.0141 0.00617 0.0166 0.0148 0.000419 0.00616 0.00547 0.00858 0.000268
2L:248660-248810:minus -13.2143 3.7156 0.688073 2.83486 -27.3061 -26.6327 -15.7523 5.19444 0.0275229
0.000714 0.00267 0.0092 0.0097 0.0315 0.0251 0.0104 0.00241 0.0183
2L:248660-248810:plus -32.2551 0.174312 2.57798 -0.33945 -18.7143 -19.4184 -19.7523 -0.25 0.614679
0.0184 0.00971 0.00466 0.0296 0.0136 0.0198 0.0197 0.0181 0.0158
2L:2492740-2492890:minus -12 0.293578 12.9358 3.86239 -17.8878 -8.65306 -13.9266 -1.68056 2.73394
0.000305 0.00932 0.0000218 0.00622 0.00704 0.00156 0.00714 0.0273 0.00756
2L:2492740-2492890:plus 15.1837 4.37615 0.87156 9.51376 -18.449 -8.45918 -8.77064 -4.36111 0.0642202
3.99e-07 0.00208 0.00864 0.000292 0.0107 0.00111 0.00245 0.0535 0.0181
2L:249280-249430:minus -12.8878 0.59633 3.68807 2.68807 -17.7143 -8.79592 -22.0917 -6.38889 -5.66972
0.000523 0.00837 0.00304 0.0104 0.00627 0.0019 0.03 0.0823 0.0829
2L:249280-249430:plus -14.3367 -0.422018 8.10092 5.22018 -17.4898 -28.4082 -6.46789 -1.61111 11.3853
0.00131 0.0119 0.000415 0.00418 0.0053 0.0615 0.00148 0.0268 0.0000815
2L:250880-251030:minus -14.3061 2.31193 -0.165138 2.44954 -8.97959 -18.0816 -16.1468 0.513889
-0.183486 0.00129 0.00448 0.0122 0.0116 0.0023 0.00934 0.0111 0.0142 0.0197
2L:250880-251030:plus -40.551 12.5505 1.88073 0.522936 -18.7143 -8.53061 -10.6789 5.44444 -0.926606
0.0418 0.0000313 0.00604 0.0234 0.0136 0.00136 0.0035 0.00215 0.0256
2L:252400-252550:minus -31.1837 -0.330275 5.88991 5.49541 0.877551 -27.051 -15.1651 -0.305556
-1.26606 0.0109 0.0115 0.00121 0.00355 0.00019 0.0315 0.00927 0.0184 0.0281
2L:252400-252550:plus -32.0408 -0.688073 6.02752 1.40367 20.1735 -8.45918 -8.38532 10.8194 1.2844
0.0174 0.013 0.00114 0.0184 5.68e-07 0.00111 0.00227 0.0000883 0.0129
2L:2575000-2575150:minus -34.0714 -0.12844 15.0459 3.56881 -17.449 0.540816 -15.3578 -0.361111
-3.82569 0.031 0.0108 1.26e-06 0.00699 0.00484 0.0002 0.00963 0.0187 0.0531
2L:2575000-2575150:plus -30.7755 -1.0367 2.34862 5.58716 -18.1224 -17.9796 -23.4862 3.18056 1.02752
0.0102 0.0145 0.00508 0.00334 0.00782 0.00764 0.038 0.00557 0.0141
2L:2580020-2580170:minus -31.8571 6.46789 4.3945 7.61468 10.6531 9.57143 -14.9908 1.34722 0.651376
0.0163 0.000847 0.00229 0.000779 0.0000102 0.0000274 0.00896 0.0108 0.0156
2L:2580020-2580170:plus -32.7041 -3.77064 8.41284 3.44037 10.9184 -0.0204082 -25.8532 0.638889
-4.44037 0.0222 0.0318 0.000351 0.00746 4.19e-06 0.000411 0.0525 0.0137 0.063
2L:2584740-2584890:minus -32.2857 3.94495 2.14679 7.33945 -18.3776 -8.30612 -2.20183 -0.888889
0.834862 0.0185 0.00245 0.00547 0.00102 0.00926 0.000973 0.000466 0.0218 0.0149
2L:2584740-2584890:plus -21.4388 5.14679 13.5872 5.94495 -17.7857 -8.65306 -22.6697 4.63889 1.78899
0.002 0.00153 0.0000122 0.00261 0.00676 0.00156 0.0332 0.00307 0.0103
2L:2585400-2585550:minus -12.1429 0.0733945 0.100917 2.69725 -8.71429 -27.5612 -19.633 4.54167
1.22936 0.000343 0.0101 0.0112 0.0104 0.00159 0.0393 0.0193 0.0032 0.0132
2L:2585400-2585550:plus -34.6327 -1.29358 -1.6055 2.25688 -19.2755 -17.2041 -26.2752 2.81944 1.59633
0.032 0.0157 0.0192 0.0127 0.0164 0.00507 0.0553 0.00639 0.0113
2L:2677560-2677710:minus -40.0204 -4.85321 2.55046 3.94495 -26.9592 -9.68367 -8.05505 -0.791667
7.37615 0.0353 0.0425 0.00471 0.00595 0.0215 0.00342 0.00213 0.0212 0.00107

2L:2677560-2677710:plus -14.1531 -0.642202 5.33028 3.00917 -16.1531 -9.27551 -7.48624 -1.34722
0.972477 0.00121 0.0128 0.00155 0.00894 0.00297 0.00244 0.00189 0.0249 0.0144
2L:271660-271810:minus -21.9286 -0.926606 12.367 3.42202 -26.8878 -8.65306 -24.8807 3.98611
-2.66972 0.0027 0.014 0.0000334 0.00751 0.0199 0.00156 0.0464 0.00404 0.0407
2L:271660-271810:plus -40.3673 14.1651 3.57798 6.00917 -35.9694 -8.45918 -24.6422 2.80556 2.63303
0.0397 6.88e-06 0.00317 0.00255 0.0709 0.00111 0.045 0.00642 0.00771
2L:2730020-2730170:minus -22.7347 -0.256881 9.40367 6.42202 -27 1.31633 -9.80734 -1.29167 -3.13761
0.00403 0.0113 0.0002 0.00188 0.0233 0.000066 0.00297 0.0245 0.0461
2L:2730020-2730170:plus -3.66327 0.541284 -1.85321 9.92661 -26.1939 -17.7857 11.2752 0.0694444
2.79817 0.0000935 0.00854 0.0207 0.000169 0.0175 0.0068 7.83e-06 0.0164 0.00743
2L:2730700-2730850:minus -32.0408 7.19266 7.82569 2.93578 -27.3061 -17.051 -16.2294 1.79167 1.2844
0.0174 0.000587 0.00048 0.00927 0.0315 0.00479 0.0113 0.00928 0.0129
2L:2730700-2730850:plus -21.9184 1.48624 4.86239 2.33945 -26.5306 -17.9796 -12.7339 3.27778 2.95413
0.00261 0.00606 0.00188 0.0122 0.0185 0.00764 0.00544 0.00536 0.0071
2L:2731820-2731970:minus -33.6633 0.513761 0.174312 1.57798 -8.0102 -27.1122 -19.9174 7.83333 0.486239
0.0291 0.00862 0.0109 0.0171 0.000826 0.0316 0.0203 0.000641 0.0163
2L:2731820-2731970:plus -31.9694 -5.29358 5.22936 6.72477 -18.9388 -17.2755 -29.9174 10.9722 1.59633
0.0168 0.0476 0.00162 0.00154 0.0151 0.00544 0.0899 0.0000781 0.0113
2L:273700-273850:minus -13.8776 13.6422 -1.33945 6.48624 -8.63265 -18.4898 -9.92661 4.90278 -3.11009
0.0011 0.0000127 0.0177 0.00177 0.0013 0.0129 0.00304 0.00274 0.0459
2L:273700-273850:plus -14.6633 -1.17431 7.80734 9.59633 -8.97959 -19.0816 -16.844 3.79167 -0.220183
0.0015 0.0151 0.000485 0.000275 0.0023 0.017 0.0125 0.00437 0.0201
2L:2739960-2740110:minus -31.8878 1.30275 2.24771 6.74312 -8.93878 -8.72449 -25.3211 -0.861111
-8.02752 0.0164 0.00649 0.00527 0.0015 0.00216 0.00169 0.0492 0.0217 0.136
2L:2739960-2740110:plus -31.4694 1.87156 2.0367 0.770642 -8.93878 -9.60204 -10.367 0.277778 -1.21101
0.0124 0.00526 0.0057 0.0219 0.00216 0.00334 0.0033 0.0153 0.0277
2L:2748520-2748670:minus -23.9592 2.66972 8.41284 7.21101 -8.93878 -27.5612 -19.1835 1.43056 0.0366972
0.00708 0.00393 0.000351 0.0011 0.00216 0.0393 0.018 0.0105 0.0182
2L:2748520-2748670:plus -22.9592 2.6422 3.06422 4.15596 -7.7449 -17.0102 -16.1101 0.611111 5.75229
0.00475 0.00397 0.00387 0.00539 0.000575 0.00453 0.0111 0.0138 0.00213
2L:2750260-2750530:minus -21.7041 3.97959 3.78899 2.15596 -9.20408 -18.051 -16.9541 -1.38889 -0.275229
0.00231 0.000669 0.00292 0.0133 0.00262 0.00886 0.0127 0.0252 0.0206
2L:2750260-2750530:plus -22.8878 3.68367 1.09174 1.77982 -28.5306 -17.9388 -11.1193 5.77778 9.45872
0.00441 0.00144 0.00801 0.0158 0.061 0.00738 0.00382 0.00184 0.000337
2L:2751060-2751210:minus -31.7041 -2.29358 -5.54128 7.51376 -7.56122 -17.0816 -15.8073 -7.38889 -1.9633
0.0146 0.0211 0.0566 0.000836 0.000419 0.00487 0.0105 0.0995 0.0329
2L:2751060-2751210:plus -31.4388 -3.0367 5.6789 7.52294 -8.60204 -27.3776 -12.2385 -1.68056 -0.247706
0.0122 0.026 0.00133 0.000819 0.00125 0.0351 0.00486 0.0273 0.0204
2L:2751540-2751690:minus -3.2551 5.69725 1.98165 0.238532 -27.9694 -18.0816 -2.33028 6.73611 9.34862
0.0000504 0.00121 0.00582 0.0251 0.0437 0.00934 0.00048 0.00115 0.000371
2L:2751540-2751690:plus -31.7041 -1.68807 -0.972477 4.19266 -17.4184 -9.79592 -22.4771 -1.55556
0.816514 0.0146 0.0177 0.0158 0.00536 0.00457 0.00377 0.0321 0.0264 0.015
2L:2752320-2752470:minus -22.5918 1.22018 3.21101 6.12844 1.21429 -27.5612 -8.57798 -4.18056 4.56881
0.00365 0.00668 0.00366 0.00231 0.0000864 0.0393 0.00236 0.0513 0.00356
2L:2752320-2752470:plus -23.5918 -2.13761 3.16514 6.56881 -18.4184 -19.051 -1.23853 0.486111 -0.275229
0.00656 0.0202 0.00373 0.0017 0.0101 0.0165 0.000374 0.0144 0.0206
2L:2753020-2753170:minus -22.6939 6.61468 2.89908 7.80734 -18.7857 -17.7857 -17.1009 2.01389 5.90826
0.00388 0.000789 0.00413 0.00063 0.0148 0.0068 0.013 0.00858 0.00198
2L:2753020-2753170:plus -3.26531 -2.00917 7.55963 4.07339 -17.0408 1.09184 0.0917431 7.90278
5.79817 0.0000515 0.0195 0.00055 0.00562 0.00329 0.000091 0.000277 0.000616 0.00208
2L:2753760-2753910:minus -21.7755 -1.77982 3.88073 1.6055 -27.1633 -16.9796 -7.61468 -7.88889 3.17431
0.00241 0.0182 0.00281 0.0169 0.025 0.0044 0.00194 0.109 0.00653
2L:2753760-2753910:plus -22.7041 2.58716 2.90826 2.26606 -26.6735 -18.051 -18.5963 2.02778 4.97248
0.00395 0.00404 0.00411 0.0126 0.0191 0.00886 0.0164 0.00853 0.00315

2L:2754400-2754550:minus -22.5612 0.192661 -1.30275 4.65138 -8.93878 -17.0816 -5.66972 0.319444 8.10092
0.0036 0.00965 0.0175 0.00471 0.00216 0.00487 0.0012 0.0151 0.000623
2L:2754400-2754550:plus -32.4796 -2.57798 -0.678899 2.25688 -18.2653 -0.132653 -18 6.25 3.77064
0.02 0.0229 0.0144 0.0127 0.00882 0.000444 0.015 0.00147 0.00491
2L:2755320-2755470:minus -22.9898 -0.853211 11.5963 2.06422 -7.56122 -35.1531 -8.55046 1.15278
1.63303 0.00482 0.0137 0.0000561 0.0139 0.000419 0.0753 0.00235 0.0115 0.0112
2L:2755320-2755470:plus -21.9694 -2.87156 5.55963 4.13761 -7.60204 -9.86735 -8.42202 3.93056 1.40367
0.00282 0.0249 0.0014 0.00546 0.000466 0.00391 0.00229 0.00413 0.0122
2L:2756900-2757050:minus -30.6327 -0.807339 8.37615 3.70642 -8.89796 -7.79592 -14.5872 5.34722
-5.50459 0.00968 0.0135 0.000358 0.0066 0.00189 0.000723 0.00824 0.00225 0.0806
2L:2756900-2757050:plus -22.551 0.513761 -0.33945 2.43119 -7.93878 -18.7551 2.48624 2.125 -3.65138
0.00359 0.00862 0.0129 0.0117 0.000767 0.0141 0.000141 0.00824 0.0512
2L:2757240-2757390:minus -23.0714 0.642202 4.76147 5.57798 -26.4898 0.897959 -10.4587 -2.56944 5.18349
0.0052 0.00823 0.00196 0.0034 0.0183 0.000144 0.00336 0.0346 0.00295
2L:2757240-2757390:plus -13.8571 1.78899 4.33028 7.33945 -28.4592 -8.79592 -16.5688 11.5278 7.83486
0.0011 0.00542 0.00235 0.00102 0.0568 0.0019 0.012 0.0000481 0.000712
2L:2759740-2759890:minus -13.449 9.7156 4.95413 9.33028 -17.9796 -18.0102 -4.85321 -5.875 3.78899
0.000914 0.000154 0.00181 0.000374 0.00726 0.00812 0.000952 0.0742 0.00487
2L:2759740-2759890:plus -31.8878 13.4592 -3.02752 5.94495 -18.4898 -8.79592 -18.8991 3.91667 -6.14679
0.0164 0.000064 0.0291 0.00261 0.0116 0.0019 0.0172 0.00416 0.0921
2L:2765600-2765750:minus -33.5816 2.6422 7.6789 3.59633 -27.9694 -36.1837 -3.34862 -2.36111 5.6055
0.0288 0.00397 0.000518 0.00693 0.0437 0.0914 0.000623 0.0328 0.00232
2L:2765600-2765750:plus -31 -5.01835 -3.02752 0.908257 -17.449 -18.1224 -17.2385 -2 2.78899 0.0105
0.0443 0.0291 0.0211 0.00484 0.00988 0.0133 0.0298 0.00745
2L:2766120-2766270:minus -23.551 -0.0733945 1.79817 2.86239 -27.6429 -9.72449 -12.6606 9.81944
7.25688 0.00648 0.0106 0.00623 0.00954 0.037 0.00352 0.00535 0.000186 0.00115
2L:2766120-2766270:plus -31.2449 -4.76147 -0.46789 3.18349 -8.93878 -27.602 -15.3119 0.361111 3.24771
0.0111 0.0414 0.0135 0.00837 0.00216 0.0416 0.00954 0.0149 0.00636
2L:2767800-2767950:minus -34.0306 7.22018 -3.33945 0.275229 -18.1531 -9.30612 -23.3578 0.958333 -1.21101
0.0307 0.000579 0.0317 0.025 0.00829 0.00257 0.0372 0.0123 0.0277
2L:2767800-2767950:plus -12.3673 13.5306 9.53211 3.42202 -9.23469 -19.4184 -23.2569 5.29167 -0.422018
0.000396 0.0000206 0.000187 0.00751 0.00277 0.0198 0.0366 0.0023 0.0213
2L:2769820-2769970:minus -31.7755 0.834862 0.788991 2.3945 -18.7143 -28.051 -12.2018 0.986111 -1.48624
0.0157 0.00769 0.00889 0.0119 0.0136 0.0563 0.00482 0.0122 0.0293
2L:2769820-2769970:plus -39.9898 5.57798 2.40367 4.12844 -18.4184 -8.65306 -16.3486 -3.63889 1.02752
0.0351 0.00128 0.00498 0.00549 0.0101 0.00156 0.0115 0.0452 0.0141
2L:277300-277450:minus -22.6939 13.4592 -5.04587 0.642202 20.4694 -18.0102 -5.02752 1.19444 -0.706422
0.00388 0.000064 0.0499 0.0227 1.65e-07 0.00812 0.001 0.0114 0.024
2L:277300-277450:plus -22.8163 2.73394 4.80734 6.29358 -7.60204 1.97959 -16.0826 6.41667 -1.88073
0.00418 0.00383 0.00193 0.00202 0.000466 0.0000486 0.011 0.00135 0.0321
2L:2808120-2808270:minus -30.6633 5.74312 1.59633 2.66972 -17.0408 -18.0102 -11.1927 0.583333 0.275229
0.00976 0.00119 0.00669 0.0106 0.00329 0.00812 0.00388 0.0139 0.0171
2L:2808120-2808270:plus -30.1735 -0.256881 1.69725 5.94495 -9.23469 -27.8265 -8.83486 2.54167
8.11009 0.00869 0.0113 0.00646 0.00261 0.00277 0.0498 0.00248 0.00709 0.000597
2L:283260-283410:minus -2.07143 -0.715596 15.1193 7.29358 -18.6837 -8.65306 -14.3945 -3.05556
-4.15596 0.0000251 0.0131 1.13e-06 0.00104 0.0126 0.00156 0.00791 0.0392 0.0578
2L:283260-283410:plus -23.5918 13.3878 10.8624 5.94495 -26.898 -18.0816 -23.2844 1.66667 3.42202
0.00656 0.0000869 0.0000868 0.00261 0.0203 0.00934 0.0368 0.00969 0.00597
2L:2856040-2856190:minus -33.5612 8.76147 1.54128 5.02752 -27.602 -8.64286 -21.5413 5.58333 -5.91743
0.0288 0.000254 0.00683 0.00435 0.0364 0.00154 0.0271 0.00202 0.0874
2L:2856040-2856190:plus -14.1122 -0.0275229 8.51376 5.42202 -18.1531 0.244898 -3.57798 -5.94444
1.16514 0.00119 0.0104 0.00033 0.00374 0.00829 0.000285 0.000663 0.0752 0.0135
2L:285700-285850:minus -22 -1.52294 0.477064 5.22936 -26.1939 -8.42857 -13.1927 0.333333 5.6055
0.00291 0.0169 0.00989 0.0041 0.0175 0.00104 0.00604 0.0151 0.00232

2L:285700-285850:plus -20.9286 3.21101 7.95413 1.6789 -26.8571 -18.1224 -16.6881 3.51389 -2.92661 0.00183 0.00322 0.000448 0.0165 0.0198 0.00988 0.0122 0.00489 0.0437
2L:2857020-2857170:minus -31.9592 0.697248 1.06422 1.56881 10.3571 -8.72449 -25.4771 2.95833 -1.11927 0.0167 0.00807 0.00808 0.0172 0.0000208 0.00169 0.0501 0.00606 0.027
2L:2857020-2857170:plus -31.2143 9.20183 -4.52294 4.3211 20.4694 -18.2755 -7.14679 -5.63889 1.38532 0.0111 0.000201 0.0436 0.00514 1.65e-07 0.0112 0.00175 0.0707 0.0123
2L:2858200-2858350:minus -31.5204 -4.05505 -0.201835 -2.59633 -28.1633 -7.5 -22.7706 1.26389 1.27523 0.0131 0.0343 0.0124 0.062 0.0462 0.000614 0.0338 0.0111 0.013
2L:2858200-2858350:plus -32.1122 0.944954 6.20183 2.68807 -18.6837 -16.9388 -17.5688 3 0.00917431 0.0178 0.00739 0.00106 0.0104 0.0126 0.00433 0.014 0.00597 0.0184
2L:2862420-2862570:minus -20.551 -1.29358 -0.761468 1.83486 -27.1939 -17.1633 -16.2752 4.02778 -5.66972 0.00168 0.0157 0.0148 0.0155 0.0267 0.005 0.0114 0.00397 0.0829
2L:2862420-2862570:plus -12.0714 -0.715596 -1.05505 10.0275 -17.3776 -8.30612 -4.56881 2.69444 3.05505 0.000318 0.0131 0.0162 0.000156 0.00419 0.000973 0.000877 0.00669 0.00692
2L:286620-286770:minus -31.3571 -1.88073 1.40367 7.46789 -18.4898 -8.60204 -10.7982 3.75 -2.36697 0.0114 0.0188 0.00717 0.000868 0.0116 0.00151 0.00359 0.00445 0.0371
2L:286620-286770:plus -12.7449 2.14679 0.146789 4.40367 -17.1122 -26.3367 -5.92661 1.48611 5.65138 0.000471 0.00476 0.011 0.00497 0.00341 0.0221 0.00129 0.0103 0.00228
2L:2872800-2872950:minus -32.7347 -0.53211 1.43119 3.81651 -17.4592 2.27551 -20.4954 -2.51389 0.201835 0.0225 0.0123 0.0071 0.00626 0.00491 0.0000444 0.0225 0.0341 0.0175
2L:2872800-2872950:plus -34.2653 -2.13761 6.49541 2.93578 -18.9796 -16.5714 -19.2385 5.56944 -2.68807 0.0316 0.0202 0.000921 0.00927 0.0155 0.00417 0.0181 0.00203 0.041
2L:2873720-2873870:minus -22.5204 -0.146789 8.41284 2.44037 -16.9286 -7.38776 -19.8899 0.791667 3.51376 0.00356 0.0108 0.000351 0.0116 0.00327 0.000563 0.0202 0.013 0.00566
2L:2873720-2873870:plus -22.8061 0.743119 1.90826 1.43119 -17.7143 -17.2041 -15.6147 1.75 1.23853 0.00414 0.00794 0.00598 0.0181 0.00627 0.00507 0.0101 0.00941 0.0132
2L:2874500-2874650:minus -31.5102 6.80734 -0.366972 5.69725 -7.33673 -8.5 -11.4679 1.27778 -2.73394 0.013 0.000716 0.0131 0.00314 0.000388 0.00126 0.00411 0.0111 0.0416
2L:2874500-2874650:plus -23.449 2.69725 0.926606 0.724771 -27.5306 -8.30612 -21.8532 4.56944 3.47706 0.00631 0.00389 0.00848 0.0223 0.0351 0.000973 0.0287 0.00317 0.00575
2L:2878280-2878430:minus -39.7245 1.75229 13.1835 7.2844 1.14286 -8.72449 -11.6697 7.69444 -1.63303 0.0337 0.0055 0.0000171 0.00105 0.000113 0.00169 0.00429 0.000693 0.0304
2L:2878280-2878430:plus -31.6224 -4.09174 6.58716 1.22936 -8.92857 -27.9286 -18.6972 2.84722 -2.62385 0.0137 0.0347 0.00088 0.0193 0.00194 0.054 0.0166 0.00632 0.0402
2L:2878780-2878930:minus -2.95918 -0.981651 1.13761 7.47706 -26.9286 -17.7857 -11.5688 -2.52778 3.78899 0.0000399 0.0143 0.00788 0.00085 0.0214 0.0068 0.0042 0.0343 0.00487
2L:2878780-2878930:plus -30.449 2.41284 15.7339 7.29358 -8.64286 -18.2755 -16.7523 -0.0972222 -4.55046 0.00925 0.00431 2.45e-07 0.00104 0.00139 0.0112 0.0123 0.0172 0.0646
2L:2879060-2879210:minus -23.7449 0.733945 15.4495 1.49541 -18.7143 -19.6429 -20.6972 1.98611 1.38532 0.00674 0.00797 9.17e-07 0.0176 0.0136 0.0204 0.0233 0.00866 0.0123
2L:2879060-2879210:plus -30.449 2.41284 12.9817 7.29358 -8.64286 -18.2755 -16.7523 -2.05556 -4.55046 0.00925 0.00431 0.0000211 0.00104 0.00139 0.0112 0.0123 0.0303 0.0646
2L:2884180-2884330:minus -32.0714 7.30275 -1.22018 8.61468 -18.7143 -19.1531 -19.9358 11.375 6.7156 0.0176 0.000554 0.0171 0.000552 0.0136 0.0177 0.0204 0.0000552 0.00144
2L:2884180-2884330:plus -20.8878 1.05505 -2.18349 11.0275 -17.3776 -27.6327 -11.6514 -4.73611 -5.58716 0.0018 0.00709 0.0228 0.0000401 0.00419 0.043 0.00427 0.0582 0.0817
2L:2884840-2884990:minus -23.4796 5.97248 5.47706 4.0367 -18.9796 -18.9796 -13.5413 -0.736111 -2.55046 0.00634 0.00107 0.00145 0.00578 0.0155 0.0155 0.00654 0.0209 0.0394
2L:2884840-2884990:plus -31.5204 -1.3945 3.66972 6.56881 -18.4184 -9.65306 -15.1835 0.888889 11.3853 0.0131 0.0162 0.00306 0.0017 0.0101 0.0034 0.00931 0.0126 0.0000815
2L:2885140-2885290:minus -21.8163 -1.56881 4.55963 2.53211 -17.9796 -9.79592 -18.0642 11.0139 3.34862 0.00244 0.0171 0.00214 0.0113 0.00726 0.00377 0.0151 0.0000754 0.00609
2L:2885140-2885290:plus -33.5612 5.45872 1.14679 2.77064 -19.3163 -7.86735 -28.4404 2.93056 2.37615 0.0288 0.00134 0.00785 0.01 0.0165 0.00073 0.073 0.00612 0.00825

2L:2885860-2886010:minus -21.8878 8.44037 13.7523 7.29358 -26.6327 -8.53061 -15.3028 0.861111 0.321101 0.00258 0.000301 9.94e-06 0.00104 0.0188 0.00136 0.00953 0.0127 0.0169
2L:2885860-2886010:plus -31.7347 13.4592 -1.70642 1.78899 -8.53061 -16.2755 -9.79817 1.15278 -0.697248 0.0149 0.000064 0.0198 0.0158 0.0011 0.00413 0.00296 0.0115 0.0239
2L:2886220-2886370:minus -31.2857 -1.05505 3.82569 6.12844 -7.85714 -9.5 -18.7064 5.16667 -3.09174 0.0113 0.0146 0.00288 0.00231 0.000594 0.00294 0.0167 0.00244 0.0456
2L:2886220-2886370:plus -32.0714 1.29358 6.05505 3.62385 -18.5204 -27.8265 -9.36697 1.18056 9.23853 0.0176 0.00651 0.00113 0.00673 0.0117 0.0498 0.00274 0.0114 0.000397
2L:2886700-2886850:minus -31.8571 1.41284 6.70642 7.27523 -18.7551 -9.02041 -22.4587 -0.166667 -2.47706 0.0163 0.00623 0.000832 0.00109 0.0144 0.00213 0.032 0.0176 0.0385
2L:2886700-2886850:plus -30.1837 -1.24771 -3.6422 3.47706 -17.7551 -8.72449 -12.7431 -0.361111 -1.79817 0.00873 0.0155 0.0345 0.00724 0.00658 0.00169 0.00545 0.0187 0.0315
2L:2888080-2888230:minus -31.3265 3.64286 7.56881 5.43119 -18.051 -19.051 -14.2018 3.08333 1.48624 0.0114 0.00159 0.000547 0.00371 0.00746 0.0165 0.00758 0.00578 0.0118
2L:2888080-2888230:plus -13.2551 0.623853 3.12844 4.50459 -17.1531 -0.244898 -4.23853 1.68056 5.59633 0.000751 0.00829 0.00378 0.00484 0.00364 0.000496 0.000798 0.00964 0.00234
2L:2908520-2908670:minus -23.4082 1.66055 2.9633 5.43119 -17.449 -26.898 -17.3394 6.75 1.2844 0.00623 0.00569 0.00403 0.00371 0.00484 0.0302 0.0135 0.00114 0.0129
2L:2908520-2908670:plus -32.9286 4.0367 -1.11927 1.16514 -7.96939 -8.72449 -21.7064 2.125 2.05505 0.025 0.00237 0.0166 0.0197 0.000785 0.00169 0.028 0.00824 0.00914
2L:2924440-2924590:minus -23.2551 6.70642 -2.42202 3.95413 -9.19388 -18.051 -9.45872 3.61111 -1.21101 0.00583 0.000754 0.0244 0.00593 0.00253 0.00886 0.00278 0.0047 0.0277
2L:2924440-2924590:plus -14.0714 0.972477 7.01835 7.17431 -17.0408 -9.16327 -29.7798 -6.36111 -12.5229 0.00117 0.00731 0.000721 0.00115 0.00329 0.00223 0.0881 0.0818 0.289
2L:2925140-2925290:minus -30.4796 0.33945 -0.844037 2.89908 -27.9388 -8.93878 -16.422 -3.26389 5.44037 0.00941 0.00917 0.0152 0.00938 0.0415 0.00201 0.0117 0.0413 0.00259
2L:2925140-2925290:plus -30.7755 -1.61468 -0.53211 2.30275 -25.9694 -26.5306 -5.00917 3.30556 12.2752 0.0102 0.0173 0.0138 0.0124 0.017 0.023 0.000995 0.0053 0.0000141
2L:2925580-2925730:minus -30.398 4.86239 8.01835 6.11009 -17.4184 -8.27551 -18.4037 -0.805556 2.06422 0.00901 0.00172 0.000433 0.00233 0.00457 0.000919 0.0159 0.0213 0.00912
2L:2925580-2925730:plus -31.551 5.31193 1.84404 -0.0642202 -18.8265 -27.449 -8.52294 0.347222 10.3394 0.0134 0.00143 0.00612 0.0274 0.0149 0.0357 0.00234 0.015 0.000112
2L:2925980-2926130:minus -23.3265 0.577982 2.88991 3.56881 -18.1531 -18.051 -12.7064 5.38889 -2.24771 0.00607 0.00843 0.00414 0.00699 0.00829 0.00886 0.00541 0.0022 0.0357
2L:2925980-2926130:plus -31.551 2.02752 3.50459 7.63303 -27.3061 -28.1939 -9.70642 4.30556 8.56881 0.0134 0.00497 0.00326 0.000745 0.0315 0.0591 0.00291 0.00354 0.000491
2L:294740-294890:minus -24.3776 14.5688 1.74312 6.3578 -18.1939 -17.9388 -18.789 -1 -3.69725 0.00793 4.39e-06 0.00635 0.00195 0.0086 0.00738 0.0169 0.0226 0.0517
2L:294740-294890:plus -32.9286 14.8716 0.146789 2.07339 -17.9796 9.37755 -26.1009 3.15278 -1.21101 0.025 2.8e-06 0.011 0.0138 0.00726 0.0000363 0.0542 0.00563 0.0277
2L:295060-295210:minus -43.0408 -0.862385 0.862385 5.90826 -19.051 -8.79592 -15.055 -0.652778 -4.84404 0.0892 0.0137 0.00867 0.00271 0.0162 0.0019 0.00907 0.0204 0.0701
2L:295060-295210:plus -4.88776 13.5306 1.90826 3.77064 -18.449 10.0918 -11.789 5.61111 -1.36697 0.000204 0.0000206 0.00598 0.00637 0.0107 9.34e-06 0.0044 0.00199 0.0286
2L:2969600-2969750:minus -33.0714 5.00917 -2.11927 2.66972 -17.1939 -0.204082 -3.41284 2.90278 -0.174312 0.0267 0.00162 0.0224 0.0106 0.00377 0.000463 0.000634 0.00619 0.0196
2L:2969600-2969750:plus -21.9184 6.31193 -0.522936 5.22018 -18.4898 -17.2755 -11.1101 6.20833 -3.27523 0.00261 0.000913 0.0137 0.00418 0.0116 0.00544 0.00382 0.0015 0.0473
2L:2972420-2972570:minus -21.0306 -1.06422 0.908257 3.26606 -17.5204 -17.2041 -8.00917 1.81944 1.6422 0.00186 0.0146 0.00853 0.00802 0.00532 0.00507 0.00211 0.00919 0.0111
2L:2972420-2972570:plus -23.6224 -1 -0.798165 4.58716 -18.7143 -19.0816 -19.5688 7.34722 -0.688073 0.00658 0.0143 0.015 0.00479 0.0136 0.017 0.0191 0.000838 0.0238
2L:2972720-2972870:minus -33.1122 -3.61468 0.908257 3.26606 -7.93878 -9.79592 -17.578 1.22222 1.6422 0.0271 0.0305 0.00853 0.00802 0.000767 0.00377 0.014 0.0113 0.0111

2L:2972720-2972870:plus -32.6939 1.62385 4.18349 3.11009 -17.8163 0.0510204 -11.8257 6.66667
-0.550459 0.0217 0.00577 0.00249 0.00862 0.00682 0.000349 0.00444 0.00119 0.0225
2L:2976980-2977130:minus -13.1122 2.22018 4.93578 5.69725 -18.6429 -9.79592 6.40367 -0.972222
-0.889908 0.000632 0.00463 0.00183 0.00314 0.0121 0.00377 0.0000367 0.0224 0.0253
2L:2976980-2977130:plus -24.3673 -0.348624 4.83486 1.66055 -9.16327 -9.34694 -10.2294 -2.375
-0.174312 0.00791 0.0116 0.00191 0.0166 0.00249 0.00261 0.00321 0.0329 0.0196
2L:297780-297930:minus -21.9286 6.54128 -1.58716 7.3578 -19.0102 -9.82653 -15.4587 4.38889 5.22936
0.0027 0.000818 0.0191 0.000965 0.0157 0.00381 0.00982 0.00342 0.00288
2L:297780-297930:plus -31.3265 -1.85321 2.23853 3.18349 -18.8265 -27.8571 -17.8073 7.48611 2.16514
0.0114 0.0186 0.00529 0.00837 0.0149 0.0508 0.0145 0.000777 0.00879
2L:2979240-2979390:minus -20.8163 2.01835 13.6606 7.45872 -26.6633 -18.2041 -15.7615 -0.430556
-4.91743 0.00177 0.00499 0.0000112 0.000894 0.0189 0.0103 0.0104 0.0191 0.0715
2L:2979240-2979390:plus -41.6327 3.55046 0.0275229 0.53211 -27.5306 -8.5 -17.633 7.29167 5.55046
0.0631 0.00284 0.0115 0.0234 0.0351 0.00126 0.0141 0.000863 0.00241
2L:298360-298510:minus -31.4694 4.14679 -1 2.6422 -16.4184 -18.7857 -1.53211 4.45833 3.88073
0.0124 0.00228 0.016 0.0107 0.00304 0.0146 0.000399 0.00332 0.00475
2L:298360-298510:plus -13.1837 5.10092 3.15596 2.23853 -17.4898 -9.30612 -13.2661 4.33333 5.44954
0.000705 0.00156 0.00374 0.0128 0.0053 0.00257 0.00614 0.0035 0.00253
2L:2987620-2987770:minus -20.3571 -2.3945 2.73394 1.45872 10.6939 -18.1224 -12.367 -2.66667 0.12844
0.00165 0.0218 0.00439 0.0179 7.35e-06 0.00988 0.005 0.0355 0.0179
2L:2987620-2987770:plus -33.0408 7.38532 -3.3945 1.63303 -28.2347 -9.30612 -27.8532 6.11111 3.52294
0.0264 0.000529 0.0322 0.0167 0.051 0.00257 0.0675 0.00157 0.00558
2L:2988020-2988170:minus -44 7.19266 -5.41284 6.12844 -18.9388 -0.469388 -24.7615 6.36111 3.51376
0.0946 0.000587 0.0548 0.00231 0.0151 0.000533 0.0457 0.00139 0.00566
2L:2988020-2988170:plus -31.7041 13.4592 13.3119 8.6055 -8.82653 -9.30612 -15.5872 -0.291667
-1.90826 0.0146 0.000064 0.0000151 0.000567 0.00163 0.00257 0.0101 0.0183 0.0324
2L:2992760-2992910:minus -31.4388 2.91743 15.5596 7.29358 -8.82653 -8.53061 -19.8165 0.763889 -2.77982
0.0122 0.00358 6.69e-07 0.00104 0.00163 0.00136 0.02 0.0131 0.042
2L:2992760-2992910:plus -31.6939 12.1193 3.6789 1.53211 -27.1939 -9.79592 -18.8073 0.875 -2.94495
0.0142 0.000043 0.00305 0.0174 0.0267 0.00377 0.0169 0.0127 0.0441
2L:2993320-2993470:minus -14.3673 1.62385 6.22018 0.366972 -18.0816 -19.2755 -19.2202 -3.38889 -9.42202
0.00132 0.00577 0.00105 0.0244 0.00759 0.0185 0.0181 0.0426 0.177
2L:2993320-2993470:plus -22.2959 -1.04587 5.56881 5.43119 -18.449 -36.449 9.22936 3.80556 3.24771
0.00335 0.0145 0.0014 0.00371 0.0107 0.109 0.0000167 0.00435 0.00636
2L:2994480-2994630:minus -30.449 -4.37615 0.385321 1.63303 -17.3469 -7.5 -21.945 5.27778 -1.15596
0.00925 0.0374 0.0102 0.0167 0.00394 0.000614 0.0292 0.00232 0.0273
2L:2994480-2994630:plus -11.9184 0.816514 5.89908 6.36697 -8.64286 -0.27551 -13.8349 -2.76389 5.44037
0.000293 0.00773 0.00121 0.00193 0.00139 0.000502 0.00699 0.0364 0.00259
2L:2999120-2999270:minus -23.7449 7.87156 2.14679 7.44037 -18.449 -0.316327 0.412844 1.52778
-1.13761 0.00674 0.000409 0.00547 0.000912 0.0107 0.000524 0.000256 0.0102 0.0271
2L:2999120-2999270:plus 6.21429 3.47706 0.110092 9.51376 -8.96939 -16.7143 -6.91743 4.11111 0.697248
4.82e-06 0.00292 0.0112 0.000292 0.00226 0.00419 0.00165 0.00384 0.0154
2L:301360-301510:minus -23.7347 0.816514 1.11009 2.41284 -18.449 -26.3061 -16.7615 -0.888889
4.11927 0.00673 0.00773 0.00796 0.0118 0.0107 0.0219 0.0123 0.0218 0.00411
2L:301360-301510:plus -22.1939 6.29358 4.70642 10.5872 -18.4592 -17.0816 -21.6147 0.25 1.01835 0.00317
0.000922 0.00201 0.000115 0.0109 0.00487 0.0275 0.0155 0.0142
2L:3018300-3018450:minus -31.1837 0.0275229 -0.724771 4.6055 -17.6837 -17.7551 -16.5413 0.569444
0.788991 0.0109 0.0102 0.0146 0.00476 0.0058 0.00643 0.0119 0.014 0.0151
2L:3018300-3018450:plus -41.1735 7.45872 2.36697 2.06422 -9.23469 -28.602 -22.789 0.541667 0.275229
0.0479 0.000509 0.00505 0.0139 0.00277 0.0648 0.0339 0.0141 0.0171
2L:3023080-3023230:minus -13.3367 13.5306 4.11927 7.27523 -18.4898 -8.30612 -12.0917 1.91667 1.95413
0.0008 0.0000206 0.00255 0.00109 0.0116 0.000973 0.00471 0.00888 0.00964
2L:3023080-3023230:plus -14.5204 7.36697 -1.11927 5.93578 -18.4184 -8.79592 -20.4128 0.569444 7.58716
0.00145 0.000535 0.0166 0.00264 0.0101 0.0019 0.0221 0.014 0.000954

2L:3029160-3029310:minus -24.5204 -0.174312 -4.36697 6.25688 -18.4898 -8.42857 -15.2844 6.20833
-6.58716 0.00813 0.0109 0.0419 0.00208 0.0116 0.00104 0.00949 0.0015 0.103
2L:3029160-3029310:plus -22.0408 -2.3578 13.844 -0.412844 -18.4898 -17.898 -6.41284 3.88889
6.11927 0.00296 0.0215 8.84e-06 0.0304 0.0116 0.00726 0.00146 0.00421 0.00178
2L:3030340-3030490:minus -30.6633 -3.51376 5.14679 2.76147 -17.3367 -19.1224 -12.5321 -5.91667 -2.98165
0.00976 0.0297 0.00167 0.01 0.00391 0.0175 0.00519 0.0748 0.0445
2L:3030340-3030490:plus -40.2959 3.88073 12.1468 7.2844 -7.37755 -9.5 -15.4587 -1.47222 8.62385
0.0389 0.00252 0.0000388 0.00105 0.000398 0.00294 0.00982 0.0258 0.00048
2L:3037300-3037450:minus -14.2551 2.18349 10.0092 3.59633 -18.4184 -38.1837 -6.98165 -1.09722 8.10092
0.00127 0.00469 0.000141 0.00693 0.0101 0.187 0.00168 0.0232 0.000623
2L:3037300-3037450:plus -31.6633 -2 13.4495 3.90826 -9.5 -18.3469 -15.0734 0.555556 5.25688 0.0141
0.0194 0.0000136 0.00603 0.00293 0.0124 0.00911 0.014 0.0028
2L:3039920-3040070:minus -23.0714 -1.44037 -0.0825688 4.12844 -27.4898 -0.0204082 -8.27523 2.05556
1.22018 0.0052 0.0164 0.0119 0.00549 0.0336 0.000411 0.00222 0.00845 0.0133
2L:3039920-3040070:plus -23.449 4.38532 15.7339 7.29358 -18.7857 -9.45918 -12.7339 1.44444 -3.99083
0.00631 0.00207 2.45e-07 0.00104 0.0148 0.00279 0.00544 0.0105 0.0556
2L:3046600-3046750:minus -22.2959 7.04587 2.79817 7.6422 -18.7449 -9.5 -9.52294 -2.81944 6.25688
0.00335 0.000633 0.00429 0.000721 0.0138 0.00294 0.00282 0.0369 0.00163
2L:3046600-3046750:plus -22.0408 4.08257 5.43119 10.8624 -27.2347 -26.8571 -12.9908 -5.04167 9.77064
0.00296 0.00233 0.00148 0.0000687 0.0283 0.0288 0.00577 0.0622 0.000228
2L:304920-305070:minus -40.7755 2.07339 0.266055 8.81651 -27.4898 -18.0816 -24.6697 0.638889 -5.11927
0.0433 0.00488 0.0106 0.000501 0.0336 0.00934 0.0451 0.0137 0.0746
2L:304920-305070:plus -23.5612 -0.357798 -2.17431 1.44954 -18.5612 -18.3469 -0.926606 1.08333
-0.284404 0.00653 0.0116 0.0227 0.0179 0.0119 0.0124 0.000349 0.0118 0.0207
2L:3055880-3056030:minus -32.4796 3.53211 -0.880734 2.46789 -18.449 -9.79592 -19.0183 3.93056
0.642202 0.02 0.00286 0.0154 0.0115 0.0107 0.00377 0.0175 0.00413 0.0157
2L:3055880-3056030:plus -31.4388 6.01835 13.5321 1.59633 0.612245 -18.3061 -12.1101 1.83333 -1.18349
0.0122 0.00105 0.0000127 0.017 0.000269 0.0114 0.00472 0.00914 0.0276
2L:3056500-3056650:minus -13 -0.669725 -1.46789 6.90826 -18.449 -27.3776 -19.422 -0.375 5.57798
0.000563 0.0129 0.0184 0.00133 0.0107 0.0351 0.0187 0.0188 0.00238
2L:3056500-3056650:plus -13.1122 -1.55963 6.87156 3.47706 -18.449 -17.9388 -10.5963 14.0694 1.16514
0.000632 0.017 0.000772 0.00724 0.0107 0.00738 0.00345 2.31e-06 0.0135
2L:3057140-3057290:minus -34.0714 -0.944954 -1.01835 2.9633 -17.4184 1.82653 -19.3578 1.68056
9.56881 0.031 0.0141 0.0161 0.00914 0.00457 0.0000498 0.0185 0.00964 0.000303
2L:3057140-3057290:plus -22.7755 -2.19266 2.06422 6.06422 -26.1939 -26.8571 -10.2294 5.84722 3.47706
0.00412 0.0205 0.00564 0.00239 0.0175 0.0288 0.00321 0.00178 0.00575
2L:305800-305950:minus -23.9592 1.55046 11.367 7.55963 -26.9694 -9.72449 -19.4312 9.83333 -0.642202
0.00708 0.00592 0.0000636 0.000807 0.023 0.00352 0.0187 0.000185 0.0234
2L:305800-305950:plus -40.551 1.19266 6.41284 6.6422 -8.63265 9.60204 -18.2477 -4.22222 3.17431
0.0418 0.00675 0.000959 0.00161 0.0013 0.0000248 0.0155 0.0518 0.00653
2L:3058640-3058790:minus -13.3776 0.862385 3.09174 7.3578 -18.3776 -8.79592 -20.5138 -0.75 2.17431
0.000831 0.00761 0.00383 0.000965 0.00926 0.0019 0.0225 0.021 0.00872
2L:3058640-3058790:plus -23 1.99083 3.21101 5.81651 -18.4898 -18.7857 -11.6514 0.611111 -0.697248
0.00498 0.00504 0.00366 0.00292 0.0116 0.0146 0.00427 0.0138 0.0239
2L:3059040-3059190:minus -24.0714 1.02752 2.72477 6.08257 -27.2347 -18.5612 -3.33028 11.4861 9.88073
0.00731 0.00717 0.00441 0.00236 0.0283 0.0132 0.00062 0.0000499 0.000206
2L:3059040-3059190:plus -22.9184 -0.440367 1.40367 7.33028 -18.449 -27.2653 -11.8899 0.0416667
-1.41284 0.00447 0.012 0.00717 0.00103 0.0107 0.0333 0.0045 0.0165 0.0289
2L:3124700-3124850:minus -11.7347 1.11927 3.50459 9.61468 -8.53061 -28.7041 1.91743 -0.666667
0.385321 0.000263 0.00693 0.00326 0.000255 0.0011 0.067 0.000169 0.0205 0.0166
2L:3124700-3124850:plus -28.8776 -2.9633 12.211 3.42202 -26.6735 -27.602 -19.6972 -1.59722 11.8532
0.00832 0.0255 0.0000371 0.00751 0.0191 0.0416 0.0196 0.0267 0.0000428
2L:3144860-3145010:minus -21.9592 -0.568807 0.394495 -0.110092 -8.64286 -18.051 -13.3119 0.333333
7.63303 0.00279 0.0125 0.0102 0.0277 0.00139 0.00886 0.00621 0.0151 0.000928

2L:3144860-3145010:plus -32.5102 6.94495 1.79817 2.29358 -26.5306 -18.6429 -7.10092 -0.152778
2.11927 0.0202 0.000669 0.00623 0.0124 0.0185 0.0139 0.00173 0.0175 0.00889
2L:3145720-3145870:minus -22.1837 14.9908 3.68807 2.70642 -18.4898 -9.72449 -19.9725 -1.86111 -1.48624
0.00315 2.1e-06 0.00304 0.0103 0.0116 0.00352 0.0205 0.0287 0.0293
2L:3145720-3145870:plus -32.1122 -1.95413 0.697248 5.57798 -18.7857 -8.68367 -14.9633 -1.90278 -2.08257
0.0178 0.0192 0.00917 0.0034 0.0148 0.00157 0.00891 0.029 0.034
2L:3171040-3171190:minus -31.7347 -1.34862 4.27523 5.86239 -18.449 -18.3061 -8.49541 4.16667 4.19266
0.0149 0.016 0.0024 0.00282 0.0107 0.0114 0.00232 0.00375 0.00401
2L:3171040-3171190:plus -30.8163 2.98165 3.66972 -1.33028 -27.5306 -24.8265 -15.1376 0.361111 1.95413
0.0103 0.0035 0.00306 0.0409 0.0351 0.0204 0.00922 0.0149 0.00964
2L:3303100-3303250:minus -21.9592 -2.66055 0.642202 6.47706 -17.0816 -8.72449 -9.17431 0.222222 2.47706
0.00279 0.0235 0.00935 0.00179 0.00334 0.00169 0.00264 0.0156 0.00801
2L:3303100-3303250:plus -23.4082 -4.20183 -1.81651 6.02752 -8.96939 -26.3776 -17.8073 0.0277778
1.12844 0.00623 0.0357 0.0205 0.00246 0.00226 0.0222 0.0145 0.0166 0.0137
2L:3303720-3303870:minus -32.7347 9.68807 6.50459 2.55963 -27.1939 -7.72449 -23.0826 5.75 -1.22936
0.0225 0.000156 0.000917 0.0111 0.0267 0.00068 0.0356 0.00187 0.0279
2L:3303720-3303870:plus -3.40816 0.605505 4.38532 7.63303 -17.7143 -9.0102 9.3945 2.59722 -0.844037
0.0000685 0.00834 0.0023 0.000745 0.00627 0.00208 0.0000159 0.00694 0.025
2L:3319860-3320010:minus -31.4796 13.4592 11.6881 0.559633 -18.5306 -8.72449 -11.2477 1.11111 -3.77982
0.0128 0.000064 0.0000527 0.0232 0.0118 0.00169 0.00393 0.0117 0.0525
2L:3319860-3320010:plus -33 13.4592 1.52294 4.34862 -17.6429 -8.53061 -14.5872 -4.05556 -4.66055
0.0258 0.000064 0.00687 0.00507 0.00562 0.00136 0.00824 0.0499 0.0667
2L:3354820-3354970:minus -3.40816 -0.321101 0.559633 9.44954 -18.7857 -8.53061 -13.0459 2.27778
1.37615 0.0000685 0.0115 0.00961 0.000326 0.0148 0.00136 0.00584 0.0078 0.0123
2L:3354820-3354970:plus -30.102 -1.26606 -3.40367 6.12844 -17.0408 -27.7857 -18.4771 -2.18056 -6.46789
0.00859 0.0156 0.0323 0.00231 0.00329 0.0466 0.0161 0.0313 0.0997
2L:3366140-3366290:minus -31.1837 2.40367 -2.10092 -0.46789 -26.9694 -27.1939 -17.9633 1.95833 2.63303
0.0109 0.00433 0.0223 0.0309 0.023 0.0327 0.0149 0.00875 0.00771
2L:3366140-3366290:plus -30.7041 6.99083 6.01835 7.43119 -8.37755 -17.5 -19.844 -7.63889 -3.61468
0.00993 0.000652 0.00114 0.000939 0.00106 0.00599 0.02 0.104 0.0509
2L:3366940-3367090:minus -24.3776 1.06422 3.6422 2.26606 -17.6735 -9.38776 -7.91743 4.80556 1.23853
0.00793 0.00707 0.00309 0.0126 0.00572 0.00266 0.00207 0.00286 0.0132
2L:3366940-3367090:plus -13.0306 0.908257 4.76147 3.13761 -18.1224 -26.602 -4.16514 5.06944 -2.52294
0.000567 0.00748 0.00196 0.00854 0.00782 0.0248 0.000782 0.00255 0.0391
2L:3373360-3373510:minus -22.8061 -1.66055 -1.04587 7.12844 -8.96939 -8.53061 -9.37615 2.65278
-0.0733945 0.00414 0.0176 0.0162 0.00118 0.00226 0.00136 0.00274 0.0068 0.0188
2L:3373360-3373510:plus -23.2245 -1.36697 -0.0275229 4.14679 -17.4184 -18.5612 -12.6697 1.33333
-4.15596 0.00576 0.0161 0.0117 0.00542 0.00457 0.0132 0.00536 0.0109 0.0578
2L:3376820-3376970:minus -32.7041 6.95413 0.651376 2.05505 -18.2245 -18.6327 -13.7339 1.375 5.29358
0.0222 0.000665 0.00932 0.0139 0.00877 0.0137 0.00684 0.0107 0.00278
2L:3376820-3376970:plus -30.7041 0.504587 -0.816514 1.56881 1.61224 -8.53061 -0.880734 5.01389
7.31193 0.00993 0.00865 0.0151 0.0172 0.0000631 0.00136 0.000346 0.00261 0.00112
2L:3414260-3414410:minus -23.6633 3.76147 5.05505 2.18349 -26.8571 -8.38776 -30.1009 1.51389 -5.11927
0.00665 0.00263 0.00174 0.0131 0.0198 0.00101 0.0922 0.0102 0.0746
2L:3414260-3414410:plus -14.551 0.284404 6.86239 7.6422 -8.64286 -8.45918 4.76147 4.40278
-0.0733945 0.00146 0.00935 0.000775 0.000721 0.00139 0.00111 0.0000627 0.0034 0.0188
2L:3449560-3449710:minus -24.449 1.38532 1.16514 -0.100917 -17.9796 -9.68367 -24.2936 3.26389
0.348624 0.00802 0.00629 0.0078 0.0277 0.00726 0.00342 0.0428 0.00539 0.0167
2L:3449560-3449710:plus -13.7449 0.944954 6.76147 6.27523 20.2041 -17.7143 -4.19266 0.875 3.62385
0.00107 0.00739 0.000811 0.00206 3.64e-07 0.00634 0.000788 0.0127 0.00523
2L:3450060-3450210:minus -3.77551 -1.62385 0.366972 -0.376147 -18.7857 -28.1122 -3.66055 5.72222
7.42202 0.000104 0.0174 0.0103 0.0299 0.0148 0.0565 0.000678 0.00189 0.00106
2L:3450060-3450210:plus -31.2143 0.394495 0.605505 4.6055 -26.7041 -26.6327 -23.0092 2.45833 0.385321
0.0111 0.00899 0.00946 0.00476 0.0193 0.0251 0.0351 0.00731 0.0166

2L:3457020-3457170:minus -32 2.00917 15.6239 2.14679 -26.9694 -26.5306 -16.1101 2.05556 9.34862
0.0171 0.005 5.44e-07 0.0133 0.023 0.023 0.0111 0.00845 0.000371
2L:3457020-3457170:plus -14.898 3.40367 2.07339 9.33028 -27.1224 -8.68367 -19.2202 1.72222 -1.21101
0.0016 0.003 0.00562 0.000374 0.0245 0.00157 0.0181 0.0095 0.0277
2L:3466080-3466230:minus -24.2959 -3.6055 15.5596 7.29358 20.4694 -18.1224 -12.3028 -5.36111 -1.6789
0.00781 0.0304 6.69e-07 0.00104 1.65e-07 0.00988 0.00493 0.0667 0.0306
2L:3466080-3466230:plus -31.5102 -1.9633 14.8532 5.59633 -8.67347 -17.3469 -22.0459 -4.05556 -3.66055
0.013 0.0192 2.28e-06 0.00331 0.00154 0.0058 0.0297 0.0499 0.0513
2L:3469940-3470090:minus -22.9592 13.844 1.7156 0.330275 -8.63265 -8.45918 -3.84404 -1.22222 3.20183
0.00475 9.98e-06 0.00642 0.0246 0.0013 0.00111 0.000714 0.024 0.0065
2L:3469940-3470090:plus -31.2143 14.7523 1.7156 3.19266 -26.9286 -25.898 -12.4128 3.90278 1.69725
0.0111 3.48e-06 0.00642 0.00829 0.0214 0.0214 0.00506 0.00418 0.0108
2L:3475680-3475830:minus -23.2653 2.6422 3.85321 3.20183 -27.2245 -18.9388 -17.8532 -7.56944 2.11009
0.00593 0.00397 0.00284 0.00824 0.0272 0.0154 0.0146 0.103 0.00898
2L:3475680-3475830:plus -21.8469 5.24771 3.27523 7.6055 -17.9286 -17.7551 -11.5505 -3.86111 3.58716
0.00247 0.00147 0.00357 0.000794 0.0071 0.00643 0.00418 0.0477 0.00534
2L:3477220-3477370:minus -30.102 3.83486 -1.49541 -0.623853 -18.9796 -9.09184 -18.5321 4.48611
3.31193 0.00859 0.00256 0.0186 0.0326 0.0155 0.00219 0.0162 0.00328 0.00618
2L:3477220-3477370:plus -32.0408 -0.165138 -1.98165 2.68807 -18.449 -9.79592 -21.1927 0.652778
-2.22936 0.0174 0.0109 0.0215 0.0104 0.0107 0.00377 0.0255 0.0136 0.0356
2L:3478200-3478350:minus -22.6633 5.46789 -0.715596 1.0367 -26.9592 -16.5 -17.4495 -2.125
1.88073 0.00384 0.00134 0.0146 0.0204 0.0215 0.00416 0.0137 0.0308 0.00987
2L:3478200-3478350:plus -30.5102 2.20183 -0.633028 4.05505 -8.70408 -17.898 -17.7339 12.4444
3.52294 0.00947 0.00466 0.0142 0.00566 0.00155 0.00726 0.0144 0.0000196 0.00558
2L:3478940-3479090:minus -40.2653 -1.69725 2.34862 3.94495 -27.2653 -8.30612 -10.4495 2.02778 5.81651
0.0383 0.0178 0.00508 0.00595 0.0302 0.000973 0.00335 0.00853 0.00204
2L:3478940-3479090:plus -12.1837 -4.6422 7.11009 6.89908 -26.2653 -36.1122 -10.8991 -6.58333 3.80734
0.000365 0.0401 0.000691 0.00134 0.018 0.0878 0.00366 0.0855 0.00484
2L:3480020-3480170:minus -21.5918 -0.440367 4.44954 6.11009 -28.1939 0.755102 -15.2202 7.77778
6.54128 0.00209 0.012 0.00224 0.00233 0.0482 0.000175 0.00937 0.000661 0.00151
2L:3480020-3480170:plus -24.5204 0.440367 0.46789 2.73394 -18.1837 -27.898 -12.3578 1.83333 2.02752
0.00813 0.00885 0.00992 0.0101 0.00835 0.0537 0.00499 0.00914 0.00925
2L:3514580-3514730:minus -24.1837 -2.49541 2.55963 7.33945 1.14286 9.79592 -22.3761 2.19444 -3.7156
0.00751 0.0224 0.0047 0.00102 0.000113 0.0000183 0.0315 0.00804 0.0518
2L:3514580-3514730:plus -33.1531 -1.77064 -0.568807 6.44037 10.398 -0.316327 -11.7064 1.56944
-4.84404 0.0273 0.0182 0.0139 0.00185 0.0000153 0.000524 0.00433 0.01 0.0701
2L:3530320-3530470:minus -13.2143 13.578 -0.972477 3.77064 -18.7143 -16.9796 -16.8624 8.16667
-0.357798 0.000714 0.0000133 0.0158 0.00637 0.0136 0.0044 0.0125 0.00053 0.021
2L:3530320-3530470:plus -23.6327 13.3878 -2.27523 4.11009 -8.86735 -16.7857 -15.1009 5.04167 4.83486
0.00661 0.0000869 0.0234 0.00553 0.00173 0.00426 0.00916 0.00258 0.00329
2L:357800-357950:minus -24.449 13.4592 -3.01835 5.06422 -26.8571 1.09184 -22.3486 1.875 -6.36697
0.00802 0.000064 0.029 0.00433 0.0198 0.000091 0.0314 0.00901 0.0972
2L:357800-357950:plus -32.1531 -5.3945 -2.54128 3.18349 -8.33673 -17.2755 -20.578 2.38889 -0.247706
0.0179 0.0489 0.0253 0.00837 0.00101 0.00544 0.0228 0.00749 0.0204
2L:3619180-3619330:minus -32.0714 5 5.93578 2.93578 -18.7143 0.683673 -16.7706 6.04167 -3.09174
0.0176 0.00163 0.00119 0.00927 0.0136 0.000184 0.0124 0.00162 0.0456
2L:3619180-3619330:plus -21.9592 3.90816 3.10092 7.70642 -27.3061 -27.2653 -10.7798 -2.09722 4.22936
0.00279 0.00102 0.00382 0.000708 0.0315 0.0333 0.00357 0.0306 0.00395
2L:3631560-3631710:minus -40.551 -4.11009 -2.09174 1.48624 -18.6735 -27.6735 -14.4495 7.43056 7.68807
0.0418 0.0348 0.0222 0.0177 0.0123 0.0443 0.008 0.000801 0.000841
2L:3631560-3631710:plus -13.7857 -2.21101 2.24771 2.97248 -18.2653 -8.72449 1.41284 3.58333 5.22018
0.00108 0.0206 0.00527 0.00909 0.00882 0.00169 0.000197 0.00475 0.00289
2L:3632220-3632370:minus -22.3673 1.04587 -3.27523 7 -27.1939 -18.7857 -12.4128 -1.70833 1.87156
0.00341 0.00712 0.0311 0.00132 0.0267 0.0146 0.00506 0.0275 0.00989

2L:3632220-3632370:plus -2.40816 1.86239 11.8991 4.05505 -27.1939 -17.2041 -14.1835 -2.66667
10.1284 0.0000315 0.00528 0.0000462 0.00566 0.0267 0.00507 0.00755 0.0355 0.000129
2L:3632960-3633110:minus -31.4796 1.15596 -5.46789 3.78899 -18.6837 -18.1224 -20.4037
4.33333 7.31193 0.0128 0.00684 0.0556 0.00632 0.0126 0.00988 0.0221 0.0035 0.00112
2L:3632960-3633110:plus -23.1837 2.20183 0.155963 3.16514 -27.6735 -19.1224 -13.2569
-2.45833 5.6055 0.00553 0.00466 0.011 0.00843 0.0375 0.0175 0.00613 0.0337 0.00232
2L:3639960-3640110:minus -31.9592 0.614679 0.981651 9.44954 -18.7857 -26.5306
0.798165 0.944444 3.42202 0.0167 0.00832 0.00832 0.000326 0.0148 0.023 0.000233 0.0124
0.00597
2L:3639960-3640110:plus -32.1837 9.88991 3.73394 -0.00917431 -18.0816 -16.0102 -24.3853
-3.22222 4.82569 0.0181 0.000141 0.00299 0.027 0.00759 0.00406 0.0434 0.0408 0.00331
2L:3656840-3656990:minus -3.30612 0.0733945 5.30275 10.0275 -18.6429 0.27551 1.0367 -1
1.87156 0.0000553 0.0101 0.00157 0.000156 0.0121 0.000257 0.000218 0.0226 0.00989
2L:3656840-3656990:plus -31.9694 2.04587 2.50459 6.45872 -27.1939 -27.602 -25.5963 1.95833 3.21101
0.0168 0.00494 0.0048 0.00182 0.0267 0.0416 0.0509 0.00875 0.00644
2L:3705660-3705810:minus -22.0408 -1 2.65138 2.19266 -18.7551 -27.5306 -12.6514 -4.625
-1.83486 0.00296 0.0143 0.00453 0.0131 0.0144 0.0374 0.00534 0.0568 0.0318
2L:3705660-3705810:plus -21.6939 3.05505 0.963303 4.0367 -8.86735 -26.8265 -4.6055 -0.611111
0.733945 0.00226 0.00341 0.00837 0.00578 0.00173 0.0285 0.000887 0.0201 0.0153
2L:3707000-3707150:minus -22.9286 1.05505 6.79817 10.7615 -18.1531 -26.5612 -9.79817
-6.18056 -1.15596 0.0046 0.00709 0.000798 0.0000826 0.00829 0.0237 0.00296 0.0789 0.0273
2L:3707000-3707150:plus -21.4694 -0.0550459 1.01835 2.58716 -26.5306 -7.79592 -3.88073
9.73611 5.22018 0.00201 0.0105 0.00821 0.011 0.0185 0.000723 0.000721 0.000197 0.00289
2L:3711660-3711810:minus -33.2653 2.95413 14.8624 2.51376 -26.5306 -9.72449 -26.6606
5.91667 -0.247706 0.0277 0.00354 2.2e-06 0.0113 0.0185 0.00352 0.058 0.00172 0.0204
2L:3711660-3711810:plus -40.8469 0.440367 11.9541 2.3578 -8.90816 -9.57143 -22.9633
-3.19444 -4.6055 0.0437 0.00885 0.0000446 0.0121 0.00192 0.00326 0.0349 0.0406 0.0656
2L:3711880-3712030:minus -33.2653 2.95413 14.8624 2.51376 -26.5306 -9.72449 -26.6606
5.91667 -0.247706 0.0277 0.00354 2.2e-06 0.0113 0.0185 0.00352 0.058 0.00172 0.0204
2L:3711880-3712030:plus -40.3673 5.02752 -3.63303 6.83486 -18.4898 -7.79592 -23.6606
-1.125 5.69725 0.0397 0.00161 0.0344 0.00142 0.0116 0.000723 0.039 0.0234 0.00225
2L:3713020-3713170:minus -40.4796 5.47706 14.8532 3.69725 -26.2347 -18.0102 -24.7248
0.972222 2.63303 0.0407 0.00133 2.28e-06 0.00665 0.0177 0.00812 0.0455 0.0123 0.00771
2L:3713020-3713170:plus -23 6.99083 1.3578 6.00917 -18.4898 -9.30612 -5.78899 4.84722 2.09174
0.00498 0.000652 0.00728 0.00255 0.0116 0.00257 0.00124 0.00281 0.00904
2L:3730320-3730470:minus -13.3265 2.2844 4.20183 7.63303 -18.2245 -28.3367 -8.31193 -5.70833 5.76147
0.000779 0.00452 0.00247 0.000745 0.00877 0.0606 0.00224 0.0717 0.0021
2L:3730320-3730470:plus -13.3367 3.94495 4.20183 10.8624 -8.30612 -8.16327 -10.9266 -4.97222 0.834862
0.0008 0.00245 0.00247 0.0000687 0.00097 0.000783 0.00368 0.0613 0.0149
2L:3771620-3771770:minus -4.44898 0.990826 6.23853 2.16514 -8.89796 -19.5714 -20.3761 3.23611 7.63303
0.000152 0.00726 0.00104 0.0132 0.00189 0.0202 0.022 0.00545 0.000928
2L:3771620-3771770:plus -22.6633 2.37615 3.9633 9.92661 -26.1939 -8.61224 4.02752 -8.30556 -0.623853
0.00384 0.00437 0.00272 0.000169 0.0175 0.00152 0.0000817 0.117 0.0232
2L:3772020-3772170:minus -31.7347 0.192661 7.05505 3.89908 -27.0408 -9.02041 -12.7431 1.40278 5.12844
0.0149 0.00965 0.000708 0.00604 0.0242 0.00213 0.00545 0.0106 0.00301
2L:3772020-3772170:plus -22.6735 -1.73394 3.07339 1.53211 -36.7143 -17.051 -16.0183 4.51389 2.11009
0.00385 0.018 0.00386 0.0174 0.0876 0.00479 0.0109 0.00324 0.00898
2L:3772880-3773030:minus -32.7347 -3.06422 -4.59633 -2.81651 -27.1939 -36.3776 -8.33028 2.11111 1.90826
0.0225 0.0262 0.0445 0.066 0.0267 0.104 0.00225 0.00828 0.00974
2L:3772880-3773030:plus -30.551 -6.17431 2.87156 2.7156 -17.4184 -26.5612 -15.4862 0.0555556
-2.62385 0.00959 0.0597 0.00417 0.0102 0.00457 0.0237 0.00987 0.0164 0.0402
2L:3776880-3777030:minus -20.6633 -1.33945 0.899083 3.13761 -17.8571 -18.3061 -5.75229 1.59722 -2.62385
0.00173 0.0159 0.00856 0.00854 0.00698 0.0114 0.00123 0.00993 0.0402
2L:3776880-3777030:plus -22.8163 1.01835 1.25688 6.36697 -18.449 -8.7551 -19.5138 0.694444 -2.94495

0.00418 0.00719 0.00755 0.00193 0.0107 0.00172 0.019 0.0134 0.0441
2L:3777760-3777910:minus -24.1531 -1.44037 7.27523 1.53211 -16.4184 -16.8265 -8.07339 6.91667 1.72477
0.00744 0.0164 0.000635 0.0174 0.00304 0.0043 0.00214 0.00105 0.0106
2L:3777760-3777910:plus -33 4.92661 0.733945 3.69725 -18.449 -19.1531 -3.40367 2.54167 5.81651
0.0258 0.00168 0.00906 0.00665 0.0107 0.0177 0.000632 0.00709 0.00204
2L:3778740-3778890:minus -31.7347 3.78899 1.00917 4.81651 -7.63265 -10.0918 -24.5046 8.65278 1.41284
0.0149 0.0026 0.00824 0.00451 0.000479 0.00404 0.0441 0.000397 0.0121
2L:3778740-3778890:plus -22.3265 3.56881 3.97248 9.59633 -17.4898 0.0204082 -10.8716 -8.125
0.963303 0.00337 0.00282 0.00271 0.000275 0.0053 0.000377 0.00364 0.114 0.0145
2L:3803420-3803570:minus -39.8776 1.00917 3.84404 6.00917 -7.93878 -9.72449 -15.4954 7.91667 0.66055
0.0341 0.00722 0.00286 0.00255 0.000767 0.00352 0.00989 0.000611 0.0156
2L:3803420-3803570:plus 6.55102 -1.42202 1.59633 2.55046 -17.3061 -9.38776 -8.75229 0.888889 4.66055
2.47e-06 0.0164 0.00669 0.0112 0.00389 0.00266 0.00244 0.0126 0.0035
2L:3813360-3813510:minus -22.9286 12.3119 10.4495 6.52294 -18.1531 -16.9388 -17.211 3.36111 -2.70642
0.0046 0.0000373 0.000108 0.00174 0.00829 0.00433 0.0132 0.00519 0.0412
2L:3813360-3813510:plus -13.2551 -2.92661 6.6422 3.44954 -26.0408 -18.9796 -10.8073 3.52778 9.56881
0.000751 0.0253 0.000858 0.00738 0.0171 0.0155 0.00359 0.00486 0.000303
2L:3862640-3862790:minus -34.1939 2 -2.75229 5.85321 -27.2653 0.173469 -32.844 2.94444 -2.43119
0.0314 0.00502 0.0269 0.00287 0.0302 0.000307 0.13 0.00609 0.038
2L:3862640-3862790:plus -32.0408 13.5306 -0.972477 3.77064 -18.7857 -8.57143 -18.578 1.97222
2.20183 0.0174 0.0000206 0.0158 0.00637 0.0148 0.00149 0.0163 0.0087 0.00869
2L:3867540-3867690:minus -30.6633 -2.10092 -3.33945 2.14679 -27.4592 -0.204082 -26.3119 3.55556
-4.3945 0.00976 0.02 0.0317 0.0133 0.0329 0.000463 0.0556 0.00481 0.062
2L:3867540-3867690:plus -33.8163 15.4771 0.559633 4.22018 -18.6429 -9.7551 -27.3578 4.15278 -0.229358
0.0299 1.08e-06 0.00961 0.00531 0.0121 0.00358 0.0633 0.00377 0.0202
2L:3872400-3872550:minus -24.2653 15.6514 16.3945 2.3578 -9.23469 -19.051 -2.30275 2.23611 -2.93578
0.00773 8.05e-07 6.75e-08 0.0121 0.00277 0.0165 0.000477 0.00792 0.0439
2L:3872400-3872550:plus -12.449 9.21101 0.146789 -0.834862 -18.7551 -17.5 -10.7615 -0.541667
-2.53211 0.000428 0.000201 0.011 0.0349 0.0144 0.00599 0.00356 0.0197 0.0392
2L:3896120-3896270:minus -20.7041 10.9083 0.302752 9.51376 -8.71429 -18.0102 -22.2752 -0.458333
1.89908 0.00175 0.0000847 0.0105 0.000292 0.00159 0.00812 0.031 0.0192 0.0098
2L:3896120-3896270:plus -40.2245 2.88991 5.99083 6.01835 -27.1633 -8.72449 -23.9908 5.29167 1.37615
0.0374 0.00362 0.00116 0.00248 0.025 0.00169 0.041 0.0023 0.0123
2L:3896680-3896830:minus -23.1939 1.75229 -1.55963 2.16514 -18.7143 -8.5 -22.3578 5.66667 -0.541284
0.00558 0.0055 0.0189 0.0132 0.0136 0.00126 0.0314 0.00194 0.0223
2L:3896680-3896830:plus -22.0714 -3.43119 2.66055 3.88991 -8.67347 -18.2755 -15.9266 3.97222 -0.899083
0.00299 0.029 0.00452 0.00608 0.00154 0.0112 0.0107 0.00407 0.0254
2L:404060-404210:minus -30.5204 0.321101 14.055 6.41284 -18.6837 -18.2755 1.85321 8.66667 -0.706422
0.00951 0.00922 6.79e-06 0.00189 0.0126 0.0112 0.000173 0.000394 0.024
2L:404060-404210:plus -13.9184 0.844037 2.68807 2.88991 -18.1837 0.530612 -5.22018 1.29167 1.6422
0.00112 0.00766 0.00447 0.00942 0.00835 0.000203 0.00106 0.011 0.0111
2L:414680-414830:minus -23.2143 6.49541 3.47706 5.02752 10.3571 -27.9286 -7.10092 -1.69444 9.87156
0.00559 0.000837 0.0033 0.00435 0.0000208 0.054 0.00173 0.0274 0.000217
2L:414680-414830:plus -3.93878 -1.84404 2.95413 4.75229 -8.71429 -27.602 -12.0367 -0.0416667 10.3486
0.000117 0.0186 0.00404 0.00461 0.00159 0.0416 0.00465 0.017 0.0000988
2L:414980-415130:minus -23.2143 1.46789 3.47706 3.57798 10.3571 -8.7551 -5.09174 2.08333 -8.11927
0.00559 0.0061 0.0033 0.00696 0.0000208 0.00172 0.00102 0.00837 0.139
2L:414980-415130:plus -31.8469 2.90826 -1.09174 3.3211 -17.8163 -9.45918 -8.05505 -2.23611 3.15596
0.0162 0.0036 0.0164 0.00782 0.00682 0.00279 0.00213 0.0317 0.00659
2L:4187760-4187910:minus -23.0408 2.13761 7.01835 3.90826 -8.16327 9.67347 -17.4679 2.40278 -0.440367
0.00512 0.00477 0.000721 0.00603 0.000873 0.0000217 0.0138 0.00746 0.0216
2L:4187760-4187910:plus -14.4082 1.59633 -1.47706 7.46789 -18.4184 -7.53061 -10.4128 7.19444 -2.52294
0.00136 0.00582 0.0185 0.000868 0.0101 0.000625 0.00333 0.000909 0.0391
2L:419720-419870:minus -31.6224 -2.11009 -2.55963 3.87156 1.87755 -0.0204082 -17.367 4.79167

6.95413 0.0137 0.0201 0.0254 0.00618 0.0000492 0.000411 0.0136 0.00288 0.00136
2L:419720-419870:plus -22.6224 1.55963 8.6422 3.70642 20.4694 19.3776 -11.2752 1.63889 -1.59633
0.00371 0.0059 0.000308 0.0066 1.65e-07 3.24e-07 0.00395 0.00978 0.0301
2L:4197400-4197550:minus -23.449 2.93578 1.13761 6.12844 -18.1531 -17.7143 -1.62385 4.83333 5.22018
0.00631 0.00356 0.00788 0.00231 0.00829 0.00634 0.000408 0.00283 0.00289
2L:4197400-4197550:plus -29.4082 0.0183486 3.66972 6.21101 -18.6837 -8.65306 -13.0459 -3.11111
11.3853 0.0084 0.0102 0.00306 0.00213 0.0126 0.00156 0.00584 0.0397 0.0000815
2L:4197960-4198110:minus -20.9286 -0.678899 15.5596 7.29358 -18.2245 -18.7857 -14.6606 3.41667
9.88073 0.00183 0.0129 6.69e-07 0.00104 0.00877 0.0146 0.00837 0.00508 0.000206
2L:4197960-4198110:plus -21.9694 3.20183 14.9266 2.41284 -18.3469 -8.53061 -22.0642 1.09722 -3.24771
0.00282 0.00323 1.9e-06 0.0118 0.00894 0.00136 0.0298 0.0118 0.0471
2L:421400-421550:minus -33.9286 15.0183 -0.908257 2.07339 -18.1531 -8.45918 -24.5229 3.86111
-4.63303 0.0303 1.96e-06 0.0155 0.0138 0.00829 0.00111 0.0442 0.00425 0.0661
2L:421400-421550:plus -31.7755 8.63303 4.6789 2.7156 -18.449 -19.1531 -29.2202 2.09722 9.66055
0.0157 0.000272 0.00203 0.0102 0.0107 0.0177 0.0814 0.00832 0.000268
2L:4226960-4227110:minus -22.6633 13.3578 3.2844 1.95413 -9.23469 -27.5612 -9.79817 1.04167 2.82569
0.00384 0.0000163 0.00356 0.0147 0.00277 0.0393 0.00296 0.012 0.0074
2L:4226960-4227110:plus -23.2959 13.5306 -5.77064 3.44954 0.653061 -7.5 -15.156 -0.263889 5.74312
0.00605 0.0000206 0.0599 0.00738 0.000239 0.000614 0.00926 0.0181 0.00215
2L:4227720-4227870:minus -41.2449 2.01835 0.284404 0.458716 -18.1122 0.897959 5.22018 3.30556 -0.59633
0.0498 0.00499 0.0105 0.024 0.00771 0.000144 0.0000534 0.0053 0.0229
2L:4227720-4227870:plus -5.16327 -0.0642202 3.75229 3.94495 2.14286 -17.9796 -24.9908 -0.986111
1.23853 0.000227 0.0105 0.00296 0.00595 0.0000316 0.00764 0.0471 0.0225 0.0132
2L:431080-431230:minus -13.0714 -0.422018 -0.862385 4.91743 -27.4898 -26.2653 -18.9266 1.51389
4.83486 0.000594 0.0119 0.0153 0.00447 0.0336 0.0218 0.0172 0.0102 0.00329
2L:431080-431230:plus -2.53061 7.66972 2.36697 7.34862 -8.30612 -8.60204 -6.84404 -0.388889 3.78899
0.0000326 0.000456 0.00505 0.000998 0.00097 0.00151 0.00163 0.0188 0.00487
2L:431700-431850:minus -30.5204 -4.29358 8.23853 7.20183 -27.2347 0.122449 -18.8991 -1.65278 -2.42202
0.00951 0.0366 0.000386 0.00112 0.0283 0.000312 0.0172 0.0271 0.0378
2L:431700-431850:plus -13.1429 1.55963 2.70642 7.80734 -17.5306 -26.898 -4.57798 -4.81944 3.40367
0.00065 0.0059 0.00444 0.00063 0.0054 0.0302 0.00088 0.0593 0.00603
2L:4332760-4332910:minus -32.8878 0.981651 -2.84404 7.80734 -18.5306 -0.27551 -6.56881 4.38889 -0.385321
0.0247 0.00729 0.0276 0.00063 0.0118 0.000502 0.00152 0.00342 0.0211
2L:4332760-4332910:plus -21.9286 2.07339 4.81651 3.78899 -17.3776 -9.45918 -12.3945 -0.625 -1.14679
0.0027 0.00488 0.00192 0.00632 0.00419 0.00279 0.00504 0.0202 0.0272
2L:4333780-4333930:minus -31.5102 2.95413 -0.0458716 4.91743 -27.0408 -18.5714 -17.7798 5.16667
3.16514 0.013 0.00354 0.0118 0.00447 0.0242 0.0136 0.0145 0.00244 0.00656
2L:4333780-4333930:plus -13.2245 -0.348624 1.41284 9.7156 -8.26531 -17.8265 -20.6789 -5.02778
-1.42202 0.000738 0.0116 0.00714 0.000242 0.000934 0.00699 0.0232 0.0621 0.0289
2L:4371820-4371970:minus -31.551 -0.697248 2.80734 1.63303 -17.4898 -7.93878 -23.4587 3.40278
1.90826 0.0134 0.013 0.00427 0.0167 0.0053 0.000736 0.0378 0.00511 0.00974
2L:4371820-4371970:plus 15.2245 4.15596 5.09174 7.00917 -17.7857 -8.5 -2.68807 -4.98611 4.80734
3.62e-07 0.00227 0.00171 0.00126 0.00676 0.00126 0.000525 0.0615 0.00334
2L:4372760-4372910:minus -24.2551 -2.06422 -1.55963 1.21101 -26.2653 -19.051 -8.21101 0.569444 -1.09174
0.00768 0.0198 0.0189 0.0194 0.018 0.0165 0.0022 0.014 0.0268
2L:4372760-4372910:plus -22.7347 1.57798 -1.62385 2.74312 -27.1939 -27.4082 -14.7064 10.6667 3.51376
0.00403 0.00586 0.0193 0.0101 0.0267 0.0356 0.00845 0.0000996 0.00566
2L:4377840-4377990:minus -21.8163 -0.761468 0.0733945 4.33945 -27.5612 -27.3776 -14.2385 10.1111
9.3945 0.00244 0.0133 0.0113 0.00509 0.0354 0.0351 0.00765 0.000151 0.000358
2L:4377840-4377990:plus -22.9898 4.46789 3.81651 1.48624 -9.23469 -18.2755 -9.27523 -0.236111
1.12844 0.00482 0.00201 0.00289 0.0177 0.00277 0.0112 0.00269 0.018 0.0137
2L:4382700-4382850:minus -31.449 1.97248 1.89908 5.31193 -9.23469 -27.602 -7.6422 2.11111 3.77064
0.0123 0.00507 0.006 0.00393 0.00277 0.0416 0.00195 0.00828 0.00491
2L:4382700-4382850:plus -32.0714 4.75229 4.74312 -0.201835 -28.0102 -18.6429 -14.2936 9.76389

0.229358 0.0176 0.0018 0.00198 0.0285 0.0449 0.0139 0.00774 0.000194 0.0173
2L:4386000-4386150:minus -41.551 0.174312 10.3119 3.90826 -18.0816 -18.5714 -22.4495 4.31944 8.11009
0.0607 0.00971 0.000118 0.00603 0.00759 0.0136 0.0319 0.00352 0.000597
2L:4386000-4386150:plus -32.4796 13.5229 -2.85321 6.66055 -18.0816 -17.7857 -16.2569 2.06944 2.99083
0.02 0.000014 0.0277 0.00158 0.00759 0.0068 0.0113 0.00841 0.00706
2L:4387020-4387170:minus -13.449 9.49541 6.07339 3.3578 -18.1531 -7.65306 -21.7339 -0.0833333
3.46789 0.000914 0.000173 0.00112 0.00767 0.00829 0.000647 0.0281 0.0172 0.00582
2L:4387020-4387170:plus -14.4082 -0.137615 2.34862 5.73394 -27.4082 -17.7143 -13.4862 0.166667
-3.13761 0.00136 0.0108 0.00508 0.00307 0.0321 0.00634 0.00646 0.0159 0.0461
2L:4393880-4394030:minus -30.7755 12.0642 -4.40367 2.11927 20.4694 -6.72449 -18.2752 3.20833 -2.72477
0.0102 0.0000445 0.0423 0.0135 1.65e-07 0.000544 0.0156 0.00551 0.0414
2L:4393880-4394030:plus -12.4082 14.2385 -1.15596 4.37615 -8.64286 -7.23469 -16.3853 2.125 -4.77982
0.000414 6.29e-06 0.0168 0.00504 0.00139 0.000557 0.0116 0.00824 0.069
2L:4403160-4403310:minus -23.9592 5.44954 -0.366972 3.76147 -8.37755 -9.57143 -14.5963 1.69444
4.77982 0.00708 0.00135 0.0131 0.00645 0.00106 0.00326 0.00826 0.0096 0.00339
2L:4403160-4403310:plus -33.9592 13.3878 -4.47706 0.522936 -17.7551 -8.72449 -17.8899 0.208333 -1.89908
0.0304 0.0000869 0.0431 0.0234 0.00658 0.00169 0.0147 0.0157 0.0323
2L:4442340-4442490:minus -34.4082 -0.486239 7.84404 3.97248 -19.051 -19.1939 -22.1927 1.77778
-0.798165 0.0319 0.0121 0.000476 0.00587 0.0162 0.0178 0.0305 0.00932 0.0246
2L:4442340-4442490:plus -13.449 4.3578 4.11927 7.47706 -18.4898 -18.0816 -7.90826 1.13889 0.330275
0.000914 0.0021 0.00255 0.00085 0.0116 0.00934 0.00206 0.0116 0.0168
2L:4444640-4444790:minus -39.9898 7.52294 2.44037 5.46789 -26.898 -9.94898 -22.1101 2.90278 -0.770642
0.0351 0.000492 0.00491 0.00362 0.0203 0.00393 0.0301 0.00619 0.0245
2L:4444640-4444790:plus -21.8878 3.58716 2.37615 7.77982 -18.4184 -9.53061 -9.34862 -1.68056 -2.3211
0.00258 0.0028 0.00503 0.000652 0.0101 0.0031 0.00273 0.0273 0.0365
2L:4446100-4446250:minus -29.9184 1.72477 -5.50459 1.69725 -17.0816 -27.4082 -14.0092 -0.0416667
1.69725 0.00854 0.00555 0.0561 0.0163 0.00334 0.0356 0.00727 0.017 0.0108
2L:4446100-4446250:plus -39.7653 -1.55046 0.100917 5.57798 -27.0408 -9.53061 -22.7339 3.69444 -3.24771
0.0339 0.017 0.0112 0.0034 0.0242 0.0031 0.0335 0.00455 0.0471
2L:4448800-4448950:minus -21.7347 -1.52294 6.54128 0.669725 -17.449 -27.898 -0.440367 8.125
-0.0733945 0.00235 0.0169 0.000901 0.0225 0.00484 0.0537 0.000313 0.000543 0.0188
2L:4448800-4448950:plus -33.3673 4.51376 3.61468 7.00917 -18.3776 0.0510204 -18.1009 3.77778
-3.52294 0.0282 0.00198 0.00313 0.00126 0.00926 0.000349 0.0152 0.0044 0.0499
2L:4454440-4454590:minus -31.7755 10.3486 4.95413 6.74312 -17.1122 -9.30612 -22.5413 7.95833 -3.99083
0.0157 0.000112 0.00181 0.0015 0.00341 0.00257 0.0324 0.000597 0.0556
2L:4454440-4454590:plus -32.898 1.14679 -0.642202 1.3945 -18.4184 -9.23469 -5.90826 4.31944
0.183486 0.0247 0.00686 0.0143 0.0184 0.0101 0.00238 0.00128 0.00352 0.0176
2L:4456920-4457070:minus -24.7551 0.944954 -3.12844 1.06422 -18.7245 -7.94898 -20.6606 3.23611 2
0.00828 0.00739 0.0299 0.0203 0.0136 0.00074 0.0231 0.00545 0.00941
2L:4456920-4457070:plus -33.4082 0.87156 -1.72477 1.84404 -17.8265 -18.2041 -6.49541 3.66667 4.88073
0.0283 0.00758 0.0199 0.0154 0.00691 0.0103 0.00149 0.0046 0.00324
2L:4458260-4458410:minus -32.4796 0.275229 0.284404 3.58716 20.4694 0.469388 -11.7798 3.625 1.23853
0.02 0.00938 0.0105 0.00694 1.65e-07 0.000212 0.00439 0.00468 0.0132
2L:4458260-4458410:plus -40.7347 -0.697248 -3.3578 1.69725 -18.5612 -27.4082 -16.2018 0.75 4.25688
0.0429 0.013 0.0319 0.0163 0.0119 0.0356 0.0112 0.0132 0.00391
2L:4459720-4459870:minus -39.9898 -2.04587 3.56881 3.14679 11.6531 -19.051 -18.6514 -0.138889
-4.25688 0.0351 0.0197 0.00318 0.00847 1.55e-06 0.0165 0.0165 0.0175 0.0593
2L:4459720-4459870:plus -22.4796 -2.11009 3.7156 4.22018 -16.4898 -9.45918 -12.6147 -2.97222 0.495413
0.00352 0.0201 0.00301 0.00531 0.00312 0.00279 0.00529 0.0384 0.0162
2L:4463020-4463170:minus -23.5204 14.945 3.29358 2.75229 -27.1939 -8.30612 -22.6972 1.18056 -4.34862
0.00645 2.29e-06 0.00354 0.0101 0.0267 0.000973 0.0333 0.0114 0.0612
2L:4463020-4463170:plus -31.7041 14.1193 14.2018 7.29358 -18.4184 -8.02041 -16.8532 3.68056 -0.284404
0.0146 7.36e-06 5.71e-06 0.00104 0.0101 0.000761 0.0125 0.00457 0.0207
2L:4463460-4463610:minus -33.2245 13.9083 0.293578 6.56881 -8.93878 -17.0102 -26.844 5.68056 -2.59633

0.0276	9.27e-06	0.0105	0.0017	0.00216	0.00453	0.0593	0.00193	0.0398
2L:4463460-4463610:plus	-30.4388	13.4592	1.48624	2.50459	-8.93878	-9.79592	-19.8073	1.69444	3.56881
0.00923	0.000064	0.00696	0.0114	0.00216	0.00377	0.0199	0.0096	0.00543
2L:452200-452350:minus	-32.8469	5.76147	0.458716	11.0183	-27.0816	-19.4184	-14.4128	3.13889	4.87156
0.0243	0.00118	0.00995	0.000052	0.0243	0.0198	0.00794	0.00566	0.00325
2L:452200-452350:plus	-24.3776	1.88073	5.14679	6.73394	-18.4184	-17.2755	-13.7339	1.69444	-6.2844
0.00793	0.00524	0.00167	0.00152	0.0101	0.00544	0.00684	0.0096	0.0954
2L:452980-453130:minus	-32.0408	0	1.90826	6.21101	11.4286	-9.53061	-14.2477	1	-0.201835
0.0174	0.0103	0.00598	0.00213	1.97e-06	0.0031	0.00766	0.0121	0.0199
2L:452980-453130:plus	-31.5816	2.78899	4.86239	2.49541	-17.9388	-9.68367	-9.17431	-1.93056	-0.981651
0.0135	0.00376	0.00188	0.0114	0.00715	0.00342	0.00264	0.0293	0.026
2L:4682680-4682830:minus	-23.449	9.44954	3.53211	9.33028	1.91837	9.23469	-13.7156	-0.958333
-0.0183486	0.00631	0.000177	0.00323	0.000374	0.0000382	0.0000427	0.00681	0.0223	0.0185
2L:4682680-4682830:plus	-33.3367	-0.247706	0.321101	2.66972	-18.4898	-17.3469	-25.0183	-0.388889
-1.63303	0.0281	0.0112	0.0104	0.0106	0.0116	0.0058	0.0473	0.0188	0.0304
2L:4690700-4690850:minus	-24.1837	-3.70642	6.79817	2.69725	-28.0102	-18.2041	-21.0367	3.84722	-0.119266
0.00751	0.0313	0.000798	0.0104	0.0449	0.0103	0.0248	0.00428	0.0192
2L:4690700-4690850:plus	-32.4694	1.44037	-0.0275229	0.862385	10.3878	-26.5306	-16.1651	1.125
5.90826	0.0196	0.00617	0.0117	0.0214	0.0000174	0.023	0.0112	0.0117	0.00198
2L:469120-469270:minus	-23.3265	5.44954	2.11009	7.88991	-17.1531	-19.3469	-10.5229	7.86111	1.02752
0.00607	0.00135	0.00554	0.00062	0.00364	0.0194	0.0034	0.000631	0.0141
2L:469120-469270:plus	-32.3673	-0.908257	13.2385	9.87156	-18.7143	-26.5306	-14.1101	8.59722	3.88073
0.0189	0.0139	0.000016	0.000195	0.0136	0.023	0.00743	0.000411	0.00475
2L:470360-470510:minus	-22.9694	-1.80734	0.733945	6.66055	-19.3163	-18.7551	-4.62385	-3.81944	11.8991
0.0048	0.0184	0.00906	0.00158	0.0165	0.0141	0.000891	0.0472	0.0000255
2L:470360-470510:plus	-32.7347	7.24771	-0.165138	3.3578	-17.1837	-36.449	-2.81651	2.06944	1.24771
0.0225	0.00057	0.0122	0.00767	0.00366	0.109	0.000542	0.00841	0.0131
2L:471400-471550:minus	-22.7041	4.05505	6.09174	2.19266	-18.7551	-19.1531	-15.0367	-2.01389	0.431193
0.00395	0.00235	0.00111	0.0131	0.0144	0.0177	0.00904	0.0299	0.0164
2L:471400-471550:plus	-22.898	-0.440367	5.11009	2.33028	-18.0816	-8.87755	-20.0917	0.444444	-7.33028
0.00442	0.012	0.0017	0.0123	0.00759	0.00198	0.0209	0.0145	0.121
2L:472180-472330:minus	-4.29592	2.6055	-0.752294	3.87156	-17.3776	-16.9796	9.15596	-3.875
11.3853	0.000135	0.00402	0.0148	0.00618	0.00419	0.0044	0.0000171	0.0478	0.0000815
2L:472180-472330:plus	-23.8061	2.33028	-0.366972	2.47706	-17.2245	-27.6735	-10.0917	3.40278	3.20183
0.0068	0.00445	0.0131	0.0115	0.00384	0.0443	0.00313	0.00511	0.0065
2L:472880-473030:minus	-32.7755	-1.9633	-0.376147	2.04587	-27.5306	-10.0204	-30.8532	8.38889
1.54128	0.0234	0.0192	0.0131	0.014	0.0351	0.00399	0.102	0.000466	0.0115
2L:472880-473030:plus	-24.3061	5.21101	5.9633	7.20183	20.4694	-27.1224	-20.3303	1.94444	7.22018
0.00782	0.00149	0.00117	0.00112	1.65e-07	0.0325	0.0218	0.00879	0.00119
2L:479140-479290:minus	-12	-2.33028	5.11009	5.21101	-18.7551	-26.602	-10.8991	0.402778	11.4771
0.000305	0.0214	0.0017	0.0042	0.0144	0.0248	0.00366	0.0147	0.0000679
2L:479140-479290:plus	-21.5102	-2.98165	-1.19266	1.77982	-26.9694	-18.0102	-10.4954	2.95833	9.87156
0.00202	0.0257	0.0169	0.0158	0.023	0.00812	0.00338	0.00606	0.000217
2L:479660-479810:minus	-13.4796	-3.78899	5.44954	7.27523	-17.8571	-17.2041	22.5321	-3.875	0.908257
0.000935	0.032	0.00147	0.00109	0.00698	0.00507	2.46e-08	0.0478	0.0147
2L:479660-479810:plus	-14.1837	2.6422	1.50459	5.94495	-26.7041	-26.602	-15.2477	1.34722	7.24771
0.00124	0.00397	0.00692	0.00261	0.0193	0.0248	0.00943	0.0108	0.00116
2L:480240-480390:minus	-21.7755	0.100917	14.2752	-0.633028	-18.3776	-27.8571	-8.79817	-0.986111
-3.62385	0.00241	0.00996	5.02e-06	0.0327	0.00926	0.0508	0.00246	0.0225	0.051
2L:480240-480390:plus	-13.5204	1.9633	6.61468	8.6055	-18.4184	-17.7857	-9.22936	-2.20833	9.04587
0.00097	0.00509	0.000869	0.000567	0.0101	0.0068	0.00267	0.0315	0.000423
2L:480860-481010:minus	-31.2143	-1.16514	14.3211	3.10092	-18.1122	-9.38776	-22.3394	-3.19444	11.3853
0.0111	0.0151	4.76e-06	0.00865	0.00771	0.00266	0.0313	0.0406	0.0000815
2L:480860-481010:plus	-33.8571	-3.23853	1.56881	2.30275	-18.4184	-19.3469	-17.2385	2.83333	2.75229

0.0301 0.0275 0.00676 0.0124 0.0101 0.0194 0.0133 0.00635 0.00748
2L:481260-481410:minus -32.551 -1.6789 6.33028 1.91743 -18.449 -9.27551 -6.81651 2.45833 3.20183
0.0207 0.0177 0.000996 0.015 0.0107 0.00244 0.00161 0.00731 0.0065
2L:481260-481410:plus -30.6633 -0.247706 -6.22018 4.34862 -27.5306 -7.57143 -21.5688 1.01389 -1.17431
0.00976 0.0112 0.0667 0.00507 0.0351 0.000644 0.0273 0.0121 0.0275
2L:4818200-4818350:minus -23.1837 3.61224 15 7.29358 -27.898 9.60204 -13.5229 0.444444 -0.642202
0.00553 0.00167 1.62e-06 0.00104 0.04 0.0000248 0.00652 0.0145 0.0234
2L:4818200-4818350:plus -22.8061 2.27523 1.57798 1.82569 11.4286 -9.57143 -17.0183 2.41667 6.95413
0.00414 0.00454 0.00674 0.0156 1.97e-06 0.00326 0.0129 0.00742 0.00136
2L:482060-482210:minus -30.3673 -0.798165 4.06422 9.49541 -17.6735 -8.45918 -17.367 1.44444
-2.25688 0.00897 0.0134 0.00261 0.000308 0.00572 0.00111 0.0136 0.0105 0.0357
2L:482060-482210:plus -13.4898 -6.12844 0.311927 0.642202 -8.0102 -27.7041 -20.4587 5.23611 -1.19266
0.000946 0.059 0.0104 0.0227 0.000826 0.0445 0.0223 0.00236 0.0276
2L:4830520-4830670:minus -24.2959 13.8349 4.66055 2.11009 -18.5612 -18.0102 -10.8073 7.13889 3.46789
0.00781 0.0000102 0.00205 0.0136 0.0119 0.00812 0.00359 0.000935 0.00582
2L:4830520-4830670:plus -13.4796 8.80734 4.13761 2.49541 -26.898 -9.0102 -0.770642 6.47222
3.45872 0.000935 0.000248 0.00254 0.0114 0.0203 0.00208 0.000337 0.00132 0.00591
2L:4837920-4838070:minus -21.6633 -2.38532 4.75229 2.75229 -18.4592 -27.5612 6.52294 0.736111 1.16514
0.00223 0.0217 0.00197 0.0101 0.0109 0.0393 0.0000355 0.0132 0.0135
2L:4837920-4838070:plus -19.5918 16.3761 0.926606 4.66972 -18.1224 -9.16327 -2.36697 -1.06944 2.6789
0.00163 1.59e-07 0.00848 0.00466 0.00782 0.00223 0.000485 0.023 0.00763
2L:484660-484810:minus -1.95918 -0.311927 -0.706422 6.86239 -8.86735 -26.602 -15.0367 -2.79167
-2.22936 0.0000238 0.0115 0.0146 0.00136 0.00173 0.0248 0.00904 0.0367 0.0356
2L:484660-484810:plus -22.6633 -1.30275 0.165138 9.23853 -7.93878 -18.051 -22.4954 -0.833333 3.87156
0.00384 0.0158 0.011 0.000424 0.000767 0.00886 0.0322 0.0215 0.00477
2L:4846940-4847090:minus -31.3673 -5.3945 5.02752 9.59633 -26.9694 0.0918367 -26.2202 -1.15278
-7.36697 0.0116 0.0489 0.00176 0.000275 0.023 0.000323 0.055 0.0236 0.121
2L:4846940-4847090:plus -30.9286 6.47706 5.95413 7.37615 -27.0816 -17.3061 -10.7064 6.59722 3.77064
0.0104 0.000843 0.00118 0.000951 0.0243 0.00548 0.00352 0.00124 0.00491
2L:4847240-4847390:minus -22.4898 3.90816 4.75229 7.80734 -17.4898 -9.42857 -14.6972 1.36111 -1.55046
0.00353 0.00102 0.00197 0.00063 0.0053 0.0027 0.00843 0.0108 0.0297
2L:4847240-4847390:plus -23.1837 2.83486 4.61468 6.02752 -17.7857 -18.0816 -12.7706 -1.09722 4.7156
0.00553 0.0037 0.00209 0.00246 0.00676 0.00934 0.00549 0.0232 0.00345
2L:4847600-4847750:minus -22.2653 13.8349 6.26606 0.559633 -26.898 -8.45918 -20.7156 1.26389 3.46789
0.00331 0.0000102 0.00103 0.0232 0.0203 0.00111 0.0234 0.0111 0.00582
2L:4847600-4847750:plus -34.3061 7.40367 7.66055 6.00917 -28.1939 9.53061 -16.8624 3.625 -4.87156
0.0317 0.000524 0.000523 0.00255 0.0482 0.0000316 0.0125 0.00468 0.0708
2L:4850480-4850630:minus -14.2143 4.90826 13.9541 3.43119 -17.7143 -29.1939 -10.8991 5.02778 3.88991
0.00124 0.00169 7.63e-06 0.00749 0.00627 0.0738 0.00366 0.00259 0.00467
2L:4850480-4850630:plus -32.0714 3.68367 5.74312 3.69725 -8.93878 -10.0918 -15.6972 1.26389 -0.394495
0.0176 0.00144 0.00129 0.00665 0.00216 0.00404 0.0103 0.0111 0.0212
2L:485500-485650:minus -30.1429 1.00917 2.3945 3.95413 0.908163 -27.3367 -10.3119 0.277778 -1.59633
0.00866 0.00722 0.00499 0.00593 0.000153 0.0347 0.00326 0.0153 0.0301
2L:485500-485650:plus -22.0306 0.0642202 -0.33945 2.83486 -8.86735 -16.7143 -23.3119 -5.58333 -2.45872
0.00293 0.0101 0.0129 0.0097 0.00173 0.00419 0.0369 0.0698 0.0383
2L:485800-485950:minus -22 0.614679 0.192661 3.09174 -8.60204 -18.051 -11.5963 0.569444 7.42202
0.00291 0.00832 0.0109 0.00867 0.00125 0.00886 0.00422 0.014 0.00106
2L:485800-485950:plus -23.1939 1.73394 2.44954 2.9633 -18.9796 -8.45918 -26.8899 4.38889 4.30275
0.00558 0.00553 0.00489 0.00914 0.0155 0.00111 0.0597 0.00342 0.00388
2L:488160-488310:minus -32.1429 -2.22936 14.2752 3.20183 -18.1224 -27.1224 -4.52294 4.29167 5.69725
0.0179 0.0208 5.02e-06 0.00824 0.00782 0.0325 0.000866 0.00356 0.00225
2L:488160-488310:plus -3.96939 1.50459 -1.80734 6.00917 -27.4184 -19.2347 -14.1835 -1.18056 -0.486239
0.000119 0.00602 0.0204 0.00255 0.0323 0.018 0.00755 0.0238 0.0219
2L:4883040-4883190:minus -23.1531 13.3878 -2.51376 9.44037 -18.4898 -8.45918 -16.2661 -2.25 5.18349

0.00542 0.0000869 0.0251 0.000339 0.0116 0.00111 0.0114 0.0319 0.00295
2L:4883040-4883190:plus -24.898 3.82569 7.73394 2.50459 -27.5612 -18.0102 -11.4679 -0.833333
3.09174 0.00831 0.00257 0.000504 0.0114 0.0354 0.00812 0.00411 0.0215 0.00678
2L:4886840-4886990:minus -13.2245 1.42202 -2.13761 6.47706 -17.0816 -7.5 -3.66972 -4.59722 -2.21101
0.000738 0.00621 0.0225 0.00179 0.00334 0.000614 0.00068 0.0564 0.0354
2L:4886840-4886990:plus -4.41837 -1.20183 -1.00917 1.3578 -18.449 -27.6327 -12.2018 -0.138889
2.6422 0.000148 0.0153 0.016 0.0186 0.0107 0.043 0.00482 0.0175 0.00768
2L:4887740-4887890:minus -23.0714 0.761468 2.52294 2.06422 -18.2245 -17.2755 -2.27523 -7.26389 4.55963
0.0052 0.00789 0.00476 0.0139 0.00877 0.00544 0.000474 0.0972 0.00357
2L:4887740-4887890:plus -22.551 -2.13761 3.22018 7.80734 -17.3776 -17.7857 -23.844 -9.20833 2.53211
0.00359 0.0202 0.00365 0.00063 0.00419 0.0068 0.0401 0.136 0.00791
2L:4891860-4892010:minus -33.1939 0.348624 2.23853 5.47706 -19.3163 -17.5714 -24.4495 0.263889 0.247706
0.0274 0.00914 0.00529 0.0036 0.0165 0.00616 0.0438 0.0154 0.0172
2L:4891860-4892010:plus -31.2449 1.37615 7.65138 9.7156 -17.1531 -9.53061 -8.82569 5.58333 3.66972
0.0111 0.00631 0.000526 0.000242 0.00364 0.0031 0.00248 0.00202 0.00514
2L:490260-490410:minus -32 0.761468 2.86239 4.0367 -17.6837 0.244898 -22.9908 4.06944 -2.61468
0.0171 0.00789 0.00418 0.00578 0.0058 0.000285 0.035 0.00391 0.0401
2L:490260-490410:plus -23 -1.06422 9.81651 3.42202 -9.45918 -17.2041 -15.633 4.69444 3.46789 0.00498
0.0146 0.000158 0.00751 0.00287 0.00507 0.0101 0.003 0.00582
2L:490620-490770:minus -21.6224 0.733945 -0.93578 6.54128 -26.9286 -17.7143 -16.7339 4.77778 8.88073
0.00213 0.00797 0.0156 0.00173 0.0214 0.00634 0.0123 0.00289 0.000468
2L:490620-490770:plus -12.2245 -0.486239 3 7.27523 -26.6327 -25.7551 -9.02752 5.90278 7.62385
0.000376 0.0121 0.00397 0.00109 0.0188 0.0208 0.00257 0.00174 0.000941
2L:4913440-4913590:minus -22.0816 0.678899 -1.19266 5.74312 -18.7143 -9.5 -15.2569 0.819444 -3.93578 0.003
0.00813 0.0169 0.00302 0.0136 0.00294 0.00944 0.0129 0.0547
2L:4913440-4913590:plus -31.8469 -1.30275 6.44037 5.3945 -26.9286 -26.898 -7.80734 7.31944 4.7156
0.0162 0.0158 0.000945 0.00379 0.0214 0.0302 0.00202 0.00085 0.00345
2L:491660-491810:minus -14.2959 3.11927 6.27523 6.42202 -17.4592 -7.23469 -15.1376 -0.0555556
3.20183 0.00129 0.00333 0.00102 0.00188 0.00491 0.000557 0.00922 0.017 0.0065
2L:491660-491810:plus -4 3.21101 -1.37615 2.3578 -18.4184 10.5714 -9.95413 1.81944 6.12844 0.000121
0.00322 0.0179 0.0121 0.0101 4.67e-06 0.00305 0.00919 0.00174
2L:492400-492550:minus -23.4898 -4.7156 4.86239 7.15596 -18.4898 -18.6327 -17.0183 -5.15278 3.72477
0.00639 0.0409 0.00188 0.00117 0.0116 0.0137 0.0129 0.0638 0.00497
2L:492400-492550:plus -22.9898 3.59633 -3.37615 -0.385321 -18.1122 -17.0816 -11.6606 3.625 1.73394
0.00482 0.00279 0.032 0.03 0.00771 0.00487 0.00428 0.00468 0.0106
2L:494240-494390:minus -22.2959 0.486239 -2.14679 3.41284 -17.7551 -17.3469 -6.56881 0.0833333
6.49541 0.00335 0.00871 0.0226 0.00756 0.00658 0.0058 0.00152 0.0163 0.00154
2L:494240-494390:plus -30.6633 3.24771 1.61468 0.917431 -8.60204 -26.1224 -7.10092 2.43056 2.75229
0.00976 0.00317 0.00665 0.021 0.00125 0.0217 0.00173 0.00738 0.00748
2L:4945480-4945630:minus -33.1531 1.50459 15.6239 9.7156 -26.6735 -17.2755 -15 7.33333 -3.91743
0.0273 0.00602 5.44e-07 0.000242 0.0191 0.00544 0.00897 0.000844 0.0544
2L:4945480-4945630:plus -31.6633 3.25688 10.3119 3.90826 -18.3776 -8.42857 -16.4037 2.31944 2.85321
0.0141 0.00316 0.000118 0.00603 0.00926 0.00104 0.0116 0.00769 0.00733
2L:4949720-4949870:minus -15.0102 0.46789 15.6239 5.48624 -28 -17.5714 -14.2202 -0.152778 0.385321
0.00162 0.00876 5.44e-07 0.00357 0.0444 0.00616 0.00761 0.0175 0.0166
2L:4949720-4949870:plus -31.7755 2.62385 1.02752 -1.19266 -8.64286 -27.1224 -15.0642 5.02778 -5.12844
0.0157 0.00399 0.00819 0.0391 0.00139 0.0325 0.00909 0.00259 0.0748
2L:495340-495490:minus -38.1837 7.34862 0.587156 4.0367 -26.4898 -19.1531 -17.4679 1.94444 3.57798
0.032 0.000541 0.00952 0.00578 0.0183 0.0177 0.0138 0.00879 0.00536
2L:495340-495490:plus -30.102 -3.27523 14.0183 7.34862 -17.3061 0.612245 -12.7156 -0.125 -2.68807
0.00859 0.0278 7.05e-06 0.000998 0.00389 0.000187 0.00542 0.0174 0.041
2L:4965700-4965850:minus -34.2245 1.20183 -0.577982 5.27523 -27.2653 -8.72449 -9.62385 4.61111
-2.43119 0.0315 0.00673 0.014 0.00402 0.0302 0.00169 0.00287 0.00311 0.038
2L:4965700-4965850:plus -23.9184 2.6055 3.56881 1.84404 -18.4184 -37.1837 -11.422 0.902778 5.90826

0.00695 0.00402 0.00318 0.0154 0.0101 0.14 0.00407 0.0125 0.00198
2L:4967720-4967870:minus -14.5612 5.73394 1.44037 -0.220183 20.4694 -17.9796 -20.6697 1.51389
2.58716 0.00147 0.00119 0.00707 0.0287 1.65e-07 0.00764 0.0232 0.0102 0.00779
2L:4967720-4967870:plus -33.2653 2.99083 -3.58716 -0.110092 -27.7551 -8.79592 -19.6147 5.80556
-0.761468 0.0277 0.00349 0.0339 0.0277 0.0385 0.0019 0.0193 0.00182 0.0244
2L:4968900-4969050:minus -21.4796 1.09174 5.40367 1.92661 -27.3061 0.316327 -23.2294 3.30556 2.54128
0.00202 0.007 0.0015 0.0148 0.0315 0.000243 0.0364 0.0053 0.00787
2L:4968900-4969050:plus 15 3.83486 -1.85321 6.00917 -8.64286 19.0816 -4.56881 6.22222 3.45872
5.44e-07 0.00256 0.0207 0.00255 0.00139 1.12e-06 0.000877 0.00149 0.00591
2L:4971480-4971630:minus -22.8878 5.11927 1.73394 5.43119 1.14286 0.0918367 -13.4312 2.47222
-5.0367 0.00441 0.00155 0.00638 0.00371 0.000113 0.000323 0.00638 0.00727 0.0734
2L:4971480-4971630:plus -32.7041 -0.926606 -1.3211 5.86239 10.6224 -18.1224 -24.4495 -0.305556
1.7156 0.0222 0.014 0.0176 0.00282 0.0000113 0.00988 0.0438 0.0184 0.0107
2L:4974000-4974150:minus -23 -1.57798 -0.0458716 6.00917 -18.2653 -9.45918 -11.2844 2.65278 6.07339
0.00498 0.0171 0.0118 0.00255 0.00882 0.00279 0.00396 0.0068 0.0018
2L:4974000-4974150:plus -13.2653 -2.6422 15.6239 2.07339 -8.64286 -26.4898 -10.3211 -5.11111 9.88073
0.000759 0.0233 5.44e-07 0.0138 0.00139 0.0225 0.00327 0.0632 0.000206
2L:4977080-4977230:minus -23.449 13.4592 2.52294 4.0367 -27.898 -8.5 -8.77064 3.15278 -4.29358
0.00631 0.000064 0.00476 0.00578 0.04 0.00126 0.00245 0.00563 0.06
2L:4977080-4977230:plus -14.449 6.46789 -3.36697 3.50459 -18.6837 -8.5 -8.93578 -0.944444 1.48624
0.0014 0.000847 0.032 0.00713 0.0126 0.00126 0.00253 0.0222 0.0118
2L:4979300-4979450:minus -32.1429 -1.42202 14.8532 3.13761 -18.449 -19.2755 -1.41284 8.19444 5.95413
0.0179 0.0164 2.28e-06 0.00854 0.0107 0.0185 0.000389 0.000522 0.00192
2L:4979300-4979450:plus -32.0714 -3.36697 14.9633 7.29358 -18.5612 -18.0102 -21.5138 7.66667 5.69725
0.0176 0.0285 1.77e-06 0.00104 0.0119 0.00812 0.027 0.000704 0.00225
2L:4981580-4981730:minus -22.6939 -1.58716 3.68807 6.12844 -18.1837 -19.0816 -19.6055 -0.0138889
-2.9633 0.00388 0.0172 0.00304 0.00231 0.00835 0.017 0.0193 0.0168 0.0442
2L:4981580-4981730:plus -14.6327 -0.678899 1.72477 7.15596 -8.93878 -18.7857 -3.16514 -3.75
1.9633 0.0015 0.0129 0.0064 0.00117 0.00216 0.0146 0.000593 0.0464 0.00951
2L:4997820-4997970:minus -23.1939 -0.385321 4.42202 1.97248 -27.2653 -17.7551 -18.5872 3.25 3.08257
0.00558 0.0117 0.00226 0.0146 0.0302 0.00643 0.0164 0.00542 0.00681
2L:4997820-4997970:plus -4.86735 -3.34862 3.30275 4.3211 -26.9694 -18.7551 -13.5505 -4.20833 -0.862385
0.000202 0.0284 0.00353 0.00514 0.023 0.0141 0.00656 0.0517 0.0251
2L:5000240-5000390:minus -30.4796 1.75229 -4.86239 1.68807 -7.93878 -17.5612 -22.2844 -2.375 4.66055
0.00941 0.0055 0.0477 0.0164 0.000767 0.00604 0.031 0.0329 0.0035
2L:5000240-5000390:plus -21.6633 -1.48624 4.26606 6.69725 -17.9796 -17.8571 -12.6514 -0.166667
5.6055 0.00223 0.0167 0.00241 0.00156 0.00726 0.00719 0.00534 0.0176 0.00232
2L:5001400-5001550:minus -31.4082 2.63303 8.81651 6.56881 -8.16327 -0.244898 -17.5872 1.30556
-9.33028 0.0119 0.00398 0.000281 0.0017 0.000873 0.000496 0.014 0.011 0.175
2L:5001400-5001550:plus -32.0408 8.73394 0.385321 5.42202 -26.9286 -18.2755 -23.9358 -1.66667 -4.52294
0.0174 0.000258 0.0102 0.00374 0.0214 0.0112 0.0407 0.0272 0.0642
2L:5007760-5007910:minus -24.2245 2.40367 -3.93578 0.357798 -17.7551 -8.79592 -19.2936 3.875 -0.12844
0.00763 0.00433 0.0373 0.0245 0.00658 0.0019 0.0183 0.00423 0.0193
2L:5007760-5007910:plus -31.2551 1.52294 3.85321 1.91743 -27.9592 -36.7449 -17.5963 3.86111 8.20183
0.0113 0.00598 0.00284 0.015 0.0416 0.119 0.0141 0.00425 0.000539
2L:5009700-5009850:minus -22.6633 11.7156 6 3.54128 -26.9286 -18.2755 -11.9541 -5.75 3.98165
0.00384 0.0000546 0.00115 0.00702 0.0214 0.0112 0.00456 0.0723 0.00439
2L:5009700-5009850:plus -32.1939 -2.9633 1.47706 9.08257 -28.602 -0.173469 -13.9266 2.55556
-3.83486 0.0181 0.0255 0.00698 0.000444 0.0631 0.000456 0.00714 0.00705 0.0533
2L:5010900-5011050:minus -22.1122 13.4592 1.49541 6.41284 -9.2449 -10.0204 -24.844 3.66667 -3.51376
0.00304 0.000064 0.00694 0.00189 0.00278 0.00399 0.0462 0.0046 0.0498
2L:5010900-5011050:plus -31.7449 1.13761 0.926606 5.37615 1.14286 -17.7857 -7.20183 -0.0694444
3.83486 0.0152 0.00689 0.00848 0.00384 0.000113 0.0068 0.00177 0.0171 0.00481
2L:502120-502270:minus -30.1735 1.76147 1.0367 4.6055 -7.67347 -19.1224 -13.0642 -0.305556

-2.13761 0.00869 0.00548 0.00816 0.00476 0.00055 0.0175 0.00587 0.0184 0.0345
2L:502120-502270:plus -23.4898 4.38532 -1.51376 5.15596 -17.7551 0.459184 -26.9908 0.194444 -1.79817
0.00639 0.00207 0.0187 0.00426 0.00658 0.000213 0.0604 0.0157 0.0315
2L:502740-502890:minus -13.1837 -1.66055 2.89908 1 -7.63265 -19.0816 -13.1835 2.66667 8.05505
0.000705 0.0176 0.00413 0.0206 0.000479 0.017 0.00603 0.00677 0.00064
2L:502740-502890:plus -31.8571 -1.70642 4.77064 2.80734 -27.898 -17.9796 -11.9266 -4.02778 0.697248
0.0163 0.0178 0.00196 0.00985 0.04 0.00764 0.00454 0.0496 0.0154
2L:5027440-5027590:minus -22.7041 1.23853 -1.11009 0.302752 -17.9796 -9.79592 -13.0367 -0.222222
9.42202 0.00395 0.00664 0.0165 0.0248 0.00726 0.00377 0.00583 0.0179 0.000349
2L:5027440-5027590:plus -23.7755 5.25688 3.87156 3.50459 -18.449 -18.1224 -9.47706 -6.26389 1.95413
0.00678 0.00146 0.00282 0.00713 0.0107 0.00988 0.00279 0.0802 0.00964
2L:5028040-5028190:minus -34.1531 0.550459 1.98165 2.23853 -18.7143 -18.3469 -20.2477 2.20833 7.37615
0.0314 0.00851 0.00582 0.0128 0.0136 0.0124 0.0215 0.008 0.00107
2L:5028040-5028190:plus -32.0714 5.84404 6.44954 3.95413 -27.2347 -25.602 -13.9083 2.68056 1.49541
0.0176 0.00113 0.000941 0.00593 0.0283 0.0207 0.00711 0.00673 0.0118
2L:5029380-5029530:minus -13.5204 -2.10092 1.56881 -0.834862 -8.93878 -18.7857 -12.2202 6.15278
2.51376 0.00097 0.02 0.00676 0.0349 0.00216 0.0146 0.00484 0.00154 0.00792
2L:5029380-5029530:plus -3.60204 0.220183 11.2018 6.80734 -17.3469 -9.72449 -5.56881 -5.93056 0.752294
0.0000856 0.00956 0.0000701 0.00145 0.00394 0.00352 0.00117 0.075 0.0152
2L:5043160-5043310:minus -23.6327 10.9266 -1.58716 2.45872 10.6531 -7.65306 -20.578 3.86111 -0.761468
0.00661 0.0000837 0.0191 0.0116 0.0000102 0.000647 0.0228 0.00425 0.0244
2L:5043160-5043310:plus -32.1122 1.44037 -2.14679 2.70642 -18.7551 -27.8571 -20.7339 4.90278 5.66055
0.0178 0.00617 0.0226 0.0103 0.0144 0.0508 0.0234 0.00274 0.00227
2L:5043640-5043790:minus -22.9898 0.0183486 0.541284 3.3945 -17.8163 0.979592 -5.6789 4.23611
-2.69725 0.00482 0.0102 0.00968 0.0076 0.00682 0.000136 0.0012 0.00364 0.0411
2L:5043640-5043790:plus -21.398 1.92661 -4.95413 0.12844 -18.1224 -18.8265 -11.5321 -8.25 9.9633
0.00197 0.00516 0.0488 0.0259 0.00782 0.0148 0.00417 0.116 0.000171
2L:5052700-5052850:minus -22.9898 2.36697 0.155963 4.6055 -8.60204 -8.20408 -12.5413 -4.77778 0.550459
0.00482 0.00439 0.011 0.00476 0.00125 0.000796 0.00521 0.0587 0.0161
2L:5052700-5052850:plus -14.4796 4.91743 15.5596 7.29358 -18.7857 -18.3061 -12.6514 8.43056 5.66055
0.00142 0.00169 6.69e-07 0.00104 0.0148 0.0114 0.00534 0.000455 0.00227
2L:505420-505570:minus -23.6633 -0.981651 1.20183 1.41284 -18.6837 -9.34694 -7.97248 3.83333
-0.0275229 0.00665 0.0143 0.0077 0.0183 0.0126 0.00261 0.00209 0.0043 0.0186
2L:505420-505570:plus -31.4796 3.76147 0.944954 3.07339 -18.5612 -35.602 -16.8807 10.3611 3.66972
0.0128 0.00263 0.00842 0.00873 0.0119 0.0803 0.0126 0.000125 0.00514
2L:5055020-5055170:minus -22.449 5.90826 1.07339 0.844037 -18.4898 -9.65306 -1.75229 5.72222 -0.917431
0.0035 0.0011 0.00806 0.0215 0.0116 0.0034 0.00042 0.00189 0.0256
2L:5055020-5055170:plus -3.89796 -4 4.61468 7 -26.5918 -9.23469 -14.4771 -1.97222 -0.40367 0.000113
0.0338 0.00209 0.00132 0.0186 0.00238 0.00805 0.0296 0.0213
2L:5058140-5058290:minus -32.7857 -1.33945 9.88991 10.5963 -27.4898 -18.0102 -20.2752 -3.63889 -2.22018
0.0235 0.0159 0.000152 0.0001 0.0336 0.00812 0.0216 0.0452 0.0355
2L:5058140-5058290:plus -31.6633 -0.587156 -1.30275 5.31193 -18.7143 -17.5714 -12.2294 4.06944
-6.33028 0.0141 0.0126 0.0175 0.00393 0.0136 0.00616 0.00485 0.00391 0.0963
2L:5059720-5059870:minus -32.1122 4.04587 0.504587 3.27523 -27.5612 -7.65306 -6.44037 -1.20833 2.01835
0.0178 0.00236 0.0098 0.00796 0.0354 0.000647 0.00147 0.0239 0.00928
2L:5059720-5059870:plus -14.5918 9.62385 0.587156 5.49541 -28.1939 -17.5714 -19.5963 8.56944 4.87156
0.00148 0.000161 0.00952 0.00355 0.0482 0.00616 0.0192 0.000418 0.00325
2L:506200-506350:minus -39 2.86239 3.22018 9.49541 -18.3776 1.02041 -12.633 2.40278 4.02752
0.0325 0.00366 0.00365 0.000308 0.00926 0.000118 0.00532 0.00746 0.00428
2L:506200-506350:plus -31.6327 5.25688 2.59633 4.0367 -17.449 -8.72449 -16.3303 3.66667 1.89908
0.0138 0.00146 0.00463 0.00578 0.00484 0.00169 0.0115 0.0046 0.0098
2L:5071060-5071210:minus -42.1122 7.24771 -2.93578 -0.724771 -27.2245 -26.898 -18.8349 6.05556
1.38532 0.0698 0.00057 0.0283 0.0338 0.0272 0.0302 0.017 0.00161 0.0123
2L:5071060-5071210:plus -32.7041 3.84404 2.47706 -1.05505 -18.4592 0.204082 -21.4037 3.11111 3.20183

0.0222 0.00255 0.00484 0.0374 0.0109 0.000296 0.0265 0.00572 0.0065
2L:5087540-5087690:minus -20.6327 -4.50459 -3.22018 2.83486 -17.3776 -9.57143 -26.7156 1.76389 -3.30275
0.00171 0.0387 0.0307 0.0097 0.00419 0.00326 0.0584 0.00937 0.0475
2L:5087540-5087690:plus -32 0.100917 4.33945 2.04587 -27.3061 -0.0204082 -28.5046 6.34722 0.541284
0.0171 0.00996 0.00234 0.014 0.0315 0.000411 0.0737 0.0014 0.0161
2L:5091160-5091310:minus -30.6327 6.25688 3.44037 2.97248 -17.4184 0.826531 -23.0183 6.09722 2.38532
0.00968 0.000938 0.00335 0.00909 0.00457 0.000153 0.0352 0.00158 0.00822
2L:5091160-5091310:plus -34.102 2.07339 3.91743 2.70642 -27.4898 -18.2755 -18.1927 8.09722 3.93578
0.0311 0.00488 0.00277 0.0103 0.0336 0.0112 0.0154 0.000552 0.00456
2L:5092020-5092170:minus -31.6735 -1.46789 3.16514 10.8624 -26.2653 -9.72449 -23.422 4.13889 -2.04587
0.0141 0.0166 0.00373 0.0000687 0.018 0.00352 0.0376 0.0038 0.0336
2L:5092020-5092170:plus -23.2551 3.44954 0.954128 6.83486 -18.4082 -8.60204 -18.1651 4.27778 -0.550459
0.00583 0.00295 0.0084 0.00142 0.00937 0.00151 0.0153 0.00358 0.0225
2L:5092980-5093130:minus -30.4388 -3.92661 -2.3578 2.98165 -26.9694 -17.2755 -12.4771 -1.43056 0.0917431
0.00923 0.0332 0.024 0.00905 0.023 0.00544 0.00513 0.0255 0.018
2L:5092980-5093130:plus -24.6735 0.0642202 0.0458716 -0.302752 -17.8878 -18.7857 0.302752
-7.47222 11.3853 0.00825 0.0101 0.0114 0.0293 0.00704 0.0146 0.000263 0.101 0.0000815
2L:5093420-5093570:minus -30.1429 0.724771 -0.504587 3.22936 -17.7551 -18.5714 -14 2.29167 9.92661
0.00866 0.008 0.0137 0.00816 0.00658 0.0136 0.00725 0.00776 0.000193
2L:5093420-5093570:plus -14.2959 3.64286 9.34862 3.59633 -28.5714 -9.09184 -11.9908 3.16667 11.3853
0.00129 0.00159 0.000207 0.00693 0.0623 0.00219 0.0046 0.0056 0.0000815
2L:5100160-5100310:minus -40.5204 -4.16514 10.4128 6.44954 -8.60204 -9.37755 -21.1009 5.18056 -2.85321
0.0413 0.0354 0.000111 0.00183 0.00125 0.00265 0.025 0.00242 0.0429
2L:5100160-5100310:plus -31.551 3.19266 10.8899 7.2844 20.4694 -9.57143 -6.77982 5.68056 -0.284404
0.0134 0.00324 0.0000856 0.00105 1.65e-07 0.00326 0.0016 0.00193 0.0207
2L:510640-510790:minus -33.0306 -0.853211 12.1101 5.57798 -18.7857 -26.051 -15.8349 -3.02778
1.90826 0.026 0.0137 0.0000401 0.0034 0.0148 0.0216 0.0105 0.0389 0.00974
2L:510640-510790:plus -22.8571 2.89908 -0.779817 4.23853 -18.1224 -25.5306 -17.5413 -3.51389 0.137615
0.00427 0.00361 0.0149 0.00522 0.00782 0.0205 0.0139 0.0439 0.0178
2L:5108340-5108490:minus -24.1531 2.0367 3.53211 3.80734 -17.6429 -9.53061 -24.2018 0.833333 -0.761468
0.00744 0.00495 0.00323 0.00627 0.00562 0.0031 0.0423 0.0128 0.0244
2L:5108340-5108490:plus -21.7347 1.72477 7.31193 0.981651 -27.2653 -17.7857 -7.22936 -3.58333 7.37615
0.00235 0.00555 0.000623 0.0207 0.0302 0.0068 0.00178 0.0446 0.00107
2L:5145460-5145610:minus -31.4388 2.25688 1.52294 -0.963303 -18.1531 -18.5714 -5.23853 10.2778
1.01835 0.0122 0.00457 0.00687 0.0362 0.00829 0.0136 0.00106 0.000134 0.0142
2L:5145460-5145610:plus -13.3367 3.63303 -0.220183 11.2936 -17.6429 -0.204082 -9.58716 2.08333
-4.00917 0.0008 0.00276 0.0125 0.0000139 0.00562 0.000463 0.00285 0.00837 0.0558
2L:5146020-5146170:minus -14.3776 4.23853 1.33028 2.7156 -7.93878 -9.57143 -27.1101 3.625 0.238532
0.00134 0.0022 0.00736 0.0102 0.000767 0.00326 0.0613 0.00468 0.0173
2L:5146020-5146170:plus -14.2959 -3.9633 -1.36697 4.33945 -26.9286 0.346939 -20.0183 1.36111 1.02752
0.00129 0.0335 0.0179 0.00509 0.0214 0.000221 0.0207 0.0108 0.0141
2L:5147600-5147750:minus -29.5204 2.05505 2.55963 2.69725 -9.54082 -8.86735 -12.9908 10.5417 8.62385
0.00845 0.00492 0.0047 0.0104 0.00294 0.00198 0.00577 0.00011 0.00048
2L:5147600-5147750:plus -22.2551 4.13761 -1.26606 2.75229 -27.2653 -17.2755 -15.422 1.36111 3.62385
0.00327 0.00228 0.0173 0.0101 0.0302 0.00544 0.00975 0.0108 0.00523
2L:516660-516810:minus -33.8469 1.22018 2.82569 2.09174 -17.7143 -37.449 -7.92661 0.930556 0.963303
0.03 0.00668 0.00424 0.0137 0.00627 0.164 0.00207 0.0124 0.0145
2L:516660-516810:plus -29.6327 3.51376 3.54128 6.31193 -8.93878 -8.16327 -23.2202 7.63889 3.99083
0.00846 0.00288 0.00322 0.00199 0.00216 0.000783 0.0364 0.000715 0.00433
2L:519340-519490:minus -22.5918 7.19266 -0.642202 5.47706 -17.3776 -18.5 -9.6789 3.5 9.92661
0.00365 0.000587 0.0143 0.0036 0.00419 0.0131 0.0029 0.00491 0.000193
2L:519340-519490:plus -21.3265 9.53211 -3.00917 3.47706 -26.6735 -8.57143 -13.3211 2.38889 3.77064
0.00193 0.00017 0.0289 0.00724 0.0191 0.00149 0.00622 0.00749 0.00491
2L:5195800-5195950:minus -23.2959 4.77064 -0.568807 1.69725 -19.3163 -9.09184 -4.3211 1.43056

-5.44037 0.00605 0.00179 0.0139 0.0163 0.0165 0.00219 0.000817 0.0105 0.0796
2L:5195800-5195950:plus -11.551 -2.0367 6.24771 6.89908 -17.1531 -26.5306 12.4128 0.555556 7.36697
0.000255 0.0196 0.00103 0.00134 0.00364 0.023 4.62e-06 0.014 0.00108
2L:5197200-5197350:minus -22.8878 -1.49541 8.52294 7.55963 -8.53061 -8.38776 -10.8165 -1.875 -1.24771
0.00441 0.0167 0.000329 0.000807 0.0011 0.00101 0.0036 0.0288 0.028
2L:5197200-5197350:plus -30.7449 -4.57798 7.40367 2.55963 -17.4184 -27.1122 -18.9083 2.27778 3.88073
0.0101 0.0395 0.000595 0.0111 0.00457 0.0316 0.0172 0.0078 0.00475
2L:521060-521210:minus -23.2245 4.86239 0.651376 3.45872 -19.051 -8.23469 -31.4954 1.18056 -4.6422
0.00576 0.00172 0.00932 0.00731 0.0162 0.000875 0.111 0.0114 0.0662
2L:521060-521210:plus -29.551 -4.41284 0.0642202 6.56881 -17.449 -19.051 -2.25688 0.875 7.6789
0.00845 0.0378 0.0113 0.0017 0.00484 0.0165 0.000472 0.0127 0.000866
2L:521840-521990:minus -23.5612 -0.330275 6.19266 4.22018 -26 -17.9388 -20.1101 -0.333333
-0.743119 0.00653 0.0115 0.00106 0.00531 0.0171 0.00738 0.021 0.0185 0.0243
2L:521840-521990:plus -14.2551 2.33945 3.18349 4.40367 -7.60204 -18.051 9.84404 0.152778 -0.0733945
0.00127 0.00443 0.0037 0.00497 0.000466 0.00886 0.0000137 0.016 0.0188
2L:524720-524870:minus -32.3265 -2.56881 6.79817 1.83486 -17.9286 -28.1122 -12.8165 -2.56944 2.6789
0.0187 0.0229 0.000798 0.0155 0.0071 0.0565 0.00554 0.0346 0.00763
2L:524720-524870:plus -31.6327 3.90816 -1.33945 2.08257 -8.64286 -18.0102 -11.0183 -0.972222 8.62385
0.0138 0.00102 0.0177 0.0137 0.00139 0.00812 0.00375 0.0224 0.00048
2L:5278400-5278550:minus -30.4388 -0.412844 -3.51376 2.55963 -18.1122 -8.94898 -21.3578 -1.19444
8.88073 0.00923 0.0119 0.0333 0.0111 0.00771 0.00203 0.0262 0.0239 0.000468
2L:5278400-5278550:plus -32.6939 6.11009 -3.82569 6.41284 -18.7143 -17.0102 -17.6697 4.80556 6.76147
0.0217 0.00101 0.0362 0.00189 0.0136 0.00453 0.0142 0.00286 0.00141
2L:5278920-5279070:minus -23.2245 3.68367 3.05505 6.00917 -27.4592 0.755102 -11.9083 2.56944 -2.98165
0.00576 0.00144 0.00389 0.00255 0.0329 0.000175 0.00452 0.00701 0.0445
2L:5278920-5279070:plus -22.2959 13.5306 2.49541 3.77064 -8.93878 -26.7551 -14.7523 1.5 3.42202
0.00335 0.0000206 0.00481 0.00637 0.00216 0.0263 0.00853 0.0103 0.00597
2L:5286120-5286270:minus -42.7755 2.50459 0.477064 5.57798 -18.9796 -8.79592 -23.3119 5.75 1.74312
0.0854 0.00417 0.00989 0.0034 0.0155 0.0019 0.0369 0.00187 0.0105
2L:5286120-5286270:plus -32.8163 -0.0366972 -1.15596 -1.20183 -19.0204 -9.02041 -13.7615 8.84722
-2.52294 0.024 0.0104 0.0168 0.0392 0.0158 0.00213 0.00688 0.000353 0.0391
2L:5289860-5290010:minus -31.4082 -4.95413 3.73394 7.37615 -18.3469 9.67347 -20.4679 -2.73611 -2.78899
0.0119 0.0436 0.00299 0.000951 0.00894 0.0000217 0.0223 0.0362 0.0422
2L:5289860-5290010:plus -41.8571 1.06422 -1.33945 -0.40367 -29.0918 -8.94898 -20.5321 5.73611 3.98165
0.0667 0.00707 0.0177 0.0302 0.0653 0.00203 0.0226 0.00188 0.00439
2L:5290460-5290610:minus -33.0816 2.12844 4.3945 5.92661 -27.8673 -9.42857 -26.6697 0 -0.504587
0.0268 0.00479 0.00229 0.00266 0.0391 0.0027 0.0581 0.0167 0.0221
2L:5290460-5290610:plus -21.6224 -1.18349 7.51376 4.0367 -6.89796 -17.8571 -11.8899 -1.30556 2.11009
0.00213 0.0152 0.000563 0.00578 0.000364 0.00719 0.0045 0.0246 0.00898
2L:5291040-5291190:minus -22.2245 2.46789 6.93578 6.00917 -27.4592 -18.3469 -23.5596 0.361111 -3.68807
0.00325 0.00422 0.00075 0.00255 0.0329 0.0124 0.0384 0.0149 0.0516
2L:5291040-5291190:plus -32.9286 2.04587 3.20183 7 -27.2653 -9.72449 -24 1.58333 7.18349 0.025
0.00494 0.00367 0.00132 0.0302 0.00352 0.041 0.00997 0.00121
2L:5291540-5291690:minus -4.59184 -1.80734 3.90826 3.41284 -17.6837 -9.23469 -5.7156 3.16667 -5.57798
0.000166 0.0184 0.00278 0.00756 0.0058 0.00238 0.00121 0.0056 0.0816
2L:5291540-5291690:plus -40.6327 7.16514 1.97248 5.22936 -17.9388 -27.7857 -14.633 1.76389 3.77982
0.0424 0.000596 0.00584 0.0041 0.00715 0.0466 0.00832 0.00937 0.00489
2L:5292440-5292590:minus -31.7347 5.37615 0.908257 6.11009 -8.11224 -8.5 -7.05505 -4.63889 -0.0183486
0.0149 0.00139 0.00853 0.00233 0.000838 0.00126 0.00171 0.057 0.0185
2L:5292440-5292590:plus -23.3673 1.45872 0.513761 6.37615 -18.449 -27.8265 -8.06422 -0.763889
5.65138 0.00617 0.00613 0.00977 0.00192 0.0107 0.0498 0.00213 0.0211 0.00228
2L:5293000-5293150:minus -21.6633 -1.81651 -0.53211 2.83486 -17.7143 -19.0102 -13.7523 -0.847222
-4.42202 0.00223 0.0184 0.0138 0.0097 0.00627 0.0159 0.00686 0.0216 0.0626
2L:5293000-5293150:plus -22.9592 0.119266 3.42202 11.0275 1.14286 -18.9388 -4.16514 -2.33333 4.13761

0.00475 0.0099 0.00337 0.0000401 0.000113 0.0154 0.000782 0.0326 0.00408
2L:5294900-5295050:minus -22.6224 0 1.08257 5.01835 -27.2245 0.173469 -12.6147 1.44444 -1.54128
0.00371 0.0103 0.00803 0.00438 0.0272 0.000307 0.00529 0.0105 0.0296
2L:5294900-5295050:plus -22.7755 7.25688 4.56881 11.2936 -17.0816 -7.72449 -8.06422 -0.180556
-3.41284 0.00412 0.000568 0.00213 0.0000139 0.00334 0.00068 0.00213 0.0177 0.0487
2L:5305500-5305650:minus -21.6633 2.89908 -0.201835 7.6055 0.877551 -18.7143 -12.0275 -2.59722
1.25688 0.00223 0.00361 0.0124 0.000794 0.00019 0.014 0.00464 0.0349 0.0131
2L:5305500-5305650:plus -21.8878 3.42202 4.26606 7.27523 -18.3776 -9.45918 -2.77064 0.930556 -0.458716
0.00258 0.00298 0.00241 0.00109 0.00926 0.00279 0.000536 0.0124 0.0217
2L:5306660-5306810:minus -22.8061 -2.77064 2.05505 1.72477 -7.67347 -26.6735 -15.4495 -0.458333
11.4771 0.00414 0.0242 0.00566 0.0161 0.00055 0.0256 0.0098 0.0192 0.0000679
2L:5306660-5306810:plus -22.3673 0.477064 -4.45872 7.00917 -7.56122 -18.7857 -6.31193 5.625 3.41284
0.00341 0.00873 0.0429 0.00126 0.000419 0.0146 0.00142 0.00198 0.006
2L:531660-531810:minus -23.9286 7.11927 3.55046 0.46789 -8.86735 -18.7857 -7.95413 5.44444 -2.05505
0.00701 0.000611 0.00321 0.0239 0.00173 0.0146 0.00208 0.00215 0.0338
2L:531660-531810:plus -13.1837 2.65138 8.88073 2.66972 -18.7143 -18.9796 -10.8807 -4.68056 -2.01835
0.000705 0.00395 0.000271 0.0106 0.0136 0.0155 0.00364 0.0575 0.0334
2L:5325220-5325370:minus -13.3673 14.6881 -0.587156 2.3578 -18.6735 -18.3061 -16.6789 -1.04167
-3.33028 0.000813 3.75e-06 0.014 0.0121 0.0123 0.0114 0.0122 0.0228 0.0478
2L:5325220-5325370:plus -31.1735 3 10.3578 7.55963 -27.2653 -17.7857 -23.7064 -2.52778 0.0275229
0.0109 0.00348 0.000115 0.000807 0.0302 0.0068 0.0393 0.0343 0.0183
2L:5326680-5326830:minus -29.9184 -2.05505 0.788991 0.40367 -8.71429 -8.72449 -18.0459 5.55556 0.669725
0.00854 0.0197 0.00889 0.0242 0.00159 0.00169 0.0151 0.00204 0.0155
2L:5326680-5326830:plus -32 2.0367 7.33028 7 -18.4592 -9.5 -3.91743 -1.13889 1.7156 0.0171
0.00495 0.000617 0.00132 0.0109 0.00294 0.000729 0.0235 0.0107
2L:5341140-5341290:minus -30.7041 1.02752 7.2844 -0.651376 -17.7143 -17.3469 -23.7431 1.79167
-4.78899 0.00993 0.00717 0.000632 0.0329 0.00627 0.0058 0.0395 0.00928 0.0691
2L:5341140-5341290:plus -33.6327 7.44037 4.9633 3.01835 -18.6735 -18.0102 -11.9358 0.611111 -1.9633
0.029 0.000515 0.00181 0.0089 0.0123 0.00812 0.00455 0.0138 0.0329
2L:5342280-5342430:minus -22.7653 -1.10092 5.63303 9.49541 -17.4082 -7.45918 -16.1009 1.75 -0.743119
0.00405 0.0148 0.00136 0.000308 0.00421 0.000582 0.011 0.00941 0.0243
2L:5342280-5342430:plus -11.8163 1.17431 -2.61468 4.86239 -28.1939 0.826531 -19.2018 2.66667 -0.366972
0.000272 0.00679 0.0259 0.00449 0.0482 0.000153 0.018 0.00677 0.021
2L:5342640-5342790:minus -32.4082 1.55046 -0.669725 1.48624 -7.63265 -27.9694 -8.78899 -0.263889
1.27523 0.0191 0.00592 0.0144 0.0177 0.000479 0.0547 0.00246 0.0181 0.013
2L:5342640-5342790:plus -22.8878 0.302752 7.11009 2.80734 -17.3061 -8.38776 -21.1284 -0.180556
-6.93578 0.00441 0.00929 0.000691 0.00985 0.00389 0.00101 0.0252 0.0177 0.111
2L:540980-541130:minus -31.6735 -3.04587 9.88991 10.0275 -8.67347 9.08163 -30.4954 1.01389 -4.04587
0.0141 0.0261 0.000152 0.000156 0.00154 0.0000431 0.0974 0.0121 0.0563
2L:540980-541130:plus -40.6633 -0.853211 0.853211 3.11009 -17.7959 -9.34694 -25.5596 5.23611 -2.40367
0.0426 0.0137 0.0087 0.00862 0.00678 0.00261 0.0507 0.00236 0.0376
2L:542520-542670:minus -23.1837 14.5688 -1.11009 6.12844 10.6224 -26.602 -6.57798 1.30556 1.36697
0.00553 4.39e-06 0.0165 0.00231 0.0000113 0.0248 0.00152 0.011 0.0124
2L:542520-542670:plus -41.8469 -0.486239 0.642202 2.6055 -18.5306 -8.72449 -30.2569 7.04167 -2.69725
0.0665 0.0121 0.00935 0.0109 0.0118 0.00169 0.0943 0.000984 0.0411
2L:544620-544770:minus -13.9286 6.31193 3.50459 5.42202 -8.67347 -27.5612 -7.72477 -1.25 -0.605505
0.00112 0.000913 0.00326 0.00374 0.00154 0.0393 0.00199 0.0242 0.023
2L:544620-544770:plus -24.2245 2.66055 3.34862 1.36697 -18.6837 -19.2755 -24.7156 0.666667 -2.7156
0.00763 0.00394 0.00347 0.0186 0.0126 0.0185 0.0454 0.0135 0.0412
2L:544980-545130:minus -31.7755 2.05505 2.79817 9.33028 -17.4898 -9.7551 -21.5963 -2.70833 -2.61468
0.0157 0.00492 0.00429 0.000374 0.0053 0.00358 0.0274 0.0359 0.0401
2L:544980-545130:plus -21.8878 4.49541 -0.0275229 9.61468 -26.1939 -28.5612 -1.75229 -2.18056 7.63303
0.00258 0.00199 0.0117 0.000255 0.0175 0.0636 0.00042 0.0313 0.000928
2L:545580-545730:minus -21.8878 4.55046 6 6.21101 -8.90816 -17.7143 -8.49541 2.70833 -1.6055

0.00258 0.00195 0.00115 0.00213 0.00192 0.00634 0.00232 0.00666 0.0302
2L:545580-545730:plus -21.4796 -2.08257 -0.972477 2.06422 0.581633 -19.1531 -10.578 1.29167 9.30275
0.00202 0.0199 0.0158 0.0139 0.000292 0.0177 0.00343 0.011 0.00038
2L:5499540-5499690:minus -11.1837 -0.211009 7.44954 6.72477 -17.7449 -18.3469 -13.2294 1.65278
-2.01835 0.000248 0.0111 0.000581 0.00154 0.0063 0.0124 0.00609 0.00974 0.0334
2L:5499540-5499690:plus -30.551 2.29358 5.48624 5.50459 -18.1939 -27.3367 -20.0459 -2.08333 0.0275229
0.00959 0.00451 0.00145 0.00349 0.0086 0.0347 0.0208 0.0305 0.0183
2L:5520160-5520310:minus -39.5918 1.0367 7.3945 2.16514 -8.33673 -8.38776 -1.80734 4.375 5.12844
0.0335 0.00714 0.000597 0.0132 0.00101 0.00101 0.000425 0.00344 0.00301
2L:5520160-5520310:plus -33.3367 -3.22936 5.12844 4.94495 -19.051 -8.5 -23.2385 2.56944 3.11009
0.0281 0.0275 0.00169 0.00444 0.0162 0.00126 0.0365 0.00701 0.00669
2L:5520760-5520910:minus -24.0714 -1.69725 10.2477 6.54128 -17.6429 -17.2755 -4.14679 4.75 -2.01835
0.00731 0.0178 0.000123 0.00173 0.00562 0.00544 0.000778 0.00293 0.0334
2L:5520760-5520910:plus -22.949 5.72477 5.51376 7 -8.89796 -8.79592 2.26606 -1.06944 -4.6422
0.0046 0.0012 0.00143 0.00132 0.00189 0.0019 0.000151 0.023 0.0662
2L:5523880-5524030:minus -23.5918 -1.83486 15.6239 -0.633028 -18.7857 -8.68367 -14.7248 5.13889
-0.440367 0.00656 0.0185 5.44e-07 0.0327 0.0148 0.00157 0.00848 0.00247 0.0216
2L:5523880-5524030:plus -32.551 1.07339 2.33945 0.899083 -27.3061 -17.5714 -27.8991 -1.36111 3.88991
0.0207 0.00705 0.0051 0.0212 0.0315 0.00616 0.0679 0.025 0.00467
2L:5524100-5524250:minus -23.5918 -0.0825688 15.6239 1.38532 -18.7857 -17.2755 -14.7248 1.68056
1.48624 0.00656 0.0106 5.44e-07 0.0185 0.0148 0.00544 0.00848 0.00964 0.0118
2L:5524100-5524250:plus -31.7755 9.3211 -1.85321 2.55046 -18.4184 -27.898 -13.8165 -1.05556 8.11009
0.0157 0.000189 0.0207 0.0112 0.0101 0.0537 0.00696 0.0229 0.000597
2L:552580-552730:minus -21.9592 -1.43119 1.40367 4.59633 -17.3776 -19.5714 -7.3945 -1.63889 3.97248
0.00279 0.0164 0.00717 0.00478 0.00419 0.0202 0.00185 0.027 0.00444
2L:552580-552730:plus -22.0306 2.3211 -1.3578 4.19266 -9.20408 -16.7857 -1.55963 0.0277778 0.12844
0.00293 0.00446 0.0178 0.00536 0.00262 0.00426 0.000402 0.0166 0.0179
2L:5525820-5525970:minus -23.6224 8.04587 6.76147 3.88991 -26.9694 -9.27551 -21.0183 -4.75 1.07339
0.00658 0.000373 0.000811 0.00608 0.023 0.00244 0.0247 0.0584 0.014
2L:5525820-5525970:plus -12.1122 13.5306 14.1835 7.45872 -18.0816 -16.7143 -11.9725 -1.25 -0.724771
0.000336 0.0000206 5.88e-06 0.000894 0.00759 0.00419 0.00458 0.0242 0.0241
2L:5526800-5526950:minus -22.7347 13.4037 15.5596 7.29358 -17.449 -17.7857 -13.8716 -3.83333 1.25688
0.00403 0.0000154 6.69e-07 0.00104 0.00484 0.0068 0.00705 0.0473 0.0131
2L:5526800-5526950:plus -30.4796 -1.74312 14.8532 3.77064 0.877551 -26.8265 -16.6972 -2.05556 -3.82569
0.00941 0.018 2.28e-06 0.00637 0.00019 0.0285 0.0122 0.0303 0.0531
2L:5527980-5528130:minus -23.4082 5.3211 1.90826 11.2936 -36.5204 -17.5714 -19.367 7.09722 -3.01835
0.00623 0.00142 0.00598 0.0000139 0.0818 0.00616 0.0185 0.000956 0.0448
2L:5527980-5528130:plus -31.2857 4.98165 10.8991 6.21101 -27 -9.30612 -17.055 3.77778 5.58716
0.0113 0.00164 0.0000848 0.00213 0.0233 0.00257 0.0129 0.0044 0.00237
2L:5532180-5532330:minus -13.9184 7.06422 -2.22936 1.12844 -27.1633 -9.5 -12.8532 1.58333 6.17431
0.00112 0.000628 0.0231 0.0198 0.025 0.00294 0.00559 0.00997 0.00171
2L:5532180-5532330:plus -14.7041 -0.908257 8.6055 2.73394 -18.1224 -27.6735 -14.4404 3.94444
1.84404 0.00154 0.0139 0.000314 0.0101 0.00782 0.0443 0.00799 0.00411 0.01
2L:553420-553570:minus -13.7449 9.05505 7.77064 10.5963 -18.4184 -27.4082 -6.22018 1.45833 7.22018
0.00107 0.000217 0.000494 0.0001 0.0101 0.0356 0.00139 0.0104 0.00119
2L:553420-553570:plus -22.2959 -1.62385 -0.59633 6.37615 -19.051 -18.3469 -18.3394 6.13889 -0.522936
0.00335 0.0174 0.0141 0.00192 0.0162 0.0124 0.0157 0.00155 0.0222
2L:5542380-5542530:minus -31.398 -2.11009 1.68807 6.31193 20.4694 -9.38776 -21.156 1.33333 -7.05505
0.0117 0.0201 0.00648 0.00199 1.65e-07 0.00266 0.0253 0.0109 0.114
2L:5542380-5542530:plus -21.5918 13.5306 -0.559633 4.47706 -17.449 -26.8265 -1.76147 -1.15278
-1.26606 0.00209 0.0000206 0.0139 0.00488 0.00484 0.0285 0.000421 0.0236 0.0281
2L:5542680-5542830:minus -23.7347 -0.00917431 5.61468 2.19266 -27.5306 -17.3469 -6.87156 5.47222
2.74312 0.00673 0.0103 0.00137 0.0131 0.0351 0.0058 0.00164 0.00212 0.00751
2L:5542680-5542830:plus -41.0612 5.66972 -0.146789 1.50459 -17.1939 -17.7857 -13.1743 1.84722

2.00917 0.0466 0.00123 0.0122 0.0176 0.00377 0.0068 0.00602 0.0091 0.00934
2L:5543000-5543150:minus -22.0714 0.256881 1.54128 3.94495 -28.2245 -8.93878 -16.1468 6.23611 4.80734
0.00299 0.00944 0.00683 0.00595 0.049 0.00201 0.0111 0.00148 0.00334
2L:5543000-5543150:plus -3.4898 -1.27523 3.81651 6.47706 -26.8571 0.0510204 -22.9633 -1.84722
11.3853 0.0000735 0.0156 0.00289 0.00179 0.0198 0.000349 0.0349 0.0286 0.0000815
2L:5546880-5547030:minus -23.1429 1.62385 -1.87156 -0.550459 -26.7449 -27.7041 -9.15596 3.31944
7.42202 0.00533 0.00577 0.0208 0.0317 0.0195 0.0445 0.00263 0.00528 0.00106
2L:5546880-5547030:plus -4.43878 9.07339 -0.972477 3.44037 -17.7551 -27.6735 10.1284 -0.0138889
10.3486 0.000149 0.000215 0.0158 0.00746 0.00658 0.0443 0.0000124 0.0168 0.0000988
2L:559440-559590:minus -30.8163 0.669725 5.95413 0.321101 -27.5306 -18.8571 -11.6789 4.23611 -0.440367
0.0103 0.00815 0.00118 0.0247 0.0351 0.0151 0.0043 0.00364 0.0216
2L:559440-559590:plus -30.4388 -3.91743 3.09174 6.74312 -18.4184 -26.2653 -14.945 -2.40278 -0.752294
0.00923 0.0331 0.00383 0.0015 0.0101 0.0218 0.00887 0.0332 0.0243
2L:560320-560470:minus -20.8776 0.724771 2.00917 5.54128 -8.60204 -17.7143 -10.2569 -0.277778
7.6422 0.00178 0.008 0.00576 0.00346 0.00125 0.00634 0.00323 0.0182 0.000893
2L:560320-560470:plus -31.398 14.5688 2.10092 0.00917431 -17.7551 -26.6735 -7.75229 3.20833 -1.47706
0.0117 4.39e-06 0.00556 0.0269 0.00658 0.0256 0.002 0.00551 0.0292
2L:564080-564230:minus -23.4898 0.302752 3.27523 5.15596 -9.5 -18.5 -9.29358 1 -6.68807 0.00639
0.00929 0.00357 0.00426 0.00293 0.0131 0.0027 0.0121 0.106
2L:564080-564230:plus -12.1122 -0.862385 6.20183 6.84404 -17.1531 -9.68367 -21.6697 -2.94444 -3.73394
0.000336 0.0137 0.00106 0.0014 0.00364 0.00342 0.0278 0.0381 0.052
2L:568220-568370:minus -32.5102 13.3878 10.2294 3.42202 -27.4592 -9.94898 -11.5596 -2.48611 1.37615
0.0202 0.0000869 0.000125 0.00751 0.0329 0.00393 0.00419 0.0339 0.0123
2L:568220-568370:plus -40.2143 -2.85321 7.3211 3.27523 11.4286 -8.57143 -16.3486 -0.388889 -5.13761
0.0372 0.0248 0.00062 0.00796 1.97e-06 0.00149 0.0115 0.0188 0.0749
2L:5720920-5721070:minus -33.3776 13.3878 -2.26606 1.12844 -19.0204 -0.316327 -20.7523 5.79167
1.95413 0.0282 0.0000869 0.0234 0.0198 0.0158 0.000524 0.0235 0.00183 0.00964
2L:5720920-5721070:plus -23.2653 13.6422 2.9633 9.59633 -17.9796 -15.9796 -17.7982 4.06944 -6.11009
0.00593 0.0000127 0.00403 0.000275 0.00726 0.00405 0.0145 0.00391 0.0912
2L:5724580-5724730:minus -13.6939 3.06422 -1.50459 1.56881 -18.051 -17.9388 -4.33945 5.02778 3.46789
0.00103 0.0034 0.0186 0.0172 0.00746 0.00738 0.000822 0.00259 0.00582
2L:5724580-5724730:plus -31.398 4.90816 10.3303 4.23853 -17.7143 -27.5612 -15.9541 -0.541667
4.66055 0.0117 0.00023 0.000117 0.00522 0.00627 0.0393 0.0108 0.0197 0.0035
2L:574240-574390:minus -21.9592 -0.486239 3.26606 3.85321 -26.0408 -19.3469 -12.7156 1.93056
3.76147 0.00279 0.0121 0.00358 0.00623 0.0171 0.0194 0.00542 0.00883 0.00494
2L:574240-574390:plus -22.9592 -3.48624 0.816514 0.183486 -27.2347 -9.5 -19.789 -0.402778 4.66055
0.00475 0.0294 0.0088 0.0256 0.0283 0.00294 0.0199 0.0189 0.0035
2L:5771360-5771510:minus -3.33673 -1.44954 3.88073 3.78899 -17.3776 -0.173469 -14.8257 -1.31944
-3.45872 0.0000599 0.0165 0.00281 0.00632 0.00419 0.000456 0.00866 0.0247 0.0492
2L:5771360-5771510:plus -31.4388 -0.688073 -0.275229 0.981651 -27.2245 -18.3469 -21.422 5.75
1.74312 0.0122 0.013 0.0127 0.0207 0.0272 0.0124 0.0266 0.00187 0.0105
2L:5772320-5772470:minus -22.7041 -3.26606 4.98165 10.0275 -26.9694 9.57143 -13.4037 -1.05556 3.45872
0.00395 0.0277 0.00179 0.000156 0.023 0.0000274 0.00634 0.0229 0.00591
2L:5772320-5772470:plus -21.8878 1.46789 3.52294 6.06422 -17.4184 -17.7143 -7.77982 4.73611 3.20183
0.00258 0.0061 0.00324 0.00239 0.00457 0.00634 0.00201 0.00295 0.0065
2L:5773300-5773450:minus -23.3367 7.11009 2.69725 3.26606 -19.0204 -26.602 -21.5688 8.23611 -3.30275
0.00614 0.000613 0.00446 0.00802 0.0158 0.0248 0.0273 0.00051 0.0475
2L:5773300-5773450:plus -22.3673 -3.53211 2.78899 1.75229 -8.86735 -8.45918 -21.0917 2.26389 -5.87156
0.00341 0.0298 0.0043 0.0159 0.00173 0.00111 0.025 0.00784 0.0865
2L:5774560-5774710:minus -22.6531 3.19266 4.73394 6.13761 -18.1939 -18.051 -2.46789 -4.51389 1.25688
0.00374 0.00324 0.00199 0.00226 0.0086 0.00886 0.000497 0.0554 0.0131
2L:5774560-5774710:plus -22.398 4.42202 -1.47706 2.83486 -7.67347 -16.9388 -12.6239 2.30556 0.816514
0.00344 0.00204 0.0185 0.0097 0.00055 0.00433 0.0053 0.00772 0.015
2L:5780740-5780890:minus -4.43878 0.724771 0.486239 2.76147 -18.5204 -17.8571 -11.2569 2.38889 9.92661

0.000149 0.008 0.00986 0.01 0.0117 0.00719 0.00393 0.00749 0.000193
2L:5780740-5780890:plus -31.9286 -2.47706 1.94495 3.14679 -18.8265 -9.02041 -24.7798 6.66667 0.293578
0.0166 0.0223 0.0059 0.00847 0.0149 0.00213 0.0458 0.00119 0.017
2L:5797840-5797990:minus -24.0714 3.56881 -0.816514 0.853211 -28.2653 -17.7143 -6.66972 7.58333
8.10092 0.00731 0.00282 0.0151 0.0214 0.0537 0.00634 0.00156 0.000737 0.000623
2L:5797840-5797990:plus -13.4082 1.56881 2.68807 4.65138 -26.898 -9.09184 -25.0275 1.70833 -5.0367
0.000867 0.00588 0.00447 0.00471 0.0203 0.00219 0.0473 0.00955 0.0734
2L:5799700-5799850:minus -24.1837 -0.192661 12.2844 7.29358 -17.6735 -9.72449 -20.7064 -5.20833
-6.11927 0.00751 0.011 0.0000351 0.00104 0.00572 0.00352 0.0233 0.0645 0.0914
2L:5799700-5799850:plus -32.3673 4.48624 -5.06422 3.18349 -8.67347 -8.72449 -15.6881
3.63889 -1.22018 0.0189 0.00199 0.0502 0.00837 0.00154 0.00169 0.0102 0.00465 0.0278
2L:5802020-5802170:minus -31.8776 4.41284 -0.321101 -0.238532 -17.4184 9.82653 -12.8624
1.69444 -6.48624 0.0163 0.00205 0.0129 0.0288 0.00457 0.0000148 0.0056 0.0096 0.1
2L:5802020-5802170:plus -31.8163 0.266055 6.88073 1.91743 2.87755 -9.42857 -2.07339 -2.88889
-2.85321 0.016 0.0094 0.000768 0.015 0.0000259 0.0027 0.000452 0.0376 0.0429
2L:5879560-5879710:minus -12.5918 -1.89908 4.61468 6.33945 -8.93878 -18.0102 -10.578
-0.652778 2.11009 0.000449 0.0189 0.00209 0.00196 0.00216 0.00812 0.00343 0.0204 0.00898
2L:5879560-5879710:plus -22.8163 4.55046 4.38532 4.91743 -18.0816 -7.72449 -20.3486 -1.22222
7.51376 0.00418 0.00195 0.0023 0.00447 0.00759 0.00068 0.0219 0.024 0.000984
2L:5907920-5908070:minus -13.1531 2.3578 3.72477 7.47706 -8.67347 -9.79592 -11.3578
1.33333 -0.33945 0.00067 0.0044 0.003 0.00085 0.00154 0.00377 0.00402 0.0109 0.0209
2L:5907920-5908070:plus -12.5204 0.256881 6.55046 1.12844 -19.051 -19.4184 -14.3211 9.59722
3.6789 0.000441 0.00944 0.000896 0.0198 0.0162 0.0198 0.00778 0.000217 0.00506
2L:5943820-5943970:minus -23.5204 2.89908 -1.12844 0.651376 -28.2347 -15.2755
-15.4128 0.0138889 11.8532 0.00645 0.00361 0.0166 0.0226 0.051 0.00404 0.00973 0.0167 0.0000428
2L:5943820-5943970:plus -13.1531 1.33028 4.25688 9.3211 -19.3163 -9.60204 -14.945 2.97222 1.80734
0.00067 0.00642 0.00242 0.000391 0.0165 0.00334 0.00887 0.00603 0.0102
2L:5949260-5949410:minus -12.449 2.45872 1.68807 2.00917 -19.0102 -9.94898 -0.908257 4.33333
1.70642 0.000428 0.00424 0.00648 0.0142 0.0157 0.00393 0.000348 0.0035 0.0108
2L:5949260-5949410:plus -30.6327 -0.0550459 -1.19266 2.19266 -8.93878 -7.79592 -12.7615
0.236111 5.80734 0.00968 0.0105 0.0169 0.0131 0.00216 0.000723 0.00547 0.0155 0.00205
2L:5971500-5971650:minus -31.0306 1.25688 -0.0366972 1.74312 -18.3776 -9.23469 -17.1376
0.0416667 7.83486 0.0105 0.00659 0.0117 0.016 0.00926 0.00238 0.0131 0.0165 0.000712
2L:5971500-5971650:plus -30.2551 1.74312 3.92661 5.08257 -18.3061 -8.45918 -12.0367 3.43056
3.05505 0.00885 0.00552 0.00276 0.00429 0.00884 0.00111 0.00465 0.00505 0.00692
2L:5972760-5972910:minus -33.0408 -0.486239 -0.201835 0.357798 -28.3061 -8.68367
-17.6697 8.52778 4.40367 0.0264 0.0121 0.0124 0.0245 0.0553 0.00157 0.0142 0.000429 0.00373
2L:5972760-5972910:plus -13.3061 7.72477 1.89908 7.62385 -17.6837 -8.16327 -17.7523 2.61111
-6.68807 0.000777 0.000443 0.006 0.000759 0.0058 0.000783 0.0144 0.00691 0.106
2L:5975760-5975910:minus -21.8469 1.44954 6.50459 6.19266 -17.7143 -17.5714 -21.367 1.55556
2.06422 0.00247 0.00615 0.000917 0.00216 0.00627 0.00616 0.0263 0.0101 0.00912
2L:5975760-5975910:plus -33.0408 6.62385 -0.53211 1.41284 -17.7857 -8.42857 -18.2294
0.777778 5.12844 0.0264 0.000785 0.0138 0.0183 0.00676 0.00104 0.0155 0.0131 0.00301
2L:5976160-5976310:minus -33.2347 -0.93578 1.3211 2.57798 -27.1939 -9.5 -29.9266 2.125
-4.19266 0.0276 0.014 0.00738 0.011 0.0267 0.00294 0.09 0.00824 0.0583
2L:5976160-5976310:plus -20.9592 0.93578 0.963303 4.05505 -18.8265 -8.72449 -15.8716
1.80556 -3.11009 0.00184 0.00741 0.00837 0.00566 0.0149 0.00169 0.0106 0.00923 0.0459
2L:5977640-5977790:minus -31.7347 -0.642202 1.41284 -0.412844 -16.8878 -27.898 -8.92661
3.55556 0.788991 0.0149 0.0128 0.00714 0.0304 0.00326 0.0537 0.00252 0.00481 0.0151
2L:5977640-5977790:plus -32.4082 6.55963 1.80734 6.74312 -7.67347 -17.898 -12.3486 1.36111 1.99083
0.0191 0.00081 0.00621 0.0015 0.00055 0.00726 0.00498 0.0108 0.00946
2L:5978640-5978790:minus -31.4388 0.412844 2.33945 -1.06422 -28 -19.1531 -0.412844 -0.833333
7.53211 0.0122 0.00893 0.0051 0.0375 0.0444 0.0177 0.000311 0.0215 0.000968
2L:5978640-5978790:plus -23.1224 -0.275229 3.02752 5.57798 -18.9796 -8.45918 -26.1284 5.5 7.22018

0.00531 0.0113 0.00393 0.0034 0.0155 0.00111 0.0543 0.00209 0.00119
2L:5980220-5980370:minus -22.7653 0.541284 -1 3.19266 -17.1122 -17.7551 -10.8532 -9.45833 3.40367
0.00405 0.00854 0.016 0.00829 0.00341 0.00643 0.00362 0.142 0.00603
2L:5980220-5980370:plus -32.4694 -1.04587 -0.385321 2.06422 -8.86735 -18.0102 -14.5596 2.11111
2.57798 0.0196 0.0145 0.0131 0.0139 0.00173 0.00812 0.00819 0.00828 0.0078
2L:5980960-5981110:minus -22.8571 2.62385 -1.48624 3.87156 10.9898 -7.45918 -16.5872 -1.05556 5.59633
0.00427 0.00399 0.0185 0.00618 3.28e-06 0.000582 0.012 0.0229 0.00234
2L:5980960-5981110:plus -3.52041 13.4592 -1.12844 4.22018 -8.89796 -9.45918 -11.5688 -0.763889
-2.6055 0.000076 0.000064 0.0166 0.00531 0.00189 0.00279 0.0042 0.0211 0.04
2L:5981980-5982130:minus -29.4388 -1.63303 -5.11927 5.47706 -8.67347 -9.94898 -25.1927 -5.56944 -1.90826
0.00842 0.0174 0.0509 0.0036 0.00154 0.00393 0.0484 0.0696 0.0324
2L:5981980-5982130:plus -20.8163 -1.08257 1.98165 2.49541 -18.1531 -17.7857 -20.8899 -0.722222
-2.3211 0.00177 0.0147 0.00582 0.0114 0.00829 0.0068 0.0241 0.0208 0.0365
2L:5982440-5982590:minus -21.7449 4.38532 5.14679 6.73394 -17.7551 -19.1224 -10.3945 1.98611 7.63303
0.00236 0.00207 0.00167 0.00152 0.00658 0.0175 0.00332 0.00866 0.000928
2L:5982440-5982590:plus -12.6327 8.90826 -0.899083 6.78899 -17.1531 -17.7143 -5.92661 3.375
6.6422 0.000455 0.000235 0.0155 0.00146 0.00364 0.00634 0.00129 0.00516 0.00148
2L:5983280-5983430:minus -34.602 3.22018 -0.816514 -1.36697 -18.7245 -17.9388 -15 -5.20833 6.25688
0.032 0.00321 0.0151 0.0416 0.0136 0.00738 0.00897 0.0645 0.00163
2L:5983280-5983430:plus -21.5918 2.82569 9.63303 6.3578 -16.0816 -17.7143 -16.1927 0.569444 5.3945
0.00209 0.00371 0.000177 0.00195 0.00294 0.00634 0.0112 0.014 0.00263
2L:5986220-5986370:minus -22 0.862385 6.15596 2.17431 -27.4592 -9.5 -7.68807 6 5.18349 0.00291
0.00761 0.00108 0.0132 0.0329 0.00294 0.00197 0.00166 0.00295
2L:5986220-5986370:plus -20.9592 4.22018 3.92661 6.89908 -8.27551 -9.23469 -5.88991 -0.555556
-6.27523 0.00184 0.00221 0.00276 0.00134 0.000951 0.00238 0.00127 0.0198 0.0952
2L:5987040-5987190:minus -32.5408 3.57143 -0.926606 -1.08257 -18.3469 -18.9388 -22.8624 7.80556
2.20183 0.0204 0.00175 0.0156 0.0378 0.00894 0.0154 0.0343 0.000651 0.00869
2L:5987040-5987190:plus 5.28571 0.486239 5.31193 10.8624 -8.64286 -8.57143 -24.9817 2.08333 -0.59633
0.0000117 0.00871 0.00156 0.0000687 0.00139 0.00149 0.047 0.00837 0.0229
2L:5997960-5998110:minus -21.5918 -2.85321 4.90826 3.29358 -17.7857 -26.7143 -13.7156 -2.125 1.22018
0.00209 0.0248 0.00185 0.00791 0.00676 0.0257 0.00681 0.0308 0.0133
2L:5997960-5998110:plus -12.1122 1.62385 2.01835 -0.834862 -19.051 -17.9388 -18.7706 -2.66667
4.09174 0.000336 0.00577 0.00574 0.0349 0.0162 0.00738 0.0168 0.0355 0.00416
2L:5999440-5999590:minus -22.398 1.90826 0.477064 1.88991 11.6531 0.27551 -20.5963 0.902778 0.495413
0.00344 0.00519 0.00989 0.0151 1.55e-06 0.000257 0.0229 0.0125 0.0162
2L:5999440-5999590:plus -32.0408 -1.16514 0.0917431 9.92661 -17.4898 -17.2041 -15.6422 -0.111111
-5.22936 0.0174 0.0151 0.0112 0.000169 0.0053 0.00507 0.0102 0.0173 0.0764
2L:602580-602730:minus -22.7653 -0.568807 8.25688 2.83486 -18.7551 -18.5714 -18.5963 -0.166667
1.27523 0.00405 0.0125 0.000382 0.0097 0.0144 0.0136 0.0164 0.0176 0.013
2L:602580-602730:plus -40.2959 0.642202 0.577982 3.05505 0.836735 1.05102 -17.2202 4.09722 1.53211
0.0389 0.00823 0.00955 0.00878 0.000215 0.000107 0.0133 0.00386 0.0116
2L:6036880-6037030:minus -22.9694 1.16514 5.78899 5.21101 -8.64286 -9.5 -20.1927 3.73611 3.88991
0.0048 0.00682 0.00127 0.0042 0.00139 0.00294 0.0213 0.00447 0.00467
2L:6036880-6037030:plus -33.1837 -0.486239 3.33028 3.27523 -27.0102 -9.08163 -11.2936 8.20833
2.05505 0.0274 0.0121 0.00349 0.00796 0.0237 0.00216 0.00396 0.000518 0.00914
2L:6045940-6046090:minus -23.3265 0.0642202 -0.412844 2.7156 -27.0816 -18.051 -6.19266 -0.180556
3.80734 0.00607 0.0101 0.0133 0.0102 0.0243 0.00886 0.00138 0.0177 0.00484
2L:6045940-6046090:plus -24.7857 0.844037 -3.19266 -1.05505 -18.1939 -8.79592 -17.3761 -0.361111
-2.24771 0.00829 0.00766 0.0304 0.0374 0.0086 0.0019 0.0136 0.0187 0.0357
2L:6047880-6048030:minus -23.6531 8.19266 3.94495 6.13761 -8.37755 -26.8265 -17.0092 -1.55556 -0.697248
0.00661 0.000343 0.00274 0.00226 0.00106 0.0285 0.0128 0.0264 0.0239
2L:6047880-6048030:plus -31.4796 0.174312 -4.46789 3.61468 20.2041 -0.540816 -16.0183 1.90278
5.9633 0.0128 0.00971 0.043 0.0068 3.64e-07 0.00054 0.0109 0.00892 0.00188
2L:6048640-6048790:minus -20.9184 13.5306 -1.04587 3.77064 -17.1837 -9.23469 -15.1835 -0.569444

-0.706422 0.00181 0.0000206 0.0162 0.00637 0.00366 0.00238 0.00931 0.0199 0.024
2L:6048640-6048790:plus -30.7347 13.578 15.5596 7.29358 -8.64286 -19.0102 -2.55046 3.75 -1.21101 0.01
0.0000133 6.69e-07 0.00104 0.00139 0.0159 0.000507 0.00445 0.0277
2L:6062780-6062930:minus -31.4796 -2.49541 10.3303 10.5872 -16.7143 -17.7857 -14.6881 -0.513889
1.02752 0.0128 0.0224 0.000117 0.000115 0.00319 0.0068 0.00842 0.0196 0.0141
2L:6062780-6062930:plus -40.7449 -0.779817 1.29358 0.752294 1.14286 -10.0204 -8.19266 3.20833
3.66972 0.0431 0.0134 0.00746 0.0221 0.000113 0.00399 0.00219 0.00551 0.00514
2L:6065940-6066090:minus -22.8469 13.844 15.5229 3.04587 -18.7857 -27.2653 -13.1651 1.58333 5.90826
0.00424 9.98e-06 7.23e-07 0.00883 0.0148 0.0333 0.006 0.00997 0.00198
2L:6065940-6066090:plus -23.4082 13.4592 4.17431 4.91743 -18.4184 -26.898 -13.9725 0.347222 3.42202
0.00623 0.000064 0.0025 0.00447 0.0101 0.0302 0.00721 0.015 0.00597
2L:6069060-6069210:minus -33.3673 3.90816 -2.36697 2.3945 -17.4184 -17.2755 -3.02752 6.5 -6.2844
0.0282 0.00102 0.0241 0.0119 0.00457 0.00544 0.000572 0.0013 0.0954
2L:6069060-6069210:plus -40.9898 0.944954 5.11009 4.6422 -18.7143 -9.5 -15.156 3.48611 -1.56881
0.0455 0.00739 0.0017 0.00473 0.0136 0.00294 0.00926 0.00494 0.0298
2L:6070980-6071130:minus -32.8878 7.84404 2.66972 3.10092 -17.3776 -9.42857 -23.6697 6.73611 -1.00917
0.0247 0.000415 0.0045 0.00865 0.00419 0.0027 0.0391 0.00115 0.0262
2L:6070980-6071130:plus -33.4082 3.26606 3.51376 9.44037 -8.67347 -8.23469 -6.77064 5.22222 2.85321
0.0283 0.00315 0.00325 0.000339 0.00154 0.000875 0.0016 0.00238 0.00733
2L:6082460-6082610:minus -31.102 -3.16514 5.37615 6.11009 -18.3469 10.3776 -25.0917 2.47222 -1.6422
0.0106 0.027 0.00152 0.00233 0.00894 6.32e-06 0.0477 0.00727 0.0304
2L:6082460-6082610:plus -4.85714 1.61468 1.11927 6.47706 -17.6429 -8.72449 -8.91743 0.138889 -0.137615
0.000202 0.00578 0.00793 0.00179 0.00562 0.00169 0.00252 0.016 0.0194
2L:6083180-6083330:minus -24 1.40367 5.17431 -1.83486 -18.3776 -0.173469 -23.8807 2.91667 -8.40367
0.00719 0.00625 0.00165 0.0484 0.00926 0.000456 0.0403 0.00616 0.147
2L:6083180-6083330:plus -40.3673 -3.54128 2.46789 4.11927 10.9898 -8.79592 -12.7156 -1.01389 11.8532
0.0397 0.0299 0.00486 0.00551 3.28e-06 0.0019 0.00542 0.0227 0.0000428
2L:6084920-6085070:minus -22.2653 -1.22018 2.6789 2.88073 -18.3776 -27.9694 -10.6147 1.59722 0.697248
0.00331 0.0154 0.00449 0.00945 0.00926 0.0547 0.00346 0.00993 0.0154
2L:6084920-6085070:plus -33.3776 6.56881 0.513761 -0.220183 -18.4898 -18.8265 -17.7064 8.70833
9.3945 0.0282 0.000806 0.00977 0.0287 0.0116 0.0148 0.0143 0.000384 0.000358
2L:6085760-6085910:minus -14.2143 3.88991 0.844037 2.72477 -27.8673 -0.0918367 -5.97248 -1.58333
-0.394495 0.00124 0.00251 0.00872 0.0102 0.0391 0.000442 0.0013 0.0266 0.0212
2L:6085760-6085910:plus -24.2551 -3.56881 0.321101 2.33945 -17.1122 -7.45918 -16.3853 2.94444 5.18349
0.00768 0.0301 0.0104 0.0122 0.00341 0.000582 0.0116 0.00609 0.00295
2L:6086000-6086150:minus -14.2143 3.88991 0.844037 2.72477 -27.8673 -0.0918367 -5.97248 -1.58333
-0.394495 0.00124 0.00251 0.00872 0.0102 0.0391 0.000442 0.0013 0.0266 0.0212
2L:6086000-6086150:plus -21.9286 8.78899 -1.21101 0.477064 -27.1224 -8.72449 -21.6514 -0.180556
0.66055 0.0027 0.00025 0.017 0.0238 0.0245 0.00169 0.0277 0.0177 0.0156
2L:6087860-6088010:minus -31.4388 -0.486239 -2.20183 -0.880734 -27.2653 -18.0816 -17.2294 4.86111
3.46789 0.0122 0.0121 0.0229 0.0353 0.0302 0.00934 0.0133 0.00279 0.00582
2L:6087860-6088010:plus -22.398 1.9633 2.84404 5.69725 -27.0102 -18.051 -10.5413 -0.569444
5.22936 0.00344 0.00509 0.00421 0.00314 0.0237 0.00886 0.00341 0.0199 0.00288
2L:6088560-6088710:minus -22.6633 4.62385 2.50459 3.18349 -18.6837 -27.2653 -9.44954 -2.26389 9.88073
0.00384 0.00189 0.0048 0.00837 0.0126 0.0333 0.00278 0.032 0.000206
2L:6088560-6088710:plus -23.6327 0.779817 -0.0366972 0.697248 -17.5612 -27.0408 2.18349 1.84722
5.74312 0.00661 0.00784 0.0117 0.0224 0.00544 0.0307 0.000156 0.0091 0.00215
2L:6098920-6099070:minus -32.7347 13.4592 3.9633 2.81651 -26.8571 -9.53061 -18.9817 1.72222 -2.26606
0.0225 0.000064 0.00272 0.00982 0.0198 0.0031 0.0174 0.0095 0.0359
2L:6098920-6099070:plus -22.2959 13.3878 14.0734 1.78899 -17.7551 -8.94898 -6.19266 5.76389 -4.36697
0.00335 0.0000869 6.63e-06 0.0158 0.00658 0.00203 0.00138 0.00185 0.0615
2L:6100240-6100390:minus -24.449 1.76147 -1.36697 5.06422 -8.16327 -10.0204 0.449541 4.43056 1.95413
0.00802 0.00548 0.0179 0.00433 0.000873 0.00399 0.000254 0.00336 0.00964
2L:6100240-6100390:plus -13.8776 3.33945 7.7156 7.17431 -17.449 -8.65306 -8.38532 3.66667 -4.76147

0.0011 0.00307 0.000509 0.00115 0.00484 0.00156 0.00227 0.0046 0.0686
2L:6323660-6323810:minus -32.2245 5.87156 0.165138 5.74312 -27.1939 -0.469388 -16.0642 6.86111
1.27523 0.0183 0.00112 0.011 0.00302 0.0267 0.000533 0.011 0.00108 0.013
2L:6323660-6323810:plus -30.4388 6.61468 1.40367 5.77982 20.2041 -8.93878 -5.50459 0.861111 -0.119266
0.00923 0.000789 0.00717 0.00294 3.64e-07 0.00201 0.00115 0.0127 0.0192
2L:6323940-6324090:minus -32.5204 1.99083 -2.57798 3.07339 -17.4082 0.27551 -17.2661 -3.23611
-0.0458716 0.0204 0.00504 0.0256 0.00873 0.00421 0.000257 0.0134 0.041 0.0187
2L:6323940-6324090:plus -23.0714 -0.944954 3.13761 3.89908 -18.449 -17.5 -6.17431 2.29167
5.33028 0.0052 0.0141 0.00377 0.00604 0.0107 0.00599 0.00137 0.00776 0.00273
2L:6324340-6324490:minus -23.7347 -1.10092 -2.27523 -1.80734 -27.0408 -37.1122 -8.2844 -0.680556
7.37615 0.00673 0.0148 0.0234 0.0479 0.0242 0.133 0.00223 0.0206 0.00107
2L:6324340-6324490:plus -31.9694 -0.0458716 0.412844 0.651376 -18.4592 -17.9796 -25.3853 0.0277778
1.11009 0.0168 0.0105 0.0101 0.0226 0.0109 0.00764 0.0496 0.0166 0.0138
2L:6337960-6338110:minus -32.398 1.6055 6.62385 2.66972 -28.1939 -27.6327 -14.2936 -1.66667 2.16514
0.019 0.0058 0.000865 0.0106 0.0482 0.043 0.00774 0.0272 0.00879
2L:6337960-6338110:plus -21.9592 5.82569 1.81651 7.29358 -18.2245 -16.9796 -18.1468 1.88889 3.56881
0.00279 0.00114 0.00619 0.00104 0.00877 0.0044 0.0153 0.00896 0.00543
2L:6338720-6338870:minus -14.449 0.770642 3.93578 3.81651 -19.0204 0.530612 -12.4128 1.5 -1.06422
0.0014 0.00786 0.00275 0.00626 0.0158 0.000203 0.00506 0.0103 0.0266
2L:6338720-6338870:plus -31.551 -3.41284 4.58716 7.10092 1.5102 0.0204082 -22.4404 -4.41667
-8.91743 0.0134 0.0289 0.00211 0.00121 0.0000722 0.000377 0.0319 0.0542 0.163
2L:6339280-6339430:minus -32.8469 7.85321 -0.715596 2.77064 -18.7143 -18.0816 2.0367 -1.02778
-1.08257 0.0243 0.000413 0.0146 0.01 0.0136 0.00934 0.000163 0.0227 0.0267
2L:6339280-6339430:plus -29.2143 -1.72477 2.57798 7.73394 -17.0408 -8.53061 -15.2844 3.47222 7.83486
0.00836 0.0179 0.00466 0.000669 0.00329 0.00136 0.00949 0.00497 0.000712
2L:6340380-6340530:minus -31.398 3.74312 -5.10092 0.633028 -7.89796 -16.3469 -20.1743 4.48611 0.862385
0.0117 0.00265 0.0506 0.0229 0.000678 0.00416 0.0212 0.00328 0.0148
2L:6340380-6340530:plus -24.4184 1.6789 0.513761 -0.0550459 -8.27551 -18.1224 -16.7706 2.97222
-1.68807 0.00797 0.00565 0.00977 0.0272 0.000951 0.00988 0.0124 0.00603 0.0308
2L:6340860-6341010:minus -24.1837 0.733945 7.91743 6.3578 -18.4184 9.37755 -6.42202 4.98611 -4.34862
0.00751 0.00797 0.000457 0.00195 0.0101 0.0000363 0.00146 0.00264 0.0612
2L:6340860-6341010:plus -31.7347 2.44954 0.137615 2.54128 -18.6837 -9.20408 -12.7798 14.0556 7.01835
0.0149 0.00425 0.0111 0.0112 0.0126 0.00225 0.0055 2.35e-06 0.00134
2L:6341340-6341490:minus -14.4796 -0.715596 5.18349 7.21101 -27.1939 -8.5 -11.0734 -0.0694444
3.41284 0.00142 0.0131 0.00165 0.0011 0.0267 0.00126 0.00379 0.0171 0.006
2L:6341340-6341490:plus -34.2959 -2.36697 0.944954 1.08257 -28.2653 -17.051 -14.1376 2.66667 5.53211
0.0316 0.0216 0.00842 0.0201 0.0537 0.00479 0.00748 0.00677 0.00245
2L:6411260-6411410:minus 6.28571 3.89908 5.9633 7.62385 -18.6837 -18.7143 -2.37615 0.0277778
0.348624 4.12e-06 0.0025 0.00117 0.000759 0.0126 0.014 0.000486 0.0166 0.0167
2L:6411260-6411410:plus -31.6224 -2.69725 3.12844 -0.587156 -27.0408 -16.5 -5.93578 6.09722
3.6789 0.0137 0.0237 0.00378 0.0322 0.0242 0.00416 0.00129 0.00158 0.00506
2L:6412080-6412230:minus -31.6633 3.77064 2.12844 5.22936 -27.3469 -9.57143 -5.07339 1.375 2.63303
0.0141 0.00262 0.00551 0.0041 0.0318 0.00326 0.00101 0.0107 0.00771
2L:6412080-6412230:plus -31.7347 -1.0367 13.7431 11.1927 -26.1939 -27.8265 -14.1835 -0.180556
3.88073 0.0149 0.0145 0.0000103 0.0000254 0.0175 0.0498 0.00755 0.0177 0.00475
2L:6422700-6422850:minus -22.9592 -0.376147 -0.486239 3.49541 -18.449 -26.5306 -5.56881 2.94444
1.31193 0.00475 0.0117 0.0136 0.00716 0.0107 0.023 0.00117 0.00609 0.0127
2L:6422700-6422850:plus -23.3776 9.59633 2.48624 2.77064 -6.67347 -27.8571 -10.8899 0.236111 -2.82569
0.00619 0.000164 0.00483 0.01 0.000359 0.0508 0.00365 0.0155 0.0425
2L:6423380-6423530:minus -13.4388 3.29358 10.3303 10.5872 -26.7449 -17.7857 -3.08257 8.36111 4.09174
0.000879 0.00312 0.000117 0.000115 0.0195 0.0068 0.00058 0.000474 0.00416
2L:6423380-6423530:plus -23.8878 2.62385 2.46789 6.56881 -8.89796 -26.8265 -6.24771 -0.638889
0.706422 0.00692 0.00399 0.00486 0.0017 0.00189 0.0285 0.0014 0.0203 0.0154
2L:6424260-6424410:minus -33.9286 3.09174 0.688073 3.05505 -18.5306 -9.5 -9.7156 2.19444 -3.74312

0.0303 0.00337 0.0092 0.00878 0.0118 0.00294 0.00292 0.00804 0.0521
2L:6424260-6424410:plus -31.7041 1.21101 -1 2.66972 2.14286 -9.5 -15.1927 3.83333 3.88991 0.0146
0.00671 0.016 0.0106 0.0000316 0.00294 0.00932 0.0043 0.00467
2L:6448320-6448470:minus -21.9286 -5.16514 1.26606 2.52294 -27.7449 -16.7857 -10.2202 1.77778 3.08257
0.0027 0.0461 0.00753 0.0113 0.0383 0.00426 0.00321 0.00932 0.00681
2L:6448320-6448470:plus -33.0408 -1.77982 2.80734 5.63303 -27.3061 -19.1224 -16.8349 5.94444 5.3945
0.0264 0.0182 0.00427 0.00326 0.0315 0.0175 0.0125 0.0017 0.00263
2L:6455140-6455290:minus -20.7449 0.440367 -1.80734 2.69725 -17.449 -17.4898 -17.0917 1.51389 8.19266
0.00176 0.00885 0.0204 0.0104 0.00484 0.0059 0.013 0.0102 0.000548
2L:6455140-6455290:plus -22.2551 3.45872 8.18349 2.90826 -18.2653 -9.02041 -3.09174 5.69444 5.22018
0.00327 0.00294 0.000397 0.00936 0.00882 0.00213 0.000582 0.00191 0.00289
2L:6465640-6465790:minus -31.551 -0.302752 3.49541 6.72477 -18.4898 -26.602 -10.3119 -2.91667
4.55963 0.0134 0.0114 0.00327 0.00154 0.0116 0.0248 0.00326 0.0378 0.00357
2L:6465640-6465790:plus -31.5918 -2.70642 2.55963 7.46789 -17.6837 -9.0102 -20.7706 4.20833 -2.21101
0.0136 0.0238 0.0047 0.000868 0.0058 0.00208 0.0236 0.00369 0.0354
2L:6467660-6467810:minus -32.4796 5.44037 2.85321 2.40367 10.9898 -9.53061 -10.7064 0.888889 3.31193
0.02 0.00135 0.0042 0.0119 3.28e-06 0.0031 0.00352 0.0126 0.00618
2L:6467660-6467810:plus -32.5204 4.41284 1.12844 0.513761 -7.82653 -9.23469 -12.5505 -0.0833333
6.73394 0.0204 0.00205 0.00791 0.0235 0.00059 0.00238 0.00522 0.0172 0.00143
2L:6473420-6473570:minus -31.449 -3.19266 0.412844 2.90826 -26.9286 -8.5 -27.2569 2.375 2.53211
0.0123 0.0272 0.0101 0.00936 0.0214 0.00126 0.0625 0.00753 0.00791
2L:6473420-6473570:plus -21.6633 0.513761 2.47706 9.33028 -17.7143 -17.0102 -17.7431 -4.05556 2.20183
0.00223 0.00862 0.00484 0.000374 0.00627 0.00453 0.0144 0.0499 0.00869
2L:6476920-6477070:minus -23.2551 13.4587 -1.27523 1.82569 -17.7143 -9.23469 -4.69725 -0.430556
-2.72477 0.00583 0.0000146 0.0174 0.0156 0.00627 0.00238 0.00091 0.0191 0.0414
2L:6476920-6477070:plus -41.8163 5.46789 1.70642 3.24771 -18.9796 -17.9388 -9.85321 2.83333 -4.65138
0.0662 0.00134 0.00644 0.00808 0.0155 0.00738 0.00299 0.00635 0.0664
2L:6479400-6479550:minus -31.2143 1.87156 5.55046 1.30275 -17.1837 -10.0918 -20.9083 -1.22222 7.58716
0.0111 0.00526 0.00141 0.0189 0.00366 0.00404 0.0242 0.024 0.000954
2L:6479400-6479550:plus -22.7449 -4.22018 4.81651 3.80734 -27.3061 -25.5306 -21.6239 0.680556 10.3394
0.00404 0.0359 0.00192 0.00627 0.0315 0.0205 0.0275 0.0135 0.000112
2L:6479740-6479890:minus -20.9184 0.247706 3.21101 6.84404 -8.71429 -19.051 -14.2936 1.11111
-0.0733945 0.00181 0.00947 0.00366 0.0014 0.00159 0.0165 0.00774 0.0117 0.0188
2L:6479740-6479890:plus -14.2245 -0.46789 -0.366972 0.0458716 0.836735 -36.9286 -17.055 5.76389
9.14679 0.00126 0.0121 0.0131 0.0266 0.000215 0.123 0.0129 0.00185 0.000414
2L:6480500-6480650:minus -3.81633 -0.220183 -3.01835 7.27523 -18.0816 -9.16327 -13.2844 -1.91667
2.16514 0.000106 0.0111 0.029 0.00109 0.00759 0.00223 0.00617 0.0291 0.00879
2L:6480500-6480650:plus -32.4082 3.61224 0.743119 2.57798 20.4694 -26.6735 -9.82569 1.77778 -3.29358
0.0191 0.00167 0.00903 0.011 1.65e-07 0.0256 0.00298 0.00932 0.0474
2L:6480820-6480970:minus -41.3265 8.00917 4.54128 0.853211 0.806122 -25.898 -13.4495 1 -3.12844
0.0541 0.00038 0.00215 0.0214 0.000229 0.0214 0.00641 0.0121 0.046
2L:6480820-6480970:plus -20.8878 -1.9633 1.79817 2.86239 -17.1531 -19.2755 -14.1651 -0.75 4.02752
0.0018 0.0192 0.00623 0.00954 0.00364 0.0185 0.00752 0.021 0.00428
2L:6481400-6481550:minus -22.7041 3.07339 2.97248 5.59633 -17.6735 -8.30612 -12.5872 1 0.642202
0.00395 0.00339 0.00401 0.00331 0.00572 0.000973 0.00526 0.0121 0.0157
2L:6481400-6481550:plus -13.1122 -1.75229 -0.165138 7.47706 -8.60204 -27.1224 -14.3945 1.59722
9.75229 0.000632 0.0181 0.0122 0.00085 0.00125 0.0325 0.00791 0.00993 0.000241
2L:6488960-6489110:minus -32.6939 -1.93578 -2.48624 4.79817 -27.2653 -18.7857 -23.9174 3.02778 -4.84404
0.0217 0.0191 0.0249 0.00453 0.0302 0.0146 0.0405 0.0059 0.0701
2L:6488960-6489110:plus -14.0102 10.9174 0.12844 7.88991 -17.7857 -9.16327 -11.4771 1.625 3.88991
0.00114 0.0000843 0.0111 0.00062 0.00676 0.00223 0.00412 0.00983 0.00467
2L:6490860-6491010:minus -14.6327 0.577982 9.91743 3.79817 11.3571 0.755102 -20.0459 9.93056 7.42202
0.0015 0.00843 0.00015 0.00629 2.44e-06 0.000175 0.0208 0.000172 0.00106
2L:6490860-6491010:plus -14.4388 0.853211 16.2202 6.21101 -17.6429 -18.0102 -17.2018 -0.583333

-0.715596 0.00137 0.00763 1.24e-07 0.00213 0.00562 0.00812 0.0132 0.02 0.0241
2L:6494520-6494670:minus -4.47959 2.82569 13.4862 10.0275 -17.0816 -17.0816 -8.08257 -0.736111
9.88073 0.000154 0.00371 0.0000132 0.000156 0.00334 0.00487 0.00214 0.0209 0.000206
2L:6494520-6494670:plus -30.102 -0.568807 1.55963 -0.761468 -26.5918 -0.469388 -20.4862
-0.791667 -8.33945 0.00859 0.0125 0.00678 0.0341 0.0186 0.000533 0.0224 0.0212 0.145
2L:6498580-6498730:minus -33.0408 2.18349 0.889908 1.57798 -26.5306 -17.5714 -23.156 6.625 1.27523
0.0264 0.00469 0.00859 0.0171 0.0185 0.00616 0.036 0.00122 0.013
2L:6498580-6498730:plus -13 3.97959 1.41284 7.43119 1.17347 -16.7857 -3.10092 -9.15278 1.01835
0.000563 0.000669 0.00714 0.000939 0.000101 0.00426 0.000583 0.135 0.0142
2L:6500160-6500310:minus -32.7755 5.83486 9.7156 1.59633 -18.9796 -18.8265 -22.8165 4.36111 1.78899
0.0234 0.00114 0.000168 0.017 0.0155 0.0148 0.034 0.00346 0.0103
2L:6500160-6500310:plus -32.7347 4.92661 3.10092 5.37615 -18.2245 -0.244898 -10.9083 0.833333
1.57798 0.0225 0.00168 0.00382 0.00384 0.00877 0.000496 0.00366 0.0128 0.0114
2L:6536160-6536310:minus 5.04082 3.97959 6.82569 11.2936 -17.4184 -9.53061 8.31193 5.44444 7.42202
0.0000162 0.000669 0.000788 0.0000139 0.00457 0.0031 0.0000218 0.00215 0.00106
2L:6536160-6536310:plus -22.9286 -2.86239 -0.724771 3.3578 -18.2245 9.60204 -20.055 -3.69444
2.74312 0.0046 0.0248 0.0146 0.00767 0.00877 0.0000248 0.0208 0.0458 0.00751
2L:6557620-6557770:minus -32.2449 6.45872 -0.678899 4.13761 10.6939 -27.8265 -13.0367 -0.888889
8.0367 0.0183 0.000851 0.0144 0.00546 7.35e-06 0.0498 0.00583 0.0218 0.000654
2L:6557620-6557770:plus -33.0714 3.09174 4.88991 2.78899 20.4694 9.60204 -20.6697 0.833333 3.88073
0.0267 0.00337 0.00186 0.00992 1.65e-07 0.0000248 0.0232 0.0128 0.00475
2L:6561580-6561730:minus -21.8571 5.17431 -2.17431 3.6055 -27.2653 -7.65306 -25.0367 3.73611 -6.11927
0.00249 0.00151 0.0227 0.00686 0.0302 0.000647 0.0474 0.00447 0.0914
2L:6561580-6561730:plus -33.5204 -1.73394 5.37615 5.06422 -26.4898 -17.3469 -26.0183 1.18056 -2.91743
0.0287 0.018 0.00152 0.00433 0.0183 0.0058 0.0536 0.0114 0.0436
2L:6562140-6562290:minus -21.9592 5.6789 -5.16514 6.66055 -17.4184 -18.9286 -12.7156 1.02778 8.0367
0.00279 0.00122 0.0515 0.00158 0.00457 0.0153 0.00542 0.012 0.000654
2L:6562140-6562290:plus -21.5918 1.30275 10.7064 4.46789 -18.1939 -18.7857 -5.36697 -3.40278 1.43119
0.00209 0.00649 0.0000942 0.0049 0.0086 0.0146 0.0011 0.0427 0.0121
2L:6610960-6611110:minus -42 0.422018 4.11927 2.81651 -26.5306 -17.4184 -25.3394 3.97222 4.51376
0.0684 0.0089 0.00255 0.00982 0.0185 0.00585 0.0493 0.00407 0.0036
2L:6610960-6611110:plus -12.9184 1.57798 13.7431 3.88991 10.6531 -18.9286 -11.5138 -0.555556
1.54128 0.000532 0.00586 0.0000103 0.00608 0.0000102 0.0153 0.00415 0.0198 0.0115
2L:6611680-6611830:minus -13.449 2.84404 -0.201835 4.46789 -27.2347 -27.3776 -9.22936 8.29167
9.04587 0.000914 0.00368 0.0124 0.0049 0.0283 0.0351 0.00267 0.000493 0.000423
2L:6611680-6611830:plus -32.8469 5.12844 10.7982 6.21101 -18.5306 -16.9796 -16.2202 1.11111 -0.59633
0.0243 0.00154 0.0000898 0.00213 0.0118 0.0044 0.0113 0.0117 0.0229
2L:6612440-6612590:minus -22.1531 1.45872 4.79817 2.9633 -17.5306 -8.45918 -23.1376 1.41667 -0.174312
0.0031 0.00613 0.00193 0.00914 0.0054 0.00111 0.0359 0.0106 0.0196
2L:6612440-6612590:plus -14.4796 0.458716 7.2844 7.21101 0.653061 -17.7857 -12.2018 0.958333 3.66972
0.00142 0.00879 0.000632 0.0011 0.000239 0.0068 0.00482 0.0123 0.00514
2L:6641060-6641210:minus -22.6633 4.04587 7.90826 7.6422 -27.1939 -0.0510204 -16.6239 1.25 11.3853
0.00384 0.00236 0.00046 0.000721 0.0267 0.000429 0.0121 0.0112 0.0000815
2L:6641060-6641210:plus -12.5918 -2.77064 0.458716 1.12844 -17.7551 -0.540816 -15.6972 0.402778
-0.0275229 0.000449 0.0242 0.00995 0.0198 0.00658 0.00054 0.0103 0.0147 0.0186
2L:6644100-6644250:minus -31.9286 -1.09174 -0.93578 9.08257 -18.2245 -27.9694 -14.3945 2.36111 2.42202
0.0166 0.0148 0.0156 0.000444 0.00877 0.0547 0.00791 0.00757 0.00816
2L:6644100-6644250:plus -31.398 -0.422018 1.16514 2.41284 -28.1939 -18.5714 -13.4954 8.65278
-3.61468 0.0117 0.0119 0.0078 0.0118 0.0482 0.0136 0.00648 0.000397 0.0509
2L:6644640-6644790:minus -22.9592 -1.82569 -2.48624 3.31193 -25.9694 -8.65306 -16.8532 -0.930556
-7.59633 0.00475 0.0185 0.0249 0.00786 0.017 0.00156 0.0125 0.0221 0.126
2L:6644640-6644790:plus -33.9184 -5.47706 -1.88073 -1.19266 -18.5612 -46.8469 -5.73394 4.48611 9.92661
0.0302 0.05 0.0209 0.0391 0.0119 0.337 0.00122 0.00328 0.000193
2L:6645320-6645470:minus -31.9184 -1.58716 9.49541 3.43119 -18.7551 -19.3061 -11.2202 1.45833 5.66055

0.0164 0.0172 0.000191 0.00749 0.0144 0.0187 0.0039 0.0104 0.00227
2L:6645320-6645470:plus -21.9184 -2.65138 9.74312 4.07339 -17.6735 -18.0102 -15.7431 2.44444 -3.57798
0.00261 0.0234 0.000166 0.00562 0.00572 0.00812 0.0104 0.00734 0.0506
2L:6647360-6647510:minus -34.449 1.33028 0.477064 2.69725 -9.20408 -17.5612 -18.5229 0.986111 3.88991
0.0319 0.00642 0.00989 0.0104 0.00262 0.00604 0.0162 0.0122 0.00467
2L:6647360-6647510:plus -20.6633 3.00917 0.302752 7.63303 -18.7143 -18.051 -9.94495 2.875 5.74312
0.00173 0.00347 0.0105 0.000745 0.0136 0.00886 0.00305 0.00626 0.00215
2L:6648140-6648290:minus -21.9184 -4.38532 -0.981651 6.36697 -8.37755 -27.5612 -20.4771 4.09722
-0.40367 0.00261 0.0375 0.0159 0.00193 0.00106 0.0393 0.0224 0.00386 0.0213
2L:6648140-6648290:plus -33.102 3.30275 -1.78899 -0.0183486 -8.96939 -9.09184 -11.7982 3.98611
-0.284404 0.0268 0.00311 0.0203 0.027 0.00226 0.00219 0.00441 0.00404 0.0207
2L:6648520-6648670:minus -21.8163 -1.89908 -0.348624 2.06422 -26 -9.08163 -5.15596 6.80556 9.9633
0.00244 0.0189 0.013 0.0139 0.0171 0.00216 0.00104 0.00111 0.000171
2L:6648520-6648670:plus -14.8163 0.174312 5.6055 0.651376 -17.7449 -27.5306 -16.5138 0.111111 1.61468
0.00159 0.00971 0.00137 0.0226 0.0063 0.0374 0.0118 0.0162 0.0112
2L:6649180-6649330:minus -31.8163 1.83486 7.3945 7.21101 10.3878 -17.8265 -22.0459 2.875 4.98165
0.016 0.00533 0.000597 0.0011 0.0000174 0.00699 0.0297 0.00626 0.00313
2L:6649180-6649330:plus -23.1837 -4.78899 8.83486 7.45872 10.6224 -26.8265 -10.2018 3.11111 1.49541
0.00553 0.0417 0.000278 0.000894 0.0000113 0.0285 0.0032 0.00572 0.0118
2L:6656300-6656450:minus -31.7347 1.9633 14.6147 2.92661 -9.5 -18.7857 -20.8899 6.08333 -0.761468
0.0149 0.00509 3.32e-06 0.00931 0.00293 0.0146 0.0241 0.00159 0.0244
2L:6656300-6656450:plus -22.9286 0.954128 7.66972 6.02752 -7.93878 -18.051 -13.1284 2.80556 1.74312
0.0046 0.00736 0.000521 0.00246 0.000767 0.00886 0.00595 0.00642 0.0105
2L:6658400-6658550:minus -34.2653 0.458716 8.07339 7.55963 1.65306 -17.2755 -26.9725 -1.81944 -2.40367
0.0316 0.00879 0.000421 0.000807 0.0000586 0.00544 0.0603 0.0284 0.0376
2L:6658400-6658550:plus -40.2857 -4 7.07339 5.58716 20.1735 19.0816 -14.3211 1.33333 -4.40367
0.0385 0.0338 0.000703 0.00334 5.68e-07 1.12e-06 0.00778 0.0109 0.0623
2L:6663900-6664050:minus -32.6327 -1.01835 4.38532 1.33028 -27.2653 -18.5714 -30.0734 -1.90278 5.18349
0.0212 0.0144 0.0023 0.0188 0.0302 0.0136 0.0919 0.029 0.00295
2L:6663900-6664050:plus -32.7755 -0.293578 -1.48624 2.25688 -26.8571 -18.2755 -2.7156 -3.19444
1.37615 0.0234 0.0114 0.0185 0.0127 0.0198 0.0112 0.000528 0.0406 0.0123
2L:6667860-6668010:minus -2.40816 0.0550459 3.27523 5.31193 -17.7551 -26.6327 -8.24771 0.402778
1.48624 0.0000315 0.0101 0.00357 0.00393 0.00658 0.0251 0.00221 0.0147 0.0118
2L:6667860-6668010:plus -23 3.77982 -0.605505 0.550459 -27.1939 -7.86735 -21.0642 5.23611 7.15596
0.00498 0.00261 0.0141 0.0233 0.0267 0.00073 0.0249 0.00236 0.00124
2L:6673300-6673450:minus -21.3571 5.05505 3.47706 10.7615 -7.56122 -27.5612 -5.59633 6.43056 1.00917
0.00194 0.00159 0.0033 0.0000826 0.000419 0.0393 0.00117 0.00134 0.0143
2L:6673300-6673450:plus -21.551 0.33945 4.22018 2.43119 -8.60204 -18.7551 -12.6972 5.22222 7.45872
0.00205 0.00917 0.00245 0.0117 0.00125 0.0141 0.00539 0.00238 0.00103
2L:6673800-6673950:minus -34.1531 2.13761 2.70642 2.23853 -27.9694 -8.79592 -14.6881 6.88889 3.66972
0.0314 0.00477 0.00444 0.0128 0.0437 0.0019 0.00842 0.00107 0.00514
2L:6673800-6673950:plus -31.5204 -1.61468 14.6789 4.33945 -18.5612 -8.27551 -19.4679 2.41667 3.88073
0.0131 0.0173 3.13e-06 0.00509 0.0119 0.000919 0.0188 0.00742 0.00475
2L:6682980-6683130:minus -20.9286 4.79817 -1.95413 9.87156 0.581633 -7.79592 -22.5688 1.83333 1.15596
0.00183 0.00177 0.0213 0.000195 0.000292 0.000723 0.0326 0.00914 0.0135
2L:6682980-6683130:plus -31.7755 -1.15596 3.80734 5.73394 -8.86735 -18.0102 -12.2018 -1.27778 0.66055
0.0157 0.0151 0.0029 0.00307 0.00173 0.00812 0.00482 0.0244 0.0156
2L:6684300-6684450:minus -3.26531 0.394495 4.55046 7.90826 20.4694 0.0204082 -19.0367 3.81944
1.16514 0.0000515 0.00899 0.00214 0.000611 1.65e-07 0.000377 0.0176 0.00433 0.0135
2L:6684300-6684450:plus -32.7755 1.53211 -3.70642 5.89908 -17.1531 0.826531 -13.0367 -1.125 -3.75229
0.0234 0.00596 0.0351 0.00272 0.00364 0.000153 0.00583 0.0234 0.0521
2L:66860-67010:minus -30.4082 4.10092 2.72477 5.45872 -8.64286 -7.16327 -20.4954 7.51389 -0.614679
0.00908 0.00231 0.00441 0.00363 0.00139 0.000547 0.0225 0.000766 0.0231
2L:66860-67010:plus -14.2551 3.94495 1.99083 9.49541 -18.4184 -8.45918 -10.5688 0.902778 -1 0.00127

0.00245 0.0058 0.000308 0.0101 0.00111 0.00343 0.0125 0.0261
2L:6709160-6709310:minus -29.9184 0.247706 -2.07339 9.7156 -26.898 -17.2755 -12.9633 -1.75 -0.192661
0.00854 0.00947 0.0221 0.000242 0.0203 0.00544 0.00573 0.0279 0.0198
2L:6709160-6709310:plus -13.5918 -1.02752 3.20183 5.55046 -18.5306 -18.0816 -19.9266 1.11111 2.58716
0.00099 0.0145 0.00367 0.00345 0.0118 0.00934 0.0203 0.0117 0.00779
2L:6717520-6717670:minus -33.5306 4.74312 -0.587156 4.02752 -27.4898 -8.65306 -29.2661 2.68056
-6.01835 0.0287 0.00181 0.014 0.00581 0.0336 0.00156 0.082 0.00673 0.0892
2L:6717520-6717670:plus -23.1837 7.58716 -3.70642 9.88073 -27.1939 -17.9388 -20.1009 7.52778 -6.01835
0.00553 0.000476 0.0351 0.000183 0.0267 0.00738 0.021 0.00076 0.0892
2L:67220-67370:minus -31.2143 0 3.57798 2.23853 -8.16327 -8.72449 -8.41284 0.375 11.8073 0.0111
0.0103 0.00317 0.0128 0.000873 0.00169 0.00229 0.0149 0.0000538
2L:67220-67370:plus -22 -2.80734 0.825688 1.74312 -18.9796 -17.3469 -14.9083 1.69444 2.33028 0.00291
0.0244 0.00878 0.016 0.0155 0.0058 0.00881 0.0096 0.00836
2L:6723340-6723490:minus -4.78571 12.844 3.90826 2.9633 -18.449 -17.2041 -18.2661 1.83333 1.22936
0.000194 0.0000255 0.00278 0.00914 0.0107 0.00507 0.0156 0.00914 0.0132
2L:6723340-6723490:plus -32.7347 11.367 2.91743 0.770642 -17.449 -18.3469 -9.92661 6.76389 5.49541
0.0225 0.000067 0.0041 0.0219 0.00484 0.0124 0.00304 0.00114 0.0025
2L:6723740-6723890:minus -33.3776 0.807339 0.33945 4.08257 -27.5 -8.5 -22.1468 3.02778 -4.68807
0.0282 0.00776 0.0104 0.00558 0.0337 0.00126 0.0303 0.0059 0.0672
2L:6723740-6723890:plus -30.898 5.73394 5.26606 6.3578 10.3878 9.86735 -28.9541 2 -7.23853
0.0103 0.00119 0.00159 0.00195 0.0000174 0.0000132 0.0784 0.00862 0.118
2L:6785820-6785970:minus -31.4082 4.85321 -1.05505 3.61468 -18.1531 -27.1224 -13.4495 4.63889 0.146789
0.0119 0.00173 0.0162 0.0068 0.00829 0.0325 0.00641 0.00307 0.0178
2L:6785820-6785970:plus -32.4796 1.46789 3.23853 2.09174 -27.1531 -17.2041 -18.844 -0.527778
4.02752 0.02 0.0061 0.00362 0.0137 0.0249 0.00507 0.017 0.0196 0.00428
2L:6786880-6787030:minus -11.0816 1.22018 -0.376147 5.84404 -27.0102 -9.20408 -6.88073 -8.36111
9.87156 0.000242 0.00668 0.0131 0.00289 0.0237 0.00225 0.00164 0.118 0.000217
2L:6786880-6787030:plus -31.7347 -1.25688 0.357798 4.91743 -8.56122 -17.1224 -6.75229 0.5 2.82569
0.0149 0.0155 0.0103 0.00447 0.00112 0.00499 0.00159 0.0143 0.0074
2L:6787420-6787570:minus -14.7143 5.38532 7.84404 6.56881 -17.4898 -18.0816 -6.83486 2.13889 0.394495
0.00154 0.00139 0.000476 0.0017 0.0053 0.00934 0.00162 0.0082 0.0166
2L:6787420-6787570:plus -24.1122 -2.04587 7.14679 7.20183 -9.0102 -27.3367 -16.7064 4.06944 3.92661
0.00738 0.0197 0.000677 0.00112 0.00236 0.0347 0.0122 0.00391 0.00461
2L:6792100-6792250:minus -29.4082 0.733945 -1.33945 4.08257 -27.2347 -18.1224 -19.7798 5.26389 7.49541
0.0084 0.00797 0.0177 0.00558 0.0283 0.00988 0.0198 0.00233 0.000998
2L:6792100-6792250:plus -31.5204 -0.0183486 -1.06422 7.6055 -25.8571 -15.9796 -21.1651 3.09722
3.07339 0.0131 0.0104 0.0163 0.000794 0.0166 0.00405 0.0253 0.00575 0.00683
2L:6794180-6794330:minus -32.1837 -1.22018 -0.412844 5.57798 -26.3061 -17.2755 -17.1651 7.34722
-0.0733945 0.0181 0.0154 0.0133 0.0034 0.0181 0.00544 0.0132 0.000838 0.0188
2L:6794180-6794330:plus -31.7041 0.110092 3.12844 -0.550459 -17.4898 -16.0102 -13.2202 0.347222
3.87156 0.0146 0.00993 0.00378 0.0317 0.0053 0.00406 0.00608 0.015 0.00477
2L:6794600-6794750:minus -23.1939 8.83486 -3.29358 10.0275 -18.6429 -18.7857 -19.578 -0.666667
3.08257 0.00558 0.000244 0.0313 0.000156 0.0121 0.0146 0.0192 0.0205 0.00681
2L:6794600-6794750:plus -22.0306 0.137615 -0.899083 4.65138 -17.7857 -7.72449 -4.44954 0.152778
1.53211 0.00293 0.00984 0.0155 0.00471 0.00676 0.00068 0.000848 0.016 0.0116
2L:6852120-6852270:minus -23.2245 2.22018 0.706422 6.00917 -17.7143 -9.5 -20.8716 3.34722 4.98165
0.00576 0.00463 0.00914 0.00255 0.00627 0.00294 0.024 0.00522 0.00313
2L:6852120-6852270:plus -22.8878 3.7551 2.0367 5.38532 20.4694 9.53061 -13.1009 3.875 -1.38532
0.00441 0.00122 0.0057 0.00382 1.65e-07 0.0000316 0.00592 0.00423 0.0288
2L:6854020-6854170:minus -32.0306 6.27523 11.4312 3.48624 -26.9694 -8.5 -20.6147 -2.06944 0.981651
0.0172 0.00093 0.0000613 0.0072 0.023 0.00126 0.0229 0.0304 0.0144
2L:6854020-6854170:plus -3.34694 -2.12844 0.66055 4.23853 0.806122 -18.0816 -15.1284 0.958333 4.04587
0.0000599 0.0202 0.00929 0.00522 0.000229 0.00934 0.00921 0.0123 0.00421
2L:6907880-6908030:minus -23.5204 0.495413 5.26606 6.42202 -18.7551 -18.3061 -6.83486 -3 -2.56881

0.00645 0.00868 0.00159 0.00188 0.0144 0.0114 0.00162 0.0386 0.0396
2L:6907880-6908030:plus -22.6633 1.54128 1.08257 2.70642 1.17347 -9.57143 -12.7615 1.94444 5.34862
0.00384 0.00594 0.00803 0.0103 0.000101 0.00326 0.00547 0.00879 0.00271
2L:6914140-6914290:minus -21.6633 -3.62385 4.99083 9.51376 0.806122 0.27551 -11.7706 -0.111111
0.66055 0.00223 0.0306 0.00178 0.000292 0.000229 0.000257 0.00439 0.0173 0.0156
2L:6914140-6914290:plus -22.8469 6.20183 2.87156 3.3578 -26.9592 -7.45918 -17.0183 2.26389 -0.981651
0.00424 0.000962 0.00417 0.00767 0.0215 0.000582 0.0129 0.00784 0.026
2L:6918720-6918870:minus -32.8469 -1.07339 3.44954 5.57798 -17.6429 0.826531 -11.4037 -0.305556
3.88991 0.0243 0.0147 0.00334 0.0034 0.00562 0.000153 0.00406 0.0184 0.00467
2L:6918720-6918870:plus -31.6633 -2.61468 1.53211 -1.2844 -17.3776 -27.8571 -15.9541 4.75 6.95413
0.0141 0.0232 0.00685 0.0402 0.00419 0.0508 0.0108 0.00293 0.00136
2L:6919260-6919410:minus -12.449 -1.21101 2.50459 -0.165138 -26.9592 -18.3469 -4.58716 4.5 -0.284404
0.000428 0.0153 0.0048 0.0282 0.0215 0.0124 0.000882 0.00326 0.0207
2L:6919260-6919410:plus -22.1939 -1.10092 -1.55046 0.220183 -27.602 -7.5 -24.9633 5.44444 3 0.00317
0.0148 0.0189 0.0253 0.0364 0.000614 0.0469 0.00215 0.00702
2L:6919900-6920050:minus -21.7347 4.97248 3.22936 -0.165138 -8.96939 -27.8571 -4.23853 2.59722
5.66055 0.00235 0.00165 0.00363 0.0282 0.00226 0.0508 0.000798 0.00694 0.00227
2L:6919900-6920050:plus -31.2143 10.2752 0.834862 7.43119 -8.64286 -17.8265 -19.5872 0.541667 -0.963303
0.0111 0.000117 0.00875 0.000939 0.00139 0.00699 0.0192 0.0141 0.0258
2L:6920200-6920350:minus -23.449 -1.29358 2.06422 6.11009 10.9898 19.0816 -19.6422 3.76389 -0.275229
0.00631 0.0157 0.00564 0.00233 3.28e-06 1.12e-06 0.0194 0.00442 0.0206
2L:6920200-6920350:plus -24.4388 1.04587 -1.9633 2.82569 -27.7551 -17.5714 -14.789 6.72222 7.16514
0.00797 0.00712 0.0214 0.00976 0.0385 0.00616 0.00859 0.00116 0.00122
2L:6920840-6920990:minus -23.0306 -2.66972 8.36697 3.89908 -8.40816 -9.16327 -9.20183 -1.08333 5.44037
0.00505 0.0235 0.00036 0.00604 0.00108 0.00223 0.00266 0.0231 0.00259
2L:6920840-6920990:plus -13.0714 3.97959 7.75229 4.07339 -17.7959 -17.051 -4.66055 -0.111111
-0.706422 0.000594 0.000669 0.000499 0.00562 0.00678 0.00479 0.000901 0.0173 0.024
2L:6921100-6921250:minus -24.449 1.10092 -0.587156 1.92661 -27.2653 -7.72449 -29.055 8.93056
3.52294 0.00802 0.00698 0.014 0.0148 0.0302 0.00068 0.0795 0.000335 0.00558
2L:6921100-6921250:plus -22.7041 -3.40367 7.65138 1.77982 -17.4082 -17.3469 -17.8624 5.81944 -2.26606
0.00395 0.0288 0.000526 0.0158 0.00421 0.0058 0.0146 0.00181 0.0359
2L:6921580-6921730:minus -30.5918 -3.79817 1.62385 9.61468 -16.4184 -0.204082 -11.7523 2.84722
5.75229 0.00963 0.032 0.00663 0.000255 0.00304 0.000463 0.00437 0.00632 0.00213
2L:6921580-6921730:plus -13.2959 -2.72477 -1.99083 11.2936 -8.63265 -17.9388 -16.3945 -7.44444 -3.78899
0.000775 0.0239 0.0216 0.0000139 0.0013 0.00738 0.0116 0.101 0.0526
2L:6922000-6922150:minus -23.2551 1.44037 0.238532 1.86239 -18.4898 -27.5612 -9.58716 -2.09722 7.58716
0.00583 0.00617 0.0107 0.0153 0.0116 0.0393 0.00285 0.0306 0.000954
2L:6922000-6922150:plus -31.5204 5.25688 5.22018 1.10092 -8.86735 -17.051 -24.8624 1.98611 2.36697
0.0131 0.00146 0.00162 0.02 0.00173 0.00479 0.0463 0.00866 0.00828
2L:6945240-6945390:minus -31.449 1.62385 8.05505 5.93578 -17.9796 -8.5 -24.3486 2.26389 -0.238532
0.0123 0.00577 0.000425 0.00264 0.00726 0.00126 0.0432 0.00784 0.0203
2L:6945240-6945390:plus -33.5612 -2.49541 -0.0458716 5.30275 -36.0102 -8.65306 -29.2018 1.29167
-6.48624 0.0288 0.0224 0.0118 0.00396 0.072 0.00156 0.0812 0.011 0.1
2L:6948720-6948870:minus -3.22449 -1.21101 0.165138 5.85321 -27.1939 -17.2755 -4.66972 5.18056 -4.10092
0.0000495 0.0153 0.011 0.00287 0.0267 0.00544 0.000903 0.00242 0.057
2L:6948720-6948870:plus -4.92857 2.45872 -0.00917431 9.7156 -17.7551 -9.53061 -24.8991 -0.625
1.48624 0.000212 0.00424 0.0116 0.000242 0.00658 0.0031 0.0465 0.0202 0.0118
2L:6950020-6950170:minus -33.5612 13.5963 9.44954 0.752294 -27.2653 -26.8571 -14.1468 4.75 3.42202
0.0288 0.0000131 0.000196 0.0221 0.0302 0.0288 0.00749 0.00293 0.00597
2L:6950020-6950170:plus -30.4796 14.0275 9.06422 7.21101 -8.64286 -9.42857 -25.0642 6.34722 -4.61468
0.00941 8.23e-06 0.000245 0.0011 0.00139 0.0027 0.0476 0.0014 0.0658
2L:6950400-6950550:minus -31.5918 13.5963 9.44954 6.84404 -27.2653 -17.6429 -14.1468 4.75 3.42202
0.0136 0.0000131 0.000196 0.0014 0.0302 0.00619 0.00749 0.00293 0.00597
2L:6950400-6950550:plus -22.898 8.6422 1.33945 7.44037 -8.20408 -9.38776 -6.07339 -1.58333 -3.11009

0.00442 0.00027 0.00733 0.000912 0.000894 0.00266 0.00134 0.0266 0.0459
2L:6956880-6957030:minus -32.9592 14.6055 -0.0458716 0.522936 -26.1939 -26.5612 -8.25688 2.93056
2.51376 0.0252 4.26e-06 0.0118 0.0234 0.0175 0.0237 0.00222 0.00612 0.00792
2L:6956880-6957030:plus -23.2959 15.3394 12.9725 9.33028 -27.1939 -9.42857 -3.92661 -0.763889
-0.605505 0.00605 1.34e-06 0.0000215 0.000374 0.0267 0.0027 0.000731 0.0211 0.023
2L:6960300-6960450:minus -32.4694 10.156 3.75229 1.91743 -27 -18.0102 -15.6147 0.152778 1.83486
0.0196 0.000124 0.00296 0.015 0.0233 0.00812 0.0101 0.016 0.0101
2L:6960300-6960450:plus -23.3776 9.54128 14.1651 7.29358 -28.2347 -17.2755 -15.6514 0.208333 5.22936
0.00619 0.000168 6.03e-06 0.00104 0.051 0.00544 0.0102 0.0157 0.00288
2L:6964060-6964210:minus -21.6633 3.34862 2.99083 4.19266 1.43878 -18.7857 -15.2385 0.291667 4.10092
0.00223 0.00306 0.00399 0.00536 0.0000769 0.0146 0.00941 0.0153 0.00413
2L:6964060-6964210:plus -32.5102 3.62385 -4.08257 4.37615 10.9184 19.3469 -25.3028 3.33333 1.2844
0.0202 0.00276 0.0388 0.00504 4.19e-06 5.04e-07 0.049 0.00525 0.0129
2L:6966420-6966570:minus -40.7449 -1.66055 2.52294 9.3211 -17.7551 -18.0816 -21.2385 0.930556 -0.449541
0.0431 0.0176 0.00476 0.000391 0.00658 0.00934 0.0257 0.0124 0.0216
2L:6966420-6966570:plus -13.4796 -4.15596 1.59633 6.18349 -7.71429 -17.7857 -16.9266 1.09722 9.55046
0.000935 0.0353 0.00669 0.00217 0.000572 0.0068 0.0127 0.0118 0.000321
2L:6967600-6967750:minus -23 2.62385 4.3211 5.46789 -28.1939 -8.45918 -13.9725 1.34722 1.57798
0.00498 0.00399 0.00235 0.00362 0.0482 0.00111 0.00721 0.0108 0.0114
2L:6967600-6967750:plus -21.9592 13.4592 -4.42202 6.3578 -7.60204 -9.30612 -20.0826 -0.291667
-3.99083 0.00279 0.000064 0.0425 0.00195 0.000466 0.00257 0.0209 0.0183 0.0556
2L:6968100-6968250:minus -23.2143 0.504587 -1.01835 5.49541 0.612245 -18.5714 -12.3761 -1.77778 -6.50459
0.00559 0.00865 0.0161 0.00355 0.000269 0.0136 0.00501 0.0281 0.101
2L:6968100-6968250:plus -4.67347 4.02752 13.4679 7.29358 -18.5306 -17.051 -9.91743 3.125 2.12844
0.000182 0.00238 0.0000134 0.00104 0.0118 0.00479 0.00303 0.00569 0.00886
2L:6971720-6971870:minus -21.4388 -0.183486 1.30275 7.34862 2.87755 -8.53061 -2.10092 2.43056
3.10092 0.002 0.011 0.00743 0.000998 0.0000259 0.00136 0.000455 0.00738 0.00674
2L:6971720-6971870:plus -41.2959 0.220183 0.926606 2.00917 -27.3061 -18.3469 -22.7615 9.93056 3.08257
0.0529 0.00956 0.00848 0.0142 0.0315 0.0124 0.0337 0.000172 0.00681
2L:7013260-7013410:minus -40.2551 -2.23853 -0.743119 -0.990826 -18.6837 -8.79592 -17.4128 0.972222
0.146789 0.0382 0.0208 0.0147 0.0366 0.0126 0.0019 0.0137 0.0123 0.0178
2L:7013260-7013410:plus -23.5306 3.66055 15.4495 5.07339 -8.89796 -18.1224 -6.90826 1.70833 5.6055
0.00646 0.00273 9.17e-07 0.0043 0.00189 0.00988 0.00165 0.00955 0.00232
2L:7023900-7024050:minus 15.8469 5.6422 0.66055 2.78899 -18.449 -18.2347 -0.0366972 3.93056
7.84404 1.84e-07 0.00124 0.00929 0.00992 0.0107 0.0104 0.000286 0.00413 0.000701
2L:7023900-7024050:plus -19.6633 -2.55963 -1.46789 1.97248 -18.4184 -9.7551 -18.4862 3.80556 7.73394
0.00164 0.0228 0.0184 0.0146 0.0101 0.00358 0.0161 0.00435 0.000784
2L:7024400-7024550:minus -31.7041 -1.87156 11.2569 -0.366972 -28.2653 -16.2755 -18.1927 6.15278
2.85321 0.0146 0.0187 0.0000682 0.0298 0.0537 0.00413 0.0154 0.00154 0.00733
2L:7024400-7024550:plus -32.8061 -1.3211 1.25688 -0.568807 -19.051 -27.1939 -21.6055 1.51389
-2.7156 0.0237 0.0159 0.00755 0.032 0.0162 0.0327 0.0275 0.0102 0.0412
2L:7027100-7027250:minus -30.6735 -1.21101 5.30275 1.92661 -17.5306 -7.72449 -8.55046 -2.13889 5.34862
0.00978 0.0153 0.00157 0.0148 0.0054 0.00068 0.00235 0.0309 0.00271
2L:7027100-7027250:plus -39.9898 1.42202 2.22018 3.6055 -7.86735 -27.602 -15.8532 -2.66667 -0.761468
0.0351 0.00621 0.00532 0.00686 0.000617 0.0416 0.0106 0.0355 0.0244
2L:7027460-7027610:minus -22.0408 6.94495 1.38532 2.25688 -8.37755 -27.1224 -15.3119 4.13889 3.15596
0.00296 0.000669 0.00721 0.0127 0.00106 0.0325 0.00954 0.0038 0.00659
2L:7027460-7027610:plus -33.0714 -3.17431 14.8624 7.29358 -8.53061 -8.5 -22.1927 -4.20833 -3.82569
0.0267 0.027 2.2e-06 0.00104 0.0011 0.00126 0.0305 0.0517 0.0531
2L:7032580-7032730:minus -24.4184 6.66055 0.311927 2.9633 -26.2653 -8.79592 -18.8257 0.680556 -0.229358
0.00797 0.000771 0.0104 0.00914 0.018 0.0019 0.017 0.0135 0.0202
2L:7032580-7032730:plus -23.4898 13.9541 0.229358 3.77064 -8.63265 -9.53061 -6.42202 -0.25 0.908257
0.00639 8.97e-06 0.0107 0.00637 0.0013 0.0031 0.00146 0.0181 0.0147
2L:7033100-7033250:minus -22.8061 1.58716 -5.3578 2.87156 -17.4898 -19.0102 -16.6697 -2.33333 -4.0367

0.00414 0.00584 0.054 0.00948 0.0053 0.0159 0.0122 0.0326 0.0562
2L:7033100-7033250:plus -32 13.4592 0.788991 4.33028 0.357143 0.0204082 -15.4587 4.47222 0.917431
0.0171 0.000064 0.00889 0.00511 0.000324 0.000377 0.00982 0.0033 0.0146
2L:7038420-7038570:minus -30.4388 -6.16514 7.27523 7.37615 -17.9388 -9.82653 -19.8991 7.47222 4.21101
0.00923 0.0595 0.000635 0.000951 0.00715 0.00381 0.0202 0.000783 0.00396
2L:7038420-7038570:plus -41.551 4.29358 -0.302752 -0.880734 -18.449 -27.8265 -17.4404 11.9028
1.74312 0.0607 0.00215 0.0128 0.0353 0.0107 0.0498 0.0137 0.000034 0.0105
2L:7042260-7042410:minus -2.36735 -3.50459 12.3394 1.26606 -8.67347 -26.3776 -15.6239 4.875 3.07339
0.0000302 0.0296 0.0000338 0.0191 0.00154 0.0222 0.0101 0.00277 0.00683
2L:7042260-7042410:plus -11.7755 7.65138 -1.84404 5.30275 -8.64286 -8.65306 -12.1468 3.09722 8.05505
0.000266 0.00046 0.0206 0.00396 0.00139 0.00156 0.00476 0.00575 0.00064
2L:7049260-7049410:minus -21.7755 -0.715596 2.75229 2.27523 -27.2653 -27.4082 -18.1009 5.43056
11.3853 0.00241 0.0131 0.00436 0.0125 0.0302 0.0356 0.0152 0.00216 0.0000815
2L:7049260-7049410:plus -24.0408 1.95413 1.0367 2.62385 -27.3367 -18.3469 -0.247706 -1.08333 1
0.00726 0.0051 0.00816 0.0108 0.0317 0.0124 0.0003 0.0231 0.0143
2L:7049960-7050110:minus -31.9184 -0.46789 7.7156 3.89908 -7.63265 -27.5306 -13.578 0.722222 4.45872
0.0164 0.0121 0.000509 0.00604 0.000479 0.0374 0.0066 0.0133 0.00366
2L:7049960-7050110:plus -24.2245 0.53211 2.44037 5.57798 -8.86735 -9.72449 -14.2477 1.47222 12.2752
0.00763 0.00856 0.00491 0.0034 0.00173 0.00352 0.00766 0.0104 0.0000141
2L:7050500-7050650:minus -31.6633 2.24771 -3.37615 2.33945 -17.9388 -8.38776 -20.3211 1.70833 -7.6055
0.0141 0.00458 0.032 0.0122 0.00715 0.00101 0.0218 0.00955 0.126
2L:7050500-7050650:plus -31.3265 9.22936 6.51376 2.50459 -18.4898 -27.051 -6.27523 0.0555556
9.77064 0.0114 0.000199 0.000912 0.0114 0.0116 0.0315 0.00141 0.0164 0.000228
2L:7056200-7056350:minus -13.1122 4.51376 0.348624 2.3945 -26.1531 9.60204 -12.8624 6.125 -0.550459
0.000632 0.00198 0.0103 0.0119 0.0172 0.0000248 0.0056 0.00156 0.0225
2L:7056200-7056350:plus -24.2551 -1.34862 -0.174312 2.07339 10.6531 -9.0102 -10.3578 -1.19444
1.63303 0.00768 0.016 0.0123 0.0138 0.0000102 0.00208 0.00329 0.0239 0.0112
2L:7065820-7065970:minus -21.8163 -0.137615 1.79817 6.17431 0.540816 9.53061 -10.3303 1.94444
-1.3945 0.00244 0.0108 0.00623 0.0022 0.000321 0.0000316 0.00328 0.00879 0.0288
2L:7065820-7065970:plus -23.9898 -2.6789 6.24771 3.34862 -17.6735 -8.16327 -20.6972 0.902778 -5.22936
0.00711 0.0236 0.00103 0.0077 0.00572 0.000783 0.0233 0.0125 0.0764
2L:7080440-7080590:minus -31.102 2.01835 -4.02752 5.43119 -17.3367 -8.86735 -20.1835 2.22222 1.94495
0.0106 0.00499 0.0383 0.00371 0.00391 0.00198 0.0213 0.00796 0.00968
2L:7080440-7080590:plus -31.4796 0.110092 3.24771 3.08257 -18.7551 -18.3061 -16.0459 12.7639 -0.12844
0.0128 0.00993 0.00361 0.0087 0.0144 0.0114 0.0109 0.0000136 0.0193
2L:7083740-7083890:minus -13.1837 3.68367 -3.07339 0.981651 -18.4184 -0.469388 1.95413 2.56944
7.16514 0.000705 0.00144 0.0294 0.0207 0.0101 0.000533 0.000167 0.00701 0.00122
2L:7083740-7083890:plus -30.7041 -1.57798 -1.6789 1.16514 -17.6429 -8.87755 -23.0642 -0.736111
2.20183 0.00993 0.0171 0.0196 0.0197 0.00562 0.00198 0.0355 0.0209 0.00869
2L:7084120-7084270:minus -24.1837 -0.119266 7.11927 7.55963 -17.9796 -18.3469 -14.7156 5.98611
1.27523 0.00751 0.0107 0.000686 0.000807 0.00726 0.0124 0.00847 0.00167 0.013
2L:7084120-7084270:plus -31.9592 3.97959 4.41284 7.6055 -9.16327 -17.9388 -27.7064 3.27778 -4.6422
0.0167 0.000669 0.00227 0.000794 0.00249 0.00738 0.0662 0.00536 0.0662
2L:7084620-7084770:minus 14.8469 1.11009 8.54128 7.78899 -17.1531 -26.5612 12.5963 -0.388889
-0.0825688 7.98e-07 0.00695 0.000326 0.000642 0.00364 0.0237 4.22e-06 0.0188 0.0189
2L:7084620-7084770:plus -33.1122 2.18349 5.75229 1.36697 -18.9796 -8.72449 -22.1009 6.31944 -8.0367
0.0271 0.00469 0.00129 0.0186 0.0155 0.00169 0.03 0.00142 0.136
2L:7086340-7086490:minus -31.4388 2.20183 -2.05505 5.55046 -18.4898 -26.3061 -7.34862 2.22222 2.36697
0.0122 0.00466 0.022 0.00345 0.0116 0.0219 0.00183 0.00796 0.00828
2L:7086340-7086490:plus -33.8469 -0.706422 -1.14679 4.6055 -8.67347 -18.3061 -14.8349 10.1806
-0.642202 0.03 0.0131 0.0167 0.00476 0.00154 0.0114 0.00868 0.000144 0.0234
2L:7086980-7087130:minus -32.9694 -0.238532 -2.50459 1.10092 -9.23469 1.09184 -25.6147 2.13889
3.17431 0.0254 0.0112 0.025 0.02 0.00277 0.000091 0.051 0.0082 0.00653
2L:7086980-7087130:plus -21.9286 -2.85321 0.59633 4.91743 -17.7551 -9.30612 -7.74312 2.65278 1.66972

0.0027 0.0248 0.00949 0.00447 0.00658 0.00257 0.00199 0.0068 0.011
2L:7087580-7087730:minus -31.6327 5.0367 1.13761 2.88991 -17.2245 -8.45918 -26.2844 -0.722222
1.07339 0.0138 0.0016 0.00788 0.00942 0.00384 0.00111 0.0554 0.0208 0.014
2L:7087580-7087730:plus -22.5102 2.11927 1.7156 3.61468 -27.3061 -17.2755 -13.7706 2.02778 6.26606
0.00354 0.00481 0.00642 0.0068 0.0315 0.00544 0.00689 0.00853 0.00162
2L:7089680-7089830:minus -2.85714 -1.44037 3.04587 9.59633 -18.2245 -19.0102 -3.90826 -2.18056 0.247706
0.0000388 0.0164 0.0039 0.000275 0.00877 0.0159 0.000727 0.0313 0.0172
2L:7089680-7089830:plus -29.9184 3.84404 2.31193 9.51376 -17.1531 -17.7143 -12.2202 -5.625 -4.77982
0.00854 0.00255 0.00515 0.000292 0.00364 0.00634 0.00484 0.0705 0.069
2L:7091580-7091730:minus -23.3673 6.46789 -1.17431 2.04587 -17.8571 -8.23469 -15.4679 0.638889 0.981651
0.00617 0.000847 0.0169 0.014 0.00698 0.000875 0.00983 0.0137 0.0144
2L:7091580-7091730:plus -31.6224 1.11927 2.13761 6.12844 -18.7551 -9.37755 -9.21101 8 1.27523
0.0137 0.00693 0.00549 0.00231 0.0144 0.00265 0.00266 0.000583 0.013
2L:7136800-7136950:minus -40.449 0.981651 -0.899083 5.93578 -27.1939 1.82653 -19.5872 1.875
5.49541 0.0402 0.00729 0.0155 0.00264 0.0267 0.0000498 0.0192 0.00901 0.0025
2L:7136800-7136950:plus -22.1122 2.80734 10.211 5.65138 -17.7143 -8.72449 -15 -2.08333 5.6055
0.00304 0.00373 0.000126 0.00322 0.00627 0.00169 0.00897 0.0305 0.00232
2L:7150980-7151130:minus -32 1.38532 2.3211 1.33028 -27.4898 -8.02041 -8.54128 0.458333 -4.75229
0.0171 0.00629 0.00513 0.0188 0.0336 0.000761 0.00234 0.0145 0.0683
2L:7150980-7151130:plus -34.1837 -5.76147 0.385321 0.46789 -26.7041 -27.6327 -15.4954 3.69444 3.09174
0.0314 0.0538 0.0102 0.0239 0.0193 0.043 0.00989 0.00455 0.00678
2L:7155300-7155450:minus -23.4796 7.74312 4.55046 2.31193 -7.71429 -19.1224 -13.8807 3.65278 7.6422
0.00634 0.000438 0.00214 0.0123 0.000572 0.0175 0.00706 0.00462 0.000893
2L:7155300-7155450:plus -4.59184 1.82569 4.05505 4.74312 -18.3878 -9.30612 -11.6881 1.45833 9.75229
0.000166 0.00535 0.00262 0.00462 0.00931 0.00257 0.00431 0.0104 0.000241
2L:7157560-7157710:minus -12.8878 3.61224 2.83486 11.0183 -18.1939 -9.23469 -13.2477 -0.736111
-3.14679 0.000523 0.00167 0.00423 0.000052 0.0086 0.00238 0.00612 0.0209 0.0463
2L:7157560-7157710:plus -31.1429 3.71429 5.12844 11.2936 -8.40816 -17.0102 -9.7156 4.43056 7.22018
0.0108 0.00129 0.00169 0.0000139 0.00108 0.00453 0.00292 0.00336 0.00119
2L:71600-71750:minus -40.4082 -3.21101 6.6422 5.57798 -27.2653 -9.68367 -17.8165 4.29167 -3.52294
0.0399 0.0273 0.000858 0.0034 0.0302 0.00342 0.0145 0.00356 0.0499
2L:71600-71750:plus -21.6633 0.53211 1.07339 9.33028 -27.0102 -27.5306 -16.0092 3.73611 1.01835
0.00223 0.00856 0.00806 0.000374 0.0237 0.0374 0.0109 0.00447 0.0142
2L:7182040-7182190:minus -23.4082 -0.844037 14.422 7.29358 -17.9796 9.60204 -28.5138 1.54167
-11.1193 0.00623 0.0136 4.18e-06 0.00104 0.00726 0.0000248 0.0738 0.0101 0.235
2L:7182040-7182190:plus -31.5102 7.41284 0.981651 1.9633 0.806122 0.27551 -17.7982 8.58333 0.348624
0.013 0.000521 0.00832 0.0146 0.000229 0.000257 0.0145 0.000415 0.0167
2L:7188120-7188270:minus -32.7755 -0.394495 -0.706422 5.73394 -18.1837 -18.0102 -6.34862 -4.36111
5.12844 0.0234 0.0118 0.0146 0.00307 0.00835 0.00812 0.00144 0.0535 0.00301
2L:7188120-7188270:plus -4.21429 -1.30275 1.3578 1.92661 -28.2653 -8.72449 -4.89908 -1.79167 7.84404
0.000129 0.0158 0.00728 0.0148 0.0537 0.00169 0.000965 0.0282 0.000701
2L:7204200-7204350:minus -24 7.22936 3.77064 5.37615 -27.3061 -8.72449 -13.156 1.15278 2.74312
0.00719 0.000576 0.00294 0.00384 0.0315 0.00169 0.00599 0.0115 0.00751
2L:7204200-7204350:plus -30.551 13.4592 0.513761 5.65138 -18.6735 -19.0102 -13.1835 4.83333 -3.74312
0.00959 0.000064 0.00977 0.00322 0.0123 0.0159 0.00603 0.00283 0.0521
2L:7207800-7207950:minus -31.8571 -2.33028 8.91743 3.9633 -18.7857 -18.7857 -14.1468 -4.34722 1.01835
0.0163 0.0214 0.000266 0.0059 0.0148 0.0146 0.00749 0.0533 0.0142
2L:7207800-7207950:plus -20.7347 -1.75229 2.54128 2.06422 -7.71429 -8.5 -13.5413 5.44444 2.47706
0.00176 0.0181 0.00473 0.0139 0.000572 0.00126 0.00654 0.00215 0.00801
2L:7213140-7213290:minus -34.1122 3.64286 0.577982 5.3945 -7.93878 -8.7551 -20.789 5.04167 1.30275
0.0312 0.00159 0.00955 0.00379 0.000767 0.00172 0.0237 0.00258 0.0128
2L:7213140-7213290:plus -20.7755 2.43119 2.51376 5.93578 -25.9286 -26.2653 -12.4954 3.91667
-0.0825688 0.00176 0.00428 0.00478 0.00264 0.0168 0.0218 0.00515 0.00416 0.0189
2L:7213720-7213870:minus -3.07143 -3.19266 1.47706 2.82569 -16.7143 -9.02041 -13.0183 2.68056 5.65138

0.0000441 0.0272 0.00698 0.00976 0.00319 0.00213 0.00581 0.00673 0.00228
2L:7213720-7213870:plus -23.3776 1.81651 2.68807 3.41284 -17.7143 -17.9796 -8.26606 -0.736111
1.30275 0.00619 0.00537 0.00447 0.00756 0.00627 0.00764 0.00222 0.0209 0.0128
2L:7217940-7218090:minus -40.7755 -1.06422 -0.155963 11.2936 -17.9796 0.979592 -26.4404 6.30556
-4.86239 0.0433 0.0146 0.0122 0.0000139 0.00726 0.000136 0.0565 0.00143 0.0705
2L:7217940-7218090:plus -32.7755 7.88991 1.95413 3.6055 -18.4898 -27.5612 -21.9817 4.68056 -0.623853
0.0234 0.000405 0.00588 0.00686 0.0116 0.0393 0.0294 0.00302 0.0232
2L:7219420-7219570:minus -31.4694 1.31193 2.17431 3.23853 -17.7143 -25.9796 -6.47706 0.930556 -2.25688
0.0124 0.00646 0.00542 0.00815 0.00627 0.0214 0.00148 0.0124 0.0357
2L:7219420-7219570:plus -39.4082 2.44037 -6.89908 2.33945 -17.3061 -8.38776 -31.3303 -1.25 -6.29358
0.0332 0.00427 0.0783 0.0122 0.00389 0.00101 0.109 0.0242 0.0955
2L:7220020-7220170:minus -2.30612 3.47706 -1.02752 4.23853 10.0918 -8.42857 -21.6972 0.180556 -4.70642
0.0000281 0.00292 0.0161 0.00522 0.0000251 0.00104 0.0279 0.0158 0.0676
2L:7220020-7220170:plus -14.4388 5.74312 -1.52294 4.22936 -26.898 -16.7857 -14.0183 6.56944 3.72477
0.00137 0.00119 0.0187 0.00525 0.0203 0.00426 0.00728 0.00125 0.00497
2L:7222980-7223130:minus -14.0816 14.1743 -0.146789 6.37615 -26.9694 -18.0102 -9.12844 -0.583333
7.36697 0.00117 6.8e-06 0.0122 0.00192 0.023 0.00812 0.00262 0.02 0.00108
2L:7222980-7223130:plus -33.602 1.91743 -0.642202 0.899083 -19.0918 -18.051 -18.2661 3.06944
-0.440367 0.0289 0.00518 0.0143 0.0212 0.0163 0.00886 0.0156 0.00581 0.0216
2L:72380-72530:minus -30.7041 -1.55963 7.05505 2.70642 -18.4184 -16.051 -9.55046 4.77778 5.00917
0.00993 0.017 0.000708 0.0103 0.0101 0.00408 0.00283 0.00289 0.0031
2L:72380-72530:plus -20.7755 5.58716 -0.449541 7.33945 -8.67347 -17.8571 -13.422 1.19444 -2.06422
0.00176 0.00127 0.0134 0.00102 0.00154 0.00719 0.00637 0.0114 0.0339
2L:7240420-7240570:minus -13.5204 1.62385 4.89908 9.59633 -18.6837 -17.8571 -18.156 7.34722 6
0.00097 0.00577 0.00185 0.000275 0.0126 0.00719 0.0153 0.000838 0.00184
2L:7240420-7240570:plus -20.5918 1.22018 6.29358 2.19266 -18.1939 -18.1224 -25.2844 3.375 2.56881
0.00169 0.00668 0.00101 0.0131 0.0086 0.00988 0.0489 0.00516 0.00783
2L:7296280-7296430:minus -32.2857 2.21101 -1.65138 0.220183 -8.93878 -26.3776 -1.36697 -0.0972222
3.66972 0.0185 0.00464 0.0195 0.0253 0.00216 0.0222 0.000385 0.0172 0.00514
2L:7296280-7296430:plus -30.9694 0.642202 2.42202 5.57798 -7.64286 -26.5306 -12.9817 -1.15278 1.82569
0.0104 0.00823 0.00495 0.0034 0.000499 0.023 0.00576 0.0236 0.0101
2L:7306920-7307070:minus -23.8163 -1.6055 -1.46789 1.91743 -17.7551 -9.30612 -23.9633 7.51389 -0.633028
0.00682 0.0173 0.0184 0.015 0.00658 0.00257 0.0408 0.000766 0.0232
2L:7306920-7307070:plus -13.1122 -1.24771 4.89908 9.08257 -8.60204 -25.4898 -12.5046 -2.73611 3.88073
0.000632 0.0155 0.00185 0.000444 0.00125 0.0205 0.00516 0.0362 0.00475
2L:73200-73350:minus -22.8061 1.2844 6.55046 -0.642202 -18.1531 -36.1531 -9.15596 10.0972 3.56881
0.00414 0.00653 0.000896 0.0328 0.00829 0.0908 0.00263 0.000153 0.00543
2L:73200-73350:plus -31.7449 0.40367 9.9633 6.42202 -27.2347 -8.5 -7.06422 7.22222 5.59633 0.0152
0.00896 0.000145 0.00188 0.0283 0.00126 0.00171 0.000895 0.00234
2L:7324980-7325130:minus -32.3367 3.97959 1.3945 6.09174 -8.93878 -17.5714 -32.3303 4.25 -4.79817
0.0187 0.000669 0.00719 0.00235 0.00216 0.00616 0.123 0.00362 0.0693
2L:7324980-7325130:plus -30.6735 -3.04587 6.44037 6.29358 -18.1939 -8.65306 -24.156 1.13889 -6.43119
0.00978 0.0261 0.000945 0.00202 0.0086 0.00156 0.042 0.0116 0.0988
2L:73880-74030:minus -32.4796 6.84404 1.81651 5.86239 -9.23469 -18.5714 -13.4037 4.09722 3.45872 0.02
0.000705 0.00619 0.00282 0.00277 0.0136 0.00634 0.00386 0.00591
2L:73880-74030:plus -32.7755 -0.247706 3.15596 6.12844 -17.8265 -18.9796 -12.9725 2.15278 6.22018
0.0234 0.0112 0.00374 0.00231 0.00691 0.0155 0.00574 0.00816 0.00165
2L:7388180-7388330:minus -23.9184 1.46789 5.72477 5.49541 -26.9286 -8.42857 -12.3394 5.95833 -6.68807
0.00695 0.0061 0.0013 0.00355 0.0214 0.00104 0.00497 0.00169 0.106
2L:7388180-7388330:plus -39.5918 0.825688 -1.37615 1.59633 -18.449 0.316327 -13.4954 2.33333 3.45872
0.0335 0.00771 0.0179 0.017 0.0107 0.000243 0.00648 0.00765 0.00591
2L:7410100-7410250:minus -33.3367 -2.89908 -2.93578 0.990826 -17.7143 -9.5 -23.5138 -0.694444 -4.55046
0.0281 0.0251 0.0283 0.0206 0.00627 0.00294 0.0381 0.0206 0.0646
2L:7410100-7410250:plus -21.8878 -1.34862 1.47706 5.43119 -8.97959 -19.3469 -22.156 2.16667 3.46789

0.00258	0.016	0.00698	0.00371	0.0023	0.0194	0.0303	0.00812	0.00582
2L:7412280-7412430:minus	-33.3673	2.26606	4.65138	1.56881	-17.5612	-9.64286	-22.6422	2.70833	-3.97248
0.0282	0.00455	0.00206	0.0172	0.00544	0.00339	0.033	0.00666	0.0553
2L:7412280-7412430:plus	-12.3367	-1.6422	-0.66055	1.91743	0.877551	-17.051	-14.8899	-3.40278	-0.394495
0.000393	0.0175	0.0143	0.015	0.00019	0.00479	0.00877	0.0427	0.0212
2L:7422440-7422590:minus	-41.2143	1.84404	3.12844	-0.394495	-9.5	-8.7551	-26.4495	1.13889	-2.53211
0.0494	0.00532	0.00378	0.0301	0.00293	0.00172	0.0565	0.0116	0.0392
2L:7422440-7422590:plus	-21.7755	-0.504587	-0.477064	11.0183	-17.449	-27.8265	-7.11927	-0.486111
7.27523	0.00241	0.0122	0.0135	0.000052	0.00484	0.0498	0.00174	0.0194	0.00113
2L:7423720-7423870:minus	-14.0306	1.21101	5.86239	0.293578	-17.5306	-27.4898	-2.94495	-1.44444	5.38532
0.00115	0.00671	0.00122	0.0248	0.0054	0.0363	0.00056	0.0256	0.00267
2L:7423720-7423870:plus	-22.1122	-3	3.14679	3.76147	-17.2653	-18.7143	-3.38532	8.25	6.12844	0.00304
0.0258	0.00375	0.00645	0.00387	0.014	0.000629	0.000505	0.00174
2L:7424580-7424730:minus	-12.7041	6.22018	2.62385	3.79817	-27.0816	-8.61224	-1.15596	2.88889	-4.76147
0.000464	0.000954	0.00458	0.00629	0.0243	0.00152	0.000367	0.00622	0.0686
2L:7424580-7424730:plus	-22.6633	-0.779817	9.14679	2.11927	-17.9388	-9.45918	-19.0917	9.91667
-1.27523	0.00384	0.0134	0.000233	0.0135	0.00715	0.00279	0.0177	0.000174	0.0282
2L:7425020-7425170:minus	-33	-1.58716	8.23853	7.55963	-8.96939	-8.27551	-21.4954	4.55556	-2.26606
0.0258	0.0172	0.000386	0.000807	0.00226	0.000919	0.0269	0.00318	0.0359
2L:7425020-7425170:plus	-30.1429	1.26606	0.908257	1.33028	-26.9694	-19.4184	11.9541	0.944444	7.68807
0.00866	0.00657	0.00853	0.0188	0.023	0.0198	5.75e-06	0.0124	0.000841
2L:7426960-7427110:minus	-23.1122	4.06422	3.37615	7.27523	-28.2347	-8.65306	-17.633	5.47222	-2.22018
0.0053	0.00235	0.00343	0.00109	0.051	0.00156	0.0141	0.00212	0.0355
2L:7426960-7427110:plus	-30.6327	4.18349	-0.366972	2.21101	10.6939	0.755102	-19.3578	-2.31944
-4.75229	0.00968	0.00224	0.0131	0.0129	7.35e-06	0.000175	0.0185	0.0325	0.0683
2L:7427560-7427710:minus	-23.9286	-0.00917431	-2.22018	-1.26606	-18.4898	-8.5	-3.14679	3.29167	3.23853
0.00701	0.0103	0.0231	0.04	0.0116	0.00126	0.00059	0.00533	0.00639
2L:7427560-7427710:plus	-30.5204	1.3578	4.49541	1.74312	-6.60204	0.0918367	-29.5872	0.819444
2.20183	0.00951	0.00635	0.00219	0.016	0.000355	0.000323	0.0858	0.0129	0.00869
2L:7433520-7433670:minus	-21.6939	-0.0366972	5.93578	10.8624	-27.2245	-19.0816	-4.38532	-1.26389
2.33028	0.00226	0.0104	0.00119	0.0000687	0.0272	0.017	0.000832	0.0243	0.00836
2L:7433520-7433670:plus	-23.0714	3.3211	3.73394	7.80734	-17.3776	-10.0918	-12.1835	6.94444	1.83486
0.0052	0.00309	0.00299	0.00063	0.00419	0.00404	0.0048	0.00103	0.0101
2L:7437220-7437370:minus	-34	3.71429	-3.22936	2.6789	-28.2653	-8.94898	-22.3028	7.63889	-3.42202
0.0307	0.00129	0.0307	0.0105	0.0537	0.00203	0.0311	0.000715	0.0488
2L:7437220-7437370:plus	-12.1531	-1.3211	3.77064	6.44037	-27.1939	-18.0102	-17.4128	0.0555556
9.87156	0.000352	0.0159	0.00294	0.00185	0.0267	0.00812	0.0137	0.0164	0.000217
2L:7473320-7473470:minus	-32.7041	1.2844	3.22936	4.22018	-18.4592	-8.42857	-23.3853	2.06944	-4.10092
0.0222	0.00653	0.00363	0.00531	0.0109	0.00104	0.0374	0.00841	0.057
2L:7473320-7473470:plus	-23.0714	1.37615	-2.11927	1.84404	-9.20408	-27.4898	-12.6789	-1.16667	8.41284
0.0052	0.00631	0.0224	0.0154	0.00262	0.0363	0.00537	0.0237	0.000519
2L:7486440-7486590:minus	-34.4898	-1.18349	-4.61468	2.0367	-16.7551	0.897959	-22.4404	4.84722	6.7156
0.0319	0.0152	0.0447	0.0141	0.00322	0.000144	0.0319	0.00281	0.00144
2L:7486440-7486590:plus	-31.7041	-1.11009	4.53211	5.65138	-9.20408	-18.1224	-19.0367	5.29167	4.83486
0.0146	0.0148	0.00216	0.00322	0.00262	0.00988	0.0176	0.0023	0.00329
2L:7495500-7495650:minus	-21.0408	-3.22018	11.3394	7.45872	-18.4592	-8.5	-23.1376	5.91667	11.3853
0.00186	0.0274	0.000065	0.000894	0.0109	0.00126	0.0359	0.00172	0.0000815
2L:7495500-7495650:plus	-23.0714	-0.0275229	4.86239	2.51376	-17.7857	-26.6735	-7.10092	8.59722
-0.788991	0.0052	0.0104	0.00188	0.0113	0.00676	0.0256	0.00173	0.000411	0.0245
2L:7496340-7496490:minus	-14.3367	-0.40367	-1.34862	3.93578	-26.898	-9.0102	-10.0092	1.01389	-0.137615
0.00131	0.0118	0.0178	0.00597	0.0203	0.00208	0.00308	0.0121	0.0194
2L:7496340-7496490:plus	-32.4694	-1.2844	-0.238532	2.52294	-27.602	-27.7041	-15.2018	1.80556
1.52294	0.0196	0.0157	0.0125	0.0113	0.0364	0.0445	0.00934	0.00923	0.0117
2L:7570660-7570950:minus	-22.8878	-1.19266	2.07339	2.82569	-18.6837	-18.5714	-20.1927	5.27778

-0.0825688 0.00441 0.0152 0.00562 0.00976 0.0126 0.0136 0.0213 0.00232 0.0189
2L:7570660-7570950:plus -22.7755 1.31193 4.29358 6.16514 -18.1122 -17.7551 -18.8165 -0.708333
-0.862385 0.00412 0.00646 0.00238 0.00223 0.00771 0.00643 0.017 0.0207 0.0251
2L:7573660-7573810:minus -31.5102 4.34862 3.13761 6.12844 -27.2347 -28.4796 -9.25688 4.29167 7.63303
0.013 0.0021 0.00377 0.00231 0.0283 0.0617 0.00268 0.00356 0.000928
2L:7573660-7573810:plus -31.3265 -0.348624 13.5872 4.44954 -18.5306 -8.5 -10.7339 -1.47222 3.33028
0.0114 0.0116 0.0000122 0.00492 0.0118 0.00126 0.00354 0.0258 0.00612
2L:7576540-7576690:minus -13.9592 3.97959 6.16514 3.6055 -26.0408 -26.898 -21.4495 -3.25 1.93578
0.00113 0.000669 0.00107 0.00686 0.0171 0.0302 0.0267 0.0411 0.00969
2L:7576540-7576690:plus -29.8776 1.62385 2.56881 5.33028 -7.64286 -36.4082 -6.55963 -1.01389 3.66972
0.00852 0.00577 0.00468 0.00389 0.000499 0.105 0.00152 0.0227 0.00514
2L:7675680-7675830:minus -40.2959 2.40367 3.55046 5.49541 -18.7551 -18.051 -20.2661 5.59722 5.70642
0.0389 0.00433 0.00321 0.00355 0.0144 0.00886 0.0216 0.002 0.00222
2L:7675680-7675830:plus -12.0306 -1.85321 9.83486 7.47706 -27.1224 -18.9796 -15.6055
1.22222 2.73394 0.000306 0.0186 0.000157 0.00085 0.0245 0.0155 0.0101 0.0113 0.00756
2L:7678480-7678630:minus -32.1429 1.43119 1.04587 3.05505 10.4286 -26.5612 -17.0367 3.70833
-0.513761 0.0179 0.00619 0.00813 0.00878 0.0000139 0.0237 0.0129 0.00452 0.0221
2L:7678480-7678630:plus -22.7653 6.13761 1.40367 3.85321 -27.5306 -19.1531 12.5963 -3.77778
2.33028 0.00405 0.000993 0.00717 0.00623 0.0351 0.0177 4.22e-06 0.0467 0.00836
2L:7701580-7701730:minus -12.1531 2.42202 1.11927 7.78899 -18.1531 -16.7143 -13.2294
6.04167 3.92661 0.000352 0.0043 0.00793 0.000642 0.00829 0.00419 0.00609 0.00162 0.00461
2L:7701580-7701730:plus -31.5918 0.733945 4.18349 5.49541 -7.56122 -9.57143 -22.2018
1.20833 1.36697 0.0136 0.00797 0.00249 0.00355 0.000419 0.00326 0.0306 0.0113 0.0124
2L:7782920-7783070:minus -24.4796 13.5306 4.66055 3.62385 -19.0102 -17.3469 -20.2844
0.0694444 1.27523 0.00804 0.0000206 0.00205 0.00673 0.0157 0.0058 0.0216 0.0164 0.013
2L:7782920-7783070:plus -40.7755 2.87156 7.40367 1.44954 -27.4592 -9.02041 -21.9358 0.541667
2.6422 0.0433 0.00365 0.000595 0.0179 0.0329 0.00213 0.0291 0.0141 0.00768
2L:7790480-7790630:minus -24.0714 -0.229358 1.88991 7.12844 -17.8571 -25.5306 -3.88991
3.23611 -2.69725 0.00731 0.0111 0.00602 0.00118 0.00698 0.0205 0.000723 0.00545 0.0411
2L:7790480-7790630:plus -21.8163 -2.70642 1.95413 6.74312 -17.3776 -8.93878 -4.92661
-1.80556 1.58716 0.00244 0.0238 0.00588 0.0015 0.00419 0.00201 0.000972 0.0283 0.0113
2L:7795900-7796050:minus -11.9184 7.33945 10.2936 5.86239 -18.0816 -19.4184 6.91743 -2.29167
1.22018 0.000293 0.000544 0.00012 0.00282 0.00759 0.0198 0.0000318 0.0322 0.0133
2L:7795900-7796050:plus -24.6327 10.1101 9.86239 7.33028 -17.4184 -9.5 -14.1101 2.01389 0.33945
0.00824 0.000126 0.000155 0.00103 0.00457 0.00294 0.00743 0.00858 0.0168
2L:7799740-7799890:minus -22.0306 4.59633 6.43119 3.70642 -18.3776 -17.3469 -7.14679
-2.25 2.88991 0.00293 0.00191 0.000949 0.0066 0.00926 0.0058 0.00175 0.0319 0.00725
2L:7799740-7799890:plus -30.5918 -1.0367 7.57798 7.37615 1.10204 -27.5612 -13.4495 2.91667 2.01835
0.00963 0.0145 0.000545 0.000951 0.000124 0.0393 0.00641 0.00616 0.00928
2L:7800100-7800250:minus -33.8163 -0.761468 4.85321 2.02752 -17.1939 1.02041 -16.2385
-1.73611 -0.220183 0.0299 0.0133 0.00189 0.0141 0.00377 0.000118 0.0113 0.0277 0.0201
2L:7800100-7800250:plus -31.3673 4.84404 -5.22936 1.62385 -18.9796 -8.72449 -25.2477
5.93056 -6.88991 0.0116 0.00174 0.0523 0.0168 0.0155 0.00169 0.0487 0.00171 0.11
2L:7809280-7809430:minus -31.5918 -2.0367 -1.41284 9.51376 -27.4592 -19.0816 -20.1835
0.402778 8.19266 0.0136 0.0196 0.0181 0.000292 0.0329 0.017 0.0213 0.0147 0.000548
2L:7809280-7809430:plus -13.1429 5.47706 0.311927 7 -18.7551 -9.27551 -3.79817
-0.791667 2.42202 0.00065 0.00133 0.0104 0.00132 0.0144 0.00244 0.000705 0.0212 0.00816
2L:7809760-7809910:minus -31.2857 -4.21101 4.47706 1.59633 -18.0816 -16.2755 -12.3303
2.48611 7.36697 0.0113 0.0358 0.00221 0.017 0.00759 0.00413 0.00496 0.00723 0.00108
2L:7809760-7809910:plus -22.8878 7.36697 -0.550459 2.09174 -26.9286 -27.5612 -9.6422 7.69444
1.27523 0.00441 0.000535 0.0139 0.0137 0.0214 0.0393 0.00288 0.000693 0.013
2L:7810720-7810870:minus -21.9592 -2.04587 4.94495 3.31193 -18.1531 -27.3367 -5.0367
-3.56944 2.06422 0.00279 0.0197 0.00182 0.00786 0.00829 0.0347 0.001 0.0445 0.00912
2L:7810720-7810870:plus -23.5204 13.5306 3.3578 6.37615 -27.2347 1.09184 -5.77064 3.66667

-2.36697 0.00645 0.0000206 0.00346 0.00192 0.0283 0.000091 0.00123 0.0046 0.0371
2L:7811280-7811430:minus -33.0714 -0.0642202 0.688073 4.81651 -27.1939 -8.79592
-10.8257 1.47222 1.2844 0.0267 0.0105 0.0092 0.00451 0.0267 0.0019 0.00361 0.0104 0.0129
2L:7811280-7811430:plus -12.8878 5.66972 1.22936 5.74312 -26.9286 -18.2755 1.18349 -0.902778
9.87156 0.000523 0.00123 0.00763 0.00302 0.0214 0.0112 0.00021 0.0219 0.000217
2L:7812920-7813070:minus -23.551 0.761468 2.7156 4.04587 -17.7143 -26.898 -21.0367 1.625
-2.04587 0.00648 0.00789 0.00442 0.00572 0.00627 0.0302 0.0248 0.00983 0.0336
2L:7812920-7813070:plus -31.8163 -4.49541 0.192661 11.0183 -17.4184 -17.8265 -18.3853
1.95833 -0.0550459 0.016 0.0386 0.0109 0.000052 0.00457 0.00699 0.0159 0.00875 0.0187
2L:7813360-7813510:minus -30.4388 -2.47706 3.95413 5.57798 -18.4184 -8.30612 -24.5413
4.75 -2.3945 0.00923 0.0223 0.00273 0.0034 0.0101 0.000973 0.0443 0.00293 0.0375
2L:7813360-7813510:plus -31.6224 1.9633 -1.81651 -0.541284 -27.2245 -27.898 -8.63303 6.09722
1.37615 0.0137 0.00509 0.0205 0.0316 0.0272 0.0537 0.00239 0.00158 0.0123
2L:7813740-7813890:minus -21.9286 -0.486239 4.11009 5.49541 -17.449 -17.051 -19.844 -0.0694444
-1.73394 0.0027 0.0121 0.00256 0.00355 0.00484 0.00479 0.02 0.0171 0.0311
2L:7813740-7813890:plus -24.7857 -4.20183 2.45872 3.92661 -18.3776 -17.9796 -21.5505 3.58333 9.56881
0.00829 0.0357 0.00488 0.00601 0.00926 0.00764 0.0272 0.00475 0.000303
2L:7814260-7814410:minus -24.449 0.605505 3.99083 7.34862 -18.3776 -8.38776 -17.2661 -0.833333
0.0458716 0.00802 0.00834 0.00269 0.000998 0.00926 0.00101 0.0134 0.0215 0.0182
2L:7814260-7814410:plus -21.4082 0.155963 1.74312 9.7156 -26.1939 -17.7143 -10.6239 0.458333 0.0825688
0.00198 0.00977 0.00635 0.000242 0.0175 0.00634 0.00347 0.0145 0.0181
2L:7814800-7814950:minus -22.9592 -0.844037 -2.34862 1.91743 -9.5 -8.68367 -23.6239 0.791667 -2.74312
0.00475 0.0136 0.0239 0.015 0.00293 0.00157 0.0388 0.013 0.0417
2L:7814800-7814950:plus -33.2245 1.93578 0.40367 1.84404 -9.16327 -8.72449 -14.6239 -0.75 0.275229
0.0276 0.00514 0.0101 0.0154 0.00249 0.00169 0.0083 0.021 0.0171
2L:7821480-7821630:minus -23.2959 14.2936 3.52294 1.78899 -17.3776 -0.173469 -11.3945 2.30556
2.84404 0.00605 6.04e-06 0.00324 0.0158 0.00419 0.000456 0.00405 0.00772 0.00736
2L:7821480-7821630:plus -21.7041 -1.18349 9.46789 0.550459 -16.449 -17.9388 -5.56881 11.1667 7.22018
0.00231 0.0152 0.000194 0.0233 0.00307 0.00738 0.00117 0.0000662 0.00119
2L:7827260-7827410:minus -13.1837 -2.62385 7.20183 2.66055 -17.7143 -8.27551 -16.6239 -1.47222 1.36697
0.000705 0.0232 0.000658 0.0106 0.00627 0.000919 0.0121 0.0258 0.0124
2L:7827260-7827410:plus -14.7755 16.3761 8.02752 6.05505 -8.64286 -18.0102 -10 -0.125 5.70642
0.00157 1.59e-07 0.000431 0.0024 0.00139 0.00812 0.00308 0.0174 0.00222
2L:7827680-7827830:minus -20.9184 -0.614679 3.25688 0.889908 -17.3776 -8.27551 -18.055 3.11111
1.78899 0.00181 0.0127 0.0036 0.0213 0.00419 0.000919 0.0151 0.00572 0.0103
2L:7827680-7827830:plus -20.6633 2.38532 -2.47706 6.36697 10.9184 -17.3469 3.52294 0.152778 3.92661
0.00173 0.00436 0.0248 0.00193 4.19e-06 0.0058 0.0000981 0.016 0.00461
2L:7828340-7828490:minus -23.2959 3.11927 -1 -0.0550459 -18.7959 -17.5714 -9.78899 1.55556 3.61468
0.00605 0.00333 0.016 0.0272 0.0148 0.00616 0.00296 0.0101 0.00526
2L:7828340-7828490:plus -21.5102 -0.669725 0.880734 -1.54128 -18.4592 -9.5 -14.0367 -1.51389 7.45872
0.00202 0.0129 0.00861 0.0439 0.0109 0.00294 0.00731 0.0261 0.00103
2L:7828940-7829090:minus -24.8265 6.72477 6.66055 0.862385 -9.27551 -17.8571 -12.8899 5.59722 3.09174
0.0083 0.000747 0.000851 0.0214 0.00284 0.00719 0.00564 0.002 0.00678
2L:7828940-7829090:plus -39.9592 -3.55046 -2.6055 2.54128 -9.16327 -17.3469 -7.93578 -0.708333
1.55046 0.0348 0.03 0.0258 0.0112 0.00249 0.0058 0.00208 0.0207 0.0115
2L:7859840-7859990:minus -32.8061 -1.00917 -1.53211 2.55046 -17.6735 -17.4184 -14.5138 10.7639 -0.623853
0.0237 0.0144 0.0188 0.0112 0.00572 0.00585 0.00811 0.0000924 0.0232
2L:7859840-7859990:plus -13.449 1.66055 -2.00917 3.33945 -7.89796 -8.7551 -19.5321 2.86111 7.42202
0.000914 0.00569 0.0217 0.00774 0.000678 0.00172 0.019 0.00629 0.00106
2L:7885440-7885590:minus -31.4388 12.5505 6.76147 5.6422 -27.898 -8.45918 -20.9266 -1.34722 3.88991
0.0122 0.0000313 0.000811 0.00325 0.04 0.00111 0.0243 0.0249 0.00467
2L:7885440-7885590:plus -24.2551 13.4592 3.62385 1.78899 -8.56122 -8.72449 -13.0092 -1.19444 1.69725
0.00768 0.000064 0.00312 0.0158 0.00112 0.00169 0.00579 0.0239 0.0108
2L:7886080-7886230:minus -32 14.2294 0.678899 3.18349 -26.2653 -19.5714 -13.5321 0.944444 -2.36697

0.0171 6.48e-06 0.00923 0.00837 0.018 0.0202 0.00653 0.0124 0.0371
2L:7886080-7886230:plus -32.8061 -2.46789 -0.963303 -0.0733945 -27.4592 -17.5 -22.367 5.36111
3.98165 0.0237 0.0222 0.0158 0.0274 0.0329 0.00599 0.0315 0.00223 0.00439
2L:7914440-7914590:minus -22 1.04587 -2.9633 3.61468 -18.4898 -27.3367 -7.62385 1.68056 3.6055
0.00291 0.00712 0.0285 0.0068 0.0116 0.0347 0.00194 0.00964 0.00532
2L:7914440-7914590:plus -42 4.97959 -1.74312 4.02752 -18.449 -0.204082 -17.6147 8.125 1.90826
0.0684 0.000156 0.02 0.00581 0.0107 0.000463 0.0141 0.000543 0.00974
2L:7914940-7915090:minus -21.7041 -2.08257 0.137615 7.90826 -9.20408 -18.3469 -14.4771 -2.22222 -2.85321
0.00231 0.0199 0.0111 0.000611 0.00262 0.0124 0.00805 0.0316 0.0429
2L:7914940-7915090:plus -22.1837 3.51376 2.10092 5.90826 -18.3469 -25.8265 -15.5872 0.708333 1.73394
0.00315 0.00288 0.00556 0.00271 0.00894 0.0212 0.0101 0.0134 0.0106
2L:7956840-7956990:minus -13.0816 6.91743 -1.24771 2.95413 -27.6327 -18.1224 -23.4495 5.54167 5.55046
0.000599 0.000678 0.0172 0.00917 0.0369 0.00988 0.0377 0.00205 0.00241
2L:7956840-7956990:plus -22.2143 -2.85321 -1.6789 3.46789 -27 -27.1224 -11.6055 4.98611 3.45872
0.00317 0.0248 0.0196 0.00726 0.0233 0.0325 0.00423 0.00264 0.00591
2L:7957420-7957570:minus -32.2245 1.21101 1.40367 1.49541 -26.9694 -17.051 -22.0826 -1.65278 -2.46789
0.0183 0.00671 0.00717 0.0176 0.023 0.00479 0.0299 0.0271 0.0384
2L:7957420-7957570:plus -13.4898 -0.605505 -0.944954 5.69725 -17.0816 0.826531 -18.9817 -0.708333
5.23853 0.000946 0.0126 0.0157 0.00314 0.00334 0.000153 0.0174 0.0207 0.00285
2L:7960900-7961050:minus -13.0408 0.807339 9.56881 8.05505 -26.6327 9.67347 -20.5138 -0.541667
-3.72477 0.000576 0.00776 0.000183 0.000578 0.0188 0.0000217 0.0225 0.0197 0.0519
2L:7960900-7961050:plus -42.7347 -1.56881 0.477064 2.01835 -27.3776 -28.4082 -15.8899 4.88889 9.92661
0.0844 0.0171 0.00989 0.0141 0.032 0.0615 0.0106 0.00276 0.000193
2L:7961860-7962010:minus -12.8469 0.522936 -1.54128 -0.183486 -17.4184 -17.051 -22.9817 7.15278
-2.13761 0.000502 0.00859 0.0188 0.0283 0.00457 0.00479 0.035 0.000928 0.0345
2L:7961860-7962010:plus -30.6633 -0.550459 7.69725 6.42202 -18.3776 0.0510204 -26.2294 5.83333
-1.50459 0.00976 0.0124 0.000513 0.00188 0.00926 0.000349 0.055 0.00179 0.0294
2L:7973680-7973830:minus -30.6327 1.25688 -3.27523 2.33945 -18.6429 -0.244898 -26.9908 -3 -6.52294
0.00968 0.00659 0.0311 0.0122 0.0121 0.000496 0.0604 0.0386 0.101
2L:7973680-7973830:plus -33.8163 -1.27523 4.72477 1.06422 -27.2653 -19.3469 -16.4954 4.36111 3.09174
0.0299 0.0156 0.00199 0.0203 0.0302 0.0194 0.0118 0.00346 0.00678
2L:7976840-7976990:minus -34.0714 -2.98165 -3.88991 -0.211009 -27 -18.1224 -15.9633 2.06944 7.73394
0.031 0.0257 0.0369 0.0286 0.0233 0.00988 0.0108 0.00841 0.000784
2L:7976840-7976990:plus -14.6735 4.91743 3.27523 -0.422018 -18.3469 -9.16327 -17.9817 3.18056
11.8532 0.00152 0.00169 0.00357 0.0305 0.00894 0.00223 0.0149 0.00557 0.0000428
2L:7984140-7984290:minus -22.398 0.889908 -1.89908 -0.40367 -18.0816 -18.6429 -4.49541 11.7083 1.54128
0.00344 0.00753 0.021 0.0302 0.00759 0.0139 0.000859 0.0000409 0.0115
2L:7984140-7984290:plus -22.398 0.669725 1.02752 5.57798 -18.1939 -9.30612 -8.81651 -0.958333
9.87156 0.00344 0.00815 0.00819 0.0034 0.0086 0.00257 0.00247 0.0223 0.000217
2L:7991680-7991830:minus -12.9592 5.72477 -0.0366972 7.80734 -17.9286 -37.1531 -5.73394 0.722222
3.07339 0.000551 0.0012 0.0117 0.00063 0.0071 0.138 0.00122 0.0133 0.00683
2L:7991680-7991830:plus -34.602 -3.41284 -0.522936 -0.834862 -8.71429 -17.2755 -16.7156 1.59722
-1.06422 0.032 0.0289 0.0137 0.0349 0.00159 0.00544 0.0122 0.00993 0.0266
2L:7992820-7992970:minus -31.1327 0.311927 -1.78899 0.275229 -18.449 -18.6429 -18.6147 -0.430556
-4.04587 0.0107 0.00925 0.0203 0.025 0.0107 0.0139 0.0164 0.0191 0.0563
2L:7992820-7992970:plus -31.4796 1.76147 -0.376147 3.75229 -8.16327 0.0204082 -17.3211 -1.22222
6.11927 0.0128 0.00548 0.0131 0.00649 0.000873 0.000377 0.0135 0.024 0.00178
2L:7993300-7993450:minus -22.6633 -1.07339 -2.75229 4.48624 -17.7857 -19.1531 -18.8899 3.98611 3.46789
0.00384 0.0147 0.0269 0.00487 0.00676 0.0177 0.0171 0.00404 0.00582
2L:7993300-7993450:plus -32 1.7156 5.92661 6.57798 -9.19388 -18.2755 -19.7339 -0.916667 -2.30275
0.0171 0.00557 0.00119 0.00167 0.00253 0.0112 0.0197 0.022 0.0363
2L:7994040-7994190:minus -4.11224 -2.22936 -0.192661 -2.38532 -18.1531 -37.6633 -8.68807 5.88889
7.84404 0.000126 0.0208 0.0123 0.058 0.00829 0.171 0.00241 0.00175 0.000701
2L:7994040-7994190:plus -13.5204 3.7551 2.21101 3.13761 -8.64286 -7.79592 -19.0642 -1.55556 5.66055

0.00097 0.00122 0.00534 0.00854 0.00139 0.000723 0.0176 0.0264 0.00227
2L:7998840-7998990:minus -41.6735 -4.51376 2.65138 2.62385 -7.85714 -9.94898 -23.6514 -0.25 -2.26606
0.0637 0.0388 0.00453 0.0108 0.000594 0.00393 0.0389 0.0181 0.0359
2L:7998840-7998990:plus -21.6939 -0.321101 0.605505 7.12844 -17.7551 -36.1531 -0.229358 12.2778
5.18349 0.00226 0.0115 0.00946 0.00118 0.00658 0.0908 0.000299 0.0000235 0.00295
2L:8000900-8001050:minus -31.102 0.330275 -0.293578 3.61468 -27.6735 -18.051 -13.9358 -0.986111
-4.01835 0.0106 0.00919 0.0128 0.0068 0.0375 0.00886 0.00715 0.0225 0.056
2L:8000900-8001050:plus -29.0612 0.623853 -2.33028 5.57798 -17.449 -9.42857 -21.211 3.75 3.10092
0.00832 0.00829 0.0238 0.0034 0.00484 0.0027 0.0256 0.00445 0.00674
2L:8001560-8001710:minus -21.5918 -3.47706 6.08257 0.651376 -26.9286 -9.7551 -24.8807 -2.76389 0.816514
0.00209 0.0294 0.00111 0.0226 0.0214 0.00358 0.0464 0.0364 0.015
2L:8001560-8001710:plus -13.4796 -0.587156 -0.12844 9.59633 -8.33673 -8.5 -9.09174 -1.08333 3.29358
0.000935 0.0126 0.0121 0.000275 0.00101 0.00126 0.0026 0.0231 0.00623
2L:8002140-8002290:minus -22.5918 -0.761468 6.18349 4.17431 -17.6429 -17.2755 -8.26606 4.69444
4.37615 0.00365 0.0133 0.00106 0.00537 0.00562 0.00544 0.00222 0.003 0.00376
2L:8002140-8002290:plus -31.5816 3.90816 4.26606 5.49541 -26.9694 -26.898 5.09174 7.01389 -0.926606
0.0135 0.00102 0.00241 0.00355 0.023 0.0302 0.0000558 0.000998 0.0256
2L:8004260-8004410:minus -32.0408 14.0275 0.697248 5.49541 -18.9388 0.826531 -20.0459 5.26389 -1.18349
0.0174 8.23e-06 0.00917 0.00355 0.0151 0.000153 0.0208 0.00233 0.0276
2L:8004260-8004410:plus -31.8469 4.86239 15.5229 4.04587 -18.7857 -26.6327 -22.1009 2.45833 2.42202
0.0162 0.00172 7.23e-07 0.00572 0.0148 0.0251 0.03 0.00731 0.00816
2L:8009380-8009530:minus -12.1939 2.0367 5.05505 6.29358 -27.1939 0.897959 -16.7339 -5.54167 0.862385
0.000366 0.00495 0.00174 0.00202 0.0267 0.000144 0.0123 0.0692 0.0148
2L:8009380-8009530:plus -30.8878 4.06422 0.981651 4.91743 1.17347 -7.45918 -12.8716 8.25 -0.541284
0.0103 0.00235 0.00832 0.00447 0.000101 0.000582 0.00561 0.000505 0.0223
2L:8029400-8029550:minus -41.5102 1.85321 0.266055 6.74312 -18.449 -19.5 -24.7339 0.513889 -2.74312
0.0582 0.0053 0.0106 0.0015 0.0107 0.0199 0.0455 0.0142 0.0417
2L:8029400-8029550:plus -24.5204 3.85321 2.20183 5.30275 1.14286 -16.9796 -8.22018 -1.43056 -1.00917
0.00813 0.00254 0.00536 0.00396 0.000113 0.0044 0.0022 0.0255 0.0262
2L:8030200-8030350:minus -22.6224 3.83673 0.321101 1.57798 -19.2755 -17.5 -11.2385 4.22222 1.7156
0.00371 0.00115 0.0104 0.0171 0.0164 0.00599 0.00392 0.00367 0.0107
2L:8030200-8030350:plus -22.1531 8.69725 5.54128 4.0367 -17.8163 -7.38776 -26.211 3.51389 1.34862
0.0031 0.000263 0.00141 0.00578 0.00682 0.000563 0.0549 0.00489 0.0125
2L:8030960-8031110:minus -32.6327 -3.3945 3.68807 1.58716 1.14286 -8.68367 -18.6881 2.125 -0.504587
0.0212 0.0287 0.00304 0.017 0.000113 0.00157 0.0166 0.00824 0.0221
2L:8030960-8031110:plus -23.1837 4.88991 1.79817 2.88991 -17.1531 -19.2755 -13.0642 -0.972222
7.45872 0.00553 0.0017 0.00623 0.00942 0.00364 0.0185 0.00587 0.0224 0.00103
2L:8031520-8031750:minus -33.1122 2.23853 6.26606 4.05505 -17.1837 -17.898 -13.9908 0.166667 3.51376
0.0271 0.0046 0.00103 0.00566 0.00366 0.00726 0.00724 0.0159 0.00566
2L:8031520-8031750:plus -22.7755 0.825688 -3.7156 2.81651 -18.1224 -19.4184 -3.06422 6.97222 11.3853
0.00412 0.00771 0.0352 0.00982 0.00782 0.0198 0.000578 0.00102 0.0000815
2L:8040960-8041110:minus -23.7857 1.47706 -2.48624 1.53211 -28.7959 -9.02041 -20.1468 6.13889 0.963303
0.00679 0.00608 0.0249 0.0174 0.0641 0.00213 0.0211 0.00155 0.0145
2L:8040960-8041110:plus -32.8878 3.50459 5.12844 9.23853 -8.60204 -8.79592 -27.6422 7.06944 5.38532
0.0247 0.00289 0.00169 0.000424 0.00125 0.0019 0.0656 0.00097 0.00267
2L:8042860-8043010:minus -23.1837 13.4592 -1.6055 1.78899 -17.1531 -8.61224 -8.16514 -4.72222 3.46789
0.00553 0.000064 0.0192 0.0158 0.00364 0.00152 0.00218 0.058 0.00582
2L:8042860-8043010:plus -33.2959 11.422 7.56881 7.00917 -9.5 0.979592 -26.1835 -0.208333 -7.0367
0.0279 0.0000644 0.000547 0.00126 0.00293 0.000136 0.0547 0.0178 0.114
2L:8043280-8043430:minus -23.398 5.92661 6.58716 1.58716 -18.4184 9.60204 -6.23853 0.305556 -0.146789
0.00619 0.00109 0.00088 0.017 0.0101 0.0000248 0.0014 0.0152 0.0194
2L:8043280-8043430:plus -31.7755 6.3578 1.94495 0.211009 -26.9694 -8.53061 -14.7431 2.41667 3.55963
0.0157 0.000892 0.0059 0.0254 0.023 0.00136 0.00851 0.00742 0.00545
2L:8071580-8071730:minus -33.0408 1.43119 2.97248 7.63303 1.10204 -18.0816 -14.8991 2.88889 0.715596

0.0264 0.00619 0.00401 0.000745 0.000124 0.00934 0.00879 0.00622 0.0154
2L:8071580-8071730:plus -33 -0.0183486 14.9633 7.29358 -19.051 -18.0102 -17.3945 6.25 -3.42202
0.0258 0.0104 1.77e-06 0.00104 0.0162 0.00812 0.0136 0.00147 0.0488
2L:8072780-8072930:minus -32.7041 -2.3211 -1.93578 4.15596 20.2041 -17.5 -19.3486 1.625 -4.49541
0.0222 0.0213 0.0212 0.00539 3.64e-07 0.00599 0.0185 0.00983 0.0638
2L:8072780-8072930:plus 6.77551 1.01835 -2.86239 3.52294 -17.4898 -8.72449 -13.8349 5.05556 0.908257
2e-06 0.00719 0.0277 0.00706 0.0053 0.00169 0.00699 0.00256 0.0147
2L:8083280-8083430:minus -31.2959 -2.99083 -1.81651 2.02752 -17.3776 -18.2755 -3.37615 -2.41667 5.00917
0.0113 0.0257 0.0205 0.0141 0.00419 0.0112 0.000628 0.0333 0.0031
2L:8083280-8083430:plus -23.1122 -1.90826 7.40367 10.8624 0.540816 -16.7143 -14.0459 -1.98611 1.72477
0.0053 0.0189 0.000595 0.0000687 0.000321 0.00419 0.00733 0.0297 0.0106
2L:8083700-8083850:minus -23.3367 -0.752294 0.449541 0.46789 -17.9796 0.0204082 -24.5688 -2.59722
11.8532 0.00614 0.0132 0.00998 0.0239 0.00726 0.000377 0.0445 0.0349 0.0000428
2L:8083700-8083850:plus -29.1735 3.2844 -0.449541 6.06422 -18.4184 -28.8571 -7.56881 2.15278
-0.798165 0.00835 0.00313 0.0134 0.00239 0.0101 0.0699 0.00192 0.00816 0.0246
2L:8084700-8084850:minus -31 -9.05505 -2.68807 0.256881 -17.1837 -19.1939 -21.3578 -0.111111 0.357798
0.0105 0.115 0.0264 0.0251 0.00366 0.0178 0.0262 0.0173 0.0166
2L:8084700-8084850:plus -32.7041 2.42202 0.899083 1.91743 -26.3061 -45.4082 -16.6055 2.44444 11.8073
0.0222 0.0043 0.00856 0.015 0.0181 0.225 0.012 0.00734 0.0000538
2L:8085640-8085790:minus -32.7347 1.66972 3.3578 9.49541 -27.1939 1.02041 -11.4495 5.69444 7.22018
0.0225 0.00567 0.00346 0.000308 0.0267 0.000118 0.00409 0.00191 0.00119
2L:8085640-8085790:plus -21.8878 -3.01835 3.53211 2.34862 -17.9796 -26.602 -17.2294 6.69444 3.88073
0.00258 0.0259 0.00323 0.0121 0.00726 0.0248 0.0133 0.00118 0.00475
2L:8086820-8087030:minus -3.37755 2.17431 2.02752 7.7156 -17.4184 -7.72449 -18.2385 3.88889 7.63303
0.0000645 0.00471 0.00572 0.000691 0.00457 0.00068 0.0155 0.00421 0.000928
2L:8086820-8087030:plus -11.6939 -4.52294 0.917431 9.3211 -8.64286 -16.7143 3.66055 -1.90278 7.45872
0.000261 0.0389 0.00851 0.000391 0.00139 0.00419 0.0000934 0.029 0.00103
2L:8087580-8087730:minus -34.0714 3.65138 8.3945 0.0733945 -17.602 -18.2755 -20.3119 -0.652778
-0.229358 0.031 0.00274 0.000354 0.0263 0.00547 0.0112 0.0217 0.0204 0.0202
2L:8087580-8087730:plus -32.2653 2.16514 1.92661 3.93578 -18.1531 -8.42857 -23.4771 -1.61111 -6.53211
0.0184 0.00473 0.00594 0.00597 0.00829 0.00104 0.0379 0.0268 0.102
2L:8097520-8097770:minus -22 -2.61468 1.23853 0.954128 -27.1224 -8.27551 -23.3853 3.73611 5.48624
0.00291 0.0232 0.0076 0.0208 0.0245 0.000919 0.0374 0.00447 0.00251
2L:8097520-8097770:plus -3.82653 5.25688 -2.3211 7.62385 -16.7143 -8.7551 -20.9908 5.63889 -2
0.000107 0.00146 0.0237 0.000759 0.00319 0.00172 0.0245 0.00196 0.0332
2L:810460-810610:minus -23.5306 2.48624 1.26606 5.49541 -27.4592 -17.898 -22.1284 1.05556 -2.3578
0.00646 0.0042 0.00753 0.00355 0.0329 0.00726 0.0302 0.0119 0.0369
2L:810460-810610:plus -31.7449 1.22018 5.13761 8.05505 -7.93878 -9.27551 -16.0917 5.15278 -2.82569
0.0152 0.00668 0.00168 0.000578 0.000767 0.00244 0.011 0.00245 0.0425
2L:811500-811650:minus -38.9898 -4.86239 8.62385 2.87156 -18.4184 -19.3776 -19.3211 -0.111111
-4.52294 0.0325 0.0426 0.000311 0.00948 0.0101 0.0195 0.0184 0.0173 0.0642
2L:811500-811650:plus -24.0306 -1.16514 12.2936 -0.46789 -27.2653 -18.5714 -12.9083 -0.666667 3.41284
0.00723 0.0151 0.0000347 0.0309 0.0302 0.0136 0.00566 0.0205 0.006
2L:8126860-8127010:minus -41.551 0.642202 15.6606 3.08257 -27.602 -19.051 -19.3028 6.13889 1.33028
0.0607 0.00823 3.94e-07 0.0087 0.0364 0.0165 0.0183 0.00155 0.0127
2L:8126860-8127010:plus -23.5204 12.3853 1.46789 6.09174 -27.2653 -16.7143 -13.8349 0.569444 2.21101
0.00645 0.0000353 0.007 0.00235 0.0302 0.00419 0.00699 0.014 0.00862
2L:8127560-8127710:minus -20.5918 -2.76147 0.651376 5.85321 -7.89796 -18.7551 -21.5229 1.43056 1.44037
0.00169 0.0241 0.00932 0.00287 0.000678 0.0141 0.027 0.0105 0.012
2L:8127560-8127710:plus -32.8571 -2.70642 -0.926606 4.37615 -17.4184 -16.8265 -16.8257 5.88889
6.6422 0.0244 0.0238 0.0156 0.00504 0.00457 0.0043 0.0125 0.00175 0.00148
2L:8128720-8128870:minus -21.4388 -2.47706 0.697248 2.09174 -26.9694 -26.602 -4.98165 -2.13889 9.87156
0.002 0.0223 0.00917 0.0137 0.023 0.0248 0.000988 0.0309 0.000217
2L:8128720-8128870:plus -32.8163 4 0.486239 3.43119 -27.5714 -10.0204 -22.3761 5.06944 1.23853 0.024

0.0024 0.00986 0.00749 0.0357 0.00399 0.0315 0.00255 0.0132
2L:8129280-8129430:minus -22.9286 1.81651 0.33945 -1.88991 -16.8878 -8.65306 -1.25688 2.34722 1.21101
0.0046 0.00537 0.0104 0.0493 0.00326 0.00156 0.000376 0.00761 0.0134
2L:8129280-8129430:plus -23.1122 8.3211 8.79817 6.57798 -17.6429 -8.5 -20.0734 -3.77778 -1.85321
0.0053 0.000321 0.000283 0.00167 0.00562 0.00126 0.0209 0.0467 0.032
2L:8156940-8157090:minus -31.5102 -1.20183 7.82569 4.07339 -8.93878 -9.23469 -11.3945 8.83333 3.66972
0.013 0.0153 0.00048 0.00562 0.00216 0.00238 0.00405 0.000356 0.00514
2L:8156940-8157090:plus -21.8571 -3.22936 15.5963 1.7156 -17.7143 0.826531 -21.1835 -2.09722 -5.69725
0.00249 0.0275 6.15e-07 0.0162 0.00627 0.000153 0.0254 0.0306 0.0833
2L:815880-816030:minus -23.5918 13.5963 0.440367 3.76147 -18.449 -7.72449 -26.9725 3.30556 -3.34862
0.00656 0.0000131 0.01 0.00645 0.0107 0.00068 0.0603 0.0053 0.048
2L:815880-816030:plus -33.0714 4.90826 2.7156 3.6055 -8.96939 -18.051 -5.72477 0.902778 -0.220183
0.0267 0.00169 0.00442 0.00686 0.00226 0.00886 0.00122 0.0125 0.0201
2L:8159660-8159810:minus -24.5204 3.00917 2.73394 -0.201835 -27.5714 -18.051 -14.5321 -3 5.81651
0.00813 0.00347 0.00439 0.0285 0.0357 0.00886 0.00814 0.0386 0.00204
2L:8159660-8159810:plus -24.0816 1.56881 -3.11009 3.45872 -27.9286 -18.1224 -21.8165 1.79167 3.45872
0.00732 0.00588 0.0297 0.00731 0.0412 0.00988 0.0285 0.00928 0.00591
2L:8162400-8162550:minus -23.5204 15.3394 6.41284 2.62385 10.3571 -18.7857 -14.4587 0.583333 -0.229358
0.00645 1.34e-06 0.000959 0.0108 0.0000208 0.0146 0.00802 0.0139 0.0202
2L:8162400-8162550:plus -32 0.293578 10.1651 2.66972 -26.5306 -18.1224 -15.4037 1.94444 -2.34862
0.0171 0.00932 0.000129 0.0106 0.0185 0.00988 0.00972 0.00879 0.0368
2L:8164140-8164290:minus -32.1531 -1.40367 -1.51376 1.36697 -26.9286 -19.2041 -7.07339 3.38889 5.53211
0.0179 0.0163 0.0187 0.0186 0.0214 0.0179 0.00172 0.00513 0.00245
2L:8164140-8164290:plus -40.7857 3.17431 9.30275 9.76147 -18.4898 -9.02041 -21.4404 5.375 1.01835
0.0433 0.00326 0.000213 0.000212 0.0116 0.00213 0.0266 0.00222 0.0142
2L:8171660-8171810:minus -12.2245 -4.95413 8.50459 3.43119 -17.7857 -9.20408 -15.6881 5.83333 5.84404
0.000376 0.0436 0.000332 0.00749 0.00676 0.00225 0.0102 0.00179 0.00202
2L:8171660-8171810:plus -12.898 -5.38532 5.7156 3.87156 -17.4184 -17.7551 -11.3028 -3.25 9.97248
0.000524 0.0488 0.00131 0.00618 0.00457 0.00643 0.00397 0.0411 0.00016
2L:819480-819630:minus -14.6735 8.40367 11.4495 7.2844 -18.6429 -8.45918 -14.4312 5.36111 -0.100917
0.00152 0.000307 0.0000608 0.00105 0.0121 0.00111 0.00797 0.00223 0.019
2L:819480-819630:plus -11.5918 8.44037 12 3.59633 -27.7959 -18.0102 -12.0459 5.65278 0.642202 0.000257
0.000301 0.0000432 0.00693 0.0386 0.00812 0.00466 0.00195 0.0157
2L:8196960-8197110:minus -23.2653 -1.11009 13.5596 7.45872 -17.4184 -0.244898 -14.3853 2.69444
2.99083 0.00593 0.0148 0.0000125 0.000894 0.00457 0.000496 0.00789 0.00669 0.00706
2L:8196960-8197110:plus -31.4082 -0.366972 1.37615 1.88073 0.908163 -17.9796 -15.422 1.55556
-2.92661 0.0119 0.0117 0.00724 0.0152 0.000153 0.00764 0.00975 0.0101 0.0437
2L:8198880-8199030:minus -32.8878 -0.899083 0.174312 5.75229 -18.449 -18.4184 -18.0275 1.5 4.77064
0.0247 0.0139 0.0109 0.00296 0.0107 0.0127 0.015 0.0103 0.0034
2L:8198880-8199030:plus -30.7041 7.63303 1.97248 6.41284 -27.3061 9.30612 -18.3853 1.23611 2.17431
0.00993 0.000464 0.00584 0.00189 0.0315 0.0000398 0.0159 0.0112 0.00872
2L:8205060-8205210:minus -13.449 3.97959 10.5229 10.5963 -26.8571 -9.42857 -3.93578 -1.76389 5.22936
0.000914 0.000669 0.000104 0.0001 0.0198 0.0027 0.000733 0.028 0.00288
2L:8205060-8205210:plus -21.6939 2.37615 11.0275 3.42202 -9.23469 -36.2245 -10.9358 6.91667 7.45872
0.00226 0.00437 0.0000785 0.00751 0.00277 0.0923 0.00369 0.00105 0.00103
2L:8211200-8211350:minus -21.6224 4.99083 -1.31193 6.49541 -8.89796 -8.79592 -13.1743 2.27778 4.66055
0.00213 0.00164 0.0176 0.00176 0.00189 0.0019 0.00602 0.0078 0.0035
2L:8211200-8211350:plus -23.5612 5.93578 1.85321 2.77982 -18.7143 -9.7551 -22.055 2.58333 -2.3945
0.00653 0.00109 0.0061 0.00997 0.0136 0.00358 0.0298 0.00698 0.0375
2L:8212920-8213070:minus -33.2959 0.807339 -1.47706 7.12844 -17.3776 -18.1224 -14.0917 7.25 0.12844
0.0279 0.00776 0.0185 0.00118 0.00419 0.00988 0.0074 0.000882 0.0179
2L:8212920-8213070:plus -21.7755 -3.81651 15.0826 7.21101 -27.2347 -17.9388 -19.3303 0.138889 2.58716
0.00241 0.0322 1.18e-06 0.0011 0.0283 0.00738 0.0184 0.016 0.00779
2L:821340-821490:minus -23.1837 1.09174 0.357798 -2.22936 -9.23469 -8.57143 -3.19266 -0.486111

0.963303 0.00553 0.007 0.0103 0.0552 0.00277 0.00149 0.000598 0.0194 0.0145
2L:821340-821490:plus -32.6327 -0.238532 3.80734 6.41284 20.2041 -17.0102 -21.9174 1.19444 3.36697
0.0212 0.0112 0.0029 0.00189 3.64e-07 0.00453 0.0291 0.0114 0.00607
2L:8218400-8218550:minus -21.2551 3.90816 7.6055 7.00917 -27.0816 -17.5714 -8.87156 1.5 3.3211
0.00191 0.00102 0.000539 0.00126 0.0243 0.00616 0.0025 0.0103 0.00616
2L:8218400-8218550:plus -22.1531 1.05505 10.5321 7.34862 -26.6633 -26.8571 -12.6514 4.68056 3.66972
0.0031 0.00709 0.000103 0.000998 0.0189 0.0288 0.00534 0.00302 0.00514
2L:8220580-8220730:minus -40.1429 13.5306 0.183486 1.99083 -18.3469 -17.0102 -18.7248 2.63889 -3.50459
0.0363 0.0000206 0.0109 0.0144 0.00894 0.00453 0.0167 0.00684 0.0497
2L:8220580-8220730:plus -41.2551 6.50459 0.688073 2.81651 -17.7143 0.122449 -34.2936 1.20833 -1.6055
0.0513 0.000833 0.0092 0.00982 0.00627 0.000312 0.15 0.0113 0.0302
2L:8229640-8229790:minus -32.0408 5.11927 0.651376 3.33028 -18.7143 -0.0204082 -28.3853 5.15278
-4.0367 0.0174 0.00155 0.00932 0.00778 0.0136 0.000411 0.0725 0.00245 0.0562
2L:8229640-8229790:plus -24.0306 4.57798 -1.80734 1.43119 -18.7551 -8.65306 -13.367 6.23611 2.07339
0.00723 0.00193 0.0204 0.0181 0.0144 0.00156 0.00629 0.00148 0.00906
2L:8230640-8230790:minus -30.6633 0.688073 3.15596 3.3578 -18.4592 -19.5 -18.9358 -1.875 5.66055
0.00976 0.0081 0.00374 0.00767 0.0109 0.0199 0.0173 0.0288 0.00227
2L:8230640-8230790:plus -31.551 7.04587 -3.00917 5.49541 -17.6735 1.7551 -15.5138 4.44444 3.45872
0.0134 0.000633 0.0289 0.00355 0.00572 0.0000519 0.00992 0.00334 0.00591
2L:8231060-8231210:minus -32.9286 1.38532 6.37615 6.19266 -17.7551 -8.23469 -24.1651 2.31944 -1.80734
0.025 0.00629 0.000974 0.00216 0.00658 0.000875 0.042 0.00769 0.0316
2L:8231060-8231210:plus -30.7041 -0.00917431 1.30275 5.16514 -8.86735 -8.79592 -26.5963 5.65278
-2.91743 0.00993 0.0103 0.00743 0.00424 0.00173 0.0019 0.0575 0.00195 0.0436
2L:8239860-8240010:minus -12.0714 7.69725 4.33945 3.88991 -27.1939 -18.1939 7.90826 -1.75 4.80734
0.000318 0.00045 0.00234 0.00608 0.0267 0.0101 0.0000244 0.0279 0.00334
2L:8239860-8240010:plus -21.8878 12.9725 -0.66055 4.0367 -18.8265 0.826531 -10.9817 -0.847222
3.63303 0.00258 0.0000227 0.0143 0.00578 0.0149 0.000153 0.00372 0.0216 0.00518
2L:8240080-8240230:minus -12.0714 7.69725 4.33945 3.88991 -27.1939 -26.5612 7.90826 -1.36111 -1.17431
0.000318 0.00045 0.00234 0.00608 0.0267 0.0237 0.0000244 0.025 0.0275
2L:8240080-8240230:plus -31.8571 1.88073 -1.59633 3.59633 -27.1939 -0.204082 -15.578 3.36111
0.697248 0.0163 0.00524 0.0192 0.00693 0.0267 0.000463 0.01 0.00519 0.0154
2L:825880-826030:minus -14.449 13.9725 3.25688 2.41284 -18.1939 -8.72449 -20.5046 -0.0972222
-1.15596 0.0014 8.89e-06 0.0036 0.0118 0.0086 0.00169 0.0225 0.0172 0.0273
2L:825880-826030:plus -4.63265 6.48624 4.90826 7.43119 -17.4184 -27.5612 -6.17431 -3.79167 3.66972
0.000175 0.00084 0.00185 0.000939 0.00457 0.0393 0.00137 0.0469 0.00514
2L:8277700-8277850:minus -22.398 -0.651376 5.65138 4.22018 -27.0408 -7.72449 -9.05505 -3.41667
7.58716 0.00344 0.0128 0.00135 0.00531 0.0242 0.00068 0.00258 0.0428 0.000954
2L:8277700-8277850:plus -24.3673 -1.41284 4.54128 3.62385 -27.7959 0.897959 -18.2936 1.81944 9.55046
0.00791 0.0163 0.00215 0.00673 0.0386 0.000144 0.0156 0.00919 0.000321
2L:8299560-8299710:minus -23.7041 -1.88073 4.89908 3.19266 -8.89796 -26.898 -14.6147 -5.52778 6.95413
0.00669 0.0188 0.00185 0.00829 0.00189 0.0302 0.00829 0.069 0.00136
2L:8299560-8299710:plus -30.2857 -4.20183 -2.50459 3.18349 -18.1939 -17.0816 1.38532 5.33333 11.4771
0.00887 0.0357 0.025 0.00837 0.0086 0.00487 0.000198 0.00226 0.0000679
2L:8300400-8300550:minus -14.0306 -1.11927 2.75229 7.27523 -26.5918 -18.2755 -14.2018 1.51389 3.09174
0.00115 0.0149 0.00436 0.00109 0.0186 0.0112 0.00758 0.0102 0.00678
2L:8300400-8300550:plus -13.1122 4.79817 5.95413 5.57798 -18.6837 -18.3469 -11.1376 10.0833 5.44037
0.000632 0.00177 0.00118 0.0034 0.0126 0.0124 0.00384 0.000154 0.00259
2L:8302860-8303010:minus -41.1735 -0.541284 10.1376 7.29358 -18.7143 -16.9796 -25.3394 3.66667
1.2844 0.0479 0.0124 0.000131 0.00104 0.0136 0.0044 0.0493 0.0046 0.0129
2L:8302860-8303010:plus -22.2245 13.5306 0.715596 5.90826 -16.7143 -27.602 -21.7523 1.80556 -1.92661
0.00325 0.0000206 0.00912 0.00271 0.00319 0.0416 0.0282 0.00923 0.0325
2L:8305520-8305670:minus -33.4082 5.15596 0.12844 -1.38532 -17.7857 -9.72449 -16.8349 7.43056 -2.88991
0.0283 0.00153 0.0111 0.0418 0.00676 0.00352 0.0125 0.000801 0.0433
2L:8305520-8305670:plus -24.2245 11.3028 -0.678899 9.59633 -27.1939 -17.0102 -19.0367 0.458333

-2.36697 0.00763 0.0000693 0.0144 0.000275 0.0267 0.00453 0.0176 0.0145 0.0371
2L:8308220-8308370:minus -5.12245 4.3211 9.30275 7.29358 -9.19388 -17.2755 -22.4679 -1.93056 -2.51376
0.000225 0.00213 0.000213 0.00104 0.00253 0.00544 0.032 0.0293 0.039
2L:8308220-8308370:plus -31.449 13.4592 -1.51376 3.18349 -18.7143 -8.45918 -24.156 -0.791667
-0.33945 0.0123 0.000064 0.0187 0.00837 0.0136 0.00111 0.042 0.0212 0.0209
2L:8311320-8311470:minus -23.4898 -1.01835 4.72477 3.19266 -8.93878 -18.2755 -27.8899 3.375 5.3945
0.00639 0.0144 0.00199 0.00829 0.00216 0.0112 0.0678 0.00516 0.00263
2L:8311320-8311470:plus -22.9592 6.21101 2.48624 3.7156 -8.64286 -8.23469 -10.633 1.25 -0.917431
0.00475 0.000958 0.00483 0.00655 0.00139 0.000875 0.00347 0.0112 0.0256
2L:8320300-8320450:minus -34.1122 -0.87156 2.3578 4.9633 -8.89796 -26.8571 -8.3211 6.93056 1.79817
0.0312 0.0138 0.00506 0.0044 0.00189 0.0288 0.00225 0.00104 0.0102
2L:8320300-8320450:plus -13.7857 1.19266 10.5596 3.70642 -17.9796 -17.2755 -22.6239 2.34722 -0.311927
0.00108 0.00675 0.000102 0.0066 0.00726 0.00544 0.0329 0.00761 0.0208
2L:8326360-8326510:minus -13.449 2.19266 2.05505 7.27523 -27.1939 -18.3469 -12.7615 0.333333 5.79817
0.000914 0.00468 0.00566 0.00109 0.0267 0.0124 0.00547 0.0151 0.00208
2L:8326360-8326510:plus -30.449 0.93578 -0.220183 6.09174 -17.5306 -7.79592 -16.4495 0.861111
1.01835 0.00925 0.00741 0.0125 0.00235 0.0054 0.000723 0.0117 0.0127 0.0142
2L:833340-833490:minus -22.2959 1.47706 2.37615 0.587156 -18.1939 -9.27551 -13.2752 2.43056 1.48624
0.00335 0.00608 0.00503 0.0231 0.0086 0.00244 0.00616 0.00738 0.0118
2L:833340-833490:plus -31.7041 2.36697 0.165138 4.13761 -18.7143 -9.45918 -22.8257 -3.25 3.56881
0.0146 0.00439 0.011 0.00546 0.0136 0.00279 0.0341 0.0411 0.00543
2L:8342460-8342610:minus -32.5102 1.76147 1.61468 3.33945 -17.4184 -28.8571 -6.69725 3.66667 6.17431
0.0202 0.00548 0.00665 0.00774 0.00457 0.0699 0.00157 0.0046 0.00171
2L:8342460-8342610:plus -32.2143 5.19266 8.48624 6.00917 -17.7857 -17.2755 -19.0734 -2.44444 1.46789
0.0182 0.0015 0.000336 0.00255 0.00676 0.00544 0.0177 0.0335 0.0119
2L:8343220-8343370:minus -42.8469 0.770642 -0.834862 5.31193 -28.5612 9.08163 -20.578 1.97222
-4.33945 0.0869 0.00786 0.0152 0.00393 0.0617 0.0000431 0.0228 0.0087 0.0609
2L:8343220-8343370:plus -32.4694 -2.41284 2.9633 3.43119 -18.2245 -36.1531 -14.2385 -3.47222 2.26606
0.0196 0.0219 0.00403 0.00749 0.00877 0.0908 0.00765 0.0434 0.00847
2L:8346440-8346590:minus -34 -3.76147 2.42202 7.73394 -17.6735 0.0204082 -21.7523 0.652778 -8.63303
0.0307 0.0317 0.00495 0.000669 0.00572 0.000377 0.0282 0.0136 0.155
2L:8346440-8346590:plus -22.7041 -1.68807 2 9.08257 -8.30612 -9.53061 -1.57798 10.6806 -4.08257
0.00395 0.0177 0.00578 0.000444 0.00097 0.0031 0.000403 0.0000985 0.0567
2L:8362340-8362490:minus -24.551 9.81651 -1.6789 2.00917 -18.5204 -8.5 -15.055 1.04167 -0.550459
0.00814 0.000147 0.0196 0.0142 0.0117 0.00126 0.00907 0.012 0.0225
2L:8362340-8362490:plus -21.8571 0.357798 5.47706 6.90826 -18.1939 -27.0408 -2.76147 1.70833 7.63303
0.00249 0.00911 0.00145 0.00133 0.0086 0.0307 0.000534 0.00955 0.000928
2L:8364680-8364830:minus -24.2653 2.05505 3.50459 2.41284 -18.4184 -27.2653 -14.6239 -3.31944 -0.275229
0.00773 0.00492 0.00326 0.0118 0.0101 0.0333 0.0083 0.0418 0.0206
2L:8364680-8364830:plus -24.449 1.34862 -1.87156 5.69725 10.3571 -17.051 -0.669725 4.90278
-1.33028 0.00802 0.00637 0.0208 0.00314 0.0000208 0.00479 0.00033 0.00274 0.0285
2L:8365720-8365870:minus -40.9898 1 0.0183486 3.11009 -27.2653 -18.2755 -26.4587 4.08333 -3.88073
0.0455 0.00724 0.0115 0.00862 0.0302 0.0112 0.0566 0.00388 0.0539
2L:8365720-8365870:plus -31.6633 6.85321 0.0366972 2.57798 -17.6429 -27.6735 -16.2752 -3.33333
-0.220183 0.0141 0.000701 0.0115 0.011 0.00562 0.0443 0.0114 0.042 0.0201
2L:8366420-8366570:minus -40.0714 0.311927 -2.29358 2.09174 0.877551 -17.2755 -15.055 4.19444 2.66972
0.0359 0.00925 0.0236 0.0137 0.00019 0.00544 0.00907 0.00371 0.00764
2L:8366420-8366570:plus -31.2959 9.37615 1.87156 3.18349 -17.4184 -17.051 -24.5872 2.29167 1.90826
0.0113 0.000184 0.00606 0.00837 0.00457 0.00479 0.0446 0.00776 0.00974
2L:8370380-8370530:minus -41.7755 2.56881 -0.275229 3.18349 -18.9796 -17.0816 -8.75229 11.3333
3.09174 0.0652 0.00407 0.0127 0.00837 0.0155 0.00487 0.00244 0.0000573 0.00678
2L:8370380-8370530:plus -12.1531 4.41284 5.40367 10.5963 -27.1939 -9.20408 -0.688073 -5.23611
4.07339 0.000352 0.00205 0.0015 0.0001 0.0267 0.00225 0.000331 0.0649 0.00417
2L:8374380-8374530:minus -33.7347 14.0275 0.908257 1.63303 -18.3776 -10.0204 -10.1193 -0.0555556

7.94495 0.0294 8.23e-06 0.00853 0.0167 0.00926 0.00399 0.00315 0.017 0.000667
2L:8374380-8374530:plus -22.9898 13.4592 5.51376 2.86239 -8.67347 -8.86735 -20.1284 -0.194444
0.963303 0.00482 0.000064 0.00143 0.00954 0.00154 0.00198 0.0211 0.0178 0.0145
2L:8376900-8377050:minus -24.4796 3.97959 -2.70642 6.29358 0.540816 -7.94898 -13.9633 2.54167 -6.62385
0.00804 0.000669 0.0265 0.00202 0.000321 0.00074 0.0072 0.00709 0.104
2L:8376900-8377050:plus -24.3265 10.6147 8.55963 3.43119 -27.6327 -17.2041 -6.70642 -5.25 5.55046
0.00784 0.0000977 0.000322 0.00749 0.0369 0.00507 0.00157 0.0651 0.00241
2L:8381600-8381750:minus -24.2653 1.49541 4.22018 7.90826 -18.4184 -18.051 7.36697 0.75 -2.63303
0.00773 0.00604 0.00245 0.000611 0.0101 0.00886 0.0000281 0.0132 0.0404
2L:8381600-8381750:plus -23.7857 15.3394 -1.68807 1.79817 -27.3061 0.530612 -29.6972 3.44444 3.16514
0.00679 1.34e-06 0.0197 0.0157 0.0315 0.000203 0.0871 0.00502 0.00656
2L:8382780-8382930:minus -23.5204 2.52294 13.5872 3.90826 -8.89796 -9.23469 -14.1009 -1.65278 -2.83486
0.00645 0.00414 0.0000122 0.00603 0.00189 0.00238 0.00742 0.0271 0.0427
2L:8382780-8382930:plus -12.0816 -1.63303 2.13761 2.66972 -9.27551 -18.0102 -13.4037 3.54167 2.75229
0.000321 0.0174 0.00549 0.0106 0.00284 0.00812 0.00634 0.00483 0.00748
2L:8384000-8384150:minus -20.6939 9.97248 -3.00917 7.34862 -7.60204 1.02041 1.63303 2.61111 -3.61468
0.00174 0.000135 0.0289 0.000998 0.000466 0.000118 0.000184 0.00691 0.0509
2L:8384000-8384150:plus -31.7041 10.3211 -2.44037 1.02752 -8.67347 -8.5 -13.0642 1.93056 10.0367
0.0146 0.000114 0.0246 0.0204 0.00154 0.00126 0.00587 0.00883 0.000146
2L:8384340-8384490:minus -20.6939 9.97248 -1.59633 7.34862 -7.60204 1.02041 1.63303 2.61111 -2.26606
0.00174 0.000135 0.0192 0.000998 0.000466 0.000118 0.000184 0.00691 0.0359
2L:8384340-8384490:plus -31.6327 4.25688 4.38532 4.33945 -26.1939 -9.45918 -22.7706 -5.84722 5.58716
0.0138 0.00218 0.0023 0.00509 0.0175 0.00279 0.0338 0.0738 0.00237
2L:8385680-8385830:minus -41.4388 2.17431 2.40367 4.13761 -26.9694 -8.79592 -17.6697 9.02778 4.56881
0.0562 0.00471 0.00498 0.00546 0.023 0.0019 0.0142 0.000315 0.00356
2L:8385680-8385830:plus -31.2857 7.61468 -3.76147 5.7156 -18.6837 -18.0816 -11.1009 2.16667 3.11009
0.0113 0.00047 0.0356 0.00309 0.0126 0.00934 0.00381 0.00812 0.00669
2L:8389580-8389730:minus -41.4796 2.87156 5.3211 -2.48624 -26.9694 -0.540816 -0.40367 6.43056
3.56881 0.0577 0.00365 0.00155 0.06 0.023 0.00054 0.000311 0.00134 0.00543
2L:8389580-8389730:plus -22.2245 -4.55963 3.34862 -0.321101 -18.4592 -27.8265 -5.72477 7.94444
9.56881 0.00325 0.0393 0.00347 0.0294 0.0109 0.0498 0.00122 0.000602 0.000303
2L:8400260-8400410:minus -33.7755 0.990826 0.926606 2.19266 -27.5306 -8.94898 -24.1193 7.18056 -2.02752
0.0297 0.00726 0.00848 0.0131 0.0351 0.00203 0.0418 0.000915 0.0335
2L:8400260-8400410:plus -30.6735 13.5306 2.94495 6.18349 10.3878 -8.42857 -28.211 5.125 -8.82569
0.00978 0.0000206 0.00405 0.00217 0.0000174 0.00104 0.0708 0.00248 0.161
2L:8403460-8403610:minus -31.8878 10.0917 3.66972 2.84404 -18.0816 -9.27551 -9.17431 2.48611 -1.01835
0.0164 0.000128 0.00306 0.00961 0.00759 0.00244 0.00264 0.00723 0.0263
2L:8403460-8403610:plus -21.8163 -1.6422 -0.926606 4.43119 10.9184 -16.051 -12.1101 -1.47222
-5.99083 0.00244 0.0175 0.0156 0.00495 4.19e-06 0.00408 0.00472 0.0258 0.0886
2L:840820-840970:minus -31.6327 -0.146789 -1.15596 -1.00917 -27.1939 -19.0102 -2.55963 -3.08333
5.6055 0.0138 0.0108 0.0168 0.0368 0.0267 0.0159 0.000508 0.0395 0.00232
2L:840820-840970:plus -33.0714 -4.63303 11.1651 3.43119 -18.1531 -19.2041 -13.9174 2.55556 -0.541284
0.0267 0.04 0.000072 0.00749 0.00829 0.0179 0.00712 0.00705 0.0223
2L:8411640-8411790:minus -31.7755 -1.21101 6.6789 9.23853 -8.93878 -17.7857 -4.17431 -6.47222 8.10092
0.0157 0.0153 0.000844 0.000424 0.00216 0.0068 0.000784 0.0836 0.000623
2L:8411640-8411790:plus -30.7041 4.55963 7.92661 5.3945 -17.3776 -18.1531 -14.9541 -1.15278 8.11009
0.00993 0.00194 0.000455 0.00379 0.00419 0.00997 0.00889 0.0236 0.000597
2L:841260-841410:minus -31.4082 4.78899 -3.12844 4.22018 -18.9796 -17.2755 -22.1468 -0.305556
-5.81651 0.0119 0.00177 0.0299 0.00531 0.0155 0.00544 0.0303 0.0184 0.0855
2L:841260-841410:plus -32.3673 1.77064 1.12844 2.26606 -17.4898 -8.02041 -19.7064 5.625 3.15596
0.0189 0.00546 0.00791 0.0126 0.0053 0.000761 0.0196 0.00198 0.00659
2L:8415180-8415330:minus -13.3776 -1.6789 2.56881 3.3945 -8.16327 1.05102 -9.77982 -4.97222 4.98165
0.000831 0.0177 0.00468 0.0076 0.000873 0.000107 0.00295 0.0613 0.00313
2L:8415180-8415330:plus -41.7347 -1.94495 -1.57798 -2.11927 -17.9388 -18.4184 -13.4771 3.27778 -0.541284

0.0643 0.0191 0.019 0.0532 0.00715 0.0127 0.00645 0.00536 0.0223
2L:8415680-8415830:minus 5.5102 -2.01835 -0.46789 5.59633 -18.4898 0.979592 -21.055 2.16667 4.66055
9.3e-06 0.0195 0.0135 0.00331 0.0116 0.000136 0.0248 0.00812 0.0035
2L:8415680-8415830:plus -14.5918 -0.183486 5.85321 1.85321 -26.9694 -8.94898 3.34862 1.88889
9.30275 0.00148 0.011 0.00123 0.0153 0.023 0.00203 0.000104 0.00896 0.00038
2L:8416400-8416550:minus -30.4694 -1.94495 -1.98165 0.504587 -18.1531 -27.6735 -14.3211 3.40278 1.73394
0.00926 0.0191 0.0215 0.0235 0.00829 0.0443 0.00778 0.00511 0.0106
2L:8416400-8416550:plus -23.0714 1.58716 -1.75229 6.11009 -8.16327 -18.051 -8.12844 1.09722 1.44954
0.0052 0.00584 0.0201 0.00233 0.000873 0.00886 0.00216 0.0118 0.0119
2L:841680-841830:minus -32.2551 0.917431 1.54128 1.6789 -18.7551 -8.72449 -23.7706 1.80556 3.47706
0.0184 0.00746 0.00683 0.0165 0.0144 0.00169 0.0397 0.00923 0.00575
2L:841680-841830:plus -32.2245 -0.192661 -0.715596 5.30275 -7.89796 -9.23469 -11.0275 4.84722
2.6789 0.0183 0.011 0.0146 0.00396 0.000678 0.00238 0.00375 0.00281 0.00763
2L:8416960-8417110:minus -30.2551 2.6422 2.94495 4.94495 -8.37755 -27.0408 -11.4954 1.45833 -0.302752
0.00885 0.00397 0.00405 0.00444 0.00106 0.0307 0.00413 0.0104 0.0208
2L:8416960-8417110:plus -14.3367 -4.00917 -0.0275229 4.49541 -18.7449 -18.051 -2.29358 -0.277778
5.13761 0.00131 0.0339 0.0117 0.00487 0.0138 0.00886 0.000476 0.0182 0.00298
2L:8418360-8418510:minus -24.2245 -0.908257 4.73394 4.59633 -17.8571 -16.5 -13.2018 4.38889
-2.3211 0.00763 0.0139 0.00199 0.00478 0.00698 0.00416 0.00605 0.00342 0.0365
2L:8418360-8418510:plus -13.5918 -1.14679 9.41284 4.9633 -26.898 -7.79592 -5.00917 4 7.42202
0.00099 0.015 0.000199 0.0044 0.0203 0.000723 0.000995 0.00402 0.00106
2L:8419620-8419770:minus -32.3673 4.33945 9.7156 7.2844 -7.67347 -27.449 -14.1101 5.13889 3.76147
0.0189 0.00211 0.000168 0.00105 0.00055 0.0357 0.00743 0.00247 0.00494
2L:8419620-8419770:plus -13.1429 5.3211 2.48624 9.41284 -17.8163 -9.45918 -7.97248 -1.52778 0.752294
0.00065 0.00142 0.00483 0.000354 0.00682 0.00279 0.00209 0.0262 0.0152
2L:8421220-8421370:minus -31.5102 -0.376147 -2.15596 4.34862 -28.2347 -18.2755 -14.1101 4.09722
2.0367 0.013 0.0117 0.0226 0.00507 0.051 0.0112 0.00743 0.00386 0.00923
2L:8421220-8421370:plus -31.4796 1.93578 -0.422018 3.49541 -28.2347 0.72449 -7.02752 6.5 5.79817
0.0128 0.00514 0.0133 0.00716 0.051 0.000175 0.0017 0.0013 0.00208
2L:8421460-8421610:minus -31.2551 0.192661 -0.256881 3.53211 -27.3367 -26.6327 -9.76147 7.13889
1.33028 0.0113 0.00965 0.0126 0.00704 0.0317 0.0251 0.00294 0.000935 0.0127
2L:8421460-8421610:plus -31.4796 1.93578 -0.422018 2.69725 -28.2347 -0.173469 -7.02752 6.5
5.79817 0.0128 0.00514 0.0133 0.0104 0.051 0.000456 0.0017 0.0013 0.00208
2L:8424080-8424230:minus -22.7449 6.50459 0.614679 6.12844 -17.3776 -27.4898 -8.29358 5.20833 5.38532
0.00404 0.000833 0.00943 0.00231 0.00419 0.0363 0.00223 0.00239 0.00267
2L:8424080-8424230:plus -14.4898 2.87156 7.98165 1.90826 -17.449 -0.469388 -19.2569 5.18056
-0.59633 0.00143 0.00365 0.000442 0.015 0.00484 0.000533 0.0182 0.00242 0.0229
2L:8426240-8426390:minus -32.7755 3.90816 -0.12844 4.75229 -18.7449 -18.0102 -10.8165 -1.29167 1.90826
0.0234 0.00102 0.0121 0.00461 0.0138 0.00812 0.0036 0.0245 0.00974
2L:8426240-8426390:plus -13.8163 1.22018 -1.47706 -0.311927 -9.23469 0.0204082 -24.7982 4.45833
-3.40367 0.00109 0.00668 0.0185 0.0294 0.00277 0.000377 0.0459 0.00332 0.0486
2L:8433700-8433850:minus -22.1735 -2.15596 5.3945 9.92661 -17.1224 -7.45918 -12.7706 -1.04167 -0.449541
0.0031 0.0203 0.00151 0.000169 0.00343 0.000582 0.00549 0.0228 0.0216
2L:8433700-8433850:plus -32.0714 6.09174 4.76147 3.88991 -17.9796 -9.02041 -15.0275 4.68056 2.0367
0.0176 0.00101 0.00196 0.00608 0.00726 0.00213 0.00902 0.00302 0.00923
2L:8437300-8437450:minus -12.7347 4.52294 7.6055 7.00917 -18.449 -26.602 -10.8257 -3.77778 -1.75229
0.000468 0.00197 0.000539 0.00126 0.0107 0.0248 0.00361 0.0467 0.0312
2L:8437300-8437450:plus -30.7755 0.733945 12.945 6.21101 -17.6429 -19.5 -16.9083 -1.52778 2.49541
0.0102 0.00797 0.0000218 0.00213 0.00562 0.0199 0.0126 0.0262 0.00794
2L:8438120-8438270:minus -32.7041 -0.247706 -1.3211 1.69725 -27.602 -9.72449 -9 2.23611 1.16514
0.0222 0.0112 0.0176 0.0163 0.0364 0.00352 0.00256 0.00792 0.0135
2L:8438120-8438270:plus -23.7857 1.86239 15.6606 1.05505 -27.3061 -0.244898 -20.8349 10.4722
6.95413 0.00679 0.00528 3.94e-07 0.0204 0.0315 0.000496 0.0239 0.000115 0.00136
2L:8449480-8449630:minus -21.7347 7.43119 15.6972 4.0367 -18.5204 -18.2755 -13.3303 -7 6.95413

0.00235 0.000517 3.11e-07 0.00578 0.0117 0.0112 0.00624 0.0926 0.00136
2L:8449480-8449630:plus -22.9286 5 -4.01835 2.42202 -27.6429 -27.4898 -10.2936 7.44444 -0.449541
0.0046 0.00163 0.0382 0.0118 0.037 0.0363 0.00325 0.000795 0.0216
2L:846060-846210:minus -23.2653 0.770642 0.00917431 6.11009 -26.9388 -9.5 -13.4495 2.81944 1.86239
0.00593 0.00786 0.0116 0.00233 0.0215 0.00294 0.00641 0.00639 0.00994
2L:846060-846210:plus -14.1837 3.90816 2.74312 5.90826 -26.9286 -26.7857 -13.2294 4.63889 2.75229
0.00124 0.00102 0.00438 0.00271 0.0214 0.0266 0.00609 0.00307 0.00748
2L:8462180-8462330:minus -31.8469 1.13761 1.20183 2.11927 -17.7857 -9.53061 -16.1651 9.5 1.27523
0.0162 0.00689 0.0077 0.0135 0.00676 0.0031 0.0112 0.000231 0.013
2L:8462180-8462330:plus 6.14286 0.917431 -2.0367 9.44954 -18.449 -17.0816 -3.3211 2.80556 6.26606
5.85e-06 0.00746 0.0219 0.000326 0.0107 0.00487 0.000618 0.00642 0.00162
2L:8463900-8464050:minus -3.7449 2.01835 2.06422 7.27523 -18.449 1.27551 -6.40367 5.125 7.84404
0.000104 0.00499 0.00564 0.00109 0.0107 0.0000717 0.00146 0.00248 0.000701
2L:8463900-8464050:plus -39.2551 1.23853 1.55963 3.25688 -17.9388 -17.2755 -12.3394 0.569444 -2.01835
0.0331 0.00664 0.00678 0.00804 0.00715 0.00544 0.00497 0.014 0.0334
2L:8464420-8464570:minus -32.7041 -0.0733945 0.348624 4.0367 -17.9796 1.2449 -22.7706 0.222222
0.963303 0.0222 0.0106 0.0103 0.00578 0.00726 0.0000797 0.0338 0.0156 0.0145
2L:8464420-8464570:plus -13.6327 13.4592 -0.752294 3.77064 -28 -9.5 -16.7156 1.88889 -2.75229
0.00101 0.000064 0.0148 0.00637 0.0444 0.00294 0.0122 0.00896 0.0418
2L:847160-847310:minus -23.9286 3.40367 2.58716 7 -7.86735 -7.5 -19.367 -0.513889 -3.77982
0.00701 0.003 0.00465 0.00132 0.000617 0.000614 0.0185 0.0196 0.0525
2L:847160-847310:plus -40.8163 -2.21101 7.84404 9.33028 -17.7857 -17.3469 -25.7615 12.3333 -2.01835
0.0436 0.0206 0.000476 0.000374 0.00676 0.0058 0.0519 0.0000221 0.0334
2L:847680-847830:minus -22.7347 0.724771 4.77982 0.559633 -27.2347 -19.1531 -10.6881 1.95833 6.73394
0.00403 0.008 0.00195 0.0232 0.0283 0.0177 0.00351 0.00875 0.00143
2L:847680-847830:plus -31.8776 2.40367 6.91743 3.04587 -27.6735 -28.4796 -10.7156 2 1.27523 0.0163
0.00433 0.000755 0.00883 0.0375 0.0617 0.00353 0.00862 0.013
2L:8485660-8485810:minus -33.3673 4.41284 1.61468 3.24771 -27.4592 -26.6327 -16.2844 -0.111111
-0.0275229 0.0282 0.00205 0.00665 0.00808 0.0329 0.0251 0.0114 0.0173 0.0186
2L:8485660-8485810:plus -31.0408 2.52294 1.26606 1.63303 0.571429 -0.244898 -21.2202 1.20833
-1.36697 0.0106 0.00414 0.00753 0.0167 0.0003 0.000496 0.0256 0.0113 0.0286
2L:8488860-8489010:minus -33.2245 -2.14679 -0.238532 2.33028 -28.2959 -18.5 -17.1009 1.75 -1.13761
0.0276 0.0203 0.0125 0.0123 0.0541 0.0131 0.013 0.00941 0.0271
2L:8488860-8489010:plus -32.9592 8.05505 13.6422 3.44037 -18.051 0.244898 3.57798 -0.472222
-2.26606 0.0252 0.000371 0.0000116 0.00746 0.00746 0.000285 0.0000962 0.0193 0.0359
2L:851000-851150:minus -12 -4.07339 5.89908 4.40367 10.9898 -9.0102 -0.385321 -2.13889 4.02752
0.000305 0.0345 0.00121 0.00497 3.28e-06 0.00208 0.000309 0.0309 0.00428
2L:851000-851150:plus -32.7347 -4 -2.9633 -1.29358 -17.1837 -19.3776 -10.0642 4.69444 2.78899 0.0225
0.0338 0.0285 0.0403 0.00366 0.0195 0.00311 0.003 0.00745
2L:8511840-8511990:minus -13.1531 14.156 -0.40367 2.19266 -27.1939 -9.5 -12.2661 7.94444 -1.70642
0.00067 6.98e-06 0.0132 0.0131 0.0267 0.00294 0.00489 0.000602 0.0309
2L:8511840-8511990:plus -23.3776 13.5306 -4.0367 2.77064 -27.8673 -8.94898 -20.422 1.09722 -0.201835
0.00619 0.0000206 0.0384 0.01 0.0391 0.00203 0.0222 0.0118 0.0199
2L:8514900-8515050:minus -30.7755 -2.18349 9.31193 2.29358 -17.0816 0.204082 -9.43119 1.25 -8.14679
0.0102 0.0205 0.000211 0.0124 0.00334 0.000296 0.00277 0.0112 0.14
2L:8514900-8515050:plus -13.2551 13.3878 9.11009 3.42202 -8.96939 -18.0102 -5.45872 0.736111 0.440367
0.000751 0.0000869 0.000238 0.00751 0.00226 0.00812 0.00113 0.0132 0.0164
2L:8517420-8517570:minus -24.5918 -1.22018 11.5138 1.81651 -18.7143 1.12245 -16.7339 -1.30556 -2.52294
0.00821 0.0154 0.0000585 0.0156 0.0136 0.000085 0.0123 0.0246 0.0391
2L:8517420-8517570:plus -23.1939 7.24771 14.9633 7.29358 -18.7551 -9.72449 -14.6055 -1.36111 -0.311927
0.00558 0.00057 1.77e-06 0.00104 0.0144 0.00352 0.00827 0.025 0.0208
2L:852740-852890:minus -21.7041 2.69725 -0.348624 6.84404 -18.449 -10.0204 -15.7248 -2.26389
-3.3945 0.00231 0.00389 0.013 0.0014 0.0107 0.00399 0.0103 0.032 0.0484
2L:852740-852890:plus -14.602 5.98165 0.93578 3.04587 -17.4898 -8.5 -4.33945 -3 0.990826 0.00149

0.00107 0.00845 0.00883 0.0053 0.00126 0.000822 0.0386 0.0143
2L:8528560-8528710:minus -21.9184 13.3878 2.66055 5.57798 -18.2245 -18.0102 -16.2569 -2.55556 -0.293578
0.00261 0.0000869 0.00452 0.0034 0.00877 0.00812 0.0113 0.0345 0.0207
2L:8528560-8528710:plus -24.2959 5.84404 3.6789 3.05505 -27.5306 1.12245 -15.6422 5.36111 0.788991
0.00781 0.00113 0.00305 0.00878 0.0351 0.000085 0.0102 0.00223 0.0151
2L:8529040-8529190:minus -23 1.15596 3.22936 5.34862 -8.60204 -9.23469 -9.22936 1.20833 0.229358
0.00498 0.00684 0.00363 0.00387 0.00125 0.00238 0.00267 0.0113 0.0173
2L:8529040-8529190:plus -22.0408 -0.211009 1.49541 9.08257 -27 -9.23469 -8.52294 -5.86111 0.394495
0.00296 0.0111 0.00694 0.000444 0.0233 0.00238 0.00234 0.074 0.0166
2L:8535760-8535910:minus -33.5612 -1.15596 -2.33028 1.89908 -28.602 -8.87755 -14.8991 2.70833 0.963303
0.0288 0.0151 0.0238 0.0151 0.0631 0.00198 0.00879 0.00666 0.0145
2L:8535760-8535910:plus -30.1735 1.26606 8.74312 0.00917431 -16.449 -27.9694 -0.954128 6.94444
5.55046 0.00869 0.00657 0.000291 0.0269 0.00307 0.0547 0.000351 0.00103 0.00241
2L:8543240-8543390:minus -40.7755 1.44954 1.70642 -1.08257 -18.7449 -17.5714 -25.633 2.90278 -2.51376
0.0433 0.00615 0.00644 0.0378 0.0138 0.00616 0.0511 0.00619 0.039
2L:8543240-8543390:plus -21.7449 2.17431 0.706422 7.27523 -17.8163 -17.3469 -19.3119 3.5 3.40367
0.00236 0.00471 0.00914 0.00109 0.00682 0.0058 0.0183 0.00491 0.00603
2L:85920-86070:minus -32.0408 -1.94495 2.44954 6.00917 -27.1531 -7.79592 -22.1193 3.15278 5.86239
0.0174 0.0191 0.00489 0.00255 0.0249 0.000723 0.0301 0.00563 0.00201
2L:85920-86070:plus -22.9184 1.37615 -1.84404 4.34862 -26.9694 -9.53061 -8.81651 1.63889 -2.7156
0.00447 0.00631 0.0206 0.00507 0.023 0.0031 0.00247 0.00978 0.0412
2L:8619820-8619970:minus -24.102 -1.7156 4.27523 5.30275 -17.5306 -27.7857 -6.63303 8.58333 7.45872
0.00733 0.0179 0.0024 0.00396 0.0054 0.0466 0.00154 0.000415 0.00103
2L:8619820-8619970:plus -31.5204 5.89908 -1.34862 6.12844 -8.33673 -9.64286 -22.3119 -4.31944 3.57798
0.0131 0.00111 0.0178 0.00231 0.00101 0.00339 0.0312 0.053 0.00536
2L:865320-865470:minus -32.2143 0.743119 3.53211 6.41284 -18.4898 -18.2755 -20.2202 -0.430556
-0.889908 0.0182 0.00794 0.00323 0.00189 0.0116 0.0112 0.0214 0.0191 0.0253
2L:865320-865470:plus -31.1735 2.09174 -2.45872 5.88073 -17.4082 -18.898 -13.1927 0.0138889 0.862385
0.0109 0.00485 0.0247 0.00275 0.00421 0.0152 0.00604 0.0167 0.0148
2L:865760-865910:minus -41.2245 -0.165138 -0.862385 -2.87156 -8.71429 -9.27551 -25.0734 1.26389
8.20183 0.0497 0.0109 0.0153 0.0668 0.00159 0.00244 0.0476 0.0111 0.000539
2L:865760-865910:plus -12.398 -2.41284 5.98165 7.02752 -18.1122 -17.9388 -16.4037 -2.75 3.17431
0.000403 0.0219 0.00116 0.00122 0.00771 0.00738 0.0116 0.0363 0.00653
2L:866400-866550:minus -31.5204 2.20183 2.52294 2.54128 -26.2347 0.316327 -16.7064 4.47222 -0.293578
0.0131 0.00466 0.00476 0.0112 0.0177 0.000243 0.0122 0.0033 0.0207
2L:866400-866550:plus -31.7041 0.678899 5.11009 2.6422 -17.5306 -26.602 -18.055 -1.29167 3.15596
0.0146 0.00813 0.0017 0.0107 0.0054 0.0248 0.0151 0.0245 0.00659
2L:866940-867090:minus -32.3571 -3.14679 1.56881 1.53211 -25.9694 -17.5714 -14.5413 0.472222 -2
0.0187 0.0268 0.00676 0.0174 0.017 0.00616 0.00816 0.0144 0.0332
2L:866940-867090:plus -21.5918 -1.22936 6.69725 1.25688 -17.3061 -27.3061 -10.0092 1.55556 -2.17431
0.00209 0.0154 0.000836 0.0192 0.00389 0.0336 0.00308 0.0101 0.035
2L:8685880-8686030:minus -13.5612 1.31193 14.5138 5.06422 -17.9286 -19.1531 -16.2661 0.347222 -3.0367
0.000984 0.00646 3.77e-06 0.00433 0.0071 0.0177 0.0114 0.015 0.045
2L:8685880-8686030:plus -30.2143 7.2844 13.5046 -0.458716 -17.7143 -19.3469 -10.5138 1.43056
1.86239 0.0088 0.000559 0.000013 0.0308 0.00627 0.0194 0.00339 0.0105 0.00994
2L:8686140-8686290:minus -13.5612 1.31193 14.5138 5.06422 -17.9286 -26.8265 -16.2661 -0.625 3.88073
0.000984 0.00646 3.77e-06 0.00433 0.0071 0.0285 0.0114 0.0202 0.00475
2L:8686140-8686290:plus -30.2143 7.2844 13.5046 -0.458716 -17.7143 -27.5612 -10.5138 9.5 -2.90826
0.0088 0.000559 0.000013 0.0308 0.00627 0.0393 0.00339 0.000231 0.0435
2L:8690560-8690710:minus -22.2245 -0.697248 3.75229 7 -17.9796 -8.23469 -16.8716 5.52778 1.45872
0.00325 0.013 0.00296 0.00132 0.00726 0.000875 0.0126 0.00207 0.0119
2L:8690560-8690710:plus -13.7143 4.37615 8.29358 4.46789 -8.97959 0.0510204 -0.908257 1.61111
0.550459 0.00105 0.00208 0.000375 0.0049 0.0023 0.000349 0.000348 0.00988 0.0161
2L:8691220-8691370:minus -21.9286 0.577982 4.80734 10.5963 -18.7143 -9.30612 -7.27523 6.20833 1.08257

0.0027 0.00843 0.00193 0.0001 0.0136 0.00257 0.0018 0.0015 0.0139
2L:8691220-8691370:plus -30.7755 -1.48624 2.80734 2.93578 -8.53061 -9.60204 -20.0826 1.375 -5.12844
0.0102 0.0167 0.00427 0.00927 0.0011 0.00334 0.0209 0.0107 0.0748
2L:8692500-8692650:minus -31.8163 0.192661 7.26606 3.01835 -18.4184 -7.72449 3.86239 -0.777778
1.79817 0.016 0.00965 0.000638 0.0089 0.0101 0.00068 0.0000868 0.0211 0.0102
2L:8692500-8692650:plus -23.449 0.33945 1.72477 3.50459 -17.9796 -18.2755 -16.9174 6.79167 -2.58716
0.00631 0.00917 0.0064 0.00713 0.00726 0.0112 0.0126 0.00112 0.0397
2L:8694540-8694690:minus -22.0816 6.22936 1.66972 6.3578 -17.7143 -9.45918 -11.7523 -3.01389 -8.20183
0.003 0.000949 0.00652 0.00195 0.00627 0.00279 0.00437 0.0388 0.141
2L:8694540-8694690:plus -40.1837 2.25688 -3.21101 -2.87156 -27.3469 -17.7551 -22.4679 4.68056 8.05505
0.0367 0.00457 0.0306 0.0668 0.0318 0.00643 0.032 0.00302 0.00064
2L:869800-869950:minus -24.4184 0.559633 4.55046 5.46789 1.87755 -26.6327 -15.7339 -1.81944 -0.981651
0.00797 0.00848 0.00214 0.00362 0.0000492 0.0251 0.0103 0.0284 0.026
2L:869800-869950:plus -32.3776 7.97248 4.31193 -1.77982 -17.5306 -0.244898 -27.0183 6.06944 -1.56881
0.0189 0.000387 0.00236 0.0474 0.0054 0.000496 0.0606 0.0016 0.0298
2L:8700020-8700170:minus -31.8061 -1.30275 5.55046 3.17431 -9.5 -9.7551 -6.75229 5.65278 1.86239
0.0158 0.0158 0.00141 0.00842 0.00293 0.00358 0.00159 0.00195 0.00994
2L:8700020-8700170:plus -22.398 0.678899 -2.15596 5.59633 -9.45918 -18.4898 -6.94495 4.69444 3.08257
0.00344 0.00813 0.0226 0.00331 0.00287 0.0129 0.00167 0.003 0.00681
2L:8700780-8700930:minus -33.0408 -0.477064 2.09174 5.48624 -18.4184 -26.2653 -16.3028 4.30556
1.79817 0.0264 0.0121 0.00558 0.00357 0.0101 0.0218 0.0114 0.00354 0.0102
2L:8700780-8700930:plus -14.2653 4.16514 3.54128 6.06422 -28.2347 -9.16327 -14.3578 0.25 1.06422
0.00128 0.00226 0.00322 0.00239 0.051 0.00223 0.00785 0.0155 0.014
2L:8701240-8701390:minus -30.2857 3.64286 5.46789 7.17431 -18.7143 -17.7857 -5.3945 -4.51389 1.99083
0.00887 0.00159 0.00146 0.00115 0.0136 0.0068 0.00111 0.0554 0.00946
2L:8701240-8701390:plus -23.5306 0.440367 3.30275 5.48624 -18.4184 -18.6429 -4.78899 7.44444 1.63303
0.00646 0.00885 0.00353 0.00357 0.0101 0.0139 0.000935 0.000795 0.0112
2L:8702260-8702410:minus -30.5102 -0.944954 4.30275 2.33028 -36.4796 -27.1224 -13.1927 1.16667
5.59633 0.00947 0.0141 0.00237 0.0123 0.0796 0.0325 0.00604 0.0115 0.00234
2L:8702260-8702410:plus -31.0612 4.84404 2.2844 5.06422 -17.9388 -10.0204 -7.29358 1.04167 -1.79817
0.0106 0.00174 0.0052 0.00433 0.00715 0.00399 0.00181 0.012 0.0315
2L:870360-870510:minus -34.6735 6.83486 -1.37615 2.27523 -18.4184 -18.2041 -21.6422 10.5833 3.46789
0.032 0.000707 0.0179 0.0125 0.0101 0.0103 0.0276 0.000106 0.00582
2L:870360-870510:plus -21.8061 -2.42202 0.394495 1.07339 1.80612 9.60204 2.61468 -3.34722 4.7156
0.00241 0.0219 0.0102 0.0201 0.0000557 0.0000248 0.000135 0.0421 0.00345
2L:8708560-8708710:minus -23.8061 2.09174 6.6055 7.6422 -19.0102 -36.7449 -7.49541 8.09722 5.70642
0.0068 0.00485 0.000872 0.000721 0.0157 0.119 0.00189 0.000552 0.00222
2L:8708560-8708710:plus -33.2347 0.651376 3.92661 5.48624 -17.1837 0.0510204 -17.9633 3.02778
5.44037 0.0276 0.00821 0.00276 0.00357 0.00366 0.000349 0.0149 0.0059 0.00259
2L:8709040-8709190:minus -22.3367 -2.19266 4.76147 0.752294 -18.4184 -18.051 -13.7248 0.777778 11.8532
0.00339 0.0205 0.00196 0.0221 0.0101 0.00886 0.00682 0.0131 0.0000428
2L:8709040-8709190:plus -32.1531 -3.22936 0.165138 1.89908 -8.89796 0.122449 -20.367 8.44444 -4.11927
0.0179 0.0275 0.011 0.0151 0.00189 0.000312 0.022 0.000451 0.0572
2L:8709540-8709690:minus -13 3.02752 -0.00917431 11.2936 -18.4592 -6.72449 -17.3945 -4.65278 -5.13761
0.000563 0.00344 0.0116 0.0000139 0.0109 0.000544 0.0136 0.0571 0.0749
2L:8709540-8709690:plus -14.5918 3.90816 0.284404 5.73394 -17.449 -26.8265 -13.3303 5.375 6.22018
0.00148 0.00102 0.0105 0.00307 0.00484 0.0285 0.00624 0.00222 0.00165
2L:871400-871550:minus -32.2653 3.57143 0.431193 1.90826 -27.1939 -8.27551 -18.8349 2.73611 -3.97248
0.0184 0.00175 0.01 0.015 0.0267 0.000919 0.017 0.00659 0.0553
2L:871400-871550:plus -32.2551 0.66055 -0.412844 0.779817 1.87755 -25.8265 -16.3945 1.29167 10.3486
0.0184 0.00818 0.0133 0.0219 0.0000492 0.0212 0.0116 0.011 0.0000988
2L:8734460-8734610:minus -39.9898 -2.41284 -4.43119 0.834862 -8.93878 -27.1122 -3.6789 -5.625 -1.97248
0.0351 0.0219 0.0426 0.0215 0.00216 0.0316 0.000682 0.0705 0.033
2L:8734460-8734610:plus -21.4796 -1.78899 -4.15596 2.47706 -18.1939 -26.898 -17.4587 -3.72222 7.24771

0.00202 0.0183 0.0396 0.0115 0.0086 0.0302 0.0138 0.0461 0.00116
2L:8735720-8735870:minus -4.52041 0.357798 -2.43119 7.27523 -18.449 -17.8265 -6.16514 -1.31944 -2.83486
0.000158 0.00911 0.0245 0.00109 0.0107 0.00699 0.00137 0.0247 0.0427
2L:8735720-8735870:plus -38.7347 0.779817 -1.11927 5.92661 -17.1122 -26.3776 -18.1927 -1.31944 2.33028
0.0322 0.00784 0.0166 0.00266 0.00341 0.0222 0.0154 0.0247 0.00836
2L:8740600-8740750:minus -32.0714 7.51376 12.0734 3.43119 10.3571 -0.0204082 -6.92661 -1.72222
7.68807 0.0176 0.000495 0.0000408 0.00749 0.0000208 0.000411 0.00166 0.0276 0.000841
2L:8740600-8740750:plus -33.0714 -4.26606 4.88991 2.23853 10.0918 -8.57143 -20.8807 -0.333333
0.12844 0.0267 0.0363 0.00186 0.0128 0.0000251 0.00149 0.0241 0.0185 0.0179
2L:874180-874450:minus -20.9592 -2.3945 3.11009 5.16514 -9.23469 -18.0102 -14.4404 1.54167 -3.14679
0.00184 0.0218 0.00381 0.00424 0.00277 0.00812 0.00799 0.0101 0.0463
2L:874180-874450:plus -42.7449 0.146789 -3.42202 -2.18349 -19.051 -9.72449 -28.7156 4.63889 -2.62385
0.0847 0.0098 0.0324 0.0545 0.0162 0.00352 0.0758 0.00307 0.0402
2L:87460-87750:minus -14.2551 -2.01835 3.12844 6.22936 -17.4898 -27.7551 -5.01835 2.81944 1.58716
0.00127 0.0195 0.00378 0.00209 0.0053 0.0458 0.000998 0.00639 0.0113
2L:87460-87750:plus -12.0714 -0.788991 3.33028 6.21101 -18.7449 -9.02041 -15.844 0.0833333
-4.09174 0.000318 0.0134 0.00349 0.00213 0.0138 0.00213 0.0105 0.0163 0.0568
2L:8825420-8825570:minus -31.2959 -1.82569 5.66972 2.14679 -27.8571 -18.051 -18.6422 -0.611111
3.94495 0.0113 0.0185 0.00134 0.0133 0.039 0.00886 0.0165 0.0201 0.00452
2L:8825420-8825570:plus -29.949 -1.09174 -0.669725 7.17431 -16.8163 -27.0408 2.69725 -0.972222
3.29358 0.00854 0.0148 0.0144 0.00115 0.00323 0.0307 0.000131 0.0224 0.00623
2L:8830980-8831130:minus -31.7755 2.66972 2.18349 2.23853 -26.7041 -17.9388 -20.055 2.04167 1.83486
0.0157 0.00393 0.0054 0.0128 0.0193 0.00738 0.0208 0.00849 0.0101
2L:8830980-8831130:plus -13.7449 1.3211 5.84404 10.5872 -28.2653 0.755102 -17.5138 0.25 12.2752
0.00107 0.00644 0.00124 0.000115 0.0537 0.000175 0.0139 0.0155 0.0000141
2L:8832980-8833130:minus -13.4082 -3.51376 -1.69725 5.43119 -18.0102 -17.2755 -18.2294 1.72222 4.66055
0.000867 0.0297 0.0197 0.00371 0.00732 0.00544 0.0155 0.0095 0.0035
2L:8832980-8833130:plus -29.3673 2.22018 1.74312 9.49541 -18.1122 -19.0102 -13.3578 0.194444 2.06422
0.00838 0.00463 0.00635 0.000308 0.00771 0.0159 0.00628 0.0157 0.00912
2L:8850260-8850410:minus -30.1735 4.40367 5.00917 3.69725 -8.57143 -16.5714 -19.3945 5.97222 -4.48624
0.00869 0.00206 0.00177 0.00665 0.00113 0.00417 0.0186 0.00168 0.0636
2L:8850260-8850410:plus -22.8163 -1.54128 7.88991 0.944954 -26.7041 -26.3776 -13.844 2.63889 9.3945
0.00418 0.0169 0.000464 0.0209 0.0193 0.0222 0.00701 0.00684 0.000358
2L:8943240-8943390:minus -23.449 8.89908 5.66972 2.78899 -27.1939 -17.2755 -27.9725 1.18056 -6.56881
0.00631 0.000236 0.00134 0.00992 0.0267 0.00544 0.0685 0.0114 0.103
2L:8943240-8943390:plus -24.5918 9.12844 -0.0458716 2.41284 0.27551 -9.53061 -7.06422 1.77778
0.174312 0.00821 0.000209 0.0118 0.0118 0.00035 0.0031 0.00171 0.00932 0.0177
2L:8948880-8949030:minus -21.5204 -2.12844 -1.09174 3.21101 -18.7551 -9.09184 -12.5229 1.97222 5.90826
0.00202 0.0202 0.0164 0.00822 0.0144 0.00219 0.00518 0.0087 0.00198
2L:8948880-8949030:plus -22.9286 -0.46789 12.6147 7.10092 -8.67347 -8.53061 -25.7798 0.458333 -0.449541
0.0046 0.0121 0.0000278 0.00121 0.00154 0.00136 0.0521 0.0145 0.0216
2L:8949820-8949970:minus -23.3776 2.51376 7.07339 3.97248 -27.3061 -8.72449 -7.90826 0.0694444
-1.12844 0.00619 0.00415 0.000703 0.00587 0.0315 0.00169 0.00206 0.0164 0.0271
2L:8949820-8949970:plus -31.6327 -2.74312 1.9633 5.69725 -18.4898 -19.0816 -21.2294 0.958333 9.04587
0.0138 0.024 0.00586 0.00314 0.0116 0.017 0.0256 0.0123 0.000423
2L:8950160-8950310:minus -31.5102 -5.33028 1.53211 6.74312 -26.8163 -0.469388 -6.57798 -1.18056
0.917431 0.013 0.0481 0.00685 0.0015 0.0195 0.000533 0.00152 0.0238 0.0146
2L:8950160-8950310:plus -24.4082 3.89908 -0.53211 0.458716 -28.3367 -18.3776 -20.2936 0.291667 1.38532
0.00797 0.0025 0.0138 0.024 0.0557 0.0125 0.0217 0.0153 0.0123
2L:8957800-8957950:minus -12.7755 -1.13761 0.981651 8.80734 -18.051 -17.7857 -7.33028 1.41667 4.73394
0.000477 0.015 0.00832 0.000518 0.00746 0.0068 0.00182 0.0106 0.00342
2L:8957800-8957950:plus -30.4082 3.12844 3.0367 7.27523 -8.92857 -9.65306 -18.9817 8.56944 -0.642202
0.00908 0.00332 0.00392 0.00109 0.00194 0.0034 0.0174 0.000418 0.0234
2L:8958340-8958490:minus -22.7755 -2.90826 -0.954128 3.95413 -18.7449 0.755102 -15.7431 0.222222

11.8532 0.00412 0.0251 0.0157 0.00593 0.0138 0.000175 0.0104 0.0156 0.0000428
2L:8958340-8958490:plus -34.3367 -2.44037 4.50459 6.72477 -27.4898 0.0204082 -30.0275 4.66667
3.11009 0.0318 0.0221 0.00219 0.00154 0.0336 0.000377 0.0913 0.00304 0.00669
2L:8959000-8959150:minus -2.29592 0.550459 9.08257 6.41284 -26.8571 -9.5 -7.22018 2.29167 -8.00917
0.000028 0.00851 0.000242 0.00189 0.0198 0.00294 0.00178 0.00776 0.136
2L:8959000-8959150:plus -21.2551 4.72477 10.0092 3.10092 -17.3776 -19.1224 -13.2294 0.0555556
6.49541 0.00191 0.00182 0.000141 0.00865 0.00419 0.0175 0.00609 0.0164 0.00154
2L:8964400-8964550:minus -14.2245 3.29358 -1.19266 6.11009 -18.6837 -8.5 -10.9358 -3.43056 -0.0825688
0.00126 0.00312 0.0169 0.00233 0.0126 0.00126 0.00369 0.043 0.0189
2L:8964400-8964550:plus -13.6429 3.97959 4.23853 1.12844 -9.23469 -19.3061 -21.156 3.09722 3.62385
0.00101 0.000669 0.00243 0.0198 0.00277 0.0187 0.0253 0.00575 0.00523
2L:8965160-8965310:minus -23.4898 1 0.311927 4.15596 -8.33673 -0.204082 -13.3211 4.23611 -2.68807
0.00639 0.00724 0.0104 0.00539 0.00101 0.000463 0.00622 0.00364 0.041
2L:8965160-8965310:plus -30.7755 5.80734 -0.963303 6.02752 -17.6429 -17.1224 -16.8624 4.19444
7.22018 0.0102 0.00116 0.0158 0.00246 0.00562 0.00499 0.0125 0.00371 0.00119
2L:8966900-8967050:minus -3.44898 -0.59633 3.6055 6.3578 -18.1224 0.387755 -11.7431
-3.33333 3.05505 0.0000713 0.0126 0.00314 0.00195 0.00782 0.000217 0.00436 0.042 0.00692
2L:8966900-8967050:plus -40.4796 -1.24771 -0.917431 6.21101 -28.5306 0.683673 -33.7156
6 -5.12844 0.0407 0.0155 0.0156 0.00213 0.061 0.000184 0.142 0.00166 0.0748
2L:8967400-8967550:minus -21.9286 2.08257 -1.65138 9.51376 -17.4898 0.316327 -21.7431
0.347222 5.44037 0.0027 0.00487 0.0195 0.000292 0.0053 0.000243 0.0282 0.015 0.00259
2L:8967400-8967550:plus -31.398 -0.46789 0.394495 8.77982 -9.16327 -8.79592 -20.2202
-0.305556 0.174312 0.0117 0.0121 0.0102 0.000533 0.00249 0.0019 0.0214 0.0184 0.0177
2L:8987920-8988070:minus -34.1122 -0.0550459 2.10092 -1.76147 -8.89796 -27.051 -22.6697
5.72222 1.07339 0.0312 0.0105 0.00556 0.0471 0.00189 0.0315 0.0332 0.00189 0.014
2L:8987920-8988070:plus -31.7041 4.62385 10.2294 5.49541 -6.93878 -8.72449 -26.2752 3.90278
-4.16514 0.0146 0.00189 0.000125 0.00355 0.000373 0.00169 0.0553 0.00418 0.0579
2L:8989160-8989310:minus -23.0714 -1.66972 2.27523 8.6055 -26.9694 -27.3367 -8.66055
2.48611 5.23853 0.0052 0.0176 0.00522 0.000567 0.023 0.0347 0.0024 0.00723 0.00285
2L:8989160-8989310:plus -22.3367 -2.02752 4.95413 7 -18.1837 -18.8265 -3.59633 -6.58333
5.7156 0.00339 0.0196 0.00181 0.00132 0.00835 0.0148 0.000666 0.0855 0.00217
2L:8997440-8997590:minus -31.8571 6.38532 4.62385 1.56881 10.9184 -7.65306 -6.54128 0.222222
-0.908257 0.0163 0.000882 0.00208 0.0172 4.19e-06 0.000647 0.00151 0.0156 0.0255
2L:8997440-8997590:plus -23.7347 8.73394 3.6789 4.79817 -18.7143 -8.5 -8.6055 1.77778 -2.05505
0.00673 0.000258 0.00305 0.00453 0.0136 0.00126 0.00237 0.00932 0.0338
2L:9125720-9125870:minus -33.602 0.724771 -3.17431 1.6789 -27.2653 -17.1224 -21.5963
0.902778 -4.43119 0.0289 0.008 0.0303 0.0165 0.0302 0.00499 0.0274 0.0125 0.0627
2L:9125720-9125870:plus -13.1122 6.6789 2.52294 8.05505 -26.9694 -26.6735 -10.4771 -7.19444
-3.68807 0.000632 0.000764 0.00476 0.000578 0.023 0.0256 0.00337 0.096 0.0516
2L:9143300-9143450:minus -31.5816 0.0183486 10.1101 6.43119 -8.71429 -26.8571 -12.8257
8.51389 5.69725 0.0135 0.0102 0.000134 0.00187 0.00159 0.0288 0.00555 0.000433 0.00225
2L:9143300-9143450:plus -22.9184 5.46789 1.17431 4.91743 -17.4592 -8.79592 -12.3578 2.88889
4.9633 0.00447 0.00134 0.00778 0.00447 0.00491 0.0019 0.00499 0.00622 0.00316
2L:9158480-9158630:minus -32.5204 1.3578 2.36697 0.633028 -8.57143 -7.20408 -30.2477
-0.555556 -0.862385 0.0204 0.00635 0.00505 0.0229 0.00113 0.000548 0.0941 0.0198 0.0251
2L:9158480-9158630:plus -32.898 4.6422 -1.3578 5.47706 -18.7143 -8.65306 -19.2936 3.80556 4.45872
0.0247 0.00188 0.0178 0.0036 0.0136 0.00156 0.0183 0.00435 0.00366
2L:9166680-9166830:minus -31.398 -4.22936 13.4862 10.5872 -17.7143 -8.5 -11.7248 -4.44444
-5.3945 0.0117 0.036 0.0000132 0.000115 0.00627 0.00126 0.00434 0.0545 0.079
2L:9166680-9166830:plus -23.449 0 15.0826 2.53211 -18.4184 -9.34694 -19.1835 1.34722 -0.302752
0.00631 0.0103 1.18e-06 0.0113 0.0101 0.00261 0.018 0.0108 0.0208
2L:9175320-9175470:minus -31.398 -1.19266 -0.0458716 1.53211 -27.5306 -17.2755 -20.7615
6.86111 3.61468 0.0117 0.0152 0.0118 0.0174 0.0351 0.00544 0.0236 0.00108 0.00526
2L:9175320-9175470:plus -23.1837 4.12844 3.9633 5.74312 -18.9796 -8.42857 -11.4404 1.70833

-2.43119 0.00553 0.00229 0.00272 0.00302 0.0155 0.00104 0.00409 0.00955 0.038
2L:9176160-9176310:minus -22.8163 2.69725 4.36697 3.97248 -8.60204 -9.0102 -13.367 1.22222
-4.76147 0.00418 0.00389 0.00231 0.00587 0.00125 0.00208 0.00629 0.0113 0.0686
2L:9176160-9176310:plus -13.0816 4.79817 12.6972 9.7156 -26.1939 -8.5 -18.1376 0.194444
-2.72477 0.000599 0.00177 0.0000264 0.000242 0.0175 0.00126 0.0153 0.0157 0.0414
2L:9176800-9176950:minus -32.8469 -1.47706 -3.93578 -0.0183486 -17.5204 -26.602
-12.4128 2.06944 5.24771 0.0243 0.0166 0.0373 0.027 0.00532 0.0248 0.00506 0.00841 0.00282
2L:9176800-9176950:plus -42.8469 -1.97248 1.92661 2.7156 -17.5204 -9.57143 -25.0459
2.63889 3 0.0869 0.0193 0.00594 0.0102 0.00532 0.00326 0.0474 0.00684 0.00702
2L:9178600-9178750:minus -24.4082 7.72477 1.68807 11.2936 -8.71429 -8.45918 -17.9174
4.02778 0.862385 0.00797 0.000443 0.00648 0.0000139 0.00159 0.00111 0.0148 0.00397 0.0148
2L:9178600-9178750:plus -21.7755 -1.31193 6.75229 4.04587 -18.6429 -8.93878 -10.0642
-2.13889 7.83486 0.00241 0.0158 0.000815 0.00572 0.0121 0.00201 0.00311 0.0309 0.000712
2L:9179320-9179470:minus -32.8163 0.733945 -1.3578 5.90826 -18.7143 -8.5 -12.1468
-0.319444 3.3211 0.024 0.00797 0.0178 0.00271 0.0136 0.00126 0.00476 0.0184 0.00616
2L:9179320-9179470:plus -24.3673 -0.926606 3.6055 8.61468 -8.67347 -26.6735 -13.2018
-2.875 10.0367 0.00791 0.014 0.00314 0.000552 0.00154 0.0256 0.00605 0.0375 0.000146
2L:9180700-9180850:minus -32.5612 2.6422 -4.02752 7.43119 -27.8265 -25.602 -22.5872
4.70833 3.52294 0.0207 0.00397 0.0383 0.000939 0.0388 0.0207 0.0327 0.00298 0.00558
2L:9180700-9180850:plus -32.9388 2.10092 -0.541284 6.00917 -27.1939 -0.204082 -26.0734
-0.361111 -4.79817 0.025 0.00484 0.0138 0.00255 0.0267 0.000463 0.054 0.0187 0.0693
2L:9182840-9182990:minus -39.0306 -4.44037 -1.83486 -2.21101 -18.1939 -26.6735
-15.2477 -2.08333 2.34862 0.0326 0.0381 0.0206 0.055 0.0086 0.0256 0.00943 0.0305 0.0083
2L:9182840-9182990:plus -23.7653 -1.65138 2.77982 2.78899 -27.0408 -15.9796 -17.2202
5.08333 1.93578 0.00675 0.0175 0.00432 0.00992 0.0242 0.00405 0.0133 0.00253 0.00969
2L:9183320-9183470:minus -40.3673 3.09174 1.57798 5.57798 -17.7143 0.897959 -22.9908
1.84722 1.16514 0.0397 0.00337 0.00674 0.0034 0.00627 0.000144 0.035 0.0091 0.0135
2L:9183320-9183470:plus -22.5306 1.78899 10.3211 4.17431 -26.898 -8.23469 -20.9725 6.31944
-0.642202 0.00356 0.00542 0.000118 0.00537 0.0203 0.000875 0.0245 0.00142 0.0234
2L:9184280-9184430:minus -40.2551 1.54128 -0.0917431 3.54128 -17.6429 -9.45918 -26.8257 -0.819444
-4.6055 0.0382 0.00594 0.0119 0.00702 0.00562 0.00279 0.0592 0.0214 0.0656
2L:9184280-9184430:plus -32.8469 -0.348624 0.724771 9.59633 -16.449 -9.53061 -6.59633 6.38889
-1.83486 0.0243 0.0116 0.00909 0.000275 0.00307 0.0031 0.00153 0.00137 0.0318
2L:9184880-9185030:minus -24.2653 4.44037 5.11927 5.74312 -27.0102 -8.27551 -6.49541 5.59722 1.75229
0.00773 0.00203 0.00169 0.00302 0.0237 0.000919 0.00149 0.002 0.0104
2L:9184880-9185030:plus -33.1939 5.33945 -0.844037 4.0367 -28.5306 1.7551 -26.3394 0.527778
-10.6147 0.0274 0.00142 0.0152 0.00578 0.061 0.0000519 0.0558 0.0142 0.217
2L:9203500-9203650:minus -23.2245 -4.41284 -1.61468 3.47706 -18.7551 -17.8571 -11.1835 0.0694444
8.20183 0.00576 0.0378 0.0193 0.00724 0.0144 0.00719 0.00387 0.0164 0.000539
2L:9203500-9203650:plus -24.4082 2.3211 4.22018 2.98165 -26.9694 -8.65306 -26.1743 0.986111 -3.66972
0.00797 0.00446 0.00245 0.00905 0.023 0.00156 0.0546 0.0122 0.0514
2L:9229800-9229950:minus -21.3673 7.48624 1.16514 4.0367 -17.6429 -8.79592 -17.5046 6.25 5.23853
0.00195 0.000502 0.0078 0.00578 0.00562 0.0019 0.0139 0.00147 0.00285
2L:9229800-9229950:plus -32.5102 -5.33028 10.8716 3.90826 -8.92857 -19.3469 -15.1835 5.63889 -0.559633
0.0202 0.0481 0.0000865 0.00603 0.00194 0.0194 0.00931 0.00196 0.0226
2L:9231220-9231370:minus -13.6735 4.25688 6.44037 6.29358 -17.449 -9.45918 -16.5413 -1.06944 5.38532
0.00103 0.00218 0.000945 0.00202 0.00484 0.00279 0.0119 0.023 0.00267
2L:9231220-9231370:plus -22.9592 6.54128 3.76147 4.05505 -18.1939 -9.57143 -5.2844 10.3611 6.6422
0.00475 0.000818 0.00295 0.00566 0.0086 0.00326 0.00108 0.000125 0.00148
2L:9231720-9231870:minus -21.8163 2.73394 -1.70642 -1.98165 -17.7857 -17.2755 -17.3486 6.05556 5.76147
0.00244 0.00383 0.0198 0.051 0.00676 0.00544 0.0135 0.00161 0.0021
2L:9231720-9231870:plus -30.8163 0.697248 -1.80734 1.77064 -19.0918 -18.051 -22.7339 12.1528 1.41284
0.0103 0.00807 0.0204 0.0159 0.0163 0.00886 0.0335 0.0000267 0.0121
2L:9237700-9237850:minus -22.9592 1.66055 -0.46789 4.22018 -27.2653 -19.3061 2.37615 3.34722 2.88991

0.00475 0.00569 0.0135 0.00531 0.0302 0.0187 0.000146 0.00522 0.00725
2L:9237700-9237850:plus -33 -1.90826 2 2.11009 -18.1939 -8.23469 -23.3486 -0.583333 -2.47706
0.0258 0.0189 0.00578 0.0136 0.0086 0.000875 0.0371 0.02 0.0385
2L:9238160-9238310:minus -32.5918 1.27523 5.06422 9.23853 -17.4898 -9.30612 -24.9725 1.81944 -4.40367
0.021 0.00655 0.00173 0.000424 0.0053 0.00257 0.047 0.00919 0.0623
2L:9238160-9238310:plus -22.2245 -0.642202 2.66055 -0.376147 -19.0102 -7.72449 -17.1193 2.55556
2.10092 0.00325 0.0128 0.00452 0.0299 0.0157 0.00068 0.0131 0.00705 0.00901
2L:9253100-9253250:minus -31.7041 1.80734 4.08257 7.78899 0.836735 -18.051 -18.7431 3.30556 3.88991
0.0146 0.00539 0.00259 0.000642 0.000215 0.00886 0.0168 0.0053 0.00467
2L:9253100-9253250:plus -21.8878 2.18349 6.08257 5.75229 -19.051 -17.3469 -6.33028 -0.5 -2.3211
0.00258 0.00469 0.00111 0.00296 0.0162 0.0058 0.00143 0.0195 0.0365
2L:9330660-9330810:minus -33.7041 0.834862 0.587156 3.19266 -18.9796 0.602041 -24.9908 3.58333 -2.59633
0.0293 0.00769 0.00952 0.00829 0.0155 0.000188 0.0471 0.00475 0.0398
2L:9330660-9330810:plus -24.2551 0.807339 3.44037 6.12844 11.6939 -9.53061 8.40367 3.75 -0.53211
0.00768 0.00776 0.00335 0.00231 1.17e-06 0.0031 0.0000213 0.00445 0.0222
2L:9387140-9387290:minus -21.6735 -1.78899 -4.3945 3.59633 -27.5306 -9.86735 -20.7706 4.15278 -0.651376
0.00224 0.0183 0.0422 0.00693 0.0351 0.00391 0.0236 0.00377 0.0235
2L:9387140-9387290:plus -13.7449 0.192661 11.8716 9.44954 -8.0102 -8.94898 1.33945 7.72222 -0.807339
0.00107 0.00965 0.0000468 0.000326 0.000826 0.00203 0.000201 0.000682 0.0247
2L:9387540-9387690:minus -31.4388 3.90816 3.06422 4.44037 -18.7857 0.979592 -9.15596 4.13889 5.53211
0.0122 0.00102 0.00387 0.00493 0.0148 0.000136 0.00263 0.0038 0.00245
2L:9387540-9387690:plus -13.3673 3.97959 7.22936 10.8624 -18.4898 -7.79592 -12.4037 3.625 3.29358
0.000813 0.000669 0.000649 0.0000687 0.0116 0.000723 0.00505 0.00468 0.00623
2L:9388180-9388330:minus -41.2959 6.33028 0.59633 1.87156 -18.4898 -9.79592 -23.211 5.72222 7.11927
0.0529 0.000904 0.00949 0.0152 0.0116 0.00377 0.0363 0.00189 0.00125
2L:9388180-9388330:plus -32.6224 6.02752 6.34862 3.0367 -27.2347 -17.8571 -0.504587 0.0694444
3.45872 0.0211 0.00104 0.000987 0.00886 0.0283 0.00719 0.000318 0.0164 0.00591
2L:9431040-9431190:minus -33.4796 2.16514 12.4312 7.29358 -26.7041 -26.4898 -8.30275 -2.47222 5.59633
0.0285 0.00473 0.0000317 0.00104 0.0193 0.0225 0.00224 0.0338 0.00234
2L:9431040-9431190:plus -23 0.229358 13.1835 7.2844 -18.7857 19.1531 -11.8991 0.388889 -2.26606
0.00498 0.00953 0.0000171 0.00105 0.0148 7.95e-07 0.00451 0.0148 0.0359
2L:9449720-9449870:minus -24.1122 -1.13761 1.55963 5.91743 -17.1531 -27.5612 -5.10092 -1.52778 8.05505
0.00738 0.015 0.00678 0.00267 0.00364 0.0393 0.00102 0.0262 0.00064
2L:9449720-9449870:plus -20.4388 1.18349 1.68807 5.93578 -17.4898 -18.9286 2.22936 1.98611 3.6789
0.00167 0.00677 0.00648 0.00264 0.0053 0.0153 0.000153 0.00866 0.00506
2L:94720-94870:minus -33 3.26606 2.90826 2.19266 -27.1633 -8.5 -19.9174 -1.84722 0.926606 0.0258
0.00315 0.00411 0.0131 0.025 0.00126 0.0203 0.0286 0.0146
2L:94720-94870:plus -11.1531 9.55963 1.33945 2.55046 -19.3163 -8.5 -11.0734 7.69444 0.385321 0.000246
0.000167 0.00733 0.0112 0.0165 0.00126 0.00379 0.000693 0.0166
2L:9494000-9494150:minus -32.0714 -0.651376 11.8991 3.2844 -26.2653 -8.61224 -12.6422 1.36111
-2.52294 0.0176 0.0128 0.0000462 0.00793 0.018 0.00152 0.00533 0.0108 0.0391
2L:9494000-9494150:plus -31.1837 -3.89908 11.7798 -0.0550459 -16.9286 -9.27551 -7.2844 3.76389
7.22018 0.0109 0.0329 0.0000501 0.0272 0.00327 0.00244 0.0018 0.00442 0.00119
2L:9494720-9494870:minus -21.8061 1.44037 -1.2844 0.238532 -27.0408 -27.9694 -6.3945 -0.0416667
5.59633 0.00241 0.00617 0.0174 0.0251 0.0242 0.0547 0.00145 0.017 0.00234
2L:9494720-9494870:plus -13.1531 3.62385 4.52294 4.05505 0.612245 -18.0102 -7.83486 -2.41667 7.63303
0.00067 0.00276 0.00217 0.00566 0.000269 0.00812 0.00203 0.0333 0.000928
2L:9495400-9495550:minus -23.2959 7.25688 1.69725 3.23853 -9.5 -10.0918 -19.6422 9.91667 3.6789
0.00605 0.000568 0.00646 0.00815 0.00293 0.00404 0.0194 0.000174 0.00506
2L:9495400-9495550:plus -31.7143 -2.09174 0.275229 3.88991 -9.0102 -9.23469 -13.7339 -0.930556
0.293578 0.0146 0.0199 0.0106 0.00608 0.00236 0.00238 0.00684 0.0221 0.017
2L:9497600-9497750:minus -14.2959 1.58716 2.97248 7 -26.9694 -26.8265 -6.3578 -4.72222 9.87156
0.00129 0.00584 0.00401 0.00132 0.023 0.0285 0.00144 0.058 0.000217
2L:9497600-9497750:plus -21.6327 4.46789 4.34862 7.17431 -18.3776 -9.38776 -18.1927 3.38889 -5.17431

0.00215 0.00201 0.00233 0.00115 0.00926 0.00266 0.0154 0.00513 0.0755
2L:9498520-9498670:minus -31.7347 2.07339 -1.34862 3.20183 -18.7857 -18.051 -16.1376 -0.541667
-6.14679 0.0149 0.00488 0.0178 0.00824 0.0148 0.00886 0.0111 0.0197 0.0921
2L:9498520-9498670:plus -14.449 6.98165 -2.19266 4.05505 -7.89796 9.60204 -14.1284 3.65278 -3.50459
0.0014 0.000656 0.0229 0.00566 0.000678 0.0000248 0.00746 0.00462 0.0497
2L:9501400-9501550:minus -32.551 3.89908 7.65138 5.29358 -18.4592 -9.7551 -13.5321 7.05556 -0.651376
0.0207 0.0025 0.000526 0.00398 0.0109 0.00358 0.00653 0.000977 0.0235
2L:9501400-9501550:plus -20.8878 6.80734 -2.08257 2.3945 -27.1531 0.163265 -16.0183 -2.06944 -2.22018
0.0018 0.000716 0.0222 0.0119 0.0249 0.000307 0.0109 0.0304 0.0355
2L:9502600-9502750:minus -22.449 -0.550459 1.42202 9.59633 -18.6429 -8.45918 -13.3119 -1.72222
1.38532 0.0035 0.0124 0.00712 0.000275 0.0121 0.00111 0.00621 0.0276 0.0123
2L:9502600-9502750:plus -41.449 0.66055 3.36697 5.34862 -17.449 -18.9796 -12.9083 3.625 3.66972
0.0564 0.00818 0.00345 0.00387 0.00484 0.0155 0.00566 0.00468 0.00514
2L:9502980-9503130:minus -14.2959 -4.45872 1.26606 2.27523 -17.7143 -36.4082 -0.0366972 3.80556
7.22018 0.00129 0.0382 0.00753 0.0125 0.00627 0.105 0.000286 0.00435 0.00119
2L:9502980-9503130:plus -32.1122 -1.13761 -4.44037 2.68807 -18.7551 -19.3469 -18.6697 7.95833 0.715596
0.0178 0.015 0.0427 0.0104 0.0144 0.0194 0.0166 0.000597 0.0154
2L:9521060-9521210:minus -42.5918 0.100917 0.93578 -0.40367 -17.7143 -18.2755 -21.4587 8 3.98165
0.0819 0.00996 0.00845 0.0302 0.00627 0.0112 0.0267 0.000583 0.00439
2L:9521060-9521210:plus -31.551 3.55046 1.00917 5.57798 -18.0204 -9.34694 -3.97248 -1.38889 -5.58716
0.0134 0.00284 0.00824 0.0034 0.00733 0.00261 0.00074 0.0252 0.0817
2L:9521360-9521510:minus -13.5612 10.1927 8.81651 0.513761 -18.4592 -0.173469 -22.5596 1.44444
11.3853 0.000984 0.000122 0.000281 0.0235 0.0109 0.000456 0.0326 0.0105 0.0000815
2L:9521360-9521510:plus -23.449 3.64286 -0.972477 2.11009 -17.7143 -9.79592 4.3211 7.65278
1.85321 0.00631 0.00159 0.0158 0.0136 0.00627 0.00377 0.0000734 0.000709 0.01
2L:9567560-9567710:minus -21.6633 4.0367 7.31193 2.93578 -27.2653 -9.30612 -15.9541 2.34722 -1.57798
0.00223 0.00237 0.000623 0.00927 0.0302 0.00257 0.0108 0.00761 0.0299
2L:9567560-9567710:plus -21.6327 -1.04587 9.30275 1.62385 -17.4898 -18.2041 -12.4404 -0.875 5.13761
0.00215 0.0145 0.000213 0.0168 0.0053 0.0103 0.00509 0.0218 0.00298
2L:9568420-9568570:minus -30.6735 1.91743 5.22018 7.34862 -18.2653 -28.0408 -13.9908 0.125 7.6789
0.00978 0.00518 0.00162 0.000998 0.00882 0.0551 0.00724 0.0161 0.000866
2L:9568420-9568570:plus -22.8571 1.56881 -0.559633 2.81651 -8.93878 -9.45918 -15.1468 -3.33333
4.18349 0.00427 0.00588 0.0139 0.00982 0.00216 0.00279 0.00924 0.042 0.00404
2L:9569280-9569430:minus -23.4184 -0.889908 4.46789 3.7156 -27.2653 0.27551 -13.7339 0.791667
-0.0825688 0.00624 0.0138 0.00222 0.00655 0.0302 0.000257 0.00684 0.013 0.0189
2L:9569280-9569430:plus -32.6224 -0.302752 -0.724771 -2.31193 -26.9388 2.05102 -14.1193 2.59722
9.45872 0.0211 0.0114 0.0146 0.0565 0.0215 0.0000468 0.00745 0.00694 0.000337
2L:9569700-9569850:minus -41.2143 6.90826 0.238532 3.53211 -27 -37.1837 4.19266 3.20833 3.46789
0.0494 0.000681 0.0107 0.00704 0.0233 0.14 0.0000769 0.00551 0.00582
2L:9569700-9569850:plus -32.1122 -0.899083 1.73394 2.90826 -8.40816 -19.3061 -11.8073 -0.652778
3.6422 0.0178 0.0139 0.00638 0.00936 0.00108 0.0187 0.00442 0.0204 0.00516
2L:9576680-9576830:minus -31.7347 -0.669725 -3.63303 2.78899 20.4694 9.45918 -6.77064 4.26389
3.14679 0.0149 0.0129 0.0344 0.00992 1.65e-07 0.0000356 0.0016 0.0036 0.00666
2L:9576680-9576830:plus -22.6224 0.0550459 -4.05505 0.550459 -18.449 -7.79592 -16.0826 1.91667
1.37615 0.00371 0.0101 0.0385 0.0233 0.0107 0.000723 0.011 0.00888 0.0123
2L:9613240-9613390:minus -11.449 7.82569 -0.449541 -0.0917431 -8.64286 -18.0102 -20.7706 5.70833
4.98165 0.000252 0.00042 0.0134 0.0276 0.00139 0.00812 0.0236 0.0019 0.00313
2L:9613240-9613390:plus -21.8878 4.00917 5.80734 5.47706 -18.6837 -19.1224 -15.8165 12.8611 2.15596
0.00258 0.0024 0.00126 0.0036 0.0126 0.0175 0.0105 0.0000122 0.00881
2L:9613900-9614050:minus -20.9286 1.77982 -2.19266 6.80734 -18.9796 0.244898 -26.2752 -1.08333 -2.73394
0.00183 0.00544 0.0229 0.00145 0.0155 0.000285 0.0553 0.0231 0.0416
2L:9613900-9614050:plus -31.1429 2.48624 1.85321 2.24771 -27.2245 -8.79592 -23.7706 3.56944 0.220183
0.0108 0.0042 0.0061 0.0127 0.0272 0.0019 0.0397 0.00478 0.0174
2L:9616740-9616890:minus -12.3061 13.5306 3.97248 6.3578 -8.89796 0.387755 -2.98165 -1.51389 -4.77064

0.000385 0.0000206 0.00271 0.00195 0.00189 0.000217 0.000565 0.0261 0.0687
2L:9616740-9616890:plus -31.5102 1.42202 0.825688 8.88073 -9.0102 0.0204082 -20.0183 -3.86111
7.53211 0.013 0.00621 0.00878 0.000479 0.00236 0.000377 0.0207 0.0477 0.000968
2L:9633800-9633950:minus -22.1122 -3.22018 0.256881 1.25688 -27.0102 -18.898 -19.6514 3.31944 4.0367
0.00304 0.0274 0.0106 0.0192 0.0237 0.0152 0.0194 0.00528 0.00424
2L:9633800-9633950:plus -14.3673 0.412844 3.00917 7.88991 -17.5306 -26.5612 -6.86239 -3.65278 6.49541
0.00132 0.00893 0.00396 0.00062 0.0054 0.0237 0.00163 0.0454 0.00154
2L:9657460-9657610:minus -41.7041 -3.17431 -1 7.61468 -18.7449 9.57143 -17.0642 -1.06944 0.495413
0.0641 0.027 0.016 0.000779 0.0138 0.0000274 0.0129 0.023 0.0162
2L:9657460-9657610:plus -33.2959 -1.23853 8.19266 6.49541 -18.3776 -17.9388 -7.79817 7.875 1.69725
0.0279 0.0154 0.000395 0.00176 0.00926 0.00738 0.00202 0.000626 0.0108
2L:9671320-9671470:minus -23.0816 -1.86239 -1.63303 0.880734 -8.60204 9.53061 -24.1835 2.18056 3.11009
0.00522 0.0187 0.0194 0.0213 0.00125 0.0000316 0.0422 0.00808 0.00669
2L:9671320-9671470:plus -31.7041 -1.97248 -0.0917431 5.88991 -18.4184 -18.8571 -15.5963 2.91667
4.66055 0.0146 0.0193 0.0119 0.00273 0.0101 0.0151 0.0101 0.00616 0.0035
2L:9699200-9699350:minus -31.7041 1.26606 4.7156 11.0183 -17.4898 -18.6327 -4.23853 -1.04167 4.21101
0.0146 0.00657 0.002 0.000052 0.0053 0.0137 0.000798 0.0228 0.00396
2L:9699200-9699350:plus -3.96939 3.11009 0.587156 2.44037 -18.4898 -17.8571 -9.63303 -2.59722 -0.256881
0.000119 0.00334 0.00952 0.0116 0.0116 0.00719 0.00288 0.0349 0.0204
2L:9699740-9699890:minus -42.1122 3.90816 -2.82569 1.20183 -28.5714 -9.09184 -17.8532 4.61111 3.09174
0.0698 0.00102 0.0275 0.0195 0.0623 0.00219 0.0146 0.00311 0.00678
2L:9699740-9699890:plus -13.5612 4.6422 3.97248 7.27523 -18.4184 -19.2347 10.2661 2.30556 5.44037
0.000984 0.00188 0.00271 0.00109 0.0101 0.018 0.0000118 0.00772 0.00259
2L:9700440-9700590:minus -23.2245 -1.01835 1.79817 1.07339 -8.67347 9.60204 -11.1376 3.48611 -2.41284
0.00576 0.0144 0.00623 0.0201 0.00154 0.0000248 0.00384 0.00494 0.0376
2L:9700440-9700590:plus -29.3265 -1.88991 1.91743 7.15596 -17.1122 -27.602 -13.9266 3.33333 3
0.00837 0.0188 0.00596 0.00117 0.00341 0.0416 0.00714 0.00525 0.00702
2L:9703100-9703250:minus -22.7347 4.22936 2.47706 6.18349 0.540816 -28.4082 -16.7156 4.38889 3.46789
0.00403 0.0022 0.00484 0.00217 0.000321 0.0615 0.0122 0.00342 0.00582
2L:9703100-9703250:plus -22.8469 1.77064 7.47706 5.90826 -18.6429 -18.2347 -21.3394 -2.04167 -5.45872
0.00424 0.00546 0.000573 0.00271 0.0121 0.0104 0.0262 0.0301 0.0799
2L:9703880-9704030:minus -43.8469 -2.15596 0.889908 5.57798 -9.23469 -9.60204 -26.2202 2.43056 -5.89908
0.0939 0.0203 0.00859 0.0034 0.00277 0.00334 0.055 0.00738 0.0869
2L:9703880-9704030:plus -32.0408 6.98165 5.44954 2.99083 -18.7857 -17.9796 -14.9358 3.26389 0.266055
0.0174 0.000656 0.00147 0.00899 0.0148 0.00764 0.00886 0.00539 0.0171
2L:9705940-9706090:minus -4.40816 3.10092 5.20183 2.26606 -26.9694 -17.7143 -10.1376 -2.43056 11.3853
0.000148 0.00335 0.00163 0.0126 0.023 0.00634 0.00316 0.0334 0.0000815
2L:9705940-9706090:plus -32.0408 -4.06422 -1.11927 7.63303 -7.56122 -9.20408 -23.1468 5.19444 -0.357798
0.0174 0.0344 0.0166 0.000745 0.000419 0.00225 0.0359 0.00241 0.021
2L:9711120-9711270:minus -30.4796 -0.669725 -1.61468 4.24771 -8.93878 -0.540816 -21.3028 2.18056
-0.12844 0.00941 0.0129 0.0193 0.00519 0.00216 0.00054 0.026 0.00808 0.0193
2L:9711120-9711270:plus -23.2959 -2.27523 1.79817 1.55046 -18.9796 -8.65306 -15.8532 2.5 -0.642202
0.00605 0.021 0.00623 0.0173 0.0155 0.00156 0.0106 0.0072 0.0234
2L:9735960-9736110:minus -21.1837 5.87156 0.321101 6.84404 -17.3469 -8.86735 -13.578 -2.05556 -3.13761
0.00189 0.00112 0.0104 0.0014 0.00394 0.00198 0.0066 0.0303 0.0461
2L:9735960-9736110:plus -24.2653 6.00917 0.357798 7.12844 -18.4184 -18.6327 -5.3578 -0.402778
4.92661 0.00773 0.00105 0.0103 0.00118 0.0101 0.0137 0.0011 0.0189 0.00318
2L:9736500-9736650:minus -21.6327 -0.87156 2.02752 5.22936 0.653061 -18.1531 -16.1743 4.75 1.99083
0.00215 0.0138 0.00572 0.0041 0.000239 0.00997 0.0112 0.00293 0.00946
2L:9736500-9736650:plus -13.2959 3.90816 4.68807 9.44954 -17.3776 -27.5612 -7.57798 -8.19444 0.275229
0.000775 0.00102 0.00203 0.000326 0.00419 0.0393 0.00192 0.115 0.0171
2L:9758180-9758330:minus -14.4082 0.770642 0.155963 5.58716 -26.898 -8.72449 -15.7156 6.25 -3.13761
0.00136 0.00786 0.011 0.00334 0.0203 0.00169 0.0103 0.00147 0.0461
2L:9758180-9758330:plus -22.6224 -4.0367 5.37615 5.57798 -17.3776 -8.42857 -16.2936 1.36111 1.59633

0.00371 0.0342 0.00152 0.0034 0.00419 0.00104 0.0114 0.0108 0.0113
2L:9758580-9758730:minus -22.1531 2.01835 3.48624 5.07339 -8.0102 -9.5 -12.5138 5.02778 -1.88073
0.0031 0.00499 0.00329 0.0043 0.000826 0.00294 0.00517 0.00259 0.0321
2L:9758580-9758730:plus -31.1837 0.165138 4.55046 4.59633 -27.1939 -7.72449 -18.156 0.416667 12.2752
0.0109 0.00974 0.00214 0.00478 0.0267 0.00068 0.0153 0.0147 0.0000141
2L:9759000-9759150:minus -22.7347 0.853211 3.87156 4.33028 -18.4184 -28.4082 -19.7156 1.04167 -1.85321
0.00403 0.00763 0.00282 0.00511 0.0101 0.0615 0.0196 0.012 0.032
2L:9759000-9759150:plus -13.4796 4.19266 -2.10092 1.97248 -18.4592 0.612245 4.13761 2.15278 2.88991
0.000935 0.00224 0.0223 0.0146 0.0109 0.000187 0.0000785 0.00816 0.00725
2L:9763280-9763430:minus -24.2143 0.577982 0.321101 1.76147 -17.6837 -8.72449 -14.2385 0.638889 5.23853
0.00754 0.00843 0.0104 0.0159 0.0058 0.00169 0.00765 0.0137 0.00285
2L:9763280-9763430:plus -14.1429 2.88073 -0.926606 -1.80734 -27.2245 -8.65306 -14.7431 0.472222
3.62385 0.0012 0.00363 0.0156 0.0479 0.0272 0.00156 0.00851 0.0144 0.00523
2L:9779880-9780030:minus -20.6224 -1.09174 3.3945 6.31193 -26.898 -27.3367 -5.7156 1.34722 -1.18349
0.0017 0.0148 0.00341 0.00199 0.0203 0.0347 0.00121 0.0108 0.0276
2L:9779880-9780030:plus -21.7041 -1.0367 4.15596 6.00917 -27.898 -18.8571 3.54128 6.25 1.37615
0.00231 0.0145 0.00251 0.00255 0.04 0.0151 0.0000975 0.00147 0.0123
2L:9782340-9782490:minus -31.3265 1.37615 1.72477 3.86239 -18.9796 -8.53061 -15.1284 -0.0694444
11.4771 0.0114 0.00631 0.0064 0.00622 0.0155 0.00136 0.00921 0.0171 0.0000679
2L:9782340-9782490:plus -22.9592 -0.862385 6.55963 4.95413 -9.23469 -19.0816 -20.7798 1.26389
-0.192661 0.00475 0.0137 0.000892 0.00442 0.00277 0.017 0.0236 0.0111 0.0198
2L:9790660-9790810:minus -22.9592 -0.568807 2.75229 6.29358 -8.89796 -8.68367 -15.7798 1.01389
-4.90826 0.00475 0.0125 0.00436 0.00202 0.00189 0.00157 0.0104 0.0121 0.0714
2L:9790660-9790810:plus -23.1531 -1.44954 0.00917431 6.36697 10.6531 10.0204 -22.7064 3.88889
-0.761468 0.00542 0.0165 0.0116 0.00193 0.0000102 0.0000106 0.0334 0.00421 0.0244
2L:9793040-9793190:minus -40.4388 0.137615 5.9633 2.31193 0.877551 -9.02041 -12.1835 3.38889 1.12844
0.0401 0.00984 0.00117 0.0123 0.00019 0.00213 0.0048 0.00513 0.0137
2L:9793040-9793190:plus -23.5204 9.43119 2.11927 2.19266 1.17347 -18.0102 -9.10092 4.30556 10.0367
0.00645 0.000179 0.00553 0.0131 0.000101 0.00812 0.00261 0.00354 0.000146
2L:9887920-9888070:minus -31.5102 7.59633 -3.46789 2.52294 -26.1939 -9.64286 -17.1284 6.76389 -2.92661
0.013 0.000474 0.0329 0.0113 0.0175 0.00339 0.0131 0.00114 0.0437
2L:9887920-9888070:plus -40.2245 5.22936 1.86239 5.57798 10.7245 -26.5306 -23.4037 5.79167 4.40367
0.0374 0.00148 0.00608 0.0034 5.2e-06 0.023 0.0375 0.00183 0.00373
2L:9890700-9890850:minus -13.5612 7.61468 -1 7.21101 -27.1531 -18.2755 -19.6514 3.01389 6.7156
0.000984 0.00047 0.016 0.0011 0.0249 0.0112 0.0194 0.00593 0.00144
2L:9890700-9890850:plus -34.3367 13.3878 -0.761468 1.89908 10.7245 -8.45918 -15.2936 5.22222
-2.98165 0.0318 0.0000869 0.0148 0.0151 5.2e-06 0.00111 0.00951 0.00238 0.0445
2L:9893400-9893550:minus -30.4796 13.4592 8.37615 3.31193 -18.4898 -17.2347 -4.62385 -1.13889 0.302752
0.00941 0.000064 0.000358 0.00786 0.0116 0.00511 0.000891 0.0235 0.0169
2L:9893400-9893550:plus -22.2245 -1.3578 0.00917431 5.22936 -18.3776 -8.79592 -27.578 5.05556
4.73394 0.00325 0.016 0.0116 0.0041 0.00926 0.0019 0.0651 0.00256 0.00342
2L:9895200-9895350:minus -22.8571 0.651376 3.49541 5.57798 -18.7143 -0.204082 -16.2294 -0.416667
-2.56881 0.00427 0.00821 0.00327 0.0034 0.0136 0.000463 0.0113 0.019 0.0396
2L:9895200-9895350:plus -32.1735 -1.08257 14.8532 1.66972 -17.9796 -9.94898 -21.422 -0.680556
-4.24771 0.018 0.0147 2.28e-06 0.0165 0.00726 0.00393 0.0266 0.0206 0.0591
2L:9897440-9897590:minus -20.7347 1.06422 1.98165 2.87156 -18.6735 -8.57143 1.10092 1.76389 3.08257
0.00176 0.00707 0.00582 0.00948 0.0123 0.00149 0.000215 0.00937 0.00681
2L:9897440-9897590:plus -31.7347 13.3878 -3.33028 2.26606 -27.9694 -8.53061 -13.8165 7.05556 -0.724771
0.0149 0.0000869 0.0316 0.0126 0.0437 0.00136 0.00696 0.000977 0.0241
2L:9907460-9907610:minus -21.9286 4.00917 4.9633 7.00917 0.612245 -25.5306 -16.0183 -1 1.89908
0.0027 0.0024 0.00181 0.00126 0.000269 0.0205 0.0109 0.0226 0.0098
2L:9907460-9907610:plus -22.898 -0.66055 -3.52294 4.9633 -18.3469 -8.42857 -3.93578 3.15278 -2.40367
0.00442 0.0129 0.0334 0.0044 0.00894 0.00104 0.000733 0.00563 0.0376
2L:9907960-9908110:minus -23.0306 4.43119 4.14679 5.94495 -26.7041 -8.57143 -17.3211 10.0833 3.6789

0.00505 0.00204 0.00252 0.00261 0.0193 0.00149 0.0135 0.000154 0.00506
2L:9907960-9908110:plus -12 -2.88991 5.54128 7.47706 -17.7551 -18.7551 -23.2569 3.68056 3.46789
0.000305 0.025 0.00141 0.00085 0.00658 0.0141 0.0366 0.00457 0.00582
2L:9908540-9908690:minus -23.6633 10.5138 -0.990826 4.36697 -27.602 -27.1939 -1.3945 5.375
1.85321 0.00665 0.000103 0.0159 0.00505 0.0364 0.0327 0.000387 0.00222 0.01
2L:9908540-9908690:plus -11.6224 -0.495413 5.93578 10.5963 -26.9694 -36.1122 -3.73394 1.48611
2.09174 0.000259 0.0122 0.00119 0.0001 0.023 0.0878 0.000692 0.0103 0.00904
2L:9911960-9912110:minus -31.5918 5.50459 13.8257 3.44954 -9.16327 -17.9796 -19.4037 -1.38889 4.56881
0.0136 0.00132 8.99e-06 0.00738 0.00249 0.00764 0.0186 0.0252 0.00356
2L:9911960-9912110:plus -20.9592 -3.78899 0.917431 4.75229 -17.6837 -18.8265 -10.5872 -0.0833333
4.6789 0.00184 0.032 0.00851 0.00461 0.0058 0.0148 0.00344 0.0172 0.00347
2L:9912680-9912830:minus -31.7041 0.633028 -0.0550459 3.27523 1.14286 -17.8571 -19.1468 1.69444
1.90826 0.0146 0.00826 0.0118 0.00796 0.000113 0.00719 0.0179 0.0096 0.00974
2L:9912680-9912830:plus -22.2653 6.89908 8.17431 4.05505 -18.7551 -19.1224 -7.97248 -0.791667
2.22936 0.00331 0.000685 0.000399 0.00566 0.0144 0.0175 0.00209 0.0212 0.00858
2L:9915120-9915270:minus -33.9898 7.73394 -5.50459 6.29358 -17.9796 -7.79592 -22.3303 5.65278 -6.94495
0.0305 0.00044 0.0561 0.00202 0.00726 0.000723 0.0313 0.00195 0.112
2L:9915120-9915270:plus -22.7347 2.55963 2.9633 2.19266 -27.1224 -18.7857 -14.367 0.722222 0.972477
0.00403 0.00409 0.00403 0.0131 0.0245 0.0146 0.00786 0.0133 0.0144
2L:9918060-9918210:minus -23.8878 -1.13761 0.0366972 3.88991 -16.7143 -18.051 -24.6606 -5.25 -3
0.00692 0.015 0.0115 0.00608 0.00319 0.00886 0.0451 0.0651 0.0447
2L:9918060-9918210:plus -21.6735 11.945 1.75229 3.80734 -18.4898 -17.7857 -12.2294 2.81944 4.04587
0.00224 0.0000475 0.00633 0.00627 0.0116 0.0068 0.00485 0.00639 0.00421
2L:9918680-9918830:minus -31.4388 14.9908 9.34862 7.45872 -18.3776 -17.5714 -12.2936 6.65278 -2.61468
0.0122 2.1e-06 0.000207 0.000894 0.00926 0.00616 0.00492 0.0012 0.0401
2L:9918680-9918830:plus -14.2653 -0.0550459 6.00917 10.8624 -16.4184 -9.45918 -13.7798 -0.458333
-0.807339 0.00128 0.0105 0.00115 0.0000687 0.00304 0.00279 0.00691 0.0192 0.0247
2L:9921580-9921730:minus -24.2959 1.81651 2.95413 1.91743 -17.4898 -7.79592 -14.5872 -0.791667 -1
0.00781 0.00537 0.00404 0.015 0.0053 0.000723 0.00824 0.0212 0.0261
2L:9921580-9921730:plus -30.5204 -2.99083 -0.880734 2.83486 -17.8163 -8.72449 -7.89908 7.38889 -4
0.00951 0.0257 0.0154 0.0097 0.00682 0.00169 0.00206 0.000819 0.0558
2L:9938100-9938250:minus -41.8878 -2.23853 4.83486 3.47706 0.877551 -26.602 -15.3028 3.54167 -4.86239
0.0672 0.0208 0.00191 0.00724 0.00019 0.0248 0.00953 0.00483 0.0705
2L:9938100-9938250:plus -23.3776 -1.0367 5.18349 9.7156 -7.93878 0.316327 -26.9908 0.180556 -0.972477
0.00619 0.0145 0.00165 0.000242 0.000767 0.000243 0.0604 0.0158 0.0259
2L:9938740-9938890:minus -22.1122 4.87156 10.8716 5.84404 -17.7143 9.37755 -12.9541 -2.25 1.58716
0.00304 0.00172 0.0000865 0.00289 0.00627 0.0000363 0.00572 0.0319 0.0113
2L:9938740-9938890:plus -31.9694 0.330275 -1.70642 2.54128 -26.9694 -18.1633 -16.9725 2.40278 7.51376
0.0168 0.00919 0.0198 0.0112 0.023 0.01 0.0128 0.00746 0.000984
2L:9947460-9947610:minus -30.8776 -1.31193 7.16514 7.52294 -8.64286 -18.2347 -13.4679 -4.55556 2.89908
0.0103 0.0158 0.000671 0.000819 0.00139 0.0104 0.00644 0.0559 0.00722
2L:9947460-9947610:plus -4.70408 3.97959 10.0092 4.05505 -8.71429 -28.1122 1.14679 2.58333 1.34862
0.000186 0.000669 0.000141 0.00566 0.00159 0.0565 0.000212 0.00698 0.0125
2L:9954080-9954230:minus -14.1837 0.642202 13.3394 7.2844 -18.1939 -18.051 -20.7431 0.444444 -1.21101
0.00124 0.00823 0.0000148 0.00105 0.0086 0.00886 0.0235 0.0145 0.0277
2L:9954080-9954230:plus -31.7347 -0.376147 13.1651 7.29358 -18.449 -17.3469 -13.9725 6.30556
-0.908257 0.0149 0.0117 0.0000173 0.00104 0.0107 0.0058 0.00721 0.00143 0.0255
2L:9957180-9957330:minus -24.6735 0.899083 -1.77982 2.81651 -19.0204 -9.27551 -27.2018 5.08333 -0.486239
0.00825 0.00751 0.0202 0.00982 0.0158 0.00244 0.062 0.00253 0.0219
2L:9957180-9957330:plus -14.2653 6.55046 -0.917431 4.04587 1.87755 -7.42857 -23.8349 2.30556
-4.47706 0.00128 0.000814 0.0156 0.00572 0.0000492 0.000567 0.04 0.00772 0.0636
2L:9963660-9963810:minus -31.6633 -2.42202 -2.16514 5.42202 10.3571 1.31633 -14.2018 -0.486111
2.47706 0.0141 0.0219 0.0227 0.00374 0.0000208 0.000066 0.00758 0.0194 0.00801
2L:9963660-9963810:plus -23.5918 3.51376 0.40367 6.83486 -27.5306 -0.173469 -17.5413 6.69444

-4.41284 0.00656 0.00288 0.0101 0.00142 0.0351 0.000456 0.0139 0.00118 0.0624
2L:9967100-9967250:minus -12.4082 14.8532 2.46789 6.85321 -17.449 -17.2755 -16.211 -0.166667
7.83486 0.000414 2.86e-06 0.00486 0.00137 0.00484 0.00544 0.0113 0.0176 0.000712
2L:9967100-9967250:plus -24.4898 4.20183 1.59633 -0.385321 -27.1633 -7.57143 -24.3211 1.73611
2.9633 0.00807 0.00223 0.00669 0.03 0.025 0.000644 0.043 0.00946 0.00709
2LHet:169040-169190:minus -33.1122 -3.26606 0.192661 6.12844 -8.60204 19.0816 -20.1009 -0.166667
1.53211 0.0271 0.0277 0.0109 0.00231 0.00125 1.12e-06 0.021 0.0176 0.0116
2LHet:169040-169190:plus -40.1837 -1.9633 -0.0183486 3.88991 -28.4898 0.530612 -29.367 5.84722
-8.44037 0.0367 0.0192 0.0117 0.00608 0.0581 0.000203 0.0831 0.00178 0.148
2LHet:31500-31650:minus -31.9592 0.0733945 1.86239 -1.46789 -17.7143 -18.0102 -25.5688 5.84722
-1.11009 0.0167 0.0101 0.00608 0.0428 0.00627 0.00812 0.0507 0.00178 0.0269
2LHet:31500-31650:plus -24.0306 0.275229 2.84404 3.41284 -17.7449 -28.4796 -13.5229 8.125 -1.43119
0.00723 0.00938 0.00421 0.00756 0.0063 0.0617 0.00652 0.000543 0.029
2LHet:32400-32550:minus -33.6327 -1.99083 -1.00917 4.38532 -18.0816 -17.5714 -22.9908 3.48611 4.45872
0.029 0.0194 0.016 0.00501 0.00759 0.00616 0.035 0.00494 0.00366
2LHet:32400-32550:plus -24.1531 13.3878 -3.10092 2.85321 -28.4592 1.7551 -29.4954 9.20833 -7.49541
0.00744 0.0000869 0.0297 0.00955 0.0568 0.0000519 0.0847 0.00028 0.124
2LHet:32860-33010:minus -22.9286 0.302752 0.119266 1.6789 -27.2347 -7.16327 -17.4587 -1.54167 0.587156
0.0046 0.00929 0.0111 0.0165 0.0283 0.000547 0.0138 0.0263 0.016
2LHet:32860-33010:plus -31.7755 4.3578 1.65138 3.6055 -9.16327 -18.6429 -21.6422 3.88889 6.02752
0.0157 0.0021 0.00656 0.00686 0.00249 0.0139 0.0276 0.00421 0.00183
2LHet:34680-34830:minus -24.5612 7.80734 0.889908 1.84404 -17.7551 -0.0510204 -25.6972 1.29167
6.95413 0.00819 0.000424 0.00859 0.0154 0.00658 0.000429 0.0515 0.011 0.00136
2LHet:34680-34830:plus -30.9286 3.80734 2.11927 2.19266 -19.0102 0.122449 -14.6055 4.33333 1.38532
0.0104 0.00259 0.00553 0.0131 0.0157 0.000312 0.00827 0.0035 0.0123
2R:10028400-10028550:minus -22.9592 1.92661 2.26606 4.40367 -27.9286 -9.72449 -19.2661 0.236111
-0.33945 0.00475 0.00516 0.00524 0.00497 0.0412 0.00352 0.0182 0.0155 0.0209
2R:10028400-10028550:plus -34.2959 8.0367 0.944954 7.46789 -18.9796 -0.173469 -26.4771 3.59722
-4.6422 0.0316 0.000375 0.00842 0.000868 0.0155 0.000456 0.0567 0.00473 0.0662
2R:10040000-10040150:minus -32.1429 5.13761 6.7156 7.37615 -9.2449 -9.34694 -26.2477 1.18056
5.3945 0.0179 0.00154 0.000828 0.000951 0.00278 0.00261 0.0551 0.0114 0.00263
2R:10040000-10040150:plus -23.2245 -4.15596 1.74312 1.81651 -8.44898 -27.7041 -2.9633 -4.83333 -2.79817
0.00576 0.0353 0.00635 0.0156 0.00109 0.0445 0.000563 0.0595 0.0423
2R:10040540-10040690:minus -21.1837 -0.798165 0.522936 6.25688 -8.93878 -18.7143 -6.24771 4.06944
5.55046 0.00189 0.0134 0.00974 0.00208 0.00216 0.014 0.0014 0.00391 0.00241
2R:10040540-10040690:plus -3.52041 -2.93578 14.7156 3.04587 -9.0102 -8.38776 -16.1009 0.555556 1.34862
0.000076 0.0253 2.9e-06 0.00883 0.00236 0.00101 0.011 0.014 0.0125
2R:10045100-10045250:minus -32.1122 -0.165138 13.6422 7.29358 -27.1939 -9.45918 -20.8532 5.38889
-2.06422 0.0178 0.0109 0.0000116 0.00104 0.0267 0.00279 0.0239 0.0022 0.0339
2R:10045100-10045250:plus -32.7449 -5.13761 3.20183 0.834862 -18.4184 -27.602 -13.2936 3.25 -0.605505
0.0228 0.0457 0.00367 0.0215 0.0101 0.0416 0.00618 0.00542 0.023
2R:10048760-10048910:minus -33.0714 0.577982 10.2294 6.43119 -18.449 -17.5714 -23.3028 0.0972222
-2.68807 0.0267 0.00843 0.000125 0.00187 0.0107 0.00616 0.0369 0.0162 0.041
2R:10048760-10048910:plus -31.2857 3.00917 5.04587 0.697248 -9.27551 -18.2755 -14.5413 -0.5 6.07339
0.0113 0.00347 0.00174 0.0224 0.00284 0.0112 0.00816 0.0195 0.0018
2R:10051880-10052030:minus -31.3673 0.486239 3.45872 1.94495 -27.2347 -18.8265 -20.6606 1.26389
1.44037 0.0116 0.00871 0.00332 0.0147 0.0283 0.0148 0.0231 0.0111 0.012
2R:10051880-10052030:plus -41.0204 -2.00917 -0.697248 6.02752 -8.85714 -19.3061 -11.2752 0 5.79817
0.0457 0.0195 0.0145 0.00246 0.00165 0.0187 0.00395 0.0167 0.00208
2R:10052320-10052470:minus -23.7449 -0.990826 1.79817 0.770642 -7.53061 -8.16327 -5.40367 -2.38889
2.78899 0.00674 0.0143 0.00623 0.0219 0.000407 0.000783 0.00111 0.0331 0.00745
2R:10052320-10052470:plus -34.1429 2.15596 -0.256881 0.899083 -18.051 -26.5612 -19.9633 7.54167
-1.97248 0.0313 0.00474 0.0126 0.0212 0.00746 0.0237 0.0205 0.000754 0.033
2R:10056000-10056270:minus -19.5918 13.9817 -5.61468 1.78899 -17.7143 -18.0102 -15.4037 2.88889

-2.01835 0.00163 8.73e-06 0.0576 0.0158 0.00627 0.00812 0.00972 0.00622 0.0334
2R:10056000-10056270:plus -23.2245 6.07339 -0.146789 6.52294 -18.1531 -9.53061 -18.3394 5.05556
0.798165 0.00576 0.00102 0.0122 0.00174 0.00829 0.0031 0.0157 0.00256 0.0151
2R:10057900-10058050:minus -30.5102 14.6789 -0.422018 1.56881 1.54082 -9.45918 -16.0917 2.48611
7.07339 0.00947 3.91e-06 0.0133 0.0172 0.0000712 0.00279 0.011 0.00723 0.0013
2R:10057900-10058050:plus -32.551 10.2202 0.981651 5.01835 -17.7857 -17.5 -14.9174 -3.09722 0.137615
0.0207 0.00012 0.00832 0.00438 0.00676 0.00599 0.00882 0.0396 0.0178
2R:10061140-10061290:minus -14.4082 5.40367 0.633028 3.74312 -7.60204 1.05102 0.761468 0.166667
-6.27523 0.00136 0.00138 0.00938 0.00651 0.000466 0.000107 0.000235 0.0159 0.0952
2R:10061140-10061290:plus -23.5204 -3.97248 0.779817 4.08257 -8.70408 -16.7143 -10.2018 -1.83333 5.12844
0.00645 0.0336 0.00892 0.00558 0.00155 0.00419 0.0032 0.0285 0.00301
2R:10062300-10062450:minus -24.2551 -0.422018 3.83486 0.779817 -17.5612 -16.9796 -14.6881 -2.63889
5.34862 0.00768 0.0119 0.00287 0.0219 0.00544 0.0044 0.00842 0.0353 0.00271
2R:10062300-10062450:plus -31.5918 2.66972 -4.80734 2.45872 -28 -6.5 -20.2202 7.47222 9.77064 0.0136
0.00393 0.047 0.0116 0.0444 0.000542 0.0214 0.000783 0.000228
2R:10063100-10063250:minus -31.1429 1.63303 -0.00917431 7 -27.2245 -19.2755 -17.9083 9.06944
-4.19266 0.0108 0.00575 0.0116 0.00132 0.0272 0.0185 0.0147 0.000307 0.0583
2R:10063100-10063250:plus -12.1531 -2.87156 -2.15596 0.220183 -17.4082 -15.9796 -9.44037 5.84722 -3.29358
0.000352 0.0249 0.0226 0.0253 0.00421 0.00405 0.00278 0.00178 0.0474
2R:10072740-10072890:minus -13.4388 4.98165 1.3578 1.16514 -18.7143 -27.3367 -6.92661 -2.44444
5.44037 0.000879 0.00164 0.00728 0.0197 0.0136 0.0347 0.00166 0.0335 0.00259
2R:10072740-10072890:plus -4.59184 3.22936 0.853211 4.36697 -17.3776 -16.0102 -9.86239 -3.84722 1.79817
0.000166 0.0032 0.0087 0.00505 0.00419 0.00406 0.003 0.0475 0.0102
2R:1009440-1009590:minus -33.7347 -1.95413 -1.47706 3.2844 -8.67347 -17.1224 -23.2385 7.26389 -2.42202
0.0294 0.0192 0.0185 0.00793 0.00154 0.00499 0.0365 0.000876 0.0378
2R:1009440-1009590:plus -21.9286 -2.37615 -2.66972 3.9633 -8.97959 -8.72449 -21.8807 9.20833 0.33945
0.0027 0.0216 0.0263 0.0059 0.0023 0.00169 0.0289 0.00028 0.0168
2R:10104020-10104170:minus -22.8469 -1.33945 0.706422 5.21101 10.9898 -8.68367 -21.3211 2.61111
2.00917 0.00424 0.0159 0.00914 0.0042 3.28e-06 0.00157 0.0261 0.00691 0.00934
2R:10104020-10104170:plus -31.7041 0.0825688 -0.66055 2.81651 -17.1531 -9.79592 -11.6514 2.70833
-2.22936 0.0146 0.01 0.0143 0.00982 0.00364 0.00377 0.00427 0.00666 0.0356
2R:10104560-10104710:minus -31.4796 -0.981651 3.7156 3.70642 -18.051 -0.469388 -30.0367
1.18056 -3.40367 0.0128 0.0143 0.00301 0.0066 0.00746 0.000533 0.0914 0.0114 0.0486
2R:10104560-10104710:plus -31.2245 -0.963303 0.321101 5.31193 -18.9796 -18.3061 -12.9083 -3.45833
2.53211 0.0111 0.0142 0.0104 0.00393 0.0155 0.0114 0.00566 0.0433 0.00791
2R:10107220-10107370:minus -32.2857 1.11927 3.50459 5.3945 -27.6327 -7.57143 -14.2385 -5.66667
4.91743 0.0185 0.00693 0.00326 0.00379 0.0369 0.000644 0.00765 0.0711 0.00321
2R:10107220-10107370:plus -21.551 1.77064 -2.3211 3.76147 -17.7551 -7.72449 -11.5872 0.402778 -4.37615
0.00205 0.00546 0.0237 0.00645 0.00658 0.00068 0.00422 0.0147 0.0617
2R:10107700-10107850:minus -22.0816 5.7156 1.27523 6.12844 -17.4898 -9.68367 -11.6422 -4.18056
-5.22936 0.003 0.0012 0.0075 0.00231 0.0053 0.00342 0.00427 0.0513 0.0764
2R:10107700-10107850:plus 4.84694 -1.63303 2.42202 2.77982 -27.5612 -19.2755 11.055 10.0278 -5.86239
0.0000199 0.0174 0.00495 0.00997 0.0354 0.0185 8.61e-06 0.000161 0.0863
2R:10122860-10123010:minus -22.1122 -3.22018 0.981651 3.95413 10.6531 -18.0816 -12.3945 -3.41667
3.24771 0.00304 0.0274 0.00832 0.00593 0.0000102 0.00934 0.00504 0.0428 0.00636
2R:10122860-10123010:plus -32.4082 -0.385321 -2.27523 5.31193 -18.7551 -9.7551 -15.8165 3.55556
5.75229 0.0191 0.0117 0.0234 0.00393 0.0144 0.00358 0.0105 0.00481 0.00213
2R:10123140-10123290:minus -22.1122 -3.22018 0.981651 3.95413 10.6531 -18.0816 -12.3945 -3.41667
3.24771 0.00304 0.0274 0.00832 0.00593 0.0000102 0.00934 0.00504 0.0428 0.00636
2R:10123140-10123290:plus -32.7755 -0.449541 4.30275 0.53211 0.877551 -8.72449 -30.7248 -0.430556
-0.192661 0.0234 0.012 0.00237 0.0234 0.00019 0.00169 0.101 0.0191 0.0198
2R:10127180-10127330:minus -23.2245 2.04587 -2.86239 2.97248 2.14286 -26.8265 -21.4862 3.09722
0.495413 0.00576 0.00494 0.0277 0.00909 0.0000316 0.0285 0.0269 0.00575 0.0162
2R:10127180-10127330:plus -29.3673 0.688073 -2.15596 6.27523 20.4694 -17.7143 -20.8991 -3.08333 -1.68807

0.00838 0.0081 0.0226 0.00206 1.65e-07 0.00634 0.0241 0.0395 0.0308
2R:10143500-10143650:minus -31.3673 3.73394 -1.26606 2.22018 -8.33673 -28.5612 -5.83486 -5.90278
2.9633 0.0116 0.00266 0.0173 0.0129 0.00101 0.0636 0.00125 0.0746 0.00709
2R:10143500-10143650:plus -3.92857 2.27523 1.94495 -0.66055 -27.5714 -18.051 -19.2844 5.16667 -0.981651
0.000116 0.00454 0.0059 0.033 0.0357 0.00886 0.0183 0.00244 0.026
2R:10143880-10144030:minus -22.7347 -0.0458716 5.88073 1.56881 10.6939 -27.2653 -9.86239 1.625
2.16514 0.00403 0.0105 0.00122 0.0172 7.35e-06 0.0333 0.003 0.00983 0.00879
2R:10143880-10144030:plus -32.7347 3.30275 2.61468 6.89908 -27.3367 -17.8571 -15.0275 2.11111 -0.798165
0.0225 0.00311 0.0046 0.00134 0.0317 0.00719 0.00902 0.00828 0.0246
2R:10146460-10146610:minus -31.4082 -1.65138 -1.3211 2.31193 -18.9796 -9.72449 -12.8073 3.93056
-5.41284 0.0119 0.0175 0.0176 0.0123 0.0155 0.00352 0.00553 0.00413 0.0791
2R:10146460-10146610:plus -24.2653 -1.46789 11.633 7.45872 -8.64286 -9.5 -12.3303 -4.22222 6.25688
0.00773 0.0166 0.0000547 0.000894 0.00139 0.00294 0.00496 0.0518 0.00163
2R:10155400-10155550:minus -4.29592 2.57798 -1.9633 7.63303 -17.4898 -26.3367 -9.01835 -0.916667
2.99083 0.000135 0.00406 0.0214 0.000745 0.0053 0.0221 0.00257 0.022 0.00706
2R:10155400-10155550:plus -14.7041 1.22936 3.27523 -0.100917 -18.7143 -9.57143 -16.0183 -0.680556
2.00917 0.00154 0.00666 0.00357 0.0277 0.0136 0.00326 0.0109 0.0206 0.00934
2R:1015740-1015890:minus -32.0408 -2.46789 4.06422 -2.40367 -18.4592 0.0510204 -21.211 4.73611
-2.17431 0.0174 0.0222 0.00261 0.0583 0.0109 0.000349 0.0256 0.00295 0.035
2R:1015740-1015890:plus -32.0816 3.97959 4.22936 1.72477 -27.5306 -8.57143 -19.0275 0.708333 1.99083
0.0176 0.000669 0.00244 0.0161 0.0351 0.00149 0.0175 0.0134 0.00946
2R:10157840-10157990:minus -24.0306 3.97959 -1.49541 5.57798 -18.5306 -25.7551 -17.8073 1.98611
7.18349 0.00723 0.000669 0.0186 0.0034 0.0118 0.0208 0.0145 0.00866 0.00121
2R:10157840-10157990:plus -22.9898 1.88073 1 7.90826 -18.0816 9.79592 -22 -0.569444 4.99083
0.00482 0.00524 0.00827 0.000611 0.00759 0.0000183 0.0295 0.0199 0.00311
2R:10219700-10219850:minus -32.0408 2.54128 -0.963303 -1.62385 -8.86735 -17.051 -22.578 8.02778
-2.09174 0.0174 0.00411 0.0158 0.0451 0.00173 0.00479 0.0327 0.000574 0.0341
2R:10219700-10219850:plus -4.11224 -1.50459 0.752294 3.88991 -18.1939 -8.38776 -11.5046 -2.625 1.31193
0.000126 0.0168 0.009 0.00608 0.0086 0.00101 0.00414 0.0351 0.0127
2R:10239660-10239810:minus -32.0714 2.3945 4.73394 2.27523 -18.0102 -17.051 -17.1376 2.16667
-6.58716 0.0176 0.00434 0.00199 0.0125 0.00732 0.00479 0.0131 0.00812 0.103
2R:10239660-10239810:plus -33.1429 3.73394 0.908257 5.85321 -18.4184 -18.051 -8.0367 5.80556 -4.85321
0.0272 0.00266 0.00853 0.00287 0.0101 0.00886 0.00212 0.00182 0.0703
2R:10244700-10244850:minus -13.1837 12.5413 6.74312 1.82569 -17.7143 -27.5612 -17.3119 6.58333
0.348624 0.000705 0.0000316 0.000818 0.0156 0.00627 0.0393 0.0135 0.00124 0.0167
2R:10244700-10244850:plus -13.3673 3.14679 2.57798 5.98165 -17.5204 -0.0204082 -4.89908 6.375
3.41284 0.000813 0.00329 0.00466 0.00258 0.00532 0.000411 0.000965 0.00138 0.006
2R:10247700-10247850:minus -23.1837 -6.31193 4.82569 4.33945 -8.67347 -37.6633 -12.5596 -7.94444
4.78899 0.00553 0.0617 0.00191 0.00509 0.00154 0.171 0.00523 0.11 0.00336
2R:10247700-10247850:plus -22.5102 -2.51376 -1.44954 0.275229 -9.20408 9.60204 -12.5321 2.61111 11.4771
0.00354 0.0225 0.0183 0.025 0.00262 0.0000248 0.00519 0.00691 0.0000679
2R:10249320-10249470:minus -39.2959 3.56881 4.12844 1.56881 -26.2653 -0.204082 -18.2018 2.80556
9.56881 0.0331 0.00282 0.00254 0.0172 0.018 0.000463 0.0154 0.00642 0.000303
2R:10249320-10249470:plus -31.7755 5.82569 5.42202 6.72477 -18.449 -27.3367 -10.3303 -1.30556 9.56881
0.0157 0.00114 0.00149 0.00154 0.0107 0.0347 0.00328 0.0246 0.000303
2R:10250380-10250530:minus -30.7347 4.97959 13.9541 3.43119 0.846939 -19.2755 -20.6881 4.20833
-0.33945 0.01 0.000156 7.63e-06 0.00749 0.000197 0.0185 0.0232 0.00369 0.0209
2R:10250380-10250530:plus -30.6735 9.11927 -2.43119 4.22018 -8.93878 -18.0102 -16.0459 -2.02778 -0.733945
0.00978 0.00021 0.0245 0.00531 0.00216 0.00812 0.0109 0.03 0.0242
2R:10299200-10299350:minus -12.2143 0.743119 2.85321 5.49541 10.6939 0.122449 1.83486 1.63889
0.0825688 0.000367 0.00794 0.0042 0.00355 7.35e-06 0.000312 0.000174 0.00978 0.0181
2R:10299200-10299350:plus -33.0714 2.44954 -0.0825688 2.97248 -27.1224 -0.0204082 -7.91743 4.31944
1.2844 0.0267 0.00425 0.0119 0.00909 0.0245 0.000411 0.00207 0.00352 0.0129
2R:1032140-1032290:minus -32.7041 -0.449541 3.54128 -0.0183486 -18.7449 -18.2755 -11.0275 6.02778

-0.33945 0.0222 0.012 0.00322 0.027 0.0138 0.0112 0.00375 0.00164 0.0209
2R:1032140-1032290:plus -23.7041 6.93578 -0.522936 5.86239 -27.4592 -0.316327 -14.2752 5.79167
-7.85321 0.00669 0.000671 0.0137 0.00282 0.0329 0.000524 0.00771 0.00183 0.132
2R:1032800-1032950:minus -34.1122 6.54128 4.51376 2.44037 -17.7143 -9.5 -11.0642 0.833333 7.25688
0.0312 0.000818 0.00218 0.0116 0.00627 0.00294 0.00378 0.0128 0.00115
2R:1032800-1032950:plus -32.6224 4 15.6606 5.34862 -18.6837 -7.23469 -12.3486 -1.65278 4.80734
0.0211 0.0024 3.94e-07 0.00387 0.0126 0.000557 0.00498 0.0271 0.00334
2R:10369380-10369530:minus -31.6633 4.99083 1.50459 7.3578 -8.33673 -26.898 -12.6697 9.375
10.3394 0.0141 0.00164 0.00692 0.000965 0.00101 0.0302 0.00536 0.000251 0.000112
2R:10369380-10369530:plus -32 -1.80734 -1.55046 -0.302752 1.36735 -28.8571 -12.1927 5.83333 1.70642
0.0171 0.0184 0.0189 0.0293 0.0000781 0.0699 0.00481 0.00179 0.0108
2R:10369760-10369910:minus -23.4592 2.58716 -1.09174 2.2844 -8.0102 -9.53061 -23.2661 1.04167
3.88073 0.00631 0.00404 0.0164 0.0124 0.000826 0.0031 0.0366 0.012 0.00475
2R:10369760-10369910:plus -24.3367 3.10092 -1.01835 2.98165 -17.9388 -26.8571 -14.5596 0.513889 3.93578
0.00789 0.00335 0.0161 0.00905 0.00715 0.0288 0.00819 0.0142 0.00456
2R:10371380-10371530:minus -31.5816 3.57143 7.81651 10.8624 -17.4898 0.0918367 -13.1927 0.930556
3.31193 0.0135 0.00175 0.000481 0.0000687 0.0053 0.000323 0.00604 0.0124 0.00618
2R:10371380-10371530:plus -22.3673 -0.247706 3.56881 5.31193 -17.9796 -9.02041 -18.3853 -1.51389
4.87156 0.00341 0.0112 0.00318 0.00393 0.00726 0.00213 0.0159 0.0261 0.00325
2R:10371860-10372010:minus -22 -0.266055 -3.02752 6.00917 -18.7551 -8.27551 -19.0459 7.69444
-4.29358 0.00291 0.0113 0.0291 0.00255 0.0144 0.000919 0.0176 0.000693 0.06
2R:10371860-10372010:plus -24.8265 -1.66972 -0.458716 1.12844 -17.3776 -8.79592 -17.1835 5.29167
3.72477 0.0083 0.0176 0.0135 0.0198 0.00419 0.0019 0.0132 0.0023 0.00497
2R:10372400-10372550:minus -23.5204 7.2844 2.83486 10.5963 -26.4898 -17.051 -19.156 2.77778
1.88991 0.00645 0.000559 0.00423 0.0001 0.0183 0.00479 0.0179 0.00649 0.00982
2R:10372400-10372550:plus -32.449 1.17431 0.220183 3.95413 -27.7959 9.72449 -17.2661 0.777778 1.88991
0.0195 0.00679 0.0108 0.00593 0.0386 0.0000214 0.0134 0.0131 0.00982
2R:10373600-10373750:minus -31.7755 0.137615 1.04587 9.23853 -18.7143 0.244898 -10.2385 -4.05556
1.36697 0.0157 0.00984 0.00813 0.000424 0.0136 0.000285 0.00322 0.0499 0.0124
2R:10373600-10373750:plus -21.9592 -0.587156 10.211 1.43119 -17.7857 -28.6327 -9.06422 0.833333
3.15596 0.00279 0.0126 0.000126 0.0181 0.00676 0.066 0.00259 0.0128 0.00659
2R:10375700-10375850:minus -31.6327 1.6055 2.31193 3.25688 -18.449 0.0510204 -15.3761 0.916667
3.88991 0.0138 0.0058 0.00515 0.00804 0.0107 0.000349 0.00966 0.0125 0.00467
2R:10375700-10375850:plus -23.449 -0.357798 -2.2844 4.6055 -18.449 -18.051 -17.0642 5.27778
4.0367 0.00631 0.0116 0.0235 0.00476 0.0107 0.00886 0.0129 0.00232 0.00424
2R:10378340-10378490:minus -22.5918 0.844037 10.5229 4.81651 -16.2245 -18.7551 -18.5688 -2.26389
-2.3211 0.00365 0.00766 0.000104 0.00451 0.00297 0.0141 0.0163 0.032 0.0365
2R:10378340-10378490:plus -21.6939 0.376147 8.41284 9.49541 -27.0408 -8.42857 -9.44954 1.375 4.98165
0.00226 0.00905 0.000351 0.000308 0.0242 0.00104 0.00278 0.0107 0.00313
2R:10390940-10391090:minus -33.0714 13.3878 9.11927 4.07339 -9.20408 -18.6327 -22.2752 -1.19444
1.59633 0.0267 0.0000869 0.000237 0.00562 0.00262 0.0137 0.031 0.0239 0.0113
2R:10390940-10391090:plus -13.4082 14.7339 8.72477 3.90826 -27.1224 -17.9796 -9.38532 0.986111 -2.53211
0.000867 3.57e-06 0.000294 0.00603 0.0245 0.00764 0.00275 0.0122 0.0392
2R:10391640-10391790:minus -22.9898 -1.9633 5.6422 0.733945 -7.85714 -25.8265 -8.59633 2.61111
-2.26606 0.00482 0.0192 0.00135 0.0222 0.000594 0.0212 0.00237 0.00691 0.0359
2R:10391640-10391790:plus -31.9592 0.669725 1.31193 5.31193 -17.6735 -7.42857 -12.6972 3.25 7.25688
0.0167 0.00815 0.00741 0.00393 0.00572 0.000567 0.00539 0.00542 0.00115
2R:10391960-10392110:minus -42.2449 -1.80734 0.0733945 -2.15596 -17.7449 0.755102 -9 3.23611
-0.862385 0.0715 0.0184 0.0113 0.0539 0.0063 0.000175 0.00256 0.00545 0.0251
2R:10391960-10392110:plus -41.398 2.66972 7.74312 4.6055 -19.051 -19.3469 -10.3303 5.11111 5.75229
0.0554 0.00393 0.000501 0.00476 0.0162 0.0194 0.00328 0.0025 0.00213
2R:10405460-10405610:minus -30.8163 -2.6789 13.8532 2.68807 -18.7551 -7.20408 -21.4862 0.0277778
-1.89908 0.0103 0.0236 8.77e-06 0.0104 0.0144 0.000548 0.0269 0.0166 0.0323
2R:10405460-10405610:plus -41.5102 -4.29358 1.22018 1.38532 -17.1837 -37.5204 -10.4495 4.73611 -1.53211

0.0582 0.0366 0.00765 0.0185 0.00366 0.166 0.00335 0.00295 0.0296
2R:10414020-10414170:minus -31.7041 5.55963 10.7248 1.87156 -27.5306 -17.2755 -7.52294 4.81944
4.10092 0.0146 0.00129 0.0000934 0.0152 0.0351 0.00544 0.0019 0.00284 0.00413
2R:10414020-10414170:plus -21.8163 -5.65138 -0.211009 2.94495 -8.64286 -17.7143 -23.2294 1.43056
-2.30275 0.00244 0.0523 0.0124 0.0092 0.00139 0.00634 0.0364 0.0105 0.0363
2R:10414280-10414430:minus -22.9286 -0.889908 -2.57798 5.59633 -17.7143 -8.7551 -17.8807 0.875
6.04587 0.0046 0.0138 0.0256 0.00331 0.00627 0.00172 0.0147 0.0127 0.00181
2R:10414280-10414430:plus -31.8469 -4.42202 1.23853 -1.34862 -26.5918 -25.7551 -7.10092 4.90278 4.80734
0.0162 0.0379 0.0076 0.0413 0.0186 0.0208 0.00173 0.00274 0.00334
2R:10460500-10460650:minus -41.2143 6.14679 6.18349 8.98165 -17.4184 -18.3469 -22.5963 5.27778
1.27523 0.0494 0.00099 0.00106 0.000461 0.00457 0.0124 0.0328 0.00232 0.013
2R:10460500-10460650:plus -32.2959 6.11927 6.11927 4.0367 -7.93878 -17.3061 -14.3761 5.47222 -2.27523
0.0186 0.001 0.00109 0.00578 0.000767 0.00548 0.00788 0.00212 0.036
2R:10464560-10464710:minus -41.6224 2.3945 3.73394 3.45872 -17.8265 -17.2755 -25.1101 5.02778
5.84404 0.0628 0.00434 0.00299 0.00731 0.00691 0.00544 0.0478 0.00259 0.00202
2R:10464560-10464710:plus -30.9694 3.44037 6.42202 1.51376 -27.4898 -17.6429 -15.0734 1.44444 1.79817
0.0104 0.00296 0.000953 0.0175 0.0336 0.00619 0.00911 0.0105 0.0102
2R:10488660-10488810:minus -21.4796 13.3878 4.46789 2.17431 -17.1531 -18.2041 -4.33945 2.33333
-0.495413 0.00202 0.0000869 0.00222 0.0132 0.00364 0.0103 0.000822 0.00765 0.022
2R:10488660-10488810:plus -31.898 8.65138 14.1927 7.29358 -27.3061 -9.42857 -19.7156 -0.833333
-4.07339 0.0164 0.000269 5.79e-06 0.00104 0.0315 0.0027 0.0196 0.0215 0.0566
2R:10491740-10491890:minus -13.2245 13.3878 0.944954 3.56881 -17.7959 -16.9796 -10.7982 1.22222
3.83486 0.000738 0.0000869 0.00842 0.00699 0.00678 0.0044 0.00359 0.0113 0.00481
2R:10491740-10491890:plus -31.9286 7.40367 -0.889908 4.04587 -27.1939 -8.60204 -15.0642 1.06944
3.29358 0.0166 0.000524 0.0154 0.00572 0.0267 0.00151 0.00909 0.0119 0.00623
2R:10492320-10492470:minus -30.5918 -2.62385 3.40367 3.27523 -18.5204 -18.0816 -19.055 7.06944
7.83486 0.00963 0.0232 0.0034 0.00796 0.0117 0.00934 0.0176 0.00097 0.000712
2R:10492320-10492470:plus -21.6327 -2.94495 3.25688 1.11009 0.653061 -18.8571 -20.6514 -4.73611 3.66972
0.00215 0.0254 0.0036 0.0199 0.000239 0.0151 0.0231 0.0582 0.00514
2R:10492860-10493010:minus -23.5204 1.12844 5.7156 10.5963 -19.0204 -17.2755 -8.98165 3.81944
0.963303 0.00645 0.00691 0.00131 0.0001 0.0158 0.00544 0.00255 0.00433 0.0145
2R:10492860-10493010:plus -31.7755 -0.614679 2.6422 3.19266 -27.2347 -16.2755 -16.9541 8.27778
-1.3945 0.0157 0.0127 0.00455 0.00829 0.0283 0.00413 0.0127 0.000497 0.0288
2R:10505580-10505730:minus -21.7755 -1.94495 4.98165 5.30275 1.14286 -0.173469 -19.6789 1.18056
7.37615 0.00241 0.0191 0.00179 0.00396 0.000113 0.000456 0.0195 0.0114 0.00107
2R:10505580-10505730:plus -38.6939 -0.201835 -4.31193 7.80734 -18.449 -9.02041 -16.5505 8.43056
-4.45872 0.0321 0.011 0.0413 0.00063 0.0107 0.00213 0.0119 0.000455 0.0633
2R:10506180-10506330:minus -41.6735 1.73394 -3.57798 0.321101 -27.1224 -8.72449 -32.1468 6.125
-8.75229 0.0637 0.00553 0.0339 0.0247 0.0245 0.00169 0.12 0.00156 0.158
2R:10506180-10506330:plus -22.8469 -0.100917 -2.65138 11.0183 -17.7143 -8.72449 -9.49541 1.375
3.6789 0.00424 0.0107 0.0261 0.000052 0.00627 0.00169 0.0028 0.0107 0.00506
2R:10509020-10509170:minus -30.398 1.17431 2.24771 1.91743 -9.16327 -18.2347 -20.4404 2.47222
-1.24771 0.00901 0.00679 0.00527 0.015 0.00249 0.0104 0.0222 0.00727 0.028
2R:10509020-10509170:plus -11.8878 -0.972477 3.73394 -0.715596 -27.7041 -8.42857 -15.0367 13.7083
3.66972 0.00029 0.0142 0.00299 0.0336 0.038 0.00104 0.00904 4.08e-06 0.00514
2R:10509420-10509570:minus -31.3673 -4.33945 7.05505 10.8624 -17.9388 -0.0204082 -13.0459 2.88889
-0.174312 0.0116 0.037 0.000708 0.0000687 0.00715 0.000411 0.00584 0.00622 0.0196
2R:10509420-10509570:plus -34.0816 -2.0367 1.47706 6.44037 -17.4184 -17.2755 -25.0092 -0.361111
-1.85321 0.0311 0.0196 0.00698 0.00185 0.00457 0.00544 0.0472 0.0187 0.032
2R:10532600-10532750:minus -34.3367 0.715596 -3.18349 0.211009 -18.9388 0.979592 -34.9908 6.13889
-8.54128 0.0318 0.00802 0.0304 0.0254 0.0151 0.000136 0.16 0.00155 0.152
2R:10532600-10532750:plus -22.4694 -0.0275229 -3.38532 0.137615 0.836735 -10.0204 -24.4495 -0.944444
-2.83486 0.0035 0.0104 0.0321 0.0259 0.000215 0.00399 0.0438 0.0222 0.0427
2R:10639780-10639930:minus -30.3673 8.3945 0.954128 4.0367 -27.2245 -18.8571 -17.5046 3.04167

-0.183486 0.00897 0.000308 0.0084 0.00578 0.0272 0.0151 0.0139 0.00587 0.0197
2R:10639780-10639930:plus -23.4082 13.3878 -0.816514 2.66972 -17.3776 -8.7551 -19.3028 1.34722
-6.98165 0.00623 0.0000869 0.0151 0.0106 0.00419 0.00172 0.0183 0.0108 0.113
2R:10643920-10644070:minus -31.4388 13.4592 -2.23853 1.89908 -17.3776 -8.79592 -12.2936 -2.70833
-6.06422 0.0122 0.000064 0.0232 0.0151 0.00419 0.0019 0.00492 0.0359 0.0902
2R:10643920-10644070:plus -30.4796 -1.49541 12.367 4.04587 -7.63265 19.1531 -21.7798 5.22222 -2.15596
0.00941 0.0167 0.0000334 0.00572 0.000479 7.95e-07 0.0283 0.00238 0.0348
2R:10645380-10645530:minus -24.2653 -1.24771 -1.12844 2.0367 -7.63265 -9.5 -8.94495 2.75 5.07339
0.00773 0.0155 0.0166 0.0141 0.000479 0.00294 0.00253 0.00656 0.00305
2R:10645380-10645530:plus -31.7755 2.06422 3.89908 2.84404 -9.5 -9.57143 -12.2844 3.90278 1.16514
0.0157 0.0049 0.00279 0.00961 0.00293 0.00326 0.00491 0.00418 0.0135
2R:10656280-10656430:minus -13.4082 -0.46789 4.24771 1.89908 -18.7143 -18.8265 -4.10092 3.47222
9.87156 0.000867 0.0121 0.00243 0.0151 0.0136 0.0148 0.000768 0.00497 0.000217
2R:10656280-10656430:plus -20.5918 2.87156 2.01835 1.41284 -18.7143 -9.79592 -21.7798 3.05556 -7.16514
0.00169 0.00365 0.00574 0.0183 0.0136 0.00377 0.0283 0.00584 0.117
2R:10656860-10657010:minus -3.22449 1.21101 -1.88991 3.33028 20.4694 0.908163 -15.3945 3.95833
5.95413 0.0000495 0.00671 0.0209 0.00778 1.65e-07 0.000143 0.0097 0.00409 0.00192
2R:10656860-10657010:plus -31.7347 2.55046 -3.36697 -0.137615 -8.97959 1.05102 -22.1927 5.61111
-2.47706 0.0149 0.0041 0.032 0.0279 0.0023 0.000107 0.0305 0.00199 0.0385
2R:10657860-10658010:minus -32.0714 14.0459 3.61468 8.61468 -26.9286 -8.79592 -24.0459 4.52778
-3.08257 0.0176 7.98e-06 0.00313 0.000552 0.0214 0.0019 0.0413 0.00322 0.0455
2R:10657860-10658010:plus -31.6735 -0.899083 3.61468 3.94495 -18.4898 1.05102 -24.5872 -0.166667
-5.59633 0.0141 0.0139 0.00313 0.00595 0.0116 0.000107 0.0446 0.0176 0.0819
2R:10658360-10658510:minus -23.1224 8.57798 0.779817 6.25688 -26.7041 -9.30612 -12.3394 -2.95833
6.17431 0.00531 0.00028 0.00892 0.00208 0.0193 0.00257 0.00497 0.0382 0.00171
2R:10658360-10658510:plus -41.2551 9.11927 -2.78899 3.44037 -18.7143 -9.53061 -22.6514 5.73611 -1.56881
0.0513 0.00021 0.0272 0.00746 0.0136 0.0031 0.0331 0.00188 0.0298
2R:10658740-10658890:minus -14.0816 -0.688073 1.81651 2.54128 -18.4184 -9.23469 -5.33028 1.80556
4.14679 0.00117 0.013 0.00619 0.0112 0.0101 0.00238 0.00109 0.00923 0.00406
2R:10658740-10658890:plus -22.1837 0.954128 2.21101 2.3578 -9.0102 -18.8571 -12.7523 6.98611 3.93578
0.00315 0.00736 0.00534 0.0121 0.00236 0.0151 0.00546 0.00101 0.00456
2R:10735700-10735850:minus -13.8878 0.844037 3.85321 3.18349 -18.4796 -18.8265 -17.3028 -1 6.7156
0.00111 0.00766 0.00284 0.00837 0.011 0.0148 0.0134 0.0226 0.00144
2R:10735700-10735850:plus -13.449 0.110092 0.752294 6.37615 -27.5 0.0918367 -22.8532 1.09722
-3.98165 0.000914 0.00993 0.009 0.00192 0.0337 0.000323 0.0342 0.0118 0.0555
2R:10740120-10740270:minus -32 9.74312 3.33028 6.69725 20.1735 -8.45918 -13.9908 1.22222 3.88991
0.0171 0.000152 0.00349 0.00156 5.68e-07 0.00111 0.00724 0.0113 0.00467
2R:10740120-10740270:plus -22.9184 -3.33945 6.40367 9.59633 -18.7143 1.09184 -15.945 4.65278 -2.98165
0.00447 0.0283 0.000962 0.000275 0.0136 0.000091 0.0107 0.00305 0.0445
2R:10742860-10743010:minus -33.9286 6.12844 -3.73394 2.19266 -26.2653 -8.72449 -21.4587 1.23611
-0.550459 0.0303 0.000997 0.0353 0.0131 0.018 0.00169 0.0267 0.0112 0.0225
2R:10742860-10743010:plus -22.1122 -0.963303 -0.220183 3.30275 0.877551 0.122449 -20.3028 -4.05556
-3.91743 0.00304 0.0142 0.0125 0.00789 0.00019 0.000312 0.0217 0.0499 0.0544
2R:10746100-10746250:minus -21.9592 4.66055 2.97248 5.29358 20.2041 -17.7551 -16.3761 0.333333
-2.78899 0.00279 0.00187 0.00401 0.00398 3.64e-07 0.00643 0.0116 0.0151 0.0422
2R:10746100-10746250:plus -31.7041 6.27523 0.119266 6.09174 -18.8265 -18.4184 -20.1468 2.70833 -0.256881
0.0146 0.00093 0.0111 0.00235 0.0149 0.0127 0.0211 0.00666 0.0204
2R:10748260-10748410:minus -23.1837 0.458716 14.1284 6.72477 -17.1531 -18.0102 -13.945 6.33333
-2.6422 0.00553 0.00879 6.14e-06 0.00154 0.00364 0.00812 0.00717 0.00141 0.0405
2R:10748260-10748410:plus -31.8469 0.825688 15.7339 7.29358 1.14286 9.30612 -17.2202 5.23611 -0.12844
0.0162 0.00771 2.45e-07 0.00104 0.000113 0.0000398 0.0133 0.00236 0.0193
2R:10758160-10758310:minus -12.9286 3.11927 -1.3578 3.33945 -18.4184 -19.0816 -17.3945 1.76389
0.0825688 0.000539 0.00333 0.0178 0.00774 0.0101 0.017 0.0136 0.00937 0.0181
2R:10758160-10758310:plus -33.6224 3.64286 -2.3211 2.21101 -9.5 -18.0816 -20.7156 1.05556 -1.2844

0.0289	0.00159	0.0237	0.0129	0.00293	0.00934	0.0234	0.0119	0.0283
2R:10758460-10758610:minus	-22.8469	1.05505	7.76147	7.37615	2.14286	-18.051	-21.8257	6.58333
2.47706	0.00424	0.00709	0.000497	0.000951	0.0000316	0.00886	0.0286	0.00124	0.00801
2R:10758460-10758610:plus	-33.3061	-2.79817	-4.77982	4.22936	-18.7143	-8.94898	-24.0092	6.33333	3.46789
0.0279	0.0244	0.0467	0.00525	0.0136	0.00203	0.0411	0.00141	0.00582
2R:10761320-10761470:minus	-40.4082	3.61224	1.43119	5.59633	-8.86735	-18.2755	-22.2661	4.04167
-3.7156	0.0399	0.00167	0.0071	0.00331	0.00173	0.0112	0.0309	0.00395	0.0518
2R:10761320-10761470:plus	-23.0408	-1.01835	1.85321	9.3211	-18.6837	-8.23469	-20.4862	3.63889	-1.6422
0.00512	0.0144	0.0061	0.000391	0.0126	0.000875	0.0224	0.00465	0.0304
2R:10768920-10769070:minus	-22.4898	3.55046	3.6422	3.87156	-18.4898	-8.86735	-12.4771	5.91667
-0.238532	0.00353	0.00284	0.00309	0.00618	0.0116	0.00198	0.00513	0.00172	0.0203
2R:10768920-10769070:plus	-31.8571	-0.183486	3.31193	9.33028	-17.1531	-8.72449	-12.5688	2.31944
11.8532	0.0163	0.011	0.00352	0.000374	0.00364	0.00169	0.00524	0.00769	0.0000428
2R:10769480-10769630:minus	-30.4796	-0.825688	8.61468	4.17431	-18.7551	-0.469388	-24.9908
5.65278	6.07339	0.00941	0.0136	0.000312	0.00537	0.0144	0.000533	0.0471	0.00195	0.0018
2R:10769480-10769630:plus	-33.2653	0.807339	7.7156	10.8624	-18.6837	-17.7143	-21.5138	13.7917	-0.559633
0.0277	0.00776	0.000509	0.0000687	0.0126	0.00634	0.027	3.59e-06	0.0226
2R:10772040-10772190:minus	-31.5204	2.31193	5.15596	2.41284	-17.9388	9.30612	-21.3945	-1.02778
-7.55963	0.0131	0.00448	0.00167	0.0118	0.00715	0.0000398	0.0264	0.0227	0.125
2R:10772040-10772190:plus	-32	1.18349	0.981651	1.68807	-17.7143	-17.8571	-14.5963	7.97222	0.798165
0.0171	0.00677	0.00832	0.0164	0.00627	0.00719	0.00826	0.000593	0.0151
2R:10772360-10772510:minus	-23.2959	-4.13761	4.14679	2.83486	-18.449	-9.30612	-11.3761	3.58333
1.22018	0.00605	0.0351	0.00252	0.0097	0.0107	0.00257	0.00403	0.00475	0.0133
2R:10772360-10772510:plus	-32	1.18349	0.981651	1.68807	-17.7143	-9.34694	-23.1101	7.97222	-4.19266
0.0171	0.00677	0.00832	0.0164	0.00627	0.00261	0.0357	0.000593	0.0583
2R:10776580-10776730:minus	-21.9286	7.50459	5.44037	7.16514	-26.9694	-19.0102	-15.0183	8.16667
1.27523	0.0027	0.000498	0.00148	0.00116	0.023	0.0159	0.00901	0.00053	0.013
2R:10776580-10776730:plus	-32.7347	3.57143	-0.266055	3.33028	-18.7551	-8.7551	-22.1009	3.05556
4.25688	0.0225	0.00175	0.0126	0.00778	0.0144	0.00172	0.03	0.00584	0.00391
2R:10786940-10787090:minus	-24.1531	5.6422	-0.669725	4.86239	-17.6837	-17.2755	0.550459	-0.513889
2.2844	0.00744	0.00124	0.0144	0.00449	0.0058	0.00544	0.000248	0.0196	0.00842
2R:10786940-10787090:plus	-14.4796	-1.69725	6.34862	5.31193	-18.3469	-17.9796	-0.972477	1.09722
-1.22018	0.00142	0.0178	0.000987	0.00393	0.00894	0.00764	0.000353	0.0118	0.0278
2R:10793680-10793830:minus	-32.2551	-2.26606	3.02752	7.43119	-17.449	-17.7551	-15.8991	0.333333
-3.04587	0.0184	0.021	0.00393	0.000939	0.00484	0.00643	0.0107	0.0151	0.0451
2R:10793680-10793830:plus	-22.2245	-1.16514	2.44954	6.6422	-17.3776	-9.27551	-9.77064	4.45833	7.42202
0.00325	0.0151	0.00489	0.00161	0.00419	0.00244	0.00295	0.00332	0.00106
2R:10795620-10795770:minus	-34.2959	1.58716	3.37615	5.94495	-8.90816	-17.4898	6.77982	9.90278
7.42202	0.0316	0.00584	0.00343	0.00261	0.00192	0.0059	0.000033	0.000176	0.00106
2R:10795620-10795770:plus	-21.8469	1.01835	-0.559633	1.88073	-27.3061	-28.1224	-2.34862	2.25	10.1284
0.00247	0.00719	0.0139	0.0152	0.0315	0.0583	0.000482	0.00788	0.000129
2R:10823080-10823230:minus	-13.1735	11.3211	6.88073	6.25688	-8.40816	-17.7143	-3.62385	2.04167
1.27523	0.000671	0.0000686	0.000768	0.00208	0.00108	0.00634	0.000671	0.00849	0.013
2R:10823080-10823230:plus	-21.6633	-2.88073	3.14679	1.40367	-19.0102	-9.23469	-20.8807	-4.29167	-0.53211
0.00223	0.0249	0.00375	0.0184	0.0157	0.00238	0.0241	0.0527	0.0222
2R:10850460-10850610:minus	-22.5102	1.57798	0.330275	4.10092	-17.6429	10.0918	-20.3394	1.52778
0.440367	0.00354	0.00586	0.0104	0.00554	0.00562	9.34e-06	0.0218	0.0102	0.0164
2R:10850460-10850610:plus	-33.2245	2.44037	5.43119	3.88991	10.398	-8.5	-12.2661	3.70833	-0.743119
0.0276	0.00427	0.00148	0.00608	0.0000153	0.00126	0.00489	0.00452	0.0243
2R:10868640-10868790:minus	-40	-2.27523	1.25688	2.40367	-27.7551	1.05102	-23.0275	5.15278	-3.50459
0.0352	0.021	0.00755	0.0119	0.0385	0.000107	0.0352	0.00245	0.0497
2R:10868640-10868790:plus	-12.5918	-1.80734	3.05505	6.29358	10.9184	-8.45918	-15.1193	-2.54167	0.0825688
0.000449	0.0184	0.00389	0.00202	4.19e-06	0.00111	0.00919	0.0344	0.0181
2R:10870000-10870150:minus	-32.6633	-0.330275	0.40367	4.9633	-26.1939	-27.051	-16.9908	4.29167

7.22018 0.0216 0.0115 0.0101 0.0044 0.0175 0.0315 0.0128 0.00356 0.00119
2R:10870000-10870150:plus -40.3571 -2.06422 -3.86239 0.146789 -18.1837 -19.6429 -12.1284 3.30556 12.2752
0.0394 0.0198 0.0366 0.0258 0.00835 0.0204 0.00474 0.0053 0.0000141
2R:10879760-10879910:minus -21.3265 -0.0550459 6.77982 8.05505 -8.64286 -17.5 -6.69725 4.23611
0.614679 0.00193 0.0105 0.000804 0.000578 0.00139 0.00599 0.00157 0.00364 0.0158
2R:10879760-10879910:plus -32.7347 13.3878 0.256881 0.862385 11.6531 0.244898 -11.4037
4.65278 -2.22936 0.0225 0.0000869 0.0106 0.0214 1.55e-06 0.000285 0.00406 0.00305 0.0356
2R:10881300-10881450:minus -23.0714 4.74312 0.220183 1.44954 1.17347 -18.3469 -16.8349
1.26389 -3.07339 0.0052 0.00181 0.0108 0.0179 0.000101 0.0124 0.0125 0.0111 0.0454
2R:10881300-10881450:plus -23.5204 -0.422018 8.62385 5.57798 -27.2959 -9.5 -10.7798
2.77778 0.908257 0.00645 0.0119 0.000311 0.0034 0.0303 0.00294 0.00357 0.00649 0.0147
2R:11016220-11016370:minus -41.4796 -3.18349 -1.73394 2.19266 -28 -8.72449 -28.6239
4.38889 -6.69725 0.0577 0.0271 0.02 0.0131 0.0444 0.00169 0.0749 0.00342 0.106
2R:11016220-11016370:plus 6.5102 4.54128 -0.825688 9.92661 -7.26531 0.27551 -8.66972 -5.36111
-3.81651 2.66e-06 0.00195 0.0151 0.000169 0.000375 0.000257 0.0024 0.0667 0.0529
2R:11022940-11023090:minus -32.4796 1.3578 8.10092 3.21101 -17.6735 -18.1224 -17.156 0.541667
9.66055 0.02 0.00635 0.000415 0.00822 0.00572 0.00988 0.0131 0.0141 0.000268
2R:11022940-11023090:plus -13.8163 3.64286 1.79817 1.62385 -17.6735 -9.42857 -21.789 -4.59722
-0.706422 0.00109 0.00159 0.00623 0.0168 0.00572 0.0027 0.0284 0.0564 0.024
2R:11033020-11033170:minus -20.8571 -0.311927 1.36697 5.57798 10.6939 -9.23469 -17.1835
0.152778 -3.40367 0.00178 0.0115 0.00726 0.0034 7.35e-06 0.00238 0.0132 0.016 0.0486
2R:11033020-11033170:plus -32.9694 3.31193 1.44954 3.86239 -18.4898 10.2347 -5.80734 2.38889
-4.13761 0.0254 0.0031 0.00705 0.00622 0.0116 7.75e-06 0.00124 0.00749 0.0575
2R:11035280-11035430:minus -22.2959 0.642202 2.58716 2.70642 10.6531 -18.9796 -19.0459
-1.27778 -3.42202 0.00335 0.00823 0.00465 0.0103 0.0000102 0.0155 0.0176 0.0244 0.0488
2R:11035280-11035430:plus -23.4898 1.7156 16.2202 3.08257 -18.4184 -8.72449 -25.0183
1.33333 -4.24771 0.00639 0.00557 1.24e-07 0.0087 0.0101 0.00169 0.0473 0.0109 0.0591
2R:11088680-11088830:minus -23.4898 13.8349 -1.33945 7.33945 -18.6837 0.387755 -16.3028
6.48611 -5.12844 0.00639 0.0000102 0.0177 0.00102 0.0126 0.000217 0.0114 0.00131 0.0748
2R:11088680-11088830:plus -24.7857 2.46789 6 0.926606 -8.97959 -26.8265 -22.422 4.97222
1.6422 0.00829 0.00422 0.00115 0.0209 0.0023 0.0285 0.0318 0.00266 0.0111
2R:11101100-11101250:minus -31.5102 11.8807 -0.0917431 -1.17431 -27.9592 -17.9388
-9.19266 -0.75 5.59633 0.013 0.0000494 0.0119 0.0388 0.0416 0.00738 0.00265 0.021 0.00234
2R:11101100-11101250:plus -31 12.1927 2.36697 6.21101 -17.7143 -8.5 -13.2202 2.18056 -4.63303
0.0105 0.000041 0.00505 0.00213 0.00627 0.00126 0.00608 0.00808 0.0661
2R:11103640-11103790:minus -32.9694 -0.954128 4.43119 9.3211 -18.7143 -8.94898 -29.1009
-0.388889 -5.65138 0.0254 0.0141 0.00225 0.000391 0.0136 0.00203 0.0801 0.0188 0.0826
2R:11103640-11103790:plus -23.1224 0.357798 -0.688073 -0.889908 -8.70408 0.826531
-22.4954 2.81944 3.45872 0.00531 0.00911 0.0145 0.0355 0.00155 0.000153 0.0322 0.00639 0.00591
2R:11105120-11105270:minus -31.8061 3.90816 15.5229 2.11927 -18.4184 -9.72449 -4.41284
1.01389 0.963303 0.0158 0.00102 7.23e-07 0.0135 0.0101 0.00352 0.000839 0.0121 0.0145
2R:11105120-11105270:plus -24.3367 13.4592 4.72477 2.82569 -27.8265 0.387755 -20.6514
5.15278 -0.963303 0.00789 0.000064 0.00199 0.00976 0.0388 0.000217 0.0231 0.00245 0.0258
2R:11105780-11105930:minus -41.2143 10.3486 3.88073 2.00917 -28.3061 -8.38776 -19.2844
7.43056 5.66055 0.0494 0.000112 0.00281 0.0142 0.0553 0.00101 0.0183 0.000801 0.00227
2R:11105780-11105930:plus -32.4694 8.42202 -0.394495 2.78899 -18.6429 -17.9388 -22.422
-0.902778 -3.52294 0.0196 0.000304 0.0132 0.00992 0.0121 0.00738 0.0318 0.0219 0.0499
2R:11147680-11147830:minus -32.8163 1.00917 1.29358 2.33028 -27.5306 -26.4898 -22.6789
7.43056 3.66972 0.024 0.00722 0.00746 0.0123 0.0351 0.0225 0.0332 0.000801 0.00514
2R:11147680-11147830:plus -22.4388 -1.65138 7.87156 8.05505 -18.9388 0.826531 -20.3394
-1.5 9.66055 0.00348 0.0175 0.000469 0.000578 0.0151 0.000153 0.0218 0.026 0.000268
2R:11177800-11177950:minus -23.102 -0.862385 2.54128 2.33028 -17.5204 -18.1224 -9.33945
-0.0416667 1.48624 0.00522 0.0137 0.00473 0.0123 0.00532 0.00988 0.00272 0.017 0.0118
2R:11177800-11177950:plus -23.1122 1.33028 5.98165 6.45872 -17.4184 -17.7857 -25.7431

4.20833 -6.48624 0.0053 0.00642 0.00116 0.00182 0.00457 0.0068 0.0518 0.00369 0.1
2R:11178220-11178370:minus -24.2245 1.3945 13.0092 7.6055 -18.1531 -17.7143 -2.33945
-0.208333 11.8073 0.00763 0.00627 0.0000207 0.000794 0.00829 0.00634 0.000481 0.0178
0.0000538
2R:11178220-11178370:plus -30.7755 -1.79817 2.33028 6.33028 -17.4898 -18.6429 -15.7248
2.25 3.66972 0.0102 0.0183 0.00511 0.00198 0.0053 0.0139 0.0103 0.00788 0.00514
2R:11178780-11178930:minus -13.4082 1.70642 0.119266 2.92661 -26.9694 -35.9286 9.44037
-8.63889 5.53211 0.000867 0.00559 0.0111 0.00931 0.023 0.0825 0.0000157 0.124 0.00245
2R:11178780-11178930:plus -20.7755 0.201835 0.706422 5.22018 -17.3367 -36.0816
-13.3761 -6.86111 3.88073 0.00176 0.00962 0.00914 0.00418 0.00391 0.0857 0.0063 0.0901 0.00475
2R:11196520-11196670:minus 5.18367 -1 1.44037 3.09174 -26.9286 -9.23469 -8.57798 -2.93056
3.88073 0.0000136 0.0143 0.00707 0.00867 0.0214 0.00238 0.00236 0.038 0.00475
2R:11196520-11196670:plus -14.449 -1.84404 10.1743 1.58716 -18.449 -9.68367 -14.1927 0.0555556
2.25688 0.0014 0.0186 0.000128 0.017 0.0107 0.00342 0.00757 0.0164 0.00853
2R:11198560-11198710:minus -31.7449 8.90826 4.02752 2.92661 -26.6633 -8.45918 -9.16514
0.125 3.20183 0.0152 0.000235 0.00265 0.00931 0.0189 0.00111 0.00264 0.0161 0.0065
2R:11198560-11198710:plus -21.9184 3.68367 0.40367 6 -17.4898 -9.5 -18.9725 9.94444
1.72477 0.00261 0.00144 0.0101 0.00256 0.0053 0.00294 0.0174 0.000171 0.0106
2R:11209960-11210110:minus -34.1122 -2.21101 0.688073 0.211009 -27.3061 0.826531
-24.7982 2.31944 -8.31193 0.0312 0.0206 0.0092 0.0254 0.0315 0.000153 0.0459 0.00769 0.144
2R:11209960-11210110:plus -24.1122 -1.6789 -1.70642 -1.40367 1.10204 -0.244898 -8.94495
9.63889 -2.53211 0.00738 0.0177 0.0198 0.042 0.000124 0.000496 0.00253 0.000211 0.0392
2R:11210440-11210590:minus -30.2551 7.18349 2.06422 7.17431 -8.64286 -27.6735 -10.211
-0.222222 4.3945 0.00885 0.00059 0.00564 0.00115 0.00139 0.0443 0.0032 0.0179 0.00375
2R:11210440-11210590:plus -30.9184 4.59633 0.302752 0.899083 -27.5306 -8.57143
-10.7156 4.09722 0.385321 0.0103 0.00191 0.0105 0.0212 0.0351 0.00149 0.00353 0.00386 0.0166
2R:11218200-11218350:minus -23.4184 -0.0458716 -1.86239 0.981651 -26.898 -8.65306
-26.9725 2.88889 -6.78899 0.00624 0.0105 0.0208 0.0207 0.0203 0.00156 0.0603 0.00622 0.108
2R:11218200-11218350:plus -32.1735 -2.33028 1.59633 0.825688 -17.7143 -28.7041
-12.2477 -3.91667 3.44954 0.018 0.0214 0.00669 0.0216 0.00627 0.067 0.00487 0.0483 0.00594
2R:11237600-11237750:minus -34.5204 3.68367 -1.74312 1.26606 -9.54082 -8.72449 -10.5688
3.72222 1.79817 0.0319 0.00144 0.02 0.0191 0.00294 0.00169 0.00343 0.0045 0.0102
2R:11237600-11237750:plus -21.8061 -1.23853 0.568807 7.80734 -8.86735 -27.2653 6.61468
-1.25 2.93578 0.00241 0.0154 0.00958 0.00063 0.00173 0.0333 0.0000346 0.0242 0.00717
2R:11240940-11241090:minus -31.5102 2.00917 2.97248 7.15596 -18.4184 -27.3367 -5.87156
-1.22222 -0.0275229 0.013 0.005 0.00401 0.00117 0.0101 0.0347 0.00127 0.024 0.0186
2R:11240940-11241090:plus -31.8061 4.46789 7.41284 9.23853 -26.9286 -17.2041 -19.4862
8.26389 -5.12844 0.0158 0.00201 0.000593 0.000424 0.0214 0.00507 0.0189 0.000502 0.0748
2R:11259040-11259190:minus -39.1429 1.42202 2.24771 1.16514 10.6531 -9.79592 -24.2661 3.59722
9.45872 0.0327 0.00621 0.00527 0.0197 0.0000102 0.00377 0.0427 0.00473 0.000337
2R:11259040-11259190:plus -34.1837 -0.0733945 -4.44037 3.75229 -18.9796 -9.60204 -27.2294 5.75 7.6789
0.0314 0.0106 0.0427 0.00649 0.0155 0.00334 0.0623 0.00187 0.000866
2R:11268120-11268270:minus -21.9286 -2.18349 4.43119 6.72477 -18.449 -19.2755 -0.522936 4.18056
6.33028 0.0027 0.0205 0.00225 0.00154 0.0107 0.0185 0.000319 0.00373 0.0016
2R:11268120-11268270:plus -23.1122 9.15596 4.06422 5.94495 -8.93878 -7.72449 -18.2202 -0.138889
1.49541 0.0053 0.000207 0.00261 0.00261 0.00216 0.00068 0.0155 0.0175 0.0118
2R:11268440-11268590:minus -24.1531 2.14679 -1.88991 5.77982 -7.33673 -8.5 -6.99083 3.98611 -1.12844
0.00744 0.00476 0.0209 0.00294 0.000388 0.00126 0.00168 0.00404 0.0271
2R:11268440-11268590:plus -31.3673 -3.33945 4.3945 1.53211 -8.53061 -8.79592 -25.0917 4.625 -2.66972
0.0116 0.0283 0.00229 0.0174 0.0011 0.0019 0.0477 0.00309 0.0407
2R:11268800-11268950:minus -41.4796 -1.04587 2.65138 2.16514 -8.96939 -0.469388 -21.9633 5.65278
-0.697248 0.0577 0.0145 0.00453 0.0132 0.00226 0.000533 0.0293 0.00195 0.0239
2R:11268800-11268950:plus -24.3367 5.02752 -0.238532 0.357798 20.2041 0.27551 -11.3853 -3.25
1.44037 0.00789 0.00161 0.0125 0.0245 3.64e-07 0.000257 0.00404 0.0411 0.012

2R:1138500-1138650:minus -23.2959 3.90816 2.95413 6.00917 0.806122 -18.0102 -7.11009 3.91667 3.40367
0.00605 0.00102 0.00404 0.00255 0.000229 0.00812 0.00173 0.00416 0.00603
2R:1138500-1138650:plus -33.0408 5.58716 -1.68807 6.00917 -18.9796 -9.45918 -10.6606 5.31944 2.84404
0.0264 0.00127 0.0197 0.00255 0.0155 0.00279 0.00349 0.00227 0.00736
2R:11398860-11399010:minus -41.5918 1.19266 0.46789 7.34862 -18.7143 -8.45918 -26.7523 4.11111
-2.59633 0.0623 0.00675 0.00992 0.000998 0.0136 0.00111 0.0587 0.00384 0.0398
2R:11398860-11399010:plus -30.7755 1.44037 15.0826 0.972477 -27.4184 -25.898 -13.5505 0.5 3.21101
0.0102 0.00617 1.18e-06 0.0207 0.0323 0.0214 0.00656 0.0143 0.00644
2R:11399100-11399250:minus -31.5102 4.33945 14.8624 2.94495 -8.67347 -9.68367 -14.8532 0.291667
-2.18349 0.013 0.00211 2.2e-06 0.0092 0.00154 0.00342 0.00871 0.0153 0.0351
2R:11399100-11399250:plus -29.9184 -2.55963 -2.12844 7.63303 -17.0408 -0.244898 -14.422 3.61111
2.88073 0.00854 0.0228 0.0224 0.000745 0.00329 0.000496 0.00796 0.0047 0.00727
2R:11419120-11419270:minus -32.7041 -1.83486 5.55046 6.01835 -18.7449 -0.397959 -20.844 5.38889
-0.440367 0.0222 0.0185 0.00141 0.00248 0.0138 0.000525 0.0239 0.0022 0.0216
2R:11419120-11419270:plus -30.8571 -2.54128 -0.0733945 2.46789 -16.449 -17.7143 -11.5321 -0.263889
-1.6055 0.0103 0.0227 0.0119 0.0115 0.00307 0.00634 0.00417 0.0181 0.0302
2R:11419720-11419870:minus -32.0714 12.7615 -0.522936 4.05505 11.6939 -26.5612 -7.36697 3.15278
5.70642 0.0176 0.0000265 0.0137 0.00566 1.17e-06 0.0237 0.00184 0.00563 0.00222
2R:11419720-11419870:plus -23.4082 1.46789 3.46789 1.56881 -27.2347 -18.0102 -13.2569 3.63889 -0.862385
0.00623 0.0061 0.00331 0.0172 0.0283 0.00812 0.00613 0.00465 0.0251
2R:11425540-11425690:minus -20.9286 0.972477 4.94495 4.27523 2.87755 -9.09184 -16.5046 5.66667
-3.00917 0.00183 0.00731 0.00182 0.00518 0.0000259 0.00219 0.0118 0.00194 0.0447
2R:11425540-11425690:plus -22.2959 -1.37615 1.55963 -3.04587 1.43878 1.05102 -13.4404 3.55556 3.88073
0.00335 0.0161 0.00678 0.0694 0.0000769 0.000107 0.00639 0.00481 0.00475
2R:11428840-11428990:minus -31.7755 13.3878 13.4771 6.73394 -19.051 -18.2755 -8.23853 -1.43056
-2.77982 0.0157 0.0000869 0.0000133 0.00152 0.0162 0.0112 0.00221 0.0255 0.042
2R:11428840-11428990:plus -13.1122 13.5306 0.495413 7 -17.4184 0.346939 -24.2936 -4.51389 -5.47706
0.000632 0.0000206 0.00983 0.00132 0.00457 0.000221 0.0428 0.0554 0.0801
2R:11429960-11430110:minus -31.6327 0.46789 -2.6789 1.24771 -18.5612 -17.1224 -16.6239 1.73611
2.33028 0.0138 0.00876 0.0263 0.0192 0.0119 0.00499 0.0121 0.00946 0.00836
2R:11429960-11430110:plus -31.2143 1.44037 15.7339 7.29358 -18.4184 -8.42857 -12.789 -0.125 6.02752
0.0111 0.00617 2.45e-07 0.00104 0.0101 0.00104 0.00551 0.0174 0.00183
2R:11453280-11453430:minus -13.4184 11.8716 9.2844 3.69725 -18.0816 -18.0102 -10.5963 4.68056
-0.908257 0.000873 0.0000496 0.000215 0.00665 0.00759 0.00812 0.00345 0.00302 0.0255
2R:11453280-11453430:plus -22.2245 13.6239 0.366972 3.77064 -27.2347 -10.0918 -10.3303 3.51389 -7.66055
0.00325 0.000013 0.0103 0.00637 0.0283 0.00404 0.00328 0.00489 0.127
2R:11453520-11453670:minus -13.4184 11.8716 9.2844 3.69725 -18.0816 -18.0102 -18.6514 6.04167
-0.908257 0.000873 0.0000496 0.000215 0.00665 0.00759 0.00812 0.0165 0.00162 0.0255
2R:11453520-11453670:plus -23.5204 1.88991 0.550459 2.70642 -27.0102 9.60204 -19.3853 4.38889 1.16514
0.00645 0.00523 0.00965 0.0103 0.0237 0.0000248 0.0186 0.00342 0.0135
2R:11456400-11456550:minus -21.7041 1.92661 1.16514 -0.752294 -27.2347 -7.65306 -21.2752 3.55556
-2.63303 0.00231 0.00516 0.0078 0.034 0.0283 0.000647 0.0259 0.00481 0.0404
2R:11456400-11456550:plus -24.5612 13.4592 -1.05505 2.50459 -26.3061 -9.53061 -20.6239 8.77778 1.38532
0.00819 0.000064 0.0162 0.0114 0.0181 0.0031 0.023 0.000368 0.0123
2R:11456740-11456890:minus -22.0306 1.62385 -1.88073 1.41284 -26.9694 -9.02041 -9.18349 2.70833
0.752294 0.00293 0.00577 0.0209 0.0183 0.023 0.00213 0.00265 0.00666 0.0152
2R:11456740-11456890:plus -21.9592 1.07339 -1.25688 6.33945 -18.3776 -17.0816 -18.7798 -1.33333 2.11009
0.00279 0.00705 0.0173 0.00196 0.00926 0.00487 0.0169 0.0248 0.00898
2R:11458960-11459110:minus -11.8469 -0.0825688 8.87156 7.2844 -18.4184 -27.3061 -15.1101 0.236111
3.45872 0.000279 0.0106 0.000273 0.00105 0.0101 0.0336 0.00917 0.0155 0.00591
2R:11458960-11459110:plus -38.9898 2.44954 10.2294 5.31193 -17.9796 -19.1224 -13.9908 4.125 -5.50459
0.0325 0.00425 0.000125 0.00393 0.00726 0.0175 0.00724 0.00382 0.0806
2R:11461320-11461470:minus -31.5102 -1.52294 11.6697 4.11927 -26.9286 -17.2755 -15.2752 10.7778
3.93578 0.013 0.0169 0.0000536 0.00551 0.0214 0.00544 0.00948 0.0000914 0.00456

2R:11461320-11461470:plus -22.7041 -4.30275 -2.33028 0.954128 -19.051 -19.3469 -18.5138 7.30556 2.47706
0.00395 0.0367 0.0238 0.0208 0.0162 0.0194 0.0162 0.000857 0.00801
2R:1146980-1147130:minus -40.102 3.72477 3.72477 5.6789 -18.1531 9.34694 -17.7706 1.93056 1.22018
0.036 0.00266 0.003 0.00315 0.00829 0.0000366 0.0144 0.00883 0.0133
2R:1146980-1147130:plus -31.8163 4.43119 7.98165 -1.05505 -17.7551 -9.79592 -18.9725 6.97222 -0.486239
0.016 0.00204 0.000442 0.0374 0.00658 0.00377 0.0174 0.00102 0.0219
2R:1147480-1147630:minus -30.8163 1.13761 -1.00917 3.44954 -18.7245 -9.34694 -10.0459 9.04167
-0.0275229 0.0103 0.00689 0.016 0.00738 0.0136 0.00261 0.0031 0.000312 0.0186
2R:1147480-1147630:plus -42.6327 0.889908 -2.66972 2.30275 -26.5306 -9.09184 -14.1009 -1.01389 0.247706
0.0829 0.00753 0.0263 0.0124 0.0185 0.00219 0.00742 0.0227 0.0172
2R:11566400-11566550:minus -20.9286 -1.46789 14.3486 7.29358 -18.6837 -17.2755 -12.8899 -3.94444
6.73394 0.00183 0.0166 4.67e-06 0.00104 0.0126 0.00544 0.00564 0.0486 0.00143
2R:11566400-11566550:plus -41.0612 -1.00917 15.6239 -0.633028 -18.6429 -0.0204082 -19.789
-0.0972222 -0.284404 0.0466 0.0144 5.44e-07 0.0327 0.0121 0.000411 0.0199 0.0172 0.0207
2R:11567560-11567710:minus -23.3367 1.99083 11.8624 3.43119 -18.4898 -17.0816 -3.95413 3.25 3.72477
0.00614 0.00504 0.0000471 0.00749 0.0116 0.00487 0.000736 0.00542 0.00497
2R:11567560-11567710:plus -21.9286 -0.477064 4.57798 0.449541 -18.4592 1.27551 -10.4771 1.63889
3.88073 0.0027 0.0121 0.00212 0.024 0.0109 0.0000717 0.00337 0.00978 0.00475
2R:11622920-11623070:minus -30.6224 4.11009 4.72477 7.47706 -18.3061 -16.9796 -25.4954 -3.625
-8.76147 0.00964 0.00231 0.00199 0.00085 0.00884 0.0044 0.0502 0.045 0.158
2R:11622920-11623070:plus -12.6939 1.56881 3.34862 9.76147 -17.3776 -16.7857 -7.11009 -2.25 4.02752
0.000462 0.00588 0.00347 0.000212 0.00419 0.00426 0.00173 0.0319 0.00428
2R:11632140-11632290:minus -24.0816 -0.688073 -4.00917 2.83486 -18.6429 -8.57143 -11.7064 -0.875
3.88991 0.00732 0.013 0.0381 0.0097 0.0121 0.00149 0.00433 0.0218 0.00467
2R:11632140-11632290:plus -22.8571 -0.825688 3.15596 7 -16.7143 -25.4898 -17.2661 1.09722 5.70642
0.00427 0.0136 0.00374 0.00132 0.00319 0.0205 0.0134 0.0118 0.00222
2R:11633060-11633210:minus -22.7347 -1.46789 4.88073 7 -17.2245 -27.8265 -11.8807 9.91667 4.7156
0.00403 0.0166 0.00187 0.00132 0.00384 0.0498 0.00449 0.000174 0.00345
2R:11633060-11633210:plus -30.8163 -0.678899 3.63303 0.256881 -9.27551 -18.8571 -20.633 1.44444
5.70642 0.0103 0.0129 0.00311 0.0251 0.00284 0.0151 0.023 0.0105 0.00222
2R:11703480-11703630:minus -22.449 3.97959 -0.642202 6.12844 -18.7857 -8.65306 -19.9083 -0.361111
-3.41284 0.0035 0.000669 0.0143 0.00231 0.0148 0.00156 0.0203 0.0187 0.0487
2R:11703480-11703630:plus -21.7755 -1.18349 -1.40367 -2.72477 -27.2653 -9.42857 -18.1743 10.5694 1.07339
0.00241 0.0152 0.0181 0.0643 0.0302 0.0027 0.0154 0.000107 0.014
2R:11704400-11704550:minus -40.3265 -0.174312 1.83486 3.44037 -18.6735 -28.8571 -16.578 8.16667
3.31193 0.0394 0.0109 0.00614 0.00746 0.0123 0.0699 0.012 0.00053 0.00618
2R:11704400-11704550:plus -14.8571 0.275229 3.04587 4.33945 -27.2245 -7.72449 -13.8807 2.30556 -0.33945
0.0016 0.00938 0.0039 0.00509 0.0272 0.00068 0.00706 0.00772 0.0209
2R:11735980-11736130:minus -41.8469 -0.40367 -0.422018 0.366972 -18.7551 -9.94898 -19.6055 9.18056
3.09174 0.0665 0.0118 0.0133 0.0244 0.0144 0.00393 0.0193 0.000285 0.00678
2R:11735980-11736130:plus -31.6224 -5.29358 -0.761468 0.899083 11.6531 -18.0102 -7.83486 -1.29167
7.15596 0.0137 0.0476 0.0148 0.0212 1.55e-06 0.00812 0.00203 0.0245 0.00124
2R:11748740-11748890:minus -23.6327 -0.0917431 3.86239 2.51376 -18.449 -26.8265 -20.3028
-0.0833333 9.92661 0.00661 0.0106 0.00283 0.0113 0.0107 0.0285 0.0217 0.0172 0.000193
2R:11748740-11748890:plus -30.9184 1.11927 10.7339 -0.46789 0.877551 -18.7857 -12.4679 2.19444 1.90826
0.0103 0.00693 0.0000931 0.0309 0.00019 0.0146 0.00512 0.00804 0.00974
2R:11750440-11750590:minus -13.2245 13.4592 0.266055 3.18349 -17.0816 -27.0408 -13.2385 -1.88889
7.6422 0.000738 0.000064 0.0106 0.00837 0.00334 0.0307 0.00611 0.0289 0.000893
2R:11750440-11750590:plus -13.1122 5.33945 6.57798 4.36697 -8.37755 -27.7041 -1.62385 1.36111 9.14679
0.000632 0.00142 0.000884 0.00505 0.00106 0.0445 0.000408 0.0108 0.000414
2R:11750960-11751110:minus -12.6939 3.0367 1.73394 3.18349 -19.051 -9.27551 -6.12844 0.569444
3.05505 0.000462 0.00343 0.00638 0.00837 0.0162 0.00244 0.00136 0.014 0.00692
2R:11750960-11751110:plus -23.2959 -3.62385 -0.302752 2.3578 -18.1939 -18.5612 -11.8991 0.111111
5.07339 0.00605 0.0306 0.0128 0.0121 0.0086 0.0132 0.00451 0.0162 0.00305

2R:11756640-11756790:minus -20.9592 7 2.90826 2.47706 -18.7143 -18.7857 -15.1651 9.16667 -3.08257
0.00184 0.000649 0.00411 0.0115 0.0136 0.0146 0.00927 0.000288 0.0455
2R:11756640-11756790:plus -40.2143 7.21101 3.55963 1.72477 -17.7143 -17.7857 -21.3119 4.55556 1.48624
0.0372 0.000581 0.0032 0.0161 0.00627 0.0068 0.026 0.00318 0.0118
2R:11783500-11783650:minus -32.2143 3 3.33945 5.49541 -18.7143 -9.94898 -18.5413 2.15278 6.25688
0.0182 0.00348 0.00348 0.00355 0.0136 0.00393 0.0162 0.00816 0.00163
2R:11783500-11783650:plus -33.551 2.25688 3.78899 2.19266 -27.5306 -17.2755 -13.2936 7.36111 -0.724771
0.0288 0.00457 0.00292 0.0131 0.0351 0.00544 0.00618 0.000832 0.0241
2R:11805840-11805990:minus -30.5204 -2.48624 -3.19266 3.41284 -17.7551 -8.5 -16.3945 2.375 4.56881
0.00951 0.0223 0.0304 0.00756 0.00658 0.00126 0.0116 0.00753 0.00356
2R:11805840-11805990:plus -31.5918 -2.41284 0.385321 6.33945 -26.8163 -8.65306 -21.1743 1.38889 -10.4587
0.0136 0.0219 0.0102 0.00196 0.0195 0.00156 0.0254 0.0107 0.211
2R:11809620-11809770:minus -3.18367 -1.12844 2.52294 6.85321 10.3878 -27.602 -7.12844 4.875
9.87156 0.0000481 0.0149 0.00476 0.00137 0.0000174 0.0416 0.00174 0.00277 0.000217
2R:11809620-11809770:plus -31.4388 -1.89908 3.70642 2.56881 -18.0816 -7.94898 -17.8165 4.75 2.47706
0.0122 0.0189 0.00302 0.0111 0.00759 0.00074 0.0145 0.00293 0.00801
2R:1181400-1181550:minus -23.2245 0.623853 3 2.6422 -18.051 -19.3061 -18 -1.02778 -3.14679 0.00576
0.00829 0.00397 0.0107 0.00746 0.0187 0.015 0.0227 0.0463
2R:1181400-1181550:plus -24.7857 3.7551 2.18349 -2.02752 -7.63265 -8.68367 -18.7248 1.66667 1.2844
0.00829 0.00122 0.0054 0.0517 0.000479 0.00157 0.0167 0.00969 0.0129
2R:11815880-11816030:minus -31.8571 7.57798 -1.79817 5.84404 -28.0102 -9.45918 -22.2294 -1.05556
-4.41284 0.0163 0.000479 0.0204 0.00289 0.0449 0.00279 0.0307 0.0229 0.0624
2R:11815880-11816030:plus -22.2551 6.31193 2.15596 6.69725 -26.1939 -27.449 -8.86239 1.68056 -0.229358
0.00327 0.000913 0.00545 0.00156 0.0175 0.0357 0.00249 0.00964 0.0202
2R:11816920-11817070:minus -31.8469 0.87156 8.38532 2.49541 -27.4898 -28.3776 -14.8624 4.26389
1.34862 0.0162 0.00758 0.000356 0.0114 0.0336 0.0609 0.00873 0.0036 0.0125
2R:11816920-11817070:plus -22 3.3578 6.3945 3.86239 -17.4898 -9.5 -20.1009 4.22222 0.93578 0.00291
0.00305 0.000966 0.00622 0.0053 0.00294 0.021 0.00367 0.0145
2R:11818520-11818670:minus -22.449 0.477064 4.13761 7.17431 -8.93878 -17.8265 -2.81651 2.36111
2.2844 0.0035 0.00873 0.00254 0.00115 0.00216 0.00699 0.000542 0.00757 0.00842
2R:11818520-11818670:plus -22.5918 -1.91743 3.22018 5.86239 -7.86735 -17.9796 -17.2294 3.75 -3.10092
0.00365 0.019 0.00365 0.00282 0.000617 0.00764 0.0133 0.00445 0.0458
2R:11819460-11819610:minus -12.7041 6.33028 2.74312 6.41284 -27.6327 -26.602 -13.5138 -1.58333
4.49541 0.000464 0.000904 0.00438 0.00189 0.0369 0.0248 0.0065 0.0266 0.00363
2R:11819460-11819610:plus -32.0408 1.01835 1.13761 3.88991 -27.7551 -8.79592 -25.6147 2.81944 1.9633
0.0174 0.00719 0.00788 0.00608 0.0385 0.0019 0.051 0.00639 0.00951
2R:11822200-11822350:minus -29.8776 3.65138 0.357798 5.57798 -18.0816 0.826531 -24.4128 1.11111
-5.54128 0.00852 0.00274 0.0103 0.0034 0.00759 0.000153 0.0436 0.0117 0.081
2R:11822200-11822350:plus -40.8469 6.04587 14.3028 1.99083 -18.449 -18.2347 -16.0917 6.43056 -2.05505
0.0437 0.00104 4.88e-06 0.0144 0.0107 0.0104 0.011 0.00134 0.0338
2R:11827220-11827370:minus -31.4796 0.633028 2.88073 2.3945 -26.6327 -17.3061 -6.88073 7.56944
-0.651376 0.0128 0.00826 0.00415 0.0119 0.0188 0.00548 0.00164 0.000743 0.0235
2R:11827220-11827370:plus -22.0816 -3.49541 1.33945 2.9633 -18.6837 -9.53061 -26.1468 -4.5 -6.91743 0.003
0.0295 0.00733 0.00914 0.0126 0.0031 0.0545 0.0552 0.111
2R:11842500-11842650:minus -41.8163 4.9633 -1.44037 5.29358 -17.5204 -9.82653 -11.8899 5.13889
-0.440367 0.0662 0.00165 0.0183 0.00398 0.00532 0.00381 0.0045 0.00247 0.0216
2R:11842500-11842650:plus -24 13.5963 11.2385 5.15596 -18.7449 -16.5 -10.4771 2.125 3.62385
0.00719 0.0000131 0.0000688 0.00426 0.0138 0.00416 0.00337 0.00824 0.00523
2R:11878740-11878890:minus -22.5102 -0.0825688 13.1284 3.27523 -17.4898 -9.65306 -12.6789 1.76389
0.293578 0.00354 0.0106 0.0000181 0.00796 0.0053 0.0034 0.00537 0.00937 0.017
2R:11878740-11878890:plus -23.7857 4.93578 14.7248 7.2844 -18.4898 -18.051 -20.5413 2.68056 4.23853
0.00679 0.00167 2.82e-06 0.00105 0.0116 0.00886 0.0226 0.00673 0.00394
2R:11879960-11880110:minus -21.7041 2.81651 5.95413 6.62385 0.806122 -0.0204082 2.72477 -1.33333
-2.2844 0.00231 0.00372 0.00118 0.00163 0.000229 0.000411 0.00013 0.0248 0.0361

2R:11879960-11880110:plus -13.3061 1.38532 2.51376 10.8624 -17.6837 -18.2755 -19.3303 1.52778 -3.14679 0.000777 0.00629 0.00478 0.0000687 0.0058 0.0112 0.0184 0.0102 0.0463
2R:11885880-11886030:minus -32.4796 -2.36697 4.6789 4.6055 -18.7143 -26.4898 0.110092 0.597222 1.27523 0.02 0.0216 0.00203 0.00476 0.0136 0.0225 0.000276 0.0139 0.013
2R:11885880-11886030:plus -31.7755 -2.04587 2.0367 3.70642 -8.89796 -18.8571 -21.1101 2.15278 1.56881 0.0157 0.0197 0.0057 0.0066 0.00189 0.0151 0.0251 0.00816 0.0114
2R:11887080-11887230:minus -23 1.55046 2.25688 7.77982 -8.33673 -28.3367 -4.2844 0.486111 7.73394 0.00498 0.00592 0.00525 0.000652 0.00101 0.0606 0.000809 0.0144 0.000784
2R:11887080-11887230:plus -30.9184 -1.3945 5.00917 5.30275 -18.3776 -8.5 -4.63303 1.13889 0.59633 0.0103 0.0162 0.00177 0.00396 0.00926 0.00126 0.000894 0.0116 0.016
2R:11887840-11887990:minus -30.5918 5.94495 0.788991 9.59633 -26.5918 0.0204082 -17.6972 -6.25 -4.72477 0.00963 0.00108 0.00889 0.000275 0.0186 0.000377 0.0143 0.08 0.068
2R:11887840-11887990:plus -14.2143 0.577982 9.04587 3.05505 -18.1531 -9.23469 -13.5229 -0.375 -0.724771 0.00124 0.00843 0.000248 0.00878 0.00829 0.00238 0.00652 0.0188 0.0241
2R:11888680-11888830:minus -21.8878 9.2844 -1.20183 6.21101 -27.0816 -9.53061 -11.0917 -5.69444 5.90826 0.00258 0.000193 0.017 0.00213 0.0243 0.0031 0.0038 0.0715 0.00198
2R:11888680-11888830:plus -30.4796 1.25688 0.100917 -0.311927 -17.449 -17.051 -15.6972 4.52778 -3.59633 0.00941 0.00659 0.0112 0.0294 0.00484 0.00479 0.0103 0.00322 0.0507
2R:11889060-11889210:minus -31.1837 -1.54128 5.55046 2.75229 -18.8265 -0.244898 -12.2661 6.95833 1.86239 0.0109 0.0169 0.00141 0.0101 0.0149 0.000496 0.00489 0.00103 0.00994
2R:11889060-11889210:plus -31.7755 7.46789 -2.16514 2.61468 10.9184 1.27551 -11.4312 10.1667 5.66055 0.0157 0.000506 0.0227 0.0109 4.19e-06 0.0000717 0.00408 0.000145 0.00227
2R:11895140-11895290:minus -21.6224 0.12844 13.6606 7.45872 -17.3061 -27.5612 -13.3394 -0.666667 3.66972 0.00213 0.00987 0.0000112 0.000894 0.00389 0.0393 0.00625 0.0205 0.00514
2R:11895140-11895290:plus -13.6735 15.3303 11.3761 9.49541 -27.602 -18.0102 -20.6697 0.277778 -2.26606 0.00103 1.39e-06 0.0000633 0.000308 0.0364 0.00812 0.0232 0.0153 0.0359
2R:11902260-11902410:minus -13.3673 7.11927 5.26606 7.00917 1.61224 -18.2041 -12.9725 -0.208333 -2.3211 0.000813 0.000611 0.00159 0.00126 0.0000631 0.0103 0.00574 0.0178 0.0365
2R:11902260-11902410:plus -23.1531 13.5413 3.81651 3.19266 -18.4592 0.755102 -8.22936 -0.527778 0.963303 0.00542 0.0000137 0.00289 0.00829 0.0109 0.000175 0.0022 0.0196 0.0145
2R:11934940-11935090:minus -31.6939 2.53211 6.40367 6.02752 -27.2653 -19.3469 -12.6881 -4.51389 0.926606 0.0142 0.00413 0.000962 0.00246 0.0302 0.0194 0.00538 0.0554 0.0146
2R:11934940-11935090:plus -22.6224 13.4592 4.12844 0.357798 -17.4184 -9.42857 -19.0275 0.277778 -1.58716 0.00371 0.000064 0.00254 0.0245 0.00457 0.0027 0.0175 0.0153 0.03
2R:1194660-1194810:minus -31.7347 0.926606 3.45872 6.13761 -9.0102 -0.204082 -7.30275 -0.222222 10.3486 0.0149 0.00743 0.00332 0.00226 0.00236 0.000463 0.00181 0.0179 0.0000988
2R:1194660-1194810:plus -32 -0.229358 13.8165 4.02752 0.612245 -9.7551 -10.1927 3.20833 -0.247706 0.0171 0.0111 9.45e-06 0.00581 0.000269 0.00358 0.00319 0.00551 0.0204
2R:1195000-1195150:minus -33.0816 8.0367 -2.31193 6.83486 -18.7143 -18.1531 -27.8349 2.44444 7.68807 0.0268 0.000375 0.0237 0.00142 0.0136 0.00997 0.0673 0.00734 0.000841
2R:1195000-1195150:plus -23.2551 1.50459 7.01835 6.6422 -18.1531 -18.0816 -19.5688 6.625 6.49541 0.00583 0.00602 0.000721 0.00161 0.00829 0.00934 0.0191 0.00122 0.00154
2R:11986060-11986210:minus -14.5918 3.87156 4.9633 10.7615 20.4694 19.3469 -18.0367 3.45833 -2.9633 0.00148 0.00252 0.00181 0.0000826 1.65e-07 5.04e-07 0.015 0.005 0.0442
2R:11986060-11986210:plus -23.9184 5.97248 11.1651 6.12844 -17.7857 0.0204082 -21.945 4.83333 3.16514 0.00695 0.00107 0.000072 0.00231 0.00676 0.000377 0.0292 0.00283 0.00656
2R:11990140-11990290:minus -30.4082 -4.6422 4.33945 4.66972 0.836735 -9.72449 -12.4679 1.51389 -2.3211 0.00908 0.0401 0.00234 0.00466 0.000215 0.00352 0.00512 0.0102 0.0365
2R:11990140-11990290:plus -4.44898 1.86239 -0.376147 6.12844 -18.1531 -18.0102 -8.37615 0.388889 1.00917 0.000152 0.00528 0.0131 0.00231 0.00829 0.00812 0.00227 0.0148 0.0143
2R:11990760-11990910:minus -5.0102 -0.174312 -0.733945 0.990826 -27.2347 -9.94898 -20.5046 9.29167 2.3211 0.000221 0.0109 0.0147 0.0206 0.0283 0.00393 0.0225 0.000266 0.00839
2R:11990760-11990910:plus -23.4082 2.31193 6.54128 9.59633 -26.9694 -0.173469 -5.07339 8.70833 1.90826 0.00623 0.00448 0.000901 0.000275 0.023 0.000456 0.00101 0.000384 0.00974

2R:11991780-11991930:minus -14.3673 -4.77064 1.3945 0.779817 -27.2653 -27.8571 -2.36697 5.625
6.6422 0.00132 0.0415 0.00719 0.0219 0.0302 0.0508 0.000485 0.00198 0.00148
2R:11991780-11991930:plus -23.1939 0.990826 -2.7156 2.98165 -18.6735 -8.79592 -25.9358 2.41667 -6.16514
0.00558 0.00726 0.0266 0.00905 0.0123 0.0019 0.0531 0.00742 0.0926
2R:11992260-11992410:minus -14.0714 2.42202 -2.47706 -0.59633 -18.7143 -17.2755 -5.05505 1.59722
7.84404 0.00117 0.0043 0.0248 0.0322 0.0136 0.00544 0.00101 0.00993 0.000701
2R:11992260-11992410:plus -21.6224 6.45872 2.08257 5.82569 -18.3776 -36.3367 -0.651376 6.97222
1.07339 0.00213 0.000851 0.0056 0.00291 0.00926 0.0974 0.000329 0.00102 0.014
2R:11993900-11994050:minus -41.1122 13.5229 -0.321101 1.05505 -17.2245 -8.02041 -11.1284 2.63889
8.19266 0.0473 0.000014 0.0129 0.0204 0.00384 0.000761 0.00383 0.00684 0.000548
2R:11993900-11994050:plus -14.5204 -0.954128 3.98165 3.19266 -9.16327 -27.602 -12.5688 -3.90278
2.48624 0.00145 0.0141 0.0027 0.00829 0.00249 0.0416 0.00524 0.0481 0.00795
2R:12012700-12012850:minus -3.40816 2.77982 1.47706 2.82569 -17.3776 -28.9286 -4.44037 4.75 5.44037
0.0000685 0.00377 0.00698 0.00976 0.00419 0.0717 0.000846 0.00293 0.00259
2R:12012700-12012850:plus -21.7347 0.220183 2.72477 2.76147 -8.64286 -26.6735 5.10092 -9.31944 8.10092
0.00235 0.00956 0.00441 0.01 0.00139 0.0256 0.0000556 0.138 0.000623
2R:12013520-12013670:minus -31.2143 1.3211 -1.16514 2.95413 -17.3776 -0.0204082 -10.6881 -0.222222
5.08257 0.0111 0.00644 0.0168 0.00917 0.00419 0.000411 0.00351 0.0179 0.00303
2R:12013520-12013670:plus -23.3367 4.92661 -1.57798 7.00917 -18.3776 -8.30612 -16.3303 -3.44444 3.24771
0.00614 0.00168 0.019 0.00126 0.00926 0.000973 0.0115 0.0431 0.00636
2R:12014540-12014690:minus -13.2653 0.275229 2.70642 3.22018 -8.89796 -9.65306 -3.11009 -1.40278
-2.11009 0.000759 0.00938 0.00444 0.00818 0.00189 0.0034 0.000585 0.0253 0.0343
2R:12014540-12014690:plus -32.3367 5.12844 -3.17431 3.59633 -18.1531 -8.23469 -9.47706 4.34722 0.137615
0.0187 0.00154 0.0303 0.00693 0.00829 0.000875 0.00279 0.00348 0.0178
2R:12016580-12016730:minus -23.0816 4.3945 -2.68807 3.45872 -26.9286 -25.8265 -13.8073 5.40278
0.963303 0.00522 0.00207 0.0264 0.00731 0.0214 0.0212 0.00695 0.00219 0.0145
2R:12016580-12016730:plus -14.7143 -3.12844 9.40367 6.86239 -17.5306 -36.0816 -9.19266 -1.45833 12.2752
0.00154 0.0267 0.0002 0.00136 0.0054 0.0857 0.00265 0.0257 0.0000141
2R:12017580-12017730:minus -30.551 7.25688 0.577982 6.45872 -27.2347 -9.79592 -20.9633 -0.611111
-3.33945 0.00959 0.000568 0.00955 0.00182 0.0283 0.00377 0.0244 0.0201 0.0479
2R:12017580-12017730:plus -31.6327 -1.55963 3.77064 4.0367 -17.0408 -9.45918 -26.8257 -6.06944 -4.11927
0.0138 0.017 0.00294 0.00578 0.00329 0.00279 0.0592 0.0772 0.0572
2R:12019360-12019510:minus -32.4388 -5.38532 0.0733945 2.01835 -16.4898 -17.8571 -5.40367 -1.04167
7.06422 0.0195 0.0488 0.0113 0.0141 0.00312 0.00719 0.00111 0.0228 0.00131
2R:12019360-12019510:plus -21.9286 -0.495413 5.44954 2.92661 -18.9796 -27.602 -14.3578 -4.63889
4.87156 0.0027 0.0122 0.00147 0.00931 0.0155 0.0416 0.00785 0.057 0.00325
2R:12023120-12023270:minus -22.0408 -5.25688 8.43119 2.66972 0.836735 -26.6735 -12.0183 -0.652778
5.38532 0.00296 0.0472 0.000347 0.0106 0.000215 0.0256 0.00463 0.0204 0.00267
2R:12023120-12023270:plus -40.3265 3.68367 0.183486 8.05505 1.17347 -8.72449 -12.4404 0.777778 7.53211
0.0394 0.00144 0.0109 0.000578 0.000101 0.00169 0.00509 0.0131 0.000968
2R:12026040-12026190:minus -31.7755 6.17431 8.77064 2.93578 -27.1939 -9.60204 -17.055 1.875
1.53211 0.0157 0.000977 0.000287 0.00927 0.0267 0.00334 0.0129 0.00901 0.0116
2R:12026040-12026190:plus -41.2551 -1.68807 1.33945 2.82569 0.846939 -9.02041 -22.3578 -0.333333
-2.05505 0.0513 0.0177 0.00733 0.00976 0.000197 0.00213 0.0314 0.0185 0.0338
2R:12026500-12026650:minus -30.8878 1.74312 -0.100917 6.00917 -18.4898 -9.27551 -16.9908 0.638889
8.19266 0.0103 0.00552 0.012 0.00255 0.0116 0.00244 0.0128 0.0137 0.000548
2R:12026500-12026650:plus -31.9286 -0.614679 4.51376 3.14679 -17.4184 -17.2041 -21.9174 4.83333
-6.52294 0.0166 0.0127 0.00218 0.00847 0.00457 0.00507 0.0291 0.00283 0.101
2R:12029580-12029730:minus -41.7449 6.80734 -3.13761 7.10092 -27 0.316327 -27.7798 2.375 -7.51376
0.0646 0.000716 0.03 0.00121 0.0233 0.000243 0.0668 0.00753 0.124
2R:12029580-12029730:plus -32.1122 13.5306 13.9908 7.2844 -36.3061 -18.7857 -23.5688 -0.944444
-6.73394 0.0178 0.0000206 7.33e-06 0.00105 0.0749 0.0146 0.0384 0.0222 0.107
2R:12030620-12030770:minus -40.2959 -0.954128 14.6881 7.15596 -18.1224 -18.8571 -10.2661 7.43056
1.02752 0.0389 0.0141 3.06e-06 0.00117 0.00782 0.0151 0.00324 0.000801 0.0141

2R:12030620-12030770:plus -13.3367 1.79817 6.87156 3.3578 -17.7143 -17.1224 -8.08257 1.98611 3.6789
0.0008 0.00541 0.000772 0.00767 0.00627 0.00499 0.00214 0.00866 0.00506
2R:12036200-12036350:minus -31.9694 0.981651 0.110092 7.34862 -8.93878 10.5714 -29.6514 1 5.9633
0.0168 0.00729 0.0112 0.000998 0.00216 4.67e-06 0.0865 0.0121 0.00188
2R:12036200-12036350:plus -15 -2.04587 2.85321 1.10092 -18.7143 -17.051 -3.07339 -0.388889 -4.11009
0.00162 0.0197 0.0042 0.02 0.0136 0.00479 0.000579 0.0188 0.0571
2R:12037160-12037310:minus -22.7041 2.56881 13.6606 7.45872 -18.7551 -19.1224 -9.56881 3.375
0.385321 0.00395 0.00407 0.0000112 0.000894 0.0144 0.0175 0.00284 0.00516 0.0166
2R:12037160-12037310:plus -21.7041 1.95413 -0.706422 4.66972 -28.1939 -17.4184 -20.3303 8.30556
4.36697 0.00231 0.0051 0.0146 0.00466 0.0482 0.00585 0.0218 0.000489 0.00379
2R:12038120-12038270:minus -13.4082 1.6789 1.29358 3.18349 -18.449 -9.20408 -15.4862 4.29167
1.17431 0.000867 0.00565 0.00746 0.00837 0.0107 0.00225 0.00987 0.00356 0.0134
2R:12038120-12038270:plus -32.449 0.385321 7.3211 3.78899 -27.9694 -18.6327 -17.6789 8.05556 0.229358
0.0195 0.00902 0.00062 0.00632 0.0437 0.0137 0.0142 0.000565 0.0173
2R:12048620-12048770:minus -33.2653 -0.394495 6.46789 1.92661 -18.7857 -0.469388 -16.578 -3.25
3.41284 0.0277 0.0118 0.000933 0.0148 0.0148 0.000533 0.012 0.0411 0.006
2R:12048620-12048770:plus -23.0306 6.54128 8.27523 0 -27.3776 -38.1837 -19.3119 7.73611 7.22018
0.00505 0.000818 0.000378 0.0269 0.032 0.187 0.0183 0.000677 0.00119
2R:12049140-12049290:minus -32.8469 3.09174 -3.90826 4.59633 -18.6837 -25.8265 -20.1009 3.36111
1.38532 0.0243 0.00337 0.037 0.00478 0.0126 0.0212 0.021 0.00519 0.0123
2R:12049140-12049290:plus -34.3776 0.275229 7.66972 3.45872 -27.2347 -8.79592 -22.6147 0.222222 -0.972477
0.0319 0.00938 0.000521 0.00731 0.0283 0.0019 0.0329 0.0156 0.0259
2R:12050980-12051130:minus -31.4796 -2.54128 2 7.02752 -18.2245 -19.0816 -14.9083 6.44444 2.36697
0.0128 0.0227 0.00578 0.00122 0.00877 0.017 0.00881 0.00133 0.00828
2R:12050980-12051130:plus -31.551 -2.98165 0.917431 5.22018 -18.7857 -18.5714 -14.0275 2.01389 -0.853211
0.0134 0.0257 0.00851 0.00418 0.0148 0.0136 0.0073 0.00858 0.0251
2R:12058340-12058490:minus -33.2959 -1.3578 -1.12844 1.25688 -17.7143 -17.2755 -22.4587 2.77778
-2.46789 0.0279 0.016 0.0166 0.0192 0.00627 0.00544 0.032 0.00649 0.0384
2R:12058340-12058490:plus -21.8469 0.633028 5.88991 3.51376 -28.5306 -18.3061 -13.8991 6.38889 -2.52294
0.00247 0.00826 0.00121 0.00709 0.061 0.0114 0.00709 0.00137 0.0391
2R:12066220-12066370:minus -31.6633 -1.16514 8.08257 6.01835 -18.2245 0.316327 -8.11009 5.97222
3.20183 0.0141 0.0151 0.000419 0.00248 0.00877 0.000243 0.00215 0.00168 0.0065
2R:12066220-12066370:plus -22.8571 12.3761 2.38532 2.88991 20.1735 -18.0102 -19.3394 -2.43056 -2.94495
0.00427 0.0000356 0.00501 0.00942 5.68e-07 0.00812 0.0184 0.0334 0.0441
2R:12066500-12066650:minus -21.7347 5.61468 -0.183486 0.899083 -18.9796 0.530612 -12.8807 3.34722
1.63303 0.00235 0.00126 0.0123 0.0212 0.0155 0.000203 0.00563 0.00522 0.0112
2R:12066500-12066650:plus -21.9286 -2.06422 -2.42202 2.53211 -27.5306 -0.0204082 -21.3853 6.83333
-4.02752 0.0027 0.0198 0.0244 0.0113 0.0351 0.000411 0.0264 0.0011 0.056
2R:12067540-12067690:minus -31.898 2.33028 -1.24771 3.88991 -16.449 -8.5 -19.4128 1.18056 1.78899
0.0164 0.00445 0.0172 0.00608 0.00307 0.00126 0.0187 0.0114 0.0103
2R:12067540-12067690:plus -31.3265 1.58716 -2.73394 3.54128 -26.9388 -25.5306 -6.40367 1.69444 12.2752
0.0114 0.00584 0.0268 0.00702 0.0215 0.0205 0.00146 0.0096 0.0000141
2R:12068160-12068310:minus -31.5612 -1.57798 11.0092 7.29358 -26.898 -17.3469 -19.5872 -0.958333
-3.10092 0.0134 0.0171 0.0000797 0.00104 0.0203 0.0058 0.0192 0.0223 0.0458
2R:12068160-12068310:plus -14.3776 -0.715596 3.81651 9.3211 -17.6837 -0.244898 -20.7706 3.76389
-1.17431 0.00134 0.0131 0.00289 0.000391 0.0058 0.000496 0.0236 0.00442 0.0275
2R:12073440-12073590:minus -32.2245 1.90826 1.66055 -0.422018 -17.1837 2.05102 -12.6697 4.16667
-2.50459 0.0183 0.00519 0.00654 0.0305 0.00366 0.0000468 0.00536 0.00375 0.0388
2R:12073440-12073590:plus -30.6531 5.17431 -2.12844 6.00917 -18.051 -9.60204 -11.3853 1.91667 -0.238532
0.00968 0.00151 0.0224 0.00255 0.00746 0.00334 0.00404 0.00888 0.0203
2R:12073980-12074130:minus -41.6327 4.49541 1.3578 4.22936 -18.449 0.0204082 -15.1651 6.36111
3.34862 0.0631 0.00199 0.00728 0.00525 0.0107 0.000377 0.00927 0.00139 0.00609
2R:12073980-12074130:plus -12.2551 1.45872 4.85321 7.88991 11.4286 1.2449 -12.6972 4.58333 -3.23853
0.000379 0.00613 0.00189 0.00062 1.97e-06 0.0000797 0.00539 0.00315 0.047

2R:12075340-12075490:minus -31.1735 0.385321 -0.798165 2.31193 -18.7143 -18.7857 -8.92661 1.02778
3.90826 0.0109 0.00902 0.015 0.0123 0.0136 0.0146 0.00252 0.012 0.00462
2R:12075340-12075490:plus -23.4082 5.2844 4.42202 2.16514 -18.6837 -9.86735 -20.6147 0 3.50459
0.00623 0.00145 0.00226 0.0132 0.0126 0.00391 0.0229 0.0167 0.00568
2R:12083780-12083930:minus -30.6633 8.37615 -2.05505 1.25688 -27.2347 -17.5714 -13.8807 3.5 5.58716
0.00976 0.00031 0.022 0.0192 0.0283 0.00616 0.00706 0.00491 0.00237
2R:12083780-12083930:plus -31.5204 1.09174 13.156 7.2844 -26.5306 -17.9796 -22.4679 -2.19444 1.02752
0.0131 0.007 0.0000178 0.00105 0.0185 0.00764 0.032 0.0314 0.0141
2R:12084480-12084630:minus -30.2857 -2.92661 0.53211 3.13761 -17.1531 -8.87755 -10.844 1.08333
1.63303 0.00887 0.0253 0.00971 0.00854 0.00364 0.00198 0.00362 0.0118 0.0112
2R:12084480-12084630:plus -12.0714 5.40367 9.73394 7.78899 -18.4184 0.244898 7.48624 -0.388889
5.70642 0.000318 0.00138 0.000166 0.000642 0.0101 0.000285 0.0000273 0.0188 0.00222
2R:12097960-12098110:minus -11.5918 0.889908 2.13761 6.12844 -18.4184 -17.2041 -21 -5.33333 -4.05505
0.000257 0.00753 0.00549 0.00231 0.0101 0.00507 0.0246 0.0663 0.0564
2R:12097960-12098110:plus -22.2245 4.20183 9.53211 10.5963 -17.1531 0.27551 -6.98165 7.04167 4.9633
0.00325 0.00223 0.000187 0.0001 0.00364 0.000257 0.00168 0.000984 0.00316
2R:12106980-12107130:minus -33.0306 3.68367 -3.51376 5.66055 -18.3776 0.979592 -16.2018 3.90278
-3.92661 0.026 0.00144 0.0333 0.00317 0.00926 0.000136 0.0112 0.00418 0.0546
2R:12106980-12107130:plus -41.4082 1.44037 -1.3578 5.57798 -19.0204 -17.3469 -25.0459 7.06944 1.2844
0.0557 0.00617 0.0178 0.0034 0.0158 0.0058 0.0474 0.00097 0.0129
2R:12108700-12108850:minus -32.6327 -2.53211 3.2844 3.3578 1.65306 -0.540816 -22.8165 -0.569444
-9.12844 0.0212 0.0226 0.00356 0.00767 0.0000586 0.00054 0.034 0.0199 0.17
2R:12108700-12108850:plus 5.36735 -2.80734 8.01835 1.92661 -18.449 -28.3367 -12.2385 -4.88889 11.8532
0.0000111 0.0244 0.000433 0.0148 0.0107 0.0606 0.00486 0.0602 0.0000428
2R:12109240-12109390:minus -23.0408 13.3878 13.9725 1.78899 -26.898 -17.7143 -13.0642 -0.638889
6.95413 0.00512 0.0000869 7.38e-06 0.0158 0.0203 0.00634 0.00587 0.0203 0.00136
2R:12109240-12109390:plus -21.1122 13.5306 -1.0367 3.59633 -18.3776 -26.3367 -10.3761 0.138889 8.62385
0.00187 0.0000206 0.0161 0.00693 0.00926 0.0221 0.0033 0.016 0.00048
2R:12113760-12113910:minus -24.2551 -0.192661 4.15596 9.92661 -27.1939 0.316327 -3.3945 -0.791667
-0.862385 0.00768 0.011 0.00251 0.000169 0.0267 0.000243 0.000631 0.0212 0.0251
2R:12113760-12113910:plus 15.8878 2.47706 3.86239 10.8624 -26.2347 -27.3776 5.02752 -1.52778 11.8991
1.77e-07 0.00421 0.00283 0.0000687 0.0177 0.0351 0.0000571 0.0262 0.0000255
2R:12117580-12117730:minus -13.7449 7.88991 -1.12844 2.23853 -19.0102 0.0918367 -17.4771 2.375
-1.3578 0.00107 0.000405 0.0166 0.0128 0.0157 0.000323 0.0138 0.00753 0.0286
2R:12117580-12117730:plus -21.5816 0.844037 14.9633 7.29358 -18.1224 -17.7551 -21.8991 5.38889 -6.41284
0.00205 0.00766 1.77e-06 0.00104 0.00782 0.00643 0.029 0.0022 0.0983
2R:12128200-12128350:minus -14.102 2.29358 2.17431 -0.899083 -18.9796 -17.0816 -15.6055 -2.75
-2.94495 0.00117 0.00451 0.00542 0.0356 0.0155 0.00487 0.0101 0.0363 0.0441
2R:12128200-12128350:plus -33.0816 4.40367 5.23853 3.51376 -9.16327 -0.244898 -12.9908 11.6806
-0.229358 0.0268 0.00206 0.00161 0.00709 0.00249 0.000496 0.00577 0.0000419 0.0202
2R:12152020-12152170:minus -41.8163 -0.0550459 0.733945 3.33028 -17.9796 -8.65306 -12.6147 -1.27778
0.697248 0.0662 0.0105 0.00906 0.00778 0.00726 0.00156 0.00529 0.0244 0.0154
2R:12152020-12152170:plus -22.4082 1.46789 6.14679 3.6422 20.4694 -9.72449 0.449541 1.58333 -2.22936
0.00345 0.0061 0.00108 0.00669 1.65e-07 0.00352 0.000254 0.00997 0.0356
2R:12157960-12158110:minus -21.9286 -2.41284 0.807339 5.85321 -18.2653 -27.1224 -13.5596 0.375
6.11927 0.0027 0.0219 0.00884 0.00287 0.00882 0.0325 0.00657 0.0149 0.00178
2R:12157960-12158110:plus -24.449 -1.19266 0.348624 7.27523 -8.93878 -9.60204 -15.8716 4.86111 11.8532
0.00802 0.0152 0.0103 0.00109 0.00216 0.00334 0.0106 0.00279 0.0000428
2R:12159360-12159510:minus -22.2653 2.89908 0.302752 2.11009 20.4694 -8.5 5.80734 5.5 4.77064
0.00331 0.00361 0.0105 0.0136 1.65e-07 0.00126 0.000044 0.00209 0.0034
2R:12159360-12159510:plus -24.5204 3.31193 -0.458716 3.57798 -18.0816 -27.5612 -5.46789 2.26389
-4.20183 0.00813 0.0031 0.0135 0.00696 0.00759 0.0393 0.00113 0.00784 0.0584
2R:12176680-12176830:minus -40.7755 0.110092 0.981651 4.08257 -27.5714 -8.5 -10.6514 6.02778 1.7156
0.0433 0.00993 0.00832 0.00558 0.0357 0.00126 0.00348 0.00164 0.0107

2R:12176680-12176830:plus -13.5204 2.77982 -0.633028 7.45872 -27.1939 -17.8265 18.6147 3.47222
3.66972 0.00097 0.00377 0.0142 0.000894 0.0267 0.00699 3.74e-07 0.00497 0.00514
2R:12178920-12179070:minus -32.3673 -2.66972 10.1376 7.29358 -18.7857 -27.898 -4.11927 7.04167
1.48624 0.0189 0.0235 0.000131 0.00104 0.0148 0.0537 0.000772 0.000984 0.0118
2R:12178920-12179070:plus -32.2857 -4.3945 2.45872 3.13761 -7.63265 -18.2755 -16.2018 3.58333 3.23853
0.0185 0.0376 0.00488 0.00854 0.000479 0.0112 0.0112 0.00475 0.00639
2R:12181780-12181930:minus -21.9592 3.02752 0.853211 6.05505 -18.7143 -9.27551 -23.633 5.11111
0.59633 0.00279 0.00344 0.0087 0.0024 0.0136 0.00244 0.0388 0.0025 0.016
2R:12181780-12181930:plus -32.1122 -0.862385 14.7248 7.2844 -26.2653 -8.53061 -24.1835 0.416667
5.34862 0.0178 0.0137 2.82e-06 0.00105 0.018 0.00136 0.0422 0.0147 0.00271
2R:12208200-12208350:minus -13.3878 -1.10092 4.6789 2.93578 20.2041 -18.8265 -12.5505 1.5 -2.73394
0.000831 0.0148 0.00203 0.00927 3.64e-07 0.0148 0.00522 0.0103 0.0416
2R:12208200-12208350:plus -33.5612 2.93578 1.3578 1.3211 -18.0102 -8.68367 -32.2477 5.875 -8.51376
0.0288 0.00356 0.00728 0.0189 0.00732 0.00157 0.122 0.00176 0.151
2R:12209580-12209730:minus -33.1122 6.6055 0.146789 1.92661 10.4286 -8.72449 -21.5138 2.72222
-0.605505 0.0271 0.000793 0.011 0.0148 0.0000139 0.00169 0.027 0.00663 0.023
2R:12209580-12209730:plus -22.6224 0.633028 12.1193 2.80734 -8.86735 -18.7551 -19.5963 0.138889 -4.0367
0.00371 0.00826 0.0000394 0.00985 0.00173 0.0141 0.0192 0.016 0.0562
2R:12223480-12223630:minus -32.5102 0.53211 -0.220183 1.80734 -18.8265 -9.34694 -14.0917 -0.236111
9.45872 0.0202 0.00856 0.0125 0.0156 0.0149 0.00261 0.0074 0.018 0.000337
2R:12223480-12223630:plus -13.4796 8.90826 14.5505 7.2844 -8.96939 -8.45918 -12.9633 -0.0555556
-6.49541 0.000935 0.000235 3.58e-06 0.00105 0.00226 0.00111 0.00573 0.017 0.101
2R:12224180-12224330:minus -21.8878 2.9633 1.66055 4.3945 -17.3776 -8.5 -16.4587 1.43056 -1.15596
0.00258 0.00353 0.00654 0.00499 0.00419 0.00126 0.0117 0.0105 0.0273
2R:12224180-12224330:plus -20.6633 -2.88991 13.7431 6.11009 -18.4184 -17.5 -7.93578 -5.11111 -2.69725
0.00173 0.025 0.0000103 0.00233 0.0101 0.00599 0.00208 0.0632 0.0411
2R:12224660-12224810:minus -23.2245 1.86239 -0.146789 -0.504587 -16.4898 0.0510204 -13.9266
4.875 5.49541 0.00576 0.00528 0.0122 0.0312 0.00312 0.000349 0.00714 0.00277 0.0025
2R:12224660-12224810:plus -31.3265 5.47706 6.85321 6.06422 -27.1531 -19.1531 -4.04587 -3.15278 5.70642
0.0114 0.00133 0.000779 0.00239 0.0249 0.0177 0.000756 0.0402 0.00222
2R:12231860-12232010:minus -32.4796 6.40367 1.02752 5.86239 -18.7857 -19.0102 -18.211 -3.08333
1.94495 0.02 0.000874 0.00819 0.00282 0.0148 0.0159 0.0154 0.0395 0.00968
2R:12231860-12232010:plus -31.7041 0.422018 -1.27523 2.53211 11.6531 -0.244898 -18.6239 -3.06944
6.69725 0.0146 0.0089 0.0174 0.0113 1.55e-06 0.000496 0.0165 0.0393 0.00145
2R:12234900-12235050:minus -21.8571 14.4862 6.70642 3.59633 -18.4898 1.05102 -9.18349 7.27778
-2.01835 0.00249 5.19e-06 0.000832 0.00693 0.0116 0.000107 0.00265 0.000869 0.0334
2R:12234900-12235050:plus -14.1429 13.8532 0.862385 1.89908 -18.4898 -17.7857 -12.3486 3.125 4.3945
0.0012 9.88e-06 0.00867 0.0151 0.0116 0.0068 0.00498 0.00569 0.00375
2R:1226780-1226930:minus -33.2959 12.9908 2.45872 5.77982 -27.4184 -9.53061 -25.8349 1.625 2.85321
0.0279 0.0000223 0.00488 0.00294 0.0323 0.0031 0.0524 0.00983 0.00733
2R:1226780-1226930:plus -33.1122 4.94495 0.366972 4.75229 -27.5306 0.244898 -15.6147 6.81944 2.04587
0.0271 0.00166 0.0103 0.00461 0.0351 0.000285 0.0101 0.0011 0.00919
2R:1228340-1228490:minus -14.8571 13.4592 3.46789 2.41284 -27.7551 -8.45918 -6.61468 6.81944 -2.87156
0.0016 0.000064 0.00331 0.0118 0.0385 0.00111 0.00154 0.0011 0.043
2R:1228340-1228490:plus -40.2143 -2.24771 2.66972 1.56881 -17.449 -18.0102 -21.789 4.125 -4.20183
0.0372 0.0209 0.0045 0.0172 0.00484 0.00812 0.0284 0.00382 0.0584
2R:12319700-12319850:minus -23.1837 0.605505 -1.82569 4.07339 -26.1939 -9.27551 -7.92661 4.09722
3.81651 0.00553 0.00834 0.0205 0.00562 0.0175 0.00244 0.00207 0.00386 0.00482
2R:12319700-12319850:plus -23.2959 0.733945 4.70642 6.00917 -17.4184 -17.6429 -10.7064 6.77778 -6.75229
0.00605 0.00797 0.00201 0.00255 0.00457 0.00619 0.00352 0.00113 0.107
2R:1237840-1237990:minus -41.3265 3.83673 0.201835 2.62385 -8.93878 -18.4184 -16.9174 1.94444 -4.11009
0.0541 0.00115 0.0108 0.0108 0.00216 0.0127 0.0126 0.00879 0.0571
2R:1237840-1237990:plus -31.7653 4.05102 -0.633028 6.21101 -18.4184 -8.72449 -7.12844 4.36111
4.40367 0.0152 0.000335 0.0142 0.00213 0.0101 0.00169 0.00174 0.00346 0.00373

2R:1238340-1238490:minus -23.1939 2.50459 1.86239 7.27523 -27.1531 -18.2755 -18.8073 6.88889 -0.669725
0.00558 0.00417 0.00608 0.00109 0.0249 0.0112 0.0169 0.00107 0.0236
2R:1238340-1238490:plus -22.8878 -1.17431 7.86239 4.55046 -9.45918 -7.79592 -14.7523 -0.319444
-2.75229 0.00441 0.0151 0.000471 0.00482 0.00287 0.000723 0.00853 0.0184 0.0418
2R:12458780-12458930:minus -12.3776 -4.06422 3.69725 2.78899 20.4694 10.0918 -13.8899 4.22222
1.23853 0.000403 0.0344 0.00303 0.00992 1.65e-07 9.34e-06 0.00708 0.00367 0.0132
2R:12458780-12458930:plus -23.449 2.16514 1.85321 0.192661 -18.4898 -8.53061 -19.9083 8.27778 0.587156
0.00631 0.00473 0.0061 0.0256 0.0116 0.00136 0.0203 0.000497 0.016
2R:12466920-12467070:minus -41.8163 6.04587 -0.46789 2.70642 -18.051 -0.173469 -21.789 -2.27778
-4.86239 0.0662 0.00104 0.0135 0.0103 0.00746 0.000456 0.0284 0.0321 0.0705
2R:12466920-12467070:plus -30.551 0.165138 5.58716 1.40367 -18.7551 -27.9694 -15.0734 3.56944 8.11009
0.00959 0.00974 0.00139 0.0184 0.0144 0.0547 0.00911 0.00478 0.000597
2R:12467880-12468030:minus -14.0816 0.440367 4.97248 2.25688 -18.1531 -9.42857 -19.6422 -3.80556
-4.99083 0.00117 0.00885 0.0018 0.0127 0.00829 0.0027 0.0194 0.047 0.0726
2R:12467880-12468030:plus -40.2143 3.26606 4.2844 6.47706 0.846939 -18.0102 -12.6606 5.18056 3.9633
0.0372 0.00315 0.00239 0.00179 0.000197 0.00812 0.00535 0.00242 0.00446
2R:12469040-12469190:minus -21.6633 1.40367 -1.86239 4.59633 -9.23469 -26.3367 -6.80734 -2.80556
-2.85321 0.00223 0.00625 0.0208 0.00478 0.00277 0.0221 0.00161 0.0368 0.0429
2R:12469040-12469190:plus -31.5918 1.55963 -0.862385 9.92661 -17.3776 -9.57143 -30.3211 -2.47222
-9.46789 0.0136 0.0059 0.0153 0.000169 0.00419 0.00326 0.0951 0.0338 0.179
2R:12469700-12469850:minus -13.0408 0.816514 1.21101 7.62385 -7.93878 -26.5612 -11.2294 2.93056
1.22018 0.000576 0.00773 0.00768 0.000759 0.000767 0.0237 0.00391 0.00612 0.0133
2R:12469700-12469850:plus -22.8163 0.93578 4.73394 2.06422 -17.4184 -9.16327 -18.7798 0.402778 3.36697
0.00418 0.00741 0.00199 0.0139 0.00457 0.00223 0.0169 0.0147 0.00607
2R:12470200-12470350:minus -24.3673 -0.238532 12.0459 7.2844 -16.4184 -26.8265 -16.4404 6.56944
9.92661 0.00791 0.0112 0.0000414 0.00105 0.00304 0.0285 0.0117 0.00125 0.000193
2R:12470200-12470350:plus -21.2245 2.53211 1.3578 3.3211 -26.9388 -8.57143 -12.4862 2.88889 1.3211
0.0019 0.00413 0.00728 0.00782 0.0215 0.00149 0.00514 0.00622 0.0127
2R:12470900-12471050:minus -3.14286 1.46789 3.16514 8.81651 -17.449 -19.0102 -4.73394 5.34722
7.15596 0.0000468 0.0061 0.00373 0.000501 0.00484 0.0159 0.00092 0.00225 0.00124
2R:12470900-12471050:plus -32.2653 -3.73394 9.36697 4.17431 -18.5306 -8.72449 -8.49541 4.33333 11.8532
0.0184 0.0315 0.000204 0.00537 0.0118 0.00169 0.00232 0.0035 0.0000428
2R:12471440-12471590:minus -22.6939 -0.0550459 1.24771 2.45872 -7.93878 -18.3469 2.36697 -2.01389
11.3853 0.00388 0.0105 0.00758 0.0116 0.000767 0.0124 0.000147 0.0299 0.0000815
2R:12471440-12471590:plus -14.7041 0.155963 2.45872 5.86239 -8.89796 -16.9388 -3.30275 1.15278 9.30275
0.00154 0.00977 0.00488 0.00282 0.00189 0.00433 0.000615 0.0115 0.00038
2R:12473160-12473310:minus -41.7041 5.3945 6.00917 2.07339 -18.7857 -17.5 -20.055 7.31944
-0.284404 0.0641 0.00138 0.00115 0.0138 0.0148 0.00599 0.0208 0.00085 0.0207
2R:12473160-12473310:plus -32.0306 5.84404 -0.688073 3.24771 -27.2959 -17.5714 -13.367 8.68056
-4.85321 0.0172 0.00113 0.0145 0.00808 0.0303 0.00616 0.00629 0.000391 0.0703
2R:12473500-12473650:minus -23.5612 4.47706 4.08257 10.5963 -8.93878 -26.8265 -16.5413 1.59722
-1.95413 0.00653 0.002 0.00259 0.0001 0.00216 0.0285 0.0119 0.00993 0.0328
2R:12473500-12473650:plus -40.6531 1.3945 3.38532 1.06422 10.9184 0.755102 -15.7798 -4.05556 1.58716
0.0425 0.00627 0.00342 0.0203 4.19e-06 0.000175 0.0104 0.0499 0.0113
2R:12474180-12474330:minus -40.5918 -2.06422 3.78899 0.963303 -8.64286 -26.6327 -16.6055 5.04167
0.394495 0.0422 0.0198 0.00292 0.0208 0.00139 0.0251 0.012 0.00258 0.0166
2R:12474180-12474330:plus -32.398 2.81651 3.23853 4.7156 -27.3469 -18.4184 -8.92661 3.31944 3.93578
0.019 0.00372 0.00362 0.00464 0.0318 0.0127 0.00252 0.00528 0.00456
2R:12476680-12476830:minus -22.7755 2.52294 12.1101 7.00917 -18.4592 -28.3776 -15.6789 1.84722
7.42202 0.00412 0.00414 0.0000401 0.00126 0.0109 0.0609 0.0102 0.0091 0.00106
2R:12476680-12476830:plus -21.7347 6.18349 1.29358 0.743119 11.3571 -17.8265 -19.4679 5.13889 -0.238532
0.00235 0.000972 0.00746 0.0221 2.44e-06 0.00699 0.0188 0.00247 0.0203
2R:1247720-1247870:minus -31 1.05505 3.13761 2.19266 -18.7143 -17.0102 -12.5413 1.16667 -2.00917
0.0105 0.00709 0.00377 0.0131 0.0136 0.00453 0.00521 0.0115 0.0333

2R:1247720-1247870:plus -32.0408 1.68807 -0.614679 3.86239 -26.9694 -18.8571 -16.6514 0.708333
3.56881 0.0174 0.00563 0.0141 0.00622 0.023 0.0151 0.0121 0.0134 0.00543
2R:12481820-12481970:minus -12.2551 3.54128 -0.917431 3.88991 -8.71429 -8.5 -1.79817 -2.05556
7.68807 0.000379 0.00285 0.0156 0.00608 0.00159 0.00126 0.000424 0.0303 0.000841
2R:12481820-12481970:plus -12.4796 0.688073 3.22018 3.97248 -9.0102 -25.5612 -6.33028 -3.95833 3.66972
0.000433 0.0081 0.00365 0.00587 0.00236 0.0206 0.00143 0.0488 0.00514
2R:1250640-1250790:minus -32.551 7.81651 -2.88073 0.807339 -27.4592 -17.5714 -19.1927 8.52778 7.63303
0.0207 0.000422 0.0279 0.0217 0.0329 0.00616 0.018 0.000429 0.000928
2R:1250640-1250790:plus -33.2653 2.85321 0.0550459 1.81651 -18.7857 0.459184 -18.8991 6.66667
1.90826 0.0277 0.00367 0.0114 0.0156 0.0148 0.000213 0.0172 0.00119 0.00974
2R:12577080-12577230:minus -23.2245 -2.66972 5.15596 5.37615 -27.1939 -9.79592 -8.09174 0.333333
6.38532 0.00576 0.0235 0.00167 0.00384 0.0267 0.00377 0.00214 0.0151 0.00157
2R:12577080-12577230:plus -12.9286 4.10092 6.50459 1.44954 -17.4184 -18.0816 -18.6972 3.93056 7.22018
0.000539 0.00231 0.000917 0.0179 0.00457 0.00934 0.0166 0.00413 0.00119
2R:12578080-12578230:minus -22.0408 4.74312 -2.93578 9.08257 -27.1939 -0.173469 -22.1101 -1.63889
-0.0275229 0.00296 0.00181 0.0283 0.000444 0.0267 0.000456 0.0301 0.027 0.0186
2R:12578080-12578230:plus -14.0102 -1.11009 0.330275 1.44037 -19.051 -8.94898 -16.8257 2.83333 -2.12844
0.00114 0.0148 0.0104 0.018 0.0162 0.00203 0.0125 0.00635 0.0345
2R:12578560-12578710:minus -31.6939 3.3945 0.458716 6.55963 -18.6429 -9.79592 -6.53211 -1.47222
-9.10092 0.0142 0.00301 0.00995 0.00172 0.0121 0.00377 0.00151 0.0258 0.169
2R:12578560-12578710:plus -20.8878 4.22936 9.09174 3.90826 -18.7551 -18.5612 -11.5872 4.01389 3.62385
0.0018 0.0022 0.000241 0.00603 0.0144 0.0132 0.00422 0.004 0.00523
2R:12579060-12579210:minus -23.3367 -1.33945 5.66972 4.59633 -27.3061 -27.898 -24.1009 6.65278
5.38532 0.00614 0.0159 0.00134 0.00478 0.0315 0.0537 0.0417 0.0012 0.00267
2R:12579060-12579210:plus -22.7041 4.42202 3.90826 9.7156 -18.4184 -28.3367 -5.68807 -0.333333
3.08257 0.00395 0.00204 0.00278 0.000242 0.0101 0.0606 0.0012 0.0185 0.00681
2R:12579540-12579690:minus -31.2857 -2.59633 4.95413 2.33028 -18.7551 -27.1122 -7.31193 2.81944
-3.76147 0.0113 0.023 0.00181 0.0123 0.0144 0.0316 0.00181 0.00639 0.0523
2R:12579540-12579690:plus -23.449 6.79817 -2.17431 2.6789 -27.4592 0.826531 -24.4312 2.09722 2.58716
0.00631 0.00072 0.0227 0.0105 0.0329 0.000153 0.0437 0.00832 0.00779
2R:12661500-12661650:minus -22.9184 9.55963 5.79817 3.44954 -18.7551 0.0918367 -14.7431 2.29167
3.66972 0.00447 0.000167 0.00126 0.00738 0.0144 0.000323 0.00851 0.00776 0.00514
2R:12661500-12661650:plus -22.1531 13.4592 -0.376147 2.50459 -18.4184 -17.9796 -21.8257 9.13889
-2.49541 0.0031 0.000064 0.0131 0.0114 0.0101 0.00764 0.0286 0.000293 0.0387
2R:12671040-12671190:minus -24.4592 -0.0733945 1.02752 -0.706422 -17.7551 0.683673 -28.4954
0.555556 -5.16514 0.00802 0.0106 0.00819 0.0335 0.00658 0.000184 0.0736 0.014 0.0753
2R:12671040-12671190:plus 7.04082 0.449541 -4.33945 2.54128 20.4694 -18.051 1.01835 1.45833 -0.412844
1.83e-06 0.00882 0.0416 0.0112 1.65e-07 0.00886 0.000219 0.0104 0.0213
2R:12671760-12671910:minus -32.6224 13.3878 -2.86239 2.55046 -8.44898 -18.0816 -10.8991 3.19444
5.66055 0.0211 0.0000869 0.0277 0.0112 0.00109 0.00934 0.00366 0.00554 0.00227
2R:12671760-12671910:plus -23.7041 1.94495 6.87156 2.75229 -17.449 -26.898 -12.1468 -1.26389 -0.302752
0.00669 0.00512 0.000772 0.0101 0.00484 0.0302 0.00476 0.0243 0.0208
2R:12672260-12672410:minus -32.9286 1.02752 -0.697248 6.21101 -19.2755 0.979592 -27.1835 0.986111
-1.6422 0.025 0.00717 0.0145 0.00213 0.0164 0.000136 0.0619 0.0122 0.0304
2R:12672260-12672410:plus -20.4388 -1.33028 1.63303 9.61468 -18.0816 -26.602 -23.0734 0.125 -3.51376
0.00167 0.0159 0.00661 0.000255 0.00759 0.0248 0.0355 0.0161 0.0498
2R:12674460-12674610:minus -12.3776 3.97959 5.55046 10.7615 -26.4898 -9.27551 -18.4312 4.375
1.95413 0.000403 0.000669 0.00141 0.0000826 0.0183 0.00244 0.016 0.00344 0.00964
2R:12674460-12674610:plus -32.7041 0.697248 -1.86239 1.94495 0.846939 -8.53061 -20.9908 0.305556 -4.02752
0.0222 0.00807 0.0208 0.0147 0.000197 0.00136 0.0245 0.0152 0.056
2R:12697900-12698050:minus -12.7347 4.66055 4.16514 -0.0917431 -18.0816 -16.0102 -11.9174 -1.45833
5.44037 0.000468 0.00187 0.00251 0.0276 0.00759 0.00406 0.00453 0.0257 0.00259
2R:12697900-12698050:plus -23.5918 -0.587156 -1.58716 2.00917 -8.89796 -26.898 -10.3578 10.7361
2.20183 0.00656 0.0126 0.0191 0.0142 0.00189 0.0302 0.00329 0.0000944 0.00869

2R:12702820-12702970:minus -33.7041 -0.348624 1.88991 3.18349 -27.898 -8.94898 -22.8532 6.73611 -2.15596 0.0293 0.0116 0.00602 0.00837 0.04 0.00203 0.0342 0.00115 0.0348
2R:12702820-12702970:plus -31.3673 2.78899 -0.33945 7.33028 -26.1939 -7.72449 -26.5505 5.84722 -8.41284 0.0116 0.00376 0.0129 0.00103 0.0175 0.00068 0.0572 0.00178 0.148
2R:12705420-12705570:minus -31.5102 -3.30275 -0.486239 3.30275 -9.0102 -18.0816 -19.3211 0.319444 3.29358 0.013 0.028 0.0136 0.00789 0.00236 0.00934 0.0184 0.0151 0.00623
2R:12705420-12705570:plus -31.6633 0.853211 14.0183 7.29358 -18.1531 -17.7857 -15.2294 1.70833 1.89908 0.0141 0.00763 7.05e-06 0.00104 0.00829 0.0068 0.00939 0.00955 0.0098
2R:12706420-12706570:minus -41.102 12.6055 1.74312 -1.50459 -28.3061 -8.5 -12.5688 4.52778 3.66972 0.0472 0.0000301 0.00635 0.0434 0.0553 0.00126 0.00524 0.00322 0.00514
2R:12706420-12706570:plus -30.398 3.98165 3.44954 4.33945 -18.2245 -36.1531 -6.25688 -2.31944 2.11009 0.00901 0.00242 0.00334 0.00509 0.00877 0.0908 0.0014 0.0325 0.00898
2R:12711580-12711730:minus -14.449 5.73394 5.09174 4.0367 -8.60204 -18.5612 -11.1009 4.26389 3.25688 0.0014 0.00119 0.00171 0.00578 0.00125 0.0132 0.00381 0.0036 0.00632
2R:12711580-12711730:plus -24.2959 -1.82569 0.238532 0.715596 -18.4898 -9.79592 -2.77982 5.41667 1.59633 0.00781 0.0185 0.0107 0.0223 0.0116 0.00377 0.000537 0.00218 0.0113
2R:12716340-12716490:minus -32.8571 -3.07339 1.76147 1.31193 -9.20408 -9.16327 -26.0367 4.47222 -2.6422 0.0244 0.0263 0.00631 0.0189 0.00262 0.00223 0.0537 0.0033 0.0405
2R:12716340-12716490:plus -14.7449 -0.59633 8.30275 2.93578 -27.5306 -17.3469 -16.0092 3.13889 4.13761 0.00157 0.0126 0.000372 0.00927 0.0351 0.0058 0.0109 0.00566 0.00408
2R:12717740-12717890:minus -14.3367 -2.88991 -1.08257 2.6422 -8.37755 -17.3469 -27 0.847222 -7.90826 0.00131 0.025 0.0164 0.0107 0.00106 0.0058 0.0605 0.0128 0.133
2R:12717740-12717890:plus -21.4082 1.93578 1.11927 6.49541 -18.4184 -18.2755 -19.1376 -2.19444 3.16514 0.00198 0.00514 0.00793 0.00176 0.0101 0.0112 0.0178 0.0314 0.00656
2R:12718160-12718310:minus -23.898 -1.6789 -2.21101 9.59633 -17.6837 -19.0102 -23.5688 2.88889 -1.68807 0.00693 0.0177 0.023 0.000275 0.0058 0.0159 0.0384 0.00622 0.0308
2R:12718160-12718310:plus -13.2959 1.82569 4.3945 6.12844 -17.3061 -9.68367 -7.30275 -3.06944 3.17431 0.000775 0.00535 0.00229 0.00231 0.00389 0.00342 0.00181 0.0393 0.00653
2R:12737820-12737970:minus -31.5918 -0.853211 -1.21101 3.6055 -17.2653 -17.9388 -23.8716 2.04167 -0.504587 0.0136 0.0137 0.017 0.00686 0.00387 0.00738 0.0403 0.00849 0.0221
2R:12737820-12737970:plus -13.2143 12.9541 11.0275 3.77064 -7.53061 -9.45918 -13.1284 -0.666667 4.80734 0.000714 0.0000231 0.0000785 0.00637 0.000407 0.00279 0.00595 0.0205 0.00334
2R:12742880-12743030:minus -21.8469 1.05505 0.0642202 2.61468 2.21429 0.0510204 -20.3945 6.15278 6.95413 0.00247 0.00709 0.0113 0.0109 0.0000277 0.000349 0.0221 0.00154 0.00136
2R:12742880-12743030:plus -33.602 1.11009 0.46789 4.14679 -27.602 -8.72449 -20.7615 7.54167 1.52294 0.0289 0.00695 0.00992 0.00542 0.0364 0.00169 0.0236 0.000754 0.0117
2R:12743660-12743810:minus -40.4388 -1.9633 2.40367 2.6789 -18.7143 -8.86735 -26.3303 6.58333 1.95413 0.0401 0.0192 0.00498 0.0105 0.0136 0.00198 0.0557 0.00124 0.00964
2R:12743660-12743810:plus -13.7041 13.8899 1.94495 4.04587 -17.7143 -7.5 -13.5963 -0.916667 1.2844 0.00104 9.42e-06 0.0059 0.00572 0.00627 0.000614 0.00663 0.022 0.0129
2R:12743980-12744130:minus -31.6224 5.37615 -1.52294 5.42202 -18.7449 -17.7857 -1.47706 1.27778 10.0367 0.0137 0.00139 0.0187 0.00374 0.0138 0.0068 0.000394 0.0111 0.000146
2R:12743980-12744130:plus -31.5918 0.311927 -3.3578 4.22018 -16.6735 -36.3061 -12.8716 -2.52778 -0.59633 0.0136 0.00925 0.0319 0.00531 0.00313 0.0959 0.00561 0.0343 0.0229
2R:12744700-12744850:minus -23.1122 -0.724771 7.07339 4.37615 -18.1837 0.316327 -15.3028 3.13889 -0.486239 0.0053 0.0131 0.000703 0.00504 0.00835 0.000243 0.00953 0.00566 0.0219
2R:12744700-12744850:plus -22.0408 0.477064 -0.183486 3.40367 -18.3469 0.0510204 -12.5963 1.15278 7.27523 0.00296 0.00873 0.0123 0.00759 0.00894 0.000349 0.00527 0.0115 0.00113
2R:12755140-12755290:minus -24.3367 -1.02752 0.981651 2.06422 -18.4184 -9.7551 -14.4862 1.19444 1.49541 0.00789 0.0145 0.00832 0.0139 0.0101 0.00358 0.00807 0.0114 0.0118
2R:12755140-12755290:plus -30.3673 -2.6789 -2.77982 1.81651 10.6531 19.0816 -11.0826 1.20833 -4.76147 0.00897 0.0236 0.0271 0.0156 0.0000102 1.12e-06 0.0038 0.0113 0.0686
2R:12755640-12755790:minus -33.3367 0.192661 -1.38532 -2.37615 -17.5612 -10.0204 -14.2294 2.36111 3.51376 0.0281 0.00965 0.018 0.0578 0.00544 0.00399 0.00763 0.00757 0.00566

2R:12755640-12755790:plus -20.4388 -1.92661 -0.825688 7.33945 2.17347 9.79592 -15.633 6.98611
3.66972 0.00167 0.019 0.0151 0.00102 0.0000296 0.0000183 0.0101 0.00101 0.00514
2R:12756720-12756870:minus -24.3265 9.88073 9.68807 6.59633 -8.93878 -18.2041 -9.90826 -0.388889
0.513761 0.00784 0.000142 0.000171 0.00164 0.00216 0.0103 0.00303 0.0188 0.0162
2R:12756720-12756870:plus -3.93878 3.47706 9.68807 10.7615 -17.6429 -8.93878 -13.7798 2.51389 5.23853
0.000117 0.00292 0.000171 0.0000826 0.00562 0.00201 0.00691 0.00716 0.00285
2R:12839420-12839570:minus -23.5918 14.8716 -0.59633 3.76147 -18.7143 -16.9388 -20.5046 9.72222
0.550459 0.00656 2.8e-06 0.0141 0.00645 0.0136 0.00433 0.0225 0.000199 0.0161
2R:12839420-12839570:plus -31.9286 4.01835 0.807339 0.311927 -8.89796 -7.57143 -21.7064 1.41667 -0.862385
0.0166 0.00239 0.00884 0.0247 0.00189 0.000644 0.028 0.0106 0.0251
2R:12842880-12843030:minus -23.1837 13.3878 1.3945 4.3211 -9.19388 -18.5612 -15.5963 4.81944
-4.75229 0.00553 0.0000869 0.00719 0.00514 0.00253 0.0132 0.0101 0.00284 0.0683
2R:12842880-12843030:plus -32.0714 9.19266 3.01835 2.29358 10.3571 -19.4184 -4.7156 1.51389 -0.770642
0.0176 0.000202 0.00394 0.0124 0.0000208 0.0198 0.000915 0.0102 0.0245
2R:12844860-12845010:minus -42 0.201835 -1.55963 3.44954 -27.2347 -8.79592 -23.8991 2.70833 1.2844
0.0684 0.00962 0.0189 0.00738 0.0283 0.0019 0.0404 0.00666 0.0129
2R:12844860-12845010:plus -22.7449 -3.44954 13.0092 7.29358 11.6939 -0.244898 -12.7523 0.861111
-0.504587 0.00404 0.0292 0.0000207 0.00104 1.17e-06 0.000496 0.00546 0.0127 0.0221
2R:12889360-12889510:minus -12.7755 1.01835 -1.82569 5.73394 20.1735 -0.244898 -18.6881 9.04167
-3.06422 0.000477 0.00719 0.0205 0.00307 5.68e-07 0.000496 0.0166 0.000312 0.0453
2R:12889360-12889510:plus -30.8061 -0.0183486 1.77064 2.47706 -18.1224 -9.5 -10.4128 5.54167 1.02752
0.0102 0.0104 0.00629 0.0115 0.00782 0.00294 0.00333 0.00205 0.0141
2R:12892860-12893010:minus -22.0816 3.83673 5.52294 7 0.877551 -7.79592 -13.2752 0.111111 0.321101
0.003 0.00115 0.00142 0.00132 0.00019 0.000723 0.00616 0.0162 0.0169
2R:12892860-12893010:plus -30.1429 2.75229 1.59633 2.88991 20.1735 0.979592 1.48624 6.93056 -1.37615
0.00866 0.00381 0.00669 0.00942 5.68e-07 0.000136 0.000192 0.00104 0.0287
2R:12896220-12896370:minus -12.8469 -2.15596 4.74312 2.95413 -26.9694 -19.1224 -3.52294 -1.19444
7.53211 0.000502 0.0203 0.00198 0.00917 0.023 0.0175 0.000653 0.0239 0.000968
2R:12896220-12896370:plus -31.4796 3.74312 7.2844 7.21101 -27.1939 9.5 -18.2202 5.27778 4.04587
0.0128 0.00265 0.000632 0.0011 0.0267 0.0000337 0.0155 0.00232 0.00421
2R:12903360-12903510:minus -22.8776 2.65138 -1.23853 6.33945 -18.6429 -26.2653 -9.95413 4.45833
1.12844 0.00428 0.00395 0.0172 0.00196 0.0121 0.0218 0.00305 0.00332 0.0137
2R:12903360-12903510:plus -23.7857 13.5306 0.366972 3.77064 -27.3469 -9.72449 -13.3761 5.98611 -2.17431
0.00679 0.0000206 0.0103 0.00637 0.0318 0.00352 0.0063 0.00167 0.035
2R:12906980-12907130:minus -34.4082 -0.495413 -1.84404 0.972477 -8.40816 -18.6429 -16.3028 -0.222222
2.33028 0.0319 0.0122 0.0206 0.0207 0.00108 0.0139 0.0114 0.0179 0.00836
2R:12906980-12907130:plus -32.398 13.4592 -0.642202 5.69725 11.4286 -19.4184 -21.2385 5.23611
-4.42202 0.019 0.000064 0.0143 0.00314 1.97e-06 0.0198 0.0257 0.00236 0.0626
2R:12907440-12907590:minus -44.1122 0.110092 -0.550459 0.486239 -27.2653 0.459184
-36.0826 10.1389 -10.367 0.0949 0.00993 0.0139 0.0237 0.0302 0.000213 0.178 0.000148 0.208
2R:12907440-12907590:plus -32.1939 2.27523 -2.13761 3.26606 10.9184 9.37755 -3.26606 4.97222
-1.61468 0.0181 0.00454 0.0225 0.00802 4.19e-06 0.0000363 0.000609 0.00266 0.0302
2R:12907880-12908030:minus -22.8163 -1.77064 2.84404 9.51376 -7.63265 -7.72449 -17.6147
-0.708333 4.88073 0.00418 0.0182 0.00421 0.000292 0.000479 0.00068 0.0141 0.0207 0.00324
2R:12907880-12908030:plus -14.2959 6.75229 4.79817 7.43119 -19.3163 -19.0816 -14.8899
1.44444 -2.11009 0.00129 0.000736 0.00193 0.000939 0.0165 0.017 0.00877 0.0105 0.0343
2R:12914260-12914410:minus -41.398 -3.93578 0.899083 1.69725 -18.1837 -36.2245 3.85321
3.81944 7.22018 0.0554 0.0333 0.00856 0.0163 0.00835 0.0923 0.0000871 0.00433 0.00119
2R:12914260-12914410:plus -24.5918 3.72477 4.19266 3.52294 -18.4184 -18.051 1.49541 7.77778
1.48624 0.00821 0.00266 0.00248 0.00706 0.0101 0.00886 0.000192 0.000661 0.0118
2R:12914600-12914750:minus -33.2959 -1.82569 4.74312 6.01835 -18.7551 -10.0204 -20.1651
1.93056 -1.37615 0.0279 0.0185 0.00198 0.00248 0.0144 0.00399 0.0212 0.00883 0.0287
2R:12914600-12914750:plus -31.4694 -3.44954 0.522936 6.12844 -18.6429 -18.9388
-2.30275 1.51389 -5.01835 0.0124 0.0292 0.00974 0.00231 0.0121 0.0154 0.000477 0.0102 0.073

2R:12915080-12915230:minus -30.7755 3.21101 2.3945 7.20183 -27 -17.051 -22.2385 3.26389 2.10092 0.0102 0.00322 0.00499 0.00112 0.0233 0.00479 0.0308 0.00539 0.00901
2R:12915080-12915230:plus -12.6633 5.05505 1.69725 0.577982 -8.64286 -9.08163 -4.94495 2.29167 3.52294 0.000458 0.00159 0.00646 0.0231 0.00139 0.00216 0.000977 0.00776 0.00558
2R:12915820-12915970:minus -13.2245 0.449541 9.3211 7.21101 -18.7245 -9.20408 -23.4037 1.54167 1.33028 0.000738 0.00882 0.00021 0.0011 0.0136 0.00225 0.0375 0.0101 0.0127
2R:12915820-12915970:plus -23.2959 -1.3211 2.46789 9.08257 -26.8571 -18.898 -14.5321 6.95833 -0.853211 0.00605 0.0159 0.00486 0.000444 0.0198 0.0152 0.00814 0.00103 0.0251
2R:12930900-12931050:minus -14.449 3.19266 3.53211 7.51376 -18.7143 -17.2755 -13.6514 2.84722 3.29358 0.0014 0.00324 0.00323 0.000836 0.0136 0.00544 0.00671 0.00632 0.00623
2R:12930900-12931050:plus -24.1837 2.61468 -1.27523 1.88991 -27.2653 -27.3367 -8.07339 1.06944 10.3486 0.00751 0.004 0.0174 0.0151 0.0302 0.0347 0.00214 0.0119 0.0000988
2R:12936380-12936530:minus -32.7041 0.00917431 14.8624 8.81651 -17.602 -17.0102 -23.4862 1.75 -6.94495 0.0222 0.0103 2.2e-06 0.000501 0.00547 0.00453 0.038 0.00941 0.112
2R:12936380-12936530:plus -14.8571 0.889908 4.77982 10.7615 -27.602 -19.051 -16.5413 6.93056 3.20183 0.0016 0.00753 0.00195 0.0000826 0.0364 0.0165 0.0119 0.00104 0.0065
2R:12941420-12941570:minus -22.6633 2.59633 0.33945 2.19266 -8.37755 -17.7857 -18.6972 -0.902778 1.65138 0.00384 0.00403 0.0104 0.0131 0.00106 0.0068 0.0166 0.0219 0.011
2R:12941420-12941570:plus -22.9592 0.366972 3.68807 7.77982 -8.33673 -7.72449 -0.944954 1.48611 2.0367 0.00475 0.00908 0.00304 0.000652 0.00101 0.00068 0.000351 0.0103 0.00923
2R:12944220-12944370:minus -30.4796 1.14679 -0.954128 6.84404 -17.3061 -0.244898 -21.1101 5.47222 -0.40367 0.00941 0.00686 0.0157 0.0014 0.00389 0.000496 0.0251 0.00212 0.0213
2R:12944220-12944370:plus -29.9898 -0.321101 10.5321 5.75229 -7.67347 -26.7143 -11.0459 -3.11111 2.20183 0.00856 0.0115 0.000103 0.00296 0.00055 0.0257 0.00377 0.0397 0.00869
2R:12950120-12950270:minus -31.7755 7.2844 2.50459 5.43119 -19.051 -26.6735 -19.8532 5.68056 3.66972 0.0157 0.000559 0.0048 0.00371 0.0162 0.0256 0.0201 0.00193 0.00514
2R:12950120-12950270:plus -32.4796 11.5688 -2.24771 3.76147 -18.4592 -18.6429 -6.24771 6.43056 1.69725 0.02 0.0000591 0.0232 0.00645 0.0109 0.0139 0.0014 0.00134 0.0108
2R:12979160-12979310:minus -14.0408 0.788991 3.21101 8.81651 -17.8878 -0.173469 -4.54128 0.222222 2.11009 0.00115 0.00781 0.00366 0.000501 0.00704 0.000456 0.000871 0.0156 0.00898
2R:12979160-12979310:plus -23.5204 7.50459 14.6881 3.43119 -27.4898 -7.72449 -21.2569 4.66667 0.229358 0.00645 0.000498 3.06e-06 0.00749 0.0336 0.00068 0.0258 0.00304 0.0173
2R:12990280-12990430:minus -13.5612 -4.50459 3.18349 6.00917 -27.2653 -6.72449 -18.4128 -3.04167 -5.17431 0.000984 0.0387 0.0037 0.00255 0.0302 0.000544 0.0159 0.0391 0.0755
2R:12990280-12990430:plus -22.4694 -3.54128 0.788991 9.24771 -27.9694 -36.9592 0.706422 -1.38889 5.22936 0.0035 0.0299 0.00889 0.000409 0.0437 0.124 0.000238 0.0252 0.00288
2R:12992840-12992990:minus -22.8878 3.25688 -1 10.1468 -17.8571 -18.2041 -13.8807 -5.80556 3.88991 0.00441 0.00316 0.016 0.000128 0.00698 0.0103 0.00706 0.0731 0.00467
2R:12992840-12992990:plus -21.7755 -1.78899 3.13761 6.68807 -18.1531 -16.8265 -19.4128 -2.90278 2.75229 0.00241 0.0183 0.00377 0.00157 0.00829 0.0043 0.0187 0.0377 0.00748
2R:12996300-12996450:minus -24.5204 0.165138 0.486239 2 -7.89796 -18.1224 -6.55963 0.513889 -4.76147 0.00813 0.00974 0.00986 0.0143 0.000678 0.00988 0.00152 0.0142 0.0686
2R:12996300-12996450:plus -13.2653 2.45872 1.55963 11.1927 -9.20408 -9.45918 -11.3394 -3.16667 -0.889908 0.000759 0.00424 0.00678 0.0000254 0.00262 0.00279 0.004 0.0403 0.0253
2R:12997420-12997570:minus -14.5612 0.779817 0.862385 4.27523 -27.2653 -7.23469 -10.5321 -2.09722 4.34862 0.00147 0.00784 0.00867 0.00518 0.0302 0.000557 0.0034 0.0306 0.00384
2R:12997420-12997570:plus -39.949 -0.633028 4.82569 2.34862 -7.71429 -18.1224 -10.4679 -3.90278 1.6422 0.0345 0.0127 0.00191 0.0121 0.000572 0.00988 0.00336 0.0481 0.0111
2R:13021440-13021590:minus -30.4388 0.155963 2.3578 9.59633 -17.1531 -26.4898 -16.8165 -2.69444 5.79817 0.00923 0.00977 0.00506 0.000275 0.00364 0.0225 0.0124 0.0358 0.00208
2R:13021440-13021590:plus -24.4898 5.62385 0.917431 9.92661 -27.7449 -26.8265 -18.9908 1.08333 10.3394 0.00807 0.00125 0.00851 0.000169 0.0383 0.0285 0.0174 0.0118 0.000112
2R:13023800-13023950:minus -23.1939 2.2844 3.3945 5.6789 -17.4898 -18.2755 -10.4037

2.80556 2.11009 0.00558 0.00452 0.00341 0.00315 0.0053 0.0112 0.00332 0.00642 0.00898
2R:13023800-13023950:plus -22.7347 3.98165 9.25688 9.41284 -18.1939 -18.7857 -11.7706
5.40278 1.72477 0.00403 0.00242 0.000218 0.000354 0.0086 0.0146 0.00439 0.00219 0.0106
2R:13024660-13024810:minus -32.8163 -1.74312 7.82569 4.17431 -27.0102 -27.6735 -17.3853
1.72222 1.6422 0.024 0.018 0.00048 0.00537 0.0237 0.0443 0.0136 0.0095 0.0111
2R:13024660-13024810:plus -21.6939 2.04587 2.41284 9.92661 -8.86735 -7.45918 -11.6606
0.263889 4.09174 0.00226 0.00494 0.00496 0.000169 0.00173 0.000582 0.00428 0.0154 0.00416
2R:13027440-13027590:minus -30.551 -0.678899 2.50459 7.10092 -18.1531 -27.6327 2.34862 8.70833
1.70642 0.00959 0.0129 0.0048 0.00121 0.00829 0.043 0.000147 0.000384 0.0108
2R:13027440-13027590:plus -23.1837 0.394495 2.3578 7.46789 -17.4898 -18.1531 -25.1193
5.40278 1.2844 0.00553 0.00899 0.00506 0.000868 0.0053 0.00997 0.0479 0.00219 0.0129
2R:1304360-1304510:minus -21.8469 -0.669725 2.00917 6.01835 -17.1122 -8.5 -15.4037
0.0277778 3.95413 0.00247 0.0129 0.00576 0.00248 0.00341 0.00126 0.00972 0.0166 0.00448
2R:1304360-1304510:plus -33.3367 3.64286 2.63303 0.990826 0.316327 -16.051 -24.9817 8.05556
-4.43119 0.0281 0.00159 0.00457 0.0206 0.000341 0.00408 0.047 0.000565 0.0627
2R:130440-130590:minus -33.0408 0.522936 4.19266 1.05505 -8.96939 -0.316327 -12.3945
8.27778 -0.238532 0.0264 0.00859 0.00248 0.0204 0.00226 0.000524 0.00504 0.000497 0.0203
2R:130440-130590:plus -13.449 7.82569 0.981651 3.6055 10.6939 -9.7551 -13.8349 2.80556 2.83486
0.000914 0.00042 0.00832 0.00686 7.35e-06 0.00358 0.00699 0.00642 0.00739
2R:1305200-1305350:minus -32.6735 -6.70642 2.43119 3.43119 -17.449 -8.5 -23.9083 3.88889
-2.15596 0.0216 0.068 0.00493 0.00749 0.00484 0.00126 0.0405 0.00421 0.0348
2R:1305200-1305350:plus -31.449 0.220183 2.97248 -1.16514 -18.6837 0.122449 -1.12844
4.68056 5.69725 0.0123 0.00956 0.00401 0.0386 0.0126 0.000312 0.000365 0.00302 0.00225
2R:1308200-1308350:minus -32.7347 1.04587 0.59633 5.06422 -18.051 -9.79592 -15.6514 3.125
-0.541284 0.0225 0.00712 0.00949 0.00433 0.00746 0.00377 0.0102 0.00569 0.0223
2R:1308200-1308350:plus -13.7449 0.458716 0.963303 1.3578 -19.0102 -18.0816 -13.0092
6.94444 8.11009 0.00107 0.00879 0.00837 0.0186 0.0157 0.00934 0.00579 0.00103 0.000597
2R:13149360-13149510:minus -29.4796 -1.33028 1.22018 1.29358 -7.82653 -8.86735 -15.6422
0.736111 2.99083 0.00844 0.0159 0.00765 0.019 0.00059 0.00198 0.0102 0.0132 0.00706
2R:13149360-13149510:plus -23.2245 1.72477 2.09174 -1.3578 -17.4184 -0.173469 -22.4954
-1.16667 -2.11009 0.00576 0.00555 0.00558 0.0415 0.00457 0.000456 0.0322 0.0237 0.0343
2R:13152880-13153030:minus -39.4082 -0.165138 2.55963 6.01835 -18.9796 0.755102 -29.6239
9.59722 -0.0275229 0.0332 0.0109 0.0047 0.00248 0.0155 0.000175 0.0862 0.000217 0.0186
2R:13152880-13153030:plus -3.40816 8.06422 1.16514 9.59633 -27.4592 -9.53061 7.50459
-0.0972222 2.58716 0.0000685 0.000369 0.0078 0.000275 0.0329 0.0031 0.0000271 0.0172
0.00779
2R:13290980-13291130:minus -32.0714 1.66972 -1.18349 2.70642 -18.2653 -16.9796 -11.6055
5.09722 -2.3945 0.0176 0.00567 0.0169 0.0103 0.00882 0.0044 0.00423 0.00251 0.0375
2R:13290980-13291130:plus -22.2245 5.90826 3.04587 7.3578 -17.9796 -18.3469 -14.2752
10.0139 7.68807 0.00325 0.0011 0.0039 0.000965 0.00726 0.0124 0.00771 0.000162 0.000841
2R:13294560-13294710:minus -24.5612 -0.788991 13.9174 3.43119 -17.7143 -9.79592 -12.5046
-2.11111 -2.17431 0.00819 0.0134 8.08e-06 0.00749 0.00627 0.00377 0.00516 0.0307 0.035
2R:13294560-13294710:plus -40.6224 -1.63303 13.7798 5.01835 -18.1939 -8.79592 -15.6147
1.33333 2.48624 0.0424 0.0174 9.84e-06 0.00438 0.0086 0.0019 0.0101 0.0109 0.00795
2R:13300180-13300330:minus -32.5918 -0.550459 1.76147 4.02752 -27.9694 -0.27551 -26.9358
-0.666667 -2.18349 0.021 0.0124 0.00631 0.00581 0.0437 0.000502 0.06 0.0205 0.0351
2R:13300180-13300330:plus -33.5918 14.8716 4.44954 5.42202 -8.93878 -9.23469 -21.7523
5.47222 3.24771 0.0289 2.8e-06 0.00224 0.00374 0.00216 0.00238 0.0282 0.00212 0.00636
2R:13307100-13307250:minus -21.8878 3.33028 4.14679 2.47706 1.17347 -9.45918 -11.7156 6.94444
7.68807 0.00258 0.00308 0.00252 0.0115 0.000101 0.00279 0.00433 0.00103 0.000841
2R:13307100-13307250:plus -31.3673 15.5963 1.82569 1.78899 -26.5306 -7.72449 -17.6789 1.06944 8.41284
0.0116 8.91e-07 0.00616 0.0158 0.0185 0.00068 0.0142 0.0119 0.000519
2R:13307700-13307970:minus -32.3265 -2.50459 9.86239 7.2844 -18.7551 -17.3469 -15.8716 1.48611
7.15596 0.0187 0.0225 0.000155 0.00105 0.0144 0.0058 0.0106 0.0103 0.00124

2R:13307700-13307970:plus -14.6633 -5.45872 2.33028 9.24771 -26.9286 -26.5612 -2.04587 -1.41667 11.8991
0.0015 0.0497 0.00511 0.000409 0.0214 0.0237 0.000449 0.0254 0.0000255
2R:13308220-13308370:minus -23.4898 -5.75229 5 2.9633 -8.12245 -17.051 -26.0917 5.25 -4.52294
0.00639 0.0536 0.00178 0.00914 0.000843 0.00479 0.0541 0.00235 0.0642
2R:13308220-13308370:plus -22.2653 8.57798 2.3578 6.00917 -18.1939 -9.53061 -1.6422 6.43056 10.3486
0.00331 0.00028 0.00506 0.00255 0.0086 0.0031 0.000409 0.00134 0.0000988
2R:13314960-13315110:minus -31.3673 -2.7156 9.93578 -0.633028 -27.3776 -18.8571 -15.7615 2.25
4.83486 0.0116 0.0238 0.000148 0.0327 0.032 0.0151 0.0104 0.00788 0.00329
2R:13314960-13315110:plus -23 2.70642 4.84404 4.04587 -7.89796 -19.1224 -15.3486 4 2.6422 0.00498
0.00387 0.0019 0.00572 0.000678 0.0175 0.00961 0.00402 0.00768
2R:13315340-13315490:minus -24.4082 5.63303 1.09174 9.44954 -27.4592 -8.09184 -21.4679 3.15278
0.908257 0.00797 0.00125 0.00801 0.000326 0.0329 0.000762 0.0268 0.00563 0.0147
2R:13315340-13315490:plus -23.2551 1.11009 1.27523 7.46789 -8.67347 -26.6327 -20.6422 -4.61111 1.34862
0.00583 0.00695 0.0075 0.000868 0.00154 0.0251 0.0231 0.0566 0.0125
2R:13318460-13318610:minus -32.4694 13.4587 -1.27523 5.7156 -19.0102 -17.898 -10.4862 0.347222
2.54128 0.0196 0.0000146 0.0174 0.00309 0.0157 0.00726 0.00337 0.015 0.00787
2R:13318460-13318610:plus -13.6327 14.3945 1.52294 3.77064 -19.0102 -17.9796 -11.0917 2.76389 -5.11927
0.00101 5.64e-06 0.00687 0.00637 0.0157 0.00764 0.0038 0.00652 0.0746
2R:13332380-13332530:minus -21.6327 9.76147 5.77982 2.9633 -27.1224 -8.65306 -26.3761 0.888889
-2.90826 0.00215 0.00015 0.00127 0.00914 0.0245 0.00156 0.056 0.0126 0.0435
2R:13332380-13332530:plus -41.102 -1.84404 4.85321 -1.66972 -18.7143 -8.87755 -27.1101 2.55556 -4.13761
0.0472 0.0186 0.00189 0.0458 0.0136 0.00198 0.0613 0.00705 0.0575
2R:13338580-13338730:minus -32.8571 -1.12844 2.74312 2.68807 -26.8571 -16.7551 -22.1284 6.875
-0.504587 0.0244 0.0149 0.00438 0.0104 0.0198 0.0042 0.0302 0.00107 0.0221
2R:13338580-13338730:plus -42.5816 -1.68807 -4.33028 2.04587 -18.6735 -8.65306 -28.6514 6.125 -7.84404
0.0807 0.0177 0.0415 0.014 0.0123 0.00156 0.0752 0.00156 0.131
2R:13339680-13339830:minus -32.8163 0.706422 5.80734 2.6422 -27.2245 -7.27551 -19.2385 4.47222
5.3945 0.024 0.00805 0.00126 0.0107 0.0272 0.000561 0.0181 0.0033 0.00263
2R:13339680-13339830:plus -33.8061 0.146789 -1.43119 0.321101 -18.7449 0.530612 -27.8624 3 2.85321
0.0298 0.0098 0.0182 0.0247 0.0138 0.000203 0.0676 0.00597 0.00733
2R:13343880-13344030:minus -33.602 -2.16514 0.761468 3.47706 -27.2347 -18.2347 -16.3211 3.23611
0.330275 0.0289 0.0204 0.00897 0.00724 0.0283 0.0104 0.0115 0.00545 0.0168
2R:13343880-13344030:plus -31.3571 -0.889908 2.98165 0.917431 -18.4184 -10.0204 -18.6514 -2.83333
-2.54128 0.0114 0.0138 0.004 0.021 0.0101 0.00399 0.0165 0.0371 0.0393
2R:13344840-13344990:minus -13.5918 8.26606 0.00917431 0.275229 -17.8265 -19.0102 -13.9908 9.33333
6.17431 0.00099 0.00033 0.0116 0.025 0.00691 0.0159 0.00724 0.000258 0.00171
2R:13344840-13344990:plus -23.9286 12.9174 -1.47706 1.89908 20.4694 -27.4898 -10.5963 -2.09722 1.74312
0.00701 0.0000238 0.0185 0.0151 1.65e-07 0.0363 0.00345 0.0306 0.0105
2R:13345100-13345250:minus -32.7347 -2.49541 -0.93578 1.63303 -18.7143 -9.5 -8.87156 5.36111 -0.908257
0.0225 0.0224 0.0156 0.0167 0.0136 0.00294 0.0025 0.00223 0.0255
2R:13345100-13345250:plus -23.9286 12.9174 -1.47706 1.89908 20.4694 -27.4898 -10.5963 -2.09722 1.74312
0.00701 0.0000238 0.0185 0.0151 1.65e-07 0.0363 0.00345 0.0306 0.0105
2R:13345800-13345950:minus -32.7041 -2.30275 -3.11009 -1.47706 -27.2653 -16.051 -11.7156 5.16667
3.40367 0.0222 0.0212 0.0297 0.0429 0.0302 0.00408 0.00433 0.00244 0.00603
2R:13345800-13345950:plus -20.8571 -0.844037 -0.633028 8.05505 -17.8571 0.602041 -8.59633 -0.944444
3.40367 0.00178 0.0136 0.0142 0.000578 0.00698 0.000188 0.00237 0.0222 0.00603
2R:13347140-13347290:minus -33.449 -0.321101 5.3211 -3.36697 -9.27551 -26.8265 -17.9633 1.91667
0.963303 0.0284 0.0115 0.00155 0.0741 0.00284 0.0285 0.0149 0.00888 0.0145
2R:13347140-13347290:plus -40.2143 -0.266055 5.56881 6.02752 -27.1531 -18.2347 -17.8807 2.36111
2.11009 0.0372 0.0113 0.0014 0.00246 0.0249 0.0104 0.0147 0.00757 0.00898
2R:13349140-13349290:minus -31.7041 -0.770642 2.78899 6.29358 -26 9.86735 -17.2385 1.65278
4.56881 0.0146 0.0133 0.0043 0.00202 0.0171 0.0000132 0.0133 0.00974 0.00356
2R:13349140-13349290:plus -34.0816 0.40367 10.3486 1.55046 -18.7143 -18.051 3.81651 0.763889 5.33028
0.0311 0.00896 0.000115 0.0173 0.0136 0.00886 0.0000882 0.0131 0.00273

2R:13349740-13349890:minus	-12.7347	1.58716	-0.486239	3.75229	-27.1633	-8.53061	2.33945	-2.94444
-0.0183486	0.000468	0.00584	0.0136	0.00649	0.025	0.00136	0.000148	0.0381	0.0185
2R:13349740-13349890:plus	-14.2959	1.22936	3.3945	6.56881	-17.1531	-18.7857	-6.43119	2.43056	12.2752
0.00129	0.00666	0.00341	0.0017	0.00364	0.0146	0.00147	0.00738	0.0000141
2R:13356620-13356770:minus	-21.9898	13.4592	-1.13761	7.61468	-26.898	19.051	-7.77064	4.70833
7.6422	0.00282	0.000064	0.0167	0.000779	0.0203	1.23e-06	0.00201	0.00298	0.000893
2R:13356620-13356770:plus	-22	13.7706	1.92661	5.51376	-27.3469	-16.7551	-19.8899	2.02778	1.33028
0.00291	0.0000111	0.00594	0.00348	0.0318	0.0042	0.0202	0.00853	0.0127
2R:13357020-13357170:minus	-41.9286	-1.17431	-2.99083	2.66972	-26.9694	-0.0204082	-22.9633	4.91667
5.53211	0.0676	0.0151	0.0288	0.0106	0.023	0.000411	0.0349	0.00272	0.00245
2R:13357020-13357170:plus	-31.6633	5.31193	6.85321	7.27523	11.6939	-8.42857	-11.5229	-1.88889	-4.19266
0.0141	0.00143	0.000779	0.00109	1.17e-06	0.00104	0.00416	0.0289	0.0583
2R:13357520-13357670:minus	-24.4082	-1.63303	0.577982	1.57798	-18.5204	-19.051	-4.57798	-1.63889
5.38532	0.00797	0.0174	0.00955	0.0171	0.0117	0.0165	0.00088	0.027	0.00267
2R:13357520-13357670:plus	-13.1531	3.64286	-1.58716	-1.61468	-18.7959	-18.898	-19.0917	-1.61111	3.51376
0.00067	0.00159	0.0191	0.0449	0.0148	0.0152	0.0177	0.0268	0.00566
2R:13401660-13401810:minus	-32.1939	-1.94495	-0.633028	1.82569	-9.23469	-8.79592	-17.6972	1.98611
-3.73394	0.0181	0.0191	0.0142	0.0156	0.00277	0.0019	0.0143	0.00866	0.052
2R:13401660-13401810:plus	-31.7755	13.5306	4.72477	6.89908	-8.57143	-17.8265	-21.2385	-1.5	-0.12844
0.0157	0.0000206	0.00199	0.00134	0.00113	0.00699	0.0257	0.026	0.0193
2R:13405700-13405850:minus	-33.1429	4.20183	14.8532	1.36697	-27	-8.53061	-14.7156	8.81944	1.73394
0.0272	0.00223	2.28e-06	0.0186	0.0233	0.00136	0.00847	0.000359	0.0106
2R:13405700-13405850:plus	-30.4796	0.66055	2.42202	1.74312	20.2041	-18.1224	-14.6055	7.68056	-0.238532
0.00941	0.00818	0.00495	0.016	3.64e-07	0.00988	0.00827	0.000698	0.0203
2R:13421480-13421630:minus	-21.9286	5.93578	0.0550459	2.54128	-26.898	-27.602	-17.8257	5.72222
-2.30275	0.0027	0.00109	0.0114	0.0112	0.0203	0.0416	0.0146	0.00189	0.0363
2R:13421480-13421630:plus	-12.3367	1.53211	4.99083	6.47706	-27.1939	-7.5	-14.7798	1.08333	-2.22936
0.000393	0.00596	0.00178	0.00179	0.0267	0.000614	0.00858	0.0118	0.0356
2R:13423060-13423210:minus	-21.4388	-1.72477	5.58716	4.08257	-17.7449	-37.449	-2.11009	-8.47222
7.31193	0.002	0.0179	0.00139	0.00558	0.0063	0.164	0.000456	0.12	0.00112
2R:13423060-13423210:plus	-12.2551	-1.86239	8.15596	-3.34862	-17.3776	-19.051	-12.5596	1.79167	3.61468
0.000379	0.0187	0.000403	0.0738	0.00419	0.0165	0.00523	0.00928	0.00526
2R:13423820-13423970:minus	-31.3265	0.275229	2.27523	5.31193	-9.2449	-8.72449	-22.4587	2.65278
0.394495	0.0114	0.00938	0.00522	0.00393	0.00278	0.00169	0.032	0.0068	0.0166
2R:13423820-13423970:plus	-13.2653	-2.08257	0.146789	3.22936	0.540816	-18.1224	-14.2385	-0.347222
-1.38532	0.000759	0.0199	0.011	0.00816	0.000321	0.00988	0.00765	0.0186	0.0288
2R:13432620-13432770:minus	-23.2551	5.55046	0.587156	-0.183486	-17.7857	-18.5714	-20.3486	2.22222
-0.440367	0.00583	0.00129	0.00952	0.0283	0.00676	0.0136	0.0219	0.00796	0.0216
2R:13432620-13432770:plus	-24.8571	13.4592	-2.49541	5.57798	-19.051	-8.42857	-12.1927	7.875	-3.62385
0.00831	0.000064	0.025	0.0034	0.0162	0.00104	0.00481	0.000626	0.051
2R:13434580-13434730:minus	-31.3265	0.651376	3.41284	7.47706	-35.5204	-36.3776	-16.3028	-3.66667
3.92661	0.0114	0.00821	0.00338	0.00085	0.0664	0.104	0.0114	0.0455	0.00461
2R:13434580-13434730:plus	-21.1531	-2.07339	5.90826	7.21101	-18.2245	-9.20408	-14.9817	0.847222	-0.972477
0.00188	0.0198	0.0012	0.0011	0.00877	0.00225	0.00894	0.0128	0.0259
2R:13435160-13435310:minus	-23.2245	16.4954	2.31193	5.66055	-17.4898	-8.45918	-16.3578	-4.375
1.44037	0.00576	7.12e-08	0.00515	0.00317	0.0053	0.00111	0.0115	0.0537	0.012
2R:13435160-13435310:plus	-23.9184	6.10092	2.83486	1.6422	0.846939	-26.8571	-15.7156	5.83333	2.33028
0.00695	0.00101	0.00423	0.0166	0.000197	0.0288	0.0103	0.00179	0.00836
2R:13435780-13435930:minus	-32.3367	5.69725	3.21101	0.366972	-18.1531	-27.5612	-15.3119	-0.722222
11.4771	0.0187	0.00121	0.00366	0.0244	0.00829	0.0393	0.00954	0.0208	0.0000679
2R:13435780-13435930:plus	-42.5816	-0.990826	-0.53211	-0.00917431	-17.3367	-17.898	-22.9817	4.375
0.917431	0.0807	0.0143	0.0138	0.027	0.00391	0.00726	0.035	0.00344	0.0146
2R:13452300-13452450:minus	-30.449	-1.14679	5.48624	5.42202	-27.4082	-8.45918	-13.5596	2.66667
2.75229	0.00925	0.015	0.00145	0.00374	0.0321	0.00111	0.00657	0.00677	0.00748

2R:13452300-13452450:plus -32.8163 -1.36697 1.34862 4.07339 -8.89796 -9.16327 -13.0826 4.41667 7.22018
0.024 0.0161 0.00731 0.00562 0.00189 0.00223 0.00589 0.00338 0.00119
2R:13455880-13456030:minus -13.449 -0.798165 0.623853 1.33028 -18.0816 -7.65306 -13.8073 12
0.495413 0.000914 0.0134 0.00941 0.0188 0.00759 0.000647 0.00695 0.0000309 0.0162
2R:13455880-13456030:plus -32.6633 8.41284 5.11927 5.46789 10.3571 -9.60204 -8.51376 0.152778 -2.19266
0.0216 0.000305 0.00169 0.00362 0.0000208 0.00334 0.00233 0.016 0.0352
2R:13456340-13456490:minus -41.6633 -1.40367 2.42202 2.16514 -8.67347 -17.4184 -16.2477 9.20833
3.29358 0.0636 0.0163 0.00495 0.0132 0.00154 0.00585 0.0113 0.00028 0.00623
2R:13456340-13456490:plus -34.0714 -1.9633 -1.78899 1.33028 -18.7143 -15.9796 -18.1835 1.13889 0.550459
0.031 0.0192 0.0203 0.0188 0.0136 0.00405 0.0154 0.0116 0.0161
2R:13460240-13460390:minus -21.1837 -0.486239 0.266055 -0.0550459 -27.5 -0.316327 -19.0459
3.01389 -2.98165 0.00189 0.0121 0.0106 0.0272 0.0337 0.000524 0.0176 0.00593 0.0445
2R:13460240-13460390:plus -33.1837 1.59633 1.79817 0.100917 -8.93878 -28.3367 -21.6147 9.59722 5.40367
0.0274 0.00582 0.00623 0.0261 0.00216 0.0606 0.0275 0.000217 0.0026
2R:13460840-13460990:minus -23.1837 -0.889908 4.44037 0.12844 -18.2245 -24.8265 -10.055 10.9583
1.23853 0.00553 0.0138 0.00224 0.0259 0.00877 0.0204 0.00311 0.000079 0.0132
2R:13460840-13460990:plus -31.7755 -2.98165 -1.37615 5.65138 -8.56122 9.82653 -17.4954 -1.41667 2.58716
0.0157 0.0257 0.0179 0.00322 0.00112 0.0000148 0.0138 0.0254 0.00779
2R:13468460-13468610:minus -23.1531 0.0642202 0.394495 -0.917431 -17.8571 -9.20408 -15.9633
2.02778 4.19266 0.00542 0.0101 0.0102 0.0358 0.00698 0.00225 0.0108 0.00853 0.00401
2R:13468460-13468610:plus -14.1122 2.0367 3.55963 1.99083 -26.9694 -18.5612 -15.1651 9.44444 -0.651376
0.00119 0.00495 0.0032 0.0144 0.023 0.0132 0.00927 0.00024 0.0235
2R:13468920-13469070:minus -40.6531 2.13761 3.85321 1.84404 1.17347 -18.1633 -16.2844 7.61111
3.56881 0.0425 0.00477 0.00284 0.0154 0.000101 0.01 0.0114 0.000726 0.00543
2R:13468920-13469070:plus -12.8878 -5.36697 2.95413 3.26606 19.9082 -18.3469 -4.25688 4.06944 4.34862
0.000523 0.0486 0.00404 0.00802 8.13e-07 0.0124 0.000802 0.00391 0.00384
2R:13473640-13473790:minus -33.0408 1.11009 14.9633 7.29358 -7.71429 0.0510204 -10.6514 -1.68056
0.0183486 0.0264 0.00695 1.77e-06 0.00104 0.000572 0.000349 0.00348 0.0273 0.0183
2R:13473640-13473790:plus -32.6633 -1.11927 9.53211 2.66055 -18.1531 -18.5714 -19.0367 3.52778 -0.954128
0.0216 0.0149 0.000187 0.0106 0.00829 0.0136 0.0176 0.00486 0.0258
2R:13478180-13478330:minus -13.2245 2.23853 2.45872 6.00917 20.4694 -18.0102 -13.2477 -2.77778
2.12844 0.000738 0.0046 0.00488 0.00255 1.65e-07 0.00812 0.00612 0.0365 0.00886
2R:13478180-13478330:plus -31.6633 -0.256881 -0.110092 2.19266 -17.3061 -9.72449 -18.4771 3.56944
5.84404 0.0141 0.0113 0.012 0.0131 0.00389 0.00352 0.0161 0.00478 0.00202
2R:13480140-13480290:minus -12.3776 8.59633 0.0183486 7.3578 -19.0102 -0.244898 -20.3303
1.19444 -6.90826 0.000403 0.000277 0.0115 0.000965 0.0157 0.000496 0.0218 0.0114 0.111
2R:13480140-13480290:plus -31.7041 13.4592 -1.77982 4.33028 -18.3776 -8.57143 -19.0275 5.72222 8.20183
0.0146 0.000064 0.0202 0.00511 0.00926 0.00149 0.0175 0.00189 0.000539
2R:13480540-13480690:minus -21.8469 12.2385 -4.01835 3.75229 -7.96939 -18.0102 -18.9266 0.0138889
-0.155963 0.00247 0.0000393 0.0382 0.00649 0.000785 0.00812 0.0172 0.0167 0.0195
2R:13480540-13480690:plus -32.6224 -1.46789 -2.97248 5.06422 -17.0408 -19.3469 -3.78899 0.597222 12.2752
0.0211 0.0166 0.0286 0.00433 0.00329 0.0194 0.000703 0.0139 0.0000141
2R:13486120-13486270:minus -23.3776 2.93578 0.440367 3.30275 -18.1531 -26.8265 -14 0.694444 0.908257
0.00619 0.00356 0.01 0.00789 0.00829 0.0285 0.00725 0.0134 0.0147
2R:13486120-13486270:plus -22.8571 0.394495 3.51376 7.27523 -17.9388 -17.5612 -20.9083 -1.22222 -0.862385
0.00427 0.00899 0.00325 0.00109 0.00715 0.00604 0.0242 0.024 0.0251
2R:13553060-13553210:minus -22.0714 13.5229 -2.47706 0.522936 -17.1939 -9.45918 -7.90826 1.97222
7.36697 0.00299 0.000014 0.0248 0.0234 0.00377 0.00279 0.00206 0.0087 0.00108
2R:13553060-13553210:plus -31.9286 3.86239 -1.48624 3.88991 -17.3061 -8.38776 -16.7615 8.34722 -4.22018
0.0166 0.00253 0.0185 0.00608 0.00389 0.00101 0.0123 0.000478 0.0586
2R:13557040-13557190:minus -31.8163 3.80734 -0.53211 4.0367 -17.1531 -26.7551 -13.1193 0.611111
4.36697 0.016 0.00259 0.0138 0.00578 0.00364 0.0263 0.00594 0.0138 0.00379
2R:13557040-13557190:plus -14.5204 -0.862385 6.86239 1.92661 -18.7143 -27.9694 -6.20183 4.48611
5.25688 0.00145 0.0137 0.000775 0.0148 0.0136 0.0547 0.00138 0.00328 0.0028

2R:13566040-13566190:minus -31.8469 9.18349 3.6422 7.6422 -18.4898 -18.7857 -17.2294 5.80556
7.73394 0.0162 0.000203 0.00309 0.000721 0.0116 0.0146 0.0133 0.00182 0.000784
2R:13566040-13566190:plus -32.9592 1.99083 5.55963 4.31193 -17.4898 -18.0816 -22.0917 4.625 -2.44037
0.0252 0.00504 0.0014 0.00516 0.0053 0.00934 0.03 0.00309 0.0381
2R:13569780-13569930:minus -31.4388 -4.10092 1.9633 7.15596 -9.19388 -19.1531 -15.0092 4.41667
-0.513761 0.0122 0.0348 0.00586 0.00117 0.00253 0.0177 0.00899 0.00338 0.0221
2R:13569780-13569930:plus -30.4796 0.550459 2.53211 5.77982 -27.4592 -8.61224 -19.5963 6.86111 7.73394
0.00941 0.00851 0.00475 0.00294 0.0329 0.00152 0.0192 0.00108 0.000784
2R:13570680-13570830:minus -23.7041 -3.88991 3.05505 -0.798165 -17.1531 -27.7857 -8.52294 10.125
5.76147 0.00669 0.0328 0.00389 0.0344 0.00364 0.0466 0.00234 0.00015 0.0021
2R:13570680-13570830:plus -32.449 0.0183486 -4.65138 -0.376147 -8.40816 -26.5306 -25.0642 1.15278
5.9633 0.0195 0.0102 0.0451 0.0299 0.00108 0.023 0.0476 0.0115 0.00188
2R:13571020-13571170:minus -22.9694 2.68807 0.440367 4.37615 -7.60204 19.3469 -23.5046 6.70833
0.807339 0.0048 0.0039 0.01 0.00504 0.000466 5.04e-07 0.0381 0.00117 0.015
2R:13571020-13571170:plus -21.8469 -0.844037 5.38532 2.12844 0.316327 -9.16327 -24.8257 -2.97222
1.02752 0.00247 0.0136 0.00151 0.0134 0.000341 0.00223 0.0461 0.0384 0.0141
2R:13585940-13586090:minus -4 -3.12844 12.6055 -0.33945 10.6531 -15.9796 -19.8716 8.95833 -4.24771
0.000121 0.0267 0.000028 0.0296 0.0000102 0.00405 0.0201 0.000329 0.0591
2R:13585940-13586090:plus -31.7347 3.90826 5.68807 1.40367 -27.5306 -18.1939 -21.2569 3.25 -0.0733945
0.0149 0.00249 0.00133 0.0184 0.0351 0.0101 0.0258 0.00542 0.0188
2R:13586520-13586670:minus -23.2653 13.3878 -5.68807 5.06422 -18.7143 -18.0102 -8.46789 1.27778
3.57798 0.00593 0.0000869 0.0587 0.00433 0.0136 0.00812 0.00231 0.0111 0.00536
2R:13586520-13586670:plus -22.0714 3.97959 -1.3945 2.88991 1.10204 -18.7143 -1.66972 4.36111 -1.88991
0.00299 0.000669 0.018 0.00942 0.000124 0.014 0.000412 0.00346 0.0322
2R:13586940-13587090:minus -22.7041 2.20183 6.25688 1.33028 -27.2653 -8.57143 -21.3028 7.25 3
0.00395 0.00466 0.00103 0.0188 0.0302 0.00149 0.026 0.000882 0.00702
2R:13586940-13587090:plus -40.2143 0 3.09174 2.44954 -18.7143 -17.2755 -26.4771 -2.15278 8.11009
0.0372 0.0103 0.00383 0.0116 0.0136 0.00544 0.0567 0.0311 0.000597
2R:13604200-13604350:minus -13.9286 -2.31193 1.04587 4.33945 -17.1837 -36.1122 -7.02752 -6 9.87156
0.00112 0.0213 0.00813 0.00509 0.00366 0.0878 0.0017 0.0761 0.000217
2R:13604200-13604350:plus -22.1837 5.86239 1.77982 7.70642 -27.0816 -17.2041 -18.9083 -6.48611 -0.495413
0.00315 0.00113 0.00627 0.000708 0.0243 0.00507 0.0172 0.0838 0.022
2R:13606840-13606990:minus -4.26531 4.46789 0.807339 7.17431 -16.7143 -0.204082 -9.94495 -0.166667
-3.10092 0.000132 0.00201 0.00884 0.00115 0.00319 0.000463 0.00305 0.0176 0.0458
2R:13606840-13606990:plus -21.6939 -0.899083 0.761468 4.0367 -17.0408 -27.602 -13.3394 -3.08333
-2.33945 0.00226 0.0139 0.00897 0.00578 0.00329 0.0416 0.00625 0.0395 0.0367
2R:136120-136270:minus -34.3061 -0.394495 -1.0367 2.66972 -17.4592 -9.57143 -15.3119 3.43056
1.54128 0.0317 0.0118 0.0161 0.0106 0.00491 0.00326 0.00954 0.00505 0.0115
2R:136120-136270:plus -31.7041 7.05505 -1.52294 5.37615 -35.9694 0.826531 -16.0642 9.375 -7.31193
0.0146 0.00063 0.0187 0.00384 0.0709 0.000153 0.011 0.000251 0.12
2R:13614320-13614470:minus -23.8878 1.40367 2.75229 7.17431 10.6531 -27.7551 -12.3211 2.56944
6.76147 0.00692 0.00625 0.00436 0.00115 0.0000102 0.0458 0.00495 0.00701 0.00141
2R:13614320-13614470:plus -31.5204 13.4592 13.9725 3.56881 -7.89796 -8.79592 -8.61468 -3.13889 -0.183486
0.0131 0.000064 7.38e-06 0.00699 0.000678 0.0019 0.00238 0.04 0.0197
2R:13620580-13620730:minus -20.9286 6.51376 -1.7156 6.09174 -18.3776 -19.2755 -11.0459 4.54167
3.46789 0.00183 0.000829 0.0198 0.00235 0.00926 0.0185 0.00377 0.0032 0.00582
2R:13620580-13620730:plus -32.8571 -1.11009 -1.34862 3.6055 -18.7551 -18.4184 -11.4862 4.125 1.6422
0.0244 0.0148 0.0178 0.00686 0.0144 0.0127 0.00413 0.00382 0.0111
2R:13624760-13624910:minus -32.6633 3.25688 3.46789 3.45872 0.836735 -18.051 -21.0367 2.88889
8.14679 0.0216 0.00316 0.00331 0.00731 0.000215 0.00886 0.0248 0.00622 0.000562
2R:13624760-13624910:plus -34.602 -1.24771 -2.56881 5.57798 -27.5306 10.3776 -35.4954 4.08333 1.2844
0.032 0.0155 0.0255 0.0034 0.0351 6.32e-06 0.168 0.00388 0.0129
2R:13627100-13627250:minus -33.1531 4.55046 5.92661 3.77064 0.877551 -8.79592 -28.2752 -1.33333
-4.61468 0.0273 0.00195 0.00119 0.00637 0.00019 0.0019 0.0714 0.0248 0.0658

2R:13627100-13627250:plus -3.40816 -2.86239 0.440367 4.22018 11.4286 -8.65306 -13.2294 -0.569444
-7.72477 0.0000685 0.0248 0.01 0.00531 1.97e-06 0.00156 0.00609 0.0199 0.129
2R:13635500-13635650:minus -31.8163 2.86239 2.61468 6.57798 20.1735 -18.4184 -13.8807 0.513889
3.05505 0.016 0.00366 0.0046 0.00167 5.68e-07 0.0127 0.00706 0.0142 0.00692
2R:13635500-13635650:plus -40.2857 6.04587 3.27523 6.57798 -18.1531 -18.9796 -26.9633 2.16667 -6.43119
0.0385 0.00104 0.00357 0.00167 0.00829 0.0155 0.0602 0.00812 0.0988
2R:13648500-13648650:minus -24.4184 -3.75229 1.13761 0.697248 -28.2245 0.979592 -25.2936 5.01389
-1.58716 0.00797 0.0316 0.00788 0.0224 0.049 0.000136 0.049 0.00261 0.03
2R:13648500-13648650:plus -23.9286 1.68807 2.25688 9.24771 -17.8571 -19.0816 -11.789 -2.84722 4.91743
0.00701 0.00563 0.00525 0.000409 0.00698 0.017 0.0044 0.0372 0.00321
2R:13654440-13654590:minus -23.1531 14.8807 3.30275 4.65138 -26.9286 -17.9388 -15.3486 8.29167
-2.33945 0.00542 2.63e-06 0.00353 0.00471 0.0214 0.00738 0.00961 0.000493 0.0367
2R:13654440-13654590:plus -31.4388 9.81651 1.14679 4.30275 -18.7551 -26.3367 -7.90826 4.94444 1.52294
0.0122 0.000147 0.00785 0.00517 0.0144 0.0221 0.00206 0.00269 0.0117
2R:13669360-13669510:minus -32.4388 -0.330275 14.2477 3.13761 -18.449 -18.0816 -17.055 4.45833
5.70642 0.0195 0.0115 5.31e-06 0.00854 0.0107 0.00934 0.0129 0.00332 0.00222
2R:13669360-13669510:plus -23.2551 10.3119 -3.66055 3.56881 -7.82653 -8.5 -15.5596 7.79167 3.58716
0.00583 0.000115 0.0346 0.00699 0.00059 0.00126 0.01 0.000656 0.00534
2R:13676640-13676790:minus -22.398 0.66055 -0.504587 0.183486 -8.70408 -36.4082 -15 4.52778
5.9633 0.00344 0.00818 0.0137 0.0256 0.00155 0.105 0.00897 0.00322 0.00188
2R:13676640-13676790:plus -14.0408 9.88073 0.0642202 7.99083 -26.5306 -8.53061 -15.7706 0.416667
-3.98165 0.00115 0.000142 0.0113 0.00059 0.0185 0.00136 0.0104 0.0147 0.0555
2R:13676980-13677130:minus -30.4082 1.43119 -5.33028 2.37615 -18.6735 -7.5 -16.789 -2.34722 -0.357798
0.00908 0.00619 0.0537 0.0119 0.0123 0.000614 0.0124 0.0327 0.021
2R:13676980-13677130:plus -13.3673 13.3878 5.98165 3.56881 -18.0816 -19.0816 0.440367 -6.83333 -0.605505
0.000813 0.0000869 0.00116 0.00699 0.00759 0.017 0.000255 0.0897 0.023
2R:13680060-13680210:minus -24.3367 4.37615 5.19266 0.321101 -16.4184 -17.9388 3.02752 2.76389
-2.56881 0.00789 0.00208 0.00164 0.0247 0.00304 0.00738 0.000117 0.00652 0.0396
2R:13680060-13680210:plus -13.1429 1.6055 5.40367 11.0275 -8.71429 -36.1122 5.63303 -3.90278 10.0367
0.00065 0.0058 0.0015 0.0000401 0.00159 0.0878 0.0000465 0.0481 0.000146
2R:13734040-13734190:minus -22.1429 3.68367 -1.76147 2.44037 -18.3776 -17.051 -22.5596 7.09722
3.20183 0.00305 0.00144 0.0201 0.0116 0.00926 0.00479 0.0326 0.000956 0.0065
2R:13734040-13734190:plus -11.1837 1.76147 0.486239 9.59633 -25.9286 -18.0102 0.972477 -2.09722 5.22936
0.000248 0.00548 0.00986 0.000275 0.0168 0.00812 0.000222 0.0306 0.00288
2R:13737720-13737870:minus -31.4796 -0.715596 0.229358 9.75229 -17.3776 -9.23469 -13.2294 -3.31944
-3.88991 0.0128 0.0131 0.0107 0.000226 0.00419 0.00238 0.00609 0.0418 0.054
2R:13737720-13737870:plus -30.7755 -4.76147 1 5.3945 -17.602 -19.3061 -12.6239 0.625 3 0.0102
0.0414 0.00827 0.00379 0.00547 0.0187 0.0053 0.0137 0.00702
2R:13738320-13738470:minus -30.4388 7.98165 14.2385 7.29358 -26.9694 -8.53061 -13.2569 3.44444
1.53211 0.00923 0.000385 5.44e-06 0.00104 0.023 0.00136 0.00613 0.00502 0.0116
2R:13738320-13738470:plus -22.9898 6.23853 4.75229 2.3945 -17.3776 -0.244898 -2.90826 -0.541667
-2.55046 0.00482 0.000946 0.00197 0.0119 0.00419 0.000496 0.000555 0.0197 0.0394
2R:13739020-13739170:minus -23.1122 3.64286 -2.33945 1.46789 -26.9694 -17.898 -26.8807 2.38889
6.07339 0.0053 0.00159 0.0239 0.0179 0.023 0.00726 0.0596 0.00749 0.0018
2R:13739020-13739170:plus -30.5204 1.20183 6.3211 6.00917 -8.93878 -9.53061 -20.5688 -0.694444
2.06422 0.00951 0.00673 0.000999 0.00255 0.00216 0.0031 0.0228 0.0206 0.00912
2R:13739820-13740110:minus -31.7449 -3.6422 3.77982 5.57798 -17.1531 -17.7143 -17.6514 0.986111
2.27523 0.0152 0.0307 0.00293 0.0034 0.00364 0.00634 0.0142 0.0122 0.00846
2R:13739820-13740110:plus -40.0612 2.66972 -3.44954 2.6789 -17.8571 -17.4898 -13.844 5.83333
-0.0825688 0.0358 0.00393 0.0327 0.0105 0.00698 0.0059 0.00701 0.00179 0.0189
2R:13740680-13740830:minus -33.0306 5 3.88073 1.08257 -18.4184 -10.0918 -11.1193 2 0.431193 0.026
0.00163 0.00281 0.0201 0.0101 0.00404 0.00382 0.00862 0.0164
2R:13740680-13740830:plus -30.6633 -5.74312 4.16514 6.33028 -26.9694 -26.5612 -15.1927 1.625 4.40367
0.00976 0.0535 0.00251 0.00198 0.023 0.0237 0.00932 0.00983 0.00373

2R:13742120-13742270:minus -34.4082 8.25688 6.15596 4.04587 -9.16327 -9.5 -22.5688 0.75 1.0367
0.0319 0.000332 0.00108 0.00572 0.00249 0.00294 0.0326 0.0132 0.0141
2R:13742120-13742270:plus -23.2551 -1.15596 -3.62385 1.77982 -18.4184 -16.9388 -13.9358 -0.347222
-2.77982 0.00583 0.0151 0.0343 0.0158 0.0101 0.00433 0.00715 0.0186 0.042
2R:13742540-13742690:minus -22.0306 4.57798 -2.42202 5.74312 -8.96939 -19.0816 -8.72477 1.81944
3.94495 0.00293 0.00193 0.0244 0.00302 0.00226 0.017 0.00243 0.00919 0.00452
2R:13742540-13742690:plus -23.1531 0.862385 1.99083 8.81651 -26.9286 -18.051 -16.1009 -0.222222
5.18349 0.00542 0.00761 0.0058 0.000501 0.0214 0.00886 0.011 0.0179 0.00295
2R:13743420-13743570:minus -23.2245 2.81651 1.55046 2.9633 -26.9694 -9.7551 -18.3303 -0.833333
7.06422 0.00576 0.00372 0.0068 0.00914 0.023 0.00358 0.0157 0.0215 0.00131
2R:13743420-13743570:plus -31.4796 -0.853211 4.04587 2.24771 -26.5306 -19.051 -16.4037 2.54167
-0.715596 0.0128 0.0137 0.00263 0.0127 0.0185 0.0165 0.0116 0.00709 0.0241
2R:13753500-13753650:minus -31.7755 8.56881 1.3211 2.43119 20.4694 -18.051 -9.53211 2.73611
4.31193 0.0157 0.000281 0.00738 0.0117 1.65e-07 0.00886 0.00282 0.00659 0.00386
2R:13753500-13753650:plus -33.5612 7.33945 0.229358 5.31193 -27.2653 -9.5 -20.1927 3.04167 -0.440367
0.0288 0.000544 0.0107 0.00393 0.0302 0.00294 0.0213 0.00587 0.0216
2R:13756420-13756570:minus -33.3061 13.7248 -1.75229 3.51376 -28.5714 -17.5 -27 7.27778 -4.29358
0.0279 0.0000116 0.0201 0.00709 0.0623 0.00599 0.0605 0.000869 0.06
2R:13756420-13756570:plus -21.5102 7.68807 0.669725 6.54128 1.61224 -9.79592 -4.50459 -0.972222
5.18349 0.00202 0.000452 0.00926 0.00173 0.0000631 0.00377 0.000862 0.0224 0.00295
2R:13756880-13757030:minus -13.7347 -4.0367 -1.18349 2.62385 -17.8265 0.27551 -23.8349 5.77778
1.00917 0.00105 0.0342 0.0169 0.0108 0.00691 0.000257 0.04 0.00184 0.0143
2R:13756880-13757030:plus -31.3367 -4.6055 -1.04587 2.53211 -17.7959 -18.3469 -22.3394 -0.388889
4.41284 0.0114 0.0398 0.0162 0.0113 0.00678 0.0124 0.0313 0.0188 0.00368
2R:13758220-13758370:minus -13.1837 3.68367 -1.66055 3.70642 -19.0102 0.0204082 -12.9083 4.72222
1.10092 0.000705 0.00144 0.0195 0.0066 0.0157 0.000377 0.00566 0.00296 0.0138
2R:13758220-13758370:plus -13.1837 2.63303 10.5229 5.94495 -19.0918 -19.4184 -4.87156 -0.819444
5.90826 0.000705 0.00398 0.000104 0.00261 0.0163 0.0198 0.000957 0.0214 0.00198
2R:13758580-13758730:minus -20.8163 -5.48624 9.08257 2.09174 -27.1939 -16.9796 -20.422 1.43056
-0.706422 0.00177 0.0501 0.000242 0.0137 0.0267 0.0044 0.0222 0.0105 0.024
2R:13758580-13758730:plus -22.449 1.9633 -0.779817 5.74312 -27.1224 -8.65306 -29.2936 -4.44444
1.41284 0.0035 0.00509 0.0149 0.00302 0.0245 0.00156 0.0823 0.0545 0.0121
2R:13758900-13759050:minus -20.6633 -4 0.568807 2.93578 -27.2959 -18.051 -2.58716 0.638889 2.54128
0.00173 0.0338 0.00958 0.00927 0.0303 0.00886 0.000511 0.0137 0.00787
2R:13758900-13759050:plus -39.949 -3.3578 -2.16514 -0.201835 -8.33673 -26.9796 -9.82569 -3.5 3.6789
0.0345 0.0284 0.0227 0.0285 0.00101 0.0306 0.00298 0.0437 0.00506
2R:13759500-13759650:minus -21.9184 4.19266 5.95413 3.70642 -27.6429 -18.2041 -15.3394 -0.180556
1.11009 0.00261 0.00224 0.00118 0.0066 0.037 0.0103 0.0096 0.0177 0.0138
2R:13759500-13759650:plus -31.4796 2.20183 -4.07339 2.34862 -27.2653 -18.8265 -18.7339 -0.458333
11.3853 0.0128 0.00466 0.0387 0.0121 0.0302 0.0148 0.0167 0.0192 0.0000815
2R:13982340-13982490:minus -23.7755 -1.45872 4.00917 6.84404 -18.4592 -9.09184 -20.3394 0.722222
7.15596 0.00678 0.0165 0.00267 0.0014 0.0109 0.00219 0.0218 0.0133 0.00124
2R:13982340-13982490:plus -14.5612 13.5306 -2.46789 9.7156 -18.9796 -17.9388 -15.1927 2.31944 -3.07339
0.00147 0.0000206 0.0248 0.000242 0.0155 0.00738 0.00932 0.00769 0.0454
2R:14013640-14013790:minus -31.5918 -2.36697 0.330275 2.24771 -18.449 -0.469388 -23.4679 0.430556
1.95413 0.0136 0.0216 0.0104 0.0127 0.0107 0.000533 0.0378 0.0146 0.00964
2R:14013640-14013790:plus -23.2245 -3.56881 7.26606 6.22936 -17.6429 -9.68367 -17.3853 5.5 9.14679
0.00576 0.0301 0.000638 0.00209 0.00562 0.00342 0.0136 0.00209 0.000414
2R:14015860-14016010:minus -24.6735 0.688073 9.10092 3.41284 -18.8265 -18.2755 -16.5413 3.02778
-2.3211 0.00825 0.0081 0.00024 0.00756 0.0149 0.0112 0.0119 0.0059 0.0365
2R:14015860-14016010:plus -22.8469 0.788991 1.22018 7.78899 -18.1939 -27.4082 -6.19266 0.402778 8.88073
0.00424 0.00781 0.00765 0.000642 0.0086 0.0356 0.00138 0.0147 0.000468
2R:14022100-14022250:minus -29.9898 3.99083 2.65138 2.3578 10.7245 -8.45918 -11.6514 12.8472
2.33028 0.00856 0.00241 0.00453 0.0121 5.2e-06 0.00111 0.00427 0.0000124 0.00836

2R:14022100-14022250:plus -40.4796 -1.22018 4.17431 1.53211 -27.1939 -0.244898 -18.7339 4.79167
-0.715596 0.0407 0.0154 0.0025 0.0174 0.0267 0.000496 0.0167 0.00288 0.0241
2R:14023020-14023170:minus -30.7755 1.62385 -2.43119 7.62385 -26.6327 -7.72449 -11.3119 -4.13889
11.8073 0.0102 0.00577 0.0245 0.000759 0.0188 0.00068 0.00398 0.0508 0.0000538
2R:14023020-14023170:plus -32.2857 -0.155963 -0.311927 6.2844 -27.3776 -7.23469 -21.2018 2.97222
1.53211 0.0185 0.0109 0.0128 0.00205 0.032 0.000557 0.0255 0.00603 0.0116
2R:14028180-14028330:minus -23.2245 -2.06422 -3.51376 2.19266 -17.4082 -8.02041 -18.7431 2.51389
1.07339 0.00576 0.0198 0.0333 0.0131 0.00421 0.000761 0.0168 0.00716 0.014
2R:14028180-14028330:plus -13 13.3878 -1.89908 5.65138 -26.898 -17.7857 -7.6055 -2.15278 2.00917
0.000563 0.0000869 0.021 0.00322 0.0203 0.0068 0.00194 0.0311 0.00934
2R:14028740-14028890:minus -22.9286 1.21101 -5.16514 0.119266 -18.2245 0.204082 -17.6422 -0.569444
1.61468 0.0046 0.00671 0.0515 0.026 0.00877 0.000296 0.0142 0.0199 0.0112
2R:14028740-14028890:plus -14.4388 -0.513761 -0.366972 3.04587 -26.7041 -8.68367 -11.1284 1.86111
7.68807 0.00137 0.0123 0.0131 0.00883 0.0193 0.00157 0.00383 0.00905 0.000841
2R:14029260-14029410:minus -31.1735 -0.385321 6.12844 3.47706 -18.7143 -27.449 -13.5505 -1.90278
3.78899 0.0109 0.0117 0.00109 0.00724 0.0136 0.0357 0.00656 0.029 0.00487
2R:14029260-14029410:plus -23.2653 1.2844 3.79817 1.05505 -17.4898 -26.7143 -12.8257 2.36111 1.33945
0.00593 0.00653 0.00291 0.0204 0.0053 0.0257 0.00555 0.00757 0.0125
2R:14038920-14039070:minus -33.3367 5.53211 6.27523 1.40367 10.3878 -8.72449 -22.0917 3.63889
-0.119266 0.0281 0.0013 0.00102 0.0184 0.0000174 0.00169 0.03 0.00465 0.0192
2R:14038920-14039070:plus -22.1735 -1.3578 5.98165 3.88991 0.571429 -18.898 -12.2569 8.26389 6.73394
0.0031 0.016 0.00116 0.00608 0.0003 0.0152 0.00488 0.000502 0.00143
2R:14044420-14044570:minus -24.2143 -0.788991 2.11009 0.183486 -17.8878 -27.5612 -5.99083 3.90278
0.33945 0.00754 0.0134 0.00554 0.0256 0.00704 0.0393 0.00131 0.00418 0.0168
2R:14044420-14044570:plus -21.7041 9.01835 4.04587 5.37615 -26.8571 -16.7857 -11.1651 -4.73611 -0.33945
0.00231 0.000221 0.00263 0.00384 0.0198 0.00426 0.00386 0.0582 0.0209
2R:14045220-14045370:minus -31.8163 -0.183486 1.20183 0.376147 -27.3367 -17.051 -19.156 2.61111
1.54128 0.016 0.011 0.0077 0.0244 0.0317 0.00479 0.0179 0.00691 0.0115
2R:14045220-14045370:plus -23.6224 2.16514 5.13761 -0.266055 -17.449 -7.45918 -15.7064 1.81944
2.85321 0.00658 0.00473 0.00168 0.029 0.00484 0.000582 0.0103 0.00919 0.00733
2R:14045580-14045730:minus -23.2245 7.00917 0.513761 3.43119 -18.5306 -27.6735 -7.12844 -1.55556
7.68807 0.00576 0.000646 0.00977 0.00749 0.0118 0.0443 0.00174 0.0264 0.000841
2R:14045580-14045730:plus -12.1837 -5.51376 -0.357798 3.19266 -17.7143 -8.57143 -8.87156 3.33333
0.0275229 0.000365 0.0504 0.013 0.00829 0.00627 0.00149 0.0025 0.00525 0.0183
2R:14047880-14048030:minus -30.4796 -2.10092 -4.20183 1.10092 -19.2755 -8.86735 -31.9908 11.5972
0.642202 0.00941 0.02 0.0401 0.02 0.0164 0.00198 0.118 0.0000452 0.0157
2R:14047880-14048030:plus -33.7347 0.788991 1.85321 2.22936 -8.37755 -26.8265 -19.2385 2.61111 -0.504587
0.0294 0.00781 0.0061 0.0128 0.00106 0.0285 0.0181 0.00691 0.0221
2R:14054600-14054750:minus -23.8061 13.7798 5.50459 1.55963 -27.2653 -0.244898 -11.7706 5.43056
4.09174 0.0068 0.0000109 0.00144 0.0173 0.0302 0.000496 0.00439 0.00216 0.00416
2R:14054600-14054750:plus -32.7449 4.81651 -2.18349 3.58716 -17.4592 9.60204 -14.1468 3.75 5.9633
0.0228 0.00175 0.0228 0.00694 0.00491 0.0000248 0.00749 0.00445 0.00188
2R:14059000-14059150:minus -12.1837 -1.78899 1.19266 6.00917 -26.2653 -18.2347 -7.29358 11.9444
3.08257 0.000365 0.0183 0.00773 0.00255 0.018 0.0104 0.00181 0.0000327 0.00681
2R:14059000-14059150:plus -14.0714 2.09174 -3.70642 -2.3211 -29.0918 -9.86735 -7.46789 2.52778 11.3853
0.00117 0.00485 0.0351 0.0569 0.0653 0.00391 0.00188 0.00712 0.0000815
2R:14059480-14059630:minus -42.4388 -0.770642 -0.623853 1.55963 -18.6735 -16.2347 -17.8991 5.625
0.587156 0.0766 0.0133 0.0142 0.0173 0.0123 0.00409 0.0147 0.00198 0.016
2R:14059480-14059630:plus -31.6633 -1.15596 8.76147 2.09174 -26.9286 -7.5 -6.68807 7.47222 3.42202
0.0141 0.0151 0.000288 0.0137 0.0214 0.000614 0.00156 0.000783 0.00597
2R:14061120-14061270:minus -32.6939 -0.137615 -1.77982 3.6055 -18.4592 -17.0102 -21.4495 8.5
0.201835 0.0217 0.0108 0.0202 0.00686 0.0109 0.00453 0.0267 0.000436 0.0175
2R:14061120-14061270:plus -33.7755 5.84404 1.74312 7.51376 -26 -17.0816 -15.7339 2.26389 3.94495
0.0297 0.00113 0.00635 0.000836 0.0171 0.00487 0.0103 0.00784 0.00452

2R:14062300-14062450:minus	-23.8163	6.77064	2.29358	5.85321	-18.4184	-9.79592	-25.1743	3.69444
3.45872	0.00682	0.000731	0.00518	0.00287	0.0101	0.00377	0.0482	0.00455	0.00591
2R:14062300-14062450:plus	-41.0714	-3.42202	7.01835	2.75229	-18.1939	-18.8571	-7.30275	-1.29167	4.01835
0.0469	0.0289	0.000721	0.0101	0.0086	0.0151	0.00181	0.0245	0.00429
2R:14066840-14066990:minus	-14.1531	3.86239	0.825688	-0.972477	-18.7551	-17.898	-11.4404	5.91667
4.56881	0.00121	0.00253	0.00878	0.0364	0.0144	0.00726	0.00409	0.00172	0.00356
2R:14066840-14066990:plus	-14.2245	6.11009	0.385321	1.50459	-17.449	-18.7551	7.38532	-5.33333	5.38532
0.00126	0.00101	0.0102	0.0176	0.00484	0.0141	0.000028	0.0663	0.00267
2R:14067620-14067770:minus	-32.2959	10.0917	-3.06422	1.05505	-18.7551	-8.38776	-8.3578	5.875
0.816514	0.0186	0.000128	0.0294	0.0204	0.0144	0.00101	0.00226	0.00176	0.015
2R:14067620-14067770:plus	-30.8469	14.1009	2.99083	0.00917431	-8.53061	-18.3469	-17.8349	1.97222
3.41284	0.0103	7.56e-06	0.00399	0.0269	0.0011	0.0124	0.0146	0.0087	0.006
2R:14071620-14071770:minus	-31.1837	-3.48624	7.11009	4.17431	-27.2347	-25.5306	-24.4495	0.291667
9.61468	0.0109	0.0294	0.000691	0.00537	0.0283	0.0205	0.0438	0.0153	0.00029
2R:14071620-14071770:plus	-41.4796	-0.155963	1.91743	-0.752294	-18.449	-18.2755	-27.2569	5.69444
-4.56881	0.0577	0.0109	0.00596	0.034	0.0107	0.0112	0.0625	0.00191	0.065
2R:14164700-14164850:minus	-24.1531	0.59633	-0.449541	6.00917	-27.1939	-19.051	-14.3761	5.73611
3.51376	0.00744	0.00837	0.0134	0.00255	0.0267	0.0165	0.00788	0.00188	0.00566
2R:14164700-14164850:plus	-14.6735	14.4128	-2.80734	3.77064	-26.8571	-18.0102	-18.7615	2.125	1.48624
0.00152	5.49e-06	0.0273	0.00637	0.0198	0.00812	0.0168	0.00824	0.0118
2R:14164860-14165010:minus	-33.0714	13.1009	3.14679	2.81651	-7.97959	-8.7551	-19.5413	4.88889
3.51376	0.0267	0.0000204	0.00375	0.00982	0.000799	0.00172	0.0191	0.00276	0.00566
2R:14164860-14165010:plus	-14.6735	14.4128	-2.80734	3.77064	-26.8571	-18.0102	-18.7615	2.125	1.48624
0.00152	5.49e-06	0.0273	0.00637	0.0198	0.00812	0.0168	0.00824	0.0118
2R:14176280-14176430:minus	-31.8571	0.12844	-3.98165	10.0275	-17.3776	-0.132653	-14.2385	1.20833
3.56881	0.0163	0.00987	0.0378	0.000156	0.00419	0.000444	0.00765	0.0113	0.00543
2R:14176280-14176430:plus	-22.8469	-2.20183	6.19266	2.81651	-18.4184	-19.0102	-9.53211	3.80556	8.11009
0.00424	0.0206	0.00106	0.00982	0.0101	0.0159	0.00282	0.00435	0.000597
2R:14195480-14195630:minus	-20.8163	2.47706	-1.6055	7.61468	-18.9796	-9.53061	-17.5872	3.20833
3.66972	0.00177	0.00421	0.0192	0.000779	0.0155	0.0031	0.014	0.00551	0.00514
2R:14195480-14195630:plus	-32.4082	0.541284	1.3945	5.54128	-8.93878	-18.7143	-19.0092	3.68056	7.73394
0.0191	0.00854	0.00719	0.00346	0.00216	0.014	0.0175	0.00457	0.000784
2R:14213280-14213430:minus	-31.2143	0.926606	11.055	3.87156	-8.90816	-9.79592	-12.1101	1.84722
-2.11927	0.0111	0.00743	0.0000772	0.00618	0.00192	0.00377	0.00472	0.0091	0.0344
2R:14213280-14213430:plus	-31.4694	-2.87156	2.44037	1.86239	-26.9286	0.316327	-7.98165	2.11111	0.816514
0.0124	0.0249	0.00491	0.0153	0.0214	0.000243	0.0021	0.00828	0.015
2R:14229320-14229470:minus	-22.8163	10.2018	9.89908	5.6422	-8.64286	0.0918367	-9.74312	-1.63889
0.93578	0.00418	0.000121	0.000151	0.00325	0.00139	0.000323	0.00293	0.027	0.0145
2R:14229320-14229470:plus	-23.4082	8	2.25688	7.00917	-18.1122	-19.0102	-14.3119	5.29167	3.09174
0.00623	0.000381	0.00525	0.00126	0.00771	0.0159	0.00777	0.0023	0.00678
2R:14239280-14239430:minus	-32.6327	2.56881	-4.52294	0.715596	-27.602	0.469388	-13.633	0.513889
6.54128	0.0212	0.00407	0.0436	0.0223	0.0364	0.000212	0.00668	0.0142	0.00151
2R:14239280-14239430:plus	-23.9184	-2.3945	3.6055	2.9633	-17.7857	0.0510204	-21.9908	-2.59722
-4.80734	0.00695	0.0218	0.00314	0.00914	0.00676	0.000349	0.0294	0.0349	0.0694
2R:14242320-14242470:minus	-23.8878	3.12844	2.61468	-0.366972	-17.1939	-18.8265	-18.945	-1.06944
9.3945	0.00692	0.00332	0.0046	0.0298	0.00377	0.0148	0.0173	0.023	0.000358
2R:14242320-14242470:plus	-12.1122	3.31193	3.21101	6.47706	-27.1939	-17.7143	-14.7706	-2.70833	3.94495
0.000336	0.0031	0.00366	0.00179	0.0267	0.00634	0.00856	0.0359	0.00452
2R:14243620-14243770:minus	-32.8776	-3.26606	4.62385	2.6422	-17.0408	-36.1122	-11.4954	3.91667
10.0367	0.0244	0.0277	0.00208	0.0107	0.00329	0.0878	0.00413	0.00416	0.000146
2R:14243620-14243770:plus	-24.0306	-0.155963	1.55963	1.82569	-18.7143	-27.3061	-9.92661	0.944444
8.41284	0.00723	0.0109	0.00678	0.0156	0.0136	0.0336	0.00304	0.0124	0.000519
2R:14244040-14244190:minus	-22.3673	-1.09174	-4.55963	9.87156	-17.3061	-18.1224	-13.4679	-6.83333
-2.30275	0.00341	0.0148	0.0441	0.000195	0.00389	0.00988	0.00644	0.0897	0.0363

2R:14244040-14244190:plus -32.4388 -3.09174 6.52294 3.97248 -18.4796 -0.244898 -14.8899 -3.125
11.8073 0.0195 0.0264 0.000908 0.00587 0.011 0.000496 0.00877 0.0399 0.0000538
2R:14286820-14286970:minus -32.0408 2.84404 -0.825688 -1.33028 -36.0102 -0.244898 -24.7431
4.13889 -1.05505 0.0174 0.00368 0.0151 0.0409 0.072 0.000496 0.0456 0.0038 0.0264
2R:14286820-14286970:plus -31.7041 -0.174312 2.25688 2.24771 -7.30612 -9.57143 -20.6972 -2.38889
-1.48624 0.0146 0.0109 0.00525 0.0127 0.000378 0.00326 0.0233 0.0331 0.0293
2R:14288000-14288150:minus -23.1531 4.86239 8.44954 6.72477 -17.9796 -9.09184 -23.1835 5.625
1.44037 0.00542 0.00172 0.000344 0.00154 0.00726 0.00219 0.0362 0.00198 0.012
2R:14288000-14288150:plus -32.0408 2.7156 6.34862 7.43119 -17.7449 -18.1224 -14.4404 13.3333 -2.61468
0.0174 0.00386 0.000987 0.000939 0.0063 0.00988 0.00799 6.96e-06 0.0401
2R:14293080-14293230:minus -40.3265 -1.05505 3.2844 2.41284 -8.93878 -18.6429 -17.2569 -0.763889
1.40367 0.0394 0.0146 0.00356 0.0118 0.00216 0.0139 0.0133 0.0211 0.0122
2R:14293080-14293230:plus -31.7041 -0.880734 5.36697 9.33028 -8.82653 1.09184 -24.1927 2.58333
-2.20183 0.0146 0.0138 0.00152 0.000374 0.00163 0.000091 0.0422 0.00698 0.0353
2R:14294280-14294430:minus -24.7755 3.68367 0.908257 6.84404 -26.8571 -18.2347 -18.8991 1.25 -4.20183
0.00828 0.00144 0.00853 0.0014 0.0198 0.0104 0.0172 0.0112 0.0584
2R:14294280-14294430:plus -30.551 3.70642 1.3211 -0.522936 -17.6429 -18.2755 -19.8257 9.68056
-1.37615 0.00959 0.00268 0.00738 0.0313 0.00562 0.0112 0.02 0.000205 0.0287
2R:14295220-14295370:minus -31.9694 4.13761 -0.0458716 0.66055 -27.8673 -0.540816 -16.7156
7.04167 -0.0733945 0.0168 0.00228 0.0118 0.0225 0.0391 0.00054 0.0122 0.000984 0.0188
2R:14295220-14295370:plus -22.7755 -2.01835 6.07339 3.0367 -18.4184 -18.2755 -16.8899 6.61111 -0.33945
0.00412 0.0195 0.00112 0.00886 0.0101 0.0112 0.0126 0.00123 0.0209
2R:14298920-14299070:minus -34.1122 14.5138 -4.72477 2.90826 -27.3367 -17.8571 -19.9633 1.59722
7.22018 0.0312 4.77e-06 0.046 0.00936 0.0317 0.00719 0.0205 0.00993 0.00119
2R:14298920-14299070:plus -31.7755 14.945 -2.91743 6.00917 -27.2653 -0.102041 -13.6697 -2.56944
-1.61468 0.0157 2.29e-06 0.0282 0.00255 0.0302 0.000443 0.00674 0.0346 0.0302
2R:14299420-14299570:minus -41.5204 0.385321 -4.7156 -1.54128 -18.7551 -18.2755 -25.2202 6.29167
-3.74312 0.0592 0.00902 0.0459 0.0439 0.0144 0.0112 0.0485 0.00144 0.0521
2R:14299420-14299570:plus -14.602 -0.577982 2.38532 5.49541 -8.64286 -8.0102 -21.4312 -4.59722
-4.09174 0.00149 0.0125 0.00501 0.00355 0.00139 0.000754 0.0266 0.0564 0.0568
2R:14299900-14300050:minus -33.2245 3.38532 -1.79817 2.07339 -26.6633 -8.38776 -10.844 1.61111
-0.220183 0.0276 0.00302 0.0204 0.0138 0.0189 0.00101 0.00362 0.00988 0.0201
2R:14299900-14300050:plus -30.8776 3.66055 -0.899083 -0.449541 -27.2959 0.602041 -18.1651 1.20833
9.9633 0.0103 0.00273 0.0155 0.0307 0.0303 0.000188 0.0153 0.0113 0.000171
2R:14301080-14301230:minus -23.3265 0.770642 1.98165 -0.0550459 -18.4184 -18.051 -11.4587 1.23611
1.43119 0.00607 0.00786 0.00582 0.0272 0.0101 0.00886 0.0041 0.0112 0.0121
2R:14301080-14301230:plus -24.6327 1.13761 1.6055 1.34862 -17.7143 0.755102 -6.97248 -1.18056 -2.15596
0.00824 0.00689 0.00667 0.0187 0.00627 0.000175 0.00168 0.0238 0.0348
2R:14301520-14301670:minus -41.7347 1.33028 4.48624 5.69725 -17.7551 -9.53061 -22.5229 0.791667
-3.51376 0.0643 0.00642 0.0022 0.00314 0.00658 0.0031 0.0323 0.013 0.0498
2R:14301520-14301670:plus -32.7755 0.798165 7.33028 4.22018 -8.93878 -26.602 -18.0734 0.277778 9.23853
0.0234 0.00779 0.000617 0.00531 0.00216 0.0248 0.0151 0.0153 0.000397
2R:14308700-14308850:minus -23.7857 1.74312 1.76147 6.3578 -8.96939 -8.72449 -11.6972 2.86111
2.47706 0.00679 0.00552 0.00631 0.00195 0.00226 0.00169 0.00432 0.00629 0.00801
2R:14308700-14308850:plus -22.7653 15.6514 5.6422 2.77064 -17.9796 0.0918367 -6.40367 -0.402778
3.66972 0.00405 8.05e-07 0.00135 0.01 0.00726 0.000323 0.00146 0.0189 0.00514
2R:14313800-14313950:minus -22.1939 -2.65138 2.92661 6.33945 -27.1224 -7.65306 -16.9817 1.75 -0.954128
0.00317 0.0234 0.00408 0.00196 0.0245 0.000647 0.0128 0.00941 0.0258
2R:14313800-14313950:plus -23.2653 3.66055 16.3945 5.90826 -17.5612 -8.68367 -15.9633 -3.15278 3.57798
0.00593 0.00273 6.75e-08 0.00271 0.00544 0.00157 0.0108 0.0402 0.00536
2R:14320640-14320790:minus 5 4.22018 -1.45872 9.7156 -18.051 -19.0816 -7.85321 -4.23611 -4.43119
0.0000168 0.00221 0.0184 0.000242 0.00746 0.017 0.00204 0.052 0.0627
2R:14320640-14320790:plus -31.9286 -0.431193 2.59633 9.7156 -8.64286 -16.7143 -25.3394 8.79167
5.23853 0.0166 0.0119 0.00463 0.000242 0.00139 0.00419 0.0493 0.000365 0.00285

2R:14321100-14321250:minus -31.398 4.88073 3.91743 7.27523 -27.2653 -27.7857 -4.25688 1.47222
-2.51376 0.0117 0.00171 0.00277 0.00109 0.0302 0.0466 0.000802 0.0104 0.039
2R:14321100-14321250:plus -22.2245 6.31193 6.37615 2.66972 -18.4592 -18.3061 -20.6789 -1.59722 -4.24771
0.00325 0.000913 0.000974 0.0106 0.0109 0.0114 0.0232 0.0267 0.0591
2R:14327860-14328010:minus -23 0.256881 -0.412844 6.12844 -26.9694 -18.051 -16.6239 8.02778
-4.66055 0.00498 0.00944 0.0133 0.00231 0.023 0.00886 0.0121 0.000574 0.0667
2R:14327860-14328010:plus -31.4082 -0.146789 0.715596 3.3578 19.9082 -19.051 -19.5872 2.875
4.18349 0.0119 0.0108 0.00912 0.00767 8.13e-07 0.0165 0.0192 0.00626 0.00404
2R:14328620-14328770:minus -31.0612 -0.201835 1.78899 -0.0550459 -17.1122 -16.9796 -21.7523
-4.48611 -8.04587 0.0106 0.011 0.00625 0.0272 0.00341 0.0044 0.0282 0.055 0.137
2R:14328620-14328770:plus -30.3673 0.293578 12.7064 5.98165 -26.9694 -27.6327 -9.06422 -2.34722 1.90826
0.00897 0.00932 0.0000263 0.00258 0.023 0.043 0.00259 0.0327 0.00974
2R:14330080-14330230:minus -33.0408 6.72477 2.33945 7.27523 -18.3469 -17.2755 -20.2844 -1.5 -2.06422
0.0264 0.000747 0.0051 0.00109 0.00894 0.00544 0.0216 0.026 0.0339
2R:14330080-14330230:plus -30.102 2.51376 0.0366972 0.743119 -27.0102 -27.9694 -15.6055 2.31944
11.4771 0.00859 0.00415 0.0115 0.0221 0.0237 0.0547 0.0101 0.00769 0.0000679
2R:14330600-14330750:minus -2.44898 1.56881 3.13761 6.47706 -27.1939 -17.7143 -9.18349 2.83333
4.82569 0.0000319 0.00588 0.00377 0.00179 0.0267 0.00634 0.00265 0.00635 0.00331
2R:14330600-14330750:plus -32.5102 0.412844 6.24771 3.02752 -17.0816 -18.5 -20.8899 -2.125 -4.87156
0.0202 0.00893 0.00103 0.00887 0.00334 0.0131 0.0241 0.0308 0.0708
2R:14333940-14334090:minus -14.0816 0.559633 -3.01835 7.62385 -19.3163 -0.132653 -16.2569 -0.152778
5.9633 0.00117 0.00848 0.029 0.000759 0.0165 0.000444 0.0113 0.0175 0.00188
2R:14333940-14334090:plus -30.4694 -4.34862 10.2385 7.29358 -17.1837 -9.45918 -4.3945 0.180556 1.73394
0.00926 0.0371 0.000123 0.00104 0.00366 0.00279 0.000835 0.0158 0.0106
2R:14338460-14338610:minus -30.4082 -1.99083 0.137615 3.18349 -18.7857 -18.7857 -18.4587 3.08333
3.30275 0.00908 0.0194 0.0111 0.00837 0.0148 0.0146 0.016 0.00578 0.00619
2R:14338460-14338610:plus -30.7041 4.90826 -0.944954 6.12844 -8.93878 -18.2041 -17.5596 3.06944
-5.88991 0.00993 0.00169 0.0157 0.00231 0.00216 0.0103 0.014 0.00581 0.0868
2R:14390960-14391110:minus -21.9592 3.25688 4.49541 1.62385 -18.449 -28.051 -22.055 3.05556
4.19266 0.00279 0.00316 0.00219 0.0168 0.0107 0.0563 0.0298 0.00584 0.00401
2R:14390960-14391110:plus -24.1837 0.770642 5.33945 10.8624 -17.3061 -8.45918 -24.8532 -1.76389
-0.0917431 0.00751 0.00786 0.00154 0.0000687 0.00389 0.00111 0.0463 0.028 0.019
2R:14417920-14418070:minus -31.6633 -1.51376 0.623853 3.65138 -7.67347 -26.6327 -0.678899 0.638889
6.17431 0.0141 0.0168 0.00941 0.00668 0.00055 0.0251 0.000331 0.0137 0.00171
2R:14417920-14418070:plus -23.5306 0.651376 4.20183 6.59633 -9.16327 0.826531 -17.4404 2.80556 1.77982
0.00646 0.00821 0.00247 0.00164 0.00249 0.000153 0.0137 0.00642 0.0103
2R:14419380-14419530:minus -23.0408 -0.321101 1.88073 6.47706 -7.86735 -17.9796 -15.7156 2.05556
7.6422 0.00512 0.0115 0.00604 0.00179 0.000617 0.00764 0.0103 0.00845 0.000893
2R:14419380-14419530:plus -21.6633 -0.697248 -1.75229 2.88991 -17.4184 -26.6735 -9.94495 -1.11111
1.78899 0.00223 0.013 0.0201 0.00942 0.00457 0.0256 0.00305 0.0233 0.0103
2R:14424060-14424210:minus -23.449 -0.633028 8.55963 -1 -8.71429 -19.051 -14.9174 2.38889
3.00917 0.00631 0.0127 0.000322 0.0367 0.00159 0.0165 0.00882 0.00749 0.00697
2R:14424060-14424210:plus -23.0408 -2.04587 6.17431 8.81651 -9.27551 -17.8265 -1.73394 15.9167 1.49541
0.00512 0.0197 0.00107 0.000501 0.00284 0.00699 0.000418 3.47e-08 0.0118
2R:14434640-14434790:minus -22.8469 4.3945 4.80734 6.06422 -26.898 -27.6735 -11.6147 3.58333
1.53211 0.00424 0.00207 0.00193 0.00239 0.0203 0.0443 0.00424 0.00475 0.0116
2R:14434640-14434790:plus -31.7449 1.44954 1.44037 3.79817 -17.4184 -7.93878 -20.9174 -0.375 -3.81651
0.0152 0.00615 0.00707 0.00629 0.00457 0.000736 0.0242 0.0188 0.0529
2R:14498720-14498870:minus -32.8469 -2.47706 4.83486 3.69725 -18.7143 -18.5612 0.486239 7.66667
4.91743 0.0243 0.0223 0.00191 0.00665 0.0136 0.0132 0.000252 0.000704 0.00321
2R:14498720-14498870:plus -23.3367 3.44954 3.80734 2.85321 -7.7449 -17.8265 -13.9358 3.20833 7.04587
0.00614 0.00295 0.0029 0.00955 0.000575 0.00699 0.00715 0.00551 0.00132
2R:14502620-14502770:minus -31.4388 13.4592 8.97248 6.37615 -17.0816 0.122449 -17.2569 -2.13889
-2.14679 0.0122 0.000064 0.000258 0.00192 0.00334 0.000312 0.0133 0.0309 0.0346

2R:14502620-14502770:plus -13.449 4.05505 0.0550459 7.3578 -26.9286 -9.0102 -14.4954 5.31944
-2.66055 0.000914 0.00235 0.0114 0.000965 0.0214 0.00208 0.00808 0.00227 0.0406
2R:14508960-14509110:minus -13.1531 0.568807 2.62385 1.12844 -9.0102 -26.5306 -19.7248 0.569444
-1.33028 0.00067 0.00845 0.00458 0.0198 0.00236 0.023 0.0196 0.014 0.0285
2R:14508960-14509110:plus -13.8878 0.366972 3.09174 0.211009 -18.5204 -18.2755 -7.79817 2.98611 4.98165
0.00111 0.00908 0.00383 0.0254 0.0117 0.0112 0.00202 0.006 0.00313
2R:14512700-14512850:minus -22.9592 6.18349 0.183486 2.82569 -18.6429 -16.2041 -2.76147 3.72222
1.94495 0.00475 0.000972 0.0109 0.00976 0.0121 0.00408 0.000534 0.0045 0.00968
2R:14512700-14512850:plus -31.7347 -2.22936 7.11927 7.7156 10.3571 -27.898 -20.7339 2.88889 4.14679
0.0149 0.0208 0.000686 0.000691 0.0000208 0.0537 0.0234 0.00622 0.00406
2R:14515340-14515490:minus -33.2245 3.91743 1.0367 2.80734 -27.5612 -17.2755 -6.24771 2.09722
-0.926606 0.0276 0.00248 0.00816 0.00985 0.0354 0.00544 0.0014 0.00832 0.0256
2R:14515340-14515490:plus -21.7755 -1.18349 1.53211 9.44037 -7.26531 -17.3469 -9.87156 9.44444 -1.11927
0.00241 0.0152 0.00685 0.000339 0.000375 0.0058 0.00301 0.00024 0.027
2R:14519340-14519490:minus -30.6633 0.761468 2.79817 3.13761 20.2041 -8.30612 -16.9358 -3.97222
1.31193 0.00976 0.00789 0.00429 0.00854 3.64e-07 0.000973 0.0127 0.0489 0.0127
2R:14519340-14519490:plus -33.1531 0.605505 8.80734 1 -9.45918 -8.79592 -19.3119 5.22222 -3.91743
0.0273 0.00834 0.000282 0.0206 0.00287 0.0019 0.0183 0.00238 0.0544
2R:14526280-14526430:minus -21.3265 -0.293578 -2.30275 2.13761 10.6224 -18.3061 -10.4954 4.09722
-2.52294 0.00193 0.0114 0.0236 0.0134 0.0000113 0.0114 0.00338 0.00386 0.0391
2R:14526280-14526430:plus -22.2857 -2.10092 1.30275 3.34862 -18.4796 -27.7551 -21.3028 4.11111 -0.137615
0.00331 0.02 0.00743 0.0077 0.011 0.0458 0.026 0.00384 0.0194
2R:14526740-14526890:minus 6.18367 2.56881 -3.23853 4.37615 -8.60204 0.244898 -10.2018 3 -2.88991
5.23e-06 0.00407 0.0308 0.00504 0.00125 0.000285 0.0032 0.00597 0.0433
2R:14526740-14526890:plus -14.1429 15.1376 -3.84404 1.10092 -27.1633 -9.45918 -8.98165 1.81944 3.45872
0.0012 1.81e-06 0.0364 0.02 0.025 0.00279 0.00255 0.00919 0.00591
2R:14539580-14539730:minus -22.7041 5.47706 -1.31193 9.08257 -26.9286 -9.53061 -16.2294 -0.694444
-0.899083 0.00395 0.00133 0.0176 0.000444 0.0214 0.0031 0.0113 0.0206 0.0254
2R:14539580-14539730:plus -32.4796 2.88991 -0.275229 3.18349 -18.6429 -16.3469 -12.3761 6.09722
1.22018 0.02 0.00362 0.0127 0.00837 0.0121 0.00416 0.00501 0.00158 0.0133
2R:14540380-14540530:minus -30.6327 0.724771 7.38532 10.7615 -18.0816 1.09184 -23.4312 -6.31944
-7.51376 0.00968 0.008 0.0006 0.0000826 0.00759 0.000091 0.0376 0.0811 0.124
2R:14540380-14540530:plus -14.4796 -2.78899 5.83486 10.8624 -18.5306 -9.57143 0.40367 2.81944 10.3486
0.00142 0.0243 0.00124 0.0000687 0.0118 0.00326 0.000257 0.00639 0.0000988
2R:14548020-14548170:minus -41.0612 -3.7156 -0.880734 -0.330275 1.07143 -8.27551 -12.9633
1.63889 -0.0825688 0.0466 0.0313 0.0154 0.0295 0.000125 0.000919 0.00573 0.00978 0.0189
2R:14548020-14548170:plus -33.2653 4.36697 1.22936 0.633028 -26.3061 -9.27551 -12.5321 1.44444 3.47706
0.0277 0.00209 0.00763 0.0229 0.0181 0.00244 0.00519 0.0105 0.00575
2R:14553340-14553490:minus -33.5612 2.3945 0.137615 -0.908257 -28.1939 -8.02041 -21.4128 12.2083
-3.61468 0.0288 0.00434 0.0111 0.0357 0.0482 0.000761 0.0265 0.0000252 0.0509
2R:14553340-14553490:plus -24.6327 -1.34862 1.52294 1.70642 -27.7959 -0.469388 -24.8073 0.847222
2.72477 0.00824 0.016 0.00687 0.0162 0.0386 0.000533 0.046 0.0128 0.00759
2R:14554080-14554230:minus -32.7449 1.44037 4.12844 4.65138 -18.2245 -9.42857 -18.7706 -2.19444
-2.77982 0.0228 0.00617 0.00254 0.00471 0.00877 0.0027 0.0168 0.0314 0.042
2R:14554080-14554230:plus -30.6735 -3.15596 10.8991 3.45872 -17.9796 -8.38776 -26.0917 5.06944 -0.816514
0.00978 0.0269 0.0000848 0.00731 0.00726 0.00101 0.0541 0.00255 0.0249
2R:14556000-14556150:minus -22.8571 -0.522936 1.89908 0.137615 -8.12245 -0.244898 -28.3486
6.27778 -3.52294 0.00427 0.0123 0.006 0.0259 0.000843 0.000496 0.0721 0.00145 0.0499
2R:14556000-14556150:plus -30.4082 -1.34862 2.17431 5.94495 -27.5306 -0.244898 -15.8807 2.16667
0.174312 0.00908 0.016 0.00542 0.00261 0.0351 0.000496 0.0106 0.00812 0.0177
2R:14556460-14556610:minus -3.07143 4.90816 5.76147 2.16514 -18.1531 -18.0102 -15.7064 0.902778
-0.284404 0.0000441 0.00023 0.00128 0.0132 0.00829 0.00812 0.0103 0.0125 0.0207
2R:14556460-14556610:plus -30.6224 -0.40367 1.42202 0.0275229 -17.4592 -26.1224 -14.9541 6.83333
5.17431 0.00964 0.0118 0.00712 0.0267 0.00491 0.0217 0.00889 0.0011 0.00296

2R:14558340-14558490:minus -32.6633 -5.5 -2.16514 0.431193 -18.2245 -8.87755 -25.9174 -0.430556
-4.62385 0.0216 0.00529 0.0227 0.0241 0.00877 0.00198 0.0529 0.0191 0.066
2R:14558340-14558490:plus -30.7041 -1.16514 3.41284 2.70642 -27 -17.9796 -20.1101 1.88889 -2.3945
0.00993 0.0151 0.00338 0.0103 0.0233 0.00764 0.021 0.00896 0.0375
2R:14560960-14561110:minus -4.85714 -1.12844 7.89908 10.5963 -17.3776 -26.898 3.24771 5.34722
11.3853 0.000202 0.0149 0.000462 0.0001 0.00419 0.0302 0.000108 0.00225 0.0000815
2R:14560960-14561110:plus -23.1531 0.0825688 3.80734 6.31193 -26.6327 -17.7143 -10.3119 -1.69444
-3.88073 0.00542 0.01 0.0029 0.00199 0.0188 0.00634 0.00326 0.0274 0.0539
2R:14575440-14575590:minus -41.2959 6.55963 -3.38532 2.25688 -19.3163 -9.72449 -16.5229 3.77778
-6.01835 0.0529 0.00081 0.0321 0.0127 0.0165 0.00352 0.0119 0.0044 0.0892
2R:14575440-14575590:plus -3.07143 9.34862 4.70642 5.81651 10.9184 -8.65306 -17 -8.22222 -0.146789
0.0000441 0.000187 0.00201 0.00292 4.19e-06 0.00156 0.0128 0.115 0.0194
2R:14576160-14576310:minus -32.102 -1.58716 4.59633 0.311927 -18.7143 -17.9388 -6.98165
-2.05556 5.13761 0.0177 0.0172 0.0021 0.0247 0.0136 0.00738 0.00168 0.0303 0.00298
2R:14576160-14576310:plus -22.5918 4.94495 -3.00917 2.88991 -18.1939 -17.7857 -12.1651
0.458333 -2.46789 0.00365 0.00166 0.0289 0.00942 0.0086 0.0068 0.00478 0.0145 0.0384
2R:14576520-14576670:minus -13.5918 -2.77982 0.0458716 2 -17.1837 -35.449 -14.4954
13.7361 10.1284 0.00099 0.0243 0.0114 0.0143 0.00366 0.0789 0.00808 3.9e-06 0.000129
2R:14576520-14576670:plus -24.4898 0.504587 1.23853 3.13761 -8.64286 -17.051 -13.2477
4.08333 3.17431 0.00807 0.00865 0.0076 0.00854 0.00139 0.00479 0.00612 0.00388 0.00653
2R:14577460-14577610:minus -24.449 5.36697 1.37615 0.825688 -18.8265 -27.3776 -15.8532
1.15278 10.3486 0.00802 0.0014 0.00724 0.0216 0.0149 0.0351 0.0106 0.0115 0.0000988
2R:14577460-14577610:plus -30.6735 -1.07339 3.31193 2.21101 -26.5918 -16.9388 -27.7248
-1.88889 -10.4954 0.00978 0.0147 0.00352 0.0129 0.0186 0.00433 0.0663 0.0289 0.212
2R:14583100-14583250:minus -31.398 -4.55046 5.66972 2.08257 -8.70408 -27.8265 -9.3578 -9.52778
11.3853 0.0117 0.0392 0.00134 0.0137 0.00155 0.0498 0.00273 0.143 0.0000815
2R:14583100-14583250:plus -23.4796 2.70642 0.0275229 3.97248 -7.89796 -9.53061 -5.87156
0.722222 1.22936 0.00634 0.00387 0.0115 0.00587 0.000678 0.0031 0.00127 0.0133 0.0132
2R:14586000-14586150:minus -2.59184 -0.293578 13.0459 11.1927 -17.8163 -9.94898 -19.633
-5.38889 -2.92661 0.0000341 0.0114 0.0000199 0.0000254 0.00682 0.00393 0.0193 0.067
0.0437
2R:14586000-14586150:plus -21.5816 5.25688 -3.07339 1.92661 -26.4898 -18.2041 -18.6697
-1.29167 -0.0825688 0.00205 0.00146 0.0294 0.0148 0.0183 0.0103 0.0166 0.0245 0.0189
2R:14593380-14593530:minus -24.4592 -1.88073 4.18349 7.27523 -28.7551 1.05102 -22.7615
4.20833 -2.34862 0.00802 0.0188 0.00249 0.00109 0.0637 0.000107 0.0337 0.00369 0.0368
2R:14593380-14593530:plus -31.4796 6.51376 1.65138 6.86239 -18.7857 0.346939 -6.63303
3.04167 -5.08257 0.0128 0.000829 0.00656 0.00136 0.0148 0.000221 0.00154 0.00587 0.0741
2R:14598300-14598450:minus -32.2245 14.4128 -0.577982 3.79817 -17.7857 9.30612 -10.2661
4.09722 -3.09174 0.0183 5.49e-06 0.014 0.00629 0.00676 0.0000398 0.00324 0.00386 0.0456
2R:14598300-14598450:plus -23.7857 14.0275 13.7064 4.9633 -28.3061 -18.3061 -28.9541
0.569444 -2.58716 0.00679 8.23e-06 0.0000106 0.0044 0.0553 0.0114 0.0784 0.014 0.0397
2R:14642380-14642530:minus -33 -4.23853 3.48624 1.53211 -27.1939 -8.53061 -14.2385
4.13889 -0.706422 0.0258 0.0361 0.00329 0.0174 0.0267 0.00136 0.00765 0.0038 0.024
2R:14642380-14642530:plus -12.1122 -0.944954 4.16514 10.8624 -17.4184 -9.34694 -18.9358
-6 -0.697248 0.000336 0.0141 0.00251 0.0000687 0.00457 0.00261 0.0173 0.0761 0.0239
2R:14658380-14658530:minus -23.5918 -4.74312 4.98165 7.17431 -18.2245 -17.9796 -15.578
-2.38889 -0.743119 0.00656 0.0412 0.00179 0.00115 0.00877 0.00764 0.01 0.0331 0.0243
2R:14658380-14658530:plus -31.8469 4.31193 -0.733945 3.92661 -18.6837 -18.7143 -23.422 5.5
1.70642 0.0162 0.00213 0.0147 0.00601 0.0126 0.014 0.0376 0.00209 0.0108
2R:14658940-14659090:minus -22.5102 3.42202 1.47706 2.7156 -18.4898 -28.602 -9.12844 -1.95833
8.10092 0.00354 0.00298 0.00698 0.0102 0.0116 0.0648 0.00262 0.0295 0.000623
2R:14658940-14659090:plus -21.3673 -0.954128 8.90826 2.66972 -26.3061 -9.42857 -2.6789
0.444444 7.36697 0.00195 0.0141 0.000267 0.0106 0.0181 0.0027 0.000523 0.0145 0.00108
2R:14662560-14662710:minus -33.102 -4.43119 11.9541 2.33028 -18.5204 -10.0204 -8.44954

0.875 4.14679 0.0268 0.038 0.0000446 0.0123 0.0117 0.00399 0.0023 0.0127 0.00406
2R:14662560-14662710:plus -22.2245 4.77064 1 6.45872 -27.898 -7.5 -11.1927 1.26389 -5.91743
0.00325 0.00179 0.00827 0.00182 0.04 0.000614 0.00388 0.0111 0.0874
2R:14662940-14663090:minus -12.6735 1.73394 -1.74312 2.07339 -17.4184 -27.4898 -9.26606
1.83333 4.14679 0.000461 0.00553 0.02 0.0138 0.00457 0.0363 0.00269 0.00914 0.00406
2R:14662940-14663090:plus -32.0714 7.29358 0.220183 4.04587 0.581633 -0.540816 -17.4404
2.5 4.19266 0.0176 0.000556 0.0108 0.00572 0.000292 0.00054 0.0137 0.0072 0.00401
2R:14664840-14664990:minus -22.2245 0.651376 3.87156 4.7156 -18.9796 -9.60204 -6.2844
1.75 -0.431193 0.00325 0.00821 0.00282 0.00464 0.0155 0.00334 0.00141 0.00941 0.0214
2R:14664840-14664990:plus -23.449 -2.04587 3.91743 6.41284 -18.1531 -9.72449 -21.1009
1.41667 1.79817 0.00631 0.0197 0.00277 0.00189 0.00829 0.00352 0.025 0.0106 0.0102
2R:14666800-14666950:minus -22.7755 1.29358 -3.06422 7.70642 -18.3776 -17.9388 -6.08257
4.72222 2.74312 0.00412 0.00651 0.0294 0.000708 0.00926 0.00738 0.00134 0.00296 0.00751
2R:14666800-14666950:plus -40.2959 5.11927 -1.43119 2.81651 -27.1939 -7.57143 -27.9817
5.51389 -5.55963 0.0389 0.00155 0.0182 0.00982 0.0267 0.000644 0.0686 0.00208 0.0813
2R:14684380-14684530:minus -23.2143 8.09174 9.97248 3.90826 -17.449 -18.8265 -18.844 -2 0.93578
0.00559 0.000363 0.000144 0.00603 0.00484 0.0148 0.017 0.0298 0.0145
2R:14684380-14684530:plus -13.2959 2.3945 9.20183 3.59633 -17.9796 0.0204082 -9.59633
2.38889 2.58716 0.000775 0.00434 0.000225 0.00693 0.00726 0.000377 0.00286 0.00749 0.00779
2R:14686840-14686990:minus -21.8878 1.73394 9.37615 0.137615 -17.0408 -27.4898 -15.9174
-0.152778 3.7156 0.00258 0.00553 0.000204 0.0259 0.00329 0.0363 0.0107 0.0175 0.00502
2R:14686840-14686990:plus -30.449 -1.34862 1.44037 -0.412844 -17.1122 -17.051 -20.789 6.33333
6.44954 0.00925 0.016 0.00707 0.0304 0.00341 0.00479 0.0237 0.00141 0.00154
2R:14688000-14688150:minus -32.7041 -0.256881 1.36697 9.3211 -8.71429 -8.23469 -23.5688
3.19444 3.78899 0.0222 0.0113 0.00726 0.000391 0.00159 0.000875 0.0384 0.00554 0.00487
2R:14688000-14688150:plus -33.2245 -1.72477 -1.69725 1.57798 -26.9286 -8.79592
-21.7156 2.73611 2.57798 0.0276 0.0179 0.0197 0.0171 0.0214 0.0019 0.028 0.00659 0.0078
2R:14690020-14690170:minus -22.5918 2.34862 1.98165 7.80734 -17.3776 -18.2755 -13.1835
8.11111 1.74312 0.00365 0.00441 0.00582 0.00063 0.00419 0.0112 0.00603 0.000547 0.0105
2R:14690020-14690170:plus -13 -1.37615 7.12844 9.24771 -8.33673 -27.5612 -12.3394
-3.54167 12.2752 0.000563 0.0161 0.000683 0.000409 0.00101 0.0393 0.00497 0.0442 0.0000141
2R:14690920-14691070:minus -22.7041 -0.431193 0.110092 2.86239 -18.6837 0.540816
-28.0642 -3.72222 4.13761 0.00395 0.0119 0.0112 0.00954 0.0126 0.0002 0.0694 0.0461 0.00408
2R:14690920-14691070:plus -34.2653 1.21101 -1.48624 1.53211 -27.8265 -9.5 -11.3761
5.51389 5.01835 0.0316 0.00671 0.0185 0.0174 0.0388 0.00294 0.00403 0.00208 0.00309
2R:14693800-14693950:minus -29.102 2.93578 -3.33945 2.58716 -7.89796 -17.7551 -12.5138
-1.94444 2.98165 0.00833 0.00356 0.0317 0.011 0.000678 0.00643 0.00517 0.0294 0.00707
2R:14693800-14693950:plus -12.8469 -0.119266 -4.02752 3.95413 -18.7143 1.7551 -20.3119
-0.208333 3.87156 0.000502 0.0107 0.0383 0.00593 0.0136 0.0000519 0.0217 0.0178 0.00477
2R:14694780-14694930:minus -24.7755 0.770642 -3.09174 5.43119 -9.23469 -18.1224
-13.5138 2.29167 7.62385 0.00828 0.00786 0.0296 0.00371 0.00277 0.00988 0.0065 0.00776 0.000941
2R:14694780-14694930:plus -22.3265 3.64286 4.14679 3.00917 -18.7143 -26.602 -8.14679 9.44444
3.14679 0.00337 0.00159 0.00252 0.00894 0.0136 0.0248 0.00217 0.00024 0.00666
2R:14706900-14707050:minus -32.3061 1.94495 3.02752 1.97248 -27.2653 -9.20408 2.20183 2.36111
1.37615 0.0186 0.00512 0.00393 0.0146 0.0302 0.00225 0.000155 0.00757 0.0123
2R:14706900-14707050:plus -21.551 1.29358 0.0825688 2.84404 10.9184 -18.2347 4.16514 -1.95833
2.47706 0.00205 0.00651 0.0113 0.00961 4.19e-06 0.0104 0.0000777 0.0295 0.00801
2R:14717180-14717330:minus -21.6327 -0.12844 2.40367 11.1927 -17.0408 -9.42857 -8.01835
-1.5 0.12844 0.00215 0.0108 0.00498 0.0000254 0.00329 0.0027 0.00211 0.026 0.0179
2R:14717180-14717330:plus -13.4796 4.97959 0.899083 2.50459 -27.2653 -17.9388 -17.4954
3.13889 -0.761468 0.000935 0.000156 0.00856 0.0114 0.0302 0.00738 0.0138 0.00566 0.0244
2R:14721360-14721510:minus -14.0714 -1.3211 0.770642 3.41284 -8.86735 -9.38776 -20.2569
-4.27778 -2.81651 0.00117 0.0159 0.00895 0.00756 0.00173 0.00266 0.0215 0.0525 0.0424
2R:14721360-14721510:plus -31.7041 -0.155963 1.04587 5.94495 -18.0204 -18.3469 -13.367

1.19444 0.93578 0.0146 0.0109 0.00813 0.00261 0.00733 0.0124 0.00629 0.0114 0.0145
2R:14722160-14722310:minus -23.1837 -0.183486 3.29358 5.93578 -18.1531 -17.0102 -16.1468
2.41667 -3.15596 0.00553 0.011 0.00354 0.00264 0.00829 0.00453 0.0111 0.00742 0.0464
2R:14722160-14722310:plus -14.1531 1 7.54128 7.21101 -8.93878 -27.1224 -20.7523 2.98611
-1.09174 0.00121 0.00724 0.000555 0.0011 0.00216 0.0325 0.0235 0.006 0.0268
2R:14722700-14722850:minus -41.9286 0.174312 -2.04587 -1.76147 -28.5306 -10.0918
-16.8991 6.75 3.95413 0.0676 0.00971 0.0219 0.0471 0.061 0.00404 0.0126 0.00114 0.00448
2R:14722700-14722850:plus -12.8571 -0.477064 0.642202 4.23853 -16.449 -17.7857 -15.3945
-3.22222 3.08257 0.000505 0.0121 0.00935 0.00522 0.00307 0.0068 0.0097 0.0408 0.00681
2R:14723240-14723390:minus -23.2653 -0.0458716 6.0367 9.75229 -8.82653 -26.602 -11.2202
3.01389 3.05505 0.00593 0.0105 0.00113 0.000226 0.00163 0.0248 0.0039 0.00593 0.00692
2R:14723240-14723390:plus -32.9694 -4.6422 7.77064 2.66972 -27.7551 -18.5714 -20.2294
2.20833 3.46789 0.0254 0.0401 0.000494 0.0106 0.0385 0.0136 0.0214 0.008 0.00582
2R:14723560-14723710:minus -34.3367 4.0367 0.137615 2.09174 -18.4184 -17.3469 -22.5963
8.54167 -0.908257 0.0318 0.00237 0.0111 0.0137 0.0101 0.0058 0.0328 0.000425 0.0255
2R:14723560-14723710:plus -32.0714 -3.88073 0.0825688 7.6422 -27.1939 -18.1224
-13.3303 3.31944 5.49541 0.0176 0.0328 0.0113 0.000721 0.0267 0.00988 0.00624 0.00528 0.0025
2R:14733080-14733230:minus -13.2551 6.20183 -1.48624 4.24771 -16.4898 -28.051 -7.66972
-1.73611 0.816514 0.000751 0.000962 0.0185 0.00519 0.00312 0.0563 0.00196 0.0277 0.015
2R:14733080-14733230:plus -24.4082 7.44954 1.23853 2.87156 -17.7551 -9.23469 -22.633 6.16667
-0.761468 0.00797 0.000512 0.0076 0.00948 0.00658 0.00238 0.033 0.00153 0.0244
2R:14733820-14733970:minus -23.6224 -4.90826 13.156 7.29358 -8.86735 -9.5 -23.5963
-2.22222 11.8532 0.00658 0.0431 0.0000178 0.00104 0.00173 0.00294 0.0386 0.0316 0.0000428
2R:14733820-14733970:plus -34.3367 -0.431193 1.86239 3.31193 -17.449 -17.2041 -19.4587
-1.54167 2.33028 0.0318 0.0119 0.00608 0.00786 0.00484 0.00507 0.0188 0.0263 0.00836
2R:14734040-14734190:minus -13.1429 8.19266 -3.43119 7.44037 -26.1939 -18.1224 -9.34862
-0.180556 2.00917 0.00065 0.000343 0.0325 0.000912 0.0175 0.00988 0.00273 0.0177 0.00934
2R:14734040-14734190:plus -33.3367 3.85321 3.84404 5.06422 -18.6837 -9.27551 -16.9083
-1.93056 -5.36697 0.0281 0.00254 0.00286 0.00433 0.0126 0.00244 0.0126 0.0293 0.0785
2R:14737800-14737950:minus -41.2551 3.53211 2.87156 5.59633 -18.7143 -17.5714 -17.0367
-1.55556 -4.79817 0.0513 0.00286 0.00417 0.00331 0.0136 0.00616 0.0129 0.0264 0.0693
2R:14737800-14737950:plus -31.5102 1.57798 5.00917 5.30275 -27 -17.2755 -12.6514 0.194444
4.40367 0.013 0.00586 0.00177 0.00396 0.0233 0.00544 0.00534 0.0157 0.00373
2R:14738100-14738250:minus -24.5204 0.733945 8.6422 1.98165 -18.449 -8.5 -14.789 -0.166667
0.385321 0.00813 0.00797 0.000308 0.0145 0.0107 0.00126 0.00859 0.0176 0.0166
2R:14738100-14738250:plus -23.6531 -1.24771 14.8532 1 -27.7551 -17.5714 -13.8716
6.80556 3 0.00661 0.0155 2.28e-06 0.0206 0.0385 0.00616 0.00705 0.00111 0.00702
2R:14742720-14742870:minus -23.8061 0.146789 8.11927 3.81651 -18.1224 -19.1224 -9.16514
-4.59722 3.20183 0.0068 0.0098 0.000411 0.00626 0.00782 0.0175 0.00264 0.0564 0.0065
2R:14742720-14742870:plus -32.5918 -4.42202 -2.12844 2.22018 -28.5306 -10.0918
-12.6881 7.61111 1.22936 0.021 0.0379 0.0224 0.0129 0.061 0.00404 0.00538 0.000726 0.0132
2R:14745040-14745190:minus -21.9592 0.889908 11.4495 7.2844 -18.4898 -9.02041 -13.9633
5.91667 5.44037 0.00279 0.00753 0.0000608 0.00105 0.0116 0.00213 0.0072 0.00172 0.00259
2R:14745040-14745190:plus -14.1122 2.16514 0.0550459 4.91743 -18.3776 -17.7551 -10.578
-1.625 3.07339 0.00119 0.00473 0.0114 0.00447 0.00926 0.00643 0.00343 0.0269 0.00683
2R:14745420-14745570:minus -13.3776 0.0825688 4.08257 6.57798 -27.898 -17.5612 -13.6789
1.51389 5.69725 0.000831 0.01 0.00259 0.00167 0.04 0.00604 0.00675 0.0102 0.00225
2R:14745420-14745570:plus -31.7347 1.27523 2.0367 6.29358 -18.4184 -18.1224 -22.1193
0.666667 7.42202 0.0149 0.00655 0.0057 0.00202 0.0101 0.00988 0.0301 0.0135 0.00106
2R:14858600-14858750:minus -21.7041 3.97248 1.63303 2.49541 -27.9388 -25.898 -14.3761 2.97222
-0.0733945 0.00231 0.00243 0.00661 0.0114 0.0415 0.0214 0.00788 0.00603 0.0188
2R:14858600-14858750:plus -23.2959 3.43119 -0.880734 6.00917 0.316327 -0.0510204 -14.3211
8.47222 -3.29358 0.00605 0.00297 0.0154 0.00255 0.000341 0.000429 0.00778 0.000443 0.0474
2R:14860000-14860150:minus -21.8571 14.7523 0.275229 7.27523 -27.1939 -8.68367 -28.3578

3.76389 -3.25688 0.00249 3.48e-06 0.0106 0.00109 0.0267 0.00157 0.0722 0.00442 0.0472
2R:14860000-14860150:plus -23.4898 2.98165 0.807339 -0.40367 -18.6837 -8.5 -16.3578
-1.15278 3.88991 0.00639 0.0035 0.00884 0.0302 0.0126 0.00126 0.0115 0.0236 0.00467
2R:14860420-14860570:minus -22 -1.66972 6.47706 4.13761 -8.93878 -9.57143 -21.5505 4.44444 -2.25688
0.00291 0.0176 0.000928 0.00546 0.00216 0.00326 0.0272 0.00334 0.0357
2R:14860420-14860570:plus -29.2143 -2.30275 0.266055 3.01835 -8.37755 -18.0102 -2.93578 2.58333 -4.68807
0.00836 0.0212 0.0106 0.0089 0.00106 0.00812 0.000559 0.00698 0.0672
2R:14863180-14863330:minus -31.1429 1.99083 7.34862 3.76147 -17.1531 -9.5 -21.0367 -1.65278 -1.47706
0.0108 0.00504 0.000611 0.00645 0.00364 0.00294 0.0248 0.0271 0.0292
2R:14863180-14863330:plus -32.3571 -3.27523 1.33028 4.04587 -17.7857 -17.0816 -18.5321 4.26389 1.95413
0.0187 0.0278 0.00736 0.00572 0.00676 0.00487 0.0162 0.0036 0.00964
2R:1491960-1492110:minus -31.6633 -1.91743 4.07339 4.22018 -18.7857 -18.4184 -18.9083 3.125 -0.669725
0.0141 0.019 0.0026 0.00531 0.0148 0.0127 0.0172 0.00569 0.0236
2R:1491960-1492110:plus -33.8061 0.412844 -3.58716 2.17431 -17.9388 -8.79592 -21.1284 -0.527778
-0.174312 0.0298 0.00893 0.0339 0.0132 0.00715 0.0019 0.0252 0.0196 0.0196
2R:1492240-1492390:minus -22.0408 -2.56881 2.30275 -0.385321 -17.1531 -8.68367 -12.5229 4.36111
-1.13761 0.00296 0.0229 0.00517 0.03 0.00364 0.00157 0.00518 0.00346 0.0271
2R:1492240-1492390:plus -32.9286 6.31193 -3.51376 4.0367 -17.6735 -9.72449 -19.7156 5.81944 3.05505
0.025 0.000913 0.0333 0.00578 0.00572 0.00352 0.0196 0.00181 0.00692
2R:1496020-1496170:minus -30.8878 8.30275 -2.55046 2.47706 -27.2653 -8.7551 -20.8165 7.375 -2.0367
0.0103 0.000324 0.0254 0.0115 0.0302 0.00172 0.0238 0.000825 0.0336
2R:1496020-1496170:plus -22.1224 1.6055 4.48624 6.52294 -18.4592 -17.2755 -5.91743 -4.18056 -5.95413
0.00304 0.0058 0.0022 0.00174 0.0109 0.00544 0.00128 0.0513 0.088
2R:1496380-1496530:minus -32.8163 2.92661 1.87156 1.57798 -19.051 -8.79592 -26.2294 8.90278 -6.89908
0.024 0.00357 0.00606 0.0171 0.0162 0.0019 0.055 0.000341 0.111
2R:1496380-1496530:plus -20.6633 3.97959 -1.91743 6.02752 -18.4184 -27.6327 -9.59633 -3.79167 -2.41284
0.00173 0.000669 0.0211 0.00246 0.0101 0.043 0.00286 0.0469 0.0376
2R:149860-150010:minus -22.8878 0.174312 4.59633 7.17431 -18.0816 -17.9388 -11.0275 -4.13889 7.22018
0.00441 0.00971 0.0021 0.00115 0.00759 0.00738 0.00375 0.0508 0.00119
2R:149860-150010:plus -31.4796 3.23853 4.52294 3.77064 -18.0816 -27.4898 -16.945 -5.06944 -0.816514
0.0128 0.00318 0.00217 0.00637 0.00759 0.0363 0.0127 0.0626 0.0249
2R:15013480-15013630:minus -21.9286 2.51376 -1.50459 7.17431 -18.3776 0.0510204 -13.3578 -3.29167
-5.66972 0.0027 0.00415 0.0186 0.00115 0.00926 0.000349 0.00628 0.0416 0.0829
2R:15013480-15013630:plus -31.6633 1.50459 -0.220183 3.13761 -18.6735 -8.65306 -21.0367 2.86111
1.41284 0.0141 0.00602 0.0125 0.00854 0.0123 0.00156 0.0248 0.00629 0.0121
2R:15013760-15013910:minus -14.7449 1.07339 13.8807 1.48624 -16.4898 -18.051 -21.1927 7.02778
0.302752 0.00157 0.00705 8.43e-06 0.0177 0.00312 0.00886 0.0255 0.000991 0.0169
2R:15013760-15013910:plus -22.8878 11.3853 1.62385 2.66972 -9.27551 -26.4898 -18.4495 0.611111 3.52294
0.00441 0.000066 0.00663 0.0106 0.00284 0.0225 0.016 0.0138 0.00558
2R:15024660-15024810:minus -23.7143 5.6055 0.880734 4.05505 -18.4898 1.12245 -17 3.23611 -1.05505
0.0067 0.00126 0.00861 0.00566 0.0116 0.000085 0.0128 0.00545 0.0264
2R:15024660-15024810:plus -13.4082 12.3761 15.211 4.3211 -18.3776 -26.5612 -20.5505 -0.208333
-1.68807 0.000867 0.0000356 1.07e-06 0.00514 0.00926 0.0237 0.0227 0.0178 0.0308
2R:15029000-15029150:minus -34.2245 14.4128 -4.59633 5.86239 -18.9388 -8.53061 -22.9266 8.01389
-1.11009 0.0315 5.49e-06 0.0445 0.00282 0.0151 0.00136 0.0347 0.000579 0.0269
2R:15029000-15029150:plus -22.6633 4.57798 5.09174 -0.587156 10.6531 -27.6327 5.41284 -2.55556
4.80734 0.00384 0.00193 0.00171 0.0322 0.0000102 0.043 5e-05 0.0345 0.00334
2R:15029700-15029850:minus -14.4082 1.3211 0.477064 3.07339 -27.3367 -19.0816 -10.6881 -3.34722
11.8532 0.00136 0.00644 0.00989 0.00873 0.0317 0.017 0.00351 0.0421 0.0000428
2R:15029700-15029850:plus -31.2449 1.58716 3.2844 3.47706 -27.2959 -28.602 -3.97248 -2.97222 1.63303
0.0111 0.00584 0.00356 0.00724 0.0303 0.0648 0.00074 0.0384 0.0112
2R:15031860-15032010:minus -24.4082 1.33945 1.07339 7.27523 -28.1633 0.122449 -6.0367 -0.777778
7.42202 0.00797 0.0064 0.00806 0.00109 0.0462 0.000312 0.00132 0.0211 0.00106
2R:15031860-15032010:plus -22.8163 0.899083 -0.220183 2.53211 -17.9796 0.27551 -22.5321 -0.347222

3.95413 0.00418 0.00751 0.0125 0.0113 0.00726 0.000257 0.0324 0.0186 0.00448
2R:15032340-15032490:minus -23.7449 5.88073 1.30275 6.45872 -17.7143 -27.602 -12.7982 2.91667
-5.02752 0.00674 0.00112 0.00743 0.00182 0.00627 0.0416 0.00552 0.00616 0.0732
2R:15032340-15032490:plus -22.4796 0.174312 -1.74312 9.7156 -26.9694 -19.3061 -5.68807 -3.75 -0.394495
0.00352 0.00971 0.02 0.000242 0.023 0.0187 0.0012 0.0464 0.0212
2R:15034980-15035130:minus -24.2245 1.41284 0.59633 -1.55963 -27.2245 -9.79592 -21.4679 1.86111
1.74312 0.00763 0.00623 0.00949 0.0442 0.0272 0.00377 0.0268 0.00905 0.0105
2R:15034980-15035130:plus -33.0408 -1.10092 -0.834862 2.31193 -27.2347 -18.051 -12.8165 1.04167
-0.59633 0.0264 0.0148 0.0152 0.0123 0.0283 0.00886 0.00554 0.012 0.0229
2R:15041840-15041990:minus -31.7041 0.00917431 8.34862 9.75229 0.908163 0.316327 -19.1835 -5.5 -4
0.0146 0.0103 0.000363 0.000226 0.000153 0.000243 0.018 0.0686 0.0558
2R:15041840-15041990:plus -21 -0.541284 2.22018 -1.20183 -18.6837 -8.79592 -13.3028 0.416667 0.605505
0.00186 0.0124 0.00532 0.0392 0.0126 0.0019 0.0062 0.0147 0.0159
2R:15042720-15042870:minus -31.3673 -0.495413 3.52294 5.47706 -16.7143 2.09184 -13.3211 2.34722
7.62385 0.0116 0.0122 0.00324 0.0036 0.00319 0.0000451 0.00622 0.00761 0.000941
2R:15042720-15042870:plus -13.8469 -3.07339 6.78899 7.33028 -17.449 -16.7857 -16.3853 -3.55556 1.89908
0.0011 0.0263 0.000801 0.00103 0.00484 0.00426 0.0116 0.0443 0.0098
2R:15051560-15051710:minus -34.0714 2.77982 4.34862 1.36697 -27.4592 -17.2755 -20.8624 3.5 0.816514
0.031 0.00377 0.00233 0.0186 0.0329 0.00544 0.024 0.00491 0.015
2R:15051560-15051710:plus -3.67347 -1.34862 -2.01835 4.0367 -19.2755 -9.20408 -20.0642 0.152778 5.38532
0.0000972 0.016 0.0217 0.00578 0.0164 0.00225 0.0208 0.016 0.00267
2R:15051880-15052030:minus -32.8571 7.59633 15.7339 7.29358 -27.1939 -9.23469 -19.8991 3.06944
1.55046 0.0244 0.000474 2.45e-07 0.00104 0.0267 0.00238 0.0202 0.00581 0.0115
2R:15051880-15052030:plus -23.4082 -0.899083 1.6789 4.91743 -27.2347 -9.42857 -23 7.125 3.25688
0.00623 0.0139 0.0065 0.00447 0.0283 0.0027 0.0351 0.000942 0.00632
2R:15052680-15052830:minus -24.1429 4.00917 -0.889908 1.31193 -8.86735 -8.65306 -14.3945 6.09722
9.97248 0.0074 0.0024 0.0154 0.0189 0.00173 0.00156 0.00791 0.00158 0.00016
2R:15052680-15052830:plus -13.9898 1.82569 -4.22936 0.293578 -18.0408 -27.6735 -9.57798 0.708333 7.6422
0.00114 0.00535 0.0404 0.0248 0.00735 0.0443 0.00285 0.0134 0.000893
2R:15057540-15057690:minus -21.551 -0.0825688 2.45872 6.59633 -27.9694 -27.4082 -7.22936 1.29167
5.79817 0.00205 0.0106 0.00488 0.00164 0.0437 0.0356 0.00178 0.011 0.00208
2R:15057540-15057690:plus -12.1837 3.64286 5.23853 10.8624 -27.1939 0.316327 -15.6972 -4.83333 -0.357798
0.000365 0.00159 0.00161 0.0000687 0.0267 0.000243 0.0103 0.0595 0.021
2R:15059420-15059570:minus -24.102 7.6055 -3.81651 2.0367 -27.1939 -9.23469 -10.5321 6.70833
5.90826 0.00733 0.000472 0.0361 0.0141 0.0267 0.00238 0.0034 0.00117 0.00198
2R:15059420-15059570:plus -31.7041 -4.09174 0.302752 1.58716 -18.7245 -17.3469 -13.4128 3.98611
-0.0275229 0.0146 0.0347 0.0105 0.017 0.0136 0.0058 0.00635 0.00404 0.0186
2R:15066180-15066330:minus -33.0306 3.21101 -2.7156 3.51376 -27.0408 -8.64286 -22.8073 4.55556
-0.899083 0.026 0.00322 0.0266 0.00709 0.0242 0.00154 0.034 0.00318 0.0254
2R:15066180-15066330:plus -13.9184 8.05505 5.07339 -1.33028 -8.71429 -9.72449 -15.3486 0.0694444
7.51376 0.00112 0.000371 0.00172 0.0409 0.00159 0.00352 0.00961 0.0164 0.000984
2R:15066680-15066830:minus -41.3673 2.27523 0.752294 3.45872 -25.9694 -8.79592 -19.5688 3.125
-0.302752 0.0551 0.00454 0.009 0.00731 0.017 0.0019 0.0191 0.00569 0.0208
2R:15066680-15066830:plus -23.1837 3.7551 1.27523 6.12844 -26.5306 -8.38776 -26.8991 -0.361111
-7.31193 0.00553 0.00122 0.0075 0.00231 0.0185 0.00101 0.0597 0.0187 0.12
2R:15114760-15114910:minus -23.4184 2.82569 -0.93578 6.3578 -28.2653 1.05102 -33.5596 -0.430556
-3.29358 0.00624 0.00371 0.0156 0.00195 0.0537 0.000107 0.14 0.0191 0.0474
2R:15114760-15114910:plus -24.1531 9.04587 2.6789 6.84404 -17.9796 -8.5 -14.9083 1.69444 -2.10092
0.00744 0.000218 0.00449 0.0014 0.00726 0.00126 0.00881 0.0096 0.0342
2R:15116460-15116610:minus -22.0408 -1.62385 4.55963 6.3578 -8.90816 -8.16327 -12.1651 -4.34722
-2.36697 0.00296 0.0174 0.00214 0.00195 0.00192 0.000783 0.00478 0.0533 0.0371
2R:15116460-15116610:plus -21.4796 4.44954 1.50459 9.59633 -8.93878 -17.3469 -3.09174 0.305556 3.29358
0.00202 0.00202 0.00692 0.000275 0.00216 0.0058 0.000582 0.0152 0.00623
2R:15171400-15171550:minus -13.5612 -1.17431 2.74312 2.62385 20.4694 9.7551 -14.7248 5.95833

-6.16514 0.000984 0.0151 0.00438 0.0108 1.65e-07 0.0000198 0.00848 0.00169 0.0926
2R:15171400-15171550:plus -30.7755 -0.440367 0.605505 9.08257 -8.67347 -8.45918 -12.6606 7.18056
-0.440367 0.0102 0.012 0.00946 0.000444 0.00154 0.00111 0.00535 0.000915 0.0216
2R:15177460-15177610:minus -32.6633 3.51376 9.38532 8.98165 -7.89796 -10.0204 -25.2936 5.27778
-2.69725 0.0216 0.00288 0.000203 0.000461 0.000678 0.00399 0.049 0.00232 0.0411
2R:15177460-15177610:plus -21.6939 1.68807 2.6055 7 -18.1531 -17.2755 -13.1284 -1.30556 -3.66972
0.00226 0.00563 0.00462 0.00132 0.00829 0.00544 0.00595 0.0246 0.0514
2R:15179740-15179890:minus -21.7041 0.513761 -0.12844 3.61468 -27.1939 -9.45918 -18.5505 -2.01389
3.21101 0.00231 0.00862 0.0121 0.0068 0.0267 0.00279 0.0163 0.0299 0.00644
2R:15179740-15179890:plus -30.3265 6.44037 -0.146789 3.25688 20.2041 9.60204 -19.1927 0.625
-5.14679 0.0089 0.000858 0.0122 0.00804 3.64e-07 0.0000248 0.018 0.0137 0.0751
2R:15180680-15180830:minus -31.0306 -1 -1.36697 1.65138 -27.3367 -35.3061 -3.07339 7.88889 12.2752
0.0105 0.0143 0.0179 0.0166 0.0317 0.0762 0.000579 0.000621 0.0000141
2R:15180680-15180830:plus -3.63265 4.10092 -4.90826 3.77064 -18.1837 -9.53061 -1 3.66667 -0.761468
0.0000917 0.00231 0.0482 0.00637 0.00835 0.0031 0.000355 0.0046 0.0244
2R:15187520-15187670:minus -21.6633 4.68807 4.38532 6.3578 -18.4592 -17.7143 -17.2385 -5.06944
2.21101 0.00223 0.00184 0.0023 0.00195 0.0109 0.00634 0.0133 0.0626 0.00862
2R:15187520-15187670:plus -22.6939 0.66055 -4.44954 9.51376 -18.4898 -27.5612 -10.4771 -1.61111 -0.192661
0.00388 0.00818 0.0428 0.000292 0.0116 0.0393 0.00337 0.0268 0.0198
2R:15192560-15192710:minus -24.4898 5.10092 6.11009 10.8624 -19.051 -0.0204082 -18.6606 3.59722
1.52294 0.00807 0.00156 0.0011 0.0000687 0.0162 0.000411 0.0165 0.00473 0.0117
2R:15192560-15192710:plus -22.4082 2.09174 2.51376 6.80734 10.6531 -27.3367 -16.055 -5.58333 5.75229
0.00345 0.00485 0.00478 0.00145 0.0000102 0.0347 0.011 0.0698 0.00213
2R:15198200-15198350:minus -31.8469 -2.85321 -0.917431 0.633028 11.3571 9.53061 -16.1284 4.55556
5.0367 0.0162 0.0248 0.0156 0.0229 2.44e-06 0.0000316 0.0111 0.00318 0.00306
2R:15198200-15198350:plus -33.2959 0.46789 0.440367 0.110092 -9.27551 -26.8265 -15.9174 2.20833 4.56881
0.0279 0.00876 0.01 0.0261 0.00284 0.0285 0.0107 0.008 0.00356
2R:15245120-15245270:minus -22.1939 -0.605505 2.65138 2.43119 -8.89796 -8.72449 -20.3578 -0.652778
0.743119 0.00317 0.0126 0.00453 0.0117 0.00189 0.00169 0.0219 0.0204 0.0153
2R:15245120-15245270:plus -22.8469 -0.706422 3.25688 9.08257 -27.1939 -8.79592 -12.8532 11.875
1.17431 0.00424 0.0131 0.0036 0.000444 0.0267 0.0019 0.00559 0.0000349 0.0134
2R:15245680-15245830:minus -13.6327 5.49541 3.41284 5.22936 -28.3061 0.244898 -22.1101 4.15278
0.110092 0.00101 0.00132 0.00338 0.0041 0.0553 0.000285 0.0301 0.00377 0.018
2R:15245680-15245830:plus 24.7755 1.86239 0.577982 8.88073 -28 -9.64286 -4.63303 -0.361111 3.90826
9.35e-09 0.00528 0.00955 0.000479 0.0444 0.00339 0.000894 0.0187 0.00462
2R:15258660-15258810:minus -30.6939 0.412844 0.853211 1.80734 -26.9694 -9.53061 -11.8807 -0.305556
3.21101 0.00979 0.00893 0.0087 0.0156 0.023 0.0031 0.00449 0.0184 0.00644
2R:15258660-15258810:plus -32.7347 -0.651376 -1.69725 2.34862 -18.4184 0.826531 -13.211 4.125
2.53211 0.0225 0.0128 0.0197 0.0121 0.0101 0.000153 0.00607 0.00382 0.00791
2R:15260160-15260310:minus -4.37755 -1.16514 3.11927 4.11927 -26.898 0.602041 -19.9633 1.40278
-4.0367 0.000144 0.0151 0.00379 0.00551 0.0203 0.000188 0.0205 0.0106 0.0562
2R:15260160-15260310:plus -21.7041 -0.174312 -0.779817 3.74312 -17.1531 -26.898 -8.7156 4.69444
4.66055 0.00231 0.0109 0.0149 0.00651 0.00364 0.0302 0.00242 0.003 0.0035
2R:15260660-15260810:minus -21.7755 1.45872 0.706422 4.50459 -17.3776 -28.3367 -8.20183 -6.27778
4.02752 0.00241 0.00613 0.00914 0.00484 0.00419 0.0606 0.00219 0.0805 0.00428
2R:15260660-15260810:plus -22.0408 5.78899 5.14679 6.47706 -7.56122 -27.2653 -8.11927 -0.541667
3.94495 0.00296 0.00117 0.00167 0.00179 0.000419 0.0333 0.00216 0.0197 0.00452
2R:15267900-15268050:minus -31.6327 7.50459 2.17431 1.69725 -26.898 -17.9796 -18.1284 1 -1.44037
0.0138 0.000498 0.00542 0.0163 0.0203 0.00764 0.0152 0.0121 0.029
2R:15267900-15268050:plus -22.6633 -3.20183 5.13761 10.7615 -17.7551 -17.9796 -10.1927 -1.75 2.25688
0.00384 0.0273 0.00168 0.0000826 0.00658 0.00764 0.00319 0.0279 0.00853
2R:15277480-15277630:minus -23.4898 1.70642 4.7156 1.21101 -18.4592 -9.02041 -24.422 4.02778
-2.47706 0.00639 0.00559 0.002 0.0194 0.0109 0.00213 0.0436 0.00397 0.0385
2R:15277480-15277630:plus -33.4082 1.98165 12.2752 0.201835 -27.1939 -17.5 -4.77064 -3.26389 1.48624

0.0283 0.00505 0.0000353 0.0255 0.0267 0.00599 0.00093 0.0413 0.0118
2R:15282600-15282750:minus -33.2245 -0.266055 3.21101 6.84404 -17.4184 -17.2755 -22.2477 4.47222
-3.0367 0.0276 0.0113 0.00366 0.0014 0.00457 0.00544 0.0308 0.0033 0.045
2R:15282600-15282750:plus -13.1837 2.61468 3.76147 3.97248 -18.6837 -26.5612 -7.77064 -2 -2.3211
0.000705 0.004 0.00295 0.00587 0.0126 0.0237 0.00201 0.0298 0.0365
2R:15283220-15283470:minus -32.6224 3.3211 3.86239 6.05505 -16.449 -8.57143 -5.92661 -1.16667
7.62385 0.0211 0.00309 0.00283 0.0024 0.00307 0.00149 0.00129 0.0237 0.000941
2R:15283220-15283470:plus -22.0816 -1.53211 4.99083 6.47706 -18.1531 -18.7857 -20.0367 -5.45833 3.09174
0.003 0.0169 0.00178 0.00179 0.00829 0.0146 0.0207 0.068 0.00678
2R:15283620-15283770:minus -32.6224 -1.02752 3.86239 6.05505 -16.449 -19.0102 -5.92661 -1.95833
7.62385 0.0211 0.0145 0.00283 0.0024 0.00307 0.0159 0.00129 0.0295 0.000941
2R:15283620-15283770:plus -30.4898 0.917431 14.8532 3.11009 -18.7551 -8.72449 -25.633 5.16667 -2.97248
0.00941 0.00746 2.28e-06 0.00862 0.0144 0.00169 0.0511 0.00244 0.0443
2R:15284120-15284270:minus -40.1837 -2.44954 0.568807 6.69725 -8.67347 -19.1531 -12.4587 3.375
1.38532 0.0367 0.0221 0.00958 0.00156 0.00154 0.0177 0.00511 0.00516 0.0123
2R:15284120-15284270:plus -34.0714 4.24771 9.25688 4.17431 -17.3776 -19.1531 -18.2477 6.86111 -0.504587
0.031 0.00219 0.000218 0.00537 0.00419 0.0177 0.0155 0.00108 0.0221
2R:15301400-15301550:minus -31.3265 -4.42202 0.376147 6.74312 -18.9796 -18.2755 -18.5046 3.86111
3.66972 0.0114 0.0379 0.0102 0.0015 0.0155 0.0112 0.0161 0.00425 0.00514
2R:15301400-15301550:plus -32 -3.82569 1.44954 9.59633 -17.1531 -9.45918 -13.2752 1.63889 0.12844
0.0171 0.0323 0.00705 0.000275 0.00364 0.00279 0.00616 0.00978 0.0179
2R:15301980-15302130:minus -22.1939 -2.48624 -0.293578 7.17431 -18.6837 -18.051 -18.7339 1.34722
0.733945 0.00317 0.0223 0.0128 0.00115 0.0126 0.00886 0.0167 0.0108 0.0153
2R:15301980-15302130:plus -3.29592 -4.21101 6.6789 7.6422 -18.1531 19.3061 -14.0459 1.18056 7.82569
0.0000549 0.0358 0.000844 0.000721 0.00829 5.97e-07 0.00733 0.0114 0.000728
2R:15306480-15306630:minus -24.1122 5.88991 7.68807 3.41284 -9.16327 -27.1939 -17.7064 1.34722
6.12844 0.00738 0.00111 0.000516 0.00756 0.00249 0.0327 0.0143 0.0108 0.00174
2R:15306480-15306630:plus -31.8163 8.93578 2.55046 2.41284 -27.7449 -18.051 -18.8624 1.54167 4.92661
0.016 0.000231 0.00471 0.0118 0.0383 0.00886 0.0171 0.0101 0.00318
2R:15307100-15307250:minus -23.6327 6.09174 -0.100917 1.55963 -27.602 -18.1224 -13.5321 5.55556
6.11927 0.00661 0.00101 0.012 0.0173 0.0364 0.00988 0.00653 0.00204 0.00178
2R:15307100-15307250:plus -34 8.42202 7.80734 3.27523 -28.1939 -9.30612 -20.9541 7.44444 7.31193
0.0307 0.000304 0.000485 0.00796 0.0482 0.00257 0.0244 0.000795 0.00112
2R:15309600-15309750:minus -21.8163 1.29358 4.6789 7.27523 -18.449 -8.72449 -22.8257 -4.22222
-3.86239 0.00244 0.00651 0.00203 0.00109 0.0107 0.00169 0.0341 0.0518 0.0535
2R:15309600-15309750:plus -32.8163 5.33945 -1.48624 1.97248 -27.4592 -17.4184 -8.78899 9.05556 1.95413
0.024 0.00142 0.0185 0.0146 0.0329 0.00585 0.00246 0.000309 0.00964
2R:15311040-15311190:minus -31.551 -0.201835 1.3945 1.9633 -18.2245 0.979592 -18.0275 4.52778
1.74312 0.0134 0.011 0.00719 0.0146 0.00877 0.000136 0.015 0.00322 0.0105
2R:15311040-15311190:plus -21.9592 -2.15596 -0.678899 0.220183 11.6939 -18.3061 1.62385 2.93056
5.25688 0.00279 0.0203 0.0144 0.0253 1.17e-06 0.0114 0.000185 0.00612 0.0028
2R:15312400-15312550:minus -23.5204 9.51376 -0.211009 1.47706 -18.4184 -18.7857 -2.6789 -2.02778
2.74312 0.00645 0.000171 0.0124 0.0178 0.0101 0.0146 0.000523 0.03 0.00751
2R:15312400-15312550:plus -22.0408 9.69725 6.81651 5.86239 -18.4592 -8.64286 -17.0642 5.38889 1.22936
0.00296 0.000155 0.000791 0.00282 0.0109 0.00154 0.0129 0.0022 0.0132
2R:15313020-15313170:minus -24.0714 5.88991 -3.11927 3.13761 -18.7143 -9.08163 -15.8349 6.625
-0.366972 0.00731 0.00111 0.0298 0.00854 0.0136 0.00216 0.0105 0.00122 0.021
2R:15313020-15313170:plus -14.4796 -0.651376 2 0.119266 -27.0408 -9.09184 -4.04587 11.2917 2.06422
0.00142 0.0128 0.00578 0.026 0.0242 0.00219 0.000756 0.0000594 0.00912
2R:15324700-15324850:minus -23.2653 -2.21101 5.13761 6.27523 20.4694 -8.86735 -16.1193 -1.47222
0.862385 0.00593 0.0206 0.00168 0.00206 1.65e-07 0.00198 0.0111 0.0258 0.0148
2R:15324700-15324850:plus -24.0306 13.578 0.366972 4.05505 -18.7857 -9.5 -10.6147 0.0416667 5.81651
0.00723 0.0000133 0.0103 0.00566 0.0148 0.00294 0.00346 0.0165 0.00204
2R:15329820-15329970:minus -29.5102 2.0367 0.486239 5.49541 -26.7449 -8.5 -10.7982 5.47222 2.04587

0.00845 0.00495 0.00986 0.00355 0.0195 0.00126 0.00359 0.00212 0.00919
2R:15329820-15329970:plus -23.102 -1.50459 0.385321 2.98165 10.6224 -8.5 -26.4037 1.65278 1.25688
0.00522 0.0168 0.0102 0.00905 0.0000113 0.00126 0.0562 0.00974 0.0131
2R:15333740-15333890:minus -4.11224 4.41284 -0.0366972 -4.08257 -7.64286 -17.051 -8.82569 -4.20833
-1.93578 0.000126 0.00205 0.0117 0.086 0.000499 0.00479 0.00248 0.0517 0.0326
2R:15333740-15333890:plus -30.5204 13.3878 0.211009 7.43119 0.877551 -18.2347 -4.75229 -4.08333 1.16514
0.00951 0.0000869 0.0108 0.000939 0.00019 0.0104 0.000925 0.0502 0.0135
2R:15334120-15334410:minus -41.8163 -0.972477 -3.22936 3.87156 -27.4898 -9.57143 -28.3028 4.31944
-4.86239 0.0662 0.0142 0.0307 0.00618 0.0336 0.00326 0.0717 0.00352 0.0705
2R:15334120-15334410:plus -33 -1.04587 -1.0367 3.49541 -26.4898 -8.7551 -14.4495 5.97222 0.550459
0.0258 0.0145 0.0161 0.00716 0.0183 0.00172 0.008 0.00168 0.0161
2R:15337300-15337450:minus -13.1735 -1.55963 7.86239 6.21101 -18.3776 -9.0102 -10.4587 5.31944
-0.0183486 0.000671 0.017 0.000471 0.00213 0.00926 0.00208 0.00336 0.00227 0.0185
2R:15337300-15337450:plus -23.0816 2.79817 -3.06422 2.6789 -8.67347 -18.2347 -21.9817 0.0833333 -2
0.00522 0.00374 0.0294 0.0105 0.00154 0.0104 0.0294 0.0163 0.0332
2R:15338200-15338350:minus -23.9286 5.53211 6.46789 2.74312 -18.3776 -18.2347 -18.4128 2.875
-4.70642 0.00701 0.0013 0.000933 0.0101 0.00926 0.0104 0.0159 0.00626 0.0676
2R:15338200-15338350:plus -39.949 -2.08257 1.88073 0.623853 -7.63265 -19.0102 -17.6606 5.625 -0.495413
0.0345 0.0199 0.00604 0.0229 0.000479 0.0159 0.0142 0.00198 0.022
2R:15338680-15338830:minus -31.398 -1.50459 2.90826 3.18349 -27.1939 -18.5714 -21.9725 -1.73611
2.88991 0.0117 0.0168 0.00411 0.00837 0.0267 0.0136 0.0293 0.0277 0.00725
2R:15338680-15338830:plus -32.3673 -0.458716 4.77982 1.44037 -17.7551 -18.051 -20.2294 2.70833
-0.0733945 0.0189 0.012 0.00195 0.018 0.00658 0.00886 0.0214 0.00666 0.0188
2R:15339000-15339150:minus -21 0.53211 2.69725 1.3945 -18.1939 -9.7551 -7.33028 15.8889 5.22936
0.00186 0.00856 0.00446 0.0184 0.0086 0.00358 0.00182 4.22e-08 0.00288
2R:15339000-15339150:plus -23.6735 -2.34862 -0.0917431 1.24771 0.540816 -18.2755 -4.88991 5.73611
0.963303 0.00665 0.0215 0.0119 0.0192 0.000321 0.0112 0.000962 0.00188 0.0145
2R:15339900-15340050:minus -22.8163 -3.56881 5.51376 2.23853 -7.93878 0.826531 -15.0734 -2.38889
7.49541 0.00418 0.0301 0.00143 0.0128 0.000767 0.000153 0.00911 0.0331 0.000998
2R:15339900-15340050:plus -4.59184 8.05505 2.89908 -0.256881 -18.3776 -8.5 -12.9908 -0.458333
-0.623853 0.000166 0.000371 0.00413 0.0289 0.00926 0.00126 0.00577 0.0192 0.0232
2R:15343540-15343690:minus -30.7449 1.77064 0.880734 1.65138 -17.5612 -18.3469 -24.9541 2.08333
-1.99083 0.0101 0.00546 0.00861 0.0166 0.00544 0.0124 0.0469 0.00837 0.0331
2R:15343540-15343690:plus -23.2245 1.23853 6.84404 3.69725 0.836735 -8.72449 -16.2018 1.09722 5.9633
0.00576 0.00664 0.000781 0.00665 0.000215 0.00169 0.0112 0.0118 0.00188
2R:15344580-15344730:minus -32.5816 5.36697 2.7156 3.18349 -17.6429 -19.2041 4.73394 -1.09722
3.31193 0.0208 0.0014 0.00442 0.00837 0.00562 0.0179 0.0000633 0.0232 0.00618
2R:15344580-15344730:plus -11.9592 -3.17431 -0.412844 6.84404 -25.9286 -17.3469 -24.4679 2.40278
1.74312 0.000301 0.027 0.0133 0.0014 0.0168 0.0058 0.0439 0.00746 0.0105
2R:15349020-15349170:minus -22.6633 2.68807 10 7.2844 -26.4898 -8.79592 -16.2477 5.58333 -2.72477
0.00384 0.0039 0.000142 0.00105 0.0183 0.0019 0.0113 0.00202 0.0414
2R:15349020-15349170:plus -31.898 -1.52294 0.211009 1.16514 -17.7143 -7.42857 -26.0459 1.20833 -8.29358
0.0164 0.0169 0.0108 0.0197 0.00627 0.000567 0.0538 0.0113 0.144
2R:15349880-15350030:minus -22.4796 -1.91743 -3.36697 1.91743 -18.6837 1.05102 -18.2385 -0.666667
7.83486 0.00352 0.019 0.032 0.015 0.0126 0.000107 0.0155 0.0205 0.000712
2R:15349880-15350030:plus -13 3.26606 1.86239 7.27523 -7.86735 -9.79592 -22.2844 4 -0.688073
0.000563 0.00315 0.00608 0.00109 0.000617 0.00377 0.031 0.00402 0.0238
2R:15350280-15350430:minus -31.4388 -1.25688 3.22018 2.9633 10.3878 0.867347 -27.3853 2.09722
-6.98165 0.0122 0.0155 0.00365 0.00914 0.0000174 0.000144 0.0635 0.00832 0.113
2R:15350280-15350430:plus -39.9898 -1.48624 0.853211 3.26606 -7.85714 -25.5306 -9.80734 3.86111 -0.59633
0.0351 0.0167 0.0087 0.00802 0.000594 0.0205 0.00297 0.00425 0.0229
2R:15351000-15351150:minus -22.0306 0.422018 3.81651 5.01835 -8.92857 0.0510204 -14.3578 -4.68056
0.12844 0.00293 0.0089 0.00289 0.00438 0.00194 0.000349 0.00785 0.0575 0.0179
2R:15351000-15351150:plus -21.9184 0.0366972 -1.34862 1.22936 -17.8878 -17.0102 -14.5596 -1.73611

0.12844 0.00261 0.0102 0.0178 0.0193 0.00704 0.00453 0.00819 0.0277 0.0179
2R:15354760-15354910:minus -30.4796 -1.12844 4.19266 1.10092 -17.4184 -9.72449 -3.56881 -6.02778
5.81651 0.00941 0.0149 0.00248 0.02 0.00457 0.00352 0.000661 0.0765 0.00204
2R:15354760-15354910:plus -14.5204 3.90816 5.30275 7 -17.0408 -8.38776 -24.1009 -6.84722 -0.0825688
0.00145 0.00102 0.00157 0.00132 0.00329 0.00101 0.0417 0.0899 0.0189
2R:15355380-15355530:minus -13.551 0.981651 -1.44954 2.53211 -17.1837 -17.3469 -9.12844 1.22222
5.18349 0.000974 0.00729 0.0183 0.0113 0.00366 0.0058 0.00262 0.0113 0.00295
2R:15355380-15355530:plus -22.7857 -3.33945 -2.65138 0.284404 -18.4796 -37 -19.4679 3.02778 11.8532
0.00412 0.0283 0.0261 0.0249 0.011 0.124 0.0188 0.0059 0.0000428
2R:15355700-15355850:minus -31.5102 3.27523 -1.78899 0.733945 -18.6837 0.0918367 -21.1651 1.48611
-4.24771 0.013 0.00314 0.0203 0.0222 0.0126 0.000323 0.0253 0.0103 0.0591
2R:15355700-15355850:plus -13.0714 -0.376147 2.78899 2.81651 -18.1531 -7.5 -21.3028 -1.44444 5.25688
0.000594 0.0117 0.0043 0.00982 0.00829 0.000614 0.026 0.0256 0.0028
2R:15356360-15356510:minus -30.6735 1.84404 4.79817 6.3578 -17.6429 1.16327 -27.4037 -2.88889
-4.52294 0.00978 0.00532 0.00193 0.00195 0.00562 0.0000831 0.0636 0.0376 0.0642
2R:15356360-15356510:plus -22.0408 -4.40367 2.17431 2.7156 -18.449 -26.2653 -14.1193 -5.5 4.82569
0.00296 0.0377 0.00542 0.0102 0.0107 0.0218 0.00745 0.0686 0.00331
2R:15374300-15374450:minus -21.1837 7.11927 0.256881 5.02752 -18.1224 -0.173469 -13.4954 -4.34722
3.05505 0.00189 0.000611 0.0106 0.00435 0.00782 0.000456 0.00648 0.0533 0.00692
2R:15374300-15374450:plus -41.4796 0.816514 -0.568807 1.49541 -18.7449 -9.86735 -23.8716 6.75 -4.26606
0.0577 0.00773 0.0139 0.0176 0.0138 0.00391 0.0403 0.00114 0.0595
2R:15379560-15379710:minus -23.3265 0.137615 3.78899 1.72477 -27.1939 -18.051 -14.5596 7.09722
3.47706 0.00607 0.00984 0.00292 0.0161 0.0267 0.00886 0.00819 0.000956 0.00575
2R:15379560-15379710:plus -13.8776 6.49541 16.3945 0.0550459 -27.4898 -8.86735 -13.4587 6.25 -3.82569
0.0011 0.000837 6.75e-08 0.0265 0.0336 0.00198 0.00642 0.00147 0.0531
2R:15384300-15384450:minus -13.449 5.95413 1.72477 0.816514 -18.1531 -27.3061 -15.055 3.5 3.92661
0.000914 0.00108 0.0064 0.0216 0.00829 0.0336 0.00907 0.00491 0.00461
2R:15384300-15384450:plus -31.551 -6.23853 4.23853 1.21101 -18.7857 -27.9694 -10.5596 1.125 -0.972477
0.0134 0.0606 0.00243 0.0194 0.0148 0.0547 0.00342 0.0117 0.0259
2R:15390440-15390590:minus -33 4.34862 2.9633 5.22936 -27.5 -8.72449 -18.945 1.52778 -6.82569
0.0258 0.0021 0.00403 0.0041 0.0337 0.00169 0.0173 0.0102 0.109
2R:15390440-15390590:plus -32.2857 0.146789 6.11009 1.21101 -18.4898 -19.051 -19 -1.91667 3.88991
0.0185 0.0098 0.0011 0.0194 0.0116 0.0165 0.0175 0.0291 0.00467
2R:1540260-1540410:minus -32.9286 -2.06422 2.6789 6.27523 20.4694 0.897959 -14.156 -3.81944 -3.51376
0.025 0.0198 0.00449 0.00206 1.65e-07 0.000144 0.00751 0.0472 0.0498
2R:1540260-1540410:plus -32 0.495413 -1.20183 6.21101 -27.4898 -16.3469 -13.578 4.59722 -2.42202
0.0171 0.00868 0.017 0.00213 0.0336 0.00416 0.0066 0.00313 0.0378
2R:1550700-1550850:minus -24.1531 3.30275 -1.87156 0.788991 -18.3878 0.0918367 -10.4037 8.15278
8.11009 0.00744 0.00311 0.0208 0.0218 0.00931 0.000323 0.00332 0.000535 0.000597
2R:1550700-1550850:plus -40.5204 7.87156 -3.68807 3.18349 -9.19388 -17.5 -25.2385 0.444444 -2.85321
0.0413 0.000409 0.0349 0.00837 0.00253 0.00599 0.0486 0.0145 0.0429
2R:1551400-1551550:minus -14.5102 1.16514 1.00917 4.05505 -26.9286 -9.30612 -4.66055 1.5 -0.972477
0.00143 0.00682 0.00824 0.00566 0.0214 0.00257 0.000901 0.0103 0.0259
2R:1551400-1551550:plus -40.5918 3.22018 -1.69725 1.36697 -19.0204 -9.79592 -25.4587 8.13889 -2.90826
0.0422 0.00321 0.0197 0.0186 0.0158 0.00377 0.05 0.000539 0.0435
2R:15556340-15556490:minus -31.1735 -1.24771 -3.88991 2.66972 -27.1531 -7.72449 -21.4495 11.6944
1.25688 0.0109 0.0155 0.0369 0.0106 0.0249 0.00068 0.0267 0.0000414 0.0131
2R:15556340-15556490:plus -33.5204 -0.825688 9.93578 3.90826 10.6531 -27.6735 -23.1927 8.16667
1.74312 0.0287 0.0136 0.000148 0.00603 0.0000102 0.0443 0.0362 0.00053 0.0105
2R:15556940-15557090:minus -40.7653 1.50459 0.146789 4.91743 -8.44898 -18.2755 -16.4679 4.61111
3.30275 0.0432 0.00602 0.011 0.00447 0.00109 0.0112 0.0118 0.00311 0.00619
2R:15556940-15557090:plus -23.9184 5.73394 2.16514 4.74312 -8.40816 -27.5612 -17.7431 5.59722 0.495413
0.00695 0.00119 0.00544 0.00462 0.00108 0.0393 0.0144 0.002 0.0162
2R:15557980-15558130:minus -33.3367 0.321101 -2.01835 1.3578 -18.7143 -9.02041 -26.7982 5.04167

-0.192661 0.0281 0.00922 0.0217 0.0186 0.0136 0.00213 0.059 0.00258 0.0198
2R:15557980-15558130:plus -33.2959 8.77064 13.9174 2.62385 -18.1224 -8.38776 -6.74312 4.54167 -1.21101
0.0279 0.000253 8.08e-06 0.0108 0.00782 0.00101 0.00159 0.0032 0.0277
2R:15561180-15561330:minus -40.449 1.08257 5.65138 -1.85321 -18.7857 -26.8265 -14.9266 0.125
0.614679 0.0402 0.00703 0.00135 0.0486 0.0148 0.0285 0.00884 0.0161 0.0158
2R:15561180-15561330:plus -32.0816 0.220183 3.22018 7.17431 -27.1531 -17.2755 -14.4495 2.47222 -0.477064
0.0176 0.00956 0.00365 0.00115 0.0249 0.00544 0.008 0.00727 0.0218
2R:1559160-1559310:minus -31.8163 -1.2844 7.08257 3.04587 -26.5306 -18.898 -2.41284 10.3056 5.23853
0.016 0.0157 0.000699 0.00883 0.0185 0.0152 0.00049 0.000131 0.00285
2R:1559160-1559310:plus -31.0714 -0.137615 1.52294 5.22018 11.6939 -8.57143 -2.04587 6.98611
5.69725 0.0106 0.0108 0.00687 0.00418 1.17e-06 0.00149 0.000449 0.00101 0.00225
2R:15614520-15614670:minus -31.0408 1.89908 5.21101 6.08257 -8.64286 -9.0102 -21.1651 1.22222
0.266055 0.0106 0.00521 0.00163 0.00236 0.00139 0.00208 0.0253 0.0113 0.0171
2R:15614520-15614670:plus -32.9286 -2.53211 1.66972 5.22936 -17.7143 -9.53061 -15.2844 -2.05556 2.85321
0.025 0.0226 0.00652 0.0041 0.00627 0.0031 0.00949 0.0303 0.00733
2R:1564620-1564770:minus -31.4388 3.43119 2.86239 9.33028 -27.2653 -16.051 -22.0459 2.375 -0.550459
0.0122 0.00297 0.00418 0.000374 0.0302 0.00408 0.0297 0.00753 0.0225
2R:1564620-1564770:plus -42.5918 1.23853 -1.34862 3.44954 -18.7143 -18.0816 -11.5596 -0.958333
1.37615 0.0819 0.00664 0.0178 0.00738 0.0136 0.00934 0.00419 0.0223 0.0123
2R:15713860-15714010:minus -24.898 1.37615 -0.669725 5.29358 -18.4898 -8.5 -7.58716 -2.18056
5.90826 0.00831 0.00631 0.0144 0.00398 0.0116 0.00126 0.00193 0.0313 0.00198
2R:15713860-15714010:plus -12.0408 -2.37615 4.40367 9.3211 -18.4184 -17.7857 -1.85321 -3.65278 2.11009
0.000311 0.0216 0.00228 0.000391 0.0101 0.0068 0.000429 0.0454 0.00898
2R:1579300-1579450:minus -23.2245 1.54128 1.66972 6.2844 -17.4898 -26.6735 -12.4862 7.80556 -0.862385
0.00576 0.00594 0.00652 0.00205 0.0053 0.0256 0.00514 0.000651 0.0251
2R:1579300-1579450:plus -12.1531 -4.87156 0.669725 7.95413 -18.7143 -26.4898 -14.5321 -4.55556 2.6789
0.000352 0.0427 0.00926 0.000599 0.0136 0.0225 0.00814 0.0559 0.00763
2R:1580840-1580990:minus -23.4898 13.5306 -0.513761 2.50459 -17.7857 -18.9388 -19.7982 -0.0555556
1.36697 0.00639 0.0000206 0.0137 0.0114 0.00676 0.0154 0.0199 0.017 0.0124
2R:1580840-1580990:plus -21.9286 3.51376 4.94495 1.63303 -9.23469 -26.3061 -7.90826 1.69444 5.44954
0.0027 0.00288 0.00182 0.0167 0.00277 0.0219 0.00206 0.0096 0.00253
2R:158200-158350:minus -32.7449 2.29358 0.954128 0.633028 -7.86735 -17.3469 -18.4679 2.11111 -5.59633
0.0228 0.00451 0.0084 0.0229 0.000617 0.0058 0.0161 0.00828 0.0819
2R:158200-158350:plus -22.6633 -6.3578 2.88991 4.33028 -17.3776 -36.1122 -16.3578 6.44444 8.11009
0.00384 0.0625 0.00414 0.00511 0.00419 0.0878 0.0115 0.00133 0.000597
2R:158420-158570:minus -23.2959 3.06422 4.55963 7.47706 -17.7551 -0.0204082 -19.1927 -0.194444
7.6422 0.00605 0.0034 0.00214 0.00085 0.00658 0.000411 0.018 0.0178 0.000893
2R:158420-158570:plus -31.7041 0.284404 6.87156 1.05505 -8.0102 -19.2755 -4.46789 3.51389 -0.495413
0.0146 0.00935 0.000772 0.0204 0.000826 0.0185 0.000852 0.00489 0.022
2R:1585240-1585390:minus -33.9286 3.64286 8.85321 3.27523 -19.051 -8.5 -17.0826 3.88889 -4.76147
0.0303 0.00159 0.000275 0.00796 0.0162 0.00126 0.013 0.00421 0.0686
2R:1585240-1585390:plus -31.2143 -0.12844 7.68807 4.22018 20.1735 -7.79592 -11.0734 8.61111 5.22936
0.0111 0.0108 0.000516 0.00531 5.68e-07 0.000723 0.00379 0.000408 0.00288
2R:1589460-1589610:minus -30.5918 7.3578 0.311927 9.44037 -17.4898 -8.23469 -17.7339 3.5 0.183486
0.00963 0.000538 0.0104 0.000339 0.0053 0.000875 0.0144 0.00491 0.0176
2R:1589460-1589610:plus -11.9592 0.110092 8.77982 9.33028 -9.0102 -8.45918 1.13761 3.47222 -0.458716
0.000301 0.00993 0.000286 0.000374 0.00236 0.00111 0.000212 0.00497 0.0217
2R:15971720-15971870:minus -31.2551 0.211009 5.84404 7.73394 -8.64286 -18.0816 -12.5138 0.902778
-0.963303 0.0113 0.00959 0.00124 0.000669 0.00139 0.00934 0.00517 0.0125 0.0258
2R:15971720-15971870:plus -31.7755 -2.01835 3.33945 4.55046 0.581633 0.173469 -13.4862 3.51389 -2.70642
0.0157 0.0195 0.00348 0.00482 0.000292 0.000307 0.00646 0.00489 0.0412
2R:16027220-16027370:minus -21.7347 2.41284 2.82569 5.76147 -17.8265 -27.3367 -5.80734 6.94444
3.26606 0.00235 0.00431 0.00424 0.00295 0.00691 0.0347 0.00124 0.00103 0.00627
2R:16027220-16027370:plus -12.9592 2.09174 10.156 9.44037 -17.8878 -8.57143 -5.36697 0.0277778

9.77064 0.000551 0.00485 0.00013 0.000339 0.00704 0.00149 0.0011 0.0166 0.000228
2R:16133000-16133150:minus -13.1837 11.0459 2.59633 2.9633 -17.1531 -8.94898 -6.41284 -1.08333
3.46789 0.000705 0.0000789 0.00463 0.00914 0.00364 0.00203 0.00146 0.0231 0.00582
2R:16133000-16133150:plus -33 1.50459 -2.94495 5.43119 -18.7551 -26.602 -16.2385 0.791667 3.29358
0.0258 0.00602 0.0284 0.00371 0.0144 0.0248 0.0113 0.013 0.00623
2R:1614420-1614570:minus -32.9592 -0.100917 6.53211 7.55963 -8.33673 -9.20408 -0.889908 -1.93056
0.12844 0.0252 0.0107 0.000904 0.000807 0.00101 0.00225 0.000346 0.0293 0.0179
2R:1614420-1614570:plus -21.3265 -0.770642 12.789 8.6055 -17.0408 -17.8571 -15.7615 -3.31944
9.97248 0.00193 0.0133 0.0000248 0.000567 0.00329 0.00719 0.0104 0.0418 0.00016
2R:16144840-16144990:minus -29.8776 -1.62385 1.30275 3.47706 20.2041 9.79592 -16.1101 6.36111
5.48624 0.00852 0.0174 0.00743 0.00724 3.64e-07 0.0000183 0.0111 0.00139 0.00251
2R:16144840-16144990:plus -31.4388 3.97959 -1.3578 5.57798 20.4694 -26.9694 -15.6606 4.27778 -4.33945
0.0122 0.000669 0.0178 0.0034 1.65e-07 0.0304 0.0102 0.00358 0.0609
2R:16145120-16145270:minus -29.8776 -1.66972 0.0917431 3.47706 20.2041 9.79592 -19.1835 6.875
1.78899 0.00852 0.0176 0.0112 0.00724 3.64e-07 0.0000183 0.018 0.00107 0.0103
2R:16145120-16145270:plus -22.7041 3.97959 -1.99083 5.57798 -18.5306 -18.3469 -18.4587 4.27778 -3
0.00395 0.000669 0.0216 0.0034 0.0118 0.0124 0.016 0.00358 0.0447
2R:16150300-16150450:minus -40.2551 -4.99083 6.84404 5.22936 0.540816 -19.1224 -17.8807 2.41667
-0.486239 0.0382 0.044 0.000781 0.0041 0.000321 0.0175 0.0147 0.00742 0.0219
2R:16150300-16150450:plus -23.449 15.4037 7.53211 2.55963 1.65306 -9.68367 -14.211 -3.04167 3.6055
0.00631 1.16e-06 0.000558 0.0111 0.0000586 0.00342 0.0076 0.0391 0.00532
2R:16151520-16151670:minus -31.9286 1.63303 1.99083 1.12844 -17.7143 -17.1224 -6.17431 6.05556
1.53211 0.0166 0.00575 0.0058 0.0198 0.00627 0.00499 0.00137 0.00161 0.0116
2R:16151520-16151670:plus -31.1837 -1.17431 2.87156 2.88073 -18.9796 -18.1633 -22.5596 -1.625 -1.80734
0.0109 0.0151 0.00417 0.00945 0.0155 0.01 0.0326 0.0269 0.0316
2R:16157720-16157870:minus -22.5612 7.69725 2.09174 3.76147 -19.0102 -9.64286 -6.22936 -0.888889
-2.26606 0.0036 0.00045 0.00558 0.00645 0.0157 0.00339 0.00139 0.0218 0.0359
2R:16157720-16157870:plus -41.2551 1.18349 -3.48624 0.798165 -8.67347 0.0510204 -15.3578 9.79167
0.550459 0.0513 0.00677 0.033 0.0218 0.00154 0.000349 0.00963 0.00019 0.0161
2R:16158600-16158750:minus -13.2143 -1.11009 10.9174 5.57798 -26.9694 -9.5 -6.2844 -2.59722 0.559633
0.000714 0.0148 0.000084 0.0034 0.023 0.00294 0.00141 0.0349 0.016
2R:16158600-16158750:plus -23.0714 2.23853 -0.0917431 2.59633 -27.1633 -17.2755 -10.1468 0.819444
2.10092 0.0052 0.0046 0.0119 0.0109 0.025 0.00544 0.00316 0.0129 0.00901
2R:16162000-16162150:minus -21.6224 -3.21101 12.6789 8.81651 -6.67347 -18.0102 -15.1284 5 5.00917
0.00213 0.0273 0.0000266 0.000501 0.000359 0.00812 0.00921 0.00263 0.0031
2R:16162000-16162150:plus -32.7449 2.33028 0.183486 -1.30275 10.4286 0.0510204 -14.1376 -3.47222
1.63303 0.0228 0.00445 0.0109 0.0404 0.0000139 0.000349 0.00748 0.0434 0.0112
2R:16166420-16166570:minus -23.2959 -2.65138 12.8716 4.14679 -27.9286 -17.7143 -13.2018 2.65278
5.90826 0.00605 0.0234 0.0000231 0.00542 0.0412 0.00634 0.00605 0.0068 0.00198
2R:16166420-16166570:plus -31.5204 -1.57798 14.1835 7.45872 -9.20408 -9.23469 -10.2018 -1.68056 2.41284
0.0131 0.0171 5.88e-06 0.000894 0.00262 0.00238 0.0032 0.0273 0.0082
2R:16196240-16196390:minus -40.2551 13.3878 3.30275 5.69725 -18.4592 -8.45918 -16.2752 0.833333
4.14679 0.0382 0.0000869 0.00353 0.00314 0.0109 0.00111 0.0114 0.0128 0.00406
2R:16196240-16196390:plus -43.0102 9.44037 -3.77982 1.84404 -26.9286 -9.94898 -23.8899 1.61111 -5.25688
0.0889 0.000178 0.0358 0.0154 0.0214 0.00393 0.0404 0.00988 0.0768
2R:16199800-16199950:minus -23 -4 5.6789 -0.110092 -8.64286 -27.602 -9.66972 -3.38889 6.04587
0.00498 0.0338 0.00133 0.0277 0.00139 0.0416 0.0029 0.0426 0.00181
2R:16199800-16199950:plus -31.4388 -0.431193 5.12844 6.13761 -18.4184 -8.38776 -14.4771 3.54167
-1.18349 0.0122 0.0119 0.00169 0.00226 0.0101 0.00101 0.00805 0.00483 0.0276
2R:16201180-16201330:minus -22.2653 2.51376 3.53211 11.0275 -18.1531 -18.051 -6.02752 8.29167
5.00917 0.00331 0.00415 0.00323 0.0000401 0.00829 0.00886 0.00132 0.000493 0.0031
2R:16201180-16201330:plus -24.6327 5.07339 3.02752 3.19266 -26.2653 -9.5 -8.77982 2.76389 4.78899
0.00824 0.00158 0.00393 0.00829 0.018 0.00294 0.00245 0.00652 0.00336
2R:16206580-16206730:minus -22.1837 2.97248 3.38532 6.33028 -7.86735 -8.72449 -15.4771 -0.597222

-2.43119 0.00315 0.00351 0.00342 0.00198 0.000617 0.00169 0.00985 0.0201 0.038
2R:16206580-16206730:plus -23.4184 0.12844 -5.88073 4.0367 10.6939 -8.45918 -13.3853 2.13889 5.90826
0.00624 0.00987 0.0615 0.00578 7.35e-06 0.00111 0.00631 0.0082 0.00198
2R:16207900-16208050:minus -40.551 -0.495413 6.05505 3.75229 0.540816 -9.53061 -25.367
-0.0277778 6.37615 0.0418 0.0122 0.00113 0.00649 0.000321 0.0031 0.0494 0.0169 0.00159
2R:16207900-16208050:plus -3.40816 3.89908 4.59633 9.7156 -26.7041 -18.0102 3.91743 -5.59722 3.29358
0.0000685 0.0025 0.0021 0.000242 0.0193 0.00812 0.000085 0.0701 0.00623
2R:16214320-16214470:minus -34.0714 -0.431193 6.48624 1.89908 11.6939 -27.7857 -19.7706 0.638889
-4.84404 0.031 0.0119 0.000925 0.0151 1.17e-06 0.0466 0.0198 0.0137 0.0701
2R:16214320-16214470:plus -23.2245 14.8807 4.00917 5.55046 0.846939 -19.051 -17.6881 1.38889 1.22936
0.00576 2.63e-06 0.00267 0.00345 0.000197 0.0165 0.0143 0.0107 0.0132
2R:16214940-16215090:minus -23.1122 2.76147 2.73394 4.15596 -8.92857 -8.82653 -7.22018 3.125
11.8532 0.0053 0.0038 0.00439 0.00539 0.00194 0.00191 0.00178 0.00569 0.0000428
2R:16214940-16215090:plus -22.6939 -1.93578 0.440367 1.87156 -18.1939 -26.4898 -11.9083 4.95833 9.30275
0.00388 0.0191 0.01 0.0152 0.0086 0.0225 0.00452 0.00267 0.00038
2R:1625420-1625570:minus -40.3265 -2.20183 -1 1.59633 -26.4898 -18.3469 -10.4128 3.36111 3.83486
0.0394 0.0206 0.016 0.017 0.0183 0.0124 0.00333 0.00519 0.00481
2R:1625420-1625570:plus -33 2.14679 -0.990826 4.46789 -9.0102 -17.7857 -14.7798 3.90278 -1.27523
0.0258 0.00476 0.0159 0.0049 0.00236 0.0068 0.00858 0.00418 0.0282
2R:1640820-1640970:minus -41.8061 3.52294 -1.90826 2.47706 -27.1224 -9.02041 -4.3578 1.38889 -3.88073
0.0654 0.00287 0.021 0.0115 0.0245 0.00213 0.000826 0.0107 0.0539
2R:1640820-1640970:plus -32.7755 2.17431 -1.99083 7.27523 -27.2653 -27.5306 -18.3761 7.65278 6.29358
0.0234 0.00471 0.0216 0.00109 0.0302 0.0374 0.0158 0.000709 0.00161
2R:1641900-1642050:minus -41.5204 -0.880734 3.77982 1.43119 -17.7857 1.82653 -20.7339 6.52778
1.2844 0.0592 0.0138 0.00293 0.0181 0.00676 0.0000498 0.0234 0.00128 0.0129
2R:1641900-1642050:plus -31.8061 1.26606 -1.88991 5.94495 20.1735 -18.3469 -19.3119 1.19444 -2.11927
0.0158 0.00657 0.0209 0.00261 5.68e-07 0.0124 0.0183 0.0114 0.0344
2R:16439580-16439730:minus -14.1122 -1.36697 13.5872 7.45872 20.2041 -9.57143 -13.2202 2.01389
-4.99083 0.00119 0.0161 0.0000122 0.000894 3.64e-07 0.00326 0.00608 0.00858 0.0726
2R:16439580-16439730:plus -24.7857 0.862385 1 -0.422018 -18.1531 -19.1224 -15.7156 -0.333333
-0.394495 0.00829 0.00761 0.00827 0.0305 0.00829 0.0175 0.0103 0.0185 0.0212
2R:1647320-1647470:minus -14.0408 4.00917 5.46789 0.119266 -17.4898 -16.9796 1.48624 0.583333 2.33028
0.00115 0.0024 0.00146 0.026 0.0053 0.0044 0.000192 0.0139 0.00836
2R:1647320-1647470:plus -32.4796 3.72477 1.11009 3.87156 -17.449 -27.1224 -23.1468 -0.0833333
-0.761468 0.02 0.00266 0.00796 0.00618 0.00484 0.0325 0.0359 0.0172 0.0244
2R:16475020-16475170:minus -40.5918 -0.201835 -0.183486 1.45872 -9.16327 -18.3061 -22.6055
1.11111 2.04587 0.0422 0.011 0.0123 0.0179 0.00249 0.0114 0.0328 0.0117 0.00919
2R:16475020-16475170:plus -31.6224 4.97959 0.807339 6.31193 -18.4898 -8.72449 -12.8349 -0.833333
3.97248 0.0137 0.000156 0.00884 0.00199 0.0116 0.00169 0.00557 0.0215 0.00444
2R:16475560-16475710:minus -31.0714 0.623853 1.54128 -0.238532 -17.7143 -7.57143 -7.25688 3.90278
3.29358 0.0106 0.00829 0.00683 0.0288 0.00627 0.000644 0.00179 0.00418 0.00623
2R:16475560-16475710:plus -23.8469 5.36697 2.25688 2.9633 -17.9388 0.122449 -18.0642 4.56944 -1.40367
0.00685 0.0014 0.00525 0.00914 0.00715 0.000312 0.0151 0.00317 0.0288
2R:16475940-16476130:minus -22.9694 1 -4.62385 0.559633 -28.8673 -8.68367 -13.3303 3.63889 1.6789
0.0048 0.00724 0.0448 0.0232 0.0652 0.00157 0.00624 0.00465 0.011
2R:16475940-16476130:plus -24.1429 0.678899 0.0825688 3.69725 -27.1224 -17.0102 -20.2844 -5.54167
-7.44037 0.0074 0.00813 0.0113 0.00665 0.0245 0.00453 0.0216 0.0692 0.123
2R:1647880-1648030:minus -14.5612 2.70642 10.6239 3.89908 -18.5306 -19.0816 -18.1376 4.04167 3.42202
0.00147 0.00387 0.0000983 0.00604 0.0118 0.017 0.0153 0.00395 0.00597
2R:1647880-1648030:plus -14.9796 4.21101 6.24771 5.48624 -9.5 1.02041 -15.0092 0.638889 -2.02752
0.00161 0.00222 0.00103 0.00357 0.00293 0.000118 0.00899 0.0137 0.0335
2R:1648260-1648410:minus -24.449 15.7248 7.3211 10.5963 -27.3061 -9.09184 -26.422 3.26389 -6.76147
0.00802 5.64e-07 0.00062 0.0001 0.0315 0.00219 0.0563 0.00539 0.107
2R:1648260-1648410:plus -13.0714 4.27523 1 5.73394 -17.6429 -9.5 -12.844 -2.90278 -6.52294 0.000594

0.00217 0.00827 0.00307 0.00562 0.00294 0.00558 0.0377 0.101
2R:16505680-16505830:minus -22.6224 7.15596 -0.275229 4.20183 -17.3061 -37.0408 18.8349 -3.47222
6.73394 0.00371 0.000599 0.0127 0.00533 0.00389 0.126 3.38e-07 0.0434 0.00143
2R:16505680-16505830:plus -23.9592 15.5321 -2.0367 6.45872 -17.3469 -9.09184 -20.6881 0.666667 -0.697248
0.00708 9.74e-07 0.0219 0.00182 0.00394 0.00219 0.0232 0.0135 0.0239
2R:16510940-16511090:minus -13.4184 14.9266 13.6606 3.90826 -17.6429 -8.53061 -23.8532 0.763889
1.01835 0.000873 2.37e-06 0.0000112 0.00603 0.00562 0.00136 0.0402 0.0131 0.0142
2R:16510940-16511090:plus -23.2653 5.88991 8.33028 3.44037 -17.7551 0.0510204 -25.0367 -1.41667
-5.88073 0.00593 0.00111 0.000367 0.00746 0.00658 0.000349 0.0474 0.0254 0.0866
2R:16528280-16528430:minus -12.1429 4.04587 9.21101 -0.165138 -18.0102 -9.53061 -8.54128 5.58333
-0.550459 0.000343 0.00236 0.000224 0.0282 0.00732 0.0031 0.00234 0.00202 0.0225
2R:16528280-16528430:plus -32.7041 3.68367 6.92661 1.83486 -17.5204 -19.4184 -12.9541 3.91667 9.23853
0.0222 0.00144 0.000753 0.0155 0.00532 0.0198 0.00572 0.00416 0.000397
2R:16529120-16529270:minus -30.4796 -1.18349 1.21101 3.51376 -18.2245 -17.2755 -23.0183 6.88889
2.16514 0.00941 0.0152 0.00768 0.00709 0.00877 0.00544 0.0352 0.00107 0.00879
2R:16529120-16529270:plus -30.8163 -4.36697 1.17431 0.458716 -27 -17.051 -11.3486 2.52778 7.06422
0.0103 0.0373 0.00778 0.024 0.0233 0.00479 0.00401 0.00712 0.00131
2R:16531400-16531550:minus -22.9184 -0.394495 -0.431193 2.78899 20.4694 -9.65306 -14.8991
-0.472222 3.6789 0.00447 0.0118 0.0133 0.00992 1.65e-07 0.0034 0.00879 0.0193 0.00506
2R:16531400-16531550:plus -32.0408 -0.137615 2.07339 2.33945 10.3571 -7.53061 -24.9083 0.805556
-0.908257 0.0174 0.0108 0.00562 0.0122 0.0000208 0.000625 0.0466 0.013 0.0255
2R:16554180-16554330:minus -24.3673 -2.42202 0.559633 3.9633 -18.7143 -9.72449 -7.12844 1.80556
1.22018 0.00791 0.0219 0.00961 0.0059 0.0136 0.00352 0.00174 0.00923 0.0133
2R:16554180-16554330:plus -31.2551 0.724771 6.55046 1.91743 -27.1531 -9.37755 -17.4771 7.22222 2.88073
0.0113 0.008 0.000896 0.015 0.0249 0.00265 0.0138 0.000895 0.00727
2R:16554740-16554890:minus -24.3776 1.08257 1.85321 2.66972 -8.71429 -26.3061 -8.13761 -7.15278
3.7156 0.00793 0.00703 0.0061 0.0106 0.00159 0.0219 0.00216 0.0953 0.00502
2R:16554740-16554890:plus -3.11224 0.761468 0.706422 0.990826 2.14286 -9.57143 -6.22018 -5.08333 3.44954
0.0000458 0.00789 0.00914 0.0206 0.0000316 0.00326 0.00139 0.0628 0.00594
2R:16555520-16555670:minus -32.1122 3.83673 -0.394495 1.62385 -8.60204 -18.4184 -13.1743 2.33333
6.38532 0.0178 0.00115 0.0132 0.0168 0.00125 0.0127 0.00602 0.00765 0.00157
2R:16555520-16555670:plus -31.6735 -1.3211 -1.24771 -0.559633 -8.86735 -17.051 -20.4771 5.375
2.54128 0.0141 0.0159 0.0172 0.0318 0.00173 0.00479 0.0224 0.00222 0.00787
2R:16558540-16558690:minus -31.4388 -1.83486 2.41284 9.44954 -18.9796 -8.53061 -16.8165 -1.54167
-5.62385 0.0122 0.0185 0.00496 0.000326 0.0155 0.00136 0.0124 0.0263 0.0822
2R:16558540-16558690:plus -22.2245 5.76147 13.6514 7.29358 -27.6327 -18.3469 -17.0092 0.194444 1.74312
0.00325 0.00118 0.0000113 0.00104 0.0369 0.0124 0.0128 0.0157 0.0105
2R:1660620-1660770:minus -14.4184 8.48624 11.6789 3.24771 -18.3776 -17.2755 -17.8532 -0.847222
8.11009 0.00137 0.000293 0.000053 0.00808 0.00926 0.00544 0.0146 0.0216 0.000597
2R:1660620-1660770:plus -23.4082 0.605505 9.58716 2.11009 -8.86735 -18.9388 -9.85321 -0.958333
3.20183 0.00623 0.00834 0.000181 0.0136 0.00173 0.0154 0.00299 0.0223 0.0065
2R:1669340-1669490:minus -12.5102 -2.04587 -0.293578 0.302752 -25.9694 -19.0816 -15.1009 6.95833
1.16514 0.000436 0.0197 0.0128 0.0248 0.017 0.017 0.00916 0.00103 0.0135
2R:1669340-1669490:plus -14.3673 -1.72477 4.22018 5.57798 -18.7857 -18.051 -15.6881 0.680556 -1.86239
0.00132 0.0179 0.00245 0.0034 0.0148 0.00886 0.0102 0.0135 0.032
2R:1669800-1669950:minus -24.1837 -1.24771 11.0459 5.31193 -17.4184 -9.23469 -6.55963 1.09722 9.92661
0.00751 0.0155 0.0000777 0.00393 0.00457 0.00238 0.00152 0.0118 0.000193
2R:1669800-1669950:plus -30.2143 -0.431193 1.29358 3.31193 -7.89796 -9.45918 -5.91743 4.16667
9.92661 0.0088 0.0119 0.00746 0.00786 0.000678 0.00279 0.00128 0.00375 0.000193
2R:1670200-1670350:minus -32.2857 -3.61468 13.1193 3.87156 -18.3776 -8.79592 -21.1927 -3.75 7.68807
0.0185 0.0305 0.0000185 0.00618 0.00926 0.0019 0.0255 0.0464 0.000841
2R:1670200-1670350:plus 5.47959 1.08257 8.15596 10.7615 -18.1939 -8.27551 -19.0092 -2.56944 9.34862
9.72e-06 0.00703 0.000403 0.0000826 0.0086 0.000919 0.0175 0.0346 0.000371
2R:16719420-16719570:minus -4.55102 2.63303 5.22936 -0.100917 -27.3776 -26.1224 -6.66055 -1.65278

11.4771 0.000159 0.00398 0.00162 0.0277 0.032 0.0217 0.00155 0.0271 0.0000679
2R:16719420-16719570:plus -30.102 -0.137615 3.27523 3.29358 -17.8163 -9.16327 -21.2936 1.09722
4.10092 0.00859 0.0108 0.00357 0.00791 0.00682 0.00223 0.0259 0.0118 0.00413
2R:16719840-16719990:minus -20.3571 6.85321 6.0367 1.72477 -17.449 -18.3061 -9.50459 0.625
-0.550459 0.00165 0.000701 0.00113 0.0161 0.00484 0.0114 0.00281 0.0137 0.0225
2R:16719840-16719990:plus -23.5612 -4.01835 2.11927 8.80734 -8.93878 -18.3469 -25.0459 6.93056 -1.79817
0.00653 0.034 0.00553 0.000518 0.00216 0.0124 0.0474 0.00104 0.0315
2R:16727100-16727250:minus -32.0408 -2.11927 -2.77064 5.90826 -9.27551 -9.79592 -25.7156 0.875
7.94495 0.0174 0.0201 0.027 0.00271 0.00284 0.00377 0.0516 0.0127 0.000667
2R:16727100-16727250:plus -30.8163 3.81651 1.97248 4.10092 -26.9694 -17.0102 -18.844 -3.59722 7.68807
0.0103 0.00258 0.00584 0.00554 0.023 0.00453 0.017 0.0448 0.000841
2R:16758200-16758350:minus -33.0408 8.22018 3.79817 3.24771 -18.449 -17.9796 -20.6697 2.88889
-3.09174 0.0264 0.000338 0.00291 0.00808 0.0107 0.00764 0.0232 0.00622 0.0456
2R:16758200-16758350:plus -41.4796 -0.688073 15.5596 7.45872 0.877551 -19.6429 -16.7798 -3.23611
0.963303 0.0577 0.013 6.69e-07 0.000894 0.00019 0.0204 0.0124 0.041 0.0145
2R:16864460-16864610:minus -3.7449 7.7156 2.68807 3.88991 20.4694 -8.5 -13.8807 -5.69444 7.94495
0.000104 0.000445 0.00447 0.00608 1.65e-07 0.00126 0.00706 0.0715 0.000667
2R:16864460-16864610:plus -33.3367 -1.43119 8 1.6789 -17.9388 -0.173469 -17.8532 5.79167 -0.862385
0.0281 0.0164 0.000438 0.0165 0.00715 0.000456 0.0146 0.00183 0.0251
2R:16881800-16881950:minus -30.4796 10.8991 8.38532 6.02752 -18.1224 0.0510204 -17.7523 5.86111
1.23853 0.00941 0.000085 0.000356 0.00246 0.00782 0.000349 0.0144 0.00177 0.0132
2R:16881800-16881950:plus -32.4796 1.6789 -0.917431 -1.86239 -27.0102 -8.27551 -25 4.625 3.16514
0.02 0.00565 0.0156 0.0487 0.0237 0.000919 0.0472 0.00309 0.00656
2R:16882320-16882470:minus -51.9286 -0.40367 -1.84404 0.899083 -37.3469 -0.540816 -28.4679 5.88889
1.90826 0.192 0.0118 0.0206 0.0212 0.13 0.00054 0.0733 0.00175 0.00974
2R:16882320-16882470:plus -31.2551 0.0366972 0.752294 6.36697 -7.60204 -16.9388 -20.578 -5.52778
-2.15596 0.0113 0.0102 0.009 0.00193 0.000466 0.00433 0.0228 0.069 0.0348
2R:16884180-16884330:minus -31.3265 -1.41284 -2.38532 2.14679 1.21429 -17.2041 -10.8165 -2.27778
-6.11927 0.0114 0.0163 0.0242 0.0133 0.0000864 0.00507 0.0036 0.0321 0.0914
2R:16884180-16884330:plus -40.2143 3.57143 4.86239 6.21101 -27.602 -18.7551 -11.0826 8.52778 10.1284
0.0372 0.00175 0.00188 0.00213 0.0364 0.0141 0.0038 0.000429 0.000129
2R:16884540-16884690:minus -23.0408 -0.119266 11.8716 7.29358 -18.7959 -17.0102 -23.9541
2.33333 2.3211 0.00512 0.0107 0.0000468 0.00104 0.0148 0.00453 0.0408 0.00765 0.00839
2R:16884540-16884690:plus -13.0714 1.2844 4.09174 6.31193 -27.1224 -9.72449 -5.08257
2.26389 0.550459 0.000594 0.00653 0.00258 0.00199 0.0245 0.00352 0.00102 0.00784 0.0161
2R:16885560-16885710:minus -33.5612 0.0917431 -3.53211 2.00917 -9.27551 0.755102
-23.3028 4.93056 5.79817 0.0288 0.00999 0.0334 0.0142 0.00284 0.000175 0.0369 0.00271 0.00208
2R:16885560-16885710:plus -32.9286 -4 0.504587 2.11009 -27.2347 -8.57143 -14.9633 2.11111 5.3211 0.025
0.0338 0.0098 0.0136 0.0283 0.00149 0.00891 0.00828 0.00275
2R:16886220-16886370:minus -13.1837 3.68367 -4.18349 5.86239 -17.449 -9.45918 -16.7248 3.26389
-2.11009 0.000705 0.00144 0.0399 0.00282 0.00484 0.00279 0.0123 0.00539 0.0343
2R:16886220-16886370:plus -30.398 -1.72477 2.76147 5.85321 -18.449 -19.3469 -14.1743 5.59722 -4.76147
0.00901 0.0179 0.00435 0.00287 0.0107 0.0194 0.00754 0.002 0.0686
2R:16887760-16887910:minus -41.0204 1.77982 0.486239 1.22936 1.14286 -17.5 -16.1468 6.18056
4.02752 0.0457 0.00544 0.00986 0.0193 0.000113 0.00599 0.0111 0.00152 0.00428
2R:16887760-16887910:plus -21.551 5.30275 0.0458716 7.80734 -17.0408 0.602041 -4.58716 0.861111
6.02752 0.00205 0.00144 0.0114 0.00063 0.00329 0.000188 0.000882 0.0127 0.00183
2R:16899320-16899470:minus -30.2143 11.0826 1.3945 4.6055 -18.7551 -17.5714 -17.8349 3.63889
4.55963 0.0088 0.0000773 0.00719 0.00476 0.0144 0.00616 0.0146 0.00465 0.00357
2R:16899320-16899470:plus -4.85714 -2.31193 1.40367 6.00917 20.4694 -8.94898 -26.3211 -5.59722 -7.92661
0.000202 0.0213 0.00717 0.00255 1.65e-07 0.00203 0.0556 0.0701 0.133
2R:16912520-16912670:minus -20.9286 9.00917 -2.88991 3.77064 -8.89796 -8.42857 -5.87156 -0.75
-2.94495 0.00183 0.000222 0.028 0.00637 0.00189 0.00104 0.00127 0.021 0.0441
2R:16912520-16912670:plus -23.9286 0.422018 15.6239 6.16514 -8.93878 -7.79592 -26.7798 -0.319444

1.85321 0.00701 0.0089 5.44e-07 0.00223 0.00216 0.000723 0.0589 0.0184 0.01
2R:16944640-16944790:minus -4.78571 0.899083 2.47706 4.19266 -18.6837 -9.42857 0.211009 0.166667
-0.678899 0.000194 0.00751 0.00484 0.00536 0.0126 0.0027 0.000269 0.0159 0.0237
2R:16944640-16944790:plus -22 11.8624 1.91743 6.17431 -26.9694 -19.3469 -6.58716 4.34722 0.440367
0.00291 0.0000498 0.00596 0.0022 0.023 0.0194 0.00153 0.00348 0.0164
2R:16945220-16945370:minus -33.7653 -0.0183486 -1.27523 5.82569 -27.5306 -8.5 -7.29358 5.61111
-0.59633 0.0295 0.0104 0.0174 0.00291 0.0351 0.00126 0.00181 0.00199 0.0229
2R:16945220-16945370:plus -13.0714 3.90816 -1.59633 2.05505 -18.5204 -18.8571 -13.4679 -0.611111
3.0367 0.000594 0.00102 0.0192 0.0139 0.0117 0.0151 0.00644 0.0201 0.00694
2R:16945740-16945890:minus -13.1122 3.3945 12.1468 7.2844 -27.1633 -18.2755 -9.11009 1.47222
2.53211 0.000632 0.00301 0.0000388 0.00105 0.025 0.0112 0.00261 0.0104 0.00791
2R:16945740-16945890:plus -29.9184 -2.13761 4.25688 5.37615 -8.67347 -18.7857 -12.6239 -4.11111 8.10092
0.00854 0.0202 0.00242 0.00384 0.00154 0.0146 0.0053 0.0505 0.000623
2R:16948080-16948230:minus -22.3571 6.92661 11.9358 3.90826 -18.2653 0.244898 -20.1101 2.80556
-0.284404 0.00339 0.000674 0.0000453 0.00603 0.00882 0.000285 0.021 0.00642 0.0207
2R:16948080-16948230:plus -21.2551 3 13.7798 7.29358 -16.449 -8.57143 -7.76147 8.45833 0.495413
0.00191 0.00348 9.84e-06 0.00104 0.00307 0.00149 0.002 0.000447 0.0162
2R:16959680-16959830:minus -33.7347 6.75229 2.77064 6.85321 -8.0102 -18.0816 -23.2844 -1.23611
0.697248 0.0294 0.000736 0.00433 0.00137 0.000826 0.00934 0.0368 0.0241 0.0154
2R:16959680-16959830:plus -41.398 -0.0733945 -2.53211 6.48624 -8.67347 -9.86735 -17.9908 4.97222
-2.00917 0.0554 0.0106 0.0252 0.00177 0.00154 0.00391 0.0149 0.00266 0.0333
2R:16960280-16960430:minus -21.551 3.92661 -0.00917431 7 -26.898 -9.79592 -23.9725 -0.763889
3.45872 0.00205 0.00247 0.0116 0.00132 0.0203 0.00377 0.0409 0.0211 0.00591
2R:16960280-16960430:plus -24.4082 -1.6789 1.43119 2.75229 -7.56122 -27.6735 -16.9266 4.40278 10.1284
0.00797 0.0177 0.0071 0.0101 0.000419 0.0443 0.0127 0.0034 0.000129
2R:16961180-16961330:minus -22.6633 4.83486 3.15596 4.0367 -18.449 -8.30612 -17.6055 -2.47222
-0.862385 0.00384 0.00174 0.00374 0.00578 0.0107 0.000973 0.0141 0.0338 0.0251
2R:16961180-16961330:plus -30.9286 0.422018 3.00917 11.0183 1.94898 0.612245 -17.4495 -2.43056 -4.33945
0.0104 0.0089 0.00396 0.000052 0.0000358 0.000187 0.0137 0.0334 0.0609
2R:16963540-16963690:minus -32.1531 -2.57798 0.00917431 7.47706 -9.5 -27.5612 -18.8991 6.41667
3.66972 0.0179 0.0229 0.0116 0.00085 0.00293 0.0393 0.0172 0.00135 0.00514
2R:16963540-16963690:plus -23.6327 -0.137615 0.495413 3.49541 -17.4082 -27.9694 -14.367 -1.11111
11.8532 0.00661 0.0108 0.00983 0.00716 0.00421 0.0547 0.00786 0.0233 0.0000428
2R:16964260-16964410:minus -21.9286 0.697248 6.37615 6.41284 -9.23469 -9.02041 -19.9633 -1.33333
-0.513761 0.0027 0.00807 0.000974 0.00189 0.00277 0.00213 0.0205 0.0248 0.0221
2R:16964260-16964410:plus -21.9286 -1.49541 0 2.23853 -18.3469 -18.7857 -3.33945 5.77778 5.38532
0.0027 0.0167 0.0116 0.0128 0.00894 0.0146 0.000621 0.00184 0.00267
2R:16978420-16978570:minus -24.8163 1.76147 2.06422 3.00917 0.540816 -19.2755 -14.0275 2.51389
5.70642 0.0083 0.00548 0.00564 0.00894 0.000321 0.0185 0.0073 0.00716 0.00222
2R:16978420-16978570:plus -33.3367 8.33028 -2.6422 -0.137615 -18.7551 -0.244898 -20.8165 3.26389
-2.59633 0.0281 0.000319 0.0261 0.0279 0.0144 0.000496 0.0238 0.00539 0.0398
2R:16984740-16984890:minus -21.8878 15.1376 -0.366972 6.00917 -27.1939 -16.9796 -10.0826 -0.444444
5.08257 0.00258 1.81e-06 0.0131 0.00255 0.0267 0.0044 0.00313 0.0192 0.00303
2R:16984740-16984890:plus -30.7041 15.1376 1.24771 7.61468 20.1735 -0.0510204 -11.3394 3.68056
2.89908 0.00993 1.81e-06 0.00758 0.000779 5.68e-07 0.000429 0.004 0.00457 0.00722
2R:17015940-17016090:minus -21.9592 13.4592 -0.119266 3.3578 -8.96939 -19.0102 -13.8073 1.27778
8.11009 0.00279 0.000064 0.0121 0.00767 0.00226 0.0159 0.00695 0.0111 0.000597
2R:17015940-17016090:plus -20.9592 13.4592 -4.19266 3.13761 -17.7551 -7.38776 -7.26606 -0.0416667
5.70642 0.00184 0.000064 0.04 0.00854 0.00658 0.000563 0.00179 0.017 0.00222
2R:17022300-17022450:minus -31 4.76147 -0.477064 2.3578 -18.7551 -8.57143 -23.7982 2.40278
4.77064 0.0105 0.00179 0.0135 0.0121 0.0144 0.00149 0.0398 0.00746 0.0034
2R:17022300-17022450:plus -31.4388 1.3211 -3.20183 -1.66972 -18.1531 -18.7143 -21.1009 -2.13889 2.25688
0.0122 0.00644 0.0305 0.0458 0.00829 0.014 0.025 0.0309 0.00853
2R:17029660-17029810:minus -4.4898 7.02752 1.73394 11.0275 10.0918 -9.23469 -2.69725 3.08333

-3.40367 0.000154 0.000639 0.00638 0.0000401 0.0000251 0.00238 0.000526 0.00578 0.0486
2R:17029660-17029810:plus -33.6327 0.779817 4.59633 9.33028 -27.6429 1.02041 -14.7615 6.84722 -2.51376
0.029 0.00784 0.0021 0.000374 0.037 0.000118 0.00855 0.00109 0.039
2R:17031000-17031150:minus -32.4694 0.256881 -3.05505 -0.844037 -27.1939 -17.7857 -17.3853 0.680556
5.01835 0.0196 0.00944 0.0293 0.035 0.0267 0.0068 0.0136 0.0135 0.00309
2R:17031000-17031150:plus -23.3367 3.51376 7.94495 7.77982 -18.4184 -9.0102 -9.40367 5.86111 5.01835
0.00614 0.00288 0.000451 0.000652 0.0101 0.00208 0.00276 0.00177 0.00309
2R:17033220-17033370:minus -30.898 -3.84404 2.06422 6.22936 -26.5306 0.755102 -9.80734 3.44444
1.73394 0.0103 0.0324 0.00564 0.00209 0.0185 0.000175 0.00297 0.00502 0.0106
2R:17033220-17033370:plus -40.4388 -0.807339 -1.00917 -1.40367 -9.16327 -18.2755 -6.88073 1.26389
1.90826 0.0401 0.0135 0.016 0.042 0.00249 0.0112 0.00164 0.0111 0.00974
2R:17043800-17043950:minus -23.0408 -0.0550459 1.91743 6.47706 -18.6429 -9.53061 -29.1927 -2.41667
0.0183486 0.00512 0.0105 0.00596 0.00179 0.0121 0.0031 0.0811 0.0333 0.0183
2R:17043800-17043950:plus -30.4082 0.40367 3.22018 6.05505 -28.4592 0.346939 -10.9908 5.88889 3.00917
0.00908 0.00896 0.00365 0.0024 0.0568 0.000221 0.00373 0.00175 0.00697
2R:17046940-17047090:minus -21.9286 -0.733945 1.55963 4.78899 -18.4592 -17.7551 -14.5596 4.58333
10.0367 0.0027 0.0132 0.00678 0.00456 0.0109 0.00643 0.00819 0.00315 0.000146
2R:17046940-17047090:plus -21.6224 -0.321101 -2.82569 1.57798 1.10204 -27.5612 -13.2202 3.59722
3.09174 0.00213 0.0115 0.0275 0.0171 0.000124 0.0393 0.00608 0.00473 0.00678
2R:17047980-17048130:minus -3.63265 11.1743 0.201835 4.04587 -26.898 -26.8265 -9.27523 0.222222
1.22936 0.0000917 0.000074 0.0108 0.00572 0.0203 0.0285 0.00269 0.0156 0.0132
2R:17047980-17048130:plus -32.7755 6.40367 0.229358 2.52294 -18.6735 -8.72449 -8.51376 0.333333 -4.78899
0.0234 0.000874 0.0107 0.0113 0.0123 0.00169 0.00233 0.0151 0.0691
2R:17056200-17056350:minus -21.9592 -3.20183 0.321101 2.55046 -17.4184 -18.051 -25.6697 1.61111
-0.284404 0.00279 0.0273 0.0104 0.0112 0.00457 0.00886 0.0514 0.00988 0.0207
2R:17056200-17056350:plus -14.1837 7.26606 3.37615 3.75229 0.836735 0.387755 -15.2844 4.61111 -4.84404
0.00124 0.000565 0.00343 0.00649 0.000215 0.000217 0.00949 0.00311 0.0701
2R:17058180-17058330:minus -31.551 0.431193 1.17431 9.59633 -8.27551 9.57143 -22.5596 2.73611
3.69725 0.0134 0.00887 0.00778 0.000275 0.000951 0.0000274 0.0326 0.00659 0.00503
2R:17058180-17058330:plus -41.7857 3.7156 -1.22936 1.76147 -18.7143 -8.65306 -22.0275 4.48611 -4.73394
0.0653 0.00267 0.0171 0.0159 0.0136 0.00156 0.0296 0.00328 0.0681
2R:17062660-17062810:minus -32.3061 5.15596 14.6147 2.61468 -17.1531 -8.79592 -12.5413 -5.81944
1.90826 0.0186 0.00153 3.32e-06 0.0109 0.00364 0.0019 0.00521 0.0734 0.00974
2R:17062660-17062810:plus -12.6939 4.05102 -0.0366972 3.34862 -19.2755 -18.2041 -4.94495 7.125
9.77064 0.000462 0.000335 0.0117 0.0077 0.0164 0.0103 0.000977 0.000942 0.000228
2R:17066500-17066650:minus -24.102 1.56881 3.80734 2.87156 -17.8878 -27.5612 3.99083 5.52778
5.00917 0.00733 0.00588 0.0029 0.00948 0.00704 0.0393 0.0000828 0.00207 0.0031
2R:17066500-17066650:plus -11.8469 -0.0183486 4.6055 2.55963 -9.0102 -27.5306 -16.4679 -0.263889
7.6422 0.000279 0.0104 0.0021 0.0111 0.00236 0.0374 0.0118 0.0181 0.000893
2R:17070700-17070850:minus -33.0714 10.5688 0.256881 3.22018 -9.5 1.82653 -23.5872 -1.19444 -2.15596
0.0267 0.000101 0.0106 0.00818 0.00293 0.0000498 0.0386 0.0239 0.0348
2R:17070700-17070850:plus -14.7551 2.25688 -2.88073 3.23853 -17.7143 -9.60204 -26.1284 8.56944 -3.07339
0.00157 0.00457 0.0279 0.00815 0.00627 0.00334 0.0543 0.000418 0.0454
2R:17088680-17088830:minus -22 4.66972 5.87156 6.3578 -17.7143 -18.0102 -11.8257 0.777778 1.02752
0.00291 0.00186 0.00122 0.00195 0.00627 0.00812 0.00444 0.0131 0.0141
2R:17088680-17088830:plus -23.4184 8.66055 -1.6422 2.50459 -18.9796 -8.38776 -24.5688 -2.93056 12.2752
0.00624 0.000268 0.0194 0.0114 0.0155 0.00101 0.0445 0.038 0.0000141
2R:17089500-17089650:minus -22.9286 1.11009 -1.3578 4.49541 -26.7449 -9.86735 -6.43119 -1.52778
-1.11009 0.0046 0.00695 0.0178 0.00487 0.0195 0.00391 0.00147 0.0262 0.0269
2R:17089500-17089650:plus -4.70408 -0.926606 7.16514 5.69725 -27.1531 0.316327 -6.25688 -7.44444
-0.0825688 0.000186 0.014 0.000671 0.00314 0.0249 0.000243 0.0014 0.101 0.0189
2R:17169800-17169950:minus -23.6224 14.945 7.11009 0.522936 -18.6837 -18.6327 -20.3211 4.09722
-0.220183 0.00658 2.29e-06 0.000691 0.0234 0.0126 0.0137 0.0218 0.00386 0.0201
2R:17169800-17169950:plus -22.1429 14.8807 10.2018 7.6422 -26.9694 -0.173469 -10.6147 0.375

0.93578 0.00305 2.63e-06 0.000126 0.000721 0.023 0.000456 0.00346 0.0149 0.0145
2R:17170180-17170330:minus -33.3367 0.183486 2.66055 7.80734 -8.96939 -10.0204 -21.055 -1.79167
0.963303 0.0281 0.00968 0.00452 0.00063 0.00226 0.00399 0.0248 0.0282 0.0145
2R:17170180-17170330:plus -23.449 1.86239 1.15596 2.33945 -26.7041 -19.2347 -8.76147 -0.430556
6.7156 0.00631 0.00528 0.00783 0.0122 0.0193 0.018 0.00245 0.0191 0.00144
2R:17179680-17179830:minus -29.7449 2 -3.09174 0.321101 0.836735 -17.7143 -16.4771 4.47222 3.6055
0.00849 0.00502 0.0296 0.0247 0.000215 0.00634 0.0118 0.0033 0.00532
2R:17179680-17179830:plus -33.2959 -1.62385 -0.605505 5.89908 -26.9286 -18.051 -19.3028 1.95833
3.83486 0.0279 0.0174 0.0141 0.00272 0.0214 0.00886 0.0183 0.00875 0.00481
2R:17181040-17181190:minus -31.7041 3.80734 -2.14679 -0.651376 -18.051 -17.9796 -19.6239 -2.61111
-4.18349 0.0146 0.00259 0.0226 0.0329 0.00746 0.00764 0.0193 0.035 0.0581
2R:17181040-17181190:plus -23.1122 -1.77982 -1.88073 3.41284 -17.8163 -8.72449 -27.7431 1.02778 -0.284404
0.0053 0.0182 0.0209 0.00756 0.00682 0.00169 0.0665 0.012 0.0207
2R:17183080-17183230:minus -31.5102 -1.76147 1.6789 1.73394 -18.4898 -18.8571 -15.6881 3.36111
-1.18349 0.013 0.0181 0.0065 0.016 0.0116 0.0151 0.0102 0.00519 0.0276
2R:17183080-17183230:plus -32.1735 -1.82569 1.20183 3.6055 -8.93878 -18.1224 -13.9174 0.680556 2.20183
0.018 0.0185 0.0077 0.00686 0.00216 0.00988 0.00712 0.0135 0.00869
2R:17183400-17183550:minus -21.7449 6.73394 3.78899 2.7156 -18.6735 -9.02041 -12.1101 3.08333
1.89908 0.00236 0.000744 0.00292 0.0102 0.0123 0.00213 0.00472 0.00578 0.0098
2R:17183400-17183550:plus -13.8878 2.86239 12.9725 7.45872 -17.9286 -18.2041 -12.422 3.04167 1.94495
0.00111 0.00366 0.0000215 0.000894 0.0071 0.0103 0.00507 0.00587 0.00968
2R:17200020-17200170:minus -23.2551 14.2661 4.22936 3.94495 -18.4898 -17.9796 -17.2752 6.93056
1.78899 0.00583 6.1e-06 0.00244 0.00595 0.0116 0.00764 0.0134 0.00104 0.0103
2R:17200020-17200170:plus -23.1531 0.0550459 3.75229 6.00917 -7.64286 -18.3469 -15.6055 1.79167
1.48624 0.00542 0.0101 0.00296 0.00255 0.000499 0.0124 0.0101 0.00928 0.0118
2R:17200400-17200550:minus -32.2653 3.2844 0.779817 2.07339 -27.1633 0.122449 -9.73394 4.125
-2.69725 0.0184 0.00313 0.00892 0.0138 0.025 0.000312 0.00293 0.00382 0.0411
2R:17200400-17200550:plus -30.449 -3.25688 0.981651 0.53211 -17.7143 -9.7551 -34.6972 0.166667 1.97248
0.00925 0.0277 0.00832 0.0234 0.00627 0.00358 0.156 0.0159 0.00948
2R:17210800-17210950:minus -14.2143 4.29358 5.36697 2.99083 10.7245 -27.1122 -15.5321 1.59722
-1.59633 0.00124 0.00215 0.00152 0.00899 5.2e-06 0.0316 0.00995 0.00993 0.0301
2R:17210800-17210950:plus -20.5918 3.24771 -0.155963 3.17431 10.6531 -9.5 -11.5229 -3.15278 -2.07339
0.00169 0.00317 0.0122 0.00842 0.0000102 0.00294 0.00416 0.0402 0.034
2R:17211180-17211330:minus -40.1837 -0.357798 -0.155963 7.02752 -18.9388 -9.45918 -18.945
-4.05556 7.18349 0.0367 0.0116 0.0122 0.00122 0.0151 0.00279 0.0173 0.0499 0.00121
2R:17211180-17211330:plus -23.1531 0.376147 4.17431 2.69725 -18.7143 -17.3469 -18.1009 1.90278 -3.01835
0.00542 0.00905 0.0025 0.0104 0.0136 0.0058 0.0152 0.00892 0.0448
2R:17262420-17262570:minus -31.1429 1.18349 3.45872 2.69725 -17.7857 -25.5612 -22.3578 3.93056
-0.0275229 0.0108 0.00677 0.00332 0.0104 0.00676 0.0206 0.0314 0.00413 0.0186
2R:17262420-17262570:plus -21.4082 8.87156 5.91743 4.3211 -26.9286 -7.5 -19.9266 -1.18056 1.89908
0.00198 0.000239 0.0012 0.00514 0.0214 0.000614 0.0203 0.0238 0.0098
2R:17267020-17267170:minus -3.14286 -1.20183 0.412844 9.61468 -18.4184 -8.45918 5.23853 -5.94444
2.09174 0.0000468 0.0153 0.0101 0.000255 0.0101 0.00111 0.000053 0.0752 0.00904
2R:17267020-17267170:plus -32.6735 7.78899 -0.229358 0.477064 -19.0204 -9.42857 -20.3211 0.763889
1.88073 0.0216 0.000429 0.0125 0.0238 0.0158 0.0027 0.0218 0.0131 0.00987
2R:17270360-17270510:minus -21.6327 2.6789 4.08257 7.72477 -26.7041 -8.45918 -16.7615 2.45833
-2.81651 0.00215 0.00391 0.00259 0.000681 0.0193 0.00111 0.0123 0.00731 0.0424
2R:17270360-17270510:plus -23.1531 0.954128 2.01835 5.63303 -26.8571 -26.602 -17.5688 -1.79167 2.33028
0.00542 0.00736 0.00574 0.00326 0.0198 0.0248 0.014 0.0282 0.00836
2R:17271740-17271890:minus -31.4694 5.63303 4.88991 0.12844 -26.9286 -37.2245 -13.2936 0.375
7.63303 0.0124 0.00125 0.00186 0.0259 0.0214 0.142 0.00618 0.0149 0.000928
2R:17271740-17271890:plus -30.6327 5.88073 0.541284 6.33028 -18.1224 0.122449 -24.2477 -1.73611 1.07339
0.00968 0.00112 0.00968 0.00198 0.00782 0.000312 0.0426 0.0277 0.014
2R:17273700-17273850:minus -21.2245 7.19266 3.07339 7.17431 -27.1531 0.0204082 -19.7064 0.430556

1.95413 0.0019 0.000587 0.00386 0.00115 0.0249 0.000377 0.0196 0.0146 0.00964
2R:17273700-17273850:plus -30.9286 0.954128 8.79817 9.33028 -26.0408 -10.0204 -8.61468 -2.51389 0.862385
0.0104 0.00736 0.000283 0.000374 0.0171 0.00399 0.00238 0.0341 0.0148
2R:17273980-17274130:minus -22 7.33028 7.0367 0.908257 -26.5306 -17.2755 -15.4771 8.05556 -0.486239
0.00291 0.000546 0.000715 0.0211 0.0185 0.00544 0.00985 0.000565 0.0219
2R:17273980-17274130:plus -10.7755 2.09174 7.76147 6.82569 -26.1939 -8.93878 -15.7156 5.06944 5.7156
0.000235 0.00485 0.000497 0.00144 0.0175 0.00201 0.0103 0.00255 0.00217
2R:17368260-17368410:minus -14.4184 1.0367 -5.84404 4.3945 -26.898 -8.42857 -15.4404 0.847222
11.8073 0.00137 0.00714 0.0609 0.00499 0.0203 0.00104 0.00978 0.0128 0.0000538
2R:17368260-17368410:plus -31.3265 -4.50459 -0.697248 -0.605505 -8.85714 1.82653 -18.6422 0.513889
-5.94495 0.0114 0.0387 0.0145 0.0323 0.00165 0.0000498 0.0165 0.0142 0.0878
2R:17368660-17368810:minus -30.4082 2.31193 2.52294 7 0.571429 -8.23469 -13.9266 1.04167 7.53211
0.00908 0.00448 0.00476 0.00132 0.0003 0.000875 0.00714 0.012 0.000968
2R:17368660-17368810:plus -31.4796 -1.08257 2.52294 4.9633 -17.7143 -8.02041 -14.6697 4.22222 3.15596
0.0128 0.0147 0.00476 0.0044 0.00627 0.000761 0.00838 0.00367 0.00659
2R:17369360-17369510:minus -34.0714 0.926606 0.59633 4.9633 -8.97959 9.30612 -25.1468 6.11111
-0.174312 0.031 0.00743 0.00949 0.0044 0.0023 0.0000398 0.0481 0.00157 0.0196
2R:17369360-17369510:plus -33 -1.56881 0.541284 5.93578 -8.93878 -7.72449 -26.7982 2.90278 -0.275229
0.0258 0.0171 0.00968 0.00264 0.00216 0.00068 0.059 0.00619 0.0206
2R:17382380-17382530:minus -30.4388 2.99083 1.33028 2.04587 -26.9592 -17.7857 -9.80734 -4.05556
-0.577982 0.00923 0.00349 0.00736 0.014 0.0215 0.0068 0.00297 0.0499 0.0227
2R:17382380-17382530:plus -23 -0.944954 1.7156 3.22018 -17.3776 -17.9796 -10.3945 -6.48611 4.18349
0.00498 0.0141 0.00642 0.00818 0.00419 0.00764 0.00332 0.0838 0.00404
2R:17384320-17384470:minus -23.2245 1.08257 2.44037 3.52294 -35.6735 -8.79592 -8.3945 0.652778
5.58716 0.00576 0.00703 0.00491 0.00706 0.0668 0.0019 0.00228 0.0136 0.00237
2R:17384320-17384470:plus -31.0714 7.77982 -1.30275 5.69725 -17.7143 -17.4184 -16.055 4.59722 6.18349
0.0106 0.00043 0.0175 0.00314 0.00627 0.00585 0.011 0.00313 0.00167
2R:17384860-17385010:minus -3.22449 1.00917 2.21101 6.47706 -7.86735 -19.051 -15.6789 2.06944
0.201835 0.0000495 0.00722 0.00534 0.00179 0.000617 0.0165 0.0102 0.00841 0.0175
2R:17384860-17385010:plus -23.3367 -2.22018 2.52294 -0.165138 -17.8265 -27.6735 -17.9725 2.69444
11.8532 0.00614 0.0207 0.00476 0.0282 0.00691 0.0443 0.0149 0.00669 0.0000428
2R:17389440-17389590:minus -3.67347 1.33028 15.0367 7.29358 -27.3061 -18.1224 -12.4495 8.52778
1.82569 0.0000972 0.00642 1.36e-06 0.00104 0.0315 0.00988 0.0051 0.000429 0.0101
2R:17389440-17389590:plus -22.8878 9.04587 -0.495413 2.62385 -27.1531 -17.9388 -15.4587 4.68056
3.56881 0.00441 0.000218 0.0136 0.0108 0.0249 0.00738 0.00982 0.00302 0.00543
2R:17393560-17393710:minus -32.0306 -0.981651 1.55046 5.69725 -18.449 -18.5714 -12.3486 2.15278
-0.853211 0.0172 0.0143 0.0068 0.00314 0.0107 0.0136 0.00498 0.00816 0.0251
2R:17393560-17393710:plus -23.2245 0.587156 -0.798165 3.6055 -27.1939 -17.9796 -17.4128 7.01389
-3.08257 0.00576 0.0084 0.015 0.00686 0.0267 0.00764 0.0137 0.000998 0.0455
2R:17394560-17394710:minus -32.3061 3.84404 7.3578 2.84404 -17.7857 -27.8265 -24.8991 1.55556
0.412844 0.0186 0.00255 0.000608 0.00961 0.00676 0.0498 0.0465 0.0101 0.0164
2R:17394560-17394710:plus -31.6633 -0.211009 -0.788991 9.61468 -17.7143 -26.898 -6.74312 7.75
1.90826 0.0141 0.0111 0.0149 0.000255 0.00627 0.0302 0.00159 0.000672 0.00974
2R:17394720-17394870:minus -13.6327 0.779817 0.53211 1.69725 -18.2245 -9.68367 -23.7156 5.19444
1.90826 0.00101 0.00784 0.00971 0.0163 0.00877 0.00342 0.0393 0.00241 0.00974
2R:17394720-17394870:plus -31.2143 4.01835 -0.0183486 2.66972 -18.4898 -26.602 -9.44954 4.48611
7.6789 0.0111 0.00239 0.0117 0.0106 0.0116 0.0248 0.00278 0.00328 0.000866
2R:17395340-17395490:minus -22.0408 -4.13761 4.65138 3.66055 -26.1939 -27.3061 -20.211 -4.94444
12.2752 0.00296 0.0351 0.00206 0.00666 0.0175 0.0336 0.0214 0.0609 0.0000141
2R:17395340-17395490:plus -32.3265 0.743119 -1.30275 2.62385 -19.0102 -18.3061 -16 -2.20833 -0.486239
0.0187 0.00794 0.0175 0.0108 0.0157 0.0114 0.0108 0.0315 0.0219
2R:17406520-17406670:minus -14.7857 12.8532 3.21101 1.3945 -9.16327 -0.173469 -16.055 4.81944
-2.99083 0.00158 0.0000252 0.00366 0.0184 0.00249 0.000456 0.011 0.00284 0.0445
2R:17406520-17406670:plus -22.9286 12.3761 2.89908 1.78899 -18.2245 -16.051 -17.7982 -4.27778 1.80734

0.0046 0.0000356 0.00413 0.0158 0.00877 0.00408 0.0145 0.0525 0.0102
2R:17409760-17409910:minus -32.4388 13.4592 4.47706 2.7156 -9.2449 -9.23469 -17.6789 3.41667
3.31193 0.0195 0.000064 0.00221 0.0102 0.00278 0.00238 0.0142 0.00508 0.00618
2R:17409760-17409910:plus -24.3367 -3.37615 -1.85321 9.59633 -8.60204 -7.53061 -1.31193 12.1389 -0.201835
0.00789 0.0286 0.0207 0.000275 0.00125 0.000625 0.00038 0.000027 0.0199
2R:17494440-17494590:minus -21.9286 6.18349 4.58716 9.44954 -27.2347 -19.051 -7.80734 2.91667
7.72477 0.0027 0.000972 0.00211 0.000326 0.0283 0.0165 0.00202 0.00616 0.000798
2R:17494440-17494590:plus -23.0816 -2.10092 -0.568807 7.27523 -27.1939 -9.86735 -22.0183 -0.625
10.1284 0.00522 0.02 0.0139 0.00109 0.0267 0.00391 0.0296 0.0202 0.000129
2R:17495700-17495850:minus -20.7755 -3.15596 7.45872 7.61468 -18.3469 -18.0816 -5.65138 -3.26389 1
0.00176 0.0269 0.000579 0.000779 0.00894 0.00934 0.00119 0.0413 0.0143
2R:17495700-17495850:plus -33.1122 -3.2844 0.431193 0.779817 -27.5 -17.3469 -14.1284 0.875 3.42202
0.0271 0.0279 0.01 0.0219 0.0337 0.0058 0.00746 0.0127 0.00597
2R:17500480-17500630:minus -22.1837 6.42202 5.25688 9.87156 -8.37755 -27.3367 -7.68807 -4.19444
1.14679 0.00315 0.000867 0.0016 0.000195 0.00106 0.0347 0.00197 0.0515 0.0136
2R:17500480-17500630:plus -22.9592 5.6055 6.83486 6.29358 -17.9286 -26.5612 1.36697 0.486111 7.15596
0.00475 0.00126 0.000785 0.00202 0.0071 0.0237 0.000199 0.0144 0.00124
2R:17504340-17504490:minus -34.0714 -1.74312 1.7156 2.61468 10.6224 19.3061 -7.6055 6.19444
-2.6055 0.031 0.018 0.00642 0.0109 0.0000113 5.97e-07 0.00194 0.00151 0.04
2R:17504340-17504490:plus -42.1531 1.51376 -0.330275 6.00917 -27.4592 -8.94898 -22.2018 -0.680556
-2.42202 0.0701 0.006 0.0129 0.00255 0.0329 0.00203 0.0306 0.0206 0.0378
2R:17511900-17512050:minus -30.4388 5.25688 -0.963303 3.30275 -17.3469 -26.6327 -20.0183 -0.472222
-0.504587 0.00923 0.00146 0.0158 0.00789 0.00394 0.0251 0.0207 0.0193 0.0221
2R:17511900-17512050:plus -21.8469 5.7156 -0.798165 3.05505 -18.1531 -27.3367 -17.3486 3.56944
3.10092 0.00247 0.0012 0.015 0.00878 0.00829 0.0347 0.0135 0.00478 0.00674
2R:17512460-17512610:minus -23.5306 -1.66972 2.93578 0.0458716 -9.23469 -18.2347 -23.2661 1.11111
-0.816514 0.00646 0.0176 0.00407 0.0266 0.00277 0.0104 0.0366 0.0117 0.0249
2R:17512460-17512610:plus -22.1122 -0.834862 12.6606 3.59633 1.80612 -9.7551 -18.4495 4.01389
6.95413 0.00304 0.0136 0.000027 0.00693 0.0000557 0.00358 0.016 0.004 0.00136
2R:17522140-17522290:minus -14.1429 0.155963 2.63303 5.33028 -26.9694 0.826531 -3.51376 9.23611
3.83486 0.0012 0.00977 0.00457 0.00389 0.023 0.000153 0.000652 0.000275 0.00481
2R:17522140-17522290:plus -31.9286 6.3211 3.24771 9.7156 -7.85714 -19.0816 -13.2752 -0.833333
5.44954 0.0166 0.000909 0.00361 0.000242 0.000594 0.017 0.00616 0.0215 0.00253
2R:17529340-17529490:minus -32.9592 -1.44954 4.62385 6.02752 -27.898 -8.53061 -15.5229 2.75 1.90826
0.0252 0.0165 0.00208 0.00246 0.04 0.00136 0.00994 0.00656 0.00974
2R:17529340-17529490:plus -21.9286 0.697248 10.6147 2.66055 -26.9286 -17.9796 -15.2569 1.06944 -2.36697
0.0027 0.00807 0.0000988 0.0106 0.0214 0.00764 0.00944 0.0119 0.0371
2R:17533620-17533770:minus -13.5204 -3.3578 6.83486 7.37615 -18.7143 -17.8265 -0.706422 1.44444
7.83486 0.00097 0.0284 0.000785 0.000951 0.0136 0.00699 0.000333 0.0105 0.000712
2R:17533620-17533770:plus -23.8878 4.3945 -3.82569 7.70642 -18.0816 -9.68367 9.08257 -2.18056 3.29358
0.00692 0.00207 0.0362 0.000708 0.00759 0.00342 0.0000175 0.0313 0.00623
2R:17545500-17545650:minus -30.3571 10.3211 5.73394 1.48624 -17.449 -26.8265 -2.42202 3.31944
4.83486 0.0089 0.000114 0.0013 0.0177 0.00484 0.0285 0.000491 0.00528 0.00329
2R:17545500-17545650:plus -23.8163 10.3945 9.07339 6.02752 -8.60204 -7.5 -1 4.27778 -7.04587 0.00682
0.000109 0.000243 0.00246 0.00125 0.000614 0.000355 0.00358 0.114
2R:17547480-17547630:minus -23.6224 3.83673 -0.605505 3.27523 10.3878 -28.4082 -15.1927 -1.69444
-1.23853 0.00658 0.00115 0.0141 0.00796 0.0000174 0.0615 0.00932 0.0274 0.0279
2R:17547480-17547630:plus -14.6429 13.5306 -1.13761 7.78899 -8.04082 -18.2347 -13.6881 -0.347222
1.17431 0.0015 0.0000206 0.0167 0.000642 0.000831 0.0104 0.00676 0.0186 0.0134
2R:17549460-17549610:minus -23.0714 3.90816 1.53211 0.376147 -17.7449 -18.5612 -9.50459 -3.90278
5.81651 0.0052 0.00102 0.00685 0.0244 0.0063 0.0132 0.00281 0.0481 0.00204
2R:17549460-17549610:plus -21.398 -0.33945 0.183486 6.12844 -26.8571 -8.45918 -19.3211 -1.45833 0.642202
0.00197 0.0116 0.0109 0.00231 0.0198 0.00111 0.0184 0.0257 0.0157
2R:17551780-17551930:minus -5.23469 -0.137615 6.48624 5.01835 -27.2653 -16.9796 -23.5688 5.16667

3.16514 0.00023 0.0108 0.000925 0.00438 0.0302 0.0044 0.0384 0.00244 0.00656
2R:17551780-17551930:plus -23 -0.0275229 0.53211 0.697248 -18.449 -17.9796 -11.7615 8.38889 5.23853
0.00498 0.0104 0.00971 0.0224 0.0107 0.00764 0.00438 0.000466 0.00285
2R:17554200-17554350:minus -33.0714 0.761468 1.02752 2.57798 -17.7551 0.683673 -17.945 2.51389
-0.770642 0.0267 0.00789 0.00819 0.011 0.00658 0.000184 0.0148 0.00716 0.0245
2R:17554200-17554350:plus -32.0408 -2.25688 5.57798 3.59633 -17.449 -17.2755 -7.76147 -1.29167 -2.40367
0.0174 0.0209 0.00139 0.00693 0.00484 0.00544 0.002 0.0245 0.0376
2R:17557560-17557710:minus -23.7755 -3.16514 -3.11009 6.54128 -17.8163 -17.3469 -16.422 -3.19444
3.45872 0.00678 0.027 0.0297 0.00173 0.00682 0.0058 0.0117 0.0406 0.00591
2R:17557560-17557710:plus -11.8469 4.9633 7.23853 2.66055 -18.1939 -0.173469 -4.30275 -1.51389
7.63303 0.000279 0.00165 0.000646 0.0106 0.0086 0.000456 0.000813 0.0261 0.000928
2R:17572920-17573070:minus -39.9184 0.266055 0.917431 1.7156 -17.3469 -18.5 -25.7064 3.54167
-0.733945 0.0344 0.0094 0.00851 0.0162 0.00394 0.0131 0.0516 0.00483 0.0242
2R:17572920-17573070:plus -31.4694 -6.98165 4.30275 3.27523 -8.93878 -37.1531 -13.6239 -5.45833 -2.36697
0.0124 0.0727 0.00237 0.00796 0.00216 0.138 0.00667 0.068 0.0371
2R:17578560-17578710:minus -14.449 0.743119 -2.06422 2.06422 -26.7449 -16.9796 -2.68807 4.95833
5.08257 0.0014 0.00794 0.022 0.0139 0.0195 0.0044 0.000525 0.00267 0.00303
2R:17578560-17578710:plus -23.2347 4.48624 0.605505 7.70642 -17.7143 -8.42857 -15.0367 -4.375 1.85321
0.00577 0.00199 0.00946 0.000708 0.00627 0.00104 0.00904 0.0537 0.01
2R:17578980-17579130:minus -42.8571 2.29358 -2.76147 2.62385 -27.2653 -0.173469 -21.3761 7.73611
-6.55963 0.0873 0.00451 0.027 0.0108 0.0302 0.000456 0.0263 0.000677 0.102
2R:17578980-17579130:plus -31.7041 4.52294 1.0367 2.70642 20.2041 0.826531 -26.0917 -2.625 -2.34862
0.0146 0.00197 0.00816 0.0103 3.64e-07 0.000153 0.0541 0.0351 0.0368
2R:17588840-17588990:minus -22.5918 5.80734 4.12844 1.40367 -18.1837 -27.3776 -17.1927 3.33333
12.2752 0.00365 0.00116 0.00254 0.0184 0.00835 0.0351 0.0132 0.00525 0.0000141
2R:17588840-17588990:plus -24.2551 1.46789 -0.431193 3.86239 -18.4592 -9.23469 -2.76147 0.930556
3.44954 0.00768 0.0061 0.0133 0.00622 0.0109 0.00238 0.000534 0.0124 0.00594
2R:17589400-17589550:minus -34.0408 0.59633 13.0183 0.53211 -17.449 -9.79592 -15.8073 9.875
3.66972 0.0309 0.00837 0.0000203 0.0234 0.00484 0.00377 0.0105 0.000179 0.00514
2R:17589400-17589550:plus -24.4082 0.0458716 3.65138 2.81651 -19.3163 -9.72449 -22.5596 7.91667
-2.90826 0.00797 0.0101 0.00308 0.00982 0.0165 0.00352 0.0326 0.000611 0.0435
2R:17590380-17590530:minus -22.3776 -4.66055 -0.926606 10.0275 -17.3776 -8.60204 -15.3578 -4.26389
0.513761 0.00342 0.0403 0.0156 0.000156 0.00419 0.00151 0.00963 0.0523 0.0162
2R:17590380-17590530:plus -23.3265 -0.495413 -0.688073 2.07339 -18.2245 -25.5612 -14.6881 1.58333
5.58716 0.00607 0.0122 0.0145 0.0138 0.00877 0.0206 0.00842 0.00997 0.00237
2R:17591000-17591150:minus -33.5612 -2.41284 1.99083 2.63303 -27.8673 -8.65306 -25.4587 7.30556
1.74312 0.0288 0.0219 0.0058 0.0107 0.0391 0.00156 0.05 0.000857 0.0105
2R:17591000-17591150:plus -22.5102 -4.08257 1.57798 5.01835 -18.1837 -9.53061 -15.0734 12.125 3
0.00354 0.0346 0.00674 0.00438 0.00835 0.0031 0.00911 0.0000274 0.00702
2R:17591400-17591550:minus -24.7449 13.4592 4.83486 7.6055 -18.9796 -9.09184 -20.211 4.61111
-0.633028 0.00827 0.000064 0.00191 0.000794 0.0155 0.00219 0.0214 0.00311 0.0232
2R:17591400-17591550:plus -32 -2.77982 -0.908257 2.6789 -18.449 -17.9796 -17.2202 0.472222 3.41284
0.0171 0.0243 0.0155 0.0105 0.0107 0.00764 0.0133 0.0144 0.006
2R:17592140-17592290:minus -31.1429 -3.55046 5.79817 2.01835 -8.85714 -17.0102 -13.0367 1.77778
-2.22018 0.0108 0.03 0.00126 0.0141 0.00165 0.00453 0.00583 0.00932 0.0355
2R:17592140-17592290:plus -31.4082 -1.11009 4.58716 4.0367 -8.67347 -9.53061 -2.17431 3.83333 3.66972
0.0119 0.0148 0.00211 0.00578 0.00154 0.0031 0.000463 0.0043 0.00514
2R:17596060-17596210:minus -21.6327 1.30275 -3.04587 3.61468 -18.1939 -18.9796 -12.6789 -8.91667
0.0275229 0.00215 0.00649 0.0292 0.0068 0.0086 0.0155 0.00537 0.13 0.0183
2R:17596060-17596210:plus -22.551 -1.04587 0.376147 7.61468 -17.7143 1.02041 -14.3394 -3.94444 -1.48624
0.00359 0.0145 0.0102 0.000779 0.00627 0.000118 0.00782 0.0486 0.0293
2R:17599100-17599250:minus -23.1939 7.7156 7.98165 10.5872 -18.6837 -8.42857 -17.4862 1.80556
10.0367 0.00558 0.000445 0.000442 0.000115 0.0126 0.00104 0.0138 0.00923 0.000146
2R:17599100-17599250:plus -31.9286 -0.00917431 6.15596 1.69725 -27.1939 -9.30612 -16.3211 2.91667

2.11009	0.0166	0.0103	0.00108	0.0163	0.0267	0.00257	0.0115	0.00616	0.00898
2R:17600720-17600870:minus	-24.1429	5.38532	-0.788991	2.78899	-8.93878	-19.0816	-14.5046	5.80556
0.917431	0.0074	0.00139	0.0149	0.00992	0.00216	0.017	0.0081	0.00182	0.0146
2R:17600720-17600870:plus	-3.81633	0.963303	2.74312	5.57798	-8.96939	-8.53061	-5.46789	-0.638889
3.20183	0.000106	0.00734	0.00438	0.0034	0.00226	0.00136	0.00113	0.0203	0.0065
2R:17601500-17601650:minus	-13.8878	5.3578	9.23853	3.26606	-18.1939	-18.6327	-16.5138	0.444444
11.8532	0.00111	0.0014	0.00022	0.00802	0.0086	0.0137	0.0118	0.0145	0.0000428
2R:17601500-17601650:plus	-13.3367	2.22018	1.94495	9.59633	-26.6327	9.64286	-14.5046	-0.0694444
9.66055	0.0008	0.00463	0.0059	0.000275	0.0188	0.0000222	0.0081	0.0171	0.000268
2R:17602260-17602410:minus	-31.5102	-0.633028	-3.07339	0.201835	-18.6429	-17.9796	-17.3028	5.375
3.14679	0.013	0.0127	0.0294	0.0255	0.0121	0.00764	0.0134	0.00222	0.00666
2R:17602260-17602410:plus	-31.4796	4.21101	1.2844	9.59633	-18.3776	1.09184	-13.9633	-3.76389	1.52294
0.0128	0.00222	0.00748	0.000275	0.00926	0.000091	0.0072	0.0466	0.0117
2R:17607460-17607610:minus	-32.8469	0.944954	-0.155963	6.17431	-17.6735	-8.5	-17.3945	3.31944
3.3211	0.0243	0.00739	0.0122	0.0022	0.00572	0.00126	0.0136	0.00528	0.00616
2R:17607460-17607610:plus	-32.6633	1.38532	0.495413	8.61468	0.918367	-9.53061	-24.4954	-0.833333
-3.08257	0.0216	0.00629	0.00983	0.000552	0.000142	0.0031	0.0441	0.0215	0.0455
2R:17607880-17608030:minus	-41.5816	13.3878	0.146789	5.47706	-19.051	0.0510204	-20.0917	8.09722
1.69725	0.061	0.0000869	0.011	0.0036	0.0162	0.000349	0.0209	0.000552	0.0108
2R:17607880-17608030:plus	-22.7347	-0.87156	-1.11009	5.47706	-17.4898	-16.9796	-4.3211	-0.569444
0.669725	0.00403	0.0138	0.0165	0.0036	0.0053	0.0044	0.000817	0.0199	0.0155
2R:17759220-17759370:minus	-20.9592	2.44037	6.22936	6.25688	-17.9286	-17.7143	-4.98165	6.06944
9.23853	0.00184	0.00427	0.00104	0.00208	0.0071	0.00634	0.000988	0.0016	0.000397
2R:17759220-17759370:plus	-3.07143	-0.321101	0.917431	3.93578	-18.3776	-8.57143	-9.31193	-1.84722
1.94495	0.0000441	0.0115	0.00851	0.00597	0.00926	0.00149	0.00271	0.0286	0.00968
2R:17860640-17860790:minus	-32.9286	-0.761468	7.29358	3.97248	-27.2347	-8.7551	-5.47706	7.29167
7.73394	0.025	0.0133	0.000628	0.00587	0.0283	0.00172	0.00114	0.000863	0.000784
2R:17860640-17860790:plus	-14.1429	6.86239	2.0367	6.52294	-8.93878	-17.1224	-13.4404	0.0972222
-3.66972	0.0012	0.000697	0.0057	0.00174	0.00216	0.00499	0.00639	0.0162	0.0514
2R:17927080-17927230:minus	-4.44898	3.47706	1.01835	5.45872	-27.1224	-19.2041	-9.90826	-2.40278
2.20183	0.000152	0.00292	0.00821	0.00363	0.0245	0.0179	0.00303	0.0332	0.00869
2R:17927080-17927230:plus	-32.2041	-1.47706	6.88991	0.100917	-26.9286	-27.5306	3.08257	1.69444	5.38532
0.0181	0.0166	0.000765	0.0261	0.0214	0.0374	0.000115	0.0096	0.00267
2R:17948320-17948470:minus	-41.2551	-1.41284	3.3945	3.49541	-18.9388	-8.72449	-26.6697	2.83333
1.70642	0.0513	0.0163	0.00341	0.00716	0.0151	0.00169	0.0581	0.00635	0.0108
2R:17948320-17948470:plus	-30.9184	13.3878	-1.30275	3.45872	-27.4082	9.53061	-15.3303	4.54167	7.83486
0.0103	0.0000869	0.0175	0.00731	0.0321	0.0000316	0.00958	0.0032	0.000712
2R:17963340-17963490:minus	-34.6327	0.724771	-1.52294	2.6789	-17.9388	-7.72449	-23.156	3.56944
-6.48624	0.032	0.008	0.0187	0.0105	0.00715	0.00068	0.036	0.00478	0.1
2R:17963340-17963490:plus	-13.9184	-0.587156	6.33945	6.21101	-8.93878	-9.45918	-0.788991	-0.708333
3.14679	0.00112	0.0126	0.000991	0.00213	0.00216	0.00279	0.000339	0.0207	0.00666
2R:17966120-17966270:minus	-21.7449	-1.19266	1.91743	4.22018	-25.9694	-36.449	-20.0183	0.513889
4.88073	0.00236	0.0152	0.00596	0.00531	0.017	0.109	0.0207	0.0142	0.00324
2R:17966120-17966270:plus	-22.1531	-1.00917	-0.889908	3.6422	-19.2755	-27.898	-12.1009	2.05556
2.6789	0.0031	0.0144	0.0154	0.00669	0.0164	0.0537	0.00472	0.00845	0.00763
2R:17966860-17967010:minus	-31.7449	6.33028	3.29358	9.49541	-7.86735	-6.79592	-27.5046	3.29167	-3
0.0152	0.000904	0.00354	0.000308	0.000617	0.000546	0.0645	0.00533	0.0447
2R:17966860-17967010:plus	-31.4796	4.6055	-2.86239	0.0550459	-18.4184	-26.602	-16.6972	-1.47222
4.78899	0.0128	0.00191	0.0277	0.0265	0.0101	0.0248	0.0122	0.0258	0.00336
2R:17967600-17967750:minus	-21.7755	2.44954	2.33945	2.83486	-9.16327	-18.5714	-16.2844	-4.05556
-0.697248	0.00241	0.00425	0.0051	0.0097	0.00249	0.0136	0.0114	0.0499	0.0239
2R:17967600-17967750:plus	-34.1837	1.18349	-0.981651	2.06422	0.806122	-9.53061	-11.0642	5.69444
3.66055	0.0314	0.00677	0.0159	0.0139	0.000229	0.0031	0.00378	0.00191	0.00515
2R:17990520-17990670:minus	-31.4796	15.5963	-0.862385	5.30275	10.7245	-9.53061	-4.06422	0.138889

0.642202 0.0128 8.91e-07 0.0153 0.00396 5.2e-06 0.0031 0.00076 0.016 0.0157
2R:17990520-17990670:plus -32.8163 -2.33945 -3.05505 0.0550459 11.6939 0.683673 -16.5688 0.361111
-1.50459 0.024 0.0214 0.0293 0.0265 1.17e-06 0.000184 0.012 0.0149 0.0294
2R:18002000-18002150:minus -32.2143 -1 2.55046 6.57798 -7.64286 -17.0102 -21.7156 -2.45833 -0.366972
0.0182 0.0143 0.00471 0.00167 0.000499 0.00453 0.028 0.0337 0.021
2R:18002000-18002150:plus -23.8061 0.458716 0.688073 2.52294 -8.96939 -18.2755 -13.156 -0.208333
1.54128 0.0068 0.00879 0.0092 0.0113 0.00226 0.0112 0.00599 0.0178 0.0115
2R:18003840-18003990:minus -13.8061 1.52294 3.40367 10.7615 -26.1939 -18.8265 11.6147 3.11111
4.91743 0.00108 0.00598 0.0034 0.0000826 0.0175 0.0148 6.73e-06 0.00572 0.00321
2R:18003840-18003990:plus -33.9286 1.2844 6.77064 3.74312 -26.2653 -36.9286 -15.9908 1.54167 6.22018
0.0303 0.00653 0.000807 0.00651 0.018 0.123 0.0108 0.0101 0.00165
2R:18009980-18010130:minus -21.8878 10.8073 1.3211 1.06422 -27.2245 -9.94898 -15.7523 1.61111
6.17431 0.00258 0.0000883 0.00738 0.0203 0.0272 0.00393 0.0104 0.00988 0.00171
2R:18009980-18010130:plus -22.2551 -2.83486 2.51376 1.57798 -17.4184 -18.2347 4.56881 5.04167 -0.33945
0.00327 0.0246 0.00478 0.0171 0.00457 0.0104 0.0000671 0.00258 0.0209
2R:18014980-18015130:minus -12.1837 3.05505 0.678899 5.98165 10.6531 -9.53061 -12.5413 -2.70833
1.00917 0.000365 0.00341 0.00923 0.00258 0.0000102 0.0031 0.00521 0.0359 0.0143
2R:18014980-18015130:plus -32.8061 7.29358 -0.376147 11.2936 -19.2755 -8.53061 -12.2844 2.08333
-2.52294 0.0237 0.000556 0.0131 0.0000139 0.0164 0.00136 0.00491 0.00837 0.0391
2R:18021480-18021630:minus -31.6224 2.0367 -0.0917431 2.12844 -18.7857 -18.3469 -10.4954 1.81944
5.6055 0.0137 0.00495 0.0119 0.0134 0.0148 0.0124 0.00338 0.00919 0.00232
2R:18021480-18021630:plus -31.7347 0.192661 11.5138 5.90826 -27.4592 -19.2755 -3.48624 -2.625 6.02752
0.0149 0.00965 0.0000585 0.00271 0.0329 0.0185 0.000647 0.0351 0.00183
2R:18024400-18024550:minus -31.3265 2.94495 -0.220183 1.3578 -18.1122 -18.5714 -6.83486 3.70833
2.41284 0.0114 0.00355 0.0125 0.0186 0.00771 0.0136 0.00162 0.00452 0.0082
2R:18024400-18024550:plus -12.8878 0.724771 -1.62385 6.44037 -8.37755 -9.68367 0.201835 8.04167 10.3394
0.000523 0.008 0.0193 0.00185 0.00106 0.00342 0.00027 0.00057 0.000112
2R:18062240-18062390:minus -14.4388 1.29358 4.05505 2.86239 -27.602 -9.53061 -11.2202 -4.11111
-0.366972 0.00137 0.00651 0.00262 0.00954 0.0364 0.0031 0.0039 0.0505 0.021
2R:18062240-18062390:plus -30.7755 -4.0367 5.3578 4.43119 20.4694 -6.72449 -21.3761 -0.611111
-7.07339 0.0102 0.0342 0.00153 0.00495 1.65e-07 0.000544 0.0263 0.0201 0.115
2R:18070260-18070410:minus -32.0408 1.7156 7.16514 6.73394 10.6531 -8.38776 -23.0459 2.65278
-2.38532 0.0174 0.00557 0.000671 0.00152 0.0000102 0.00101 0.0353 0.0068 0.0374
2R:18070260-18070410:plus -41.551 -1.48624 4.53211 -0.577982 -27.3061 -17.2755 -25.3945 3.81944
2.75229 0.0607 0.0167 0.00216 0.0321 0.0315 0.00544 0.0496 0.00433 0.00748
2R:18073380-18073530:minus -32.2959 6.2844 0.330275 2.12844 -18.7143 -8.45918 -4 -1.875 5.44954
0.0186 0.000926 0.0104 0.0134 0.0136 0.00111 0.000746 0.0288 0.00253
2R:18073380-18073530:plus -23.8776 13.3878 5.72477 7.43119 -18.7143 -19.0816 -17.7798 2.09722 -5.08257
0.00687 0.0000869 0.0013 0.000939 0.0136 0.017 0.0145 0.00832 0.0741
2R:18080680-18080830:minus -12.6224 -5.01835 1.3578 9.51376 -26.898 -27.5612 -8.75229 0.555556
1.44037 0.000453 0.0443 0.00728 0.000292 0.0203 0.0393 0.00244 0.014 0.012
2R:18080680-18080830:plus -32.8469 0.201835 5.17431 4.33028 -18.0816 -9.45918 -2.55963 -0.805556
6.6422 0.0243 0.00962 0.00165 0.00511 0.00759 0.00279 0.000508 0.0213 0.00148
2R:18081980-18082130:minus -30.7347 -2.90826 -0.59633 3.87156 -18.7857 -8.72449 -19.8624 -1.76389
-2.13761 0.01 0.0251 0.0141 0.00618 0.0148 0.00169 0.0201 0.028 0.0345
2R:18081980-18082130:plus -24.2959 4.26606 0.651376 9.49541 -18.7143 -17.1224 -10.2569 4.91667 -2.27523
0.00781 0.00217 0.00932 0.000308 0.0136 0.00499 0.00323 0.00272 0.036
2R:18084760-18084910:minus -22.7755 2.19266 -1.22936 3.20183 -8.56122 -17.1224 -10.3578 8.75 5.55046
0.00412 0.00468 0.0171 0.00824 0.00112 0.00499 0.00329 0.000374 0.00241
2R:18084760-18084910:plus -12.3367 1.38532 -1.85321 4.56881 -8.30612 -8.65306 -0.12844 3.68056 1.59633
0.000393 0.00629 0.0207 0.0048 0.00097 0.00156 0.000292 0.00457 0.0113
2R:18086600-18086750:minus -22.5204 1.94495 0.238532 2.69725 -8.60204 -18.9796 -7.16514 -0.0416667
5.65138 0.00356 0.00512 0.0107 0.0104 0.00125 0.0155 0.00175 0.017 0.00228
2R:18086600-18086750:plus -13.3673 -1.77982 3.18349 6.31193 -7.89796 -17.7857 -10.4771 0.138889 -0.577982

0.000813 0.0182 0.0037 0.00199 0.000678 0.0068 0.00337 0.016 0.0227
2R:18100040-18100190:minus -32.9694 3.3945 15.4862 6.29358 -18.449 -8.5 -13.8257 -0.847222
3.08257 0.0254 0.00301 7.92e-07 0.00202 0.0107 0.00126 0.00698 0.0216 0.00681
2R:18100040-18100190:plus -13.1122 8.6422 -3.84404 2.11009 10.7245 -18.2041 -7.21101 1.41667 3.66972
0.000632 0.00027 0.0364 0.0136 5.2e-06 0.0103 0.00177 0.0106 0.00514
2R:18100400-18100550:minus -12 4.14679 3.09174 6.21101 -17.3776 0.0918367 -20.1835 5.91667
-6.41284 0.000305 0.00228 0.00383 0.00213 0.00419 0.000323 0.0213 0.00172 0.0983
2R:18100400-18100550:plus -24.449 4.29358 7.21101 5.48624 -28.2347 -26.602 -9.22936 11.7222 3.05505
0.00802 0.00215 0.000655 0.00357 0.051 0.0248 0.00267 0.0000403 0.00692
2R:18131220-18131370:minus -33.602 4.92661 0.614679 3.76147 -27.5306 0.72449 -16.3211 7.47222
3.50459 0.0289 0.00168 0.00943 0.00645 0.0351 0.000175 0.0115 0.000783 0.00568
2R:18131220-18131370:plus -22.2245 -4.88073 9.01835 10.5872 -8.40816 -26.6735 -13.0367 -4.88889 9.23853
0.00325 0.0428 0.000251 0.000115 0.00108 0.0256 0.00583 0.0602 0.000397
2R:18185380-18185530:minus -42.4388 -2.50459 0.706422 3.75229 -17.9388 0.0204082 -11.8257 1.375
-1.47706 0.0766 0.0225 0.00914 0.00649 0.00715 0.000377 0.00444 0.0107 0.0292
2R:18185380-18185530:plus -33.4388 2.11927 -0.33945 5.73394 -18.5612 -8.5 -14.3945 1.33333 -0.220183
0.0284 0.00481 0.0129 0.00307 0.0119 0.00126 0.00791 0.0109 0.0201
2R:18198840-18198990:minus -12.1429 2.76147 -1.88073 6.36697 -17.4898 -27.3367 -2.77064 -2.18056
1.95413 0.000343 0.0038 0.0209 0.00193 0.0053 0.0347 0.000536 0.0313 0.00964
2R:18198840-18198990:plus -31.2551 -1.44037 3.98165 2.3578 -7.96939 -27.8265 -22.1835 0.152778 9.9633
0.0113 0.0164 0.0027 0.0121 0.000785 0.0498 0.0305 0.016 0.000171
2R:18199460-18199610:minus -10.6224 -1.17431 -0.137615 7.27523 -17.7551 -17.2755 -7.45872 -6.18056
1.54128 0.000234 0.0151 0.0121 0.00109 0.00658 0.00544 0.00187 0.0789 0.0115
2R:18199460-18199610:plus -30.551 0.0917431 -0.788991 -0.431193 0.836735 -16.0102 -14.8073
10.1667 1.23853 0.00959 0.00999 0.0149 0.0306 0.000215 0.00406 0.00863 0.000145 0.0132
2R:18204900-18205050:minus -32.7041 2.50459 -1.40367 1.99083 -18.7551 -8.86735 -14.1376 5.23611
3.83486 0.0222 0.00417 0.0181 0.0144 0.0144 0.00198 0.00748 0.00236 0.00481
2R:18204900-18205050:plus -32.7041 13.4592 8.48624 10.7615 -27.1939 -17.3061 -19.7982 -2.16667 -5.12844
0.0222 0.000064 0.000336 0.0000826 0.0267 0.00548 0.0199 0.0312 0.0748
2R:18205560-18205710:minus -41.551 0.761468 4.89908 1.9633 -18.9796 -10.0204 -17.2477 -1.61111
3.66972 0.0607 0.00789 0.00185 0.0146 0.0155 0.00399 0.0133 0.0268 0.00514
2R:18205560-18205710:plus -24.1531 1.45872 8.75229 7.21101 -17.1531 -16.051 -12.0275 -3.66667 1.79817
0.00744 0.00613 0.000289 0.0011 0.00364 0.00408 0.00464 0.0455 0.0102
2R:18210500-18210650:minus -29.7449 1.00917 5.59633 3.04587 -18.9796 -19.4184 -18.2936 0.513889
9.56881 0.00849 0.00722 0.00138 0.00883 0.0155 0.0198 0.0156 0.0142 0.000303
2R:18210500-18210650:plus -40.5204 1.85321 8.66055 6.73394 -18.7857 -27.9286 -17.8807 6.02778 3.94495
0.0413 0.0053 0.000305 0.00152 0.0148 0.054 0.0147 0.00164 0.00452
2R:18260660-18260810:minus -22.7755 1.46789 -2.15596 5.57798 -18.7245 -8.72449 -8.02752 -1.26389
3.88073 0.00412 0.0061 0.0226 0.0034 0.0136 0.00169 0.00212 0.0243 0.00475
2R:18260660-18260810:plus -32.4796 1.77064 2.42202 2.34862 -17.0408 -0.173469 -11.3945 4.79167
8.10092 0.02 0.00546 0.00495 0.0121 0.00329 0.000456 0.00405 0.00288 0.000623
2R:18262480-18262630:minus -32.1939 1.80734 1.17431 9.59633 -9.5 9.57143 -20.789 2.31944 1.33028
0.0181 0.00539 0.00778 0.000275 0.00293 0.0000274 0.0237 0.00769 0.0127
2R:18262480-18262630:plus -22.6531 13.4592 -4.00917 2.77064 -16.6735 -17.9388 -2.91743 0.75 2.33028
0.00374 0.000064 0.0381 0.01 0.00313 0.00738 0.000556 0.0132 0.00836
2R:18299000-18299150:minus -31.6939 -3.86239 2.45872 10.0275 -8.93878 -9.53061 -14.1376 -3.88889
-3.13761 0.0142 0.0326 0.00488 0.000156 0.00216 0.0031 0.00748 0.048 0.0461
2R:18299000-18299150:plus -13.602 13.4592 1.7156 6.00917 -27.4592 1.09184 -20.0917 2.73611 -4.27523
0.000995 0.000064 0.00642 0.00255 0.0329 0.000091 0.0209 0.00659 0.0596
2R:18303180-18303330:minus -22.9184 6.63303 -0.40367 3.44037 -18.1939 -27.0408 -7.59633 3.73611
-0.449541 0.00447 0.000782 0.0132 0.00746 0.0086 0.0307 0.00193 0.00447 0.0216
2R:18303180-18303330:plus -12.9592 5.3945 0.0366972 3.76147 -26.9286 -36.1122 -7.14679 1.45833
6.17431 0.000551 0.00138 0.0115 0.00645 0.0214 0.0878 0.00175 0.0104 0.00171
2R:18307860-18308010:minus -31.3673 14.0917 2.18349 2.61468 -18.4184 -18.0102 -20.7798 -3.56944

3.3211 0.0116 7.62e-06 0.0054 0.0109 0.0101 0.00812 0.0236 0.0445 0.00616
2R:18307860-18308010:plus -32.4796 15.0183 4.09174 2.11927 -17.6837 -26.3367 -12.7064 3.09722 3.58716
0.02 1.96e-06 0.00258 0.0135 0.0058 0.0221 0.00541 0.00575 0.00534
2R:18308440-18308590:minus -41.551 1.90826 0.321101 0.899083 -26.5306 -10.0918 -17.0734 3.55556
-7.00917 0.0607 0.00519 0.0104 0.0212 0.0185 0.00404 0.013 0.00481 0.113
2R:18308440-18308590:plus -23.2959 2.33945 1.90826 7 -18.4898 -19.2755 -17.9083 6.11111 5.80734
0.00605 0.00443 0.00598 0.00132 0.0116 0.0185 0.0147 0.00157 0.00205
2R:18314460-18314610:minus -21.2959 1.27523 0.275229 4.30275 -26.898 -9.5 -10.6972 3.05556 3.49541
0.00192 0.00655 0.0106 0.00517 0.0203 0.00294 0.00352 0.00584 0.0057
2R:18314460-18314610:plus -30.6327 -0.46789 -1.17431 7.61468 -18.4184 1.02041 -17.5688 2.90278 -1.22018
0.00968 0.0121 0.0169 0.000779 0.0101 0.000118 0.014 0.00619 0.0278
2R:18370820-18370970:minus -30.5102 -0.495413 3.91743 9.49541 -26.7041 -7.42857 -22.7339 2.83333
-4.98165 0.00947 0.0122 0.00277 0.000308 0.0193 0.000567 0.0335 0.00635 0.0725
2R:18370820-18370970:plus -32.7347 14.0459 6.51376 7.10092 -8.93878 -7.79592 -19.578 1.5 3.66972
0.0225 7.98e-06 0.000912 0.00121 0.00216 0.000723 0.0192 0.0103 0.00514
2R:18379580-18379730:minus -42.0816 -1.85321 -0.110092 5.57798 -17.7551 -0.173469 -21.7064
4.56944 -0.59633 0.0694 0.0186 0.012 0.0034 0.00658 0.000456 0.028 0.00317 0.0229
2R:18379580-18379730:plus -14.4796 13.5306 6.15596 7 -18.4898 -9.42857 -5.36697 0.916667 -4.76147
0.00142 0.0000206 0.00108 0.00132 0.0116 0.0027 0.0011 0.0125 0.0686
2R:18380-18530:minus -24.7041 -2.14679 -1.30275 2.74312 -18.7857 -9.5 -27.7064 6.875 -3.9633 0.00826
0.0203 0.0175 0.0101 0.0148 0.00294 0.0662 0.00107 0.0551
2R:18380-18530:plus -23.3367 0.972477 2.38532 2.93578 -8.60204 -9.5 -20.2202 6.09722 7.16514 0.00614
0.00731 0.00501 0.00927 0.00125 0.00294 0.0214 0.00158 0.00122
2R:18427180-18427330:minus -30.2551 -4.11009 11.6697 4.6422 -8.71429 -18.051 -20.422 6.83333
4.13761 0.00885 0.0348 0.0000536 0.00473 0.00159 0.00886 0.0222 0.0011 0.00408
2R:18427180-18427330:plus -21.9184 0.183486 7.10092 9.92661 -17.6429 -17.5714 2.76147 -1.23611 -2.26606
0.00261 0.00968 0.000693 0.000169 0.00562 0.00616 0.000128 0.0241 0.0359
2R:18434180-18434330:minus -21.7755 0.706422 3.88073 11.2936 -17.0408 -9.16327 -18.6881 -3.72222
4.09174 0.00241 0.00805 0.00281 0.0000139 0.00329 0.00223 0.0166 0.0461 0.00416
2R:18434180-18434330:plus -31.8776 -0.541284 1.19266 7.80734 -18.1531 -28.1122 -7.99083 -1.02778
1.02752 0.0163 0.0124 0.00773 0.00063 0.00829 0.0565 0.0021 0.0227 0.0141
2R:18437440-18437590:minus -30.2143 1.07339 5.18349 9.7156 -18.4184 -18.1531 -9.11927 3.98611
-1.44037 0.0088 0.00705 0.00165 0.000242 0.0101 0.00997 0.00262 0.00404 0.029
2R:18437440-18437590:plus -30.5102 -0.357798 5.09174 10.0275 -18.7857 -8.45918 3.26606 -2.98611
9.23853 0.00947 0.0116 0.00171 0.000156 0.0148 0.00111 0.000108 0.0385 0.000397
2R:18441560-18441710:minus -13.2653 7.91743 -5.21101 2.77064 -26.8571 -8.38776 -22.9083 -0.0694444
-9.78899 0.000759 0.000399 0.0521 0.01 0.0198 0.00101 0.0345 0.0171 0.187
2R:18441560-18441710:plus -30.4388 -2.40367 4.76147 2.88991 -7.93878 -19.4184 -14.156 3.08333 1.74312
0.00923 0.0218 0.00196 0.00942 0.000767 0.0198 0.00751 0.00578 0.0105
2R:18449460-18449610:minus -39.9592 5.05505 8.7156 2.55046 0.877551 -18.2041 -15.5505 -7.76389
-3.13761 0.0348 0.00159 0.000296 0.0112 0.00019 0.0103 0.00999 0.106 0.0461
2R:18449460-18449610:plus -22.6224 -3.68807 14.422 7.29358 -17.7143 -18.1224 -10.7523 -2.375 7.6422
0.00371 0.0311 4.18e-06 0.00104 0.00627 0.00988 0.00355 0.0329 0.000893
2R:18457400-18457550:minus -23.8469 -1.21101 -3.6789 3.17431 -18.6837 -17.7551 -5.38532 -0.986111
11.3853 0.00685 0.0153 0.0348 0.00842 0.0126 0.00643 0.00111 0.0225 0.0000815
2R:18457400-18457550:plus -31.8163 3.02752 -0.321101 3.13761 -18.4898 -8.5 -12.8257 -3.70833 -2.89908
0.016 0.00344 0.0129 0.00854 0.0116 0.00126 0.00555 0.046 0.0434
2R:18474440-18474590:minus -22.1122 2.25688 1.95413 9.7156 -17.1531 -17.6429 -20.9083 8.91667
11.8073 0.00304 0.00457 0.00588 0.000242 0.00364 0.00619 0.0242 0.000338 0.0000538
2R:18474440-18474590:plus -41.0612 0.53211 -2.52294 4.81651 -28.1224 -19.2755 -8.23853 4.41667 2.75229
0.0466 0.00856 0.0252 0.00451 0.0457 0.0185 0.00221 0.00338 0.00748
2R:18480460-18480610:minus -30.4388 -0.550459 3.33028 6.00917 -7.89796 -9.7551 -8.18349 2.69444
-3.14679 0.00923 0.0124 0.00349 0.00255 0.000678 0.00358 0.00218 0.00669 0.0463
2R:18480460-18480610:plus -42.3673 -0.275229 -4.82569 -2.06422 -18.3776 -0.469388 -27.211 3.63889

4.0367	0.0755	0.0113	0.0472	0.0523	0.00926	0.000533	0.0621	0.00465	0.00424
2R:18493220-18493370:minus	-24.1837	15.4037	-3.07339	4.86239	-19.051	9.60204	-23.844	4.88889
5.70642	0.00751	1.16e-06	0.0294	0.00449	0.0162	0.0000248	0.0401	0.00276	0.00222
2R:18493220-18493370:plus	-32.5918	2.54128	10.9266	2.33945	-8.82653	0.0510204	-18.2752	-2.54167
-0.0183486	0.021	0.00411	0.0000837	0.0122	0.00163	0.000349	0.0156	0.0344	0.0185
2R:18493540-18493690:minus	-32.1429	4.09174	-2.41284	1.42202	-28.3061	-8.79592	-15.8807	4.29167
1.70642	0.0179	0.00232	0.0244	0.0182	0.0553	0.0019	0.0106	0.00356	0.0108
2R:18493540-18493690:plus	-32.5408	-2.77064	1.88991	2.88991	10.6939	-8.27551	-9.83486	-5.05556	0.816514
0.0204	0.0242	0.00602	0.00942	7.35e-06	0.000919	0.00298	0.0624	0.015
2R:18495680-18495830:minus	-33.2245	-2.74312	7.38532	3.27523	-9.16327	-9.7551	-23.0092	6.54167
-5.10092	0.0276	0.024	0.0006	0.00796	0.00249	0.00358	0.0351	0.00127	0.0743
2R:18495680-18495830:plus	-33.8163	2.36697	1.19266	2.41284	-17.3776	-19.2347	-24.5321	1.09722	-1.11927
0.0299	0.00439	0.00773	0.0118	0.00419	0.018	0.0443	0.0118	0.027
2R:18525160-18525310:minus	-31.7755	14.2936	11.0917	3.14679	-8.67347	-8.23469	-17.9358	-2.06944
-0.605505	0.0157	6.04e-06	0.0000753	0.00847	0.00154	0.000875	0.0148	0.0304	0.023
2R:18525160-18525310:plus	-23.3367	11.0459	12.1468	4.0367	-7.71429	-9.34694	-12.7156	2.70833	-1.14679
0.00614	0.0000789	0.0000388	0.00578	0.000572	0.00261	0.00542	0.00666	0.0272
2R:18527520-18527770:minus	-23.1837	15.0183	-0.174312	3.77064	-17.7857	-17.9388	-15.4037	-0.319444
-0.385321	0.00553	1.96e-06	0.0123	0.00637	0.00676	0.00738	0.00972	0.0184	0.0211
2R:18527520-18527770:plus	-34.0408	-0.0733945	2.6789	7.27523	-27.0408	-9.09184	-19	-0.0555556
-0.0183486	0.0309	0.0106	0.00449	0.00109	0.0242	0.00219	0.0175	0.017	0.0185
2R:18528880-18529030:minus	-14.5918	4.69725	-1.79817	1.55963	-18.449	-18.0102	-19.6881	4.27778
0.550459	0.00148	0.00184	0.0204	0.0173	0.0107	0.00812	0.0195	0.00358	0.0161
2R:18528880-18529030:plus	-23.1939	0.0642202	6.76147	4.04587	-18.9388	-7.79592	-20.156	1.33333
-4.33945	0.00558	0.0101	0.000811	0.00572	0.0151	0.000723	0.0212	0.0109	0.0609
2R:18531500-18531650:minus	-13.449	2.79817	-2.08257	10.0275	-17.3776	-8.45918	-6.82569	0.458333
6.80734	0.000914	0.00374	0.0222	0.000156	0.00419	0.00111	0.00162	0.0145	0.0014
2R:18531500-18531650:plus	-31.7755	0.706422	2.76147	9.7156	-17.3469	-16.9796	-12	5.04167	1.34862
0.0157	0.00805	0.00435	0.000242	0.00394	0.0044	0.00461	0.00258	0.0125
2R:18533780-18533930:minus	-22.3673	6.53211	0.330275	11.2936	-27.3061	-18.8571	-18.7156	0.611111
0.183486	0.00341	0.000822	0.0104	0.0000139	0.0315	0.0151	0.0167	0.0138	0.0176
2R:18533780-18533930:plus	-31.4796	11.0367	4.51376	3.14679	-7.89796	-7.94898	-13.8349	0.305556	2.36697
0.0128	0.0000797	0.00218	0.00847	0.000678	0.00074	0.00699	0.0152	0.00828
2R:1855260-1855410:minus	-32.8469	11.1376	-1.58716	-2.77982	-18.4898	-18.6327	-15.7248	4.22222	7.94495
0.0243	0.0000754	0.0191	0.0654	0.0116	0.0137	0.0103	0.00367	0.000667
2R:1855260-1855410:plus	-32.4694	3.68367	-2.90826	-0.807339	0.581633	-16.7857	-8.77064	1.55556
-0.0733945	0.0196	0.00144	0.0281	0.0345	0.000292	0.00426	0.00245	0.0101	0.0188
2R:18558200-18558350:minus	-22.3367	-0.834862	10.5046	3.79817	-18.4898	-8.72449	-7	-2.13889
-0.275229	0.00339	0.0136	0.000105	0.00629	0.0116	0.00169	0.00169	0.0309	0.0206
2R:18558200-18558350:plus	-24.4898	15.2202	0.211009	2.72477	-27.898	-8.5	-16.3761	-0.194444	1.54128
0.00807	1.59e-06	0.0108	0.0102	0.04	0.00126	0.0116	0.0178	0.0115
2R:18568480-18568630:minus	-22.3673	-0.66055	1.08257	3.47706	-18.4898	-45.7755	0.816514	3.81944
7.22018	0.00341	0.0129	0.00803	0.00724	0.0116	0.244	0.000231	0.00433	0.00119
2R:18568480-18568630:plus	-21.6633	6.02752	3	10.0275	-26.5918	-9.23469	-6.00917	-9.95833	1.48624
0.00223	0.00104	0.00397	0.000156	0.0186	0.00238	0.00131	0.153	0.0118
2R:18573680-18573830:minus	-31.2857	-2.25688	2.45872	5.59633	-18.5612	-8.79592	-14.8349	9.93056
-0.440367	0.0113	0.0209	0.00488	0.00331	0.0119	0.0019	0.00868	0.000172	0.0216
2R:18573680-18573830:plus	-30.7041	4.25688	5.82569	7.47706	-18.3061	0.897959	-22.5963	1.52778	-5.88073
0.00993	0.00218	0.00125	0.00085	0.00884	0.000144	0.0328	0.0102	0.0866
2R:18574340-18574490:minus	-29.398	7.58716	3.90826	7.88991	-26.9694	-9.72449	-17.2294	-2.04167
5.87156	0.00839	0.000476	0.00278	0.00062	0.023	0.00352	0.0133	0.0301	0.00199
2R:18574340-18574490:plus	-21.2143	-0.422018	6.85321	7.90826	-18.1531	-9.27551	-11.8257	7.55556
3.56881	0.00189	0.0119	0.000779	0.000611	0.00829	0.00244	0.00444	0.000748	0.00543
2R:18576480-18576630:minus	-20.8163	-3.65138	1.77064	9.59633	-18.2245	0.0918367	-3.51376	0.5

1.27523 0.00177 0.0308 0.00629 0.000275 0.00877 0.000323 0.000652 0.0143 0.013
2R:18576480-18576630:plus -10.8163 -0.256881 2.44954 10.0275 -8.60204 -8.27551 -3.75229 -3.47222
7.83486 0.000236 0.0113 0.00489 0.000156 0.00125 0.000919 0.000696 0.0434 0.000712
2R:18588420-18588570:minus -30.3673 13.5306 1.26606 3.94495 -26.898 -18.0816 -7.80734 4 -1.26606
0.00897 0.0000206 0.00753 0.00595 0.0203 0.00934 0.00202 0.00402 0.0281
2R:18588420-18588570:plus -32.5102 13.5229 -0.0733945 0.522936 -26.3061 1.02041 5.52294 3.69444
9.66055 0.0202 0.000014 0.0119 0.0234 0.0181 0.000118 0.0000482 0.00455 0.000268
2R:1861840-1861990:minus -14.3673 13.4592 2.2844 5.22936 -26.2653 -17.051 -25.1101 1.31944 9.45872
0.00132 0.000064 0.0052 0.0041 0.018 0.00479 0.0478 0.0109 0.000337
2R:1861840-1861990:plus -31.7347 -1.78899 7.87156 5.22018 -18.4592 -18.6429 -7.3945 5.09722 3.46789
0.0149 0.0183 0.000469 0.00418 0.0109 0.0139 0.00185 0.00251 0.00582
2R:1868240-1868390:minus -22.9286 13.3878 -3.40367 3.18349 -27.5612 0.0510204 -23.1009 -0.0972222
1.95413 0.0046 0.0000869 0.0323 0.00837 0.0354 0.000349 0.0357 0.0172 0.00964
2R:1868240-1868390:plus -34.1531 3 -2.13761 -1.88991 -27.5306 -7.79592 -22.7064 7.98611 7.68807
0.0314 0.00348 0.0225 0.0493 0.0351 0.000723 0.0334 0.000588 0.000841
2R:18686300-18686450:minus -23.2551 2.00917 7.24771 5.49541 -7.85714 -18.2755 -10.2385 4.08333
2.46789 0.00583 0.005 0.000644 0.00355 0.000594 0.0112 0.00322 0.00388 0.00805
2R:18686300-18686450:plus -23.0102 -1.58716 0.587156 4.75229 -17.8878 -18.051 -15.1651 -4.38889 9.66055
0.00499 0.0172 0.00952 0.00461 0.00704 0.00886 0.00927 0.0538 0.000268
2R:18686880-18687030:minus -28.9898 7.7156 -1.65138 3.06422 -18.7551 -8.45918 -9.44954 -0.736111
2.6789 0.00832 0.000445 0.0195 0.00874 0.0144 0.00111 0.00278 0.0209 0.00763
2R:18686880-18687030:plus -23.2653 -5.02752 0.348624 1.44954 -18.7551 -9.27551 -15.7523 -2.41667 0.0275229
0.00593 0.0444 0.0103 0.0179 0.0144 0.00244 0.0104 0.0333 0.0183
2R:18694100-18694250:minus -13.7857 2.04587 -3.93578 -0.53211 -18.4592 -28.6735 -15.7706 0.875
11.4771 0.00108 0.00494 0.0373 0.0314 0.0109 0.0667 0.0104 0.0127 0.0000679
2R:18694100-18694250:plus -22.1122 2.46789 3.51376 3.46789 -26.1939 -9.5 -15.9541 -1 2.0367 0.00304
0.00422 0.00325 0.00726 0.0175 0.00294 0.0108 0.0226 0.00923
2R:18694440-18694590:minus -14.5612 3.45872 3.12844 3.54128 -17.0816 -18.3469 -12.1193 1.15278
-2.26606 0.00147 0.00294 0.00378 0.00702 0.00334 0.0124 0.00473 0.0115 0.0359
2R:18694440-18694590:plus -23.2245 13.5306 -0.724771 3.95413 -26.8571 -18.0102 -17.6239 6.94444
-0.477064 0.00576 0.0000206 0.0146 0.00593 0.0198 0.00812 0.0141 0.00103 0.0218
2R:1869560-1869710:minus -23 0.12844 1.42202 1.48624 -17.602 -8.5 -26.3853 -0.0694444 -3.81651
0.00498 0.00987 0.00712 0.0177 0.00547 0.00126 0.0561 0.0171 0.0529
2R:1869560-1869710:plus -22.9286 4.43119 1.78899 3.13761 -17.1837 -18.5 -16.8807 0.138889 5.38532
0.0046 0.00204 0.00625 0.00854 0.00366 0.0131 0.0126 0.016 0.00267
2R:18741280-18741430:minus -32.4388 2.61468 10.7156 3.70642 -27.7041 -27.7041 -15.7982 0.819444 2
0.0195 0.004 0.000094 0.0066 0.038 0.0445 0.0105 0.0129 0.00941
2R:18741280-18741430:plus -33.4082 1.08257 1.01835 10.1468 -18.4898 -9.53061 -25.8624 8.40278 -0.183486
0.0283 0.00703 0.00821 0.000128 0.0116 0.0031 0.0526 0.000462 0.0197
2R:18741740-18741890:minus -33.3673 -4.44954 9.22936 6.43119 0.877551 -17.0816 -11.7706 -1.45833
-4.7156 0.0282 0.0381 0.000221 0.00187 0.00019 0.00487 0.00439 0.0257 0.0678
2R:18741740-18741890:plus -23.0408 1.3211 -1.44954 2.61468 1.10204 0.979592 -22.3578 4.125 -1.59633
0.00512 0.00644 0.0183 0.0109 0.000124 0.000136 0.0314 0.00382 0.0301
2R:18770200-18770350:minus -22.7041 3.61468 11.4128 2.78899 11.6939 9.79592 -20.0367 4.38889
-3.82569 0.00395 0.00277 0.0000621 0.00992 1.17e-06 0.0000183 0.0207 0.00342 0.0531
2R:18770200-18770350:plus -41.8163 3.64286 -0.284404 -0.284404 -18.7143 -8.86735 -20.2844 1.83333
-0.220183 0.0662 0.00159 0.0127 0.0291 0.0136 0.00198 0.0216 0.00914 0.0201
2R:18773520-18773670:minus -21.4286 1.58716 10.4679 1.24771 -26.898 -18.4184 -15.633 -5.59722
-0.816514 0.00198 0.00584 0.000108 0.0192 0.0203 0.0127 0.0101 0.0701 0.0249
2R:18773520-18773670:plus -22.0306 -0.449541 -1.86239 2.34862 -27.898 -18.051 -12.789 -1.31944
1.44037 0.00293 0.012 0.0208 0.0121 0.04 0.00886 0.00551 0.0247 0.012
2R:18774040-18774190:minus -22.7041 4.0367 2.55046 2.92661 -8.86735 -18.7857 -19.1651 3.61111
3.55963 0.00395 0.00237 0.00471 0.00931 0.00173 0.0146 0.0179 0.0047 0.00545
2R:18774040-18774190:plus -30.1429 -0.119266 3.37615 4.05505 -26.9694 -17.7551 -20.055 -0.597222

-3.52294 0.00866 0.0107 0.00343 0.00566 0.023 0.00643 0.0208 0.0201 0.0499
2R:18776360-18776510:minus -23.8571 -0.669725 12.4771 5.91743 0.908163 -18.5612 -17.8624 6.47222
2.01835 0.00687 0.0129 0.0000306 0.00267 0.000153 0.0132 0.0146 0.00132 0.00928
2R:18776360-18776510:plus -33.2347 0.321101 1.79817 2.88073 20.2041 -8.72449 -24.3211 -1.55556 1.07339
0.0276 0.00922 0.00623 0.00945 3.64e-07 0.00169 0.043 0.0264 0.014
2R:18799540-18799690:minus -12.3367 -2.08257 -1.92661 2.61468 -16.3776 -18.9796 -19.6514 -3.63889
-2.63303 0.000393 0.0199 0.0212 0.0109 0.003 0.0155 0.0194 0.0452 0.0404
2R:18799540-18799690:plus -31.6224 -3.11009 -0.688073 2.51376 -26.5306 -45.6633 3.47706 -5.70833
7.94495 0.0137 0.0266 0.0145 0.0113 0.0185 0.238 0.0000998 0.0717 0.000667
2R:18812440-18812590:minus -32.8163 0.513761 1.95413 4.12844 -17.4184 -26.898 -15.6606 0.736111
-0.541284 0.024 0.00862 0.00588 0.00549 0.00457 0.0302 0.0102 0.0132 0.0223
2R:18812440-18812590:plus -5.26531 3.90816 -0.357798 3.45872 -18.449 -16.7143 -10.7156 1.47222
3.72477 0.000231 0.00102 0.013 0.00731 0.0107 0.00419 0.00353 0.0104 0.00497
2R:18813200-18813350:minus -22.7041 0.376147 2.25688 4.44954 -8.70408 -8.20408 -14.6972 6.69444
0.954128 0.00395 0.00905 0.00525 0.00492 0.00155 0.000796 0.00843 0.00118 0.0145
2R:18813200-18813350:plus -31.9694 1.92661 1.66972 2.78899 -16.3776 -9.02041 -12.4312 -2.83333 -1.90826
0.0168 0.00516 0.00652 0.00992 0.003 0.00213 0.00508 0.0371 0.0324
2R:18821680-18821830:minus -30.2143 -2.11009 3.12844 3.36697 -18.1939 -24.8265 -15.2936 5.18056
6.37615 0.0088 0.0201 0.00378 0.00764 0.0086 0.0204 0.00951 0.00242 0.00159
2R:18821680-18821830:plus -23.1429 -5.2844 0.174312 -1.22018 -18.4898 -25.5306 -13.3853 1.09722 9.56881
0.00533 0.0475 0.0109 0.0394 0.0116 0.0205 0.00631 0.0118 0.000303
2R:18822240-18822390:minus -22.0408 -4.42202 3.33945 10.8624 -17.5612 -9.53061 -12.0183 1.76389
1.6422 0.00296 0.0379 0.00348 0.0000687 0.00544 0.0031 0.00463 0.00937 0.0111
2R:18822240-18822390:plus -32.7857 2.65138 1.30275 0.321101 -28.2041 -18.051 -25.5688 -2.09722 2.20183
0.0235 0.00395 0.00743 0.0247 0.0483 0.00886 0.0507 0.0306 0.00869
2R:18824580-18824730:minus -21.898 3.90816 2.07339 6.76147 10.9184 -18.051 -6.42202 5.625
1.01835 0.00259 0.00102 0.00562 0.00148 4.19e-06 0.00886 0.00146 0.00198 0.0142
2R:18824580-18824730:plus -22.9694 10.0092 1.86239 11.1927 -26.898 -18.0816 -20.5321 1.68056 1.77982
0.0048 0.000132 0.00608 0.0000254 0.0203 0.00934 0.0226 0.00964 0.0103
2R:18825080-18825230:minus -24.0306 10.578 0.788991 1.46789 -17.1531 -9.57143 -24.367 3.23611
-3.07339 0.00723 0.0001 0.00889 0.0179 0.00364 0.00326 0.0433 0.00545 0.0454
2R:18825080-18825230:plus -21.6633 -1.49541 -0.688073 6.20183 -26.9694 1.12245 -19.1743 0.986111
5.48624 0.00223 0.0167 0.0145 0.00214 0.023 0.000085 0.0179 0.0122 0.00251
2R:18828580-18828730:minus -13.1122 4.65138 7.51376 10.7615 -17.0408 -26.4898 -20.0183 -0.708333
1.08257 0.000632 0.00187 0.000563 0.0000826 0.00329 0.0225 0.0207 0.0207 0.0139
2R:18828580-18828730:plus -14.1122 3.98165 2.6422 -1.17431 -8.93878 -17.8571 1.58716 -2.93056 10.3486
0.00119 0.00242 0.00455 0.0388 0.00216 0.00719 0.000187 0.038 0.0000988
2R:18886300-18886450:minus -21.3265 0.605505 3.10092 7.95413 -8.33673 -8.53061 -9.0367 -4.41667
0.12844 0.00193 0.00834 0.00382 0.000599 0.00101 0.00136 0.00258 0.0542 0.0179
2R:18886300-18886450:plus -22.7755 0.137615 0.266055 -0.321101 -8.89796 -36.4082 -3.90826 -0.930556
3.20183 0.00412 0.00984 0.0106 0.0294 0.00189 0.105 0.000727 0.0221 0.0065
2R:18933680-18933830:minus -21.8878 -1.02752 0.449541 1.69725 -18.0816 -7.45918 -14.3486 -3.20833
10.3486 0.00258 0.0145 0.00998 0.0163 0.00759 0.000582 0.00783 0.0407 0.0000988
2R:18933680-18933830:plus -21.8571 2.40367 5.94495 10.5872 -27.2347 -17.7857 3.20183 -1 2.73394
0.00249 0.00433 0.00118 0.000115 0.0283 0.0068 0.00011 0.0226 0.00756
2R:18936060-18936210:minus -24.0408 -1.0367 -2.49541 2.05505 -27.0102 -26.5306 -16.7523 5.54167
4.10092 0.00726 0.0145 0.025 0.0139 0.0237 0.023 0.0123 0.00205 0.00413
2R:18936060-18936210:plus -30.3673 0.53211 0.165138 3.76147 -17.8878 -9.23469 -21.7523 5.65278 0.229358
0.00897 0.00856 0.011 0.00645 0.00704 0.00238 0.0282 0.00195 0.0173
2R:18938440-18938590:minus -23.1837 0.651376 7.07339 3.26606 1.10204 -7.79592 -11.7615 1.08333
-1.59633 0.00553 0.00821 0.000703 0.00802 0.000124 0.000723 0.00438 0.0118 0.0301
2R:18938440-18938590:plus -31.7041 1.12844 5.83486 6.74312 -17.4184 -27.6327 -15.9633 0.5 -0.669725
0.0146 0.00691 0.00124 0.0015 0.00457 0.043 0.0108 0.0143 0.0236
2R:18939560-18939710:minus -30.5102 0.422018 -0.33945 4.3211 -17.8163 -27.9286 -15.3761 8.97222

1.27523 0.00947 0.0089 0.0129 0.00514 0.00682 0.054 0.00966 0.000326 0.013
2R:18939560-18939710:plus -31.4694 6.41284 1.92661 -0.944954 -17.1837 -17.7857 -7.37615 8.45833 3
0.0124 0.00087 0.00594 0.0361 0.00366 0.0068 0.00184 0.000447 0.00702
2R:18950020-18950170:minus -21.8469 10.6147 1.61468 5.86239 -18.7143 -17.9388 -12.7156 2.61111
7.68807 0.00247 0.0000977 0.00665 0.00282 0.0136 0.00738 0.00542 0.00691 0.000841
2R:18950020-18950170:plus -24.5612 5.11009 -1.68807 -0.275229 -27.898 -9.5 -19.367 9.08333 -4.77982
0.00819 0.00156 0.0197 0.029 0.04 0.00294 0.0185 0.000304 0.069
2R:18954980-18955130:minus -31.102 -0.834862 3.3211 5.90826 -8.97959 -17.2755 -18.9633 -1.18056
-1.16514 0.0106 0.0136 0.00351 0.00271 0.0023 0.00544 0.0173 0.0238 0.0274
2R:18954980-18955130:plus -13.5204 -0.972477 2 10.0275 -18.6429 -8.45918 -12.4495 1.75 7.51376
0.00097 0.0142 0.00578 0.000156 0.0121 0.00111 0.0051 0.00941 0.000984
2R:18962220-18962370:minus -23.4898 2.30275 4.82569 -0.137615 2.14286 1.02041 -2.55046 1.90278
-3.52294 0.00639 0.00449 0.00191 0.0279 0.0000316 0.000118 0.000507 0.00892 0.0499
2R:18962220-18962370:plus -33.5816 -1.27523 3.23853 3.61468 -18.7551 -17.0816 -8.08257 1.5 7.73394
0.0288 0.0156 0.00362 0.0068 0.0144 0.00487 0.00214 0.0103 0.000784
2R:18966600-18966750:minus -38.0306 -1.15596 13.3761 2.22018 -18.3776 -8.93878 -18.156 2.40278
-2.37615 0.032 0.0151 0.0000145 0.0129 0.00926 0.00201 0.0153 0.00746 0.0373
2R:18966600-18966750:plus -23.0816 10.5046 -1.78899 1.78899 -28.8265 -19.6429 -10.7248 2.43056 1.47706
0.00522 0.000104 0.0203 0.0158 0.0648 0.0204 0.00353 0.00738 0.0119
2R:1899720-1899870:minus -23.2959 3.71429 -0.990826 5.01835 -18.4898 -9.42857 -22.1009 3.66667
-5.0367 0.00605 0.00129 0.0159 0.00438 0.0116 0.0027 0.03 0.0046 0.0734
2R:1899720-1899870:plus -41.7755 -1.53211 -4.11927 -1.77982 -18.9796 -9.79592 -28.0734 3.05556 -2.15596
0.0652 0.0169 0.0392 0.0474 0.0155 0.00377 0.0695 0.00584 0.0348
2R:1899980-1900130:minus -23.2959 3.71429 -0.990826 5.01835 -18.4898 -9.64286 -22.1009 1.11111
-5.0367 0.00605 0.00129 0.0159 0.00438 0.0116 0.00339 0.03 0.0117 0.0734
2R:1899980-1900130:plus -24.2959 -3.84404 4.12844 3.52294 0.581633 0.173469 -21.6055 1.98611 -2.18349
0.00781 0.0324 0.00254 0.00706 0.000292 0.000307 0.0275 0.00866 0.0351
2R:1904480-1904630:minus -32.2653 2.3578 0.889908 2.07339 -17.7857 -9.60204 -20.7064 -0.0416667
-2.82569 0.0184 0.0044 0.00859 0.0138 0.00676 0.00334 0.0233 0.017 0.0425
2R:1904480-1904630:plus -30.4388 -3.75229 4.68807 6.21101 -8.86735 0.244898 -19.4771 -4.875 2.33028
0.00923 0.0316 0.00203 0.00213 0.00173 0.000285 0.0189 0.06 0.00836
2R:1905120-1905270:minus -32.449 -5.15596 -0.724771 3.85321 -17.7143 1.12245 -14.4954 0.597222
-0.385321 0.0195 0.046 0.0146 0.00623 0.00627 0.000085 0.00808 0.0139 0.0211
2R:1905120-1905270:plus -40.2245 1.11009 -2.69725 3.69725 -16.8878 -16.7857 -23.1101 -3.48611 -1.17431
0.0374 0.00695 0.0265 0.00665 0.00326 0.00426 0.0357 0.0436 0.0275
2R:1910660-1910810:minus -13.2959 -0.880734 -2.3211 9.7156 -26.8571 -18.0102 -13.7156
-0.875 6.80734 0.000775 0.0138 0.0237 0.000242 0.0198 0.00812 0.00681 0.0218 0.0014
2R:1910660-1910810:plus -21.6327 2.48624 -1.55046 0.0733945 -17.7551 -27.3367 -10.6881
0.847222 2.53211 0.00215 0.0042 0.0189 0.0263 0.00658 0.0347 0.00351 0.0128 0.00791
2R:19216700-19216850:minus -22.7755 0.733945 7.72477 1.83486 -18.4184 -18.2347 -1.77064
-1.93056 3.92661 0.00412 0.00797 0.000506 0.0155 0.0101 0.0104 0.000421 0.0293 0.00461
2R:19216700-19216850:plus -31.7041 1.86239 7.7156 7.6422 1.87755 -18.0816 -26.5413 -4.88889
-4.24771 0.0146 0.00528 0.000509 0.000721 0.0000492 0.00934 0.0572 0.0602 0.0591
2R:1923000-1923150:minus -32.102 -1.12844 11.7615 4.33028 -18.4592 -18.1224 -16.8991 -2.77778 -0.990826
0.0177 0.0149 0.0000506 0.00511 0.0109 0.00988 0.0126 0.0365 0.026
2R:1923000-1923150:plus -11.8878 3.88991 5.54128 3.27523 -17.0816 -27.5612 -12.2936 -4.26389 -1.3211
0.00029 0.00251 0.00141 0.00796 0.00334 0.0393 0.00492 0.0523 0.0284
2R:1923960-1924110:minus -13.4082 -2.17431 2.55046 4.75229 -17.8878 -9.30612 -15.6422 -5.65278 3.78899
0.000867 0.0204 0.00471 0.00461 0.00704 0.00257 0.0102 0.0709 0.00487
2R:1923960-1924110:plus -10.8469 -4.13761 13.1193 4.0367 -17.1531 -27.602 0.752294 -5.65278 3.88073
0.000237 0.0351 0.0000185 0.00578 0.00364 0.0416 0.000235 0.0709 0.00475
2R:19242600-19242750:minus -33.5612 1.9633 3.43119 1.69725 -17.449 -9.79592 -13.6514 5.54167
3.6055 0.0288 0.00509 0.00336 0.0163 0.00484 0.00377 0.00671 0.00205 0.00532
2R:19242600-19242750:plus -14.3673 3.11927 3.16514 0.651376 -8.93878 -26.7551 -12.633 2.23611 3.20183

0.00132	0.00333	0.00373	0.0226	0.00216	0.0263	0.00532	0.00792	0.0065
2R:19244860-19245010:minus	-31.551	-1.26606	7.48624	4.47706	-17.449	0.0510204	-5.17431	0.805556
-3.3211	0.0134	0.0156	0.00057	0.00488	0.00484	0.000349	0.00104	0.013	0.0478
2R:19244860-19245010:plus	-32.6633	4.45872	1.97248	1.92661	-19.0102	-18.6429	-18.6514	2.88889	-0.440367
0.0216	0.00202	0.00584	0.0148	0.0157	0.0139	0.0165	0.00622	0.0216
2R:19246740-19246890:minus	-12.5204	1.21101	2.0367	5.34862	-7.60204	-18.6429	-12.3211	2.51389
-2.61468	0.000441	0.00671	0.0057	0.00387	0.000466	0.0139	0.00495	0.00716	0.0401
2R:19246740-19246890:plus	-29.8776	-1.3578	2.22018	5.49541	-17.1531	-19.1531	-10.3761	-0.791667
3.52294	0.00852	0.016	0.00532	0.00355	0.00364	0.0177	0.0033	0.0212	0.00558
2R:19253840-19253990:minus	-4.59184	9.22936	5	7.21101	-26.9286	-9.86735	-9.40367	4.11111	-4.42202
0.000166	0.000199	0.00178	0.0011	0.0214	0.00391	0.00276	0.00384	0.0626
2R:19253840-19253990:plus	-40.5918	0.733945	3.07339	9.92661	-17.6429	-0.316327	-17.6972	6.43056
8.0367	0.0422	0.00797	0.00386	0.000169	0.00562	0.000524	0.0143	0.00134	0.000654
2R:19254320-19254470:minus	-19.8571	-0.834862	3.88991	2.66972	-27.9694	-8.5	-14.4128	1.68056
4.40367	0.00164	0.0136	0.0028	0.0106	0.0437	0.00126	0.00794	0.00964	0.00373
2R:19254320-19254470:plus	-14.7449	14.2477	-0.293578	2.14679	-26.898	-0.173469	-17.9725	7.38889
4.13761	0.00157	6.21e-06	0.0128	0.0133	0.0203	0.000456	0.0149	0.000819	0.00408
2R:19264480-19264630:minus	-12.9592	14.1009	3.44037	2.07339	-26.9286	-9.72449	-2.3945	3.40278
-4.09174	0.000551	7.56e-06	0.00335	0.0138	0.0214	0.00352	0.000488	0.00511	0.0568
2R:19264480-19264630:plus	-32.4796	14.945	4.41284	4.86239	20.4694	-8.53061	-5.83486	4.11111	3.20183
0.02	2.29e-06	0.00227	0.00449	1.65e-07	0.00136	0.00125	0.00384	0.0065
2R:19270900-19271050:minus	-31.2857	-4.00917	0.330275	1.74312	10.9898	-8.27551	-22.5872	7.125
5.44037	0.0113	0.0339	0.0104	0.016	3.28e-06	0.000919	0.0327	0.000942	0.00259
2R:19270900-19271050:plus	-30.7041	13.3878	-0.954128	3.26606	-17.3776	-18.2755	-18.7982	3.02778
1.36697	0.00993	0.0000869	0.0157	0.00802	0.00419	0.0112	0.0169	0.0059	0.0124
2R:19273880-19274030:minus	-22.4082	-1.76147	4.74312	5.30275	-18.449	-7.5	-20.945	-1.22222	-4.16514
0.00345	0.0181	0.00198	0.00396	0.0107	0.000614	0.0243	0.024	0.0579
2R:19273880-19274030:plus	-14.3673	13.5306	9.74312	3.70642	-27.1939	-16.9796	-22.2477	-0.791667
-0.192661	0.00132	0.0000206	0.000166	0.0066	0.0267	0.0044	0.0308	0.0212	0.0198
2R:19284160-19284310:minus	-21.7041	9.0367	-2	0.46789	-27.2653	-0.469388	-4.07339	-0.986111
-0.954128	0.00231	0.000219	0.0216	0.0239	0.0302	0.000533	0.000762	0.0225	0.0258
2R:19284160-19284310:plus	-13.2653	-1.50459	0.0183486	5.06422	-18.3878	-26.8265	-10.5872	5.56944
11.8991	0.000759	0.0168	0.0115	0.00433	0.00931	0.0285	0.00344	0.00203	0.0000255
2R:19289780-19289930:minus	-20.6633	2.27523	13.4954	0.220183	0.877551	-27.2653	-7.85321	2.15278
1.57798	0.00173	0.00454	0.000013	0.0253	0.00019	0.0333	0.00204	0.00816	0.0114
2R:19289780-19289930:plus	-22.9286	1.54128	13.9266	7.2844	1.17347	-17.9796	-14.2569	-0.847222
3.72477	0.0046	0.00594	7.79e-06	0.00105	0.000101	0.00764	0.00768	0.0216	0.00497
2R:1930100-1930250:minus	-22.2959	0.146789	6.08257	0.477064	11.4286	-18.1224	-8.55046	2.98611	3.3211
0.00335	0.0098	0.00111	0.0238	1.97e-06	0.00988	0.00235	0.006	0.00616
2R:1930100-1930250:plus	-32	3.89908	3.43119	6.01835	-17.449	-17.051	-27.6422	6.06944	-4.3578
0.0171	0.0025	0.00336	0.00248	0.00484	0.00479	0.0656	0.0016	0.0614
2R:19316400-19316550:minus	-22.7755	-2.15596	0	6.33945	-18.1531	-18.5	-10.6055	-1.70833	9.97248
0.00412	0.0203	0.0116	0.00196	0.00829	0.0131	0.00345	0.0275	0.00016
2R:19316400-19316550:plus	-20.8571	2.17431	-2.16514	3.93578	-17.7857	-9.72449	-9.10092	3.59722	-3.08257
0.00178	0.00471	0.0227	0.00597	0.00676	0.00352	0.00261	0.00473	0.0455
2R:1938160-1938310:minus	-12.8571	-0.752294	0.944954	3.29358	-19.0102	-0.469388	-23.156	4.45833
-0.954128	0.000505	0.0132	0.00842	0.00791	0.0157	0.000533	0.036	0.00332	0.0258
2R:1938160-1938310:plus	-24.2245	0.825688	4.81651	3.54128	-18.7551	-27.5612	-18.844	-3.98611	6.26606
0.00763	0.00771	0.00192	0.00702	0.0144	0.0393	0.017	0.0491	0.00162
2R:19411080-19411230:minus	-24.2245	7.09174	16.2202	6.56881	-26.8571	-28.3367	-4.22018	2.06944
-2.52294	0.00763	0.000619	1.24e-07	0.0017	0.0198	0.0606	0.000794	0.00841	0.0391
2R:19411080-19411230:plus	-23.0714	1.37615	8.50459	3.16514	-8.93878	-27.1224	-11.0367	-2.47222	1.48624
0.0052	0.00631	0.000332	0.00843	0.00216	0.0325	0.00376	0.0338	0.0118
2R:19431560-19431710:minus	-39.9592	4.24771	3.63303	3.14679	-17.4898	-8.72449	-17.844	0.847222

5.69725 0.0348 0.00219 0.00311 0.00847 0.0053 0.00169 0.0146 0.0128 0.00225
2R:19431560-19431710:plus -21.9694 2.06422 0.651376 2.62385 -18.4184 -26.8571 -9.73394 1.16667 2.74312
0.00282 0.0049 0.00932 0.0108 0.0101 0.0288 0.00293 0.0115 0.00751
2R:19435440-19435590:minus -30.1735 5.05505 5.00917 3.57798 -18.7143 -17.8265 -21.4679 -1.54167
-2.85321 0.00869 0.00159 0.00177 0.00696 0.0136 0.00699 0.0268 0.0263 0.0429
2R:19435440-19435590:plus -21.9592 5.97248 1.2844 7.27523 -19.051 -18.1531 -11.8532 -1.41667 3.61468
0.00279 0.00107 0.00748 0.00109 0.0162 0.00997 0.00447 0.0254 0.00526
2R:19439040-19439190:minus -31.102 -4.66055 7.94495 7.37615 -9.16327 -8.42857 -12.3119 -3.72222
-10.2661 0.0106 0.0403 0.000451 0.000951 0.00249 0.00104 0.00494 0.0461 0.204
2R:19439040-19439190:plus -22.4388 0.440367 -0.889908 -0.284404 -17.5612 -45.9592 -2.36697 3.94444
8.56881 0.00348 0.00885 0.0154 0.0291 0.00544 0.276 0.000485 0.00411 0.000491
2R:19441480-19441630:minus -23.6224 -0.715596 15.4495 1.7156 -8.92857 -27.1224 -8.45872 -2.47222
-0.0825688 0.00658 0.0131 9.17e-07 0.0162 0.00194 0.0325 0.00231 0.0338 0.0189
2R:19441480-19441630:plus -30.5204 3.36697 1.65138 4.36697 -17.7551 -8.93878 -13.8624 -2.19444 2.16514
0.00951 0.00304 0.00656 0.00505 0.00658 0.00201 0.00704 0.0314 0.00879
2R:19442120-19442270:minus -12.7449 12.1193 -0.0642202 6.74312 -17.449 -18.2755 -13.6239 -0.972222
1.53211 0.000471 0.000043 0.0118 0.0015 0.00484 0.0112 0.00667 0.0224 0.0116
2R:19442120-19442270:plus -31.4388 14.4954 3.15596 2.94495 -27.5714 -8.45918 -23.6239 0.430556 1.44037
0.0122 5.09e-06 0.00374 0.0092 0.0357 0.00111 0.0388 0.0146 0.012
2R:19443720-19443870:minus -20.6327 -4.87156 12.367 3.42202 -17.7143 0.122449 -13.367 -4.02778
-1.44954 0.00171 0.0427 0.0000334 0.00751 0.00627 0.000312 0.00629 0.0496 0.0291
2R:19443720-19443870:plus -13.3776 7.81651 0.192661 7.3578 -27.1633 -18.2755 -4.14679 5.88889 -1.22018
0.000831 0.000422 0.0109 0.000965 0.025 0.0112 0.000778 0.00175 0.0278
2R:19452300-19452450:minus -22.7347 -0.137615 15.0092 1.12844 -8.96939 -9.79592 -16.5138 2.25
3.49541 0.00403 0.0108 1.55e-06 0.0198 0.00226 0.00377 0.0118 0.00788 0.0057
2R:19452300-19452450:plus -11.8163 2.66972 15.211 1.82569 -17.1122 0.0918367 -17.2018 -0.986111
9.30275 0.000272 0.00393 1.07e-06 0.0156 0.00341 0.000323 0.0132 0.0225 0.00038
2R:19468300-19468450:minus -30.2551 -2.59633 3.06422 -0.155963 -17.0408 -17.9388 -14.7156 2
4.77064 0.00885 0.023 0.00387 0.0282 0.00329 0.00738 0.00847 0.00862 0.0034
2R:19468300-19468450:plus -22.5102 -1.63303 -1.66055 6.82569 -26.2653 -17.8571 1.19266 2.18056 3.66972
0.00354 0.0174 0.0195 0.00144 0.018 0.00719 0.000209 0.00808 0.00514
2R:19546120-19546270:minus -31.8163 0.733945 6.13761 7.37615 -9.23469 -9.5 -14.9817 -1.13889 1.95413
0.016 0.00797 0.00109 0.000951 0.00277 0.00294 0.00894 0.0235 0.00964
2R:19546120-19546270:plus -22.3776 -0.926606 5.33945 11.0183 -26.9286 -0.244898 -20.9633 4.625
-3.91743 0.00342 0.014 0.00154 0.000052 0.0214 0.000496 0.0244 0.00309 0.0544
2R:19546700-19546850:minus -34.602 0.348624 6.88073 -0.605505 -27.1531 -9.16327 -20.8807 5.5
10.3394 0.032 0.00914 0.000768 0.0323 0.0249 0.00223 0.0241 0.00209 0.000112
2R:19546700-19546850:plus -22.8163 -1.55046 0.733945 1.26606 -17.4184 -0.173469 -14.4679 -2.20833
-3.34862 0.00418 0.017 0.00906 0.0191 0.00457 0.000456 0.00803 0.0315 0.048
2R:19547520-19547670:minus -22.7347 -0.981651 2.82569 5.22936 -8.67347 -19.3061 -14.6514 1.625
1.02752 0.00403 0.0143 0.00424 0.0041 0.00154 0.0187 0.00835 0.00983 0.0141
2R:19547520-19547670:plus -23.0408 -3.53211 -3.23853 0.366972 -27.3061 -9.53061 -21.6697 0.472222 1.89908
0.00512 0.0298 0.0308 0.0244 0.0315 0.0031 0.0278 0.0144 0.0098
2R:19554460-19554610:minus -31.3265 -0.12844 11.1651 3.42202 -8.64286 -37.0408 -13.6697 1.80556
3.36697 0.0114 0.0108 0.000072 0.00751 0.00139 0.126 0.00674 0.00923 0.00607
2R:19554460-19554610:plus -30.2143 -0.211009 13.0459 10.5872 -7.60204 -17.7857 -12.3119 -7.66667
-0.862385 0.0088 0.0111 0.0000199 0.000115 0.000466 0.0068 0.00494 0.105 0.0251
2R:19555420-19555570:minus -21.4796 0.963303 -4.19266 0.53211 -17.4184 -26.3776 -16.1376 -1.01389
3.19266 0.00202 0.00734 0.04 0.0234 0.00457 0.0222 0.0111 0.0227 0.00652
2R:19555420-19555570:plus -38.9184 -2.15596 -1.93578 5.82569 -26.1531 -26.5612 -14.5688 -2.97222 1.48624
0.0323 0.0203 0.0212 0.00291 0.0172 0.0237 0.00821 0.0384 0.0118
2R:19581400-19581550:minus -23.449 13.4592 -1.23853 7.70642 -17.3061 -8.5 -21.844 3.375 -0.926606
0.00631 0.000064 0.0172 0.000708 0.00389 0.00126 0.0287 0.00516 0.0256
2R:19581400-19581550:plus -33.1429 -0.137615 13.6422 7.12844 -18.7449 -18.1531 -17.9358 -3.29167

3.88073 0.0272 0.0108 0.0000116 0.00118 0.0138 0.00997 0.0148 0.0416 0.00475
2R:19585320-19585470:minus -23.0408 1.58716 2.6422 6.84404 -26.8163 -18.7143 -13.578 3.27778
4.80734 0.00512 0.00584 0.00455 0.0014 0.0195 0.014 0.0066 0.00536 0.00334
2R:19585320-19585470:plus -40.1837 2.33945 15.7339 7.29358 -8.97959 -26.5612 -12.8899 7.25 9.61468
0.0367 0.00443 2.45e-07 0.00104 0.0023 0.0237 0.00564 0.000882 0.00029
2R:19589880-19590030:minus -23.4184 -0.944954 11.7064 10.0275 1.17347 -9.5 -24.3486 -1.88889
-0.0733945 0.00624 0.0141 0.0000523 0.000156 0.000101 0.00294 0.0432 0.0289 0.0188
2R:19589880-19590030:plus -22 6.0367 6.2844 6.16514 20.4694 -18.6429 -23.3394 -2.73611 0.394495
0.00291 0.00104 0.00102 0.00223 1.65e-07 0.0139 0.0371 0.0362 0.0166
2R:19594480-19594630:minus -12.9592 8.9633 -0.146789 3.18349 -17.3367 -18.0816 -6.68807 3.29167
5.59633 0.000551 0.000228 0.0122 0.00837 0.00391 0.00934 0.00156 0.00533 0.00234
2R:19594480-19594630:plus -20.398 -0.504587 -0.825688 2.78899 10.6531 -17.7857 -17.4587 -3.54167
11.8532 0.00166 0.0122 0.0151 0.00992 0.0000102 0.0068 0.0138 0.0442 0.0000428
2R:19597260-19597410:minus -31.7755 13.6239 14.8532 7 -18.4184 -9.02041 -19.6422 -3.08333 2.73394
0.0157 0.000013 2.28e-06 0.00132 0.0101 0.00213 0.0194 0.0395 0.00756
2R:19597260-19597410:plus -14.1429 14.9266 1.16514 6.49541 -8.60204 -8.45918 -12.2661 2.38889 3.44954
0.0012 2.37e-06 0.0078 0.00176 0.00125 0.00111 0.00489 0.00749 0.00594
2R:19599820-19599970:minus -13.1837 -0.0366972 -1.52294 5.57798 -19.051 -18.0102 -14.4495 6.23611
1.89908 0.000705 0.0104 0.0187 0.0034 0.0162 0.00812 0.008 0.00148 0.0098
2R:19599820-19599970:plus -30.4388 -3.85321 2.23853 1.26606 -17.4592 -18.1224 -17.8991 -2.45833 -0.981651
0.00923 0.0325 0.00529 0.0191 0.00491 0.00988 0.0147 0.0337 0.026
2R:19601380-19601530:minus -23.8469 3.83673 13.8257 3.3578 -18.1224 -27.3776 -6.99083 -2.375
10.3486 0.00685 0.00115 8.99e-06 0.00767 0.00782 0.0351 0.00168 0.0329 0.0000988
2R:19601380-19601530:plus -3.26531 3.97959 7.22936 10.8624 -18.6429 -9.45918 -8.74312 -5.11111 5.66055
0.0000515 0.000669 0.000649 0.0000687 0.0121 0.00279 0.00244 0.0632 0.00227
2R:19611660-19611810:minus -20.3673 -1.85321 6.15596 3.21101 -18.4184 -17.5 -26.8073 3.52778
-1.47706 0.00166 0.0186 0.00108 0.00822 0.0101 0.00599 0.0591 0.00486 0.0292
2R:19611660-19611810:plus -23.1837 -2.15596 10.4771 5.6789 -8.33673 -26.7551 -7.02752 -1.02778 1.16514
0.00553 0.0203 0.000107 0.00315 0.00101 0.0263 0.0017 0.0227 0.0135
2R:19612260-19612410:minus -31.0408 5.68807 -2.66972 3.46789 -19.051 -17.3469 -13.1927 5.51389
-2.51376 0.0106 0.00122 0.0263 0.00726 0.0162 0.0058 0.00604 0.00208 0.039
2R:19612260-19612410:plus -24.1939 -3.84404 7.56881 7.21101 -8.40816 -9.53061 -19.1193 1.68056 0.174312
0.00753 0.0324 0.000547 0.0011 0.00108 0.0031 0.0178 0.00964 0.0177
2R:19634260-19634410:minus -3.47959 3.97959 1.65138 2.88991 -18.0816 9.89796 -22.8991 -5.48611
-7.11009 0.0000728 0.000669 0.00656 0.00942 0.00759 0.0000111 0.0345 0.0684 0.116
2R:19634260-19634410:plus -22.8163 -1.22018 -0.155963 -0.605505 -18.1939 9.82653 -13.1468 2.31944
5.6055 0.00418 0.0154 0.0122 0.0323 0.0086 0.0000148 0.00598 0.00769 0.00232
2R:1963840-1963990:minus -32 9.70642 3.29358 1.99083 -18.449 -8.86735 -18.0367 1.93056 -0.862385
0.0171 0.000155 0.00354 0.0144 0.0107 0.00198 0.015 0.00883 0.0251
2R:1963840-1963990:plus -24.3776 2.19266 -3.36697 3.74312 -17.4898 -8.64286 -12.8624 0.569444 1.75229
0.00793 0.00468 0.032 0.00651 0.0053 0.00154 0.0056 0.014 0.0104
2R:19640900-19641050:minus -21.8469 -1.0367 -2 1.78899 -17.4898 -9.09184 -11.5138 3.80556 1.27523
0.00247 0.0145 0.0216 0.0158 0.0053 0.00219 0.00415 0.00435 0.013
2R:19640900-19641050:plus -32.0408 -0.669725 -1.51376 9.08257 -27.4898 -9.86735 -21.2844 3.875
7.22018 0.0174 0.0129 0.0187 0.000444 0.0336 0.00391 0.0259 0.00423 0.00119
2R:1964300-1964450:minus -33 2.11927 -3.18349 1.79817 -27.3061 -8.7551 -9.11927 5.11111 -2.83486
0.0258 0.00481 0.0304 0.0157 0.0315 0.00172 0.00262 0.0025 0.0427
2R:1964300-1964450:plus -32.6939 -5.54128 14.1835 7.45872 -18.449 -16.9796 -13.7615 5.66667 3.05505
0.0217 0.0508 5.88e-06 0.000894 0.0107 0.0044 0.00688 0.00194 0.00692
2R:19696560-19696710:minus -23.1939 -0.12844 2.69725 1.74312 -8.20408 -9.0102 -24.2569 1.77778
2.37615 0.00558 0.0108 0.00446 0.016 0.000894 0.00208 0.0426 0.00932 0.00825
2R:19696560-19696710:plus -40.7755 -0.0642202 1.83486 3.10092 -8.67347 -8.53061 -13.789 7.43056
1.99083 0.0433 0.0105 0.00614 0.00865 0.00154 0.00136 0.00692 0.000801 0.00946
2R:19699460-19699610:minus -31.8163 0.0642202 -1.73394 5.21101 10.6939 -8.5 -20.7615 -4.52778

6.02752 0.016 0.0101 0.02 0.0042 7.35e-06 0.00126 0.0236 0.0556 0.00183
2R:19699460-19699610:plus -33.0714 -2.68807 0.724771 2.7156 -17.7143 -9.02041 -17.2936 6.66667 -4.34862
0.0267 0.0236 0.00909 0.0102 0.00627 0.00213 0.0134 0.00119 0.0612
2R:19727580-19727730:minus -41.8163 3.55963 -4.19266 -0.183486 -18.7551 -17.0102 -25.4128 2.125
-1.49541 0.0662 0.00283 0.04 0.0283 0.0144 0.00453 0.0497 0.00824 0.0293
2R:19727580-19727730:plus -20.8878 0.541284 0.201835 6.41284 -17.6837 1.05102 -12.8899 3.70833 5.90826
0.0018 0.00854 0.0108 0.00189 0.0058 0.000107 0.00564 0.00452 0.00198
2R:19730720-19730870:minus -31.398 6.80734 5.27523 7 -27.4898 -18.7857 -0.706422 -1.94444
3.47706 0.0117 0.000716 0.00158 0.00132 0.0336 0.0146 0.000333 0.0294 0.00575
2R:19730720-19730870:plus -22.398 8.17431 3.30275 1.76147 -18.1531 -18.0816 -16.9908 5.59722 6.02752
0.00344 0.000347 0.00353 0.0159 0.00829 0.00934 0.0128 0.002 0.00183
2R:1973080-1973230:minus -33.3367 6.21101 0.715596 2.62385 20.1735 -8.34694 -18.9083 5.73611 3.72477
0.0281 0.000958 0.00912 0.0108 5.68e-07 0.000993 0.0172 0.00188 0.00497
2R:1973080-1973230:plus -32.2143 14.8257 4.48624 0.00917431 -18.4592 -17.3469 -26.7431 4.125
-6.95413 0.0182 2.99e-06 0.0022 0.0269 0.0109 0.0058 0.0586 0.00382 0.112
2R:19732340-19732490:minus -32 13.3878 -0.752294 4.3211 -17.7143 -9.53061 -21.9908 5.875
-0.504587 0.0171 0.0000869 0.0148 0.00514 0.00627 0.0031 0.0294 0.00176 0.0221
2R:19732340-19732490:plus -32.8878 2.80734 2.53211 6.11009 -26.1939 -7.45918 -18.6789 5.52778 2.88073
0.0247 0.00373 0.00475 0.00233 0.0175 0.000582 0.0166 0.00207 0.00727
2R:19737760-19737910:minus -32.0714 11.0367 -0.394495 3.56881 -27.2653 -18.3776 -14.0183 -2.88889
5.6055 0.0176 0.0000797 0.0132 0.00699 0.0302 0.0125 0.00728 0.0376 0.00232
2R:19737760-19737910:plus -22.3673 13.4592 5.27523 7 -26.9694 -17.8265 -17.945 3.34722 0.394495
0.00341 0.000064 0.00158 0.00132 0.023 0.00699 0.0148 0.00522 0.0166
2R:19738600-19738750:minus -22 -3.87156 -3.2844 6.02752 -27.4082 -17.2755 -10.4128 0.944444 1.01835
0.00291 0.0327 0.0312 0.00246 0.0321 0.00544 0.00333 0.0124 0.0142
2R:19738600-19738750:plus -14.0408 5.79817 -0.853211 2.93578 -28.5714 0.530612 -9.36697 6.58333
-0.908257 0.00115 0.00116 0.0152 0.00927 0.0623 0.000203 0.00274 0.00124 0.0255
2R:19738960-19739110:minus -31.2551 -0.0550459 6.45872 7.55963 -17.7143 -18.1939 -14.0826 0.5
1.56881 0.0113 0.0105 0.000937 0.000807 0.00627 0.0101 0.00739 0.0143 0.0114
2R:19738960-19739110:plus -32.0306 0.541284 -1.0367 3.56881 -26.5306 0.244898 -14.6422 -0.472222
5.3945 0.0172 0.00854 0.0161 0.00699 0.0185 0.000285 0.00834 0.0193 0.00263
2R:19744400-19744550:minus -13.7755 15.844 -1.52294 3.20183 -17.8163 -8.27551 -11.6789 3.80556
2.20183 0.00107 3.15e-07 0.0187 0.00824 0.00682 0.000919 0.0043 0.00435 0.00869
2R:19744400-19744550:plus -21.6633 15.1376 -1.55963 1.78899 -26.3061 -17.0102 -17.2569 4.48611 1.55963
0.00223 1.81e-06 0.0189 0.0158 0.0181 0.00453 0.0133 0.00328 0.0114
2R:19749960-19750110:minus -32.9592 0.12844 -1.13761 3.33028 -27.2653 -8.57143 -9.09174 1.75 7.58716
0.0252 0.00987 0.0167 0.00778 0.0302 0.00149 0.0026 0.00941 0.000954
2R:19749960-19750110:plus -40.3265 -0.577982 -2.56881 0.752294 -9.23469 -0.469388 -29.2936 6.01389
-0.12844 0.0394 0.0125 0.0255 0.0221 0.00277 0.000533 0.0823 0.00165 0.0193
2R:19752100-19752250:minus -12.449 7.11927 3.57798 2.25688 -17.3367 -8.5 -8.9633 3.63889 2.42202
0.000428 0.000611 0.00317 0.0127 0.00391 0.00126 0.00254 0.00465 0.00816
2R:19752100-19752250:plus -31.6224 -3.09174 2.6055 6.74312 -8.89796 -17.7857 -5.88073 -3.19444 1.16514
0.0137 0.0264 0.00462 0.0015 0.00189 0.0068 0.00127 0.0406 0.0135
2R:19752420-19752570:minus -33.8163 0.422018 1.56881 5.42202 -17.4082 -25.5306 -14.0275 2.875
-3.50459 0.0299 0.0089 0.00676 0.00374 0.00421 0.0205 0.0073 0.00626 0.0497
2R:19752420-19752570:plus -21.9286 1.75229 1.46789 0.449541 -26.7041 -18.8265 -25.4495 2.08333 1.33028
0.0027 0.0055 0.007 0.024 0.0193 0.0148 0.05 0.00837 0.0127
2R:19752780-19752930:minus -31.9082 -1.94495 -1 2.69725 10.6531 -9.82653 -18.4954 1.55556 -4.3211
0.0164 0.0191 0.016 0.0104 0.0000102 0.00381 0.0161 0.0101 0.0605
2R:19752780-19752930:plus -20.3265 14.3394 -0.550459 10.0275 10.6939 9.57143 -10.6972 0.75 -4.25688
0.00165 5.96e-06 0.0139 0.000156 7.35e-06 0.0000274 0.00352 0.0132 0.0593
2R:19755960-19756110:minus -12.6224 1.68807 8.57798 7.90826 -18.449 -18.1531 -2.95413 1.30556
7.42202 0.000453 0.00563 0.000318 0.000611 0.0107 0.00997 0.000561 0.011 0.00106
2R:19755960-19756110:plus -22.9592 8.21101 10.9358 5.57798 -18.6837 -17.9388 -9.7156 0.347222 2.89908

0.00475 0.00034 0.0000828 0.0034 0.0126 0.00738 0.00292 0.015 0.00722
2R:19757740-19757890:minus -40.5816 5.49541 -4.08257 3.24771 -26.2653 -0.469388 -26.9358 5.98611
1.54128 0.0419 0.00132 0.0388 0.00808 0.018 0.000533 0.06 0.00167 0.0115
2R:19757740-19757890:plus -21.7347 0.33945 -3.15596 1.48624 -8.67347 -18.0102 -8.9633 1.36111 3.40367
0.00235 0.00917 0.0301 0.0177 0.00154 0.00812 0.00254 0.0108 0.00603
2R:19758720-19758870:minus -33.1939 -4.3578 1.53211 9.59633 -18.6429 -9.45918 -29.1468 1.65278
5.29358 0.0274 0.0372 0.00685 0.000275 0.0121 0.00279 0.0806 0.00974 0.00278
2R:19758720-19758870:plus -13.0714 0.146789 7.12844 6.47706 -18.3776 -27.2653 -1.49541 -3.29167 1.82569
0.000594 0.0098 0.000683 0.00179 0.00926 0.0333 0.000396 0.0416 0.0101
2R:19759160-19759310:minus -12.4082 -2.94495 2.0367 0.779817 -27.2347 -10.0918 -13.0275 2.72222
4.75229 0.000414 0.0254 0.0057 0.0219 0.0283 0.00404 0.00582 0.00663 0.00341
2R:19759160-19759310:plus -32.5918 -0.53211 1.20183 3.53211 -17.1531 -9.72449 -23.8899 -3.41667 7.6789
0.021 0.0123 0.0077 0.00704 0.00364 0.00352 0.0404 0.0428 0.000866
2R:19759840-19759990:minus -21.7347 6.58716 -4.26606 -1.05505 -26.9286 -35.1531 -4.51376 -4.91667
9.97248 0.00235 0.000799 0.0408 0.0374 0.0214 0.0753 0.000864 0.0606 0.00016
2R:19759840-19759990:plus -22.9592 1.97248 4.82569 0.504587 -8.71429 -19.3469 -21.6239 1.54167 9.23853
0.00475 0.00507 0.00191 0.0235 0.00159 0.0194 0.0275 0.0101 0.000397
2R:19760460-19760610:minus -22.8469 3.90816 -0.807339 4.22018 -27.1531 -17.7857 -7.42202 -0.277778
3.66972 0.00424 0.00102 0.015 0.00531 0.0249 0.0068 0.00186 0.0182 0.00514
2R:19760460-19760610:plus -13 1.94495 -3.88073 5.37615 -18.3469 -18.2755 -13.5321 -1.98611 -0.0733945
0.000563 0.00512 0.0368 0.00384 0.00894 0.0112 0.00653 0.0297 0.0188
2R:19760660-19760870:minus -31.8469 3.93578 7.38532 5.57798 -18.4898 -26.602 -9.51376 -0.694444
5.23853 0.0162 0.00246 0.0006 0.0034 0.0116 0.0248 0.00281 0.0206 0.00285
2R:19760660-19760870:plus -23.1837 -3.18349 0.284404 4.50459 -7.85714 0.0510204 1.45872 -3.15278
6.12844 0.00553 0.0271 0.0105 0.00484 0.000594 0.000349 0.000194 0.0402 0.00174
2R:19769820-19769970:minus -31.551 3.22936 7.24771 4.05505 -18.1122 -9.42857 -22.2936 1.20833
3.14679 0.0134 0.0032 0.000644 0.00566 0.00771 0.0027 0.0311 0.0113 0.00666
2R:19769820-19769970:plus -21.7755 3.52294 -0.559633 4.94495 -18.8265 -8.23469 -18.7431 1.79167
7.22018 0.00241 0.00287 0.0139 0.00444 0.0149 0.000875 0.0168 0.00928 0.00119
2R:19777900-19778050:minus -39.2959 -0.743119 1.10092 2.74312 -27.0102 -18.8571 -16.5413 1.19444
4.7156 0.0331 0.0132 0.00798 0.0101 0.0237 0.0151 0.0119 0.0114 0.00345
2R:19777900-19778050:plus -22.8469 13.4592 1.48624 4.02752 -27.1939 -9.82653 -9.3211 3.09722 1.22936
0.00424 0.000064 0.00696 0.00581 0.0267 0.00381 0.00271 0.00575 0.0132
2R:19782460-19782610:minus 5 4.22936 1.42202 6.21101 -17.1531 -8.5 -1.54128 -2.66667 -0.770642
0.0000168 0.0022 0.00712 0.00213 0.00364 0.00126 0.0004 0.0355 0.0245
2R:19782460-19782610:plus -33.4388 2.3211 3.2844 2.6055 -9.23469 -9.57143 -10.6881 5.93056 7.94495
0.0284 0.00446 0.00356 0.0109 0.00277 0.00326 0.00351 0.00171 0.000667
2R:19785780-19785930:minus -41.6633 -3.66972 0.697248 5.85321 -9.16327 -28.449 -16.7798 1.125
-0.541284 0.0636 0.031 0.00917 0.00287 0.00249 0.0616 0.0124 0.0117 0.0223
2R:19785780-19785930:plus -30.6633 3.56881 1.99083 2.50459 -8.93878 -9.5 -5.37615 0.847222 6.44954
0.00976 0.00282 0.0058 0.0114 0.00216 0.00294 0.0011 0.0128 0.00154
2R:19794440-19794590:minus -31.8878 5.22018 -1.61468 4.0367 -17.449 -18.2755 -0.110092 -3.29167
6.95413 0.0164 0.00149 0.0193 0.00578 0.00484 0.0112 0.000291 0.0416 0.00136
2R:19794440-19794590:plus -23.0714 4.87156 1.49541 7.70642 -28.2347 -19.3061 -14.5229 -3.125 1.75229
0.0052 0.00172 0.00694 0.000708 0.051 0.0187 0.00813 0.0399 0.0104
2R:19795100-19795250:minus -31.9592 13.5505 14.2477 2.78899 -26.2653 1.12245 -12.6055 -0.513889
-0.605505 0.0167 0.0000136 5.31e-06 0.00992 0.018 0.000085 0.00528 0.0196 0.023
2R:19795100-19795250:plus -41.1429 0.256881 -2.54128 1.63303 -8.89796 0.755102 -24.3486 7.81944 -2.44037
0.0477 0.00944 0.0253 0.0167 0.00189 0.000175 0.0432 0.000646 0.0381
2R:19795580-19795730:minus -33.0408 -1.02752 6.01835 1.14679 -27.2347 -18.3469 -21.5596 5.90278
6.12844 0.0264 0.0145 0.00114 0.0198 0.0283 0.0124 0.0272 0.00174 0.00174
2R:19795580-19795730:plus -30.0612 0.155963 -2.31193 5.31193 -19.0102 -8.72449 -13.9083 3.79167 -4.86239
0.00856 0.00977 0.0237 0.00393 0.0157 0.00169 0.00711 0.00437 0.0705
2R:19821000-19821150:minus -22.2245 1.45872 -1.25688 4.16514 -18.0918 -18.051 -20.211 0.986111

5.23853 0.00325 0.00613 0.0173 0.00538 0.00761 0.00886 0.0214 0.0122 0.00285
2R:19821000-19821150:plus -32 -1.11927 3.90826 0.46789 -8.63265 -9.45918 -23.8807 1.91667 5.69725
0.0171 0.0149 0.00278 0.0239 0.0013 0.00279 0.0403 0.00888 0.00225
2R:19827200-19827350:minus -31.7755 15.7706 7.33028 3.76147 -17.4898 -17.0102 -15.1927 -2.01389
-1.80734 0.0157 4.56e-07 0.000617 0.00645 0.0053 0.00453 0.00932 0.0299 0.0316
2R:19827200-19827350:plus -14.7041 14.8807 4.78899 1.78899 -18.2245 -9.5 -16.0642 5.90278 3.16514
0.00154 2.63e-06 0.00194 0.0158 0.00877 0.00294 0.011 0.00174 0.00656
2R:19827820-19827970:minus -40.2551 13.3878 -0.880734 2.21101 -19.0102 -18.4184 -22.1101 9.51389
0.146789 0.0382 0.0000869 0.0154 0.0129 0.0157 0.0127 0.0301 0.000229 0.0178
2R:19827820-19827970:plus -24.1122 -1.69725 -0.66055 3.12844 -8.64286 -8.93878 -2.90826 11.5278 3
0.00738 0.0178 0.0143 0.00857 0.00139 0.00201 0.000555 0.0000481 0.00702
2R:19828220-19828370:minus -32.5918 2.40367 -2.36697 5.76147 -16.7143 -9.45918 -9.6055 8.69444
-3.18349 0.021 0.00433 0.0241 0.00295 0.00319 0.00279 0.00286 0.000387 0.0465
2R:19828220-19828370:plus -39.9592 -2.23853 3.83486 1.26606 -26.9286 -18.9796 -13.5505 3.84722 3.42202
0.0348 0.0208 0.00287 0.0191 0.0214 0.0155 0.00656 0.00428 0.00597
2R:19829900-19830050:minus -22.4796 2.43119 2.77064 1.41284 -28.0102 -9.72449 -13.1376 1.38889
1.11009 0.00352 0.00428 0.00433 0.0183 0.0449 0.00352 0.00597 0.0107 0.0138
2R:19829900-19830050:plus -31.551 15.0183 -3.45872 2.20183 -8.63265 -19.1224 -14.6881 5.48611 -0.605505
0.0134 1.96e-06 0.0328 0.013 0.0013 0.0175 0.00842 0.00211 0.023
2R:19830780-19830930:minus -22.2245 1.79817 -2.09174 6.37615 -27.9388 -16.051 -26.2477 5.09722
-4.46789 0.00325 0.00541 0.0222 0.00192 0.0415 0.00408 0.0551 0.00251 0.0635
2R:19830780-19830930:plus -30.6633 -0.201835 2.23853 1.43119 -18.7857 -18.3469 -20.3028 10.3333
-5.66055 0.00976 0.011 0.00529 0.0181 0.0148 0.0124 0.0217 0.000128 0.0827
2R:19836780-19836930:minus -23.8163 13.4592 5.22018 1.99083 -27.2347 -8.30612 -8.22018 -1.875 -1
0.00682 0.000064 0.00162 0.0144 0.0283 0.000973 0.0022 0.0288 0.0261
2R:19836780-19836930:plus -24.6735 3.21101 -3.21101 0.926606 -17.3776 10.3061 -24.2294 4.43056 -3.52294
0.00825 0.00322 0.0306 0.0209 0.00419 7.25e-06 0.0424 0.00336 0.0499
2R:19841380-19841530:minus -31.4388 2.43119 4.75229 2.88991 11.6939 -8.86735 -12.6697 1.125
0.40367 0.0122 0.00428 0.00197 0.00942 1.17e-06 0.00198 0.00536 0.0117 0.0165
2R:19841380-19841530:plus -14.1837 6.44037 5.54128 9.33028 0.540816 -8.45918 -13.6789 4.48611 -0.697248
0.00124 0.000858 0.00141 0.000374 0.000321 0.00111 0.00675 0.00328 0.0239
2R:19844880-19845030:minus -40.2857 -3.83486 4.0367 3.45872 10.6531 -17.3469 -12.1835 -2.22222
-6.44954 0.0385 0.0324 0.00264 0.00731 0.0000102 0.0058 0.0048 0.0316 0.0993
2R:19844880-19845030:plus -30.449 -1.72477 -1.25688 1 -18.4184 -18.5612 -8.57798 -0.402778 -0.59633
0.00925 0.0179 0.0173 0.0206 0.0101 0.0132 0.00236 0.0189 0.0229
2R:19852160-19852310:minus -23.449 6.88991 0.183486 4.22018 -18.6837 -18.051 -10.4771 -1.88889
0.972477 0.00631 0.000688 0.0109 0.00531 0.0126 0.00886 0.00337 0.0289 0.0144
2R:19852160-19852310:plus -24.1837 -0.33945 1.36697 2.44037 -27.1531 -17.5 -14.4954 2.48611 -0.284404
0.00751 0.0116 0.00726 0.0116 0.0249 0.00599 0.00808 0.00723 0.0207
2R:19854460-19854610:minus -22.7041 0.247706 -4.12844 2.27523 -18.0102 -17.6429 -21.3303 5.08333
-4.31193 0.00395 0.00947 0.0393 0.0125 0.00732 0.00619 0.0261 0.00253 0.0604
2R:19854460-19854610:plus -32.8469 5.34862 3.93578 5.74312 -26.9694 -0.244898 -18.2936 2.48611
-2.17431 0.0243 0.00141 0.00275 0.00302 0.023 0.000496 0.0156 0.00723 0.035
2R:19855280-19855430:minus -32.9286 3.92661 -2.27523 3.30275 -26.6327 0.602041 -15.6881 3.93056
-4.86239 0.025 0.00247 0.0234 0.00789 0.0188 0.000188 0.0102 0.00413 0.0705
2R:19855280-19855430:plus -30.7755 0.376147 12.7064 5.93578 -27.7551 -0.132653 -25.5321 6.01389
-0.651376 0.0102 0.00905 0.0000263 0.00264 0.0385 0.000444 0.0505 0.00165 0.0235
2R:19857680-19857830:minus -32.7347 11.1193 2.99083 5.86239 -16.7143 -18.0102 -19.4862 2.02778
-3.42202 0.0225 0.000076 0.00399 0.00282 0.00319 0.00812 0.0189 0.00853 0.0488
2R:19857680-19857830:plus -22.8878 8.38532 -1.01835 11.2936 -17.7551 -0.0510204 -7.62385 0.958333
-6.05505 0.00441 0.000309 0.0161 0.0000139 0.00658 0.000429 0.00194 0.0123 0.09
2R:19859820-19859970:minus -22.8878 3.7551 -2.93578 6.00917 -17.7143 -18.0816 -20.7431 0.888889
3.36697 0.00441 0.00122 0.0283 0.00255 0.00627 0.00934 0.0235 0.0126 0.00607
2R:19859820-19859970:plus -12.3776 14.4862 0.614679 7.62385 -18.7857 -18.7857 -12.9083 -0.791667

1.48624 0.000403 5.19e-06 0.00943 0.000759 0.0148 0.0146 0.00566 0.0212 0.0118
2R:19864640-19864790:minus -33 14.1009 2.44954 6.41284 0.540816 -8.53061 -14.6881 3.41667 -2.42202 0.0258 7.56e-06 0.00489 0.00189 0.000321 0.00136 0.00842 0.00508 0.0378
2R:19864640-19864790:plus -33.0714 16.3761 1.7156 6.00917 -17.9796 -9.5 -16.789 6.70833 3.6055 0.0267 1.59e-07 0.00642 0.00255 0.00726 0.00294 0.0124 0.00117 0.00532
2R:19865780-19865930:minus -30.7755 -0.66055 2.84404 7.12844 -18.1939 1.09184 -7.6422 4.29167 9.66055 0.0102 0.0129 0.00421 0.00118 0.0086 0.000091 0.00195 0.00356 0.000268
2R:19865780-19865930:plus -32.9694 -2.40367 2.74312 1.41284 -27.2653 -8.79592 -21.9266 9.81944 1.90826 0.0254 0.0218 0.00438 0.0183 0.0302 0.0019 0.0291 0.000186 0.00974
2R:19866240-19866390:minus -21.8571 -0.761468 1.38532 10.8624 -18.4184 -9.57143 -19.5688 -6.48611 9.45872 0.00249 0.0133 0.00721 0.0000687 0.0101 0.00326 0.0191 0.0838 0.000337
2R:19866240-19866390:plus 6.59184 -2.85321 2.55046 4.12844 -25.8571 -17.2755 -10.055 4.69444 -4.81651 2.35e-06 0.0248 0.00471 0.00549 0.0166 0.00544 0.00311 0.003 0.0697
2R:19866980-19867130:minus -30.6939 -3.26606 -1.30275 3.45872 -17.7551 -17.051 -13.1835 -2.79167 2.25688 0.00979 0.0277 0.0175 0.00731 0.00658 0.00479 0.00603 0.0367 0.00853
2R:19866980-19867130:plus -23.0306 3.68367 -0.623853 9.59633 -18.4184 0.0204082 -1.66055 4.125 8.53211 0.00505 0.00144 0.0142 0.000275 0.0101 0.000377 0.000411 0.00382 0.000502
2R:19868460-19868610:minus -31.449 -0.0550459 11.1009 3.19266 -26.6327 -7.5 -13.2385 3.68056 6.73394 0.0123 0.0105 0.000075 0.00829 0.0188 0.000614 0.00611 0.00457 0.00143
2R:19868460-19868610:plus -20.7347 0.0275229 3.53211 2.33945 -27.2347 -19.2755 -5.77064 3.94444 4.83486 0.00176 0.0102 0.00323 0.0122 0.0283 0.0185 0.00123 0.00411 0.00329
2R:19868920-19869070:minus -33.9184 0.614679 -0.174312 3.86239 -18.5612 -36.1837 -9.45872 2.68056 7.42202 0.0302 0.00832 0.0123 0.00622 0.0119 0.0914 0.00278 0.00673 0.00106
2R:19868920-19869070:plus -22.6633 -0.834862 10.2661 6.06422 -18.1224 -16.7857 -9.55963 2.91667 2.36697 0.00384 0.0136 0.000122 0.00239 0.00782 0.00426 0.00284 0.00616 0.00828
2R:19875860-19876010:minus -24.2245 13.5306 1.98165 3.77064 -25.9286 -25.602 -23.2661 7.76389 12.2752 0.00763 0.0000206 0.00582 0.00637 0.0168 0.0207 0.0366 0.000666 0.0000141
2R:19875860-19876010:plus -22.398 13.3878 -0.12844 4.05505 -27.0408 -18.6429 -13.6606 6.75 0.513761 0.00344 0.0000869 0.0121 0.00566 0.0242 0.0139 0.00672 0.00114 0.0162
2R:19882560-19882710:minus -22 5.91743 1.86239 5.3945 -18.7143 -9.09184 -10.4128 0.944444 -0.697248 0.00291 0.0011 0.00608 0.00379 0.0136 0.00219 0.00333 0.0124 0.0239
2R:19882560-19882710:plus -32 2.17431 4.38532 7.17431 -26.5306 -17.8571 -27.5138 7.69444 -2.93578 0.0171 0.00471 0.0023 0.00115 0.0185 0.00719 0.0646 0.000693 0.0439
2R:19909180-19909330:minus -30.8776 8.41284 -2.55963 7.63303 -8.64286 -8.27551 -12.8899 0.458333 -1.11009 0.0103 0.000305 0.0254 0.000745 0.00139 0.000919 0.00564 0.0145 0.0269
2R:19909180-19909330:plus -30.4796 -3.43119 5.90826 7.77982 -17.1531 -27.3061 -13.8991 3.19444 9.56881 0.00941 0.029 0.0012 0.000652 0.00364 0.0336 0.00709 0.00554 0.000303
2R:19909640-19909790:minus -23.5204 13.7798 -0.862385 1.43119 -18.9388 -16.3469 -14.7248 -2.15278 -4.10092 0.00645 0.0000109 0.0153 0.0181 0.0151 0.00416 0.00848 0.0311 0.057
2R:19909640-19909790:plus -21.6327 6.37615 -2.27523 2.05505 10.9898 0.0510204 -13.7064 -1.31944 3.10092 0.00215 0.000885 0.0234 0.0139 3.28e-06 0.000349 0.00679 0.0247 0.00674
2R:19911680-19911830:minus -31.5918 14.6789 2.86239 2.86239 -17.602 -0.173469 -12.5505 4 -3.72477 0.0136 3.91e-06 0.00418 0.00954 0.00547 0.000456 0.00522 0.00402 0.0519
2R:19911680-19911830:plus -31.4796 -0.183486 -1.3578 6.49541 -26.7041 -7.94898 -21.1468 3.83333 2.6422 0.0128 0.011 0.0178 0.00176 0.0193 0.00074 0.0253 0.0043 0.00768
2R:19915980-19916130:minus -23.7041 -0.844037 -0.559633 1.44037 -25.9286 -17.3469 -16.8532 0.458333 0.697248 0.00669 0.0136 0.0139 0.018 0.0168 0.0058 0.0125 0.0145 0.0154
2R:19915980-19916130:plus -31.1429 6.52294 4.65138 6.18349 -18.0816 -9.53061 -6.27523 -0.152778 -1.26606 0.0108 0.000826 0.00206 0.00217 0.00759 0.0031 0.00141 0.0175 0.0281
2R:19916260-19916410:minus -22.7449 2.44954 0.761468 2.29358 -16.9286 -26.6735 -7.45872 5.90278 7.31193 0.00404 0.00425 0.00897 0.0124 0.00327 0.0256 0.00187 0.00174 0.00112
2R:19916260-19916410:plus -41.0306 -2.09174 -2.29358 -3.65138 -27.4898 -17.9796 -24.0917 5.27778 3.66972 0.0464 0.0199 0.0236 0.0783 0.0336 0.00764 0.0416 0.00232 0.00514
2R:19916740-19916890:minus -30.7449 3.88991 2.6055 4.3211 -28.2245 -8.7551 -25.422 7.90278

-6.05505 0.0101 0.00251 0.00462 0.00514 0.049 0.00172 0.0498 0.000616 0.09
2R:19916740-19916890:plus -23.2959 5.65138 0.293578 6.84404 -17.6429 0.0918367 5.0367 1.20833
5.59633 0.00605 0.00124 0.0105 0.0014 0.00562 0.000323 0.0000569 0.0113 0.00234
2R:19921420-19921570:minus -5 10.2569 2.82569 9.33028 -9.27551 -19.2755 0.972477 0.375 7.58716
0.000219 0.000118 0.00424 0.000374 0.00284 0.0185 0.000222 0.0149 0.000954
2R:19921420-19921570:plus -13.4898 4.66055 2.14679 0.706422 -18.5612 -16.7551 -11.3394 -5.47222 2.42202
0.000946 0.00187 0.00547 0.0223 0.0119 0.0042 0.004 0.0682 0.00816
2R:19925260-19925410:minus -31.4694 -2.37615 -0.853211 2.78899 20.1735 -19.6429 -22.9174 2
-2.9633 0.0124 0.0216 0.0152 0.00992 5.68e-07 0.0204 0.0346 0.00862 0.0442
2R:19925260-19925410:plus -32.5102 0.981651 5.95413 1.46789 20.2041 -28.602 -17.5596 6.5 -0.550459
0.0202 0.00729 0.00118 0.0179 3.64e-07 0.0648 0.014 0.0013 0.0225
2R:19933840-19933990:minus -23 1.22018 6.15596 7.88991 -7.85714 -27.6735 -14.1468 2.19444 3.88073
0.00498 0.00668 0.00108 0.00062 0.000594 0.0443 0.00749 0.00804 0.00475
2R:19933840-19933990:plus -24.5612 3.27523 1.48624 -0.146789 -18.7857 -17.1224 -15.8624 3.16667
7.07339 0.00819 0.00314 0.00696 0.028 0.0148 0.00499 0.0106 0.0056 0.0013
2R:19934300-19934450:minus -12.1531 3.64286 0.66055 3.76147 -8.93878 -26.2653 -15.5138 -0.361111
3.66972 0.000352 0.00159 0.00929 0.00645 0.00216 0.0218 0.00992 0.0187 0.00514
2R:19934300-19934450:plus -30.4796 5.11927 -1.9633 3.70642 -16.449 -8.79592 -16.6606 5.26389 -2.75229
0.00941 0.00155 0.0214 0.0066 0.00307 0.0019 0.0121 0.00233 0.0418
2R:19936100-19936250:minus -31.0714 6.97248 1.3578 -0.146789 -19.051 -26.898 -17.9541 2.95833
-4.06422 0.0106 0.000659 0.00728 0.028 0.0162 0.0302 0.0148 0.00606 0.0565
2R:19936100-19936250:plus -41.2449 3.55963 5.66055 4.31193 -17.9796 -9.57143 -13.6881 -1.27778 -5.38532
0.0498 0.00283 0.00134 0.00516 0.00726 0.00326 0.00676 0.0244 0.0788
2R:19945240-19945390:minus -31.6633 0.770642 3.6789 9.92661 -17.7143 -8.45918 -24.055 4.84722
-4.20183 0.0141 0.00786 0.00305 0.000169 0.00627 0.00111 0.0414 0.00281 0.0584
2R:19945240-19945390:plus -4.96939 5.89908 -1.95413 7.62385 -18.449 -16.7551 -10.1284 0.819444 1.22936
0.000218 0.00111 0.0213 0.000759 0.0107 0.0042 0.00315 0.0129 0.0132
2R:19947360-19947510:minus -42.7041 13.5306 1.52294 4.22018 -27.1939 0.826531 -31.2661 -0.375
-4.93578 0.0842 0.0000206 0.00687 0.00531 0.0267 0.000153 0.108 0.0188 0.0718
2R:19947360-19947510:plus -30.9286 13.3878 5.33945 7.44037 -28.2245 -7.79592 -17.8165 -1.27778 3.72477
0.0104 0.0000869 0.00154 0.000912 0.049 0.000723 0.0145 0.0244 0.00497
2R:19947860-19948010:minus -30.5102 4 -1.41284 3.95413 -28.4898 -17.3469 -4.46789 6.29167 5.08257
0.00947 0.0024 0.0181 0.00593 0.0581 0.0058 0.000852 0.00144 0.00303
2R:19947860-19948010:plus -15.0408 13.3878 2.72477 2.58716 -17.8571 -7.42857 -10.9541 -0.652778
-0.724771 0.00162 0.0000869 0.00441 0.011 0.00698 0.000567 0.0037 0.0204 0.0241
2R:19948900-19949050:minus -21.7755 -0.642202 6.29358 9.7156 -27.2653 -10.0204 -12.4862 7.16667
-1.69725 0.00241 0.0128 0.00101 0.000242 0.0302 0.00399 0.00514 0.000922 0.0308
2R:19948900-19949050:plus -32.5102 0.229358 -0.412844 4.91743 -17.4898 -9.30612 -1.3578 2.19444
4.25688 0.0202 0.00953 0.0133 0.00447 0.0053 0.00257 0.000384 0.00804 0.00391
2R:19949880-19950030:minus -30.6633 -4.78899 -1.04587 2.92661 -18.4592 -8.27551 -12.2477 9.22222
1.46789 0.00976 0.0417 0.0162 0.00931 0.0109 0.000919 0.00487 0.000278 0.0119
2R:19949880-19950030:plus -31.4796 -2.63303 2.22936 5.49541 -28.2347 -8.02041 -23.5872 6.16667 -0.522936
0.0128 0.0233 0.00531 0.00355 0.051 0.000761 0.0386 0.00153 0.0222
2R:19950620-19950770:minus -24.2959 14.7523 1.77064 1.78899 -27 -17.5714 -16.055 2.18056 3.93578
0.00781 3.48e-06 0.00629 0.0158 0.0233 0.00616 0.011 0.00808 0.00456
2R:19950620-19950770:plus -32.2245 5.75229 -3.14679 5.69725 -27.2347 -9.53061 -21.1468 4.23611 -6.95413
0.0183 0.00118 0.03 0.00314 0.0283 0.0031 0.0253 0.00364 0.112
2R:19951180-19951330:minus -31.4082 5.49541 0.238532 3.51376 -26.1939 -8.5 -17.4037 3.48611 -0.504587
0.0119 0.00132 0.0107 0.00709 0.0175 0.00126 0.0136 0.00494 0.0221
2R:19951180-19951330:plus -40.2143 4.47706 1.88073 -0.752294 -17.2245 -16.3469 -25.1651 5.5 -0.926606
0.0372 0.002 0.00604 0.034 0.00384 0.00416 0.0482 0.00209 0.0256
2R:19951820-19951970:minus -22.2245 3.97959 1.55963 7.3578 -17.1531 -16.7143 -6.13761 -0.777778
-2.88991 0.00325 0.000669 0.00678 0.000965 0.00364 0.00419 0.00136 0.0211 0.0433
2R:19951820-19951970:plus -23.6633 -1.43119 0.348624 6.12844 -17.1122 -9.5 -9.00917 -2.52778 3.20183

0.00665 0.0164 0.0103 0.00231 0.00341 0.00294 0.00256 0.0343 0.0065
2R:19953000-19953150:minus -23.4898 6.04587 7.77064 3.33945 -17.7143 -8.5 -25.4587 -1.09722 -1.73394
0.00639 0.00104 0.000494 0.00774 0.00627 0.00126 0.05 0.0232 0.0311
2R:19953000-19953150:plus -22.0306 4.70642 1.3945 -1.33028 -26.9694 -18.1224 -2.00917 4.84722 9.45872
0.00293 0.00183 0.00719 0.0409 0.023 0.00988 0.000445 0.00281 0.000337
2R:19955960-19956110:minus -40.2551 -1.9633 -0.220183 6.05505 -27.4592 -8.45918 -29.4771 3.73611
-2.58716 0.0382 0.0192 0.0125 0.0024 0.0329 0.00111 0.0844 0.00447 0.0397
2R:19955960-19956110:plus -22.6939 0.761468 -0.568807 2.78899 -26.9694 -27.898 -7.55963 -0.833333
-3.80734 0.00388 0.00789 0.0139 0.00992 0.023 0.0537 0.00192 0.0215 0.0528
2R:19956500-19956650:minus -22.7041 5.16514 0.844037 4.19266 -8.86735 -19.0102 -14.2936 1.72222
5.75229 0.00395 0.00152 0.00872 0.00536 0.00173 0.0159 0.00774 0.0095 0.00213
2R:19956500-19956650:plus -30.8163 7.10092 2.95413 6.78899 -17.7143 -17.3469 -8.88991 -2.36111 7.15596
0.0103 0.000615 0.00404 0.00146 0.00627 0.0058 0.00251 0.0328 0.00124
2R:19974040-19974190:minus -14.6735 8.2844 0.761468 6.57798 -17.4898 -17.3469 -2.15596 2.47222 -2
0.00152 0.000327 0.00897 0.00167 0.0053 0.0058 0.000461 0.00727 0.0332
2R:19974040-19974190:plus -4.18367 0.0366972 -0.788991 2.21101 -17.3776 0.612245 -6.61468 5.90278
-2.51376 0.000128 0.0102 0.0149 0.0129 0.00419 0.000187 0.00154 0.00174 0.039
2R:19986060-19986210:minus -31.6633 3.0367 6.2844 1.59633 -7.37755 0.0510204 11.0367 6.86111
1.00917 0.0141 0.00343 0.00102 0.017 0.000398 0.000349 8.68e-06 0.00108 0.0143
2R:19986060-19986210:plus -32.4388 4.68807 13.3945 7.29358 10.9184 1.31633 -24.367 11.5278 7.73394
0.0195 0.00184 0.0000143 0.00104 4.19e-06 0.000066 0.0433 0.0000481 0.000784
2R:19986660-19986810:minus -41.102 2.77982 0.66055 -1.90826 -17.4898 -16.5 -16.9817 1.56944
9.97248 0.0472 0.00377 0.00929 0.0497 0.0053 0.00416 0.0128 0.01 0.00016
2R:19986660-19986810:plus -32.7755 2.40367 -0.477064 8.77982 -7.89796 -26.4898 -20.2661 7.31944
-0.550459 0.0234 0.00433 0.0135 0.000533 0.000678 0.0225 0.0216 0.00085 0.0225
2R:20001720-20001870:minus -21.8878 2.99083 7.88991 7.55963 -18.1531 -17.2755 -15.0367 3 3.6789
0.00258 0.00349 0.000464 0.000807 0.00829 0.00544 0.00904 0.00597 0.00506
2R:20001720-20001870:plus -22.7041 4.43119 8.41284 5.58716 10.6531 -18.7857 -1.27523 1.52778 -0.724771
0.00395 0.00204 0.000351 0.00334 0.0000102 0.0146 0.000377 0.0102 0.0241
2R:20018540-20018690:minus -22.1939 3.52294 5.06422 7 -8.90816 -18.0102 -12.7615 3.81944 -0.642202
0.00317 0.00287 0.00173 0.00132 0.00192 0.00812 0.00547 0.00433 0.0234
2R:20018540-20018690:plus -31.9694 -4.25688 3.22936 2.75229 -18.7143 -26.898 -20.0459 4.15278 4.91743
0.0168 0.0362 0.00363 0.0101 0.0136 0.0302 0.0208 0.00377 0.00321
2R:20019040-20019190:minus -12.4082 6.65138 0.908257 6.37615 -18.4898 -27.3367 -5.6422 -1.34722
6.25688 0.000414 0.000775 0.00853 0.00192 0.0116 0.0347 0.00119 0.0249 0.00163
2R:20019040-20019190:plus -11.7755 -2.94495 0.825688 3.18349 -18.7143 -35.449 -14.9908 -0.0833333
4.19266 0.000266 0.0254 0.00878 0.00837 0.0136 0.0789 0.00896 0.0172 0.00401
2R:20020960-20021110:minus -30.7449 0.330275 2.88991 7.61468 -18.4898 -18.051 -23.0734 3.11111
-6.05505 0.0101 0.00919 0.00414 0.000779 0.0116 0.00886 0.0355 0.00572 0.09
2R:20020960-20021110:plus -24.5918 2.0367 0.477064 -1.6422 -17.7551 -28.6735 -2.25688 -1.70833 0.238532
0.00821 0.00495 0.00989 0.0453 0.00658 0.0667 0.000472 0.0275 0.0173
2R:20025580-20025730:minus -20.8469 -0.0550459 2.36697 3.08257 20.2041 -17.5 -21.055 3.77778
-0.449541 0.00178 0.0105 0.00505 0.0087 3.64e-07 0.00599 0.0248 0.0044 0.0216
2R:20025580-20025730:plus -31.0408 -2.70642 -1.06422 7.61468 -8.64286 19.1224 -23.7615 1.66667 3.20183
0.0106 0.0238 0.0163 0.000779 0.00139 9e-07 0.0396 0.00969 0.0065
2R:20046800-20046950:minus -40.8469 -0.724771 14.9633 7.29358 -18.1939 -8.65306 -24.9908 4.48611
-3.43119 0.0437 0.0131 1.77e-06 0.00104 0.0086 0.00156 0.0471 0.00328 0.0489
2R:20046800-20046950:plus -13.2551 11.8624 4.9633 10.7615 -8.67347 -0.469388 -12.5321 0.125
4.09174 0.000751 0.0000498 0.00181 0.0000826 0.00154 0.000533 0.00519 0.0161 0.00416
2R:20061940-20062090:minus -21.8878 13.3878 12.1101 3.74312 -26.9286 -7.94898 -6.49541 -3.80556
-5.25688 0.00258 0.0000869 0.0000401 0.00651 0.0214 0.00074 0.00149 0.047 0.0768
2R:20061940-20062090:plus -33.1531 0.357798 -0.266055 8.80734 -18.9388 -18.1224 -16.6606 3.77778
0.293578 0.0273 0.00911 0.0126 0.000518 0.0151 0.00988 0.0121 0.0044 0.017
2R:20062300-20062450:minus -31.9286 1.34862 1.33945 1.56881 -8.23469 -18.2347 -23.7064 4.65278

-4.92661 0.0166 0.00637 0.00733 0.0172 0.000927 0.0104 0.0393 0.00305 0.0717
2R:20062300-20062450:plus -4.14286 15.1376 -1.81651 2.11009 1.65306 -8.72449 -11.7248 -4.69444 3.40367
0.000127 1.81e-06 0.0205 0.0136 0.0000586 0.00169 0.00434 0.0577 0.00603
2R:20074060-20074210:minus -3.56122 6.37615 5.78899 2.08257 -27.1939 0.0510204 -6.04587 0.375
3.40367 0.0000803 0.000885 0.00127 0.0137 0.0267 0.000349 0.00133 0.0149 0.00603
2R:20074060-20074210:plus -14.0306 1.47706 6.66055 2.54128 -18.6429 -9.53061 -14.156 3.41667 7.63303
0.00115 0.00608 0.000851 0.0112 0.0121 0.0031 0.00751 0.00508 0.000928
2R:20075040-20075190:minus -30.2551 8.07339 5.77064 0.669725 -17.4184 -18.9796 -26.1468 0.972222
6.42202 0.00885 0.000367 0.00128 0.0225 0.00457 0.0155 0.0545 0.0123 0.00156
2R:20075040-20075190:plus -32.7755 -3.36697 4.63303 2.41284 -8.93878 -27.3776 -14.2752 -2.98611 3.6789
0.0234 0.0285 0.00207 0.0118 0.00216 0.0351 0.00771 0.0385 0.00506
2R:20083000-20083150:minus -22.2245 -3.66055 11.3119 7.29358 -17.9286 -8.68367 -14.1376 0.708333
7.94495 0.00325 0.0309 0.0000659 0.00104 0.0071 0.00157 0.00748 0.0134 0.000667
2R:20083000-20083150:plus -21.8878 9.05505 11.2661 7.45872 -8.33673 -8.27551 -12.7798 -2.09722 1.38532
0.00258 0.000217 0.0000678 0.000894 0.00101 0.000919 0.0055 0.0306 0.0123
2R:20084300-20084450:minus -31.6633 -3.43119 -0.431193 3.52294 -18.0816 -17.4184 -15.1927 1.19444
10.1284 0.0141 0.029 0.0133 0.00706 0.00759 0.00585 0.00932 0.0114 0.000129
2R:20084300-20084450:plus -31.4388 -3.78899 3.22936 4.76147 -17.3776 -27.6327 -4.77064 -4.55556 0.394495
0.0122 0.032 0.00363 0.00459 0.00419 0.043 0.00093 0.0559 0.0166
2R:20084740-20084890:minus -33.2245 2.01835 2.80734 8.77982 -9.16327 -8.72449 -30.9541 6.91667
-1.3578 0.0276 0.00499 0.00427 0.000533 0.00249 0.00169 0.104 0.00105 0.0286
2R:20084740-20084890:plus -20.9694 3.07339 -1.88073 1.78899 -27.2755 -0.469388 -24.789 7.13889
5.0367 0.00184 0.00339 0.0209 0.0158 0.0302 0.000533 0.0459 0.000935 0.00306
2R:20085020-20085170:minus -34.102 13.5306 1.15596 3.57798 -17.4082 -17.2755 -0.431193 -0.805556
-3.20183 0.0311 0.0000206 0.00783 0.00696 0.00421 0.00544 0.000313 0.0213 0.0467
2R:20085020-20085170:plus -30.2143 10.3853 11.1468 5.85321 -18.1224 -17.7857 -11.2844 0.944444 7.6422
0.0088 0.00011 0.0000727 0.00287 0.00782 0.0068 0.00396 0.0124 0.000893
2R:20145440-20145590:minus -23.1837 2.6789 9.47706 6.84404 -26.898 -17.3469 -7.13761 2.36111
12.2752 0.00553 0.00391 0.000193 0.0014 0.0203 0.0058 0.00174 0.00757 0.0000141
2R:20145440-20145590:plus -2.15306 -1.94495 3.82569 3.97248 -27.2347 -8.16327 -1.12844 -6.125 4.02752
0.0000259 0.0191 0.00288 0.00587 0.0283 0.000783 0.000365 0.078 0.00428
2R:20148280-20148430:minus -32.0816 0.550459 4.74312 7.27523 -18.5306 -8.57143 -21.1651 3.09722
1.53211 0.0176 0.00851 0.00198 0.00109 0.0118 0.00149 0.0253 0.00575 0.0116
2R:20148280-20148430:plus -23.9286 0.229358 0.715596 6.2844 -16.7551 -28.6327 -9.13761 5.77778 2.78899
0.00701 0.00953 0.00912 0.00205 0.00322 0.066 0.00262 0.00184 0.00745
2R:20162820-20162970:minus -20.8878 -2.34862 3.31193 7 -8.82653 -8.72449 -22.8532 2.69444 1.52294
0.0018 0.0215 0.00352 0.00132 0.00163 0.00169 0.0342 0.00669 0.0117
2R:20162820-20162970:plus -3.81633 0.119266 8.56881 6.72477 -8.57143 -18.7857 -15.2018 -6.16667 3.01835
0.000106 0.0099 0.00032 0.00154 0.00113 0.0146 0.00934 0.0787 0.00697
2R:20163600-20163750:minus -22.9286 3.99083 2.41284 -0.311927 -18.3469 -0.244898 -18.4128
-5.125 -1.11009 0.0046 0.00241 0.00496 0.0294 0.00894 0.000496 0.0159 0.0634 0.0269
2R:20163600-20163750:plus -11.551 -0.0183486 1.84404 3.78899 -18.449 -27.602 -12.5688 4.93056
-0.46789 0.000255 0.0104 0.00612 0.00632 0.0107 0.0416 0.00524 0.00271 0.0218
2R:20181460-20181610:minus -31.1429 -1.33945 8.00917 2.3945 -18.1939 -17.7143 -17.6789 4.97222
11.8073 0.0108 0.0159 0.000435 0.0119 0.0086 0.00634 0.0142 0.00266 0.0000538
2R:20181460-20181610:plus -13.2551 -4.48624 7.53211 11.0275 -26.7041 -18.051 -8.82569 14.6389 1.26606
0.000751 0.0385 0.000558 0.0000401 0.0193 0.00886 0.00248 9.23e-07 0.013
2R:20193280-20193430:minus -33.2959 -1.6789 11.6055 7.45872 -9.23469 0.0204082 -16.5229 2.33333
-2.63303 0.0279 0.0177 0.0000557 0.000894 0.00277 0.000377 0.0119 0.00765 0.0404
2R:20193280-20193430:plus -23.0816 -0.40367 9.50459 10.5963 -18.4592 -18.2755 -17.9725 -5.65278 -3.31193
0.00522 0.0118 0.000189 0.0001 0.0109 0.0112 0.0149 0.0709 0.0477
2R:20194860-20195010:minus -23 3.90816 -2.29358 3.88991 -18.4184 -18.9796 -4.80734 0.763889 7.53211
0.00498 0.00102 0.0236 0.00608 0.0101 0.0155 0.00094 0.0131 0.000968
2R:20194860-20195010:plus -31.2551 -0.761468 -1.86239 0.201835 -28.2755 -8.34694 -15.5872 3.19444

3.17431 0.0113 0.0133 0.0208 0.0255 0.0539 0.000993 0.0101 0.00554 0.00653
2R:2019560-2019710:minus -21.9694 3.02752 5.29358 3.94495 -17.1122 18.8571 -21.5229 -0.902778
-6.10092 0.00282 0.00344 0.00157 0.00595 0.00341 1.47e-06 0.027 0.0219 0.091
2R:2019560-2019710:plus -20.7347 -0.46789 2.44037 5.21101 -26.8571 -25.602 -10.8532 2.47222 1.88073
0.00176 0.0121 0.00491 0.0042 0.0198 0.0207 0.00362 0.00727 0.00987
2R:2021940-2022090:minus -21.9694 -0.165138 3.2844 6.6422 -18.3776 -17.7857 -7.51376 10.3194
7.24771 0.00282 0.0109 0.00356 0.00161 0.00926 0.0068 0.0019 0.000129 0.00116
2R:2021940-2022090:plus -24.1939 -2.11009 1.00917 2.31193 -27.1633 0.0510204 -20.7064 -4.875
-3.29358 0.00753 0.0201 0.00824 0.0123 0.025 0.000349 0.0233 0.06 0.0474
2R:2025520-2025670:minus -31.9694 4.87156 6.98165 5.43119 -8.93878 -17.5714 -11.9908 -5.19444 2.57798
0.0168 0.00172 0.000733 0.00371 0.00216 0.00616 0.0046 0.0643 0.0078
2R:2025520-2025670:plus -24.2857 2.06422 9.04587 6.17431 -17.3367 -27.6327 -9.53211 -1.69444 5.01835
0.00773 0.0049 0.000248 0.0022 0.00391 0.043 0.00282 0.0274 0.00309
2R:20257300-20257450:minus -22.9286 -2.6422 6.40367 10.0275 -18.6429 -18.0102 -4.37615 -4.625
3.61468 0.0046 0.0233 0.000962 0.000156 0.0121 0.00812 0.00083 0.0568 0.00526
2R:20257300-20257450:plus -31 -0.284404 6.21101 6.00917 -8.37755 -25.898 -2.24771 -0.111111
3.49541 0.0105 0.0114 0.00105 0.00255 0.00106 0.0214 0.000471 0.0173 0.0057
2R:2026300-2026450:minus -21.7449 3.90816 -0.954128 1.98165 -27.2347 -9.86735 -18.1009 3.68056
2.73394 0.00236 0.00102 0.0157 0.0145 0.0283 0.00391 0.0152 0.00457 0.00756
2R:2026300-2026450:plus -14.2653 5.88073 7.73394 10.5963 -18.1531 -9.5 -22.422 5.02778 2.53211
0.00128 0.00112 0.000504 0.0001 0.00829 0.00294 0.0318 0.00259 0.00791
2R:20279940-20280090:minus -31.8571 -0.53211 3.42202 3.74312 -27.9286 -18.8265 -19.4771 -0.0694444
1.79817 0.0163 0.0123 0.00337 0.00651 0.0412 0.0148 0.0189 0.0171 0.0102
2R:20279940-20280090:plus -23.3776 9.88073 -2.74312 1.78899 -19.051 -9.5 -22.4954 2.15278 -0.229358
0.00619 0.000142 0.0268 0.0158 0.0162 0.00294 0.0322 0.00816 0.0202
2R:2028080-2028230:minus -41.7041 -1.00917 -1.69725 2.0367 -18.4592 -9.02041 -19.1927 1.94444 -0.559633
0.0641 0.0144 0.0197 0.0141 0.0109 0.00213 0.018 0.00879 0.0226
2R:2028080-2028230:plus -32.3367 -1.81651 4.17431 6.00917 -8.67347 -8.34694 -14.211 1.25 2.93578
0.0187 0.0184 0.0025 0.00255 0.00154 0.000993 0.0076 0.0112 0.00717
2R:20287240-20287390:minus -23.0306 3.57143 2.78899 2.66972 -8.56122 -17.8265 -10.5046 0.986111
3.85321 0.00505 0.00175 0.0043 0.0106 0.00112 0.00699 0.00339 0.0122 0.00479
2R:20287240-20287390:plus -12.8878 0.669725 -3.6789 3.44954 -27.2347 -17.9796 -9.47706 5.51389 1.38532
0.000523 0.00815 0.0348 0.00738 0.0283 0.00764 0.00279 0.00208 0.0123
2R:2028900-2029050:minus -32.398 9.97248 -2.48624 1.95413 -18.1531 -27.5612 -13.8257 3.875 3.88991
0.019 0.000135 0.0249 0.0147 0.00829 0.0393 0.00698 0.00423 0.00467
2R:2028900-2029050:plus -31.3571 -4.94495 -1.57798 7.02752 -17.5612 -26.6327 -17.1651 9.26389 -3.40367
0.0114 0.0435 0.019 0.00122 0.00544 0.0251 0.0132 0.00027 0.0486
2R:20289360-20289510:minus -31.4388 -0.46789 1.34862 -1.46789 -18.2245 -27.1939 -9.22936 5.38889
1.3211 0.0122 0.0121 0.00731 0.0428 0.00877 0.0327 0.00267 0.0022 0.0127
2R:20289360-20289510:plus -22.7755 -2.05505 6.63303 6.31193 -17.3776 -27.3367 -14.633 -2.01389 1.63303
0.00412 0.0197 0.000861 0.00199 0.00419 0.0347 0.00832 0.0299 0.0112
2R:20289860-20290010:minus -14.2245 -0.605505 0.146789 3.98165 -9.0102 -26.8265 -12.422 0.444444
8.10092 0.00126 0.0126 0.011 0.00582 0.00236 0.0285 0.00507 0.0145 0.000623
2R:20289860-20290010:plus -23.8469 -1.52294 1.11009 0.779817 -26.2245 -27.602 -15.0275 -1.66667 10.3486
0.00685 0.0169 0.00796 0.0219 0.0175 0.0416 0.00902 0.0272 0.0000988
2R:2029760-2029910:minus -41.5204 4.65138 4.3211 6.85321 -17.7143 -17.5714 -25.0275 1.94444 -1.63303
0.0592 0.00187 0.00235 0.00137 0.00627 0.00616 0.0473 0.00879 0.0304
2R:2029760-2029910:plus -20.6633 1.69725 5.26606 7.20183 -18.4082 -27.602 -4.50459 -0.305556
3.94495 0.00173 0.00561 0.00159 0.00112 0.00937 0.0416 0.000862 0.0184 0.00452
2R:20299300-20299450:minus -24.5204 -1.99083 -1.11927 5.69725 -17.449 -9.57143 -9.73394 6.97222
3.16514 0.00813 0.0194 0.0166 0.00314 0.00484 0.00326 0.00293 0.00102 0.00656
2R:20299300-20299450:plus -22.8571 -1.85321 3.16514 3.13761 1.87755 -8.72449 -13.9725 4.91667 -4.0367
0.00427 0.0186 0.00373 0.00854 0.0000492 0.00169 0.00721 0.00272 0.0562
2R:2031500-2031650:minus -13.5918 1.46789 3 2.6422 -27.1531 -0.102041 -23.4679 5.69444 0.669725

0.00099	0.0061	0.00397	0.0107	0.0249	0.000443	0.0378	0.00191	0.0155
2R:2031500-2031650:plus	-23.4898	-1.11927	7.89908	6.02752	-18.7143	-8.65306	-6.51376	11.0417	-4.93578
0.00639	0.0149	0.000462	0.00246	0.0136	0.00156	0.0015	0.0000736	0.0718
2R:2032260-2032410:minus	-32.7449	-0.522936	-1.45872	5.07339	1.10204	-9.5	-22.2936	-0.680556
1.53211	0.0228	0.0123	0.0184	0.0043	0.000124	0.00294	0.0311	0.0206	0.0116
2R:2032260-2032410:plus	-30.9898	0.165138	5.84404	2.74312	-26.9286	-28.1939	-1.3578	2.95833	9.04587
0.0105	0.00974	0.00124	0.0101	0.0214	0.0591	0.000384	0.00606	0.000423
2R:2032960-2033110:minus	-14.4184	-1.11009	-3.33945	3.05505	-9.54082	-8.45918	-21.5872	3.30556	2.53211
0.00137	0.0148	0.0317	0.00878	0.00294	0.00111	0.0274	0.0053	0.00791
2R:2032960-2033110:plus	-13.1122	-0.788991	7.0367	1.98165	-17.0408	0.204082	-13.1468	1.44444
-2.00917	0.000632	0.0134	0.000715	0.0145	0.00329	0.000296	0.00598	0.0105	0.0333
2R:2034520-2034670:minus	-23.2959	-0.522936	-4.80734	3.74312	-18.4592	-9.16327	-8.83486	-1.20833
2.36697	0.00605	0.0123	0.047	0.00651	0.0109	0.00223	0.00248	0.0239	0.00828
2R:2034520-2034670:plus	-12.551	6.23853	-5.89908	1.6789	-8.33673	-27.051	-11.0734	5.72222	3.41284
0.000444	0.000946	0.0618	0.0165	0.00101	0.0315	0.00379	0.00189	0.006
2R:2035500-2035650:minus	-13.2959	-1.57798	2.81651	3.10092	-18.4184	-17.051	-12.8165	-1.93056	7.11009
0.000775	0.0171	0.00426	0.00865	0.0101	0.00479	0.00554	0.0293	0.00128
2R:2035500-2035650:plus	-21.7347	-3.97248	2.38532	7.63303	0.581633	-16.9796	-13.4128	-1.81944	1.78899
0.00235	0.0336	0.00501	0.000745	0.000292	0.0044	0.00635	0.0284	0.0103
2R:20402540-20402810:minus	-31.0714	3.64286	3.11009	3.26606	1.5102	-9.0102	-21.7615	-0.736111
0.238532	0.0106	0.00159	0.00381	0.00802	0.0000722	0.00208	0.0282	0.0209	0.0173
2R:20402540-20402810:plus	-30.8469	-3.3945	1.57798	1.58716	-27.1531	-8.16327	-13.9817	1.63889	7.68807
0.0103	0.0287	0.00674	0.017	0.0249	0.000783	0.00722	0.00978	0.000841
2R:20413580-20413730:minus	-22.2653	12.0275	4.47706	3.0367	-25.7041	-8.38776	-17.2018	5.43056
-1.52294	0.00331	0.0000451	0.00221	0.00886	0.0165	0.00101	0.0132	0.00216	0.0295
2R:20413580-20413730:plus	-22.0714	1.6055	-0.522936	1.05505	-18.7143	-26.4898	-8.58716	0.736111
-2.69725	0.00299	0.0058	0.0137	0.0204	0.0136	0.0225	0.00236	0.0132	0.0411
2R:20419160-20419310:minus	-31.5204	6.34862	3.40367	0.577982	-18.1224	-17.898	-12.2752	7.27778
6.02752	0.0131	0.000897	0.0034	0.0231	0.00782	0.00726	0.0049	0.000869	0.00183
2R:20419160-20419310:plus	-33.3061	-1.86239	4.30275	7	-18.6837	-18.3061	-23.3578	2.22222	-0.761468
0.0279	0.0187	0.00237	0.00132	0.0126	0.0114	0.0372	0.00796	0.0244
2R:20419500-20419650:minus	-32.8061	-0.46789	9.0367	6.72477	-17.4082	-28.602	-18.1651	-2.15278	6
0.0237	0.0121	0.000249	0.00154	0.00421	0.0648	0.0153	0.0311	0.00184
2R:20419500-20419650:plus	-30.6327	5.61468	2.57798	9.61468	-17.6429	-8.16327	-17.1468	9.38889	-0.174312
0.00968	0.00126	0.00466	0.000255	0.00562	0.000783	0.0131	0.000249	0.0196
2R:20422880-20423030:minus	-21.8571	6.53211	5.09174	7.00917	-18.4184	-8.53061	-4.88073	0.694444
6.76147	0.00249	0.000822	0.00171	0.00126	0.0101	0.00136	0.00096	0.0134	0.00141
2R:20422880-20423030:plus	-22.1837	13.5306	-0.93578	4.3211	-36.4082	-17.3469	-10.8991	6.84722	-0.559633
0.00315	0.0000206	0.0156	0.00514	0.0765	0.0058	0.00366	0.00109	0.0226
2R:20426940-20427090:minus	-13.449	0.458716	-4.22018	1.53211	-8.57143	-17.7143	-9.50459	0.791667
3.87156	0.000914	0.00879	0.0403	0.0174	0.00113	0.00634	0.00281	0.013	0.00477
2R:20426940-20427090:plus	-31.2857	-0.972477	-4.08257	2.12844	-8.60204	-26.3776	-6.25688	-1.30556
5.66055	0.0113	0.0142	0.0388	0.0134	0.00125	0.0222	0.0014	0.0246	0.00227
2R:20427400-20427550:minus	-32.0408	0.495413	3.57798	0.954128	-18.1837	-8.5	-10.844	2.34722	7.22018
0.0174	0.00868	0.00317	0.0208	0.00835	0.00126	0.00362	0.00761	0.00119
2R:20427400-20427550:plus	-21.551	2.22018	2.18349	5.69725	-18.4184	-8.16327	-12.9358	1.93056	3.09174
0.00205	0.00463	0.0054	0.00314	0.0101	0.000783	0.0057	0.00883	0.00678
2R:20428100-20428250:minus	-31.8061	5.04587	1.41284	1.62385	-18.7959	-9.45918	-12.6972	1.125
7.22018	0.0158	0.0016	0.00714	0.0168	0.0148	0.00279	0.00539	0.0117	0.00119
2R:20428100-20428250:plus	-24.1122	8	2.3945	3.10092	-17.4898	-17.7857	-0.577982	4.72222	7.73394
0.00738	0.000381	0.00499	0.00865	0.0053	0.0068	0.000323	0.00296	0.000784
2R:20430900-20431050:minus	-40.5918	-1.68807	-2.3945	1.41284	-19.2755	-17.3469	-26.0459	6.66667
5.9633	0.0422	0.0177	0.0242	0.0183	0.0164	0.0058	0.0538	0.00119	0.00188
2R:20430900-20431050:plus	-31.8469	0.256881	-2.56881	2.27523	-27.898	-18.5714	-7.63303	1.76389	1.00917

0.0162 0.00944 0.0255 0.0125 0.04 0.0136 0.00195 0.00937 0.0143
2R:2044100-2044250:minus -31.7347 3.44037 4.44037 5.49541 -17.6735 -9.68367 -11.6697 4.01389 3.42202
0.0149 0.00296 0.00224 0.00355 0.00572 0.00342 0.00429 0.004 0.00597
2R:2044100-2044250:plus -32.4388 -1.94495 4.47706 5.75229 -17.7143 -7.79592 -26.0917 -1.81944 0.12844
0.0195 0.0191 0.00221 0.00296 0.00627 0.000723 0.0541 0.0284 0.0179
2R:2044640-2044790:minus 6.47959 -0.623853 7.45872 9.59633 -18.2245 -9.45918 -2.83486 -1.38889
7.42202 3e-06 0.0127 0.000579 0.000275 0.00877 0.00279 0.000544 0.0252 0.00106
2R:2044640-2044790:plus -39.2245 -2.23853 2.3211 2.80734 -17.3776 -8.38776 -2.00917 -1.83333 -0.862385
0.0329 0.0208 0.00513 0.00985 0.00419 0.00101 0.000445 0.0285 0.0251
2R:20448540-20448690:minus 6.55102 -1.63303 0.440367 5.57798 -26.898 -17.7551 -19.2752 -2.29167
-0.220183 2.47e-06 0.0174 0.01 0.0034 0.0203 0.00643 0.0182 0.0322 0.0201
2R:20448540-20448690:plus -23.1531 13.4592 0.284404 2.83486 -17.7551 -18.1531 -14.4587 -0.472222
3.55963 0.00542 0.000064 0.0105 0.0097 0.00658 0.00997 0.00802 0.0193 0.00545
2R:20448980-20449130:minus -21.9592 -1.6055 3.12844 6.89908 -26.1939 -8.27551 -5.75229 -0.0833333
-5.33945 0.00279 0.0173 0.00378 0.00134 0.0175 0.000919 0.00123 0.0172 0.0781
2R:20448980-20449130:plus -22.9592 11.7248 -1.20183 4.11927 -8.93878 -17.2755 -19.7615 -1.30556 -0.495413
0.00475 0.0000542 0.017 0.00551 0.00216 0.00544 0.0198 0.0246 0.022
2R:20452740-20452890:minus -33.4694 2.12844 2.16514 2.66972 -28.2959 -26.7857 -13.3394 1.79167
-4.20183 0.0285 0.00479 0.00544 0.0106 0.0541 0.0266 0.00625 0.00928 0.0584
2R:20452740-20452890:plus -30.2959 0.981651 -0.431193 0.330275 20.1735 -8.94898 -15.9358 1.25 7.72477
0.00887 0.00729 0.0133 0.0246 5.68e-07 0.00203 0.0107 0.0112 0.000798
2R:20459060-20459210:minus -30.7449 -4.97248 5.61468 5.43119 -9.19388 -19.4184 -12.2018 2.38889
7.42202 0.0101 0.0438 0.00137 0.00371 0.00253 0.0198 0.00482 0.00749 0.00106
2R:20459060-20459210:plus -24.2245 2.38532 12.4862 11.2936 -17.7857 -8.72449 -24.7431 1.68056 -3.33028
0.00763 0.00436 0.0000304 0.0000139 0.00676 0.00169 0.0456 0.00964 0.0478
2R:20462020-20462170:minus -31.6633 6.93578 -1.58716 5.3945 -18.0816 -17.0816 -23.4312 3.09722
-1.68807 0.0141 0.000671 0.0191 0.00379 0.00759 0.00487 0.0376 0.00575 0.0308
2R:20462020-20462170:plus -23.0714 8.16514 14.9266 6.25688 0.846939 1.38776 -11.8532 -2.41667 -2.95413
0.0052 0.000349 1.9e-06 0.00208 0.000197 0.0000559 0.00447 0.0333 0.0441
2R:20487320-20487470:minus -33 5.80734 2.6055 7.33945 10.3878 -17.8571 -12.6055 2.98611 0.174312
0.0258 0.00116 0.00462 0.00102 0.0000174 0.00719 0.00528 0.006 0.0177
2R:20487320-20487470:plus -24.2551 10.0642 -3.56881 3.75229 -19.0102 -8.79592 -17.2844 4.84722 1.21101
0.00768 0.00013 0.0338 0.00649 0.0157 0.0019 0.0134 0.00281 0.0134
2R:20494600-20494750:minus -13.1429 1.34862 5.56881 2.98165 -18.4184 -27.898 -20.2936 -1.59722
-2.98165 0.00065 0.00637 0.0014 0.00905 0.0101 0.0537 0.0217 0.0267 0.0445
2R:20494600-20494750:plus -31.4796 4.20183 15.0367 7.29358 -26.9694 -18.8571 -8.59633 -0.722222
7.6789 0.0128 0.00223 1.36e-06 0.00104 0.023 0.0151 0.00237 0.0208 0.000866
2R:20499040-20499190:minus -31.6735 -5.44037 2.58716 4.04587 -18.449 -17.9796 -10.4954 2.75 4.23853
0.0141 0.0495 0.00465 0.00572 0.0107 0.00764 0.00338 0.00656 0.00394
2R:20499040-20499190:plus -32.4286 2.14679 15.6239 5.74312 -18.7143 -9.5 -11.5505 1.54167 5.13761
0.0191 0.00476 5.44e-07 0.00302 0.0136 0.00294 0.00418 0.0101 0.00298
2R:20515660-20515810:minus -32.0714 0.357798 -3.57798 9.59633 -26.9694 -8.79592 -9.10092 5.80556
-0.642202 0.0176 0.00911 0.0339 0.000275 0.023 0.0019 0.00261 0.00182 0.0234
2R:20515660-20515810:plus -21.9286 6.41284 -2.77982 3.33945 -17.4898 -8.5 -23.4771 3.59722 -3.89908
0.0027 0.00087 0.0271 0.00774 0.0053 0.00126 0.0379 0.00473 0.0541
2R:20518460-20518610:minus -14.5204 1.9633 -3.22018 3.95413 -27.0408 -17.051 -10.4862 4.63889
5.65138 0.00145 0.00509 0.0307 0.00593 0.0242 0.00479 0.00337 0.00307 0.00228
2R:20518460-20518610:plus -30.4082 -1.19266 4.44954 4.08257 -17.4898 -7.45918 -10.0092 7.77778 9.45872
0.00908 0.0152 0.00224 0.00558 0.0053 0.000582 0.00308 0.000661 0.000337
2R:20548900-20549050:minus -33.2245 -1.33028 -1.78899 5.06422 -8.93878 -9.7551 -16.5413 0.847222
0.963303 0.0276 0.0159 0.0203 0.00433 0.00216 0.00358 0.0119 0.0128 0.0145
2R:20548900-20549050:plus -22.2143 0.238532 14.1284 6.21101 -18.3469 0.0204082 -25.2936 2.08333
-1.58716 0.00317 0.0095 6.14e-06 0.00213 0.00894 0.000377 0.049 0.00837 0.03
2R:20551820-20551970:minus -23.5918 12.5688 5.88073 1.86239 -16.7143 -7.45918 -9.86239 2.47222

-2.11927 0.00656 0.000031 0.00122 0.0153 0.00319 0.000582 0.003 0.00727 0.0344
2R:20551820-20551970:plus -23.2959 14.1468 14.3486 7.29358 -27.2653 -18.0816 -11.5229 4.11111 -4.04587
0.00605 7.12e-06 4.67e-06 0.00104 0.0302 0.00934 0.00416 0.00384 0.0563
2R:20555820-20555970:minus -31.7041 -0.972477 14.9633 7.29358 -27.2653 1.05102 -10.7431 1.72222
-2.42202 0.0146 0.0142 1.77e-06 0.00104 0.0302 0.000107 0.00355 0.0095 0.0378
2R:20555820-20555970:plus -3.33673 3.85321 10.3486 2.66972 -17.6837 -10.0204 -17.055 2.27778 -2.10092
0.0000599 0.00254 0.000115 0.0106 0.0058 0.00399 0.0129 0.0078 0.0342
2R:20556800-20556950:minus -22.7755 -0.59633 13.3394 1.29358 -8.86735 -17.7857 -12.5321 2.38889
3.36697 0.00412 0.0126 0.0000148 0.019 0.00173 0.0068 0.00519 0.00749 0.00607
2R:20556800-20556950:plus -21.7347 -1.66972 13.055 7.29358 -16.7143 -19.3469 -14.3945 -0.5 -2.61468
0.00235 0.0176 0.0000195 0.00104 0.00319 0.0194 0.00791 0.0195 0.0401
2R:20572760-20572910:minus -24 0.137615 6.51376 10.5963 -27.1531 -18.0816 -9.38532 -0.75 3.88073
0.00719 0.00984 0.000912 0.0001 0.0249 0.00934 0.00275 0.021 0.00475
2R:20572760-20572910:plus -31.8061 0.155963 4.09174 2.83486 -17.449 -36.1531 -7 -0.263889 1.44037
0.0158 0.00977 0.00258 0.0097 0.00484 0.0908 0.00169 0.0181 0.012
2R:20574820-20574970:minus -4.52041 5.97248 4.6422 7.78899 -17.0816 -17.7857 -10.1651 1.13889
6.37615 0.000158 0.00107 0.00206 0.000642 0.00334 0.0068 0.00317 0.0116 0.00159
2R:20574820-20574970:plus -21.8878 -0.706422 1.87156 9.7156 -26.9286 -9.42857 -19.4037 -3.13889
-6.3578 0.00258 0.0131 0.00606 0.000242 0.0214 0.0027 0.0186 0.04 0.097
2R:20575840-20575990:minus -4.66327 0.266055 -1.3945 9.92661 -18.4082 -27.6327 4.66055 7.90278
3.08257 0.000177 0.0094 0.018 0.000169 0.00937 0.043 0.0000649 0.000616 0.00681
2R:20575840-20575990:plus -24.0408 3.22018 -2.59633 3.27523 -18.7143 0.755102 -22.2752 2.25 -4.33945
0.00726 0.00321 0.0257 0.00796 0.0136 0.000175 0.031 0.00788 0.0609
2R:20577160-20577310:minus -21.9286 -1.31193 2.86239 6.84404 -26.9388 -8.23469 -24 3.51389 -4.42202
0.0027 0.0158 0.00418 0.0014 0.0215 0.000875 0.041 0.00489 0.0626
2R:20577160-20577310:plus -29.8776 0.0825688 10.367 5.57798 -17.6429 -18.0102 -17.4404 -4.19444
5.29358 0.00852 0.01 0.000114 0.0034 0.00562 0.00812 0.0137 0.0515 0.00278
2R:20631020-20631170:minus -14.8571 14.4862 -4.73394 5.42202 -18.4898 -17.1224 -10.844 7.61111
1.01835 0.0016 5.19e-06 0.0461 0.00374 0.0116 0.00499 0.00362 0.000726 0.0142
2R:20631020-20631170:plus -40.6224 5.42202 0.678899 1.3211 1.91837 1.27551 -15.6697 0.902778
-0.0642202 0.0424 0.00137 0.00923 0.0189 0.0000382 0.0000717 0.0102 0.0125 0.0188
2R:20631760-20631910:minus -22.4388 0.174312 2.75229 6.27523 0.846939 -18.051 -10.3945 6.38889
0.293578 0.00348 0.00971 0.00436 0.00206 0.000197 0.00886 0.00332 0.00137 0.017
2R:20631760-20631910:plus -13.3265 4.48624 0.816514 3.07339 -27 -36.8878 -12.7798 3.94444 7.15596
0.000779 0.00199 0.0088 0.00873 0.0233 0.123 0.0055 0.00411 0.00124
2R:20632860-20633010:minus -33.3367 1.44954 1.18349 6.84404 -18.449 -8.72449 -13.1284 4.02778
-3.41284 0.0281 0.00615 0.00775 0.0014 0.0107 0.00169 0.00595 0.00397 0.0487
2R:20632860-20633010:plus -31.4796 2.06422 5.2844 4.12844 -8.53061 -17.9796 -4.85321 7.52778 -0.559633
0.0128 0.0049 0.00158 0.00549 0.0011 0.00764 0.000952 0.00076 0.0226
2R:20650580-20650730:minus -31.7347 -1.11009 13.4495 10.8624 -27.2347 -17.898 -15.6697 -3.81944
1.48624 0.0149 0.0148 0.0000136 0.0000687 0.0283 0.00726 0.0102 0.0472 0.0118
2R:20650580-20650730:plus -30.7755 1.30275 0.293578 3.45872 11.6939 -18.051 -17.5688 1.88889
-3.81651 0.0102 0.00649 0.0105 0.00731 1.17e-06 0.00886 0.014 0.00896 0.0529
2R:20655640-20655790:minus -22.8061 0.0366972 -1.12844 6.05505 -17.3776 -18.7857
-18.7156 1.86111 2.47706 0.00414 0.0102 0.0166 0.0024 0.00419 0.0146 0.0167 0.00905 0.00801
2R:20655640-20655790:plus -23.4898 0.522936 -3.47706 0.963303 -18.7551 -0.0204082
-20.7064 1.88889 -1.68807 0.00639 0.00859 0.0329 0.0208 0.0144 0.000411 0.0233 0.00896 0.0308
2R:20667340-20667490:minus -13.7449 7.88991 -3.09174 -0.302752 -18.3776 -18.3469
-13.6422 2.48611 5.59633 0.00107 0.000405 0.0296 0.0293 0.00926 0.0124 0.00669 0.00723 0.00234
2R:20667340-20667490:plus -14.7041 3.64286 0.385321 6.78899 -9.19388 -9.94898 -11.3028
1.13889 4.13761 0.00154 0.00159 0.0102 0.00146 0.00253 0.00393 0.00397 0.0116 0.00408
2R:20668340-20668490:minus -22.2653 -0.486239 4.66972 5.59633 -17.7143 -28.898 -10.7615 -3.625
4.77982 0.00331 0.0121 0.00204 0.00331 0.00627 0.0713 0.00356 0.045 0.00339
2R:20668340-20668490:plus -23.2245 4.72477 -1.58716 3.05505 -18.4184 -18.7857 -24.211 1.33333 1.02752

0.00576 0.00182 0.0191 0.00878 0.0101 0.0146 0.0423 0.0109 0.0141
2R:20670980-20671130:minus -11.5816 -4.84404 14.3486 9.88073 -8.56122 -19.3469 -5.95413 2.02778
-4.52294 0.000256 0.0423 4.67e-06 0.000183 0.00112 0.0194 0.00129 0.00853 0.0642
2R:20670980-20671130:plus -23.5204 1.45872 14.6881 6.11009 -18.5306 -35.3776 3.86239 -0.638889
0.165138 0.00645 0.00613 3.06e-06 0.00233 0.0118 0.0783 0.0000868 0.0203 0.0177
2R:20674720-20674870:minus -31.3265 -0.504587 4.01835 3.61468 -7.89796 -18.8571 -23.2752 8.33333
9.55046 0.0114 0.0122 0.00266 0.0068 0.000678 0.0151 0.0367 0.000481 0.000321
2R:20674720-20674870:plus -22.6633 2.33028 -2.88073 4.86239 -7.93878 -18.1939 -20.3486 3.77778 -2.84404
0.00384 0.00445 0.0279 0.00449 0.000767 0.0101 0.0219 0.0044 0.0428
2R:20680820-20680970:minus -22.9286 2.93578 2.54128 1.77982 -18.3367 -18.9388 -17.633 -5 0.238532
0.0046 0.00356 0.00473 0.0158 0.00885 0.0154 0.0141 0.0617 0.0173
2R:20680820-20680970:plus -21.7347 3.44954 3.80734 5.6422 -18.4184 -18.051 17.8165 -2.66667 9.77064
0.00235 0.00295 0.0029 0.00325 0.0101 0.00886 5.17e-07 0.0355 0.000228
2R:20681260-20681410:minus -22.4388 4.02752 0.330275 2.26606 -18.449 -7.38776 -19.2569 4.94444
-2.06422 0.00348 0.00238 0.0104 0.0126 0.0107 0.000563 0.0182 0.00269 0.0339
2R:20681260-20681410:plus -13.0714 -2.87156 3.3578 4.66972 -18.1531 -27.1224 -12.5413 -1.54167 -0.550459
0.000594 0.0249 0.00346 0.00466 0.00829 0.0325 0.00521 0.0263 0.0225
2R:20684940-20685090:minus -23.2143 -1.18349 -0.788991 1.20183 -27.2347 -9.42857 -13.2018 -0.305556
-0.743119 0.00559 0.0152 0.0149 0.0195 0.0283 0.0027 0.00605 0.0184 0.0243
2R:20684940-20685090:plus -33.5204 5.3578 0.963303 5.73394 -17.4592 0.27551 -26 10.0139 -3.41284
0.0287 0.0014 0.00837 0.00307 0.00491 0.000257 0.0535 0.000162 0.0487
2R:20702180-20702330:minus -22.8878 0.715596 6.29358 3.21101 -18.0816 -28.898 5.73394 -7.81944
9.3945 0.00441 0.00802 0.00101 0.00822 0.00759 0.0713 0.0000451 0.108 0.000358
2R:20702180-20702330:plus -22.551 -2.18349 5.33028 7.90826 -18.0816 -28.2653 -6.57798 -7.375 3.87156
0.00359 0.0205 0.00155 0.000611 0.00759 0.0595 0.00152 0.0993 0.00477
2R:20757380-20757530:minus -13 13.4592 2.66055 6.00917 -18.4898 -25.602 -8.6055 6.29167 4.10092
0.000563 0.000064 0.00452 0.00255 0.0116 0.0207 0.00237 0.00144 0.00413
2R:20757380-20757530:plus -22.2245 11.2202 2.06422 3.51376 -17.7143 -8.20408 -18.5046 -1.77778 -1.70642
0.00325 0.0000727 0.00564 0.00709 0.00627 0.000796 0.0161 0.0281 0.0309
2R:20762440-20762590:minus -30.9286 13.4592 2.08257 9.49541 -26.1939 -18.0102 -21.422 -1.04167
6.69725 0.0104 0.000064 0.0056 0.000308 0.0175 0.00812 0.0266 0.0228 0.00145
2R:20762440-20762590:plus -33.8571 -0.100917 3.58716 9.3211 -17.7551 9.79592 -14.8899 1.59722
3.46789 0.0301 0.0107 0.00316 0.000391 0.00658 0.0000183 0.00877 0.00993 0.00582
2R:20763260-20763410:minus -33.6327 -1.34862 6.56881 6.27523 10.6531 -17.2755 -24.0826 1.15278
5.6055 0.029 0.016 0.000888 0.00206 0.0000102 0.00544 0.0415 0.0115 0.00232
2R:20763260-20763410:plus -41.4796 5.33028 -4.91743 6.00917 -17.7551 -9.02041 -29.9174 2.69444 -4.84404
0.0577 0.00142 0.0483 0.00255 0.00658 0.00213 0.0899 0.00669 0.0701
2R:20769460-20769610:minus -21.8878 6.80734 1.6789 2.49541 -18.2245 -18.2755 -21.1835 0.736111
2.16514 0.00258 0.000716 0.0065 0.0114 0.00877 0.0112 0.0254 0.0132 0.00879
2R:20769460-20769610:plus -11.9592 -0.825688 5.24771 1.84404 -27.5 -25.7551 -1.30275 -3.97222
6.29358 0.000301 0.0136 0.0016 0.0154 0.0337 0.0208 0.000379 0.0489 0.00161
2R:20769980-20770130:minus -14.2959 -4.27523 2.97248 4.13761 -18.7143 -9.53061 -17.9908 2.5 3.14679
0.00129 0.0364 0.00401 0.00546 0.0136 0.0031 0.0149 0.0072 0.00666
2R:20769980-20770130:plus -23.5204 2.61468 3.23853 5.58716 -17.3776 -27.6327 -0.541284 -2.98611
-0.229358 0.00645 0.004 0.00362 0.00334 0.00419 0.043 0.000321 0.0385 0.0202
2R:20770260-20770410:minus -34.1122 -4.14679 7.26606 0.825688 -18.449 -19.3061 -14.2936 0.0138889
7.88991 0.0312 0.0352 0.000638 0.0216 0.0107 0.0187 0.00774 0.0167 0.000688
2R:20770260-20770410:plus -23.1122 3.57798 4.18349 1.84404 -18.5306 -8.5 -22.0367 -1.125 9.42202
0.0053 0.00281 0.00249 0.0154 0.0118 0.00126 0.0297 0.0234 0.000349
2R:2077680-2077830:minus -3.33673 0.431193 3.57798 4.3211 -18.1224 -8.86735 -23.7248 -3.94444 -0.440367
0.0000599 0.00887 0.00317 0.00514 0.00782 0.00198 0.0394 0.0486 0.0216
2R:2077680-2077830:plus -32.1531 9.11009 -0.348624 2.29358 -26.7041 -8.87755 -14.9725 4 4.21101
0.0179 0.000212 0.013 0.0124 0.0193 0.00198 0.00892 0.00402 0.00396
2R:20791460-20791610:minus -21.8163 -0.724771 -1.69725 2.77064 -18.449 -17.9388 -4.69725 1.48611

2.06422 0.00244 0.0131 0.0197 0.01 0.0107 0.00738 0.00091 0.0103 0.00912
2R:20791460-20791610:plus -20.5918 -1.11927 1.11927 -0.0733945 -27.3367 -18.5 -19.422 0.5 0.275229
0.00169 0.0149 0.00793 0.0274 0.0317 0.0131 0.0187 0.0143 0.0171
2R:20815820-20815970:minus -19.8878 -1.50459 2.65138 5.16514 -17.0816 -8.30612 -21.2294 4.15278
0.752294 0.00164 0.0168 0.00453 0.00424 0.00334 0.000973 0.0256 0.00377 0.0152
2R:20815820-20815970:plus -41.3673 -2.93578 2.11927 5.59633 1.87755 -8.57143 -17.5596 6.29167 2.27523
0.0551 0.0253 0.00553 0.00331 0.0000492 0.00149 0.014 0.00144 0.00846
2R:20825820-20825970:minus -14.7449 13.7706 7 10.5963 -27.2347 -8.45918 -3.85321 4.22222 -1.11009
0.00157 0.0000111 0.000727 0.0001 0.0283 0.00111 0.000716 0.00367 0.0269
2R:20825820-20825970:plus -22.9286 13.4592 3.59633 2.98165 -18.5204 -18.0816 -17.945 7.15278 1.53211
0.0046 0.000064 0.00315 0.00905 0.0117 0.00934 0.0148 0.000928 0.0116
2R:20827960-20828110:minus -23.9694 1.78899 0.330275 2.95413 -18.4592 -18.4898 -6.79817 -1.80556
9.88073 0.0071 0.00542 0.0104 0.00917 0.0109 0.0129 0.00161 0.0283 0.000206
2R:20827960-20828110:plus -22.7347 -3.6422 8.69725 1.09174 -27.3061 -8.53061 -6.20183 -2.58333 0.321101
0.00403 0.0307 0.000299 0.02 0.0315 0.00136 0.00138 0.0348 0.0169
2R:20830320-20830470:minus -22.898 0.192661 -0.697248 7.16514 -9.16327 -9.72449 -20.1101 5.86111
-0.53211 0.00442 0.00965 0.0145 0.00116 0.00249 0.00352 0.021 0.00177 0.0222
2R:20830320-20830470:plus -22.9592 -3.25688 2.49541 9.59633 -8.56122 -18.0816 -10.1376 1.88889 1.23853
0.00475 0.0277 0.00481 0.000275 0.00112 0.00934 0.00316 0.00896 0.0132
2R:20832140-20832290:minus -23.0714 0.40367 5.45872 2.93578 -8.86735 -18.7551 -11.1743 -3.83333
-2.93578 0.0052 0.00896 0.00147 0.00927 0.00173 0.0141 0.00387 0.0473 0.0439
2R:20832140-20832290:plus -13.2143 1.43119 0.495413 2.53211 -18.4592 -18.7551 -5.57798 -10.8056 9
0.000714 0.00619 0.00983 0.0113 0.0109 0.0141 0.00117 0.173 0.000437
2R:20832960-20833110:minus -12.7347 -7.76147 0.53211 1.42202 -17.4082 -16.9388 -13.1009 -4.13889
6.7156 0.000468 0.0871 0.00971 0.0182 0.00421 0.00433 0.00592 0.0508 0.00144
2R:20832960-20833110:plus -12.8878 -0.431193 1.77982 1.38532 -18.7857 -18.3469 -12.4862 -2.91667
7.73394 0.000523 0.0119 0.00627 0.0185 0.0148 0.0124 0.00514 0.0378 0.000784
2R:20835820-20835970:minus -23.9592 1.66055 11.7248 3.42202 -8.93878 -18.3061 -20.7615 1.16667
2.99083 0.00708 0.00569 0.0000516 0.00751 0.00216 0.0114 0.0236 0.0115 0.00706
2R:20835820-20835970:plus -23.0714 3.30275 11.1835 2.66055 -17.449 -9.79592 -13.156 -2.30556 4.02752
0.0052 0.00311 0.000071 0.0106 0.00484 0.00377 0.00599 0.0323 0.00428
2R:20850060-20850210:minus -20.398 -2.00917 0.513761 6.02752 -8.26531 -18.4184 -13.422 10.8194
3.01835 0.00166 0.0195 0.00977 0.00246 0.000934 0.0127 0.00637 0.0000883 0.00697
2R:20850060-20850210:plus -31.2143 0.266055 -1.15596 2.89908 -18.6837 -18.2755 -11.422 6.16667 -1.75229
0.0111 0.0094 0.0168 0.00938 0.0126 0.0112 0.00407 0.00153 0.0312
2R:20850980-20851130:minus -4.89796 -0.963303 2.85321 5.94495 -18.7857 -19.0816 -13.2752 6.11111
11.8532 0.000208 0.0142 0.0042 0.00261 0.0148 0.017 0.00616 0.00157 0.0000428
2R:20850980-20851130:plus -24.2551 0.522936 1.6789 0.449541 -18.7857 0.0510204 -6.20183 1.40278
4.56881 0.00768 0.00859 0.0065 0.024 0.0148 0.000349 0.00138 0.0106 0.00356
2R:20851720-20851870:minus -30.9286 8.33028 -4.31193 3.6055 -17.6735 -19.3776 -14.4862 -1.51389
6.11927 0.0104 0.000319 0.0413 0.00686 0.00572 0.0195 0.00807 0.0261 0.00178
2R:20851720-20851870:plus -22.7755 3.73394 1.56881 5.74312 -8.97959 -18.1224 -16.2661 -0.597222
5.33945 0.00412 0.00266 0.00676 0.00302 0.0023 0.00988 0.0114 0.0201 0.00271
2R:20852580-20852730:minus -31.1735 0.697248 6.26606 3.50459 -18.3061 -7.45918 -16.3486 12.8611
1.90826 0.0109 0.00807 0.00103 0.00713 0.00884 0.000582 0.0115 0.0000122 0.00974
2R:20852580-20852730:plus -22.2245 8.29358 -0.46789 3.76147 -18.8265 -18.3776 -22.633 2.5 3.52294
0.00325 0.000326 0.0135 0.00645 0.0149 0.0125 0.033 0.0072 0.00558
2R:20856980-20857130:minus -22.4388 13.4592 1.34862 2.93578 -17.7143 -17.4286 -17.7706 -1.16667
1.85321 0.00348 0.000064 0.00731 0.00927 0.00627 0.00586 0.0144 0.0237 0.01
2R:20856980-20857130:plus -31.7347 -0.669725 10.0275 6.89908 20.1735 10.5306 -14.5321 1.27778
1.38532 0.0149 0.0129 0.000139 0.00134 5.68e-07 5.67e-06 0.00814 0.0111 0.0123
2R:20874440-20874590:minus -32.3673 2.65138 3.22018 3.26606 -8.60204 -17.9796 -20.0734 -4.80556
-3.66055 0.0189 0.00395 0.00365 0.00802 0.00125 0.00764 0.0209 0.0591 0.0513
2R:20874440-20874590:plus -31.398 1.20183 12.7156 7.29358 -18.4184 -27.5306 -5.20183 -3.80556 1.37615

0.0117	0.00673	0.000026	0.00104	0.0101	0.0374	0.00105	0.047	0.0123
2R:20886860-20887010:minus	-22.9286	8.33945	3.17431	3.62385	-26.7449	0.0204082	-16.3119	5.75
0.93578	0.0046	0.000317	0.00371	0.00673	0.0195	0.000377	0.0114	0.00187	0.0145
2R:20886860-20887010:plus	-32.7755	-0.40367	3.19266	9.33028	-18.3776	-10.0204	-11.9083	5.19444	6.7156
0.0234	0.0118	0.00369	0.000374	0.00926	0.00399	0.00452	0.00241	0.00144
2R:20888100-20888250:minus	-12.1122	-3.6422	5.33028	10.5872	-18.4592	-17.7857	-19.2752	-1.15278
0.330275	0.000336	0.0307	0.00155	0.000115	0.0109	0.0068	0.0182	0.0236	0.0168
2R:20888100-20888250:plus	-24.4796	5.3945	-4.3945	5.55046	-17.449	-17.2755	-19.1009	1.27778	1.70642
0.00804	0.00138	0.0422	0.00345	0.00484	0.00544	0.0177	0.0111	0.0108
2R:20889300-20889450:minus	-5.15306	5.08257	3.65138	2.55046	-18.3469	-18.2041	-10.0734	-0.277778
2.21101	0.000226	0.00158	0.00308	0.0112	0.00894	0.0103	0.00312	0.0182	0.00862
2R:20889300-20889450:plus	-42.551	6.78899	2.48624	6.74312	-18.2245	-36.7449	-12	0.263889	3.51376
0.0803	0.000723	0.00483	0.0015	0.00877	0.119	0.00461	0.0154	0.00566
2R:20889640-20889790:minus	-31.9286	2.3945	-4.11927	3.62385	-16.449	-26.8265	-15.6789	2.66667
7.46789	0.0166	0.00434	0.0392	0.00673	0.00307	0.0285	0.0102	0.00677	0.00102
2R:20889640-20889790:plus	-32.9592	0.733945	7.77982	4.17431	-18.6837	-9.5	-7.02752	3.52778	7.53211
0.0252	0.00797	0.000492	0.00537	0.0126	0.00294	0.0017	0.00486	0.000968
2R:20898220-20898370:minus	-33.7755	4.91743	4.54128	3.75229	-18.4184	-27.6327	-13.5046	-0.430556
5.66055	0.0297	0.00169	0.00215	0.00649	0.0101	0.043	0.00649	0.0191	0.00227
2R:20898220-20898370:plus	-32.1429	-0.724771	3.41284	5.57798	-7.67347	-18.2347	-6.97248	2.47222
9.66055	0.0179	0.0131	0.00338	0.0034	0.00055	0.0104	0.00168	0.00727	0.000268
2R:20898940-20899090:minus	-23.3367	1.44037	-1.56881	7.95413	-18.2653	-28.1837	-15.9174	2.65278
7.58716	0.00614	0.00617	0.019	0.000599	0.00882	0.0584	0.0107	0.0068	0.000954
2R:20898940-20899090:plus	-14.7041	11.1468	3.55046	0.568807	-8.93878	-19.2755	-1.89908	6.25	0.66055
0.00154	0.000075	0.00321	0.0232	0.00216	0.0185	0.000434	0.00147	0.0156
2R:20899460-20899610:minus	-21.5918	6.87156	-3.10092	3.52294	0.877551	-16.3469	-21.3578	1.80556
-3.14679	0.00209	0.000694	0.0297	0.00706	0.00019	0.00416	0.0262	0.00923	0.0463
2R:20899460-20899610:plus	-12.7755	1.3211	3.18349	9.44954	-9.27551	0.122449	-14.5872	-0.333333
-0.908257	0.000477	0.00644	0.0037	0.000326	0.00284	0.000312	0.00824	0.0185	0.0255
2R:20899840-20899990:minus	-31.2551	-5.06422	1.3578	5.06422	-26.1939	-26.4898	-15.2936	3.44444
-0.385321	0.0113	0.0449	0.00728	0.00433	0.0175	0.0225	0.00951	0.00502	0.0211
2R:20899840-20899990:plus	-31.5204	1.07339	-3.55963	6.00917	-18.7959	-16.051	-21.3394	1.625	6.6422
0.0131	0.00705	0.0337	0.00255	0.0148	0.00408	0.0262	0.00983	0.00148
2R:20940000-20940150:minus	-3.22449	1.53211	-2.98165	6.21101	-17.3061	-8.72449	-8.91743	1.15278
7.37615	0.0000495	0.00596	0.0287	0.00213	0.00389	0.00169	0.00252	0.0115	0.00107
2R:20940000-20940150:plus	-38.949	3.13761	2.38532	6.31193	-17.7143	-27.2653	-14.6514	0	3.88073
0.0323	0.00331	0.00501	0.00199	0.00627	0.0333	0.00835	0.0167	0.00475
2R:20949200-20949350:minus	-22.4898	0.422018	-0.46789	11.1927	-17.4184	0.0510204	-28.4771	8.13889
2.33028	0.00353	0.0089	0.0135	0.0000254	0.00457	0.000349	0.0734	0.000539	0.00836
2R:20949200-20949350:plus	-31.4796	-1.56881	1.79817	9.33028	-18.449	-19.0102	-20.633	-1.79167	0.12844
0.0128	0.0171	0.00623	0.000374	0.0107	0.0159	0.023	0.0282	0.0179
2R:20968340-20968490:minus	-23.8878	-2.02752	0.798165	2	-9.12245	-18.0102	-16.5413	6.80556	1.95413
0.00692	0.0196	0.00886	0.0143	0.00238	0.00812	0.0119	0.00111	0.00964
2R:20968340-20968490:plus	-33.4082	0.458716	3.68807	9.92661	-17.6429	-9.02041	-4.62385	3.125	4.80734
0.0283	0.00879	0.00304	0.000169	0.00562	0.00213	0.000891	0.00569	0.00334
2R:20971920-20972070:minus	-33.5918	0.688073	7.44954	9.76147	-18.6429	1.09184	-6.44037	1.44444
2.75229	0.0289	0.0081	0.000581	0.000212	0.0121	0.000091	0.00147	0.0105	0.00748
2R:20971920-20972070:plus	-32.9184	4.74312	-0.633028	7.00917	-27.1531	-17.2755	-14.6789	4.30556
7.68807	0.0248	0.00181	0.0142	0.00126	0.0249	0.00544	0.0084	0.00354	0.000841
2R:2101660-2101810:minus	-31.8469	9.54128	8.66972	0.00917431	-27.2653	-19.1224	-11.1743	1.90278
9.88073	0.0162	0.000168	0.000303	0.0269	0.0302	0.0175	0.00387	0.00892	0.000206
2R:2101660-2101810:plus	-14.3673	1.19266	13.8532	7.2844	-9.20408	-8.61224	-16.2569	-3.76389	-0.981651
0.00132	0.00675	8.77e-06	0.00105	0.00262	0.00152	0.0113	0.0466	0.026
2R:2106920-2107070:minus	-33.5918	3.7551	-0.522936	1.05505	-26.3061	-9.02041	-16.789	5.77778

-1.7156 0.0289 0.00122 0.0137 0.0204 0.0181 0.00213 0.0124 0.00184 0.0309
2R:2106920-2107070:plus -32.4388 -3.11927 12.0275 3.40367 -18.7143 -36.1122 -18.3211 5.84722 4.14679
0.0195 0.0266 0.0000422 0.00759 0.0136 0.0878 0.0157 0.00178 0.00406
2R:2107300-2107450:minus -3.82653 -2.06422 4.33945 5.30275 -18.6837 -8.72449 -3.55963 2.83333 1.48624
0.000107 0.0198 0.00234 0.00396 0.0126 0.00169 0.00066 0.00635 0.0118
2R:2107300-2107450:plus -40.398 -3.36697 3.40367 -0.302752 -18.051 -9.57143 -14.6422 -0.125
2.22936 0.0397 0.0285 0.0034 0.0293 0.00746 0.00326 0.00834 0.0174 0.00858
2R:2108560-2108710:minus -22.9592 -3.80734 -4.84404 -0.110092 -27.7551 -9.79592 -21.578 4.88889
7.31193 0.00475 0.0321 0.0474 0.0277 0.0385 0.00377 0.0273 0.00276 0.00112
2R:2108560-2108710:plus -32.6633 5.16514 -0.477064 2.69725 -18.5612 -28.4082 -16.9083 0.208333
3.6422 0.0216 0.00152 0.0135 0.0104 0.0119 0.0615 0.0126 0.0157 0.00516
2R:2108940-2109090:minus -24.2245 2.17431 5.22936 0.862385 -18.2245 -18.0816 -15.0642 8.47222 6.18349
0.00763 0.00471 0.00162 0.0214 0.00877 0.00934 0.00909 0.000443 0.00167
2R:2108940-2109090:plus -32.551 9.44954 -0.733945 -2.37615 -17.7551 -18.4184 -14.945 11 7.04587
0.0207 0.000177 0.0147 0.0578 0.00658 0.0127 0.00887 0.0000763 0.00132
2R:2109960-2110110:minus -32.6327 0.706422 1.40367 3.70642 -26.7041 -9.64286 -15.9817 2.25 -1.06422
0.0212 0.00805 0.00717 0.0066 0.0193 0.00339 0.0108 0.00788 0.0266
2R:2109960-2110110:plus -33.1122 3.02752 5.01835 2.81651 -19.051 -8.79592 -18.3119 4.22222 -2.69725
0.0271 0.00344 0.00176 0.00982 0.0162 0.0019 0.0157 0.00367 0.0411
2R:2111440-2111590:minus -31.6327 -3.52294 0.0458716 5.43119 -9.5 -17.9796 -17.9174 -1.88889 1.34862
0.0138 0.0297 0.0114 0.00371 0.00293 0.00764 0.0148 0.0289 0.0125
2R:2111440-2111590:plus -22.5816 3.97959 -1.81651 2.49541 -18.3776 -9.16327 -13.3211 7.69444 6.12844
0.00361 0.000669 0.0205 0.0114 0.00926 0.00223 0.00622 0.000693 0.00174
2R:21140680-21140830:minus -32.3265 -2.99083 -0.541284 -0.495413 -18.3776 -8.16327 -25.0642
4.90278 5.70642 0.0187 0.0257 0.0138 0.0311 0.00926 0.000783 0.0476 0.00274 0.00222
2R:21140680-21140830:plus -32.9184 -1 -1.55046 0.981651 -17.2245 1.05102 -17.9817 3.97222 7.68807
0.0248 0.0143 0.0189 0.0207 0.00384 0.000107 0.0149 0.00407 0.000841
2R:2123520-2123670:minus -22.7041 -1.77064 1.25688 5.88073 -18.4184 -37.1531 -20.5963 -2.55556 -2.72477
0.00395 0.0182 0.00755 0.00275 0.0101 0.138 0.0229 0.0345 0.0414
2R:2123520-2123670:plus -30.6327 -0.862385 3.6422 3.00917 -25.9286 -0.173469 -10.3211 -0.305556
9.66055 0.00968 0.0137 0.00309 0.00894 0.0168 0.000456 0.00327 0.0184 0.000268
2R:2129400-2129550:minus -31.398 1.51376 1.36697 7.46789 -17.7143 -8.60204 -9.11927 1.31944 -0.724771
0.0117 0.006 0.00726 0.000868 0.00627 0.00151 0.00262 0.0109 0.0241
2R:2129400-2129550:plus -22.8571 -2.94495 0.917431 9.3211 -8.93878 -8.23469 -3.29358 -1.48611 2.93578
0.00427 0.0254 0.00851 0.000391 0.00216 0.000875 0.000614 0.0259 0.00717
2R:2154880-2155030:minus -22.2245 13.5306 1.13761 11.2936 -18.4082 -18.051 -20.7798 1.36111 -4.6789
0.00325 0.0000206 0.00788 0.0000139 0.00937 0.00886 0.0236 0.0108 0.067
2R:2154880-2155030:plus -21.9694 4.81651 -0.715596 3.87156 -19.0204 -9.20408 -9.66972 3.45833
1.70642 0.00282 0.00175 0.0146 0.00618 0.0158 0.00225 0.0029 0.005 0.0108
2R:2389280-2389430:minus -22.4694 -1 0.981651 3.85321 -17.2245 -37.449 -8.47706 0.180556 7.84404
0.0035 0.0143 0.00832 0.00623 0.00384 0.164 0.00232 0.0158 0.000701
2R:2389280-2389430:plus -2.07143 -3.41284 6.41284 11.0183 -7.60204 -26.3367 -10.6147 -4.15278 5.70642
0.0000251 0.0289 0.000959 0.000052 0.000466 0.0221 0.00346 0.051 0.00222
2R:2501120-2501270:minus -12.8469 -0.46789 14.0917 3.61468 -18.6429 -9.57143 -12.0092 2.22222 2.98165
0.000502 0.0121 6.39e-06 0.0068 0.0121 0.00326 0.00462 0.00796 0.00707
2R:2501120-2501270:plus -41.449 -0.0825688 2 -0.0366972 -17.1531 -26.8265 -18.7798 0.416667
-0.642202 0.0564 0.0106 0.00578 0.0271 0.00364 0.0285 0.0169 0.0147 0.0234
2R:2515780-2515930:minus -14.8163 1.62385 2.81651 8.80734 -17.4898 0.122449 -19.1651 3.47222 4.40367
0.00159 0.00577 0.00426 0.000518 0.0053 0.000312 0.0179 0.00497 0.00373
2R:2515780-2515930:plus -13.1531 3.70642 8.00917 4.11009 -9.27551 -7.23469 -14.0826 7.72222 6.25688
0.00067 0.00268 0.000435 0.00553 0.00284 0.000557 0.00739 0.000682 0.00163
2R:2516640-2516790:minus -14.6735 5.40367 4.81651 6.00917 -17.4184 -17.051 1.62385 -1.70833 2.37615
0.00152 0.00138 0.00192 0.00255 0.00457 0.00479 0.000185 0.0275 0.00825
2R:2516640-2516790:plus -13.7041 6.09174 0.440367 1.55963 -18.5306 -18.2755 -8.83486 1.16667 0.394495

0.00104 0.00101 0.01 0.0173 0.0118 0.0112 0.00248 0.0115 0.0166
2R:2517120-2517270:minus -31.3265 3.11009 6.33945 5.42202 -27.1939 -8.79592 -20.9817 4.125 2.33028
0.0114 0.00334 0.000991 0.00374 0.0267 0.0019 0.0245 0.00382 0.00836
2R:2517120-2517270:plus -13.1837 1.24771 8.7156 2.83486 -16.7143 -26.602 -5.99083 0.111111 3.98165
0.000705 0.00661 0.000296 0.0097 0.00319 0.0248 0.00131 0.0162 0.00439
2R:2517500-2517650:minus -30.8163 -5.06422 8.55046 1.84404 -17.7143 -18.2755 -16.6239 -0.944444
-0.183486 0.0103 0.0449 0.000323 0.0154 0.00627 0.0112 0.0121 0.0222 0.0197
2R:2517500-2517650:plus -23.6633 -1.68807 2.66055 1.88073 -18.7143 -9.64286 -16.9083 -1.59722 -1.21101
0.00665 0.0177 0.00452 0.0152 0.0136 0.00339 0.0126 0.0267 0.0277
2R:2517960-2518110:minus -13.0408 -2.21101 7.08257 7.55963 -17.3776 -18.0816 -20.9725 1.75 -2.11009
0.000576 0.0206 0.000699 0.000807 0.00419 0.00934 0.0245 0.00941 0.0343
2R:2517960-2518110:plus -22.6327 3.64286 1.6789 4.94495 -9.16327 -18.5612 -19.8716 4.45833 1.36697
0.00373 0.00159 0.0065 0.00444 0.00249 0.0132 0.0201 0.00332 0.0124
2R:2519000-2519150:minus -22.7755 0.357798 -0.440367 2.27523 0.806122 -18.2041 -17.7798 8.26389
-0.688073 0.00412 0.00911 0.0134 0.0125 0.000229 0.0103 0.0145 0.000502 0.0238
2R:2519000-2519150:plus -13.1837 1.06422 1.82569 1.45872 -8.96939 -25.602 8.15596 3.77778 10.3394
0.000705 0.00707 0.00616 0.0179 0.00226 0.0207 0.0000228 0.0044 0.000112
2R:2519960-2520110:minus -23.1122 8.38532 0.522936 4.0367 -8.64286 -7.86735 -13.7615 2.44444 3.66972
0.0053 0.000309 0.00974 0.00578 0.00139 0.00073 0.00688 0.00734 0.00514
2R:2519960-2520110:plus -23.1122 -2.7156 3.22936 3.41284 -17.1837 -17.8571 -16.5138 -1.36111 4.91743
0.0053 0.0238 0.00363 0.00756 0.00366 0.00719 0.0118 0.025 0.00321
2R:2520540-2520690:minus -30.4388 0.614679 4.6422 7.61468 -18.449 -18.0816 -12.1835 10.1806 5.80734
0.00923 0.00832 0.00206 0.000779 0.0107 0.00934 0.0048 0.000144 0.00205
2R:2520540-2520690:plus -24.7449 3.24771 -3.02752 0.00917431 -8.63265 -19.1531 -18.3945 6.33333
1.36697 0.00827 0.00317 0.0291 0.0269 0.0013 0.0177 0.0159 0.00141 0.0124
2R:2523720-2523870:minus -31.9286 13.4592 3.08257 5.46789 0.836735 -10.0204 -23.4862 -1.08333 -5.01835
0.0166 0.000064 0.00385 0.00362 0.000215 0.00399 0.038 0.0231 0.073
2R:2523720-2523870:plus -32.9286 5.66055 10.9908 3.97248 -18.6837 -25.8265 -20.6422 -1.5 11.8532 0.025
0.00123 0.0000806 0.00587 0.0126 0.0212 0.0231 0.026 0.0000428
2R:2532640-2532790:minus -30.6633 6.15596 0.440367 8.98165 -8.93878 -19.051 1.93578 2.88889 3.46789
0.00976 0.000984 0.01 0.000461 0.00216 0.0165 0.000168 0.00622 0.00582
2R:2532640-2532790:plus -13.1122 1.41284 1.21101 6.08257 -26.9694 -17.4898 -18.1101 -0.805556
7.07339 0.000632 0.00623 0.00768 0.00236 0.023 0.0059 0.0152 0.0213 0.0013
2R:2536080-2536230:minus -12.6224 1.83486 -1.79817 2.66972 -8.37755 -18.2755 -1.98165 -7.625 3.29358
0.000453 0.00533 0.0204 0.0106 0.00106 0.0112 0.000442 0.104 0.00623
2R:2536080-2536230:plus -31.4388 1.61468 -0.587156 -1.33028 -18.2245 -36.9286 -16.7248 -2.44444
5.12844 0.0122 0.00578 0.014 0.0409 0.00877 0.123 0.0123 0.0335 0.00301
2R:2537960-2538110:minus -33.2653 1.73394 -0.862385 5.90826 -18.8265 -18.051 -28.8532 4.56944
1.70642 0.0277 0.00553 0.0153 0.00271 0.0149 0.00886 0.0773 0.00317 0.0108
2R:2537960-2538110:plus -40.2959 9.0367 4.59633 2.14679 -18.0816 -18.5714 -1.3578 0.972222 1.00917
0.0389 0.000219 0.0021 0.0133 0.00759 0.0136 0.000384 0.0123 0.0143
2R:2540380-2540530:minus -30.3265 1.33028 -2.38532 1.89908 -27.2245 -27.5612 -14.0092 8.52778 7.31193
0.0089 0.00642 0.0242 0.0151 0.0272 0.0393 0.00727 0.000429 0.00112
2R:2540380-2540530:plus -41.2959 -4.12844 -2.55963 2.47706 -8.93878 -9.53061 -12.8165 3.29167 -1.00917
0.0529 0.035 0.0254 0.0115 0.00216 0.0031 0.00554 0.00533 0.0262
2R:2540540-2540690:minus -32.3673 3.97959 0.917431 3.52294 -18.6837 0.602041 -25.6147 6.90278 2.20183
0.0189 0.000669 0.00851 0.00706 0.0126 0.000188 0.051 0.00106 0.00869
2R:2540540-2540690:plus -23.1429 4.89908 4.22018 2.88073 -17.4184 -0.173469 -12.8532 -0.236111
0.495413 0.00533 0.0017 0.00245 0.00945 0.00457 0.000456 0.00559 0.018 0.0162
2R:2543820-2543970:minus -24.449 -0.477064 2.6789 3.45872 -18.4898 1.31633 -17.0734 0.263889
2.98165 0.00802 0.0121 0.00449 0.00731 0.0116 0.000066 0.013 0.0154 0.00707
2R:2543820-2543970:plus -22.7755 4.9633 -0.330275 2.82569 -18.7143 -19.4184 -17.6606 -3.27778
0.165138 0.00412 0.00165 0.0129 0.00976 0.0136 0.0198 0.0142 0.0414 0.0177
2R:2549680-2549830:minus -23.7755 -4.3211 6.6789 6.72477 20.4694 9.7551 -19.1009 1.5 0.357798

0.00678 0.0369 0.000844 0.00154 1.65e-07 0.0000198 0.0177 0.0103 0.0166
2R:2549680-2549830:plus -32.9286 0.486239 2.59633 2.08257 -26.7449 -17.7143 -17.1651 -7.06944 2.21101
0.025 0.00871 0.00463 0.0137 0.0195 0.00634 0.0132 0.0938 0.00862
2R:2550260-2550410:minus -22.5918 4.13761 5.44954 7.00917 -17.8878 -27.6327 -18.2385 0.180556 1.75229
0.00365 0.00228 0.00147 0.00126 0.00704 0.043 0.0155 0.0158 0.0104
2R:2550260-2550410:plus -22.7041 -1.6422 3.14679 2.19266 -26.7449 -19.1939 -20.6055 3.48611 4.91743
0.00395 0.0175 0.00375 0.0131 0.0195 0.0178 0.0229 0.00494 0.00321
2R:2550680-2550830:minus -22.7653 9.06422 1.37615 6.66055 -17.7143 -27.6327 -6.06422 1.94444 4.55963
0.00405 0.000216 0.00724 0.00158 0.00627 0.043 0.00133 0.00879 0.00357
2R:2550680-2550830:plus -21.4388 -0.330275 2.37615 1.40367 -7.89796 -16.2755 -22.8165 7.52778
-0.743119 0.002 0.0115 0.00503 0.0184 0.000678 0.00413 0.034 0.00076 0.0243
2R:2551220-2551370:minus -31.1429 -2.13761 6.95413 2.6422 -18.1224 -27.0408 -0.559633 -4.05556
3.78899 0.0108 0.0202 0.000743 0.0107 0.00782 0.0307 0.000322 0.0499 0.00487
2R:2551220-2551370:plus -21.898 0.779817 -0.0825688 1.68807 -17.7857 -9.34694 -7.63303 -3.84722
-0.862385 0.00259 0.00784 0.0119 0.0164 0.00676 0.00261 0.00195 0.0475 0.0251
2R:2553700-2553850:minus -24.2959 5.46789 6.09174 3.92661 -8.16327 -18.2755 -15.5963 0.527778 -0.348624
0.00781 0.00134 0.00111 0.00601 0.000873 0.0112 0.0101 0.0142 0.0209
2R:2553700-2553850:plus -30.6224 0.733945 5.01835 5.93578 -26.9694 -18.5714 -15.8807 0.611111 -4.14679
0.00964 0.00797 0.00176 0.00264 0.023 0.0136 0.0106 0.0138 0.0576
2R:2555680-2555830:minus -31.1837 -0.752294 4.00917 5.56881 -17.4184 -7.93878 -9.40367 1.76389
-4.68807 0.0109 0.0132 0.00267 0.00342 0.00457 0.000736 0.00276 0.00937 0.0672
2R:2555680-2555830:plus -24.3265 1.44954 13.7523 7.29358 -9.0102 -18.5 -16.8624 2.80556 -0.614679
0.00784 0.00615 9.94e-06 0.00104 0.00236 0.0131 0.0125 0.00642 0.0231
2R:2556420-2556570:minus -13.9184 -0.504587 9.52294 2.66972 -17.7857 -26.8265 -11.0367 1.79167
0.165138 0.00112 0.0122 0.000187 0.0106 0.00676 0.0285 0.00376 0.00928 0.0177
2R:2556420-2556570:plus -22.8878 -3.12844 2.7156 4.91743 -26.2653 -19.3469 -4.63303 -2.91667 -3.15596
0.00441 0.0267 0.00442 0.00447 0.018 0.0194 0.000894 0.0378 0.0464
2R:2560860-2561010:minus -24.1429 -1.11009 4.53211 6.11009 -18.6429 -8.38776 -9.89908 6.52778 7.6422
0.0074 0.0148 0.00216 0.00233 0.0121 0.00101 0.00302 0.00128 0.000893
2R:2560860-2561010:plus -14.4796 9.18349 0.87156 5.02752 -18.5306 -9.82653 -0.944954 -0.236111
3.45872 0.00142 0.000203 0.00864 0.00435 0.0118 0.00381 0.000351 0.018 0.00591
2R:2563260-2563410:minus -23.9184 8.77064 1.24771 -0.174312 -28.2653 -18.4184 -24.3853 0.902778
-6.37615 0.00695 0.000253 0.00758 0.0283 0.0537 0.0127 0.0434 0.0125 0.0975
2R:2563260-2563410:plus -22.9184 13.4592 0.972477 1.78899 0.27551 -8.02041 -7.63303 3.15278 1.22936
0.00447 0.000064 0.00835 0.0158 0.00035 0.000761 0.00195 0.00563 0.0132
2R:2567040-2567190:minus -31.8163 -1.68807 5.9633 0.321101 -27.3367 -18.5714 -16.1376 -2.56944 1.90826
0.016 0.0177 0.00117 0.0247 0.0317 0.0136 0.0111 0.0346 0.00974
2R:2567040-2567190:plus -13.3776 -1.90826 6.11009 10.8624 -17.3469 -8.16327 -13.9266 -0.944444
5.58716 0.000831 0.0189 0.0011 0.0000687 0.00394 0.000783 0.00714 0.0222 0.00237
2R:2584120-2584270:minus -22.2959 13.3878 0.431193 3.56881 -18.4898 0.244898 -6.9633 3.58333 -0.972477
0.00335 0.0000869 0.01 0.00699 0.0116 0.000285 0.00167 0.00475 0.0259
2R:2584120-2584270:plus -23.6224 4.19266 1.54128 5.37615 10.6531 -19.1531 -3.00917 6.45833 -0.743119
0.00658 0.00224 0.00683 0.00384 0.0000102 0.0177 0.000569 0.00133 0.0243
2R:2584600-2584750:minus -31.4388 1.26606 5.83486 2.80734 -18.1939 -18.2755 -11.9633 2.06944 -1.75229
0.0122 0.00657 0.00124 0.00985 0.0086 0.0112 0.00457 0.00841 0.0312
2R:2584600-2584750:plus -22.6939 -4.58716 4.88991 3.19266 -18.4184 -27.5612 -11.7248 4.05556 6.7156
0.00388 0.0396 0.00186 0.00829 0.0101 0.0393 0.00434 0.00393 0.00144
2R:2595460-2595610:minus -21.4796 3.88991 5.45872 2.9633 -17.3061 -17.051 -19.4862 -5.68056 1.99083
0.00202 0.00251 0.00147 0.00914 0.00389 0.00479 0.0189 0.0713 0.00946
2R:2595460-2595610:plus -13.0714 -2.45872 0.33945 3.23853 -17.6837 -17.4898 -15.8624 -2.22222 -5.3945
0.000594 0.0222 0.0104 0.00815 0.0058 0.0059 0.0106 0.0316 0.079
2R:2603280-2603430:minus -14.8163 6.16514 2.04587 7.00917 -27.3061 -9.57143 -5.42202 0.208333 5.48624
0.00159 0.00098 0.00568 0.00126 0.0315 0.00326 0.00112 0.0157 0.00251
2R:2603280-2603430:plus -21.6224 2.90826 8.43119 3.41284 -7.56122 -26.898 -10.6055 3.56944 -4.68807

0.00213 0.0036 0.000347 0.00756 0.000419 0.0302 0.00345 0.00478 0.0672
2R:2629300-2629450:minus -32.9592 3.64286 1.59633 2.58716 -18.449 -8.82653 -18.2294 0.458333 -7.83486
0.0252 0.00159 0.00669 0.011 0.0107 0.00191 0.0155 0.0145 0.131
2R:2629300-2629450:plus -30.3673 14.6789 -0.0825688 2.9633 -16.449 -9.72449 -1.92661 -1 5.17431
0.00897 3.91e-06 0.0119 0.00914 0.00307 0.00352 0.000437 0.0226 0.00296
2R:2629800-2629950:minus -21.7041 2.31193 15.6606 6.52294 -8.37755 -9.45918 -13.5688 2.19444 5.80734
0.00231 0.00448 3.94e-07 0.00174 0.00106 0.00279 0.00658 0.00804 0.00205
2R:2629800-2629950:plus -32.8163 -3.6789 14.1835 4.02752 -18.449 -35.602 -12.0734 3.54167 3.66972
0.024 0.031 5.88e-06 0.00581 0.0107 0.0803 0.00469 0.00483 0.00514
2R:2633840-2633990:minus -14.3673 0.12844 2.88991 5.2844 -26.9694 -26.8265 -19.6697 -1.15278 7.6422
0.00132 0.00987 0.00414 0.004 0.023 0.0285 0.0195 0.0236 0.000893
2R:2633840-2633990:plus -14.2959 8.49541 13.3761 9.44954 -16.8878 -8.5 -18.422 1.22222 -8.74312
0.00129 0.000292 0.0000145 0.000326 0.00326 0.00126 0.0159 0.0113 0.158
2R:2636880-2637030:minus -32.7755 -1.55963 -1.94495 7.43119 0.918367 -28.6327 -14.4771 -2.61111 12.2752
0.0234 0.017 0.0213 0.000939 0.000142 0.066 0.00805 0.035 0.0000141
2R:2636880-2637030:plus -24.3673 4.82569 3.34862 5.30275 -27.7041 -16.7857 -4.73394 0.861111 3.88991
0.00791 0.00175 0.00347 0.00396 0.038 0.00426 0.00092 0.0127 0.00467
2R:2644660-2644810:minus -14.9286 13.4592 3.56881 6.02752 -9.54082 -18.0102 -16.5321 4.47222 7.68807
0.0016 0.000064 0.00318 0.00246 0.00294 0.00812 0.0119 0.0033 0.000841
2R:2644660-2644810:plus -31.6633 4.68807 10.0092 7.55963 -18.0816 -17.7857 -18.1927 1.31944 1.29358
0.0141 0.00184 0.000141 0.000807 0.00759 0.0068 0.0154 0.0109 0.0128
2R:2646400-2646550:minus -13.1837 5.88991 11.3119 4.65138 -17.8571 -28.449 -11.9083 -1.58333 5.44954
0.000705 0.00111 0.0000659 0.00471 0.00698 0.0616 0.00452 0.0266 0.00253
2R:2646400-2646550:plus -21 2.2844 7.25688 3.69725 -9.12245 -9.72449 -18.2018 -1.02778 -0.825688
0.00186 0.00452 0.000641 0.00665 0.00238 0.00352 0.0154 0.0227 0.0249
2R:2669080-2669230:minus -22.3265 -1.50459 -0.908257 6.83486 -18.4898 -18.0816 -7.16514 9.22222
6.29358 0.00337 0.0168 0.0155 0.00142 0.0116 0.00934 0.00175 0.000278 0.00161
2R:2669080-2669230:plus -40.2551 3.90816 1.50459 2.51376 -18.3776 -17.5714 -15.4404 2.73611 -6.05505
0.0382 0.00102 0.00692 0.0113 0.00926 0.00616 0.00978 0.00659 0.09
2R:2676560-2676710:minus -4.66327 2.30275 2.48624 4.36697 -18.4898 -8.79592 0.889908 3.09722 -0.678899
0.000177 0.00449 0.00483 0.00505 0.0116 0.0019 0.000227 0.00575 0.0237
2R:2676560-2676710:plus -13.0714 0.238532 2.16514 5.34862 -27.1939 -17.7857 -6.66972 1.41667 -3.83486
0.000594 0.0095 0.00544 0.00387 0.0267 0.0068 0.00156 0.0106 0.0533
2R:2697100-2697250:minus -31.7347 2.85321 -0.587156 3.47706 -26.8878 -27.7143 -12.1835 -1.09722
5.3945 0.0149 0.00367 0.014 0.00724 0.0199 0.0447 0.0048 0.0232 0.00263
2R:2697100-2697250:plus -40.7449 0.330275 -3.98165 6.17431 -18.6735 -9.72449 -11.0826 1.20833 2.46789
0.0431 0.00919 0.0378 0.0022 0.0123 0.00352 0.0038 0.0113 0.00805
2R:2697880-2698030:minus -22.2551 1.68807 -2.84404 4.27523 -18.7143 -9.5 1.50459 0.611111 -0.220183
0.00327 0.00563 0.0276 0.00518 0.0136 0.00294 0.000191 0.0138 0.0201
2R:2697880-2698030:plus -13.7857 14.9266 -3.3945 1.46789 -18.4184 0.0510204 -14.1009 1.11111
0.220183 0.00108 2.37e-06 0.0322 0.0179 0.0101 0.000349 0.00742 0.0117 0.0174
2R:2698460-2698610:minus -32.1837 1.10092 -2.01835 3.08257 -8.93878 -17.7857 -14.7982 7.83333 6.17431
0.0181 0.00698 0.0217 0.0087 0.00216 0.0068 0.00861 0.000641 0.00171
2R:2698460-2698610:plus -40.6939 10.4587 -4.7156 7.27523 -18.449 -7.72449 -16.7982 -1.08333 -0.220183
0.0427 0.000106 0.0459 0.00109 0.0107 0.00068 0.0124 0.0231 0.0201
2R:2699860-2700010:minus -41.6633 0.577982 1.48624 7.33945 -9.20408 -9.37755 -7.31193 3.52778 0.908257
0.0636 0.00843 0.00696 0.00102 0.00262 0.00265 0.00181 0.00486 0.0147
2R:2699860-2700010:plus -22.7041 3.75229 4.51376 9.76147 -8.57143 -9.5 -15.7615 1.13889 9.87156
0.00395 0.00264 0.00218 0.000212 0.00113 0.00294 0.0104 0.0116 0.000217
2R:2758840-2758990:minus -23.3367 0.954128 5.11009 2.22018 -18.6735 -9.20408 -14.2477 -0.0694444
3.63303 0.00614 0.00736 0.0017 0.0129 0.0123 0.00225 0.00766 0.0171 0.00518
2R:2758840-2758990:plus -32.5918 2.40367 6.09174 6.12844 -18.9388 -8.94898 -12.6606 -1.38889 6.7156
0.021 0.00433 0.00111 0.00231 0.0151 0.00203 0.00535 0.0252 0.00144
2R:2759580-2759730:minus -14.5918 1.48624 2.46789 7.80734 -26.1531 -8.94898 -12.6147 1.90278 5.59633

0.00148 0.00606 0.00486 0.00063 0.0172 0.00203 0.00529 0.00892 0.00234
2R:2759580-2759730:plus -30.8367 -5.2844 3.65138 0.66055 -7.56122 -17.2755 -5.6789 -2.625 0.0275229
0.0103 0.0475 0.00308 0.0225 0.000419 0.00544 0.0012 0.0351 0.0183
2R:2760140-2760290:minus -21.5816 2.87156 4.9633 1.91743 -18.0816 0.540816 -11.8165 -2.66667 1.25688
0.00205 0.00365 0.00181 0.015 0.00759 0.0002 0.00443 0.0355 0.0131
2R:2760140-2760290:plus -13.2245 2.75229 6.52294 6.22936 -17.3776 -19.0102 -4 -0.194444 9.87156
0.000738 0.00381 0.000908 0.00209 0.00419 0.0159 0.000746 0.0178 0.000217
2R:2766240-2766390:minus -22.4082 -0.963303 7.27523 10.8624 -8.0102 -19.0102 -22.0642 11.2639
7.88991 0.00345 0.0142 0.000635 0.0000687 0.000826 0.0159 0.0298 0.0000608 0.000688
2R:2766240-2766390:plus -22.0306 5.88991 -2.22018 7 -9.5 1.02041 -15.8532 -2.13889 1.68807 0.00293
0.00111 0.0231 0.00132 0.00293 0.000118 0.0106 0.0309 0.0109
2R:2768320-2768470:minus -31.551 -2.19266 2.30275 0.688073 -17.1531 -0.540816 -12.5138 3.59722
1.34862 0.0134 0.0205 0.00517 0.0224 0.00364 0.00054 0.00517 0.00473 0.0125
2R:2768320-2768470:plus -12.2959 -2.40367 -0.366972 9.7156 -17.3776 -9.57143 -18.2018 -6.79167
-1.59633 0.000384 0.0218 0.0131 0.000242 0.00419 0.00326 0.0154 0.089 0.0301
2R:2773280-2773430:minus -41.9694 6.6055 5.65138 5.30275 -27.2347 -8.45918 -22.6055 2.375 1.2844
0.068 0.000793 0.00135 0.00396 0.0283 0.00111 0.0328 0.00753 0.0129
2R:2773280-2773430:plus -31.8061 1.55963 1.91743 0.568807 -27.2653 -17.2755 -19.2294 6.75 0.587156
0.0158 0.0059 0.00596 0.0232 0.0302 0.00544 0.0181 0.00114 0.016
2R:2859120-2859270:minus -13.1531 2.31193 2.3578 7.78899 20.2041 -17.051 22.2844 -2.44444 1.37615
0.00067 0.00448 0.00506 0.000642 3.64e-07 0.00479 3.16e-08 0.0335 0.0123
2R:2859120-2859270:plus -22.6633 3.45872 2.30275 2.58716 -17.4592 -26.5612 -10.4495 -1.72222 -0.715596
0.00384 0.00294 0.00517 0.011 0.00491 0.0237 0.00335 0.0276 0.0241
2R:2862500-2862650:minus -31.7041 -0.357798 3.23853 6.29358 -17.3776 -18.2347 -23.8073 0.208333
1.73394 0.0146 0.0116 0.00362 0.00202 0.00419 0.0104 0.0399 0.0157 0.0106
2R:2862500-2862650:plus -40.9184 0.899083 3.56881 5.93578 -18.7857 -27.9694 -6.57798 2.63889 3.16514
0.0443 0.00751 0.00318 0.00264 0.0148 0.0547 0.00152 0.00684 0.00656
2R:2862740-2862890:minus -31.7041 -0.357798 3.23853 6.29358 -27.1939 -0.244898 -23.8073 0.208333
1.73394 0.0146 0.0116 0.00362 0.00202 0.0267 0.000496 0.0399 0.0157 0.0106
2R:2862740-2862890:plus -32.398 14.8257 0.926606 0.00917431 -18.9796 -10.0918 -14.6606 3.44444
-1.22936 0.019 2.99e-06 0.00848 0.0269 0.0155 0.00404 0.00837 0.00502 0.0279
2R:2873200-2873350:minus -14.1122 -2.48624 6.59633 7.47706 20.1735 -18.051 6.56881 -1.31944 2.93578
0.00119 0.0223 0.000876 0.00085 5.68e-07 0.00886 0.000035 0.0247 0.00717
2R:2873200-2873350:plus -23.3367 13.4592 -1.18349 3.77064 0.836735 -8.5 -12.6239 3.75 -1.07339 0.00614
0.000064 0.0169 0.00637 0.000215 0.00126 0.0053 0.00445 0.0267
2R:2880260-2880410:minus -13.1531 -1 7.14679 7.21101 -17.4898 -18.0816 -1.68807 1.08333 -0.229358
0.00067 0.0143 0.000677 0.0011 0.0053 0.00934 0.000414 0.0118 0.0202
2R:2880260-2880410:plus -40.2143 2.7156 3.02752 8.6055 -7.82653 -27.4082 -13.0917 0.791667 5.66055
0.0372 0.00386 0.00393 0.000567 0.00059 0.0356 0.0059 0.013 0.00227
2R:2898720-2898870:minus -13.8776 0.972477 -2.88991 4.19266 -17.7143 -18.3469 -13.0459 -1.43056 -1.27523
0.0011 0.00731 0.028 0.00536 0.00627 0.0124 0.00584 0.0255 0.0282
2R:2898720-2898870:plus -14.3673 -1.82569 11.0183 4.76147 -18.4898 -18.2347 -24.4771 4.29167 -4.22936
0.00132 0.0185 0.0000791 0.00459 0.0116 0.0104 0.0439 0.00356 0.0588
2R:2899660-2899810:minus -31.9694 10.789 -2.14679 2.19266 -19.2755 -0.316327 -34.5138 0.736111
-7.48624 0.0168 0.0000894 0.0226 0.0131 0.0164 0.000524 0.153 0.0132 0.123
2R:2899660-2899810:plus -22.5102 1.56881 9.51376 -0.53211 20.4694 -9.34694 1.19266 6.54167 7.73394
0.00354 0.00588 0.000189 0.0314 1.65e-07 0.00261 0.000209 0.00127 0.000784
2R:2900280-2900430:minus -24.8265 1.29358 -1.26606 1.98165 -27.9694 -18.8265 -15.1651 3.11111 9.04587
0.0083 0.00651 0.0173 0.0145 0.0437 0.0148 0.00927 0.00572 0.000423
2R:2900280-2900430:plus -32.7347 3.64286 4.81651 6.84404 -26.9286 0.826531 -18.1101 6.65278 -6.33028
0.0225 0.00159 0.00192 0.0014 0.0214 0.000153 0.0152 0.0012 0.0963
2R:2900880-2901030:minus -39.3265 3.01835 4.00917 5.76147 -8.7449 -27.7857 -12.7431 2.31944 3.42202
0.0332 0.00346 0.00267 0.00295 0.00161 0.0466 0.00545 0.00769 0.00597
2R:2900880-2901030:plus -31.8061 1.7156 4.52294 2.51376 -27.4898 -18.8265 -13.2661 5.02778 -1.10092

0.0158 0.00557 0.00217 0.0113 0.0336 0.0148 0.00614 0.00259 0.0268
2R:2901800-2901950:minus -22 -0.366972 0.688073 4.31193 -17.9796 0.0510204 -26.5138 2.55556
-0.440367 0.00291 0.0117 0.0092 0.00516 0.00726 0.000349 0.057 0.00705 0.0216
2R:2901800-2901950:plus -40.2143 -0.733945 -1.24771 2.12844 -18.6429 -18.051 -16.5505 5.97222
7.04587 0.0372 0.0132 0.0172 0.0134 0.0121 0.00886 0.0119 0.00168 0.00132
2R:2902180-2902330:minus -29.4082 -0.431193 7.61468 2.63303 -27.1531 -9.42857 0.110092 0.333333
3.51376 0.0084 0.0119 0.000536 0.0107 0.0249 0.0027 0.000276 0.0151 0.00566
2R:2902180-2902330:plus -33.1122 -0.779817 0.853211 9.92661 0.540816 -0.244898 -19.1927 -2.15278
7.24771 0.0271 0.0134 0.0087 0.000169 0.000321 0.000496 0.018 0.0311 0.00116
2R:2903000-2903150:minus -32.8163 -2.25688 4.18349 9.33028 -27.2347 -17.2755 -20.0826 2.26389 1.55046
0.024 0.0209 0.00249 0.000374 0.0283 0.00544 0.0209 0.00784 0.0115
2R:2903000-2903150:plus -3.30612 4.10092 0.0642202 5.93578 -18.449 -16.9796 -2.40367 -0.0277778
-0.495413 0.0000553 0.00231 0.0113 0.00264 0.0107 0.0044 0.000489 0.0169 0.022
2R:2912480-2912630:minus -22.9592 4.27523 8.11927 -1.3945 -18.4184 -25.5612 -17.6789 -0.152778
-1.3578 0.00475 0.00217 0.000411 0.0419 0.0101 0.0206 0.0142 0.0175 0.0286
2R:2912480-2912630:plus -23 3.51376 5.3211 7 -8.44898 0.0204082 -11.0459 -2 5.44954 0.00498
0.00288 0.00155 0.00132 0.00109 0.000377 0.00377 0.0298 0.00253
2R:2937140-2937290:minus -30.4388 3.64286 7.07339 8.6055 -18.1531 0.346939 -16.3761 0.902778 -1.31193
0.00923 0.00159 0.000703 0.000567 0.00829 0.000221 0.0116 0.0125 0.0284
2R:2937140-2937290:plus -21.7755 -2.27523 -1.09174 9.7156 -26.5306 -27.8571 -5.88073 -1.75 12.2752
0.00241 0.021 0.0164 0.000242 0.0185 0.0508 0.00127 0.0279 0.0000141
2R:2949340-2949490:minus -20.8878 -1.13761 5.72477 5.22936 -27.4898 -26.8265 -11.5688 -1.58333 4.92661
0.0018 0.015 0.0013 0.0041 0.0336 0.0285 0.0042 0.0266 0.00318
2R:2949340-2949490:plus -32.4388 -0.669725 0.302752 4.37615 -18.7143 -8.23469 -20.7706 5.01389
-4.61468 0.0195 0.0129 0.0105 0.00504 0.0136 0.000875 0.0236 0.00261 0.0658
2R:3074540-3074690:minus -23.1531 0.944954 2.11009 6.45872 0.316327 -27.4898 -18.3211 11.0972 -1.43119
0.00542 0.00739 0.00554 0.00182 0.000341 0.0363 0.0157 0.0000703 0.029
2R:3074540-3074690:plus -2.37755 0.788991 5.91743 10.5872 -8.60204 -27.3367 -2.37615 -0.722222
5.44037 0.0000305 0.00781 0.0012 0.000115 0.00125 0.0347 0.000486 0.0208 0.00259
2R:3130260-3130410:minus -40.949 -0.137615 5.85321 9.23853 -27.2653 -18.8571 -15.6422 4.54167
7.68807 0.0445 0.0108 0.00123 0.000424 0.0302 0.0151 0.0102 0.0032 0.000841
2R:3130260-3130410:plus -23.9184 6.6055 9.78899 3.43119 -17.4898 -26.8265 -11.0734 7.38889 1.43119
0.00695 0.000793 0.000161 0.00749 0.0053 0.0285 0.00379 0.000819 0.0121
2R:3130740-3130890:minus -32.7653 4.81651 2.2844 1.97248 -8.93878 -18.1224 -12.4128 4.15278 7.63303
0.0228 0.00175 0.0052 0.0146 0.00216 0.00988 0.00506 0.00377 0.000928
2R:3130740-3130890:plus -32.3367 3.85321 10.4862 1.43119 -18.7551 -8.65306 -19.2018 5.06944 4.92661
0.0187 0.00254 0.000106 0.0181 0.0144 0.00156 0.018 0.00255 0.00318
2R:3133260-3133410:minus -32.8163 4.25688 1.29358 4.58716 -27.1939 -9.27551 -21.3394 5.19444 1.05505
0.024 0.00218 0.00746 0.00479 0.0267 0.00244 0.0262 0.00241 0.014
2R:3133260-3133410:plus -21.3571 7.19266 5.51376 4.6422 -18.1531 -36.1122 1.70642 1.88889 9.61468
0.00194 0.000587 0.00143 0.00473 0.00829 0.0878 0.00018 0.00896 0.00029
2R:3136600-3136750:minus -31.7755 -2.16514 5.11009 6.21101 -17.4082 -9.37755 -23.1468 -4.61111 -0.559633
0.0157 0.0204 0.0017 0.00213 0.00421 0.00265 0.0359 0.0566 0.0226
2R:3136600-3136750:plus -4.96939 1.9633 -1.89908 1.84404 -8.44898 -27.6735 -4.38532 12.7778 11.8073
0.000218 0.00509 0.021 0.0154 0.00109 0.0443 0.000832 0.0000134 0.0000538
2R:3137760-3137910:minus -32.9694 0.990826 1.14679 6.83486 -27.2653 0.94898 -15.4128 4.97222 5.18349
0.0254 0.00726 0.00785 0.00142 0.0302 0.00014 0.00973 0.00266 0.00295
2R:3137760-3137910:plus -40.551 1.14679 -0.146789 3.13761 -27.7551 -18.2755 -14.6697 6.41667
9.56881 0.0418 0.00686 0.0122 0.00854 0.0385 0.0112 0.00838 0.00135 0.000303
2R:3165780-3165930:minus -32.6939 -0.357798 1.6789 5.58716 -18.1122 -18.4184 -12.4128 0.125
4.92661 0.0217 0.0116 0.0065 0.00334 0.00771 0.0127 0.00506 0.0161 0.00318
2R:3165780-3165930:plus -23.6224 1.59633 6.40367 3.78899 -18.7551 -27.602 -15.8624 5 -0.40367
0.00658 0.00582 0.000962 0.00632 0.0144 0.0416 0.0106 0.00263 0.0213
2R:3257500-3257650:minus -41.8571 5.74312 -1.42202 1.44037 -9.45918 -9.79592 -17.5505 3.76389

-0.0733945 0.0667 0.00119 0.0182 0.018 0.00287 0.00377 0.014 0.00442 0.0188
2R:3257500-3257650:plus -22.2959 -1.55046 5.91743 4.79817 -27.6429 -18.3469 -10.6239 1.97222 7.11009
0.00335 0.017 0.0012 0.00453 0.037 0.0124 0.00347 0.0087 0.00128
2R:3258180-3258330:minus -31.9694 0.486239 0.53211 6.22936 -18.4898 -27.1224 -10.5505 0.305556 -4.43119
0.0168 0.00871 0.00971 0.00209 0.0116 0.0325 0.00342 0.0152 0.0627
2R:3258180-3258330:plus -12.9184 0.449541 0.33945 0.229358 -17.4184 -18.051 -14.1193 4.18056 2.36697
0.000532 0.00882 0.0104 0.0252 0.00457 0.00886 0.00745 0.00373 0.00828
2R:3262900-3263050:minus -24.3367 -0.0550459 0.155963 5.86239 -17.7857 -18.0102 -12.3394 1.63889
3.42202 0.00789 0.0105 0.011 0.00282 0.00676 0.00812 0.00497 0.00978 0.00597
2R:3262900-3263050:plus -22 2 1.43119 7.27523 -17.4184 -19.3061 -9.3211 4 4.04587 0.00291
0.00502 0.0071 0.00109 0.00457 0.0187 0.00271 0.00402 0.00421
2R:3263640-3263790:minus -32.5816 2.75229 1.72477 5.31193 -27.602 -8.60204 -9.92661 6.44444 3.46789
0.0208 0.00381 0.0064 0.00393 0.0364 0.00151 0.00304 0.00133 0.00582
2R:3263640-3263790:plus -30.7449 1.79817 0.954128 1.74312 -7.89796 -19.0816 -19.5872 7.65278 3.66972
0.0101 0.00541 0.0084 0.016 0.000678 0.017 0.0192 0.000709 0.00514
2R:3264120-3264270:minus -30.7449 -1.81651 2.16514 9.7156 -7.97959 -17.3469 -16.0275 -2.41667 11.8991
0.0101 0.0184 0.00544 0.000242 0.000799 0.0058 0.0109 0.0333 0.0000255
2R:3264120-3264270:plus -24.551 2.31193 0.715596 3.26606 -18.1837 -26.602 -9.78899 3.95833 3.20183
0.00814 0.00448 0.00912 0.00802 0.00835 0.0248 0.00296 0.00409 0.0065
2R:3265940-3266090:minus -14.2959 -1.43119 5.66055 2.66972 -18.1939 -17.051 -11.9358 3.43056 1.08257
0.00129 0.0164 0.00134 0.0106 0.0086 0.00479 0.00455 0.00505 0.0139
2R:3265940-3266090:plus -13.6735 4.11927 -0.0458716 3.6055 -27.3061 -8.65306 -15.7798 1.02778
7.6422 0.00103 0.0023 0.0118 0.00686 0.0315 0.00156 0.0104 0.012 0.000893
2R:3266900-3267050:minus -22.2959 3.54128 10.211 11.2936 -27.9388 -17.5612 -21.1009 -4.68056 5.6055
0.00335 0.00285 0.000126 0.0000139 0.0415 0.00604 0.025 0.0575 0.00232
2R:3266900-3267050:plus -31.5204 -2.36697 -0.394495 3.18349 -7.71429 -9.60204 -18.578 8.41667
4.40367 0.0131 0.0216 0.0132 0.00837 0.000572 0.00334 0.0163 0.000458 0.00373
2R:3269360-3269510:minus -20.8469 4.41284 0.853211 10.0275 -27.9286 0.0918367 -13.2844 -1.625
1.43119 0.00178 0.00205 0.0087 0.000156 0.0412 0.000323 0.00617 0.0269 0.0121
2R:3269360-3269510:plus -21.8878 4.57798 1.21101 4.81651 -18.1122 -27.2653 -7.6789 0.611111 -1.17431
0.00258 0.00193 0.00768 0.00451 0.00771 0.0333 0.00197 0.0138 0.0275
2R:3274780-3274930:minus -23.1224 13.5306 3.82569 4.37615 10.6224 9.86735 -20.2018 -3.31944 8.56881
0.00531 0.0000206 0.00288 0.00504 0.0000113 0.0000132 0.0213 0.0418 0.000491
2R:3274780-3274930:plus -29.4796 -0.504587 1.11927 4.10092 2.17347 9.60204 -8.66055 4.90278
7.15596 0.00844 0.0122 0.00793 0.00554 0.0000296 0.0000248 0.0024 0.00274 0.00124
2R:3283400-3283550:minus -30.6633 2.68807 8.12844 2.46789 11.6531 -8.27551 -20.0826 0.194444 7.62385
0.00976 0.0039 0.000408 0.0115 1.55e-06 0.000919 0.0209 0.0157 0.000941
2R:3283400-3283550:plus -24.0102 -4.23853 1.16514 2.12844 -18.449 -19.3469 -21.4679 2.94444 -1.06422
0.00719 0.0361 0.0078 0.0134 0.0107 0.0194 0.0268 0.00609 0.0266
2R:3294660-3294810:minus -32.4388 -0.238532 3.59633 5.59633 -17.7143 -0.244898 -16.7064 5.34722
-1.21101 0.0195 0.0112 0.00315 0.00331 0.00627 0.000496 0.0122 0.00225 0.0277
2R:3294660-3294810:plus -23.1531 -0.633028 6.30275 1.6789 -17.449 -17.9796 -19.0275 -0.652778
-4.52294 0.00542 0.0127 0.00101 0.0165 0.00484 0.00764 0.0175 0.0204 0.0642
2R:3295260-3295410:minus -22.0408 4.12844 7.44954 2.33028 -8.37755 1.05102 -7.40367 3.31944 7.68807
0.00296 0.00229 0.000581 0.0123 0.00106 0.000107 0.00185 0.00528 0.000841
2R:3295260-3295410:plus -22.0306 8.85321 -0.0550459 -0.192661 -18.1939 -26.8265 -11.7982 -0.236111
4.14679 0.00293 0.000241 0.0118 0.0284 0.0086 0.0285 0.00441 0.018 0.00406
2R:3345180-3345330:minus -22.5918 -0.59633 0.266055 1.61468 -8.86735 -26.7551 -15.9725 5.98611 0.908257
0.00365 0.0126 0.0106 0.0169 0.00173 0.0263 0.0108 0.00167 0.0147
2R:3345180-3345330:plus -30.449 8.34862 8.82569 7.55963 -18.9796 1.09184 -11.1651 2.72222 3.25688
0.00925 0.000315 0.000279 0.000807 0.0155 0.000091 0.00386 0.00663 0.00632
2R:3346300-3346450:minus -11.8469 -2.02752 3.11009 7.88991 -18.2245 -17.0102 -9.86239 12.6111 3.51376
0.000279 0.0196 0.00381 0.00062 0.00877 0.00453 0.003 0.0000163 0.00566
2R:3346300-3346450:plus -22.2653 2.91743 8.41284 4.12844 -26.8571 -8.23469 -16.789 -5.20833 3.49541

0.00331 0.00358 0.000351 0.00549 0.0198 0.000875 0.0124 0.0645 0.0057
2R:3350640-3350790:minus -13.1837 -0.100917 5.77064 6.25688 -27.1939 -8.16327 -10.7798 7.41667
2.85321 0.000705 0.0107 0.00128 0.00208 0.0267 0.000783 0.00357 0.000807 0.00733
2R:3350640-3350790:plus -41.8878 0.889908 1.79817 -0.311927 -17.449 -9.57143 -17.8991 3.625
-2.3578 0.0672 0.00753 0.00623 0.0294 0.00484 0.00326 0.0147 0.00468 0.0369
2R:3354240-3354390:minus -30.6735 -1.20183 1.88073 6.57798 20.2041 -16.9796 -21.422 -6.04167 -3.81651
0.00978 0.0153 0.00604 0.00167 3.64e-07 0.0044 0.0266 0.0767 0.0529
2R:3354240-3354390:plus -32 6.88991 8.48624 10.5963 -18.7143 -18.2755 -17.9725 10.4306 3.46789
0.0171 0.000688 0.000336 0.0001 0.0136 0.0112 0.0149 0.000119 0.00582
2R:3357820-3357970:minus -23.2245 2.27523 11.9083 7.29358 -16.1531 -26.3367 -22.7156 4.54167 0.293578
0.00576 0.00454 0.0000457 0.00104 0.00297 0.0221 0.0334 0.0032 0.017
2R:3357820-3357970:plus -30.6939 0.770642 5.75229 7.37615 -18.0408 -27.602 -12 -7.38889 -0.238532
0.00979 0.00786 0.00129 0.000951 0.00735 0.0416 0.00461 0.0995 0.0203
2R:3358440-3358590:minus -13.9592 -5.55046 7.34862 7.12844 -27.4898 -9.38776 -18.3211 1.02778 5.12844
0.00113 0.0509 0.000611 0.00118 0.0336 0.00266 0.0157 0.012 0.00301
2R:3358440-3358590:plus -22.9898 -0.0183486 3.13761 2.16514 -8.86735 -19.4184 -11.3761 -2.51389
-6.04587 0.00482 0.0104 0.00377 0.0132 0.00173 0.0198 0.00403 0.0341 0.0898
2R:3359560-3359710:minus -20.8163 2.07339 5.68807 6.74312 -8.7449 -18.3061 -13.8073 1.34722 -0.550459
0.00177 0.00488 0.00133 0.0015 0.00161 0.0114 0.00695 0.0108 0.0225
2R:3359560-3359710:plus -30.1837 6.47706 0.862385 8.05505 -17.8571 -16.7143 -15.1468 4.45833 -4.40367
0.00873 0.000843 0.00867 0.000578 0.00698 0.00419 0.00924 0.00332 0.0623
2R:3361140-3361290:minus -31.7347 -3.49541 4.52294 1.26606 -26.9286 -9.57143 -0.504587 1.36111
1.16514 0.0149 0.0295 0.00217 0.0191 0.0214 0.00326 0.000318 0.0108 0.0135
2R:3361140-3361290:plus -30.1429 -2.83486 1.53211 3.76147 -8.64286 -9.42857 -17.4404 -1.95833 -2.59633
0.00866 0.0246 0.00685 0.00645 0.00139 0.0027 0.0137 0.0295 0.0398
2R:3361640-3361790:minus -22.7755 -2.20183 -2.69725 5.55046 20.4694 -17.8571 -10.156 -0.666667
1.46789 0.00412 0.0206 0.0265 0.00345 1.65e-07 0.00719 0.00317 0.0205 0.0119
2R:3361640-3361790:plus -32.7449 5.6422 -1.73394 3.36697 -27.5306 -17.3469 -23.2661 5.76389 5.75229
0.0228 0.00124 0.02 0.00764 0.0351 0.0058 0.0366 0.00185 0.00213
2R:3376300-3376450:minus -10.9592 0.238532 -1.37615 11.2936 -18.7143 -17.2755 -9.6055 -2.27778 7.53211
0.00024 0.0095 0.0179 0.0000139 0.0136 0.00544 0.00286 0.0321 0.000968
2R:3376300-3376450:plus -29.8776 0.550459 2.94495 1.56881 -17.0408 -8.42857 -6.97248 6.47222 -0.412844
0.00852 0.00851 0.00405 0.0172 0.00329 0.00104 0.00168 0.00132 0.0213
2R:3377340-3377490:minus -21.4796 -3.92661 0.963303 6.21101 -27.2755 -18.2755 -11.6239 6.18056 5.80734
0.00202 0.0332 0.00837 0.00213 0.0302 0.0112 0.00425 0.00152 0.00205
2R:3377340-3377490:plus -31.3265 0.12844 0.119266 2.25688 20.2041 -9.72449 -17.6147 -2.31944 -3.85321
0.0114 0.00987 0.0111 0.0127 3.64e-07 0.00352 0.0141 0.0325 0.0534
2R:3377980-3378130:minus -33.2959 6.09174 -0.238532 3.41284 -17.4592 -17.7857 -15.1651 1 2.16514
0.0279 0.00101 0.0125 0.00756 0.00491 0.0068 0.00927 0.0121 0.00879
2R:3377980-3378130:plus -22.102 -3.88991 1.52294 -0.568807 -27.1939 -9.42857 -16.6789 2.45833
6.33028 0.003 0.0328 0.00687 0.032 0.0267 0.0027 0.0122 0.00731 0.0016
2R:3378380-3378530:minus -33 -0.522936 -3.88073 4.14679 -8.63265 -17.8571 -23.7982 3.27778 -0.642202
0.0258 0.0123 0.0368 0.00542 0.0013 0.00719 0.0398 0.00536 0.0234
2R:3378380-3378530:plus -22.8163 -0.486239 -0.0825688 11.0183 -18.9796 -17.7857 -22.8073 2.81944
-1.55046 0.00418 0.0121 0.0119 0.000052 0.0155 0.0068 0.034 0.00639 0.0297
2R:3379920-3380070:minus -23.9592 -1.23853 1.10092 2.93578 -18.6837 1.31633 -12.7339 -3.72222 -0.155963
0.00708 0.0154 0.00798 0.00927 0.0126 0.000066 0.00544 0.0461 0.0195
2R:3379920-3380070:plus -31.7347 -2.53211 9.20183 2.08257 -18.1531 -19.0816 -7.25688 -0.0833333
3.05505 0.0149 0.0226 0.000225 0.0137 0.00829 0.017 0.00179 0.0172 0.00692
2R:3380580-3380730:minus -21.8571 1.15596 -0.0917431 2.55046 -8.64286 -17.2755 -25.9266 6.19444
-4.68807 0.00249 0.00684 0.0119 0.0112 0.00139 0.00544 0.053 0.00151 0.0672
2R:3380580-3380730:plus -29.4082 -2.7156 2.6422 6.72477 -27.1939 -7.45918 -1.17431 0.902778 1.6789
0.0084 0.0238 0.00455 0.00154 0.0267 0.000582 0.000369 0.0125 0.011
2R:3383780-3383930:minus -31.7449 0.293578 3.59633 2.0367 1.14286 -17.3469 -9.31193 -0.875 0.816514

0.0152	0.00932	0.00315	0.0141	0.000113	0.0058	0.00271	0.0218	0.015
2R:3383780-3383930:plus	-31.102	2.17431	0.146789	2.53211	-26.9694	-9.45918	-23.5321	7.16667	-1.33945
0.0106	0.00471	0.011	0.0113	0.023	0.00279	0.0382	0.000922	0.0285
2R:3387420-3387670:minus	-23.3776	2.27523	2.11927	2.07339	-8.33673	-17.9388	-8.3945	3	-0.431193
0.00619	0.00454	0.00553	0.0138	0.00101	0.00738	0.00228	0.00597	0.0214
2R:3387420-3387670:plus	-40.4694	-5.57798	0.0458716	9.23853	-8.12245	-7.42857	-19.1927	-0.0416667
3.97248	0.0402	0.0513	0.0114	0.000424	0.000843	0.000567	0.018	0.017	0.00444
2R:3397820-3397970:minus	-11.8878	-3.04587	16.2202	6.14679	-26.9694	-8.42857	-19.0275	-4.375	-5.76147
0.00029	0.0261	1.24e-07	0.00224	0.023	0.00104	0.0175	0.0537	0.0844
2R:3397820-3397970:plus	-33.102	-1.21101	6.13761	1.68807	-18.4898	-19.3469	-15.0734	8.40278	-0.880734
0.0268	0.0153	0.00109	0.0164	0.0116	0.0194	0.00911	0.000462	0.0252
2R:3400200-3400350:minus	-32.7449	13.4592	-2.84404	0.642202	-17.4898	-9.5	-21.578	9.20833	-0.651376
0.0228	0.000064	0.0276	0.0227	0.0053	0.00294	0.0273	0.00028	0.0235
2R:3400200-3400350:plus	-24.2551	13.4592	0.93578	4.11009	-17.6837	-16.7857	-14.1101	2.41667	1.38532
0.00768	0.000064	0.00845	0.00553	0.0058	0.00426	0.00743	0.00742	0.0123
2R:3413160-3413310:minus	-21.398	-1.49541	7.0367	3.18349	-18.1939	-27.0408	-3.51376	2.30556	3.6789
0.00197	0.0167	0.000715	0.00837	0.0086	0.0307	0.000652	0.00772	0.00506
2R:3413160-3413310:plus	-30.7755	-1.31193	9.84404	9.88073	-17.6429	-19.1224	-2.68807	8.48611	5.9633
0.0102	0.0158	0.000156	0.000183	0.00562	0.0175	0.000525	0.00044	0.00188
2R:3416720-3416870:minus	-22.7347	2.10092	9.47706	6.59633	-18.4592	-17.7143	3	2.06944	5.66055
0.00403	0.00484	0.000193	0.00164	0.0109	0.00634	0.000118	0.00841	0.00227
2R:3416720-3416870:plus	-33.2245	2.90826	-1.76147	9.49541	-27.1531	-17.8265	-21.1376	5.94444	1.61468
0.0276	0.0036	0.0201	0.000308	0.0249	0.00699	0.0252	0.0017	0.0112
2R:3417440-3417590:minus	-24.6327	1.43119	-1.44037	1.6422	-29.0918	-16.2755	-18.1835	3.54167	1.48624
0.00824	0.00619	0.0183	0.0166	0.0653	0.00413	0.0154	0.00483	0.0118
2R:3417440-3417590:plus	-40.8061	1.18349	3.17431	1.30275	-17.7143	-16.5	-13.3486	0.430556	8.10092
0.0434	0.00677	0.00371	0.0189	0.00627	0.00416	0.00626	0.0146	0.000623
2R:3417780-3417930:minus	-23.1531	7.3211	6.56881	2.6422	-18.1939	-26.898	-0.119266	6.73611
0.614679	0.00542	0.000548	0.000888	0.0107	0.0086	0.0302	0.000291	0.00115	0.0158
2R:3417780-3417930:plus	-22.8878	1.25688	3.41284	5.74312	-8.20408	-19.0816	-16.5963	-1.63889	4.88073
0.00441	0.00659	0.00338	0.00302	0.000894	0.017	0.012	0.027	0.00324
2R:3418360-3418510:minus	-40.1735	3.18349	2.9633	9.33028	-18.5204	-17.0102	-17.9817	-4.34722	-3.20183
0.0363	0.00325	0.00403	0.000374	0.0117	0.00453	0.0149	0.0533	0.0467
2R:3418360-3418510:plus	-40.2857	-4.04587	1.31193	0.944954	-17.4184	-9.42857	-16.5688	10.3472	-4.15596
0.0385	0.0342	0.00741	0.0209	0.00457	0.0027	0.012	0.000127	0.0578
2R:3421000-3421150:minus	-22.5612	14.6055	2.27523	2.61468	20.4694	-17.7551	-12.8532	-3.86111	-0.275229
0.0036	4.26e-06	0.00522	0.0109	1.65e-07	0.00643	0.00559	0.0477	0.0206
2R:3421000-3421150:plus	-23.7755	1.44954	4.48624	5.3945	-8.86735	-27.1224	-3.77064	1.27778	7.83486
0.00678	0.00615	0.0022	0.00379	0.00173	0.0325	0.000699	0.0111	0.000712
2R:3421220-3421370:minus	-4.29592	-2.24771	8.57798	7.45872	-7.60204	-26.4898	-6.04587	-5.94444	9.3945
0.000135	0.0209	0.000318	0.000894	0.000466	0.0225	0.00133	0.0752	0.000358
2R:3421220-3421370:plus	-14.3367	5.33945	0.513761	3.33945	-8.63265	-19.2755	-17.0917	1.44444	-0.559633
0.00131	0.00142	0.00977	0.00774	0.0013	0.0185	0.013	0.0105	0.0226
2R:3427120-3427270:minus	-32.7347	-0.00917431	3.92661	2.44037	10.9898	-18.5714	-21.0459	3.72222
-2.89908	0.0225	0.0103	0.00276	0.0116	3.28e-06	0.0136	0.0248	0.0045	0.0434
2R:3427120-3427270:plus	-31.1735	-4.0367	3.62385	3.3578	-26.9286	-28.2653	-6.86239	6.79167	6.95413
0.0109	0.0342	0.00312	0.00767	0.0214	0.0595	0.00163	0.00112	0.00136
2R:3453080-3453230:minus	-14.7755	2.23853	4.00917	3.11009	-27.5306	-17.5	-20.4495	-1.36111	1.59633
0.00157	0.0046	0.00267	0.00862	0.0351	0.00599	0.0223	0.025	0.0113
2R:3453080-3453230:plus	-13.8163	5.88991	-1.40367	0.981651	-18.0816	-9.02041	3.81651	3.75	0.440367
0.00109	0.00111	0.0181	0.0207	0.00759	0.00213	0.0000882	0.00445	0.0164
2R:3454240-3454390:minus	-23.5612	-3.47706	15.5596	8.05505	-17.0816	-17.7143	-1.19266	-4.25	2.74312
0.00653	0.0294	6.69e-07	0.000578	0.00334	0.00634	0.00037	0.0522	0.00751
2R:3454240-3454390:plus	-21.8878	-3.91743	15.5963	1.41284	-18.4898	-0.244898	-4.04587	6.84722

4.98165 0.00258 0.0331 6.15e-07 0.0183 0.0116 0.000496 0.000756 0.00109 0.00313
2R:3474580-3474730:minus -20.9592 -4.3945 3.41284 5.42202 -18.4184 -27.0408 -8.04587 -1.95833 6.73394
0.00184 0.0376 0.00338 0.00374 0.0101 0.0307 0.00212 0.0295 0.00143
2R:3474580-3474730:plus -31 0.550459 -0.752294 6.12844 -27.3061 -19.0816 -18.9358 3.27778 -0.486239
0.0105 0.00851 0.0148 0.00231 0.0315 0.017 0.0173 0.00536 0.0219
2R:3477580-3477730:minus -22.5204 3.31193 2.63303 4.75229 -8.97959 -9.72449 -21.8716 3.20833 1.11927
0.00356 0.0031 0.00457 0.00461 0.0023 0.00352 0.0288 0.00551 0.0137
2R:3477580-3477730:plus -14.0306 -2.38532 3.58716 3.18349 -27.0102 -9.72449 -2.3578 -2.01389 3.6789
0.00115 0.0217 0.00316 0.00837 0.0237 0.00352 0.000484 0.0299 0.00506
2R:3495200-3495350:minus -12.4082 0.0825688 3.18349 2.30275 -17.9286 -16.7143 -11.4404 -3.79167
2.56881 0.000414 0.01 0.0037 0.0124 0.0071 0.00419 0.00409 0.0469 0.00783
2R:3495200-3495350:plus -30.6735 4.30275 4.11009 -1.05505 -8.7449 -9.45918 -20.1101 1.73611 5.6055
0.00978 0.00214 0.00256 0.0374 0.00161 0.00279 0.021 0.00946 0.00232
2R:3501800-3501950:minus -31.2551 -2.01835 -1.06422 5.58716 -19.051 -28.2653 -10.7156 0.111111
-0.0458716 0.0113 0.0195 0.0163 0.00334 0.0162 0.0595 0.00353 0.0162 0.0187
2R:3501800-3501950:plus -30.9184 -0.302752 -1.61468 5.59633 -8.11224 -27.602 -3.30275 4.05556
11.4771 0.0103 0.0114 0.0193 0.00331 0.000838 0.0416 0.000615 0.00393 0.0000679
2R:3503480-3503630:minus -43.1122 -2.74312 -5.42202 1.41284 -27.0408 0.683673 -22.6606 1.41667 4.19266
0.0897 0.024 0.0549 0.0183 0.0242 0.000184 0.0331 0.0106 0.00401
2R:3503480-3503630:plus -23.1429 -0.678899 2.9633 9.33028 -17.449 -7.57143 -12.4679 1.29167
-2.47706 0.00533 0.0129 0.00403 0.000374 0.00484 0.000644 0.00512 0.011 0.0385
2R:3510580-3510730:minus -12.2653 0.0733945 7.38532 6.72477 -18.449 -9.16327 -18.4771 -1.69444
0.779817 0.000381 0.0101 0.0006 0.00154 0.0107 0.00223 0.0161 0.0274 0.0151
2R:3510580-3510730:plus 14.8878 2.45872 -2.02752 7.17431 -18.4898 -28.7857 -3.52294 -0.791667
1.01835 7.48e-07 0.00424 0.0218 0.00115 0.0116 0.0679 0.000653 0.0212 0.0142
2R:3511080-3511230:minus -21.9184 0.46789 3.66055 1.31193 -8.60204 -18.1224 -10.422 3.38889 -7.48624
0.00261 0.00876 0.00307 0.0189 0.00125 0.00988 0.00333 0.00513 0.123
2R:3511080-3511230:plus -13.1429 1.13761 7.93578 0.321101 -18.5204 -37.1122 0.165138 1.34722 2.88073
0.00065 0.00689 0.000453 0.0247 0.0117 0.133 0.000272 0.0108 0.00727
2R:3513900-3514050:minus -23.3673 -1.88991 2.22018 6.22936 -17.3776 -9.82653 -22 1.73611 -0.0733945
0.00617 0.0188 0.00532 0.00209 0.00419 0.00381 0.0295 0.00946 0.0188
2R:3513900-3514050:plus -21.9286 2.3211 -3.19266 3.23853 -18.1531 -28.051 -19.945 -2.31944 2.73394
0.0027 0.00446 0.0304 0.00815 0.00829 0.0563 0.0204 0.0325 0.00756
2R:3520140-3520290:minus -13.4388 -0.46789 2.44954 5.2844 -8.60204 -27.6735 1.04587 -1.13889 1.95413
0.000879 0.0121 0.00489 0.004 0.00125 0.0443 0.000218 0.0235 0.00964
2R:3520140-3520290:plus -23 -4.75229 13.0092 7.45872 -26.9286 -17.7857 -3.81651 3.75 2.33028 0.00498
0.0413 0.0000207 0.000894 0.0214 0.0068 0.000708 0.00445 0.00836
2R:3541480-3541630:minus -12.9184 3.3945 -0.688073 9.51376 -17.3776 -9.45918 -13.0092 0.555556
3.46789 0.000532 0.00301 0.0145 0.000292 0.00419 0.00279 0.00579 0.014 0.00582
2R:3541480-3541630:plus -30.7041 4.97248 -0.577982 1.89908 -26.5306 -8.57143 -24.7615 0.902778
-0.477064 0.00993 0.00165 0.014 0.0151 0.0185 0.00149 0.0457 0.0125 0.0218
2R:3542740-3542890:minus -22.1122 -1.44037 5.54128 -1.33028 -26.2653 -9.65306 -17.0275 -1.09722 0.642202
0.00304 0.0164 0.00141 0.0409 0.018 0.0034 0.0129 0.0232 0.0157
2R:3542740-3542890:plus -22 15.2202 14.2477 4.33945 -27.0408 1.7551 -20.3853 -0.375 2.04587
0.00291 1.59e-06 5.31e-06 0.00509 0.0242 0.0000519 0.022 0.0188 0.00919
2R:3551160-3551310:minus -23.0306 -2.95413 2.76147 -0.541284 -28.2653 -18.9796 -4.19266 9.19444
1.73394 0.00505 0.0255 0.00435 0.0316 0.0537 0.0155 0.000788 0.000283 0.0106
2R:3551160-3551310:plus -13.6327 0.954128 -1.93578 6.05505 -17.2245 -17.3469 -1.88991 13.0417 2.11009
0.00101 0.00736 0.0212 0.0024 0.00384 0.0058 0.000433 9.9e-06 0.00898
2R:3552700-3552850:minus -31.7041 2.16514 3.93578 6.29358 -18.7143 -8.68367 -29.5963 -0.694444
4.56881 0.0146 0.00473 0.00275 0.00202 0.0136 0.00157 0.0859 0.0206 0.00356
2R:3552700-3552850:plus -23.1837 1.2844 1.29358 8.81651 -8.67347 -8.7551 -10.7156 2.61111 -0.926606
0.00553 0.00653 0.00746 0.000501 0.00154 0.00172 0.00353 0.00691 0.0256
2R:3553200-3553350:minus -31.4796 2.77064 -2.56881 2.47706 -18.7449 -7.57143 -20.3761 6.33333 5.34862

0.0128 0.00378 0.0255 0.0115 0.0138 0.000644 0.022 0.00141 0.00271
2R:3553200-3553350:plus -31.4898 9.6055 13.6422 7 -18.7143 -9.86735 -16.8624 5 2.42202 0.0128
0.000164 0.0000116 0.00132 0.0136 0.00391 0.0125 0.00263 0.00816
2R:3556440-3556590:minus -42.6633 14.4128 13.8257 3.44037 -26.3061 -17.3469 -17.6422 5.83333 7.73394
0.0837 5.49e-06 8.99e-06 0.00746 0.0181 0.0058 0.0142 0.00179 0.000784
2R:3556440-3556590:plus -33.7755 3.97959 0.302752 2.18349 -17.1939 0.755102 -23.3945 1.76389 0.93578
0.0297 0.000669 0.0105 0.0131 0.00377 0.000175 0.0374 0.00937 0.0145
2R:3561860-3562010:minus -30.4388 -3.25688 14.7248 7.2844 -8.93878 -9.5 -21.8165 -3.18056 -6.05505
0.00923 0.0277 2.82e-06 0.00105 0.00216 0.00294 0.0285 0.0404 0.09
2R:3561860-3562010:plus -21.6224 3.90816 4.25688 6.74312 -28.3061 -9.42857 -17.8257 -0.402778
4.09174 0.00213 0.00102 0.00242 0.0015 0.0553 0.0027 0.0146 0.0189 0.00416
2R:3563860-3564010:minus -22.6633 15.4037 5.11009 2.31193 -18.7551 -18.0102 -18.5321 -4.48611 3.46789
0.00384 1.16e-06 0.0017 0.0123 0.0144 0.00812 0.0162 0.055 0.00582
2R:3563860-3564010:plus -40.2857 5.21101 -0.46789 1.88073 -18.0102 -27.602 -23.0459 4.19444 1.63303
0.0385 0.00149 0.0135 0.0152 0.00732 0.0416 0.0353 0.00371 0.0112
2R:3569460-3569610:minus -31.7347 -1.02752 11.6697 2.19266 -18.3776 -26.602 11.8624 -2.36111 -0.59633
0.0149 0.0145 0.0000536 0.0131 0.00926 0.0248 6.01e-06 0.0328 0.0229
2R:3569460-3569610:plus -40.4796 0.651376 -0.119266 7.99083 -27.2347 -8.79592 -22.4587 2.98611
-4.84404 0.0407 0.00821 0.0121 0.00059 0.0283 0.0019 0.032 0.006 0.0701
2R:3575800-3575950:minus -23.2959 7.20183 -2.22018 2.77064 -8.60204 -18.2755 -22.6697 2.65278 7.16514
0.00605 0.000584 0.0231 0.01 0.00125 0.0112 0.0332 0.0068 0.00122
2R:3575800-3575950:plus -39.2551 -3.01835 -1.01835 5.93578 -17.7143 -27.3367 -1.34862 6.56944 2.88991
0.0331 0.0259 0.0161 0.00264 0.00627 0.0347 0.000383 0.00125 0.00725
2R:3576460-3576610:minus -33.2245 3.68367 -2.04587 6.84404 -18.4184 -17.5714 -22.6881 3.05556 -1.29358
0.0276 0.00144 0.0219 0.0014 0.0101 0.00616 0.0333 0.00584 0.0283
2R:3576460-3576610:plus -22.2959 -4.63303 0.348624 5.15596 -17.7551 -18.4184 -7.01835 1.75 0.761468
0.00335 0.04 0.0103 0.00426 0.00658 0.0127 0.00169 0.00941 0.0152
2R:3579040-3579190:minus -31.7449 -3.05505 4.88991 4.17431 -18.7959 -8.79592 -19.6881 -0.958333
-0.275229 0.0152 0.0262 0.00186 0.00537 0.0148 0.0019 0.0195 0.0223 0.0206
2R:3579040-3579190:plus -3.96939 1.3945 -0.495413 6.44037 -17.4184 -8.42857 -15.9633 0.513889
-0.440367 0.000119 0.00627 0.0136 0.00185 0.00457 0.00104 0.0108 0.0142 0.0216
2R:3581680-3581830:minus -22.8878 0.477064 2.56881 1.90826 -26.9694 -27.1939 -9.63303 7.97222 5.9633
0.00441 0.00873 0.00468 0.015 0.023 0.0327 0.00288 0.000593 0.00188
2R:3581680-3581830:plus -13.5204 3.97248 -0.247706 7.90826 -18.1531 -18.051 -7.84404 1.125
0.357798 0.00097 0.00243 0.0126 0.000611 0.00829 0.00886 0.00204 0.0117 0.0166
2R:358320-358470:minus -32.3367 3.83486 0.862385 7.34862 -18.8265 -17.0816 -11.4128 5.08333 3.66972
0.0187 0.00256 0.00867 0.000998 0.0149 0.00487 0.00406 0.00253 0.00514
2R:358320-358470:plus -32.2245 3.97248 0.513761 2.41284 -18.7143 -17.5714 -10.9083 -0.583333 -0.192661
0.0183 0.00243 0.00977 0.0118 0.0136 0.00616 0.00366 0.02 0.0198
2R:358540-358690:minus -42.2143 -0.623853 -1.45872 0.715596 20.1735 -8.02041 -15.4954 6.90278
-1.52294 0.0712 0.0127 0.0184 0.0223 5.68e-07 0.000761 0.00989 0.00106 0.0295
2R:358540-358690:plus -32.0408 0.477064 1.57798 7.10092 -17.7143 -9.53061 -9.63303 3.18056 2.6422
0.0174 0.00873 0.00674 0.00121 0.00627 0.0031 0.00288 0.00557 0.00768
2R:3586560-3586710:minus -32.5816 -0.412844 7.0367 5.81651 20.2041 -19.1224 -16.8991 2.23611
-0.302752 0.0208 0.0119 0.000715 0.00292 3.64e-07 0.0175 0.0126 0.00792 0.0208
2R:3586560-3586710:plus -22 4.20183 0.559633 7.27523 -27.1939 -18.9388 -13.8991 6.13889 -2.88991
0.00291 0.00223 0.00961 0.00109 0.0267 0.0154 0.00709 0.00155 0.0433
2R:358980-359130:minus -32.5612 2.31193 -0.963303 5.37615 -17.4898 -18.3469 -14.8073 5.44444
-2.36697 0.0207 0.00448 0.0158 0.00384 0.0053 0.0124 0.00863 0.00215 0.0371
2R:358980-359130:plus -32.7041 -1.14679 -0.0642202 5.22018 -18.6429 -7.5 -24.5413 5.68056 -0.137615
0.0222 0.015 0.0118 0.00418 0.0121 0.000614 0.0443 0.00193 0.0194
2R:3610160-3610310:minus -20.2143 1.51376 -0.146789 7.17431 -17.1122 -27.898 6.12844
-0.513889 5.70642 0.00165 0.006 0.0122 0.00115 0.00341 0.0537 0.0000398 0.0196 0.00222
2R:3610160-3610310:plus -14.6327 1.3945 0.321101 0.59633 -16.7143 -28.7857 -19.6422

-3.79167 12.2752 0.0015 0.00627 0.0104 0.023 0.00319 0.0679 0.0194 0.0469 0.0000141
2R:3610520-3610670:minus -32.3265 -1.86239 0.165138 -0.211009 -17.7143 -17.9796
-12.3211 0.513889 12.2752 0.0187 0.0187 0.011 0.0286 0.00627 0.00764 0.00495 0.0142 0.0000141
2R:3610520-3610670:plus -30.7755 -0.155963 1.36697 8.81651 -18.7857 -26.5306 -16.156 -0.458333
9.88073 0.0102 0.0109 0.00726 0.000501 0.0148 0.023 0.0111 0.0192 0.000206
2R:3622340-3622490:minus -5.19388 -3.13761 -4.06422 1.83486 -18.7959 -17.2041
-15.7248 5.875 11.8991 0.000229 0.0268 0.0386 0.0155 0.0148 0.00507 0.0103 0.00176 0.0000255
2R:3622340-3622490:plus -33.6939 1.37615 4.77982 2.23853 -8.96939 -18.1633 -12.0917 -0.402778
1.38532 0.0291 0.00631 0.00195 0.0128 0.00226 0.01 0.00471 0.0189 0.0123
2R:3623040-3623190:minus -21.7755 6.21101 2.88991 7.6422 -27.1939 -18.0102 -8.53211
6.375 7.68807 0.00241 0.000958 0.00414 0.000721 0.0267 0.00812 0.00234 0.00138 0.000841
2R:3623040-3623190:plus -33.602 -2.69725 0.247706 0.642202 -26.2653 -0.204082 -11.4679 2.33333
-0.284404 0.0289 0.0237 0.0107 0.0227 0.018 0.000463 0.00411 0.00765 0.0207
2R:3632820-3632970:minus -21.5102 0.807339 3.75229 6.84404 -18.1837 -0.173469 -5.11009 -3.45833
1.52294 0.00202 0.00776 0.00296 0.0014 0.00835 0.000456 0.00102 0.0433 0.0117
2R:3632820-3632970:plus -32.2653 -1.10092 1.00917 7.77982 -7.85714 -9.0102 -9.17431 -2.91667 -4.57798
0.0184 0.0148 0.00824 0.000652 0.000594 0.00208 0.00264 0.0378 0.0651
2R:3639060-3639210:minus -34.4082 1.66055 -3.3578 4.22018 -27.2347 -8.72449 -23.1927 1.98611 -7.24771
0.0319 0.00569 0.0319 0.00531 0.0283 0.00169 0.0362 0.00866 0.119
2R:3639060-3639210:plus -23.1531 -1.78899 6.24771 3.27523 -27.4082 -18.1224 -17.0642 -2.08333 1.56881
0.00542 0.0183 0.00103 0.00796 0.0321 0.00988 0.0129 0.0305 0.0114
2R:3645040-3645190:minus -31.8163 3.81651 1.56881 5.21101 -17.7143 -17.4184 -23.5963 0.305556 8.11009
0.016 0.00258 0.00676 0.0042 0.00627 0.00585 0.0386 0.0152 0.000597
2R:3645040-3645190:plus -22.0408 -0.733945 9.29358 2.66972 -17.9388 -17.2755 -11.7523 2.55556
10.1284 0.00296 0.0132 0.000214 0.0106 0.00715 0.00544 0.00437 0.00705 0.000129
2R:3646540-3646690:minus -12.8163 -1.80734 3.12844 11.0275 -27.4592 -26.602 -17.7339 -1.61111 3.44954
0.000489 0.0184 0.00378 0.0000401 0.0329 0.0248 0.0144 0.0268 0.00594
2R:3646540-3646690:plus -21.6939 2.79817 4.91743 3.31193 -17.6429 0.459184 2.99083 2.04167 0.715596
0.00226 0.00374 0.00184 0.00786 0.00562 0.000213 0.000119 0.00849 0.0154
2R:3659140-3659290:minus -32.0102 -2.13761 -2.69725 2.33028 -18.4796 -7.57143 -13.2569 3.88889 0.0642202
0.0172 0.0202 0.0265 0.0123 0.011 0.000644 0.00613 0.00421 0.0181
2R:3659140-3659290:plus -32.7347 1.24771 2.45872 7.72477 -8.97959 -25.8265 -13.633 1.93056 -1.26606
0.0225 0.00661 0.00488 0.000681 0.0023 0.0212 0.00668 0.00883 0.0281
2R:3661360-3661510:minus -13.4898 2.01835 -2.43119 4.23853 -18.449 -17.0102 -13.0092 1.48611 0.990826
0.000946 0.00499 0.0245 0.00522 0.0107 0.00453 0.00579 0.0103 0.0143
2R:3661360-3661510:plus -32 1.80734 -0.899083 -1.30275 -8.92857 -8.72449 -19.3028 -0.152778
-5.97248 0.0171 0.00539 0.0155 0.0404 0.00194 0.00169 0.0183 0.0175 0.0883
2R:3664180-3664330:minus -21.8878 -2.33028 1.13761 1.77064 -8.86735 -19.051 -11.4862 -3.38889 -2.18349
0.00258 0.0214 0.00788 0.0159 0.00173 0.0165 0.00413 0.0426 0.0351
2R:3664180-3664330:plus -32 2.37615 -2.46789 2.19266 -18.6837 -8.16327 -5.30275 2.625 -2.22018
0.0171 0.00437 0.0248 0.0131 0.0126 0.000783 0.00108 0.00687 0.0355
2R:3667060-3667210:minus -30.2143 -1.66972 1.09174 3.13761 -27.9694 -36.8878 0.330275 1.13889 7.94495
0.0088 0.0176 0.00801 0.00854 0.0437 0.123 0.000262 0.0116 0.000667
2R:3667060-3667210:plus -22.8571 1.46789 -1.08257 3.78899 -17.4184 0.897959 -17.211 -1.66667 -3.59633
0.00427 0.0061 0.0164 0.00632 0.00457 0.000144 0.0132 0.0272 0.0507
2R:3667780-3667930:minus -13.2245 1.88991 -0.761468 3.05505 -27.2347 -8.65306 -13.7523 3.5 0.697248
0.000738 0.00523 0.0148 0.00878 0.0283 0.00156 0.00686 0.00491 0.0154
2R:3667780-3667930:plus -40.9592 4.11009 2.12844 3.24771 -18.7551 -8.68367 -20.8807 -2.13889 -2.33028
0.0449 0.00231 0.00551 0.00808 0.0144 0.00157 0.0241 0.0309 0.0366
2R:3670000-3670150:minus -12.9592 4.16514 0.183486 -1.66972 -18.4592 -18.3469 0.110092 -3.47222 5.59633
0.000551 0.00226 0.0109 0.0458 0.0109 0.0124 0.000276 0.0434 0.00234
2R:3670000-3670150:plus -12.6224 -0.504587 9.30275 0.844037 -18.1224 -27.4082 -12.8257 -2.81944
1.23853 0.000453 0.0122 0.000213 0.0215 0.00782 0.0356 0.00555 0.0369 0.0132
2R:3670680-3670830:minus -20.551 8.69725 -0.899083 1.77982 -7.89796 -18.0102 -17.7982 0.166667

-2.47706 0.00168 0.000263 0.0155 0.0158 0.000678 0.00812 0.0145 0.0159 0.0385
2R:3670680-3670830:plus -31.7041 4.20183 4.02752 4.12844 -26.6735 -9.72449 -18.4862 6.81944 -0.220183
0.0146 0.00223 0.00265 0.00549 0.0191 0.00352 0.0161 0.0011 0.0201
2R:3673480-3673630:minus -31.9592 -2.23853 0.12844 4.86239 -8.16327 1.05102 -24.7982 2.75 -7.95413
0.0167 0.0208 0.0111 0.00449 0.000873 0.000107 0.0459 0.00656 0.134
2R:3673480-3673630:plus -24.2959 0.293578 6.83486 2.78899 10.6224 -7.5 -5.6055 -1.36111 1.59633
0.00781 0.00932 0.000785 0.00992 0.0000113 0.000614 0.00118 0.025 0.0113
2R:3674000-3674210:minus -22.1837 -1.52294 1.14679 -0.220183 -18.2245 -8.57143 -12.8165 4.16667
-3.75229 0.00315 0.0169 0.00785 0.0287 0.00877 0.00149 0.00554 0.00375 0.0521
2R:3674000-3674210:plus -14.2245 -1.05505 1.33945 0.752294 -28.3061 -25.602 -6.17431 3.61111 -0.449541
0.00126 0.0146 0.00733 0.0221 0.0553 0.0207 0.00137 0.0047 0.0216
2R:3679100-3679250:minus -14.2245 4.88991 3.05505 6.36697 -26.9286 -16.9388 -10.8991 1.04167 -0.201835
0.00126 0.0017 0.00389 0.00193 0.0214 0.00433 0.00366 0.012 0.0199
2R:3679100-3679250:plus -23.449 0.541284 0.587156 2.62385 -18.8265 -18.051 -22.4771 6.54167 1.2844
0.00631 0.00854 0.00952 0.0108 0.0149 0.00886 0.0321 0.00127 0.0129
2R:3680720-3680870:minus -31.8469 0.137615 -0.669725 7.63303 -18.7143 -27.7449 -12.4954 2.69444
5.23853 0.0162 0.00984 0.0144 0.000745 0.0136 0.0448 0.00515 0.00669 0.00285
2R:3680720-3680870:plus -33.6327 13.4592 -1.18349 1.99083 2.87755 -8.45918 -21.4312 2.59722 -3.12844
0.029 0.000064 0.0169 0.0144 0.0000259 0.00111 0.0266 0.00694 0.046
2R:3693120-3693270:minus -21.8878 1.55046 -5.10092 2.04587 -17.3776 -19.3061 8.6422 9.04167 5.34862
0.00258 0.00592 0.0506 0.014 0.00419 0.0187 0.0000199 0.000312 0.00271
2R:3693120-3693270:plus -31.3367 -0.0917431 3.62385 6.82569 -27.0102 -16.051 -19.1835 7.33333
8.10092 0.0114 0.0106 0.00312 0.00144 0.0237 0.00408 0.018 0.000844 0.000623
2R:3693840-3693990:minus -13.9898 -4.2844 3.77064 4.33945 -18.4796 -18.0816 -12.8716 4.83333 -0.889908
0.00114 0.0365 0.00294 0.00509 0.011 0.00934 0.00561 0.00283 0.0253
2R:3693840-3693990:plus -5.19388 13.5413 -3.61468 2.00917 -18.5612 -0.173469 -6.01835 3.70833
-0.605505 0.000229 0.0000137 0.0342 0.0142 0.0119 0.000456 0.00132 0.00452 0.023
2R:3694400-3694550:minus -22.0714 -0.642202 1.50459 1.51376 -18.449 -17.0102 -4.88073 -1.68056
-0.440367 0.00299 0.0128 0.00692 0.0175 0.0107 0.00453 0.00096 0.0273 0.0216
2R:3694400-3694550:plus -31.6633 2.61468 5.66055 7.99083 -17.2653 0.826531 -12.3119 -3.40278 4.87156
0.0141 0.004 0.00134 0.00059 0.00387 0.000153 0.00494 0.0427 0.00325
2R:3699600-3699750:minus -30.1429 -2.08257 -1.91743 2.62385 0.877551 -17.7143 -18.4312 -2.23611 6.17431
0.00866 0.0199 0.0211 0.0108 0.00019 0.00634 0.016 0.0317 0.00171
2R:3699600-3699750:plus -30.7755 4.86239 0.981651 0.862385 -27 -25.898 -18.0275 4.45833 1.54128
0.0102 0.00172 0.00832 0.0214 0.0233 0.0214 0.015 0.00332 0.0115
2R:3699940-3700090:minus -31.4388 -1.10092 12.6697 7.29358 10.6224 -27.6735 -20.7156 -1.65278 5.84404
0.0122 0.0148 0.0000269 0.00104 0.0000113 0.0443 0.0234 0.0271 0.00202
2R:3699940-3700090:plus -32.5408 4.02752 0.302752 1.06422 -17.7857 -18.4184 -11.1101 -1.01389 1.52294
0.0204 0.00238 0.0105 0.0203 0.00676 0.0127 0.00382 0.0227 0.0117
2R:3702340-3702490:minus -31.9184 3.68367 2.87156 5.38532 -7.93878 -17.3469 -14.2018 3.33333 2.13761
0.0164 0.00144 0.00417 0.00382 0.000767 0.0058 0.00758 0.00525 0.00885
2R:3702340-3702490:plus -22.6633 3.86239 -1.9633 7.90826 -26.5306 -26.3367 -10.5046 1.77778 -2.59633
0.00384 0.00253 0.0214 0.000611 0.0185 0.0221 0.00339 0.00932 0.0398
2R:3702980-3703130:minus -31.7041 0.633028 9.3578 10.7615 -19.051 -10.0918 -15.4404 1.15278 3.05505
0.0146 0.00826 0.000206 0.0000826 0.0162 0.00404 0.00978 0.0115 0.00692
2R:3702980-3703130:plus -14.7449 1.54128 10.7706 7.55963 -18.449 -9.53061 -16.3578 1.98611 7.22018
0.00157 0.00594 0.0000912 0.000807 0.0107 0.0031 0.0115 0.00866 0.00119
2R:3711020-3711170:minus -22.9592 1.58716 0.688073 6.21101 -27.2653 -9.30612 -7.47706 2.48611 2.80734
0.00475 0.00584 0.0092 0.00213 0.0302 0.00257 0.00188 0.00723 0.00742
2R:3711020-3711170:plus -29.4082 7.00917 -3.80734 2.74312 -28.4592 1.31633 -14.1743 -0.416667
3.92661 0.0084 0.000646 0.036 0.0101 0.0568 0.000066 0.00754 0.019 0.00461
2R:3711180-3711330:minus -22.9592 1.58716 0.688073 6.21101 -27.2653 -9.30612 -7.47706 2.48611 2.80734
0.00475 0.00584 0.0092 0.00213 0.0302 0.00257 0.00188 0.00723 0.00742
2R:3711180-3711330:plus -4.40816 7.11927 1.78899 0.53211 -26.5306 -7.72449 -4.68807 3.375 9.3945

0.000148 0.000611 0.00625 0.0234 0.0185 0.00068 0.000908 0.00516 0.000358
2R:3712780-3712930:minus -14.5204 13.5306 8.19266 5.43119 -8.86735 -19.3061 -15.7982 5.61111 5.70642
0.00145 0.0000206 0.000395 0.00371 0.00173 0.0187 0.0105 0.00199 0.00222
2R:3712780-3712930:plus -31.4388 7.11927 14.8257 5.22936 -8.16327 -18.2347 -17.0275 3.19444 3.72477
0.0122 0.000611 2.55e-06 0.0041 0.000873 0.0104 0.0129 0.00554 0.00497
2R:3804300-3804450:minus -33.3367 4.93578 -2.0367 5.30275 1.87755 -19.051 -21.1376 8.90278 -0.908257
0.0281 0.00167 0.0219 0.00396 0.0000492 0.0165 0.0252 0.000341 0.0255
2R:3804300-3804450:plus -22.3265 3.83673 -1.58716 2.3578 2.14286 -0.0204082 -5.14679 1.84722 2
0.00337 0.00115 0.0191 0.0121 0.0000316 0.000411 0.00104 0.0091 0.00941
2R:3804780-3804930:minus -31.3673 -1.76147 -2.45872 7.90826 -17.0408 -36.6735 -10.4037 5.23611 6.17431
0.0116 0.0181 0.0247 0.000611 0.00329 0.118 0.00332 0.00236 0.00171
2R:3804780-3804930:plus -21.4796 8.17431 1.06422 0.981651 -18.449 -18.7551 -12 -2.84722 3.46789
0.00202 0.000347 0.00808 0.0207 0.0107 0.0141 0.00461 0.0372 0.00582
2R:3819760-3819910:minus -24.449 9.00917 5.21101 2.05505 -27.1939 1.31633 -19.5505 0.0138889
-0.229358 0.00802 0.000222 0.00163 0.0139 0.0267 0.000066 0.0191 0.0167 0.0202
2R:3819760-3819910:plus -22.2551 -0.12844 1.7156 7.00917 -18.1224 -17.4184 -7.04587 1.45833 0.972477
0.00327 0.0108 0.00642 0.00126 0.00782 0.00585 0.00171 0.0104 0.0144
2R:3843460-3843610:minus -14.8163 8.9633 7.52294 3.45872 -25.9286 -17.9388 -7.66972 7.43056 1.72477
0.00159 0.000228 0.00056 0.00731 0.0168 0.00738 0.00196 0.000801 0.0106
2R:3843460-3843610:plus -29.1429 2.26606 -4.80734 -0.761468 -27.2653 -0.469388 -19.6972 6.33333
-2.66972 0.00834 0.00455 0.047 0.0341 0.0302 0.000533 0.0196 0.00141 0.0407
2R:3847380-3847530:minus -22.8469 -0.431193 10.9083 6.84404 -8.64286 -9.45918 -21.8349 -1.75
-0.761468 0.00424 0.0119 0.0000846 0.0014 0.00139 0.00279 0.0286 0.0279 0.0244
2R:3847380-3847530:plus -41.3673 1.12844 -0.0733945 4.02752 -27.1939 19.1531 -24.789 1.51389
-8.22018 0.0551 0.00691 0.0119 0.00581 0.0267 7.95e-07 0.0459 0.0102 0.142
2R:3849520-3849670:minus -40.7653 13.3878 7.82569 2.25688 -18.0816 -18.0816 -15.5229 5.01389 4.61468
0.0432 0.0000869 0.00048 0.0127 0.00759 0.00934 0.00994 0.00261 0.00351
2R:3849520-3849670:plus -29.8776 0.550459 4.22936 2.66972 -19.051 0.173469 -19.3578 3.31944 1.61468
0.00852 0.00851 0.00244 0.0106 0.0162 0.000307 0.0185 0.00528 0.0112
2R:3849860-3850010:minus -13.6327 0.752294 -1.17431 5.57798 -18.7143 -9.72449 -2.76147 5.875 -3.9633
0.00101 0.00791 0.0169 0.0034 0.0136 0.00352 0.000534 0.00176 0.0551
2R:3849860-3850010:plus -31.1735 7.79817 -0.522936 5.59633 -28.0408 -19.0816 -15.0367 -1.23611
3.02752 0.0109 0.000426 0.0137 0.00331 0.0455 0.017 0.00904 0.0241 0.00695
2R:3859360-3859510:minus -22.8571 13.4592 0.669725 3.7156 -27.2347 -27.0408 -15.0734 4.94444 -1.73394
0.00427 0.000064 0.00926 0.00655 0.0283 0.0307 0.00911 0.00269 0.0311
2R:3859360-3859510:plus -41.4796 13.4592 1.06422 6.12844 -18.1531 -7.86735 -17.156 -0.583333
1.16514 0.0577 0.000064 0.00808 0.00231 0.00829 0.00073 0.0131 0.02 0.0135
2R:3859960-3860110:minus -22.0306 -2.98165 -2.40367 -4.3211 -28.8265 -10.0918 -15.9083 3.22222 4.7156
0.00293 0.0257 0.0243 0.0908 0.0648 0.00404 0.0107 0.00548 0.00345
2R:3859960-3860110:plus -23.3367 15.844 -1.16514 0.651376 -18.4898 0.122449 -16.2661 2.20833 2.04587
0.00614 3.15e-07 0.0168 0.0226 0.0116 0.000312 0.0114 0.008 0.00919
2R:3871680-3871830:minus -21.2857 2.38532 1.79817 9.7156 -27.0408 -18.7857 -21.9725 -1.81944 -0.12844
0.00191 0.00436 0.00623 0.000242 0.0242 0.0146 0.0293 0.0284 0.0193
2R:3871680-3871830:plus -20.9592 0.807339 6.88991 2.72477 -17.7143 -18.1224 -17.945 -0.583333
0.733945 0.00184 0.00776 0.000765 0.0102 0.00627 0.00988 0.0148 0.02 0.0153
2R:3875640-3875790:minus -21.7041 -1.01835 6.80734 3.97248 -18.7143 -26.898 -13.844 0.222222 1.38532
0.00231 0.0144 0.000794 0.00587 0.0136 0.0302 0.00701 0.0156 0.0123
2R:3875640-3875790:plus -22.8878 -5.33028 6.51376 6.12844 -18.449 -17.9796 -4.59633 -3.31944 -0.220183
0.00441 0.0481 0.000912 0.00231 0.0107 0.00764 0.000884 0.0418 0.0201
2R:3876020-3876170:minus -21.6327 3.97959 3.37615 5.74312 -19.051 -18.6429 -5.98165 0.430556 1.16514
0.00215 0.000669 0.00343 0.00302 0.0162 0.0139 0.0013 0.0146 0.0135
2R:3876020-3876170:plus -19.8878 2.57798 -3.24771 6.02752 -17.1531 -18.7857 -20.4862 -0.152778
-4.19266 0.00164 0.00406 0.0309 0.00246 0.00364 0.0146 0.0224 0.0175 0.0583
2R:3876640-3876790:minus -14.2245 1.88073 1.50459 5.22936 -26.9694 0.27551 -19.8716 0.361111 10.3394

0.00126 0.00524 0.00692 0.0041 0.023 0.000257 0.0201 0.0149 0.000112
2R:3876640-3876790:plus -31.7755 1.47706 0.697248 3.62385 -25.898 -8.5 -13.7248 3.13889 1.22018
0.0157 0.00608 0.00917 0.00673 0.0166 0.00126 0.00682 0.00566 0.0133
2R:3877160-3877310:minus -32.7347 1.90826 6.68807 7.72477 -17.449 -27.6327 -7.14679 3.55556 5.59633
0.0225 0.00519 0.000839 0.000681 0.00484 0.043 0.00175 0.00481 0.00234
2R:3877160-3877310:plus -11.7755 -4.47706 6.14679 2.19266 -26.5918 -26.602 -12.3945 4.29167 -1.59633
0.000266 0.0384 0.00108 0.0131 0.0186 0.0248 0.00504 0.00356 0.0301
2R:3955180-3955330:minus -24.4796 13.4592 6.3578 9.44954 -27.2245 -8.53061 -16.3853 -1.20833 -4.44037
0.00804 0.000064 0.000982 0.000326 0.0272 0.00136 0.0116 0.0239 0.063
2R:3955180-3955330:plus -13.7041 8.76147 -2.04587 4.20183 10.3571 -17.7857 -13.055 4.59722 1.69725
0.00104 0.000254 0.0219 0.00533 0.0000208 0.0068 0.00585 0.00313 0.0108
2R:3956420-3956570:minus -23.1122 -0.926606 6.18349 2.21101 -17.1837 -18.2755 -21.8899 2.13889
1.22936 0.0053 0.014 0.00106 0.0129 0.00366 0.0112 0.0289 0.0082 0.0132
2R:3956420-3956570:plus -31.3673 -3.11009 6.66972 5.74312 -17.4184 -19.2755 -15.3394 -2.5 -4.79817
0.0116 0.0266 0.000847 0.00302 0.00457 0.0185 0.0096 0.034 0.0693
2R:3967480-3967630:minus -23.7755 -1.45872 -1.77064 1.68807 10.9184 -18.2041 -17.8716 4.79167 1.2844
0.00678 0.0165 0.0202 0.0164 4.19e-06 0.0103 0.0147 0.00288 0.0129
2R:3967480-3967630:plus -30.551 -0.293578 -4.36697 3.47706 -18.1122 -9.30612 -23.5413 3.5 5.9633
0.00959 0.0114 0.0419 0.00724 0.00771 0.00257 0.0383 0.00491 0.00188
2R:3970340-3970490:minus -23.5204 -2.47706 4.73394 1.40367 -18.7449 -8.45918 -10.9266 8.45833 -0.697248
0.00645 0.0223 0.00199 0.0184 0.0138 0.00111 0.00368 0.000447 0.0239
2R:3970340-3970490:plus -22.6531 0.853211 9.49541 11.2936 -17.0408 -26.6735 -1.97248 -4.5 -2.27523
0.00374 0.00763 0.000191 0.0000139 0.00329 0.0256 0.000441 0.0552 0.036
2R:3986020-3986170:minus -31.7449 1.66972 2.23853 0.605505 -9.5 -0.469388 -20.1284 5.15278 2.17431
0.0152 0.00567 0.00529 0.023 0.00293 0.000533 0.0211 0.00245 0.00872
2R:3986020-3986170:plus -31.5918 15.844 -3.22936 1.83486 -27.2653 -18.0102 -18.6055 3.29167 1.57798
0.0136 3.15e-07 0.0307 0.0155 0.0302 0.00812 0.0164 0.00533 0.0114
2R:3986500-3986650:minus -33.3673 6.91743 -2.22018 6.17431 -18.6429 -8.7551 -10.7798 2.29167 1.95413
0.0282 0.000678 0.0231 0.0022 0.0121 0.00172 0.00357 0.00776 0.00964
2R:3986500-3986650:plus -24.449 4.86239 -0.0825688 -0.146789 -26.9694 -27.1224 -16.211 1.59722
2.16514 0.00802 0.00172 0.0119 0.028 0.023 0.0325 0.0113 0.00993 0.00879
2R:3993760-3993910:minus -23.5204 13.5306 -2.46789 3.56881 -26.9286 -17.9388 -13.8716 -0.958333
5.18349 0.00645 0.0000206 0.0248 0.00699 0.0214 0.00738 0.00705 0.0223 0.00295
2R:3993760-3993910:plus -4.52041 14.6055 -1.66972 1.57798 -17.1939 -18.0102 -19.8257 2.59722 0.93578
0.000158 4.26e-06 0.0196 0.0171 0.00377 0.00812 0.02 0.00694 0.0145
2R:4010380-4010530:minus -23.4694 0.449541 2.38532 1.44037 -17.8265 -36.449 -14.8716 8.33333 3.44954
0.00632 0.00882 0.00501 0.018 0.00691 0.109 0.00874 0.000481 0.00594
2R:4010380-4010530:plus -31.5102 8.6422 5.6789 6.3578 -17.7143 9.93878 -17.9358 3.41667 2.12844
0.013 0.00027 0.00133 0.00195 0.00627 0.0000108 0.0148 0.00508 0.00886
2R:4014760-4014910:minus -32.6939 1.24771 -4.45872 5.31193 -17.449 -9.86735 -9.6422 2.54167 -1.22018
0.0217 0.00661 0.0429 0.00393 0.00484 0.00391 0.00288 0.00709 0.0278
2R:4014760-4014910:plus -31.7755 14.7523 2.06422 1.78899 -18.4898 -9.94898 -17.2752 2.20833 4.13761
0.0157 3.48e-06 0.00564 0.0158 0.0116 0.00393 0.0134 0.008 0.00408
2R:4018880-4019030:minus -41.3673 -3.21101 -3.43119 1.44037 -9.19388 -8.79592 -16.6514 2.98611 7.73394
0.0551 0.0273 0.0325 0.018 0.00253 0.0019 0.0121 0.006 0.000784
2R:4018880-4019030:plus -23.9898 5.85321 3.44954 6.00917 -18.5306 0.755102 -4.11009 3.22222 3.20183
0.00711 0.00113 0.00334 0.00255 0.0118 0.000175 0.00077 0.00548 0.0065
2R:4019420-4019570:minus -32.8469 1.89908 7.05505 7.20183 -18.5306 -17.3469 -20.2752 7.69444 9.45872
0.0243 0.00521 0.000708 0.00112 0.0118 0.0058 0.0216 0.000693 0.000337
2R:4019420-4019570:plus -31.4796 -0.990826 4.43119 6.14679 -17.7143 -28.9694 -9.55046 -2.19444
3.40367 0.0128 0.0143 0.00225 0.00224 0.00627 0.0721 0.00283 0.0314 0.00603
2R:4030960-4031110:minus -30.1837 -0.568807 -0.834862 3.52294 -17.449 -16.7551 -21.9541 -1.34722
11.8991 0.00873 0.0125 0.0152 0.00706 0.00484 0.0042 0.0292 0.0249 0.0000255
2R:4030960-4031110:plus -42.6633 0.880734 0.0917431 4.12844 -18.4898 -10.0918 -15.3761 2.72222

3.20183 0.0837 0.00756 0.0112 0.00549 0.0116 0.00404 0.00966 0.00663 0.0065
2R:4031440-4031590:minus -22.9286 6.50459 0.651376 2.70642 -26.1939 -26.6735 -18.5963 -1.05556 1.33945
0.0046 0.000833 0.00932 0.0103 0.0175 0.0256 0.0164 0.0229 0.0125
2R:4031440-4031590:plus -22.6939 5.76147 -5.16514 0.853211 -27.8265 -27.602 -1.26606 0.513889 7.83486
0.00388 0.00118 0.0515 0.0214 0.0388 0.0416 0.000376 0.0142 0.000712
2R:4032680-4032830:minus -23.1224 1.77982 4.22936 2.9633 -17.6429 -16.9796 -22.5229 -3.08333 -9.10092
0.00531 0.00544 0.00244 0.00914 0.00562 0.0044 0.0323 0.0395 0.169
2R:4032680-4032830:plus -31.7041 -0.486239 -0.577982 2.09174 -18.7449 -17.5714 -7.51376 7.15278
5.24771 0.0146 0.0121 0.014 0.0137 0.0138 0.00616 0.0019 0.000928 0.00282
2R:4033360-4033510:minus -24.3265 3.97959 2.93578 9.92661 -17.3776 -27.5612 -12.1651 1.40278 2.05505
0.00784 0.000669 0.00407 0.000169 0.00419 0.0393 0.00478 0.0106 0.00914
2R:4033360-4033510:plus -31.1122 0.0642202 1.10092 3.45872 -18.4898 -17.0102 -14.8257 3.55556
11.8532 0.0107 0.0101 0.00798 0.00731 0.0116 0.00453 0.00866 0.00481 0.0000428
2R:4033940-4034090:minus -13.0306 3.93578 1.70642 1.05505 -17.7143 -18.051 -3.79817 12.7361 12.2752
0.000567 0.00246 0.00644 0.0204 0.00627 0.00886 0.000705 0.0000141 0.0000141
2R:4033940-4034090:plus -22.5918 -1.22936 2.16514 2.57798 -17.4184 -18.3469 -11.8349 3.47222 3.94495
0.00365 0.0154 0.00544 0.011 0.00457 0.0124 0.00445 0.00497 0.00452
2R:403460-403610:minus -21.9286 1.83486 1.3945 3.52294 -27.0102 -17.2755 -19.7248 5.94444 -2.3578
0.0027 0.00533 0.00719 0.00706 0.0237 0.00544 0.0196 0.0017 0.0369
2R:403460-403610:plus -30.9592 2.20183 -0.917431 5.22936 -18.4184 -0.244898 -4.81651 2.56944
-2.83486 0.0104 0.00466 0.0156 0.0041 0.0101 0.000496 0.000942 0.00701 0.0427
2R:403920-404070:minus -24.0408 6.43119 -4.56881 2.06422 -9.23469 -17.7143 -18.633 6.13889 -2.69725
0.00726 0.000862 0.0442 0.0139 0.00277 0.00634 0.0165 0.00155 0.0411
2R:403920-404070:plus -14.6735 5.29358 3.22936 3.33945 -18.9796 -9.79592 -20.8532 12.2778 -0.642202
0.00152 0.00144 0.00363 0.00774 0.0155 0.00377 0.0239 0.0000235 0.0234
2R:4039660-4039810:minus -13.6327 0.477064 -3 1.10092 -19.051 -9.42857 -15.0275 -1.55556 -4.42202
0.00101 0.00873 0.0288 0.02 0.0162 0.0027 0.00902 0.0264 0.0626
2R:4039660-4039810:plus -22.398 2.75229 0.761468 5.73394 -17.8571 -9.45918 -6.91743 -3.31944 4.56881
0.00344 0.00381 0.00897 0.00307 0.00698 0.00279 0.00165 0.0418 0.00356
2R:4041800-4041950:minus -20.7041 2.9633 11.8991 4.05505 -17.9796 -18.0102 3.99083 -2.22222 1.33028
0.00175 0.00353 0.0000462 0.00566 0.00726 0.00812 0.0000828 0.0316 0.0127
2R:4041800-4041950:plus -14.4796 5.6789 6.85321 2.42202 -8.96939 -27.8571 -4.51376 1.90278 7.15596
0.00142 0.00122 0.000779 0.0118 0.00226 0.0508 0.000864 0.00892 0.00124
2R:4042760-4042910:minus -23.1837 3.64286 1.6055 2.33945 -18.1939 -17.9388 -14.211 1.90278 1.44037
0.00553 0.00159 0.00667 0.0122 0.0086 0.00738 0.0076 0.00892 0.012
2R:4042760-4042910:plus -21.7041 13.3878 6.3945 3.90826 -8.89796 -26.3061 -9.82569 2.375 -0.816514
0.00231 0.0000869 0.000966 0.00603 0.00189 0.0219 0.00298 0.00753 0.0249
2R:4044080-4044230:minus -21.6633 0.0183486 10.156 7.45872 10.6531 0.0510204 -13.4862 -0.180556
2.98165 0.00223 0.0102 0.00013 0.000894 0.0000102 0.000349 0.00646 0.0177 0.00707
2R:4044080-4044230:plus -22.6224 8.66055 -0.798165 3.22018 -8.33673 -8.79592 -13.7248 -4.18056
2.99083 0.00371 0.000268 0.015 0.00818 0.00101 0.0019 0.00682 0.0513 0.00706
2R:4050920-4051070:minus -20.898 15.6514 13.0459 5.84404 0.877551 -18.1224 -10.9908 4.98611 3.42202
0.0018 8.05e-07 0.0000199 0.00289 0.00019 0.00988 0.00373 0.00264 0.00597
2R:4050920-4051070:plus -14.7449 13.4592 11.5321 7.29358 11.4286 -26.5612 -19.0917 2.76389 1.22936
0.00157 0.000064 0.0000579 0.00104 1.97e-06 0.0237 0.0177 0.00652 0.0132
2R:4052220-4052370:minus -23.2245 7.11009 13.9908 7.2844 -19.051 -9.86735 -13.3394 -1.31944 -3.81651
0.00576 0.000613 7.33e-06 0.00105 0.0162 0.00391 0.00625 0.0247 0.0529
2R:4052220-4052370:plus -21.8878 3.47706 1.19266 2.34862 -26 -18.7551 -15.2294 1.09722 -2.83486
0.00258 0.00292 0.00773 0.0121 0.0171 0.0141 0.00939 0.0118 0.0427
2R:4055340-4055490:minus -14.1122 -0.678899 -2.17431 3.02752 -26.9694 -19.5 -18.7339 0.541667
-0.917431 0.00119 0.0129 0.0227 0.00887 0.023 0.0199 0.0167 0.0141 0.0256
2R:4055340-4055490:plus -24.7857 13.844 -1.41284 3.77064 -17.7143 -8.7551 -24.633 6.95833 6.95413
0.00829 9.98e-06 0.0181 0.00637 0.00627 0.00172 0.0449 0.00103 0.00136
2R:4057680-4057830:minus -14.8571 6.10092 -1.38532 6.00917 -26.2653 -8.79592 -23.8624 7.90278 -4.84404

0.0016 0.00101 0.018 0.00255 0.018 0.0019 0.0402 0.000616 0.0701
2R:4057680-4057830:plus -21.5918 -1.49541 0.880734 -1.72477 10.6224 -18.2755 -15.8073 2.25 1.31193
0.00209 0.0167 0.00861 0.0465 0.0000113 0.0112 0.0105 0.00788 0.0127
2R:4061540-4061690:minus -21.8061 4.49541 0.449541 5.57798 -26.9286 -0.540816 -4.69725 -0.0138889
3.6789 0.00241 0.00199 0.00998 0.0034 0.0214 0.00054 0.00091 0.0168 0.00506
2R:4061540-4061690:plus -4.52041 4.55963 0.229358 8.05505 -17.8571 -17.7143 3.97248 3.19444 3.66972
0.000158 0.00194 0.0107 0.000578 0.00698 0.00634 0.0000833 0.00554 0.00514
2R:4061940-4062090:minus -32.4388 -4.6422 -1.20183 -1.06422 -17.4082 -8.72449 0.412844 -0.0972222
1.26606 0.0195 0.0401 0.017 0.0375 0.00421 0.00169 0.000256 0.0172 0.013
2R:4061940-4062090:plus -14.1939 -1.18349 -0.788991 -0.422018 -18.2653 -26.2653 -11.8349 2.59722
11.8073 0.00124 0.0152 0.0149 0.0305 0.00882 0.0218 0.00445 0.00694 0.0000538
2R:4062520-4062670:minus -3.44898 -0.220183 1.99083 7.21101 -27.0408 -27.6735 -16.4862 6.01389
12.2752 0.0000713 0.0111 0.0058 0.0011 0.0242 0.0443 0.0118 0.00165 0.0000141
2R:4062520-4062670:plus -32.3367 -1.56881 2.53211 1.58716 -27.1939 -17.9796 -11.1376 1.72222 1.2844
0.0187 0.0171 0.00475 0.017 0.0267 0.00764 0.00384 0.0095 0.0129
2R:4124700-4124850:minus -30.1837 0.201835 1.22936 3.41284 -7.85714 -8.79592 -21.8807 3.44444 8.05505
0.00873 0.00962 0.00763 0.00756 0.000594 0.0019 0.0289 0.00502 0.00064
2R:4124700-4124850:plus -22.2245 -5.45872 4.29358 -0.33945 -8.60204 -18.3469 -15.8899 4.75 3.98165
0.00325 0.0497 0.00238 0.0296 0.00125 0.0124 0.0106 0.00293 0.00439
2R:4125140-4125290:minus -22.5918 0.311927 -0.302752 2.58716 -18.2653 -26.602 -20.8349 0.319444
7.68807 0.00365 0.00925 0.0128 0.011 0.00882 0.0248 0.0239 0.0151 0.000841
2R:4125140-4125290:plus -20.4388 0.486239 2.77982 2.08257 1.17347 -17.3469 4.27523 6.76389 9.87156
0.00167 0.00871 0.00432 0.0137 0.000101 0.0058 0.0000746 0.00114 0.000217
2R:4130080-4130230:minus -32.8878 -2.27523 1.25688 5.89908 -18.7449 -18.0816 -14.6239 7.26389 -1.51376
0.0247 0.021 0.00755 0.00272 0.0138 0.00934 0.0083 0.000876 0.0294
2R:4130080-4130230:plus -3.7449 4.93578 -0.00917431 9.7156 -17.7143 -9.79592 -16.5229 0.319444
1.0367 0.000104 0.00167 0.0116 0.000242 0.00627 0.00377 0.0119 0.0151 0.0141
2R:4214780-4214930:minus -22.1429 -1.27523 4.0367 2.44954 -16.0816 -35.0816 -6.56881 1.04167 7.83486
0.00305 0.0156 0.00264 0.0116 0.00294 0.0746 0.00152 0.012 0.000712
2R:4214780-4214930:plus -23.2653 0.293578 6.46789 11.2936 -18.1531 -27.8265 -12.7064 -7.58333 2.78899
0.00593 0.00932 0.000933 0.0000139 0.00829 0.0498 0.00541 0.103 0.00745
2R:427800-427950:minus -32.5102 -2.54128 1.44037 -0.293578 -8.44898 -19.0816 -12.422 1.59722 9
0.0202 0.0227 0.00707 0.0292 0.00109 0.017 0.00507 0.00993 0.000437
2R:427800-427950:plus -33.1122 -1 1.73394 7.7156 -18.3776 -18.2755 -19.2936 7.19444 3.98165 0.0271
0.0143 0.00638 0.000691 0.00926 0.0112 0.0183 0.000909 0.00439
2R:4308260-4308410:minus -41.4388 -4.23853 7.0367 2.02752 -17.4184 -18.5714 -15.7248 11.375 1.78899
0.0562 0.0361 0.000715 0.0141 0.00457 0.0136 0.0103 0.0000552 0.0103
2R:4308260-4308410:plus -24.4898 10.7339 -2.66055 8.05505 20.4694 0.244898 -9.27523 3.72222 -4.89908
0.00807 0.0000914 0.0262 0.000578 1.65e-07 0.000285 0.00269 0.0045 0.0712
2R:4332080-4332230:minus -30.3265 -0.0550459 2.3945 7.33028 -18.3776 -7.86735 -2.88991 6.48611
-6.6055 0.0089 0.0105 0.00499 0.00103 0.00926 0.00073 0.000552 0.00131 0.104
2R:4332080-4332230:plus -29.1837 0.844037 1.88991 1.62385 -17.3469 -7.5 -1.33945 10.4722 -2.99083
0.00835 0.00766 0.00602 0.0168 0.00394 0.000614 0.000383 0.000115 0.0445
2R:434120-434270:minus -42.7449 -3.14679 0.770642 -0.990826 -9.16327 -0.540816 -24.9725 5.80556
-3.43119 0.0847 0.0268 0.00895 0.0366 0.00249 0.00054 0.047 0.00182 0.0489
2R:434120-434270:plus -33.0102 2.38532 -1.92661 1.05505 -9.0102 -18.5714 -25.5046 7.25 1.33028 0.0259
0.00436 0.0212 0.0204 0.00236 0.0136 0.0503 0.000882 0.0127
2R:434860-435010:minus -31.5918 6.01835 7.73394 -2.53211 -9.5 -18.3469 -16 5.45833 -0.440367
0.0136 0.00105 0.000504 0.0608 0.00293 0.0124 0.0108 0.00214 0.0216
2R:434860-435010:plus -40.8163 2.69725 1.57798 1.6789 -27.1633 -8.42857 -30.4771 4.16667 -5 0.0436
0.00389 0.00674 0.0165 0.025 0.00104 0.0972 0.00375 0.0727
2R:4485260-4485410:minus -23 4.81651 6.18349 3.45872 -18.3878 -27.4082 -6.26606 -2.04167 1.00917
0.00498 0.00175 0.00106 0.00731 0.00931 0.0356 0.00141 0.0301 0.0143
2R:4485260-4485410:plus -20.3673 -0.40367 3.41284 9.08257 -17.8265 -9.0102 -12.6789 0.402778 6.76147

0.00166 0.0118 0.00338 0.000444 0.00691 0.00208 0.00537 0.0147 0.00141
2R:4486180-4486330:minus -11.8163 4.6055 8.11009 7.37615 -26.2653 -27.7041 -9.29358 -5.84722 1.86239
0.000272 0.00191 0.000413 0.000951 0.018 0.0445 0.0027 0.0738 0.00994
2R:4486180-4486330:plus -10.8571 -1.12844 4.21101 3.9633 -18.4184 -28.3061 -11.6514 -4.02778 3.57798
0.000237 0.0149 0.00246 0.0059 0.0101 0.0597 0.00427 0.0496 0.00536
2R:4486680-4486830:minus -30.4388 -2.18349 3.24771 2.76147 -17.4184 -18.5612 -26.5138 1.13889 0.146789
0.00923 0.0205 0.00361 0.01 0.00457 0.0132 0.057 0.0116 0.0178
2R:4486680-4486830:plus -31.4796 3.97248 7.90826 7.12844 -26.898 -8.45918 -18.6147 -0.0833333
4.98165 0.0128 0.00243 0.00046 0.00118 0.0203 0.00111 0.0164 0.0172 0.00313
2R:4487720-4487870:minus -22.2245 -0.266055 1.0367 -1.09174 -18.7143 -9.57143 -20.8165 5.25 1.61468
0.00325 0.0113 0.00816 0.0379 0.0136 0.00326 0.0238 0.00235 0.0112
2R:4487720-4487870:plus -21.8878 -1.26606 4.99083 6.6422 10.9184 -18.051 -13.3028 -3.18056 3.42202
0.00258 0.0156 0.00178 0.00161 4.19e-06 0.00886 0.0062 0.0404 0.00597
2R:4495500-4495650:minus -22.3367 -2.02752 5.27523 4.9633 -18.7857 -9.79592 -4.15596 0.972222 3.51376
0.00339 0.0196 0.00158 0.0044 0.0148 0.00377 0.00078 0.0123 0.00566
2R:4495500-4495650:plus -3.36735 5.75229 0.46789 0.568807 -18.3469 -18.1633 -12.2385 2.30556 3.17431
0.000063 0.00118 0.00992 0.0232 0.00894 0.01 0.00486 0.00772 0.00653
2R:4502100-4502250:minus -41.6327 -3.10092 -2.88073 4.22018 -18.6429 1.05102 -26.1284 7.70833 -0.541284
0.0631 0.0265 0.0279 0.00531 0.0121 0.000107 0.0543 0.000687 0.0223
2R:4502100-4502250:plus -32.7041 -0.93578 5.19266 2.62385 -27.6633 0.0204082 9.76147 -1.875
3.66972 0.0222 0.014 0.00164 0.0108 0.0372 0.000377 0.0000141 0.0288 0.00514
2R:4512820-4512970:minus -32.2959 3.97248 1.63303 4.78899 -18.7143 -8.72449 -17.0367 0.180556 -2.15596
0.0186 0.00243 0.00661 0.00456 0.0136 0.00169 0.0129 0.0158 0.0348
2R:4512820-4512970:plus -34.3367 -2.21101 8.05505 1.74312 10.4286 -9.72449 -16.8899 3.125 -2.98165
0.0318 0.0206 0.000425 0.016 0.0000139 0.00352 0.0126 0.00569 0.0445
2R:4515080-4515230:minus -21.8878 3.68807 0.93578 3.13761 1.21429 0.979592 -20.9174 8.20833 -2.47706
0.00258 0.0027 0.00845 0.00854 0.0000864 0.000136 0.0242 0.000518 0.0385
2R:4515080-4515230:plus -31.8469 -0.706422 -1.69725 6.36697 -19.0102 -0.244898 -20.9358 3.36111
-0.440367 0.0162 0.0131 0.0197 0.00193 0.0157 0.000496 0.0243 0.00519 0.0216
2R:4519320-4519470:minus -24.7857 1.25688 0.211009 3.6055 20.2041 -27.5612 -19 -0.986111 3.97248
0.00829 0.00659 0.0108 0.00686 3.64e-07 0.0393 0.0175 0.0225 0.00444
2R:4519320-4519470:plus -21.7449 2.24771 6.24771 2.81651 -27.2347 -9.37755 -13.4495 4.43056 3.62385
0.00236 0.00458 0.00103 0.00982 0.0283 0.00265 0.00641 0.00336 0.00523
2R:4537000-4537150:minus -23.3367 5.77064 -1.81651 1.44037 -18.1939 -18.7143 -9.72477 3.375 1.97248
0.00614 0.00117 0.0205 0.018 0.0086 0.014 0.00292 0.00516 0.00948
2R:4537000-4537150:plus -13.5204 0.53211 0.715596 5.76147 -8.89796 -27.3061 22.5138 -0.736111
9.30275 0.00097 0.00856 0.00912 0.00295 0.00189 0.0336 2.51e-08 0.0209 0.00038
2R:4537520-4537670:minus -30.4082 2.05505 2.10092 2.87156 -7.93878 -9.16327 -17.0642 7.06944 2.73394
0.00908 0.00492 0.00556 0.00948 0.000767 0.00223 0.0129 0.00097 0.00756
2R:4537520-4537670:plus -23.0306 1.93578 -1.41284 2.27523 -18.7449 -36.0408 -9.01835 5.45833 5.18349
0.00505 0.00514 0.0181 0.0125 0.0138 0.0837 0.00257 0.00214 0.00295
2R:4539160-4539310:minus -22.4184 -2.05505 5.6422 7.47706 -8.96939 0.0510204 -11.3119 -2.22222
-1.31193 0.00345 0.0197 0.00135 0.00085 0.00226 0.000349 0.00398 0.0316 0.0284
2R:4539160-4539310:plus -13.0816 -0.46789 7 7.62385 -18.4184 -8.5 -19.4679 -6.59722 2.75229 0.000599
0.0121 0.000727 0.000759 0.0101 0.00126 0.0188 0.0857 0.00748
2R:4553520-4553670:minus -22.6939 5.97248 -1.07339 1.50459 -18.6735 -19.3469 -18.4862 -1.84722 -4.77064
0.00388 0.00107 0.0163 0.0176 0.0123 0.0194 0.0161 0.0286 0.0687
2R:4553520-4553670:plus -21.5918 -0.981651 -2.72477 2.93578 -28.0816 -26.4898 -8.62385 -0.263889
5.3945 0.00209 0.0143 0.0267 0.00927 0.0455 0.0225 0.00238 0.0181 0.00263
2R:4555240-4555390:minus -4.77551 -3.61468 5.31193 10.8624 -18.1837 -9.34694 -8.01835 8.125 11.3853
0.000192 0.0305 0.00156 0.0000687 0.00835 0.00261 0.00211 0.000543 0.0000815
2R:4555240-4555390:plus -13.2959 1.89908 -1.21101 5.24771 -17.4184 -18.2755 -17.6697 -2.83333 6.02752
0.000775 0.00521 0.017 0.00403 0.00457 0.0112 0.0142 0.0371 0.00183
2R:4556380-4556530:minus -4 -4.11009 2.33028 1.92661 -17.9388 -18.1224 -13.6697 4.75 7.42202 0.000121

0.0348 0.00511 0.0148 0.00715 0.00988 0.00674 0.00293 0.00106
2R:4556380-4556530:plus -23.0714 -2.54128 -2.6055 6.33945 -18.2245 -17.9388 -12.5046 0.125 -3.94495
0.0052 0.0227 0.0258 0.00196 0.00877 0.00738 0.00516 0.0161 0.0549
2R:4558040-4558190:minus -31.4796 2.25688 -0.87156 -1.77982 -27.1939 -16.2755 -12.7615 5.22222 5.90826
0.0128 0.00457 0.0153 0.0474 0.0267 0.00413 0.00547 0.00238 0.00198
2R:4558040-4558190:plus -31.4388 -1.75229 -2.18349 5.49541 -17.602 -18.2041 -18.8532 -2.97222 -5.77064
0.0122 0.0181 0.0228 0.00355 0.00547 0.0103 0.017 0.0384 0.0846
2R:4558660-4558810:minus -33.1122 0.87156 -1.13761 7.63303 -8.60204 -27.1224 -14.7156 8.09722 3.20183
0.0271 0.00758 0.0167 0.000745 0.00125 0.0325 0.00847 0.000552 0.0065
2R:4558660-4558810:plus -22.5102 2.22936 6.81651 7.6422 -18.2245 -27.6735 -3.09174 -1.19444 8.95413
0.00354 0.00461 0.000791 0.000721 0.00877 0.0443 0.000582 0.0239 0.000458
2R:4561040-4561190:minus -41.102 -0.541284 2.99083 1.06422 -18.4898 -27.602 -9.46789 6.08333
1.33945 0.0472 0.0124 0.00399 0.0203 0.0116 0.0416 0.00279 0.00159 0.0125
2R:4561040-4561190:plus -29.2143 0.917431 4.01835 3.14679 -17.4898 -18.9388 -9.93578 2.75 6.73394
0.00836 0.00746 0.00266 0.00847 0.0053 0.0154 0.00304 0.00656 0.00143
2R:4609280-4609430:minus -13.2245 -1.18349 9.55963 7.55963 -18.449 -17.3469 -12.1927 3.18056 0.954128
0.000738 0.0152 0.000184 0.000807 0.0107 0.0058 0.00481 0.00557 0.0145
2R:4609280-4609430:plus -22.9694 -1.75229 7.53211 7.61468 -18.7551 -8.53061 -18.6239 -2.05556 -2.43119
0.0048 0.0181 0.000558 0.000779 0.0144 0.00136 0.0165 0.0303 0.038
2R:4609960-4610110:minus -31.7347 -2.99083 -1.3578 5.91743 0.653061 -7.79592 -29.0092 3.70833 -2.94495
0.0149 0.0257 0.0178 0.00267 0.000239 0.000723 0.079 0.00452 0.0441
2R:4609960-4610110:plus -42 5.53211 -3.63303 0.880734 -18.7551 -17.1633 -16.7339 2.55556 -3.92661
0.0684 0.0013 0.0344 0.0213 0.0144 0.005 0.0123 0.00705 0.0546
2R:4611060-4611210:minus -13.1735 2.6789 1.69725 6.11009 -26.9286 -18.2755 -7.12844 -4.02778 0.0275229
0.000671 0.00391 0.00646 0.00233 0.0214 0.0112 0.00174 0.0496 0.0183
2R:4611060-4611210:plus -22.2245 3.64286 4.48624 3.61468 -18.6735 -18.2347 -28.055 8.90278 -4.44037
0.00325 0.00159 0.0022 0.0068 0.0123 0.0104 0.0693 0.000341 0.063
2R:4611680-4611830:minus -43.4082 6.05505 -0.412844 5.74312 -9.0102 -16.3469 -30.0459 4.04167
-2.91743 0.0912 0.00103 0.0133 0.00302 0.00236 0.00416 0.0915 0.00395 0.0436
2R:4611680-4611830:plus -30.6224 3.0367 2.26606 0.412844 -17.7551 -27.9694 -1.08257 3.875 7.6422
0.00964 0.00343 0.00524 0.0242 0.00658 0.0547 0.000361 0.00423 0.000893
2R:4611900-4612050:minus -22.1122 1.68807 1.51376 3.62385 -18.9796 -8.45918 -15.2661 5.5 -0.284404
0.00304 0.00563 0.00689 0.00673 0.0155 0.00111 0.00946 0.00209 0.0207
2R:4611900-4612050:plus -32.8571 0.697248 5.58716 2.72477 -18.1939 -0.173469 -21.7248 8.75 2.16514
0.0244 0.00807 0.00139 0.0102 0.0086 0.000456 0.0281 0.000374 0.00879
2R:4613020-4613170:minus -32.8061 -2.66972 -4.16514 1.91743 -27.5714 -9.86735 -17.6147 3.52778 -1.18349
0.0237 0.0235 0.0397 0.015 0.0357 0.00391 0.0141 0.00486 0.0276
2R:4613020-4613170:plus -23.8163 0.972477 0.366972 6.33945 -18.7551 -9.42857 -9.73394 4.58333 -0.972477
0.00682 0.00731 0.0103 0.00196 0.0144 0.0027 0.00293 0.00315 0.0259
2R:4778920-4779070:minus -4.29592 -2.21101 3.3211 0.908257 -9.20408 -8.72449 -1.93578 -2.45833 7.84404
0.000135 0.0206 0.00351 0.0211 0.00262 0.00169 0.000438 0.0337 0.000701
2R:4778920-4779070:plus -31.7041 1.6422 0.834862 3.14679 -18.3776 -19.2347 -23.3028 10 -0.220183
0.0146 0.00573 0.00875 0.00847 0.00926 0.018 0.0369 0.000164 0.0201
2R:4781060-4781210:minus -33.2959 0.605505 -1.89908 7.34862 -18.4184 -8.60204 -9.85321 4.68056 -2.6055
0.0279 0.00834 0.021 0.000998 0.0101 0.00151 0.00299 0.00302 0.04
2R:4781060-4781210:plus -29.3265 3.97959 0.807339 3.3578 20.4694 0.0510204 -12.9358 3.15278
1.52294 0.00837 0.000669 0.00884 0.00767 1.65e-07 0.000349 0.0057 0.00563 0.0117
2R:4787720-4787870:minus -14.7143 13.7431 2.22936 6.56881 -16.4898 -9.20408 -18.7156 1.75 -1.43119
0.00154 0.0000115 0.00531 0.0017 0.00312 0.00225 0.0167 0.00941 0.029
2R:4787720-4787870:plus -30.5102 -2.69725 4.40367 0.00917431 -17.1531 -19.051 -12.6422 0.652778
1.54128 0.00947 0.0237 0.00228 0.0269 0.00364 0.0165 0.00533 0.0136 0.0115
2R:4789040-4789190:minus -21.7041 7.2844 5.33945 9.33028 -26.9286 -18.0102 -13.4037 0.958333 4.91743
0.00231 0.000559 0.00154 0.000374 0.0214 0.00812 0.00634 0.0123 0.00321
2R:4789040-4789190:plus -24 2.44037 0.724771 1.3578 -18.4184 -0.204082 -19.1835 6.77778 -2.2844

0.00719 0.00427 0.00909 0.0186 0.0101 0.000463 0.018 0.00113 0.0361
2R:4789380-4789530:minus -22.2653 6.66972 0.59633 4.20183 -8.60204 -19.1224 2.63303 2.44444 0.908257
0.00331 0.000767 0.00949 0.00533 0.00125 0.0175 0.000134 0.00734 0.0147
2R:4789380-4789530:plus -24.4898 1.46789 4.33945 2.02752 -27.0102 -18.9388 -27.0183 -2.55556 0.972477
0.00807 0.0061 0.00234 0.0141 0.0237 0.0154 0.0606 0.0345 0.0144
2R:4794420-4794570:minus -14.3265 7.33028 1.16514 -0.412844 -8.37755 -18.0816 -15.3486 1.88889
-6.47706 0.0013 0.000546 0.0078 0.0304 0.00106 0.00934 0.00961 0.00896 0.1
2R:4794420-4794570:plus -30.8878 13.5306 -1.42202 3.51376 -26.2347 -8.65306 -19.8165 -2.77778 -6.05505
0.0103 0.0000206 0.0182 0.00709 0.0177 0.00156 0.02 0.0365 0.09
2R:4794920-4795070:minus -50.3265 -2.53211 1.77982 1.45872 -27.4898 -10.0204 -23.3119 2.08333 -2.05505
0.13 0.0226 0.00627 0.0179 0.0336 0.00399 0.0369 0.00837 0.0338
2R:4794920-4795070:plus -30.398 2.97248 1.16514 2.33945 -9.23469 0.27551 -8.25688 8.11111 0.146789
0.00901 0.00351 0.0078 0.0122 0.00277 0.000257 0.00222 0.000547 0.0178
2R:4801420-4801570:minus -23.9592 4.52294 1.99083 9.41284 -18.1531 -8.30612 -15.2294 0.347222 5.01835
0.00708 0.00197 0.0058 0.000354 0.00829 0.000973 0.00939 0.015 0.00309
2R:4801420-4801570:plus -12.3776 2.09174 1.20183 6.09174 -16.8163 -18.5 -6.40367 5.45833 1.34862
0.000403 0.00485 0.0077 0.00235 0.00323 0.0131 0.00146 0.00214 0.0125
2R:4801980-4802130:minus -22 -1.99083 -3.45872 1.77982 -18.9796 0.316327 -21.9174 2.26389 4.13761
0.00291 0.0194 0.0328 0.0158 0.0155 0.000243 0.0291 0.00784 0.00408
2R:4801980-4802130:plus -32 0.678899 2.52294 1.51376 -8.64286 -18.5 -5.18349 -0.0555556 1.27523
0.0171 0.00813 0.00476 0.0175 0.00139 0.0131 0.00105 0.017 0.013
2R:4810180-4810330:minus -31.6224 -3.74312 6.23853 1.3578 -18.449 -17.5 -5.52294 4.22222
0.00917431 0.0137 0.0316 0.00104 0.0186 0.0107 0.00599 0.00115 0.00367 0.0184
2R:4810180-4810330:plus -31.4694 2.7156 0.926606 5.6422 10.9898 -19.4184 -10.3028 0.319444 4.45872
0.0124 0.00386 0.00848 0.00325 3.28e-06 0.0198 0.00326 0.0151 0.00366
2R:4813080-4813230:minus -31.8878 -1.11009 7.92661 2.19266 -16.4898 -8.72449 -16.6147 6.20833 1.27523
0.0164 0.0148 0.000455 0.0131 0.00312 0.00169 0.012 0.0015 0.013
2R:4813080-4813230:plus -22.7755 6.84404 1.90826 7 -17.1837 -8.53061 -10.1468 7.33333 -0.651376
0.00412 0.000705 0.00598 0.00132 0.00366 0.00136 0.00316 0.000844 0.0235
2R:4815620-4815770:minus -32.2551 -7.88073 1.72477 -0.688073 -17.4898 -35.1122 -13.5413 3.52778
0.614679 0.0184 0.0894 0.0064 0.0333 0.0053 0.0748 0.00654 0.00486 0.0158
2R:4815620-4815770:plus -23.1122 1.97248 5.50459 6.57798 1.87755 0.979592 -20.4128 0.597222 7.84404
0.0053 0.00507 0.00144 0.00167 0.0000492 0.000136 0.0221 0.0139 0.000701
2R:482740-482890:minus -23.7449 8.62385 -0.394495 5.57798 -17.4184 -17.9388 -26.1101 -5.70833
-6.73394 0.00674 0.000273 0.0132 0.0034 0.00457 0.00738 0.0542 0.0717 0.107
2R:482740-482890:plus -15.0102 7.27523 -2.9633 4.0367 -9.23469 -18.1633 -17.9358 -2.01389 -4.29358
0.00162 0.000561 0.0285 0.00578 0.00277 0.01 0.0148 0.0299 0.06
2R:483120-483270:minus -31.7755 -2.33028 3.33028 -1.70642 -18.4184 -9.65306 -21.422 4.11111 3.07339
0.0157 0.0214 0.00349 0.0462 0.0101 0.0034 0.0266 0.00384 0.00683
2R:483120-483270:plus -30.6327 -0.477064 1.94495 2.09174 -26.5306 -26.602 -19.5321 8.31944 4.49541
0.00968 0.0121 0.0059 0.0137 0.0185 0.0248 0.019 0.000485 0.00363
2R:4834780-4834930:minus -31.2857 0.0458716 2.16514 -0.100917 11.6531 -36.2245 -13.9725 4.97222
7.62385 0.0113 0.0101 0.00544 0.0277 1.55e-06 0.0923 0.00721 0.00266 0.000941
2R:4834780-4834930:plus -23 -2.56881 7.74312 4.17431 -17.7143 -18.3061 -11.9817 -2.95833 -3.05505
0.00498 0.0229 0.000501 0.00537 0.00627 0.0114 0.00459 0.0382 0.0452
2R:4836820-4836970:minus -23.7755 1.29358 4.69725 3.81651 -17.1531 -27.6327 -17.4679 -0.458333
11.3853 0.00678 0.00651 0.00202 0.00626 0.00364 0.043 0.0138 0.0192 0.0000815
2R:4836820-4836970:plus -22.9592 1.11927 -3.25688 5.48624 -28.602 -26.8265 -11.7064 7.30556 -0.761468
0.00475 0.00693 0.031 0.00357 0.0631 0.0285 0.00433 0.000857 0.0244
2R:4838540-4838690:minus -31.5918 1.07339 1.05505 4.07339 -17.3367 -0.173469 -19.9358 -6.55556
-6.33945 0.0136 0.00705 0.00811 0.00562 0.00391 0.000456 0.0204 0.085 0.0966
2R:4838540-4838690:plus -31.2959 8.21101 4 2.93578 -28.2347 0.602041 -14.6147 -2.94444 3.54128
0.0113 0.00034 0.00268 0.00927 0.051 0.000188 0.00829 0.0381 0.0055
2R:4839100-4839250:minus -30.898 8.52294 13.6422 6.00917 -18.7143 -9.7551 -22.844 8.5 2.11009

0.0103 0.000288 0.0000116 0.00255 0.0136 0.00358 0.0342 0.000436 0.00898
2R:4839100-4839250:plus -23.449 7.69725 13.9541 7.2844 -8.96939 -9.23469 -9.61468 1.31944 1.57798
0.00631 0.00045 7.63e-06 0.00105 0.00226 0.00238 0.00287 0.0109 0.0114
2R:4839680-4839830:minus -30.6633 4.10092 -3.95413 5.57798 -18.4184 -17.2041 -17.7431 4.91667 2.11927
0.00976 0.00231 0.0375 0.0034 0.0101 0.00507 0.0144 0.00272 0.00889
2R:4839680-4839830:plus -30.1429 -0.0183486 0.100917 6.48624 20.4694 -18.0102 -20.1468 -2.18056
1.46789 0.00866 0.0104 0.0112 0.00177 1.65e-07 0.00812 0.0211 0.0313 0.0119
2R:4975220-4975370:minus -50.0714 -1.05505 -1.06422 5.49541 -27.4898 -18.0816 -19.0092 6.04167 -2.36697
0.122 0.0146 0.0163 0.00355 0.0336 0.00934 0.0175 0.00162 0.0371
2R:4975220-4975370:plus -23.2245 14.055 0.293578 1.78899 -26.9694 -17.9796 -4.06422 2.40278 0.743119
0.00576 7.8e-06 0.0105 0.0158 0.023 0.00764 0.00076 0.00746 0.0153
2R:4980380-4980530:minus -21.551 -0.633028 5.18349 8.81651 -18.4184 -18.0816 -11.0275 1.31944
11.3853 0.00205 0.0127 0.00165 0.000501 0.0101 0.00934 0.00375 0.0109 0.0000815
2R:4980380-4980530:plus -32.6224 1.04587 1.46789 7.72477 -26.9694 -9.79592 -23.1193 9.19444 7.16514
0.0211 0.00712 0.007 0.000681 0.023 0.00377 0.0358 0.000283 0.00122
2R:4982700-4982850:minus -41.9592 -0.788991 -3.02752 -0.348624 -17.7449 -10.0918 -13.3119 5.625
3.46789 0.0679 0.0134 0.0291 0.0296 0.0063 0.00404 0.00621 0.00198 0.00582
2R:4982700-4982850:plus -31 3.90816 -3.23853 3.57798 1.14286 -9.68367 -18.2569 5.16667 7.53211
0.0105 0.00102 0.0308 0.00696 0.000113 0.00342 0.0155 0.00244 0.000968
2R:4990840-4990990:minus -31.2143 -0.678899 5.61468 3.11009 -17.1939 -17.8571 -13.789 2.09722
3.78899 0.0111 0.0129 0.00137 0.00862 0.00377 0.00719 0.00692 0.00832 0.00487
2R:4990840-4990990:plus -33.8469 -2.70642 5.11927 2.00917 -18.1939 -27.6735 -21.4587 6.72222 -4.33028
0.03 0.0238 0.00169 0.0142 0.0086 0.0443 0.0267 0.00116 0.0607
2R:4993960-4994110:minus -40.0714 -3.92661 3.6789 6.36697 -18.3878 0.755102 -22.1009 9.94444 -3.94495
0.0359 0.0332 0.00305 0.00193 0.00931 0.000175 0.03 0.000171 0.0549
2R:4993960-4994110:plus -14.7143 1.66055 -1.85321 5.57798 -27.4592 -18.2041 -15.5872 8.84722 -7.66972
0.00154 0.00569 0.0207 0.0034 0.0329 0.0103 0.0101 0.000353 0.128
2R:4997060-4997210:minus -21.8878 0.486239 3.56881 8.61468 -17.0816 -17.0102 -4.11009 -0.916667
0.12844 0.00258 0.00871 0.00318 0.000552 0.00334 0.00453 0.00077 0.022 0.0179
2R:4997060-4997210:plus -22.8469 4.40367 0.477064 3.94495 -7.93878 -9.45918 -11.1835 2.61111 4.80734
0.00424 0.00206 0.00989 0.00595 0.000767 0.00279 0.00387 0.00691 0.00334
2R:4998240-4998390:minus -33.0714 4.15596 6.54128 -2.30275 -17.4898 -16.7857 -24.1376 2.45833 -4.42202
0.0267 0.00227 0.000901 0.0564 0.0053 0.00426 0.0419 0.00731 0.0626
2R:4998240-4998390:plus -32.4694 1.53211 3.46789 2.99083 -17.4898 -18.0102 -8.6422 -1.66667 3.31193
0.0196 0.00596 0.00331 0.00899 0.0053 0.00812 0.00239 0.0272 0.00618
2R:5001640-5001790:minus -12.0714 -1.66055 -2.61468 2.0367 2.14286 -17.051 10.6422 -0.347222
1.90826 0.000318 0.0176 0.0259 0.0141 0.0000316 0.00479 0.0000102 0.0186 0.00974
2R:5001640-5001790:plus -34 0.550459 9.01835 7 10.7245 -17.0816 -19.3119 -0.902778 2.53211
0.0307 0.00851 0.000251 0.00132 5.2e-06 0.00487 0.0183 0.0219 0.00791
2R:5003560-5003710:minus 5.14286 0.844037 1.22018 3.3578 -18.6837 -8.5 -19.844 -2.34722 -4.75229
0.0000147 0.00766 0.00765 0.00767 0.0126 0.00126 0.02 0.0327 0.0683
2R:5003560-5003710:plus -22.9286 9.69725 -2.15596 4.19266 -17.9286 -18.6327 -15.6789 -3.43056 1.08257
0.0046 0.000155 0.0226 0.00536 0.0071 0.0137 0.0102 0.043 0.0139
2R:5003980-5004130:minus -24.6327 -0.0458716 2.87156 1.91743 -27.0102 -9.23469 -13.9174 -4.61111
-0.366972 0.00824 0.0105 0.00417 0.015 0.0237 0.00238 0.00712 0.0566 0.021
2R:5003980-5004130:plus -21.7347 -5.06422 -2.6422 5.98165 -18.5612 -8.27551 1.63303 3.44444 5.84404
0.00235 0.0449 0.0261 0.00258 0.0119 0.000919 0.000184 0.00502 0.00202
2R:5016340-5016490:minus -21.6224 5.00917 0.550459 7.80734 -17.7959 -18.1224 -21.4404 3.33333 -3.29358
0.00213 0.00162 0.00965 0.00063 0.00678 0.00988 0.0266 0.00525 0.0474
2R:5016340-5016490:plus -32.5918 -3.52294 7.75229 3.69725 -18.6735 -17.1224 -17.0459 5.11111 -4.2844
0.021 0.0297 0.000499 0.00665 0.0123 0.00499 0.0129 0.0025 0.0597
2R:5017340-5017490:minus -24.0408 -1.34862 7.55046 1.33028 -18.1531 -26.898 -12.2202 3.45833 1.59633
0.00726 0.016 0.000552 0.0188 0.00829 0.0302 0.00484 0.005 0.0113
2R:5017340-5017490:plus -32.0408 0.211009 0.844037 -0.642202 -9.12245 -8.79592 -10.1468 6.58333

-1.68807 0.0174 0.00959 0.00872 0.0328 0.00238 0.0019 0.00316 0.00124 0.0308
2R:5017820-5017970:minus -32 -2.17431 7.30275 6.84404 -8.93878 -19.0102 -16.633 -3.33333 1.29358
0.0171 0.0204 0.000626 0.0014 0.00216 0.0159 0.0121 0.042 0.0128
2R:5017820-5017970:plus -32.2449 1.74312 -0.46789 3.7156 -17.8163 -19.0816 -16.9633 -1.93056 5.65138
0.0183 0.00552 0.0135 0.00655 0.00682 0.017 0.0127 0.0293 0.00228
2R:5018980-5019130:minus -21.5918 -0.220183 6.6789 5.57798 -17.3469 -26.6735 -15.4037 -5.44444
2.89908 0.00209 0.0111 0.000844 0.0034 0.00394 0.0256 0.00972 0.0678 0.00722
2R:5018980-5019130:plus -22.9592 -0.146789 6.63303 2.75229 -7.93878 -19.4184 -16.8899 -2.04167
0.229358 0.00475 0.0108 0.000861 0.0101 0.000767 0.0198 0.0126 0.0301 0.0173
2R:5019380-5019530:minus -31.7143 1.46789 -1.47706 0.201835 10.6531 0.0918367 -23.1927 8.63889
-2.90826 0.0146 0.0061 0.0185 0.0255 0.0000102 0.000323 0.0362 0.000401 0.0435
2R:5019380-5019530:plus -24.5612 -1.92661 4.22018 1.22936 -26.9694 -9.23469 -14.789 6.31944 -2.26606
0.00819 0.019 0.00245 0.0193 0.023 0.00238 0.00859 0.00142 0.0359
2R:5019840-5019990:minus -14.4796 1.89908 -2.59633 -1 -8.86735 -9.45918 -22.4771 2.72222 1.72477
0.00142 0.00521 0.0257 0.0367 0.00173 0.00279 0.0321 0.00663 0.0106
2R:5019840-5019990:plus -30.1735 -0.238532 3.85321 7.47706 10.7245 -7.79592 -0.862385 2.58333
0.348624 0.00869 0.0112 0.00284 0.00085 5.2e-06 0.000723 0.000344 0.00698 0.0167
2R:5025340-5025490:minus -31.1429 1.43119 0.146789 3.13761 -18.3776 -8.72449 -6.06422 8.41667 -3.66055
0.0108 0.00619 0.011 0.00854 0.00926 0.00169 0.00133 0.000458 0.0513
2R:5025340-5025490:plus -23.4592 5 11.055 3.90826 -18.1837 -17.2755 -19.8532 1.30556 -3.08257
0.00631 0.00163 0.0000772 0.00603 0.00835 0.00544 0.0201 0.011 0.0455
2R:5026260-5026410:minus -24.2653 -1.6422 3.63303 3.23853 -27.2347 -17.3469 -23.5413 1.73611 7.58716
0.00773 0.0175 0.00311 0.00815 0.0283 0.0058 0.0383 0.00946 0.000954
2R:5026260-5026410:plus -22.8878 4.22936 1.20183 2.93578 -16.7143 -26.7551 -5.84404 -2.13889 -1.27523
0.00441 0.0022 0.0077 0.00927 0.00319 0.0263 0.00126 0.0309 0.0282
2R:5031320-5031470:minus -14.449 6.92661 4.11927 4.11009 -18.7143 -26.3367 -18.7064 0.472222 3.55963
0.0014 0.000674 0.00255 0.00553 0.0136 0.0221 0.0167 0.0144 0.00545
2R:5031320-5031470:plus -31.1735 -0.761468 9.18349 4.07339 1.17347 -8.57143 -10.9817 1.58333
-0.0275229 0.0109 0.0133 0.000228 0.00562 0.000101 0.00149 0.00372 0.00997 0.0186
2R:5032720-5032870:minus -30.3673 -1.10092 2.6789 6.00917 2.21429 -0.204082 -16.1009 0.347222
-1.52294 0.00897 0.0148 0.00449 0.00255 0.0000277 0.000463 0.011 0.015 0.0295
2R:5032720-5032870:plus -4.04082 -2.3211 8.3211 5.31193 -26.8571 -8.79592 -13.6606 5.77778 -1.11009
0.000123 0.0213 0.000368 0.00393 0.0198 0.0019 0.00672 0.00184 0.0269
2R:5052520-5052670:minus -31.6327 3.68367 7.2844 10.7615 -27.3061 -18.0816 -20.7982 2.44444 1.16514
0.0138 0.00144 0.000632 0.0000826 0.0315 0.00934 0.0237 0.00734 0.0135
2R:5052520-5052670:plus -33.7755 3.05505 0.706422 4.61468 -16.3776 -16.2755 -14.2018 2.48611 7.22018
0.0297 0.00341 0.00914 0.00475 0.003 0.00413 0.00758 0.00723 0.00119
2R:5052880-5053030:minus -22.7041 -5.42202 0.963303 7.63303 -17.7857 -27.3367 -10.8899 6.23611 5.58716
0.00395 0.0493 0.00837 0.000745 0.00676 0.0347 0.00365 0.00148 0.00237
2R:5052880-5053030:plus -33.0714 3.90816 0.788991 2.98165 -27.3367 -8.79592 -15.3028 2.66667 1.44037
0.0267 0.00102 0.00889 0.00905 0.0317 0.0019 0.00953 0.00677 0.012
2R:5053220-5053370:minus -31.8163 13.3878 -3.65138 2.31193 -8.93878 -18.1224 -22.0183 5.91667 -2.27523
0.016 0.0000869 0.0345 0.0123 0.00216 0.00988 0.0296 0.00172 0.036
2R:5053220-5053370:plus -15.0102 13.4592 -1.74312 2.20183 -18.4898 -18.0102 -20.2752 6.40278 3.88991
0.00162 0.000064 0.02 0.013 0.0116 0.00812 0.0216 0.00136 0.00467
2R:5054960-5055110:minus -13.6735 0.321101 -0.550459 -0.844037 -19.0102 9.60204 -18.0275 4.44444
2.73394 0.00103 0.00922 0.0139 0.035 0.0157 0.0000248 0.015 0.00334 0.00756
2R:5054960-5055110:plus -33.3061 4.83486 3.81651 4.13761 -17.7143 9.08163 -18.3394 5.58333 -3.94495
0.0279 0.00174 0.00289 0.00546 0.00627 0.0000431 0.0157 0.00202 0.0549
2R:506440-506590:minus -33.8878 0.53211 0.522936 3.18349 -28.5306 -17.2755 -21.578 7.48611 9.61468
0.0302 0.00856 0.00974 0.00837 0.061 0.00544 0.0273 0.000777 0.00029
2R:506440-506590:plus -32.9694 0.275229 -2.72477 11.2936 -18.4898 -0.0204082 -19.6881 0.833333 -8.11009
0.0254 0.00938 0.0267 0.0000139 0.0116 0.000411 0.0195 0.0128 0.139
2R:5064740-5064890:minus -23.1837 11.1193 0.53211 0.642202 -18.4184 -10.0204 -23.6881 2.65278 -6.0367

0.00553 0.000076 0.00971 0.0227 0.0101 0.00399 0.0392 0.0068 0.0896
2R:5064740-5064890:plus -13.449 14.5596 3.72477 5.57798 -17.3469 -8.23469 -12.4495 -0.263889
-1.22936 0.000914 4.47e-06 0.003 0.0034 0.00394 0.000875 0.0051 0.0181 0.0279
2R:5073840-5073990:minus -22.0816 0.669725 0.0733945 9.08257 -25.9694 -8.72449 -18.1468 0.0972222
-0.211009 0.003 0.00815 0.0113 0.000444 0.017 0.00169 0.0153 0.0162 0.02
2R:5073840-5073990:plus -23.7857 -1.44037 -1.73394 5.06422 -17.4898 -0.0204082 -15.0734 3.04167
1.16514 0.00679 0.0164 0.02 0.00433 0.0053 0.000411 0.00911 0.00587 0.0135
2R:5079540-5079690:minus -13.4082 3.97959 2.66972 3.33945 10.3878 -8.5 -12.6514 5.18056 3.88073
0.000867 0.000669 0.0045 0.00774 0.0000174 0.00126 0.00534 0.00242 0.00475
2R:5079540-5079690:plus -41.0306 5.99083 3.59633 4.12844 -8.93878 -9.86735 -9.6055 7.73611 -0.963303
0.0464 0.00106 0.00315 0.00549 0.00216 0.00391 0.00286 0.000677 0.0258
2R:5094920-5095070:minus -3.66327 -1.0367 4.07339 3.69725 -17.3776 -9.72449 -9.33028 7.29167 -3.05505
0.0000935 0.0145 0.0026 0.00665 0.00419 0.00352 0.00272 0.000863 0.0452
2R:5094920-5095070:plus -22.5918 0.981651 11.7064 7.2844 -8.82653 -8.42857 -7.2844 -4.52778 5.18349
0.00365 0.00729 0.0000523 0.00105 0.00163 0.00104 0.0018 0.0556 0.00295
2R:5139480-5139630:minus -34.0714 1.91743 4.12844 1.9633 -8.93878 -18.0102 -18.3303 -3.94444 1.16514
0.031 0.00518 0.00254 0.0146 0.00216 0.00812 0.0157 0.0486 0.0135
2R:5139480-5139630:plus -12.1531 2.58716 15.0092 7.37615 -9.5 -26.8265 -9.36697 2.66667 -1.6422
0.000352 0.00404 1.55e-06 0.000951 0.00293 0.0285 0.00274 0.00677 0.0304
2R:5162480-5162630:minus -31.4082 -4.98165 0.174312 9.7156 -26.9388 -16.7143 -16.9725 -0.805556
-2.90826 0.0119 0.0439 0.0109 0.000242 0.0215 0.00419 0.0128 0.0213 0.0435
2R:5162480-5162630:plus -3.70408 1.3211 -1.63303 2.92661 -18.1531 -36.8571 -4.93578 0.958333 11.8073
0.000101 0.00644 0.0194 0.00931 0.00829 0.121 0.000975 0.0123 0.0000538
2R:5171740-5171890:minus -32.7041 7.66055 7.6422 3.78899 -27.3061 -27.3367 -7.93578 7.69444 5.70642
0.0222 0.000457 0.000528 0.00632 0.0315 0.0347 0.00208 0.000693 0.00222
2R:5171740-5171890:plus -31.7041 0.513761 2.72477 2.9633 -17.7143 -9.57143 -18.0917 -0.958333
1.38532 0.0146 0.00862 0.00441 0.00914 0.00627 0.00326 0.0152 0.0223 0.0123
2R:5172500-5172650:minus -30.7143 -3.3578 1.44037 2.78899 20.4694 -8.5 -25.3028 3.23611 -3.38532
0.00993 0.0284 0.00707 0.00992 1.65e-07 0.00126 0.049 0.00545 0.0483
2R:5172500-5172650:plus -32.3367 4.94495 6.31193 3.59633 -18.9796 -17.5 -21.9633 8.58333 3.6789
0.0187 0.00166 0.001 0.00693 0.0155 0.00599 0.0293 0.000415 0.00506
2R:5174060-5174210:minus -33 4.51376 7.61468 2.66972 -19.051 -19.0816 -23.7982 3.33333 -4.24771
0.0258 0.00198 0.000536 0.0106 0.0162 0.017 0.0398 0.00525 0.0591
2R:5174060-5174210:plus -33.8878 -3.44037 9.95413 3.87156 -7.60204 -17.2755 -19 2.93056 3.88073
0.0302 0.0291 0.000146 0.00618 0.000466 0.00544 0.0175 0.00612 0.00475
2R:5179140-5179290:minus -42.7755 1.33028 1.89908 2.82569 -8.63265 0.0510204 -29.7706 4.66667
-3.42202 0.0854 0.00642 0.006 0.00976 0.0013 0.000349 0.088 0.00304 0.0488
2R:5179140-5179290:plus -23.1837 13.3878 1.76147 4.22018 -17.7857 -17.2755 -13.1376 2.375 1.74312
0.00553 0.0000869 0.00631 0.00531 0.00676 0.00544 0.00597 0.00753 0.0105
2R:5190620-5190770:minus -13.1122 0.275229 -3.85321 1.91743 -18.7143 -9.7551 -11.4587 2.93056 -0.394495
0.000632 0.00938 0.0365 0.015 0.0136 0.00358 0.0041 0.00612 0.0212
2R:5190620-5190770:plus -22.2551 7.58716 3.62385 6.83486 -17.1531 -26.6327 -7.65138 3.125 -1.20183
0.00327 0.000476 0.00312 0.00142 0.00364 0.0251 0.00195 0.00569 0.0276
2R:5195880-5196030:minus -23.8469 1.3211 -0.862385 7.16514 -7.33673 -36.9592 2.48624 1.375
7.15596 0.00685 0.00644 0.0153 0.00116 0.000388 0.124 0.000141 0.0107 0.00124
2R:5195880-5196030:plus -30.3265 -4.44037 0.0458716 1.91743 -7.56122 -8.42857 -9.69725 -6.25
-4.89908 0.0089 0.0381 0.0114 0.015 0.000419 0.00104 0.00291 0.08 0.0712
2R:5196160-5196310:minus -4.59184 8.33945 7.3945 4.20183 -18.1939 -27.6735 1.87156 -3.84722 2.84404
0.000166 0.000317 0.000597 0.00533 0.0086 0.0443 0.000172 0.0475 0.00736
2R:5196160-5196310:plus -22.1531 -2.49541 6.55046 3.76147 -6.67347 -9.09184 -20.2018 1.58333 1.48624
0.0031 0.0224 0.000896 0.00645 0.000359 0.00219 0.0213 0.00997 0.0118
2R:5286220-5286370:minus -12.1122 7.44037 3.83486 2.88991 -17.4898 -8.42857 -12.0275 -3.06944 -2.62385
0.000336 0.000515 0.00287 0.00942 0.0053 0.00104 0.00464 0.0393 0.0402
2R:5286220-5286370:plus -41.5612 -1.95413 -1.22018 4.07339 -9.20408 -18.5714 -17.2752 5 3.87156

0.0609	0.0192	0.0171	0.00562	0.00262	0.0136	0.0134	0.00263	0.00477
2R:5287660-5287810:minus	-32	6.94495	-2.51376	4.11009	10.9898	10.7959	-23.8899	9.95833	-2.42202
0.0171	0.000669	0.0251	0.00553	3.28e-06	2.99e-06	0.0404	0.000169	0.0378
2R:5287660-5287810:plus	-23.3673	7.47706	-1.24771	2.22018	-27.5306	-8.93878	-15.7982	2.91667	3.20183
0.00617	0.000505	0.0172	0.0129	0.0351	0.00201	0.0105	0.00616	0.0065
2R:5297040-5297190:minus	-31.8469	0.431193	2.7156	4.3945	-18.4898	-27.9694	-13.3394	5.02778	7.83486
0.0162	0.00887	0.00442	0.00499	0.0116	0.0547	0.00625	0.00259	0.000712
2R:5297040-5297190:plus	-32.7041	-0.926606	0.431193	7.73394	-27.2347	-19.0816	-13.9725	5.18056
11.4771	0.0222	0.014	0.01	0.000669	0.0283	0.017	0.00721	0.00242	0.0000679
2R:5311540-5311690:minus	-34.1531	-1.61468	-4.33945	1.25688	-18.051	-9.02041	-20.8716	0.458333	-9.07339
0.0314	0.0173	0.0416	0.0192	0.00746	0.00213	0.024	0.0145	0.168
2R:5311540-5311690:plus	-20.6633	-0.247706	10.055	2.56881	-17.449	0.0510204	-19.8073	3.41667
0.0275229	0.00173	0.0112	0.000138	0.0111	0.00484	0.000349	0.0199	0.00508	0.0183
2R:5313920-5314070:minus	-20.7041	-1.73394	5.61468	1.77982	-26.9694	-27.602	-2.55963	-3.69444	7.6422
0.00175	0.018	0.00137	0.0158	0.023	0.0416	0.000508	0.0458	0.000893
2R:5313920-5314070:plus	-13.3673	-0.174312	1.49541	2.53211	-26.1939	-17.7143	-6.42202	-2.125
-0.192661	0.000813	0.0109	0.00694	0.0113	0.0175	0.00634	0.00146	0.0308	0.0198
2R:5318220-5318370:minus	-4.36735	0.311927	1.79817	9.23853	-18.6837	-8.53061	-3.02752	-6.75	4.14679
0.000143	0.00925	0.00623	0.000424	0.0126	0.00136	0.000572	0.0883	0.00406
2R:5318220-5318370:plus	-22.8878	3.80734	2.41284	1.08257	-17.449	-18.2755	-10.3761	-2.58333	7.15596
0.00441	0.00259	0.00496	0.0201	0.00484	0.0112	0.0033	0.0348	0.00124
2R:5318820-5318970:minus	-24.5204	6.44037	2.29358	6.29358	-18.6837	-26.7143	-20.7523	-1.13889	-1.97248
0.00813	0.000858	0.00518	0.00202	0.0126	0.0257	0.0235	0.0235	0.033
2R:5318820-5318970:plus	-22.898	-3.65138	-0.376147	4.23853	-26.3061	0.0510204	-17.7615	5.875
3.92661	0.00442	0.0308	0.0131	0.00522	0.0181	0.000349	0.0144	0.00176	0.00461
2R:5323180-5323330:minus	-30.9694	-0.954128	8.01835	0.431193	-27.9694	-6.72449	-6.29358	5.41667
1.93578	0.0104	0.0141	0.000433	0.0241	0.0437	0.000544	0.00142	0.00218	0.00969
2R:5323180-5323330:plus	-32.5816	2.36697	4.24771	2.40367	-17.1939	-19.051	-16.3303	3.5	5.12844
0.0208	0.00439	0.00243	0.0119	0.00377	0.0165	0.0115	0.00491	0.00301
2R:5326760-5326910:minus	-23.3061	4.89908	3.10092	3.33028	-19.051	-8.53061	-17.844	5.09722	-0.715596
0.00606	0.0017	0.00382	0.00778	0.0162	0.00136	0.0146	0.00251	0.0241
2R:5326760-5326910:plus	-31.5918	3.57143	-0.53211	3.98165	-18.0102	-8.93878	-20.1651	1.38889	7.63303
0.0136	0.00175	0.0138	0.00582	0.00732	0.00201	0.0212	0.0107	0.000928
2R:5327300-5327450:minus	-32	-3.45872	3.01835	11.1927	-18.1939	-18.0816	-19.6606	2.26389	6.11927
0.0171	0.0292	0.00394	0.0000254	0.0086	0.00934	0.0194	0.00784	0.00178
2R:5327300-5327450:plus	-23.4184	-2.19266	1.21101	9.59633	-26.6327	-9.45918	-30.8165	1.80556	-5.99083
0.00624	0.0205	0.00768	0.000275	0.0188	0.00279	0.102	0.00923	0.0886
2R:5328320-5328470:minus	15.102	4.90826	6.66972	3.6055	-26.5918	-16.9796	-8.81651	0.194444	0.963303
4.37e-07	0.00169	0.000847	0.00686	0.0186	0.0044	0.00247	0.0157	0.0145
2R:5328320-5328470:plus	-21.8469	2.79817	-0.110092	6.11009	-17.3061	-16.9388	-4.00917	-7.06944
-6.18349	0.00247	0.00374	0.012	0.00233	0.00389	0.00433	0.000748	0.0938	0.093
2R:5328980-5329130:minus	-20.9592	3.46789	5.31193	3.59633	-7.67347	-9.42857	-16.1468	-1.70833	4.30275
0.00184	0.00293	0.00156	0.00693	0.00055	0.0027	0.0111	0.0275	0.00388
2R:5328980-5329130:plus	-22.5918	4.43119	2.42202	7.80734	-17.4184	-27.8265	-2.33945	-4.61111	11.8532
0.00365	0.00204	0.00495	0.00063	0.00457	0.0498	0.000481	0.0566	0.0000428
2R:5329540-5329690:minus	-24.1837	-2.31193	-2.77982	5.30275	-17.1122	-8.20408	-12.9817	0.972222	-2.05505
0.00751	0.0213	0.0271	0.00396	0.00341	0.000796	0.00576	0.0123	0.0338
2R:5329540-5329690:plus	-22.8571	-4.14679	6.10092	4.23853	-8.70408	1.27551	-17.6881	0.611111	-5.25688
0.00427	0.0352	0.0011	0.00522	0.00155	0.0000717	0.0143	0.0138	0.0768
2R:5332280-5332430:minus	-23.8163	1.58716	-1.82569	0.119266	-26.1939	-8.72449	-12.7706	-2.09722	-4.42202
0.00682	0.00584	0.0205	0.026	0.0175	0.00169	0.00549	0.0306	0.0626
2R:5332280-5332430:plus	-22.551	1.06422	3.66055	4.33945	-26.8571	-9.16327	-16.1284	4.31944	3.72477
0.00359	0.00707	0.00307	0.00509	0.0198	0.00223	0.0111	0.00352	0.00497
2R:5333320-5333470:minus	-31.8571	2.26606	-1.50459	5.30275	-27.4592	0.0510204	-19.9633	3	-5.25688

0.0163 0.00455 0.0186 0.00396 0.0329 0.000349 0.0205 0.00597 0.0768
2R:5333320-5333470:plus -40.1429 4.27523 6.79817 2.40367 -17.7143 -8.82653 -20.8257 -2.34722 -7.30275
0.0363 0.00217 0.000798 0.0119 0.00627 0.00191 0.0238 0.0327 0.12
2R:5333720-5333870:minus -22.9592 8.92661 -0.917431 1.38532 -17.8163 -19.1224 -5.11009 -0.791667
-0.715596 0.00475 0.000233 0.0156 0.0185 0.00682 0.0175 0.00102 0.0212 0.0241
2R:5333720-5333870:plus -21.4082 0.798165 6.89908 2.62385 -8.30612 -17.7143 -12.0092 0.125 3.20183
0.00198 0.00779 0.000762 0.0108 0.00097 0.00634 0.00462 0.0161 0.0065
2R:5428000-5428150:minus -32 6.57798 -2.11009 6.3578 -8.89796 0.387755 -18.6422 7.84722 -4.40367
0.0171 0.000803 0.0223 0.00195 0.00189 0.000217 0.0165 0.000636 0.0623
2R:5428000-5428150:plus -32.9592 4.02752 5.55963 3.21101 -18.2245 -27.9694 -16.4404 0.375 1.48624
0.0252 0.00238 0.0014 0.00822 0.00877 0.0547 0.0117 0.0149 0.0118
2R:5431680-5431830:minus -30.2143 0.229358 1.72477 7.27523 -18.4592 -9.02041 -10.2477 -0.819444
1.73394 0.0088 0.00953 0.0064 0.00109 0.0109 0.00213 0.00322 0.0214 0.0106
2R:5431680-5431830:plus -22.9592 -2.07339 3.26606 1.83486 -18.7959 -9.5 -15.7982 1.47222 0.440367
0.00475 0.0198 0.00358 0.0155 0.0148 0.00294 0.0105 0.0104 0.0164
2R:5434020-5434170:minus -3.04082 1.04587 -0.0550459 5.48624 -18.3776 -8.42857 -13.5138 2.76389
7.31193 0.0000423 0.00712 0.0118 0.00357 0.00926 0.00104 0.0065 0.00652 0.00112
2R:5434020-5434170:plus -14.4082 -3.52294 2.11927 2.92661 -16.8571 -6.5 -16.7064 -2.30556 2.07339
0.00136 0.0297 0.00553 0.00931 0.00324 0.000542 0.0122 0.0323 0.00906
2R:5434460-5434610:minus -21.6939 -4.53211 -1.55046 7.17431 -27.1531 -9.57143 -2.74312 -2.20833 10.0367
0.00226 0.039 0.0189 0.00115 0.0249 0.00326 0.000532 0.0315 0.000146
2R:5434460-5434610:plus -33.7755 1.46789 1.06422 8.80734 -17.4184 -27.602 -4.86239 1.33333 4.23853
0.0297 0.0061 0.00808 0.000518 0.00457 0.0416 0.000955 0.0109 0.00394
2R:5437660-5437810:minus -29.6531 1.55963 2.49541 5.88073 -18.0816 -36.4082 -10.8807 1.91667 7.02752
0.00846 0.0059 0.00481 0.00275 0.00759 0.105 0.00364 0.00888 0.00133
2R:5437660-5437810:plus -24.2245 -1.27523 4.56881 3.95413 -8.89796 -19.0102 -10.7523 1.29167 2.33028
0.00763 0.0156 0.00213 0.00593 0.00189 0.0159 0.00355 0.011 0.00836
2R:5438160-5438310:minus -22.0306 -0.93578 10.2477 2.66972 -27.5306 -18.5 -14.1101 -2.58333 1.73394
0.00293 0.014 0.000123 0.0106 0.0351 0.0131 0.00743 0.0348 0.0106
2R:5438160-5438310:plus -23.2653 1.90826 7.88991 2.56881 -17.8571 -8.79592 -20.8716 5.19444 1.27523
0.00593 0.00519 0.000464 0.0111 0.00698 0.0019 0.024 0.00241 0.013
2R:5439520-5439670:minus -31.6633 3.88991 5.22936 -0.110092 -8.26531 -36.3776 1.07339 3.19444
4.44037 0.0141 0.00251 0.00162 0.0277 0.000934 0.104 0.000216 0.00554 0.00367
2R:5439520-5439670:plus -22.8878 -0.0275229 1.73394 9.59633 -27.0408 -18.2755 -7.55046 -6.43056
0.486239 0.00441 0.0104 0.00638 0.000275 0.0242 0.0112 0.00191 0.0829 0.0163
2R:5440140-5440290:minus -30.4082 2.68807 4.73394 -0.541284 -17.1837 -17.9796 -22.6422 -4.73611
-3.68807 0.00908 0.0039 0.00199 0.0316 0.00366 0.00764 0.033 0.0582 0.0516
2R:5440140-5440290:plus 5.10204 3.90816 -2.3945 7.47706 -18.4184 -8.42857 -1.49541 -4.68056 2.58716
0.0000155 0.00102 0.0242 0.00085 0.0101 0.00104 0.000396 0.0575 0.00779
2R:5440820-5440970:minus -22.5102 3.58716 4.3945 2.70642 -26.2653 -27.4898 -14.5229 0.472222 7.83486
0.00354 0.0028 0.00229 0.0103 0.018 0.0363 0.00813 0.0144 0.000712
2R:5440820-5440970:plus -23.5918 0.238532 3.59633 6.56881 -17.3469 -17.0102 -21.2844 -5.40278 0.12844
0.00656 0.0095 0.00315 0.0017 0.00394 0.00453 0.0259 0.0672 0.0179
2R:5441600-5441750:minus -31.2143 -2.46789 -0.376147 6.84404 -17.3367 -18.0102 -22.4679 0.958333
1.54128 0.0111 0.0222 0.0131 0.0014 0.00391 0.00812 0.032 0.0123 0.0115
2R:5441600-5441750:plus -21.9694 3.6789 0.743119 2.55046 -27 -8.72449 -7.90826 3.25 5.18349 0.00282
0.00271 0.00903 0.0112 0.0233 0.00169 0.00206 0.00542 0.00295
2R:5442280-5442430:minus -30.7755 3.2844 -3.16514 2.00917 -19.3163 -17.2755 -12.0459 -0.736111
-1.01835 0.0102 0.00313 0.0302 0.0142 0.0165 0.00544 0.00466 0.0209 0.0263
2R:5442280-5442430:plus -13.4796 0.733945 0.119266 2.93578 -8.89796 -0.0204082 -2.86239 4.23611
7.63303 0.000935 0.00797 0.0111 0.00927 0.00189 0.000411 0.000548 0.00364 0.000928
2R:5442700-5442850:minus -23.8878 6.79817 -0.229358 1.89908 -26.9388 -18.7551 -13.8807 -0.541667
1.48624 0.00692 0.00072 0.0125 0.0151 0.0215 0.0141 0.00706 0.0197 0.0118
2R:5442700-5442850:plus -23.4796 0.697248 1.10092 5.45872 -26.8878 -18.051 -21.4128 1.63889 -0.504587

0.00634 0.00807 0.00798 0.00363 0.0199 0.00886 0.0265 0.00978 0.0221
2R:5452640-5452790:minus -33.0408 -3.80734 1.08257 5.50459 -17.7143 -8.64286 -22.1376 -1.69444 -0.440367
0.0264 0.0321 0.00803 0.00349 0.00627 0.00154 0.0302 0.0274 0.0216
2R:5452640-5452790:plus -30.9184 -1.51376 -0.486239 5.22018 -17.5612 -17.051 -7.04587 1.76389
5.90826 0.0103 0.0168 0.0136 0.00418 0.00544 0.00479 0.00171 0.00937 0.00198
2R:5453240-5453390:minus -29.102 3.7551 1.76147 6.12844 -18.6429 -9.53061 -15.3853 2.91667 -4.40367
0.00833 0.00122 0.00631 0.00231 0.0121 0.0031 0.00968 0.00616 0.0623
2R:5453240-5453390:plus -23.1531 3.84404 2.01835 5.49541 -17.449 -17.0816 -20.9633 1.93056
-2.77982 0.00542 0.00255 0.00574 0.00355 0.00484 0.00487 0.0244 0.00883 0.042
2R:5464960-5465110:minus -23.8163 -1.13761 3.6422 1.99083 -19.0102 -9.7551 -15.6422
1.66667 8.62385 0.00682 0.015 0.00309 0.0144 0.0157 0.00358 0.0102 0.00969 0.00048
2R:5464960-5465110:plus -24.2245 -3.76147 -3.93578 -1.77982 -8.0102 -19.0816 -16.7339
-1.54167 10.3486 0.00763 0.0317 0.0373 0.0474 0.000826 0.017 0.0123 0.0263 0.0000988
2R:5465800-5465950:minus -32.551 3.11009 3.93578 -0.165138 -17.4184 -27.5612 -12.055 2.75
7.42202 0.0207 0.00334 0.00275 0.0282 0.00457 0.0393 0.00467 0.00656 0.00106
2R:5465800-5465950:plus -22.9286 2.22018 1.48624 5.69725 -26.9694 -8.79592 -24.6422 -2.43056
-2.11009 0.0046 0.00463 0.00696 0.00314 0.023 0.0019 0.045 0.0334 0.0343
2R:5466420-5466570:minus -24.7041 4.05102 0.981651 7.3578 -17.3776 -8.79592 -9.22936
1.97222 -0.12844 0.00826 0.000335 0.00832 0.000965 0.00419 0.0019 0.00267 0.0087 0.0193
2R:5466420-5466570:plus -32.8571 8.18349 1.14679 9.51376 -17.6429 -17.2755 -16.1101 -1.19444
4.73394 0.0244 0.000345 0.00785 0.000292 0.00562 0.00544 0.0111 0.0239 0.00342
2R:5466820-5466970:minus -41.7449 7.24771 -0.0366972 3.19266 -36.3469 0.755102 -15.6789
5.73611 -3.52294 0.0646 0.00057 0.0117 0.00829 0.0752 0.000175 0.0102 0.00188 0.0499
2R:5466820-5466970:plus -31.551 -0.12844 -5.43119 4.10092 2.14286 19.6429 -14.8349 2 -0.33945
0.0134 0.0108 0.055 0.00554 0.0000316 7.32e-08 0.00868 0.00862 0.0209
2R:5472040-5472190:minus -21.8571 13.3878 4.89908 2.66972 -27.2347 -8.94898 -17.5688
1.05556 3.11009 0.00249 0.0000869 0.00185 0.0106 0.0283 0.00203 0.014 0.0119 0.00669
2R:5472040-5472190:plus -32.8163 -1.37615 -0.825688 2.50459 -18.4082 -28.6327 -12.3853 -2.97222
4.13761 0.024 0.0161 0.0151 0.0114 0.00937 0.066 0.00503 0.0384 0.00408
2R:5474580-5474730:minus -43.9592 -4.63303 -2.05505 3.75229 -28.7959 -18.3469 -22.5413 6.69444 5.3945
0.0946 0.04 0.022 0.00649 0.0641 0.0124 0.0324 0.00118 0.00263
2R:5474580-5474730:plus -23.1531 3.47706 0.0275229 2.82569 1.14286 -8.5 -22 -6.91667 3.26606
0.00542 0.00292 0.0115 0.00976 0.000113 0.00126 0.0295 0.0911 0.00627
2R:5476440-5476590:minus -30.8878 3.55963 2.42202 -0.46789 -17.2245 -17.5714 -18.2569 9.69444 4.77982
0.0103 0.00283 0.00495 0.0309 0.00384 0.00616 0.0155 0.000203 0.00339
2R:5476440-5476590:plus -22.9592 -2.40367 2.52294 1.44954 -7.97959 -19.1224 -14.2752 7.44444 4.19266
0.00475 0.0218 0.00476 0.0179 0.000799 0.0175 0.00771 0.000795 0.00401
2R:5477880-5478030:minus -41.2551 4.14679 2.88991 3.31193 0.540816 -18.8571 -21.3211 3.61111 3.55963
0.0513 0.00228 0.00414 0.00786 0.000321 0.0151 0.0261 0.0047 0.00545
2R:5477880-5478030:plus -31.4796 14.7523 -3.95413 2.07339 -28.0408 -17.051 -18.1835 3.44444 1.2844
0.0128 3.48e-06 0.0375 0.0138 0.0455 0.00479 0.0154 0.00502 0.0129
2R:5478200-5478350:minus -21.9184 6.61468 4.27523 1.68807 -8.16327 -9.57143 -22.4495 -2.86111 6.42202
0.00261 0.000789 0.0024 0.0164 0.000873 0.00326 0.0319 0.0373 0.00156
2R:5478200-5478350:plus -3.47959 -0.238532 -0.990826 1.87156 1.87755 -18.0102 1.45872 0.625
10.0367 0.0000728 0.0112 0.0159 0.0152 0.0000492 0.00812 0.000194 0.0137 0.000146
2R:5492720-5492870:minus -23.1939 6.38532 -0.486239 6.00917 -17.6837 -10.0204 -24.1651 6.15278
-6.47706 0.00558 0.000882 0.0136 0.00255 0.0058 0.00399 0.042 0.00154 0.1
2R:5492720-5492870:plus -32.7449 -2.14679 3.55046 6.00917 -18.7143 -17.9796 -10.8257 -4.43056 -9.38532
0.0228 0.0203 0.00321 0.00255 0.0136 0.00764 0.00361 0.0544 0.176
2R:5493400-5493550:minus -24.5918 -0.93578 6.85321 4.6422 -18.051 -19.3776 -13.0183 5.81944 6.17431
0.00821 0.014 0.000779 0.00473 0.00746 0.0195 0.00581 0.00181 0.00171
2R:5493400-5493550:plus -31.8469 1.21101 1.40367 1.55963 -17.8571 -9.65306 -19.9908 -0.166667
-2.44037 0.0162 0.00671 0.00717 0.0173 0.00698 0.0034 0.0206 0.0176 0.0381
2R:5494720-5494870:minus -23.3673 1.20183 1.06422 1.12844 -19.3163 -17.3469 -18.0092 9.68056 5.18349

0.00617 0.00673 0.00808 0.0198 0.0165 0.0058 0.015 0.000205 0.00295
2R:5494720-5494870:plus -32.9286 0.192661 6.47706 5.90826 -27.4592 -9.72449 -20.7339 -5.16667 -4.00917
0.025 0.00965 0.000928 0.00271 0.0329 0.00352 0.0234 0.0639 0.0558
2R:5571340-5571490:minus -23.2959 -1.37615 8.55963 9.51376 -7.89796 -18.0102 -16.6789 2.08333 -2.77064
0.00605 0.0161 0.000322 0.000292 0.000678 0.00812 0.0122 0.00837 0.0419
2R:5571340-5571490:plus -21.7041 -1.65138 9.07339 -0.0275229 -8.44898 -35.1531 -11.9266 3 7.22018
0.00231 0.0175 0.000243 0.027 0.00109 0.0753 0.00454 0.00597 0.00119
2R:5574660-5574810:minus -24.4082 -0.275229 8.23853 6.01835 -17.4184 -18.7857 -17.7523 2.65278
5.70642 0.00797 0.0113 0.000386 0.00248 0.00457 0.0146 0.0144 0.0068 0.00222
2R:5574660-5574810:plus -24.5918 0.816514 9.23853 3.0367 -17.1122 0.122449 -5.21101 4.29167 8.97248
0.00821 0.00773 0.00022 0.00886 0.00341 0.000312 0.00105 0.00356 0.000449
2R:5579500-5579650:minus 5.95918 1.13761 -0.972477 6.3578 -27.4592 0.387755 -10.5872 -2.40278
-2.01835 6.88e-06 0.00689 0.0158 0.00195 0.0329 0.000217 0.00344 0.0332 0.0334
2R:5579500-5579650:plus -12.7449 13.5306 1.40367 2.50459 -26.898 -17.5 -14.1835 -1.23611 -4.36697
0.000471 0.0000206 0.00717 0.0114 0.0203 0.00599 0.00755 0.0241 0.0615
2R:5580220-5580370:minus -14.0714 -4.57798 6.31193 5.30275 -27.3061 -18.1224 -6.3211 -1.41667 4.31193
0.00117 0.0395 0.001 0.00396 0.0315 0.00988 0.00143 0.0254 0.00386
2R:5580220-5580370:plus -30.4694 -2.98165 5.72477 0.0733945 -18.6837 -8.72449 -29.5229 -4.29167
-7.12844 0.00926 0.0257 0.0013 0.0263 0.0126 0.00169 0.085 0.0527 0.116
2R:5580800-5580950:minus -14.2143 1.59633 0.93578 4.36697 10.6224 10.6429 -2.72477 2.84722 4.04587
0.00124 0.00582 0.00845 0.00505 0.0000113 3.35e-06 0.000529 0.00632 0.00421
2R:5580800-5580950:plus -21.9592 14.9266 3.14679 3.78899 -17.4898 -17.2755 -19.2018 -0.875 -0.119266
0.00279 2.37e-06 0.00375 0.00632 0.0053 0.00544 0.018 0.0218 0.0192
2R:559260-559410:minus -14.7143 -0.559633 -2.22018 0.642202 -27.2347 -26.8265 -24.1284 5.45833
-3.80734 0.00154 0.0124 0.0231 0.0227 0.0283 0.0285 0.0418 0.00214 0.0528
2R:559260-559410:plus -41.5102 5.48624 4.46789 0.788991 -27.4592 -9.45918 -18.5229 0.861111 -2.42202
0.0582 0.00133 0.00222 0.0218 0.0329 0.00279 0.0162 0.0127 0.0378
2R:5599080-5599230:minus -31.5204 -1.41284 -0.376147 3.18349 -17.7143 1.12245 -11.7339 0.416667
6.33028 0.0131 0.0163 0.0131 0.00837 0.00627 0.000085 0.00435 0.0147 0.0016
2R:5599080-5599230:plus -13.602 -3.83486 3.22018 0 -17.2245 -26.6735 1.61468 -0.638889 11.8532
0.000995 0.0324 0.00365 0.0269 0.00384 0.0256 0.000185 0.0203 0.0000428
2R:5599680-5599830:minus -23.0714 1.44954 2.12844 2.82569 -8.26531 -27.7857 -0.642202 -1.69444
7.53211 0.0052 0.00615 0.00551 0.00976 0.000934 0.0466 0.000328 0.0274 0.000968
2R:5599680-5599830:plus 5.43878 3.68807 -2.77982 5.37615 -8.60204 -16.5 -2.46789 -3.09722 10.3394
0.0000102 0.0027 0.0271 0.00384 0.00125 0.00416 0.000497 0.0396 0.000112
2R:5600180-5600330:minus -22.8163 -3.66055 3.52294 2.90826 -17.3776 -9.53061 -23.2569 1.56944 11.8073
0.00418 0.0309 0.00324 0.00936 0.00419 0.0031 0.0366 0.01 0.0000538
2R:5600180-5600330:plus -24 -2.11927 1.74312 1.51376 -26.898 -26.8265 -11.4862 1.01389 4.14679
0.00719 0.0201 0.00635 0.0175 0.0203 0.0285 0.00413 0.0121 0.00406
2R:5698320-5698470:minus -32.9286 -3.7156 -2.91743 2.27523 -8.93878 -8.72449 -13.7156 4.84722 -10.2385
0.025 0.0313 0.0282 0.0125 0.00216 0.00169 0.00681 0.00281 0.202
2R:5698320-5698470:plus -33.7347 0.715596 1.58716 7.34862 -19.051 -8.60204 -22.9817 2.125 -2.05505
0.0294 0.00802 0.00672 0.000998 0.0162 0.00151 0.035 0.00824 0.0338
2R:5701840-5701990:minus -23.1429 7.72477 0.981651 4.91743 -8.67347 -8.42857 -21.8257 2.97222 -1.54128
0.00533 0.000443 0.00832 0.00447 0.00154 0.00104 0.0286 0.00603 0.0296
2R:5701840-5701990:plus -22.4388 5.44954 1.9633 -0.321101 -27.2347 -27.1224 -11.6055 7.54167
7.25688 0.00348 0.00135 0.00586 0.0294 0.0283 0.0325 0.00423 0.000754 0.00115
2R:5707600-5707750:minus -12.6224 1.25688 -3.55046 7.80734 -18.4184 -17.2755 -2.78899 7.48611 1.00917
0.000453 0.00659 0.0336 0.00063 0.0101 0.00544 0.000538 0.000777 0.0143
2R:5707600-5707750:plus -22.7041 3.61468 3.57798 2.27523 -7.86735 -9.23469 -8.70642 1.59722 9.87156
0.00395 0.00277 0.00317 0.0125 0.000617 0.00238 0.00242 0.00993 0.000217
2R:5710440-5710590:minus -22.2959 -1.89908 4.11009 6.21101 1.54082 0.0510204 -12.0092 1.375
-0.458716 0.00335 0.0189 0.00256 0.00213 0.0000712 0.000349 0.00462 0.0107 0.0217
2R:5710440-5710590:plus -22.5612 13.5306 3.50459 5.57798 -17.7143 -8.79592 -8.51376 6.65278 7.51376

0.0036 0.0000206 0.00326 0.0034 0.00627 0.0019 0.00233 0.0012 0.000984
2R:5724400-5724550:minus -13.449 15.1376 2.13761 3.46789 -26.2653 -9.34694 -20.9541 2.97222 3.20183
0.000914 1.81e-06 0.00549 0.00726 0.018 0.00261 0.0244 0.00603 0.0065
2R:5724400-5724550:plus -4.59184 3.83673 1.42202 1.2844 -27.4898 -17.4184 -11.4587 -0.0277778
-0.174312 0.000166 0.00115 0.00712 0.019 0.0336 0.00585 0.0041 0.0169 0.0196
2R:5727060-5727210:minus -31.6327 13.3878 1.82569 4.6055 -17.9286 -26.5612 -13 2.20833 3.46789
0.0138 0.0000869 0.00616 0.00476 0.0071 0.0237 0.00578 0.008 0.00582
2R:5727060-5727210:plus -32.1122 -2.88991 4.66972 3.81651 -27.3061 -27.5306 -21.2936 -2.75 7.07339
0.0178 0.025 0.00204 0.00626 0.0315 0.0374 0.0259 0.0363 0.0013
2R:5730000-5730150:minus -24.5612 11.0367 5.57798 3.62385 -18.4898 0.0510204 -18.7706 3.65278
-3.21101 0.00819 0.0000797 0.00139 0.00673 0.0116 0.000349 0.0168 0.00462 0.0468
2R:5730000-5730150:plus -13.1429 6.77064 3.3945 9.44954 -26.9286 -9.5 -6.18349 6.11111 7.63303
0.00065 0.000731 0.00341 0.000326 0.0214 0.00294 0.00138 0.00157 0.000928
2R:5731120-5731270:minus -21.8878 0.211009 -1.97248 0.53211 -18.7857 -17.1224 -24.9817 4.45833 0.559633
0.00258 0.00959 0.0214 0.0234 0.0148 0.00499 0.047 0.00332 0.016
2R:5731120-5731270:plus -20.3673 8.30275 -0.0917431 4.04587 -17.4184 -25.8265 -9.41284 4.41667
7.46789 0.00166 0.000324 0.0119 0.00572 0.00457 0.0212 0.00276 0.00338 0.00102
2R:5733980-5734130:minus -30.6633 5.50459 12.9358 3.93578 -17.449 -17.9796 -9.44037 5.86111 -0.504587
0.00976 0.00132 0.0000218 0.00597 0.00484 0.00764 0.00278 0.00177 0.0221
2R:5733980-5734130:plus -32.0714 3.73394 -3.31193 1.42202 -27.1939 -0.0510204 -13.3486 7.98611
2.06422 0.0176 0.00266 0.0315 0.0182 0.0267 0.000429 0.00626 0.000588 0.00912
2R:5735420-5735570:minus -13.7347 4.55046 0.00917431 3.78899 -17.6429 -26.4898 -13.789 0.291667
-0.605505 0.00105 0.00195 0.0116 0.00632 0.00562 0.0225 0.00692 0.0153 0.023
2R:5735420-5735570:plus -10.551 3.11927 -2.43119 2.87156 -26.5306 0.826531 -11.2936 -1.48611 -1.68807
0.000233 0.00333 0.0245 0.00948 0.0185 0.000153 0.00396 0.0259 0.0308
2R:5748180-5748330:minus -3.7449 2.3578 6.17431 6.01835 -18.1122 -18.2041 -1.86239 0.486111 1.40367
0.000104 0.0044 0.00107 0.00248 0.00771 0.0103 0.00043 0.0144 0.0122
2R:5748180-5748330:plus -22.7755 1.13761 0.522936 2.0367 -27.1224 -17.8265 -9.38532 5.20833 2.15596
0.00412 0.00689 0.00974 0.0141 0.0245 0.00699 0.00275 0.00239 0.00881
2R:5749360-5749510:minus -11.8061 1.93578 0.917431 3.14679 -17.7551 -26.8571 -4.51376 -0.263889
1.21101 0.000268 0.00514 0.00851 0.00847 0.00658 0.0288 0.000864 0.0181 0.0134
2R:5749360-5749510:plus -30.551 5.74312 1.89908 3.47706 -17.3776 -17.8571 -11.3028 -2.48611 10.3486
0.00959 0.00119 0.006 0.00724 0.00419 0.00719 0.00397 0.0339 0.0000988
2R:5751080-5751230:minus -31.7755 0.00917431 1.22018 1.65138 -18.4592 -19.3469 -18.0734 2.73611
0.816514 0.0157 0.0103 0.00765 0.0166 0.0109 0.0194 0.0151 0.00659 0.015
2R:5751080-5751230:plus -23.6327 1.73394 13.9541 7.2844 -17.7857 -9.23469 -22.367 2.54167 5.44954
0.00661 0.00553 7.63e-06 0.00105 0.00676 0.00238 0.0315 0.00709 0.00253
2R:5752980-5753130:minus -22.2959 -2.73394 14.8257 7.29358 -17.4184 -7.16327 -9.42202 0.458333 6.11927
0.00335 0.0239 2.55e-06 0.00104 0.00457 0.000547 0.00277 0.0145 0.00178
2R:5752980-5753130:plus -13.2551 3.22018 -3.40367 0.100917 -9.12245 -17.2347 -16.7615 0.138889 0.715596
0.000751 0.00321 0.0323 0.0261 0.00238 0.00511 0.0123 0.016 0.0154
2R:5762640-5762790:minus -31.4388 4.19266 0.12844 6.54128 -17.4082 -18.051 -13.1193 3.66667 5.75229
0.0122 0.00224 0.0111 0.00173 0.00421 0.00886 0.00594 0.0046 0.00213
2R:5762640-5762790:plus -13.5204 -0.256881 11.1284 2.66972 -9.16327 -17.9796 0.504587 2.41667
5.25688 0.00097 0.0113 0.0000734 0.0106 0.00249 0.00764 0.000251 0.00742 0.0028
2R:5769820-5769970:minus -24.7449 0.944954 1.0367 2.11927 20.1735 -8.7551 3.29358 6.43056 1.73394
0.00827 0.00739 0.00816 0.0135 5.68e-07 0.00172 0.000107 0.00134 0.0106
2R:5769820-5769970:plus -39.4796 0.137615 -4.3945 1.84404 -17.9796 -8.7551 -11.9083 7.15278 -0.119266
0.0334 0.00984 0.0422 0.0154 0.00726 0.00172 0.00452 0.000928 0.0192
2R:5779040-5779190:minus -23.0408 11.1651 2.07339 2 -27.2347 -8.65306 -1.44954 -4.01389 3.29358
0.00512 0.0000741 0.00562 0.0143 0.0283 0.00156 0.000392 0.0494 0.00623
2R:5779040-5779190:plus -30.2551 -2.50459 1.44954 6.56881 -26.9592 -17.9796 -15.6972 1.30556 -3.14679
0.00885 0.0225 0.00705 0.0017 0.0215 0.00764 0.0103 0.011 0.0463
2R:5779400-5779550:minus -31.551 -2.11927 1.46789 4.40367 11.6939 0.204082 -13.1743 0.277778 -5.01835

0.0134 0.0201 0.007 0.00497 1.17e-06 0.000296 0.00602 0.0153 0.073
2R:5779400-5779550:plus -33 0.568807 -0.284404 6.72477 10.6939 -8.79592 -29.0734 1.30556 -6.88991
0.0258 0.00845 0.0127 0.00154 7.35e-06 0.0019 0.0797 0.011 0.11
2R:5798380-5798530:minus -44.1122 4.48624 -3.68807 3.88073 -27.5714 -17.4184 -25.7982 5.34722 3.51376
0.0949 0.00199 0.0349 0.0061 0.0357 0.00585 0.0522 0.00225 0.00566
2R:5798380-5798530:plus -21.7449 0.183486 8.55963 5.90826 -17.6429 -8.5 -11.2202 4.625 5.38532
0.00236 0.00968 0.000322 0.00271 0.00562 0.00126 0.0039 0.00309 0.00267
2R:5817240-5817390:minus -23.1429 1.14679 2.54128 9.59633 -27.1939 1.2449 -11.945 -2.58333 -0.761468
0.00533 0.00686 0.00473 0.000275 0.0267 0.0000797 0.00456 0.0348 0.0244
2R:5817240-5817390:plus -20.6735 -1.37615 -1.43119 6.3578 -17.7959 -17.7143 -15.9725 -2.875 7.62385
0.00173 0.0161 0.0182 0.00195 0.00678 0.00634 0.0108 0.0375 0.000941
2R:5837120-5837270:minus -3.64286 1.33945 -0.385321 4.23853 -17.7551 -8.72449 -26.6147 -1.30556
1.26606 0.000092 0.0064 0.0131 0.00522 0.00658 0.00169 0.0577 0.0246 0.013
2R:5837120-5837270:plus -30.8163 -4.3211 0.669725 1.98165 -9.5 -8.34694 -14.4954 3 6.26606 0.0103
0.0369 0.00926 0.0145 0.00293 0.000993 0.00808 0.00597 0.00162
2R:5846040-5846190:minus -3.18367 -0.844037 -0.605505 -0.46789 -18.449 -17.4184 6.77064 3.13889
7.37615 0.0000481 0.0136 0.0141 0.0309 0.0107 0.00585 0.0000331 0.00566 0.00107
2R:5846040-5846190:plus -11.898 -2.62385 -0.183486 3.9633 -8.63265 -17.2755 -5.0367 -1.38889
9.88073 0.00029 0.0232 0.0123 0.0059 0.0013 0.00544 0.001 0.0252 0.000206
2R:5847280-5847430:minus -22.8878 1.58716 4.14679 6.57798 -6.60204 1.16327 -19.0183 4.625 3.66055
0.00441 0.00584 0.00252 0.00167 0.000355 0.0000831 0.0175 0.00309 0.00515
2R:5847280-5847430:plus -23.9184 1.3578 4.77064 7.6055 -27.2245 -17.3469 -11.9633 0.388889 1.23853
0.00695 0.00635 0.00196 0.000794 0.0272 0.0058 0.00457 0.0148 0.0132
2R:5847900-5848050:minus -31.5918 3.9633 7.77064 3.0367 -8.67347 -17.7857 -19.4771 -1.41667 4.78899
0.0136 0.00244 0.000494 0.00886 0.00154 0.0068 0.0189 0.0254 0.00336
2R:5847900-5848050:plus -21.3673 5.14679 7.2844 5.49541 -18.1224 -0.316327 -13.8532 -0.263889
1.01835 0.00195 0.00153 0.000632 0.00355 0.00782 0.000524 0.00702 0.0181 0.0142
2R:5856640-5856790:minus -32.551 0.211009 0.348624 0.715596 -27.0102 0.0204082 -15.3761 -4.375
4.10092 0.0207 0.00959 0.0103 0.0223 0.0237 0.000377 0.00966 0.0537 0.00413
2R:5856640-5856790:plus -23.0714 -1.44037 4.97248 5.90826 -8.64286 -26.7857 -16.3486 -1.45833 8.10092
0.0052 0.0164 0.0018 0.00271 0.00139 0.0266 0.0115 0.0257 0.000623
2R:5858700-5858850:minus -12.6735 1.85321 0.440367 2.07339 0.877551 -18.6429 -0.917431 2.75 3.21101
0.000461 0.0053 0.01 0.0138 0.00019 0.0139 0.000348 0.00656 0.00644
2R:5858700-5858850:plus -31.0408 1.18349 -0.834862 4.78899 -27.5714 -17.7551 -25.0459 2.22222
3.45872 0.0106 0.00677 0.0152 0.00456 0.0357 0.00643 0.0474 0.00796 0.00591
2R:5876560-5876710:minus -31.1429 1.29358 8.46789 3.0367 -27.2347 -17.2041 -19.1743 3.47222 -0.541284
0.0108 0.00651 0.00034 0.00886 0.0283 0.00507 0.0179 0.00497 0.0223
2R:5876560-5876710:plus -31.4082 0.522936 3.3578 2.55046 -26.6327 -26.7857 -2.38532 0.222222 -2.30275
0.0119 0.00859 0.00346 0.0112 0.0188 0.0266 0.000487 0.0156 0.0363
2R:5876800-5876950:minus -23.0408 -4.81651 -0.697248 5.85321 -26.9694 -9.42857 -22.4954 2.40278
-1.59633 0.00512 0.042 0.0145 0.00287 0.023 0.0027 0.0322 0.00746 0.0301
2R:5876800-5876950:plus -22.6224 0.321101 0.302752 9.41284 -8.96939 -18.5612 -18.2202 0.555556 0.0733945
0.00371 0.00922 0.0105 0.000354 0.00226 0.0132 0.0155 0.014 0.0181
2R:5877080-5877230:minus -23.0714 1.82569 -1.81651 6.3578 -26.8571 -9.16327 -8.9633 -0.0833333
0.12844 0.0052 0.00535 0.0205 0.00195 0.0198 0.00223 0.00254 0.0172 0.0179
2R:5877080-5877230:plus -21.551 2.36697 5.82569 7.47706 -8.90816 -9.20408 -25.4771 -1.01389 -10.1835
0.00205 0.00439 0.00125 0.00085 0.00192 0.00225 0.0501 0.0227 0.2
2R:5913460-5913610:minus -31.7449 -0.908257 1.10092 2.6422 -18.2245 0.826531 -22.2936 8.65278
4.77982 0.0152 0.0139 0.00798 0.0107 0.00877 0.000153 0.0311 0.000397 0.00339
2R:5913460-5913610:plus -21.6735 -2.47706 3.51376 4.91743 -27.3367 -9.86735 -13.5963 3.38889 7.04587
0.00224 0.0223 0.00325 0.00447 0.0317 0.00391 0.00663 0.00513 0.00132
2R:5914220-5914370:minus -31.9286 1.40367 4.51376 6.12844 -17.4184 -19.3776 -20.8349 1.125 0.348624
0.0166 0.00625 0.00218 0.00231 0.00457 0.0195 0.0239 0.0117 0.0167
2R:5914220-5914370:plus -32.449 6.19266 -1.36697 5.98165 -9.23469 -7.5 -15.6422 3.18056 -3.84404

0.0195 0.000967 0.0179 0.00258 0.00277 0.000614 0.0102 0.00557 0.0533
2R:5920420-5920570:minus -32.7347 0.0366972 -2.21101 4.22018 -18.4184 -8.72449 -10.5321 -3.79167
-0.284404 0.0225 0.0102 0.023 0.00531 0.0101 0.00169 0.0034 0.0469 0.0207
2R:5920420-5920570:plus -32.6633 4 1.6789 7.27523 -7.93878 -7.72449 -31.4771 6.66667 -6.11927
0.0216 0.0024 0.0065 0.00109 0.000767 0.00068 0.111 0.00119 0.0914
2R:5920720-5920870:minus -33.0306 0.46789 1.82569 2.11927 -18.7143 -9.57143 -15.8991 6.22222 -2.68807
0.026 0.00876 0.00616 0.0135 0.0136 0.00326 0.0107 0.00149 0.041
2R:5920720-5920870:plus -31.8878 1.36697 2.53211 5.85321 -27.1939 -8.86735 -23.1927 0.583333 -0.541284
0.0164 0.00633 0.00475 0.00287 0.0267 0.00198 0.0362 0.0139 0.0223
2R:5933220-5933370:minus -20.7347 5.43119 0.284404 2.07339 -27.1531 -9.68367 -6.57798 5.11111 3.23853
0.00176 0.00136 0.0105 0.0138 0.0249 0.00342 0.00152 0.0025 0.00639
2R:5933220-5933370:plus -24.7449 3.57143 1.45872 7.27523 -26.2653 -16.2755 -25.8991 5.83333 -4.73394
0.00827 0.00175 0.00703 0.00109 0.018 0.00413 0.0528 0.00179 0.0681
2R:5934620-5934770:minus -41.3265 -1.6789 13.578 7.29358 -26.9694 -17.051 -13.1376 -0.458333
0.862385 0.0541 0.0177 0.0000123 0.00104 0.023 0.00479 0.00597 0.0192 0.0148
2R:5934620-5934770:plus -23.3367 9.12844 -0.211009 5.49541 -26.5306 -18.2755 -0.146789 1.97222
3.97248 0.00614 0.000209 0.0124 0.00355 0.0185 0.0112 0.000293 0.0087 0.00444
2R:5935720-5935870:minus -23.4388 -4.30275 4.16514 2.84404 -18.449 -18.8265 -14.6055 11.125 6.49541
0.00625 0.0367 0.00251 0.00961 0.0107 0.0148 0.00827 0.0000686 0.00154
2R:5935720-5935870:plus -31.7755 -0.669725 -1.06422 5.74312 -27.1531 -8.16327 -20.789 10.8194
-9.43119 0.0157 0.0129 0.0163 0.00302 0.0249 0.000783 0.0237 0.0000883 0.178
2R:5936080-5936230:minus -21.7041 1.58716 -1.10092 5.06422 -17.7143 0.755102 4.78899 4.04167
-0.0183486 0.00231 0.00584 0.0165 0.00433 0.00627 0.000175 0.000062 0.00395 0.0185
2R:5936080-5936230:plus -30.7755 4.76147 -3.82569 2 -16.7143 -9.86735 -10.5688 3.81944 5.48624
0.0102 0.00179 0.0362 0.0143 0.00319 0.00391 0.00343 0.00433 0.00251
2R:5936720-5936870:minus -13.6735 7.65138 -3.33945 -0.651376 -25.898 -9.7551 -12.9817 3.79167
-4.84404 0.00103 0.00046 0.0317 0.0329 0.0166 0.00358 0.00576 0.00437 0.0701
2R:5936720-5936870:plus -32 6.7156 6.95413 5.31193 -8.33673 -18.051 -14.8624 0.0694444 -0.220183
0.0171 0.00075 0.000743 0.00393 0.00101 0.00886 0.00873 0.0164 0.0201
2R:5937120-5937270:minus -30.6327 0.678899 8.25688 3.02752 -18.4898 -9.7551 -27.1009 -1.77778 -4.45872
0.00968 0.00813 0.000382 0.00887 0.0116 0.00358 0.0613 0.0281 0.0633
2R:5937120-5937270:plus -24.2653 3.68367 -1.20183 5.86239 -8.64286 -19.3469 -10.633 6.66667 4.66055
0.00773 0.00144 0.017 0.00282 0.00139 0.0194 0.00347 0.00119 0.0035
2R:5939600-5939750:minus -40.2857 14.5596 2.18349 3.24771 -8.33673 -16.9796 -13.844 4.13889 1.37615
0.0385 4.47e-06 0.0054 0.00808 0.00101 0.0044 0.00701 0.0038 0.0123
2R:5939600-5939750:plus -31.9184 13.4592 0.963303 5.45872 -26.2347 -9.02041 -18.7615 2.65278 1.69725
0.0164 0.000064 0.00837 0.00363 0.0177 0.00213 0.0168 0.0068 0.0108
2R:5954040-5954190:minus -32.6633 3.90816 13.4771 5.06422 -17.449 -27.5306 -13.844 4.69444 -2.83486
0.0216 0.00102 0.0000133 0.00433 0.00484 0.0374 0.00701 0.003 0.0427
2R:5954040-5954190:plus -22.2245 5.92661 10.3578 9.08257 -17.7143 0.826531 -13.8899 1.84722 0.486239
0.00325 0.00109 0.000115 0.000444 0.00627 0.000153 0.00708 0.0091 0.0163
2R:5963080-5963230:minus -33.4082 -2.92661 2.86239 3.45872 10.9184 -8.23469 -26.8899 2.09722 -0.926606
0.0283 0.0253 0.00418 0.00731 4.19e-06 0.000875 0.0597 0.00832 0.0256
2R:5963080-5963230:plus -13.6327 5.65138 -2.82569 2.62385 -18.4898 -0.540816 -21.789 7.06944
-0.440367 0.00101 0.00124 0.0275 0.0108 0.0116 0.00054 0.0284 0.00097 0.0216
2R:5963740-5963890:minus -30.2143 -0.963303 -4.82569 1.80734 -27.2653 -9.5 -13.7523 1.33333 1.54128
0.0088 0.0142 0.0472 0.0156 0.0302 0.00294 0.00686 0.0109 0.0115
2R:5963740-5963890:plus -21.8878 -0.577982 3.49541 7 -18.4592 -17.7857 -22.1376 1.88889 1.55963
0.00258 0.0125 0.00327 0.00132 0.0109 0.0068 0.0302 0.00896 0.0114
2R:5964580-5964730:minus -21.6224 0.522936 0.458716 3.19266 -18.6429 -8.23469 -12.1193 -2.90278 0.752294
0.00213 0.00859 0.00995 0.00829 0.0121 0.000875 0.00473 0.0377 0.0152
2R:5964580-5964730:plus -22.398 0.972477 2.97248 4.33028 -9.23469 -18.051 -4.79817 -3.81944 2.15596
0.00344 0.00731 0.00401 0.00511 0.00277 0.00886 0.000937 0.0472 0.00881
2R:5965060-5965210:minus -31.2551 -0.844037 0.40367 3.08257 -26.6735 -9.79592 -19.4037 1.80556

1.05505 0.0113 0.0136 0.0101 0.0087 0.0191 0.00377 0.0186 0.00923 0.014
2R:5965060-5965210:plus -24.1837 -0.825688 2.2844 1.48624 -17.4898 -9.72449 -15.1927 7.76389
3.46789 0.00751 0.0136 0.0052 0.0177 0.0053 0.00352 0.00932 0.000666 0.00582
2R:5965760-5965910:minus -4.21429 -1.20183 2.06422 0.366972 -27.3776 -19.0816 9.3578 -2.51389 7.68807
0.000129 0.0153 0.00564 0.0244 0.032 0.017 0.0000161 0.0341 0.000841
2R:5965760-5965910:plus -2.91837 0.633028 -0.844037 9.61468 -16.3776 -8.68367 0.917431 3.94444
3.88991 0.0000392 0.00826 0.0152 0.000255 0.003 0.00157 0.000225 0.00411 0.00467
2R:5966300-5966450:minus -31.7041 3.31193 14.6881 3.84404 -17.2245 -27.5612 -15.1284 -0.694444 2
0.0146 0.0031 3.06e-06 0.00624 0.00384 0.0393 0.00921 0.0206 0.00941
2R:5966300-5966450:plus -30.949 1.11927 12.0367 6.52294 -8.53061 -16.7857 -17.5229 -4.94444 -1.77064
0.0104 0.00693 0.000042 0.00174 0.0011 0.00426 0.0139 0.0609 0.0313
2R:5984520-5984670:minus -32 -0.412844 1.33945 -0.0458716 -18.4592 -7.45918 -14.7523 -1.11111
-0.862385 0.0171 0.0119 0.00733 0.0271 0.0109 0.000582 0.00853 0.0233 0.0251
2R:5984520-5984670:plus -21.9592 -5.77064 5.45872 6.05505 -18.0816 -18.0816 -12.9725 -3.47222 1.6422
0.00279 0.0539 0.00147 0.0024 0.00759 0.00934 0.00574 0.0434 0.0111
2R:5988420-5988570:minus -31.9592 3.07339 -2.72477 4.91743 -16.7551 -18.0816 -8.54128 7.80556 5.44954
0.0167 0.00339 0.0267 0.00447 0.00322 0.00934 0.00234 0.000651 0.00253
2R:5988420-5988570:plus -23.1939 4.44954 1.21101 9.59633 -17.3776 0.27551 -19.3303 -4.45833 0.550459
0.00558 0.00202 0.00768 0.000275 0.00419 0.000257 0.0184 0.0547 0.0161
2R:5988940-5989090:minus -29.3673 1.02752 1.11009 2.44037 -18.4184 -8.79592 -24.8349 9.88889 -3.43119
0.00838 0.00717 0.00796 0.0116 0.0101 0.0019 0.0461 0.000178 0.0489
2R:5988940-5989090:plus -24.0714 0.522936 -2.44037 -0.0550459 -9.0102 -8.94898 -10.4312 -0.305556
-2.76147 0.00731 0.00859 0.0246 0.0272 0.00236 0.00203 0.00334 0.0184 0.0418
2R:5990820-5990970:minus -13.5204 2.88991 2.31193 5.3945 -18.7143 -16.7143 -22.422 5.22222 3.15596
0.00097 0.00362 0.00515 0.00379 0.0136 0.00419 0.0318 0.00238 0.00659
2R:5990820-5990970:plus -21.398 -0.330275 1.90826 3.48624 -17.449 -17.3469 -16.6514 2.26389
-3.99083 0.00197 0.0115 0.00598 0.0072 0.00484 0.0058 0.0121 0.00784 0.0556
2R:5991160-5991310:minus -32.9184 4.97248 -1.20183 -1.46789 -17.1531 -26.8265 -14.5505 6.01389 1.07339
0.0248 0.00165 0.017 0.0428 0.00364 0.0285 0.00818 0.00165 0.014
2R:5991160-5991310:plus -40.6224 1.91743 3.81651 5.31193 -18.7143 -17.5 -21.4771 4.80556 -1.20183
0.0424 0.00518 0.00289 0.00393 0.0136 0.00599 0.0268 0.00286 0.0276
2R:5998340-5998490:minus -33.0408 5.44954 6.91743 3.97248 -27.2347 -18.051 -19.5872 1.48611 -0.192661
0.0264 0.00135 0.000755 0.00587 0.0283 0.00886 0.0192 0.0103 0.0198
2R:5998340-5998490:plus -21.9592 0.559633 13.0092 3.43119 -27.3061 -27.5612 -6.19266 -0.986111
-1.3578 0.00279 0.00848 0.0000207 0.00749 0.0315 0.0393 0.00138 0.0225 0.0286
2R:6005100-6005250:minus -33 -0.311927 -0.0366972 6.02752 -27.1939 -17.5 -14.8991 4.59722
-0.0733945 0.0258 0.0115 0.0117 0.00246 0.0267 0.00599 0.00879 0.00313 0.0188
2R:6005100-6005250:plus -3.29592 13.4592 -0.66055 4.22018 -18.1939 -7.5 -4.74312 2.16667 -3.83486
0.0000549 0.000064 0.0143 0.00531 0.0086 0.000614 0.000922 0.00812 0.0533
2R:6011640-6011790:minus -32.5204 -3.88073 -3.44954 5.73394 -7.64286 -16.9796 -6.55963 0.694444 -1.58716
0.0204 0.0328 0.0327 0.00307 0.000499 0.0044 0.00152 0.0134 0.03
2R:6011640-6011790:plus -31.6327 3.83486 5.53211 7.34862 -18.449 10.5714 -14.6422 2.19444 -6
0.0138 0.00256 0.00142 0.000998 0.0107 4.67e-06 0.00834 0.00804 0.0889
2R:6012420-6012570:minus -30.449 2.16514 -0.59633 4.23853 -18.3469 -18.8265 -9.50459 -1.75 5.22936
0.00925 0.00473 0.0141 0.00522 0.00894 0.0148 0.00281 0.0279 0.00288
2R:6012420-6012570:plus -14.449 1.55046 0.275229 0.963303 -26.8571 0.540816 -17.8073 -0.847222
1.79817 0.0014 0.00592 0.0106 0.0208 0.0198 0.0002 0.0145 0.0216 0.0102
2R:6013300-6013450:minus -24.0408 -3.25688 1.6055 5.45872 -17.6429 -9.45918 -11.7339 7.15278 5.53211
0.00726 0.0277 0.00667 0.00363 0.00562 0.00279 0.00435 0.000928 0.00245
2R:6013300-6013450:plus -5.04082 -0.486239 0.220183 2.94495 -18.2245 -26.898 0.440367 -3.44444
1.73394 0.000223 0.0121 0.0108 0.0092 0.00877 0.0302 0.000255 0.0431 0.0106
2R:6015720-6015870:minus -31.6224 0.522936 5.84404 9.92661 -9.16327 -9.30612 -14.8624 -0.263889
5.38532 0.0137 0.00859 0.00124 0.000169 0.00249 0.00257 0.00873 0.0181 0.00267
2R:6015720-6015870:plus -32.4796 -0.559633 4.66055 3.69725 -18.7551 -19.0816 -11.6881 4.02778

9.75229 0.02 0.0124 0.00205 0.00665 0.0144 0.017 0.00431 0.00397 0.000241
2R:6029760-6029910:minus -32.4796 -0.816514 1.25688 1.94495 -18.4184 -17.3469 -17.1009 6.56944
-1.01835 0.02 0.0135 0.00755 0.0147 0.0101 0.0058 0.013 0.00125 0.0263
2R:6029760-6029910:plus -31.9592 -1.69725 0.880734 -0.577982 1.54082 -28.1837 -4.85321 2.04167
2.73394 0.0167 0.0178 0.00861 0.0321 0.0000712 0.0584 0.000952 0.00849 0.00756
2R:6043640-6043790:minus -39.8776 3.42202 1.77982 -1 -7.93878 -16.3469 -23.2477 0.513889 6.12844
0.0341 0.00298 0.00627 0.0367 0.000767 0.00416 0.0365 0.0142 0.00174
2R:6043640-6043790:plus -30.2959 0.192661 0.477064 3.87156 -17.3776 1.20408 -16.4495 10.6806 5.38532
0.00887 0.00965 0.00989 0.00618 0.00419 0.0000826 0.0117 0.0000985 0.00267
2R:6046100-6046250:minus -3.18367 1.29358 -0.0733945 2.99083 -8.90816 -17.9796 -8.73394 -7.22222
5.38532 0.0000481 0.00651 0.0119 0.00899 0.00192 0.00764 0.00243 0.0965 0.00267
2R:6046100-6046250:plus -21.6633 -1.23853 -3.09174 5.6422 -17.1531 -0.173469 -11.4679 -1.75
1.38532 0.00223 0.0154 0.0296 0.00325 0.00364 0.000456 0.00411 0.0279 0.0123
2R:6047540-6047690:minus -40.0714 -3.88073 13.4128 7.29358 -27.2347 -9.45918 -2.53211 -4.09722 -0.770642
0.0359 0.0328 0.0000141 0.00104 0.0283 0.00279 0.000505 0.0504 0.0245
2R:6047540-6047690:plus -21.8163 -2.77064 8.77064 9.7156 -8.64286 -17.7143 -1.87156 0.222222 7.22018
0.00244 0.0242 0.000287 0.000242 0.00139 0.00634 0.000431 0.0156 0.00119
2R:6115580-6115730:minus -4.63265 8 2.3945 5.77982 -17.4592 -17.7857 -9.94495 -2.86111 0.642202
0.000175 0.000381 0.00499 0.00294 0.00491 0.0068 0.00305 0.0373 0.0157
2R:6115580-6115730:plus -31.5102 1.31193 4.56881 1.91743 -17.8878 1.20408 -13.9083 5 11.8532 0.013
0.00646 0.00213 0.015 0.00704 0.0000826 0.00711 0.00263 0.0000428
2R:6139540-6139690:minus -23 -3.01835 -1.6789 3.6055 -27.8878 -8.65306 -27.8165 -5.16667 -2.22018
0.00498 0.0259 0.0196 0.00686 0.0392 0.00156 0.0671 0.0639 0.0355
2R:6139540-6139690:plus -24.1122 5.07339 3.25688 3.01835 -17.1531 -18.3061 -12.6972 3.5 3.46789
0.00738 0.00158 0.0036 0.0089 0.00364 0.0114 0.00539 0.00491 0.00582
2R:6158520-6158670:minus -22.6224 -1.81651 4.14679 5.22936 -27.3061 -9.30612 -9.58716 1 2.94495
0.00371 0.0184 0.00252 0.0041 0.0315 0.00257 0.00285 0.0121 0.00714
2R:6158520-6158670:plus -24.5918 0.495413 -0.0642202 2.04587 -26.9694 -26.8265 -7.26606 0.444444
7.18349 0.00821 0.00868 0.0118 0.014 0.023 0.0285 0.00179 0.0145 0.00121
2R:6159140-6159290:minus -22 0.266055 0.40367 -0.853211 -8.86735 -9.5 -10.5872 -1.93056 1.79817
0.00291 0.0094 0.0101 0.035 0.00173 0.00294 0.00344 0.0293 0.0102
2R:6159140-6159290:plus -22.4388 3.66972 3.00917 0.752294 -27.2347 -9.20408 -6.74312 -2.70833 5.59633
0.00348 0.00272 0.00396 0.0221 0.0283 0.00225 0.00159 0.0359 0.00234
2R:6160300-6160450:minus -22.4286 -0.715596 0.788991 3.95413 -18.0918 -17.3469 -12.0092 0.416667
5.55046 0.00345 0.0131 0.00889 0.00593 0.00761 0.0058 0.00462 0.0147 0.00241
2R:6160300-6160450:plus -22.1122 5.42202 4.23853 7 -26.898 -17.3469 -16.3761 8.45833 9.45872
0.00304 0.00137 0.00243 0.00132 0.0203 0.0058 0.0116 0.000447 0.000337
2R:616460-616610:minus -40.2551 -2.59633 -4.13761 2.69725 10.6224 -17.2755 -16.5596 1.5 -0.743119
0.0382 0.023 0.0394 0.0104 0.0000113 0.00544 0.0119 0.0103 0.0243
2R:616460-616610:plus -31.8571 -1.69725 -2.3211 3.41284 -17.7143 -17.051 -23.1468 6.26389 4.37615
0.0163 0.0178 0.0237 0.00756 0.00627 0.00479 0.0359 0.00146 0.00376
2R:6164800-6164950:minus -33.2245 7.88991 -0.59633 2.19266 -26.2653 0.826531 -20.4128 3.375 1.86239
0.0276 0.000405 0.0141 0.0131 0.018 0.000153 0.0221 0.00516 0.00994
2R:6164800-6164950:plus -21.9286 -5.3578 4.57798 0.302752 10.6939 -27.5306 -7.63303 3.65278 0.183486
0.0027 0.0484 0.00212 0.0248 7.35e-06 0.0374 0.00195 0.00462 0.0176
2R:6166500-6166650:minus -23.7857 5.31193 -0.0458716 0.0917431 -26.6633 1.05102 -16.0275 0.625
-2.66055 0.00679 0.00143 0.0118 0.0262 0.0189 0.000107 0.0109 0.0137 0.0406
2R:6166500-6166650:plus -12.6224 0.422018 -0.926606 9.7156 -18.4898 -9.45918 -9.89908 -3.51389
5.90826 0.000453 0.0089 0.0156 0.000242 0.0116 0.00279 0.00302 0.0439 0.00198
2R:6190380-6190530:minus -23 8.55963 2.19266 2.77064 -18.0816 -18.1224 -22.2294 -3.68056 11.4771
0.00498 0.000283 0.00538 0.01 0.00759 0.00988 0.0307 0.0457 0.0000679
2R:6190380-6190530:plus -22.0816 6.93578 5.83486 10.8624 -17.4184 -17.7143 -7.73394 -2.97222 -0.770642
0.003 0.000671 0.00124 0.0000687 0.00457 0.00634 0.00199 0.0384 0.0245
2R:6197280-6197430:minus -31.8163 6.21101 16.2202 1.86239 -17.1837 -18.7857 -21.7339 0.25 -0.908257

0.016	0.000958	1.24e-07	0.0153	0.00366	0.0146	0.0281	0.0155	0.0255
2R:6197280-6197430:plus	-23.3367	1.01835	-3.29358	6.52294	10.4286	-8.86735	-19.1284	8.86111	-2.87156
0.00614	0.00719	0.0313	0.00174	0.0000139	0.00198	0.0178	0.000349	0.043
2R:6309020-6309170:minus	-31.9592	-2.08257	4.20183	2.63303	-28.2347	-8.45918	-24.1376	4.43056	-1.80734
0.0167	0.0199	0.00247	0.0107	0.051	0.00111	0.0419	0.00336	0.0316
2R:6309020-6309170:plus	-31.9286	3.64286	-0.348624	-0.394495	-17.4898	-26.602	-21.1835	2.05556
6.18349	0.0166	0.00159	0.013	0.0301	0.0053	0.0248	0.0254	0.00845	0.00167
2R:6316060-6316210:minus	-21.3265	2.19266	3.76147	3.92661	-8.89796	-7.23469	-12.9633	0.388889	-7.41284
0.00193	0.00468	0.00295	0.00601	0.00189	0.000557	0.00573	0.0148	0.122
2R:6316060-6316210:plus	-34.3367	3.64286	-0.137615	5.3945	-18.1531	-27.3776	-10.0092	5.86111
3.20183	0.0318	0.00159	0.0121	0.00379	0.00829	0.0351	0.00308	0.00177	0.0065
2R:6317200-6317350:minus	-32.7755	2.99083	-0.247706	2.78899	-18.0918	-8.27551	-23.2936	1.125
-0.559633	0.0234	0.00349	0.0126	0.00992	0.00761	0.000919	0.0368	0.0117	0.0226
2R:6317200-6317350:plus	4.62245	14.6147	2.94495	4.05505	-27.1224	-7.79592	-9.94495	4.40278	-3.33028
0.0000219	4.19e-06	0.00405	0.00566	0.0245	0.000723	0.00305	0.0034	0.0478
2R:6317940-6318090:minus	-13.3367	-4.02752	3.13761	3.31193	-28.0102	-17.1224	-5.53211	-0.986111
11.8532	0.0008	0.0341	0.00377	0.00786	0.0449	0.00499	0.00115	0.0225	0.0000428
2R:6317940-6318090:plus	-30.0612	-2.77982	-7.08257	7.17431	11.6939	-35.8163	-21.7982	-1.22222	-1.11009
0.00856	0.0243	0.0816	0.00115	1.17e-06	0.0815	0.0284	0.024	0.0269
2R:631880-632030:minus	-33.2653	4.45872	9.6422	3.13761	-18.7551	-17.3061	-21.1743	3.30556	-0.798165
0.0277	0.00202	0.000176	0.00854	0.0144	0.00548	0.0254	0.0053	0.0246
2R:631880-632030:plus	-33.1122	0.275229	7.46789	6.89908	-9.5	-8.79592	-20.367	5.95833	3.51376	0.0271
0.00938	0.000575	0.00134	0.00293	0.0019	0.022	0.00169	0.00566
2R:6320040-6320190:minus	-30.1837	-1.02752	11.6422	4.17431	-18.449	-25.5306	-18.1835	-1.76389	9.92661
0.00873	0.0145	0.0000543	0.00537	0.0107	0.0205	0.0154	0.028	0.000193
2R:6320040-6320190:plus	-32.7755	0.844037	12.7339	0.46789	-28	-26.898	-22.6147	3.77778	-0.284404
0.0234	0.00766	0.0000257	0.0239	0.0444	0.0302	0.0329	0.0044	0.0207
2R:6324840-6324990:minus	-31.6327	13.5306	-0.256881	2.34862	-17.4898	-8.79592	-21.8716	0.652778
-10.5321	0.0138	0.0000206	0.0126	0.0121	0.0053	0.0019	0.0288	0.0136	0.214
2R:6324840-6324990:plus	-40.8878	2.58716	0.981651	4.79817	-16.449	-0.469388	-15.1193	11.4583
1.16514	0.0439	0.00404	0.00832	0.00453	0.00307	0.000533	0.00919	0.0000512	0.0135
2R:6325400-6325550:minus	-21.1837	-4.88073	12.6055	0.944954	-18.4898	-26.1224	-9.6789	-1.16667	0.642202
0.00189	0.0428	0.000028	0.0209	0.0116	0.0217	0.0029	0.0237	0.0157
2R:6325400-6325550:plus	-14.0714	0.137615	16.2202	7.47706	-26.8571	-18.1224	4.43119	-3.66667	11.8532
0.00117	0.00984	1.24e-07	0.00085	0.0198	0.00988	0.0000705	0.0455	0.0000428
2R:6355920-6356070:minus	-33.2653	7.36697	-1.73394	0.0917431	2.87755	-9.5	-12.156	3.02778	2.37615
0.0277	0.000535	0.02	0.0262	0.0000259	0.00294	0.00477	0.0059	0.00825
2R:6355920-6356070:plus	-13.7449	8.56881	0.853211	2.77064	-18.4592	-17.9388	-5.55046	3.33333	3.62385
0.00107	0.000281	0.0087	0.01	0.0109	0.00738	0.00116	0.00525	0.00523
2R:6362980-6363130:minus	-22.6939	3.97959	1.85321	9.51376	-8.16327	-18.7857	-3.27523	9.06944	6.26606
0.00388	0.000669	0.0061	0.000292	0.000873	0.0146	0.000611	0.000307	0.00162
2R:6362980-6363130:plus	-23.2347	5.50459	2.55046	3.76147	-9.0102	-8.5	-3.57798	-2.54167	7.15596
0.00577	0.00132	0.00471	0.00645	0.00236	0.00126	0.000663	0.0344	0.00124
2R:6365300-6365450:minus	-31.551	4.81651	5.47706	-0.642202	-8.63265	-27.3061	-15.3211	-1.22222
10.1284	0.0134	0.00175	0.00145	0.0328	0.0013	0.0336	0.00956	0.024	0.000129
2R:6365300-6365450:plus	-31.2143	3.46789	1.90826	2.62385	-27.1939	-18.1531	-15.9817	4.72222	3.31193
0.0111	0.00293	0.00598	0.0108	0.0267	0.00997	0.0108	0.00296	0.00618
2R:6366020-6366170:minus	-20.551	-2.38532	-1.54128	10.0275	-8.93878	-8.45918	3.06422	-2.27778	0.348624
0.00168	0.0217	0.0188	0.000156	0.00216	0.00111	0.000116	0.0321	0.0167
2R:6366020-6366170:plus	-23.2653	-4.18349	1.86239	1.06422	-18.4898	-37.1531	0.862385	-2.15278	9.92661
0.00593	0.0355	0.00608	0.0203	0.0116	0.138	0.000229	0.0311	0.000193
2R:6367560-6367710:minus	-23.9184	2.77064	0.926606	0.238532	-17.7551	-18.3469	-10.8073	3.59722	1.48624
0.00695	0.00378	0.00848	0.0251	0.00658	0.0124	0.00359	0.00473	0.0118
2R:6367560-6367710:plus	-32.7041	0.889908	4.21101	3.26606	-18.3776	-9.82653	-20.7431	-0.486111

-0.798165 0.0222 0.00753 0.00246 0.00802 0.00926 0.00381 0.0235 0.0194 0.0246
2R:6416120-6416270:minus -13.8571 4.33028 0.449541 3.23853 -18.7551 -10.0204 -14.9541 6.23611 -3.12844
0.0011 0.00212 0.00998 0.00815 0.0144 0.00399 0.00889 0.00148 0.046
2R:6416120-6416270:plus -22.2959 -1.55963 3.06422 1.56881 -9.0102 -9.72449 -17.0826 5.93056 2.15596
0.00335 0.017 0.00387 0.0172 0.00236 0.00352 0.013 0.00171 0.00881
2R:6419140-6419290:minus -23.3367 -0.587156 -0.174312 4.22018 -28.602 0.826531 -9.05505 4.34722
7.6422 0.00614 0.0126 0.0123 0.00531 0.0631 0.000153 0.00258 0.00348 0.000893
2R:6419140-6419290:plus -32.9694 -3.68807 0.504587 2.27523 -27.5306 -8.87755 -17.4404 -0.541667
0.972477 0.0254 0.0311 0.0098 0.0125 0.0351 0.00198 0.0137 0.0197 0.0144
2R:6421760-6421910:minus -31.6633 6.98165 5.23853 7.80734 -8.96939 -9.16327 -11.367 0.166667 2.49541
0.0141 0.000656 0.00161 0.00063 0.00226 0.00223 0.00403 0.0159 0.00794
2R:6421760-6421910:plus -24.1122 -1.08257 -2.06422 7.80734 -17.8571 -26.3776 -12.5505 7.125 9.55046
0.00738 0.0147 0.022 0.00063 0.00698 0.0222 0.00522 0.000942 0.000321
2R:6422480-6422630:minus -12.2653 1.22018 1.13761 9.41284 -7.89796 -17.2041 -18.5688 -4.5 -4.42202
0.000381 0.00668 0.00788 0.000354 0.000678 0.00507 0.0163 0.0552 0.0626
2R:6422480-6422630:plus -14.5918 -2.20183 0.87156 0.211009 -17.7551 -27.3367 -11.8991 -2.01389 9.77064
0.00148 0.0206 0.00864 0.0254 0.00658 0.0347 0.00451 0.0299 0.000228
2R:6429160-6429310:minus -23.449 3.72477 4.18349 11.0183 -26.9286 0.316327 -15.2752 -2.63889 -0.678899
0.00631 0.00266 0.00249 0.000052 0.0214 0.000243 0.00948 0.0353 0.0237
2R:6429160-6429310:plus -22.9286 -0.623853 2.97248 7.12844 -18.1531 -7.72449 -22.7156 -0.472222
0.59633 0.0046 0.0127 0.00401 0.00118 0.00829 0.00068 0.0334 0.0193 0.016
2R:6445160-6445310:minus -31.7041 0.495413 2.30275 6.74312 -28.2347 -8.5 -26.9908 6.40278 2.36697
0.0146 0.00868 0.00517 0.0015 0.051 0.00126 0.0604 0.00136 0.00828
2R:6445160-6445310:plus -29.9184 2.27523 1.6789 1.77982 -17.8878 -9.02041 -20.1009 3.54167 5.70642
0.00854 0.00454 0.0065 0.0158 0.00704 0.00213 0.021 0.00483 0.00222
2R:6445620-6445770:minus -22.9184 7.61468 -3.09174 0.651376 -8.63265 -9.02041 -17.789 1.875 -0.550459
0.00447 0.00047 0.0296 0.0226 0.0013 0.00213 0.0145 0.00901 0.0225
2R:6445620-6445770:plus -13.2245 1.04587 0.889908 1.06422 -36.1429 -25.5306 -13.8716 2.59722 7.62385
0.000738 0.00712 0.00859 0.0203 0.0741 0.0205 0.00705 0.00694 0.000941
2R:6453440-6453590:minus -31.4694 3.90816 -0.733945 0.816514 -27.1939 -18.8571 -13.945 8.55556
1.27523 0.0124 0.00102 0.0147 0.0216 0.0267 0.0151 0.00717 0.000422 0.013
2R:6453440-6453590:plus -31.5102 4.48624 7.50459 5.93578 -8.93878 -9.94898 -15.2018 2.19444 -4.55046
0.013 0.00199 0.000565 0.00264 0.00216 0.00393 0.00934 0.00804 0.0646
2R:6454180-6454330:minus -31.551 2.06422 -1.63303 3.69725 -17.0408 -17.2755 -19.0734 0.208333 5.44037
0.0134 0.0049 0.0194 0.00665 0.00329 0.00544 0.0177 0.0157 0.00259
2R:6454180-6454330:plus -31.7755 -5.23853 7.7156 10.0459 -18.1837 -26.8571 -11.9725 -6.75 0.174312
0.0157 0.047 0.000509 0.000139 0.00835 0.0288 0.00458 0.0883 0.0177
2R:6456400-6456550:minus -14.5204 2.46789 2.49541 7.78899 -7.60204 -27.4082 -7.74312 2.63889 2.75229
0.00145 0.00422 0.00481 0.000642 0.000466 0.0356 0.00199 0.00684 0.00748
2R:6456400-6456550:plus -31.4388 4.91743 -0.926606 6.47706 -17.1122 -8.5 -22.211 -1.90278 -8.01835
0.0122 0.00169 0.0156 0.00179 0.00341 0.00126 0.0306 0.029 0.136
2R:6457080-6457230:minus -14.5204 -0.788991 4.86239 6.6422 -27.0408 -28.6327 -13.9633 -0.444444
12.2752 0.00145 0.0134 0.00188 0.00161 0.0242 0.066 0.0072 0.0192 0.0000141
2R:6457080-6457230:plus -22.8571 -1.37615 -2.62385 3.88991 -8.86735 -9.57143 -28.2752 1.72222 -1.36697
0.00427 0.0161 0.0259 0.00608 0.00173 0.00326 0.0714 0.0095 0.0286
2R:6488240-6488390:minus -4.85714 -4.10092 -0.00917431 2.44954 -18.051 -17.051 -15.9633 2.72222
2.54128 0.000202 0.0348 0.0116 0.0116 0.00746 0.00479 0.0108 0.00663 0.00787
2R:6488240-6488390:plus -31.7755 1.50459 0.59633 5.43119 -18.7245 9.60204 -20.9541 -1.5 7.18349
0.0157 0.00602 0.00949 0.00371 0.0136 0.0000248 0.0244 0.026 0.00121
2R:6488620-6488770:minus -40.0306 -0.449541 1.08257 6.31193 -18.6735 -17.051 -16.367 6.75 -0.642202
0.0357 0.012 0.00803 0.00199 0.0123 0.00479 0.0116 0.00114 0.0234
2R:6488620-6488770:plus -32.1939 -3.77064 -4.24771 1.69725 -9.20408 -16.9388 -0.623853 8.80556
-2.22018 0.0181 0.0318 0.0406 0.0163 0.00262 0.00433 0.000326 0.000362 0.0355
2R:6489460-6489610:minus -21.398 -0.651376 -2.17431 9.23853 -8.33673 -26.2653 -18.0183 -3.26389

1.72477 0.00197 0.0128 0.0227 0.000424 0.00101 0.0218 0.015 0.0413 0.0106
2R:6489460-6489610:plus -22.1939 -1.61468 7.7156 10.8624 -26.6327 -8.45918 -9.54128 -2.38889 -1.36697
0.00317 0.0173 0.000509 0.0000687 0.0188 0.00111 0.00283 0.0331 0.0286
2R:6490520-6490670:minus -14.6735 4.69725 12.8257 3.90826 -26.7041 -7.57143 -13.0092 -1.19444 11.4771
0.00152 0.00184 0.0000239 0.00603 0.0193 0.000644 0.00579 0.0239 0.0000679
2R:6490520-6490670:plus -23.4592 -1.77064 3.73394 8.77982 -17.6735 -17.7551 -20.156 -4.55556 2.73394
0.00631 0.0182 0.00299 0.000533 0.00572 0.00643 0.0212 0.0559 0.00756
2R:6505020-6505170:minus -41.2857 7.05505 1.2844 -0.146789 -18.7143 -18.2755 -29.1743 3.19444
-8.2844 0.0521 0.00063 0.00748 0.028 0.0136 0.0112 0.0809 0.00554 0.143
2R:6505020-6505170:plus -20.7755 -4.6055 -0.743119 5.37615 -17.7143 0.27551 -14.7064 0.0694444
5.22936 0.00176 0.0398 0.0147 0.00384 0.00627 0.000257 0.00845 0.0164 0.00288
2R:6505920-6506070:minus -32.2551 -1.74312 0.66055 1.56881 -16.7143 1.82653 -17.8257 0.333333 3.45872
0.0184 0.018 0.00929 0.0172 0.00319 0.0000498 0.0146 0.0151 0.00591
2R:6505920-6506070:plus -40.551 -3.3945 14.6147 3.19266 -9.20408 -26.7857 -13.0734 -1.15278 9.61468
0.0418 0.0287 3.32e-06 0.00829 0.00262 0.0266 0.00588 0.0236 0.00029
2R:6533100-6533250:minus -13.7755 2.59633 11.0459 6.44954 20.2041 -9.23469 -3.43119 2.61111 1.58716
0.00107 0.00403 0.0000777 0.00183 3.64e-07 0.00238 0.000637 0.00691 0.0113
2R:6533100-6533250:plus -33 -3.44037 8.38532 2.41284 -18.4184 -19.3061 -15.6422 3.47222 1.89908
0.0258 0.0291 0.000356 0.0118 0.0101 0.0187 0.0102 0.00497 0.0098
2R:6618920-6619070:minus -22.9592 -2.6789 13.4862 7.45872 -27.602 10.8265 -3.62385 4.19444 5.38532
0.00475 0.0236 0.0000132 0.000894 0.0364 2.5e-06 0.000671 0.00371 0.00267
2R:6618920-6619070:plus -33.7347 3.97959 1.37615 4.81651 11.3571 -16.5 -16.3211 -0.805556
1.21101 0.0294 0.000669 0.00724 0.00451 2.44e-06 0.00416 0.0115 0.0213 0.0134
2R:6627340-6627490:minus -33.3367 8.79817 -1.52294 1.06422 10.0918 -18.2755 -11.9633 1.06944 2.15596
0.0281 0.000249 0.0187 0.0203 0.0000251 0.0112 0.00457 0.0119 0.00881
2R:6627340-6627490:plus -24.3265 2.56881 -2.85321 1.38532 -18.9796 -7.79592 -11.0734 -1.94444 -0.917431
0.00784 0.00407 0.0277 0.0185 0.0155 0.000723 0.00379 0.0294 0.0256
2R:6662180-6662330:minus -31.5408 7.41284 2.3211 9.41284 -26.3061 -17.8571 -3.19266 -0.791667
-1.88991 0.0131 0.000521 0.00513 0.000354 0.0181 0.00719 0.000598 0.0212 0.0322
2R:6662180-6662330:plus -22.0816 1.77982 0.422018 6.3578 -18.4898 -9.7551 -27.1651 6.68056 -2.41284
0.003 0.00544 0.0101 0.00195 0.0116 0.00358 0.0618 0.00119 0.0376
2R:6664980-6665130:minus -29.7755 0.00917431 9.69725 5.74312 -18.6429 -6.5 -10.5413 1.18056 -4.2844
0.0085 0.0103 0.00017 0.00302 0.0121 0.000542 0.00341 0.0114 0.0597
2R:6664980-6665130:plus -22.2653 3.0367 1.85321 6.84404 -18.4592 -17.2755 -11.055 12.6667 6.26606
0.00331 0.00343 0.0061 0.0014 0.0109 0.00544 0.00377 0.0000153 0.00162
2R:6669540-6669690:minus -30.4388 3.64286 5.36697 1.21101 -17.4898 -16.2755 -23.9908 7.63889 -2.79817
0.00923 0.00159 0.00152 0.0194 0.0053 0.00413 0.041 0.000715 0.0423
2R:6669540-6669690:plus -31.7347 -0.330275 0.422018 -0.0458716 -27.7449 -17.2755 -16.7798 4.88889
3.46789 0.0149 0.0115 0.0101 0.0271 0.0383 0.00544 0.0124 0.00276 0.00582
2R:6670140-6670290:minus -34.4082 0.311927 -3.89908 2.54128 -18.8265 -8.23469 -16.6697 0.875
-0.0825688 0.0319 0.00925 0.037 0.0112 0.0149 0.000875 0.0122 0.0127 0.0189
2R:6670140-6670290:plus -29.9184 3.68367 16.2202 -0.412844 10.9184 -9.23469 -15.3578 -0.680556
7.15596 0.00854 0.00144 1.24e-07 0.0304 4.19e-06 0.00238 0.00963 0.0206 0.00124
2R:6696400-6696550:minus -13 0.504587 3.53211 10.0275 -18.7551 -8.72449 2.11009 3.27778 1.78899
0.000563 0.00865 0.00323 0.000156 0.0144 0.00169 0.000159 0.00536 0.0103
2R:6696400-6696550:plus -22.8061 1.04587 8.94495 5.89908 -18.4898 -27.6327 -6.95413 -2.40278 7.6422
0.00414 0.00712 0.000262 0.00272 0.0116 0.043 0.00167 0.0332 0.000893
2R:6697000-6697150:minus -33 -0.899083 3.21101 1.2844 -17.4184 -18.5714 -18.2385 7.56944 6.11927
0.0258 0.0139 0.00366 0.019 0.00457 0.0136 0.0155 0.000743 0.00178
2R:6697000-6697150:plus -23.0306 -2.05505 1.88991 5.85321 -18.9796 -17.8571 -1.77982 -7.45833 1.45872
0.00505 0.0197 0.00602 0.00287 0.0155 0.00719 0.000422 0.101 0.0119
2R:6698400-6698550:minus -32.2041 1.3578 1.66972 -1.6055 -17.4184 -7.72449 -15.4037 4.38889 -1.83486
0.0181 0.00635 0.00652 0.0448 0.00457 0.00068 0.00972 0.00342 0.0318
2R:6698400-6698550:plus -31.9286 13.5306 -2.77982 2.62385 -8.93878 -0.316327 -14.6606 2.51389

-0.449541	0.0166	0.0000206	0.0271	0.0108	0.00216	0.000524	0.00837	0.00716	0.0216
2R:6700960-6701110:minus	-13.5918	-1.6789	0.834862	3.30275	-18.3776	-27.602	-4.88991	4.97222	-1.68807
0.00099	0.0177	0.00875	0.00789	0.00926	0.0416	0.000962	0.00266	0.0308
2R:6700960-6701110:plus	-32.7347	8.17431	1.30275	2.43119	-18.4184	-27.6735	-19.5229	1.11111	1.24771
0.0225	0.000347	0.00743	0.0117	0.0101	0.0443	0.019	0.0117	0.0131
2R:6707440-6707590:minus	-31.6735	-4.53211	2.3578	2.44954	-27.1224	0.826531	-20.1927	4.375	-2.62385
0.0141	0.039	0.00506	0.0116	0.0245	0.000153	0.0213	0.00344	0.0402
2R:6707440-6707590:plus	-23.449	1.26606	3.07339	2.0367	10.6939	-8.23469	-19.8349	2.43056	0.394495
0.00631	0.00657	0.00386	0.0141	7.35e-06	0.000875	0.02	0.00738	0.0166
2R:6709060-6709210:minus	-24.4082	14.156	4.94495	5.94495	-26.2653	-18.0816	-18.0917	6.41667	5.70642
0.00797	6.98e-06	0.00182	0.00261	0.018	0.00934	0.0152	0.00135	0.00222
2R:6709060-6709210:plus	-30.3673	-4	3.6789	1.40367	-18.7857	-9.23469	-19.2385	0.277778	5.9633
0.00897	0.0338	0.00305	0.0184	0.0148	0.00238	0.0181	0.0153	0.00188
2R:6711000-6711150:minus	-40.0204	1.02752	11.6881	2.09174	-17.9388	-9.7551	-10.0917	-2.79167	5.01835
0.0353	0.00717	0.0000527	0.0137	0.00715	0.00358	0.00313	0.0367	0.00309
2R:6711000-6711150:plus	-4.14286	-2.09174	3.65138	2.74312	-27.7959	-8.02041	-10.5963	3.125	-0.40367
0.000127	0.0199	0.00308	0.0101	0.0386	0.000761	0.00345	0.00569	0.0213
2R:6726180-6726330:minus	-32.3673	-3.18349	2.58716	3.44037	-18.7857	-8.68367	-21.0642	3.55556	-0.862385
0.0189	0.0271	0.00465	0.00746	0.0148	0.00157	0.0249	0.00481	0.0251
2R:6726180-6726330:plus	-33.2245	-2.77982	5.42202	7.37615	20.4694	-8.72449	-19.0642	2.11111	7.25688
0.0276	0.0243	0.00149	0.000951	1.65e-07	0.00169	0.0176	0.00828	0.00115
2R:6753980-6754130:minus	-4.89796	0.66055	7.20183	10.8624	-26.9694	-9.45918	-2.38532	-1.65278	-0.87156
0.000208	0.00818	0.000658	0.0000687	0.023	0.00279	0.000487	0.0271	0.0252
2R:6753980-6754130:plus	-22.7347	3.27523	5.66055	5.7156	-17.449	-27.898	-5.98165	2.09722	3.62385
0.00403	0.00314	0.00134	0.00309	0.00484	0.0537	0.0013	0.00832	0.00523
2R:6754880-6755030:minus	-23.2551	-1.45872	0.53211	6.44037	-16.449	-26.3776	-7.90826	-1.84722	0.486239
0.00583	0.0165	0.00971	0.00185	0.00307	0.0222	0.00206	0.0286	0.0163
2R:6754880-6755030:plus	-30.3265	3.90816	1.3211	3.26606	-9.23469	-17.5714	-16.3761	4.44444	3.98165
0.0089	0.00102	0.00738	0.00802	0.00277	0.00616	0.0116	0.00334	0.00439
2R:6758500-6758650:minus	-31.8469	-2.52294	1.94495	0.40367	-17.4184	-16.2041	-9.17431	6.75	7.22018
0.0162	0.0226	0.0059	0.0242	0.00457	0.00408	0.00264	0.00114	0.00119
2R:6758500-6758650:plus	-24.2551	0.431193	-2.82569	2.66055	-17.5306	-27.6735	-14.6147	0.513889	1.78899
0.00768	0.00887	0.0275	0.0106	0.0054	0.0443	0.00829	0.0142	0.0103
2R:6762180-6762330:minus	-3.04082	1.19266	1.48624	9.49541	-17.3469	-8.16327	-19.2569	5.45833	-0.816514
0.0000423	0.00675	0.00696	0.000308	0.00394	0.000783	0.0182	0.00214	0.0249
2R:6762180-6762330:plus	-23.2245	-0.715596	1.13761	2.25688	-17.1531	-0.0204082	-8.31193	1.80556
-0.311927	0.00576	0.0131	0.00788	0.0127	0.00364	0.000411	0.00224	0.00923	0.0208
2R:676220-676370:minus	-41.4796	-0.963303	0.889908	6.00917	10.0918	-17.0816	-19.7798	7.76389
-6.29358	0.0577	0.0142	0.00859	0.00255	0.0000251	0.00487	0.0198	0.000666	0.0955
2R:676220-676370:plus	-30.8061	-1.48624	1.54128	3.46789	-7.56122	-17.2755	-11.2202	5.11111	2.88991
0.0102	0.0167	0.00683	0.00726	0.000419	0.00544	0.0039	0.0025	0.00725
2R:6763160-6763310:minus	-40.551	3.90816	-1.38532	3.27523	-17.9796	0.602041	-20.2202	-0.625	-0.440367
0.0418	0.00102	0.018	0.00796	0.00726	0.000188	0.0214	0.0202	0.0216
2R:6763160-6763310:plus	-22.2245	3.7551	-0.183486	5.86239	10.3571	0.0510204	-24.1009	3.84722
1.95413	0.00325	0.00122	0.0123	0.00282	0.0000208	0.000349	0.0417	0.00428	0.00964
2R:6767100-6767250:minus	-39.2143	2.06422	0.559633	6.00917	11.4286	0.795918	-10.844	3.40278	-4.11927
0.0329	0.0049	0.00961	0.00255	1.97e-06	0.000155	0.00362	0.00511	0.0572
2R:6767100-6767250:plus	-13.4184	3.77982	-0.477064	7.3578	-9.23469	-17.8265	-15.2661	2.13889
-0.715596	0.000873	0.00261	0.0135	0.000965	0.00277	0.00699	0.00946	0.0082	0.0241
2R:6774220-6774370:minus	-33.6327	1.3578	8.33028	-0.165138	-28.1939	-0.397959	-22.211	6.86111
1.16514	0.029	0.00635	0.000367	0.0282	0.0482	0.000525	0.0306	0.00108	0.0135
2R:6774220-6774370:plus	-12.7347	0.293578	-2.74312	2.70642	-19.0102	-9.72449	-13.211	0.486111	1.70642
0.000468	0.00932	0.0268	0.0103	0.0157	0.00352	0.00607	0.0144	0.0108
2R:6786520-6786670:minus	-29.3673	-1.52294	-2.13761	1.59633	0.612245	-8.5	-22.7064	2.77778	3.30275

0.00838	0.0169	0.0225	0.017	0.000269 0.00126	0.0334	0.00649	0.00619
2R:6786520-6786670:plus	-23.2653 1.22936	4.0367	0.926606 -7.93878	-26.8265	-22.1927	3.56944	9.45872
0.00593	0.00666	0.00264	0.0209	0.000767 0.0285	0.0305	0.00478	0.000337
2R:6933800-6933950:minus	-23.0714	-0.816514	10.3761	3.62385	-7.67347	-27.3776	-15.2202	-4.58333
2.6422	0.0052	0.0135	0.000113 0.00673	0.00055	0.0351	0.00937	0.0563	0.00768
2R:6933800-6933950:plus	-23.2245	0.119266 4.90826	7	-16.7143	-18.7857	-18.1743	-1.27778	-0.541284
0.00576	0.0099	0.00185	0.00132	0.00319	0.0146	0.0154	0.0244	0.0223
2R:6953180-6953330:minus	-23.2551	-0.844037	0.733945 5.49541	-8.89796	-26.9694	-8.2844	5.22222
-0.440367	0.00583	0.0136	0.00906	0.00355	0.00189	0.0304	0.00223	0.00238	0.0216
2R:6953180-6953330:plus	-12.3061	-0.623853	0.53211	0.651376 -18.4184	-8.42857	-23.211	2.02778
-6.3578	0.000385 0.0127	0.00971	0.0226	0.0101	0.00104	0.0363	0.00853	0.097
2R:6958180-6958330:minus	-24.0306	5.93578	9.05505	3.04587	-18.4184	-18.0816	-2.08257	-2.91667
-0.0733945	0.00723	0.00109	0.000246 0.00883	0.0101	0.00934	0.000453 0.0378	0.0188
2R:6958180-6958330:plus	14.7755	2.94495	5.89908	7.47706	-18.3469	-18.7143	-13.422	-3.43056	-9.10092
8.5e-07	0.00355	0.00121	0.00085	0.00894	0.014	0.00637	0.043	0.169
2R:6959320-6959470:minus	-32.551	3.65138	5.40367	5.49541	-27	-19.051	-16.8807	-1.13889	3.62385
0.0207	0.00274	0.0015	0.00355	0.0233	0.0165	0.0126	0.0235	0.00523
2R:6959320-6959470:plus	-22.1531	-0.357798	6.51376	10.8624	-18.1531	-18.0816	-21.4679	1.16667
-0.422018	0.0031	0.0116	0.000912 0.0000687	0.00829	0.00934	0.0268	0.0115	0.0213
2R:6976680-6976830:minus	-32.0102	1.49541	2.26606	7.27523	-8.93878	-9.57143	-24	0.680556 4.51376
0.0172	0.00604	0.00524	0.00109	0.00216	0.00326	0.041	0.0135	0.0036
2R:6976680-6976830:plus	-41.7653	-0.981651	-2.84404 2.3945	-27.5306	-9.72449	-16.3303	9.59722
7.94495	0.0647	0.0143	0.0276	0.0119	0.0351	0.00352	0.0115	0.000217 0.000667
2R:6990660-6990810:minus	-31.1837	-0.0458716	-2.07339 6.47706	-27.2347	-9.7551	-24.6606	-2.77778
2.6422	0.0109	0.0105	0.0221	0.00179	0.0283	0.00358	0.0451	0.0365	0.00768
2R:6990660-6990810:plus	-23.9592	-0.513761	2.85321	5.69725	-8.40816	-17.2755	-15.5413	-2.55556
-0.715596	0.00708	0.0123	0.0042	0.00314	0.00108	0.00544	0.00997	0.0345	0.0241
2R:6994260-6994410:minus	-23.8878	5.17431	0.449541 1.56881	-17.7143	-19.6429	-10.6514	-2.22222	5.22936
0.00692	0.00151	0.00998	0.0172	0.00627	0.0204	0.00348	0.0316	0.00288
2R:6994260-6994410:plus	-33.7449	-2.97248	4.09174	3.05505	-27.1939	-8.86735	-28.2844	2.33333	2.20183
0.0295	0.0256	0.00258	0.00878	0.0267	0.00198	0.0715	0.00765	0.00869
2R:7037440-7037590:minus	-33.0714	0.743119 13.8532	2.62385	-8.86735	-17.3469	-23.3578	0.916667 -4.24771
0.0267	0.00794	8.77e-06	0.0108	0.00173	0.0058	0.0372	0.0125	0.0591
2R:7037440-7037590:plus	-24	13.3878	15.0183	4.04587	-18.1122	-17.4184	-15.367	1.15278	-2.63303
0.00719	0.0000869	1.42e-06	0.00572	0.00771	0.00585	0.00965	0.0115	0.0404
2R:7061140-7061290:minus	-22.7755	6.58716	6.01835	7.70642	-27.4592	-9.94898	-12.1376	-3.97222	9.45872
0.00412	0.000799 0.00114	0.000708 0.0329	0.00393	0.00475	0.0489	0.000337
2R:7061140-7061290:plus	-22.3571	1.82569	-2.41284	2.23853	-18.0816	-18.9796	-9.07339	-3.43056	-1.06422
0.00339	0.00535	0.0244	0.0128	0.00759	0.0155	0.00259	0.043	0.0266
2R:7063420-7063570:minus	-22.0408	1.61468	4.55963	5.98165	-18.5204	-9.53061	-16.8899	-1.58333	-4.29358
0.00296	0.00578	0.00214	0.00258	0.0117	0.0031	0.0126	0.0266	0.06
2R:7063420-7063570:plus	-32.4694	3.99083	1.92661	1.43119	-27.2653	-9.79592	-19.8073	4.83333	-2.34862
0.0196	0.00241	0.00594	0.0181	0.0302	0.00377	0.0199	0.00283	0.0368
2R:7071940-7072090:minus	-22.4388	-2.05505	1.89908	3.13761	0.877551 -27.3367	-12.5413	4.58333	11.8532
0.00348	0.0197	0.006	0.00854	0.00019	0.0347	0.00521	0.00315	0.0000428
2R:7071940-7072090:plus	-31.398	-0.165138	0.522936 -0.844037	-18.4898	-7.79592	-20.6239
-0.0416667	-2.77982 0.0117	0.0109	0.00974	0.035	0.0116	0.000723 0.023	0.017	0.042
2R:7073280-7073430:minus	-23.6327	3.68367	5.81651	6.55963	-18.7551	-9.7551	-10.0367	0.819444 9.92661
0.00661	0.00144	0.00125	0.00172	0.0144	0.00358	0.0031	0.0129	0.000193
2R:7073280-7073430:plus	-30.551	2.17431	3.40367	2.19266	-27.2347	0.204082 -17.2477	5.48611	5.13761
0.00959	0.00471	0.0034	0.0131	0.0283	0.000296 0.0133	0.00211	0.00298
2R:7075180-7075330:minus	-32.9286	4.44037	-0.0458716	11.2936	-8.60204	-8.94898	-16.3028	-0.861111
-3.34862 0.025	0.00203	0.0118	0.0000139	0.00125	0.00203	0.0114	0.0217	0.048
2R:7075180-7075330:plus	-14.2245	3.90816	-1.26606	2.08257	-27.2653	-16.5714	-9.57798	4.31944	0.330275

0.00126 0.00102 0.0173 0.0137 0.0302 0.00417 0.00285 0.00352 0.0168
2R:7079660-7079810:minus -31.2449 0.614679 -1.01835 5.55046 -26.8571 -25.898 -8.56881 4 3.66972
0.0111 0.00832 0.0161 0.00345 0.0198 0.0214 0.00236 0.00402 0.00514
2R:7079660-7079810:plus -21.9184 1.89908 3.3578 7.72477 -8.93878 -16.051 -12.7339 -5.09722 3.44954
0.00261 0.00521 0.00346 0.000681 0.00216 0.00408 0.00544 0.063 0.00594
2R:7080620-7080770:minus -4.85714 3.41284 0.201835 5.58716 0.612245 -18.8265 -19.1468 -7.25 3.51376
0.000202 0.00299 0.0108 0.00334 0.000269 0.0148 0.0179 0.097 0.00566
2R:7080620-7080770:plus -30.7755 -3 4.14679 6.14679 -19.051 -16.9388 -16.8073 5.34722 -2.9633
0.0102 0.0258 0.00252 0.00224 0.0162 0.00433 0.0124 0.00225 0.0442
2R:7084360-7084510:minus -22.2857 0.302752 5.86239 8.77982 -18.7143 0.826531 -13.2752 7.65278 3.52294
0.00331 0.00929 0.00122 0.000533 0.0136 0.000153 0.00616 0.000709 0.00558
2R:7084360-7084510:plus -32.551 0.0275229 0.486239 0.587156 -19.0102 -18.4184 -14.7523 0.972222
4.6055 0.0207 0.0102 0.00986 0.0231 0.0157 0.0127 0.00853 0.0123 0.00352
2R:7084820-7084970:minus -13.449 3.97959 4.53211 2.66972 -26.8571 -17.9388 -19.7156 2.79167 1.78899
0.000914 0.000669 0.00216 0.0106 0.0198 0.00738 0.0196 0.00646 0.0103
2R:7084820-7084970:plus -22.1224 -4.23853 1.18349 5.57798 -8.37755 -9.42857 -21.9358 -7.69444 -4.36697
0.00304 0.0361 0.00775 0.0034 0.00106 0.0027 0.0291 0.105 0.0615
2R:7086360-7086510:minus -31.551 -0.779817 -6.20183 3.18349 -26.8163 -0.469388 -30.578 3.02778
8.20183 0.0134 0.0134 0.0665 0.00837 0.0195 0.000533 0.0985 0.0059 0.000539
2R:7086360-7086510:plus 5.36735 -2.77982 1.23853 5.33028 -17.4898 -8.79592 -15.1743 -0.472222
7.6422 0.0000111 0.0243 0.0076 0.00389 0.0053 0.0019 0.00929 0.0193 0.000893
2R:7096180-7096330:minus -21.9286 1.56881 2.24771 6.21101 -18.3469 -9.20408 -19.6606 -6.5 -1.79817
0.0027 0.00588 0.00527 0.00213 0.00894 0.00225 0.0194 0.0841 0.0315
2R:7096180-7096330:plus -29.1429 -0.963303 4.31193 3.53211 -17.1224 -36.0408 6.23853 0.0277778
2.84404 0.00834 0.0142 0.00236 0.00704 0.00343 0.0837 0.0000385 0.0166 0.00736
2R:7106860-7107010:minus -21.5102 -2.36697 7.2844 4.0367 -17.2653 -17.8571 -12.6422 6.86111 4.11927
0.00202 0.0216 0.000632 0.00578 0.00387 0.00719 0.00533 0.00108 0.00411
2R:7106860-7107010:plus -22.8878 1.79817 -0.972477 1.56881 1.54082 0.316327 -14.0092 0.0972222
1.90826 0.00441 0.00541 0.0158 0.0172 0.0000712 0.000243 0.00727 0.0162 0.00974
2R:7110200-7110350:minus -23.1429 3.90816 12.4404 -0.266055 -18.5306 -18.2755 -12.9725 -3.68056
9.61468 0.00533 0.00102 0.0000315 0.029 0.0118 0.0112 0.00574 0.0457 0.00029
2R:7110200-7110350:plus -29.7755 -0.155963 7.44037 7.21101 -27.9694 0.0918367 -9.41284 -5.75
5.90826 0.0085 0.0109 0.000584 0.0011 0.0437 0.000323 0.00276 0.0723 0.00198
2R:7110900-7111050:minus -32.6939 -0.697248 1.10092 -0.165138 -17.449 -19.3469 -12.2569 3.45833
-1.75229 0.0217 0.013 0.00798 0.0282 0.00484 0.0194 0.00488 0.005 0.0312
2R:7110900-7111050:plus -22.9694 -0.119266 2.94495 0.862385 -18.8265 -9.79592 -17.1927 2.68056
1.16514 0.0048 0.0107 0.00405 0.0214 0.0149 0.00377 0.0132 0.00673 0.0135
2R:711800-711950:minus -23.1531 5.25688 3.20183 5.94495 -17.9286 -17.7857 -17.2661 3.55556 -0.954128
0.00542 0.00146 0.00367 0.00261 0.0071 0.0068 0.0134 0.00481 0.0258
2R:711800-711950:plus -32.9694 4.11927 -5.86239 2.33945 -18.7143 -8.72449 -22.3853 8.88889 9.55046
0.0254 0.0023 0.0612 0.0122 0.0136 0.00169 0.0316 0.000344 0.000321
2R:716680-716830:minus -40.7449 3.68367 -1.79817 5.22936 -18.6429 -0.0510204 -28.3119 1.73611
-2.34862 0.0431 0.00144 0.0204 0.0041 0.0121 0.000429 0.0717 0.00946 0.0368
2R:716680-716830:plus -23.2653 3.64286 0.266055 5.43119 -17.7959 -9.86735 -11.1835 2.94444 3.29358
0.00593 0.00159 0.0106 0.00371 0.00678 0.00391 0.00387 0.00609 0.00623
2R:7167120-7167270:minus -31.6224 13.6422 14.1927 7.29358 1.87755 -18.0816 -13.7339 0.416667 -2.11009
0.0137 0.0000127 5.79e-06 0.00104 0.0000492 0.00934 0.00684 0.0147 0.0343
2R:7167120-7167270:plus -31.898 3.68367 0.587156 6.55963 -17.449 -17.5714 -23.4771 -1.125 0.348624
0.0164 0.00144 0.00952 0.00172 0.00484 0.00616 0.0379 0.0234 0.0167
2R:7171400-7171550:minus -30.9184 -0.66055 -2.23853 -0.853211 -27.0102 -19.0816 -14.0734 -2.04167
11.3853 0.0103 0.0129 0.0232 0.035 0.0237 0.017 0.00737 0.0301 0.0000815
2R:7171400-7171550:plus -29.102 -5.95413 5.95413 2.93578 -8.67347 -19.051 -20.1743 -2.69444 2.63303
0.00833 0.0565 0.00118 0.00927 0.00154 0.0165 0.0212 0.0358 0.00771
2R:7171840-7171990:minus -32.7041 2.87156 3.72477 6.51376 1.43878 -9.30612 -22.9266 5.22222 3.56881

0.0222 0.00365 0.003 0.00175 0.0000769 0.00257 0.0347 0.00238 0.00543
2R:7171840-7171990:plus -31.5102 0.440367 -2.77982 3.18349 -17.8878 -18.5714 -17.6697 6.84722 0.614679
0.013 0.00885 0.0271 0.00837 0.00704 0.0136 0.0142 0.00109 0.0158
2R:7176520-7176670:minus -22.7755 7.83486 -4.27523 4.33945 -18.1939 -8.72449 -11.1743 8.75 4.75229
0.00412 0.000417 0.0409 0.00509 0.0086 0.00169 0.00387 0.000374 0.00341
2R:7176520-7176670:plus -22.6327 -1.97248 4.3578 7.37615 -7.37755 0.826531 -16.0826 2.125 -0.651376
0.00373 0.0193 0.00232 0.000951 0.000398 0.000153 0.011 0.00824 0.0235
2R:7176920-7177070:minus -21.4388 -1.77982 -0.40367 2.23853 -27.6327 -17.3469 5.30275 0.138889 5.59633
0.002 0.0182 0.0132 0.0128 0.0369 0.0058 0.0000519 0.016 0.00234
2R:7176920-7177070:plus -23.0408 3.97959 -2.00917 2.88991 -8.93878 -8.5 -24.8073 0.180556 -6.9633
0.00512 0.000669 0.0217 0.00942 0.00216 0.00126 0.046 0.0158 0.112
2R:7180540-7180690:minus -31.898 7.56881 6.55046 3.04587 -27.5714 1.05102 -15.422 1.58333 2.41284
0.0164 0.000481 0.000896 0.00883 0.0357 0.000107 0.00975 0.00997 0.0082
2R:7180540-7180690:plus -22.6224 4.65138 6.36697 6.49541 -8.86735 -17.0102 -8.73394 -1.59722 4.14679
0.00371 0.00187 0.000979 0.00176 0.00173 0.00453 0.00243 0.0267 0.00406
2R:7183720-7183870:minus -23.8469 4.37615 5.10092 10.1468 -27.5306 -10.0918 -18.6789 0.625 7.68807
0.00685 0.00208 0.0017 0.000128 0.0351 0.00404 0.0166 0.0137 0.000841
2R:7183720-7183870:plus -14.5204 1.42202 -4.02752 3.31193 1.07143 -26.6735 -14.7798 5.375 9
0.00145 0.00621 0.0383 0.00786 0.000125 0.0256 0.00858 0.00222 0.000437
2R:7184940-7185090:minus -21.8469 10.0642 2.75229 2.80734 -18.6735 -8.38776 -18.4954 0.777778 0.669725
0.00247 0.00013 0.00436 0.00985 0.0123 0.00101 0.0161 0.0131 0.0155
2R:7184940-7185090:plus -23.602 8.22018 4.00917 4.81651 20.1735 -27.6327 -13.6789 0.0972222
2.33028 0.00657 0.000338 0.00267 0.00451 5.68e-07 0.043 0.00675 0.0162 0.00836
2R:7194760-7194910:minus -23 2.50459 -0.302752 6.12844 -18.1531 -8.16327 -14.4312 1.97222 7.18349
0.00498 0.00417 0.0128 0.00231 0.00829 0.000783 0.00797 0.0087 0.00121
2R:7194760-7194910:plus -14.1531 -0.412844 4.41284 6.29358 -18.3878 -9.5 -22.9908 -4.54167 4.88073
0.00121 0.0119 0.00227 0.00202 0.00931 0.00294 0.035 0.0557 0.00324
2R:7195080-7195330:minus -31.6735 0.110092 0.302752 9.51376 10.6939 -8.20408 -10.789 8.59722 2.06422
0.0141 0.00993 0.0105 0.000292 7.35e-06 0.000796 0.00358 0.000411 0.00912
2R:7195080-7195330:plus -31.6633 4.75229 7.09174 6.27523 20.4694 -9.57143 -14.5688 -1.97222 -0.633028
0.0141 0.0018 0.000696 0.00206 1.65e-07 0.00326 0.00821 0.0296 0.0232
2R:7246540-7246690:minus -24.3673 8.54128 5.0367 3.61468 -16.7143 -26.8265 -10.5321 2.06944 5.66055
0.00791 0.000285 0.00175 0.0068 0.00319 0.0285 0.0034 0.00841 0.00227
2R:7246540-7246690:plus -22.6633 7.27523 5.73394 -0.46789 -17.4184 -8.72449 -20.9725 8.79167 -0.908257
0.00384 0.000561 0.0013 0.0309 0.00457 0.00169 0.0245 0.000365 0.0255
2R:7247940-7248090:minus -11.0408 3.34862 6.47706 7.34862 -18.051 -9.64286 -12.1009 1.97222 1.16514
0.000241 0.00306 0.000928 0.000998 0.00746 0.00339 0.00472 0.0087 0.0135
2R:7247940-7248090:plus -24.7857 6.51376 12.5046 3.23853 -18.4898 -26.8265 -6.46789 8.04167 -3.40367
0.00829 0.000829 3e-05 0.00815 0.0116 0.0285 0.00148 0.00057 0.0486
2R:7253280-7253430:minus -41.102 -3.66972 -2.3211 -2.78899 -17.4898 -6.5 -25.945 0.291667 6.7156
0.0472 0.031 0.0237 0.0655 0.0053 0.000542 0.0531 0.0153 0.00144
2R:7253280-7253430:plus -12.449 1.94495 1.29358 7.52294 -17.3367 -27.051 -10.3303 -0.194444
-0.33945 0.000428 0.00512 0.00746 0.000819 0.00391 0.0315 0.00328 0.0178 0.0209
2R:7253940-7254090:minus -29.6531 -0.0733945 -5.99083 0.0183486 -7.63265 -45.7755 -11.3028 -2.95833
1.44037 0.00846 0.0106 0.0632 0.0268 0.000479 0.244 0.00397 0.0382 0.012
2R:7253940-7254090:plus -13 -1.61468 0.926606 5.49541 -7.33673 -18.7857 3.62385 -3.72222 1.46789
0.000563 0.0173 0.00848 0.00355 0.000388 0.0146 0.0000946 0.0461 0.0119
2R:7255620-7255770:minus -13.4796 2.65138 -0.165138 6.21101 -28.2347 0.122449 -16.8349 0.166667
-0.513761 0.000935 0.00395 0.0122 0.00213 0.051 0.000312 0.0125 0.0159 0.0221
2R:7255620-7255770:plus -32.2551 1.56881 -0.788991 2.61468 20.4694 -8.79592 -15.5872 0.138889
0.394495 0.0184 0.00588 0.0149 0.0109 1.65e-07 0.0019 0.0101 0.016 0.0166
2R:7258580-7258730:minus 6.32653 -2.6422 1.15596 1.52294 -26.6735 -25.602 -11.5872 4.88889 9.61468
3.97e-06 0.0233 0.00783 0.0175 0.0191 0.0207 0.00422 0.00276 0.00029
2R:7258580-7258730:plus -31.4796 1.93578 -1.44954 11.1927 20.2041 -27.9694 -9.0367 4.29167 0.311927

0.0128 0.00514 0.0183 0.0000254 3.64e-07 0.0547 0.00258 0.00356 0.0169
2R:7271640-7271790:minus -13.2551 5.87156 -0.211009 4.3211 -18.0816 -18.1224 -7.41284 -7.55556
1.72477 0.000751 0.00112 0.0124 0.00514 0.00759 0.00988 0.00186 0.103 0.0106
2R:7271640-7271790:plus -21.9184 3.33945 -1.46789 4.15596 -17.1531 -18.051 -20.9266 0.208333 5.22936
0.00261 0.00307 0.0184 0.00539 0.00364 0.00886 0.0243 0.0157 0.00288
2R:7276160-7276310:minus -31.6735 0.266055 10.156 7.55963 -17.9388 -8.72449 -27.4312 0.166667 -7.61468
0.0141 0.0094 0.00013 0.000807 0.00715 0.00169 0.0639 0.0159 0.126
2R:7276160-7276310:plus -24.5204 -0.880734 5.62385 6.69725 -7.89796 -19.051 -15.8165 1.25 -5.98165
0.00813 0.0138 0.00136 0.00156 0.000678 0.0165 0.0105 0.0112 0.0885
2R:7339720-7339870:minus -32.1735 -2.66055 0.706422 2.43119 -8.93878 -18.2755 -2.22936 2.83333 1.37615
0.018 0.0235 0.00914 0.0117 0.00216 0.0112 0.000469 0.00635 0.0123
2R:7339720-7339870:plus -23 -0.00917431 2.31193 7.27523 -17.0816 -17.0102 -14.5505 2.72222 1.55046
0.00498 0.0103 0.00515 0.00109 0.00334 0.00453 0.00818 0.00663 0.0115
2R:7340480-7340630:minus -31.1429 -3.36697 -0.853211 2.44037 -17.6735 -8.20408 -18.9908 0.305556
1.75229 0.0108 0.0285 0.0152 0.0116 0.00572 0.000796 0.0174 0.0152 0.0104
2R:7340480-7340630:plus -3.26531 -4.23853 -0.183486 3.95413 -27.3061 -18.2755 -20.2477 8.06944
2.04587 0.0000515 0.0361 0.0123 0.00593 0.0315 0.0112 0.0215 0.000561 0.00919
2R:7479920-7480070:minus -30.6633 3.46789 -1.66972 4.75229 -26.8571 -0.173469 -2.75229 2.875
11.3853 0.00976 0.00293 0.0196 0.00461 0.0198 0.000456 0.000533 0.00626 0.0000815
2R:7479920-7480070:plus -32.3571 -0.743119 0.348624 6.11009 -18.9388 -27.3367 -13.6697
-1.88889 5.22936 0.0187 0.0132 0.0103 0.00233 0.0151 0.0347 0.00674 0.0289 0.00288
2R:7480920-7481070:minus -32.551 -0.633028 1.16514 2.00917 -9.23469 -18.5 -14.6239 5.19444
5.70642 0.0207 0.0127 0.0078 0.0142 0.00277 0.0131 0.0083 0.00241 0.00222
2R:7480920-7481070:plus -40.3265 -2.08257 -1.68807 3.52294 -18.2653 -18.1224 -5.42202
4.625 6.11927 0.0394 0.0199 0.0197 0.00706 0.00882 0.00988 0.00112 0.00309 0.00178
2R:7481780-7481930:minus -32.4796 1.72477 7.37615 3.13761 -18.2245 -28.4082 -15.9908
3.38889 11.8532 0.02 0.00555 0.000603 0.00854 0.00877 0.0615 0.0108 0.00513 0.0000428
2R:7481780-7481930:plus -22.0816 0.963303 -1.46789 3.6055 -17.449 -9.45918 -17.6789 3.5
4.98165 0.003 0.00734 0.0184 0.00686 0.00484 0.00279 0.0142 0.00491 0.00313
2R:7482660-7482810:minus -32.1122 0.614679 -0.633028 2.66055 -18.449 -36.9592 -17.7339
0.25 3.56881 0.0178 0.00832 0.0142 0.0106 0.0107 0.124 0.0144 0.0155 0.00543
2R:7482660-7482810:plus -31.7755 -0.0825688 -0.0733945 2.23853 -27.2653 -8.38776 -20.6881
-0.486111 7.62385 0.0157 0.0106 0.0119 0.0128 0.0302 0.00101 0.0232 0.0194 0.000941
2R:7489040-7489190:minus -21.8163 1.40367 6.74312 6.29358 -26.9388 -7.5 -24.6972 -1.625
-5.11009 0.00244 0.00625 0.000818 0.00202 0.0215 0.000614 0.0453 0.0269 0.0744
2R:7489040-7489190:plus -24.3776 2.40367 2.82569 -0.33945 -17.2653 -26.3776 -8.79817
2.23611 9.55046 0.00793 0.00433 0.00424 0.0296 0.00387 0.0222 0.00246 0.00792 0.000321
2R:7489780-7489930:minus -14.5204 3.01835 3.86239 7.61468 -18.449 -8.38776 -24.3761 2.08333
0.0825688 0.00145 0.00346 0.00283 0.000779 0.0107 0.00101 0.0433 0.00837 0.0181
2R:7489780-7489930:plus -21.2959 2.22936 1.6055 5.34862 -27 -18.2755 2.31193 2.75 9.66055 0.00192
0.00461 0.00667 0.00387 0.0233 0.0112 0.000149 0.00656 0.000268
2R:7490300-7490450:minus -14.7449 -1.37615 9.37615 5.86239 -8.64286 -26.5612 -14.8349 -3.19444
5.38532 0.00157 0.0161 0.000204 0.00282 0.00139 0.0237 0.00868 0.0406 0.00267
2R:7490300-7490450:plus -22.0816 -2.16514 -0.211009 -0.357798 -17.3776 -17.2041 -24.4679 2.13889
-2.62385 0.003 0.0204 0.0124 0.0297 0.00419 0.00507 0.0439 0.0082 0.0402
2R:7490880-7491030:minus -23.0306 -5.26606 0.330275 6.25688 -26.6327 -8.16327 -7.92661 -1.38889
-0.0733945 0.00505 0.0473 0.0104 0.00208 0.0188 0.000783 0.00207 0.0252 0.0188
2R:7490880-7491030:plus -21.8878 -0.0275229 -3.68807 6.02752 -17.3367 -9.16327 -10.0734 -5.72222
-5.14679 0.00258 0.0104 0.0349 0.00246 0.00391 0.00223 0.00312 0.0719 0.0751
2R:7492560-7492710:minus -23.4592 1.99083 0.990826 7.77982 -8.33673 -8.5 -5.7156 -6.41667 3.66972
0.00631 0.00504 0.00829 0.000652 0.00101 0.00126 0.00121 0.0827 0.00514
2R:7492560-7492710:plus -31.5918 -2.30275 -3.18349 -1.56881 -17.8878 -19.1939 -14.1376 2.86111 11.8073
0.0136 0.0212 0.0304 0.0442 0.00704 0.0178 0.00748 0.00629 0.0000538
2R:7493540-7493690:minus -23 -1.86239 3.91743 6.00917 -7.64286 -18.051 -16.3486 -1.59722 -2.92661

0.00498 0.0187 0.00277 0.00255 0.000499 0.00886 0.0115 0.0267 0.0437
2R:7493540-7493690:plus -31.1837 1.58716 2.18349 4.38532 -18.4184 -16.051 -13.1101 2.45833 3.66972
0.0109 0.00584 0.0054 0.00501 0.0101 0.00408 0.00593 0.00731 0.00514
2R:7501660-7501810:minus -23.1122 -1.73394 5.56881 3.89908 -8.70408 -9.53061 -21.8165 -0.986111
4.82569 0.0053 0.018 0.0014 0.00604 0.00155 0.0031 0.0285 0.0225 0.00331
2R:7501660-7501810:plus -22.8061 -2.41284 -2.63303 3.13761 -28.3367 -10.0204 -7.70642 8.51389 6.95413
0.00414 0.0219 0.026 0.00854 0.0557 0.00399 0.00198 0.000433 0.00136
2R:7502640-7502790:minus -32.6633 -3.2844 -3.22936 -1.85321 0.836735 -8.72449 -14.5046 2.45833 3.52294
0.0216 0.0279 0.0307 0.0486 0.000215 0.00169 0.0081 0.00731 0.00558
2R:7502640-7502790:plus -22.8878 -2.05505 -0.87156 4.79817 -27.3061 -17.1224 -4.9633 9.33333 9.04587
0.00441 0.0197 0.0153 0.00453 0.0315 0.00499 0.000983 0.000258 0.000423
2R:7503600-7503750:minus 5.36735 5.44954 1.47706 6.09174 -8.60204 -16.7143 3.76147 5.33333 10.3486
0.0000111 0.00135 0.00698 0.00235 0.00125 0.00419 9e-05 0.00226 0.0000988
2R:7503600-7503750:plus -24.2653 3.97248 -2.48624 4.79817 -18.1837 -18.051 -12.6055 0.75 -4.49541
0.00773 0.00243 0.0249 0.00453 0.00835 0.00886 0.00528 0.0132 0.0638
2R:7506500-7506650:minus -14.2143 14.3394 8.2844 4.17431 1.94898 -8.86735 -2.53211 0.319444 -2.47706
0.00124 5.96e-06 0.000376 0.00537 0.0000358 0.00198 0.000505 0.0151 0.0385
2R:7506500-7506650:plus -39.9898 4.19266 13.4128 7.45872 -27.2653 -8.23469 -13.5229 7.25 0.651376
0.0351 0.00224 0.0000141 0.000894 0.0302 0.000875 0.00652 0.000882 0.0156
2R:7520060-7520210:minus -2.40816 0.146789 13.5872 7.45872 -26.9694 -18.0102 -0.394495 4.73611
1.63303 0.0000315 0.0098 0.0000122 0.000894 0.023 0.00812 0.00031 0.00295 0.0112
2R:7520060-7520210:plus -39.9184 0.93578 3.92661 4.74312 -17.8265 1.27551 -27.844 2.875 -1.14679
0.0344 0.00741 0.00276 0.00462 0.00691 0.0000717 0.0674 0.00626 0.0272
2R:7520900-7521050:minus -22.9184 3.24771 0.0917431 -1.15596 -27.3061 -17.898 -11.6055 10.8194
1.37615 0.00447 0.00317 0.0112 0.0386 0.0315 0.00726 0.00423 0.0000883 0.0123
2R:7520900-7521050:plus -22.9592 -3.86239 1.58716 1.77064 -18.4796 0.204082 -6.13761 10.1389 5.22936
0.00475 0.0326 0.00672 0.0159 0.011 0.000296 0.00136 0.000148 0.00288
2R:7524960-7525110:minus -13.2653 -1.92661 2.99083 7.99083 -18.449 -8.38776 -15.8349 -2.69444 11.8532
0.000759 0.019 0.00399 0.00059 0.0107 0.00101 0.0105 0.0358 0.0000428
2R:7524960-7525110:plus -31.3673 3.01835 -0.623853 -0.880734 -27 -27.5612 -7.17431 0.902778
5.59633 0.0116 0.00346 0.0142 0.0353 0.0233 0.0393 0.00176 0.0125 0.00234
2R:7525740-7525890:minus -33.0408 -3.82569 1.94495 3.95413 -18.4898 -17.0102 -22.9725 9.36111 -1.51376
0.0264 0.0323 0.0059 0.00593 0.0116 0.00453 0.0349 0.000254 0.0294
2R:7525740-7525890:plus -23.9592 0.174312 2.65138 5.63303 -7.86735 -8.72449 -14.8899 0.305556 5.17431
0.00708 0.00971 0.00453 0.00326 0.000617 0.00169 0.00877 0.0152 0.00296
2R:7527080-7527230:minus -22.2245 1.21101 2.7156 2.3578 -18.0102 -8.38776 -2.61468 5.61111 1.00917
0.00325 0.00671 0.00442 0.0121 0.00732 0.00101 0.000515 0.00199 0.0143
2R:7527080-7527230:plus -41.4082 -1.61468 3.20183 4.79817 -27.2653 -26.7857 -18.6147 0.166667 4.18349
0.0557 0.0173 0.00367 0.00453 0.0302 0.0266 0.0164 0.0159 0.00404
2R:7527280-7527430:minus -33.6633 1.44954 1.77982 -1.01835 -18.6837 0.755102 -32.7431 0.847222 -8.20183
0.0291 0.00615 0.00627 0.0369 0.0126 0.000175 0.128 0.0128 0.141
2R:7527280-7527430:plus -32.7347 1.24771 0.834862 1.83486 -8.27551 -9.57143 -25.7339 1.09722 -0.651376
0.0225 0.00661 0.00875 0.0155 0.000951 0.00326 0.0518 0.0118 0.0235
2R:7527600-7527750:minus -23.1429 -2.46789 -0.981651 2.69725 -18.4898 -8.93878 -25.5505 -2.91667
5.18349 0.00533 0.0222 0.0159 0.0104 0.0116 0.00201 0.0506 0.0378 0.00295
2R:7527600-7527750:plus -31.4388 -2.91743 11.7798 7.45872 -7.63265 -17.4286 -19.2569 -3.15278 -4.18349
0.0122 0.0252 0.0000501 0.000894 0.000479 0.00586 0.0182 0.0402 0.0581
2R:7528400-7528550:minus -32.1735 -1.12844 12.367 2.83486 1.17347 -17.9796 -18.7431 -1.33333 -2.51376
0.018 0.0149 0.0000334 0.0097 0.000101 0.00764 0.0168 0.0248 0.039
2R:7528400-7528550:plus -42.102 -1.42202 -1.65138 0.201835 -27.5612 -9.09184 -14.3486 2.75 3.88991
0.0696 0.0164 0.0195 0.0255 0.0354 0.00219 0.00783 0.00656 0.00467
2R:7533600-7533750:minus -21.7041 -5.20183 1.3578 2.27523 -18.1531 -36.6633 -14.6972 -1.30556 1.73394
0.00231 0.0465 0.00728 0.0125 0.00829 0.115 0.00843 0.0246 0.0106
2R:7533600-7533750:plus -29.1429 3.38532 1.37615 4.14679 -19.051 -18.3061 -14.7156 0.25 1.23853

0.00834 0.00302 0.00724 0.00542 0.0162 0.0114 0.00847 0.0155 0.0132
2R:7538500-7538650:minus -32.3673 0.715596 -0.275229 2.31193 -17.4898 -17.0816 -2.20183 6.25 5.74312
0.0189 0.00802 0.0127 0.0123 0.0053 0.00487 0.000466 0.00147 0.00215
2R:7538500-7538650:plus -32.9694 -4.34862 4.34862 10.0459 -18.3061 -7.42857 -19.4587 -7.22222 -12.6789
0.0254 0.0371 0.00233 0.000139 0.00884 0.000567 0.0188 0.0965 0.297
2R:7539100-7539250:minus -39.0306 -0.0458716 3.55963 5.36697 2.14286 0.897959 -5.25688 0.944444
-4.68807 0.0326 0.0105 0.0032 0.00386 0.0000316 0.000144 0.00107 0.0124 0.0672
2R:7539100-7539250:plus -22.9184 -1.45872 14.8257 7.29358 -9.23469 0.897959 -13.945 0.805556 -6.89908
0.00447 0.0165 2.55e-06 0.00104 0.00277 0.000144 0.00717 0.013 0.111
2R:7549560-7549710:minus -24.7449 1.59633 4.05505 4.59633 -18.7143 -18.2041 -4.02752 11.1667 -2.26606
0.00827 0.00582 0.00262 0.00478 0.0136 0.0103 0.000752 0.0000662 0.0359
2R:7549560-7549710:plus -22.9592 5.37615 -0.495413 4.22018 -26.898 -17.2755 -15.3945 -4.29167
1.48624 0.00475 0.00139 0.0136 0.00531 0.0203 0.00544 0.0097 0.0527 0.0118
2R:7551160-7551310:minus -33.551 0.59633 3.09174 -1.22936 -27 -27.6735 -14.0826 1.16667 7.68807
0.0288 0.00837 0.00383 0.0395 0.0233 0.0443 0.00739 0.0115 0.000841
2R:7551160-7551310:plus -14.2143 2.45872 3.17431 2.05505 -18.1224 -18.5612 14.9266 -2.5 3.92661
0.00124 0.00424 0.00371 0.0139 0.00782 0.0132 1.43e-06 0.034 0.00461
2R:7553000-7553150:minus -21.4796 -0.651376 3.6789 6.72477 -17.1224 -8.72449 -11.8899 -6.08333
10.3394 0.00202 0.0128 0.00305 0.00154 0.00343 0.00169 0.0045 0.0774 0.000112
2R:7553000-7553150:plus -24.1837 -0.541284 -0.788991 3.78899 -17.7857 -0.244898 -15.2936
11.2917 -3.81651 0.00751 0.0124 0.0149 0.00632 0.00676 0.000496 0.00951 0.0000594 0.0529
2R:75620-75770:minus -32.0408 -3.48624 2.80734 1.56881 0.540816 -18.3469 -14.8991 -0.930556 -6.22018
0.0174 0.0294 0.00427 0.0172 0.000321 0.0124 0.00879 0.0221 0.0938
2R:75620-75770:plus -31.5204 6.61468 -0.623853 2 0.316327 -27.0408 -17.6697 5.625 4.88073
0.0131 0.000789 0.0142 0.0143 0.000341 0.0307 0.0142 0.00198 0.00324
2R:7566780-7566930:minus -24.7143 2.11009 -1.77064 5.49541 10.6531 -8.72449 -32.055 -4.33333 -2.18349
0.00826 0.00482 0.0202 0.00355 0.0000102 0.00169 0.119 0.0532 0.0351
2R:7566780-7566930:plus -3.84694 2.9633 -0.59633 6.72477 -27.2347 -8.72449 -3.94495 -4.93056 2.11009
0.000107 0.00353 0.0141 0.00154 0.0283 0.00169 0.000734 0.0608 0.00898
2R:7577920-7578070:minus -13.5204 1.61468 -0.110092 1.68807 1.21429 -28.4082 -10.9908 4.63889
1.56881 0.00097 0.00578 0.012 0.0164 0.0000864 0.0615 0.00373 0.00307 0.0114
2R:7577920-7578070:plus -21 0.798165 1.23853 6.11009 -18.4184 -26.898 -12.422 -6.09722 3.16514
0.00186 0.00779 0.0076 0.00233 0.0101 0.0302 0.00507 0.0776 0.00656
2R:7578620-7578770:minus -13.7449 -0.284404 15.0092 5.57798 -17.4898 -18.2041 -12.8716 0.652778
-4.52294 0.00107 0.0114 1.55e-06 0.0034 0.0053 0.0103 0.00561 0.0136 0.0642
2R:7578620-7578770:plus -33 -2.47706 3.89908 1.72477 0.836735 -9.53061 -22.0183 -0.319444 -2.92661
0.0258 0.0223 0.00279 0.0161 0.000215 0.0031 0.0296 0.0184 0.0437
2R:7599620-7599770:minus -33.9286 2.08257 1.49541 1.86239 -18.1939 -9.30612 -16.8991 3.90278 3.51376
0.0303 0.00487 0.00694 0.0153 0.0086 0.00257 0.0126 0.00418 0.00566
2R:7599620-7599770:plus -21.2143 0.798165 0.33945 9.59633 -17.7143 1.09184 -3.40367 -3.13889 0.559633
0.00189 0.00779 0.0104 0.000275 0.00627 0.000091 0.000632 0.04 0.016
2R:7604800-7604950:minus -24.602 0.229358 4.6789 6.57798 -8.70408 -17.8265 -20.2202 1.61111 1.43119
0.00822 0.00953 0.00203 0.00167 0.00155 0.00699 0.0214 0.00988 0.0121
2R:7604800-7604950:plus -32.1122 -5.44954 -2.10092 -1.63303 -18.4184 -17.9796 -10.7064 1.84722 8.05505
0.0178 0.0496 0.0223 0.0452 0.0101 0.00764 0.00352 0.0091 0.00064
2R:7667640-7667790:minus -33.2245 0.706422 -0.926606 2.6789 -19.2755 -9.79592 -19.3119 5 1.36697
0.0276 0.00805 0.0156 0.0105 0.0164 0.00377 0.0183 0.00263 0.0124
2R:7667640-7667790:plus -33.2653 -0.165138 0.495413 3.41284 -17.4898 -8.79592 -10.6055 5.86111
7.42202 0.0277 0.0109 0.00983 0.00756 0.0053 0.0019 0.00345 0.00177 0.00106
2R:7712740-7712890:minus -32.7041 0.311927 1.01835 5.22936 -18.7143 -18.5714 -19.367 6.38889 1.70642
0.0222 0.00925 0.00821 0.0041 0.0136 0.0136 0.0185 0.00137 0.0108
2R:7712740-7712890:plus -32.7347 13.3878 2.55963 0.486239 10.6224 -0.0204082 -6.82569 3.48611
-0.59633 0.0225 0.0000869 0.0047 0.0237 0.0000113 0.000411 0.00162 0.00494 0.0229
2R:7727080-7727230:minus -13.2551 -0.174312 5.84404 3.00917 -28.1939 -27.7449 -11.633 0.958333

3.15596 0.000751 0.0109 0.00124 0.00894 0.0482 0.0448 0.00426 0.0123 0.00659
2R:7727080-7727230:plus -22.8163 3.43119 10.5596 7.72477 -26.6633 -18.0102 -12.789 -3.94444 -2.76147
0.00418 0.00297 0.000102 0.000681 0.0189 0.00812 0.00551 0.0486 0.0418
2R:7729060-7729210:minus -33.2653 3.21101 -1.04587 5.49541 -18.6837 -7.79592 -28.0642 1.36111 -4.84404
0.0277 0.00322 0.0162 0.00355 0.0126 0.000723 0.0694 0.0108 0.0701
2R:7729060-7729210:plus -24.5204 0.568807 14.9633 7.29358 -17.0816 -0.244898 -34.9174 1.27778
-8.81651 0.00813 0.00845 1.77e-06 0.00104 0.00334 0.000496 0.159 0.0111 0.16
2R:7731680-7731830:minus -23.1531 13.4592 0.926606 4.3211 -18.7857 0.0510204 -19.1651 3.25 2.88073
0.00542 0.000064 0.00848 0.00514 0.0148 0.000349 0.0179 0.00542 0.00727
2R:7731680-7731830:plus -4.85714 13.4592 1.44037 4.22018 -18.4184 -18.1224 -9.9633 2.36111 4.30275
0.000202 0.000064 0.00707 0.00531 0.0101 0.00988 0.00306 0.00757 0.00388
2R:7740040-7740190:minus -32 2.77064 -0.348624 5.86239 -27.1939 -18.2347 1.27523 8.75 5.01835
0.0171 0.00378 0.013 0.00282 0.0267 0.0104 0.000204 0.000374 0.00309
2R:7740040-7740190:plus -33.1837 14.4954 -2.63303 5.57798 -17.7143 -8.45918 -20.055 -1.34722 3.25688
0.0274 5.09e-06 0.026 0.0034 0.00627 0.00111 0.0208 0.0249 0.00632
2R:7773060-7773210:minus -21.8571 -1.66972 4.30275 6.12844 0.908163 -17.9796 -5.98165 5.83333 4.19266
0.00249 0.0176 0.00237 0.00231 0.000153 0.00764 0.0013 0.00179 0.00401
2R:7773060-7773210:plus -29.4388 0.201835 -1.34862 -1.78899 -16.8571 -8.42857 -3.61468 -5.83333 1.02752
0.00842 0.00962 0.0178 0.0476 0.00324 0.00104 0.00067 0.0736 0.0141
2R:7777900-7778050:minus -21.7755 -3.82569 -1.93578 3.47706 -18.4898 0.204082 -17.5505 2.76389 8.88073
0.00241 0.0323 0.0212 0.00724 0.0116 0.000296 0.014 0.00652 0.000468
2R:7777900-7778050:plus -21.7347 4.11927 0.183486 7.78899 -18.2245 -28.1122 -9.88073 0.736111 3.29358
0.00235 0.0023 0.0109 0.000642 0.00877 0.0565 0.00301 0.0132 0.00623
2R:7778440-7778590:minus -21.9592 4.08257 3.37615 9.49541 -27.7041 -8.45918 -7.40367 5.5 6.11927
0.00279 0.00233 0.00343 0.000308 0.038 0.00111 0.00185 0.00209 0.00178
2R:7778440-7778590:plus -32.1122 0.669725 2.82569 5.65138 1.14286 -8.79592 -15.0826 5.13889 -2.06422
0.0178 0.00815 0.00424 0.00322 0.000113 0.0019 0.00912 0.00247 0.0339
2R:7779100-7779250:minus -40.7755 1.53211 -2.29358 2.09174 -18.7143 -8.23469 -13.2936 6.02778 -0.229358
0.0433 0.00596 0.0236 0.0137 0.0136 0.000875 0.00618 0.00164 0.0202
2R:7779100-7779250:plus -23.7857 5.06422 1.97248 3.88991 -27.5306 -8.79592 -27.8349 4.09722 0.275229
0.00679 0.00159 0.00584 0.00608 0.0351 0.0019 0.0673 0.00386 0.0171
2R:7779580-7779730:minus -24.2551 7.16514 0.541284 5.57798 -9.16327 -9.7551 -0.66055 0.333333 -0.981651
0.00768 0.000596 0.00968 0.0034 0.00249 0.00358 0.000329 0.0151 0.026
2R:7779580-7779730:plus -21.6224 -4.13761 -0.513761 5.3945 -8.60204 -9.45918 -10.7339 -2.72222
1.68807 0.00213 0.0351 0.0137 0.00379 0.00125 0.00279 0.00354 0.036 0.0109
2R:7780400-7780550:minus -22.9694 2.82569 4.38532 1.47706 -8.67347 -18.898 -7.63303 8 11.8532
0.0048 0.00371 0.0023 0.0178 0.00154 0.0152 0.00195 0.000583 0.0000428
2R:7780400-7780550:plus -41.7041 5.98165 8.3578 9.7156 -27.5306 -9.27551 -22.211 0.458333 2.11927
0.0641 0.00107 0.000361 0.000242 0.0351 0.00244 0.0306 0.0145 0.00889
2R:7786940-7787090:minus -22.7755 1.25688 1.62385 0.779817 0.908163 -17.7551 -14.578 9.09722 3.87156
0.00412 0.00659 0.00663 0.0219 0.000153 0.00643 0.00822 0.000301 0.00477
2R:7786940-7787090:plus -12.9898 -1.29358 4.0367 9.41284 -26.5918 -18.2041 -23.1193 9.18056 1.0367
0.000553 0.0157 0.00264 0.000354 0.0186 0.0103 0.0358 0.000285 0.0141
2R:7791380-7791530:minus -24.3776 -0.458716 -3.18349 2.31193 20.1735 -9.42857 -26.2569 -1.38889
-2.89908 0.00793 0.012 0.0304 0.0123 5.68e-07 0.0027 0.0552 0.0252 0.0434
2R:7791380-7791530:plus -21.1531 13.4592 0.366972 5.57798 -8.33673 -0.316327 -16.1101 4.125
-2.6055 0.00188 0.000064 0.0103 0.0034 0.00101 0.000524 0.0111 0.00382 0.04
2R:7856320-7856470:minus -32 13.5306 -1.58716 4.78899 -18.7143 -17.2755 -19.9817 1.27778 -2.92661
0.0171 0.0000206 0.0191 0.00456 0.0136 0.00544 0.0205 0.0111 0.0437
2R:7856320-7856470:plus -14.1122 14.9908 10.6789 5.57798 -8.97959 -9.20408 -20.1743 -1.25 3.10092
0.00119 2.1e-06 0.0000957 0.0034 0.0023 0.00225 0.0212 0.0242 0.00674
2R:7857640-7857790:minus -42.551 13.3878 -0.614679 3.56881 -27.3061 -8.53061 -26.578 5.73611
2.42202 0.0803 0.0000869 0.0141 0.00699 0.0315 0.00136 0.0574 0.00188 0.00816
2R:7857640-7857790:plus -23.5204 13.5306 2.82569 5.58716 -7.33673 -7.45918 -19.6606 4.13889 5.24771

0.00645 0.0000206 0.00424 0.00334 0.000388 0.000582 0.0194 0.0038 0.00282
2R:7858080-7858230:minus -23.9592 0.00917431 -2.88073 2.74312 -18.449 -8.61224 -16.7248 7.69444
3.46789 0.00708 0.0103 0.0279 0.0101 0.0107 0.00152 0.0123 0.000693 0.00582
2R:7858080-7858230:plus -32.4082 0.449541 10.3578 7.55963 -17.449 -9.30612 -14.4587 -2.43056 3.33028
0.0191 0.00882 0.000115 0.000807 0.00484 0.00257 0.00802 0.0334 0.00612
2R:7872100-7872250:minus -22.8163 2.38532 -1.12844 2.33945 -17.449 -18.5 -12.945 2.34722 -1.44954
0.00418 0.00436 0.0166 0.0122 0.00484 0.0131 0.00571 0.00761 0.0291
2R:7872100-7872250:plus -21.7755 -3.36697 7.17431 2.55963 -18.6837 -0.244898 -21.3578 3.30556
0.12844 0.00241 0.0285 0.000668 0.0111 0.0126 0.000496 0.0262 0.0053 0.0179
2R:7872740-7872890:minus -13.1429 2.84404 1.84404 2.66972 -18.6429 -17.2755 -2.9633 1.61111 1.25688
0.00065 0.00368 0.00612 0.0106 0.0121 0.00544 0.000563 0.00988 0.0131
2R:7872740-7872890:plus -31.0408 3.64286 -0.348624 1.66972 -18.0204 -9.57143 -24.8807 3.44444
2.22018 0.0106 0.00159 0.013 0.0165 0.00733 0.00326 0.0464 0.00502 0.00859
2R:7873260-7873410:minus -23.7755 -2.05505 14.9633 7.29358 -18.6735 -18.0102 -17.0183 1.30556 5.79817
0.00678 0.0197 1.77e-06 0.00104 0.0123 0.00812 0.0129 0.011 0.00208
2R:7873260-7873410:plus -22 13.8532 13.5046 2.53211 -27.7959 0.0510204 -19.4771 2.56944 -4.42202
0.00291 9.88e-06 0.000013 0.0113 0.0386 0.000349 0.0189 0.00701 0.0626
2R:7876460-7876610:minus -41.1735 -4.55963 3.80734 1.91743 1.87755 -8.5 -16.6239 4.40278 -2.83486
0.0479 0.0393 0.0029 0.015 0.0000492 0.00126 0.0121 0.0034 0.0427
2R:7876460-7876610:plus -24.1531 13.3878 3.7156 2.50459 -28.2653 -25.8265 -25.1284 0.972222 5.23853
0.00744 0.0000869 0.00301 0.0114 0.0537 0.0212 0.048 0.0123 0.00285
2R:7915340-7915490:minus -22.398 0.146789 11.1284 -0.174312 -18.4898 -17.2041 -16 3.70833 -0.119266
0.00344 0.0098 0.0000734 0.0283 0.0116 0.00507 0.0108 0.00452 0.0192
2R:7915340-7915490:plus -22.8878 -4.26606 0.0917431 -2 1.87755 -26.602 -16.0275 6.43056 -0.697248
0.00441 0.0363 0.0112 0.0513 0.0000492 0.0248 0.0109 0.00134 0.0239
2R:7915720-7915870:minus -11.8469 8.55963 0.422018 2.62385 -18.4184 -8.72449 -14.5321 -5.11111 1.10092
0.000279 0.000283 0.0101 0.0108 0.0101 0.00169 0.00814 0.0632 0.0138
2R:7915720-7915870:plus -22.8163 0.0275229 -0.238532 3.78899 -8.33673 -9.38776 -7.88991 0.166667
-2.19266 0.00418 0.0102 0.0125 0.00632 0.00101 0.00266 0.00206 0.0159 0.0352
2R:7921360-7921510:minus -33.5612 8.83486 -0.0550459 2.84404 -27.4898 -16.2755 -25.7248 6.26389
-0.651376 0.0288 0.000244 0.0118 0.00961 0.0336 0.00413 0.0517 0.00146 0.0235
2R:7921360-7921510:plus -31.7755 1.13761 1.79817 3.41284 -17.4184 0.122449 -14.0183 -0.930556
-3.11009 0.0157 0.00689 0.00623 0.00756 0.00457 0.000312 0.00728 0.0221 0.0459
2R:7924700-7924850:minus -32.7041 0.550459 -1.29358 1.90826 0.908163 -17.1224 -20.2385 2.13889 -4.11009
0.0222 0.00851 0.0175 0.015 0.000153 0.00499 0.0215 0.0082 0.0571
2R:7924700-7924850:plus -12.1837 13.5306 5.50459 3.77064 -8.96939 0.530612 -6.29358 1.65278 2.55963
0.000365 0.0000206 0.00144 0.00637 0.00226 0.000203 0.00142 0.00974 0.00784
2R:7926460-7926610:minus -32.3571 5.0367 -2.13761 2.33945 -17.9388 -8.94898 -23.3028 5.81944 0.59633
0.0187 0.0016 0.0225 0.0122 0.00715 0.00203 0.0369 0.00181 0.016
2R:7926460-7926610:plus -12.4082 13.4592 1.42202 2.58716 -17.6429 -18.2755 -10.1651 1.94444 -1.06422
0.000414 0.000064 0.00712 0.011 0.00562 0.0112 0.00317 0.00879 0.0266
2R:7932100-7932250:minus -23.4592 0.100917 1.41284 -2.46789 -26.4898 0.602041 -11.0183 3.90278 -9.47706
0.00631 0.00996 0.00714 0.0596 0.0183 0.000188 0.00375 0.00418 0.179
2R:7932100-7932250:plus -13.3673 -0.376147 2.01835 5.59633 -26.9286 -9.23469 -6.12844 14.25
2.73394 0.000813 0.0117 0.00574 0.00331 0.0214 0.00238 0.00136 1.8e-06 0.00756
2R:8026180-8026330:minus -31.1837 0.733945 2.10092 2.83486 -18.7857 -8.30612 -14.4862 12.4861 2.44037
0.0109 0.00797 0.00556 0.0097 0.0148 0.000973 0.00807 0.0000188 0.00809
2R:8026180-8026330:plus -33.0714 -3.70642 13.844 2.88991 20.1735 -18.1224 -19.2294 -0.888889
-0.807339 0.0267 0.0313 8.84e-06 0.00942 5.68e-07 0.00988 0.0181 0.0218 0.0247
2R:8027940-8028090:minus -29.3673 -3.43119 1.10092 1.99083 0.877551 -18.0102 -16.7339 5.65278 -6.62385
0.00838 0.029 0.00798 0.0144 0.00019 0.00812 0.0123 0.00195 0.104
2R:8027940-8028090:plus -31.6224 0.0458716 0.0917431 1.91743 -17.449 9.23469 -4.19266 8.18056
-0.678899 0.0137 0.0101 0.0112 0.015 0.00484 0.0000427 0.000788 0.000526 0.0237
2R:8033140-8033290:minus -23.4796 10.2202 0.211009 2.26606 -19.2755 0.0918367 -20.055 5.09722

-4.24771 0.00634 0.00012 0.0108 0.0126 0.0164 0.000323 0.0208 0.00251 0.0591
2R:8033140-8033290:plus -39.9898 4.51376 -0.954128 1.74312 10.4286 -17.3469 -6.47706 3.48611
-2.05505 0.0351 0.00198 0.0157 0.016 0.0000139 0.0058 0.00148 0.00494 0.0338
2R:8038280-8038430:minus -30.5102 -2.85321 -0.504587 0.853211 -17.8265 -26.6327 -13.4495 3.95833
8.11009 0.00947 0.0248 0.0137 0.0214 0.00691 0.0251 0.00641 0.00409 0.000597
2R:8038280-8038430:plus -23.0408 1.29358 5.33028 2.44037 -27.5306 -8.23469 -31.1284 1.79167 9
0.00512 0.00651 0.00155 0.0116 0.0351 0.000875 0.106 0.00928 0.000437
2R:8050420-8050570:minus -32.2959 2.44037 -0.0366972 3.77064 -18.5306 -19.5714 -19.7339 13.1944
-0.550459 0.0186 0.00427 0.0117 0.00637 0.0118 0.0202 0.0197 8.22e-06 0.0225
2R:8050420-8050570:plus -4.04082 13.3878 0.880734 7.6055 0.806122 -0.204082 -6.33028 3.40278
3.20183 0.000123 0.0000869 0.00861 0.000794 0.000229 0.000463 0.00143 0.00511 0.0065
2R:8053260-8053410:minus -22 7.38532 0.623853 3.10092 -17.7857 -27.2653 -5.55046 10.5833 3.26606
0.00291 0.000529 0.00941 0.00865 0.00676 0.0333 0.00116 0.000106 0.00627
2R:8053260-8053410:plus -31.5918 2.86239 15.0459 5.06422 -8.71429 1.31633 -19.4312 3.02778 2.27523
0.0136 0.00366 1.26e-06 0.00433 0.00159 0.000066 0.0187 0.0059 0.00846
2R:8056220-8056370:minus -21.551 6.70642 4.72477 6.09174 -27.602 -25.602 -13.6972 6.52778 5.18349
0.00205 0.000754 0.00199 0.00235 0.0364 0.0207 0.00678 0.00128 0.00295
2R:8056220-8056370:plus -22.8469 -2.38532 -4.38532 0.972477 -17.449 -27.4082 -12.2018 1.75 9.77064
0.00424 0.0217 0.0421 0.0207 0.00484 0.0356 0.00482 0.00941 0.000228
2R:8058040-8058190:minus -14.5918 3.90816 7.27523 3.20183 -27.2653 -27.6327 -7.90826 7.25 9.92661
0.00148 0.00102 0.000635 0.00824 0.0302 0.043 0.00206 0.000882 0.000193
2R:8058040-8058190:plus -13.1531 -0.733945 7.7156 3.04587 -8.97959 -27.8265 -9.92661 0.555556
5.38532 0.00067 0.0132 0.000509 0.00883 0.0023 0.0498 0.00304 0.014 0.00267
2R:8058720-8058870:minus -21.6633 1.77982 1.19266 11.0183 -18.7857 -26.602 -6.98165 5.36111 1.73394
0.00223 0.00544 0.00773 0.000052 0.0148 0.0248 0.00168 0.00223 0.0106
2R:8058720-8058870:plus -11.8469 -2.94495 0.651376 6 -17.4898 -18.8571 -4.51376 -0.361111 9.56881
0.000279 0.0254 0.00932 0.00256 0.0053 0.0151 0.000864 0.0187 0.000303
2R:8060420-8060570:minus -32.6633 3.56881 5.23853 2.6422 -26.898 -8.23469 -26.4771 3.80556 9.88073
0.0216 0.00282 0.00161 0.0107 0.0203 0.000875 0.0567 0.00435 0.000206
2R:8060420-8060570:plus -33.8878 5.40367 0.752294 -0.917431 -26.9694 -18.7857 1.87156 8.47222
2.54128 0.0302 0.00138 0.009 0.0358 0.023 0.0146 0.000172 0.000443 0.00787
2R:8060760-8060910:minus -31.9694 0.605505 9.41284 3.95413 -18.7245 9.60204 -13.578 -0.347222
-0.633028 0.0168 0.00834 0.000199 0.00593 0.0136 0.0000248 0.0066 0.0186 0.0232
2R:8060760-8060910:plus -31.2857 -2.44037 -0.376147 0.0642202 -8.64286 -27.6735 -15.0917 4.63889
1.16514 0.0113 0.0221 0.0131 0.0264 0.00139 0.0443 0.00914 0.00307 0.0135
2R:8061180-8061330:minus -32.1735 0.926606 -0.366972 0.550459 -18.7857 -0.244898 -24.0734 3.375
-0.275229 0.018 0.00743 0.0131 0.0233 0.0148 0.000496 0.0415 0.00516 0.0206
2R:8061180-8061330:plus -32 -2.02752 -1.29358 2.3578 -6.93878 -18.1224 -16.3028 -3.15278 0.238532
0.0171 0.0196 0.0175 0.0121 0.000373 0.00988 0.0114 0.0402 0.0173
2R:8061680-8061830:minus -23.9592 -1.29358 3.27523 9.59633 -26.2653 9.82653 -16.0092 12.5139 2.25688
0.00708 0.0157 0.00357 0.000275 0.018 0.0000148 0.0109 0.0000182 0.00853
2R:8061680-8061830:plus -22.8163 -1.75229 5.97248 1.14679 19.9082 -18.051 -19.6606 -2.02778 -3.43119
0.00418 0.0181 0.00117 0.0198 8.13e-07 0.00886 0.0194 0.03 0.0489
2R:8062180-8062330:minus -33.2245 14.0275 0.568807 2.51376 -28.4592 -0.469388 -33.367 1.79167
-6.33945 0.0276 8.23e-06 0.00958 0.0113 0.0568 0.000533 0.137 0.00928 0.0966
2R:8062180-8062330:plus -3.63265 14.5688 3.86239 11.0275 -8.20408 -9.5 -18.4404 -0.888889 -1.23853
0.0000917 4.39e-06 0.00283 0.0000401 0.000894 0.00294 0.016 0.0218 0.0279
2R:8066000-8066150:minus -14.0714 0.0917431 1.26606 4.40367 19.9082 0.122449 -23.3394 0.0694444
1.54128 0.00117 0.00999 0.00753 0.00497 8.13e-07 0.000312 0.0371 0.0164 0.0115
2R:8066000-8066150:plus -22.7041 6.77064 3.05505 2 -17.6837 -18.3061 -12.211 5.30556 1.86239
0.00395 0.000731 0.00389 0.0143 0.0058 0.0114 0.00483 0.00229 0.00994
2R:8066380-8066530:minus -29.7449 7.54128 2.3578 5.57798 11.4286 -18.2347 -17.5046 6.61111 0.642202
0.00849 0.000487 0.00506 0.0034 1.97e-06 0.0104 0.0139 0.00123 0.0157
2R:8066380-8066530:plus -24.5918 3.3578 4.45872 3.62385 -27.2653 1.05102 -16.2202 4.44444 3

0.00821 0.00305 0.00223 0.00673 0.0302 0.000107 0.0113 0.00334 0.00702
2R:8066960-8067110:minus -24.7143 2 -3.33028 2.19266 -18.7449 -9.02041 -15.1376 7.05556 -0.12844
0.00826 0.00502 0.0316 0.0131 0.0138 0.00213 0.00922 0.000977 0.0193
2R:8066960-8067110:plus -21.5102 -1.11009 4.02752 6.02752 -17.449 -18.9796 -15.1835 -0.777778
-0.449541 0.00202 0.0148 0.00265 0.00246 0.00484 0.0155 0.00931 0.0211 0.0216
2R:8067300-8067450:minus -32.2653 4.9633 1.63303 9.7156 10.3878 10.3061 -8.14679 3.15278 3.6789
0.0184 0.00165 0.00661 0.000242 0.0000174 7.25e-06 0.00217 0.00563 0.00506
2R:8067300-8067450:plus -31.9184 5.08257 7.45872 4.79817 10.3571 -17.7857 -17.2018 0.75 0.229358
0.0164 0.00158 0.000579 0.00453 0.0000208 0.0068 0.0132 0.0132 0.0173
2R:8068020-8068170:minus -32 3.64286 5.45872 5.38532 -18.051 -18.5714 -5.56881 2.65278 -0.761468
0.0171 0.00159 0.00147 0.00382 0.00746 0.0136 0.00117 0.0068 0.0244
2R:8068020-8068170:plus -41.2551 -1.88073 5.65138 5.31193 19.9082 -18.1531 -18.211 2.11111 1.86239
0.0513 0.0188 0.00135 0.00393 8.13e-07 0.00997 0.0154 0.00828 0.00994
2R:8069980-8070130:minus -31.4082 0.0550459 6.93578 3.97248 -9.0102 -9.5 -26.3486 -2.98611 -1.6422
0.0119 0.0101 0.00075 0.00587 0.00236 0.00294 0.0558 0.0385 0.0304
2R:8069980-8070130:plus -31.8163 0.174312 2.08257 0.59633 -26.4898 -27.5306 -25.2202 5.19444 1.95413
0.016 0.00971 0.0056 0.023 0.0183 0.0374 0.0485 0.00241 0.00964
2R:8086360-8086510:minus -32.8469 -0.110092 13.156 4.43119 10.3878 -27.3776 -13.3303 -0.25
9.55046 0.0243 0.0107 0.0000178 0.00495 0.0000174 0.0351 0.00624 0.0181 0.000321
2R:8086360-8086510:plus -32.0714 0.761468 4.62385 5.66055 -17.4184 -8.45918 -26.9083 2.90278 -2.36697
0.0176 0.00789 0.00208 0.00317 0.00457 0.00111 0.0598 0.00619 0.0371
2R:8088360-8088510:minus -24.1837 -0.412844 -2.02752 2.50459 10.9898 -18.1633 -15.6697 -5.40278
3.44954 0.00751 0.0119 0.0218 0.0114 3.28e-06 0.01 0.0102 0.0672 0.00594
2R:8088360-8088510:plus -23.1531 -0.412844 1.66972 6.2844 -18.7143 -8.5 -8.16514 3.36111 -2.3578
0.00542 0.0119 0.00652 0.00205 0.0136 0.00126 0.00218 0.00519 0.0369
2R:8146280-8146430:minus -33.5612 2.3211 4.9633 1.69725 -18.9796 0.795918 -17.0092 4.45833 3.83486
0.0288 0.00446 0.00181 0.0163 0.0155 0.000155 0.0128 0.00332 0.00481
2R:8146280-8146430:plus -42.551 5.22018 5.23853 5.22018 -19.051 0.683673 -27.4312 5.25 -1.22018
0.0803 0.00149 0.00161 0.00418 0.0162 0.000184 0.0639 0.00235 0.0278
2R:8146800-8146950:minus -32.4388 0.348624 0.779817 2.9633 -8.04082 -16.9796 -16.4495 -1.83333 5.18349
0.0195 0.00914 0.00892 0.00914 0.000831 0.0044 0.0117 0.0285 0.00295
2R:8146800-8146950:plus -21.9286 0.513761 -0.678899 5.91743 -18.1939 -17.0816 -5.98165 13.1667
0.550459 0.0027 0.00862 0.0144 0.00267 0.0086 0.00487 0.0013 8.51e-06 0.0161
2R:8148960-8149110:minus -40.102 -0.0183486 0.577982 -0.211009 -18.7551 -28.4082 -6.20183 6.77778
11.8532 0.036 0.0104 0.00955 0.0286 0.0144 0.0615 0.00138 0.00113 0.0000428
2R:8148960-8149110:plus -30.551 1.53211 2.48624 0.66055 -17.3776 -18.1224 -13.2018 -2.72222 7.02752
0.00959 0.00596 0.00483 0.0225 0.00419 0.00988 0.00605 0.036 0.00133
2R:8149780-8149930:minus -30.7041 -1.40367 5.74312 9.24771 -26.9694 -18.1224 -15.5688 3.06944 -0.669725
0.00993 0.0163 0.00129 0.000409 0.023 0.00988 0.01 0.00581 0.0236
2R:8149780-8149930:plus -30.9286 -4.3211 11.2936 8.98165 -18.4592 -9.64286 -18.8257 1.38889 -0.330275
0.0104 0.0369 0.0000665 0.000461 0.0109 0.00339 0.017 0.0107 0.0208
2R:8151380-8151670:minus -31.7755 0.752294 2.49541 2.30275 -18.449 -18.2041 -26.0917 8.93056 -0.302752
0.0157 0.00791 0.00481 0.0124 0.0107 0.0103 0.0541 0.000335 0.0208
2R:8151380-8151670:plus -14.449 4.47706 0.59633 2.6422 -17.7143 0.826531 -14.2018 -0.583333
0.816514 0.0014 0.002 0.00949 0.0107 0.00627 0.000153 0.00758 0.02 0.015
2R:8201500-8201650:minus -33.2653 -1.86239 -4.81651 0.908257 -19.051 -9.02041 -15.1743 2.86111 -1.3211
0.0277 0.0187 0.0471 0.0211 0.0162 0.00213 0.00929 0.00629 0.0284
2R:8201500-8201650:plus -20.6633 0.577982 10.0183 9.49541 -18.1939 0.316327 -2.22936 -5.58333 -3.04587
0.00173 0.00843 0.00014 0.000308 0.0086 0.000243 0.000469 0.0698 0.0451
2R:8208660-8208810:minus -32.0714 -3.99083 -0.211009 11.0275 -18.9796 0.755102 -23.8624 1.55556
3.46789 0.0176 0.0337 0.0124 0.0000401 0.0155 0.000175 0.0402 0.0101 0.00582
2R:8208660-8208810:plus -51.4388 6.54128 0.899083 -0.12844 -18.4184 -17.6429 -19.0734 1.58333 1.95413
0.181 0.000818 0.00856 0.0278 0.0101 0.00619 0.0177 0.00997 0.00964
2R:8213620-8213890:minus -13.8878 -3.81651 1.43119 11.2936 -7.93878 -17.8265 -13.5138 -2.5 2.00917

0.00111 0.0322 0.0071 0.0000139 0.000767 0.00699 0.0065 0.034 0.00934
2R:8213620-8213890:plus -32.398 -2.19266 -0.706422 2.62385 -18.6429 -19.3776 -11.8716 2.47222
-1.11009 0.019 0.0205 0.0146 0.0108 0.0121 0.0195 0.00448 0.00727 0.0269
2R:8222680-8222830:minus -23.3776 -0.110092 -0.275229 -1 -8.67347 -18.3061 -11.0183 -6.59722
-4.6055 0.00619 0.0107 0.0127 0.0367 0.00154 0.0114 0.00375 0.0857 0.0656
2R:8222680-8222830:plus -22.1531 13.9725 -1.69725 6.52294 -18.1531 -8.5 -13.3028 3.26389 -0.486239
0.0031 8.89e-06 0.0197 0.00174 0.00829 0.00126 0.0062 0.00539 0.0219
2R:8225440-8225590:minus -22.7755 -1.82569 -1.54128 1.62385 -17.1531 -16.5 -21.9541 1.38889 -2.30275
0.00412 0.0185 0.0188 0.0168 0.00364 0.00416 0.0292 0.0107 0.0363
2R:8225440-8225590:plus -32.2653 9.23853 7.98165 3.31193 -27.5306 1.09184 -21.8899 0.819444 -0.541284
0.0184 0.000198 0.000442 0.00786 0.0351 0.000091 0.0289 0.0129 0.0223
2R:8230480-8230630:minus -30.8163 0.183486 5.40367 2.78899 -27.1531 -17.051 -20.578 -1.79167 0.642202
0.0103 0.00968 0.0015 0.00992 0.0249 0.00479 0.0228 0.0282 0.0157
2R:8230480-8230630:plus -31.6633 1.85321 1.04587 6.54128 -16.3776 -17.2755 -16.5963 0.833333 -0.33945
0.0141 0.0053 0.00813 0.00173 0.003 0.00544 0.012 0.0128 0.0209
2R:8231520-8231670:minus -23.3367 4.22018 -0.513761 1.56881 -8.86735 -36.8878 -20.1743 -1.375
3.05505 0.00614 0.00221 0.0137 0.0172 0.00173 0.123 0.0212 0.0251 0.00692
2R:8231520-8231670:plus -23.398 4.20183 2.41284 2.66972 -7.71429 -17.5 -15.2477 2 6.63303
0.00619 0.00223 0.00496 0.0106 0.000572 0.00599 0.00943 0.00862 0.00149
2R:8231980-8232130:minus -14.9796 1.79817 7.36697 4.22018 -18.0816 -17.2755 -18.8349 7.36111 1.79817
0.00161 0.00541 0.000605 0.00531 0.00759 0.00544 0.017 0.000832 0.0102
2R:8231980-8232130:plus -32.9694 15.4771 0.266055 2.34862 -18.7857 -9.72449 -24.5321 1.88889 -0.229358
0.0254 1.08e-06 0.0106 0.0121 0.0148 0.00352 0.0443 0.00896 0.0202
2R:8272500-8272650:minus -31.3265 13.7431 3.74312 2.73394 -16.7143 -9.30612 -7.09174 -2.16667 1.2844
0.0114 0.0000115 0.00297 0.0101 0.00319 0.00257 0.00172 0.0312 0.0129
2R:8272500-8272650:plus -29.1429 13.4592 1.25688 4.49541 -26.898 -17.7857 -19.0275 -3.51389 -7.90826
0.00834 0.000064 0.00755 0.00487 0.0203 0.0068 0.0175 0.0439 0.133
2R:827940-828090:minus -22.2653 0.275229 3.46789 5.69725 -18.4184 -9.82653 -20.3578 4.625 3.45872
0.00331 0.00938 0.00331 0.00314 0.0101 0.00381 0.0219 0.00309 0.00591
2R:827940-828090:plus -33.7755 0.541284 2.6422 3.13761 0.877551 -18.3061 -20.7523 -1.54167 -6.14679
0.0297 0.00854 0.00455 0.00854 0.00019 0.0114 0.0235 0.0263 0.0921
2R:8310960-8311110:minus -34.602 2.81651 -3.57798 -1.62385 -17.7857 -8.72449 -25.211 4.06944 3.24771
0.032 0.00372 0.0339 0.0451 0.00676 0.00169 0.0485 0.00391 0.00636
2R:8310960-8311110:plus -31.551 2.2844 14.4128 7.29358 -7.93878 -8.57143 -15.9083 -0.194444
-2.11009 0.0134 0.00452 4.28e-06 0.00104 0.000767 0.00149 0.0107 0.0178 0.0343
2R:8313000-8313150:minus -41.0306 8.83486 -2.89908 2.19266 -28.7959 -17.2755 -26.4771 6.02778 1.72477
0.0464 0.000244 0.028 0.0131 0.0641 0.00544 0.0567 0.00164 0.0106
2R:8313000-8313150:plus -13.0816 4.59633 13.6422 7.62385 -17.9796 -18.7857 -13.4128 -1.72222 -1.14679
0.000599 0.00191 0.0000116 0.000759 0.00726 0.0146 0.00635 0.0276 0.0272
2R:8318560-8318710:minus -32.6735 -2.53211 0.697248 3.52294 10.3878 -17.2755 -22.211 1.73611 7.16514
0.0216 0.0226 0.00917 0.00706 0.0000174 0.00544 0.0306 0.00946 0.00122
2R:8318560-8318710:plus -24.1429 15.4771 1.15596 3.30275 10.3878 -18.0102 -21.4128 1.13889 -4.37615
0.0074 1.08e-06 0.00783 0.00789 0.0000174 0.00812 0.0265 0.0116 0.0617
2R:8323360-8323510:minus -31.7041 7.94495 1.88991 10.0275 -8.60204 -26.3367 -14.3853 -2.84722 -3.48624
0.0146 0.000394 0.00602 0.000156 0.00125 0.0221 0.00789 0.0372 0.0494
2R:8323360-8323510:plus -23.2959 4.02752 15.211 2.95413 -26.3061 -8.79592 -7.80734 4.375 -4.04587
0.00605 0.00238 1.07e-06 0.00917 0.0181 0.0019 0.00202 0.00344 0.0563
2R:8324680-8324830:minus -23.0306 1.7156 1.86239 -0.0733945 -18.7551 -9.5 -14.7064 3.36111 3.93578
0.00505 0.00557 0.00608 0.0274 0.0144 0.00294 0.00845 0.00519 0.00456
2R:8324680-8324830:plus -23.2245 2.72477 0.605505 7.27523 10.3878 -19.2041 -19.6055 -2.08333 2.6422
0.00576 0.00385 0.00946 0.00109 0.0000174 0.0179 0.0193 0.0305 0.00768
2R:8340720-8340870:minus -30.7041 3.59633 5.78899 9.24771 -18.5612 -9.45918 -16.7615 1.84722 8.11009
0.00993 0.00279 0.00127 0.000409 0.0119 0.00279 0.0123 0.0091 0.000597
2R:8340720-8340870:plus -13.5204 -1.33028 3.42202 5.73394 -17.7857 -26.1224 4.14679 8.06944 7.06422

0.00097 0.0159 0.00337 0.00307 0.00676 0.0217 0.0000782 0.000561 0.00131
2R:8341580-8341730:minus -32.2143 -3.12844 7.27523 6.73394 -18.7551 -18.051 -15.5413 3.20833 -0.743119
0.0182 0.0267 0.000635 0.00152 0.0144 0.00886 0.00997 0.00551 0.0243
2R:8341580-8341730:plus -23.0408 -5.31193 0.137615 9.59633 -17.7143 0.0918367 -20.6239 -5.13889
1.10092 0.00512 0.0479 0.0111 0.000275 0.00627 0.000323 0.023 0.0636 0.0138
2R:8355680-8355830:minus -31.5918 -1.00917 0.366972 3.54128 -17.7857 -18.3469 -20.2018 -0.75 -2.25688
0.0136 0.0144 0.0103 0.00702 0.00676 0.0124 0.0213 0.021 0.0357
2R:8355680-8355830:plus -22.2551 -1.20183 -0.357798 5.57798 -19.0102 -18.5 -15.8899 1.47222
1.11927 0.00327 0.0153 0.013 0.0034 0.0157 0.0131 0.0106 0.0104 0.0137
2R:8393640-8393790:minus -21.9286 -0.394495 7.9633 3.69725 -18.0102 -18.0102 -20.5229 5.06944
5.00917 0.0027 0.0118 0.000446 0.00665 0.00732 0.00812 0.0226 0.00255 0.0031
2R:8393640-8393790:plus -34.1429 0 1.79817 1.91743 -9.23469 -16.3469 -18.633 3.01389 -0.706422
0.0313 0.0103 0.00623 0.015 0.00277 0.00416 0.0165 0.00593 0.024
2R:8394180-8394330:minus -39 -1.08257 -0.522936 2.93578 -26.8571 -18.0816 -20.844 0.597222 6.89908
0.0325 0.0147 0.0137 0.00927 0.0198 0.00934 0.0239 0.0139 0.00137
2R:8394180-8394330:plus -31.6224 1.65138 6.58716 3.87156 -8.89796 -17.0102 -16.5505 2.875 -1.43119
0.0137 0.00571 0.00088 0.00618 0.00189 0.00453 0.0119 0.00626 0.029
2R:8394820-8394970:minus -32.7041 1.72477 5.99083 10.5872 -26.9694 -0.102041 -22.9541 4.47222
-0.486239 0.0222 0.00555 0.00116 0.000115 0.023 0.000443 0.0348 0.0033 0.0219
2R:8394820-8394970:plus -40.6633 -0.12844 1.77982 2.83486 -27.2653 -9.08163 -7.3945 5.54167 2.82569
0.0426 0.0108 0.00627 0.0097 0.0302 0.00216 0.00185 0.00205 0.0074
2R:8402100-8402250:minus -24.5612 15.5963 -1.72477 5.73394 -17.0816 -17.5 -22.7156 0.694444 0.816514
0.00819 8.91e-07 0.0199 0.00307 0.00334 0.00599 0.0334 0.0134 0.015
2R:8402100-8402250:plus -42.1837 -1.55963 -2.63303 1.56881 -8.70408 -18.3776 -11.7706 2.31944 6.17431
0.0706 0.017 0.026 0.0172 0.00155 0.0125 0.00439 0.00769 0.00171
2R:8402560-8402710:minus -30.4796 2.49541 -0.541284 1.92661 -18.1531 -17.7143 -19.633 5.09722
1.15596 0.00941 0.00418 0.0138 0.0148 0.00829 0.00634 0.0193 0.00251 0.0135
2R:8402560-8402710:plus -31.7755 14.7523 -1.74312 0.522936 -18.7143 -8.42857 -19.5413 7.94444 -2.17431
0.0157 3.48e-06 0.02 0.0234 0.0136 0.00104 0.0191 0.000602 0.035
2R:8406500-8406650:minus -40.1429 4.40367 3.21101 1.91743 10.6531 -9.27551 -12.6239 -1.65278 -0.12844
0.0363 0.00206 0.00366 0.015 0.0000102 0.00244 0.0053 0.0271 0.0193
2R:8406500-8406650:plus -12.3673 3.92661 4.65138 6.84404 -17.1531 -18.5714 -5.3945 6.80556 0.495413
0.000396 0.00247 0.00206 0.0014 0.00364 0.0136 0.00111 0.00111 0.0162
2R:8416400-8416550:minus -14.4082 7.13761 6.53211 6.19266 -18.6735 -9.09184 -21.8624 3.48611 3.42202
0.00136 0.000604 0.000904 0.00216 0.0123 0.00219 0.0288 0.00494 0.00597
2R:8416400-8416550:plus -12.7755 4.73394 0.642202 8.05505 -27.6735 0.826531 -13.9358 -3.91667 -2.14679
0.000477 0.00181 0.00935 0.000578 0.0375 0.000153 0.00715 0.0483 0.0346
2R:8416800-8416950:minus -23 7.85321 3.55963 6.33028 -36.0408 -7.5 -21.5046 6.11111 -2.26606 0.00498
0.000413 0.0032 0.00198 0.0725 0.000614 0.027 0.00157 0.0359
2R:8416800-8416950:plus -13.8163 3.83673 0.376147 1.44954 -27.3367 -18.051 -13.0459 2.72222 4.51376
0.00109 0.00115 0.0102 0.0179 0.0317 0.00886 0.00584 0.00663 0.0036
2R:8457280-8457430:minus -22.8878 -1.3211 4.10092 2.61468 -27.602 -18.4184 -12.9174 3.90278 -2.56881
0.00441 0.0159 0.00257 0.0109 0.0364 0.0127 0.00567 0.00418 0.0396
2R:8457280-8457430:plus -30.5918 3.2844 2.11927 -1.22936 -8.90816 -17.2347 -27.6606 -2.44444 3.56881
0.00963 0.00313 0.00553 0.0395 0.00192 0.00511 0.0658 0.0335 0.00543
2R:8459460-8459610:minus -24.2245 4.77064 1.3211 1.88073 -27.2347 -8.53061 -12.6239 4.06944 5.65138
0.00763 0.00179 0.00738 0.0152 0.0283 0.00136 0.0053 0.00391 0.00228
2R:8459460-8459610:plus -31.5918 3.83673 0.743119 7.17431 -17.1531 9.60204 -21.422 -1.33333 -6.33028
0.0136 0.00115 0.00903 0.00115 0.00364 0.0000248 0.0266 0.0248 0.0963
2R:8474980-8475130:minus -21.9286 1.13761 12.7339 5.49541 -18.1939 -9.7551 -15.8624 1.01389 5.84404
0.0027 0.00689 0.0000257 0.00355 0.0086 0.00358 0.0106 0.0121 0.00202
2R:8474980-8475130:plus -12.8571 -1.21101 -0.770642 6.84404 -17.7551 -8.34694 -30.6697 -4.52778
5.25688 0.000505 0.0153 0.0149 0.0014 0.00658 0.000993 0.0998 0.0556 0.0028
2R:8475700-8475850:minus -14.0408 0.394495 -0.706422 5.55046 -18.3469 -8.16327 5.34862 -3.41667 -9

0.00115 0.00899 0.0146 0.00345 0.00894 0.000783 0.0000511 0.0428 0.166
2R:8475700-8475850:plus -13.5204 -1.59633 0.486239 9.44037 -27.3061 -18.8571 -2.56881 4.91667 3.45872
0.00097 0.0172 0.00986 0.000339 0.0315 0.0151 0.000509 0.00272 0.00591
2R:8476700-8476850:minus -4.26531 5.68807 7.11009 1.3211 -18.7143 -9.38776 -17.7523 1.38889 -2.46789
0.000132 0.00122 0.000691 0.0189 0.0136 0.00266 0.0144 0.0107 0.0384
2R:8476700-8476850:plus -23.1939 -4.44954 2.74312 5.57798 -18.1531 -9.5 -19.5963 0.5 3.77064 0.00558
0.0381 0.00438 0.0034 0.00829 0.00294 0.0192 0.0143 0.00491
2R:8477160-8477310:minus -21.5918 1.46789 -4.07339 2.45872 -17.0816 -17.2041 -13.6881 -1.58333 1.06422
0.00209 0.0061 0.0387 0.0116 0.00334 0.00507 0.00676 0.0266 0.014
2R:8477160-8477310:plus -23.6224 5.0367 0.522936 4.34862 -7.67347 -9.60204 7.80734 2.95833
-0.0642202 0.00658 0.0016 0.00974 0.00507 0.00055 0.00334 0.000025 0.00606 0.0188
2R:8479240-8479390:minus -14.449 1.73394 1.69725 7.70642 -17.3776 -0.173469 -14.2385 -1.81944
8.88073 0.0014 0.00553 0.00646 0.000708 0.00419 0.000456 0.00765 0.0284 0.000468
2R:8479240-8479390:plus -22.3673 -3.22936 3.44954 5.34862 -17.1837 -9.45918 -14.2752 -2.77778 7.15596
0.00341 0.0275 0.00334 0.00387 0.00366 0.00279 0.00771 0.0365 0.00124
2R:8479680-8479830:minus -31.6327 -2.36697 8.13761 5.90826 -9.16327 -19.0102 -19.4954 0.25 2.36697
0.0138 0.0216 0.000407 0.00271 0.00249 0.0159 0.0189 0.0155 0.00828
2R:8479680-8479830:plus -22.7449 -4.82569 1.38532 -1.08257 -27.7551 -17.5714 -18.7798 -3.52778 12.2752
0.00404 0.0421 0.00721 0.0378 0.0385 0.00616 0.0169 0.044 0.0000141
2R:8481040-8481190:minus -21.6224 -1.15596 6.7156 7.55963 -18.4082 -27.5612 -6.11009 -1.93056 8.10092
0.00213 0.0151 0.000828 0.000807 0.00937 0.0393 0.00135 0.0293 0.000623
2R:8481040-8481190:plus -14.2245 0.0550459 4.00917 3.20183 -27.1224 -8.79592 4.6422 -2.02778
5.59633 0.00126 0.0101 0.00267 0.00824 0.0245 0.0019 0.0000654 0.03 0.00234
2R:8488480-8488630:minus -11.449 0.688073 4.57798 5.57798 -27 -9.23469 -13.4128 3.88889 -0.440367
0.000252 0.0081 0.00212 0.0034 0.0233 0.00238 0.00635 0.00421 0.0216
2R:8488480-8488630:plus -14.2551 -1.49541 3.92661 2.13761 -26.1939 -25.898 2.55963 4.68056 5.65138
0.00127 0.0167 0.00276 0.0134 0.0175 0.0214 0.000138 0.00302 0.00228
2R:8500580-8500730:minus -31.7041 3.97959 -1.73394 2.88991 -17.7143 -18.8571 -19.6881 0.180556 3.19266
0.0146 0.000669 0.02 0.00942 0.00627 0.0151 0.0195 0.0158 0.00652
2R:8500580-8500730:plus -32.2857 -2.07339 2.86239 4.0367 -7.60204 -9.57143 -12.633 1.80556 1.33028
0.0185 0.0198 0.00418 0.00578 0.000466 0.00326 0.00532 0.00923 0.0127
2R:8502040-8502190:minus -33.3265 1.45872 -1.02752 6.62385 -18.7449 -8.53061 -18.1009 5.09722 3.20183
0.0279 0.00613 0.0161 0.00163 0.0138 0.00136 0.0152 0.00251 0.0065
2R:8502040-8502190:plus -32.102 -0.348624 2.80734 5.22936 -17.7551 -18.0102 -13.3119 1.43056
7.6422 0.0177 0.0116 0.00427 0.0041 0.00658 0.00812 0.00621 0.0105 0.000893
2R:8513120-8513270:minus -31.551 0.0183486 0.431193 0.733945 -28.5612 -8.79592 -11.5321 6.91667
3.56881 0.0134 0.0102 0.01 0.0222 0.0617 0.0019 0.00417 0.00105 0.00543
2R:8513120-8513270:plus -13.1531 -2.91743 -1.81651 1.53211 -17.7959 -18.9286 -17.6606 2.375 3.83486
0.00067 0.0252 0.0205 0.0174 0.00678 0.0153 0.0142 0.00753 0.00481
2R:8513640-8513790:minus -24.5612 3.90816 -0.752294 1.18349 -9.23469 0.755102 -28.2752 1.27778
3.20183 0.00819 0.00102 0.0148 0.0196 0.00277 0.000175 0.0714 0.0111 0.0065
2R:8513640-8513790:plus -32.8061 1.75229 -3.38532 4.9633 -18.7551 0.0510204 -27.0734 4.45833
-6.00917 0.0237 0.0055 0.0321 0.0044 0.0144 0.000349 0.061 0.00332 0.089
2R:8519780-8519930:minus -12.1122 0.247706 14.2385 7.29358 -18.0816 -17.7143 -19.844 -1.86111 4.19266
0.000336 0.00947 5.44e-06 0.00104 0.00759 0.00634 0.02 0.0287 0.00401
2R:8519780-8519930:plus -29.7041 4.55046 -0.00917431 1.98165 -18.4898 -9.57143 -15.5138 2.875
0.146789 0.00848 0.00195 0.0116 0.0145 0.0116 0.00326 0.00992 0.00626 0.0178
2R:8523320-8523470:minus -23.0714 2.45872 -1.18349 7.15596 -8.63265 -28.5306 -11.6881 1.18056 6.18349
0.0052 0.00424 0.0169 0.00117 0.0013 0.0625 0.00431 0.0114 0.00167
2R:8523320-8523470:plus -22.4898 0.412844 0.201835 9.7156 -28.3061 -9.79592 -18.5596 -3.15278 5.0367
0.00353 0.00893 0.0108 0.000242 0.0553 0.00377 0.0163 0.0402 0.00306
2R:8524060-8524210:minus -32.8163 2.46789 -1.44037 2.07339 -17.4898 -8.82653 -17.6972 1.80556 -2.2844
0.024 0.00422 0.0183 0.0138 0.0053 0.00191 0.0143 0.00923 0.0361
2R:8524060-8524210:plus -30.8367 10.1835 1.11927 2.72477 -26.9286 -16.9388 -10.9358 -2.86111 1.22936

0.0103 0.000122 0.00793 0.0102 0.0214 0.00433 0.00369 0.0373 0.0132
2R:8540040-8540190:minus -31.2449 14.7523 15.6239 6.02752 -18.4184 -8.93878 -19.6514 3.30556 -2.6055
0.0111 3.48e-06 5.44e-07 0.00246 0.0101 0.00201 0.0194 0.0053 0.04
2R:8540040-8540190:plus -40.551 6.79817 6.91743 1.07339 -18.4184 -18.1939 -15.7064 2.84722 5.23853
0.0418 0.00072 0.000755 0.0201 0.0101 0.0101 0.0103 0.00632 0.00285
2R:8553340-8553490:minus -3.03061 -0.192661 6.70642 10.8624 -8.16327 -9.30612 -9.94495 0.708333
-0.522936 0.0000417 0.011 0.000832 0.0000687 0.000873 0.00257 0.00305 0.0134 0.0222
2R:8553340-8553490:plus -23.7449 4.65138 9.37615 4.11927 -17.4184 -17.051 -8.9633 3.25 2.21101
0.00674 0.00187 0.000204 0.00551 0.00457 0.00479 0.00254 0.00542 0.00862
2R:8557700-8557850:minus -12.8571 4.21101 1.44954 9.61468 -18.449 -16.7143 -7.52294 -1.875 -4.26606
0.000505 0.00222 0.00705 0.000255 0.0107 0.00419 0.0019 0.0288 0.0595
2R:8557700-8557850:plus -12.6735 4.14679 2.22936 6.3578 -9.27551 -18.7143 -11.3761 2.13889 -3.29358
0.000461 0.00228 0.00531 0.00195 0.00284 0.014 0.00403 0.0082 0.0474
2R:8565380-8565530:minus -23.0714 5.56881 0.357798 0.990826 -28.0408 -8.5 -15.789 0.75 8.20183 0.0052
0.00128 0.0103 0.0206 0.0455 0.00126 0.0104 0.0132 0.000539
2R:8565380-8565530:plus -24.1837 -2.44954 -3.48624 0.119266 0.581633 -9.7551 -26.8532 3.27778 -10.6697
0.00751 0.0221 0.033 0.026 0.000292 0.00358 0.0594 0.00536 0.219
2R:8566040-8566190:minus -31.5918 -0.908257 1.25688 6.86239 -18.4898 -10.0204 -22.4495 0.819444
1.84404 0.0136 0.0139 0.00755 0.00136 0.0116 0.00399 0.0319 0.0129 0.01
2R:8566040-8566190:plus -22 0.33945 5.22936 5.54128 -18.9796 -9.60204 -16.4587 3.23611 -2.34862
0.00291 0.00917 0.00162 0.00346 0.0155 0.00334 0.0117 0.00545 0.0368
2R:8567100-8567250:minus -14.449 6.79817 4.05505 2.18349 -18.7857 0.0510204 -0.944954 1.77778
7.31193 0.0014 0.00072 0.00262 0.0131 0.0148 0.000349 0.000351 0.00932 0.00112
2R:8567100-8567250:plus -23.3673 -1.04587 3.6789 1.44954 -17.0408 -18.1939 -5.57798 11.2083 1.48624
0.00617 0.0145 0.00305 0.0179 0.00329 0.0101 0.00117 0.0000639 0.0118
2R:8592640-8592790:minus -31.7755 3.79817 0.798165 11.1927 -17.8163 -18.7143 -25.844 1.13889 1.95413
0.0157 0.00259 0.00886 0.0000254 0.00682 0.014 0.0525 0.0116 0.00964
2R:8592640-8592790:plus 8.04082 0.651376 -0.917431 11.2936 -26.9694 -17.4184 -7.61468 4.34722
-0.412844 1.35e-06 0.00821 0.0156 0.0000139 0.023 0.00585 0.00194 0.00348 0.0213
2R:8628640-8628790:minus -21.7755 -4.33945 6.40367 2.68807 -26.7449 -17.7143 -3.88991 3.55556 1.78899
0.00241 0.037 0.000962 0.0104 0.0195 0.00634 0.000723 0.00481 0.0103
2R:8628640-8628790:plus -13.5612 3.64286 5.43119 -0.0550459 -27.3061 -26.602 -12.945 2.98611
4.09174 0.000984 0.00159 0.00148 0.0272 0.0315 0.0248 0.00571 0.006 0.00416
2R:8643320-8643470:minus -23.7857 13.4592 2.54128 5.58716 -18.4082 -17.898 -16.7982 3.61111 -1.3578
0.00679 0.000064 0.00473 0.00334 0.00937 0.00726 0.0124 0.0047 0.0286
2R:8643320-8643470:plus -14.8571 5.82569 2.62385 9.44037 -18.7245 -26.051 -18.8532 3.40278 7.16514
0.0016 0.00114 0.00458 0.000339 0.0136 0.0216 0.017 0.00511 0.00122
2R:8643740-8643890:minus -33.2959 -1.12844 15.6972 10.5963 -27.2653 -8.53061 -27.1376 2.36111 -2.52294
0.0279 0.0149 3.11e-07 0.0001 0.0302 0.00136 0.0615 0.00757 0.0391
2R:8643740-8643890:plus -32.2959 4.15596 -0.697248 9.61468 10.0918 -8.72449 -14.2661 2.25 -2.00917
0.0186 0.00227 0.0145 0.000255 0.0000251 0.00169 0.00769 0.00788 0.0333
2R:8644980-8645130:minus -24.8265 -1.27523 13.1284 7.29358 -18.7143 -8.53061 -15.8532 4.11111 -0.275229
0.0083 0.0156 0.0000181 0.00104 0.0136 0.00136 0.0106 0.00384 0.0206
2R:8644980-8645130:plus -31.7755 4.51376 -0.0458716 2.62385 20.4694 -8.20408 -20.7615 1.77778
-0.146789 0.0157 0.00198 0.0118 0.0108 1.65e-07 0.000796 0.0236 0.00932 0.0194
2R:8648280-8648430:minus -21.5918 2.43119 -3.25688 2.45872 1.91837 -7.45918 -12.3394 0.791667 1.53211
0.00209 0.00428 0.031 0.0116 0.0000382 0.000582 0.00497 0.013 0.0116
2R:8648280-8648430:plus -24 3.83673 -1.7156 2.54128 -8.93878 -9.79592 -20.9174 2.65278 -5.72477
0.00719 0.00115 0.0198 0.0112 0.00216 0.00377 0.0242 0.0068 0.0837
2R:8737680-8737830:minus -31.398 -0.807339 2.43119 3.31193 -27.1939 -18.0102 -15.6881 -1.22222
-1.53211 0.0117 0.0135 0.00493 0.00786 0.0267 0.00812 0.0102 0.024 0.0296
2R:8737680-8737830:plus -23.9592 4.27523 2.91743 2.55046 -16.3776 -26.5612 -15.5138 -2.79167 -0.33945
0.00708 0.00217 0.0041 0.0112 0.003 0.0237 0.00992 0.0367 0.0209
2R:8756180-8756330:minus -23.0408 5.21101 2.14679 2.9633 -27.5306 9.67347 -16.2385 5.73611 12.2752

0.00512 0.00149 0.00547 0.00914 0.0351 0.0000217 0.0113 0.00188 0.0000141
2R:8756180-8756330:plus -34.6327 -2.70642 1.9633 2.17431 -17.1122 -17.2041 -14.8165 3.875 1.33028
0.032 0.0238 0.00586 0.0132 0.00341 0.00507 0.00864 0.00423 0.0127
2R:8757060-8757210:minus -21.5816 4.87156 1.47706 6.02752 -18.7143 -19.5714 -17.6697 0.986111 -0.770642
0.00205 0.00172 0.00698 0.00246 0.0136 0.0202 0.0142 0.0122 0.0245
2R:8757060-8757210:plus -41.551 5.24771 -0.486239 4.22018 -9.5 0.979592 -21.9817 1.56944 -4.23853
0.0607 0.00147 0.0136 0.00531 0.00293 0.000136 0.0294 0.01 0.0589
2R:8757640-8757790:minus -32.7857 -3.25688 -1.89908 1.84404 -27.5306 -8.45918 -21.7064 2.19444 -1.05505
0.0235 0.0277 0.021 0.0154 0.0351 0.00111 0.028 0.00804 0.0264
2R:8757640-8757790:plus -22.449 0.990826 3.79817 1.44037 -17.1531 -0.173469 -20.0092 -0.277778
2.06422 0.0035 0.00726 0.00291 0.018 0.00364 0.000456 0.0206 0.0182 0.00912
2R:8758900-8759050:minus -32.0102 3.97959 -1.00917 -0.623853 -9.20408 0.122449 -18.156 -1.41667
8.10092 0.0172 0.000669 0.016 0.0326 0.00262 0.000312 0.0153 0.0254 0.000623
2R:8758900-8759050:plus -31.4286 -0.0917431 1.83486 2.83486 -17.449 -9.30612 -15.8716 8.55556
1.02752 0.0119 0.0106 0.00614 0.0097 0.00484 0.00257 0.0106 0.000422 0.0141
2R:8759360-8759510:minus -31.6633 5.16514 6.00917 6.19266 0.836735 -17.0102 -17.8532 0.722222 -0.798165
0.0141 0.00152 0.00115 0.00216 0.000215 0.00453 0.0146 0.0133 0.0246
2R:8759360-8759510:plus -13.0306 2.3578 3.21101 7.63303 -17.9796 -8.5 -6.85321 -3.36111 7.6789
0.000567 0.0044 0.00366 0.000745 0.00726 0.00126 0.00163 0.0423 0.000866
2R:8760040-8760190:minus -42.6224 4.09174 1.57798 5.22936 -27.3776 0.459184 -13.2844 6.84722 -1.11927
0.0826 0.00232 0.00674 0.0041 0.032 0.000213 0.00617 0.00109 0.027
2R:8760040-8760190:plus -39.9184 -2.22018 4.99083 2.55963 -18.5204 -16.051 -19.8624 3.54167 4.0367
0.0344 0.0207 0.00178 0.0111 0.0117 0.00408 0.0201 0.00483 0.00424
2R:8766380-8766530:minus -13.6735 0.651376 2.68807 5.06422 -18.4184 -19.2755 -13.0092 -4.55556 3.14679
0.00103 0.00821 0.00447 0.00433 0.0101 0.0185 0.00579 0.0559 0.00666
2R:8766380-8766530:plus -23.2959 0.899083 3.57798 2.66972 -7.89796 -28.602 -11.5413 3.86111 2.82569
0.00605 0.00751 0.00317 0.0106 0.000678 0.0648 0.00418 0.00425 0.0074
2R:8766980-8767130:minus -21.9592 -1.59633 2.29358 6.22936 -18.3469 -7.5 -22.3853 2.54167 -0.972477
0.00279 0.0172 0.00518 0.00209 0.00894 0.000614 0.0316 0.00709 0.0259
2R:8766980-8767130:plus -22.8878 -0.220183 -3.24771 1.84404 -18.9388 -9.5 -22.7431 -0.541667
-6.27523 0.00441 0.0111 0.0309 0.0154 0.0151 0.00294 0.0336 0.0197 0.0952
2R:8767680-8767830:minus -31.7755 -1.82569 2.6789 2.51376 -26.2653 -9.86735 -26.0183 3.20833 -2.05505
0.0157 0.0185 0.00449 0.0113 0.018 0.00391 0.0536 0.00551 0.0338
2R:8767680-8767830:plus -31.1735 14.2477 -1.31193 1.90826 -27.2347 -8.53061 -12.1193 6.95833 1.69725
0.0109 6.21e-06 0.0176 0.015 0.0283 0.00136 0.00473 0.00103 0.0108
2R:8803320-8803470:minus -23.5204 5.82569 3.48624 5.06422 -9.5 -18.3469 -3.02752 6.13889 -2.52294
0.00645 0.00114 0.00329 0.00433 0.00293 0.0124 0.000572 0.00155 0.0391
2R:8803320-8803470:plus -14.5612 9.43119 2.33945 2.55046 -8.71429 -17.9388 5.48624 7.11111 -1.01835
0.00147 0.000179 0.0051 0.0112 0.00159 0.00738 0.0000488 0.000949 0.0263
2R:8811240-8811390:minus -22.9286 -2.40367 -1.30275 9.3211 -8.60204 -8.23469 -14.7982 2.48611 -0.440367
0.0046 0.0218 0.0175 0.000391 0.00125 0.000875 0.00861 0.00723 0.0216
2R:8811240-8811390:plus -24.5918 6.17431 0.46789 2.41284 1.87755 -8.72449 -22.0734 0.347222 -2.26606
0.00821 0.000977 0.00992 0.0118 0.0000492 0.00169 0.0299 0.015 0.0359
2R:8812160-8812310:minus -31.1429 4.05505 0.256881 4.65138 -8.60204 -9.60204 -19.5963 4.97222 5.00917
0.0108 0.00235 0.0106 0.00471 0.00125 0.00334 0.0192 0.00266 0.0031
2R:8812160-8812310:plus -20.9286 2.90826 2.05505 2.22018 -8.86735 -17.4898 -22.5872 5 -4.57798
0.00183 0.0036 0.00566 0.0129 0.00173 0.0059 0.0327 0.00263 0.0651
2R:8814960-8815110:minus -29.9184 0.926606 1.15596 6.00917 -17.1531 -26.5612 -16.2018 0.0694444
2.3211 0.00854 0.00743 0.00783 0.00255 0.00364 0.0237 0.0112 0.0164 0.00839
2R:8814960-8815110:plus -31.8469 1.17431 0.752294 2.81651 -17.4898 -9.53061 -17.3394 8.02778 -0.174312
0.0162 0.00679 0.009 0.00982 0.0053 0.0031 0.0135 0.000574 0.0196
2R:8815920-8816070:minus -32.8061 -0.724771 2.75229 6.00917 -18.4184 -18.0816 -6.78899 2.97222
7.6422 0.0237 0.0131 0.00436 0.00255 0.0101 0.00934 0.0016 0.00603 0.000893
2R:8815920-8816070:plus -13.4184 -5.21101 3.15596 6.66055 -17.0816 -16.9796 -12.8165 -9.30556 0.59633

0.000873 0.0466 0.00374 0.00158 0.00334 0.0044 0.00554 0.138 0.016
2R:8817000-8817150:minus -12.8469 5.0367 4.59633 7.47706 -16.7143 -26.5612 -13.9725 -0.208333
-2.66055 0.000502 0.0016 0.0021 0.00085 0.00319 0.0237 0.00721 0.0178 0.0406
2R:8817000-8817150:plus -32.4388 3.25688 0.0642202 3.78899 -18.7143 -8.79592 -21.156 7.90278
3.88073 0.0195 0.00316 0.0113 0.00632 0.0136 0.0019 0.0253 0.000616 0.00475
2R:8821440-8821590:minus -40.0612 1.53211 5.11009 2.04587 -9.23469 -8.72449 -13.3853 0.625 1.3211
0.0358 0.00596 0.0017 0.014 0.00277 0.00169 0.00631 0.0137 0.0127
2R:8821440-8821590:plus -31.5204 14.5138 3.54128 6.6422 -26.9694 -9.0102 -22.4679 3.36111 -0.605505
0.0131 4.77e-06 0.00322 0.00161 0.023 0.00208 0.032 0.00519 0.023
2R:8823920-8824070:minus -31.2551 1.59633 0.559633 3.45872 -8.90816 -27.602 -14.1284 1.04167 1.66972
0.0113 0.00582 0.00961 0.00731 0.00192 0.0416 0.00746 0.012 0.011
2R:8823920-8824070:plus -32.551 1.80734 0.743119 10.0275 -8.23469 -19.1224 -25.3394 -7.84722 10.3486
0.0207 0.00539 0.00903 0.000156 0.000927 0.0175 0.0493 0.108 0.0000988
2R:8844980-8845130:minus -31.3265 -0.477064 2.11009 1.92661 -17.9388 -0.173469 -19.2661 -3.97222
-3.92661 0.0114 0.0121 0.00554 0.0148 0.00715 0.000456 0.0182 0.0489 0.0546
2R:8844980-8845130:plus -33 -0.220183 -0.431193 9.92661 -17.5612 10.5306 -18.4587 3.65278
-0.0366972 0.0258 0.0111 0.0133 0.000169 0.00544 5.67e-06 0.016 0.00462 0.0187
2R:8846520-8846670:minus -24.1429 -2.90826 3.97248 1.92661 -27 -8.45918 -12.0092 4.13889 0.513761
0.0074 0.0251 0.00271 0.0148 0.0233 0.00111 0.00462 0.0038 0.0162
2R:8846520-8846670:plus -13.9592 -0.431193 4.09174 4.31193 -17.4898 -27.6327 -13.7982 1.08333
5.53211 0.00113 0.0119 0.00258 0.00516 0.0053 0.043 0.00693 0.0118 0.00245
2R:8867900-8868050:minus -12.8878 -2.33028 0.944954 4.33945 -18.7245 -8.20408 -5.04587 7.41667 3.66972
0.000523 0.0214 0.00842 0.00509 0.0136 0.000796 0.00101 0.000807 0.00514
2R:8867900-8868050:plus -32.6224 -2.91743 5.08257 5.43119 0.581633 -9.23469 -12.6789 7.72222 -0.880734
0.0211 0.0252 0.00172 0.00371 0.000292 0.00238 0.00537 0.000682 0.0252
2R:8868240-8868390:minus -3.55102 -1.22936 3.10092 7.34862 -18.7143 -8.93878 13.789 5.22222 5.18349
0.0000767 0.0154 0.00382 0.000998 0.0136 0.00201 2.36e-06 0.00238 0.00295
2R:8868240-8868390:plus -31.102 1.08257 0.568807 8.05505 -8.60204 -17.9388 -16.9817 3.45833 -0.394495
0.0106 0.00703 0.00958 0.000578 0.00125 0.00738 0.0128 0.005 0.0212
2R:8872360-8872510:minus -30.7041 -1.80734 6.30275 3.87156 -17.449 -16.2755 -17.4312 -3.11111 1.52294
0.00993 0.0184 0.00101 0.00618 0.00484 0.00413 0.0137 0.0397 0.0117
2R:8872360-8872510:plus -32.8469 -6.18349 4.55963 6.13761 -18.3776 -17.1224 -12.5688 -0.138889
2.72477 0.0243 0.0598 0.00214 0.00226 0.00926 0.00499 0.00524 0.0175 0.00759
2R:8878320-8878470:minus -32.4388 2.16514 7.90826 6.41284 -7.67347 -8.27551 -27.6239 -0.319444
-4.41284 0.0195 0.00473 0.00046 0.00189 0.00055 0.000919 0.0655 0.0184 0.0624
2R:8878320-8878470:plus -21.449 0.0275229 9.00917 5.94495 -18.0816 -18.0816 -10.5872 1.86111
2.29358 0.002 0.0102 0.000253 0.00261 0.00759 0.00934 0.00344 0.00905 0.0084
2R:8878560-8878710:minus -32.4388 2.16514 7.90826 6.41284 -7.67347 -8.27551 -27.6239 -0.319444
-4.41284 0.0195 0.00473 0.00046 0.00189 0.00055 0.000919 0.0655 0.0184 0.0624
2R:8878560-8878710:plus -24.2143 1.17431 -1.06422 3.29358 -17.8265 -18.9286 -12.9908 1.61111 5.80734
0.00754 0.00679 0.0163 0.00791 0.00691 0.0153 0.00577 0.00988 0.00205
2R:8883860-8884010:minus -32.1531 -2.23853 4.88073 2.83486 -18.7857 -16.2755 -16.2018 5.41667 10.1284
0.0179 0.0208 0.00187 0.0097 0.0148 0.00413 0.0112 0.00218 0.000129
2R:8883860-8884010:plus -12.8776 2.74312 5.77064 9.76147 -18.1531 -17.8265 -4.73394 -1.27778 4.09174
0.000507 0.00382 0.00128 0.000212 0.00829 0.00699 0.00092 0.0244 0.00416
2R:8884600-8884750:minus -23.6224 2.94495 0.275229 2.09174 -17.1531 -17.5 -23.6881 -0.166667
10.3394 0.00658 0.00355 0.0106 0.0137 0.00364 0.00599 0.0392 0.0176 0.000112
2R:8884600-8884750:plus -22.9286 1.38532 4.54128 10.8624 -27.1939 0.27551 -14.8716 -2.26389 7.15596
0.0046 0.00629 0.00215 0.0000687 0.0267 0.000257 0.00874 0.032 0.00124
2R:8885100-8885250:minus -13.602 -5.01835 7.16514 7.45872 -17.8878 -18.7551 -14.8257 3.31944 3.56881
0.000995 0.0443 0.000671 0.000894 0.00704 0.0141 0.00866 0.00528 0.00543
2R:8885100-8885250:plus -21.7755 0.504587 0.183486 2.69725 -8.86735 -17.3469 -27.5872 3.70833 1.36697
0.00241 0.00865 0.0109 0.0104 0.00173 0.0058 0.0652 0.00452 0.0124
2R:8886240-8886390:minus -24.3673 5.10092 -0.605505 -0.100917 -18.4184 -9.0102 0.908257 -1.98611

8.10092 0.00791 0.00156 0.0141 0.0277 0.0101 0.00208 0.000226 0.0297 0.000623
2R:8886240-8886390:plus -32 4.33945 0.743119 1.90826 -18.449 -0.469388 -6.6055 0.861111 -2.81651
0.0171 0.00211 0.00903 0.015 0.0107 0.000533 0.00153 0.0127 0.0424
2R:8892640-8892790:minus -23.0408 -1.72477 3.66972 -1.93578 -27.0408 -27.5306 -12.4404 6.20833 9.34862
0.00512 0.0179 0.00306 0.0502 0.0242 0.0374 0.00509 0.0015 0.000371
2R:8892640-8892790:plus -21.6633 -3.13761 4.09174 7.12844 -17.1531 -8.65306 -13.4771 -1.88889 1.23853
0.00223 0.0268 0.00258 0.00118 0.00364 0.00156 0.00645 0.0289 0.0132
2R:8893660-8893810:minus -31.3673 4.42202 3.33028 4.0367 -27.4592 0.27551 -18.6514 5.91667 -4.02752
0.0116 0.00204 0.00349 0.00578 0.0329 0.000257 0.0165 0.00172 0.056
2R:8893660-8893810:plus -14.602 1.27523 2.00917 7.62385 -8.0102 -9.20408 -17.8257 1.02778 1.17431
0.00149 0.00655 0.00576 0.000759 0.000826 0.00225 0.0146 0.012 0.0134
2R:8901920-8902070:minus -14.7449 7.6422 -3.26606 6.00917 -9.23469 -18.2347 -6.95413 0.791667 3
0.00157 0.000462 0.0311 0.00255 0.00277 0.0104 0.00167 0.013 0.00702
2R:8901920-8902070:plus -30.1429 -1.92661 1.04587 5.69725 -26.9694 -9.0102 -16.5413 5.20833 1.6422
0.00866 0.019 0.00813 0.00314 0.023 0.00208 0.0119 0.00239 0.0111
2R:8903280-8903430:minus -21.8469 2.29358 7.55963 1.66055 -16.8163 -19.1939 -13.8349 1.84722 11.8073
0.00247 0.00451 0.00055 0.0166 0.00323 0.0178 0.00699 0.0091 0.0000538
2R:8903280-8903430:plus -13.6327 0.53211 3.80734 7.27523 -26.6327 0.0918367 -16.2202 0.791667
-6.82569 0.00101 0.00856 0.0029 0.00109 0.0188 0.000323 0.0113 0.013 0.109
2R:8919820-8919970:minus -29.4796 0.330275 7.50459 6.72477 -27.3061 0.683673 -27.6606 3.05556 9.9633
0.00844 0.00919 0.000565 0.00154 0.0315 0.000184 0.0658 0.00584 0.000171
2R:8919820-8919970:plus -22.1837 4.02752 -3.19266 2.66972 -18.3776 -18.0102 -20.2661 -1.01389 -6.10092
0.00315 0.00238 0.0304 0.0106 0.00926 0.00812 0.0216 0.0227 0.091
2R:8923020-8923170:minus -30.7755 -0.770642 4.77982 -1.01835 -18.4898 -8.5 -21.5505 4.80556 0.394495
0.0102 0.0133 0.00195 0.0369 0.0116 0.00126 0.0272 0.00286 0.0166
2R:8923020-8923170:plus -20.6939 6.10092 0.733945 -0.706422 -18.1531 0.867347 -7.66055 5.375
0.12844 0.00174 0.00101 0.00906 0.0335 0.00829 0.000144 0.00196 0.00222 0.0179
2R:8926340-8926490:minus -22.7755 5.14679 1.36697 3.48624 -17.3776 -17.9796 -12.055 -2.16667 11.8073
0.00412 0.00153 0.00726 0.0072 0.00419 0.00764 0.00467 0.0312 0.0000538
2R:8926340-8926490:plus -30.2143 2.75229 -0.944954 11.0183 -7.63265 -8.57143 -12.1009 2.38889
0.908257 0.0088 0.00381 0.0157 0.000052 0.000479 0.00149 0.00472 0.00749 0.0147
2R:9020520-9020670:minus -32.7347 3.69725 3.14679 6.57798 -18.7551 -17.5714 -22.8257 8.36111 -1.09174
0.0225 0.00269 0.00375 0.00167 0.0144 0.00616 0.0341 0.000474 0.0268
2R:9020520-9020670:plus -21.8469 2.88991 1.33945 6.57798 -28.1939 -16.9796 -5.97248 1.84722 3.66972
0.00247 0.00362 0.00733 0.00167 0.0482 0.0044 0.0013 0.0091 0.00514
2R:9040120-9040270:minus -21.3265 -4.91743 0.201835 3.74312 1.87755 -19.0102 -13.367 0.819444 3.44954
0.00193 0.0432 0.0108 0.00651 0.0000492 0.0159 0.00629 0.0129 0.00594
2R:9040120-9040270:plus -23.2245 4.90816 -1.02752 7.44037 -27.4592 -9.0102 -17.4954 3.625 -3.50459
0.00576 0.00023 0.0161 0.000912 0.0329 0.00208 0.0138 0.00468 0.0497
2R:9045840-9045990:minus -22.9592 -0.706422 2.26606 6.68807 -8.67347 -17.9388 -1.43119 -2.59722
6.37615 0.00475 0.0131 0.00524 0.00157 0.00154 0.00738 0.00039 0.0349 0.00159
2R:9045840-9045990:plus -32.551 6.6055 -3.78899 0.724771 -17.3776 19.0816 -12.1651 3.36111 2.93578
0.0207 0.000793 0.0359 0.0223 0.00419 1.12e-06 0.00478 0.00519 0.00717
2R:9046040-9046190:minus -29.4388 -4.85321 4.73394 3.69725 -18.6429 19.3776 -19.0826 2.16667 -3.40367
0.00842 0.0425 0.00199 0.00665 0.0121 3.24e-07 0.0177 0.00812 0.0486
2R:9046040-9046190:plus -23.7041 3.68367 -4.62385 3.7156 20.4694 -9.60204 -24.2936 3.36111 2.93578
0.00669 0.00144 0.0448 0.00655 1.65e-07 0.00334 0.0428 0.00519 0.00717
2R:9055520-9055670:minus -20.7041 4.14679 8.00917 9.59633 -8.67347 -18.0102 -13.6514 -1.54167 -1.22018
0.00175 0.00228 0.000435 0.000275 0.00154 0.00812 0.00671 0.0263 0.0278
2R:9055520-9055670:plus -23.0408 -0.697248 3 6.13761 -17.5306 -8.72449 -13.1101 -4.81944 -0.275229
0.00512 0.013 0.00397 0.00226 0.0054 0.00169 0.00593 0.0593 0.0206
2R:9056060-9056210:minus -32.5918 5.80734 0.908257 1.20183 -18.7551 -16.9796 -20.1193 2.47222 2.53211
0.021 0.00116 0.00853 0.0195 0.0144 0.0044 0.021 0.00727 0.00791
2R:9056060-9056210:plus -23.3367 3.64286 3.37615 7.27523 -17.4184 -9.7551 -17.8257 4.90278 7.16514

0.00614 0.00159 0.00343 0.00109 0.00457 0.00358 0.0146 0.00274 0.00122
2R:9058760-9058910:minus -5.19388 5.22018 14.9633 7.29358 -27.9286 -17.2755 -4.0367 4.22222 -0.33945
0.000229 0.00149 1.77e-06 0.00104 0.0412 0.00544 0.000754 0.00367 0.0209
2R:9058760-9058910:plus -30.8163 0.302752 11.0459 6 -17.7143 -26.9694 -14.0092 4.73611 1.25688
0.0103 0.00929 0.0000777 0.00256 0.00627 0.0304 0.00727 0.00295 0.0131
2R:9085800-9085950:minus -21.6224 4.68807 0.192661 3.36697 11.4286 9.67347 -22.8991 -0.472222
-2.72477 0.00213 0.00184 0.0109 0.00764 1.97e-06 0.0000217 0.0345 0.0193 0.0414
2R:9085800-9085950:plus -30.7041 7.90826 2.13761 1.41284 -9.23469 -17.6429 -15.7982 5.34722 -2.33028
0.00993 0.000401 0.00549 0.0183 0.00277 0.00619 0.0105 0.00225 0.0366
2R:9086300-9086450:minus -42.8163 3.93578 2.31193 1.33028 -27.4184 -0.316327 -20.6239 4.34722
-3.38532 0.0865 0.00246 0.00515 0.0188 0.0323 0.000524 0.023 0.00348 0.0483
2R:9086300-9086450:plus -33.3367 -1.43119 2.99083 6.16514 -8.60204 -7.94898 -20.3945 -1.76389 -6.3211
0.0281 0.0164 0.00399 0.00223 0.00125 0.00074 0.0221 0.028 0.096
2R:9092780-9092930:minus -30.4388 0.59633 12.1376 5.69725 -17.4898 -18.8571 -6.6789 4.05556 0.59633
0.00923 0.00837 0.000039 0.00314 0.0053 0.0151 0.00156 0.00393 0.016
2R:9092780-9092930:plus -21.5102 1.6789 12.844 7.29358 -26.9388 -7.27551 -0.165138 3.52778
1.31193 0.00202 0.00565 0.0000237 0.00104 0.0215 0.000561 0.000294 0.00486 0.0127
2R:9094120-9094270:minus -33.0714 0.431193 -2.73394 1.68807 -17.7143 -37.1837 -17.4312 2.68056 1.68807
0.0267 0.00887 0.0268 0.0164 0.00627 0.14 0.0137 0.00673 0.0109
2R:9094120-9094270:plus -22.9184 13.5306 13.4128 7.99083 -8.44898 -9.57143 -17.9725 -3 -4.91743
0.00447 0.0000206 0.0000141 0.00059 0.00109 0.00326 0.0149 0.0386 0.0715
2R:9102140-9102290:minus -31.102 5.88073 8.20183 11.1927 -18.0816 -18.8571 -19.7798 3.19444 6.37615
0.0106 0.00112 0.000393 0.0000254 0.00759 0.0151 0.0198 0.00554 0.00159
2R:9102140-9102290:plus -22.8163 14.4862 12.8899 3.90826 -17.1939 -17.051 -22.422 1.29167 0.93578
0.00418 5.19e-06 0.0000227 0.00603 0.00377 0.00479 0.0318 0.011 0.0145
2R:9109820-9109970:minus -20.7755 2.38532 1.95413 3.18349 10.9898 -17.5 -14.7431 3.66667 3.94495
0.00176 0.00436 0.00588 0.00837 3.28e-06 0.00599 0.00851 0.0046 0.00452
2R:9109820-9109970:plus -22.9184 -0.706422 1.61468 7.27523 -17.1531 -9.27551 -9.21101
1.30556 3.42202 0.00447 0.0131 0.00665 0.00109 0.00364 0.00244 0.00266 0.011 0.00597
2R:9110180-9110330:minus -22 -3.42202 4.33028 2.09174 -7.89796 -9.45918 -10.1376
-1.41667 5.12844 0.00291 0.0289 0.00235 0.0137 0.000678 0.00279 0.00316 0.0254 0.00301
2R:9110180-9110330:plus -31.551 -3.77982 1.51376 3.24771 -8.30612 -17.1633 -10.9358 -3.52778
-0.917431 0.0134 0.0319 0.00689 0.00808 0.00097 0.005 0.00369 0.044 0.0256
2R:9111120-9111270:minus -23.0714 -2.56881 6.3211 0.266055 -18.1531 -26.602 -15.0826
2.81944 5.44037 0.0052 0.0229 0.000999 0.0251 0.00829 0.0248 0.00912 0.00639 0.00259
2R:9111120-9111270:plus -24.7449 3.77064 4.13761 5.77982 -27.2755 -17.051 -13.8807 3.16667 2.25688
0.00827 0.00262 0.00254 0.00294 0.0302 0.00479 0.00706 0.0056 0.00853
2R:9117680-9117830:minus -31.4796 5.81651 5.29358 3.49541 -8.64286 -18.0816 -20.4495
4.80556 -0.678899 0.0128 0.00115 0.00157 0.00716 0.00139 0.00934 0.0223 0.00286 0.0237
2R:9117680-9117830:plus -33.3367 0.853211 6.44037 7.47706 -19.0102 -9.45918 -23.9725
2.13889 -7.24771 0.0281 0.00763 0.000945 0.00085 0.0157 0.00279 0.0409 0.0082 0.119
2R:9124700-9124850:minus -33.1122 -0.559633 7.02752 6.69725 -27.2347 -18.1224 -18.6789
3.70833 -5.10092 0.0271 0.0124 0.000718 0.00156 0.0283 0.00988 0.0166 0.00452 0.0743
2R:9124700-9124850:plus -33.6327 -1.66972 0.706422 7.10092 -28.1939 -9.53061 -18.7523
3.54167 -5.02752 0.029 0.0176 0.00914 0.00121 0.0482 0.0031 0.0168 0.00483 0.0732
2R:9126940-9127090:minus -31 -6.12844 13.4128 7.45872 2.14286 -17.0102 -14.3486 0.333333
0.614679 0.0105 0.059 0.0000141 0.000894 0.0000316 0.00453 0.00783 0.0151 0.0158
2R:9126940-9127090:plus 5.91837 3.25688 2.11927 9.08257 -27.3061 -7.42857 -3.88991 3.54167
-2.94495 7.25e-06 0.00316 0.00553 0.000444 0.0315 0.000567 0.000723 0.00483 0.0441
2R:9221120-9221270:minus -12.0408 1.05505 3 9.59633 -19.051 -8.5 -23.0275 7.5 1.58716
0.000311 0.00709 0.00397 0.000275 0.0162 0.00126 0.0352 0.000771 0.0113
2R:9221120-9221270:plus -23.5612 4.01835 3 7.00917 -9.0102 -28.4082 -5.01835 9.16667 3.92661
0.00653 0.00239 0.00397 0.00126 0.00236 0.0615 0.000998 0.000288 0.00461
2R:9222520-9222670:minus -21.551 6.91743 8.70642 6.11009 -18.3776 -18.7857 -17.8624 2.73611

-6.50459 0.00205 0.000678 0.000297 0.00233 0.00926 0.0146 0.0146 0.00659 0.101
2R:9222520-9222670:plus -23.1122 -2.19266 -0.0458716 0.40367 -18.4898 -9.5 -6.80734
3.94444 4.3945 0.0053 0.0205 0.0118 0.0242 0.0116 0.00294 0.00161 0.00411 0.00375
2R:9223380-9223530:minus -11.6327 -1.66055 3.3211 2.08257 -18.6429 -16.2755 -10.211 0.0277778
4.56881 0.000259 0.0176 0.00351 0.0137 0.0121 0.00413 0.0032 0.0166 0.00356
2R:9223380-9223530:plus -13.2245 -0.275229 0.889908 4.66972 -7.96939 -17.2347 -17.578 -0.680556
-1.21101 0.000738 0.0113 0.00859 0.00466 0.000785 0.00511 0.014 0.0206 0.0277
2R:9224200-9224350:minus -23.9286 0.146789 3.70642 1.06422 -27.1939 -18.051 -18.3394 -1.93056 5.25688
0.00701 0.0098 0.00302 0.0203 0.0267 0.00886 0.0157 0.0293 0.0028
2R:9224200-9224350:plus -41.2551 -1.6055 -1.74312 1.45872 -18.7857 -17.1224 -11.9541 2 1.84404
0.0513 0.0173 0.02 0.0179 0.0148 0.00499 0.00456 0.00862 0.01
2R:9224980-9225130:minus -32.8571 1.88073 -4.49541 10.0459 -26.1939 -25.8265 -3.63303 3.59722 8.0367
0.0244 0.00524 0.0433 0.000139 0.0175 0.0212 0.000673 0.00473 0.000654
2R:9224980-9225130:plus -23.0408 -2.22936 5.52294 -0.201835 -17.7551 -8.87755 -10.9174 -0.0277778
2.22018 0.00512 0.0208 0.00142 0.0285 0.00658 0.00198 0.00367 0.0169 0.00859
2R:9225600-9225750:minus -23.1122 1.66055 4.58716 4.04587 -18.1939 -18.0816 -17.4862 3.34722 7.11927
0.0053 0.00569 0.00211 0.00572 0.0086 0.00934 0.0138 0.00522 0.00125
2R:9225600-9225750:plus -31.8571 2.13761 9.87156 2.69725 -18.4082 -9.42857 -21.055 5.98611 -0.504587
0.0163 0.00477 0.000153 0.0104 0.00937 0.0027 0.0248 0.00167 0.0221
2R:9230360-9230510:minus -33 -0.174312 5.66972 3.13761 -18.3776 -18.2755 -8.38532 -3.80556 -0.495413
0.0258 0.0109 0.00134 0.00854 0.00926 0.0112 0.00227 0.047 0.022
2R:9230360-9230510:plus -24.4082 -1.20183 5.31193 2.66972 -17.7857 -17.0102 -16.2661 -1.91667 3.66972
0.00797 0.0153 0.00156 0.0106 0.00676 0.00453 0.0114 0.0291 0.00514
2R:9231820-9231970:minus -30.398 -1.54128 0.238532 4.65138 -18.7551 -25.602 -13.4771 4.47222 2.36697
0.00901 0.0169 0.0107 0.00471 0.0144 0.0207 0.00645 0.0033 0.00828
2R:9231820-9231970:plus -20.7449 0 2.86239 2.99083 -27.1531 0.387755 -12.3394 0.791667 6.04587
0.00176 0.0103 0.00418 0.00899 0.0249 0.000217 0.00497 0.013 0.00181
2R:9232320-9232470:minus -21.898 0.220183 0.93578 5.69725 -18.1939 -17.7857 -22.7064 -5.02778 3.08257
0.00259 0.00956 0.00845 0.00314 0.0086 0.0068 0.0334 0.0621 0.00681
2R:9232320-9232470:plus -22.2959 -1.27523 1.2844 2.95413 -26.1939 -17.7143 -11.6147 1.77778 10.1284
0.00335 0.0156 0.00748 0.00917 0.0175 0.00634 0.00424 0.00932 0.000129
2R:9326020-9326170:minus -14.5204 2.26606 9.46789 1.48624 -27.5306 -28.1939 -15.6055 4.56944 6.17431
0.00145 0.00455 0.000194 0.0177 0.0351 0.0591 0.0101 0.00317 0.00171
2R:9326020-9326170:plus -4.63265 -1.33028 7.63303 3.46789 -25.9694 -27.449 -9.15596 -3.79167 5.65138
0.000175 0.0159 0.000531 0.00726 0.017 0.0357 0.00263 0.0469 0.00228
2R:9326440-9326590:minus -24.1122 2.6055 10.0459 5.90826 -8.67347 -27.3367 -14.945 2.94444 3
0.00738 0.00402 0.000138 0.00271 0.00154 0.0347 0.00887 0.00609 0.00702
2R:9326440-9326590:plus -33.2245 -0.40367 -4.77982 5.49541 -18.3776 -16.3469 -30.5229 7.20833 -3.41284
0.0276 0.0118 0.0467 0.00355 0.00926 0.00416 0.0978 0.000902 0.0487
2R:9336240-9336390:minus -24.0306 -3.41284 -0.449541 2.11009 -27.1224 -27.7551 -6.54128 2.15278
4.56881 0.00723 0.0289 0.0134 0.0136 0.0245 0.0458 0.00151 0.00816 0.00356
2R:9336240-9336390:plus -23.602 0.724771 5.3578 6.6422 -18.4898 -27.5612 -16.7064 0.0277778
2.11927 0.00657 0.008 0.00153 0.00161 0.0116 0.0393 0.0122 0.0166 0.00889
2R:9336680-9336830:minus -21.7041 5.33028 0.733945 3.88991 -27.1633 -9.72449 -13.6972 0.361111 -1.22018
0.00231 0.00142 0.00906 0.00608 0.025 0.00352 0.00678 0.0149 0.0278
2R:9336680-9336830:plus -2.36735 0.807339 1.07339 0.146789 -26.7041 -28.1837 -8.20183 -0.0972222
5.18349 0.0000302 0.00776 0.00806 0.0258 0.0193 0.0584 0.00219 0.0172 0.00295
2R:9337460-9337610:minus -22.6633 -1.41284 7.38532 7.45872 -17.9286 -26.5612 -11.5138 -6.875 5.29358
0.00384 0.0163 0.0006 0.000894 0.0071 0.0237 0.00415 0.0904 0.00278
2R:9337460-9337610:plus -13.4082 -1.06422 7.88991 4.08257 0.877551 -28.4082 0.0275229 -6.02778
9.66055 0.000867 0.0146 0.000464 0.00558 0.00019 0.0615 0.000281 0.0765 0.000268
2R:9337960-9338110:minus -23.2551 0.293578 15.5229 5.57798 -19.0204 -18.051 -12.4954 -0.805556
1.90826 0.00583 0.00932 7.23e-07 0.0034 0.0158 0.00886 0.00515 0.0213 0.00974
2R:9337960-9338110:plus -23.1531 12.2661 0.0642202 3.62385 -18.2245 -18.7143 -11.4679 -2.31944

-1.56881 0.00542 0.0000384 0.0113 0.00673 0.00877 0.014 0.00411 0.0325 0.0298
2R:9369420-9369570:minus -4.66327 -4.09174 8.16514 3.26606 -18.5306 -9.65306 -8.88073 2.58333 3.9633
0.000177 0.0347 0.0004 0.00802 0.0118 0.0034 0.0025 0.00698 0.00446
2R:9369420-9369570:plus -12.9184 -1.22018 9.19266 0.504587 -8.93878 -8.42857 -7.86239 0.388889 0.348624
0.000532 0.0154 0.000226 0.0235 0.00216 0.00104 0.00204 0.0148 0.0167
2R:9370640-9370790:minus -32.1429 0.669725 3.16514 2.88991 20.4694 -18.1224 -11.6881 -1.01389 9.45872
0.0179 0.00815 0.00373 0.00942 1.65e-07 0.00988 0.00431 0.0227 0.000337
2R:9370640-9370790:plus -41.551 2.87156 2.84404 3.11009 -27.2653 -8.5 -24.3303 1.72222 1.70642
0.0607 0.00365 0.00421 0.00862 0.0302 0.00126 0.0431 0.0095 0.0108
2R:9372980-9373130:minus -14.6327 13.4592 12.8624 5.86239 -27.5714 -8.79592 -9 -0.0833333 -1.14679
0.0015 0.000064 0.0000233 0.00282 0.0357 0.0019 0.00256 0.0172 0.0272
2R:9372980-9373130:plus -14.4082 5.17431 -3.83486 4.22936 20.4694 -9.09184 -12.2385 4.54167 7.04587
0.00136 0.00151 0.0363 0.00525 1.65e-07 0.00219 0.00486 0.0032 0.00132
2R:9379900-9380050:minus -20.9184 0.266055 2.00917 1.90826 -18.1224 -9.23469 1.3945 0.0694444
0.917431 0.00181 0.0094 0.00576 0.015 0.00782 0.00238 0.000198 0.0164 0.0146
2R:9379900-9380050:plus -30.2551 3.97959 3.58716 5.77982 -7.60204 -19.0816 -3.41284 -1.01389 3.46789
0.00885 0.000669 0.00316 0.00294 0.000466 0.017 0.000634 0.0227 0.00582
2R:9381100-9381250:minus -21.6327 1.02752 7.3211 5.86239 -18.7857 -28.3367 -22.2844 -5.36111 4.88073
0.00215 0.00717 0.00062 0.00282 0.0148 0.0606 0.031 0.0667 0.00324
2R:9381100-9381250:plus -14.2143 3.10092 7.37615 10.5872 -26.9286 -8.57143 2.12844 -5.51389 -2.69725
0.00124 0.00335 0.000603 0.000115 0.0214 0.00149 0.000158 0.0688 0.0411
2R:9382420-9382570:minus -30.102 -1.79817 -4.77982 6.12844 20.4694 0.27551 -13.3394 0.416667 -0.963303
0.00859 0.0183 0.0467 0.00231 1.65e-07 0.000257 0.00625 0.0147 0.0258
2R:9382420-9382570:plus -40.4082 3.68807 9.18349 6.37615 -17.449 -17.9796 -15.0367 1.5 -0.284404
0.0399 0.0027 0.000228 0.00192 0.00484 0.00764 0.00904 0.0103 0.0207
2R:9384420-9384570:minus -42.3571 -2.10092 -0.614679 5.22936 -19.0102 -28.4082 -16.9541 -0.236111
6.69725 0.0748 0.02 0.0141 0.0041 0.0157 0.0615 0.0127 0.018 0.00145
2R:9384420-9384570:plus -30.398 -2.21101 3.22018 2.24771 -17.7857 -18.3469 -21.2844 2.55556 -2.50459
0.00901 0.0206 0.00365 0.0127 0.00676 0.0124 0.0259 0.00705 0.0388
2R:9389780-9389930:minus -13 -2.36697 2.24771 6.00917 -17.6429 10.0918 -14.9725 -8.29167 12.2752
0.000563 0.0216 0.00527 0.00255 0.00562 9.34e-06 0.00892 0.117 0.0000141
2R:9389780-9389930:plus -21.6633 4.49541 5.94495 2.6422 -8.37755 -9.5 -23.4771 -4.08333 3.42202
0.00223 0.00199 0.00118 0.0107 0.00106 0.00294 0.0379 0.0502 0.00597
2R:9390220-9390370:minus -24.3673 0.577982 -0.146789 5.22936 10.9898 -18.1224 -12.3211 4.02778
-0.0733945 0.00791 0.00843 0.0122 0.0041 3.28e-06 0.00988 0.00495 0.00397 0.0188
2R:9390220-9390370:plus -23.3367 -0.715596 3.6789 6.12844 10.6531 -9.45918 -20.6055 3.95833
1.53211 0.00614 0.0131 0.00305 0.00231 0.0000102 0.00279 0.0229 0.00409 0.0116
2R:9393840-9393990:minus -41.8571 -2.77982 -0.880734 1.86239 -27.5612 -18.051 -18.6697 2.25 -2.00917
0.0667 0.0243 0.0154 0.0153 0.0354 0.00886 0.0166 0.00788 0.0333
2R:9393840-9393990:plus -23 1.53211 -5.56881 2.34862 -7.33673 0.469388 -12.3303 6.38889 0.614679
0.00498 0.00596 0.057 0.0121 0.000388 0.000212 0.00496 0.00137 0.0158
2R:9394340-9394490:minus -22.9286 1.10092 -0.807339 5.98165 -18.1531 -18.4898 -18.0092 5.98611
6.26606 0.0046 0.00698 0.015 0.00258 0.00829 0.0129 0.015 0.00167 0.00162
2R:9394340-9394490:plus -31.9286 -3.77064 4.97248 2.11009 -9.16327 9.89796 -24.0642 3.63889 0.862385
0.0166 0.0318 0.0018 0.0136 0.00249 0.0000111 0.0414 0.00465 0.0148
2R:9394780-9394930:minus -23.0408 0.422018 -4.11927 3.18349 -18.9796 -0.244898 -18.8991 3.38889
5.53211 0.00512 0.0089 0.0392 0.00837 0.0155 0.000496 0.0172 0.00513 0.00245
2R:9394780-9394930:plus -30.7041 -0.862385 0.00917431 2.52294 -8.64286 -18.051 -7.66972 -3.77778
-2.11009 0.00993 0.0137 0.0116 0.0113 0.00139 0.00886 0.00196 0.0467 0.0343
2R:9397000-9397150:minus -32.2653 3.68367 0.137615 5.74312 -27.2653 -8.02041 -20.7523 3.80556 -0.651376
0.0184 0.00144 0.0111 0.00302 0.0302 0.000761 0.0235 0.00435 0.0235
2R:9397000-9397150:plus -33.8878 3.64286 10.8624 4.07339 -18.7143 -18.4184 4.42202 2.86111 0.697248
0.0302 0.00159 0.0000868 0.00562 0.0136 0.0127 0.0000708 0.00629 0.0154
2R:9427400-9427550:minus -24.449 -1 1.57798 2.86239 -26.8163 -16.9388 -17.9358 3.33333 -1.56881

0.00802 0.0143 0.00674 0.00954 0.0195 0.00433 0.0148 0.00525 0.0298
2R:9427400-9427550:plus -24.602 3.51376 -0.293578 -0.733945 -18.1224 -27.7449 -12.1101 -4.98611
5.44954 0.00822 0.00288 0.0128 0.0339 0.00782 0.0448 0.00472 0.0615 0.00253
2R:9434380-9434530:minus -30.1735 -0.211009 9.45872 1.62385 -8.40816 2.05102 -11.7615 0.305556
-2.51376 0.00869 0.0111 0.000195 0.0168 0.00108 0.0000468 0.00438 0.0152 0.039
2R:9434380-9434530:plus -32.7347 1.40367 8.90826 7.20183 19.9082 -7.65306 -19.9083 1.33333 -0.706422
0.0225 0.00625 0.000267 0.00112 8.13e-07 0.000647 0.0203 0.0109 0.024
2R:9439920-9440070:minus -22 5.14679 4.3211 5.46789 -17.7143 -8.45918 -11.4495 3.05556 3.88073
0.00291 0.00153 0.00235 0.00362 0.00627 0.00111 0.00409 0.00584 0.00475
2R:9439920-9440070:plus -22.7347 13.3878 4.41284 3.23853 -8.20408 -19.0102 -13.2661 0.847222 5.22936
0.00403 0.0000869 0.00227 0.00815 0.000894 0.0159 0.00614 0.0128 0.00288
2R:9441480-9441630:minus -31.4796 13.5306 7.6789 4.07339 -26.9286 -9.57143 -3.38532 -3.11111 1.16514
0.0128 0.0000206 0.000518 0.00562 0.0214 0.00326 0.000629 0.0397 0.0135
2R:9441480-9441630:plus -30.7755 13.3878 5.29358 3.77064 -8.44898 -27.3776 -14.9174 -2.59722 10.1284
0.0102 0.0000869 0.00157 0.00637 0.00109 0.0351 0.00882 0.0349 0.000129
2R:9445620-9445770:minus -22.9286 -1.54128 7.3578 6.45872 2.17347 0.316327 -20.789 -0.805556
3.58716 0.0046 0.0169 0.000608 0.00182 0.0000296 0.000243 0.0237 0.0213 0.00534
2R:9445620-9445770:plus -34.3673 0.376147 2.20183 2.04587 -19.0102 -9.79592 -21.1101 7.33333 -0.12844
0.0318 0.00905 0.00536 0.014 0.0157 0.00377 0.0251 0.000844 0.0193
2R:9466140-9466290:minus -31.6224 -1.3578 0.0275229 4.9633 -18.449 -17.8571 -15.5229 2.56944
5.18349 0.0137 0.016 0.0115 0.0044 0.0107 0.00719 0.00994 0.00701 0.00295
2R:9466140-9466290:plus -13.3878 -3.24771 7.90826 10.5872 -18.3878 -8.27551 -25.6789 -8.18056 0.733945
0.000831 0.0276 0.00046 0.000115 0.00931 0.000919 0.0514 0.115 0.0153
2R:9472680-9472830:minus -32.5918 2.33945 -4.00917 2.26606 -17.6429 -18.7143 -21.7706 13.0278 0.816514
0.021 0.00443 0.0381 0.0126 0.00562 0.014 0.0283 1e-05 0.015
2R:9472680-9472830:plus -31.898 4.93578 4.22936 2.9633 -19.051 -26.5612 -19.7798 4.31944 -0.908257
0.0164 0.00167 0.00244 0.00914 0.0162 0.0237 0.0198 0.00352 0.0255
2R:9473180-9473330:minus -14.7449 4.57798 2.65138 6.06422 -17.3061 -7.86735 -20.844 4.97222 -3
0.00157 0.00193 0.00453 0.00239 0.00389 0.00073 0.0239 0.00266 0.0447
2R:9473180-9473330:plus -22.9592 -2.0367 7.45872 3.02752 -28 -8.5 -18.9174 6.38889 -4.14679 0.00475
0.0196 0.000579 0.00887 0.0444 0.00126 0.0172 0.00137 0.0576
2R:9473780-9473930:minus -22.3776 3.22936 -0.550459 5.84404 -17.1531 -8.72449 -18.5413 -5.41667
-4.75229 0.00342 0.0032 0.0139 0.00289 0.00364 0.00169 0.0162 0.0674 0.0683
2R:9473780-9473930:plus -23.2245 9.6789 1.49541 4.14679 -18.9796 -9.7551 -23.9083 8.55556 3.62385
0.00576 0.000157 0.00694 0.00542 0.0155 0.00358 0.0405 0.000422 0.00523
2R:9474360-9474510:minus -33.3367 0.568807 2.48624 8.6055 -27.0816 -18.3469 -22.1651 -0.680556
7.73394 0.0281 0.00845 0.00483 0.000567 0.0243 0.0124 0.0304 0.0206 0.000784
2R:9474360-9474510:plus -32.2653 4.22936 -2.33028 5.59633 -26.1939 -9.09184 -10.633 5.83333 7.6422
0.0184 0.0022 0.0238 0.00331 0.0175 0.00219 0.00347 0.00179 0.000893
2R:9480460-9480610:minus -32.5918 8.99083 -0.486239 2.55046 -17.7551 -36.3367 -10.6239 10.4444
9.92661 0.021 0.000225 0.0136 0.0112 0.00658 0.0974 0.00347 0.000118 0.000193
2R:9480460-9480610:plus -30.5102 -1.10092 4.49541 3.31193 -18.051 -9.60204 -6.63303 -1.01389 -0.293578
0.00947 0.0148 0.00219 0.00786 0.00746 0.00334 0.00154 0.0227 0.0207
2R:9480760-9480910:minus -33 -0.495413 6.22936 9.49541 -17.4184 -7.72449 -8.68807 -1.11111 -5.23853
0.0258 0.0122 0.00104 0.000308 0.00457 0.00068 0.00241 0.0233 0.0766
2R:9480760-9480910:plus -31.9286 0.908257 -0.348624 2 -27.2653 -9.7551 -12.3119 2.44444 6.22018
0.0166 0.00748 0.013 0.0143 0.0302 0.00358 0.00494 0.00734 0.00165
2R:9709740-9709890:minus -41.5918 1 3.47706 -0.550459 -18.7857 -27.6735 -15.4954 2.95833 7.68807
0.0623 0.00724 0.0033 0.0317 0.0148 0.0443 0.00989 0.00606 0.000841
2R:9709740-9709890:plus -41.5102 0.256881 0.330275 2.19266 -9.23469 -7.72449 -21.5321 5.93056 -6.59633
0.0582 0.00944 0.0104 0.0131 0.00277 0.00068 0.0271 0.00171 0.103
2R:9710520-9710670:minus -22.9592 -5.42857 4.99083 3.6055 -27.6327 -26.7551 -16.945 -2.59722 10.3486
0.00475 0.00443 0.00178 0.00686 0.0369 0.0263 0.0127 0.0349 0.0000988
2R:9710520-9710670:plus -31.9286 4.05505 6.53211 6.19266 -17.6837 -10.0204 -20.3853 5.66667 -8.3578

0.0166 0.00235 0.000904 0.00216 0.0058 0.00399 0.022 0.00194 0.146
2R:9711740-9711890:minus -21.5102 -4.77064 -0.669725 -0.0458716 -17.8571 -15.9796 -13.9817 -3.16667
9.66055 0.00202 0.0415 0.0144 0.0271 0.00698 0.00405 0.00722 0.0403 0.000268
2R:9711740-9711890:plus -21.2245 6.00917 -2.82569 0.284404 0.846939 -28.2653 -9.26606 -6.45833 -2.16514
0.0019 0.00105 0.0275 0.0249 0.000197 0.0595 0.00269 0.0834 0.0348
2R:9741820-9741970:minus -21.6633 -2.92661 4.46789 2.78899 2.14286 -19.0816 -13.1468 -2.76389 7.53211
0.00223 0.0253 0.00222 0.00992 0.0000316 0.017 0.00598 0.0364 0.000968
2R:9741820-9741970:plus -23 1.55046 11.4404 2.6789 -17.4898 -17.3469 -19.5872 1.40278 -2.19266
0.00498 0.00592 0.000061 0.0105 0.0053 0.0058 0.0192 0.0106 0.0352
2R:9746160-9746310:minus -31.398 -1.26606 4.77064 5.73394 20.2041 -26.5306 -4.24771 4.59722 1.27523
0.0117 0.0156 0.00196 0.00307 3.64e-07 0.023 0.0008 0.00313 0.013
2R:9746160-9746310:plus -22.7347 4.82569 0.899083 2.66972 -19.0102 -17.9388 -15.0459 5.04167 -0.761468
0.00403 0.00175 0.00856 0.0106 0.0157 0.00738 0.00906 0.00258 0.0244
2R:9749460-9749610:minus -33.4898 3.16514 -0.522936 2.33945 10.3571 -9.94898 -26.6514 0.166667
-1.21101 0.0286 0.00327 0.0137 0.0122 0.0000208 0.00393 0.0579 0.0159 0.0277
2R:9749460-9749610:plus -32.3673 13.4592 3.87156 3.01835 -8.30612 9.82653 -3.74312 1.33333 2
0.0189 0.000064 0.00282 0.0089 0.00097 0.0000148 0.000694 0.0109 0.00941
2R:978200-978350:minus -39.0714 -0.40367 -1.87156 1.33028 -17.7857 -26.5306 -20.0459 2.55556 1.33028
0.0326 0.0118 0.0208 0.0188 0.00676 0.023 0.0208 0.00705 0.0127
2R:978200-978350:plus -34.3367 -4.46789 0.862385 9.08257 -8.16327 0.0918367 -12.0734 -2.02778 -1.68807
0.0318 0.0383 0.00867 0.000444 0.000873 0.000323 0.00469 0.03 0.0308
2R:9785220-9785370:minus -31.4796 1.78899 7.22018 7.73394 -26.898 -27.4898 -6.37615 -1.55556 5.65138
0.0128 0.00542 0.000652 0.000669 0.0203 0.0363 0.00145 0.0264 0.00228
2R:9785220-9785370:plus -22.8878 4.69725 12 3.59633 -18.7143 -26.8265 -9.99083 3.01389 1.70642
0.00441 0.00184 0.0000432 0.00693 0.0136 0.0285 0.00307 0.00593 0.0108
2R:9812960-9813110:minus 4.80612 3.97959 2.48624 9.41284 -17.7143 -16.7857 -3.72477 -0.125 1.59633
0.0000206 0.000669 0.00483 0.000354 0.00627 0.00426 0.000691 0.0174 0.0113
2R:9812960-9813110:plus -20.7041 3.20183 3.17431 3.01835 -9.20408 -9.5 -0.155963 1.22222 9.61468
0.00175 0.00323 0.00371 0.0089 0.00262 0.00294 0.000294 0.0113 0.00029
2R:9829680-9829830:minus -22.2959 1.44037 8.78899 3.70642 -17.4898 -26.3367 -16.1835 2.29167 1.74312
0.00335 0.00617 0.000284 0.0066 0.0053 0.0221 0.0112 0.00776 0.0105
2R:9829680-9829830:plus -31.1837 0.376147 -3.92661 4.3211 10.4286 -0.173469 -18.6881 -1.54167
-5.12844 0.0109 0.00905 0.0372 0.00514 0.0000139 0.000456 0.0166 0.0263 0.0748
2R:9841200-9841350:minus -24.449 0.146789 -1.79817 2.09174 -18.1837 -18.0816 -10.1101 5.98611 5.48624
0.00802 0.0098 0.0204 0.0137 0.00835 0.00934 0.00314 0.00167 0.00251
2R:9841200-9841350:plus -23.2653 -1.3211 -4 3.18349 -8.26531 -9.02041 -25.8165 10.2083 -2.15596
0.00593 0.0159 0.038 0.00837 0.000934 0.00213 0.0523 0.000141 0.0348
2R:9841520-9841670:minus -31.5612 -4.6055 -1.6789 4.9633 -7.63265 -17.2755 -9.13761 1.91667 1.31193
0.0134 0.0398 0.0196 0.0044 0.000479 0.00544 0.00262 0.00888 0.0127
2R:9841520-9841670:plus -22.9286 8.14679 12.8624 7.45872 -18.0816 -9.23469 -21.5046 -2.45833 1.99083
0.0046 0.000352 0.0000233 0.000894 0.00759 0.00238 0.027 0.0337 0.00946
2R:9845000-9845150:minus -31.5102 -1.59633 6.16514 4.33945 0.540816 -26.898 -7.51376 2.76389
-0.0733945 0.013 0.0172 0.00107 0.00509 0.000321 0.0302 0.0019 0.00652 0.0188
2R:9845000-9845150:plus -21.4388 0.807339 -1.56881 3.86239 -18.0408 -17.4898 -24.5596 5.84722 2.98165
0.002 0.00776 0.019 0.00622 0.00735 0.0059 0.0445 0.00178 0.00707
2R:9845580-9845730:minus -34.3061 -1.7156 3.73394 6.12844 -17.1837 -10.0204 -16.789 4.93056 -1.33945
0.0317 0.0179 0.00299 0.00231 0.00366 0.00399 0.0124 0.00271 0.0285
2R:9845580-9845730:plus -13.2245 0.899083 0.0642202 -1.31193 -17.5612 -9.0102 -22.211 2.09722
11.3853 0.000738 0.00751 0.0113 0.0405 0.00544 0.00208 0.0306 0.00832 0.0000815
2R:9848060-9848210:minus -40.8878 8.33028 -1.47706 4.3211 -17.449 -0.244898 -13.844 1.13889
-1.80734 0.0439 0.000319 0.0185 0.00514 0.00484 0.000496 0.00701 0.0116 0.0316
2R:9848060-9848210:plus -22.9592 0.559633 5.23853 3.29358 -19.051 -18.3469 -21.1284 0.666667 7.94495
0.00475 0.00848 0.00161 0.00791 0.0162 0.0124 0.0252 0.0135 0.000667
2R:9848800-9848950:minus -23.5204 0.577982 -2.42202 1.61468 -18.3469 -8.65306 -15.6147 -0.222222

-7.87156 0.00645 0.00843 0.0244 0.0169 0.00894 0.00156 0.0101 0.0179 0.132
2R:9848800-9848950:plus -41.6939 2.57798 4.0367 9.24771 -18.7143 9.5 -0.697248 3.18056 -0.972477
0.0637 0.00406 0.00264 0.000409 0.0136 0.0000337 0.000332 0.00557 0.0259
2R:9850100-9850250:minus -31.6633 -4.09174 6.10092 4.61468 -7.85714 -7.38776 -11.7248 -3.94444 2.11927
0.0141 0.0347 0.0011 0.00475 0.000594 0.000563 0.00434 0.0486 0.00889
2R:9850100-9850250:plus -31.1837 -3.08257 3.52294 5.65138 -17.449 -27.3061 -5.65138 -1.13889 4.45872
0.0109 0.0264 0.00324 0.00322 0.00484 0.0336 0.00119 0.0235 0.00366
2R:9851300-9851450:minus -33.2143 2.17431 8.59633 4.17431 -17.3776 -9.16327 -17.0092 -1.41667 -3.97248
0.0274 0.00471 0.000315 0.00537 0.00419 0.00223 0.0128 0.0254 0.0553
2R:9851300-9851450:plus -14.8163 2.90826 3.94495 4.05505 -18.7857 -19.3469 1.05505 5.75 6.11927
0.00159 0.0036 0.00274 0.00566 0.0148 0.0194 0.000217 0.00187 0.00178
2R:9852020-9852170:minus -13.0408 -2.45872 5.37615 6.20183 -18.7245 -18.9388 -21.9358 1.36111 4.55963
0.000576 0.0222 0.00152 0.00214 0.0136 0.0154 0.0291 0.0108 0.00357
2R:9852020-9852170:plus -21.4286 -2.69725 2.9633 3.29358 -27.1939 0.826531 9.44954 -3.01389 5.18349
0.00198 0.0237 0.00403 0.00791 0.0267 0.000153 0.0000156 0.0388 0.00295
2R:9853220-9853370:minus -23.6735 0.577982 2.11927 3.10092 -8.93878 0.683673 -4.10092 -5.31944 0.0275229
0.00665 0.00843 0.00553 0.00865 0.00216 0.000184 0.000768 0.0661 0.0183
2R:9853220-9853370:plus -23.6327 5.44037 -3.29358 0.981651 -26.9694 -17.9796 -18.1743 5.375 5.23853
0.00661 0.00135 0.0313 0.0207 0.023 0.00764 0.0154 0.00222 0.00285
2R:9855720-9855870:minus -32.1837 2.09174 4.46789 1.63303 -36.4082 -16.2755 -15.6972 3.02778 7.58716
0.0181 0.00485 0.00222 0.0167 0.0765 0.00413 0.0103 0.0059 0.000954
2R:9855720-9855870:plus -21.6633 14.7523 8.91743 2.11927 -19.051 -17.0102 -17.6697 0.722222 -6.19266
0.00223 3.48e-06 0.000266 0.0135 0.0162 0.00453 0.0142 0.0133 0.0932
2R:9866020-9866170:minus -22.9592 -5.22018 5.9633 6.59633 -17.6735 -16.7857 -17.2294 -4.15278 -0.486239
0.00475 0.0467 0.00117 0.00164 0.00572 0.00426 0.0133 0.051 0.0219
2R:9866020-9866170:plus -22.9592 1.38532 0.12844 4.19266 -17.1122 -17.051 -9.22936 3.08333 9.77064
0.00475 0.00629 0.0111 0.00536 0.00341 0.00479 0.00267 0.00578 0.000228
2R:9867040-9867190:minus -12.8776 5.14679 1.91743 7.2844 -25.9694 -26.5612 10.422 10.5556 -6.81651
0.000507 0.00153 0.00596 0.00105 0.017 0.0237 0.0000111 0.000108 0.109
2R:9867040-9867190:plus -22.8061 -0.87156 -3.83486 3.7156 -18.449 -9.72449 -7.14679 -3.5 2.25688
0.00414 0.0138 0.0363 0.00655 0.0107 0.00352 0.00175 0.0437 0.00853
2R:9867600-9867750:minus -13.7041 7.62385 -2.92661 5.31193 -18.7143 -8.5 1.97248 1.52778 -0.807339
0.00104 0.000468 0.0283 0.00393 0.0136 0.00126 0.000166 0.0102 0.0247
2R:9867600-9867750:plus -23.4898 -0.669725 -1.00917 4.78899 19.9082 0.27551 -15.3761 4.90278
1.33945 0.00639 0.0129 0.016 0.00456 8.13e-07 0.000257 0.00966 0.00274 0.0125
2R:9870880-9871030:minus -13.1837 0.862385 -0.743119 4.75229 -7.93878 -17.2755 -17.9266 -1.11111
2.55963 0.000705 0.00761 0.0147 0.00461 0.000767 0.00544 0.0148 0.0233 0.00784
2R:9870880-9871030:plus -29.1429 1.84404 3.30275 5.57798 -27.4898 -10.0204 -17.0367 3.09722 -4.73394
0.00834 0.00532 0.00353 0.0034 0.0336 0.00399 0.0129 0.00575 0.0681
2R:9878100-9878250:minus -33.1429 1.84404 1.3578 11.1927 -8.64286 -27.602 -0.715596 2.34722
-0.211009 0.0272 0.00532 0.00728 0.0000254 0.00139 0.0416 0.000333 0.00761 0.02
2R:9878100-9878250:plus -32.4796 2.13761 1.33028 4.22018 -8.93878 -0.204082 -20.2661 1.375
5.24771 0.02 0.00477 0.00736 0.00531 0.00216 0.000463 0.0216 0.0107 0.00282
2R:9878580-9878730:minus -33.3367 0.477064 1.44037 3.08257 -17.6429 -9.02041 -27.6789 4.01389 -4.19266
0.0281 0.00873 0.00707 0.0087 0.00562 0.00213 0.0659 0.004 0.0583
2R:9878580-9878730:plus -2.33673 1.21101 1.27523 7.63303 -18.1224 -16.7143 -12.4404 3.5 7.36697
0.0000294 0.00671 0.0075 0.000745 0.00782 0.00419 0.00509 0.00491 0.00108
2R:9879300-9879450:minus -24.4082 0.779817 0.40367 1.26606 -17.6429 -9.65306 -21.7706 1.13889 9.92661
0.00797 0.00784 0.0101 0.0191 0.00562 0.0034 0.0283 0.0116 0.000193
2R:9879300-9879450:plus -14.7449 1.18349 3.06422 7.47706 -18.6837 -17.9388 -23.5413 2.56944 -2.69725
0.00157 0.00677 0.00387 0.00085 0.0126 0.00738 0.0383 0.00701 0.0411
2R:9883220-9883370:minus -4.2551 10.4587 -3.97248 3.18349 -17.7143 -18.051 -11.6422 1.22222 -4.70642
0.000131 0.000106 0.0377 0.00837 0.00627 0.00886 0.00427 0.0113 0.0676
2R:9883220-9883370:plus -32.8163 -4.37615 0.761468 0.788991 -18.4898 -19.2755 -23.8165 2.19444 -2.17431

0.024 0.0374 0.00897 0.0218 0.0116 0.0185 0.0399 0.00804 0.035
2R:9894580-9894730:minus -21.7755 1.19266 -2.15596 -0.477064 -28 -26.3367 3.22936 3.875 4.55963
0.00241 0.00675 0.0226 0.0309 0.0444 0.0221 0.000109 0.00423 0.00357
2R:9894580-9894730:plus -23.3776 -2.93578 1.98165 3.7156 -18.6837 -8.53061 -20.5963 2.01389 4.14679
0.00619 0.0253 0.00582 0.00655 0.0126 0.00136 0.0229 0.00858 0.00406
2R:9896640-9896790:minus -21.8061 0.944954 4.11009 0.486239 -26.3061 -18.051 -18.0642 -0.708333
2.2844 0.00241 0.00739 0.00256 0.0237 0.0181 0.00886 0.0151 0.0207 0.00842
2R:9896640-9896790:plus -21.9184 -3.48624 7.37615 9.75229 -17.6429 -18.7857 2.99083 -8.15278 -1.63303
0.00261 0.0294 0.000603 0.000226 0.00562 0.0146 0.000119 0.114 0.0304
2R:9918920-9919070:minus -12.4082 1.83486 6.24771 1.9633 -26.898 -7.57143 -16.2385 3.20833 2.20183
0.000414 0.00533 0.00103 0.0146 0.0203 0.000644 0.0113 0.00551 0.00869
2R:9918920-9919070:plus -21.4388 -0.568807 5.43119 4.30275 -18.1224 -17.7143 -14.6055 -4.08333
3.88073 0.002 0.0125 0.00148 0.00517 0.00782 0.00634 0.00827 0.0502 0.00475
2R:9971200-9971350:minus -21.8163 -3.52294 -1.01835 5.73394 -18.9796 -8.45918 -15.8716 8.41667 7.46789
0.00244 0.0297 0.0161 0.00307 0.0155 0.00111 0.0106 0.000458 0.00102
2R:9971200-9971350:plus -30.8163 -2.58716 1.15596 -1.40367 -18.5204 -7.65306 -15.5872 5.19444 7.31193
0.0103 0.023 0.00783 0.042 0.0117 0.000647 0.0101 0.00241 0.00112
2RHet:1259580-1259730:minus -31.8163 4.12844 2.19266 -0.0458716 -17.4898 -26.8265 -9.37615 4.90278
2.94495 0.016 0.00229 0.00538 0.0271 0.0053 0.0285 0.00274 0.00274 0.00714
2RHet:1259580-1259730:plus -31.7755 -2.42202 2.55963 9.76147 -17.4184 -9.53061 -15.6239 -0.833333
-0.669725 0.0157 0.0219 0.0047 0.000212 0.00457 0.0031 0.0101 0.0215 0.0236
2RHet:1335540-1335690:minus -23.1531 0.587156 -5.46789 7.47706 -26.2245 -17.2755 -21.3028 3.80556
-3.93578 0.00542 0.0084 0.0556 0.00085 0.0175 0.00544 0.026 0.00435 0.0547
2RHet:1335540-1335690:plus -23.5204 2.44954 -2 7.62385 -18.7551 -9.7551 -17.3303 -0.708333
1.62385 0.00645 0.00425 0.0216 0.000759 0.0144 0.00358 0.0135 0.0207 0.0112
2RHet:1336020-1336170:minus -34.6327 3.22936 -0.963303 1.84404 -27.1224 -9.23469 -16.2477 9.26389
-3.3945 0.032 0.0032 0.0158 0.0154 0.0245 0.00238 0.0113 0.00027 0.0484
2RHet:1336020-1336170:plus -33.4898 3.16514 3.62385 -1.73394 -26.2653 -10.0204 -22.2661 6.80556
-1.43119 0.0286 0.00327 0.00312 0.0466 0.018 0.00399 0.0309 0.00111 0.029
2RHet:1547200-1547350:minus -24.1837 3.51376 8.7156 2.83486 -7.67347 -18.8265 -15.7339 0.875
3.25688 0.00751 0.00288 0.000296 0.0097 0.00055 0.0148 0.0103 0.0127 0.00632
2RHet:1547200-1547350:plus -32.6633 5.10092 2.02752 1.40367 -26.5306 0.0510204 -28.8165 4.79167
-5.62385 0.0216 0.00156 0.00572 0.0184 0.0185 0.000349 0.0769 0.00288 0.0822
2RHet:1621520-1621670:minus -32.3061 0.440367 8 0.972477 -27.3061 -9.57143 -24.1651 3.875 -4.76147
0.0186 0.00885 0.000438 0.0207 0.0315 0.00326 0.042 0.00423 0.0686
2RHet:1621520-1621670:plus -32.1939 0.0917431 5.30275 6.3578 -27.3061 0.387755 -21.4312
-0.0972222 2.3211 0.0181 0.00999 0.00157 0.00195 0.0315 0.000217 0.0266 0.0172 0.00839
2RHet:1631800-1631950:minus -40.4796 13.7798 3.44954 6.51376 -17.7857 0.540816 -26.8807 4.27778
-4.63303 0.0407 0.0000109 0.00334 0.00175 0.00676 0.0002 0.0596 0.00358 0.0661
2RHet:1631800-1631950:plus -40.5918 -1.15596 2.07339 6.17431 -7.67347 -7.45918 -17.211 9.81944
-0.119266 0.0422 0.0151 0.00562 0.0022 0.00055 0.000582 0.0132 0.000186 0.0192
2RHet:1777360-1777510:minus -39.9592 3.57143 0.40367 -0.211009 -27.3061 -17.2755 -18.9725 -2.09722
-2.3578 0.0348 0.00175 0.0101 0.0286 0.0315 0.00544 0.0174 0.0306 0.0369
2RHet:1777360-1777510:plus -31.398 5.92661 2.75229 3.18349 -17.7143 -18.7857 4.73394 1.5 6.17431
0.0117 0.00109 0.00436 0.00837 0.00627 0.0146 0.0000633 0.0103 0.00171
2RHet:198040-198190:minus-33.0714 0.431193 9.08257 6.43119 -19.2755 -7.94898 -22.0092 2.44444 0.486239
0.0267 0.00887 0.000242 0.00187 0.0164 0.00074 0.0295 0.00734 0.0163
2RHet:198040-198190:plus -13.4898 -1.66972 6.44037 3.40367 0.540816 -8.79592 -23.7523 -0.569444
-2.42202 0.000946 0.0176 0.000945 0.00759 0.000321 0.0019 0.0396 0.0199 0.0378
2RHet:198880-199030:minus-33 4.90826 -0.605505 2.04587 -17.6837 -9.45918 -22.8073 0.708333 0.385321
0.0258 0.00169 0.0141 0.014 0.0058 0.00279 0.034 0.0134 0.0166
2RHet:198880-199030:plus -31.7347 -0.633028 0.00917431 1.53211 -27.9694 0.316327 -5.3211 -2
-0.899083 0.0149 0.0127 0.0116 0.0174 0.0437 0.000243 0.00109 0.0298 0.0254
2RHet:2775760-2775910:minus -4.70408 -1.02752 9.27523 9.7156 -17.7857 0.0204082 -21.7982 8

0.110092 0.000186 0.0145 0.000216 0.000242 0.00676 0.000377 0.0284 0.000583 0.018
2RHet:2775760-2775910:plus -33.9592 -1.2844 2.6055 2.9633 -17.7143 -0.204082 -10.3211 4.41667
-2.98165 0.0304 0.0157 0.00462 0.00914 0.00627 0.000463 0.00327 0.00338 0.0445
2RHet:2776160-2776310:minus -30.4388 4.52294 2.20183 7.00917 -17.6837 -18.9796 -10.6147 -0.972222
0.0183486 0.00923 0.00197 0.00536 0.00126 0.0058 0.0155 0.00346 0.0224 0.0183
2RHet:2776160-2776310:plus -12.8878 5.18349 -3.99083 2.52294 -18.449 -18.8571 -6.55046 2.44444
-0.100917 0.000523 0.00151 0.0379 0.0113 0.0107 0.0151 0.00151 0.00734 0.019
2RHet:2901860-2902010:minus -32.5102 1.72477 -5.0367 -1.58716 -18.4082 -17.5714 -10.7431 1.66667
-2.15596 0.0202 0.00555 0.0498 0.0445 0.00937 0.00616 0.00355 0.00969 0.0348
2RHet:2901860-2902010:plus -22.0714 0.302752 2.47706 6.00917 -27.0816 -0.469388 -11.8257 4.91667
5.81651 0.00299 0.00929 0.00484 0.00255 0.0243 0.000533 0.00444 0.00272 0.00204
2RHet:3178880-3179030:minus -31.7755 13.4592 3.66055 2.70642 -18.7857 9.08163 -22.6055 5.44444
-2.36697 0.0157 0.000064 0.00307 0.0103 0.0148 0.0000431 0.0328 0.00215 0.0371
2RHet:3178880-3179030:plus -32.0102 1.01835 4.95413 7 -27.9694 -10.0204 -15.6147 5.625 -3.57798
0.0172 0.00719 0.00181 0.00132 0.0437 0.00399 0.0101 0.00198 0.0506
2RHet:3179360-3179510:minus -24.1837 1.6422 1.21101 2.66972 -17.7449 -27.6327 -17.367 5.36111
10.0367 0.00751 0.00573 0.00768 0.0106 0.0063 0.043 0.0136 0.00223 0.000146
2RHet:3179360-3179510:plus -41.4388 0.486239 5.49541 1.84404 -8.97959 -8.57143 -22.4312 1.90278
-0.12844 0.0562 0.00871 0.00144 0.0154 0.0023 0.00149 0.0318 0.00892 0.0193
2RHet:3199260-3199410:minus -32.2143 6.9633 10.0367 0.944954 -26.9694 -18.898 -16.367 4.59722
-2.47706 0.0182 0.000662 0.000139 0.0209 0.023 0.0152 0.0116 0.00313 0.0385
2RHet:3199260-3199410:plus -14.1531 13.5306 2.16514 7.44037 -27.3061 -18.0102 -12.789 4.88889 3
0.00121 0.0000206 0.00544 0.000912 0.0315 0.00812 0.00551 0.00276 0.00702
2RHet:3199700-3199850:minus -14.449 3.52294 1.54128 2.78899 -18.4184 0.122449 -14.3119 3.68056
1.24771 0.0014 0.00287 0.00683 0.00992 0.0101 0.000312 0.00777 0.00457 0.0131
2RHet:3199700-3199850:plus -24.5204 -0.972477 2.55046 1.63303 -26.5918 -18.0816 -10.3853 2.27778
2.11927 0.00813 0.0142 0.00471 0.0167 0.0186 0.00934 0.00331 0.0078 0.00889
2RHet:337860-338010:minus-32.7755 -0.201835 -2.22936 1.53211 -26.3061 1.05102 -15.6697 7.43056
0.963303 0.0234 0.011 0.0231 0.0174 0.0181 0.000107 0.0102 0.000801 0.0145
2RHet:337860-338010:plus -40.5918 -3.89908 0.0825688 2.77064 -17.7143 -18.3061 -21.9174 2.36111
-0.908257 0.0422 0.0329 0.0113 0.01 0.00627 0.0114 0.0291 0.00757 0.0255
2RHet:391020-391170:minus-33.8878 1.37615 -1.07339 1.38532 -17.7857 -17.2755 -14.4587 0.75 8.11009
0.0302 0.00631 0.0163 0.0185 0.00676 0.00544 0.00802 0.0132 0.000597
2RHet:391020-391170:plus -13.6735 4.77064 -1.83486 9.08257 -26.898 1.38776 -28.4679 2.98611 -5.14679
0.00103 0.00179 0.0206 0.000444 0.0203 0.0000559 0.0733 0.006 0.0751
2RHet:391300-391450:minus-22.8571 2.89908 3.41284 2.19266 -27.1939 -26.602 -14.1743 3.66667 7.22018
0.00427 0.00361 0.00338 0.0131 0.0267 0.0248 0.00754 0.0046 0.00119
2RHet:391300-391450:plus -30.7755 -1.56881 14.3578 3.90826 -9.16327 -27.5306 -8.65138 3.65278 0.697248
0.0102 0.0171 4.55e-06 0.00603 0.00249 0.0374 0.00239 0.00462 0.0154
2RHet:471720-471870:minus-23.7143 -2.21101 -1.02752 2.3578 -8.67347 -8.72449 -17.7615 1.36111 3.46789
0.0067 0.0206 0.0161 0.0121 0.00154 0.00169 0.0144 0.0108 0.00582
2RHet:471720-471870:plus -33.5204 3.57798 -0.522936 6.12844 -18.1939 0.72449 -17.7431 3.375
1.23853 0.0287 0.00281 0.0137 0.00231 0.0086 0.000175 0.0144 0.00516 0.0132
3L:1005940-1006090:minus -30.4796 0.669725 -2.6422 7.27523 -17.602 -9.02041 -13.7706 3.26389 1.46789
0.00941 0.00815 0.0261 0.00109 0.00547 0.00213 0.00689 0.00539 0.0119
3L:1005940-1006090:plus -24.3673 -4.58716 4.58716 1.22936 -17.1122 -27.051 -17.8807 2.22222 1.69725
0.00791 0.0396 0.00211 0.0193 0.00341 0.0315 0.0147 0.00796 0.0108
3L:1006440-1006590:minus -30.5204 5.80734 2.97248 6.45872 -26.898 -18.5714 -13.1835 0.861111 -0.605505
0.00951 0.00116 0.00401 0.00182 0.0203 0.0136 0.00603 0.0127 0.023
3L:1006440-1006590:plus -24.4796 -3.25688 3.76147 7.27523 -8.85714 -9.79592 -8.30275 1.13889 -0.486239
0.00804 0.0277 0.00295 0.00109 0.00165 0.00377 0.00224 0.0116 0.0219
3L:1006900-1007050:minus -31.898 3.16514 0.504587 3.6055 -18.2245 -8.65306 -4.07339 0.930556 5.57798
0.0164 0.00327 0.0098 0.00686 0.00877 0.00156 0.000762 0.0124 0.00238
3L:1006900-1007050:plus -21.2551 3.68367 1.7156 3.88991 -27.2347 -18.5714 -9.77064 3.11111 6.16514

0.00191 0.00144 0.00642 0.00608 0.0283 0.0136 0.00295 0.00572 0.00172
3L:1007740-1007890:minus -21.5918 3.40367 1.70642 9.49541 -17.1531 -26.5612 -23.6972 -2.29167 7.84404
0.00209 0.003 0.00644 0.000308 0.00364 0.0237 0.0392 0.0322 0.000701
3L:1007740-1007890:plus -31.6633 4.53211 -4.22018 2.77064 -17.449 -27.4898 -16.9541 -1.98611 3.77064
0.0141 0.00196 0.0403 0.01 0.00484 0.0363 0.0127 0.0297 0.00491
3L:1008100-1008250:minus -32.7755 -1.17431 11.0092 7.45872 -17.6429 -9.42857 -20.6239 -3.75 7.15596
0.0234 0.0151 0.0000797 0.000894 0.00562 0.0027 0.023 0.0464 0.00124
3L:1008100-1008250:plus -24.4592 0.0550459 1.6422 2.81651 10.398 -7.72449 -28.5963 1.44444
-6.77064 0.00802 0.0101 0.00659 0.00982 0.0000153 0.00068 0.0746 0.0105 0.107
3L:10216740-10216890:minus -31.1429 1.6789 10.055 10.5963 -18.3469 -17.4898 -7.79817 -3.05556
-0.422018 0.0108 0.00565 0.000138 0.0001 0.00894 0.0059 0.00202 0.0392 0.0213
3L:10216740-10216890:plus -31.3673 2.22936 6.72477 4.49541 -26.6327 -9.72449 -23.5688 2.93056 -5.98165
0.0116 0.00461 0.000825 0.00487 0.0188 0.00352 0.0384 0.00612 0.0885
3L:10218480-10218630:minus -22.9694 -1.31193 5.75229 9.51376 1.87755 9.82653 -11.8624 3.33333
-5.89908 0.0048 0.0158 0.00129 0.000292 0.0000492 0.0000148 0.00447 0.00525 0.0869
3L:10218480-10218630:plus -32.2653 1.6789 -0.0642202 9.08257 -28.3061 0.459184 -20.2202 7.54167
1.27523 0.0184 0.00565 0.0118 0.000444 0.0553 0.000213 0.0214 0.000754 0.013
3L:1034340-1034490:minus -23.1531 -3.79817 -2.74312 2.0367 -17.3367 -16.7143 -12.9725 3.51389 1.97248
0.00542 0.032 0.0268 0.0141 0.00391 0.00419 0.00574 0.00489 0.00948
3L:1034340-1034490:plus -20.5102 -2.19266 -1.86239 5.90826 -17.6735 -8.45918 -11.7156 3.54167 3.23853
0.00168 0.0205 0.0208 0.00271 0.00572 0.00111 0.00433 0.00483 0.00639
3L:10347840-10347990:minus -14.5612 8.61468 5.05505 5.58716 0.877551 -27.3367 -13.4404 0.0277778
9 0.00147 0.000275 0.00174 0.00334 0.00019 0.0347 0.00639 0.0166 0.000437
3L:10347840-10347990:plus -33.0714 0.192661 8.43119 10.5872 1.14286 1.31633 -16.2569 4.26389 5.66055
0.0267 0.00965 0.000347 0.000115 0.000113 0.000066 0.0113 0.0036 0.00227
3L:1034820-1034970:minus -30.551 0.348624 5.55963 9.59633 11.4286 -17.7857 -14.367 2.41667 11.3853
0.00959 0.00914 0.0014 0.000275 1.97e-06 0.0068 0.00786 0.00742 0.0000815
3L:1034820-1034970:plus -13.3673 11.3486 0.788991 3.77064 -18.7143 -27.602 -16.4037 4.625 -2.11927
0.000813 0.0000677 0.00889 0.00637 0.0136 0.0416 0.0116 0.00309 0.0344
3L:10357000-10357150:minus -23.0714 0 2.55963 2.90826 -17.449 -18.9796 -18.3028 1.90278 -4.81651
0.0052 0.0103 0.0047 0.00936 0.00484 0.0155 0.0157 0.00892 0.0697
3L:10357000-10357150:plus -21.8163 3.30275 5.23853 10.8624 -26.9286 -9.45918 1.20183 -4.40278 1.44037
0.00244 0.00311 0.00161 0.0000687 0.0214 0.00279 0.000209 0.054 0.012
3L:10359620-10359770:minus -23.1122 9.0367 2.2844 10.1468 -8.93878 -8.45918 -23.3028 5.29167
-2.88991 0.0053 0.000219 0.0052 0.000128 0.00216 0.00111 0.0369 0.0023 0.0433
3L:10359620-10359770:plus -33.3367 -2.04587 -1.11009 -0.110092 -18.2245 -19.2755 -8.22018 5.34722
12.2752 0.0281 0.0197 0.0165 0.0277 0.00877 0.0185 0.0022 0.00225 0.0000141
3L:10365480-10365630:minus -24.5612 1.69725 1.00917 3.50459 -27.1531 -9.02041 -15.7798 9.66667
5.33028 0.00819 0.00561 0.00824 0.00713 0.0249 0.00213 0.0104 0.000207 0.00273
3L:10365480-10365630:plus -24.0408 6.40367 -2.85321 5.94495 -17.7143 -27.898 -18.0367 1.34722 1.88073
0.00726 0.000874 0.0277 0.00261 0.00627 0.0537 0.015 0.0108 0.00987
3L:10377960-10378110:minus -29.6531 -6.09174 -3.90826 3.17431 -18.4184 1.05102 -9.13761 0.972222
1.52294 0.00846 0.0585 0.037 0.00842 0.0101 0.000107 0.00262 0.0123 0.0117
3L:10377960-10378110:plus -21.9286 -1.93578 1.14679 9.51376 -7.67347 -8.79592 -12.7706 -5.65278 0.59633
0.0027 0.0191 0.00785 0.000292 0.00055 0.0019 0.00549 0.0709 0.016
3L:104240-104390:minus -22.6633 4.49541 -0.972477 2.69725 -18.0918 -26.8265 -17.1009 -1.47222
-0.697248 0.00384 0.00199 0.0158 0.0104 0.00761 0.0285 0.013 0.0258 0.0239
3L:104240-104390:plus -23.1939 2.83486 1.30275 11.2936 -17.7143 -18.6429 -3.79817 -0.0694444 -1.57798
0.00558 0.0037 0.00743 0.0000139 0.00627 0.0139 0.000705 0.0171 0.0299
3L:10509000-10509150:minus -33.0714 -0.211009 0.0917431 1.10092 -18.7143 -10.0204 -25.4495
5.16667 2.42202 0.0267 0.0111 0.0112 0.02 0.0136 0.00399 0.05 0.00244 0.00816
3L:10509000-10509150:plus -22.8571 1 6.23853 2.83486 -17.9388 -19.4184 -12.5505 0.0972222 0.963303
0.00427 0.00724 0.00104 0.0097 0.00715 0.0198 0.00522 0.0162 0.0145
3L:10509540-10509690:minus -31.7755 -1.98165 -2.3945 0.0642202 -18.2245 -26.7551 -5.57798 0.444444

9.9633	0.0157	0.0193	0.0242	0.0264	0.00877	0.0263	0.00117	0.0145	0.000171
3L:10509540-10509690:plus -21.551	-4.55046 2.25688	5.49541	-27.4592 -17.9388 -3.42202 1.79167	1.48624
0.00205	0.0392	0.00525	0.00355	0.0329	0.00738	0.000635 0.00928	0.0118
3L:10515760-10515910:minus	-24.4082 2.88991	1.34862	0.862385 -18.4898 -27.1224 -14.055	5.54167
3.72477	0.00797	0.00362	0.00731	0.0214	0.0116	0.0325	0.00734	0.00205	0.00497
3L:10515760-10515910:plus -38.9184 4.3578	2.23853	3.77064	-8.60204 -8.27551 -28.4862 1.36111	1.47706
0.0323	0.0021	0.00529	0.00637	0.00125	0.000919 0.0735	0.0108	0.0119
3L:10624180-10624330:minus	-32.1122 -2.25688 1.11009	-0.899083	-8.64286 0.826531 -18.9174 7
3.20183	0.0178	0.0209	0.00796	0.0356	0.00139	0.000153 0.0172	0.00101	0.0065
3L:10624180-10624330:plus -24.1531 7.08257	0.761468 3.47706	-27.1939 1.31633	-22.4587 -1.51389 -5.65138
0.00744	0.000622 0.00897	0.00724	0.0267	0.000066 0.032	0.0261	0.0826
3L:10631080-10631230:minus	-21.6633 -3.97248 12.0367	4.34862	20.4694	-18.2755 -10.945	5.11111
1.95413	0.00223	0.0336	0.000042 0.00507	1.65e-07	0.0112	0.00369	0.0025	0.00964
3L:10631080-10631230:plus -30.7347 0.211009 -1.22936 3.44037	-18.4184 -27.5612 -19.8073 8.22222	7.31193
0.01 0.00959	0.0171	0.00746	0.0101	0.0393	0.0199	0.000514 0.00112
3L:10648820-10648970:minus	-30.7041 6.42202	0.688073 11.0183	-8.64286 -9.30612 -23.1835 6.79167
5.53211	0.00993	0.000867 0.0092	0.000052 0.00139	0.00257	0.0362	0.00112	0.00245
3L:10648820-10648970:plus -33	-0.220183	1.57798	6.02752	-17.4184 -16.7143 -30.7064 -3.73611 5.33945
0.0258	0.0111	0.00674	0.00246	0.00457	0.00419	0.1	0.0463	0.00271
3L:10649640-10649790:minus	-32.1939 2.40367	4.34862	11.1927	-17.4184 -18.0816 -26.5688 1.01389
-1.63303 0.0181	0.00433	0.00233	0.0000254	0.00457	0.00934	0.0573	0.0121	0.0304
3L:10649640-10649790:plus -32.7755 1.08257	-2.11927 1.04587	-17.449	-9.86735 -15.4679 3.18056	-2.52294
0.0234	0.00703	0.0224	0.0204	0.00484	0.00391	0.00983	0.00557	0.0391
3L:10651480-10651630:minus	-33.5102 -0.256881	3.83486	3.59633	-17.602	-18.3061 -22.9908 0.736111
5.00917	0.0286	0.0113	0.00287	0.00693	0.00547	0.0114	0.035	0.0132	0.0031
3L:10651480-10651630:plus -24.1122 3.68367	-1.58716 5.45872	-18.3776 -8.57143 -23.2202 3.11111	-1.50459
0.00738	0.00144	0.0191	0.00363	0.00926	0.00149	0.0364	0.00572	0.0294
3L:10652000-10652150:minus	-23.1837 -0.477064	-0.40367 2.23853	-26.9694 -8.79592 -4.55963 3.40278
9.45872	0.00553	0.0121	0.0132	0.0128	0.023	0.0019	0.000875 0.00511	0.000337
3L:10652000-10652150:plus -23.2653 2.41284	2.90826	6.00917	-17.3469 9.60204	-27.156	1.98611	-8.6055
0.00593	0.00431	0.00411	0.00255	0.00394	0.0000248	0.0617	0.00866	0.154
3L:10653640-10653790:minus	-23.1531 -1.75229 3.68807	0.541284 -18.9388 -9.53061 -21.4404 1.16667
-0.87156 0.00542	0.0181	0.00304	0.0233	0.0151	0.0031	0.0266	0.0115	0.0252
3L:10653640-10653790:plus -33.0408 -1.51376 3.07339	5.31193	-27.4184 -0.204082	-12.2661 2.47222
3.81651	0.0264	0.0168	0.00386	0.00393	0.0323	0.000463 0.00489	0.00727	0.00482
3L:10654040-10654190:minus	-14.2959 0.174312 8.99083	3.78899	10.9898	-8.42857 -0.577982	-2.11111
-2.46789 0.00129	0.00971	0.000256 0.00632	3.28e-06	0.00104	0.000323 0.0307	0.0384
3L:10654040-10654190:plus -31.8163 -0.275229	1.92661	-1.17431 -19.051	-28.3776 -3.15596 5.47222
2.82569	0.016	0.0113	0.00594	0.0388	0.0162	0.0609	0.000592 0.00212	0.0074
3L:10656340-10656490:minus	-43.1531 3.20183	2.04587	5.93578	-18.4592 0.602041 -37.3578 0.291667
-3.99083 0.0899	0.00323	0.00568	0.00264	0.0109	0.000188 0.203	0.0153	0.0556
3L:10656340-10656490:plus -23.3673 13.5306	14.3119	6.72477	-18.9796 -0.469388	-13.5413 0.777778
0.963303 0.00617	0.0000206	4.82e-06	0.00154	0.0155	0.000533 0.00654	0.0131	0.0145
3L:10657740-10657890:minus	-31.5102 -0.633028	-1.90826 1.68807	1.10204	-9.38776 -20.5413 -3.06944
-3.00917 0.013	0.0127	0.021	0.0164	0.000124 0.00266	0.0226	0.0393	0.0447
3L:10657740-10657890:plus -12.8878 8.05505	0.0458716	3.88991	-18.1531 -8.94898 -2.66055 -2.09722
2.16514	0.000523 0.000371 0.0114	0.00608	0.00829	0.00203	0.000521 0.0306	0.00879
3L:10658540-10658690:minus	-32.1429 -1.82569 -1.59633 2.68807	-18.1224 -9.79592 -25.0917 1.43056
2.82569	0.0179	0.0185	0.0192	0.0104	0.00782	0.00377	0.0477	0.0105	0.0074
3L:10658540-10658690:plus -32.6633 2.36697	-2.3578	4.02752	-17.0408 -8.7551	-17.3303 5.13889	7.31193
0.0216	0.00439	0.024	0.00581	0.00329	0.00172	0.0135	0.00247	0.00112
3L:10659100-10659250:minus	-31.5408 -0.981651	5.44037	2.06422	-28	-17.3469 -20.5963 0.930556
3.66972	0.0131	0.0143	0.00148	0.0139	0.0444	0.0058	0.0229	0.0124	0.00514
3L:10659100-10659250:plus -31.4082 -2.14679 10.3853	7.45872	-26.6735 -9.5	-14.8073 -2.68056 -1.93578

0.0119	0.0203	0.000113 0.000894 0.0191	0.00294	0.00863	0.0356	0.0326
3L:10671560-10671710:minus	-32.2959 2.13761	-0.00917431	3.44954	-17.3776 -10.0918 -8.10092 2.52778
1.16514	0.0186	0.00477	0.0116	0.00738	0.00419	0.00404	0.00215	0.00712	0.0135
3L:10671560-10671710:plus -33.3367 0.669725 -0.587156	-1.91743 -9.0102	-10.0204 -19.0642 6.94444
1.33945	0.0281	0.00815	0.014	0.0499	0.00236	0.00399	0.0176	0.00103	0.0125
3L:10672080-10672230:minus	-22.2245 -2.58716 -0.504587	2.70642	-26.1939 -26.898	-6.44954 4.91667
-2.11009 0.00325	0.023	0.0137	0.0103	0.0175	0.0302	0.00147	0.00272	0.0343
3L:10672080-10672230:plus -21.7041 -2.93578 -0.330275	7.77982	-18.4184 -8.93878 -10.8899 -1.15278
1.70642	0.00231	0.0253	0.0129	0.000652 0.0101	0.00201	0.00365	0.0236	0.0108
3L:10673020-10673170:minus	-24.2959 -1.0367	-0.577982	2.27523	-8.89796 -17.9796 -15.4495 4.90278
4.36697	0.00781	0.0145	0.014	0.0125	0.00189	0.00764	0.0098	0.00274	0.00379
3L:10673020-10673170:plus -33.1531 -2.22018 2.78899	9.59633	-8.93878 9.57143	-20.8716 3.83333	-2.08257
0.0273	0.0207	0.0043	0.000275 0.00216	0.0000274	0.024	0.0043	0.034
3L:10674120-10674270:minus	-32.4388 1.53211	-1.41284 2.09174	-8.37755 -17.3469 -20.6881 1.22222
6.89908	0.0195	0.00596	0.0181	0.0137	0.00106	0.0058	0.0232	0.0113	0.00137
3L:10674120-10674270:plus -23.0714 3.6055	3.6789	2.21101	-26.9694 -9.72449 -6.66055 1.31944	-0.174312
0.0052	0.00278	0.00305	0.0129	0.023	0.00352	0.00155	0.0109	0.0196
3L:10675080-10675230:minus	-21.551	-5.27523 -1.36697 -1.46789 -8.70408 -18.5612 -9.54128 -0.875
12.2752	0.00205	0.0474	0.0179	0.0428	0.00155	0.0132	0.00283	0.0218	0.0000141
3L:10675080-10675230:plus -13.0408 2.09174	6.44954	7	-8.60204 -17.0102 -13.1835 -0.805556	-1.47706
0.000576 0.00485	0.000941 0.00132	0.00125	0.00453	0.00603	0.0213	0.0292
3L:10675740-10675890:minus	-23.1531 3.08257	2.6422	5.21101	-7.93878 -8.42857 -26.2661 2.90278
1.33028	0.00542	0.00338	0.00455	0.0042	0.000767 0.00104	0.0553	0.00619	0.0127
3L:10675740-10675890:plus -22.6633 -1.45872 6.38532	-0.302752	-8.63265 -18.051	-17.2661 1.83333
-0.33945 0.00384	0.0165	0.00097	0.0293	0.0013	0.00886	0.0134	0.00914	0.0209
3L:10684040-10684190:minus	-41.551	4.12844	0.963303 5.74312	-18.6837 -10.0204 -23.2294 7.22222
-5.31193 0.0607	0.00229	0.00837	0.00302	0.0126	0.00399	0.0364	0.000895 0.0776
3L:10684040-10684190:plus -32	14.6055	4.11009	0.522936 -17.1837 -17.0102 -18.2477 1.01389	-2.88991
0.0171	4.26e-06 0.00256	0.0234	0.00366	0.00453	0.0155	0.0121	0.0433
3L:10687200-10687350:minus	-31.551	-1.19266 14.8991	2.54128	-18.7857 -8.79592 -11.7706 3.625
-2.6422	0.0134	0.0152	1.97e-06 0.0112	0.0148	0.0019	0.00439	0.00468	0.0405
3L:10687200-10687350:plus -13.7449 5.6422	1.76147	3.70642	-28.3061 -9.86735 -12.9817 10.6806	1.2844
0.00107	0.00124	0.00631	0.0066	0.0553	0.00391	0.00576	0.0000985	0.0129
3L:10706020-10706170:minus	-32.7449 -0.357798	-0.917431	-0.559633	-18.6837 -8.5 -20.5046 5.125
3.29358	0.0228	0.0116	0.0156	0.0318	0.0126	0.00126	0.0225	0.00248	0.00623
3L:10706020-10706170:plus -32.8163 -0.816514	-0.880734	-0.449541	-28.1939 1.82653	-22.5138
2.51389	2.58716	0.024	0.0135	0.0154	0.0307	0.0482	0.0000498	0.0323	0.00716	0.00779
3L:10729960-10730110:minus	-32.7755 -1.20183 1.92661	2.83486	-17.9388 -0.244898	-25.1651 1.58333
-2.18349 0.0234	0.0153	0.00594	0.0097	0.00715	0.000496 0.0482	0.00997	0.0351
3L:10729960-10730110:plus -22.2959 -0.825688	0.256881 7.90826	-17.3061 -18.1224 -13.9633 9.98611
2.82569	0.00335	0.0136	0.0106	0.000611 0.00389	0.00988	0.0072	0.000166 0.0074
3L:10730460-10730610:minus	-32.6531 1.62385	-0.110092	1.74312	-8.70408 -27.9286 -15.0642 1.83333
5.12844	0.0212	0.00577	0.012	0.016	0.00155	0.054	0.00909	0.00914	0.00301
3L:10730460-10730610:plus -30.4388 1.34862	2.0367	0.844037 -18.3469 0.122449 -28.055	-2.06944 -0.853211
0.00923	0.00637	0.0057	0.0215	0.00894	0.000312 0.0693	0.0304	0.0251
3L:10731540-10731690:minus	-23.9184 -0.330275	-0.0275229	5.59633	-27.1531 -0.469388	-14.2477
-0.652778	2.33028	0.00695	0.0115	0.0117	0.00331	0.0249	0.000533 0.00766	0.0204	0.00836
3L:10731540-10731690:plus -31.4388 -0.651376	-0.990826	2.99083	-18.1939 -17.9796 -14.2477 -0.236111
-4.54128 0.0122	0.0128	0.0159	0.00899	0.0086	0.00764	0.00766	0.018	0.0645
3L:10734340-10734490:minus	-23.0612 -2.3578	2.56881	2.70642	-18.0816 -19.2041 -1.76147 -3.20833
1.48624	0.00512	0.0215	0.00468	0.0103	0.00759	0.0179	0.000421 0.0407	0.0118
3L:10734340-10734490:plus -21.0816 -3.29358 -3.06422 1.58716	-17.6429 -8.42857 -20.0367 -4.65278 -8.09174
0.00187	0.028	0.0294	0.017	0.00562	0.00104	0.0207	0.0571	0.138
3L:10737180-10737330:minus	-31.5918 2.13761	2.69725	2.46789	-18.2245 -8.53061 -16.1927 -1.29167

7.51376 0.0136 0.00477 0.00446 0.0115 0.00877 0.00136 0.0112 0.0245 0.000984
3L:10737180-10737330:plus -22.7755 1.68807 6.0367 7.00917 -26.5306 -18.5714 -6.55046 -0.680556
3.40367 0.00412 0.00563 0.00113 0.00126 0.0185 0.0136 0.00151 0.0206 0.00603
3L:10764800-10764950:minus -12.1837 3.55046 0.238532 5.74312 -18.7143 0.0204082 -4.99083 3.45833
5.29358 0.000365 0.00284 0.0107 0.00302 0.0136 0.000377 0.00099 0.005 0.00278
3L:10764800-10764950:plus -23.0306 1.84404 1.24771 -0.422018 -27.0408 -17.7143 -13.3028 6.18056 3
0.00505 0.00532 0.00758 0.0305 0.0242 0.00634 0.0062 0.00152 0.00702
3L:10765460-10765610:minus -21.9592 5.9633 1.21101 7.15596 -28.4592 -16.2755 -7.33945 -3.05556
-4.88991 0.00279 0.00107 0.00768 0.00117 0.0568 0.00413 0.00182 0.0392 0.0711
3L:10765460-10765610:plus -31.7041 0.605505 0.633028 6.00917 -18.8265 -28.4796 -13.0183 3.75 12.2752
0.0146 0.00834 0.00938 0.00255 0.0149 0.0617 0.00581 0.00445 0.0000141
3L:10766840-10766990:minus -31.8163 0.53211 2.2844 1.43119 -18.4184 -27.5612 -18.7431 5.75 7.6789
0.016 0.00856 0.0052 0.0181 0.0101 0.0393 0.0168 0.00187 0.000866
3L:10766840-10766990:plus -32.7041 -1.68807 3.63303 4.66972 -7.67347 0.755102 -24.7523 -0.416667
-5.58716 0.0222 0.0177 0.00311 0.00466 0.00055 0.000175 0.0456 0.019 0.0817
3L:10769740-10769890:minus -23.9592 1.01835 1.72477 -1.66055 -8.67347 -35.449 -16.4679 0.361111
6.49541 0.00708 0.00719 0.0064 0.0456 0.00154 0.0789 0.0118 0.0149 0.00154
3L:10769740-10769890:plus -21.898 2.02752 3.22018 6.25688 -17.9286 -17.051 -22.2752 0.972222 -0.706422
0.00259 0.00497 0.00365 0.00208 0.0071 0.00479 0.031 0.0123 0.024
3L:10771000-10771150:minus -23.3776 -4.11927 1.89908 7.15596 -8.33673 -26.5612 -11.1743 1.93056
5.34862 0.00619 0.0349 0.006 0.00117 0.00101 0.0237 0.00387 0.00883 0.00271
3L:10771000-10771150:plus -22.398 -0.412844 7.3578 5.3945 -18.2653 -37.1837 5.92661 -2.55556
3.20183 0.00344 0.0119 0.000608 0.00379 0.00882 0.14 0.0000424 0.0345 0.0065
3L:10776300-10776450:minus -31.7041 -1.42202 1.94495 0.990826 -17.4898 -27.5612 -16.6239 3 2.84404
0.0146 0.0164 0.0059 0.0206 0.0053 0.0393 0.0121 0.00597 0.00736
3L:10776300-10776450:plus -40.6224 -0.715596 -1.00917 4.23853 -17.6735 -0.540816 -14.0917 6.41667
2.33028 0.0424 0.0131 0.016 0.00522 0.00572 0.00054 0.0074 0.00135 0.00836
3L:10776840-10776990:minus -33.0714 4.80734 8.18349 0.137615 -27.3061 -27.6735 -12.2385 -4.52778
0.93578 0.0267 0.00176 0.000397 0.0259 0.0315 0.0443 0.00486 0.0556 0.0145
3L:10776840-10776990:plus -32.8571 -1.79817 9.84404 4.78899 -9.23469 -26.8265 -21.1927 5.36111 5.24771
0.0244 0.0183 0.000156 0.00456 0.00277 0.0285 0.0255 0.00223 0.00282
3L:10777560-10777710:minus -31.3673 -4.52294 2.77064 3.70642 -17.4898 -9.72449 -15.5321 3.81944
5.84404 0.0116 0.0389 0.00433 0.0066 0.0053 0.00352 0.00995 0.00433 0.00202
3L:10777560-10777710:plus -4.33673 4.3211 -2.20183 9.7156 -18.7143 -9.53061 -7.77064 6.52778 2.11009
0.00014 0.00213 0.0229 0.000242 0.0136 0.0031 0.00201 0.00128 0.00898
3L:10778460-10778610:minus -33.2245 0.275229 1.53211 5.31193 -18.7143 -9.02041 -17.8349 -0.472222
0.486239 0.0276 0.00938 0.00685 0.00393 0.0136 0.00213 0.0146 0.0193 0.0163
3L:10778460-10778610:plus -32.1122 6.07339 3.68807 1.69725 -18.5204 -27.1224 -11.6972 0.972222 7.6422
0.0178 0.00102 0.00304 0.0163 0.0117 0.0325 0.00432 0.0123 0.000893
3L:10779480-10779630:minus -23.8469 5.15596 -0.550459 11.1927 -17.3367 -8.38776 -13.9541 0.583333
-7.17431 0.00685 0.00153 0.0139 0.0000254 0.00391 0.00101 0.00718 0.0139 0.117
3L:10779480-10779630:plus -12.8878 -2.41284 0.256881 6.44037 -9.0102 -17.5714 -17.7615 0.486111 -4.40367
0.000523 0.0219 0.0106 0.00185 0.00236 0.00616 0.0144 0.0144 0.0623
3L:10781760-10781910:minus -22.8878 0.53211 6.16514 6.43119 -17.3776 -18.5 -17.2844 -0.652778
7.11927 0.00441 0.00856 0.00107 0.00187 0.00419 0.0131 0.0134 0.0204 0.00125
3L:10781760-10781910:plus -33.5408 -2.88991 3.79817 0.93578 -26 -6.72449 -10.8532 -0.791667
-0.0733945 0.0287 0.025 0.00291 0.0209 0.0171 0.000544 0.00362 0.0212 0.0188
3L:10782620-10782770:minus -19.6633 2.66055 3.21101 6.90826 -27.5306 -8.86735 -6.90826 2.09722
3.08257 0.00164 0.00394 0.00366 0.00133 0.0351 0.00198 0.00165 0.00832 0.00681
3L:10782620-10782770:plus -31.2857 1.24771 -0.678899 4.27523 -27.6735 -7.5 -6.10092 3.15278 1.6422
0.0113 0.00661 0.0144 0.00518 0.0375 0.000614 0.00135 0.00563 0.0111
3L:10831180-10831330:minus -30.9286 0.706422 1.24771 9.7156 -16.7143 9.86735 -11.9725 0.125
-8.47706 0.0104 0.00805 0.00758 0.000242 0.00319 0.0000132 0.00458 0.0161 0.15
3L:10831180-10831330:plus -23.7347 9.54128 0.155963 0.00917431 -18.1939 -28.4082 -12.5229 2.06944

5.23853 0.00673 0.000168 0.011 0.0269 0.0086 0.0615 0.00518 0.00841 0.00285
3L:10831500-10831650:minus -13.0816 9.2844 -0.46789 6.08257 -27.9694 -9.0102 -18.5046 2.06944
1.85321 0.000599 0.000193 0.0135 0.00236 0.0437 0.00208 0.0161 0.00841 0.01
3L:10831500-10831650:plus -23.2245 1.88073 4.47706 -2.70642 -26.898 -28.5612 -12.0367 2.44444 5.23853
0.00576 0.00524 0.00221 0.0641 0.0203 0.0636 0.00465 0.00734 0.00285
3L:10835180-10835330:minus -22 -2.62385 8.38532 7.37615 -7.64286 -26.3061 -12.0734 5.5 8.62385
0.00291 0.0232 0.000356 0.000951 0.000499 0.0219 0.00469 0.00209 0.00048
3L:10835180-10835330:plus -32.551 0.376147 5.49541 7.20183 -25.8571 -8.79592 -1.2844 2.11111 5.3945
0.0207 0.00905 0.00144 0.00112 0.0166 0.0019 0.000378 0.00828 0.00263
3L:10842200-10842350:minus -31.7347 -1.46789 7.16514 1.91743 -18.3469 -7.5 -19.2018 -0.972222
-1.79817 0.0149 0.0166 0.000671 0.015 0.00894 0.000614 0.018 0.0224 0.0315
3L:10842200-10842350:plus -22.4184 0.724771 -1.3211 2.98165 -18.4592 -8.93878 -16.9633 -1.27778 -0.247706
0.00345 0.008 0.0176 0.00905 0.0109 0.00201 0.0127 0.0244 0.0204
3L:10845620-10845890:minus -13 1.13761 1.76147 3.18349 -17.449 -25.898 -6.33945 -1.19444 1.89908
0.000563 0.00689 0.00631 0.00837 0.00484 0.0214 0.00143 0.0239 0.0098
3L:10845620-10845890:plus -14.9286 4.92661 5.12844 7.00917 -17.8571 -27.6327 -13.2202 7.18056 3.72477
0.0016 0.00168 0.00169 0.00126 0.00698 0.043 0.00608 0.000915 0.00497
3L:10849820-10849970:minus -22.8571 -2.41284 1.77064 6.22936 0.806122 -8.79592 -14.6422 3.04167
11.4771 0.00427 0.0219 0.00629 0.00209 0.000229 0.0019 0.00834 0.00587 0.0000679
3L:10849820-10849970:plus -13.1837 7.95413 0.137615 1.3211 -8.82653 -8.79592 -11.9541 0.736111 0.422018
0.000705 0.000392 0.0111 0.0189 0.00163 0.0019 0.00456 0.0132 0.0164
3L:10850740-10850890:minus -21.1122 -1.58716 -4.36697 6.78899 -26.9286 0.27551 -18.2202 1.65278
2.89908 0.00187 0.0172 0.0419 0.00146 0.0214 0.000257 0.0155 0.00974 0.00722
3L:10850740-10850890:plus -20.6633 3.90816 -1.75229 9.7156 -18.1531 -8.45918 -15.3578 1.45833 -0.798165
0.00173 0.00102 0.0201 0.000242 0.00829 0.00111 0.00963 0.0104 0.0246
3L:10853260-10853410:minus -23.9286 4.16514 12.7431 7.2844 0.877551 -18.5612 -19.6881 5.30556
10.3394 0.00701 0.00226 0.0000253 0.00105 0.00019 0.0132 0.0195 0.00229 0.000112
3L:10853260-10853410:plus -4.19388 2.70642 7.06422 6.3578 -18.4184 -8.42857 -15.211 -3.5 4.92661
0.000129 0.00387 0.000706 0.00195 0.0101 0.00104 0.00936 0.0437 0.00318
3L:10858180-10858330:minus -12.5918 -2.15596 -1.12844 6.25688 -18.5306 -18.0102 -15.4587 5.36111
3.98165 0.000449 0.0203 0.0166 0.00208 0.0118 0.00812 0.00982 0.00223 0.00439
3L:10858180-10858330:plus -31.6633 -0.220183 0.614679 3.61468 -18.2245 -16.5714 -20.0367 3.19444
6.26606 0.0141 0.0111 0.00943 0.0068 0.00877 0.00417 0.0207 0.00554 0.00162
3L:10861240-10861390:minus -31.3265 -5.49541 -1.80734 0.165138 -18.4184 -27.6735 -7.50459 4.93056
11.4771 0.0114 0.0502 0.0204 0.0258 0.0101 0.0443 0.00189 0.00271 0.0000679
3L:10861240-10861390:plus -14.4592 -2.77982 -3 2.08257 -18.3469 0.826531 -28.7798 -1.20833 -5.22936
0.0014 0.0243 0.0288 0.0137 0.00894 0.000153 0.0765 0.0239 0.0764
3L:10872080-10872230:minus -23.1939 5.53211 5 5.90826 10.3878 -9.5 -16.7982 -6.625 -2.48624
0.00558 0.0013 0.00178 0.00271 0.0000174 0.00294 0.0124 0.0861 0.0387
3L:10872080-10872230:plus -23.2245 -0.376147 7.68807 5.3945 20.2041 -9.57143 -21.0275 -4.05556
1.11009 0.00576 0.0117 0.000516 0.00379 3.64e-07 0.00326 0.0247 0.0499 0.0138
3L:10879100-10879250:minus -31.398 -0.623853 0.642202 4.79817 -7.56122 -19.5714 -13.0642 2.33333
3.20183 0.0117 0.0127 0.00935 0.00453 0.000419 0.0202 0.00587 0.00765 0.0065
3L:10879100-10879250:plus -3.11224 -4.15596 14.1468 1.05505 -7.93878 -28.3367 -21.5046 5.54167 5.66055
0.0000458 0.0353 6.07e-06 0.0204 0.000767 0.0606 0.027 0.00205 0.00227
3L:10891060-10891210:minus -23.0714 5.12844 5.33945 0.697248 -18.6837 -28.8571 -9.98165 4.09722
5.58716 0.0052 0.00154 0.00154 0.0224 0.0126 0.0699 0.00307 0.00386 0.00237
3L:10891060-10891210:plus -30.7041 -2.7156 5.55046 3.91743 -17.0816 -17.0102 -10.6147 -4.06944 -0.972477
0.00993 0.0238 0.00141 0.00601 0.00334 0.00453 0.00346 0.05 0.0259
3L:10891420-10891570:minus -32.1429 -0.66055 -3.10092 0.0183486 -17.5204 -36.1837 -9.65138 3.875
5.12844 0.0179 0.0129 0.0297 0.0268 0.00532 0.0914 0.00289 0.00423 0.00301
3L:10891420-10891570:plus -33.9184 -0.40367 -0.458716 1.86239 -26.3061 -9.30612 -9.85321 3.80556
5.69725 0.0302 0.0118 0.0135 0.0153 0.0181 0.00257 0.00299 0.00435 0.00225
3L:10891740-10891890:minus -39.1837 -0.522936 -1.19266 5.69725 -27.898 -9.45918 -23.844 0.819444

-4.76147 0.0328 0.0123 0.0169 0.00314 0.04 0.00279 0.0401 0.0129 0.0686
3L:10891740-10891890:plus -21.9286 -0.0825688 -0.53211 3.22936 -26.9694 0.897959 -0.155963 -1.125
11.8073 0.0027 0.0106 0.0138 0.00816 0.023 0.000144 0.000294 0.0234 0.0000538
3L:10892180-10892330:minus -39.5918 4.9633 0.0642202 -0.357798 11.6939 -8.30612 -13.4679
8.93056 1.70642 0.0335 0.00165 0.0113 0.0297 1.17e-06 0.000973 0.00644 0.000335 0.0108
3L:10892180-10892330:plus -32.0714 3.49541 1.78899 1.76147 -27.602 -18.5 -24.9908 10.375 -2.68807
0.0176 0.0029 0.00625 0.0159 0.0364 0.0131 0.0471 0.000124 0.041
3L:109880-110030:minus -31.8163 0.256881 3.99083 0.899083 -17.4898 -27.1939 -21.6972 2.90278 4.0367
0.016 0.00944 0.00269 0.0212 0.0053 0.0327 0.0279 0.00619 0.00424
3L:109880-110030:plus -21.7347 -1.87156 6.30275 3.31193 -18.7143 -17.5714 -6.61468 0.722222 2.27523
0.00235 0.0187 0.00101 0.00786 0.0136 0.00616 0.00154 0.0133 0.00846
3L:11047060-11047210:minus -24.449 0.0458716 0.770642 1.22936 -8.89796 -9.45918 0.119266 3.31944
0.00917431 0.00802 0.0101 0.00895 0.0193 0.00189 0.00279 0.000275 0.00528 0.0184
3L:11047060-11047210:plus -14.398 -0.247706 3.59633 2.9633 -8.67347 -16.9796 -8.63303 4.23611
-0.825688 0.00134 0.0112 0.00315 0.00914 0.00154 0.0044 0.00239 0.00364 0.0249
3L:11055660-11055810:minus -23.1122 1.58716 12.4587 7.63303 -8.37755 -17.5612 0.293578 -1.43056
3.20183 0.0053 0.00584 0.000031 0.000745 0.00106 0.00604 0.000264 0.0255 0.0065
3L:11055660-11055810:plus -32.5816 6.46789 1.73394 4.11009 -26 -9.02041 -3.99083 6.47222 6.29358
0.0208 0.000847 0.00638 0.00553 0.0171 0.00213 0.000744 0.00132 0.00161
3L:11057840-11057990:minus -21.8571 2.90826 5.88073 5.57798 -18.6837 -8.16327 -19.2294 2.73611
-5.97248 0.00249 0.0036 0.00122 0.0034 0.0126 0.000783 0.0181 0.00659 0.0883
3L:11057840-11057990:plus -11.8469 -1.79817 15.4495 4.50459 -18.3776 -18.5714 -11.3028 -1.38889 1.2844
0.000279 0.0183 9.17e-07 0.00484 0.00926 0.0136 0.00397 0.0252 0.0129
3L:11062920-11063070:minus -21.8571 5.16514 -1.3578 9.87156 -17.449 -18.3469 -19.8716 7.93056
-5.33945 0.00249 0.00152 0.0178 0.000195 0.00484 0.0124 0.0201 0.000607 0.0781
3L:11062920-11063070:plus -31.7143 -0.568807 -1.04587 0.66055 -18.4184 -8.27551 -26.1101 1.31944
5.75229 0.0146 0.0125 0.0162 0.0225 0.0101 0.000919 0.0542 0.0109 0.00213
3L:11063580-11063730:minus -30.7755 -4.3211 9.3945 5.94495 -18.7857 -8.94898 -10.8991 2.79167
1.6422 0.0102 0.0369 0.000202 0.00261 0.0148 0.00203 0.00366 0.00646 0.0111
3L:11063580-11063730:plus -12.8163 0.348624 4.45872 7.47706 -7.82653 -9.79592 -4.80734 8.93056 11.8073
0.000489 0.00914 0.00223 0.00085 0.00059 0.00377 0.00094 0.000335 0.0000538
3L:11066400-11066550:minus -32 4.27523 1.0367 5.49541 -28.1939 -26.8265 -20.0367 1.66667 -2
0.0171 0.00217 0.00816 0.00355 0.0482 0.0285 0.0207 0.00969 0.0332
3L:11066400-11066550:plus -24.5306 2.85321 -2.18349 1.24771 -8.97959 -7.5 -16.5229 9.13889 3.80734
0.00814 0.00367 0.0228 0.0192 0.0023 0.000614 0.0119 0.000293 0.00484
3L:11067180-11067330:minus -22.9184 3.64286 11.2018 3.86239 -17.5612 -19.0816 -13.0092 10 4.18349
0.00447 0.00159 0.0000701 0.00622 0.00544 0.017 0.00579 0.000164 0.00404
3L:11067180-11067330:plus -23.9184 4.14679 -0.990826 1.84404 -19.0102 -18.9388 -7.19266 2.05556
5.44037 0.00695 0.00228 0.0159 0.0154 0.0157 0.0154 0.00176 0.00845 0.00259
3L:11067540-11067690:minus -12.0408 1.22936 -3.10092 6.78899 1.17347 -8.5 -21.6055 -1.45833 3.66972
0.000311 0.00666 0.0297 0.00146 0.000101 0.00126 0.0275 0.0257 0.00514
3L:11067540-11067690:plus -21.8163 -2.91743 2.55046 1.34862 -18.4184 -18.3469 -16.7064 2.11111
-0.0275229 0.00244 0.0252 0.00471 0.0187 0.0101 0.0124 0.0122 0.00828 0.0186
3L:11085400-11085550:minus -31.9592 -1.75229 -5.68807 2.19266 -17.4082 -9.16327 -17.7523 -2.06944
-8.50459 0.0167 0.0181 0.0587 0.0131 0.00421 0.00223 0.0144 0.0304 0.151
3L:11085400-11085550:plus -31.3673 6.88073 0.504587 11.0275 -17.9388 0.602041 -26.6055 3.33333 -3.81651
0.0116 0.000691 0.0098 0.0000401 0.00715 0.000188 0.0576 0.00525 0.0529
3L:11085960-11086110:minus -23.8163 -0.275229 6.91743 3.04587 -27.2347 -18.2347 -5.30275 3.76389
11.8073 0.00682 0.0113 0.000755 0.00883 0.0283 0.0104 0.00108 0.00442 0.0000538
3L:11085960-11086110:plus -30.398 -1.36697 5.25688 7.17431 -17.449 -18.2041 -16.3853 -1.06944 0.972477
0.00901 0.0161 0.0016 0.00115 0.00484 0.0103 0.0116 0.023 0.0144
3L:11089560-11089710:minus -29.5918 2.94495 14.9266 7.61468 -17.3061 0.0204082 -17.5596 0.513889
1.72477 0.00846 0.00355 1.9e-06 0.000779 0.00389 0.000377 0.014 0.0142 0.0106
3L:11089560-11089710:plus -22.8469 1.30275 -2.56881 3.7156 -8.93878 -28.7041 -11.7064 2.79167 9.87156

0.00424 0.00649 0.0255 0.00655 0.00216 0.067 0.00433 0.00646 0.000217
3L:11089960-11090110:minus -20.6735 1.97248 -2.11927 3.51376 -27.2347 -8.20408 -19.4404 7.15278
0.385321 0.00173 0.00507 0.0224 0.00709 0.0283 0.000796 0.0187 0.000928 0.0166
3L:11089960-11090110:plus -23.7857 3.49541 2.29358 5.19266 -18.4898 -9.79592 -9.46789 0.0833333
0.550459 0.00679 0.0029 0.00518 0.00422 0.0116 0.00377 0.00279 0.0163 0.0161
3L:11091520-11091670:minus -30.7347 2.45872 15.6239 2.09174 -8.93878 -18.2755 -15.7982 -0.0277778
6.89908 0.01 0.00424 5.44e-07 0.0137 0.00216 0.0112 0.0105 0.0169 0.00137
3L:11091520-11091670:plus -33.1122 -2.92661 0.807339 4.44037 -27.8265 -9.30612 -23.1009 3.01389 -4.41284
0.0271 0.0253 0.00884 0.00493 0.0388 0.00257 0.0357 0.00593 0.0624
3L:11106740-11106890:minus -33.2245 1.48624 5.23853 2.6055 10.6224 -17.0102 -22.6697 0.430556
-2.12844 0.0276 0.00606 0.00161 0.0109 0.0000113 0.00453 0.0332 0.0146 0.0345
3L:11106740-11106890:plus -41.7449 -0.816514 -0.963303 2.25688 10.398 -0.244898 -14.6514
-0.847222 0.348624 0.0646 0.0135 0.0158 0.0127 0.0000153 0.000496 0.00835 0.0216 0.0167
3L:11111760-11111910:minus -24.1837 0.183486 -1.98165 -1.77064 -27.0816 1.97959 -8.11009 3.02778
11.3853 0.00751 0.00968 0.0215 0.0473 0.0243 0.0000486 0.00215 0.0059 0.0000815
3L:11111760-11111910:plus -39.9898 -1.12844 -3.0367 7.95413 -18.5612 -25.8265 -19.844 4.125 1.27523
0.0351 0.0149 0.0291 0.000599 0.0119 0.0212 0.02 0.00382 0.013
3L:11113000-11113150:minus -14.602 7.77982 -1.20183 4.36697 -18.6429 -7.72449 -20.7156 -2.11111
-2.87156 0.00149 0.00043 0.017 0.00505 0.0121 0.00068 0.0234 0.0307 0.043
3L:11113000-11113150:plus -31.7347 -0.12844 0.0642202 5.77982 -27.2653 -17.9388 -21.8807 8.77778
-2.10092 0.0149 0.0108 0.0113 0.00294 0.0302 0.00738 0.0289 0.000368 0.0342
3L:11113560-11113710:minus -23.2143 -2.3578 1.59633 1.52294 -18.8265 -27.5306 -17.1468 5.08333
11.8532 0.00559 0.0215 0.00669 0.0175 0.0149 0.0374 0.0131 0.00253 0.0000428
3L:11113560-11113710:plus -4.96939 -1.15596 1.6789 9.44954 -8.93878 -18.2347 -1.18349 -3.5 4.83486
0.000218 0.0151 0.0065 0.000326 0.00216 0.0104 0.00037 0.0437 0.00329
3L:11116280-11116430:minus -13.3367 -0.376147 1.22936 -1.6055 20.2041 -19.3469 -18.6239 2.59722
1.2844 0.0008 0.0117 0.00763 0.0448 3.64e-07 0.0194 0.0165 0.00694 0.0129
3L:11116280-11116430:plus -14.2143 3.46789 -0.614679 0.926606 -18.3776 -17.8571 -6.97248 1.69444
-0.623853 0.00124 0.00293 0.0141 0.0209 0.00926 0.00719 0.00168 0.0096 0.0232
3L:11129060-11129210:minus -23.9592 -5.72477 2.3578 5.86239 -17.9388 -8.65306 -7.07339 -0.791667
-8.22018 0.00708 0.0532 0.00506 0.00282 0.00715 0.00156 0.00172 0.0212 0.142
3L:11129060-11129210:plus 16.0306 3.42202 8.66055 10.5963 -27.2347 -18.0816 5.65138 -0.277778
7.15596 1.16e-07 0.00298 0.000305 0.0001 0.0283 0.00934 0.0000462 0.0182 0.00124
3L:11157960-11158110:minus -23.4082 3.36697 0.954128 7.27523 -26.5306 1.97959 -26.9083
1.30556 -3.09174 0.00623 0.00304 0.0084 0.00109 0.0185 0.0000486 0.0598 0.011 0.0456
3L:11157960-11158110:plus -30.1429 -3.14679 4.77064 4.77064 -27.2653 10.3061 -2.52294
2.06944 -2.3211 0.00866 0.0268 0.00196 0.00457 0.0302 7.25e-06 0.000503 0.00841 0.0365
3L:11162700-11162850:minus -32.0408 2.56881 1.97248 5.92661 11.6531 1.09184 -21.211 3.52778 -2.66055
0.0174 0.00407 0.00584 0.00266 1.55e-06 0.000091 0.0256 0.00486 0.0406
3L:11162700-11162850:plus -31.0714 -0.247706 1.27523 7.34862 -18.7143 9.30612 -21.1743
0.472222 -5.27523 0.0106 0.0112 0.0075 0.000998 0.0136 0.0000398 0.0254 0.0144 0.0771
3L:11162960-11163110:minus -24.7857 -1.82569 -3.11927 5.22936 -19.051 -18.0102 -23.1651
2.90278 3 0.00829 0.0185 0.0298 0.0041 0.0162 0.00812 0.036 0.00619 0.00702
3L:11162960-11163110:plus -30.0306 0.100917 8.92661 3.69725 -17.9286 -18.051 -12.3211
0.722222 4.25688 0.00856 0.00996 0.000264 0.00665 0.0071 0.00886 0.00495 0.0133 0.00391
3L:11176040-11176190:minus -23.2653 4.68807 0.0733945 3.50459 -27.6429 -7.72449 -14.8257
4.83333 3.16514 0.00593 0.00184 0.0113 0.00713 0.037 0.00068 0.00866 0.00283 0.00656
3L:11176040-11176190:plus -23.3265 0.348624 5.85321 1.36697 -8.89796 -18.0102 -13.1927
3.48611 0.807339 0.00607 0.00914 0.00123 0.0186 0.00189 0.00812 0.00604 0.00494 0.015
3L:11177240-11177390:minus -24.102 -0.293578 -0.981651 6.82569 -18.7857 -9.37755 0.559633
2.86111 2.75229 0.00733 0.0114 0.0159 0.00144 0.0148 0.00265 0.000247 0.00629 0.00748
3L:11177240-11177390:plus -12.4082 6.19266 1.31193 6.09174 -9.20408 -8.16327 -18.4954
9.61111 2.75229 0.000414 0.000967 0.00741 0.00235 0.00262 0.000783 0.0161 0.000215 0.00748
3L:11177760-11177910:minus -41.2245 7.57798 3.01835 -1.29358 -27.2347 -9.23469 -28.3394

2.69444 -2.17431 0.0497 0.000479 0.00394 0.0403 0.0283 0.00238 0.072 0.00669 0.035
3L:11177760-11177910:plus -21.898 -0.137615 5.10092 5.43119 -18.1531 -18.2755 -16.4679
2.11111 3.42202 0.00259 0.0108 0.0017 0.00371 0.00829 0.0112 0.0118 0.00828 0.00597
3L:11178500-11178650:minus -24.3776 0.889908 4.87156 5.59633 -17.6429 -18.1224 -16.9725
-1.48611 6.80734 0.00793 0.00753 0.00188 0.00331 0.00562 0.00988 0.0128 0.0259 0.0014
3L:11178500-11178650:plus -21.7449 -0.504587 5.16514 1.63303 -25.6327 -17.1224 -11.8807
-0.458333 6.44954 0.00236 0.0122 0.00166 0.0167 0.0165 0.00499 0.00449 0.0192 0.00154
3L:11190980-11191130:minus -14.5204 -2.22018 6.91743 3.97248 -17.7143 -19.1939 -7.10092
-1.80556 2.11009 0.00145 0.0207 0.000755 0.00587 0.00627 0.0178 0.00173 0.0283 0.00898
3L:11190980-11191130:plus -30.9184 5.09174 13.8165 4.31193 -17.1939 -9.53061 -26.1009
-4.875 3.00917 0.0103 0.00157 9.45e-06 0.00516 0.00377 0.0031 0.0542 0.06 0.00697
3L:11199020-11199170:minus -22.6327 3.20183 4.55046 4.11927 -17.1837 -9.23469 -17.0642
-1.59722 -0.651376 0.00373 0.00323 0.00214 0.00551 0.00366 0.00238 0.0129 0.0267 0.0235
3L:11199020-11199170:plus -13.5204 3.84404 13.3394 7.2844 -27.1939 -9.64286 -10.2018
3.36111 5.33028 0.00097 0.00255 0.0000148 0.00105 0.0267 0.00339 0.0032 0.00519 0.00273
3L:11203740-11203890:minus -23.9184 11.422 0.844037 4.02752 -8.89796 -8.79592 -12.8257 4.09722
1.22936 0.00695 0.0000644 0.00872 0.00581 0.00189 0.0019 0.00555 0.00386 0.0132
3L:11203740-11203890:plus -22.9184 3.77064 13.2018 7.29358 -18.449 -8.23469 -11.3853 2.65278 -2.62385
0.00447 0.00262 0.0000167 0.00104 0.0107 0.000875 0.00404 0.0068 0.0402
3L:11212060-11212210:minus -33.8878 0.522936 2.56881 6.31193 -26.9694 -17.7857 -16.2385 -0.180556
-0.0733945 0.0302 0.00859 0.00468 0.00199 0.023 0.0068 0.0113 0.0177 0.0188
3L:11212060-11212210:plus -31.4388 -0.53211 3.34862 -0.284404 -28.5 -18.3469 -11.2752 3.76389
0.174312 0.0122 0.0123 0.00347 0.0291 0.0585 0.0124 0.00395 0.00442 0.0177
3L:11239380-11239530:minus -32.8571 -4.80734 1.73394 5.57798 -17.1531 -7.94898 -20.5505 1.66667
3.23853 0.0244 0.0419 0.00638 0.0034 0.00364 0.00074 0.0227 0.00969 0.00639
3L:11239380-11239530:plus -24.5612 -2.22018 -1.61468 2.20183 -17.1531 -26.898 -15.3211 2.76389 0.633028
0.00819 0.0207 0.0193 0.013 0.00364 0.0302 0.00956 0.00652 0.0158
3L:11241040-11241190:minus -22.6939 -4.3945 13.5505 7.45872 -26.5918 -18.9796 -18.6147 -0.125
1.08257 0.00388 0.0376 0.0000126 0.000894 0.0186 0.0155 0.0164 0.0174 0.0139
3L:11241040-11241190:plus -32.5204 -3.08257 1.2844 6.86239 -18.6735 -19.3061 -4.11009 4.65278 6.17431
0.0204 0.0264 0.00748 0.00136 0.0123 0.0187 0.00077 0.00305 0.00171
3L:11243420-11243570:minus -22.8061 4.81651 -0.633028 -1.05505 0.612245 -17.9796 -2.41284 -0.236111
3.62385 0.00414 0.00175 0.0142 0.0374 0.000269 0.00764 0.00049 0.018 0.00523
3L:11243420-11243570:plus -32.9694 -1.19266 5.49541 6.44037 -27.1939 -8.94898 -17.3119 2.15278 3.66972
0.0254 0.0152 0.00144 0.00185 0.0267 0.00203 0.0135 0.00816 0.00514
3L:11245340-11245490:minus -32.3265 -5.50459 -3.45872 -3.33028 -26.8571 -18.051 -15.4312 5.75 7.88991
0.0187 0.0503 0.0328 0.0736 0.0198 0.00886 0.00977 0.00187 0.000688
3L:11245340-11245490:plus -31.2857 3.0367 -1.88991 1.97248 -8.11224 -26.898 -9.01835 15.875 4.19266
0.0113 0.00343 0.0209 0.0146 0.000838 0.0302 0.00257 4.89e-08 0.00401
3L:11246020-11246170:minus -22.449 5.88991 -0.93578 6.09174 -17.3776 -8.16327 -7.22018 -5.47222
-0.798165 0.0035 0.00111 0.0156 0.00235 0.00419 0.000783 0.00178 0.0682 0.0246
3L:11246020-11246170:plus -32.4796 0.963303 -1.13761 1.49541 -17.7857 -25.7551 -16.2385 3.22222 10.0367
0.02 0.00734 0.0167 0.0176 0.00676 0.0208 0.0113 0.00548 0.000146
3L:11246480-11246630:minus -31.6224 -1.3945 -1.3945 0.944954 -18.5306 -38.5204 -7.72477 5.84722
7.68807 0.0137 0.0162 0.018 0.0209 0.0118 0.195 0.00199 0.00178 0.000841
3L:11246480-11246630:plus -30.1735 5.23853 7.47706 7.44037 -18.1122 -18.2347 -26.6055 -5.48611 -0.293578
0.00869 0.00148 0.000573 0.000912 0.00771 0.0104 0.0576 0.0684 0.0207
3L:11248000-11248150:minus -30.102 -2.08257 -0.899083 -0.981651 -16.7143 -9.79592 -2.91743
6.20833 4.41284 0.00859 0.0199 0.0155 0.0365 0.00319 0.00377 0.000556 0.0015 0.00368
3L:11248000-11248150:plus -32.4796 0.192661 0.0458716 -0.678899 -18.2245 -9.57143 -18.367 1.98611
0.0275229 0.02 0.00965 0.0114 0.0332 0.00877 0.00326 0.0158 0.00866 0.0183
3L:11269120-11269270:minus -21.8878 3.93578 5.55963 7 -18.051 -18.5612 -20.945 -1.66667 -1.58716
0.00258 0.00246 0.0014 0.00132 0.00746 0.0132 0.0243 0.0272 0.03
3L:11269120-11269270:plus -13.4184 1.24771 5.70642 2.56881 -17.5306 -17.5612 -16.2477 -1.41667 7.11927

0.000873 0.00661 0.00131 0.0111 0.0054 0.00604 0.0113 0.0254 0.00125
3L:11277360-11277510:minus -23.6939 3.25688 6.80734 5.31193 0.877551 -9.02041 -6.25688 1.16667
-1.50459 0.00667 0.00316 0.000794 0.00393 0.00019 0.00213 0.0014 0.0115 0.0294
3L:11277360-11277510:plus -22.2245 2.61468 7.30275 1.92661 -7.64286 -8.57143 -15.9908 0.430556 2.88073
0.00325 0.004 0.000626 0.0148 0.000499 0.00149 0.0108 0.0146 0.00727
3L:11278960-11279110:minus -22.5918 0.412844 2.29358 3.61468 -26.9694 -17.7857 -24.1101 0.805556
6.54128 0.00365 0.00893 0.00518 0.0068 0.023 0.0068 0.0417 0.013 0.00151
3L:11278960-11279110:plus -24.1837 1.58716 -1.33028 1.56881 -17.7143 -18.2755 -20.2018 3.52778 -0.963303
0.00751 0.00584 0.0177 0.0172 0.00627 0.0112 0.0213 0.00486 0.0258
3L:11283460-11283610:minus -31.6735 6.05505 2.07339 3.45872 -8.93878 -10.0204 -26.7339 -1.41667
-2.63303 0.0141 0.00103 0.00562 0.00731 0.00216 0.00399 0.0585 0.0254 0.0404
3L:11283460-11283610:plus -23.3776 4.29358 1.49541 1.02752 -7.93878 -9.53061 -21.1009 -2.625 -3.6422
0.00619 0.00215 0.00694 0.0204 0.000767 0.0031 0.025 0.0351 0.0511
3L:11284240-11284390:minus -32.6327 1.07339 0.807339 3.20183 -26.9286 -26.6735 -13.633 -1.47222
2.25688 0.0212 0.00705 0.00884 0.00824 0.0214 0.0256 0.00668 0.0258 0.00853
3L:11284240-11284390:plus -21.7347 3.31193 3.41284 7.15596 -18.5204 -18.4184 -10.3486 5.45833 3
0.00235 0.0031 0.00338 0.00117 0.0117 0.0127 0.00329 0.00214 0.00702
3L:11285360-11285510:minus -31.8469 -3.33945 6.6789 7.55963 -26.9694 -19.0102 -14.5963 0.513889
4.10092 0.0162 0.0283 0.000844 0.000807 0.023 0.0159 0.00826 0.0142 0.00413
3L:11285360-11285510:plus -32.2857 -2.66055 6.50459 6.72477 0.846939 -16.2755 -9.83486 -0.861111
3.30275 0.0185 0.0235 0.000917 0.00154 0.000197 0.00413 0.00298 0.0217 0.00619
3L:11286660-11286810:minus -21.3265 -3.72477 4.52294 0.00917431 -17.6837 -17.9796 -6.6055 0.625
7.15596 0.00193 0.0314 0.00217 0.0269 0.0058 0.00764 0.00153 0.0137 0.00124
3L:11286660-11286810:plus -21.6633 -3.05505 4.6422 2.11927 -17.4082 -37.3061 -7.74312 -5.76389 3.40367
0.00223 0.0262 0.00206 0.0135 0.00421 0.146 0.00199 0.0725 0.00603
3L:11288180-11288330:minus -31.1429 3.89908 7.27523 4.02752 -18.3776 -17.7551 -12.7798 -0.958333
-1.06422 0.0108 0.0025 0.000635 0.00581 0.00926 0.00643 0.0055 0.0223 0.0266
3L:11288180-11288330:plus -31.6327 2.33945 0.12844 2.45872 -18.7245 0.27551 -20.8624 10.0278 -8.90826
0.0138 0.00443 0.0111 0.0116 0.0136 0.000257 0.024 0.000161 0.163
3L:11288720-11288870:minus -33.6224 -2.3578 5.61468 3.44954 -18.5204 -37.1837 -11.0275 1.56944
10.3394 0.0289 0.0215 0.00137 0.00738 0.0117 0.14 0.00375 0.01 0.000112
3L:11288720-11288870:plus -12.5204 -4.89908 5.72477 3.48624 -18.3878 -9.02041 5.92661 4.68056 1.31193
0.000441 0.043 0.0013 0.0072 0.00931 0.00213 0.0000424 0.00302 0.0127
3L:11340020-11340170:minus -22.2245 0.880734 -2.3211 7.27523 -17.4898 -8.23469 -13.5138 3.30556
0.440367 0.00325 0.00756 0.0237 0.00109 0.0053 0.000875 0.0065 0.0053 0.0164
3L:11340020-11340170:plus -20.7041 3.64286 3.52294 1.14679 -17.449 -0.540816 -10.578 -1.09722
-0.12844 0.00175 0.00159 0.00324 0.0198 0.00484 0.00054 0.00343 0.0232 0.0193
3L:11342060-11342210:minus 5 5.21101 4.84404 6.00917 -17.1531 -18.2755 -1.25688 2.22222 7.42202
0.0000168 0.00149 0.0019 0.00255 0.00364 0.0112 0.000376 0.00796 0.00106
3L:11342060-11342210:plus -14.4082 5.91743 2.23853 9.24771 -17.7143 -19.3469 -7.89908 0.388889 10.3486
0.00136 0.0011 0.00529 0.000409 0.00627 0.0194 0.00206 0.0148 0.0000988
3L:11342740-11342890:minus -14.1531 0.0458716 0.0183486 0.733945 -8.93878 -28.7041 -15.3945
4.04167 2.20183 0.00121 0.0101 0.0115 0.0222 0.00216 0.067 0.0097 0.00395 0.00869
3L:11342740-11342890:plus -13.4082 0.165138 2.46789 2.69725 -27.3367 -27.8265 -1.57798 -3.06944 5.22018
0.000867 0.00974 0.00486 0.0104 0.0317 0.0498 0.000403 0.0393 0.00289
3L:11343420-11343570:minus -23.8163 0.917431 3.89908 2.6789 -27.5714 -17.8265 -10.6789 6.19444
0.816514 0.00682 0.00746 0.00279 0.0105 0.0357 0.00699 0.0035 0.00151 0.015
3L:11343420-11343570:plus -40.3265 -1.10092 9.86239 3.47706 -8.40816 -17.3469 -11.5872 8.5 -1.36697
0.0394 0.0148 0.000155 0.00724 0.00108 0.0058 0.00422 0.000436 0.0286
3L:11344140-11344290:minus -14.2143 -2.3578 7.97248 3.18349 -17.7143 -27.602 -14.156 -1.41667
-0.0183486 0.00124 0.0215 0.000444 0.00837 0.00627 0.0416 0.00751 0.0254 0.0185
3L:11344140-11344290:plus -34.2551 3.68367 1.62385 3.05505 -26.1531 -18.2755 -23.4495 6.36111 4.13761
0.0315 0.00144 0.00663 0.00878 0.0172 0.0112 0.0377 0.00139 0.00408
3L:11345760-11345910:minus -32.8571 1.94495 -1.46789 -0.972477 -17.1939 -8.7551 -17.5963 2.375

5.53211 0.0244 0.00512 0.0184 0.0364 0.00377 0.00172 0.0141 0.00753 0.00245
3L:11345760-11345910:plus -23.1939 3.71429 1.94495 3.18349 -18.4592 -18.1224 -4.97248 -0.791667
3.47706 0.00558 0.00129 0.0059 0.00837 0.0109 0.00988 0.000985 0.0212 0.00575
3L:11346020-11346170:minus -10.8469 -1.9633 0.40367 6.78899 -27.5714 -25.5306 -10.1835 2.01389
4.6789 0.000237 0.0192 0.0101 0.00146 0.0357 0.0205 0.00319 0.00858 0.00347
3L:11346020-11346170:plus -34.2245 -0.798165 -3.90826 5.69725 -17.3776 -18.0102 -11.0275 4.59722
5.38532 0.0315 0.0134 0.037 0.00314 0.00419 0.00812 0.00375 0.00313 0.00267
3L:11346440-11346590:minus -23.3061 -0.724771 0.137615 3.94495 -18.4184 -17.9796 -16.3853 1.66667
-3.77064 0.00606 0.0131 0.0111 0.00595 0.0101 0.00764 0.0116 0.00969 0.0524
3L:11346440-11346590:plus -15.0816 1.45872 -0.422018 1.98165 -27 -26.5306 -7.74312 4.08333 3.66972
0.00163 0.00613 0.0133 0.0145 0.0233 0.023 0.00199 0.00388 0.00514
3L:11347480-11347630:minus -30.102 2.22018 0.944954 8.81651 -17.8571 -18.7143 -4.38532 3.55556
5.22936 0.00859 0.00463 0.00842 0.000501 0.00698 0.014 0.000832 0.00481 0.00288
3L:11347480-11347630:plus -31.4082 4.48624 -0.256881 -1.70642 -18.1837 -18.1224 -23.4771 8.69444
0.201835 0.0119 0.00199 0.0126 0.0462 0.00835 0.00988 0.0379 0.000387 0.0175
3L:11358240-11358390:minus -22.6224 1.17431 6.42202 2.57798 -18.0816 -8.72449 -15.055 -1.15278
-1.26606 0.00371 0.00679 0.000953 0.011 0.00759 0.00169 0.00907 0.0236 0.0281
3L:11358240-11358390:plus -41.3265 -3.88991 2.12844 2.09174 -27 -46.4796 -10.367 2.13889 7.31193
0.0541 0.0328 0.00551 0.0137 0.0233 0.312 0.0033 0.0082 0.00112
3L:11399880-11400030:minus -22.7755 1.92661 8.85321 7.20183 -18.8265 -18.8571 -4.3211 2.38889
7.83486 0.00412 0.00516 0.000275 0.00112 0.0149 0.0151 0.000817 0.00749 0.000712
3L:11399880-11400030:plus -14.3061 -1.29358 12.5596 7.95413 -17.8571 -7.93878 -15.4404 6.94444 -0.394495
0.00129 0.0157 0.000029 0.000599 0.00698 0.000736 0.00978 0.00103 0.0212
3L:11425120-11425270:minus -32.1531 4.80734 3.78899 3.33945 -18.1531 -8.5 -17.1743 5.43056 -5.40367
0.0179 0.00176 0.00292 0.00774 0.00829 0.00126 0.0132 0.00216 0.0791
3L:11425120-11425270:plus -22.8469 3.41284 1.74312 5.38532 -17.3776 -18.1224 -21.3578 -3.56944 -0.798165
0.00424 0.00299 0.00635 0.00382 0.00419 0.00988 0.0262 0.0445 0.0246
3L:11480360-11480510:minus -33.2653 6.41284 4.38532 6.57798 -27.4184 -17.5 -18.156 -1.125
-4.84404 0.0277 0.00087 0.0023 0.00167 0.0323 0.00599 0.0153 0.0234 0.0701
3L:11480360-11480510:plus -22.4694 -1.00917 6.01835 6.31193 -18.7551 -8.57143 1.93578 13.6111 1.27523
0.0035 0.0144 0.00114 0.00199 0.0144 0.00149 0.000168 4.71e-06 0.013
3L:11480860-11481010:minus -31.1735 6.27523 5.11009 5.94495 -7.37755 -26.898 -2.91743 1.81944
7.15596 0.0109 0.00093 0.0017 0.00261 0.000398 0.0302 0.000556 0.00919 0.00124
3L:11480860-11481010:plus -32.5102 0.0550459 1.89908 7.80734 -17.7449 -18.3469 -13.156 6.15278
3.15596 0.0202 0.0101 0.006 0.00063 0.0063 0.0124 0.00599 0.00154 0.00659
3L:11485660-11485810:minus -30.551 2.05505 7.6422 -2.84404 -7.93878 -17.0102 -13.7064 6.76389
1.88073 0.00959 0.00492 0.000528 0.0664 0.000767 0.00453 0.00679 0.00114 0.00987
3L:11485660-11485810:plus -23.102 2.83486 -0.376147 1.63303 -18.7551 -18.051 -13.7982 -0.513889
7.63303 0.00522 0.0037 0.0131 0.0167 0.0144 0.00886 0.00693 0.0196 0.000928
3L:11486680-11486830:minus -13.4796 -0.110092 8.55963 9.61468 -8.67347 -7.94898 -12.5229 -3.08333
-4.24771 0.000935 0.0107 0.000322 0.000255 0.00154 0.00074 0.00518 0.0395 0.0591
3L:11486680-11486830:plus -31.9286 2.84404 14.1927 7.29358 -18.7449 -9.42857 -13.7339 -5.08333 -4.31193
0.0166 0.00368 5.79e-06 0.00104 0.0138 0.0027 0.00684 0.0628 0.0604
3L:11513900-11514050:minus -4.47959 -2.44954 7.2844 10.7615 -8.67347 -18.0816 2.3578 -3.41667
7.84404 0.000154 0.0221 0.000632 0.0000826 0.00154 0.00934 0.000147 0.0428 0.000701
3L:11513900-11514050:plus -21.7347 -3.13761 6.57798 3.87156 -26.5918 -17.3469 0.330275 -4.22222 5.00917
0.00235 0.0268 0.000884 0.00618 0.0186 0.0058 0.000262 0.0518 0.0031
3L:11527020-11527170:minus -13.4082 5.41284 -0.0366972 2.15596 -17.449 -19.3061 -17.4404 2.76389
2.73394 0.000867 0.00137 0.0117 0.0133 0.00484 0.0187 0.0137 0.00652 0.00756
3L:11527020-11527170:plus -14.5204 0.165138 4.77064 3.48624 -7.89796 -17.9796 -8.06422 1.77778 -0.87156
0.00145 0.00974 0.00196 0.0072 0.000678 0.00764 0.00213 0.00932 0.0252
3L:11527740-11527890:minus -12.9898 -2.08257 3.48624 5.59633 -16.8878 -18.7857 -11.2202 -10.5833
6.95413 0.000553 0.0199 0.00329 0.00331 0.00326 0.0146 0.0039 0.168 0.00136
3L:11527740-11527890:plus -23.8469 -0.93578 3.93578 5.93578 -8.63265 -18.051 -10.9266 -0.472222

-3.99083 0.00685 0.014 0.00275 0.00264 0.0013 0.00886 0.00368 0.0193 0.0556
3L:11537720-11537870:minus -32.1939 0.605505 0.40367 2.62385 -17.8265 -8.02041 -29.7248 5.875
-0.275229 0.0181 0.00834 0.0101 0.0108 0.00691 0.000761 0.0875 0.00176 0.0206
3L:11537720-11537870:plus -24.2959 1.02752 4.20183 10.8624 -27.2653 -19.0102 -12.5046 5.44444 7.63303
0.00781 0.00717 0.00247 0.0000687 0.0302 0.0159 0.00516 0.00215 0.000928
3L:11546500-11546650:minus -30.3265 1.52294 2.55046 5.45872 -18.7857 0.897959 -24.5505 1.41667
-0.0733945 0.0089 0.00598 0.00471 0.00363 0.0148 0.000144 0.0444 0.0106 0.0188
3L:11546500-11546650:plus -32.7041 -1.83486 12.9817 7.29358 -18.6837 -26.5306 -6.0367 -5.58333 -0.770642
0.0222 0.0185 0.0000211 0.00104 0.0126 0.023 0.00132 0.0698 0.0245
3L:11548920-11549070:minus -24.5204 -1.85321 8.3578 4.17431 -18.6837 -0.244898 -21.7706 5.38889
-2.88991 0.00813 0.0186 0.000361 0.00537 0.0126 0.000496 0.0283 0.0022 0.0433
3L:11548920-11549070:plus -32 -1.99083 7.85321 3.27523 0.316327 -26.6327 -15.1009 2.63889 -0.0825688
0.0171 0.0194 0.000473 0.00796 0.000341 0.0251 0.00916 0.00684 0.0189
3L:11550040-11550190:minus -23.6939 3.16514 -2.36697 4.37615 -17.2245 -9.23469 -19.5138 5.40278
-1.18349 0.00667 0.00327 0.0241 0.00504 0.00384 0.00238 0.019 0.00219 0.0276
3L:11550040-11550190:plus -21.5918 -4.30275 4.12844 1.61468 -8.60204 -19.1531 -15.4495 0.680556 3.76147
0.00209 0.0367 0.00254 0.0169 0.00125 0.0177 0.0098 0.0135 0.00494
3L:11555240-11555390:minus -22.6939 14.6789 0.0917431 1.6055 -17.1939 -27.898 -10.1651 1.51389
0.614679 0.00388 3.91e-06 0.0112 0.0169 0.00377 0.0537 0.00317 0.0102 0.0158
3L:11555240-11555390:plus -24.2245 6.47706 10.4128 5.31193 -8.90816 -17.5714 -23.1468 0.263889 -0.559633
0.00763 0.000843 0.000111 0.00393 0.00192 0.00616 0.0359 0.0154 0.0226
3L:11555560-11555710:minus -30.7755 3.83673 9.75229 7.21101 -17.7857 -27.6327 -17.3211 3.29167
-0.0733945 0.0102 0.00115 0.000164 0.0011 0.00676 0.043 0.0135 0.00533 0.0188
3L:11555560-11555710:plus -31.6633 14.0459 13.6422 6.33028 -8.60204 -19.1224 -15.0734 6.73611 1.6422
0.0141 7.98e-06 0.0000116 0.00198 0.00125 0.0175 0.00911 0.00115 0.0111
3L:11602440-11602590:minus -33.1122 2.55963 1.3578 3.47706 10.6531 9.7551 -22.9174 5.375
-1.22018 0.0271 0.00409 0.00728 0.00724 0.0000102 0.0000198 0.0346 0.00222 0.0278
3L:11602440-11602590:plus -29.7041 0.357798 3.38532 6.11009 -27.0408 -9.72449 -18.2661 4.41667 2.46789
0.00848 0.00911 0.00342 0.00233 0.0242 0.00352 0.0156 0.00338 0.00805
3L:11615300-11615450:minus -24.5204 -3.74312 4.26606 2.19266 -17.1939 -8.72449 -12.0642 -0.402778
-0.192661 0.00813 0.0316 0.00241 0.0131 0.00377 0.00169 0.00468 0.0189 0.0198
3L:11615300-11615450:plus -31.9286 2.3945 -2.33028 9.08257 -19.0102 -18.9388 -19.2569 -0.833333
7.02752 0.0166 0.00434 0.0238 0.000444 0.0157 0.0154 0.0182 0.0215 0.00133
3L:11616760-11616910:minus -22.2245 7.33945 13.8165 9.49541 -18.4184 -17.0102 -18.3394 11.3194
5.58716 0.00325 0.000544 9.45e-06 0.000308 0.0101 0.00453 0.0157 0.000058 0.00237
3L:11616760-11616910:plus -14.5204 1.08257 3.02752 -0.633028 -27.4592 -18.051 -5.20183 6.375
6.11927 0.00145 0.00703 0.00393 0.0327 0.0329 0.00886 0.00105 0.00138 0.00178
3L:11621040-11621190:minus -14.7041 5.20183 -2.95413 1.87156 -27.2245 -17.8265 -17.6697 5.38889
1.2844 0.00154 0.0015 0.0285 0.0152 0.0272 0.00699 0.0142 0.0022 0.0129
3L:11621040-11621190:plus -12.4082 3.97959 6.55046 6.27523 -7.67347 -17.0102 -8.68807 -0.0416667
4.09174 0.000414 0.000669 0.000896 0.00206 0.00055 0.00453 0.00241 0.017 0.00416
3L:11627780-11627930:minus -31.6633 2.22936 3.34862 2.15596 -27.4184 0.602041 -18.8073 -2.41667
-10.8624 0.0141 0.00461 0.00347 0.0133 0.0323 0.000188 0.0169 0.0333 0.227
3L:11627780-11627930:plus -23.6633 -1.3945 -3.12844 2.55046 -18.7857 -25.602 -12.1284 -3.68056 7.83486
0.00665 0.0162 0.0299 0.0112 0.0148 0.0207 0.00474 0.0457 0.000712
3L:11641500-11641650:minus 5.18367 -0.440367 -1.29358 3.61468 -18.7551 -9.79592 -12.3578 3.73611
-2.46789 0.0000136 0.012 0.0175 0.0068 0.0144 0.00377 0.00499 0.00447 0.0384
3L:11641500-11641650:plus -40.2959 2.73394 0.00917431 0.899083 19.9082 10.602 -23.7798 5.38889
-1.6422 0.0389 0.00383 0.0116 0.0212 8.13e-07 4.1e-06 0.0397 0.0022 0.0304
3L:11641740-11641890:minus 5.18367 -0.440367 -1.29358 3.61468 -18.7551 -9.79592 -12.3578 3.73611
-2.46789 0.0000136 0.012 0.0175 0.0068 0.0144 0.00377 0.00499 0.00447 0.0384
3L:11641740-11641890:plus -31.7755 -0.770642 0.00917431 1.21101 19.9082 -0.0204082 -14.4954
2.93056 3.40367 0.0157 0.0133 0.0116 0.0194 8.13e-07 0.000411 0.00808 0.00612 0.00603
3L:11675060-11675210:minus -24.5612 0.385321 -1.44037 1.92661 -18.7143 0.755102 -24.4862 1.01389

3.41284 0.00819 0.00902 0.0183 0.0148 0.0136 0.000175 0.044 0.0121 0.006
3L:11675060-11675210:plus -32.5918 4.17431 -3.74312 -2.66972 0.836735 -9.27551 -14.0183 0.361111 0.614679
0.021 0.00225 0.0354 0.0635 0.000215 0.00244 0.00728 0.0149 0.0158
3L:11677160-11677310:minus -28.3673 -0.284404 0.908257 6.25688 -17.5204 -18.7143 -20.1193 0.319444
5.80734 0.00831 0.0114 0.00853 0.00208 0.00532 0.014 0.021 0.0151 0.00205
3L:11677160-11677310:plus -4.43878 6.05505 3.02752 11.2936 -8.16327 -9.45918 -12.4312 -4.43056 -2.6055
0.000149 0.00103 0.00393 0.0000139 0.000873 0.00279 0.00508 0.0544 0.04
3L:11684580-11684730:minus -24 0.688073 4.12844 3.04587 -27.5306 -17.7143 -15.3303 3.88889 3.52294
0.00719 0.0081 0.00254 0.00883 0.0351 0.00634 0.00958 0.00421 0.00558
3L:11684580-11684730:plus -31.6735 -1.78899 15.7339 9.59633 -18.8265 -0.173469 -22.1927 7.52778
-2.49541 0.0141 0.0183 2.45e-07 0.000275 0.0149 0.000456 0.0305 0.00076 0.0387
3L:11689520-11689670:minus -22.9592 4.84404 5.29358 7.02752 -17.4184 -27.8571 -7.37615 2.06944
1.89908 0.00475 0.00174 0.00157 0.00122 0.00457 0.0508 0.00184 0.00841 0.0098
3L:11689520-11689670:plus -24.0306 1.43119 2.51376 3.13761 -27.602 -7.72449 -18.0917 -0.597222
-1.49541 0.00723 0.00619 0.00478 0.00854 0.0364 0.00068 0.0152 0.0201 0.0293
3L:11690020-11690170:minus -13.4082 2.77982 14.3486 7.29358 -17.2245 -27.5306 8.33028 -2.45833
5.81651 0.000867 0.00377 4.67e-06 0.00104 0.00384 0.0374 0.0000217 0.0337 0.00204
3L:11690020-11690170:plus -32.9286 -3.43119 -0.880734 1 -9.20408 -8.72449 -19.7156 5.375 3.42202
0.025 0.029 0.0154 0.0206 0.00262 0.00169 0.0196 0.00222 0.00597
3L:11701100-11701250:minus -31.8571 -0.0366972 2.12844 9.59633 -17.0408 1.12245 -32.8991 0.277778
-10 0.0163 0.0104 0.00551 0.000275 0.00329 0.000085 0.131 0.0153 0.194
3L:11701100-11701250:plus -22.1531 -0.743119 -0.40367 5.69725 -27.5306 -9.65306 -10.6514 -0.652778
-0.40367 0.0031 0.0132 0.0132 0.00314 0.0351 0.0034 0.00348 0.0204 0.0213
3L:11701840-11701990:minus -12.6531 3.22018 2.46789 6.05505 -26.1939 -17.2755 -1.47706 3.40278
1.56881 0.000455 0.00321 0.00486 0.0024 0.0175 0.00544 0.000394 0.00511 0.0114
3L:11701840-11701990:plus -21 -3.15596 2.49541 2.54128 -18.3469 -17.2755 -15.7706 0.611111 0.183486
0.00186 0.0269 0.00481 0.0112 0.00894 0.00544 0.0104 0.0138 0.0176
3L:11704440-11704590:minus -40.9184 -2.77064 1.55046 6.74312 20.4694 19.0816 -20.6606 2.66667
-1.73394 0.0443 0.0242 0.0068 0.0015 1.65e-07 1.12e-06 0.0231 0.00677 0.0311
3L:11704440-11704590:plus -33.0408 2.06422 1.69725 2.84404 -18.6429 -18.3469 -20.1651 3.44444 -2.51376
0.0264 0.0049 0.00646 0.00961 0.0121 0.0124 0.0212 0.00502 0.039
3L:1177180-1177330:minus -22.1939 -0.944954 -0.954128 9.7156 -27.5306 -8.45918 -9.63303 -0.944444
1.80734 0.00317 0.0141 0.0157 0.000242 0.0351 0.00111 0.00288 0.0222 0.0102
3L:1177180-1177330:plus -23.4694 -0.40367 9.49541 7.2844 -26.5918 -19.3776 -17.6514 1.18056 -0.761468
0.00632 0.0118 0.000191 0.00105 0.0186 0.0195 0.0142 0.0114 0.0244
3L:11782140-11782290:minus -23.2653 1.79817 5.11009 7.17431 -17.1531 -17.2041 -15.3028 -2.55556
1.37615 0.00593 0.00541 0.0017 0.00115 0.00364 0.00507 0.00953 0.0345 0.0123
3L:11782140-11782290:plus -23.2245 4.88991 2.31193 5.02752 -27.3061 -18.3776 -9.66055 0.875 11.8532
0.00576 0.0017 0.00515 0.00435 0.0315 0.0125 0.00289 0.0127 0.0000428
3L:11782860-11783110:minus -13.3367 13.5306 2.23853 2.74312 -18.7857 -8.65306 -22.7431 3.44444
-6.77064 0.0008 0.0000206 0.00529 0.0101 0.0148 0.00156 0.0336 0.00502 0.107
3L:11782860-11783110:plus -24.4898 13.6972 -0.477064 5.22936 -27.2347 -8.45918 -3.66972 2.22222
1.86239 0.00807 0.0000119 0.0135 0.0041 0.0283 0.00111 0.00068 0.00796 0.00994
3L:11808460-11808610:minus -21.8878 6.7156 -0.0366972 3.01835 -18.7143 -0.397959 -15.6697
0.680556 0.963303 0.00258 0.00075 0.0117 0.0089 0.0136 0.000525 0.0102 0.0135 0.0145
3L:11808460-11808610:plus -22.0714 4.44954 15.6606 6.00917 -17.5306 -9.42857 -17.1927 3.73611 3.88073
0.00299 0.00202 3.94e-07 0.00255 0.0054 0.0027 0.0132 0.00447 0.00475
3L:11813020-11813170:minus -14.1122 1.52294 0.587156 7.27523 -7.33673 -17.051 -11.7615 4.23611
-2.66972 0.00119 0.00598 0.00952 0.00109 0.000388 0.00479 0.00438 0.00364 0.0407
3L:11813020-11813170:plus -13.2959 1.3578 -1.23853 4.15596 -17.6429 -16.9388 -24.6147 5.625 1.07339
0.000775 0.00635 0.0172 0.00539 0.00562 0.00433 0.0448 0.00198 0.014
3L:11815320-11815470:minus -31.6327 1.25688 7.53211 2.7156 -18.9388 -17.9796 -25.3394 2.63889
-2.93578 0.0138 0.00659 0.000558 0.0102 0.0151 0.00764 0.0493 0.00684 0.0439
3L:11815320-11815470:plus -32.6633 3.64286 7.27523 6.73394 -27.0408 -18.6429 -19.2661 3.23611 -0.972477

0.0216	0.00159	0.000635	0.00152	0.0242	0.0139	0.0182	0.00545	0.0259
3L:11815860-11816010:minus	-41.2143	1.02752	-0.568807	4.14679	-18.7857	-9.82653	-14.5688	3.16667
3.31193	0.0494	0.00717	0.0139	0.00542	0.0148	0.00381	0.00821	0.0056	0.00618
3L:11815860-11816010:plus	-13.449	6.6422	2.48624	1.70642	-28.2755	-18.1224	-17.6606	0.333333	2.33028
0.000914	0.000778	0.00483	0.0162	0.0539	0.00988	0.0142	0.0151	0.00836
3L:11818760-11818910:minus	-40.5918	13.5306	1.01835	4.13761	-28.5612	-26.051	-17.945	5.36111
-2.57798	0.0422	0.0000206	0.00821	0.00546	0.0617	0.0216	0.0148	0.00223	0.0397
3L:11818760-11818910:plus	-22.6224	13.5306	2.05505	4.05505	-18.4184	-8.45918	-16.1009	5.02778	-0.963303
0.00371	0.0000206	0.00566	0.00566	0.0101	0.00111	0.011	0.00259	0.0258
3L:11825620-11825770:minus	-31.7041	5.21101	-0.577982	5.31193	-18.4796	-8.7551	-7.79817	6.81944
5.33945	0.0146	0.00149	0.014	0.00393	0.011	0.00172	0.00202	0.0011	0.00271
3L:11825620-11825770:plus	-31.7755	-5.11009	-6.08257	2.49541	-8.86735	-16.2041	-10.5413	2.86111	4.10092
0.0157	0.0454	0.0646	0.0114	0.00173	0.00408	0.00341	0.00629	0.00413
3L:11826280-11826430:minus	-23.8061	-3.78899	3.65138	3.44954	-18.7857	-35.8163	-15.2661	-1.61111
9.92661	0.0068	0.032	0.00308	0.00738	0.0148	0.0815	0.00946	0.0268	0.000193
3L:11826280-11826430:plus	-32.7755	7.09174	2.88073	1.86239	-17.1837	-17.898	-14.7339	3.04167	0.137615
0.0234	0.000619	0.00415	0.0153	0.00366	0.00726	0.0085	0.00587	0.0178
3L:119460-119610:minus	-13.6735	0.0458716	3.98165	9.33028	-7.89796	-8.53061	-12.1927	7.27778
10.3486	0.00103	0.0101	0.0027	0.000374	0.000678	0.00136	0.00481	0.000869	0.0000988
3L:119460-119610:plus	-33	1.0367	11.3761	-0.642202	-27.6735	-9.5	-19.156	2.75	4.61468	0.0258
0.00714	0.0000633	0.0328	0.0375	0.00294	0.0179	0.00656	0.00351
3L:11986160-11986310:minus	-33.2653	-2.51376	-2.00917	9.59633	-26.9694	0.0918367	-16.8257	11.7361
3.51376	0.0277	0.0225	0.0217	0.000275	0.023	0.000323	0.0125	0.0000398	0.00566
3L:11986160-11986310:plus	-23.5612	6.31193	0.715596	1.48624	-17.449	-26.051	-14.4037	-2.83333	7.53211
0.00653	0.000913	0.00912	0.0177	0.00484	0.0216	0.00792	0.0371	0.000968
3L:11991220-11991370:minus	-33.8163	2.7156	-0.0917431	2.88991	10.9184	-7.5	-7.57798	2.80556
-2.0367	0.0299	0.00386	0.0119	0.00942	4.19e-06	0.000614	0.00192	0.00642	0.0336
3L:11991220-11991370:plus	-40.1735	1.63303	-0.577982	3.08257	-18.449	0.316327	-22.7798	4.80556
-0.862385	0.0363	0.00575	0.014	0.0087	0.0107	0.000243	0.0338	0.00286	0.0251
3L:11996100-11996250:minus	-21.2959	13.4592	2.3945	4.22018	-17.3776	-8.45918	-15.5596	-0.0555556
9.88073	0.00192	0.000064	0.00499	0.00531	0.00419	0.00111	0.01	0.017	0.000206
3L:11996100-11996250:plus	-24.2143	2.6422	12.1376	7.55963	0.540816	-17.6429	-9.06422	4.94444	3.94495
0.00754	0.00397	0.000039	0.000807	0.000321	0.00619	0.00259	0.00269	0.00452
3L:11996580-11996730:minus	-31.4082	4.62385	2.46789	11.0275	-18.4898	-10.0204	-23.1651	3.18056
1.89908	0.0119	0.00189	0.00486	0.0000401	0.0116	0.00399	0.036	0.00557	0.0098
3L:11996580-11996730:plus	-32.0714	3.13761	3.80734	5.73394	-18.1531	-17.8571	-11.6972	4.88889	2.51376
0.0176	0.00331	0.0029	0.00307	0.00829	0.00719	0.00432	0.00276	0.00792
3L:12049640-12049790:minus	-22.6633	1.26606	-0.633028	9.49541	-26.9286	-9.23469	4.3945	1.875
1.38532	0.00384	0.00657	0.0142	0.000308	0.0214	0.00238	0.0000715	0.00901	0.0123
3L:12049640-12049790:plus	-31.0714	0.59633	1.98165	2.22936	-17.9388	-26.5612	2.48624	0.180556	9.66055
0.0106	0.00837	0.00582	0.0128	0.00715	0.0237	0.000141	0.0158	0.000268
3L:12074880-12075030:minus	-30.4796	7.74312	2.17431	9.59633	-17.4184	-8.45918	-6.93578	-7.16667
-1.79817	0.00941	0.000438	0.00542	0.000275	0.00457	0.00111	0.00166	0.0955	0.0315
3L:12074880-12075030:plus	-22.8163	6.97248	1.45872	11.0183	-18.7143	-18.8571	-1.77064	0.680556	11.8532
0.00418	0.000659	0.00703	0.000052	0.0136	0.0151	0.000421	0.0135	0.0000428
3L:12109060-12109210:minus	-31.6327	1.11009	-3.07339	6.83486	-18.7143	-18.1531	-16.789	2.55556
1.22018	0.0138	0.00695	0.0294	0.00142	0.0136	0.00997	0.0124	0.00705	0.0133
3L:12109060-12109210:plus	-23.2143	7.09174	-0.266055	1.36697	-19.2755	-17.2755	-19.0642	0.111111
1.95413	0.00559	0.000619	0.0126	0.0186	0.0164	0.00544	0.0176	0.0162	0.00964
3L:12109340-12109490:minus	-24.5204	13.7798	0.412844	5.69725	-18.7143	-9.30612	-20.2294	5.91667
-3.99083	0.00813	0.0000109	0.0101	0.00314	0.0136	0.00257	0.0214	0.00172	0.0556
3L:12109340-12109490:plus	-23.0306	0.155963	-2.76147	11.1927	-17.3776	-27.5612	-2.90826	-3.11111	7.6422
0.00505	0.00977	0.027	0.0000254	0.00419	0.0393	0.000555	0.0397	0.000893
3L:12111280-12111430:minus	-24.5204	15.4037	-1.6789	4.10092	-19.051	-9.0102	-24.1101	3.88889

-2.36697 0.00813 1.16e-06 0.0196 0.00554 0.0162 0.00208 0.0417 0.00421 0.0371
3L:12111280-12111430:plus -14.5204 13.3878 5.19266 1.56881 -17.7143 -8.72449 -14.7982 3.56944 -1.29358
0.00145 0.0000869 0.00164 0.0172 0.00627 0.00169 0.00861 0.00478 0.0283
3L:12113020-12113170:minus -40.449 13.3878 -4.73394 2.25688 -17.7143 -18.051 -15.8165 3.13889
3.88991 0.0402 0.0000869 0.0461 0.0127 0.00627 0.00886 0.0105 0.00566 0.00467
3L:12113020-12113170:plus -22.6224 -3.76147 5.25688 1.33028 -18.4898 -0.397959 -19.1284 0.972222
2.75229 0.00371 0.0317 0.0016 0.0188 0.0116 0.000525 0.0178 0.0123 0.00748
3L:12117380-12117530:minus -32.7755 0.229358 -4.30275 -0.0183486 -26.9694 -19.3061 -16.5872 2.06944
0.495413 0.0234 0.00953 0.0412 0.027 0.023 0.0187 0.012 0.00841 0.0162
3L:12117380-12117530:plus -2.84694 2.23853 1.34862 6.00917 -18.4592 -9.30612 -17.0367 -3.04167 7.46789
0.0000385 0.0046 0.00731 0.00255 0.0109 0.00257 0.0129 0.0391 0.00102
3L:12118220-12118370:minus -29.1429 5.41284 1.31193 1.78899 -18.1531 -18.051 -11.9725 -0.861111
1.2844 0.00834 0.00137 0.00741 0.0158 0.00829 0.00886 0.00458 0.0217 0.0129
3L:12118220-12118370:plus -22.7041 13.6422 1.88073 2.3578 -9.16327 -27.3367 -19.0367 -1.63889 2.84404
0.00395 0.0000127 0.00604 0.0121 0.00249 0.0347 0.0176 0.027 0.00736
3L:12129860-12130010:minus -31.4796 2.69725 5.04587 0.834862 -17.7143 -9.5 -21.5596 6.51389 1.95413
0.0128 0.00389 0.00174 0.0215 0.00627 0.00294 0.0272 0.00129 0.00964
3L:12129860-12130010:plus -23.4082 15.3853 4.24771 3.08257 -17.7143 -9.60204 -23.4771 -0.111111
-5.50459 0.00623 1.25e-06 0.00243 0.0087 0.00627 0.00334 0.0379 0.0173 0.0806
3L:12131760-12131910:minus -30.8163 14.0275 8.19266 2.55963 -17.3776 -18.0816 -20.5872 2.47222
1.90826 0.0103 8.23e-06 0.000395 0.0111 0.00419 0.00934 0.0228 0.00727 0.00974
3L:12131760-12131910:plus -31.6735 8.83486 -1.6422 1.62385 -18.1531 0.0204082 -11.7615 3.61111
-4.00917 0.0141 0.000244 0.0194 0.0168 0.00829 0.000377 0.00438 0.0047 0.0558
3L:12138900-12139250:minus -33.0408 13.9725 1.18349 5.73394 20.2041 -17.3469 -27.4587 -1.40278
-8.31193 0.0264 8.89e-06 0.00775 0.00307 3.64e-07 0.0058 0.0641 0.0253 0.144
3L:12138900-12139250:plus -21.8469 15.7248 0.174312 2.50459 -18.9796 -9.5 -18.8624 -1.81944 -4.43119
0.00247 5.64e-07 0.0109 0.0114 0.0155 0.00294 0.0171 0.0284 0.0627
3L:12139520-12139670:minus -33.0714 -1.79817 -0.155963 0.899083 -28.4592 -8.79592 -18.7064 6.01389
1.48624 0.0267 0.0183 0.0122 0.0212 0.0568 0.0019 0.0167 0.00165 0.0118
3L:12139520-12139670:plus -13.2959 -0.770642 6.53211 6.89908 20.4694 -7.23469 -9.66972 -3.5 3.87156
0.000775 0.0133 0.000904 0.00134 1.65e-07 0.000557 0.0029 0.0437 0.00477
3L:12140140-12140290:minus -23.7857 6.84404 4.01835 6.29358 -26.0408 -26.5612 -10.2844 2.29167
9.88073 0.00679 0.000705 0.00266 0.00202 0.0171 0.0237 0.00325 0.00776 0.000206
3L:12140140-12140290:plus -23.9184 1.78899 6.83486 6.89908 -26.9694 -8.45918 -18.2936 0.694444 6.7156
0.00695 0.00542 0.000785 0.00134 0.023 0.00111 0.0156 0.0134 0.00144
3L:12196980-12197130:minus -21.8163 5.68807 1.45872 9.41284 -7.56122 -26.5612 -5.84404 -3.44444
5.57798 0.00244 0.00122 0.00703 0.000354 0.000419 0.0237 0.00126 0.0431 0.00238
3L:12196980-12197130:plus -23.1837 -3.42202 -1.04587 5.85321 -17.9796 -8.79592 -18.9908 -2.41667 -6.76147
0.00553 0.0289 0.0162 0.00287 0.00726 0.0019 0.0174 0.0333 0.107
3L:12258700-12258850:minus -34.2653 2.43119 0.293578 2.25688 -28.3061 -8.5 -29.3853 4.13889 -4.2844
0.0316 0.00428 0.0105 0.0127 0.0553 0.00126 0.0833 0.0038 0.0597
3L:12258700-12258850:plus -32.2143 1.79817 -2 5.65138 -8.7449 -28.1837 0.458716 -1.84722 1.69725
0.0182 0.00541 0.0216 0.00322 0.00161 0.0584 0.000254 0.0286 0.0108
3L:12259060-12259210:minus -24.1837 8.11009 -0.541284 3.24771 -18.449 -18.0102 -24.3853 7.09722
11.3853 0.00751 0.000359 0.0138 0.00808 0.0107 0.00812 0.0434 0.000956 0.0000815
3L:12259060-12259210:plus -40.2857 5.31193 4.56881 2.26606 -18.3061 -17.5714 -9.52294 7.22222 -0.440367
0.0385 0.00143 0.00213 0.0126 0.00884 0.00616 0.00282 0.000895 0.0216
3L:12259900-12260050:minus -11.0408 -3.00917 2.65138 9.3211 1.36735 1.31633 -20.2477 1.15278
-5.55046 0.000241 0.0258 0.00453 0.000391 0.0000781 0.000066 0.0215 0.0115 0.0811
3L:12259900-12260050:plus -31.4388 1.46789 2.73394 -0.357798 2.14286 -18.3776 -13.6697 2.66667
2.42202 0.0122 0.0061 0.00439 0.0297 0.0000316 0.0125 0.00674 0.00677 0.00816
3L:12260520-12260670:minus -32.2653 2.77064 4.40367 2.75229 -28.5306 -9.02041 -24.2202 5.05556
1.36697 0.0184 0.00378 0.00228 0.0101 0.061 0.00213 0.0424 0.00256 0.0124
3L:12260520-12260670:plus -31.6224 6.40367 13.4128 7.45872 -8.11224 -26.602 -11.3394 1.72222 3.51376

0.0137 0.000874 0.0000141 0.000894 0.000838 0.0248 0.004 0.0095 0.00566
3L:12262940-12263090:minus -13.1429 3.04587 -1.23853 7.27523 -18.1531 -17.1224 8.20183 -3.06944
3.66972 0.00065 0.00342 0.0172 0.00109 0.00829 0.00499 0.0000225 0.0393 0.00514
3L:12262940-12263090:plus -32.6735 -0.348624 -1.82569 1.89908 -27 -8.79592 -8.49541 0.333333 7.62385
0.0216 0.0116 0.0205 0.0151 0.0233 0.0019 0.00232 0.0151 0.000941
3L:12329580-12329730:minus -4.33673 4.76147 6.97248 2.15596 -17.4184 -9.02041 -19.1835 8.70833
0.93578 0.00014 0.00179 0.000737 0.0133 0.00457 0.00213 0.018 0.000384 0.0145
3L:12329580-12329730:plus -13.1429 4.90816 -3.16514 1.78899 -18.7143 0.602041 -17.1927 3.36111 -0.59633
0.00065 0.00023 0.0302 0.0158 0.0136 0.000188 0.0132 0.00519 0.0229
3L:12384500-12384650:minus -12.1122 3.97959 0.87156 11.0183 -18.0816 -9.72449 -12.578 -2.94444
-2.52294 0.000336 0.000669 0.00864 0.000052 0.00759 0.00352 0.00525 0.0381 0.0391
3L:12384500-12384650:plus -13.6735 -0.87156 3.20183 4.65138 -18.3878 -16.9796 1.75229 -2.27778 2.36697
0.00103 0.0138 0.00367 0.00471 0.00931 0.0044 0.000178 0.0321 0.00828
3L:12392440-12392590:minus -32.8878 3.68367 0.972477 4.6055 10.9184 0.27551 -9.43119 1.47222
1.38532 0.0247 0.00144 0.00835 0.00476 4.19e-06 0.000257 0.00277 0.0104 0.0123
3L:12392440-12392590:plus -31.8878 2.36697 0.724771 7.27523 -18.7143 -18.8571 -21.6055 0.819444 2.04587
0.0164 0.00439 0.00909 0.00109 0.0136 0.0151 0.0275 0.0129 0.00919
3L:12401660-12401810:minus -23.7041 -0.559633 1.81651 1.0367 -17.7143 -18.1224 -8.75229 5.65278
7.36697 0.00669 0.0124 0.00619 0.0204 0.00627 0.00988 0.00244 0.00195 0.00108
3L:12401660-12401810:plus -31.9694 -1.51376 -5.07339 5.3945 -18.1837 0.826531 -11.7706 6.375 3.87156
0.0168 0.0168 0.0503 0.00379 0.00835 0.000153 0.00439 0.00138 0.00477
3L:12403760-12403910:minus -22.8163 -5.42202 2.89908 4.33028 -18.2653 9.60204 -21.3211 0 4.88073
0.00418 0.0493 0.00413 0.00511 0.00882 0.0000248 0.0261 0.0167 0.00324
3L:12403760-12403910:plus -23.6633 1.97248 11.4128 4.07339 -27.9694 -8.23469 -17.2936 3.84722 -0.642202
0.00665 0.00507 0.0000621 0.00562 0.0437 0.000875 0.0134 0.00428 0.0234
3L:12404380-12404530:minus -23.1122 8.95413 -0.0733945 5.59633 -9.16327 -8.45918 -20.0183 2.48611
-3.55963 0.0053 0.00023 0.0119 0.00331 0.00249 0.00111 0.0207 0.00723 0.0503
3L:12404380-12404530:plus -24.3367 2.85321 4.55046 0.366972 -27.5714 -18.2755 -7.07339 4.75 -0.211009
0.00789 0.00367 0.00214 0.0244 0.0357 0.0112 0.00172 0.00293 0.02
3L:12407820-12407970:minus -23.2245 1.29358 -4.88991 2.19266 20.2041 -0.244898 -10.3119 4.08333
-5.82569 0.00576 0.00651 0.048 0.0131 3.64e-07 0.000496 0.00326 0.00388 0.0856
3L:12407820-12407970:plus -22.6327 0.376147 1.10092 2.29358 -18.4184 -9.65306 -9.50459 0.236111 -3
0.00373 0.00905 0.00798 0.0124 0.0101 0.0034 0.00281 0.0155 0.0447
3L:12410580-12410730:minus -22.7449 9.3578 4.79817 6.13761 -16.1531 0.244898 -19.1743 1.76389
0.275229 0.00404 0.000186 0.00193 0.00226 0.00297 0.000285 0.0179 0.00937 0.0171
3L:12410580-12410730:plus -33.9184 14.8073 -0.495413 0.522936 0.540816 -17.2755 -8.88991 1.83333
-1.75229 0.0302 3.09e-06 0.0136 0.0234 0.000321 0.00544 0.00251 0.00914 0.0312
3L:12464580-12464730:minus -33.0714 2.17431 4.48624 -1.74312 -27.5306 -9.45918 -27.4954 6.77778
-1.18349 0.0267 0.00471 0.0022 0.0467 0.0351 0.00279 0.0644 0.00113 0.0276
3L:12464580-12464730:plus -31.5204 9.09174 0.614679 5.42202 -17.3776 -19.2755 -17.9725 -0.263889
1.22936 0.0131 0.000213 0.00943 0.00374 0.00419 0.0185 0.0149 0.0181 0.0132
3L:1247940-1248090:minus -24.2551 13.4592 -0.908257 0.642202 -18.449 -19.2755 -2.18349 -1.29167
1.95413 0.00768 0.000064 0.0155 0.0227 0.0107 0.0185 0.000464 0.0245 0.00964
3L:1247940-1248090:plus -31.7347 -0.669725 7.43119 6.43119 -17.4082 -17.2755 -12.6972 4.48611
2.15596 0.0149 0.0129 0.000587 0.00187 0.00421 0.00544 0.00539 0.00328 0.00881
3L:12483220-12483370:minus -22.8469 1.55963 -0.321101 9.33028 -28.4592 -18.7857 -16.3486 0.25
10.1284 0.00424 0.0059 0.0129 0.000374 0.0568 0.0146 0.0115 0.0155 0.000129
3L:12483220-12483370:plus -21.1735 -0.807339 -1.16514 2.77982 -16.3776 -18.5714 -11.5046 -1.09722
-0.321101 0.00189 0.0135 0.0168 0.00997 0.003 0.0136 0.00414 0.0232 0.0208
3L:12483620-12483770:minus -22.6633 0.688073 1.80734 7.17431 -17.3776 -9.23469 -10.9633 3.625
-2.15596 0.00384 0.0081 0.00621 0.00115 0.00419 0.00238 0.00371 0.00468 0.0348
3L:12483620-12483770:plus -13.4082 -1.16514 -2.46789 9.49541 -26.1939 -16.0102 -2.14679 2.88889 5.12844
0.000867 0.0151 0.0248 0.000308 0.0175 0.00406 0.00046 0.00622 0.00301
3L:12484020-12484170:minus -21.7449 -0.486239 2.58716 2.62385 -18.7449 -18.051 -10.8073 0.888889

7.88991 0.00236 0.0121 0.00465 0.0108 0.0138 0.00886 0.00359 0.0126 0.000688
3L:12484020-12484170:plus -24.1122 3.6789 5.90826 4.0367 -18.7143 -26.5306 -16.055 4.875 3.88991
0.00738 0.00271 0.0012 0.00578 0.0136 0.023 0.011 0.00277 0.00467
3L:12494500-12494650:minus -32.1122 5.37615 0.844037 0.633028 -8.60204 -8.68367 -19.6606 1.65278
-0.93578 0.0178 0.00139 0.00872 0.0229 0.00125 0.00157 0.0194 0.00974 0.0257
3L:12494500-12494650:plus -32 -0.972477 14.8991 2.63303 -18.4184 -8.72449 -22.1651 4.11111 -4.24771
0.0171 0.0142 1.97e-06 0.0107 0.0101 0.00169 0.0304 0.00384 0.0591
3L:12497300-12497450:minus -21.8878 3.92661 -0.651376 2.25688 20.4694 -18.1939 -15.7064 -2.70833
5.7156 0.00258 0.00247 0.0143 0.0127 1.65e-07 0.0101 0.0103 0.0359 0.00217
3L:12497300-12497450:plus -31.551 5.0367 2.24771 2.66972 -9.19388 -18.1633 -22.9908 2.65278 0.220183
0.0134 0.0016 0.00527 0.0106 0.00253 0.01 0.035 0.0068 0.0174
3L:12507940-12508090:minus -22.7755 -0.66055 -1.33945 3.05505 -26.6327 -9.5 2.68807 1.30556 5.18349
0.00412 0.0129 0.0177 0.00878 0.0188 0.00294 0.000132 0.011 0.00295
3L:12507940-12508090:plus -21.6633 3.01835 -1.97248 5.57798 -25.8571 -17.9388 -5.66055 -3.43056 2.78899
0.00223 0.00346 0.0214 0.0034 0.0166 0.00738 0.0012 0.043 0.00745
3L:12517160-12517310:minus -31.6224 5.7156 -0.293578 0.770642 -7.93878 -18.3469 -18.4037 1.38889
0.275229 0.0137 0.0012 0.0128 0.0219 0.000767 0.0124 0.0159 0.0107 0.0171
3L:12517160-12517310:plus -31.7041 5.0367 -2.16514 0.743119 -19.0918 -9.09184 -17.6514 4.31944 -4.6422
0.0146 0.0016 0.0227 0.0221 0.0163 0.00219 0.0142 0.00352 0.0662
3L:12519780-12519930:minus -22.2245 -3.85321 3.77064 7.88991 10.9184 0.0918367 -12.5596 -1.90278
-6.88073 0.00325 0.0325 0.00294 0.00062 4.19e-06 0.000323 0.00523 0.029 0.11
3L:12519780-12519930:plus -39.551 2.58716 4.99083 3.51376 -18.6735 -17.2755 -19.3303 4.51389 -8.3211
0.0335 0.00404 0.00178 0.00709 0.0123 0.00544 0.0184 0.00324 0.144
3L:12523000-12523150:minus -23.3776 1.40367 -0.192661 3.3578 -17.7143 -9.27551 -8.37615 -2.97222
0.651376 0.00619 0.00625 0.0123 0.00767 0.00627 0.00244 0.00227 0.0384 0.0156
3L:12523000-12523150:plus -32.398 -3.29358 -2.36697 2.62385 20.4694 -9.45918 -4.89908 -0.722222
-2.3945 0.019 0.028 0.0241 0.0108 1.65e-07 0.00279 0.000965 0.0208 0.0375
3L:12526840-12526990:minus -31.9388 -1.22936 1.10092 2.18349 10.3878 -8.79592 -19.8349 2.75 -1.06422
0.0166 0.0154 0.00798 0.0131 0.0000174 0.0019 0.02 0.00656 0.0266
3L:12526840-12526990:plus -31.6327 -3.44037 7.3578 2.34862 -8.37755 -8.65306 -20.7064 -0.5 -6.52294
0.0138 0.0291 0.000608 0.0121 0.00106 0.00156 0.0233 0.0195 0.101
3L:12527260-12527410:minus -24.0408 -0.825688 -1.52294 2.7156 -17.4898 0.826531 -14.6514 -0.875
1.23853 0.00726 0.0136 0.0187 0.0102 0.0053 0.000153 0.00835 0.0218 0.0132
3L:12527260-12527410:plus -32.6633 5.2844 0.155963 2.46789 -17.3469 -18.3061 -11.4495 3.25 -0.559633
0.0216 0.00145 0.011 0.0115 0.00394 0.0114 0.00409 0.00542 0.0226
3L:12527620-12527770:minus -30.898 1.14679 -2.88073 5.58716 -27.4898 -10.0204 -16.4037 6.68056
2.09174 0.0103 0.00686 0.0279 0.00334 0.0336 0.00399 0.0116 0.00119 0.00904
3L:12527620-12527770:plus -23.9592 5.2844 -0.412844 2.84404 -26.8571 -9.79592 -18.9633 6.54167
-2.27523 0.00708 0.00145 0.0133 0.00961 0.0198 0.00377 0.0173 0.00127 0.036
3L:12530340-12530490:minus -21.398 -0.688073 6.30275 -0.311927 -27.602 -8.5 -13.4771 6.66667
2.11927 0.00197 0.013 0.00101 0.0294 0.0364 0.00126 0.00645 0.00119 0.00889
3L:12530340-12530490:plus -33.0408 3.6789 7.68807 7.61468 -27.2347 9.57143 -16.4679 1.33333 4.77982
0.0264 0.00271 0.000516 0.000779 0.0283 0.0000274 0.0118 0.0109 0.00339
3L:12565380-12565530:minus -23.6327 2.86239 3.24771 7.80734 -18.1531 -9.5 -16.4312 -2.23611 2.16514
0.00661 0.00366 0.00361 0.00063 0.00829 0.00294 0.0117 0.0317 0.00879
3L:12565380-12565530:plus -30.5102 -3.82569 4.63303 3.45872 0.877551 -19.6429 -18.1193 4.30556 1.00917
0.00947 0.0323 0.00207 0.00731 0.00019 0.0204 0.0152 0.00354 0.0143
3L:12727420-12727570:minus -30.4388 2.04587 3.02752 2.88073 -17.6429 -0.102041 -8.72477 0.194444
3.72477 0.00923 0.00494 0.00393 0.00945 0.00562 0.000443 0.00243 0.0157 0.00497
3L:12727420-12727570:plus -32.9184 -0.853211 2.30275 1.26606 -18.4184 -9.72449 -11.055 -0.458333
-1.26606 0.0248 0.0137 0.00517 0.0191 0.0101 0.00352 0.00377 0.0192 0.0281
3L:12728540-12728690:minus -32.4388 -0.174312 -0.0917431 3.31193 -27.1939 -17.1224 -22.1009
4.88889 -1.68807 0.0195 0.0109 0.0119 0.00786 0.0267 0.00499 0.03 0.00276 0.0308
3L:12728540-12728690:plus -40.5204 -0.743119 -1.45872 5.77982 -27.4898 -0.244898 -27.2661 5.54167

-4.33028 0.0413 0.0132 0.0184 0.00294 0.0336 0.000496 0.0625 0.00205 0.0607
3L:12734740-12734890:minus -33.2959 13.5306 3.72477 6.09174 -18.3878 0.204082 -15.1468 2.83333
-4.55963 0.0279 0.0000206 0.003 0.00235 0.00931 0.000296 0.00924 0.00635 0.0648
3L:12734740-12734890:plus -23 15.5963 -0.816514 5.59633 -18.8265 -0.469388 -12.0734 -1.36111
-1.77982 0.00498 8.91e-07 0.0151 0.00331 0.0149 0.000533 0.00469 0.025 0.0314
3L:12738400-12738550:minus -30.8163 -2.31193 -1.34862 9.59633 -25.9694 -8.65306 -12.5229 7.97222
6.69725 0.0103 0.0213 0.0178 0.000275 0.017 0.00156 0.00518 0.000593 0.00145
3L:12738400-12738550:plus -13.898 -0.146789 0.0917431 6.84404 20.2041 0.0204082 -17.7156
2.88889 -2.50459 0.00111 0.0108 0.0112 0.0014 3.64e-07 0.000377 0.0143 0.00622 0.0388
3L:12742860-12743010:minus -32.4694 -1.99083 2.55046 0.917431 -28 -17.0102 -27.5321 6.01389 -0.220183
0.0196 0.0194 0.00471 0.021 0.0444 0.00453 0.0647 0.00165 0.0201
3L:12742860-12743010:plus -31.4898 3.64286 2.42202 5.85321 -26.9286 -16.9796 -21.4587 4.55556 -2.72477
0.0128 0.00159 0.00495 0.00287 0.0214 0.0044 0.0267 0.00318 0.0414
3L:12743720-12743870:minus -24.7449 4.3945 4.18349 2.15596 -28.2347 -18.3469 -13.4037 4.75 3.46789
0.00827 0.00207 0.00249 0.0133 0.051 0.0124 0.00634 0.00293 0.00582
3L:12743720-12743870:plus -32.1531 -2.01835 0.220183 1.66055 -26.1939 -19.1224 -7.36697 1.56944 4.19266
0.0179 0.0195 0.0108 0.0166 0.0175 0.0175 0.00184 0.01 0.00401
3L:12747980-12748130:minus -32.4082 2.63303 -3.82569 6.52294 -28.3061 -9.45918 -19.8073 0.666667
-3.33945 0.0191 0.00398 0.0362 0.00174 0.0553 0.00279 0.0199 0.0135 0.0479
3L:12747980-12748130:plus -40.7755 0.944954 5.88991 2.41284 -27.3061 -9.53061 -22.8257 6.48611 -0.816514
0.0433 0.00739 0.00121 0.0118 0.0315 0.0031 0.0341 0.00131 0.0249
3L:12761800-12761950:minus -13.4796 -0.201835 -4.33945 2.19266 -17.6429 -9.79592 -1.88073 9.72222
1.74312 0.000935 0.011 0.0416 0.0131 0.00562 0.00377 0.000432 0.000199 0.0105
3L:12761800-12761950:plus -14.551 4.82569 2.65138 0.816514 -18.1939 -18.3469 -8.70642 8.30556 5.23853
0.00146 0.00175 0.00453 0.0216 0.0086 0.0124 0.00242 0.000489 0.00285
3L:12766000-12766150:minus -32 0.559633 7.43119 7.00917 -8.96939 -19.2755 -20.789 0 -2.80734
0.0171 0.00848 0.000587 0.00126 0.00226 0.0185 0.0237 0.0167 0.0423
3L:12766000-12766150:plus -32.5102 -3.57798 8.57798 3.69725 -17.4898 -17.6429 -13.3119 -1.06944 5.22936
0.0202 0.0302 0.000318 0.00665 0.0053 0.00619 0.00621 0.023 0.00288
3L:12766560-12766710:minus -31.5102 13.4592 6.76147 1.33028 -17.6735 -17.2755 -22.5963 4.45833
-1.18349 0.013 0.000064 0.000811 0.0188 0.00572 0.00544 0.0328 0.00332 0.0276
3L:12766560-12766710:plus -32.8571 13.4592 7.40367 0.917431 -19.051 -17.9796 -17.8349 5.63889 1.79817
0.0244 0.000064 0.000595 0.021 0.0162 0.00764 0.0146 0.00196 0.0102
3L:12781720-12781870:minus -24.449 3.05505 -0.33945 2.70642 -17.7143 -18.051 -12.2018 -4.73611
2.09174 0.00802 0.00341 0.0129 0.0103 0.00627 0.00886 0.00482 0.0582 0.00904
3L:12781720-12781870:plus -31.7041 -2.10092 8.61468 11.2936 -18.1531 -8.93878 -4.06422 0.152778 5.90826
0.0146 0.02 0.000312 0.0000139 0.00829 0.00201 0.00076 0.016 0.00198
3L:12783200-12783350:minus -22.6633 0.229358 -0.266055 1.56881 -28.5306 -9.79592 -19.6972 4.77778
2.54128 0.00384 0.00953 0.0126 0.0172 0.061 0.00377 0.0196 0.00289 0.00787
3L:12783200-12783350:plus -4.59184 5.22936 3.30275 5.90826 -18.4898 -18.2347 -9.87156 2.77778 -3.08257
0.000166 0.00148 0.00353 0.00271 0.0116 0.0104 0.00301 0.00649 0.0455
3L:12784160-12784310:minus -32.6633 -0.40367 -1.72477 9.33028 -17.3367 -17.051 -21.367 -3.34722
-4.69725 0.0216 0.0118 0.0199 0.000374 0.00391 0.00479 0.0263 0.0421 0.0673
3L:12784160-12784310:plus -24 1.0367 -1.41284 11.0183 -18.1531 -9.27551 0.889908 -5.06944 5.22018
0.00719 0.00714 0.0181 0.000052 0.00829 0.00244 0.000227 0.0626 0.00289
3L:12787780-12787930:minus -19.9286 -5 7.31193 7.47706 -18.0816 -9.42857 -15.8349 -0.319444
0.238532 0.00164 0.0441 0.000623 0.00085 0.00759 0.0027 0.0105 0.0184 0.0173
3L:12787780-12787930:plus -31.0714 3.61224 7.33945 7.37615 -7.56122 -8.53061 -15.0917 6.23611 1.85321
0.0106 0.00167 0.000614 0.000951 0.000419 0.00136 0.00914 0.00148 0.01
3L:12794640-12794790:minus -20.8878 3.59633 3.77982 3.14679 -27.3367 -9.86735 -5.54128 1.45833
3.42202 0.0018 0.00279 0.00293 0.00847 0.0317 0.00391 0.00116 0.0104 0.00597
3L:12794640-12794790:plus -31.5102 1.53211 -1.13761 5.49541 -18.7143 -27.3061 -14.5596 2.02778 4.83486
0.013 0.00596 0.0167 0.00355 0.0136 0.0336 0.00819 0.00853 0.00329
3L:12797620-12797770:minus -13 -0.40367 0.0183486 0.779817 -27.2347 -36.9286 -11.9083 -5.80556

12.2752 0.000563 0.0118 0.0115 0.0219 0.0283 0.123 0.00452 0.0731 0.0000141
3L:12797620-12797770:plus -22.0408 3.68367 0.385321 5.74312 -26.9694 -17.2347 -20.0642 -2.88889 -2.42202
0.00296 0.00144 0.0102 0.00302 0.023 0.00511 0.0208 0.0376 0.0378
3L:12798060-12798210:minus -30.7041 -0.926606 2.06422 2.81651 10.9184 -17.2041 -12.1101 -7.09722
-5.89908 0.00993 0.014 0.00564 0.00982 4.19e-06 0.00507 0.00472 0.0943 0.0869
3L:12798060-12798210:plus -31.8163 1.95413 -0.33945 1.26606 -26.2347 -0.173469 -17.1927 5.375
-3.05505 0.016 0.0051 0.0129 0.0191 0.0177 0.000456 0.0132 0.00222 0.0452
3L:12798940-12799090:minus -23.8571 -2.81651 -2.55963 5.30275 -18.7143 -18.051 -22.5321 4.75 -3.10092
0.00687 0.0245 0.0254 0.00396 0.0136 0.00886 0.0324 0.00293 0.0458
3L:12798940-12799090:plus -41.4388 2.38532 -1.24771 6.44037 10.0918 -0.244898 -16.4495 0.486111
9.88073 0.0562 0.00436 0.0172 0.00185 0.0000251 0.000496 0.0117 0.0144 0.000206
3L:12807320-12807470:minus -32 14.7339 -1.51376 3.77064 -18.4184 -10.0204 -9.92661 -0.0694444
-2.3211 0.0171 3.57e-06 0.0187 0.00637 0.0101 0.00399 0.00304 0.0171 0.0365
3L:12807320-12807470:plus -31.7041 -2.04587 -0.66055 2.88991 20.1735 0.683673 -15.7431 6.69444 -0.458716
0.0146 0.0197 0.0143 0.00942 5.68e-07 0.000184 0.0104 0.00118 0.0217
3L:12808040-12808190:minus -13.7755 -0.321101 4.98165 7.37615 -8.97959 -18.7857 -15.6606 -4.22222
3.88991 0.00107 0.0115 0.00179 0.000951 0.0023 0.0146 0.0102 0.0518 0.00467
3L:12808040-12808190:plus -32.4388 1.85321 6.49541 1.75229 0.540816 -19.5714 -8.62385 -1.13889
0.00917431 0.0195 0.0053 0.000921 0.0159 0.000321 0.0202 0.00238 0.0235 0.0184
3L:12824260-12824410:minus -22.1837 2.73394 -1.44037 4.08257 -18.7857 -8.53061 -13.0275 1.13889
0.66055 0.00315 0.00383 0.0183 0.00558 0.0148 0.00136 0.00582 0.0116 0.0156
3L:12824260-12824410:plus -33.7041 0.697248 4.16514 6.84404 -18.7857 -7.72449 -30.2936 5.93056 -4.81651
0.0293 0.00807 0.00251 0.0014 0.0148 0.00068 0.0947 0.00171 0.0697
3L:12827760-12827910:minus -13.1531 -1.16514 2.50459 5.43119 -26.5306 -8.72449 -18.7523 0.25 5.74312
0.00067 0.0151 0.0048 0.00371 0.0185 0.00169 0.0168 0.0155 0.00215
3L:12827760-12827910:plus -24.5204 1.16514 1.26606 7.00917 -18.449 -0.397959 -12.7248 1.47222
-2.2844 0.00813 0.00682 0.00753 0.00126 0.0107 0.000525 0.00543 0.0104 0.0361
3L:12836140-12836290:minus 7.70408 2.10092 3.26606 11.0275 -26.9694 -8.45918 5.92661 2.43056
6.63303 1.4e-06 0.00484 0.00358 0.0000401 0.023 0.00111 0.0000424 0.00738 0.00149
3L:12836140-12836290:plus -22.2245 1.6422 2.6055 -0.724771 -18.1531 -27.4898 -11.7156 5.375
9.61468 0.00325 0.00573 0.00462 0.0338 0.00829 0.0363 0.00433 0.00222 0.00029
3L:12836440-12836590:minus -22.2143 2.10092 1.55046 0.0917431 -18.0816 1.31633 -18.1284 2.43056
6.63303 0.00317 0.00484 0.0068 0.0262 0.00759 0.000066 0.0152 0.00738 0.00149
3L:12836440-12836590:plus -24.5612 -3.27523 -1.56881 7 -26.9694 1.02041 -20.5872 0.430556 1.22018
0.00819 0.0278 0.019 0.00132 0.023 0.000118 0.0228 0.0146 0.0133
3L:12837280-12837430:minus -23.7857 -1.89908 1.25688 4.65138 -19.0102 -9.86735 -18.4587 2.09722
8.41284 0.00679 0.0189 0.00755 0.00471 0.0157 0.00391 0.016 0.00832 0.000519
3L:12837280-12837430:plus -31.9694 2.45872 -0.587156 3.70642 -27.1633 -8.42857 -10.2936 3.56944
-2.69725 0.0168 0.00424 0.014 0.0066 0.025 0.00104 0.00325 0.00478 0.0411
3L:12843060-12843210:minus -30.5102 -1.40367 0 5.86239 -17.1122 0.826531 -15.6514 2.22222 -2.2844
0.00947 0.0163 0.0116 0.00282 0.00341 0.000153 0.0102 0.00796 0.0361
3L:12843060-12843210:plus -32.4388 0.990826 13.1651 9.3211 -7.63265 -9.23469 -18.3578 3.72222 -4.76147
0.0195 0.00726 0.0000173 0.000391 0.000479 0.00238 0.0158 0.0045 0.0686
3L:12885580-12885730:minus -14.2653 1.43119 -1.07339 3.04587 -7.93878 -7.5 -26.3303 5.38889 -1.18349
0.00128 0.00619 0.0163 0.00883 0.000767 0.000614 0.0557 0.0022 0.0276
3L:12885580-12885730:plus -23.0306 0.798165 9.46789 11.2936 -18.1224 -26.9694 -14.5229 7.56944 2.74312
0.00505 0.00779 0.000194 0.0000139 0.00782 0.0304 0.00813 0.000743 0.00751
3L:12895880-12896030:minus -14.6633 3.68367 3.46789 7.33945 0.836735 -8.30612 -7.26606 -1.02778
-4.34862 0.0015 0.00144 0.00331 0.00102 0.000215 0.000973 0.00179 0.0227 0.0612
3L:12895880-12896030:plus -23.5612 3.64286 14.9633 7.29358 -28.1939 -25.602 -13.2202 0.388889 -2.17431
0.00653 0.00159 1.77e-06 0.00104 0.0482 0.0207 0.00608 0.0148 0.035
3L:12907060-12907210:minus -41.102 2.29358 -0.568807 4.58716 -9.5 -18.2755 -22.3028 0.458333
1.68807 0.0472 0.00451 0.0139 0.00479 0.00293 0.0112 0.0311 0.0145 0.0109
3L:12907060-12907210:plus -22.1837 7.38532 -3.57798 3.33945 -27.1224 -18.1939 -7.88991 -4.08333 10.3394

0.00315 0.000529 0.0339 0.00774 0.0245 0.0101 0.00206 0.0502 0.000112
3L:129320-129470:minus -23.1122 -0.853211 -1.49541 1.05505 -16.4898 -8.57143 -15.1009 5.45833
10.3486 0.0053 0.0137 0.0186 0.0204 0.00312 0.00149 0.00916 0.00214 0.0000988
3L:129320-129470:plus -4.37755 0.908257 0.963303 8.05505 -17.4184 -17.7857 -11.0642 -3.94444 -0.743119
0.000144 0.00748 0.00837 0.000578 0.00457 0.0068 0.00378 0.0486 0.0243
3L:1295780-1295930:minus -32.4796 2.65138 0.972477 9.08257 -17.7143 -9.45918 -25.1743 5.40278 3.46789
0.02 0.00395 0.00835 0.000444 0.00627 0.00279 0.0482 0.00219 0.00582
3L:1295780-1295930:plus -22.4694 -0.844037 5.09174 9.51376 -8.64286 -18.051 -15.578 8.25 -0.183486
0.0035 0.0136 0.00171 0.000292 0.00139 0.00886 0.01 0.000505 0.0197
3L:1298580-1298730:minus -31 -1.11009 0.33945 2.11009 -27.5714 -16.2755 -23.6881 0.625 5.81651
0.0105 0.0148 0.0104 0.0136 0.0357 0.00413 0.0392 0.0137 0.00204
3L:1298580-1298730:plus -13.0306 4.55963 0.926606 9.49541 -17.4082 -9.53061 16.4128 0.666667 2.00917
0.000567 0.00194 0.00848 0.000308 0.00421 0.0031 8.4e-07 0.0135 0.00934
3L:12999760-12999910:minus -33.3367 14.633 -0.201835 6.11009 -26.9286 -0.204082 -13.2752
-1.56944 5.65138 0.0281 4.09e-06 0.0124 0.00233 0.0214 0.000463 0.00616 0.0265 0.00228
3L:12999760-12999910:plus -24.7857 13.844 1.88073 0.990826 -28.1633 -8.5 -16.4954 5.81944 -2.17431
0.00829 9.98e-06 0.00604 0.0206 0.0462 0.00126 0.0118 0.00181 0.035
3L:1300040-1300190:minus -22.8878 2.56881 6.30275 5.49541 -18.6837 -26.3367 -19.5046 0.0277778
1.78899 0.00441 0.00407 0.00101 0.00355 0.0126 0.0221 0.0189 0.0166 0.0103
3L:1300040-1300190:plus -22.9592 2.82569 5.89908 6.13761 -8.67347 -27.2653 -13.5505 -1.81944 3.31193
0.00475 0.00371 0.00121 0.00226 0.00154 0.0333 0.00656 0.0284 0.00618
3L:13001260-13001410:minus -22.8469 13.4592 -6.24771 3.33028 -17.7551 9.5 -25.6972 2.59722 2.25688
0.00424 0.000064 0.0672 0.00778 0.00658 0.0000337 0.0515 0.00694 0.00853
3L:13001260-13001410:plus -33.0408 9.51376 13.6422 7.29358 0.612245 -27.1224 -20.2477 5.875 1.56881
0.0264 0.000171 0.0000116 0.00104 0.000269 0.0325 0.0215 0.00176 0.0114
3L:13004680-13004830:minus -31.4694 3.83673 -1.14679 6.83486 -27.2653 -18.8265 -7.58716 4.59722
2.16514 0.0124 0.00115 0.0167 0.00142 0.0302 0.0148 0.00193 0.00313 0.00879
3L:13004680-13004830:plus -31.9694 -3.22936 -1.08257 -2.74312 -18.7143 -7.65306 -18.2294 0.888889 -0.174312
0.0168 0.0275 0.0164 0.0646 0.0136 0.000647 0.0155 0.0126 0.0196
3L:1300500-1300650:minus -39.9184 -0.0183486 -0.0275229 3.41284 -9.16327 -28.3367 -21.7064 0.277778
4.04587 0.0344 0.0104 0.0117 0.00756 0.00249 0.0606 0.028 0.0153 0.00421
3L:1300500-1300650:plus -33 1.27523 1.52294 4.44037 -17.3367 -9.82653 -18.2844 4.43056 -0.899083
0.0258 0.00655 0.00687 0.00493 0.00391 0.00381 0.0156 0.00336 0.0254
3L:13008300-13008450:minus -24 16.3761 6.31193 4.13761 -8.16327 -8.57143 -12.2385 1.13889 -0.651376
0.00719 1.59e-07 0.001 0.00546 0.000873 0.00149 0.00486 0.0116 0.0235
3L:13008300-13008450:plus -14.6327 13.4592 4.56881 7.43119 -18.9796 -9.53061 -15.1193 5.16667 -0.697248
0.0015 0.000064 0.00213 0.000939 0.0155 0.0031 0.00919 0.00244 0.0239
3L:13009120-13009270:minus -22.398 3.7156 3.97248 2.09174 -18.4898 -18.0816 -17.8073 -1.63889
-0.394495 0.00344 0.00267 0.00271 0.0137 0.0116 0.00934 0.0145 0.027 0.0212
3L:13009120-13009270:plus -12.8163 3.10092 2.72477 6.49541 10.6531 -18.1939 -19.9725 0.347222 1.83486
0.000489 0.00335 0.00441 0.00176 0.0000102 0.0101 0.0205 0.015 0.0101
3L:13011000-13011150:minus -22.1531 13.4592 -3.16514 5.47706 -18.9796 -18.6429 -16.2569 8.80556
0.669725 0.0031 0.000064 0.0302 0.0036 0.0155 0.0139 0.0113 0.000362 0.0155
3L:13011000-13011150:plus -31.7755 9.07339 5.94495 2.19266 -18.6429 -18.1224 -15.3394 0.236111 -2.27523
0.0157 0.000215 0.00118 0.0131 0.0121 0.00988 0.0096 0.0155 0.036
3L:13011280-13011430:minus -23.9286 9.6422 -2.88991 2.66055 -9.23469 -10.0204 -18.4037 3.75 -2.22936
0.00701 0.00016 0.028 0.0106 0.00277 0.00399 0.0159 0.00445 0.0356
3L:13011280-13011430:plus -31.7755 9.07339 1.13761 2.22018 -18.6429 0.540816 -15.3394 -1.36111 -2.27523
0.0157 0.000215 0.00788 0.0129 0.0121 0.0002 0.0096 0.025 0.036
3L:13015660-13015810:minus -13.5102 0.247706 6.10092 3.19266 10.3878 -36.1531 -12.1927 -1.54167
1.85321 0.000947 0.00947 0.0011 0.00829 0.0000174 0.0908 0.00481 0.0263 0.01
3L:13015660-13015810:plus -32.6224 0.284404 4.68807 3.78899 1.17347 -18.1224 -7.73394 0.819444 9.45872
0.0211 0.00935 0.00203 0.00632 0.000101 0.00988 0.00199 0.0129 0.000337
3L:13016400-13016550:minus -4.40816 15.2202 1.10092 3.94495 -18.7143 -18.0102 -15 5.15278 -2.01835

0.000148 1.59e-06 0.00798 0.00595 0.0136 0.00812 0.00897 0.00245 0.0334
3L:13016400-13016550:plus -23.2959 1.29358 6.55963 3.76147 -18.7551 -8.65306 -10.3761 -3.18056 -2.69725
0.00605 0.00651 0.000892 0.00645 0.0144 0.00156 0.0033 0.0404 0.0411
3L:1302800-1302950:minus -33 -5.10092 5.04587 4.34862 20.4694 9.30612 -21.6055 -1.40278 -2.06422
0.0258 0.0453 0.00174 0.00507 1.65e-07 0.0000398 0.0275 0.0253 0.0339
3L:1302800-1302950:plus -40.1429 3.59633 2.30275 5.19266 11.4286 -8.79592 -31.1927 0.347222 -0.119266
0.0363 0.00279 0.00517 0.00422 1.97e-06 0.0019 0.107 0.015 0.0192
3L:13029940-13030090:minus -31.2143 -1.29358 2.49541 2.47706 -27.9694 -27.1939 -20.5963 1.02778
-0.669725 0.0111 0.0157 0.00481 0.0115 0.0437 0.0327 0.0229 0.012 0.0236
3L:13029940-13030090:plus -21.4388 6.52294 -2.50459 -0.495413 -26.3061 -7.94898 -17.9266 -1.18056
1.9633 0.002 0.000826 0.025 0.0311 0.0181 0.00074 0.0148 0.0238 0.00951
3L:13034820-13034970:minus -32.7347 -0.137615 -2.29358 2.6789 -17.1531 -9.7551 -24.2477 6.5
-3.10092 0.0225 0.0108 0.0236 0.0105 0.00364 0.00358 0.0426 0.0013 0.0458
3L:13034820-13034970:plus -22.4286 -5.72477 6.17431 2.3945 1.10204 -6.72449 -15.9174 0.180556 -3.31193
0.00345 0.0532 0.00107 0.0119 0.000124 0.000544 0.0107 0.0158 0.0477
3L:13037780-13037930:minus -30.4388 -4.6789 2.33028 1.20183 -17.8571 -8.65306 -10.3853 -1.06944
6.54128 0.00923 0.0405 0.00511 0.0195 0.00698 0.00156 0.00331 0.023 0.00151
3L:13037780-13037930:plus -22.9592 -0.247706 3.3945 6.57798 -18.8265 -8.27551 -17.6514 -2.27778
-0.0733945 0.00475 0.0112 0.00341 0.00167 0.0149 0.000919 0.0142 0.0321 0.0188
3L:13038760-13038910:minus -21.9286 3.64286 -1.55046 3.45872 -26.898 -7.53061 -16.8624 4.79167
5.49541 0.0027 0.00159 0.0189 0.00731 0.0203 0.000625 0.0125 0.00288 0.0025
3L:13038760-13038910:plus -23.3776 4.68807 -0.880734 3.51376 -18.7143 -18.051 -14.4954 0.541667
-4.11009 0.00619 0.00184 0.0154 0.00709 0.0136 0.00886 0.00808 0.0141 0.0571
3L:13039980-13040130:minus -38.6939 2.73394 -0.174312 3.79817 -17.1122 -19.6429 -13.422 5.84722
3.98165 0.0321 0.00383 0.0123 0.00629 0.00341 0.0204 0.00637 0.00178 0.00439
3L:13039980-13040130:plus -24 13.3878 -2.34862 2.33945 -18.7551 -8.65306 -13.6972 3.68056 1.11009
0.00719 0.0000869 0.0239 0.0122 0.0144 0.00156 0.00678 0.00457 0.0138
3L:1307140-1307290:minus -22.4388 7.36697 5.66972 3.29358 -18.1939 -18.0816 -19.1468 7.43056 7.6422
0.00348 0.000535 0.00134 0.00791 0.0086 0.00934 0.0179 0.000801 0.000893
3L:1307140-1307290:plus -29.9184 2.44037 -3.6055 7.15596 -18.1224 -8.42857 -10.0459 -1.45833 5.53211
0.00854 0.00427 0.0341 0.00117 0.00782 0.00104 0.0031 0.0257 0.00245
3L:1307360-1307510:minus -21.6224 5.22018 4.3211 7.45872 -18.1224 -9.30612 -15.0092 3.59722 -0.348624
0.00213 0.00149 0.00235 0.000894 0.00782 0.00257 0.00899 0.00473 0.0209
3L:1307360-1307510:plus -22.6224 6.95413 4.52294 4.22018 -19.0918 -7.5 -4.70642 -2.81944 1.22936
0.00371 0.000665 0.00217 0.00531 0.0163 0.000614 0.000913 0.0369 0.0132
3L:13107100-13107250:minus -33.398 -1.17431 0.0642202 -0.559633 -25.8571 -26.898 -10.1743
7.33333 3.40367 0.0282 0.0151 0.0113 0.0318 0.0166 0.0302 0.00318 0.000844 0.00603
3L:13107100-13107250:plus 15.2143 7.33945 2.24771 3.97248 -18.4898 -8.53061 -7.79817 -2.08333 2.00917
3.74e-07 0.000544 0.00527 0.00587 0.0116 0.00136 0.00202 0.0305 0.00934
3L:1313200-1313350:minus -31.3673 -3.30275 6.99083 7.72477 -17.7551 -18.0102 -14.1927 7.19444 -2.41284
0.0116 0.028 0.000731 0.000681 0.00658 0.00812 0.00757 0.000909 0.0376
3L:1313200-1313350:plus -42.0408 8.65138 1.22018 2.86239 -9.16327 -9.02041 -28.6972 3.875 -7.26606
0.069 0.000269 0.00765 0.00954 0.00249 0.00213 0.0757 0.00423 0.119
3L:1313360-1313510:minus -31.3673 -3.30275 6.99083 7.72477 -17.7551 -18.0102 -14.1927 7.19444 -2.41284
0.0116 0.028 0.000731 0.000681 0.00658 0.00812 0.00757 0.000909 0.0376
3L:1313360-1313510:plus -42.0408 8.65138 1.22018 2.86239 -9.16327 -9.02041 -28.6972 3.875 -7.26606
0.069 0.000269 0.00765 0.00954 0.00249 0.00213 0.0757 0.00423 0.119
3L:1313680-1313830:minus -12.8469 2.27523 -0.183486 1.41284 -27.5306 -17.2755 -24.367 3.81944
0.330275 0.000502 0.00454 0.0123 0.0183 0.0351 0.00544 0.0433 0.00433 0.0168
3L:1313680-1313830:plus -23.1837 -0.394495 6.50459 6.73394 -18.4592 -8.42857 -15.6055 0.583333
-3.88991 0.00553 0.0118 0.000917 0.00152 0.0109 0.00104 0.0101 0.0139 0.054
3L:131520-131670:minus -14.3673 1.05505 -1.68807 11.2936 -25.9286 -17.9388 -3.78899 6.83333 11.8532
0.00132 0.00709 0.0197 0.0000139 0.0168 0.00738 0.000703 0.0011 0.0000428
3L:131520-131670:plus -13.6633 -0.706422 3.59633 7.16514 -17.8878 -18.5714 12.0367 -2.84722 -2.63303

0.00101 0.0131 0.00315 0.00116 0.00704 0.0136 5.54e-06 0.0372 0.0404
3L:1318280-1318430:minus -40.1837 0.761468 0.229358 1.36697 -18.7449 -18.7551 -22.4954 4.68056 -3.77982
0.0367 0.00789 0.0107 0.0186 0.0138 0.0141 0.0322 0.00302 0.0525
3L:1318280-1318430:plus -11.5918 4.10092 11.5688 9.44037 -27.1939 -18.8571 -17.156 -2.84722 1.44037
0.000257 0.00231 0.0000567 0.000339 0.0267 0.0151 0.0131 0.0372 0.012
3L:13221700-13221850:minus -24.2653 -4.56881 -2.58716 4.6422 -17.4898 -26.3367 -21.3394 2.65278
2.16514 0.00773 0.0394 0.0256 0.00473 0.0053 0.0221 0.0262 0.0068 0.00879
3L:13221700-13221850:plus -23.2653 1.11927 -2.76147 1.11009 0.836735 -26.8265 -14.633 0.736111 7.06422
0.00593 0.00693 0.027 0.0199 0.000215 0.0285 0.00832 0.0132 0.00131
3L:1323920-1324070:minus -33.551 -0.40367 3.6422 3.41284 -27.2347 -17.4184 -20.6147 1.19444 8.20183
0.0288 0.0118 0.00309 0.00756 0.0283 0.00585 0.0229 0.0114 0.000539
3L:1323920-1324070:plus -30.7449 -1.97248 0.440367 0.853211 -18.4184 -17.7551 -21.1284 0.75 -2.69725
0.0101 0.0193 0.01 0.0214 0.0101 0.00643 0.0252 0.0132 0.0411
3L:132400-132550:minus -30.5204 -2.54128 -3.21101 1.46789 -27.4898 -9.02041 -30.3028 5.30556 -2.78899
0.00951 0.0227 0.0306 0.0179 0.0336 0.00213 0.0949 0.00229 0.0422
3L:132400-132550:plus -23.0306 5.33028 -3.59633 0.46789 -18.7857 -8.5 -15.5229 2.18056 9.45872 0.00505
0.00142 0.034 0.0239 0.0148 0.00126 0.00994 0.00808 0.000337
3L:1324340-1324490:minus -40.551 1.41284 5.44037 5.51376 -8.23469 -27.7041 -17.8624 -0.125 2.36697
0.0418 0.00623 0.00148 0.00348 0.000927 0.0445 0.0146 0.0174 0.00828
3L:1324340-1324490:plus -23.7857 -2.15596 -1.3945 5.85321 -17.1122 -18.3469 -7.33028 0.125 6.95413
0.00679 0.0203 0.018 0.00287 0.00341 0.0124 0.00182 0.0161 0.00136
3L:132900-133050:minus -13.2551 0.33945 -1.97248 3.62385 -9.0102 -8.7551 6.88073 7.01389 2.33028
0.000751 0.00917 0.0214 0.00673 0.00236 0.00172 0.0000321 0.000998 0.00836
3L:132900-133050:plus -33.2959 -4.18349 -0.623853 6.22936 -27.2653 -17.5714 -19.9908 4.59722 -2.31193
0.0279 0.0355 0.0142 0.00209 0.0302 0.00616 0.0206 0.00313 0.0363
3L:1329860-1330010:minus -14.8163 9.44037 7.54128 3.56881 -27.7041 -8.93878 -9.85321 2.77778 2.20183
0.00159 0.000178 0.000555 0.00699 0.038 0.00201 0.00299 0.00649 0.00869
3L:1329860-1330010:plus -38.9184 -3.57798 7.21101 2.23853 20.4694 -8.38776 -20.422 -3.36111
-0.59633 0.0323 0.0302 0.000655 0.0128 1.65e-07 0.00101 0.0222 0.0423 0.0229
3L:1331640-1331790:minus -32.0306 9.05505 -3.10092 3.59633 -28.2653 -8.72449 -5.93578
3.77778 1.95413 0.0172 0.000217 0.0297 0.00693 0.0537 0.00169 0.00129 0.0044 0.00964
3L:1331640-1331790:plus -31.2857 3.01835 0.770642 6.85321 0.877551 -18.1531 -13.7248
0.527778 1.90826 0.0113 0.00346 0.00895 0.00137 0.00019 0.00997 0.00682 0.0142 0.00974
3L:1333120-1333270:minus -21.2857 13.5306 -1.11009 3.54128 -18.7449 -28.1224 -9.21101
6.06944 4.83486 0.00191 0.0000206 0.0165 0.00702 0.0138 0.0583 0.00266 0.0016 0.00329
3L:1333120-1333270:plus -14.2551 13.3878 4.86239 3.6055 -18.449 -18.5612 -14.5688 4.72222
-0.724771 0.00127 0.0000869 0.00188 0.00686 0.0107 0.0132 0.00821 0.00296 0.0241
3L:1334740-1334890:minus -32.1735 7.02752 0.963303 7.45872 -18.4184 -0.0204082 -11.3211
0.708333 4.19266 0.018 0.000639 0.00837 0.000894 0.0101 0.000411 0.00399 0.0134 0.00401
3L:1334740-1334890:plus -30.1429 0.165138 4.41284 7.95413 11.6939 1.7551 -12.5413 2.08333
-3.99083 0.00866 0.00974 0.00227 0.000599 1.17e-06 0.0000519 0.00521 0.00837 0.0556
3L:13349060-13349210:minus -41.9694 1.45872 -1.18349 3.08257 -19.0204 0.979592 -29.8073
4.11111 -0.0733945 0.068 0.00613 0.0169 0.0087 0.0158 0.000136 0.0885 0.00384 0.0188
3L:13349060-13349210:plus -34.5612 -0.834862 -3.31193 1.84404 -17.4898 -9.86735
-30.3028 2.09722 -8.7156 0.032 0.0136 0.0315 0.0154 0.0053 0.00391 0.0949 0.00832 0.157
3L:13349480-13349630:minus -12.3776 -1.12844 13.6422 1.18349 -9.23469 -18.0102 -11.1743
-2.11111 -0.201835 0.000403 0.0149 0.0000116 0.0196 0.00277 0.00812 0.00387 0.0307 0.0199
3L:13349480-13349630:plus -14.5918 2.79817 6.22936 7.46789 -18.7857 -17.3469 -5.6422 -2.33333
3.20183 0.00148 0.00374 0.00104 0.000868 0.0148 0.0058 0.00119 0.0326 0.0065
3L:1335060-1335210:minus -30.2551 1.88991 2.53211 -0.504587 -8.89796 -36.1531 -7.88991
4.68056 4.45872 0.00885 0.00523 0.00475 0.0312 0.00189 0.0908 0.00206 0.00302 0.00366
3L:1335060-1335210:plus -22.9286 -1.33945 -0.513761 -0.908257 -26.3061 -17.051 -14.8716
5.09722 9.45872 0.0046 0.0159 0.0137 0.0357 0.0181 0.00479 0.00874 0.00251 0.000337
3L:1335460-1335610:minus -20.9286 0.100917 4.72477 5.90826 -18.051 -17.051 -9.50459 2

3.66972 0.00183 0.00996 0.00199 0.00271 0.00746 0.00479 0.00281 0.00862 0.00514
3L:1335460-1335610:plus -32.3673 -1.77064 2.22936 2.51376 -18.6429 -0.469388 -11.7064
1.26389 0.348624 0.0189 0.0182 0.00531 0.0113 0.0121 0.000533 0.00433 0.0111 0.0167
3L:1336280-1336430:minus -23.2959 0.844037 3.44954 3.30275 -18.449 -9.30612 1.21101 7.09722
-0.00917431 0.00605 0.00766 0.00334 0.00789 0.0107 0.00257 0.000208 0.000956 0.0184
3L:1336280-1336430:plus -31.4796 2.3945 -0.908257 4.16514 -18.5612 -9.02041 -16.5321
0.138889 1.49541 0.0128 0.00434 0.0155 0.00538 0.0119 0.00213 0.0119 0.016 0.0118
3L:1336840-1336990:minus -21.9286 -0.651376 0.192661 2.47706 -27.5306 -8.57143
-12.1101 6.86111 9.55046 0.0027 0.0128 0.0109 0.0115 0.0351 0.00149 0.00472 0.00108 0.000321
3L:1336840-1336990:plus -30.3265 -0.541284 0.302752 2.94495 -18.4898 -18.9388 -7.12844
-2.72222 1.9633 0.0089 0.0124 0.0105 0.0092 0.0116 0.0154 0.00174 0.036 0.00951
3L:13383080-13383230:minus -23.4796 5.76147 -2.16514 5.43119 10.6531 -18.7857 -16.5596
1.44444 -3.07339 0.00634 0.00118 0.0227 0.00371 0.0000102 0.0146 0.0119 0.0105 0.0454
3L:13383080-13383230:plus -41.2551 6.83486 9.91743 2.33028 -18.4184 -18.3469 -15.5046
5.83333 0.972477 0.0513 0.000707 0.00015 0.0123 0.0101 0.0124 0.0099 0.00179 0.0144
3L:133960-134110:minus -22.9694 5.04587 0.908257 -4.57798 -27.5714 -18.1224 -28.4771
6.88889 3.7156 0.0048 0.0016 0.00853 0.0963 0.0357 0.00988 0.0734 0.00107 0.00502
3L:133960-134110:plus -2.40816 0.33945 0.697248 1.33028 -18.7551 -7.5 -14.9908 1.09722
5.3945 0.0000315 0.00917 0.00917 0.0188 0.0144 0.000614 0.00896 0.0118 0.00263
3L:13405840-13405990:minus -51.5204 2.30275 -2.25688 6.00917 -27.7551 0.530612 -26.5688
5.43056 3.51376 0.183 0.00449 0.0233 0.00255 0.0385 0.000203 0.0573 0.00216 0.00566
3L:13405840-13405990:plus -33.3673 11.3211 -3.77064 5.06422 -19.0918 0.122449 -12.5872
2.68056 -3.36697 0.0282 0.0000686 0.0357 0.00433 0.0163 0.000312 0.00526 0.00673 0.0482
3L:1342280-1342430:minus -23.2959 5.3945 5.70642 7.47706 -8.64286 9.53061 -19.9174 2.625 2.74312
0.00605 0.00138 0.00131 0.00085 0.00139 0.0000316 0.0203 0.00687 0.00751
3L:1342280-1342430:plus -22.5918 13.3878 8.54128 2.14679 -8.60204 -8.79592 -24.3945 6.05556 -2.6055
0.00365 0.0000869 0.000326 0.0133 0.00125 0.0019 0.0434 0.00161 0.04
3L:13443340-13443490:minus -30.7449 -0.220183 -1.40367 -1.76147 -17.449 -16.9796 -17.5688 -2.125
6.11927 0.0101 0.0111 0.0181 0.0471 0.00484 0.0044 0.014 0.0308 0.00178
3L:13443340-13443490:plus -14.4184 -1.17431 -2.3578 2.30275 -17.7551 -19.0816 -15.3761 0.458333 1.47706
0.00137 0.0151 0.024 0.0124 0.00658 0.017 0.00966 0.0145 0.0119
3L:13443720-13443870:minus -31.8469 -0.614679 -5.61468 1.84404 -27.1939 -19.3061 -10.2936 5.58333
1.27523 0.0162 0.0127 0.0576 0.0154 0.0267 0.0187 0.00325 0.00202 0.013
3L:13443720-13443870:plus -12.8061 -0.623853 1.65138 3.42202 10.9898 1.05102 -6.33028 -1.59722
2.11009 0.000481 0.0127 0.00656 0.00751 3.28e-06 0.000107 0.00143 0.0267 0.00898
3L:13473700-13473850:minus -21.7449 6.18349 15.7798 5.90826 -18.6837 -8.72449 -9.97248 0.5 8.95413
0.00236 0.000972 1.81e-07 0.00271 0.0126 0.00169 0.00306 0.0143 0.000458
3L:13473700-13473850:plus -30.7347 3.61224 -1.25688 2.47706 -17.3776 -18.2755 -12.4679 4.98611 -3.65138
0.01 0.00167 0.0173 0.0115 0.00419 0.0112 0.00512 0.00264 0.0512
3L:13479380-13479530:minus -12.6224 13.5306 3.30275 2.44954 -8.97959 -18.2755 -15.9725 1.47222
-0.807339 0.000453 0.0000206 0.00353 0.0116 0.0023 0.0112 0.0108 0.0104 0.0247
3L:13479380-13479530:plus -22.6633 13.3878 2.22018 0.642202 -8.86735 -17.9388 -24.7615 1.97222 2.42202
0.00384 0.0000869 0.00532 0.0227 0.00173 0.00738 0.0457 0.0087 0.00816
3L:13479740-13479890:minus -32.4082 0.651376 4.3945 5.46789 -27.5714 -17.4184 -18.7156 4.875
5.87156 0.0191 0.00821 0.00229 0.00362 0.0357 0.00585 0.0167 0.00277 0.00199
3L:13479740-13479890:plus -22.7653 10.6606 -2.3945 2.55046 -18.6837 -18.5714 -6.82569 0.388889 -2.59633
0.00405 0.0000954 0.0242 0.0112 0.0126 0.0136 0.00162 0.0148 0.0398
3L:13481800-13481950:minus -22.9592 -1.40367 -1.11927 2.09174 -7.82653 -18.2755 -18.3945 0.319444
-3.89908 0.00475 0.0163 0.0166 0.0137 0.00059 0.0112 0.0159 0.0151 0.0541
3L:13481800-13481950:plus -33.0408 6.52294 1.21101 7.15596 -8.86735 -9.23469 -15.7431 4.29167 -0.137615
0.0264 0.000826 0.00768 0.00117 0.00173 0.00238 0.0104 0.00356 0.0194
3L:13487680-13487830:minus -29.8776 -0.93578 3 6.3578 -18.1224 1.38776 -19.6606 -0.791667
-0.798165 0.00852 0.014 0.00397 0.00195 0.00782 0.0000559 0.0194 0.0212 0.0246
3L:13487680-13487830:plus -21.9592 -4.02752 -0.908257 9.33028 -17.3776 -17.7857 -20.3303 -0.888889

-1.57798 0.00279 0.0341 0.0155 0.000374 0.00419 0.0068 0.0218 0.0218 0.0299
3L:13488320-13488470:minus -22.9286 -0.733945 3.73394 6.72477 -16.7551 -8.65306 -10.4587 -3.02778
1.59633 0.0046 0.0132 0.00299 0.00154 0.00322 0.00156 0.00336 0.0389 0.0113
3L:13488320-13488470:plus -21.3265 3.68367 3.31193 2.23853 -17.7143 -16.0102 -14.4037 -0.513889
1.90826 0.00193 0.00144 0.00352 0.0128 0.00627 0.00406 0.00792 0.0196 0.00974
3L:13495720-13495870:minus -34.3776 1.90826 -0.201835 6.21101 -17.7143 0.0204082 -32.6147
1.80556 1.90826 0.0319 0.00519 0.0124 0.00213 0.00627 0.000377 0.127 0.00923 0.00974
3L:13495720-13495870:plus -13.1429 4.9633 1.36697 3.61468 -8.71429 -0.0510204 -3.38532 -1.18056
1.21101 0.00065 0.00165 0.00726 0.0068 0.00159 0.000429 0.000629 0.0238 0.0134
3L:13506240-13506390:minus -30.551 1.27523 3.24771 2.11927 -18.4898 -18.9796 -8.3945 5.06944
-1.80734 0.00959 0.00655 0.00361 0.0135 0.0116 0.0155 0.00228 0.00255 0.0316
3L:13506240-13506390:plus -31.8469 15.0183 4.44037 2.6422 -27.1939 -9.53061 -7.55046 5.22222 1.0367
0.0162 1.96e-06 0.00224 0.0107 0.0267 0.0031 0.00191 0.00238 0.0141
3L:1350640-1350790:minus -23.7041 6.6422 3.05505 5.21101 -18.449 -18.7857 -13.7523 -2.375 -5.11009
0.00669 0.000778 0.00389 0.0042 0.0107 0.0146 0.00686 0.0329 0.0744
3L:1350640-1350790:plus -34.1122 -1.31193 -2.33945 1.6422 -19.2755 -8.45918 -22.2294 -1.625 1.66972
0.0312 0.0158 0.0239 0.0166 0.0164 0.00111 0.0307 0.0269 0.011
3L:1352220-1352370:minus -32.2245 -0.926606 -1.00917 1.9633 -7.89796 -8.94898 -16.4587 2.34722
-1.6055 0.0183 0.014 0.016 0.0146 0.000678 0.00203 0.0117 0.00761 0.0302
3L:1352220-1352370:plus -31.6633 1.45872 -1.11927 3.88991 -18.1531 -17.0102 -18.367 5.19444 1.42202
0.0141 0.00613 0.0166 0.00608 0.00829 0.00453 0.0158 0.00241 0.0121
3L:1367000-1367150:minus -31.8571 -0.981651 2.27523 -1.48624 -18.1224 -18.9388 -21.8349 5.25 5.58716
0.0163 0.0143 0.00522 0.0431 0.00782 0.0154 0.0286 0.00235 0.00237
3L:1367000-1367150:plus -31.7755 -2.04587 0.238532 3.31193 -18.4898 -8.5 -11.8073 -0.5 -1.17431 0.0157
0.0197 0.0107 0.00786 0.0116 0.00126 0.00442 0.0195 0.0275
3L:1376060-1376210:minus -30.1837 -2.66055 0.990826 2.25688 -26.9694 -18.8571 -23.2752 -0.333333
1.08257 0.00873 0.0235 0.00829 0.0127 0.023 0.0151 0.0367 0.0185 0.0139
3L:1376060-1376210:plus -14.3776 2.00917 0.201835 4.22018 -18.6837 -0.0204082 -19.8807 -0.902778
-4.26606 0.00134 0.005 0.0108 0.00531 0.0126 0.000411 0.0202 0.0219 0.0595
3L:1378660-1378810:minus -21.8878 0.321101 2.06422 9.59633 -27.5714 -18.1224 -11.7798 3.66667 4.22936
0.00258 0.00922 0.00564 0.000275 0.0357 0.00988 0.00439 0.0046 0.00395
3L:1378660-1378810:plus -32.1122 8.55963 3.33028 -0.0642202 -27.0408 -9.79592 -15.7615 -0.180556
3.56881 0.0178 0.000283 0.00349 0.0274 0.0242 0.00377 0.0104 0.0177 0.00543
3L:13855760-13855910:minus -22.3367 3.62385 1.68807 4.37615 -18.2245 -8.23469 -14.8257 4.30556
1.05505 0.00339 0.00276 0.00648 0.00504 0.00877 0.000875 0.00866 0.00354 0.014
3L:13855760-13855910:plus -14.3367 0.220183 0.229358 6.57798 -17.1531 -17.3469 -13.4037 1.18056 -1.59633
0.00131 0.00956 0.0107 0.00167 0.00364 0.0058 0.00634 0.0114 0.0301
3L:13910840-13910990:minus -13.8163 -3.99083 -0.110092 9.23853 -17.7143 -9.23469 -15.2294 0.666667
-1.78899 0.00109 0.0337 0.012 0.000424 0.00627 0.00238 0.00939 0.0135 0.0314
3L:13910840-13910990:plus -23.1531 3.85321 -2.94495 3.61468 -18.7143 -8.42857 -22.1651 1.93056 0.0275229
0.00542 0.00254 0.0284 0.0068 0.0136 0.00104 0.0304 0.00883 0.0183
3L:13911280-13911430:minus -31.2857 -3.34862 11.9633 5.49541 -27.6327 -25.898 -12.7798 5.59722
9.45872 0.0113 0.0284 0.0000442 0.00355 0.0369 0.0214 0.0055 0.002 0.000337
3L:13911280-13911430:plus -22.1531 -1.00917 0.0917431 7.00917 -28.6429 -8.94898 -17.5688 0.319444
5.24771 0.0031 0.0144 0.0112 0.00126 0.0631 0.00203 0.014 0.0151 0.00282
3L:13913080-13913230:minus -13.7755 1.14679 6.44954 4.3945 -17.3776 -18.4898 -15.3028 -0.861111
5.3211 0.00107 0.00686 0.000941 0.00499 0.00419 0.0129 0.00953 0.0217 0.00275
3L:13913080-13913230:plus -22.0408 0.761468 -0.412844 7.17431 -17.6429 -17.9796 -9.21101 1.16667
2.42202 0.00296 0.00789 0.0133 0.00115 0.00562 0.00764 0.00266 0.0115 0.00816
3L:13913660-13913810:minus -31.5918 0.550459 3.94495 7 -18.8265 -16.5 -10.6422 3.77778 8.88073
0.0136 0.00851 0.00274 0.00132 0.0149 0.00416 0.00348 0.0044 0.000468
3L:13913660-13913810:plus -32.7347 6.01835 3.50459 5.73394 -9.16327 18.8571 -21.1927 6.48611 -0.697248
0.0225 0.00105 0.00326 0.00307 0.00249 1.47e-06 0.0255 0.00131 0.0239
3L:13957780-13957930:minus -14.1122 0.53211 7.44954 4.17431 0.877551 -9.42857 -7.70642 -5.98611

9.56881 0.00119 0.00856 0.000581 0.00537 0.00019 0.0027 0.00198 0.0759 0.000303
3L:13957780-13957930:plus -30.7449 6.54128 3.58716 2.6789 -18.7143 -17.7857 -20.1468 1.91667 -3.76147
0.0101 0.000818 0.00316 0.0105 0.0136 0.0068 0.0211 0.00888 0.0523
3L:13958260-13958410:minus -41.8878 -1.37615 -1.55963 1.46789 -18.9796 -10.0204 -19.7523 2.55556
-5.99083 0.0672 0.0161 0.0189 0.0179 0.0155 0.00399 0.0197 0.00705 0.0886
3L:13958260-13958410:plus -22.8469 4.14679 7.25688 6.02752 -8.71429 -7.79592 -8.66972 -4.65278 5.90826
0.00424 0.00228 0.000641 0.00246 0.00159 0.000723 0.0024 0.0571 0.00198
3L:13987000-13987150:minus -23 0.0275229 0.899083 -0.0458716 -18.2245 -8.65306 -17.3394 -1.59722
0.220183 0.00498 0.0102 0.00856 0.0271 0.00877 0.00156 0.0135 0.0267 0.0174
3L:13987000-13987150:plus -14.7857 5.57798 4.57798 4.40367 -18.6735 -17.7857 -13.8624 5.63889 -0.53211
0.00158 0.00128 0.00212 0.00497 0.0123 0.0068 0.00704 0.00196 0.0222
3L:13987260-13987410:minus -24.449 13.4592 0.825688 2.77064 -28.5306 -8.68367 -22.4495 4.33333
-0.100917 0.00802 0.000064 0.00878 0.01 0.061 0.00157 0.0319 0.0035 0.019
3L:13987260-13987410:plus -14.5612 2.00917 4.57798 4.05505 -18.6735 -9.64286 -3.36697 -0.694444
1.61468 0.00147 0.005 0.00212 0.00566 0.0123 0.00339 0.000626 0.0206 0.0112
3L:13991700-13991850:minus -23.9592 3.3211 2.41284 3.26606 -8.63265 -36.1122 -8.52294 2.06944
5.75229 0.00708 0.00309 0.00496 0.00802 0.0013 0.0878 0.00234 0.00841 0.00213
3L:13991700-13991850:plus -23.1531 -2.88991 -1.02752 3.17431 -27.1939 -27.7857 -16.5596 0.527778 7.73394
0.00542 0.025 0.0161 0.00842 0.0267 0.0466 0.0119 0.0142 0.000784
3L:13992300-13992450:minus -23.1224 0.816514 -2.85321 2.87156 -8.37755 9.7551 -14.4954 1.29167
0.990826 0.00531 0.00773 0.0277 0.00948 0.00106 0.0000198 0.00808 0.011 0.0143
3L:13992300-13992450:plus -33.9286 1.82569 12.9817 7.29358 -27.5714 -9.42857 -24.5505 2.81944 -2.43119
0.0303 0.00535 0.0000211 0.00104 0.0357 0.0027 0.0444 0.00639 0.038
3L:13995460-13995610:minus -22.2653 2.30275 -4.55046 -1.11927 -8.89796 -16.9796 -10.2661 -1.5 -1.14679
0.00331 0.00449 0.044 0.0381 0.00189 0.0044 0.00324 0.026 0.0272
3L:13995460-13995610:plus -23.1531 0.788991 -2.2844 3.95413 -17.7551 -9.79592 -9.88991 3.36111 5.34862
0.00542 0.00781 0.0235 0.00593 0.00658 0.00377 0.00302 0.00519 0.00271
3L:13995960-13996110:minus -32.3265 -4.88991 -0.330275 1.99083 -18.0816 -8.42857 -26.8624 1.98611
-9.04587 0.0187 0.0429 0.0129 0.0144 0.00759 0.00104 0.0595 0.00866 0.167
3L:13995960-13996110:plus -13.5918 -0.431193 1.93578 5.63303 -8.93878 -17.2041 6.25688 -1.625
-2.52294 0.00099 0.0119 0.00592 0.00326 0.00216 0.00507 0.0000383 0.0269 0.0391
3L:13996720-13996870:minus -32.8061 -0.422018 7.33945 2.54128 -8.63265 -27.0408 -16.0917
-0.0277778 7.83486 0.0237 0.0119 0.000614 0.0112 0.0013 0.0307 0.011 0.0169 0.000712
3L:13996720-13996870:plus -30.3673 7.46789 -1.77982 7.34862 -17.449 0.826531 -28.8073 5.86111 -0.853211
0.00897 0.000506 0.0202 0.000998 0.00484 0.000153 0.0768 0.00177 0.0251
3L:13997240-13997390:minus -30.2143 -1.12844 -0.697248 0.0275229 -17.6735 -17.2755 -18.2752
5.11111 3.77064 0.0088 0.0149 0.0145 0.0267 0.00572 0.00544 0.0156 0.0025 0.00491
3L:13997240-13997390:plus -23.1837 0.46789 1.97248 3.88991 -18.7857 -8.72449 -17.1468 4.29167 -2.18349
0.00553 0.00876 0.00584 0.00608 0.0148 0.00169 0.0131 0.00356 0.0351
3L:13999180-13999330:minus -13.4082 6.90826 1.42202 2.9633 -18.7143 -0.0204082 -21.2385 -1.36111
-1.38532 0.000867 0.000681 0.00712 0.00914 0.0136 0.000411 0.0257 0.025 0.0288
3L:13999180-13999330:plus -30.7041 4.52294 3.21101 4.07339 -18.7143 -8.23469 -18.4587 -1.40278 3.24771
0.00993 0.00197 0.00366 0.00562 0.0136 0.000875 0.016 0.0253 0.00636
3L:13999900-14000050:minus -21.6224 5.66055 1.37615 3.76147 -18.7143 -7.5 -23.0092 0.236111 -6.59633
0.00213 0.00123 0.00724 0.00645 0.0136 0.000614 0.0351 0.0155 0.103
3L:13999900-14000050:plus -34.0714 13.4592 -0.146789 0.00917431 -17.7857 -8.72449 -22.4862 5.65278
-2.43119 0.031 0.000064 0.0122 0.0269 0.00676 0.00169 0.0321 0.00195 0.038
3L:14014240-14014390:minus -23.2245 0.366972 -1.76147 7.70642 -17.9286 -18.051 -14.1101 -2.58333
3.31193 0.00576 0.00908 0.0201 0.000708 0.0071 0.00886 0.00743 0.0348 0.00618
3L:14014240-14014390:plus -23.9592 4.59633 7.47706 6.33945 -17.4184 -8.72449 -20.0275 5.33333 -2.68807
0.00708 0.00191 0.000573 0.00196 0.00457 0.00169 0.0207 0.00226 0.041
3L:14021700-14021850:minus -23.0408 -7.9633 2.69725 1.97248 -8.37755 -27.6735 -13.6055 9.01389
9.30275 0.00512 0.0911 0.00446 0.0146 0.00106 0.0443 0.00664 0.000318 0.00038
3L:14021700-14021850:plus -23.449 1.94495 4.33028 2.90826 0.846939 -19.0102 -8.14679 -3.19444

-0.0733945 0.00631 0.00512 0.00235 0.00936 0.000197 0.0159 0.00217 0.0406 0.0188
3L:14022180-14022330:minus -32.7755 16.3761 2.30275 1.78899 -7.89796 -18.0102 -8.90826 0.958333
3.88073 0.0234 1.59e-07 0.00517 0.0158 0.000678 0.00812 0.00251 0.0123 0.00475
3L:14022180-14022330:plus -31.7041 2.00917 -1.82569 2.25688 -26.5306 -9.79592 -20.9725 4.02778 -5.59633
0.0146 0.005 0.0205 0.0127 0.0185 0.00377 0.0245 0.00397 0.0819
3L:14024680-14024830:minus -23.1837 8.48624 5.34862 7.61468 -27.2653 -9.53061 -20.6514 -0.291667
2.11927 0.00553 0.000293 0.00154 0.000779 0.0302 0.0031 0.0231 0.0183 0.00889
3L:14024680-14024830:plus -33.0714 11.7248 5.86239 4.04587 -17.7857 -17.4286 -13.8807 -4.54167 -4.44037
0.0267 0.0000542 0.00122 0.00572 0.00676 0.00586 0.00706 0.0557 0.063
3L:14025060-14025210:minus -41.7449 3.37615 -1.3945 -1.06422 -27.2653 0.683673 -22.3761 1.30556
-4.87156 0.0646 0.00303 0.018 0.0375 0.0302 0.000184 0.0315 0.011 0.0708
3L:14025060-14025210:plus -22.9184 7.15596 4.97248 1.53211 -18.4184 -17.0102 -13.055 -0.0555556
9.45872 0.00447 0.000599 0.0018 0.0174 0.0101 0.00453 0.00585 0.017 0.000337
3L:14030000-14030150:minus -23.4898 14.5046 -2.49541 3.33945 -9.27551 -9.79592 -29.3394 0.222222
-0.220183 0.00639 4.87e-06 0.025 0.00774 0.00284 0.00377 0.0828 0.0156 0.0201
3L:14030000-14030150:plus -13.449 9.97248 -0.899083 6.83486 -18.7551 -17.9388 -5.22018 3.83333
-3.09174 0.000914 0.000135 0.0155 0.00142 0.0144 0.00738 0.00106 0.0043 0.0456
3L:14030480-14030630:minus -33.6327 0.486239 0.211009 5.77982 -8.67347 -0.102041 -19 1.95833
11.4771 0.029 0.00871 0.0108 0.00294 0.00154 0.000443 0.0175 0.00875 0.0000679
3L:14030480-14030630:plus -32.8163 3.68367 1.93578 6.85321 0.806122 -18.2347 -16.4404 0 -1.41284 0.024
0.00144 0.00592 0.00137 0.000229 0.0104 0.0117 0.0167 0.0289
3L:14031420-14031570:minus -32.3367 -4.83486 4.69725 2.27523 -18.7245 -17.9796 -19.1835 2.76389
5.55963 0.0187 0.0422 0.00202 0.0125 0.0136 0.00764 0.018 0.00652 0.0024
3L:14031420-14031570:plus -23.7347 -1.12844 4.56881 -0.807339 -8.93878 -26.898 -9.11009 3.20833
3.36697 0.00673 0.0149 0.00213 0.0345 0.00216 0.0302 0.00261 0.00551 0.00607
3L:14044240-14044390:minus -24.1429 -0.330275 3.81651 5.57798 -18.1531 -19.0816 -17.1376 1.04167
0.816514 0.0074 0.0115 0.00289 0.0034 0.00829 0.017 0.0131 0.012 0.015
3L:14044240-14044390:plus -30.7755 2.98165 4.55046 -0.0642202 20.4694 -8.93878 -16.0275 2.68056
-0.192661 0.0102 0.0035 0.00214 0.0274 1.65e-07 0.00201 0.0109 0.00673 0.0198
3L:14046240-14046390:minus -22 -2.44954 6.72477 2.31193 2.21429 -9.5 -17.9174 3.88889 -2.49541
0.00291 0.0221 0.000825 0.0123 0.0000277 0.00294 0.0148 0.00421 0.0387
3L:14046240-14046390:plus -11.8571 -5.29358 -0.513761 7.77982 20.4694 -17.9796 -9.20183 -6.36111
1.40367 0.000281 0.0476 0.0137 0.000652 1.65e-07 0.00764 0.00266 0.0818 0.0122
3L:14049140-14049290:minus -14.5918 -0.33945 0.412844 6.41284 -18.3776 -27.8265 -19.0734 8.52778
4.44037 0.00148 0.0116 0.0101 0.00189 0.00926 0.0498 0.0177 0.000429 0.00367
3L:14049140-14049290:plus -31.4796 5.21101 1.29358 9.23853 -18.1531 -9.30612 -13.9083 8.15278 7.63303
0.0128 0.00149 0.00746 0.000424 0.00829 0.00257 0.00711 0.000535 0.000928
3L:14049920-14050070:minus -32.4796 8.51376 2.70642 3.11927 -18.7857 -17.7857 -13.8624 -2.41667
3.88991 0.02 0.000289 0.00444 0.00858 0.0148 0.0068 0.00704 0.0333 0.00467
3L:14049920-14050070:plus -21.6633 6.53211 0.330275 1.55963 -8.64286 -16.2041 -24.8165 -4.05556 -4.2844
0.00223 0.000822 0.0104 0.0173 0.00139 0.00408 0.046 0.0499 0.0597
3L:14051560-14051710:minus -21.7347 0.238532 2.23853 5.47706 -17.7857 -17.9388 -9.05505 4.22222
-0.100917 0.00235 0.0095 0.00529 0.0036 0.00676 0.00738 0.00258 0.00367 0.019
3L:14051560-14051710:plus -32.2959 5.74312 8.74312 1.43119 -17.5306 -7.38776 -13 5.69444 4.56881
0.0186 0.00119 0.000291 0.0181 0.0054 0.000563 0.00578 0.00191 0.00356
3L:14052420-14052570:minus -24.3265 0.247706 5.02752 5.43119 -19.051 -18.5714 -14.4312 6.73611
0.917431 0.00784 0.00947 0.00176 0.00371 0.0162 0.0136 0.00797 0.00115 0.0146
3L:14052420-14052570:plus -31.3673 3.59633 -3.06422 2.14679 -27.898 -17.3469 -14.8532 6.06944 10.3486
0.0116 0.00279 0.0294 0.0133 0.04 0.0058 0.00871 0.0016 0.0000988
3L:14054860-14055010:minus -34.0714 3.85321 5.6422 3.87156 -18.1837 -8.79592 -11.6422 1.73611
0.00917431 0.031 0.00254 0.00135 0.00618 0.00835 0.0019 0.00427 0.00946 0.0184
3L:14054860-14055010:plus -23.8469 -2.00917 5.6055 -0.201835 -18.1531 -17.7551 -6.90826 2.18056
1.06422 0.00685 0.0195 0.00137 0.0285 0.00829 0.00643 0.00165 0.00808 0.014
3L:14055620-14055770:minus -40.5918 -1.16514 8.68807 7.55963 -27.8265 -9.53061 -19.6697 7.05556

3.31193 0.0422 0.0151 0.0003 0.000807 0.0388 0.0031 0.0195 0.000977 0.00618
3L:14055620-14055770:plus -11.1122 0.972477 3.68807 5.65138 -25.8571 -9.65306 -19.8807 9.65278 -1.85321
0.000244 0.00731 0.00304 0.00322 0.0166 0.0034 0.0202 0.000209 0.032
3L:14056560-14056710:minus -24.2959 -0.238532 -1.17431 1.41284 -17.7551 -18.3469 -13.7431 8.44444
-2.41284 0.00781 0.0112 0.0169 0.0183 0.00658 0.0124 0.00685 0.000451 0.0376
3L:14056560-14056710:plus -39.2143 -2.14679 0.211009 6.18349 -17.3776 -18.051 -19.1101 4.09722 0.495413
0.0329 0.0203 0.0108 0.00217 0.00419 0.00886 0.0178 0.00386 0.0162
3L:14064860-14065010:minus -24.5612 -1 -3.94495 4.02752 -18.9796 -9.09184 -28.211 3.84722 -4.33028
0.00819 0.0143 0.0374 0.00581 0.0155 0.00219 0.0708 0.00428 0.0607
3L:14064860-14065010:plus -22.9592 0.211009 12.2752 7.45872 -27.6735 -26.9694 -13.2844 1.26389 7.42202
0.00475 0.00959 0.0000353 0.000894 0.0375 0.0304 0.00617 0.0111 0.00106
3L:1413360-1413510:minus -29.7755 4.0367 4.20183 3.78899 -8.53061 -26.4898 -9.77064 -5.81944 4.82569
0.0085 0.00237 0.00247 0.00632 0.0011 0.0225 0.00295 0.0734 0.00331
3L:1413360-1413510:plus -23.449 5.17431 3.38532 7.00917 -18.0816 -27.7857 -12.5229 -2.02778 1.25688
0.00631 0.00151 0.00342 0.00126 0.00759 0.0466 0.00518 0.03 0.0131
3L:14267260-14267410:minus -20.8163 0.779817 2.33945 2.6055 -7.56122 -19.2755 -10.1193 -3.80556
11.3853 0.00177 0.00784 0.0051 0.0109 0.000419 0.0185 0.00315 0.047 0.0000815
3L:14267260-14267410:plus -13.1122 -0.678899 2.14679 9.7156 -18.5306 -18.8571 -10.156 -2.90278
11.3853 0.000632 0.0129 0.00547 0.000242 0.0118 0.0151 0.00317 0.0377 0.0000815
3L:14402600-14402750:minus -32.8061 9 9.89908 3.90826 -18.449 -26.898 -17.4312 5.09722 -0.972477
0.0237 0.000224 0.000151 0.00603 0.0107 0.0302 0.0137 0.00251 0.0259
3L:14402600-14402750:plus -24.0714 0.688073 3.55963 6.54128 -18.4898 -17.5612 -20.7523 5 4.7156
0.00731 0.0081 0.0032 0.00173 0.0116 0.00604 0.0235 0.00263 0.00345
3L:14402920-14403070:minus -20.3673 1.77982 10.5688 7.90826 -17.0816 -9.16327 -9.98165 0.0277778
-0.623853 0.00166 0.00544 0.000101 0.000611 0.00334 0.00223 0.00307 0.0166 0.0232
3L:14402920-14403070:plus -13.0408 -1.77064 -0.486239 1.34862 -26.6735 -36.6735 5.98165 -7.25
11.3853 0.000576 0.0182 0.0136 0.0187 0.0191 0.118 0.0000417 0.097 0.0000815
3L:14403460-14403610:minus -22.6633 1.41284 7.54128 7.21101 -17.449 -18.7551 -21.789 0.305556
3.36697 0.00384 0.00623 0.000555 0.0011 0.00484 0.0141 0.0284 0.0152 0.00607
3L:14403460-14403610:plus -30.4388 -2.59633 5.06422 1.77982 -17.4184 -8.68367 -20.2936 1.16667 8.11009
0.00923 0.023 0.00173 0.0158 0.00457 0.00157 0.0217 0.0115 0.000597
3L:14420460-14420610:minus -4.03061 2.34862 3 9.44954 -18.449 -26.5612 -5.63303 -7.06944 7.6789
0.000121 0.00441 0.00397 0.000326 0.0107 0.0237 0.00119 0.0938 0.000866
3L:14420460-14420610:plus -19.8878 -1.05505 13.7431 9.92661 -17.3061 -17.7143 -2.63303 -1.06944 10.3486
0.00164 0.0146 0.0000103 0.000169 0.00389 0.00634 0.000517 0.023 0.0000988
3L:14530600-14530750:minus -42.8571 6.72477 0.853211 6.84404 -9.5 -8.79592 -29.1743 1.29167 -4.66055
0.0873 0.000747 0.0087 0.0014 0.00293 0.0019 0.0809 0.011 0.0667
3L:14530600-14530750:plus -31.1327 3.66972 -3.23853 5.90826 -18.4184 -27.3367 -7.29358 1.23611 0.238532
0.0107 0.00272 0.0308 0.00271 0.0101 0.0347 0.00181 0.0112 0.0173
3L:14541280-14541430:minus -22.3265 -0.486239 -2.27523 1.84404 -9.45918 -18.3061 -10.0183 1.47222
1.37615 0.00337 0.0121 0.0234 0.0154 0.00287 0.0114 0.00309 0.0104 0.0123
3L:14541280-14541430:plus -20.6224 6.88073 3.06422 5.92661 -27.2347 0.94898 -18.7706 1.63889 1.95413
0.0017 0.000691 0.00387 0.00266 0.0283 0.00014 0.0168 0.00978 0.00964
3L:14544080-14544230:minus 15.9592 -0.0183486 -3.48624 7.27523 -27.602 -9.68367 -2.85321 0.333333
3.31193 1.52e-07 0.0104 0.033 0.00109 0.0364 0.00342 0.000547 0.0151 0.00618
3L:14544080-14544230:plus -13.4082 0.0733945 -1.04587 -0.678899 -17.7143 -26.1224 1.85321 -0.236111
9.92661 0.000867 0.0101 0.0162 0.0332 0.00627 0.0217 0.000173 0.018 0.000193
3L:14552960-14553110:minus -13.7143 0.908257 1.3211 7.3578 -36.449 1.05102 -7.31193 4.20833
5.75229 0.00105 0.00748 0.00738 0.000965 0.0783 0.000107 0.00181 0.00369 0.00213
3L:14552960-14553110:plus -24.8571 10.1376 2.01835 3.79817 -18.7857 -17.7857 -15.4495 3.06944 -2.85321
0.00831 0.000125 0.00574 0.00629 0.0148 0.0068 0.0098 0.00581 0.0429
3L:14606620-14606770:minus -21.4388 5.27523 -1.81651 2.22018 -16.4184 -17.3061 -18.8624 6.97222
7.83486 0.002 0.00145 0.0205 0.0129 0.00304 0.00548 0.0171 0.00102 0.000712
3L:14606620-14606770:plus -24.5918 0.981651 4.04587 5.31193 -8.71429 -17.2755 -8.44954 0.166667 6.7156

0.00821 0.00729 0.00263 0.00393 0.00159 0.00544 0.0023 0.0159 0.00144
3L:14625080-14625230:minus -22.2551 9.81651 13.9174 5.46789 -18.4184 -25.8265 -9.12844 -4.65278
1.48624 0.00327 0.000147 8.08e-06 0.00362 0.0101 0.0212 0.00262 0.0571 0.0118
3L:14625080-14625230:plus -22.8571 11.3761 -2.0367 2.05505 -26.2653 -18.051 -23.2018 3.61111 0.926606
0.00427 0.0000665 0.0219 0.0139 0.018 0.00886 0.0363 0.0047 0.0146
3L:14625600-14625750:minus -34.4898 0.256881 -0.541284 -1.3211 -18.4796 1.2449 -26.2752 2.43056
-8.24771 0.0319 0.00944 0.0138 0.0407 0.011 0.0000797 0.0553 0.00738 0.142
3L:14625600-14625750:plus -31.1735 5.58716 1.23853 9.41284 -17.7143 -9.53061 -16.3211 2.72222 5.76147
0.0109 0.00127 0.0076 0.000354 0.00627 0.0031 0.0115 0.00663 0.0021
3L:146320-146470:minus -32.4082 1.19266 3.58716 2.19266 -19.0102 -9.72449 -26 4.73611 5.69725
0.0191 0.00675 0.00316 0.0131 0.0157 0.00352 0.0535 0.00295 0.00225
3L:146320-146470:plus -14.6327 14.4954 4.48624 2.62385 19.9082 -19.051 -10.7798 4.55556 6.17431
0.0015 5.09e-06 0.0022 0.0108 8.13e-07 0.0165 0.00357 0.00318 0.00171
3L:1463680-1463830:minus 16.1837 0.577982 3.04587 6.12844 -8.60204 -17.051 14.6972 -4.16667 4.99083
8.69e-08 0.00843 0.0039 0.00231 0.00125 0.00479 1.57e-06 0.0512 0.00311
3L:1463680-1463830:plus -12.4796 5.86239 6.7156 10.5872 -9.16327 -27.3367 -4.44037 10.0972 7.36697
0.000433 0.00113 0.000828 0.000115 0.00249 0.0347 0.000846 0.000153 0.00108
3L:14699700-14699850:minus -13.1939 4.93578 2.2844 3.94495 -27.6633 -17.051 -3.66972 6.05556
9.45872 0.000709 0.00167 0.0052 0.00595 0.0372 0.00479 0.00068 0.00161 0.000337
3L:14699700-14699850:plus 6.81633 0.431193 5.66055 11.0183 -17.4184 -17.7143 -3.99083 -6.33333 -3.3945
1.94e-06 0.00887 0.00134 0.000052 0.00457 0.00634 0.000744 0.0814 0.0484
3L:14748380-14748530:minus -31.2857 -2.7156 2.88991 4.31193 -28.3061 -16.9388 -17.1835 0.763889
8.88073 0.0113 0.0238 0.00414 0.00516 0.0553 0.00433 0.0132 0.0131 0.000468
3L:14748380-14748530:plus -14.4796 -1.66972 -0.155963 7.46789 -17.3469 -8.60204 -6.08257 -3.84722
-0.678899 0.00142 0.0176 0.0122 0.000868 0.00394 0.00151 0.00134 0.0475 0.0237
3L:14750480-14750630:minus -33.0714 1.3945 -0.0275229 9.59633 -36.5204 -8.23469 -5.88073 -0.986111
7.73394 0.0267 0.00627 0.0117 0.000275 0.0818 0.000875 0.00127 0.0225 0.000784
3L:14750480-14750630:plus -32.5408 -1.17431 -0.522936 5.24771 -17.449 -19.3061 -16.3486 8.20833
3.05505 0.0204 0.0151 0.0137 0.00403 0.00484 0.0187 0.0115 0.000518 0.00692
3L:14751120-14751270:minus -30.2551 3.22018 -3.27523 9.92661 -8.7449 -26.5612 -11.7798 -2.15278
5.22018 0.00885 0.00321 0.0311 0.000169 0.00161 0.0237 0.00439 0.0311 0.00289
3L:14751120-14751270:plus -32.449 1.74312 6.7156 2.93578 -17.4184 -7.79592 -12.9633 0.222222 7.88991
0.0195 0.00552 0.000828 0.00927 0.00457 0.000723 0.00573 0.0156 0.000688
3L:14752240-14752390:minus -22.9592 0.853211 0.00917431 6.12844 -16.8571 -27.5612 -25.0642 -3.31944
-1.81651 0.00475 0.00763 0.0116 0.00231 0.00324 0.0393 0.0476 0.0418 0.0317
3L:14752240-14752390:plus -32.7041 0.137615 0.238532 3.84404 -18.4184 -7.57143 -12.5046 3.20833 4.82569
0.0222 0.00984 0.0107 0.00624 0.0101 0.000644 0.00516 0.00551 0.00331
3L:14752880-14753030:minus -12.2653 -2.42202 1.94495 9.59633 -27.0408 9.64286 -14.1835 1.375
4.10092 0.000381 0.0219 0.0059 0.000275 0.0242 0.0000222 0.00755 0.0107 0.00413
3L:14752880-14753030:plus -40.2551 5.99083 -2.07339 5.47706 -18.9796 -8.79592 -22.2477 1.13889 4.73394
0.0382 0.00106 0.0221 0.0036 0.0155 0.0019 0.0308 0.0116 0.00342
3L:14753180-14753330:minus -13.4796 3.97959 4.10092 6.09174 -8.93878 -8.23469 -13.0183 0.708333
-4.51376 0.000935 0.000669 0.00257 0.00235 0.00216 0.000875 0.00581 0.0134 0.064
3L:14753180-14753330:plus -33 2.56881 10.1743 3.56881 -18.6837 -18.2755 -18.789 4.84722 -3.31193
0.0258 0.00407 0.000128 0.00699 0.0126 0.0112 0.0169 0.00281 0.0477
3L:14755000-14755150:minus -32.4694 -0.697248 4.26606 2.09174 -27.1939 -26.5612 -15.4862 5.02778
2.25688 0.0196 0.013 0.00241 0.0137 0.0267 0.0237 0.00987 0.00259 0.00853
3L:14755000-14755150:plus -13.3367 4.9633 2.6055 2.9633 -8.71429 0.122449 -17.9633 -1.20833 3.31193
0.0008 0.00165 0.00462 0.00914 0.00159 0.000312 0.0149 0.0239 0.00618
3L:14768800-14768950:minus -22.8878 -4.63303 8.04587 5.93578 -17.4898 -9.27551 -19.844 2.75 -4.87156
0.00441 0.04 0.000428 0.00264 0.0053 0.00244 0.02 0.00656 0.0708
3L:14768800-14768950:plus -31.551 -5.49541 -4.19266 2.13761 -17.3367 -18.4286 -20.3119 -0.847222
0.486239 0.0134 0.0502 0.04 0.0134 0.00391 0.0128 0.0217 0.0216 0.0163
3L:14769140-14769290:minus -32 1.31193 3.46789 1.84404 10.9184 -8.57143 -24.5505 2.69444 -0.761468

0.0171 0.00646 0.00331 0.0154 4.19e-06 0.00149 0.0444 0.00669 0.0244
3L:14769140-14769290:plus -23.1837 9.13761 1.77064 1.63303 -28.2347 -8.23469 -15.1468 6.30556 0.293578
0.00553 0.000208 0.00629 0.0167 0.051 0.000875 0.00924 0.00143 0.017
3L:14772540-14772690:minus -33.6939 1.11009 3.78899 5.29358 -27 -28.8571 -10.0917 0.680556 0.513761
0.0291 0.00695 0.00292 0.00398 0.0233 0.0699 0.00313 0.0135 0.0162
3L:14772540-14772690:plus -13.3367 0.293578 1.69725 6.41284 -27.4592 -18.051 -15.9817 1.23611 1.42202
0.0008 0.00932 0.00646 0.00189 0.0329 0.00886 0.0108 0.0112 0.0121
3L:14779500-14779650:minus -23.2245 3.19266 5.94495 5.69725 -26.9694 -27.3367 -16.5596 14.5278
9.87156 0.00576 0.00324 0.00118 0.00314 0.023 0.0347 0.0119 1.13e-06 0.000217
3L:14779500-14779650:plus -32.2653 13.4592 0.862385 4.58716 -25.9694 -17.2041 -21.0642 0.458333 -0.449541
0.0184 0.000064 0.00867 0.00479 0.017 0.00507 0.0249 0.0145 0.0216
3L:14783360-14783510:minus -32.7347 3.97959 1.95413 8.80734 -18.4184 -9.23469 -15.4037 6.13889
-0.862385 0.0225 0.000669 0.00588 0.000518 0.0101 0.00238 0.00972 0.00155 0.0251
3L:14783360-14783510:plus -22.3265 0.816514 9.3578 5.69725 -8.89796 -17.7143 -20.5505 -3.56944 -1.12844
0.00337 0.00773 0.000206 0.00314 0.00189 0.00634 0.0227 0.0445 0.0271
3L:14795220-14795370:minus -31.1429 0.908257 3.11927 3.04587 20.4694 -8.57143 -16.6147 4.38889
1.07339 0.0108 0.00748 0.00379 0.00883 1.65e-07 0.00149 0.012 0.00342 0.014
3L:14795220-14795370:plus -31.4796 2.40367 0.431193 5.34862 -17.449 -27.5306 -17.0092 6.27778 1.37615
0.0128 0.00433 0.01 0.00387 0.00484 0.0374 0.0128 0.00145 0.0123
3L:1495840-1495990:minus -22.9592 4.74312 6.23853 2.52294 -18.6429 -18.0102 -7.00917 8.97222 -0.284404
0.00475 0.00181 0.00104 0.0113 0.0121 0.00812 0.00169 0.000326 0.0207
3L:1495840-1495990:plus -23.3367 1.20183 -2.93578 3.21101 -17.8571 -17.051 -19.2569 3.80556 3
0.00614 0.00673 0.0283 0.00822 0.00698 0.00479 0.0182 0.00435 0.00702
3L:1496100-1496250:minus -31.7347 -0.238532 4.00917 3.51376 -17.1837 0.826531 -11.9908 2.23611
1.53211 0.0149 0.0112 0.00267 0.00709 0.00366 0.000153 0.0046 0.00792 0.0116
3L:1496100-1496250:plus -23.3367 1.57798 13.367 7.2844 -17.8571 -17.4898 -15.3119 0.875 3.08257
0.00614 0.00586 0.0000145 0.00105 0.00698 0.0059 0.00954 0.0127 0.00681
3L:14970720-14970870:minus -31.0714 1.59633 0.284404 2.66055 -8.67347 -28.3367 -8.55963 13.3889
-5.65138 0.0106 0.00582 0.0105 0.0106 0.00154 0.0606 0.00235 6.49e-06 0.0826
3L:14970720-14970870:plus -38.6531 2.74312 1.30275 1.40367 -17.1122 -27.9694 -18.0459 1.19444 7.22018
0.0321 0.00382 0.00743 0.0184 0.00341 0.0547 0.0151 0.0114 0.00119
3L:14975840-14975990:minus -39.2551 -1.04587 0 5.06422 -18.7143 -10.0204 -19.9541 2.29167 0.146789
0.0331 0.0145 0.0116 0.00433 0.0136 0.00399 0.0204 0.00776 0.0178
3L:14975840-14975990:plus -32.0714 11.4771 -0.247706 5.06422 -18.7857 0.826531 -16.3853 7.66667
3.61468 0.0176 0.0000625 0.0126 0.00433 0.0148 0.000153 0.0116 0.000704 0.00526
3L:14979220-14979370:minus -13.1837 -0.862385 -0.761468 5.31193 -17.2245 0.826531 -13.7248
1.88889 5.76147 0.000705 0.0137 0.0148 0.00393 0.00384 0.000153 0.00682 0.00896 0.0021
3L:14979220-14979370:plus -32.8469 3.68367 3.63303 2.83486 -19.051 -17.3469 -19.1376 11.375 1.95413
0.0243 0.00144 0.00311 0.0097 0.0162 0.0058 0.0178 0.0000552 0.00964
3L:14980120-14980270:minus -31.6327 2.42202 1.19266 2.09174 -8.86735 -8.23469 -20.844 3.81944
-2.94495 0.0138 0.0043 0.00773 0.0137 0.00173 0.000875 0.0239 0.00433 0.0441
3L:14980120-14980270:plus -31.3265 1.76147 6.6422 5.38532 -18.7551 -18.7857 -11.6514 4.36111 7.62385
0.0114 0.00548 0.000858 0.00382 0.0144 0.0146 0.00427 0.00346 0.000941
3L:14980720-14980870:minus -23.3776 0.972477 3.22018 9.7156 -27.0408 -8.38776 -19.0183 -3.84722
-6.16514 0.00619 0.00731 0.00365 0.000242 0.0242 0.00101 0.0175 0.0475 0.0926
3L:14980720-14980870:plus -32.8469 1.6055 -3.06422 5.37615 -8.89796 -17.8571 -13.5872 8.88889 3.46789
0.0243 0.0058 0.0294 0.00384 0.00189 0.00719 0.00661 0.000344 0.00582
3L:14992800-14992950:minus -40.0306 -0.889908 2.38532 1.3211 -19.051 0.0510204 -29.6789
-2.38889 1.22018 0.0357 0.0138 0.00501 0.0189 0.0162 0.000349 0.0869 0.0331 0.0133
3L:14992800-14992950:plus -32 13.5306 0.550459 4.77064 -18.3367 -10.0204 -21.0734 5.88889 -2.85321
0.0171 0.0000206 0.00965 0.00457 0.00885 0.00399 0.0249 0.00175 0.0429
3L:1499780-1499930:minus -14.7041 -2.06422 4.40367 5.93578 -26.9694 0.602041 -18.0917 5.26389 0.513761
0.00154 0.0198 0.00228 0.00264 0.023 0.000188 0.0152 0.00233 0.0162
3L:1499780-1499930:plus -32.0408 8.55963 5.18349 5.37615 -18.7857 -18.3469 -15.8349 8.68056 12.2752

0.0174 0.000283 0.00165 0.00384 0.0148 0.0124 0.0105 0.000391 0.0000141
3L:15001420-15001570:minus -22.1837 -0.93578 1.25688 3.17431 -26.8571 -18.051 -11.8073 0.111111
3.66972 0.00315 0.014 0.00755 0.00842 0.0198 0.00886 0.00442 0.0162 0.00514
3L:15001420-15001570:plus -22.1939 -0.779817 5.08257 6.84404 -7.71429 -9.23469 -18.1835 4.375
5.84404 0.00317 0.0134 0.00172 0.0014 0.000572 0.00238 0.0154 0.00344 0.00202
3L:15002020-15002170:minus -31.7755 3 7.49541 6.44037 -8.86735 -18.1531 -15.5872 -0.152778
0.247706 0.0157 0.00348 0.000568 0.00185 0.00173 0.00997 0.0101 0.0175 0.0172
3L:15002020-15002170:plus -31.1837 -2.16514 8.77064 2.66055 -17.3776 -27.051 -14.6239 -3.88889 -5.66055
0.0109 0.0204 0.000287 0.0106 0.00419 0.0315 0.0083 0.048 0.0827
3L:15002760-15002910:minus -33.551 -1.13761 0.724771 1.21101 -7.96939 -27.4082 -15.0642 6.73611
0.816514 0.0288 0.015 0.00909 0.0194 0.000785 0.0356 0.00909 0.00115 0.015
3L:15002760-15002910:plus -31.3673 0.844037 11.4404 6.36697 -17.8571 -27.6327 -19.1284 8.83333
-0.0825688 0.0116 0.00766 0.000061 0.00193 0.00698 0.043 0.0178 0.000356 0.0189
3L:15003500-15003650:minus -22.0408 -0.87156 1.59633 1.88991 -18.6837 -8.42857 -22.422 -3.69444
2.98165 0.00296 0.0138 0.00669 0.0151 0.0126 0.00104 0.0318 0.0458 0.00707
3L:15003500-15003650:plus -13.3367 0.541284 4.72477 7.62385 -18.7143 -18.5714 -10.6055 0.694444 -2.2844
0.0008 0.00854 0.00199 0.000759 0.0136 0.0136 0.00345 0.0134 0.0361
3L:15005080-15005230:minus -32.2857 8.73394 1.81651 6.49541 -17.3776 -7.79592 -25.7798 1.86111
2.74312 0.0185 0.000258 0.00619 0.00176 0.00419 0.000723 0.0521 0.00905 0.00751
3L:15005080-15005230:plus -21.3265 -1.05505 1.25688 0.0733945 -18.1939 -18.9388 -10.6514 -1.38889
1.07339 0.00193 0.0146 0.00755 0.0263 0.0086 0.0154 0.00348 0.0252 0.014
3L:15007060-15007210:minus -12.6633 -3.31193 4.54128 2.83486 10.6939 -26.7857 -8.26606 -0.236111
0.752294 0.000458 0.0281 0.00215 0.0097 7.35e-06 0.0266 0.00222 0.018 0.0152
3L:15007060-15007210:plus -31.1735 -5.21101 2.85321 1.86239 -8.97959 -18.2347 -18.5596 -0.25 4.91743
0.0109 0.0466 0.0042 0.0153 0.0023 0.0104 0.0163 0.0181 0.00321
3L:1502600-1502750:minus -22.8163 8.18349 -0.486239 4.04587 -18.7143 -17.051 -16.578 -3.38889
4.09174 0.00418 0.000345 0.0136 0.00572 0.0136 0.00479 0.012 0.0426 0.00416
3L:1502600-1502750:plus -30.398 6.72477 1.10092 7.43119 -17.449 -18.3469 3.42202 1.375 3.66972
0.00901 0.000747 0.00798 0.000939 0.00484 0.0124 0.000102 0.0107 0.00514
3L:15065260-15065410:minus -31.7041 4.38532 6.20183 10.7615 -17.1531 -17.8571 -8.87156 -5.77778
5.24771 0.0146 0.00207 0.00106 0.0000826 0.00364 0.00719 0.0025 0.0727 0.00282
3L:15065260-15065410:plus -41.6633 0.0642202 0.247706 -0.412844 -18.7449 -17.6429 -25.3945 1.19444
7.82569 0.0636 0.0101 0.0107 0.0304 0.0138 0.00619 0.0496 0.0114 0.000728
3L:15072540-15072690:minus -12.9592 -0.53211 5.3211 10.0275 -17.3776 -9.45918 -9.89908 3.125
-1.15596 0.000551 0.0123 0.00155 0.000156 0.00419 0.00279 0.00302 0.00569 0.0273
3L:15072540-15072690:plus -31.4082 2.2844 -0.678899 2.01835 -17.3061 -16.7143 -27.4587 6.02778
-0.12844 0.0119 0.00452 0.0144 0.0141 0.00389 0.00419 0.0641 0.00164 0.0193
3L:15072840-15072990:minus -31.4796 -4.17431 1.88991 3.00917 -17.7857 -26.602 -16.1743 4.31944
9.92661 0.0128 0.0354 0.00602 0.00894 0.00676 0.0248 0.0112 0.00352 0.000193
3L:15072840-15072990:plus -32.1531 -0.844037 -1.85321 -0.614679 -18.4184 -9.08163 -17.844 7.86111
6.54128 0.0179 0.0136 0.0207 0.0324 0.0101 0.00216 0.0146 0.000631 0.00151
3L:15077440-15077590:minus -32.2857 -0.522936 1.16514 6.62385 -17.4184 -26.7857 -16.1743 -3.13889
0.486239 0.0185 0.0123 0.0078 0.00163 0.00457 0.0266 0.0112 0.04 0.0163
3L:15077440-15077590:plus -21.6633 -1.25688 2.99083 3.18349 -8.70408 -18.6429 -12.3945 2.58333 -4.06422
0.00223 0.0155 0.00399 0.00837 0.00155 0.0139 0.00504 0.00698 0.0565
3L:15087320-15087470:minus -21.9694 -2.04587 3.36697 5.21101 0.806122 -9.42857 -8.00917 -0.0694444
5.38532 0.00282 0.0197 0.00345 0.0042 0.000229 0.0027 0.00211 0.0171 0.00267
3L:15087320-15087470:plus -22.6633 0.834862 4.3211 4.13761 -26.8571 -18.5612 -25.6147 -0.0694444
1.02752 0.00384 0.00769 0.00235 0.00546 0.0198 0.0132 0.051 0.0171 0.0141
3L:15087840-15087990:minus -13.4082 13.4592 -0.862385 6.6422 -28.1939 -0.173469 -23.6697
3.13889 2.6422 0.000867 0.000064 0.0153 0.00161 0.0482 0.000456 0.0391 0.00566 0.00768
3L:15087840-15087990:plus -13.6735 14.2294 1.44037 0.431193 -27.898 -18.9796 -16.3945 -0.347222
1.6422 0.00103 6.48e-06 0.00707 0.0241 0.04 0.0155 0.0116 0.0186 0.0111
3L:15091180-15091330:minus -12.5204 -1.02752 2.6055 4.33945 -26.9286 -19.3776 -23.5505 3.5 1.37615

0.000441 0.0145 0.00462 0.00509 0.0214 0.0195 0.0383 0.00491 0.0123
3L:15091180-15091330:plus -14.3367 -1.12844 -1.37615 2.14679 0.540816 -19.3469 -12.0642 8.76389 1.59633
0.00131 0.0149 0.0179 0.0133 0.000321 0.0194 0.00468 0.000371 0.0113
3L:15091680-15091830:minus -34.1429 4 6.66055 1.22018 -9.27551 -19.1531 -13.3394 1.66667 4.0367
0.0313 0.0024 0.000851 0.0194 0.00284 0.0177 0.00625 0.00969 0.00424
3L:15091680-15091830:plus -21.8163 -0.944954 1.6422 -1.6789 -18.7857 -27.3367 -19.9908 3.01389
12.2752 0.00244 0.0141 0.00659 0.0459 0.0148 0.0347 0.0206 0.00593 0.0000141
3L:15094840-15094990:minus -31.8163 1.6789 0.174312 -2.81651 -27.3367 -17.9796 -16.844 0.944444
9.56881 0.016 0.00565 0.0109 0.066 0.0317 0.00764 0.0125 0.0124 0.000303
3L:15094840-15094990:plus -31.6327 -5.27523 4.52294 3.45872 -27.5918 -9.5 -16.8899 -0.375 -4.87156
0.0138 0.0474 0.00217 0.00731 0.0358 0.00294 0.0126 0.0188 0.0708
3L:15098880-15099030:minus -21.3571 -2.83486 3.33945 -0.33945 -8.12245 -18.9796 -12.8073 -7.80556
-2.74312 0.00194 0.0246 0.00348 0.0296 0.000843 0.0155 0.00553 0.107 0.0417
3L:15098880-15099030:plus -24.2245 2.23853 7.2844 10.8624 -26.1531 -8.5 -21.1009 -2.91667 0.825688
0.00763 0.0046 0.000632 0.0000687 0.0172 0.00126 0.025 0.0378 0.0149
3L:15100600-15100750:minus -22.2653 3.16514 -0.247706 7.80734 -17.4184 0.244898 -6.44037 4.05556
-0.587156 0.00331 0.00327 0.0126 0.00063 0.00457 0.000285 0.00147 0.00393 0.0227
3L:15100600-15100750:plus -29.102 5.31193 -0.825688 0.486239 -27.8571 -16.3469 -24.2752 4.44444
-5.45872 0.00833 0.00143 0.0151 0.0237 0.039 0.00416 0.0427 0.00334 0.0799
3L:15101300-15101450:minus -12.9592 1.01835 0.678899 2.52294 -27.0816 -27.4898 -8.09174 -1.68056
7.73394 0.000551 0.00719 0.00923 0.0113 0.0243 0.0363 0.00214 0.0273 0.000784
3L:15101300-15101450:plus -40.102 3.36697 2.48624 1.40367 -17.9796 -18.2755 -13.8349 -6.40278 3.14679
0.036 0.00304 0.00483 0.0184 0.00726 0.0112 0.00699 0.0825 0.00666
3L:15102400-15102550:minus -22.0408 -2.11009 2.6789 2.54128 -27.4592 -17.2755 -16.9174 -0.861111
1.33028 0.00296 0.0201 0.00449 0.0112 0.0329 0.00544 0.0126 0.0217 0.0127
3L:15102400-15102550:plus -23.2959 8.30275 -0.385321 2.50459 -17.602 -19.0816 -2.83486 -2.34722
5.44037 0.00605 0.000324 0.0131 0.0114 0.00547 0.017 0.000544 0.0327 0.00259
3L:15109740-15109890:minus -41.6633 2.65138 0.00917431 4.02752 -28.8265 -0.469388 -20.8349
1.97222 3.88991 0.0636 0.00395 0.0116 0.00581 0.0648 0.000533 0.0239 0.0087 0.00467
3L:15109740-15109890:plus -23.2653 7.70642 -1.30275 7.3578 -18.6837 -8.53061 -21.7706 4.79167 1.23853
0.00593 0.000447 0.0175 0.000965 0.0126 0.00136 0.0283 0.00288 0.0132
3L:15129620-15129770:minus -22.6633 -1.15596 5.08257 2.91743 -17.0816 -8.79592 -17.4862 10.2917
-0.623853 0.00384 0.0151 0.00172 0.00932 0.00334 0.0019 0.0138 0.000132 0.0232
3L:15129620-15129770:plus -32.2143 4.55046 -1.21101 4.08257 -18.1531 -8.72449 -18.2385 4.26389 2.85321
0.0182 0.00195 0.017 0.00558 0.00829 0.00169 0.0155 0.0036 0.00733
3L:15129920-15130070:minus -30.7041 3.90816 2.98165 4.22936 -18.7143 -17.7857 -10.2569 -1.29167
0.697248 0.00993 0.00102 0.004 0.00525 0.0136 0.0068 0.00323 0.0245 0.0154
3L:15129920-15130070:plus -39.9898 2.65138 9.44037 7.29358 -27.1939 -16.7551 -17.4771 -0.888889
-1.15596 0.0351 0.00395 0.000197 0.00104 0.0267 0.0042 0.0138 0.0218 0.0273
3L:15130340-15130490:minus -41.7449 -1.59633 0.779817 1.84404 -17.7551 -7.72449 -32.5138 0.236111
-7.93578 0.0646 0.0172 0.00892 0.0154 0.00658 0.00068 0.125 0.0155 0.134
3L:15130340-15130490:plus -33.2653 13.4592 1.12844 1.18349 -27.5306 1.09184 -13.6606 0.611111 -2.15596
0.0277 0.000064 0.00791 0.0196 0.0351 0.000091 0.00672 0.0138 0.0348
3L:15130960-15131110:minus -23.9592 4.88073 12.844 11.0275 -26.898 -17.2755 -18.8349 2.34722
1.78899 0.00708 0.00171 0.0000237 0.0000401 0.0203 0.00544 0.017 0.00761 0.0103
3L:15130960-15131110:plus -31.102 -1.59633 7 6.68807 -17.8571 -9.5 -25.1193 -2.38889 0.137615 0.0106
0.0172 0.000727 0.00157 0.00698 0.00294 0.0479 0.0331 0.0178
3L:15133560-15133710:minus -32.3673 -1.0367 8.77982 7.90826 -18.1939 -26.7551 -17.9817 0.805556
2.11009 0.0189 0.0145 0.000286 0.000611 0.0086 0.0263 0.0149 0.013 0.00898
3L:15133560-15133710:plus -22.1122 -1.92661 2.63303 2.76147 -17.3469 -26.8265 -13.1376 4.48611 4.78899
0.00304 0.019 0.00457 0.01 0.00394 0.0285 0.00597 0.00328 0.00336
3L:15134820-15134970:minus -24.5612 -0.192661 4.33028 2.24771 -17.8265 0.826531 -16.1193 0.777778
-1.75229 0.00819 0.011 0.00235 0.0127 0.00691 0.000153 0.0111 0.0131 0.0312
3L:15134820-15134970:plus -31.5102 6.22936 1.78899 0.889908 -8.96939 -18.0816 -10.7064 5.77778 11.3853

0.013	0.000949	0.00625	0.0213	0.00226	0.00934	0.00352	0.00184	0.0000815
3L:15135680-15135830:minus	-31.4082	-3.44954	1.75229	6.84404	-17.4184	-16.3469	-19.4771	8.93056
-0.247706	0.0119	0.0292	0.00633	0.0014	0.00457	0.00416	0.0189	0.000335	0.0204
3L:15135680-15135830:plus	-21.6735	13.6514	0.495413	4.04587	-25.898	-8.34694	-3.11009	0.0972222
-0.642202	0.00224	0.0000126	0.00983	0.00572	0.0166	0.000993	0.000585	0.0162	0.0234
3L:15136820-15136970:minus	-12.7857	1.3578	2.77982	3.97248	-18.3878	-17.7551	-10.6422	4.44444
5.66055	0.000478	0.00635	0.00432	0.00587	0.00931	0.00643	0.00348	0.00334	0.00227
3L:15136820-15136970:plus	-31.5102	1.31193	9.0367	5.94495	-18.1531	-9.57143	-18.9908	0.583333	1.84404
0.013	0.00646	0.000249	0.00261	0.00829	0.00326	0.0174	0.0139	0.01
3L:15137280-15137430:minus	-13.0714	7.70642	1.27523	2.14679	-27.1939	-18.0102	-13.0826	0.388889
3.21101	0.000594	0.000447	0.0075	0.0133	0.0267	0.00812	0.00589	0.0148	0.00644
3L:15137280-15137430:plus	-22.4388	4.11927	1.22018	4.12844	-27.0408	-27.8265	-13.5596	4.98611	4.14679
0.00348	0.0023	0.00765	0.00549	0.0242	0.0498	0.00657	0.00264	0.00406
3L:15151600-15151750:minus	-21.6633	1.73394	-1.22018	4.37615	-9.16327	9.86735	-3.46789	2.11111
2.11009	0.00223	0.00553	0.0171	0.00504	0.00249	0.0000132	0.000643	0.00828	0.00898
3L:15151600-15151750:plus	-32.7347	3.7156	-1.33028	-0.715596	-27.1939	18.7857	-13.5688	5.36111
-0.431193	0.0225	0.00267	0.0177	0.0336	0.0267	1.6e-06	0.00658	0.00223	0.0214
3L:15154760-15154910:minus	-13.2653	1.78899	0.376147	2.95413	-18.4184	-18.0102	-13.422	3.47222
7.04587	0.000759	0.00542	0.0102	0.00917	0.0101	0.00812	0.00637	0.00497	0.00132
3L:15154760-15154910:plus	-33	1.01835	10.9725	7.45872	1.65306	-17.2755	-22.2385	4.68056	1.2844
0.0258	0.00719	0.0000812	0.000894	0.0000586	0.00544	0.0308	0.00302	0.0129
3L:15156620-15156770:minus	-33.7041	1.46789	11.2202	1.87156	-18.4184	-9.57143	-18.578	0.0972222
-4.6055	0.0293	0.0061	0.0000694	0.0152	0.0101	0.00326	0.0163	0.0162	0.0656
3L:15156620-15156770:plus	-32.4796	2.76147	3.97248	3.7156	-26.1939	-9.53061	-21.2477	4.04167	4.98165
0.02	0.0038	0.00271	0.00655	0.0175	0.0031	0.0257	0.00395	0.00313
3L:1516020-1516170:minus	-22.7041	-0.807339	7.77064	2.22018	-27.1939	-7.93878	-20.7615	1.01389
-2.2844	0.00395	0.0135	0.000494	0.0129	0.0267	0.000736	0.0236	0.0121	0.0361
3L:1516020-1516170:plus	-12.6633	1.42202	0.733945	-0.486239	-8.44898	-28.449	-19.4037	-2.36111
-2.2844	0.000458	0.00621	0.00906	0.031	0.00109	0.0616	0.0186	0.0328	0.0361
3L:1516560-1516710:minus	-23.1531	-1.79817	3.65138	2.93578	-18.3367	-8.68367	-13.7615	-0.791667
3.42202	0.00542	0.0183	0.00308	0.00927	0.00885	0.00157	0.00688	0.0212	0.00597
3L:1516560-1516710:plus	-30.1837	4.09174	4.80734	4.12844	-17.2245	-26.602	-21.8624	4.34722	3.78899
0.00873	0.00232	0.00193	0.00549	0.00384	0.0248	0.0288	0.00348	0.00487
3L:1517300-1517450:minus	-21.7755	3.15596	2.77982	2.0367	-17.3776	-9.5	-7.78899	1.90278	1.12844
0.00241	0.00328	0.00432	0.0141	0.00419	0.00294	0.00201	0.00892	0.0137
3L:1517300-1517450:plus	-22.8878	-3.27523	-2.21101	1.78899	-18.449	-18.0102	-6.49541	3.66667	7.46789
0.00441	0.0278	0.023	0.0158	0.0107	0.00812	0.00149	0.0046	0.00102
3L:1517540-1517690:minus	-24.1939	4.05505	-0.788991	5.06422	-27.2347	0.826531	-15.8807	1.08333
5.53211	0.00753	0.00235	0.0149	0.00433	0.0283	0.000153	0.0106	0.0118	0.00245
3L:1517540-1517690:plus	-23.7041	-3.02752	5.50459	2.11927	-8.89796	0.0510204	-23.3394	-0.944444
-4.44037	0.00669	0.026	0.00144	0.0135	0.00189	0.000349	0.0371	0.0222	0.063
3L:15222320-15222470:minus	-31.4082	6.75229	1.42202	5.49541	-17.3061	-17.9796	-8.59633	10.6528
-1.3578	0.0119	0.000736	0.00712	0.00355	0.00389	0.00764	0.00237	0.000101	0.0286
3L:15222320-15222470:plus	-23.1837	-1.33028	2.76147	7	-26.9694	-0.469388	-11.6147	-1.125	6.49541
0.00553	0.0159	0.00435	0.00132	0.023	0.000533	0.00424	0.0234	0.00154
3L:15223400-15223550:minus	-21.7041	7.10092	2.20183	-2.13761	-18.2245	-7.72449	-16.0459	3.90278
7.58716	0.00231	0.000615	0.00536	0.0535	0.00877	0.00068	0.0109	0.00418	0.000954
3L:15223400-15223550:plus	-13.2653	-2.47706	3.78899	6.31193	-18.5612	-18.0102	-15.6789	-5.01389	-0.972477
0.000759	0.0223	0.00292	0.00199	0.0119	0.00812	0.0102	0.0619	0.0259
3L:15223940-15224090:minus	-21.7041	1.47706	6.11927	6.3578	-18.4898	-8.16327	-25.0734	-1.11111
9.87156	0.00231	0.00608	0.00109	0.00195	0.0116	0.000783	0.0476	0.0233	0.000217
3L:15223940-15224090:plus	-4.03061	0.330275	7.63303	0.981651	-25.9694	-26.602	-5.45872	1.58333	5.23853
0.000121	0.00919	0.000531	0.0207	0.017	0.0248	0.00113	0.00997	0.00285
3L:15225120-15225270:minus	-24.7857	3.63303	9.22018	6.72477	-28.5612	-0.102041	-30.4312	-1.31944

-7.41284 0.00829 0.00276 0.000223 0.00154 0.0617 0.000443 0.0966 0.0247 0.122
3L:15225120-15225270:plus -32.4694 3.90816 -3.11009 0.46789 -18.449 -36.449 -8.63303 8.95833 1.06422
0.0196 0.00102 0.0297 0.0239 0.0107 0.109 0.00239 0.000329 0.014
3L:15227520-15227670:minus -14.1122 3.90816 6.27523 5.57798 -28.5306 -19.1224 -9.98165 2.81944
7.88991 0.00119 0.00102 0.00102 0.0034 0.061 0.0175 0.00307 0.00639 0.000688
3L:15227520-15227670:plus -33.3061 -0.981651 -0.174312 2.95413 -17.4184 -18.2755 -20.6606 -4.90278
-0.889908 0.0279 0.0143 0.0123 0.00917 0.00457 0.0112 0.0231 0.0604 0.0253
3L:15228580-15228730:minus -21.9286 -0.211009 0.926606 0.743119 -17.4898 -17.898 -17.156 -0.166667
1.23853 0.0027 0.0111 0.00848 0.0221 0.0053 0.00726 0.0131 0.0176 0.0132
3L:15228580-15228730:plus -12.7347 -0.146789 4.78899 9.33028 -26.3673 -18.0102 25.4679 -0.972222
12.2752 0.000468 0.0108 0.00194 0.000374 0.0182 0.00812 2.89e-10 0.0224 0.0000141
3L:15297640-15297790:minus -30.3673 5.77064 6.58716 6.3578 -27.4184 -8.65306 -21.5138 -2.36111
3.46789 0.00897 0.00117 0.00088 0.00195 0.0323 0.00156 0.027 0.0328 0.00582
3L:15297640-15297790:plus -23.2245 -2.33028 6.34862 5.86239 -7.67347 -26.5612 -12.8165 3.22222 2.63303
0.00576 0.0214 0.000987 0.00282 0.00055 0.0237 0.00554 0.00548 0.00771
3L:15298400-15298550:minus -41.7755 3.34862 -4.11009 -0.40367 -19.051 -18.3061 -30.055 3.95833
-4.78899 0.0652 0.00306 0.0391 0.0302 0.0162 0.0114 0.0916 0.00409 0.0691
3L:15298400-15298550:plus -23.9592 -1.75229 2.87156 6 1.87755 -18.0102 -12.5872 -0.361111 1.90826
0.00708 0.0181 0.00417 0.00256 0.0000492 0.00812 0.00526 0.0187 0.00974
3L:1534020-1534170:minus -20.6633 1.98165 2.20183 2.88991 10.9898 -8.79592 -13.7982 0.555556 1.2844
0.00173 0.00505 0.00536 0.00942 3.28e-06 0.0019 0.00693 0.014 0.0129
3L:1534020-1534170:plus -32.0714 0.779817 1.84404 6.11009 -26.9286 -7.94898 -21.4771 5.98611 -3.6055
0.0176 0.00784 0.00612 0.00233 0.0214 0.00074 0.0268 0.00167 0.0507
3L:1536880-1537030:minus -13.1939 -2.76147 0.00917431 5.58716 -26.1939 -36.2245 -5.65138 -1.56944
7.15596 0.000709 0.0241 0.0116 0.00334 0.0175 0.0923 0.00119 0.0265 0.00124
3L:1536880-1537030:plus -31.9694 -4.75229 0.33945 5.30275 10.3878 -8.45918 -25.9541 1.01389 -4.45872
0.0168 0.0413 0.0104 0.00396 0.0000174 0.00111 0.0532 0.0121 0.0633
3L:1537320-1537470:minus -34.2551 -1.41284 -4 -2.10092 -8.89796 -18.2755 -20.8257 -4.33333 -7.83486
0.0315 0.0163 0.038 0.0529 0.00189 0.0112 0.0238 0.0532 0.131
3L:1537320-1537470:plus -13.1837 -0.0825688 -1.21101 0.779817 -17.1837 -9.23469 -8.85321 0.486111
5.12844 0.000705 0.0106 0.017 0.0219 0.00366 0.00238 0.00249 0.0144 0.00301
3L:1546060-1546210:minus -31.7449 9.81651 1.82569 6.00917 -18.6429 -9.68367 -32.2202 4.90278 -1.94495
0.0152 0.000147 0.00616 0.00255 0.0121 0.00342 0.121 0.00274 0.0327
3L:1546060-1546210:plus -24.1837 -0.541284 5.46789 0.87156 -18.4184 -18.3061 -5.63303 -3.84722
-0.550459 0.00751 0.0124 0.00146 0.0213 0.0101 0.0114 0.00119 0.0475 0.0225
3L:1546540-1546690:minus -31.1429 -0.623853 -0.0275229 -0.752294 -18.4592 -18.4898 -14 3.55556
4.78899 0.0108 0.0127 0.0117 0.034 0.0109 0.0129 0.00725 0.00481 0.00336
3L:1546540-1546690:plus -13.0816 -2.10092 2.3211 2.34862 0.653061 -9.16327 -15.2018 -4.125 1.27523
0.000599 0.02 0.00513 0.0121 0.000239 0.00223 0.00934 0.0507 0.013
3L:1548080-1548230:minus -31.6633 6.31193 -0.247706 -0.366972 -8.63265 -17.5714 -20.4037 0.430556
-2.26606 0.0141 0.000913 0.0126 0.0298 0.0013 0.00616 0.0221 0.0146 0.0359
3L:1548080-1548230:plus -30.7041 13.4592 6.59633 2.50459 -27.9694 -19.0816 -22.7156 3.375 -0.211009
0.00993 0.000064 0.000876 0.0114 0.0437 0.017 0.0334 0.00516 0.02
3L:1549040-1549190:minus -21.551 -0.623853 -0.972477 3.86239 -17.7143 -19.3061 -14.2294 6.02778
6.7156 0.00205 0.0127 0.0158 0.00622 0.00627 0.0187 0.00763 0.00164 0.00144
3L:1549040-1549190:plus -31.7347 -3.33028 -1.82569 2.53211 -18.1122 -28.898 -10.6697 1.05556 5.18349
0.0149 0.0282 0.0205 0.0113 0.00771 0.0713 0.0035 0.0119 0.00295
3L:15491900-15492050:minus -32.9286 -1.05505 13.9541 7.2844 10.0918 -9.72449 -18.2477 -1.95833
6.33028 0.025 0.0146 7.63e-06 0.00105 0.0000251 0.00352 0.0155 0.0295 0.0016
3L:15491900-15492050:plus -14.4388 1.15596 12.9817 7.29358 0.836735 -9.02041 -12.9633 0.138889 -0.284404
0.00137 0.00684 0.0000211 0.00104 0.000215 0.00213 0.00573 0.016 0.0207
3L:15494020-15494230:minus -30.4796 1.21101 3.40367 3.95413 -28.3061 -28.051 -14.5229 0.138889
2.18349 0.00941 0.00671 0.0034 0.00593 0.0553 0.0563 0.00813 0.016 0.00871
3L:15494020-15494230:plus -30.4388 6.77982 1.56881 7.77982 -17.8878 1.02041 -2.66972 8.97222 -2.92661

0.00923	0.000727	0.00676	0.000652	0.00704	0.000118	0.000522	0.000326	0.0437
3L:15494460-15494610:minus	-30.7755	3.97959	0.256881	5.86239	-17.7551	-18.3469	-14.9817	-0.402778
6.18349	0.0102	0.000669	0.0106	0.00282	0.00658	0.0124	0.00894	0.0189	0.00167
3L:15494460-15494610:plus	-32.4388	-0.431193	3.44954	1.97248	-27.1939	-17.1224	-17.7523	2.81944
7.6422	0.0195	0.0119	0.00334	0.0146	0.0267	0.00499	0.0144	0.00639	0.000893
3L:1549720-1549870:minus	-32.2857	-4.51376	0.366972	3.74312	-8.37755	-27.5612	-7.13761	-1.75	0.238532
0.0185	0.0388	0.0103	0.00651	0.00106	0.0393	0.00174	0.0279	0.0173
3L:1549720-1549870:plus	-13.1531	8.76147	7.2844	2.55963	-26.898	-16.0102	-4.10092	3.30556	1.21101
0.00067	0.000254	0.000632	0.0111	0.0203	0.00406	0.000768	0.0053	0.0134
3L:15497940-15498090:minus	-14.2143	5.48624	7.7156	4.37615	1.94898	0.244898	-15.9817	8.40278
-2.77982	0.00124	0.00133	0.000509	0.00504	0.0000358	0.000285	0.0108	0.000462	0.042
3L:15497940-15498090:plus	-31.8469	3.14679	7.88991	-0.256881	-17.6429	9.30612	-19.5596	1	-2.27523
0.0162	0.00329	0.000464	0.0289	0.00562	0.0000398	0.0191	0.0121	0.036
3L:15500700-15500850:minus	-24.449	3.00917	-0.0642202	1.08257	-19.0102	-16.2755	-17.3303	-1.13889
-4.24771	0.00802	0.00347	0.0118	0.0201	0.0157	0.00413	0.0135	0.0235	0.0591
3L:15500700-15500850:plus	-33.0408	8.34862	15.4495	3.76147	-8.37755	-8.7551	-18.2844	0.194444	5.53211
0.0264	0.000315	9.17e-07	0.00645	0.00106	0.00172	0.0156	0.0157	0.00245
3L:15501060-15501210:minus	-12.4082	0.0458716	5.85321	7.6422	-17.1939	-26.602	-11.1193	-1.52778
6.73394	0.000414	0.0101	0.00123	0.000721	0.00377	0.0248	0.00382	0.0262	0.00143
3L:15501060-15501210:plus	-21.551	1.66972	-0.944954	2.76147	-8.0102	-9.5	-14.7798	-2.29167	-3.24771
0.00205	0.00567	0.0157	0.01	0.000826	0.00294	0.00858	0.0322	0.0471
3L:15502620-15502770:minus	-23.2653	1.12844	1.6789	9.33028	-18.1939	-8.02041	-10.789	3.29167
3.52294	0.00593	0.00691	0.0065	0.000374	0.0086	0.000761	0.00358	0.00533	0.00558
3L:15502620-15502770:plus	-20.5918	-3.23853	14.1835	7.45872	20.2041	-27.5612	-10.0734	-7.36111	5.80734
0.00169	0.0275	5.88e-06	0.000894	3.64e-07	0.0393	0.00312	0.099	0.00205
3L:15504000-15504150:minus	-23.2551	-5.76147	2.77064	3.44037	-8.64286	1.82653	-11.6055	4.27778
0.0183486	0.00583	0.0538	0.00433	0.00746	0.00139	0.0000498	0.00423	0.00358	0.0183
3L:15504000-15504150:plus	-23.4796	0.220183	0.954128	9.44954	-17.7857	-9.37755	-10.0917	11.4444	7.73394
0.00634	0.00956	0.0084	0.000326	0.00676	0.00265	0.00313	0.0000519	0.000784
3L:15507600-15507750:minus	-41.6633	4.08257	-5.63303	-2.11927	-19.2755	-9.30612	-24.9083	1.91667
-5.53211	0.0636	0.00233	0.0579	0.0532	0.0164	0.00257	0.0466	0.00888	0.0809
3L:15507600-15507750:plus	-32.3367	3.59633	4.88073	1.92661	-28.2347	-17.3469	-21.3578	2.34722	1.36697
0.0187	0.00279	0.00187	0.0148	0.051	0.0058	0.0262	0.00761	0.0124
3L:15508120-15508270:minus	-23.6224	2.6789	1.55046	1.63303	-27.2245	-9.65306	-18.6514	-1.375
1.40367	0.00658	0.00391	0.0068	0.0167	0.0272	0.0034	0.0165	0.0251	0.0122
3L:15508120-15508270:plus	-32.551	-0.834862	2.3211	7.78899	1.43878	-8.79592	-6.15596	1.54167
3.98165	0.0207	0.0136	0.00513	0.000642	0.0000769	0.0019	0.00137	0.0101	0.00439
3L:15509600-15509750:minus	-33.1122	-1.37615	4.01835	7	-27.3061	-8.79592	-20.6055	8.48611	-3.94495
0.0271	0.0161	0.00266	0.00132	0.0315	0.0019	0.0229	0.00044	0.0549
3L:15509600-15509750:plus	-13.7449	14.0367	-0.899083	7.02752	-17.7143	-18.7143	-16.2752	0.180556
-2.52294	0.00107	8.06e-06	0.0155	0.00122	0.00627	0.014	0.0114	0.0158	0.0391
3L:15517160-15517310:minus	-41.2245	3.97959	2.02752	5.07339	0.806122	-9.09184	-19.0092	3.5	-2.53211
0.0497	0.000669	0.00572	0.0043	0.000229	0.00219	0.0175	0.00491	0.0392
3L:15517160-15517310:plus	-31.7041	4.23853	1.61468	3.54128	-17.449	-9.0102	-19.578	4.875	0.788991
0.0146	0.0022	0.00665	0.00702	0.00484	0.00208	0.0192	0.00277	0.0151
3L:15525500-15525650:minus	-21.9592	2.58716	-2.25688	2.19266	-26.9286	-0.316327	-12.6239	9.90278
7.63303	0.00279	0.00404	0.0233	0.0131	0.0214	0.000524	0.0053	0.000176	0.000928
3L:15525500-15525650:plus	-22.4796	-5.22936	-0.807339	2.37615	-26.6327	-36.1531	-14.1009	-2.19444
3.87156	0.00352	0.0469	0.015	0.0119	0.0188	0.0908	0.00742	0.0314	0.00477
3L:15526440-15526590:minus	-13.2245	4.34862	-3.42202	2.06422	-27.2653	0.826531	-13.367	8	10.0367
0.000738	0.0021	0.0324	0.0139	0.0302	0.000153	0.00629	0.000583	0.000146
3L:15526440-15526590:plus	-24.2959	6.13761	5.17431	7	-25.9694	-19.0816	-12.0367	1.94444	4.40367
0.00781	0.000993	0.00165	0.00132	0.017	0.017	0.00465	0.00879	0.00373
3L:15527760-15527910:minus	-32	2.31193	4.53211	2.23853	-17.3367	-18.1224	-16.9725	-2.59722	3.49541

0.0171 0.00448 0.00216 0.0128 0.00391 0.00988 0.0128 0.0349 0.0057
3L:15527760-15527910:plus -23.1531 2.88991 -2.34862 3.3578 -17.4898 -26.6735 -16.4404 -3.44444 -4.42202
0.00542 0.00362 0.0239 0.00767 0.0053 0.0256 0.0117 0.0431 0.0626
3L:15528360-15528510:minus -31.7347 5.29358 6.73394 3.44954 -27.2653 -8.72449 -20.8165 2.73611
-4.06422 0.0149 0.00144 0.000821 0.00738 0.0302 0.00169 0.0238 0.00659 0.0565
3L:15528360-15528510:plus -13.1122 5.84404 4.3211 7.90826 0.918367 -8.79592 -8.62385 -4.40278 -5.12844
0.000632 0.00113 0.00235 0.000611 0.000142 0.0019 0.00238 0.054 0.0748
3L:15529060-15529210:minus -13.4082 -3.9633 5.3578 1.52294 -17.9388 -0.397959 -10.1101 3.08333
1.88073 0.000867 0.0335 0.00153 0.0175 0.00715 0.000525 0.00314 0.00578 0.00987
3L:15529060-15529210:plus -32.9286 -3.80734 -4.22018 -4.18349 -27 -27.9694 -15.7431 8.95833 9.92661 0.025
0.0321 0.0403 0.0879 0.0233 0.0547 0.0104 0.000329 0.000193
3L:15530840-15530990:minus -31.5918 0.908257 -1.95413 5.59633 -17.7959 -0.27551 -19.5413 -0.0277778
-1.73394 0.0136 0.00748 0.0213 0.00331 0.00678 0.000502 0.0191 0.0169 0.0311
3L:15530840-15530990:plus -32.7755 5.02752 -0.477064 6.84404 -9.0102 -25.898 -19.3211 -2.11111
1.74312 0.0234 0.00161 0.0135 0.0014 0.00236 0.0214 0.0184 0.0307 0.0105
3L:15531280-15531430:minus -31.2857 3.64286 4.53211 1.61468 -18.0816 -18.1633 -10.6789 -3.86111
9.66055 0.0113 0.00159 0.00216 0.0169 0.00759 0.01 0.0035 0.0477 0.000268
3L:15531280-15531430:plus -23.2653 -3.43119 2.55046 3.44037 -18.7143 -8.23469 -22.9541 7.55556 3.52294
0.00593 0.029 0.00471 0.00746 0.0136 0.000875 0.0348 0.000748 0.00558
3L:15547280-15547430:minus -20.6224 13.5306 0.59633 1.99083 -8.93878 -16.5714 -14.0092 2.43056
3.08257 0.0017 0.0000206 0.00949 0.0144 0.00216 0.00417 0.00727 0.00738 0.00681
3L:15547280-15547430:plus -24.3367 0.614679 2.19266 8.80734 -17.3776 -18.8571 -14.1927 8.44444 1.61468
0.00789 0.00832 0.00538 0.000518 0.00419 0.0151 0.00757 0.000451 0.0112
3L:15547560-15547710:minus -23.1939 3.61224 4.68807 3.19266 -18.7551 -17.2755 -16.0917 1.91667
-1.6055 0.00558 0.00167 0.00203 0.00829 0.0144 0.00544 0.011 0.00888 0.0302
3L:15547560-15547710:plus -41.4796 6.18349 4.77982 3.97248 -27.7959 -19.5714 -14.2018 -1.94444 -3.13761
0.0577 0.000972 0.00195 0.00587 0.0386 0.0202 0.00758 0.0294 0.0461
3L:15556140-15556290:minus -31.6224 5.84404 6.66972 6.74312 -18.1939 -9.7551 -17.7156 1.01389
3.76147 0.0137 0.00113 0.000847 0.0015 0.0086 0.00358 0.0143 0.0121 0.00494
3L:15556140-15556290:plus -22.6224 13.3878 -1.04587 6.49541 -8.86735 1.31633 -10.5688 3.27778 -0.440367
0.00371 0.0000869 0.0162 0.00176 0.00173 0.000066 0.00343 0.00536 0.0216
3L:15556680-15556830:minus -12.0714 -1.48624 -0.715596 1.68807 -9.27551 -8.5 -11.4037 2.375
-0.220183 0.000318 0.0167 0.0146 0.0164 0.00284 0.00126 0.00406 0.00753 0.0201
3L:15556680-15556830:plus -14.102 0.422018 3.47706 6.76147 -27.7959 -17.7857 -11.6147 -1.61111 0.183486
0.00117 0.0089 0.0033 0.00148 0.0386 0.0068 0.00424 0.0268 0.0176
3L:15559060-15559210:minus -32.5918 6.74312 -0.633028 2.57798 -7.86735 -18.1224 -20.6239 8.45833
-0.651376 0.021 0.00074 0.0142 0.011 0.000617 0.00988 0.023 0.000447 0.0235
3L:15559060-15559210:plus -21.9286 7.29358 4.33945 4.3211 -18.7551 -8.72449 -15.5688 1.23611 -0.990826
0.0027 0.000556 0.00234 0.00514 0.0144 0.00169 0.01 0.0112 0.026
3L:15559300-15559450:minus -30.8878 6.04587 0.357798 4.36697 -17.1837 -9.5 -4.68807 -2.88889 -2.87156
0.0103 0.00104 0.0103 0.00505 0.00366 0.00294 0.000908 0.0376 0.043
3L:15559300-15559450:plus -21.551 -1.27523 5.82569 7.47706 -25.9286 0.122449 -14.4128 -3.16667 -2.47706
0.00205 0.0156 0.00125 0.00085 0.0168 0.000312 0.00794 0.0403 0.0385
3L:15559780-15559930:minus -32.7755 3.80734 14.5872 -0.633028 -17.1224 -19.1531 -17.789 4.70833
1.79817 0.0234 0.00259 3.48e-06 0.0327 0.00343 0.0177 0.0145 0.00298 0.0102
3L:15559780-15559930:plus -31.3265 -0.376147 2.53211 1.53211 -27.3776 -17.3469 -9.33945 3.30556
4.88073 0.0114 0.0117 0.00475 0.0174 0.032 0.0058 0.00272 0.0053 0.00324
3L:15560780-15560930:minus -32.0408 3.41284 -1.78899 1.31193 -17.6429 -26.8265 -16.2844 6.72222
3.21101 0.0174 0.00299 0.0203 0.0189 0.00562 0.0285 0.0114 0.00116 0.00644
3L:15560780-15560930:plus -23.5204 3.06422 6.61468 3.3211 -18.4898 10.2347 -17.5596 3.95833 -0.284404
0.00645 0.0034 0.000869 0.00782 0.0116 7.75e-06 0.014 0.00409 0.0207
3L:15569480-15569630:minus -24.8571 3.90816 2.6422 1.33028 -27.2653 -6.79592 -16.0367 3.40278
3.63303 0.00831 0.00102 0.00455 0.0188 0.0302 0.000546 0.0109 0.00511 0.00518
3L:15569480-15569630:plus -31.2551 14.945 10.1927 8.98165 -17.8878 -27.1939 -20.1284 5.55556 1.3211

0.0113 2.29e-06 0.000128 0.000461 0.00704 0.0327 0.0211 0.00204 0.0127
3L:15573740-15573890:minus -32.5102 13.1101 -1.24771 1.77982 -18.4898 -17.6429 -17.6606 0.875
1.95413 0.0202 0.0000203 0.0172 0.0158 0.0116 0.00619 0.0142 0.0127 0.00964
3L:15573740-15573890:plus -32.1735 14.4862 10.7431 7.55963 -17.3776 0.826531 -18.3578 1.16667 0.357798
0.018 5.19e-06 0.0000926 0.000807 0.00419 0.000153 0.0158 0.0115 0.0166
3L:15576660-15576810:minus -2.33673 9.04587 0.0275229 2.62385 10.3878 -17.0102 -14.3761 2.84722
-2.48624 0.0000294 0.000218 0.0115 0.0108 0.0000174 0.00453 0.00788 0.00632 0.0387
3L:15576660-15576810:plus -31.8571 4.09174 -0.220183 -0.899083 -26.9286 9.60204 -9.2844 0.236111
-0.33945 0.0163 0.00232 0.0125 0.0356 0.0214 0.0000248 0.0027 0.0155 0.0209
3L:15581200-15581350:minus -31.7857 -1.97248 -0.651376 2.20183 -7.60204 -17.9796 -16.4495 -2.66667
-0.0733945 0.0157 0.0193 0.0143 0.013 0.000466 0.00764 0.0117 0.0355 0.0188
3L:15581200-15581350:plus -31.9694 5.62385 -2.08257 6.84404 -28.2653 -18.2755 -19.1101 5.88889 1.93578
0.0168 0.00125 0.0222 0.0014 0.0537 0.0112 0.0178 0.00175 0.00969
3L:15581860-15582010:minus -23 2.04587 -0.220183 1.99083 -27.5306 -27.4082 -5.29358 -2.18056
-2.3945 0.00498 0.00494 0.0125 0.0144 0.0351 0.0356 0.00108 0.0313 0.0375
3L:15581860-15582010:plus -21.4796 3.58716 -0.513761 4.02752 -7.89796 -19.3776 -16.0367 2.13889
4.98165 0.00202 0.0028 0.0137 0.00581 0.000678 0.0195 0.0109 0.0082 0.00313
3L:1558300-1558450:minus -24.3673 -1.07339 2.88991 -1.24771 -18.7449 -27.5612 -5.17431 5.20833 7.68807
0.00791 0.0147 0.00414 0.0398 0.0138 0.0393 0.00104 0.00239 0.000841
3L:1558300-1558450:plus -33.1531 1 -1.08257 0.321101 -18.7551 -28.7449 -20.2202 2.23611
6.37615 0.0273 0.00724 0.0164 0.0247 0.0144 0.0672 0.0214 0.00792 0.00159
3L:15583420-15583570:minus -15.0102 5.80734 -1.62385 5.43119 -18.0816 -8.5 -24.3486
-3.54167 5.7156 0.00162 0.00116 0.0193 0.00371 0.00759 0.00126 0.0432 0.0442 0.00217
3L:15583420-15583570:plus -30.9694 7.19266 -1.37615 5.73394 -27.1939 -9.5 -22.9358
2.76389 -4.54128 0.0104 0.000587 0.0179 0.00307 0.0267 0.00294 0.0347 0.00652 0.0645
3L:15587560-15587710:minus -23.0306 0.53211 4.77982 6.21101 -18.6429 -9.65306 -13.6239
4.34722 7.04587 0.00505 0.00856 0.00195 0.00213 0.0121 0.0034 0.00667 0.00348 0.00132
3L:15587560-15587710:plus -22.1122 1.08257 -3.70642 3.26606 -25.898 -9.09184 -19.1743
4.61111 -0.798165 0.00304 0.00703 0.0351 0.00802 0.0166 0.00219 0.0179 0.00311 0.0246
3L:15588300-15588450:minus -21.5918 -5.44037 3.79817 4.3211 -26.9694 -17.7857 -18.9083
2.06944 4.18349 0.00209 0.0495 0.00291 0.00514 0.023 0.0068 0.0172 0.00841 0.00404
3L:15588300-15588450:plus -23.0408 3.38532 2.87156 5.22936 -18.7551 -9.30612 -16.6147
-1.19444 2.13761 0.00512 0.00302 0.00417 0.0041 0.0144 0.00257 0.012 0.0239 0.00885
3L:15588960-15589110:minus -32.7347 1.22936 4.70642 5.94495 -18.4796 -27.6327 -3.89908
6.31944 3 0.0225 0.00666 0.00201 0.00261 0.011 0.043 0.000725 0.00142 0.00702
3L:15588960-15589110:plus -30.3673 -0.0366972 6.45872 5.49541 -18.4898 -17.051 -12.7064
3.40278 -0.302752 0.00897 0.0104 0.000937 0.00355 0.0116 0.00479 0.00541 0.00511 0.0208
3L:15589460-15589610:minus -11.1122 2.22936 2.04587 7.78899 -7.67347 -8.93878 -11.3578
2.27778 -4.93578 0.000244 0.00461 0.00568 0.000642 0.00055 0.00201 0.00402 0.0078 0.0718
3L:15589460-15589610:plus -24.1837 2.89908 6.69725 7.12844 -17.449 0.826531 -20.2844 7.09722
-4.17431 0.00751 0.00361 0.000836 0.00118 0.00484 0.000153 0.0216 0.000956 0.058
3L:15590380-15590530:minus -32.4796 -0.220183 1.91743 11.2936 -8.60204 -18.1224 -3.41284
-2.94444 7.72477 0.02 0.0111 0.00596 0.0000139 0.00125 0.00988 0.000634 0.0381 0.000798
3L:15590380-15590530:plus -31.5102 -0.550459 0.449541 4.04587 -8.57143 -17.8265
-3.22018 -3.72222 -2.0367 0.013 0.0124 0.00998 0.00572 0.00113 0.00699 0.000602 0.0461 0.0336
3L:15593440-15593590:minus -13.8571 0.311927 -5.25688 1.57798 -17.1531 -9.0102 -21.5505
0.152778 7.68807 0.0011 0.00925 0.0527 0.0171 0.00364 0.00208 0.0272 0.016 0.000841
3L:15593440-15593590:plus -14.1429 3.23853 3.87156 1.19266 -17.7143 -9.30612 -3.20183
-0.0972222 -0.146789 0.0012 0.00318 0.00282 0.0195 0.00627 0.00257 0.000599 0.0172 0.0194
3L:15603380-15603530:minus -33.7347 13.8807 -2.80734 4.11009 -17.9796 -8.23469 -15.8349
-0.333333 -4.66055 0.0294 9.55e-06 0.0273 0.00553 0.00726 0.000875 0.0105 0.0185 0.0667
3L:15603380-15603530:plus -14.4898 13.5505 4.09174 9.59633 0.316327 19.3469 -17.4495 0.305556
-4.36697 0.00143 0.0000136 0.00258 0.000275 0.000341 5.04e-07 0.0137 0.0152 0.0615
3L:15612860-15613010:minus -31.1122 4.89908 9.38532 3.90826 -18.7143 -9.79592 -11.6514 8.5

7.22018 0.0107 0.0017 0.000203 0.00603 0.0136 0.00377 0.00427 0.000436 0.00119
3L:15612860-15613010:plus -21.6633 -0.412844 -1.01835 0.568807 -27.0408 0.540816
-15.8532 2.52778 11.8532 0.00223 0.0119 0.0161 0.0232 0.0242 0.0002 0.0106 0.00712 0.0000428
3L:15619260-15619410:minus -22.8061 3.33028 3.44037 2.21101 -18.449 -27.602 1.01835 0.375 9.87156
0.00414 0.00308 0.00335 0.0129 0.0107 0.0416 0.000219 0.0149 0.000217
3L:15619260-15619410:plus -4.33673 4.57798 2.36697 2.33945 -27.1939 -17.9796 -17.1743
-2.66667 9.92661 0.00014 0.00193 0.00505 0.0122 0.0267 0.00764 0.0132 0.0355 0.000193
3L:1568260-1568410:minus -20.7755 -0.220183 0.0366972 4.0367 -18.449 -18.9388 -11.3211
-2.875 7.01835 0.00176 0.0111 0.0115 0.00578 0.0107 0.0154 0.00399 0.0375 0.00134
3L:1568260-1568410:plus -31.2959 1.08257 -1.83486 0.53211 -17.1224 -9.16327 -2.86239
1.19444 8.19266 0.0113 0.00703 0.0206 0.0234 0.00343 0.00223 0.000548 0.0114 0.000548
3L:1569060-1569210:minus -31.449 2.70642 3.88073 3.6055 10.4286 -8.5 -17.4404 2.33333 0.385321
0.0123 0.00387 0.00281 0.00686 0.0000139 0.00126 0.0137 0.00765 0.0166
3L:1569060-1569210:plus -14.4388 0.926606 2.37615 5.06422 -6.67347 -7.5 -19.3578 2.11111
-4.93578 0.00137 0.00743 0.00503 0.00433 0.000359 0.000614 0.0185 0.00828 0.0718
3L:15693000-15693150:minus -13.9286 0.963303 3.66055 9.33028 -17.8878 -7.45918 -11.6697
-0.916667 -3.08257 0.00112 0.00734 0.00307 0.000374 0.00704 0.000582 0.00429 0.022 0.0455
3L:15693000-15693150:plus -24.1429 4.37615 0.486239 6.74312 -17.1122 -27.7143 -10.4037
-3.79167 1.33028 0.0074 0.00208 0.00986 0.0015 0.00341 0.0447 0.00332 0.0469 0.0127
3L:15809300-15809450:minus -22 -6.6789 3.43119 2.33945 -8.23469 -7.42857 -6.68807 5.66667
5.23853 0.00291 0.0676 0.00336 0.0122 0.000927 0.000567 0.00156 0.00194 0.00285
3L:15809300-15809450:plus -32.2245 0.807339 2.86239 9.41284 -18.051 0.244898 -18.6055
1.86111 -6.00917 0.0183 0.00776 0.00418 0.000354 0.00746 0.000285 0.0164 0.00905 0.089
3L:15809940-15810090:minus -22.2959 2.87156 3.48624 -0.770642 -27.2347 -17.8571 -14.6972
9.36111 5.9633 0.00335 0.00365 0.00329 0.0342 0.0283 0.00719 0.00843 0.000254 0.00188
3L:15809940-15810090:plus -32 15.4037 0.376147 1.99083 -26.898 -17.9796 -13.7431 -1.93056
-2.70642 0.0171 1.16e-06 0.0102 0.0144 0.0203 0.00764 0.00685 0.0293 0.0412
3L:15818800-15818950:minus -23 8.29358 -3.19266 1.9633 -18.7449 -27.1224 -1.65138
1.93056 11.8532 0.00498 0.000326 0.0304 0.0146 0.0138 0.0325 0.00041 0.00883 0.0000428
3L:15818800-15818950:plus -22.2245 4.66972 0.761468 7.3578 -8.89796 -8.72449 -14.4587
2.84722 -6.40367 0.00325 0.00186 0.00897 0.000965 0.00189 0.00169 0.00802 0.00632 0.0981
3L:15819440-15819590:minus -22.9694 -2.11927 4.37615 7.21101 -18.0102 9.82653 -13.6514 0.722222
7.73394 0.0048 0.0201 0.0023 0.0011 0.00732 0.0000148 0.00671 0.0133 0.000784
3L:15819440-15819590:plus -31.1429 0.963303 5.25688 3.97248 -18.4184 -28.898 -18.844 3.25 -0.678899
0.0108 0.00734 0.0016 0.00587 0.0101 0.0713 0.017 0.00542 0.0237
3L:15819900-15820050:minus -22.5612 13.4592 3.45872 1.43119 -17.9388 -8.45918 -20.2202 2.5 -0.853211
0.0036 0.000064 0.00332 0.0181 0.00715 0.00111 0.0214 0.0072 0.0251
3L:15819900-15820050:plus -5.11224 13.4592 -1.37615 3.57798 -8.93878 -17.2755 -7.50459 -1.91667 -5.25688
0.000224 0.000064 0.0179 0.00696 0.00216 0.00544 0.00189 0.0291 0.0768
3L:15820620-15820770:minus -22.3367 9.50459 2.54128 3.70642 -19.0204 -19.5714 -20.4954 1.44444
-2.34862 0.00339 0.000172 0.00473 0.0066 0.0158 0.0202 0.0225 0.0105 0.0368
3L:15820620-15820770:plus -31.8163 -3.88073 2.12844 2.06422 -17.4898 -25.8265 -18.3945 2.83333 5.75229
0.016 0.0328 0.00551 0.0139 0.0053 0.0212 0.0159 0.00635 0.00213
3L:15825460-15825610:minus -3.29592 7.76147 -0.275229 5.49541 10.6939 -9.30612 -12.3761 1.34722
-1.16514 0.0000549 0.000434 0.0127 0.00355 7.35e-06 0.00257 0.00501 0.0108 0.0274
3L:15825460-15825610:plus -40.4796 -1.77982 15.6606 3.11009 -18.6837 0.826531 -17.8624 3.26389 -2.40367
0.0407 0.0182 3.94e-07 0.00862 0.0126 0.000153 0.0146 0.00539 0.0376
3L:15828420-15828570:minus -24.449 3.79817 0.908257 6.41284 -7.86735 -8.79592 -13.7431 5.80556
1.63303 0.00802 0.00259 0.00853 0.00189 0.000617 0.0019 0.00685 0.00182 0.0112
3L:15828420-15828570:plus -33.1122 -0.00917431 5.95413 0.825688 -18.1939 -17.6429 -19.4128 7.05556
-1.57798 0.0271 0.0103 0.00118 0.0216 0.0086 0.00619 0.0187 0.000977 0.0299
3L:15830480-15830630:minus -33.3367 -0.220183 -1.59633 6.55963 -7.86735 -8.79592 -13.0734 1.22222
-0.743119 0.0281 0.0111 0.0192 0.00172 0.000617 0.0019 0.00588 0.0113 0.0243
3L:15830480-15830630:plus -41.5612 6.27523 4.86239 5.30275 -18.3776 -16.9796 -7.66972 5.69444 -1.22018

0.0609 0.00093 0.00188 0.00396 0.00926 0.0044 0.00196 0.00191 0.0278
3L:1586620-1586770:minus -23.2245 8.42202 2.93578 2.55963 -18.5204 -37.449 -11.3486 4.61111 12.2752
0.00576 0.000304 0.00407 0.0111 0.0117 0.164 0.00401 0.00311 0.0000141
3L:1586620-1586770:plus -3.29592 -2.98165 4.91743 3.34862 -8.93878 -17.0102 -7.78899 -1.38889 2.09174
0.0000549 0.0257 0.00184 0.0077 0.00216 0.00453 0.00201 0.0252 0.00904
3L:15948140-15948290:minus -31.4388 -0.293578 5.30275 3.56881 -18.7143 -18.3776 -22.5596 3.48611
-4.51376 0.0122 0.0114 0.00157 0.00699 0.0136 0.0125 0.0326 0.00494 0.064
3L:15948140-15948290:plus -31.9592 -1.24771 5.24771 1.26606 20.2041 -0.244898 -23.9633 4.30556
-4.52294 0.0167 0.0155 0.0016 0.0191 3.64e-07 0.000496 0.0408 0.00354 0.0642
3L:15949620-15949770:minus -32.7857 -4.21101 6.78899 7.55963 -18.6837 -17.3469 -19.4679 0.222222
0.321101 0.0235 0.0358 0.000801 0.000807 0.0126 0.0058 0.0188 0.0156 0.0169
3L:15949620-15949770:plus -39.9592 -2.10092 6.86239 9.41284 10.398 -35.602 -16.844 0.0972222
-0.174312 0.0348 0.02 0.000775 0.000354 0.0000153 0.0803 0.0125 0.0162 0.0196
3L:15961220-15961370:minus -34.0816 0.366972 -0.0183486 3.48624 -19.051 -8.27551 -16.2661 4.80556
1.2844 0.0311 0.00908 0.0117 0.0072 0.0162 0.000919 0.0114 0.00286 0.0129
3L:15961220-15961370:plus -13.8469 3.77982 1.3211 9.51376 20.4694 -8.72449 -10.578 0.625 1.23853
0.0011 0.00261 0.00738 0.000292 1.65e-07 0.00169 0.00343 0.0137 0.0132
3L:15969980-15970130:minus -23.2245 -0.357798 -0.633028 2.23853 -28.7959 9.23469 -30.1743
8.05556 -0.642202 0.00576 0.0116 0.0142 0.0128 0.0641 0.0000427 0.0932 0.000565 0.0234
3L:15969980-15970130:plus -22.5816 -1.86239 12.367 11.1927 -18.4184 -8.53061 -7.04587 5.77778 0.394495
0.00361 0.0187 0.0000334 0.0000254 0.0101 0.00136 0.00171 0.00184 0.0166
3L:15974560-15974710:minus -32.6224 1.26606 8.11009 5.49541 -26.6327 -27.1224 -10.8716 1.83333
3.20183 0.0211 0.00657 0.000413 0.00355 0.0188 0.0325 0.00364 0.00914 0.0065
3L:15974560-15974710:plus -12.8469 -0.366972 12.4771 3.89908 -18.449 -8.79592 -12.6697 5.5 4.19266
0.000502 0.0117 0.0000306 0.00604 0.0107 0.0019 0.00536 0.00209 0.00401
3L:15975960-15976110:minus -3.26531 -0.440367 0.321101 0.724771 -17.3776 -18.2755 -24.4128 1.66667
7.68807 0.0000515 0.012 0.0104 0.0223 0.00419 0.0112 0.0436 0.00969 0.000841
3L:15975960-15976110:plus -14.0408 -2.99083 -2.02752 2.98165 -17.449 -36.1122 -8.22018 0.472222 0.963303
0.00115 0.0257 0.0218 0.00905 0.00484 0.0878 0.0022 0.0144 0.0145
3L:15981260-15981410:minus -23.2245 1.19266 0.963303 -0.779817 -17.6837 19.1531 -20.0642 3.06944
2.85321 0.00576 0.00675 0.00837 0.0343 0.0058 7.95e-07 0.0208 0.00581 0.00733
3L:15981260-15981410:plus -22.8061 1.89908 5.40367 6.02752 -17.449 -19.0102 -19.2661 -6.19444 -2.95413
0.00414 0.00521 0.0015 0.00246 0.00484 0.0159 0.0182 0.0791 0.0441
3L:15997660-15997810:minus -40.0306 0.0825688 5.47706 3.47706 -27.9694 -17.7551 -11.0734 8.31944
7.94495 0.0357 0.01 0.00145 0.00724 0.0437 0.00643 0.00379 0.000485 0.000667
3L:15997660-15997810:plus -13.7449 0 -1.77982 -0.788991 -27.602 -27.602 -10.844 -0.0972222
3.47706 0.00107 0.0103 0.0202 0.0343 0.0364 0.0416 0.00362 0.0172 0.00575
3L:15998000-15998150:minus -41.0612 7.34862 7.6055 3.18349 -17.7143 -18.5714 -23.3028 2.56944
-3.13761 0.0466 0.000541 0.000539 0.00837 0.00627 0.0136 0.0369 0.00701 0.0461
3L:15998000-15998150:plus -20.9184 -1.66972 4.48624 3.54128 -17.4184 -26.5612 -16.1468 5.13889 -2.26606
0.00181 0.0176 0.0022 0.00702 0.00457 0.0237 0.0111 0.00247 0.0359
3L:16004840-16004990:minus -23.2245 8.54128 -2.86239 0.844037 1.87755 -9.16327 -6.13761 3.44444
-0.293578 0.00576 0.000285 0.0277 0.0215 0.0000492 0.00223 0.00136 0.00502 0.0207
3L:16004840-16004990:plus -14.4898 6.55963 2.15596 0.385321 -18.7959 -17.2755 -15.0642 4.47222 1.69725
0.00143 0.00081 0.00545 0.0243 0.0148 0.00544 0.00909 0.0033 0.0108
3L:1602100-1602250:minus -23.2959 5.93578 9.30275 2.53211 -7.82653 -8.79592 -17.6055 -2.08333 4.91743
0.00605 0.00109 0.000213 0.0113 0.00059 0.0019 0.0141 0.0305 0.00321
3L:1602100-1602250:plus -23.1837 1.44037 9.38532 3.69725 -26.5918 -18.7857 -10.1284 -0.958333
6.7156 0.00553 0.00617 0.000203 0.00665 0.0186 0.0146 0.00315 0.0223 0.00144
3L:1602660-1602810:minus -23.9184 -0.165138 0.376147 2.61468 20.1735 -9.02041 -13.7156 6.375
-1.43119 0.00695 0.0109 0.0102 0.0109 5.68e-07 0.00213 0.00681 0.00138 0.029
3L:1602660-1602810:plus -31.6633 0.275229 4.37615 11.2936 -17.0816 -9.23469 -20.1009 -0.861111
-7.51376 0.0141 0.00938 0.0023 0.0000139 0.00334 0.00238 0.021 0.0217 0.124
3L:16039280-16039430:minus -4.26531 -1.97248 2.52294 2.33945 -18.7551 0.897959 -18.6147 10.3889

1.27523 0.000132 0.0193 0.00476 0.0122 0.0144 0.000144 0.0164 0.000123 0.013
3L:16039280-16039430:plus -21.8163 3.49541 2.6789 -0.155963 -17.5612 -8.5 -26.1284 0.0972222
-7.41284 0.00244 0.0029 0.00449 0.0282 0.00544 0.00126 0.0543 0.0162 0.122
3L:16044160-16044310:minus -22.8878 -2.10092 11.6606 2.66055 -17.9796 -0.244898 -6.87156 4.875
1.72477 0.00441 0.02 0.0000539 0.0106 0.00726 0.000496 0.00164 0.00277 0.0106
3L:16044160-16044310:plus -22.2959 7.15596 4.41284 5.30275 -27.2653 -17.8571 -3.22018 6 -0.688073
0.00335 0.000599 0.00227 0.00396 0.0302 0.00719 0.000602 0.00166 0.0238
3L:16054440-16054590:minus -12.7347 -0.366972 -4.72477 6.2844 -18.1837 -27.0408 -0.0366972
-1.73611 1.43119 0.000468 0.0117 0.046 0.00205 0.00835 0.0307 0.000286 0.0277 0.0121
3L:16054440-16054590:plus -31.4082 -3.42202 3.78899 2.34862 -27.1939 -26.5306 -14.3761 2.80556 2.46789
0.0119 0.0289 0.00292 0.0121 0.0267 0.023 0.00788 0.00642 0.00805
3L:16080660-16080810:minus -21.9286 0.46789 2.82569 7.47706 10.9898 0.979592 -3.22936 2.34722
0.743119 0.0027 0.00876 0.00424 0.00085 3.28e-06 0.000136 0.000603 0.00761 0.0153
3L:16080660-16080810:plus -31.2143 -0.385321 7.51376 1.31193 -7.93878 -9.02041 -22.3394 5.65278
1.6422 0.0111 0.0117 0.000563 0.0189 0.000767 0.00213 0.0313 0.00195 0.0111
3L:16083480-16083630:minus -3.59184 3.90816 -2.2844 7.00917 -8.60204 -8.23469 -13.0092 0.208333
-0.40367 0.0000835 0.00102 0.0235 0.00126 0.00125 0.000875 0.00579 0.0157 0.0213
3L:16083480-16083630:plus -22.1122 13.4592 -3.88991 4.86239 20.2041 -8.45918 -12.0642 0.791667 -7.20183
0.00304 0.000064 0.0369 0.00449 3.64e-07 0.00111 0.00468 0.013 0.118
3L:160840-160990:minus -22.9592 2.53211 -3.84404 3.50459 -27.2653 10.602 -1.31193 5.43056 3.61468
0.00475 0.00413 0.0364 0.00713 0.0302 4.1e-06 0.00038 0.00216 0.00526
3L:160840-160990:plus -23.449 2.91743 0.880734 -0.522936 -27.2653 -0.244898 -19.3761 4.63889
4.73394 0.00631 0.00358 0.00861 0.0313 0.0302 0.000496 0.0185 0.00307 0.00342
3L:16093420-16093570:minus -23.2245 -1.09174 2.0367 2.70642 -26.898 -17.3469 -11.5229 6.93056
6.18349 0.00576 0.0148 0.0057 0.0103 0.0203 0.0058 0.00416 0.00104 0.00167
3L:16093420-16093570:plus -30.2449 -0.0642202 -0.0183486 6.21101 -18.3776 -27.051 -5.94495 -5.58333
4.02752 0.00881 0.0105 0.0117 0.00213 0.00926 0.0315 0.00129 0.0698 0.00428
3L:16098760-16098910:minus -20.551 1.17431 -3.53211 6.08257 -9.20408 -9.0102 -9.42202 4.23611
-1.79817 0.00168 0.00679 0.0334 0.00236 0.00262 0.00208 0.00277 0.00364 0.0315
3L:16098760-16098910:plus -23.1429 -1.54128 3.05505 2.11009 -8.86735 -8.45918 -25.211 0.180556 -7.31193
0.00533 0.0169 0.00389 0.0136 0.00173 0.00111 0.0485 0.0158 0.12
3L:16105220-16105370:minus -32.9286 -3.13761 4.16514 3.04587 -26.9694 -28.449 -10.2202 0.375
5.7156 0.025 0.0268 0.00251 0.00883 0.023 0.0616 0.00321 0.0149 0.00217
3L:16105220-16105370:plus -30.8878 13.5306 4.3211 3.54128 -7.60204 -26.051 -21.2385 0.319444 -2.15596
0.0103 0.0000206 0.00235 0.00702 0.000466 0.0216 0.0257 0.0151 0.0348
3L:16105400-16105550:minus -34 -0.513761 -1.59633 2.88991 20.1735 -17.3469 -7.17431 -0.847222
3.24771 0.0307 0.0123 0.0192 0.00942 5.68e-07 0.0058 0.00176 0.0216 0.00636
3L:16105400-16105550:plus -42.6327 -2.77982 -2.49541 1.57798 -9.19388 0.0510204 -23.9633 0.805556
-1.07339 0.0829 0.0243 0.025 0.0171 0.00253 0.000349 0.0408 0.013 0.0267
3L:16105720-16105870:minus -23.2959 4.58716 -0.376147 4.43119 -9.0102 10.8673 -13.055 -0.263889
-1.14679 0.00605 0.00192 0.0131 0.00495 0.00236 2.19e-06 0.00585 0.0181 0.0272
3L:16105720-16105870:plus -13.2959 14.7339 1.13761 4.37615 -18.1939 -8.57143 -14.7248 5.84722 -0.449541
0.000775 3.57e-06 0.00788 0.00504 0.0086 0.00149 0.00848 0.00178 0.0216
3L:16106220-16106370:minus -30.9694 1.50459 2.55046 -0.40367 -28.2653 -9.53061 -10.8349 4.13889
-3.50459 0.0104 0.00602 0.00471 0.0302 0.0537 0.0031 0.00361 0.0038 0.0497
3L:16106220-16106370:plus -21.6633 -0.908257 0.559633 6.25688 20.4694 -18.5 -12.4587 1.02778 -5
0.00223 0.0139 0.00961 0.00208 1.65e-07 0.0131 0.00511 0.012 0.0727
3L:16106560-16106710:minus -12.9286 -4.86239 4.05505 3.85321 -18.7143 -8.79592 -8.2844 2.81944
1.55963 0.000539 0.0426 0.00262 0.00623 0.0136 0.0019 0.00223 0.00639 0.0114
3L:16106560-16106710:plus -33.1122 2.77064 -4.23853 3.78899 -18.6735 -27.5612 -17.4679 1.01389 -0.651376
0.0271 0.00378 0.0405 0.00632 0.0123 0.0393 0.0138 0.0121 0.0235
3L:16115000-16115150:minus -32.7041 0.853211 1.05505 6.00917 -27.1939 -18.898 -13.6422 5.93056
4.92661 0.0222 0.00763 0.00811 0.00255 0.0267 0.0152 0.00669 0.00171 0.00318
3L:16115000-16115150:plus -43.8878 4.74312 0.816514 1.53211 -18.9796 -26.8265 -25.2844 5.875 3.46789

0.0943 0.00181 0.0088 0.0174 0.0155 0.0285 0.0489 0.00176 0.00582
3L:16121560-16121710:minus -22.2245 3.55046 4.14679 7 -7.89796 -18.8265 -9.77982 -2.16667 -0.33945
0.00325 0.00284 0.00252 0.00132 0.000678 0.0148 0.00295 0.0312 0.0209
3L:16121560-16121710:plus -30.7041 1.90826 0.0275229 9.87156 -18.7143 -18.7857 -22.3303 2.65278
1.19266 0.00993 0.00519 0.0115 0.000195 0.0136 0.0146 0.0313 0.0068 0.0134
3L:16122020-16122170:minus -33.0408 1.6422 0.449541 4.31193 -17.8265 0.0204082 -19.5505 3.55556
2.09174 0.0264 0.00573 0.00998 0.00516 0.00691 0.000377 0.0191 0.00481 0.00904
3L:16122020-16122170:plus -21.6633 -3.20183 12.6055 2 -27.5612 -18.5 -15.4587 2.01389 8.95413
0.00223 0.0273 0.000028 0.0143 0.0354 0.0131 0.00982 0.00858 0.000458
3L:16124600-16124750:minus -40.551 -2.13761 -1.73394 1.56881 20.2041 0.0510204 -28.1284 0.458333
-5.6055 0.0418 0.0202 0.02 0.0172 3.64e-07 0.000349 0.07 0.0145 0.0821
3L:16124600-16124750:plus -33 3.90816 -2.14679 1.6055 -27.0102 -18.051 -18.156 5.08333 3.51376
0.0258 0.00102 0.0226 0.0169 0.0237 0.00886 0.0153 0.00253 0.00566
3L:16125100-16125250:minus -31.5918 -3.72477 2.17431 2.70642 -18.7143 -18.3469 -12.9817 4.15278
4.10092 0.0136 0.0314 0.00542 0.0103 0.0136 0.0124 0.00576 0.00377 0.00413
3L:16125100-16125250:plus -23.9592 2.81651 3.93578 5.58716 -27.3061 -17.7857 -13.1743 1.31944 7.63303
0.00708 0.00372 0.00275 0.00334 0.0315 0.0068 0.00602 0.0109 0.000928
3L:16125260-16125410:minus -31.5918 -3.72477 2.17431 2.70642 -18.7143 -18.3469 -12.9817 4.15278
4.10092 0.0136 0.0314 0.00542 0.0103 0.0136 0.0124 0.00576 0.00377 0.00413
3L:16125260-16125410:plus -23.9592 2.81651 3.93578 5.58716 -27.3061 -17.7857 -13.1743 1.31944 7.63303
0.00708 0.00372 0.00275 0.00334 0.0315 0.0068 0.00602 0.0109 0.000928
3L:16151380-16151530:minus -14.5918 3.22018 7.82569 5.31193 -26.9286 -18.051 -16.4771 2.16667
5.23853 0.00148 0.00321 0.00048 0.00393 0.0214 0.00886 0.0118 0.00812 0.00285
3L:16151380-16151530:plus -23.8571 7.86239 4.3211 1.21101 -9.23469 -17.9796 -23.1927 8.56944 6.7156
0.00687 0.000411 0.00235 0.0194 0.00277 0.00764 0.0362 0.000418 0.00144
3L:16152520-16152670:minus -31.4388 13.4592 -2.62385 3.77064 -9.0102 -18.0102 -6.78899 -0.0416667
2.33028 0.0122 0.000064 0.0259 0.00637 0.00236 0.00812 0.0016 0.017 0.00836
3L:16152520-16152670:plus -39.9592 -0.229358 2.95413 1.41284 -19.0204 0.122449 -14.6789 3.75 0.348624
0.0348 0.0111 0.00404 0.0183 0.0158 0.000312 0.0084 0.00445 0.0167
3L:16153820-16153970:minus -21.7449 -0.605505 -1.46789 2.80734 -18.3776 -19.0816 -15.7156 3
2.99083 0.00236 0.0126 0.0184 0.00985 0.00926 0.017 0.0103 0.00597 0.00706
3L:16153820-16153970:plus -33.1122 -1.12844 -1.57798 1.12844 -17.7143 -8.86735 -11.5596 1.375 -3.12844
0.0271 0.0149 0.019 0.0198 0.00627 0.00198 0.00419 0.0107 0.046
3L:16155280-16155430:minus -31.5102 1.6055 4.21101 3.30275 -17.4184 -26.7857 -11.6422 -0.0416667
2.25688 0.013 0.0058 0.00246 0.00789 0.00457 0.0266 0.00427 0.017 0.00853
3L:16155280-16155430:plus -23.449 2.0367 7.52294 1.6422 -18.4592 -26.602 -7.16514 -0.25 5.70642
0.00631 0.00495 0.00056 0.0166 0.0109 0.0248 0.00175 0.0181 0.00222
3L:16156540-16156690:minus -23.8878 1.68807 2.10092 2.6422 -17.4082 -26.8571 -11.6422 9.91667
5.18349 0.00692 0.00563 0.00556 0.0107 0.00421 0.0288 0.00427 0.000174 0.00295
3L:16156540-16156690:plus -22 8.44037 3.93578 3.02752 1.87755 -18.1224 -14.6514 2.77778 4.98165
0.00291 0.000301 0.00275 0.00887 0.0000492 0.00988 0.00835 0.00649 0.00313
3L:16157760-16157910:minus -13 3.04587 -2.25688 4.0367 -27.4592 -7.42857 -0.155963 -1.625
2.78899 0.000563 0.00342 0.0233 0.00578 0.0329 0.000567 0.000294 0.0269 0.00745
3L:16157760-16157910:plus -32.9592 0.201835 4.06422 -0.715596 -18.7449 -8.72449 -23.3028 4.98611
-4.87156 0.0252 0.00962 0.00261 0.0336 0.0138 0.00169 0.0369 0.00264 0.0708
3L:1618280-1618430:minus -13.2143 11.367 -0.33945 2.29358 -18.2653 -17.9796 -3.7156 0.555556 7.04587
0.000714 0.000067 0.0129 0.0124 0.00882 0.00764 0.000689 0.014 0.00132
3L:1618280-1618430:plus -39.3265 -0.275229 13.1284 -0.633028 -17.4184 -8.94898 -12.1284 -2.38889
9.97248 0.0332 0.0113 0.0000181 0.0327 0.00457 0.00203 0.00474 0.0331 0.00016
3L:16221820-16221970:minus -32.398 -1.9633 7.20183 10.8624 -17.4184 -9.45918 -20.8257 -1.38889
1.82569 0.019 0.0192 0.000658 0.0000687 0.00457 0.00279 0.0238 0.0252 0.0101
3L:16221820-16221970:plus -22.9592 -3.61468 5.81651 4.33028 -18.1122 -0.0204082 -15.3853 3.72222
-1.41284 0.00475 0.0305 0.00125 0.00511 0.00771 0.000411 0.00968 0.0045 0.0289
3L:16222080-16222230:minus -41.7347 0.0275229 -4.44037 0.981651 -27.7551 -17.3469 -18.9817 5.75

1.74312 0.0643 0.0102 0.0427 0.0207 0.0385 0.0058 0.0174 0.00187 0.0105
3L:16222080-16222230:plus -22.3776 -2.06422 7.22936 9.75229 0.612245 0.755102 -20.4771 -0.847222
5.84404 0.00342 0.0198 0.000649 0.000226 0.000269 0.000175 0.0224 0.0216 0.00202
3L:16226140-16226290:minus -22.2551 3.89908 14.055 5.06422 -8.93878 -10.0204 -14.3578 1.86111
-2.52294 0.00327 0.0025 6.79e-06 0.00433 0.00216 0.00399 0.00785 0.00905 0.0391
3L:16226140-16226290:plus -15.0102 6.08257 14.9633 9.08257 -18.7143 -9.53061 -17.6789 2.79167 -6.53211
0.00162 0.00102 1.77e-06 0.000444 0.0136 0.0031 0.0142 0.00646 0.102
3L:16345040-16345190:minus -31.8061 -1.63303 14.6881 2.00917 -19.0102 -19.2755 -11.5872 -1.98611
6.69725 0.0158 0.0174 3.06e-06 0.0142 0.0157 0.0185 0.00422 0.0297 0.00145
3L:16345040-16345190:plus -20.4388 -2.77064 2.81651 3.9633 1.54082 -0.0204082 -13.5596 -2.69444
-0.247706 0.00167 0.0242 0.00426 0.0059 0.0000712 0.000411 0.00657 0.0358 0.0204
3L:16352840-16352990:minus -14.0408 6.3578 -4.20183 0.990826 -28.1224 0.0510204 -12.5321 7.59722
-3.87156 0.00115 0.000892 0.0401 0.0206 0.0457 0.000349 0.00519 0.000731 0.0537
3L:16352840-16352990:plus -13.5612 -4.17431 -4.11009 6.82569 -8.67347 9.72449 -11.4862 3.77778 9.04587
0.000984 0.0354 0.0391 0.00144 0.00154 0.0000214 0.00413 0.0044 0.000423
3L:16370560-16370710:minus -41.1837 2.23853 -0.642202 4.02752 -18.7143 -9.23469 -29.9817 6.29167
1.70642 0.0485 0.0046 0.0143 0.00581 0.0136 0.00238 0.0907 0.00144 0.0108
3L:16370560-16370710:plus -33.5612 0.238532 1.46789 5.16514 -27.2653 -8.72449 -24.5138 0.513889
-0.0183486 0.0288 0.0095 0.007 0.00424 0.0302 0.00169 0.0442 0.0142 0.0185
3L:16371000-16371150:minus -22.7755 -0.816514 12.0459 -0.642202 -17.3776 -19.2755 -17.3028
0.694444 5.65138 0.00412 0.0135 0.0000414 0.0328 0.00419 0.0185 0.0134 0.0134 0.00228
3L:16371000-16371150:plus -31 1.55963 1.20183 3.95413 -17.5612 -17.9388 -13.4312 2.04167 3.98165
0.0105 0.0059 0.0077 0.00593 0.00544 0.00738 0.00638 0.00849 0.00439
3L:16371620-16371770:minus -12.7755 -0.311927 7.24771 7.61468 10.6531 -9.53061 -10.9541 -4.55556
-4.09174 0.000477 0.0115 0.000644 0.000779 0.0000102 0.0031 0.0037 0.0559 0.0568
3L:16371620-16371770:plus -31.1429 7.69725 13.5872 7.45872 1.87755 -17.3061 -17.3028 -2.59722 -1.49541
0.0108 0.00045 0.0000122 0.000894 0.0000492 0.00548 0.0134 0.0349 0.0293
3L:16374600-16374750:minus -31.6633 1.01835 2.27523 3.88073 -26.9592 -18.3469 -11.7706 8.63889
-3.61468 0.0141 0.00719 0.00522 0.0061 0.0215 0.0124 0.00439 0.000401 0.0509
3L:16374600-16374750:plus -40.1735 1.46789 5.87156 5.31193 -7.89796 -27.5612 -13.9358 0.125 -0.981651
0.0363 0.0061 0.00122 0.00393 0.000678 0.0393 0.00715 0.0161 0.026
3L:16378560-16378710:minus -13.7041 4.9633 4.7156 4.14679 -18.2245 -9.72449 -9.40367 4.79167
-0.449541 0.00104 0.00165 0.002 0.00542 0.00877 0.00352 0.00276 0.00288 0.0216
3L:16378560-16378710:plus -12.9694 -4.00917 10.156 7.45872 -26.5306 -9.79592 -18.0459 0.277778 7.73394
0.000552 0.0339 0.00013 0.000894 0.0185 0.00377 0.0151 0.0153 0.000784
3L:16378980-16379130:minus -13.6735 3.68367 -1 5.86239 1.10204 -16.9388 -11.9633 4.02778 1.95413
0.00103 0.00144 0.016 0.00282 0.000124 0.00433 0.00457 0.00397 0.00964
3L:16378980-16379130:plus -39.2143 2.90826 -0.917431 4.02752 -17.4592 18.8571 -19.1651 0.166667
-0.926606 0.0329 0.0036 0.0156 0.00581 0.00491 1.47e-06 0.0179 0.0159 0.0256
3L:1639380-1639530:minus -32 7.68807 3.01835 9.51376 -17.7143 -17.7857 -11.1743 -0.444444 -1.21101
0.0171 0.000452 0.00394 0.000292 0.00627 0.0068 0.00387 0.0192 0.0277
3L:1639380-1639530:plus -13.0408 4.80734 7.72477 10.5872 -18.1939 0.204082 -8.85321 4.98611 -1.06422
0.000576 0.00176 0.000506 0.000115 0.0086 0.000296 0.00249 0.00264 0.0266
3L:16404880-16405030:minus -11.9286 -1.0367 0.0183486 5.59633 -17.9388 -18.5 -14.1927 -4.75
9.87156 0.000296 0.0145 0.0115 0.00331 0.00715 0.0131 0.00757 0.0584 0.000217
3L:16404880-16405030:plus -22.4082 3.64286 -0.376147 4.48624 -18.449 -26.602 -9.70642 4.59722
-0.12844 0.00345 0.00159 0.0131 0.00487 0.0107 0.0248 0.00291 0.00313 0.0193
3L:16405580-16405730:minus -34.3367 0.623853 5.17431 6.21101 -26.6633 -19.2041 -22 -2.29167 1.27523
0.0318 0.00829 0.00165 0.00213 0.0189 0.0179 0.0295 0.0322 0.013
3L:16405580-16405730:plus -23.3673 3.90816 0.00917431 6.11009 -9.16327 -9.72449 -24.6697 6.63889
0.495413 0.00617 0.00102 0.0116 0.00233 0.00249 0.00352 0.0451 0.00121 0.0162
3L:16412620-16412770:minus -13.0714 -2.77982 14.7156 6.44037 -8.86735 -18.2041 -19.3853 1.66667
-0.59633 0.000594 0.0243 2.9e-06 0.00185 0.00173 0.0103 0.0186 0.00969 0.0229
3L:16412620-16412770:plus -11.5918 -0.192661 3.31193 9.44037 10.3571 -8.79592 -21.7064 8.04167

-0.40367 0.000257 0.011 0.00352 0.000339 0.0000208 0.0019 0.028 0.00057 0.0213
3L:1645120-1645270:minus -30.7449 2.11009 4.65138 2.57798 0.836735 9.89796 -20.8257 -0.0277778
0.614679 0.0101 0.00482 0.00206 0.011 0.000215 0.0000111 0.0238 0.0169 0.0158
3L:1645120-1645270:plus -31.8878 0 -0.513761 2.62385 -8.97959 -7.72449 -23.1927 0.472222 -0.541284
0.0164 0.0103 0.0137 0.0108 0.0023 0.00068 0.0362 0.0144 0.0223
3L:16467140-16467290:minus -42.2857 -1.26606 0.366972 1.55963 -18.5204 -17.9796 -20.8624 1.05556
9.61468 0.0731 0.0156 0.0103 0.0173 0.0117 0.00764 0.024 0.0119 0.00029
3L:16467140-16467290:plus -42.4796 2.13761 3.88073 6.00917 -27.4184 -17.3469 -22.4404 5.5 -4.69725
0.0777 0.00477 0.00281 0.00255 0.0323 0.0058 0.0319 0.00209 0.0673
3L:16467640-16467790:minus -31.5918 -0.449541 2.22018 2.43119 -17.7551 -8.65306 -26.0459 -3.09722
-1.6422 0.0136 0.012 0.00532 0.0117 0.00658 0.00156 0.0538 0.0396 0.0304
3L:16467640-16467790:plus -31.2959 -2.37615 4.6055 5.31193 -7.63265 -18.051 -10.7523 3.48611 8.19266
0.0113 0.0216 0.0021 0.00393 0.000479 0.00886 0.00355 0.00494 0.000548
3L:16469360-16469510:minus -41.551 2.13761 1.92661 2.97248 -8.7449 -9.08163 -14.4128 4.94444
-0.642202 0.0607 0.00477 0.00594 0.00909 0.00161 0.00216 0.00794 0.00269 0.0234
3L:16469360-16469510:plus -33.0714 -0.12844 -2.13761 2.37615 -16.9286 -17.5714 -19.7064 6.47222 -1.88073
0.0267 0.0108 0.0225 0.0119 0.00327 0.00616 0.0196 0.00132 0.0321
3L:16470280-16470430:minus -32.0102 -1.84404 -3.78899 2.10092 -28.0102 -8.57143 -30.0642 3.98611
-0.12844 0.0172 0.0186 0.0359 0.0136 0.0449 0.00149 0.0917 0.00404 0.0193
3L:16470280-16470430:plus -33.0408 -3.59633 2.97248 5.49541 -18.2245 -8.42857 0.53211 -1.40278 2.93578
0.0264 0.0303 0.00401 0.00355 0.00877 0.00104 0.000249 0.0253 0.00717
3L:16474020-16474170:minus -31.3673 -4.13761 4.9633 1.65138 -18.2653 -27.7449 -15.5963 -0.986111
9.04587 0.0116 0.0351 0.00181 0.0166 0.00882 0.0448 0.0101 0.0225 0.000423
3L:16474020-16474170:plus -23.5306 -2.3211 1.09174 5.90826 -18.6837 1.05102 -18.1193 6.80556 0.0183486
0.00646 0.0213 0.00801 0.00271 0.0126 0.000107 0.0152 0.00111 0.0183
3L:16474860-16475010:minus -23.449 3.7551 4.36697 6.00917 -17.4898 -8.79592 -18.5138 5.83333
11.4771 0.00631 0.00122 0.00231 0.00255 0.0053 0.0019 0.0162 0.00179 0.0000679
3L:16474860-16475010:plus 14.4694 3.88991 -0.770642 7.62385 -17.3469 -8.5 -5.80734 3 -0.688073
1.23e-06 0.00251 0.0149 0.000759 0.00394 0.00126 0.00124 0.00597 0.0238
3L:16476640-16476790:minus -1.73469 4.90826 2.36697 9.44954 -18.4184 -8.72449 -16.0917 -2.73611
9.34862 0.0000234 0.00169 0.00505 0.000326 0.0101 0.00169 0.011 0.0362 0.000371
3L:16476640-16476790:plus -32.3367 2.48624 1.06422 6.02752 -18.4898 -10.0204 -22.7798 7.08333 7.88991
0.0187 0.0042 0.00808 0.00246 0.0116 0.00399 0.0338 0.000963 0.000688
3L:16477160-16477310:minus -42.9694 0.0366972 -2.3945 1.89908 -27.5306 -17.2755 -27.9083 5.54167
-3.81651 0.0887 0.0102 0.0242 0.0151 0.0351 0.00544 0.068 0.00205 0.0529
3L:16477160-16477310:plus -23.8878 1.82569 3.2844 0.926606 -18.7551 -17.9388 -23.5596 1.125 9.92661
0.00692 0.00535 0.00356 0.0209 0.0144 0.00738 0.0384 0.0117 0.000193
3L:16486960-16487110:minus -11.5816 -0.944954 2.04587 6.51376 -18.3776 -9.0102 -8.55046 3.97222
4.10092 0.000256 0.0141 0.00568 0.00175 0.00926 0.00208 0.00235 0.00407 0.00413
3L:16486960-16487110:plus -23.0408 3.64286 3.11927 1.91743 -18.6429 -8.65306 -9.75229 -4.41667 -2.37615
0.00512 0.00159 0.00379 0.015 0.0121 0.00156 0.00294 0.0542 0.0373
3L:16487840-16487990:minus -13.8571 1.37615 6.05505 3.62385 -19.051 -26.898 -0.146789 5.41667
3.20183 0.0011 0.00631 0.00113 0.00673 0.0162 0.0302 0.000293 0.00218 0.0065
3L:16487840-16487990:plus -33.0714 -1.23853 0.944954 1.14679 -17.7143 -9.82653 -14.055 8.26389 -6.05505
0.0267 0.0154 0.00842 0.0198 0.00627 0.00381 0.00734 0.000502 0.09
3L:16488320-16488470:minus -40.0714 -3.80734 0.834862 0.183486 -19.051 -8.5 -17.2385 3.45833 8.97248
0.0359 0.0321 0.00875 0.0256 0.0162 0.00126 0.0133 0.005 0.000449
3L:16488320-16488470:plus -33.1429 3.64286 3.83486 5.66055 -7.93878 9.53061 0.642202 9.44444 -1.01835
0.0272 0.00159 0.00287 0.00317 0.000767 0.0000316 0.000242 0.00024 0.0263
3L:1652820-1652970:minus -23 2.48624 -0.724771 5.42202 10.7245 0.469388 -21.1743 0.805556 -2.15596
0.00498 0.0042 0.0146 0.00374 5.2e-06 0.000212 0.0254 0.013 0.0348
3L:1652820-1652970:plus -22.9592 -3.29358 4.81651 10.7615 -26.9694 -9.42857 -2.63303 -1.94444 -2.51376
0.00475 0.028 0.00192 0.0000826 0.023 0.0027 0.000517 0.0294 0.039
3L:16567500-16567650:minus -31.4796 13.3878 15.6972 7.70642 -27.898 -7.79592 -11.2018 -0.138889

0.568807 0.0128 0.0000869 3.11e-07 0.000708 0.04 0.000723 0.00389 0.0175 0.016
3L:16567500-16567650:plus -32.9286 -1.45872 6.27523 3.08257 0.571429 0.826531 -23.1927 -1.01389 2.0367
0.025 0.0165 0.00102 0.0087 0.0003 0.000153 0.0362 0.0227 0.00923
3L:16573260-16573410:minus -23.2245 0.770642 -1.05505 0.844037 -19.0102 -17.9796 -15.5505 5.73611
-2.52294 0.00576 0.00786 0.0162 0.0215 0.0157 0.00764 0.00999 0.00188 0.0391
3L:16573260-16573410:plus -31.7347 4.18349 0.330275 4.30275 -8.97959 -18.1224 -5.55046 -0.222222
-3.88991 0.0149 0.00224 0.0104 0.00517 0.0023 0.00988 0.00116 0.0179 0.054
3L:16574520-16574670:minus -12.3776 13.4592 -1.22018 5.49541 -9.19388 0.755102 -18.2477 12.1111
3.46789 0.000403 0.000064 0.0171 0.00355 0.00253 0.000175 0.0155 0.0000278 0.00582
3L:16574520-16574670:plus -24.5918 13.4592 1.69725 4.04587 -18.7449 -18.0102 -5.33945 4.72222 7.42202
0.00821 0.000064 0.00646 0.00572 0.0138 0.00812 0.00109 0.00296 0.00106
3L:16575960-16576110:minus -34.2347 13.5306 -1.57798 3.77064 -18.9796 -17.1224 -20.6697 3.31944
-1.7156 0.0315 0.0000206 0.019 0.00637 0.0155 0.00499 0.0232 0.00528 0.0309
3L:16575960-16576110:plus -32.7041 7.6055 3.77064 2.00917 -27.1939 -18.051 -13.3303 4.5 5.23853
0.0222 0.000472 0.00294 0.0142 0.0267 0.00886 0.00624 0.00326 0.00285
3L:16577380-16577530:minus -3 -1.25688 -3.11927 3.76147 -8.33673 -18.3469 -17.1651 -1.58333 7.11009
0.000041 0.0155 0.0298 0.00645 0.00101 0.0124 0.0132 0.0266 0.00128
3L:16577380-16577530:plus -31.2857 0.504587 -3.2844 2.43119 10.6224 -16.3469 -13.1193 0.236111 7.06422
0.0113 0.00865 0.0312 0.0117 0.0000113 0.00416 0.00594 0.0155 0.00131
3L:16580160-16580310:minus -23.1531 1.22936 14.422 7.29358 -26.9286 -8.65306 -27.2936 -4.16667
-1.11927 0.00542 0.00666 4.18e-06 0.00104 0.0214 0.00156 0.0628 0.0512 0.027
3L:16580160-16580310:plus -33.6633 1.02752 4 3.6055 0.877551 -17.3469 -16.5046 0.708333 -2.65138
0.0291 0.00717 0.00268 0.00686 0.00019 0.0058 0.0118 0.0134 0.0406
3L:16581280-16581430:minus -31.7347 0.201835 2.3945 1.16514 10.3571 0.204082 -23.0459 9.06944
-3.62385 0.0149 0.00962 0.00499 0.0197 0.0000208 0.000296 0.0353 0.000307 0.051
3L:16581280-16581430:plus -30.7755 0.715596 -0.908257 0.366972 -7.97959 -17.5714 -22.6881 5.47222
1.59633 0.0102 0.00802 0.0155 0.0244 0.000799 0.00616 0.0333 0.00212 0.0113
3L:16583960-16584110:minus -41.6633 1.2844 5.49541 3.87156 -18.2245 -27.5612 -18.5229 -2.31944
7.68807 0.0636 0.00653 0.00144 0.00618 0.00877 0.0393 0.0162 0.0325 0.000841
3L:16583960-16584110:plus -22.2653 1.2844 8.62385 5.57798 0.540816 -17.5612 -6.16514 7.41667 -4.18349
0.00331 0.00653 0.000311 0.0034 0.000321 0.00604 0.00137 0.000807 0.0581
3L:16588280-16588430:minus -30.4388 7.23853 -1.13761 4.79817 1.21429 -10.0204 -14.3303 1.48611
3.6789 0.00923 0.000573 0.0167 0.00453 0.0000864 0.00399 0.0078 0.0103 0.00506
3L:16588280-16588430:plus -31.9592 4.20183 0.862385 1.99083 -18.7551 -26.898 -13.945 2.22222 -0.155963
0.0167 0.00223 0.00867 0.0144 0.0144 0.0302 0.00717 0.00796 0.0195
3L:16595840-16595990:minus -31.6224 7.23853 -1.78899 6.56881 -18.7449 -10.0204 -13.8991 1.48611
3.6789 0.0137 0.000573 0.0203 0.0017 0.0138 0.00399 0.00709 0.0103 0.00506
3L:16595840-16595990:plus -22.7245 0.46789 -5.01835 6.12844 -8.71429 -16.9796 -5.7156 -3.04167 -2.75229
0.00395 0.00876 0.0496 0.00231 0.00159 0.0044 0.00121 0.0391 0.0418
3L:16602340-16602490:minus -13.3367 0.477064 5.44954 10.1468 -17.4184 -17.9388 -22.5138 -3.63889
1.99083 0.0008 0.00873 0.00147 0.000128 0.00457 0.00738 0.0323 0.0452 0.00946
3L:16602340-16602490:plus -41.2143 -0.559633 -1.02752 -1.51376 -26.9694 -8.65306 -3.02752 1 1.95413
0.0494 0.0124 0.0161 0.0436 0.023 0.00156 0.000572 0.0121 0.00964
3L:16603480-16603630:minus -32 13.4592 2.74312 5.43119 10.6531 -8.65306 -18.633 -0.0555556
-3.00917 0.0171 0.000064 0.00438 0.00371 0.0000102 0.00156 0.0165 0.017 0.0447
3L:16603480-16603630:plus -22.8571 14.0917 -0.733945 6.08257 -28.1939 9.53061 -23.2569 1.66667
-2.74312 0.00427 7.62e-06 0.0147 0.00236 0.0482 0.0000316 0.0366 0.00969 0.0417
3L:16605280-16605430:minus -32.7041 0.651376 3.47706 2.6789 0.653061 10.602 -11.4587 6.63889
-3.04587 0.0222 0.00821 0.0033 0.0105 0.000239 4.1e-06 0.0041 0.00121 0.0451
3L:16605280-16605430:plus -21.9592 -2.51376 5.66972 1.78899 -27.5306 9.60204 -7.16514 4.02778 -4.76147
0.00279 0.0225 0.00134 0.0158 0.0351 0.0000248 0.00175 0.00397 0.0686
3L:16606120-16606270:minus -32.4388 0.0183486 1.19266 1.9633 -18.3776 -26.9694 -12.8991 -2.34722
4.19266 0.0195 0.0102 0.00773 0.0146 0.00926 0.0304 0.00565 0.0327 0.00401
3L:16606120-16606270:plus -21.9388 -7.04587 -0.00917431 -1.47706 -8.67347 -9.53061 -18.578 -0.958333

5.7156	0.0027	0.0738	0.0116	0.0429	0.00154	0.0031	0.0163	0.0223	0.00217
3L:16623760-16623910:minus	-14.5612	-0.899083	1	1.68807	-18.2245	-8.27551	-21.1193	2.75	1.90826
0.00147	0.0139	0.00827	0.0164	0.00877	0.000919	0.0251	0.00656	0.00974
3L:16623760-16623910:plus	-38.6939	6.72477	3.99083	9.49541	-18.6837	-9.79592	-28.0459	3.01389	4.30275
0.0321	0.000747	0.00269	0.000308	0.0126	0.00377	0.0692	0.00593	0.00388
3L:16624120-16624270:minus	-34.5612	-2.33028	-0.743119	5.74312	-17.9796	-8.09184	-26.7706	2.93056
-5.99083	0.032	0.0214	0.0147	0.00302	0.00726	0.000762	0.0588	0.00612	0.0886
3L:16624120-16624270:plus	-22.2245	5.58716	2.6422	7.3578	-27.4184	-9.82653	-14.055	10	-3.49541
0.00325	0.00127	0.00455	0.000965	0.0323	0.00381	0.00734	0.000164	0.0495
3L:16625300-16625450:minus	-32.6633	-3.94495	5.88991	2.6422	-17.4184	-0.244898	-12.6422	3.31944
-0.137615	0.0216	0.0333	0.00121	0.0107	0.00457	0.000496	0.00533	0.00528	0.0194
3L:16625300-16625450:plus	-13.9184	4.02752	2.83486	1.00917	-17.0816	-19.051	-2.13761	0.694444	0.33945
0.00112	0.00238	0.00423	0.0205	0.00334	0.0165	0.000459	0.0134	0.0168
3L:16631520-16631670:minus	-20.7755	0.412844	0.293578	5.73394	-18.0204	-8.23469	-12.789	5.88889
-2.68807	0.00176	0.00893	0.0105	0.00307	0.00733	0.000875	0.00551	0.00175	0.041
3L:16631520-16631670:plus	-30.6735	0.614679	-1.36697	9.08257	-17.9796	-7.93878	-14.7982	4.06944	1.48624
0.00978	0.00832	0.0179	0.000444	0.00726	0.000736	0.00861	0.00391	0.0118
3L:16640980-16641130:minus	-12.8163	0.165138	-0.761468	4.11009	10.6224	-7.5	-3.80734	3.16667
0.293578	0.000489	0.00974	0.0148	0.00553	0.0000113	0.000614	0.000707	0.0056	0.017
3L:16640980-16641130:plus	-29.5918	13.4592	0.926606	7.77982	-27.2653	-8.93878	-23.2202	2.33333	-0.229358
0.00846	0.000064	0.00848	0.000652	0.0302	0.00201	0.0364	0.00765	0.0202
3L:16646800-16646950:minus	-20.9694	5.61468	10.6239	10.5963	-27.602	-8.42857	-18.5596	2.63889
-2.94495	0.00184	0.00126	0.0000983	0.0001	0.0364	0.00104	0.0163	0.00684	0.0441
3L:16646800-16646950:plus	-22.449	5.30275	6.59633	7	-18.0102	-27.5612	-7.44954	-4.15278	-2.68807
0.0035	0.00144	0.000876	0.00132	0.00732	0.0393	0.00187	0.051	0.041
3L:1665060-1665210:minus	-32.0408	0.229358	6.93578	3.26606	20.2041	-9.08163	-27.6147	3.33333	6.29358
0.0174	0.00953	0.00075	0.00802	3.64e-07	0.00216	0.0654	0.00525	0.00161
3L:1665060-1665210:plus	-40.3673	0.394495	-5.13761	2.0367	10.6224	-18.5714	-15.0826	-2.31944	3.24771
0.0397	0.00899	0.0511	0.0141	0.0000113	0.0136	0.00912	0.0325	0.00636
3L:16654620-16654770:minus	-3.81633	7.66972	0.788991	9.7156	-26.898	0.0918367	-5.49541	7.68056
-2.49541	0.000106	0.000456	0.00889	0.000242	0.0203	0.000323	0.00114	0.000698	0.0387
3L:16654620-16654770:plus	-11.6327	-6.06422	-1.33945	2.88991	-7.67347	-18.051	-13.0642	-4.25	-0.40367
0.000259	0.058	0.0177	0.00942	0.00055	0.00886	0.00587	0.0522	0.0213
3L:16660960-16661110:minus	-14.3673	0.605505	1.92661	3.31193	-19.051	-9.27551	-6.65138	5.43056
1.38532	0.00132	0.00834	0.00594	0.00786	0.0162	0.00244	0.00155	0.00216	0.0123
3L:16660960-16661110:plus	-23.4082	1.23853	1.3211	9.44954	0.877551	-17.0102	0.733945	2.09722	5.70642
0.00623	0.00664	0.00738	0.000326	0.00019	0.00453	0.000237	0.00832	0.00222
3L:16666580-16666730:minus	-23.8878	-1.20183	0.0917431	4.40367	10.0918	9.7551	-16.6514	4.08333
1.66055	0.00692	0.0153	0.0112	0.00497	0.0000251	0.0000198	0.0121	0.00388	0.011
3L:16666580-16666730:plus	-31.6735	5.80734	2.31193	-1.77064	-17.4184	0.459184	-18.5596	0.138889	-0.972477
0.0141	0.00116	0.00515	0.0473	0.00457	0.000213	0.0163	0.016	0.0259
3L:1668580-1668730:minus	-32.5816	-0.0642202	7.18349	9.49541	-18.4898	-9.68367	-18.6789	6.30556
2.82569	0.0208	0.0105	0.000665	0.000308	0.0116	0.00342	0.0166	0.00143	0.0074
3L:1668580-1668730:plus	-13.3776	-3.0367	4.82569	2.93578	0.836735	-8.45918	-21.5596	-0.541667
5.18349	0.000831	0.026	0.00191	0.00927	0.000215	0.00111	0.0272	0.0197	0.00295
3L:16691400-16691550:minus	-22.0714	3.97959	3.27523	2.78899	-17.3776	-27.2653	-14.8073	-2.90278
0.568807	0.00299	0.000669	0.00357	0.00992	0.00419	0.0333	0.00863	0.0377	0.016
3L:16691400-16691550:plus	-13.2959	3.06422	6.93578	7.37615	1.10204	-18.7857	1.12844	3.73611	5.23853
0.000775	0.0034	0.00075	0.000951	0.000124	0.0146	0.000213	0.00447	0.00285
3L:16692000-16692150:minus	-22.5918	6.77982	1.21101	4.13761	-16.7143	-18.1224	-6.93578	1.91667
0.642202	0.00365	0.000727	0.00768	0.00546	0.00319	0.00988	0.00166	0.00888	0.0157
3L:16692000-16692150:plus	-40.5204	-4.69725	-0.816514	5.30275	-27.5306	0.0918367	-27.6881	0.986111
-2.01835	0.0413	0.0407	0.0151	0.00396	0.0351	0.000323	0.066	0.0122	0.0334
3L:16703800-16703950:minus	-31.8163	-0.366972	6.97248	3.13761	-18.3469	0.387755	-15.3028	1.75

-2.78899 0.016 0.0117 0.000737 0.00854 0.00894 0.000217 0.00953 0.00941 0.0422
3L:16703800-16703950:plus -30.4388 4.33028 2.18349 2.27523 -17.2245 -19.5 -21.4495 -1.52778 -0.146789
0.00923 0.00212 0.0054 0.0125 0.00384 0.0199 0.0267 0.0262 0.0194
3L:16704700-16704850:minus -30.2449 7.25688 4.45872 6.54128 10.6939 -8.30612 12.5046 2.01389
2.93578 0.00881 0.000568 0.00223 0.00173 7.35e-06 0.000973 4.42e-06 0.00858 0.00717
3L:16704700-16704850:plus -31.9286 5.97248 1.02752 4.79817 -9.0102 -19.5714 -25.6697 6.66667 3.09174
0.0166 0.00107 0.00819 0.00453 0.00236 0.0202 0.0514 0.00119 0.00678
3L:16705040-16705190:minus -24.2959 5.91743 1.52294 2.3578 20.2041 -36.3776 -11.4037 3.13889
7.68807 0.00781 0.0011 0.00687 0.0121 3.64e-07 0.104 0.00406 0.00566 0.000841
3L:16705040-16705190:plus -14.3673 8.11009 3.3211 0.834862 -18.449 -8.16327 -22.1835 -3.26389 -1.77982
0.00132 0.000359 0.00351 0.0215 0.0107 0.000783 0.0305 0.0413 0.0314
3L:16723840-16723990:minus -4.55102 13.4592 0.155963 6.00917 -26.8163 -8.79592 -14.3303 -0.875
1.3211 0.000159 0.000064 0.011 0.00255 0.0195 0.0019 0.0078 0.0218 0.0127
3L:16723840-16723990:plus -20.6633 13.9083 0.486239 0.93578 -17.3776 -17.9796 -14.9174 3.08333 -4.0367
0.00173 9.27e-06 0.00986 0.0209 0.00419 0.00764 0.00882 0.00578 0.0562
3L:16759040-16759190:minus -39.9898 -2.72477 -1.99083 -1.05505 -28.2347 -9.65306 -20.1743 3.72222
3.46789 0.0351 0.0239 0.0216 0.0374 0.051 0.0034 0.0212 0.0045 0.00582
3L:16759040-16759190:plus -30.1429 4.90826 2.55046 9.3211 -26.8571 0.316327 -12.8807 1.31944
-0.0275229 0.00866 0.00169 0.00471 0.000391 0.0198 0.000243 0.00563 0.0109 0.0186
3L:16772700-16772850:minus -22.1531 4.31193 -5.38532 3.10092 -27.2347 -27.602 -16.0734 1.20833
-2.19266 0.0031 0.00213 0.0544 0.00865 0.0283 0.0416 0.011 0.0113 0.0352
3L:16772700-16772850:plus -33.0714 7.65138 1.90826 -1.20183 -8.27551 -8.09184 -23.0826 2.70833 2.94495
0.0267 0.00046 0.00598 0.0392 0.000951 0.000762 0.0356 0.00666 0.00714
3L:16773160-16773310:minus -34.6327 3.71429 0.908257 2.72477 -27 -17.5 -20.0642 2.40278 -4.44037
0.032 0.00129 0.00853 0.0102 0.0233 0.00599 0.0208 0.00746 0.063
3L:16773160-16773310:plus -21.8878 3.83673 0.733945 3.13761 1.21429 0.0510204 -15.1193 3.08333
-2.22936 0.00258 0.00115 0.00906 0.00854 0.0000864 0.000349 0.00919 0.00578 0.0356
3L:16776060-16776210:minus -24.1837 4.66972 2.77982 7.00917 19.9082 -17.2347 -9.99083 0.361111
3.3211 0.00751 0.00186 0.00432 0.00126 8.13e-07 0.00511 0.00307 0.0149 0.00616
3L:16776060-16776210:plus -33.1122 -3.19266 4.25688 2.69725 -27.2347 -18.5612 -15.6881 8.13889 5.95413
0.0271 0.0272 0.00242 0.0104 0.0283 0.0132 0.0102 0.000539 0.00192
3L:16779900-16780050:minus -29.949 -3.9633 -3.97248 3.33028 10.6531 -9.45918 -18.2844 -1.93056
7.24771 0.00854 0.0335 0.0377 0.00778 0.0000102 0.00279 0.0156 0.0293 0.00116
3L:16779900-16780050:plus -41.4082 0.220183 4.13761 5.90826 -27.7551 -9.7551 -22.3303 2.34722 -3.68807
0.0557 0.00956 0.00254 0.00271 0.0385 0.00358 0.0313 0.00761 0.0516
3L:16789620-16789770:minus -40.0714 -2.20183 4.3211 6.72477 -18.7143 -26.3367 -22.1835 5.27778
11.8073 0.0359 0.0206 0.00235 0.00154 0.0136 0.0221 0.0305 0.00232 0.0000538
3L:16789620-16789770:plus -22.1122 -2.42202 5.11009 9.41284 -17.1531 -9.53061 -12.3028 9.08333 -5.07339
0.00304 0.0219 0.0017 0.000354 0.00364 0.0031 0.00493 0.000304 0.0739
3L:16791280-16791430:minus -32.2959 2.88991 -0.715596 5.89908 -18.4184 -8.79592 -17.5138 10.875
3.16514 0.0186 0.00362 0.0146 0.00272 0.0101 0.0019 0.0139 0.0000846 0.00656
3L:16791280-16791430:plus -31.6224 3.04587 -2.81651 5.47706 -19.0102 -17.5714 -20.7982 4.68056 2.05505
0.0137 0.00342 0.0274 0.0036 0.0157 0.00616 0.0237 0.00302 0.00914
3L:16793300-16793450:minus -23.4082 0.357798 4.40367 7.17431 -26.9286 -27.3367 -9.99083 -2.88889
-0.440367 0.00623 0.00911 0.00228 0.00115 0.0214 0.0347 0.00307 0.0376 0.0216
3L:16793300-16793450:plus -21.6224 0.0366972 0.834862 2.44037 -18.1122 -18.3469 -16.2018 4.44444
6.7156 0.00213 0.0102 0.00875 0.0116 0.00771 0.0124 0.0112 0.00334 0.00144
3L:16794600-16794750:minus -32.7347 0.944954 -0.422018 11.2936 -17.7857 -17.5714 -12.9358 2.38889
-0.229358 0.0225 0.00739 0.0133 0.0000139 0.00676 0.00616 0.0057 0.00749 0.0202
3L:16794600-16794750:plus -4.63265 0.66055 -0.825688 3.14679 -16.4898 -17.7857 -6.3211 -1.30556
11.3853 0.000175 0.00818 0.0151 0.00847 0.00312 0.0068 0.00143 0.0246 0.0000815
3L:16796440-16796590:minus -13.1122 -0.0275229 6.6789 6.00917 -16.8163 0.867347 -14.4679 -3.52778
-8.16514 0.000632 0.0104 0.000844 0.00255 0.00323 0.000144 0.00803 0.044 0.14
3L:16796440-16796590:plus -3.78571 6.78899 3.12844 0.293578 -27.2347 -27.4082 -18.2936 6.06944

-0.0733945 0.000105 0.000723 0.00378 0.0248 0.0283 0.0356 0.0156 0.0016 0.0188
3L:16797980-16798130:minus -21.6224 0.165138 1.42202 4.04587 -26.6327 -27.5612 -6.38532 0.180556
3.6789 0.00213 0.00974 0.00712 0.00572 0.0188 0.0393 0.00145 0.0158 0.00506
3L:16797980-16798130:plus -14.9286 3.49541 4.85321 9.44954 -26.9694 -27.5612 -16.2385 9.08333 2.85321
0.0016 0.0029 0.00189 0.000326 0.023 0.0393 0.0113 0.000304 0.00733
3L:16798640-16798790:minus -30.6327 4.12844 -0.0642202 7.27523 -7.26531 -9.5 -3.3211 1.22222 1
0.00968 0.00229 0.0118 0.00109 0.000375 0.00294 0.000618 0.0113 0.0143
3L:16798640-16798790:plus -39.102 3.61468 -1.2844 3.51376 -27.3061 -18.2041 -23.0734 1.25 3.6055
0.0326 0.00277 0.0174 0.00709 0.0315 0.0103 0.0355 0.0112 0.00532
3L:16799660-16799810:minus -21.6633 3.04587 5.26606 5.69725 -18.3776 -9.38776 -13.789 -3.59722
-0.697248 0.00223 0.00342 0.00159 0.00314 0.00926 0.00266 0.00692 0.0448 0.0239
3L:16799660-16799810:plus -14 -2.20183 -0.715596 0.201835 -27.3776 -18.6327 -16.156 3.43056 5.53211
0.00114 0.0206 0.0146 0.0255 0.032 0.0137 0.0111 0.00505 0.00245
3L:16800340-16800490:minus -24.3673 0.0275229 14.3578 3.90826 -27.3776 -9.45918 -16.1743 5.97222
9.56881 0.00791 0.0102 4.55e-06 0.00603 0.032 0.00279 0.0112 0.00168 0.000303
3L:16800340-16800490:plus -14.2245 5.47706 -5.04587 0.201835 -17.6735 -8.16327 -27.5321 -2.65278 -0.174312
0.00126 0.00133 0.0499 0.0255 0.00572 0.000783 0.0647 0.0354 0.0196
3L:16800760-16800910:minus -12.2653 1.55046 -2.51376 7.17431 -18.1531 0.387755 -2.85321 0.375
1.11009 0.000381 0.00592 0.0251 0.00115 0.00829 0.000217 0.000547 0.0149 0.0138
3L:16800760-16800910:plus -22.8469 -0.183486 -1.05505 1.12844 -28.2653 -37.1122 -3.6055 1.375
11.8073 0.00424 0.011 0.0162 0.0198 0.0537 0.133 0.000668 0.0107 0.0000538
3L:16801520-16801670:minus -23.551 -0.733945 3.97248 5.30275 -8.93878 -8.72449 -19.6972 1.27778
-2.45872 0.00648 0.0132 0.00271 0.00396 0.00216 0.00169 0.0196 0.0111 0.0383
3L:16801520-16801670:plus -21.7347 2.55046 -0.633028 4.91743 1.54082 -9.53061 -2.25688 5.04167
-0.550459 0.00235 0.0041 0.0142 0.00447 0.0000712 0.0031 0.000472 0.00258 0.0225
3L:16835460-16835610:minus -23.1837 0.889908 0.0458716 -2.30275 1.83673 -9.79592 -20.0367 3.79167
2.82569 0.00553 0.00753 0.0114 0.0564 0.0000532 0.00377 0.0207 0.00437 0.0074
3L:16835460-16835610:plus -23.5612 -0.53211 -0.669725 5.49541 10.6531 -9.65306 -16.5872 2.09722
6.76147 0.00653 0.0123 0.0144 0.00355 0.0000102 0.0034 0.012 0.00832 0.00141
3L:16838360-16838510:minus -31.7041 8.30275 2.0367 -0.458716 -8.16327 -17.8571 -15.9633 1.70833
-1.59633 0.0146 0.000324 0.0057 0.0308 0.000873 0.00719 0.0108 0.00955 0.0301
3L:16838360-16838510:plus -22.1837 3.01835 1.63303 3.93578 -18.3776 -0.27551 -18.8624 1.13889 -3.14679
0.00315 0.00346 0.00661 0.00597 0.00926 0.000502 0.0171 0.0116 0.0463
3L:16846760-16846910:minus -31.5102 3.90816 5.19266 9.33028 -9.5 -18.051 -4.02752 2.58333 0.348624
0.013 0.00102 0.00164 0.000374 0.00293 0.00886 0.000752 0.00698 0.0167
3L:16846760-16846910:plus -14.1122 5.6789 3.09174 7.51376 -18.7857 -26.8265 1.33945 -1.05556 -0.220183
0.00119 0.00122 0.00383 0.000836 0.0148 0.0285 0.000201 0.0229 0.0201
3L:16847400-16847550:minus -21 -0.798165 6.25688 2.25688 -8.60204 -17.7551 -20.0183 -6.31944
-2.42202 0.00186 0.0134 0.00103 0.0127 0.00125 0.00643 0.0207 0.0811 0.0378
3L:16847400-16847550:plus -32.5816 -3.22936 1.25688 1.09174 -17.7143 -17.8571 -4.89908 4.18056 3.49541
0.0208 0.0275 0.00755 0.02 0.00627 0.00719 0.000965 0.00373 0.0057
3L:16856260-16856410:minus -13.2551 1.51376 2.63303 0.93578 -18.4898 -18.051 -13.4037 0.527778
-0.651376 0.000751 0.006 0.00457 0.0209 0.0116 0.00886 0.00634 0.0142 0.0235
3L:16856260-16856410:plus -23.1837 1.33028 3.41284 6.21101 -7.89796 -28.6327 -7.77064 1.27778 0.972477
0.00553 0.00642 0.00338 0.00213 0.000678 0.066 0.00201 0.0111 0.0144
3L:16858400-16858550:minus -41.8878 7.10092 3.25688 -2.11009 -27.5306 -17.2755 -16.4771 6.77778
3.24771 0.0672 0.000615 0.0036 0.053 0.0351 0.00544 0.0118 0.00113 0.00636
3L:16858400-16858550:plus 5.03061 -2.93578 3.2844 2.45872 -18.6429 1.82653 -5.22936 1.34722 6.95413
0.0000166 0.0253 0.00356 0.0116 0.0121 0.0000498 0.00106 0.0108 0.00136
3L:16879620-16879770:minus -32.6735 -1.61468 0.422018 11.0183 -8.37755 -8.93878 -23.7523 7.13889
4.0367 0.0216 0.0173 0.0101 0.000052 0.00106 0.00201 0.0396 0.000935 0.00424
3L:16879620-16879770:plus -13.2959 -1.33945 11.1743 10.7615 -8.33673 -26.2653 -7.45872 -0.986111
11.3853 0.000775 0.0159 0.0000714 0.0000826 0.00101 0.0218 0.00187 0.0225 0.0000815
3L:16884100-16884250:minus -3.52041 -0.394495 2.98165 2.24771 -18.7551 -27.3061 -12.2477 2.79167

11.8532 0.000076 0.0118 0.004 0.0127 0.0144 0.0336 0.00487 0.00646 0.0000428
3L:16884100-16884250:plus -22.1939 -0.954128 4.37615 9.08257 -18.2245 -9.57143 -3.18349 -2.83333
5.6055 0.00317 0.0141 0.0023 0.000444 0.00877 0.00326 0.000596 0.0371 0.00232
3L:1688620-1688770:minus -41.8163 -3.05505 0.394495 1.69725 -27.5714 -8.5 -22.8624 9.27778 4.14679
0.0662 0.0262 0.0102 0.0163 0.0357 0.00126 0.0343 0.000268 0.00406
3L:1688620-1688770:plus -3.53061 1.74312 3.38532 9.92661 -17.4184 -16.7143 -9 -3.44444 -3.87156
0.0000763 0.00552 0.00342 0.000169 0.00457 0.00419 0.00256 0.0431 0.0537
3L:16887520-16887670:minus -32 -0.614679 3.82569 -1.05505 -18.3776 -9.5 -30.4404 5.93056
-0.0183486 0.0171 0.0127 0.00288 0.0374 0.00926 0.00294 0.0967 0.00171 0.0185
3L:16887520-16887670:plus -33.0816 1.61468 1.79817 7.27523 -8.93878 -8.27551 -23.9541 -3.01389 9.04587
0.0268 0.00578 0.00623 0.00109 0.00216 0.000919 0.0408 0.0388 0.000423
3L:16948520-16948670:minus -33.3367 0.541284 3.52294 2.7156 -27.5306 -0.204082 -18.0459 1.77778
-6.51376 0.0281 0.00854 0.00324 0.0102 0.0351 0.000463 0.0151 0.00932 0.101
3L:16948520-16948670:plus -14.7857 -0.844037 -0.376147 4.3211 -8.63265 -18.3469 2.95413 3.5
-0.926606 0.00158 0.0136 0.0131 0.00514 0.0013 0.0124 0.00012 0.00491 0.0256
3L:16950420-16950570:minus -22.0306 3.90816 -1.0367 -0.678899 -8.7449 -27.5612 -10.1468 5.88889
7.45872 0.00293 0.00102 0.0161 0.0332 0.00161 0.0393 0.00316 0.00175 0.00103
3L:16950420-16950570:plus -29.7755 2.09174 0.816514 11.2936 -26.1531 0.826531 -7.99083 1.76389 1.94495
0.0085 0.00485 0.0088 0.0000139 0.0172 0.000153 0.0021 0.00937 0.00968
3L:16951180-16951330:minus -23.6939 7.10092 2.81651 2.99083 -9.0102 -28.5612 -9.07339 2.66667
7.42202 0.00667 0.000615 0.00426 0.00899 0.00236 0.0636 0.00259 0.00677 0.00106
3L:16951180-16951330:plus -32.8571 4.94495 0.770642 1.70642 -18.4592 -19.0816 -8.12844 -0.402778
1.06422 0.0244 0.00166 0.00895 0.0162 0.0109 0.017 0.00216 0.0189 0.014
3L:16962920-16963070:minus -31.6327 0.853211 1.43119 1.61468 -18.7551 -9.82653 -24.7982 -1.88889
1.12844 0.0138 0.00763 0.0071 0.0169 0.0144 0.00381 0.0459 0.0289 0.0137
3L:16962920-16963070:plus -30.4388 2.65138 -2.6422 10.0459 -8.89796 0.316327 -15.0275 -2.61111 -1.59633
0.00923 0.00395 0.0261 0.000139 0.00189 0.000243 0.00902 0.035 0.0301
3L:16979700-16979850:minus -13.6327 -2.14679 6.50459 7.37615 -17.3776 -8.65306 -18.7798 3.27778
7.15596 0.00101 0.0203 0.000917 0.000951 0.00419 0.00156 0.0169 0.00536 0.00124
3L:16979700-16979850:plus -24.8163 3.88073 6.18349 7.00917 -9.23469 -27.6735 -18.3211 4.75 -0.119266
0.0083 0.00252 0.00106 0.00126 0.00277 0.0443 0.0157 0.00293 0.0192
3L:16993420-16993570:minus -22.7347 6.46789 5.98165 3.19266 -18.4898 -9.72449 -11.6239 9.72222
1.94495 0.00403 0.000847 0.00116 0.00829 0.0116 0.00352 0.00425 0.000199 0.00968
3L:16993420-16993570:plus -40.4796 1.92661 1.77982 2.33945 -18.9796 -17.4184 -14.7064 5.59722 2.33028
0.0407 0.00516 0.00627 0.0122 0.0155 0.00585 0.00845 0.002 0.00836
3L:16995660-16995810:minus -14.5204 1.49541 7.14679 4.07339 -18.4898 -36.2245 -0.0917431 -2.26389
1.37615 0.00145 0.00604 0.000677 0.00562 0.0116 0.0923 0.000289 0.032 0.0123
3L:16995660-16995810:plus -31.1429 -4.08257 4.47706 3.61468 -8.93878 -8.79592 -17.9083 -1.55556 2.20183
0.0108 0.0346 0.00221 0.0068 0.00216 0.0019 0.0147 0.0264 0.00869
3L:16996780-16996930:minus -31.4694 -2.69725 4.02752 -1.34862 -19.0918 -27.9694 -14.8257 5.47222 -2
0.0124 0.0237 0.00265 0.0413 0.0163 0.0547 0.00866 0.00212 0.0332
3L:16996780-16996930:plus -24.1429 -1.34862 -1.55046 -1.81651 -18.5204 -27.9694 -7.78899 10.4167 7.42202
0.0074 0.016 0.0189 0.0481 0.0117 0.0547 0.00201 0.00012 0.00106
3L:17001400-17001550:minus -14.3367 -0.770642 1.59633 3 -27.9694 -27.602 -13.3211 5.20833
5.18349 0.00131 0.0133 0.00669 0.00896 0.0437 0.0416 0.00622 0.00239 0.00295
3L:17001400-17001550:plus -29.1429 -0.587156 7.2844 2.73394 -26.9286 -9.45918 -21.4862 -2.125
-2.77982 0.00834 0.0126 0.000632 0.0101 0.0214 0.00279 0.0269 0.0308 0.042
3L:17003300-17003450:minus -21.3265 -0.247706 3.05505 4.3945 -26.2653 -18.0102 -5.14679 6.55556
5.18349 0.00193 0.0112 0.00389 0.00499 0.018 0.00812 0.00104 0.00126 0.00295
3L:17003300-17003450:plus -12.9694 6.87156 2.98165 4.36697 -16.4898 -18.898 -9.98165 -0.0138889
7.6422 0.000552 0.000694 0.004 0.00505 0.00312 0.0152 0.00307 0.0168 0.000893
3L:17004180-17004330:minus -22.2857 6.3211 -0.972477 5.88991 -18.1531 -19.0816 -11.1651 1.01389
1.69725 0.00331 0.000909 0.0158 0.00273 0.00829 0.017 0.00386 0.0121 0.0108
3L:17004180-17004330:plus -32.551 1.48624 4.53211 5.93578 -27.2653 -7.72449 -20.2936 0.194444 1.83486

0.0207	0.00606	0.00216	0.00264	0.0302	0.00068	0.0217	0.0157	0.0101
3L:17005180-17005330:minus	-22.7347	6.87156	3.54128	6.47706	-26.1939	-18.7143	-8.0367	6.38889
4.80734	0.00403	0.000694	0.00322	0.00179	0.0175	0.014	0.00212	0.00137	0.00334
3L:17005180-17005330:plus	-21.551	7.26606	-0.247706	7.80734	-26.1939	-9.30612	-21.3761	6.94444
4.24771	0.00205	0.000565	0.0126	0.00063	0.0175	0.00257	0.0263	0.00103	0.00393
3L:17005680-17005830:minus	-30.1837	-3.18349	5.23853	4.07339	-17.2653	-8.42857	-10.3303	5.09722
1.25688	0.00873	0.0271	0.00161	0.00562	0.00387	0.00104	0.00328	0.00251	0.0131
3L:17005680-17005830:plus	-40.4082	-4.30275	3.22018	5.93578	-18.449	-17.8265	-7.40367	3.54167	-0.504587
0.0399	0.0367	0.00365	0.00264	0.0107	0.00699	0.00185	0.00483	0.0221
3L:17009120-17009270:minus	-20.7041	1.99083	-2.12844	4.37615	11.6939	0.316327	-16.1284	3.52778
1.79817	0.00175	0.00504	0.0224	0.00504	1.17e-06	0.000243	0.0111	0.00486	0.0102
3L:17009120-17009270:plus	-22.4388	-0.0458716	1.74312	4.75229	-8.16327	-27.7041	-11.5321	-1.81944
5.23853	0.00348	0.0105	0.00635	0.00461	0.000873	0.0445	0.00417	0.0284	0.00285
3L:17009460-17009610:minus	-32.7347	2.02752	-1.19266	4.22018	-8.16327	-18.5714	-17.6697	5.11111
-1.68807	0.0225	0.00497	0.0169	0.00531	0.000873	0.0136	0.0142	0.0025	0.0308
3L:17009460-17009610:plus	-30.8367	4.74312	6.78899	3.14679	0.836735	-16.9796	-7.17431	2.79167	3.6789
0.0103	0.00181	0.000801	0.00847	0.000215	0.0044	0.00176	0.00646	0.00506
3L:17017320-17017470:minus	-31.7755	2.16514	1.22936	3.56881	-17.4898	0.826531	-23.2752	0.208333
-2.93578	0.0157	0.00473	0.00763	0.00699	0.0053	0.000153	0.0367	0.0157	0.0439
3L:17017320-17017470:plus	-12.2959	13.4592	0.587156	7.16514	-18.8265	-17.7143	-8.98165	2.75	9.34862
0.000384	0.000064	0.00952	0.00116	0.0149	0.00634	0.00255	0.00656	0.000371
3L:17025180-17025330:minus	-30.1429	1.92661	0.706422	4.19266	-17.9286	-27.5306	-11.6422	11.7222
4.52294	0.00866	0.00516	0.00914	0.00536	0.0071	0.0374	0.00427	0.0000403	0.00358
3L:17025180-17025330:plus	-29.949	5.17431	2.14679	5.70642	-17.7857	0.27551	-12.2202	-0.277778
6.02752	0.00854	0.00151	0.00547	0.0031	0.00676	0.000257	0.00484	0.0182	0.00183
3L:17028580-17028730:minus	-24.4796	5.83486	-1	2.93578	-18.7143	-9.53061	-18.0183	-0.75	-1.05505
0.00804	0.00114	0.016	0.00927	0.0136	0.0031	0.015	0.021	0.0264
3L:17028580-17028730:plus	-33	14.8716	14.1651	7.29358	-26.6327	-18.051	-19.8165	1.73611	-5.6422
0.0258	2.8e-06	6.03e-06	0.00104	0.0188	0.00886	0.02	0.00946	0.0824
3L:17035660-17035810:minus	-24.0408	-1.44954	0.0642202	2.49541	10.6531	-18.3469	-24.4312	2.81944
0.614679	0.00726	0.0165	0.0113	0.0114	0.0000102	0.0124	0.0437	0.00639	0.0158
3L:17035660-17035810:plus	-22.7755	-3.08257	3.07339	9.61468	-7.26531	-18.5	-13.1468	2.22222	-0.431193
0.00412	0.0264	0.00386	0.000255	0.000375	0.0131	0.00598	0.00796	0.0214
3L:17044200-17044350:minus	-32.1122	2.38532	-2.00917	1.6789	-18.7857	-18.3469	-20.945	6.34722
1.69725	0.0178	0.00436	0.0217	0.0165	0.0148	0.0124	0.0243	0.0014	0.0108
3L:17044200-17044350:plus	-23.1837	-2.83486	6.43119	5.49541	0.581633	-18.2041	-13	4.30556	-4.01835
0.00553	0.0246	0.000949	0.00355	0.000292	0.0103	0.00578	0.00354	0.056
3L:17045900-17046050:minus	-22.8878	2.52294	6.22936	3.31193	-25.9286	-26.4898	-14.3486	1.55556
7.06422	0.00441	0.00414	0.00104	0.00786	0.0168	0.0225	0.00783	0.0101	0.00131
3L:17045900-17046050:plus	-32.8163	10.2752	3.90826	1.40367	-18.1224	-28.6327	-9.45872	0.916667	3.49541
0.024	0.000117	0.00278	0.0184	0.00782	0.066	0.00278	0.0125	0.0057
3L:17235480-17235630:minus	-32	1.48624	6.10092	4.10092	-17.7143	10.0918	-21.4495	3.36111	10.3486
0.0171	0.00606	0.0011	0.00554	0.00627	9.34e-06	0.0267	0.00519	0.0000988
3L:17235480-17235630:plus	-30.7347	4.08257	1.0367	5.30275	-7.60204	-8.5	-3.86239	7.08333	-4.81651	0.01
0.00233	0.00816	0.00396	0.000466	0.00126	0.000718	0.000963	0.0697
3L:1728380-1728530:minus	-31.551	1.15596	0.311927	4.24771	-27.2653	-8.45918	-14.945	11.2222	7.04587
0.0134	0.00684	0.0104	0.00519	0.0302	0.00111	0.00887	0.0000631	0.00132
3L:1728380-1728530:plus	-13.4082	-4.76147	3.87156	4.08257	-18.7551	-17.8265	-3.47706	3.20833	0.825688
0.000867	0.0414	0.00282	0.00558	0.0144	0.00699	0.000645	0.00551	0.0149
3L:1729920-1730070:minus	-23.5204	5.47706	1.04587	9.44954	-26.2347	-17.2755	-17.1193	1.65278	-4.54128
0.00645	0.00133	0.00813	0.000326	0.0177	0.00544	0.0131	0.00974	0.0645
3L:1729920-1730070:plus	-31.5204	6.55046	8.92661	6.84404	-27.2347	-9.20408	-26.3853	-3.09722	-4.04587
0.0131	0.000814	0.000264	0.0014	0.0283	0.00225	0.0561	0.0396	0.0563
3L:17332380-17332530:minus	-23.8878	1.6789	4.21101	3.01835	-17.6429	-9.5	-19.2477	-4.13889	-6.6422

0.00692	0.00565	0.00246	0.0089	0.00562	0.00294	0.0182	0.0508	0.105
3L:17332380-17332530:plus	-31.8061	3.99083	-0.0183486	3.76147	-7.67347	-8.5	-16.8807	-6.18056	-0.119266
0.0158	0.00241	0.0117	0.00645	0.00055	0.00126	0.0126	0.0789	0.0192
3L:17346020-17346170:minus	-34.2245	13.3878	-5.21101	0.522936	-27.5306	-0.397959	-29.367	8.5	-2
0.0315	0.0000869	0.0521	0.0234	0.0351	0.000525	0.0831	0.000436	0.0332
3L:17346020-17346170:plus	-12.8878	5.73394	13.4128	7.2844	-27.8265	-28.3367	-14.8899	6.875	-2.69725
0.000523	0.00119	0.0000141	0.00105	0.0388	0.0606	0.00877	0.00107	0.0411
3L:1738400-1738550:minus	-31.6327	-1.9633	7.63303	3.34862	-17.4184	-18.2755	-12.5688	2.375	-3.82569
0.0138	0.0192	0.000531	0.0077	0.00457	0.0112	0.00524	0.00753	0.0531
3L:1738400-1738550:plus	-22.6327	-0.0825688	7.3578	7.21101	-8.37755	19.3776	-22.4954	3.22222
5.70642	0.00373	0.0106	0.000608	0.0011	0.00106	3.24e-07	0.0322	0.00548	0.00222
3L:17414580-17414730:minus	-13.1122	3.3211	5.09174	7.3578	-17.6429	-26.2653	-3.52294	-8.47222
-1.6789	0.000632	0.00309	0.00171	0.000965	0.00562	0.0218	0.000653	0.12	0.0306
3L:17414580-17414730:plus	-33.9592	-0.825688	7.3578	2	-16.7143	-9.72449	-21.4312	9.52778	-1.09174
0.0304	0.0136	0.000608	0.0143	0.00319	0.00352	0.0266	0.000227	0.0268
3L:17418340-17418490:minus	-32.7449	3.83673	15.1193	7.29358	-27.3367	-27.4082	-9.80734	0.708333
-0.706422	0.0228	0.00115	1.13e-06	0.00104	0.0317	0.0356	0.00297	0.0134	0.024
3L:17418340-17418490:plus	-24.1939	-1.11927	5.07339	3.43119	20.4694	-8.5	-6.11927	5.44444	-0.275229
0.00753	0.0149	0.00172	0.00749	1.65e-07	0.00126	0.00135	0.00215	0.0206
3L:17420260-17420410:minus	-31.8061	1.68807	15.4495	2.07339	-8.71429	-18.9796	-5.51376	3.47222
-2.49541	0.0158	0.00563	9.17e-07	0.0138	0.00159	0.0155	0.00115	0.00497	0.0387
3L:17420260-17420410:plus	-41.2143	3.07339	-1.57798	3.78899	-17.9796	0.0204082	-6.95413	4.45833
6.6422	0.0494	0.00339	0.019	0.00632	0.00726	0.000377	0.00167	0.00332	0.00148
3L:17421660-17421810:minus	-23.3265	-0.238532	0.431193	-0.669725	-18.2653	-37.6633	-10.2202
-3.70833	4.10092	0.00607	0.0112	0.01	0.0331	0.00882	0.171	0.00321	0.046	0.00413
3L:17421660-17421810:plus	-31.7041	-1.3211	3.37615	2	-8.67347	-18.9796	-21.3945	0.847222	-3.77982
0.0146	0.0159	0.00343	0.0143	0.00154	0.0155	0.0264	0.0128	0.0525
3L:17422000-17422150:minus	-12.2959	-1.23853	0.926606	8.05505	-18.4898	0.897959	-17.211	-2.91667
-1.48624	0.000384	0.0154	0.00848	0.000578	0.0116	0.000144	0.0132	0.0378	0.0293
3L:17422000-17422150:plus	-21.4796	-4.42202	2.44954	4.43119	-18.4184	-19.1531	-2.44954	3.84722	7.06422
0.00202	0.0379	0.00489	0.00495	0.0101	0.0177	0.000494	0.00428	0.00131
3L:17424320-17424470:minus	-24.5612	3.81651	1.66972	2.15596	-9.16327	-18.5714	-24.5596	3.75	-0.688073
0.00819	0.00258	0.00652	0.0133	0.00249	0.0136	0.0445	0.00445	0.0238
3L:17424320-17424470:plus	-23.2245	3.57143	-2.62385	6.22936	-8.89796	-17.5	-22.0734	4.15278	-3.47706
0.00576	0.00175	0.0259	0.00209	0.00189	0.00599	0.0299	0.00377	0.0493
3L:17424900-17425050:minus	-24.1531	-1.0367	-0.93578	7.27523	-18.1837	-8.5	-14.211	-2.66667	0.743119
0.00744	0.0145	0.0156	0.00109	0.00835	0.00126	0.0076	0.0355	0.0153
3L:17424900-17425050:plus	-21.1429	2.65138	0.541284	4.22018	-17.3469	-26.8265	-10.6422	-1.08333	2.85321
0.00188	0.00395	0.00968	0.00531	0.00394	0.0285	0.00348	0.0231	0.00733
3L:17426280-17426430:minus	-30.2959	-0.706422	6.41284	3.58716	10.9184	-0.244898	-16.1284
2.90278	8.95413	0.00887	0.0131	0.000959	0.00694	4.19e-06	0.000496	0.0111	0.00619	0.000458
3L:17426280-17426430:plus	-33.2245	-1.15596	4.37615	2.09174	-18.4184	-9.72449	-22.4587	-1.31944	-0.220183
0.0276	0.0151	0.0023	0.0137	0.0101	0.00352	0.032	0.0247	0.0201
3L:17426680-17426830:minus	-20.7041	1.43119	14.8899	0.119266	-9.20408	-0.173469	-17.5596	2.375
-1.44037	0.00175	0.00619	2.06e-06	0.026	0.00262	0.000456	0.014	0.00753	0.029
3L:17426680-17426830:plus	-32.3673	1.44954	0.614679	-0.412844	0.94898	-8.42857	-22.0734	10.625
1.95413	0.0189	0.00615	0.00943	0.0304	0.000134	0.00104	0.0299	0.000103	0.00964
3L:17458860-17459010:minus	-32.8878	4.20183	3.7156	5.31193	-17.5204	-9.02041	-12.7156	3.58333
-2.11009	0.0247	0.00223	0.00301	0.00393	0.00532	0.00213	0.00542	0.00475	0.0343
3L:17458860-17459010:plus	-31.1122	-1.68807	2.00917	1.86239	19.9082	-17.8571	-21.9817	5.29167	2.88073
0.0107	0.0177	0.00576	0.0153	8.13e-07	0.00719	0.0294	0.0023	0.00727
3L:174620-174770:minus	-13.4796	14.2018	-1.21101	1.99083	-19.051	-9.20408	-13.6881	11.5833	-4.19266
0.000935	6.73e-06	0.017	0.0144	0.0162	0.00225	0.00676	0.0000457	0.0583
3L:174620-174770:plus	-14.2959	8.69725	5.47706	9.7156	-17.7551	-8.68367	-10.7798	2.08333	-2.92661

0.00129 0.000263 0.00145 0.000242 0.00658 0.00157 0.00357 0.00837 0.0437
3L:17468000-17468150:minus -31.7755 -0.0366972 2.18349 -0.678899 -27.3061 -8.45918 -16.1376
1.08333 4.25688 0.0157 0.0104 0.0054 0.0332 0.0315 0.00111 0.0111 0.0118 0.00391
3L:17468000-17468150:plus -30.5102 0.0733945 1.6789 1.78899 20.4694 -7.5 -7.27523 1.34722 7.72477
0.00947 0.0101 0.0065 0.0158 1.65e-07 0.000614 0.0018 0.0108 0.000798
3L:17469140-17469290:minus -32.4388 -2.07339 14.3119 4.17431 -18.4184 -9.79592 -10.7523 0.694444
4.14679 0.0195 0.0198 4.82e-06 0.00537 0.0101 0.00377 0.00355 0.0134 0.00406
3L:17469140-17469290:plus -23.2551 11.4312 15.0183 5.66055 -17.449 -27.7449 -8.70642 2.59722
2.36697 0.00583 0.0000639 1.42e-06 0.00317 0.00484 0.0448 0.00242 0.00694 0.00828
3L:17480420-17480570:minus -29.102 8.49541 6.08257 1.85321 -8.26531 0.0510204 -24.9633 8
-2.90826 0.00833 0.000292 0.00111 0.0153 0.000934 0.000349 0.0469 0.000583 0.0435
3L:17480420-17480570:plus -22.551 1.07339 -0.0458716 7.02752 -17.6837 -9.42857 -11.7706
11.4583 -2.26606 0.00359 0.00705 0.0118 0.00122 0.0058 0.0027 0.00439 0.0000512 0.0359
3L:17484480-17484630:minus -32.4796 2.34862 2.86239 4.31193 -18.5204 -9.53061 -18.5688
2.93056 2.53211 0.02 0.00441 0.00418 0.00516 0.0117 0.0031 0.0163 0.00612 0.00791
3L:17484480-17484630:plus -32.3673 -0.275229 0.302752 3.07339 -18.5204 -37.5204
-6.59633 5.16667 7.6422 0.0189 0.0113 0.0105 0.00873 0.0117 0.166 0.00153 0.00244 0.000893
3L:17507040-17507190:minus -23.7449 -1.0367 0.541284 0.743119 -8.93878 0.0204082
-19.7615 -1.02778 0.513761 0.00674 0.0145 0.00968 0.0221 0.00216 0.000377 0.0198 0.0227
0.0162
3L:17507040-17507190:plus -32.1327 4.51376 -0.0825688 4.43119 -17.8878 -18.2347 -20.2844
5.72222 -4.25688 0.0178 0.00198 0.0119 0.00495 0.00704 0.0104 0.0216 0.00189 0.0593
3L:17522700-17522850:minus -30.449 -1.65138 4.16514 2.76147 -18.7959 -26.602 -17.0459 2.47222
10.3486 0.00925 0.0175 0.00251 0.01 0.0148 0.0248 0.0129 0.00727 0.0000988
3L:17522700-17522850:plus -31.6327 4.2844 1.73394 4.37615 -17.7143 9.86735 -20.945 7.30556
-6.48624 0.0138 0.00216 0.00638 0.00504 0.00627 0.0000132 0.0243 0.000857 0.1
3L:17523080-17523230:minus -23.1939 -1.50459 2.19266 4.37615 -7.40816 1.05102 -11.0183
-3.55556 3.62385 0.00558 0.0168 0.00538 0.00504 0.000401 0.000107 0.00375 0.0443 0.00523
3L:17523080-17523230:plus -30.3673 6.68807 1.17431 5.93578 10.9898 -7.72449 -27.0917 -1.29167
1.01835 0.00897 0.00076 0.00778 0.00264 3.28e-06 0.00068 0.0612 0.0245 0.0142
3L:17526140-17526290:minus -40.2143 2.95413 10.156 2.0367 -8.56122 -9.94898 -25.8165
-4.94444 -4.04587 0.0372 0.00354 0.00013 0.0141 0.00112 0.00393 0.0523 0.0609 0.0563
3L:17526140-17526290:plus -39.2143 1.04587 -2.17431 6.00917 20.1735 0.755102 -12.3211
2.97222 -0.59633 0.0329 0.00712 0.0227 0.00255 5.68e-07 0.000175 0.00495 0.00603 0.0229
3L:17526560-17526710:minus -3.03061 1.6789 -2.27523 1.41284 -18.4184 -18.051 6.86239 -2.40278
2.93578 0.0000417 0.00565 0.0234 0.0183 0.0101 0.00886 0.0000322 0.0332 0.00717
3L:17526560-17526710:plus -22.2959 -2.89908 -0.247706 5.57798 1.87755 -8.38776 -10.9908
-5.33333 5.65138 0.00335 0.0251 0.0126 0.0034 0.0000492 0.00101 0.00373 0.0663 0.00228
3L:17550820-17550970:minus -24.5612 -3.51376 -0.899083 3.18349 -7.67347 -17.9796
-11.4679 -2.41667 -2 0.00819 0.0297 0.0155 0.00837 0.00055 0.00764 0.00411 0.0333 0.0332
3L:17550820-17550970:plus -33.3673 13.4592 5.01835 1.41284 -8.97959 -0.244898 -14.2752
2.31944 -4.33945 0.0282 0.000064 0.00176 0.0183 0.0023 0.000496 0.00771 0.00769 0.0609
3L:17572780-17572930:minus -12.0408 -1.17431 2.43119 9.23853 -8.60204 -17.4898 -17.1468
-5.77778 -2.6789 0.000311 0.0151 0.00493 0.000424 0.00125 0.0059 0.0131 0.0727 0.0408
3L:17572780-17572930:plus -23.9184 1.66972 0.568807 4.22018 -19.051 -27.6327 -4.90826
-5.30556 2.75229 0.00695 0.00567 0.00958 0.00531 0.0162 0.043 0.000967 0.0659 0.00748
3L:176120-176270:minus -31.3673 3.93578 -0.807339 1.55046 2.10204 -8.79592 -22.7248 5.34722
5.33028 0.0116 0.00246 0.015 0.0173 0.0000336 0.0019 0.0335 0.00225 0.00273
3L:176120-176270:plus -32.0714 3.61224 7.07339 3.88073 -18.7551 -17.5 -19.4495 5.34722 -0.908257
0.0176 0.00167 0.000703 0.0061 0.0144 0.00599 0.0188 0.00225 0.0255
3L:17612420-17612570:minus -31.4796 -2.53211 5.33945 3.09174 -26.7041 -9.23469 -18.9358
2.97222 1.16514 0.0128 0.0226 0.00154 0.00867 0.0193 0.00238 0.0173 0.00603 0.0135
3L:17612420-17612570:plus -22.5102 -2.07339 -0.174312 0.504587 -16.3776 -36.1122
-11.1376 -4.52778 4.13761 0.00354 0.0198 0.0123 0.0235 0.003 0.0878 0.00384 0.0556 0.00408

3L:17614660-17614810:minus -39.9898 -3.55963 15.6239 1.49541 -18.4898 -18.1224 -12.9908
2.93056 3.46789 0.0351 0.03 5.44e-07 0.0176 0.0116 0.00988 0.00577 0.00612 0.00582
3L:17614660-17614810:plus -24.4592 -0.211009 3.33028 3.52294 1.14286 9.23469 -12.3486 7.54167
1.58716 0.00802 0.0111 0.00349 0.00706 0.000113 0.0000427 0.00498 0.000754 0.0113
3L:17637520-17637670:minus -30.2143 3.68367 1.86239 5.22936 -17.3367 -17.7857 -13.8349
-0.583333 -3.49541 0.0088 0.00144 0.00608 0.0041 0.00391 0.0068 0.00699 0.02 0.0495
3L:17637520-17637670:plus -31.7755 2.80734 -0.0275229 1.43119 -28.2347 -26.898 -24.2752
6.95833 -1.17431 0.0157 0.00373 0.0117 0.0181 0.051 0.0302 0.0427 0.00103 0.0275
3L:17647040-17647190:minus -31.551 4.33945 -3.26606 4.22018 -26.9286 -37.4796 -3.66972
6.25 11.3853 0.0134 0.00211 0.0311 0.00531 0.0214 0.165 0.00068 0.00147 0.0000815
3L:17647040-17647190:plus -31.6633 1.34862 4.44037 2.52294 -8.93878 0.346939 -15.6422
-0.527778 -0.743119 0.0141 0.00637 0.00224 0.0113 0.00216 0.000221 0.0102 0.0196 0.0243
3L:17661000-17661150:minus -4.63265 7.06422 7.33028 4.23853 -17.8571 -8.72449 -8.11927
4.98611 5.08257 0.000175 0.000628 0.000617 0.00522 0.00698 0.00169 0.00216 0.00264 0.00303
3L:17661000-17661150:plus -33.3061 1.83486 -2.47706 1.24771 -27.7959 0.0918367 -32.0734
1.72222 -1.11009 0.0279 0.00533 0.0248 0.0192 0.0386 0.000323 0.119 0.0095 0.0269
3L:17662300-17662450:minus -13.1122 6.76147 1.73394 1.9633 -17.7857 -36.1122 -15.4312
-1.29167 3.76147 0.000632 0.000733 0.00638 0.0146 0.00676 0.0878 0.00977 0.0245 0.00494
3L:17662300-17662450:plus -22.0714 6.99083 -1.94495 2.55046 -17.7551 -18.8571 -23.6147
1.22222 4.10092 0.00299 0.000652 0.0213 0.0112 0.00658 0.0151 0.0387 0.0113 0.00413
3L:17733620-17733770:minus -5.08163 7.37615 4.22018 3.62385 -18.051 -8.7551 -15.4587 8.18056
0.816514 0.000224 0.000532 0.00245 0.00673 0.00746 0.00172 0.00982 0.000526 0.015
3L:17733620-17733770:plus -12.1122 -0.0825688 4.12844 10.8624 -27.1939 -9.0102 -10.7156
4.06944 5.0367 0.000336 0.0106 0.00254 0.0000687 0.0267 0.00208 0.00353 0.00391 0.00306
3L:1775100-1775250:minus -24.7857 13.5306 8.6789 3.77064 -18.449 -10.0204 -20.2936 3.22222
3.55963 0.00829 0.0000206 0.000302 0.00637 0.0107 0.00399 0.0217 0.00548 0.00545
3L:1775100-1775250:plus -30.6633 13.5306 -1.15596 3.75229 -18.4184 -9.86735 -13.055 9.45833
-4.04587 0.00976 0.0000206 0.0168 0.00649 0.0101 0.00391 0.00585 0.000238 0.0563
3L:1775520-1775670:minus -21.9286 13.3878 6.54128 6.12844 -18.7143 -18.1224 -14.1101
1.90278 -0.697248 0.0027 0.0000869 0.000901 0.00231 0.0136 0.00988 0.00743 0.00892 0.0239
3L:1775520-1775670:plus -31.8469 -4.94495 7.02752 1.16514 -27.2653 -18.1224 -17.9541
1.45833 1.6789 0.0162 0.0435 0.000718 0.0197 0.0302 0.00988 0.0148 0.0104 0.011
3L:17757280-17757430:minus -3.67347 0.119266 3.42202 11.0275 -25.9694 -17.0102 -13.4128
-0.847222 8.14679 0.0000972 0.0099 0.00337 0.0000401 0.017 0.00453 0.00635 0.0216 0.000562
3L:17757280-17757430:plus -30.6224 -0.238532 -2.06422 2.61468 -25.8571 -18.051 -11.1651
6.20833 4.24771 0.00964 0.0112 0.022 0.0109 0.0166 0.00886 0.00386 0.0015 0.00393
3L:1777400-1777550:minus -22.6224 9.33945 0.412844 1.69725 -17.5306 0.755102 -6.12844
1.27778 11.8073 0.00371 0.000188 0.0101 0.0163 0.0054 0.000175 0.00136 0.0111 0.0000538
3L:1777400-1777550:plus -31.1735 5.95413 14.8624 7.29358 -17.6837 -18.3061 -19.7523 2.47222 -2.37615
0.0109 0.00108 2.2e-06 0.00104 0.0058 0.0114 0.0197 0.00727 0.0373
3L:17795400-17795550:minus -31.3673 2.63303 -1.80734 4.34862 -16.8163 1.12245 -15.5229 -0.194444
-5.54128 0.0116 0.00398 0.0204 0.00507 0.00323 0.000085 0.00994 0.0178 0.081
3L:17795400-17795550:plus -31.7347 -1.29358 5.75229 2.62385 -26.2245 -9.94898 -20.1376 1.25 -2.3578
0.0149 0.0157 0.00129 0.0108 0.0175 0.00393 0.0211 0.0112 0.0369
3L:17797680-17797830:minus -32 2.27523 2.3945 -0.284404 0.908163 -10.0204 -3.76147 -0.708333
-1.83486 0.0171 0.00454 0.00499 0.0291 0.000153 0.00399 0.000698 0.0207 0.0318
3L:17797680-17797830:plus -34.5612 2.13761 -4.15596 -0.40367 -17.7143 -9.02041 -15.3211 1.13889 5.70642
0.032 0.00477 0.0396 0.0302 0.00627 0.00213 0.00956 0.0116 0.00222
3L:17834240-17834390:minus -22.1837 13.1927 2.04587 -1.77982 -18.4184 -9.27551 -23.6055 7.08333
4.14679 0.00315 0.0000187 0.00568 0.0474 0.0101 0.00244 0.0387 0.000963 0.00406
3L:17834240-17834390:plus -31.102 -2.52294 -1.78899 0.779817 -18.0408 -27.602 2.98165 -5.16667 4.13761
0.0106 0.0226 0.0203 0.0219 0.00735 0.0416 0.000119 0.0639 0.00408
3L:17836580-17836730:minus -31.2143 6.29358 13.1468 5.22018 -18.7551 -18.6429 -22.8899 2.41667
0.522936 0.0111 0.000922 0.0000179 0.00418 0.0144 0.0139 0.0344 0.00742 0.0162

3L:17836580-17836730:plus -32.6633 -3.89908 13.7431 5.15596 -17.7143 -8.79592 -13.3486 1.91667 2.63303
0.0216 0.0329 0.0000103 0.00426 0.00627 0.0019 0.00626 0.00888 0.00771
3L:17839560-17839710:minus -22.6633 3.90826 2.86239 7.51376 20.4694 -8.53061 -19.5872 -1.90278
1.27523 0.00384 0.00249 0.00418 0.000836 1.65e-07 0.00136 0.0192 0.029 0.013
3L:17839560-17839710:plus -22 5.49541 2.83486 -1.31193 -18.4184 -36.3776 -6.40367 -0.847222 2.93578
0.00291 0.00132 0.00423 0.0405 0.0101 0.104 0.00146 0.0216 0.00717
3L:17840060-17840210:minus -40.551 -0.844037 -0.963303 -0.633028 -26.9286 -8.94898 -17.2385
0.319444 -2.90826 0.0418 0.0136 0.0158 0.0327 0.0214 0.00203 0.0133 0.0151 0.0435
3L:17840060-17840210:plus -14.102 8.17431 0.183486 3.10092 -18.0102 -18.2041 -8.7156 -3.125 -2.52294
0.00117 0.000347 0.0109 0.00865 0.00732 0.0103 0.00242 0.0399 0.0391
3L:17849840-17849990:minus -22.7041 2.56881 3.9633 1.10092 -26.7041 -17.8571 -13.3761 -0.513889
7.58716 0.00395 0.00407 0.00272 0.02 0.0193 0.00719 0.0063 0.0196 0.000954
3L:17849840-17849990:plus -32.5102 -2.11009 5.9633 6.74312 -8.67347 -27.7143 -8.6789 -1.25 3.81651
0.0202 0.0201 0.00117 0.0015 0.00154 0.0447 0.00241 0.0242 0.00482
3L:17850420-17850570:minus -23.8469 7.51376 -0.238532 2.19266 -8.97959 -9.72449 -14.7064 7.44444
1.04587 0.00685 0.000495 0.0125 0.0131 0.0023 0.00352 0.00845 0.000795 0.014
3L:17850420-17850570:plus -41.8469 -4.07339 -1.66055 6.57798 -18.4184 -9.42857 -23.0183 2.34722 2.42202
0.0665 0.0345 0.0195 0.00167 0.0101 0.0027 0.0352 0.00761 0.00816
3L:17872080-17872230:minus -24.3776 0.779817 3.44037 0.633028 -18.7551 -18.0816 -22.9083 -2.75
11.8532 0.00793 0.00784 0.00335 0.0229 0.0144 0.00934 0.0345 0.0363 0.0000428
3L:17872080-17872230:plus -21.3673 -1.6055 -0.211009 4.08257 -27.1224 -8.82653 4.84404 -2.38889
-1.41284 0.00195 0.0173 0.0124 0.00558 0.0245 0.00191 0.0000609 0.0331 0.0289
3L:17900820-17900970:minus -22.0408 3.04587 5.54128 2.80734 -27.6429 -17.5714 -10.8899 6.80556
4.7156 0.00296 0.00342 0.00141 0.00985 0.037 0.00616 0.00365 0.00111 0.00345
3L:17900820-17900970:plus -22 -2.33028 4.11009 3.13761 -8.93878 -27.7041 -9.42202 1.84722 3.6789
0.00291 0.0214 0.00256 0.00854 0.00216 0.0445 0.00277 0.0091 0.00506
3L:17905660-17905930:minus -31.4388 0.486239 14.2294 7.29358 -26.7041 -18.8571 -16.367 5.47222
-4.76147 0.0122 0.00871 5.53e-06 0.00104 0.0193 0.0151 0.0116 0.00212 0.0686
3L:17905660-17905930:plus -24.4082 -0.587156 5.74312 7.61468 -27.9286 0.0204082 -8.66972 8.125
-1.01835 0.00797 0.0126 0.00129 0.000779 0.0412 0.000377 0.0024 0.000543 0.0263
3L:17906300-17906450:minus -23.9898 0.779817 5.46789 3.61468 -18.6837 -9.30612 -18.4495 4.45833
-0.697248 0.00711 0.00784 0.00146 0.0068 0.0126 0.00257 0.016 0.00332 0.0239
3L:17906300-17906450:plus -12.8469 5.41284 -4.91743 3.76147 -17.4184 -8.94898 -18.2936 5.27778 4.66055
0.000502 0.00137 0.0483 0.00645 0.00457 0.00203 0.0156 0.00232 0.0035
3L:17907100-17907250:minus -31.8163 0.513761 -0.917431 5.22018 -19.3163 -9.7551 -18.6697 4.29167
2.99083 0.016 0.00862 0.0156 0.00418 0.0165 0.00358 0.0166 0.00356 0.00706
3L:17907100-17907250:plus -32.4388 -1.29358 4.19266 5.30275 -18.6837 -8.65306 -24.9817 5.66667 -3.59633
0.0195 0.0157 0.00248 0.00396 0.0126 0.00156 0.047 0.00194 0.0507
3L:17909440-17909590:minus -33.6633 1.80734 5.84404 7.63303 -17.9796 -9.23469 -19.7431 -1.13889
3.62385 0.0291 0.00539 0.00124 0.000745 0.00726 0.00238 0.0197 0.0235 0.00523
3L:17909440-17909590:plus -24.4082 4.70642 0.119266 1.57798 -8.16327 -18.6429 -17.422 0.25 3.02752
0.00797 0.00183 0.0111 0.0171 0.000873 0.0139 0.0137 0.0155 0.00695
3L:17909660-17909810:minus -32.6633 -1.59633 1.66972 6.12844 -18.6837 -8.30612 -15.367 1.11111
-4.49541 0.0216 0.0172 0.00652 0.00231 0.0126 0.000973 0.00965 0.0117 0.0638
3L:17909660-17909810:plus -33.0408 1.79817 -0.394495 1.18349 -18.6837 -8.7551 -23.4862 2.80556
-0.0366972 0.0264 0.00541 0.0132 0.0196 0.0126 0.00172 0.038 0.00642 0.0187
3L:17911320-17911470:minus -14.1122 0.348624 1.3578 10.8624 -18.1224 -17.8571 -13.3578 0.305556
9.66055 0.00119 0.00914 0.00728 0.0000687 0.00782 0.00719 0.00628 0.0152 0.000268
3L:17911320-17911470:plus -20.3265 -1.6055 2 4.22018 -26.8571 -9.20408 -15.2936 -2.51389 -2.3578
0.00165 0.0173 0.00578 0.00531 0.0198 0.00225 0.00951 0.0341 0.0369
3L:17914700-17914850:minus -24.2245 -0.275229 8.55046 2.69725 -27.1224 -8.93878 -23.2018 0.875
-1.97248 0.00763 0.0113 0.000323 0.0104 0.0245 0.00201 0.0363 0.0127 0.033
3L:17914700-17914850:plus -22.3673 4 4.3945 3.87156 -17.1939 -27.4082 -8.3578 5.01389 5.65138
0.00341 0.0024 0.00229 0.00618 0.00377 0.0356 0.00226 0.00261 0.00228

3L:17915880-17916030:minus -31.1327 1.70642 2.3211 5.49541 -26.898 -17.7857 -18.4312 4.16667
-4.66055 0.0107 0.00559 0.00513 0.00355 0.0203 0.0068 0.016 0.00375 0.0667
3L:17915880-17916030:plus -39.7347 1.05505 3.99083 6.74312 -7.86735 -17.7551 -16.633 4.56944 -2.68807
0.0338 0.00709 0.00269 0.0015 0.000617 0.00643 0.0121 0.00317 0.041
3L:17918240-17918390:minus -3.92857 4.37615 9.83486 7.88991 -17.4184 -16.7143 1.98165 -3 -0.412844
0.000116 0.00208 0.000157 0.00062 0.00457 0.00419 0.000166 0.0386 0.0213
3L:17918240-17918390:plus -32.8163 -0.33945 3.76147 3.7156 -18.2245 -28.7041 -17.4037 3 1.55963 0.024
0.0116 0.00295 0.00655 0.00877 0.067 0.0136 0.00597 0.0114
3L:17920340-17920490:minus -40.1429 1.22018 5.78899 2.84404 -17.6429 -17.1224 -18.3028 5.26389
3.88991 0.0363 0.00668 0.00127 0.00961 0.00562 0.00499 0.0157 0.00233 0.00467
3L:17920340-17920490:plus -34.1531 0.770642 -1.43119 1.63303 -27.5306 -18.6429 -16.9358 8.18056 7.82569
0.0314 0.00786 0.0182 0.0167 0.0351 0.0139 0.0127 0.000526 0.000728
3L:1793300-1793450:minus -32.6633 -4.04587 1.3211 -2.52294 -17.7449 -18.7143 -16.4495 7.15278 -0.100917
0.0216 0.0342 0.00738 0.0606 0.0063 0.014 0.0117 0.000928 0.019
3L:1793300-1793450:plus -23.8571 -4.55046 6.61468 7 -27.1939 -9.45918 -10.4679 -3.40278 -4.36697
0.00687 0.0392 0.000869 0.00132 0.0267 0.00279 0.00336 0.0427 0.0615
3L:17940340-17940490:minus -41.8878 1.10092 -1.44037 3.51376 -17.6429 -7.72449 -25.1009 1.68056
2.11009 0.0672 0.00698 0.0183 0.00709 0.00562 0.00068 0.0478 0.00964 0.00898
3L:17940340-17940490:plus -31.102 0.733945 -1.91743 2.3945 1.17347 -8.5 -19.9725 -3.30556 -0.541284
0.0106 0.00797 0.0211 0.0119 0.000101 0.00126 0.0205 0.0417 0.0223
3L:17942520-17942670:minus -31.8469 2.12844 4.89908 -0.614679 -19.051 -18.4184 -20.5321 9.18056
3.47706 0.0162 0.00479 0.00185 0.0324 0.0162 0.0127 0.0226 0.000285 0.00575
3L:17942520-17942670:plus -30.2143 -5.3578 1.63303 2.23853 -27.8571 -0.397959 -23.0092 -4.80556
0.0550459 0.0088 0.0484 0.00661 0.0128 0.039 0.000525 0.0351 0.0591 0.0182
3L:1794360-1794510:minus -14.1837 -1.29358 2.3945 3.78899 -17.8163 -8.38776 -21.4771 -0.152778
-0.733945 0.00124 0.0157 0.00499 0.00632 0.00682 0.00101 0.0268 0.0175 0.0242
3L:1794360-1794510:plus -32.7755 -0.633028 -1.90826 -1.15596 -18.4898 -27.9694 -10.4128 -1.125
0.0825688 0.0234 0.0127 0.021 0.0386 0.0116 0.0547 0.00333 0.0234 0.0181
3L:17943620-17943770:minus -23.4184 7 4.59633 1.97248 -18.3469 0.0510204 -18.1376 -1.80556
3.47706 0.00624 0.000649 0.0021 0.0146 0.00894 0.000349 0.0153 0.0283 0.00575
3L:17943620-17943770:plus -31.7755 3.86239 -2.52294 -0.587156 -17.7551 -19.0102 -12.5413 2.16667
11.8991 0.0157 0.00253 0.0252 0.0322 0.00658 0.0159 0.00521 0.00812 0.0000255
3L:1794780-1794930:minus -14.7857 -4.98165 0.990826 -3.77982 -28.2347 -8.72449 -10.8257 3.625 3
0.00158 0.0439 0.00829 0.0804 0.051 0.00169 0.00361 0.00468 0.00702
3L:1794780-1794930:plus -32.1327 -1.61468 0.816514 3.88991 -17.1837 -9.53061 -14.3853 -0.694444
3.66972 0.0178 0.0173 0.0088 0.00608 0.00366 0.0031 0.00789 0.0206 0.00514
3L:1795240-1795390:minus -24.2959 4.42202 2.44037 4.48624 -18.1531 -18.0816 -4.38532 1.56944 3.52294
0.00781 0.00204 0.00491 0.00487 0.00829 0.00934 0.000832 0.01 0.00558
3L:1795240-1795390:plus -22.8061 1.47706 -1.26606 2.90826 20.1735 0.0510204 -5.22018 3.90278
5.3211 0.00414 0.00608 0.0173 0.00936 5.68e-07 0.000349 0.00106 0.00418 0.00275
3L:17954860-17955010:minus -13.7755 2.12844 -0.944954 2.68807 -18.9796 -7.57143 -20.9633 -1.5
-2.17431 0.00107 0.00479 0.0157 0.0104 0.0155 0.000644 0.0244 0.026 0.035
3L:17954860-17955010:plus -23.0714 3.7156 2.48624 7.6422 -28.2653 -36.3061 -3.17431 5.72222 10.3486
0.0052 0.00267 0.00483 0.000721 0.0537 0.0959 0.000595 0.00189 0.0000988
3L:1795540-1795690:minus -24.2959 13.4592 2.44037 4.48624 -18.4898 -18.0816 -4.38532 5.81944 1.69725
0.00781 0.000064 0.00491 0.00487 0.0116 0.00934 0.000832 0.00181 0.0108
3L:1795540-1795690:plus -33 1.47706 0.12844 4.33945 20.1735 0.0510204 -5.22018 3.90278 3.46789
0.0258 0.00608 0.0111 0.00509 5.68e-07 0.000349 0.00106 0.00418 0.00582
3L:17964300-17964450:minus -3.37755 -0.963303 4.66055 7.27523 -26.898 -8.72449 -7.48624 -2.38889
5.66055 0.0000645 0.0142 0.00205 0.00109 0.0203 0.00169 0.00189 0.0331 0.00227
3L:17964300-17964450:plus -13.949 -2.3578 2.33945 4.02752 -16.0816 -9.20408 -4.3211 -1.34722 1.73394
0.00112 0.0215 0.0051 0.00581 0.00294 0.00225 0.000817 0.0249 0.0106
3L:1798700-1798850:minus -31.8061 3.68367 3.78899 5.86239 -18.8265 -10.0204 -15.211 1.5 -4.36697
0.0158 0.00144 0.00292 0.00282 0.0149 0.00399 0.00936 0.0103 0.0615

3L:1798700-1798850:plus	-21.6939	10.2477	15.7339	7.29358	-8.37755	-19.051	-14.0826	-3.90278	3.88991
0.00226	0.000118	2.45e-07	0.00104	0.00106	0.0165	0.00739	0.0481	0.00467
3L:17992580-17992730:minus	-11.6224	0.0275229	1.3945	2.89908	-18.4184	-18.9796	-12.8073	-0.861111
3.40367	0.000259	0.0102	0.00719	0.00938	0.0101	0.0155	0.00553	0.0217	0.00603
3L:17992580-17992730:plus	-22.3265	0.550459	2.01835	9.59633	-26.8571	0.0918367	-7.50459	0.486111
3.29358	0.00337	0.00851	0.00574	0.000275	0.0198	0.000323	0.00189	0.0144	0.00623
3L:17994740-17994890:minus	-22.9694	-1.11927	-1.3945	3.45872	-28.0408	-18.7857	-17.0092	8.06944
11.8073	0.0048	0.0149	0.018	0.00731	0.0455	0.0146	0.0128	0.000561	0.0000538
3L:17994740-17994890:plus	-29.3673	0.743119	9.02752	10.1468	-18.051	-8.45918	-31.4771	9.11111	-3.83486
0.00838	0.00794	0.00025	0.000128	0.00746	0.00111	0.111	0.000298	0.0533
3L:17998060-17998210:minus	-23.0816	-0.541284	-3.00917	1.58716	-26.1939	-17.7143	-14.1376	7.69444
7.84404	0.00522	0.0124	0.0289	0.017	0.0175	0.00634	0.00748	0.000693	0.000701
3L:17998060-17998210:plus	-23.2551	9.3578	-0.357798	3.77064	-17.2653	-18.9286	-11.0275	7.44444
1.73394	0.00583	0.000186	0.013	0.00637	0.00387	0.0153	0.00375	0.000795	0.0106
3L:1802900-1803050:minus	-13.8163	0.229358	5.73394	1.6422	10.6531	-27.7041	-12.5321	5.79167	3.42202
0.00109	0.00953	0.0013	0.0166	0.0000102	0.0445	0.00519	0.00183	0.00597
3L:1802900-1803050:plus	-31.5816	-1.86239	-0.706422	-1.02752	-27.7959	-8.79592	-13.1101	2.625
4.80734	0.0135	0.0187	0.0146	0.037	0.0386	0.0019	0.00593	0.00687	0.00334
3L:18040240-18040390:minus	-22.9694	-5.3211	6.3945	3.61468	-17.8571	-18.2041	-14.1376	4.45833
0.944954	0.0048	0.048	0.000966	0.0068	0.00698	0.0103	0.00748	0.00332	0.0145
3L:18040240-18040390:plus	-41.2857	0.238532	8.00917	6.74312	-27.2653	-27.9286	-17.0183	2.93056	0.0183486
0.0521	0.0095	0.000435	0.0015	0.0302	0.054	0.0129	0.00612	0.0183
3L:18054920-18055070:minus	-22.2959	-2.55046	1.08257	9.51376	-18.7857	-9.16327	6.2844	4.86111
3.52294	0.00335	0.0227	0.00803	0.000292	0.0148	0.00223	0.000038	0.00279	0.00558
3L:18054920-18055070:plus	-30.5102	-0.87156	-2.68807	2.07339	-27	-25.8265	-10.0459	4.79167	7.68807
0.00947	0.0138	0.0264	0.0138	0.0233	0.0212	0.0031	0.00288	0.000841
3L:18066840-18066990:minus	-23	2.10092	5.38532	9.61468	-26.1939	-18.051	-5.21101	3.43056	-2.21101
0.00498	0.00484	0.00151	0.000255	0.0175	0.00886	0.00105	0.00505	0.0354
3L:18066840-18066990:plus	-34.0408	-5.55046	3.44037	-0.614679	-18.7857	-8.53061	-20.1101	8.22222
8.11009	0.0309	0.0509	0.00335	0.0324	0.0148	0.00136	0.021	0.000514	0.000597
3L:18099300-18099450:minus	-30.8776	0.330275	1.44954	11.1927	-8.60204	-18.8571	-17.4312	8.44444
-1.22018	0.0103	0.00919	0.00705	0.0000254	0.00125	0.0151	0.0137	0.000451	0.0278
3L:18099300-18099450:plus	-32.4796	-2.44037	4.37615	1.86239	-18.8265	-7.5	-15.211	-0.180556	0.917431
0.02	0.0221	0.0023	0.0153	0.0149	0.000614	0.00936	0.0177	0.0146
3L:18102760-18102910:minus	-22.9286	8.54128	6.43119	6.19266	-7.93878	-18.7857	-12.4037	2.05556
-2.94495	0.0046	0.000285	0.000949	0.00216	0.000767	0.0146	0.00505	0.00845	0.0441
3L:18102760-18102910:plus	-13.0714	3.31193	1.66972	1.69725	-8.93878	-26.7857	-11.156	5.54167	-0.972477
0.000594	0.0031	0.00652	0.0163	0.00216	0.0266	0.00385	0.00205	0.0259
3L:18105940-18106090:minus	-22	-1.0367	-0.761468	6.12844	-18.0102	-16.3469	-25.2569	5.27778
3.87156	0.00291	0.0145	0.0148	0.00231	0.00732	0.00416	0.0488	0.00232	0.00477
3L:18105940-18106090:plus	-23.449	-0.642202	14.055	9.59633	-18.7551	-8.94898	-14.0917	3.72222
5.22936	0.00631	0.0128	6.79e-06	0.000275	0.0144	0.00203	0.0074	0.0045	0.00288
3L:18107460-18107610:minus	-33.7449	0.862385	4.30275	6.69725	-19.051	-17.0816	-15.2752	3.52778
-1.6789	0.0295	0.00761	0.00237	0.00156	0.0162	0.00487	0.00948	0.00486	0.0306
3L:18107460-18107610:plus	-22.551	-4.43119	0.798165	1.48624	-18.1939	-18.051	-20.4495	-3.01389	6.49541
0.00359	0.038	0.00886	0.0177	0.0086	0.00886	0.0223	0.0388	0.00154
3L:18132280-18132430:minus	-31.7041	-1.27523	12.9266	7.80734	-17.1531	-8.57143	-20.7523	-1.79167
-3.81651	0.0146	0.0156	0.0000219	0.00063	0.00364	0.00149	0.0235	0.0282	0.0529
3L:18132280-18132430:plus	-31.2449	0.577982	2.82569	9.7156	-17.0816	-9.72449	5.11009	0.861111	-1.17431
0.0111	0.00843	0.00424	0.000242	0.00334	0.00352	0.0000555	0.0127	0.0275
3L:18391520-18391670:minus	-11.9184	2.77064	2.33028	10.8624	-18.7143	-8.82653	1.81651	13.5972
7.88991	0.000293	0.00378	0.00511	0.0000687	0.0136	0.00191	0.000175	4.83e-06	0.000688
3L:18391520-18391670:plus	-4.18367	-0.678899	0.46789	7.17431	-17.1939	9.60204	-14.578	0.722222
7.24771	0.000128	0.0129	0.00992	0.00115	0.00377	0.0000248	0.00822	0.0133	0.00116

3L:18394380-18394530:minus -23.398 6.46789 3.3578 6.48624 -26.2653 -19.3469 -20.8991 4.94444
9.66055 0.00619 0.000847 0.00346 0.00177 0.018 0.0194 0.0241 0.00269 0.000268
3L:18394380-18394530:plus -13.8163 -0.119266 9.29358 3.59633 0.612245 -16.2755 -14.7615 -0.0833333
-9.34862 0.00109 0.0107 0.000214 0.00693 0.000269 0.00413 0.00855 0.0172 0.175
3L:18395040-18395190:minus -24.1122 4.02752 2.22018 1.81651 -18.051 -18.2755 -11.5505 0.166667
4.77982 0.00738 0.00238 0.00532 0.0156 0.00746 0.0112 0.00418 0.0159 0.00339
3L:18395040-18395190:plus -21.7755 2.61468 7.3211 4.3211 -27.1633 2.05102 -10.367 -0.611111
-6.52294 0.00241 0.004 0.00062 0.00514 0.025 0.0000468 0.0033 0.0201 0.101
3L:18395500-18395650:minus -3.89796 -0.229358 1.70642 6.09174 -27.9388 -26.2653 1.58716 -2.31944
3.49541 0.000113 0.0111 0.00644 0.00235 0.0415 0.0218 0.000187 0.0325 0.0057
3L:18395500-18395650:plus -31.2551 0.412844 5.52294 6.02752 -17.6429 -9.94898 -16.1468 0.416667 3.20183
0.0113 0.00893 0.00142 0.00246 0.00562 0.00393 0.0111 0.0147 0.0065
3L:184520-184670:minus -22.9184 7.95413 10.1193 3.43119 -25.898 -7.5 7.92661 1.16667 -1.54128
0.00447 0.000392 0.000133 0.00749 0.0166 0.000614 0.0000242 0.0115 0.0296
3L:184520-184670:plus -31.4388 3.97959 0.844037 6.21101 1.10204 -17.4184 -24.945 1.47222 -5.69725
0.0122 0.000669 0.00872 0.00213 0.000124 0.00585 0.0468 0.0104 0.0833
3L:1857000-1857150:minus -23.2551 5.44037 0.486239 3.24771 -17.7143 -18.3776 -17.3853 2.125 1.68807
0.00583 0.00135 0.00986 0.00808 0.00627 0.0125 0.0136 0.00824 0.0109
3L:1857000-1857150:plus -40.102 -0.862385 1.9633 7 20.2041 -9.34694 -23.8899 3.54167 4.6789
0.036 0.0137 0.00586 0.00132 3.64e-07 0.00261 0.0404 0.00483 0.00347
3L:18614460-18614610:minus -13.6531 -0.825688 3.26606 1.44037 -18.7143 -18.5612 -10.1193 1.5
4.36697 0.00101 0.0136 0.00358 0.018 0.0136 0.0132 0.00315 0.0103 0.00379
3L:18614460-18614610:plus -22.6939 -1.2844 5.37615 6.49541 -26.898 -17.9796 -2.83486 -2.26389 0.137615
0.00388 0.0157 0.00152 0.00176 0.0203 0.00764 0.000544 0.032 0.0178
3L:18614860-18615010:minus -23.9184 1.61468 4.51376 5.22936 -8.60204 -17.4898 -17.9083 4.52778
1.95413 0.00695 0.00578 0.00218 0.0041 0.00125 0.0059 0.0147 0.00322 0.00964
3L:18614860-18615010:plus -23.4388 2.40367 2.36697 0.577982 -18.6837 -18.051 -10.5229 -0.0972222
-0.669725 0.00625 0.00433 0.00505 0.0231 0.0126 0.00886 0.0034 0.0172 0.0236
3L:18615180-18615330:minus -21.5918 3.30275 13.4312 -0.449541 20.2041 -16.051 -15.6881 4.73611
3.66972 0.00209 0.00311 0.0000138 0.0307 3.64e-07 0.00408 0.0102 0.00295 0.00514
3L:18615180-18615330:plus -21 -1.9633 13.4037 6.73394 -18.4184 10.602 -13.2202 0.458333 2.11927
0.00186 0.0192 0.0000142 0.00152 0.0101 4.1e-06 0.00608 0.0145 0.00889
3L:1861740-1861890:minus -30.6633 -0.834862 1.56881 9.44037 -27.2653 -27.5612 -15.1468 1.36111
10.3394 0.00976 0.0136 0.00676 0.000339 0.0302 0.0393 0.00924 0.0108 0.000112
3L:1861740-1861890:plus -30.2143 -5.54128 4.26606 6.31193 -8.11224 -18.3061 9.84404 2.86111 0.908257
0.0088 0.0508 0.00241 0.00199 0.000838 0.0114 0.0000137 0.00629 0.0147
3L:18618980-18619130:minus -33.2653 -0.0917431 4.80734 -1.07339 -18.7551 -0.244898 -23.3578
3.81944 1.2844 0.0277 0.0106 0.00193 0.0377 0.0144 0.000496 0.0372 0.00433 0.0129
3L:18618980-18619130:plus -23 3.15596 13.055 1.88991 -17.1531 -0.316327 -5.6055 4.34722 -5.05505
0.00498 0.00328 0.0000195 0.0151 0.00364 0.000524 0.00118 0.00348 0.0736
3L:1865220-1865370:minus -22.1837 -5.74312 3.16514 2.55046 0.571429 -26.5612 -9.45872 4.36111 1.16514
0.00315 0.0535 0.00373 0.0112 0.0003 0.0237 0.00278 0.00346 0.0135
3L:1865220-1865370:plus -23.8571 -4.40367 -1.36697 0.513761 10.6531 1.2449 -4.82569 7.86111 3.88991
0.00687 0.0377 0.0179 0.0235 0.0000102 0.0000797 0.000945 0.000631 0.00467
3L:18665500-18665650:minus -20.9694 -0.0366972 2.6422 5.38532 -17.1531 -9.20408 6.61468 0.986111
5.22936 0.00184 0.0104 0.00455 0.00382 0.00364 0.00225 0.0000346 0.0122 0.00288
3L:18665500-18665650:plus -31.2551 -2.53211 -2.65138 -2.6789 -27.0408 -37.5204 -6.23853 6.625 6.26606
0.0113 0.0226 0.0261 0.0637 0.0242 0.166 0.0014 0.00122 0.00162
3L:18666240-18666390:minus -21.9286 1.59633 6.53211 7.6422 -27.3367 -0.173469 2.45872 -1.91667
7.72477 0.0027 0.00582 0.000904 0.000721 0.0317 0.000456 0.000142 0.0291 0.000798
3L:18666240-18666390:plus -31.5204 13.4592 2.55963 6.06422 -17.4898 -18.2755 -13.7706 -2.20833 -2.42202
0.0131 0.000064 0.0047 0.00239 0.0053 0.0112 0.00689 0.0315 0.0378
3L:18668560-18668710:minus -31.4388 2.31193 1.77064 6.44037 -8.33673 -8.7551 -3.40367 2.09722
1.06422 0.0122 0.00448 0.00629 0.00185 0.00101 0.00172 0.000632 0.00832 0.014

3L:18668560-18668710:plus -14.8163 0.146789 15 7.29358 -18.7143 -18.2755 -14.1101 3.80556 7.11927
0.00159 0.0098 1.62e-06 0.00104 0.0136 0.0112 0.00743 0.00435 0.00125
3L:18675120-18675270:minus -31.9184 -4.53211 5.84404 5.47706 -17.4184 -18.5714 -17.578 0.638889
-0.486239 0.0164 0.039 0.00124 0.0036 0.00457 0.0136 0.014 0.0137 0.0219
3L:18675120-18675270:plus -32.8469 1.3578 4.33945 0.733945 10.6531 -18.3061 -18.9266 4.375 2.36697
0.0243 0.00635 0.00234 0.0222 0.0000102 0.0114 0.0172 0.00344 0.00828
3L:18677060-18677210:minus -22.5204 -1.56881 3.82569 6.08257 10.3571 -25.7551 -15.2752 8.09722
1.90826 0.00356 0.0171 0.00288 0.00236 0.0000208 0.0208 0.00948 0.000552 0.00974
3L:18677060-18677210:plus -39.551 -0.908257 3.88073 5.65138 -18.4898 -17.7551 -5.22936 2.19444
-4.58716 0.0335 0.0139 0.00281 0.00322 0.0116 0.00643 0.00106 0.00804 0.0652
3L:18738860-18739010:minus -33.3367 4.89908 1.63303 7.00917 -28.8265 -8.79592 -19.7064 7.98611
3.46789 0.0281 0.0017 0.00661 0.00126 0.0648 0.0019 0.0196 0.000588 0.00582
3L:18738860-18739010:plus -22.8163 -2.11009 -0.651376 0.917431 20.2041 -8.65306 -20.7982 7.20833
3.42202 0.00418 0.0201 0.0143 0.021 3.64e-07 0.00156 0.0237 0.000902 0.00597
3L:18741640-18741790:minus -31.7041 7.95413 -3.61468 6.27523 20.1735 9.7551 -5.10092 3.44444
3.46789 0.0146 0.000392 0.0342 0.00206 5.68e-07 0.0000198 0.00102 0.00502 0.00582
3L:18741640-18741790:plus -20.6633 -0.137615 6.08257 7 -18.4184 -9.02041 -14.4404 -0.263889
10.1284 0.00173 0.0108 0.00111 0.00132 0.0101 0.00213 0.00799 0.0181 0.000129
3L:18758940-18759090:minus -24.2653 5.27523 2.22018 7.43119 -18.7143 -8.42857 -14.8349 -6.5 1.24771
0.00773 0.00145 0.00532 0.000939 0.0136 0.00104 0.00868 0.0841 0.0131
3L:18758940-18759090:plus -33 2.09174 -1.77982 2.29358 -17.9796 -9.57143 -0.238532 -2.38889 7.58716
0.0258 0.00485 0.0202 0.0124 0.00726 0.00326 0.000299 0.0331 0.000954
3L:1876320-1876470:minus -30.5204 -2.87156 5.25688 3.54128 2.21429 9.86735 -4.01835 2.95833 9.66055
0.00951 0.0249 0.0016 0.00702 0.0000277 0.0000132 0.00075 0.00606 0.000268
3L:1876320-1876470:plus -41.2857 0.926606 8.24771 6.73394 -18.9796 -27.5612 -24.0092 1.86111 -0.807339
0.0521 0.00743 0.000384 0.00152 0.0155 0.0393 0.0411 0.00905 0.0247
3L:18784140-18784290:minus -30.7041 -3.22936 10.2477 6.21101 -27.3367 -19.0816 -7.20183 3.29167
9.61468 0.00993 0.0275 0.000123 0.00213 0.0317 0.017 0.00177 0.00533 0.00029
3L:18784140-18784290:plus -31.7041 4.3211 2.10092 4.07339 -17.7857 -8.72449 -11.6789 -0.180556
-0.33945 0.0146 0.00213 0.00556 0.00562 0.00676 0.00169 0.0043 0.0177 0.0209
3L:18793480-18793630:minus -23.1837 2.22018 5.11927 0.110092 -26.9694 -8.79592 -9.48624 9.77778
11.8532 0.00553 0.00463 0.00169 0.0261 0.023 0.0019 0.0028 0.000192 0.0000428
3L:18793480-18793630:plus -14.5204 -1.6055 0.357798 5.69725 -27.1224 -19.2755 -5.82569 3.44444 5.08257
0.00145 0.0173 0.0103 0.00314 0.0245 0.0185 0.00125 0.00502 0.00303
3L:18794460-18794610:minus -30.4796 0.165138 -2.04587 6.78899 -26.898 0.387755 -21.1927 -3.75
2.18349 0.00941 0.00974 0.0219 0.00146 0.0203 0.000217 0.0255 0.0464 0.00871
3L:18794460-18794610:plus -14.7449 5.77982 6.78899 7.55963 -27.1939 -9.30612 -17.4312 7.23611 3.20183
0.00157 0.00117 0.000801 0.000807 0.0267 0.00257 0.0137 0.000889 0.0065
3L:18805500-18805650:minus -22.2551 -2.41284 0.458716 3.00917 -25.9286 -19.3469 -10.0734 1.93056
7.68807 0.00327 0.0219 0.00995 0.00894 0.0168 0.0194 0.00312 0.00883 0.000841
3L:18805500-18805650:plus -34.2245 2.58716 2.20183 -0.0550459 -18.7143 -27.6735 -15.0826 8.16667
4.92661 0.0315 0.00404 0.00536 0.0272 0.0136 0.0443 0.00912 0.00053 0.00318
3L:18807420-18807570:minus -14.3673 -1.81651 3.85321 2.22936 -26.6327 1.09184 -13.8991 -2.05556
3.40367 0.00132 0.0184 0.00284 0.0128 0.0188 0.000091 0.00709 0.0303 0.00603
3L:18807420-18807570:plus -31.9286 2.08257 5.89908 7.47706 -27.4592 0.0918367 -26.9817 -0.833333
-8.77064 0.0166 0.00487 0.00121 0.00085 0.0329 0.000323 0.0603 0.0215 0.159
3L:18813120-18813270:minus -31 -0.697248 -0.33945 -0.146789 -28.2245 -17.2755 -23.6514 8.16667
2.98165 0.0105 0.013 0.0129 0.028 0.049 0.00544 0.0389 0.00053 0.00707
3L:18813120-18813270:plus -23.1531 -1.68807 -0.59633 -0.954128 -27.0816 0.826531 -20.2844 0.208333
3.17431 0.00542 0.0177 0.0141 0.0362 0.0243 0.000153 0.0216 0.0157 0.00653
3L:18813500-18813650:minus -23.3776 -4.97248 -1.17431 -1.13761 -28.3061 -9.72449 -10.9633 2.08333
10.0367 0.00619 0.0438 0.0169 0.0383 0.0553 0.00352 0.00371 0.00837 0.000146
3L:18813500-18813650:plus -30.4388 2.53211 -1.33028 2.70642 -26.1939 -8.65306 -10.211 -2.45833 -1.29358
0.00923 0.00413 0.0177 0.0103 0.0175 0.00156 0.0032 0.0337 0.0283

3L:18818160-18818310:minus -32.0408 0.788991 0.963303 2 -18.4184 -19.1531 -18.156 2.25 1.70642
0.0174 0.00781 0.00837 0.0143 0.0101 0.0177 0.0153 0.00788 0.0108
3L:18818160-18818310:plus -23.0714 0.752294 -1.3578 4.48624 -17.4898 -26.8265 -11.8807 4.43056 -0.541284
0.0052 0.00791 0.0178 0.00487 0.0053 0.0285 0.00449 0.00336 0.0223
3L:18831220-18831370:minus -20.9286 3.86239 5.37615 3.43119 -18.7551 -16.9796 -21.4587 -1.47222
3.83486 0.00183 0.00253 0.00152 0.00749 0.0144 0.0044 0.0267 0.0258 0.00481
3L:18831220-18831370:plus -40.5102 7.49541 3.61468 3.81651 -8.86735 -18.0102 -11.7431 4.54167 2.33028
0.0409 0.0005 0.00313 0.00626 0.00173 0.00812 0.00436 0.0032 0.00836
3L:18833520-18833670:minus 6.36735 6.11009 -1.62385 11.2936 -17.9286 -26.9694 -2.3211 -2.80556
4.66055 3.84e-06 0.00101 0.0193 0.0000139 0.0071 0.0304 0.000479 0.0368 0.0035
3L:18833520-18833670:plus -31.0306 -3.53211 0.633028 3.91743 -8.64286 -26.6327 -13.2018 -1.09722 9.87156
0.0105 0.0298 0.00938 0.00601 0.00139 0.0251 0.00605 0.0232 0.000217
3L:18834500-18834650:minus -22.4898 8.33028 6.30275 -0.880734 -7.71429 -17.2755 -24.5321 3.58333
2.04587 0.00353 0.000319 0.00101 0.0353 0.000572 0.00544 0.0443 0.00475 0.00919
3L:18834500-18834650:plus -23.1531 3.54128 1.14679 6.00917 1.61224 -18.1224 -26.7798 -1.20833 -3.27523
0.00542 0.00285 0.00785 0.00255 0.0000631 0.00988 0.0589 0.0239 0.0473
3L:1884820-1884970:minus -22.8878 1.45872 14.8624 7.37615 -17.1531 -27.6327 -7.06422 -0.333333
-3.77064 0.00441 0.00613 2.2e-06 0.000951 0.00364 0.043 0.00171 0.0185 0.0524
3L:1884820-1884970:plus -14.3061 7.01835 12.3853 7.2844 -17.5204 -9.57143 -4.11009 0.361111 0.715596
0.00129 0.000643 0.0000329 0.00105 0.00532 0.00326 0.00077 0.0149 0.0154
3L:18859180-18859330:minus -22.6633 4.46789 -5.50459 4.07339 -27.8673 1.7551 -22.578 5.95833
-0.504587 0.00384 0.00201 0.0561 0.00562 0.0391 0.0000519 0.0327 0.00169 0.0221
3L:18859180-18859330:plus -22 0.972477 3.68807 5.49541 -26.9694 1.31633 -12.5321 2.77778 -0.633028
0.00291 0.00731 0.00304 0.00355 0.023 0.000066 0.00519 0.00649 0.0232
3L:18860760-18860910:minus -14.3776 0.825688 1.84404 7.00917 -18.5204 9.7551 -21.1193 3.44444
2.25688 0.00134 0.00771 0.00612 0.00126 0.0117 0.0000198 0.0251 0.00502 0.00853
3L:18860760-18860910:plus -30.1735 9.0367 14.9266 5.90826 -27.2653 -18.2347 -19.211 2.34722 1.46789
0.00869 0.000219 1.9e-06 0.00271 0.0302 0.0104 0.018 0.00761 0.0119
3L:18865760-18865910:minus -34.0714 -1.48624 4.38532 1.75229 -8.85714 -16.2041 -18.2844 2.41667
1.25688 0.031 0.0167 0.0023 0.0159 0.00165 0.00408 0.0156 0.00742 0.0131
3L:18865760-18865910:plus -39.9592 -2.22936 3.6422 9.44954 20.2041 -27.9694 -14.2385 -1.25 -1.01835
0.0348 0.0208 0.00309 0.000326 3.64e-07 0.0547 0.00765 0.0242 0.0263
3L:18881260-18881410:minus -31.9286 4.09174 2.25688 2.57798 -17.1939 -19.0816 -4.95413 8.66667
0.697248 0.0166 0.00232 0.00525 0.011 0.00377 0.017 0.00098 0.000394 0.0154
3L:18881260-18881410:plus -31.8469 -1.49541 0.0366972 9.44954 -27.5714 -28.1224 -17.3394 5.06944
4.0367 0.0162 0.0167 0.0115 0.000326 0.0357 0.0583 0.0135 0.00255 0.00424
3L:18883580-18883730:minus -21.9286 1.55963 1.58716 9.61468 -26.9694 -8.53061 -9.89908 -5.97222
-1.01835 0.0027 0.0059 0.00672 0.000255 0.023 0.00136 0.00302 0.0757 0.0263
3L:18883580-18883730:plus -14.1122 0.807339 2.62385 7.16514 -17.2245 -28.4082 -10.8716 2.72222 7.22018
0.00119 0.00776 0.00458 0.00116 0.00384 0.0615 0.00364 0.00663 0.00119
3L:18891400-18891550:minus -32.1837 -1.85321 2.75229 11.2936 -8.57143 -17.9796 -11.9633 2.77778
-0.440367 0.0181 0.0186 0.00436 0.0000139 0.00113 0.00764 0.00457 0.00649 0.0216
3L:18891400-18891550:plus -21 0.917431 6.20183 0.917431 -27.3061 -17.7857 -20.7706 3.72222 3.21101
0.00186 0.00746 0.00106 0.021 0.0315 0.0068 0.0236 0.0045 0.00644
3L:18894600-18894750:minus -13.6735 -0.100917 -2.08257 -0.825688 -18.4184 1.05102 -13.4771
6.68056 1.27523 0.00103 0.0107 0.0222 0.0347 0.0101 0.000107 0.00645 0.00119 0.013
3L:18894600-18894750:plus -30.8469 14.6789 5.78899 6.02752 -8.93878 -8.57143 -13.8349 5.56944 2.99083
0.0103 3.91e-06 0.00127 0.00246 0.00216 0.00149 0.00699 0.00203 0.00706
3L:18898300-18898450:minus -23.2959 2.31193 1.13761 5.31193 -18.7551 -0.173469 -19.9083 3.75
-0.761468 0.00605 0.00448 0.00788 0.00393 0.0144 0.000456 0.0203 0.00445 0.0244
3L:18898300-18898450:plus -24.7857 13.5963 0.302752 4.7156 -27.7551 0.755102 -12.6789 3.22222 5.13761
0.00829 0.0000131 0.0105 0.00464 0.0385 0.000175 0.00537 0.00548 0.00298
3L:18986040-18986190:minus -40.0306 5.70642 7.37615 5.47706 -18.3061 -26.898 -19.4862 1.95833
3.62385 0.0357 0.00121 0.000603 0.0036 0.00884 0.0302 0.0189 0.00875 0.00523

3L:18986040-18986190:plus -31.102 6.44037 -3.61468 2.9633 -18.3776 -27.051 -2.95413 -1.06944 -0.798165
0.0106 0.000858 0.0342 0.00914 0.00926 0.0315 0.000561 0.023 0.0246
3L:18987780-18987930:minus -22.6633 -2.31193 3.59633 -0.633028 -27.2653 -17.7857 -17.4954 5.23611
9.34862 0.00384 0.0213 0.00315 0.0327 0.0302 0.0068 0.0138 0.00236 0.000371
3L:18987780-18987930:plus -22 -2.93578 0.201835 9.59633 -27.2653 -18.1224 -10.7339 3.625 8.11009
0.00291 0.0253 0.0108 0.000275 0.0302 0.00988 0.00354 0.00468 0.000597
3L:18988940-18989090:minus -23.8571 5.24771 6.81651 2.66972 -18.1531 -27.2653 -6.61468 9.27778
3.72477 0.00687 0.00147 0.000791 0.0106 0.00829 0.0333 0.00154 0.000268 0.00497
3L:18988940-18989090:plus -21.1531 2.80734 3.65138 10.1468 -27.6327 -9.08163 -11.6055 -4.19444 -0.715596
0.00188 0.00373 0.00308 0.000128 0.0369 0.00216 0.00423 0.0515 0.0241
3L:18989820-18989970:minus -22.2857 -1.42202 8.04587 2.3578 -17.7857 -44.7041 -14.9725 -3.29167
4.11927 0.00331 0.0164 0.000428 0.0121 0.00676 0.203 0.00892 0.0416 0.00411
3L:18989820-18989970:plus -30.5918 -2.38532 2.04587 3.05505 -18.7551 -8.72449 -23.0275 -0.902778
-1.9633 0.00963 0.0217 0.00568 0.00878 0.0144 0.00169 0.0352 0.0219 0.0329
3L:18990340-18990490:minus -30.4388 0.963303 1.59633 2.54128 -17.9286 -17.8571 -12 3.48611 1.54128
0.00923 0.00734 0.00669 0.0112 0.0071 0.00719 0.00461 0.00494 0.0115
3L:18990340-18990490:plus -13.3367 3.3578 -1.43119 6.00917 -7.60204 -8.42857 -20.2477 8.22222 -4.11927
0.0008 0.00305 0.0182 0.00255 0.000466 0.00104 0.0215 0.000514 0.0572
3L:18993560-18993710:minus -30.7755 -2.61468 4.55963 1.69725 20.4694 -9.20408 -11.8073 4.45833
-2.04587 0.0102 0.0232 0.00214 0.0163 1.65e-07 0.00225 0.00442 0.00332 0.0336
3L:18993560-18993710:plus -23.0306 2.09174 3.33945 6.16514 -18.449 -16.7551 -14.422 12.25 5.3945
0.00505 0.00485 0.00348 0.00223 0.0107 0.0042 0.00796 0.0000241 0.00263
3L:18996560-18996710:minus -21.9286 0.0642202 8.56881 7 -26.5918 -18.5612 -13.7706 8.31944
-2.68807 0.0027 0.0101 0.00032 0.00132 0.0186 0.0132 0.00689 0.000485 0.041
3L:18996560-18996710:plus -30.6327 5.33945 -1.91743 6.21101 -8.93878 -8.09184 -17.5229 3.77778 -2.42202
0.00968 0.00142 0.0211 0.00213 0.00216 0.000762 0.0139 0.0044 0.0378
3L:19038480-19038630:minus -30.7755 5.21101 2.82569 5.43119 -18.7143 -8.45918 3.36697 -1.94444
1.9633 0.0102 0.00149 0.00424 0.00371 0.0136 0.00111 0.000104 0.0294 0.00951
3L:19038480-19038630:plus -13.5204 4.21101 3.07339 3.76147 -18.7551 -18.7143 -1.38532 -5.18056 3.20183
0.00097 0.00222 0.00386 0.00645 0.0144 0.014 0.000386 0.0641 0.0065
3L:19056020-19056170:minus -22.5918 0.743119 7.94495 7.37615 -27.1939 -17.9388 -18.6422 1.15278
1.43119 0.00365 0.00794 0.000451 0.000951 0.0267 0.00738 0.0165 0.0115 0.0121
3L:19056020-19056170:plus -31.4388 -4.6789 0.715596 -1.11927 10.6939 -26.2653 -15.4037 -1.70833 -2.47706
0.0122 0.0405 0.00912 0.0381 7.35e-06 0.0218 0.00972 0.0275 0.0385
3L:19064480-19064630:minus -30.9694 3.52294 4.06422 1.69725 -18.7143 -8.42857 -16.4495 7.41667
5.25688 0.0104 0.00287 0.00261 0.0163 0.0136 0.00104 0.0117 0.000807 0.0028
3L:19064480-19064630:plus -22.8163 1.94495 2.66972 4.07339 -6.89796 -8.5 -22.5413 1.59722 -0.504587
0.00418 0.00512 0.0045 0.00562 0.000364 0.00126 0.0324 0.00993 0.0221
3L:19065380-19065530:minus -23.8163 0.633028 4.6422 0.12844 -27.2755 -17.7551 -8.84404 -5.94444
2.58716 0.00682 0.00826 0.00206 0.0259 0.0302 0.00643 0.00248 0.0752 0.00779
3L:19065380-19065530:plus -31.5204 -1.41284 -0.0366972 0.889908 -18.449 -8.94898 -15.5596 1.38889
5.7156 0.0131 0.0163 0.0117 0.0213 0.0107 0.00203 0.01 0.0107 0.00217
3L:19068780-19068930:minus -23.0816 -1.55046 -0.853211 2.33945 -27.898 -17.9796 -13.2385 4.33333
-1.90826 0.00522 0.017 0.0152 0.0122 0.04 0.00764 0.00611 0.0035 0.0324
3L:19068780-19068930:plus -24.3367 9.63303 -0.394495 5.31193 -28.4184 -8.45918 -24.2569 5.79167
-2.22018 0.00789 0.000161 0.0132 0.00393 0.056 0.00111 0.0426 0.00183 0.0355
3L:19077560-19077710:minus -21.5102 1.99083 -1.11009 6.29358 -19.051 -16.3469 -13.6606 6.38889
-3.74312 0.00202 0.00504 0.0165 0.00202 0.0162 0.00416 0.00672 0.00137 0.0521
3L:19077560-19077710:plus -22.8878 -0.247706 -3.16514 9.24771 0.581633 -9.53061 -14.6697 -0.0833333
7.72477 0.00441 0.0112 0.0302 0.000409 0.000292 0.0031 0.00838 0.0172 0.000798
3L:19079520-19079670:minus -21.8878 -1.34862 -0.577982 -0.394495 -26.8571 -19.2755 -10.6881
8.65278 9.56881 0.00258 0.016 0.014 0.0301 0.0198 0.0185 0.00351 0.000397 0.000303
3L:19079520-19079670:plus -22.9898 1.11927 -0.972477 1.95413 -8.40816 -18.051 5.77982 5.875
3.29358 0.00482 0.00693 0.0158 0.0147 0.00108 0.00886 0.0000444 0.00176 0.00623

3L:19080080-19080230:minus -14.3367 -4.22936 1.74312 -0.642202 -9.20408 -9.34694 -13.9358 -0.152778
10.0367 0.00131 0.036 0.00635 0.0328 0.00262 0.00261 0.00715 0.0175 0.000146
3L:19080080-19080230:plus -32.3265 4.61468 -0.972477 2.53211 -18.1224 -26.3367 -8.10092 -1.41667
-0.899083 0.0187 0.0019 0.0158 0.0113 0.00782 0.0221 0.00215 0.0254 0.0254
3L:19082300-19082450:minus -32.6633 3.26606 0.834862 3.56881 -26 -26.898 -21.4495 6.76389 4.75229
0.0216 0.00315 0.00875 0.00699 0.0171 0.0302 0.0267 0.00114 0.00341
3L:19082300-19082450:plus -31.7449 2.37615 -0.834862 2.41284 -18.4184 -18.7857 -5.44037 -5.22222
7.11009 0.0152 0.00437 0.0152 0.0118 0.0101 0.0146 0.00112 0.0647 0.00128
3L:19082840-19082990:minus -12.1122 -2.44037 1.55046 2.43119 -17.6429 -8.72449 -12.6422 0.361111
5.79817 0.000336 0.0221 0.0068 0.0117 0.00562 0.00169 0.00533 0.0149 0.00208
3L:19082840-19082990:plus -31.0102 -0.33945 -1.95413 1.48624 -17.3776 -9.5 -20.0642 -1.27778 -0.0275229
0.0105 0.0116 0.0213 0.0177 0.00419 0.00294 0.0208 0.0244 0.0186
3L:19084780-19084930:minus -22.7755 -1.14679 8.61468 7.52294 -18.7245 1.12245 -6 3.61111 5.66055
0.00412 0.015 0.000312 0.000819 0.0136 0.000085 0.00131 0.0047 0.00227
3L:19084780-19084930:plus -31.4388 6.21101 1.98165 4.33028 -17.2245 -17.8571 -12.633 -1.86111 -2.47706
0.0122 0.000958 0.00582 0.00511 0.00384 0.00719 0.00532 0.0287 0.0385
3L:19085660-19085810:minus -30.6735 13.6239 1.04587 1.99083 1.54082 -8.23469 -13.5596 6.52778
-3.06422 0.00978 0.000013 0.00813 0.0144 0.0000712 0.000875 0.00657 0.00128 0.0453
3L:19085660-19085810:plus -23.2551 -0.174312 14.8624 2.78899 20.2041 -18.8571 -21.1101 0.208333
3.36697 0.00583 0.0109 2.2e-06 0.00992 3.64e-07 0.0151 0.0251 0.0157 0.00607
3L:19090920-19091070:minus -31.551 8.18349 1.14679 1.6422 -8.67347 -17.3469 -9.99083 -1.16667
1.3211 0.0134 0.000345 0.00785 0.0166 0.00154 0.0058 0.00307 0.0237 0.0127
3L:19090920-19091070:plus -23.2245 6.44954 5.15596 4.40367 -27.6327 -28.8265 -7.9633 -0.5 -0.504587
0.00576 0.000854 0.00167 0.00497 0.0369 0.0692 0.00209 0.0195 0.0221
3L:19093380-19093530:minus -11.1122 0.577982 0.862385 4.27523 20.4694 -17.7857 -15.0734 -3.375
-0.853211 0.000244 0.00843 0.00867 0.00518 1.65e-07 0.0068 0.00911 0.0424 0.0251
3L:19093380-19093530:plus -31.7041 0.880734 -4.61468 1.84404 -18.1531 -17.7857 -12.0917 -0.277778
1.78899 0.0146 0.00756 0.0447 0.0154 0.00829 0.0068 0.00471 0.0182 0.0103
3L:19163660-19163810:minus -2.33673 2.29358 6.17431 10.7615 -18.4184 -17.8265 -2.58716 -8.58333
7.83486 0.0000294 0.00451 0.00107 0.0000826 0.0101 0.00699 0.000511 0.123 0.000712
3L:19163660-19163810:plus -32.9286 -0.495413 1.07339 1.41284 -9.23469 -8.42857 -10.5229 6.375
2.58716 0.025 0.0122 0.00806 0.0183 0.00277 0.00104 0.0034 0.00138 0.00779
3L:19228560-19228710:minus -39.2551 -3.43119 6.57798 10.5872 -9.0102 -19.1224 -13.1376 -0.833333
2.98165 0.0331 0.029 0.000884 0.000115 0.00236 0.0175 0.00597 0.0215 0.00707
3L:19228560-19228710:plus -23.2959 3.90816 0.211009 2.77064 -18.4898 -19.1224 -5.61468 7.625 4.56881
0.00605 0.00102 0.0108 0.01 0.0116 0.0175 0.00118 0.00072 0.00356
3L:19248080-19248230:minus -22.9286 -0.688073 14.844 -0.174312 -8.93878 0.0510204 -4.88991
3.58333 2.73394 0.0046 0.013 2.33e-06 0.0283 0.00216 0.000349 0.000962 0.00475 0.00756
3L:19248080-19248230:plus -22.4388 3.07339 1.23853 0.844037 -18.1122 -27.4082 -13.3853 2.70833 1.6422
0.00348 0.00339 0.0076 0.0215 0.00771 0.0356 0.00631 0.00666 0.0111
3L:19253460-19253610:minus -22.8878 -0.587156 1.86239 9.33028 -17.3776 -26.5612 -17.4679 -1.59722
-0.0733945 0.00441 0.0126 0.00608 0.000374 0.00419 0.0237 0.0138 0.0267 0.0188
3L:19253460-19253610:plus -14.449 -1.19266 -1.05505 -0.53211 -17.6837 -27.3367 -17.0367 -0.305556
7.6422 0.0014 0.0152 0.0162 0.0314 0.0058 0.0347 0.0129 0.0184 0.000893
3L:19254480-19254630:minus -13 -1.33028 -0.504587 6.00917 -18.4184 -19.2347 -18.1009 2.61111
4.80734 0.000563 0.0159 0.0137 0.00255 0.0101 0.018 0.0152 0.00691 0.00334
3L:19254480-19254630:plus -33.5612 2.15596 -1.41284 1.89908 20.2041 -18.2755 -21.9358 5.44444 0.669725
0.0288 0.00474 0.0181 0.0151 3.64e-07 0.0112 0.0291 0.00215 0.0155
3L:19257260-19257490:minus -22.8061 -4.22936 -0.770642 2.47706 10.7245 -8.79592 -15.3028 2.11111
-0.229358 0.00414 0.036 0.0149 0.0115 5.2e-06 0.0019 0.00953 0.00828 0.0202
3L:19257260-19257490:plus -14.3673 3.79817 7.18349 6.37615 0.877551 -0.244898 -2.98165 -0.236111
2.85321 0.00132 0.00259 0.000665 0.00192 0.00019 0.000496 0.000565 0.018 0.00733
3L:19262000-19262150:minus -30.1735 5.41284 8.69725 11.0183 -8.53061 -18.7857 -9.22018 -0.902778
-0.440367 0.00869 0.00137 0.000299 0.000052 0.0011 0.0146 0.00266 0.0219 0.0216

3L:19262000-19262150:plus -32.5204 -1.27523 5.93578 6.69725 -18.7551 -9.20408 -19.7982 0.277778 1.82569
0.0204 0.0156 0.00119 0.00156 0.0144 0.00225 0.0199 0.0153 0.0101
3L:19285640-19285790:minus -30.5102 -0.146789 5.47706 10.7615 -8.96939 -9.5 -10.9541 -1.86111
5.18349 0.00947 0.0108 0.00145 0.0000826 0.00226 0.00294 0.0037 0.0287 0.00295
3L:19285640-19285790:plus -33.2245 0.376147 -4.07339 0.366972 -26.5714 -0.244898 -24.789 5.58333
5.3945 0.0276 0.00905 0.0387 0.0244 0.0185 0.000496 0.0459 0.00202 0.00263
3L:19287940-19288090:minus -31.7347 9.26606 0.53211 -1.25688 -18.7857 -27.5612 -9.84404 -3.66667
-2.11009 0.0149 0.000195 0.00971 0.0399 0.0148 0.0393 0.00299 0.0455 0.0343
3L:19287940-19288090:plus -23.0816 8.66055 -1.17431 3.88991 -18.2653 -17.051 -16.6881 -2.18056 2.11927
0.00522 0.000268 0.0169 0.00608 0.00882 0.00479 0.0122 0.0313 0.00889
3L:1940940-1941090:minus -22.9898 4.66972 -4.80734 2.52294 -8.93878 -9.79592 -15.9174 6.81944 -0.12844
0.00482 0.00186 0.047 0.0113 0.00216 0.00377 0.0107 0.0011 0.0193
3L:1940940-1941090:plus -33.2653 13.5306 -2.61468 4.91743 -27.1224 -9.79592 -21.422 0.138889 -4.21101
0.0277 0.0000206 0.0259 0.00447 0.0245 0.00377 0.0266 0.016 0.0586
3L:1947640-1947790:minus -13.0816 2.87156 1.74312 2.93578 -27.4592 -8.45918 -11.8624 10.1806 -4
0.000599 0.00365 0.00635 0.00927 0.0329 0.00111 0.00447 0.000144 0.0558
3L:1947640-1947790:plus -3.63265 5.62385 0.752294 4.04587 -8.64286 -9.68367 -7.88073 -2.16667 -4.55963
0.0000917 0.00125 0.009 0.00572 0.00139 0.00342 0.00205 0.0312 0.0648
3L:19545400-19545550:minus -24.0612 15 0.119266 0.00917431 -18.8265 -36.3776 4.55963 4.09722
10.3486 0.00726 2.04e-06 0.0111 0.0269 0.0149 0.104 0.0000673 0.00386 0.0000988
3L:19545400-19545550:plus -21.7347 1.59633 2.77064 6.12844 -18.449 -9.5 -16.3394 -0.722222 -3.19266
0.00235 0.00582 0.00433 0.00231 0.0107 0.00294 0.0115 0.0208 0.0466
3L:19579100-19579250:minus -41.7041 4.11009 4.59633 2.26606 -19.051 -18.2755 -15.7615 6.04167
-2.94495 0.0641 0.00231 0.0021 0.0126 0.0162 0.0112 0.0104 0.00162 0.0441
3L:19579100-19579250:plus -23.4184 4.00917 0.706422 3.6055 10.9898 -9.72449 -20.6147 0.277778 -2.55046
0.00624 0.0024 0.00914 0.00686 3.28e-06 0.00352 0.0229 0.0153 0.0394
3L:19583620-19583770:minus -30.5102 -2.09174 11.5505 2.70642 -8.37755 -0.540816 -16.2661 3.73611
-3.10092 0.00947 0.0199 0.0000571 0.0103 0.00106 0.00054 0.0114 0.00447 0.0458
3L:19583620-19583770:plus -30.5102 1.87156 0.146789 2.66972 -26.9286 -18.3061 -16.5413 1.34722 -2.62385
0.00947 0.00526 0.011 0.0106 0.0214 0.0114 0.0119 0.0108 0.0402
3L:19591880-19592030:minus -31.9592 -3.11009 8.88991 6.54128 11.6531 0.979592 -20.211 1.80556
0.917431 0.0167 0.0266 0.00027 0.00173 1.55e-06 0.000136 0.0214 0.00923 0.0146
3L:19591880-19592030:plus -24.5204 0.816514 2.45872 3.47706 0.877551 -9.02041 -13.0367 3.30556 1.07339
0.00813 0.00773 0.00488 0.00724 0.00019 0.00213 0.00583 0.0053 0.014
3L:19605380-19605530:minus -23.5612 5.17431 4.89908 7.7156 1.17347 -27.2653 -24.0826 -0.638889
-0.229358 0.00653 0.00151 0.00185 0.000691 0.000101 0.0333 0.0415 0.0203 0.0202
3L:19605380-19605530:plus -40.4388 0.981651 -1.62385 2.81651 -27.4592 -9.45918 -2.05505 1.05556 1.57798
0.0401 0.00729 0.0193 0.00982 0.0329 0.00279 0.00045 0.0119 0.0114
3L:19611620-19611770:minus -14.102 6.98165 7.91743 3.88991 -18.7143 1.09184 -18.7248 -3.73611
-3.98165 0.00117 0.000656 0.000457 0.00608 0.0136 0.000091 0.0167 0.0463 0.0555
3L:19611620-19611770:plus -22.0306 3.37615 1.90826 3.94495 -18.4898 -18.051 -11.9083 -4.25 -2.73394
0.00293 0.00303 0.00598 0.00595 0.0116 0.00886 0.00452 0.0522 0.0416
3L:19611840-19611990:minus -23.4898 7.59633 5.46789 4.0367 -18.4184 -17.2041 -26.844 0.0416667
3.40367 0.00639 0.000474 0.00146 0.00578 0.0101 0.00507 0.0593 0.0165 0.00603
3L:19611840-19611990:plus -22.0306 -1.43119 12.0459 7.2844 1.5102 -9.53061 -2.68807 0.194444 7.37615
0.00293 0.0164 0.0000414 0.00105 0.0000722 0.0031 0.000525 0.0157 0.00107
3L:19626160-19626310:minus -15 4.11927 0.889908 7.44037 0.94898 -18.7143 -3.63303 0 1.45872
0.00162 0.0023 0.00859 0.000912 0.000134 0.014 0.000673 0.0167 0.0119
3L:19626160-19626310:plus -31.8163 0.412844 4.76147 1.69725 -8.57143 -17.051 -10.7523 1.63889 1.33028
0.016 0.00893 0.00196 0.0163 0.00113 0.00479 0.00355 0.00978 0.0127
3L:19635840-19635990:minus -12.8776 5.52294 3.94495 6.56881 1.61224 9.30612 -10.8349 1.22222
1.38532 0.000507 0.00131 0.00274 0.0017 0.0000631 0.0000398 0.00361 0.0113 0.0123
3L:19635840-19635990:plus -31.9286 2.20183 3 10.0275 -18.1939 -8.23469 -21.8073 1.38889 -0.651376
0.0166 0.00466 0.00397 0.000156 0.0086 0.000875 0.0285 0.0107 0.0235

3L:1964220-1964370:minus -14.551 -1.56881 1.36697 6.02752 -18.4898 -38.2551 -5.25688 2.27778 5.13761
0.00146 0.0171 0.00726 0.00246 0.0116 0.188 0.00107 0.0078 0.00298
3L:1964220-1964370:plus -31.6327 1.00917 13.5872 7.45872 -26.8571 -9.72449 -21.5138 -7.56944 -4.11009
0.0138 0.00722 0.0000122 0.000894 0.0198 0.00352 0.027 0.103 0.0571
3L:19642960-19643110:minus -31.2143 3.84404 4.33028 9.08257 -27.4082 -27.5612 -10.0826 14.1944 3
0.0111 0.00255 0.00235 0.000444 0.0321 0.0393 0.00313 1.94e-06 0.00702
3L:19642960-19643110:plus -23.9592 -4.20183 1.56881 -0.834862 -17.7143 -17.7551 -11.6972 6.11111
9.56881 0.00708 0.0357 0.00676 0.0349 0.00627 0.00643 0.00432 0.00157 0.000303
3L:1967700-1967850:minus -32.0714 4.11927 7.80734 3.61468 -27.3367 -27.5612 -18.633 8.45833 4.56881
0.0176 0.0023 0.000485 0.0068 0.0317 0.0393 0.0165 0.000447 0.00356
3L:1967700-1967850:plus -31.6633 2.26606 0.137615 5.37615 -8.96939 -17.3469 -16.3853 7.79167 1.2844
0.0141 0.00455 0.0111 0.00384 0.00226 0.0058 0.0116 0.000656 0.0129
3L:19680460-19680610:minus -31.4388 -4.41284 15.5596 7.29358 0.94898 -18.0102 -12.7431 2.63889
2.33028 0.0122 0.0378 6.69e-07 0.00104 0.000134 0.00812 0.00545 0.00684 0.00836
3L:19680460-19680610:plus -32.9592 2.05505 -0.651376 1.66972 -18.6429 -8.7551 -25.3028 5.95833
3.46789 0.0252 0.00492 0.0143 0.0165 0.0121 0.00172 0.049 0.00169 0.00582
3L:1969700-1969850:minus -23.2653 2.14679 1.06422 1.56881 -17.4082 -18.0816 -3.31193 8.98611 -4
0.00593 0.00476 0.00808 0.0172 0.00421 0.00934 0.000617 0.000323 0.0558
3L:1969700-1969850:plus -30.4388 2.62385 5.52294 6.72477 -18.1939 -0.102041 -13.633 3.04167
0.550459 0.00923 0.00399 0.00142 0.00154 0.0086 0.000443 0.00668 0.00587 0.0161
3L:1970040-1970190:minus -23.4898 0.944954 11.8349 10.5963 10.6224 -17.7143 -13.6881 -3.11111 8.10092
0.00639 0.00739 0.0000478 0.0001 0.0000113 0.00634 0.00676 0.0397 0.000623
3L:1970040-1970190:plus -31.7755 6.12844 11.1835 6.12844 -18.4592 -18.1531 -13.8165 -0.0833333
10.0367 0.0157 0.000997 0.000071 0.00231 0.0109 0.00997 0.00696 0.0172 0.000146
3L:19780900-19781050:minus -40.4898 -0.889908 -3.78899 2.13761 -27.1939 -16.0102 -22.8624 5.47222
-3.3578 0.0407 0.0138 0.0359 0.0134 0.0267 0.00406 0.0343 0.00212 0.0481
3L:19780900-19781050:plus -30.8776 2.95413 1.17431 2.97248 -18.4898 -8.72449 -25.7706 0.0555556
-2.77982 0.0103 0.00354 0.00778 0.00909 0.0116 0.00169 0.052 0.0164 0.042
3L:19783020-19783170:minus -13.551 0.834862 8.57798 5.86239 -8.60204 -26.051 -7.80734 1.52778
3.20183 0.000974 0.00769 0.000318 0.00282 0.00125 0.0216 0.00202 0.0102 0.0065
3L:19783020-19783170:plus -21.6633 1.3211 -2.3211 3.45872 -18.1531 -8.87755 -13.3945 -2 -2.88073
0.00223 0.00644 0.0237 0.00731 0.00829 0.00198 0.00633 0.0298 0.0432
3L:19784060-19784210:minus -42.3673 -1.24771 -0.0917431 -1.33945 -17.8878 -0.540816 -12.789
5.76389 0.220183 0.0755 0.0155 0.0119 0.0411 0.00704 0.00054 0.00551 0.00185 0.0174
3L:19784060-19784210:plus -40.1837 -1.34862 0.311927 2.7156 -18.6429 0.122449 -33.7798 5.22222 -1.80734
0.0367 0.016 0.0104 0.0102 0.0121 0.000312 0.143 0.00238 0.0316
3L:19784800-19784950:minus -24.0408 0.357798 -0.816514 5.22936 -17.5306 -9.72449 -15.1743 -5.59722
7.6789 0.00726 0.00911 0.0151 0.0041 0.0054 0.00352 0.00929 0.0701 0.000866
3L:19784800-19784950:plus -32.551 -0.0458716 3.6055 3.17431 -8.60204 -26.7143 -10.7798 0.583333
5.66055 0.0207 0.0105 0.00314 0.00842 0.00125 0.0257 0.00357 0.0139 0.00227
3L:19786020-19786170:minus -31.7041 1.66055 -2.77982 1.16514 -17.449 -0.469388 -28.9633 5.86111
-2.11009 0.0146 0.00569 0.0271 0.0197 0.00484 0.000533 0.0785 0.00177 0.0343
3L:19786020-19786170:plus -13.4898 -4.33945 10.6239 10.5963 -19.0204 -18.0102 -12.9817 -1.08333 11.8532
0.000946 0.037 0.0000983 0.0001 0.0158 0.00812 0.00576 0.0231 0.0000428
3L:19786860-19787010:minus -23.5204 -2.58716 -2.75229 0 10.6531 -9.30612 -13.844 1.375 0.642202
0.00645 0.023 0.0269 0.0269 0.0000102 0.00257 0.00701 0.0107 0.0157
3L:19786860-19787010:plus -23.8571 8.33028 6.22936 5.58716 -27.1939 -26.8265 -15.1376 2.81944 3.41284
0.00687 0.000319 0.00104 0.00334 0.0267 0.0285 0.00922 0.00639 0.006
3L:19787860-19788010:minus -41.551 -3.53211 5.04587 0.0275229 -27.2653 0.979592 -20.1651 2.5
-1.68807 0.0607 0.0298 0.00174 0.0267 0.0302 0.000136 0.0212 0.0072 0.0308
3L:19787860-19788010:plus -14.1429 1.68807 1.81651 2.00917 -8.37755 -18.0102 -9.26606 1.93056 4.80734
0.0012 0.00563 0.00619 0.0142 0.00106 0.00812 0.00269 0.00883 0.00334
3L:19794340-19794490:minus -32.7755 14.7523 7 2.66972 -17.7551 -9.64286 -25.4679 10.6667 2.82569
0.0234 3.48e-06 0.000727 0.0106 0.00658 0.00339 0.0501 0.0000996 0.0074

3L:19794340-19794490:plus -32.8878 15 12.2018 3.90826 -18.7857 -9.57143 -14.8807 4.95833 0.394495 0.0247 2.04e-06 0.0000374 0.00603 0.0148 0.00326 0.00876 0.00267 0.0166
3L:19798140-19798290:minus -24.7857 13.9083 2.76147 4.21101 -18.9388 9.86735 -15.7615 1.625 -2.90826 0.00829 9.27e-06 0.00435 0.00532 0.0151 0.0000132 0.0104 0.00983 0.0435
3L:19798140-19798290:plus -24.2245 13.5306 0.422018 3.77064 -28.4592 -18.6429 -22.0459 11.3056 0.174312 0.00763 0.0000206 0.0101 0.00637 0.0568 0.0139 0.0297 0.0000587 0.0177
3L:19799140-19799290:minus -20.8878 13.4592 -2.13761 4.3211 -18.4898 -26.898 -10.367 1.66667 0.816514 0.0018 0.000064 0.0225 0.00514 0.0116 0.0302 0.0033 0.00969 0.015
3L:19799140-19799290:plus -23.1531 13.4592 2.33028 5.31193 -17.4898 -8.65306 -6.07339 0.486111 -2.31193 0.00542 0.000064 0.00511 0.00393 0.0053 0.00156 0.00134 0.0144 0.0363
3L:19800380-19800530:minus -30.5102 14.9908 11.7523 4.13761 -8.93878 -19.3061 -7.33945 1.125 3.46789 0.00947 2.1e-06 0.0000507 0.00546 0.00216 0.0187 0.00182 0.0117 0.00582
3L:19800380-19800530:plus -31.5918 -5.90816 -0.944954 -1.07339 -18.0816 -19.2755 -23.8165 3.68056 3.51376 0.0136 0.0134 0.0157 0.0377 0.00759 0.0185 0.0399 0.00457 0.00566
3L:19810780-19810930:minus -24.2245 9.74312 5.55963 3.41284 10.6939 -9.53061 -14.2202 4.125 -1.16514 0.00763 0.000152 0.0014 0.00756 7.35e-06 0.0031 0.00761 0.00382 0.0274
3L:19810780-19810930:plus -32.5408 8.46789 -0.119266 3.41284 -17.4898 -18.1531 -18.2844 4.61111 0.926606 0.0204 0.000296 0.0121 0.00756 0.0053 0.00997 0.0156 0.00311 0.0146
3L:19811160-19811310:minus -24.449 4.83486 -3.6055 -0.385321 -18.9388 -10.0204 -23.9633 4.16667 -0.385321 0.00802 0.00174 0.0341 0.03 0.0151 0.00399 0.0408 0.00375 0.0211
3L:19811160-19811310:plus -13.2245 2.04587 5.42202 2.44037 -18.7143 -0.316327 -21.1927 -3.27778 7.73394 0.000738 0.00494 0.00149 0.0116 0.0136 0.000524 0.0255 0.0414 0.000784
3L:19827480-19827630:minus -22.0714 13.4592 5.90826 2.72477 -8.60204 -18.2041 -12.6422 -2.97222 8.14679 0.00299 0.000064 0.0012 0.0102 0.00125 0.0103 0.00533 0.0384 0.000562
3L:19827480-19827630:plus -22.5204 13.4592 3.30275 3.77064 -26.9694 -0.173469 -26.2844 6.01389 -1.93578 0.00356 0.000064 0.00353 0.00637 0.023 0.000456 0.0554 0.00165 0.0326
3L:19838340-19838490:minus -12.9184 4.30275 12.8532 7.45872 -9.27551 -27.6327 -4.77064 -0.861111 -0.541284 0.000532 0.00214 0.0000235 0.000894 0.00284 0.043 0.00093 0.0217 0.0223
3L:19838340-19838490:plus -22.7041 5.29358 -1.53211 1.78899 -19.3163 -8.20408 -20.0183 1.18056 -3.81651 0.00395 0.00144 0.0188 0.0158 0.0165 0.000796 0.0207 0.0114 0.0529
3L:19854440-19854590:minus -23.7653 -4.77982 5.26606 3.31193 -17.4898 -18.2755 -13.3853 0.138889 -1 0.00675 0.0416 0.00159 0.00786 0.0053 0.0112 0.00631 0.016 0.0261
3L:19854440-19854590:plus -22.7041 -2.3211 -1.91743 4.66972 -18.1531 -18.2755 -9.75229 1.625 1.40367 0.00395 0.0213 0.0211 0.00466 0.00829 0.0112 0.00294 0.00983 0.0122
3L:19864100-19864250:minus -38.6939 8.26606 -1.09174 5.69725 -26.9694 -17.5 -19.2294 -0.680556 4.92661 0.0321 0.00033 0.0164 0.00314 0.023 0.00599 0.0181 0.0206 0.00318
3L:19864100-19864250:plus -21.8878 14.6972 15.6972 5.22018 -27.0816 -19.051 -8.92661 2.20833 5.3945 0.00258 3.63e-06 3.11e-07 0.00418 0.0243 0.0165 0.00252 0.008 0.00263
3L:19866180-19866330:minus -23.2551 4.66055 0.440367 2.66055 -27.602 -8.79592 -9.98165 5.13889 1.38532 0.00583 0.00187 0.01 0.0106 0.0364 0.0019 0.00307 0.00247 0.0123
3L:19866180-19866330:plus -32.6633 -5.18349 -1.22936 0.752294 -18.4184 -9.7551 -15.0367 1.48611 -2.87156 0.0216 0.0463 0.0171 0.0221 0.0101 0.00358 0.00904 0.0103 0.043
3L:19888760-19888910:minus -32.5102 0.834862 5.16514 1.92661 -17.1837 -35.8571 -12.7156 4.40278 10.3394 0.0202 0.00769 0.00166 0.0148 0.00366 0.0818 0.00542 0.0034 0.000112
3L:19888760-19888910:plus -33.9694 2.21101 7.98165 3.27523 -18.3776 0.530612 -24.156 3.11111 -5.31193 0.0305 0.00464 0.000442 0.00796 0.00926 0.000203 0.042 0.00572 0.0776
3L:19889560-19889710:minus -4.81633 7.68807 3.83486 5.22936 -26.7041 -17.9388 -10.6055 -0.722222 9.92661 0.000197 0.000452 0.00287 0.0041 0.0193 0.00738 0.00345 0.0208 0.000193
3L:19889560-19889710:plus -23.4184 1.93578 2.37615 3.94495 -19.051 -7.5 -25.578 -0.555556 1.2844 0.00624 0.00514 0.00503 0.00595 0.0162 0.000614 0.0508 0.0198 0.0129
3L:19890200-19890350:minus -14.3673 3.66972 -1.42202 0.779817 -17.2653 -9.27551 -0.816514 8.80556 11.3853 0.00132 0.00272 0.0182 0.0219 0.00387 0.00244 0.000341 0.000362 0.0000815
3L:19890200-19890350:plus -14.1122 13.5306 14.9633 7.29358 -17.1122 -9.34694 -21.0917 -3.16667 -4.04587 0.00119 0.0000206 1.77e-06 0.00104 0.00341 0.00261 0.025 0.0403 0.0563

3L:19890640-19890790:minus -14.8163 8.33945 -0.862385 4.20183 -18.8265 -28.1837 -11.2477 1.38889
7.48624 0.00159 0.000317 0.0153 0.00533 0.0149 0.0584 0.00393 0.0107 0.00101
3L:19890640-19890790:plus -31.7041 -1.44037 -2 1.58716 -17.6429 10.3776 -14.7431 -1.69444 -4.37615
0.0146 0.0164 0.0216 0.017 0.00562 6.32e-06 0.00851 0.0274 0.0617
3L:19892520-19892670:minus -21.9286 3.64286 1.25688 0.93578 -17.6429 -8.65306 -13.2752 -5.375
9.87156 0.0027 0.00159 0.00755 0.0209 0.00562 0.00156 0.00616 0.0669 0.000217
3L:19892520-19892670:plus -22.8163 -2.00917 -0.816514 0.137615 -9.20408 -28.7857 -17.2018 -1.83333
6.63303 0.00418 0.0195 0.0151 0.0259 0.00262 0.0679 0.0132 0.0285 0.00149
3L:19893340-19893490:minus -12.3776 0.0733945 -2.68807 3.45872 -27.1224 0.826531 -16.5413 2.73611
-6.52294 0.000403 0.0101 0.0264 0.00731 0.0245 0.000153 0.0119 0.00659 0.101
3L:19893340-19893490:plus -21.7041 5.16514 7.53211 7.00917 -18.7959 -8.64286 -9.3211 -0.958333
1.08257 0.00231 0.00152 0.000558 0.00126 0.0148 0.00154 0.00271 0.0223 0.0139
3L:19893600-19893750:minus -33.1429 2.75229 1.7156 1.44037 -28.1633 -8.34694 -22.3211 12.8333
-0.201835 0.0272 0.00381 0.00642 0.018 0.0462 0.000993 0.0312 0.0000126 0.0199
3L:19893600-19893750:plus -4.66327 0.926606 5.2844 3.19266 -17.7449 -9.30612 -17.2936 6.70833 0.798165
0.000177 0.00743 0.00158 0.00829 0.0063 0.00257 0.0134 0.00117 0.0151
3L:19896200-19896350:minus -22.7653 2.6055 -0.889908 6.33945 -17.3776 -28.3367 -0.963303
2.61111 1.0367 0.00405 0.00402 0.0154 0.00196 0.00419 0.0606 0.000352 0.00691 0.0141
3L:19896200-19896350:plus -41.4388 1.10092 4.33945 2.34862 -27.3061 -9.27551 -18.9083 7.40278 3.6789
0.0562 0.00698 0.00234 0.0121 0.0315 0.00244 0.0172 0.000813 0.00506
3L:19896580-19896730:minus -31.4082 0.412844 5.56881 5.85321 -8.93878 1.12245 -13.945 -1.73611
-8.04587 0.0119 0.00893 0.0014 0.00287 0.00216 0.000085 0.00717 0.0277 0.137
3L:19896580-19896730:plus -23.0306 1.12844 4.95413 2.69725 -19.0102 -9.72449 -3.36697 2.41667 9.77064
0.00505 0.00691 0.00181 0.0104 0.0157 0.00352 0.000626 0.00742 0.000228
3L:19899260-19899410:minus -31.6327 -1.36697 0.100917 1.77982 -18.3776 -17.2041 -16.0826 -0.736111
-0.944954 0.0138 0.0161 0.0112 0.0158 0.00926 0.00507 0.011 0.0209 0.0257
3L:19899260-19899410:plus -32.3265 2.69725 3.95413 3.19266 -27.2653 0.316327 -2.85321 2.47222 1.44037
0.0187 0.00389 0.00273 0.00829 0.0302 0.000243 0.000547 0.00727 0.012
3L:19900180-19900330:minus -31.7041 2.04587 10.0917 5.43119 -18.3469 -9.23469 -29.6055 1.05556
10.0367 0.0146 0.00494 0.000135 0.00371 0.00894 0.00238 0.086 0.0119 0.000146
3L:19900180-19900330:plus -13.2551 4.73394 4.40367 2.83486 -26.5306 -37.1837 1.55046 3.69444 9.92661
0.000751 0.00181 0.00228 0.0097 0.0185 0.14 0.000189 0.00455 0.000193
3L:19901540-19901690:minus -23.2653 -2.41284 -0.733945 1.79817 -8.60204 -7.57143 -9.91743 5.05556
3.05505 0.00593 0.0219 0.0147 0.0157 0.00125 0.000644 0.00303 0.00256 0.00692
3L:19901540-19901690:plus -31.5918 -1.24771 10.578 -0.633028 -27.3776 -26.6735 -14.5872 3.76389
10.3486 0.0136 0.0155 0.000101 0.0327 0.032 0.0256 0.00824 0.00442 0.0000988
3L:19902000-19902150:minus -22.2857 -3.55046 -4.3945 3.29358 -17.8163 -25.4898 -8.49541 0.625
-2.33945 0.00331 0.03 0.0422 0.00791 0.00682 0.0205 0.00232 0.0137 0.0367
3L:19902000-19902150:plus -32.8469 1.34862 1.95413 5.66055 -27.5714 -18.8571 -19.055 3.36111 9.14679
0.0243 0.00637 0.00588 0.00317 0.0357 0.0151 0.0176 0.00519 0.000414
3L:19902520-19902670:minus -41.4796 -0.376147 4.02752 4.11927 -18.4898 -8.38776 -23.8624 -5.11111
-2.26606 0.0577 0.0117 0.00265 0.00551 0.0116 0.00101 0.0402 0.0632 0.0359
3L:19902520-19902670:plus -41.2857 -1.57798 4.92661 0.366972 0.806122 -8.7551 -21.6697 0.75 -4.0367
0.0521 0.0171 0.00183 0.0244 0.000229 0.00172 0.0278 0.0132 0.0562
3L:19903040-19903190:minus -22.8571 1.17431 -0.229358 11.2936 -27.3061 -18.1224 -7.11927
2.26389 8.11009 0.00427 0.00679 0.0125 0.0000139 0.0315 0.00988 0.00174 0.00784 0.000597
3L:19903040-19903190:plus -23.3673 0.642202 -6.12844 -1.91743 20.4694 -7.65306 5.44037
8.23611 7.88991 0.00617 0.00823 0.0653 0.0499 1.65e-07 0.000647 0.0000496 0.00051 0.000688
3L:19906740-19906890:minus -41.7755 9.44037 2.33945 9.49541 -27.1939 -7.45918 -19.8807
7.125 -2.94495 0.0652 0.000178 0.0051 0.000308 0.0267 0.000582 0.0202 0.000942 0.0441
3L:19906740-19906890:plus -41.4796 5.19266 -0.807339 0.752294 -27.5306 -8.79592
-23.5872 3.76389 -9.06422 0.0577 0.0015 0.015 0.0221 0.0351 0.0019 0.0386 0.00442 0.168
3L:19908000-19908150:minus -32.449 13.5306 4.69725 2.50459 -17.6837 -17.5 -23.1835 2.41667
0.743119 0.0195 0.0000206 0.00202 0.0114 0.0058 0.00599 0.0362 0.00742 0.0153

3L:19908000-19908150:plus	-4.85714	-0.238532	7.01835 2.78899 20.4694 -25.8265	-12.8716
2.66667 5.23853 0.000202	0.0112	0.000721	0.00992 1.65e-07	0.0212	0.00561 0.00677 0.00285
3L:19916940-19917090:minus	-31.8163	0.495413	9.49541 3.49541 -18.9796	-9.5	-17.7798
-0.125	3.56881 0.016	0.00868 0.000191	0.00716 0.0155	0.00294 0.0145	0.0174	0.00543
3L:19916940-19917090:plus	-24.102 -0.155963	8.77982 3.42202 -18.1224	-35.1122	-5.22936
-2.15278	3.40367 0.00733 0.0109	0.000286	0.00751 0.00782 0.0748	0.00106 0.0311	0.00603
3L:19917880-19918030:minus	-33.2959	1.74312 0.724771	5.06422 -27.1939	-9.60204	-15.4128
6.47222 -4.24771	0.0279	0.00552 0.00909 0.00433 0.0267	0.00334 0.00973 0.00132 0.0591
3L:19917880-19918030:plus	-24.5612	-1.84404	4.93578 -0.211009	0.846939	-18.898 -16.3945
1.27778 2.33028 0.00819 0.0186	0.00183 0.0286	0.000197	0.0152	0.0116	0.0111	0.00836
3L:19921700-19921850:minus	-21.5918	0.798165	13.9174 1.83486 -17.6837	-9.42857	-25.8899
-2.375	-4.24771	0.00209 0.00779 8.08e-06	0.0155	0.0058	0.0027	0.0528	0.0329	0.0591
3L:19921700-19921850:plus	-22.2551	-3.41284	-0.247706	2.24771 1.91837 0.0510204	-9.6789
5.41667 -2.48624	0.00327 0.0289	0.0126	0.0127	0.0000382	0.000349	0.0029	0.00218 0.0387
3L:19922180-19922330:minus	-40.2857	1.93578 3.02752 3.16514 0.918367	-8.61224	-11.2294
2.66667 -2.50459	0.0385	0.00514 0.00393 0.00843 0.000142	0.00152 0.00391 0.00677 0.0388
3L:19922180-19922330:plus	-31.1735	0.431193	6.11927 9.7156	0.836735	-9.34694	-14.2477
-2.84722	-8.62385	0.0109	0.00887 0.00109 0.000242	0.000215	0.00261 0.00766 0.0372	0.155
3L:199300-199450:minus	-30.0306	0.366972	0.321101	5.38532 -26.9286	0.867347	-9	-0.75
8.10092 0.00856 0.00908 0.0104	0.00382 0.0214	0.000144	0.00256 0.021	0.000623
3L:199300-199450:plus	-40.1735	-0.0366972	5.54128 4.61468 -17.9388	-8.72449	-12.1101
0.569444	-1.36697	0.0363	0.0104	0.00141 0.00475 0.00715 0.00169 0.00472 0.014	0.0286
3L:19932240-19932390:minus	-22.1122	3.12844 -0.963303	7.43119 -27.0408	0.387755	-10.4954
1.11111 11.8532 0.00304 0.00332 0.0158	0.000939	0.0242	0.000217	0.00338 0.0117	0.0000428
3L:19932240-19932390:plus	5.14286 3.88073 -0.954128	9.92661 -17.3776	-16.7551	-18.9083
-3.13889	5.65138 0.0000147	0.00252 0.0157	0.000169	0.00419 0.0042	0.0172	0.04	0.00228
3L:19985180-19985330:minus	-31.9286	-0.954128	1.40367 2.78899 -26.9286	-9.53061	-18.633
4.98611 -2.43119	0.0166	0.0141	0.00717 0.00992 0.0214	0.0031	0.0165	0.00264 0.038
3L:19985180-19985330:plus	-41.5612	3.05505 1.94495 -0.917431	-27.2653	-17.1224	-26.789
3.26389 4.25688 0.0609	0.00341 0.0059	0.0358	0.0302	0.00499 0.0589	0.00539 0.00391
3L:19994100-19994250:minus	-41.551 -0.862385	0.00917431	2.93578 -18.4898	-0.244898	-32.5046
2.47222 0.275229	0.0607	0.0137	0.0116	0.00927 0.0116	0.000496	0.125	0.00727 0.0171
3L:19994100-19994250:plus	-31.7041	13.5306 6.02752 2.50459 -26.9286	-9.72449	-10.5872
2.625	-2.47706	0.0146	0.0000206	0.00114 0.0114	0.0214	0.00352 0.00344 0.00687 0.0385
3L:19995940-19996090:minus	-24	-1.41284	6.02752 2.83486 -8.86735	-26.5612	-6.61468
-4.61111	9.66055 0.00719 0.0163	0.00114 0.0097	0.00173 0.0237	0.00154 0.0566	0.000268
3L:19995940-19996090:plus	-30.7347	4.42202 -0.46789	2.78899 -27.5306	-18.1224	-15.5046
0.263889	5.44954 0.01	0.00204 0.0135	0.00992 0.0351	0.00988 0.0099	0.0154	0.00253
3L:19997760-19997910:minus	-23.2959	-0.605505	0.275229	1.41284 -27.1531	-9.53061
-13.7339	5.36111 3.66972 0.00605 0.0126	0.0106	0.0183	0.0249	0.0031	0.00684 0.00223 0.00514
3L:19997760-19997910:plus	-32.8163	-0.605505	0.541284	1.92661 -18.449 -18.3469	-20.8624
7	1.22018 0.024	0.0126	0.00968 0.0148	0.0107	0.0124	0.024	0.00101 0.0133
3L:19999460-19999610:minus	-31.7041	6.08257 -2.68807	1.99083 -8.93878	-18.7857	-13.3761
5.68056 5.69725 0.0146	0.00102 0.0264	0.0144	0.00216 0.0146	0.0063	0.00193 0.00225
3L:19999460-19999610:plus	-14.2245	2.53211 5.74312 4.17431 -26.9694	-26.602 -12.0642	3.5
11.8532 0.00126 0.00413 0.00129 0.00537 0.023	0.0248	0.00468 0.00491 0.0000428
3L:19999940-20000090:minus	-31.7347	-3.55963	-0.779817	9.88073 -7.96939	-18.0102
-18.6881	1.38889 -2.2844 0.0149	0.03	0.0149	0.000183	0.000785	0.00812 0.0166	0.0107	0.0361
3L:19999940-20000090:plus	-32.5918	-0.743119	2.05505 1.88073 -17.7551	-17.5714	-13.5872
1.95833 -6.44037	0.021	0.0132	0.00566 0.0152	0.00658 0.00616 0.00661 0.00875 0.099
3L:20022040-20022190:minus	-33.0306	6.94495 3.36697 1.6789	-27.5306	-18.3469	-11.2569
6.05556 0.238532	0.026	0.000669	0.00345 0.0165	0.0351	0.0124	0.00393 0.00161 0.0173
3L:20022040-20022190:plus	-33.6735	13.5306 11.7064 2.66972 -27.4898	-18.5714	-26.5688
6.83333 -3.3211 0.0291	0.0000206	0.0000523	0.0106	0.0336	0.0136	0.0573	0.0011	0.0478

3L:20122160-20122310:minus -41.1735 3.17431 2.16514 4.02752 -17.7143 -0.27551 -29.5046 4.33333 -10.6697 0.0479 0.00326 0.00544 0.00581 0.00627 0.000502 0.0848 0.0035 0.219
3L:20122160-20122310:plus -20.6633 -1.99083 13.6239 7.45872 10.3878 -18.2755 -14.8991 5.38889 -2.50459 0.00173 0.0194 0.0000118 0.000894 0.0000174 0.0112 0.00879 0.0022 0.0388
3L:20133720-20133870:minus -32.8061 4.82569 10.9083 4.36697 -17.7143 -17.2755 -13.8716 4.875 -0.541284 0.0237 0.00175 0.0000846 0.00505 0.00627 0.00544 0.00705 0.00277 0.0223
3L:20133720-20133870:plus -21.9592 7.76147 -3.18349 4.9633 -8.33673 -0.540816 -4.14679 2.72222 9.61468 0.00279 0.000434 0.0304 0.0044 0.00101 0.00054 0.000778 0.00663 0.00029
3L:20134120-20134270:minus -22.0714 6.3945 4.6055 4.86239 -27.1224 -18.0816 -13.6055 2.34722 9.45872 0.00299 0.000878 0.0021 0.00449 0.0245 0.00934 0.00664 0.00761 0.000337
3L:20134120-20134270:plus -34.0306 -0.110092 -3.09174 1.22936 -27.3061 -9.94898 -23.1376 5.51389 -4.19266 0.0307 0.0107 0.0296 0.0193 0.0315 0.00393 0.0359 0.00208 0.0583
3L:20179180-20179330:minus -12.6735 3.97959 1.3945 9.33028 -18.1224 0.387755 -16.2202 4.01389 7.6422 0.000461 0.000669 0.00719 0.000374 0.00782 0.000217 0.0113 0.004 0.000893
3L:20179180-20179330:plus -40.2143 -1.37615 0.0183486 6.00917 -7.63265 -27.898 -15.5138 8.41667 -3.61468 0.0372 0.0161 0.0115 0.00255 0.000479 0.0537 0.00992 0.000458 0.0509
3L:20186680-20186830:minus -22.8163 -3.17431 7.91743 11.0183 -18.3776 -17.9388 -16.2018 2.63889 4.77982 0.00418 0.027 0.000457 0.000052 0.00926 0.00738 0.0112 0.00684 0.00339
3L:20186680-20186830:plus -31.4388 -2.83486 1.06422 7.34862 -17.2245 -9.5 -3.53211 -0.458333 2.41284 0.0122 0.0246 0.00808 0.000998 0.00384 0.00294 0.000655 0.0192 0.0082
3L:20186980-20187130:minus -23.2551 1.15596 -3.88073 3.05505 -18.4898 -27.898 -16.8257 11.5556 1.9633 0.00583 0.00684 0.0368 0.00878 0.0116 0.0537 0.0125 0.0000469 0.00951
3L:20186980-20187130:plus -40.1837 -2.26606 1.24771 2.18349 -27.2653 -19.3469 -16.9817 0.625 -1.06422 0.0367 0.021 0.00758 0.0131 0.0302 0.0194 0.0128 0.0137 0.0266
3L:20190500-20190650:minus -33.6939 1.16514 3.6055 6.02752 -8.89796 -18.3776 -17.0275 7.02778 7.22018 0.0291 0.00682 0.00314 0.00246 0.00189 0.0125 0.0129 0.000991 0.00119
3L:20190500-20190650:plus -22.6939 4.15596 4.31193 3.08257 -18.4898 1.12245 -13.0459 0.680556 -1.79817 0.00388 0.00227 0.00236 0.0087 0.0116 0.000085 0.00584 0.0135 0.0315
3L:20190900-20191050:minus -23.3673 0.844037 0.137615 9.59633 -27.9286 0.0918367 -8.23853 1.54167 -0.504587 0.00617 0.00766 0.0111 0.000275 0.0412 0.000323 0.00221 0.0101 0.0221
3L:20190900-20191050:plus -23.2551 -1.66972 14.4037 3.11009 -18.1531 -18.2041 -12.9725 7.15278 5.66055 0.00583 0.0176 4.33e-06 0.00862 0.00829 0.0103 0.00574 0.000928 0.00227
3L:20191420-20191570:minus -20.4388 1.77064 5.74312 10.8624 -25.9286 -9.42857 -12.4771 2.05556 2.09174 0.00167 0.00546 0.00129 0.0000687 0.0168 0.0027 0.00513 0.00845 0.00904
3L:20191420-20191570:plus -32.2143 1.52294 13.4679 7.29358 -18.7857 -16.051 -21.8807 1.25 3.22018 0.0182 0.00598 0.0000134 0.00104 0.0148 0.00408 0.0289 0.0112 0.0064
3L:20219400-20219550:minus -23.0714 13.8532 2.20183 3.59633 -17.7551 -8.27551 7.19266 3.76389 -0.59633 0.0052 9.88e-06 0.00536 0.00693 0.00658 0.000919 0.0000295 0.00442 0.0229
3L:20219400-20219550:plus -24.3061 -1.81651 5.09174 0.93578 -8.40816 -8.02041 -21.4404 5.375 2 0.00782 0.0184 0.00171 0.0209 0.00108 0.000761 0.0266 0.00222 0.00941
3L:20234420-20234570:minus -23.5612 -1.00917 -1.10092 0.477064 -18.4898 -18.9388 -14.2294 8 3.46789 0.00653 0.0144 0.0165 0.0238 0.0116 0.0154 0.00763 0.000583 0.00582
3L:20234420-20234570:plus -22.1122 1.75229 -1.07339 6.21101 -18.4592 -8.65306 -4.72477 -4.45833 -4.57798 0.00304 0.0055 0.0163 0.00213 0.0109 0.00156 0.000918 0.0547 0.0651
3L:20234860-20235010:minus -31.551 1.13761 5.44954 7.10092 -27.3367 -17.2755 -18.211 3.76389 3.15596 0.0134 0.00689 0.00147 0.00121 0.0317 0.00544 0.0154 0.00442 0.00659
3L:20234860-20235010:plus -13.1429 2.0367 1.18349 7.00917 -9.16327 -18.3469 -17.0275 1.73611 -1.68807 0.00065 0.00495 0.00775 0.00126 0.00249 0.0124 0.0129 0.00946 0.0308
3L:20235560-20235710:minus -34.3367 -0.605505 -2.72477 0.724771 -27.5306 0.826531 -9.63303 1.19444 -2.77064 0.0318 0.0126 0.0267 0.0223 0.0351 0.000153 0.00288 0.0114 0.0419
3L:20235560-20235710:plus -22.2245 3.9633 6.20183 5.58716 -27.2347 -9.53061 -17.3119 3.54167 -0.697248 0.00325 0.00244 0.00106 0.00334 0.0283 0.0031 0.0135 0.00483 0.0239
3L:20289980-20290130:minus -31.7449 1.11927 4.76147 3.44037 -28.3367 -8.5 -15.0367 2.5 -0.229358

0.0152	0.00693	0.00196	0.00746	0.0557	0.00126	0.00904	0.0072	0.0202
3L:20289980-20290130:plus	-41.398	-1.6422	-1.48624	3.59633	-19.051	-0.27551	-30.9817	3.80556	2.26606
0.0554	0.0175	0.0185	0.00693	0.0162	0.000502	0.104	0.00435	0.00847
3L:20295360-20295510:minus	-22.1837	8.01835	7.15596	2.19266	-9.0102	-8.65306	-7.44954	0.111111
-2.10092	0.00315	0.000378	0.000673	0.0131	0.00236	0.00156	0.00187	0.0162	0.0342
3L:20295360-20295510:plus	-22.7755	13.4592	0.302752	2.55046	-18.4898	-9.72449	-6.84404	0.0555556
-1.9633	0.00412	0.000064	0.0105	0.0112	0.0116	0.00352	0.00163	0.0164	0.0329
3L:20303000-20303150:minus	-33.102	1.45872	1.0367	4.22018	-26.5306	-18.0102	-16.789	6.15278
3.81651	0.0268	0.00613	0.00816	0.00531	0.0185	0.00812	0.0124	0.00154	0.00482
3L:20303000-20303150:plus	-12.8163	3.2844	9.04587	7.55963	1.07143	-9.5	2.43119	4.40278	3.24771
0.000489	0.00313	0.000248	0.000807	0.000125	0.00294	0.000144	0.0034	0.00636
3L:20306560-20306710:minus	-31.3265	-5.57798	11.789	7.88991	-9.23469	-9.82653	-17.8257	2.875
-2.3211	0.0114	0.0513	0.0000494	0.00062	0.00277	0.00381	0.0146	0.00626	0.0365
3L:20306560-20306710:plus	-31.4388	5.14679	2.82569	4.0367	10.6224	0.173469	-18.3028	-1.59722	1.44037
0.0122	0.00153	0.00424	0.00578	0.0000113	0.000307	0.0157	0.0267	0.012
3L:20310180-20310330:minus	-22.551	-1.47706	3.29358	9.44954	-8.60204	-36.1122	-11.6881	-7.30556
8.10092	0.00359	0.0166	0.00354	0.000326	0.00125	0.0878	0.00431	0.098	0.000623
3L:20310180-20310330:plus	-20.898	2.99083	12.2752	9.41284	-8.33673	-9.5	-12.1651	-5.95833	0.229358
0.0018	0.00349	0.0000353	0.000354	0.00101	0.00294	0.00478	0.0755	0.0173
3L:20341020-20341170:minus	-30.6327	-2.70642	5.77064	10.5872	-17.7959	-9.5	-16.0275	5.36111	2.0367
0.00968	0.0238	0.00128	0.000115	0.00678	0.00294	0.0109	0.00223	0.00923
3L:20341020-20341170:plus	-12.8878	5.86239	-2.11927	1.47706	-27.2245	-8.79592	-11.2936	1	-0.12844
0.000523	0.00113	0.0224	0.0178	0.0272	0.0019	0.00396	0.0121	0.0193
3L:20341340-20341490:minus	-13.4082	13.4592	0.192661	1.21101	-9.23469	-17.0102	3.72477	0.75	-0.12844
0.000867	0.000064	0.0109	0.0194	0.00277	0.00453	0.0000912	0.0132	0.0193
3L:20341340-20341490:plus	-32.9592	14.6881	-1.89908	1.0367	-18.4184	0.0918367	-9.33945	-0.152778
-0.440367	0.0252	3.75e-06	0.021	0.0204	0.0101	0.000323	0.00272	0.0175	0.0216
3L:20342600-20342750:minus	-24.0816	-3.33945	1.42202	-0.880734	-8.97959	-27.602	-20.156	-2.88889
4.61468	0.00732	0.0283	0.00712	0.0353	0.0023	0.0416	0.0212	0.0376	0.00351
3L:20342600-20342750:plus	-22.8878	-1.43119	0.211009	3.52294	-7.56122	-18.9286	-16.0367	-2.13889	3.7156
0.00441	0.0164	0.0108	0.00706	0.000419	0.0153	0.0109	0.0309	0.00502
3L:20349880-20350030:minus	-22.7449	13.6606	4.24771	1.99083	-17.449	-9.79592	-25.4954	0.805556
3.25688	0.00404	0.0000125	0.00243	0.0144	0.00484	0.00377	0.0502	0.013	0.00632
3L:20349880-20350030:plus	-23.8163	-1.13761	-0.944954	2.78899	-7.67347	-17.051	-17.4128	-1.41667
1.46789	0.00682	0.015	0.0157	0.00992	0.00055	0.00479	0.0137	0.0254	0.0119
3L:20354680-20354830:minus	-31.7041	15.7248	12.6789	8.61468	-18.2245	-8.53061	-11.9908	0.458333
1.31193	0.0146	5.64e-07	0.0000266	0.000552	0.00877	0.00136	0.0046	0.0145	0.0127
3L:20354680-20354830:plus	-2.33673	14.7523	13.2294	10.5872	-27.1939	-8.45918	-18.3394	-1.27778	-2.68807
0.0000294	3.48e-06	0.0000162	0.000115	0.0267	0.00111	0.0157	0.0244	0.041
3L:20368940-20369090:minus	-28.9184	-4.06422	11.6881	5.21101	-18.1224	-0.0204082	-20.4587	-0.875
-0.495413	0.00832	0.0344	0.0000527	0.0042	0.00782	0.000411	0.0223	0.0218	0.022
3L:20368940-20369090:plus	-31.8878	-3.42202	-1.09174	3.31193	-26.3061	-17.5714	-21.4587	3.31944	1.95413
0.0164	0.0289	0.0164	0.00786	0.0181	0.00616	0.0267	0.00528	0.00964
3L:20369200-20369350:minus	-28.9184	0.642202	3.15596	5.21101	-17.4184	-17.8265	-11.5596	3.54167
-0.495413	0.00832	0.00823	0.00374	0.0042	0.00457	0.00699	0.00419	0.00483	0.022
3L:20369200-20369350:plus	-22.8469	-5.73394	-2.29358	6.16514	-8.60204	-26.5612	-0.357798	-0.638889
6.95413	0.00424	0.0534	0.0236	0.00223	0.00125	0.0237	0.000308	0.0203	0.00136
3L:203700-203850:minus	-30.4388	-5.14679	3.78899	-1.07339	-17.449	-18.9796	-9.3211	-1.08333	5.81651
0.00923	0.0459	0.00292	0.0377	0.00484	0.0155	0.00271	0.0231	0.00204
3L:203700-203850:plus	-24.1429	5.89908	5.27523	3.61468	-7.89796	-18.4184	-11.3211	-0.138889	9.92661
0.0074	0.00111	0.00158	0.0068	0.000678	0.0127	0.00399	0.0175	0.000193
3L:20377360-20377510:minus	-32.1531	0.477064	3.95413	6.74312	-26.6327	-10.0204	-26.6606	3.79167
-2.62385	0.0179	0.00873	0.00273	0.0015	0.0188	0.00399	0.058	0.00437	0.0402
3L:20377360-20377510:plus	-31.7041	13.5306	14.8624	7.29358	-18.7143	-19.4184	-20.5596	3.04167	-2.90826

0.0146 0.0000206 2.2e-06 0.00104 0.0136 0.0198 0.0227 0.00587 0.0435
3L:20378680-20378830:minus -12.0714 -1.45872 -0.522936 6.05505 0.612245 -27.7041 -16.4404 4.36111
-1.18349 0.000318 0.0165 0.0137 0.0024 0.000269 0.0445 0.0117 0.00346 0.0276
3L:20378680-20378830:plus -31.7041 6.58716 -3.24771 3.07339 -27.1939 -0.0204082 -13.7248 3.13889
-2.94495 0.0146 0.000799 0.0309 0.00873 0.0267 0.000411 0.00682 0.00566 0.0441
3L:20379120-20379270:minus -13.8878 -2.74312 0.972477 3.44954 -8.60204 -26.2653 -18.5413 5.625
1.73394 0.00111 0.024 0.00835 0.00738 0.00125 0.0218 0.0162 0.00198 0.0106
3L:20379120-20379270:plus -24 0.669725 5.02752 5.49541 -26.9694 -9.37755 -6.75229 -4.66667 1.22018
0.00719 0.00815 0.00176 0.00355 0.023 0.00265 0.00159 0.0573 0.0133
3L:20386780-20386930:minus -22.0816 -1.62385 5.78899 2.7156 -18.6735 -17.2041 -10.1468 -0.0277778
-0.724771 0.003 0.0174 0.00127 0.0102 0.0123 0.00507 0.00316 0.0169 0.0241
3L:20386780-20386930:plus -42.0612 1.43119 5.11927 0.302752 -26.2653 -9.02041 -20.7615 4.36111 -1.11009
0.0692 0.00619 0.00169 0.0248 0.018 0.00213 0.0236 0.00346 0.0269
3L:20388540-20388690:minus -30.7041 6.84404 1.69725 6.09174 -8.64286 -8.68367 -18.8716 1.625
-3.19266 0.00993 0.000705 0.00646 0.00235 0.00139 0.00157 0.0171 0.00983 0.0466
3L:20388540-20388690:plus -24.5204 -2.37615 5.13761 5.57798 -9.45918 -9.82653 -20.3578 -1.63889 -0.798165
0.00813 0.0216 0.00168 0.0034 0.00287 0.00381 0.0219 0.027 0.0246
3L:20389060-20389210:minus -24.3367 -3.77064 7.63303 2.75229 -26.9694 -27.602 -19.1743 -2.94444
2.53211 0.00789 0.0318 0.000531 0.0101 0.023 0.0416 0.0179 0.0381 0.00791
3L:20389060-20389210:plus -23.1531 -1.79817 -0.119266 2.27523 -18.1531 -17.2755 -16.6606 -4.77778
-5.3211 0.00542 0.0183 0.0121 0.0125 0.00829 0.00544 0.0121 0.0587 0.0777
3L:20392240-20392390:minus -32.8469 5.6055 1.77064 -0.688073 1.83673 -17.0102 -8.01835 4.91667
2.37615 0.0243 0.00126 0.00629 0.0333 0.0000532 0.00453 0.00211 0.00272 0.00825
3L:20392240-20392390:plus -22.8469 6.27523 5.26606 5.91743 -27.2347 -8.72449 -8.06422 1.01389 3.52294
0.00424 0.00093 0.00159 0.00267 0.0283 0.00169 0.00213 0.0121 0.00558
3L:20392680-20392830:minus -11.0408 -0.486239 1.33945 3.19266 -17.7143 -16.8265 -10.3578 3.29167
1.2844 0.000241 0.0121 0.00733 0.00829 0.00627 0.0043 0.00329 0.00533 0.0129
3L:20392680-20392830:plus -31.7755 -1.47706 1.44037 3.36697 -17.4898 -19.0816 -21.2936 -0.75 0.174312
0.0157 0.0166 0.00707 0.00764 0.0053 0.017 0.0259 0.021 0.0177
3L:20393320-20393470:minus -31.7449 5.46789 2.24771 7.17431 -26.9694 10.0918 -13.1835 3.58333
7.62385 0.0152 0.00134 0.00527 0.00115 0.023 9.34e-06 0.00603 0.00475 0.000941
3L:20393320-20393470:plus -41.2245 4.76147 1.11927 5.48624 -27.5 0.204082 -9.2844 4.27778 -2.52294
0.0497 0.00179 0.00793 0.00357 0.0337 0.000296 0.0027 0.00358 0.0391
3L:20394720-20394870:minus 5.32653 -0.53211 0.385321 2.45872 1.61224 -16.9796 -10.0092 -2.40278
1.74312 0.0000114 0.0123 0.0102 0.0116 0.0000631 0.0044 0.00308 0.0332 0.0105
3L:20394720-20394870:plus -32.4388 5.05505 2.10092 0.651376 -18.7143 1.09184 -9.59633 0.472222 5.70642
0.0195 0.00159 0.00556 0.0226 0.0136 0.000091 0.00286 0.0144 0.00222
3L:20396360-20396510:minus -3.7449 4.55963 -3.31193 7.70642 -18.051 -19.4184 -3.88073 2.79167
9.45872 0.000104 0.00194 0.0315 0.000708 0.00746 0.0198 0.000721 0.00646 0.000337
3L:20396360-20396510:plus -40.3571 3.04587 5.06422 1.43119 -8.96939 -25.9796 -11.0642 -3.58333 5.84404
0.0394 0.00342 0.00173 0.0181 0.00226 0.0214 0.00378 0.0446 0.00202
3L:20397060-20397210:minus -30.4796 -1.3578 -0.256881 3.97248 -17.449 -19.051 -16.1284 -0.472222
1.48624 0.00941 0.016 0.0126 0.00587 0.00484 0.0165 0.0111 0.0193 0.0118
3L:20397060-20397210:plus -22.4388 -1.69725 2.87156 5.43119 -8.70408 -17.3469 -18.9725 1.09722 1.55963
0.00348 0.0178 0.00417 0.00371 0.00155 0.0058 0.0174 0.0118 0.0114
3L:20423580-20423730:minus -31.6224 0.0733945 8.24771 2.66972 10.6531 -9.64286 -23.6697 -2.375
-0.33945 0.0137 0.0101 0.000384 0.0106 0.0000102 0.00339 0.0391 0.0329 0.0209
3L:20423580-20423730:plus -33.3673 6.77064 0.0275229 2.81651 -17.8265 -8.79592 -12.4495 6.98611
3.51376 0.0282 0.000731 0.0115 0.00982 0.00691 0.0019 0.0051 0.00101 0.00566
3L:20425580-20425730:minus -30.4796 4.81651 0.504587 1.55963 0.877551 -8.72449 -12.2936 3.77778
1.44037 0.00941 0.00175 0.0098 0.0173 0.00019 0.00169 0.00492 0.0044 0.012
3L:20425580-20425730:plus -31.7347 12.1376 -0.825688 7.99083 -8.33673 -18.4184 -14.3578 6.11111
5.12844 0.0149 0.0000424 0.0151 0.00059 0.00101 0.0127 0.00785 0.00157 0.00301
3L:20429220-20429370:minus -40.5204 -0.100917 5.34862 3.48624 -7.60204 9.82653 -15.0826 7.02778

-2.90826 0.0413 0.0107 0.00154 0.0072 0.000466 0.0000148 0.00912 0.000991 0.0435
3L:20429220-20429370:plus -40.1429 -0.807339 2.01835 5.77982 1.14286 -18.7857 -13.2752 -2.19444
1.48624 0.0363 0.0135 0.00574 0.00294 0.000113 0.0146 0.00616 0.0314 0.0118
3L:20445900-20446050:minus -30.2857 6.73394 -2.88991 5.57798 -18.1531 -27.602 -6.76147 1.38889
8.88073 0.00887 0.000744 0.028 0.0034 0.00829 0.0416 0.00159 0.0107 0.000468
3L:20445900-20446050:plus -33.7041 4.76147 -2.40367 5.69725 -18.7143 9.45918 -21.7064 4.73611 -4.75229
0.0293 0.00179 0.0243 0.00314 0.0136 0.0000356 0.028 0.00295 0.0683
3L:20446300-20446450:minus -13.6735 -0.458716 3.3945 1.84404 -18.7551 -17.2755 -20.3578 5.55556
-0.642202 0.00103 0.012 0.00341 0.0154 0.0144 0.00544 0.0219 0.00204 0.0234
3L:20446300-20446450:plus -31.9694 2.92661 0.486239 3.98165 -17.9286 -9.57143 -12.2569 -3.91667 1.25688
0.0168 0.00357 0.00986 0.00582 0.0071 0.00326 0.00488 0.0483 0.0131
3L:20465020-20465170:minus -32.0408 -0.40367 3.22018 6.17431 -18.4592 -9.79592 -21.8899 -1.22222
2.63303 0.0174 0.0118 0.00365 0.0022 0.0109 0.00377 0.0289 0.024 0.00771
3L:20465020-20465170:plus -21.5918 6.3211 4.44037 4.0367 -26.898 -0.244898 -17.4862 9.15278
1.15596 0.00209 0.000909 0.00224 0.00578 0.0203 0.000496 0.0138 0.000291 0.0135
3L:20466460-20466610:minus -22.6939 5.3945 7.62385 4.05505 20.2041 -27.051 -7.13761 -0.0416667
7.53211 0.00388 0.00138 0.000533 0.00566 3.64e-07 0.0315 0.00174 0.017 0.000968
3L:20466460-20466610:plus -22.7347 1.44037 12.1927 0.0275229 0.877551 -28.4082 -21.0092 0.555556
1.9633 0.00403 0.00617 0.0000376 0.0267 0.00019 0.0615 0.0246 0.014 0.00951
3L:20467940-20468090:minus -14.6327 -1.72477 5.44954 9.7156 0.540816 -17.3469 -17.945 10.6667
1.33945 0.0015 0.0179 0.00147 0.000242 0.000321 0.0058 0.0148 0.0000996 0.0125
3L:20467940-20468090:plus -14.7143 3.90816 13.6239 3.90826 -19.0204 -9.5 -14.156 6.61111 -2.43119
0.00154 0.00102 0.0000118 0.00603 0.0158 0.00294 0.00751 0.00123 0.038
3L:20488300-20488450:minus -32.9592 8.69725 -1.98165 3.59633 -7.93878 -9.7551 -29.4128 5.75 -3.81651
0.0252 0.000263 0.0215 0.00693 0.000767 0.00358 0.0837 0.00187 0.0529
3L:20488300-20488450:plus -32.0714 0.321101 3.19266 5.85321 20.4694 -0.316327 -12.6239 6.33333
-3.65138 0.0176 0.00922 0.00369 0.00287 1.65e-07 0.000524 0.0053 0.00141 0.0512
3L:20490340-20490490:minus -40.4388 3.58716 2.05505 3.41284 -26.4898 -8.94898 -22.7431 0.930556
-3.24771 0.0401 0.0028 0.00566 0.00756 0.0183 0.00203 0.0336 0.0124 0.0471
3L:20490340-20490490:plus -30.2143 -3.29358 4.98165 5.94495 -27.1939 -17.7143 -10.1101 4.31944 -2.83486
0.0088 0.028 0.00179 0.00261 0.0267 0.00634 0.00314 0.00352 0.0427
3L:20491000-20491150:minus -31.7755 7.06422 14.0734 2.50459 -7.93878 -9.42857 -19.1743 -8.25
-6.70642 0.0157 0.000628 6.63e-06 0.0114 0.000767 0.0027 0.0179 0.116 0.106
3L:20491000-20491150:plus -24.0714 -3.88073 5.92661 6.45872 -27.0816 -28.7041 -11.5505 -1.15278 7.6789
0.00731 0.0328 0.00119 0.00182 0.0243 0.067 0.00418 0.0236 0.000866
3L:20501200-20501350:minus -24.5204 -0.779817 0.862385 3.11009 -27 -25.7551 -10.1743 2.81944
-2.42202 0.00813 0.0134 0.00867 0.00862 0.0233 0.0208 0.00318 0.00639 0.0378
3L:20501200-20501350:plus -32.6327 -1.77064 0.422018 7.27523 -26.5714 -8.79592 -24.6055 3.05556 4.30275
0.0212 0.0182 0.0101 0.00109 0.0185 0.0019 0.0447 0.00584 0.00388
3L:20501640-20501790:minus -30.4388 2.50459 -3.14679 2.29358 -17.4184 0.0510204 -20.0183 2.98611
6.11927 0.00923 0.00417 0.03 0.0124 0.00457 0.000349 0.0207 0.006 0.00178
3L:20501640-20501790:plus -23 5.50459 6.37615 6.00917 0.846939 -17.2041 -9.09174 0.486111 1.78899
0.00498 0.00132 0.000974 0.00255 0.000197 0.00507 0.0026 0.0144 0.0103
3L:20502340-20502490:minus -31.2143 0.422018 -4.85321 4.31193 -27.5306 -17.6429 -12.211 9 5.79817
0.0111 0.0089 0.0475 0.00516 0.0351 0.00619 0.00483 0.00032 0.00208
3L:20502340-20502490:plus -13.5204 1.6422 8.3578 6.73394 -18.6837 -18.6429 -14.8624 -2.52778 7.68807
0.00097 0.00573 0.000361 0.00152 0.0126 0.0139 0.00873 0.0343 0.000841
3L:20502820-20502970:minus -31.7755 1.20183 5.18349 -0.807339 -27.1939 -8.57143 -12.5963 3.125
-4.76147 0.0157 0.00673 0.00165 0.0345 0.0267 0.00149 0.00527 0.00569 0.0686
3L:20502820-20502970:plus -3.33673 -0.211009 0.431193 6.84404 -17.7143 -26.7551 -10.7523 3.29167
3.49541 0.0000599 0.0111 0.01 0.0014 0.00627 0.0263 0.00355 0.00533 0.0057
3L:20509880-20510030:minus -13.2551 5.6055 -0.541284 2.16514 -9.2449 -16.051 2.6789 4.90278
-0.798165 0.000751 0.00126 0.0138 0.0132 0.00278 0.00408 0.000132 0.00274 0.0246
3L:20509880-20510030:plus -31.5102 3.78899 4.29358 1.07339 -8.93878 -9.30612 -14.367 12.4722 1.70642

0.013 0.0026 0.00238 0.0201 0.00216 0.00257 0.00786 0.0000191 0.0108
3L:20510680-20510830:minus -30.5612 -1.59633 -5.37615 -3.85321 -28.2347 -27.602 -9.66055 3.375
11.8532 0.00959 0.0172 0.0543 0.0817 0.051 0.0416 0.00289 0.00516 0.0000428
3L:20510680-20510830:plus -32.5816 -3.55963 2.3945 5.69725 -17.4184 -18.7551 -17.3486 2.77778 -0.504587
0.0208 0.03 0.00499 0.00314 0.00457 0.0141 0.0135 0.00649 0.0221
3L:20511600-20511750:minus -22.8571 -5.95413 7.80734 10.5872 -26.8571 -9.5 -22.7798 -3.625 -6.55046
0.00427 0.0565 0.000485 0.000115 0.0198 0.00294 0.0338 0.045 0.102
3L:20511600-20511750:plus -14.5612 -5.2844 4.22936 2.63303 -27.602 -17.1633 -12.3578 3.01389 9.45872
0.00147 0.0475 0.00244 0.0107 0.0364 0.005 0.00499 0.00593 0.000337
3L:20513820-20513970:minus -23.1122 -2.18349 9.18349 6.41284 1.61224 -8.53061 -17.8257 1.30556
-3.13761 0.0053 0.0205 0.000228 0.00189 0.0000631 0.00136 0.0146 0.011 0.0461
3L:20513820-20513970:plus -32.0408 -0.642202 -0.0275229 3.62385 20.1735 9.7551 -19.2294 6.69444
-3.3211 0.0174 0.0128 0.0117 0.00673 5.68e-07 0.0000198 0.0181 0.00118 0.0478
3L:20517700-20517850:minus -30.2857 2.98165 -5.6789 1.92661 -18.6429 -25.898 -7.91743 0.680556
3.62385 0.00887 0.0035 0.0585 0.0148 0.0121 0.0214 0.00207 0.0135 0.00523
3L:20517700-20517850:plus -22.551 0.504587 1.59633 -0.770642 -18.7857 -9.94898 -6.00917 4.91667
0.348624 0.00359 0.00865 0.00669 0.0342 0.0148 0.00393 0.00131 0.00272 0.0167
3L:20519360-20519510:minus -30.6633 6.56881 2.7156 3.54128 -8.7449 -9.16327 -16.5963 -1.36111
-4.98165 0.00976 0.000806 0.00442 0.00702 0.00161 0.00223 0.012 0.025 0.0725
3L:20519360-20519510:plus -22.0408 -1.51376 15.6239 3.07339 -17.6429 -9.30612 -9.02752 3.55556 0.495413
0.00296 0.0168 5.44e-07 0.00873 0.00562 0.00257 0.00257 0.00481 0.0162
3L:20528460-20528610:minus -31.6633 0.559633 -2.91743 7.45872 -8.63265 -8.79592 -20.0826 -3.88889
-7.45872 0.0141 0.00848 0.0282 0.000894 0.0013 0.0019 0.0209 0.048 0.123
3L:20528460-20528610:plus -31.7041 -1.66055 2.3945 5.43119 -17.1837 -18.051 -30.9358 4.20833 -2.46789
0.0146 0.0176 0.00499 0.00371 0.00366 0.00886 0.103 0.00369 0.0384
3L:20530640-20530790:minus -21.4388 1.66055 0.486239 1.57798 -18.9796 -18.0102 -11.1835 -0.402778
3.23853 0.002 0.00569 0.00986 0.0171 0.0155 0.00812 0.00387 0.0189 0.00639
3L:20530640-20530790:plus -23.0714 0.256881 0.559633 5.33028 -8.89796 -27.602 -17.5046 4.01389 2.44037
0.0052 0.00944 0.00961 0.00389 0.00189 0.0416 0.0139 0.004 0.00809
3L:20531080-20531230:minus -31.4388 3.64286 -0.917431 1.25688 -17.7143 -8.57143 -8.3211 0.708333
2.74312 0.0122 0.00159 0.0156 0.0192 0.00627 0.00149 0.00225 0.0134 0.00751
3L:20531080-20531230:plus -29.4388 4.50459 1.30275 5.74312 -18.4898 -17.051 -21.5046 -0.486111
-4.24771 0.00842 0.00198 0.00743 0.00302 0.0116 0.00479 0.027 0.0194 0.0591
3L:20531540-20531690:minus -21.8878 7.55046 -1.40367 -1.23853 -19.051 -18.3061 -18.8165 -0.75
-0.174312 0.00258 0.000485 0.0181 0.0397 0.0162 0.0114 0.017 0.021 0.0196
3L:20531540-20531690:plus -32.5816 -0.504587 3.88991 0.724771 -27.3061 -9.65306 -14.5046 0 4.02752
0.0208 0.0122 0.0028 0.0223 0.0315 0.0034 0.0081 0.0167 0.00428
3L:20533500-20533650:minus -12.449 4.97248 3 7.3578 -18.7857 -26.7857 -13.9633 3.59722 9.34862
0.000428 0.00165 0.00397 0.000965 0.0148 0.0266 0.0072 0.00473 0.000371
3L:20533500-20533650:plus -22.9184 0.357798 -5.80734 1.87156 -18.1531 -8.38776 -18.5963 -4.73611 1.25688
0.00447 0.00911 0.0604 0.0152 0.00829 0.00101 0.0164 0.0582 0.0131
3L:20533980-20534130:minus -24.2959 13.3878 -3.19266 2.6789 -7.71429 -19.3469 -7.49541 4.05556
-3.00917 0.00781 0.0000869 0.0304 0.0105 0.000572 0.0194 0.00189 0.00393 0.0447
3L:20533980-20534130:plus -23.7755 4.20183 3.90826 5.22936 -18.449 -9.5 -19.8349 2.76389 4.40367
0.00678 0.00223 0.00278 0.0041 0.0107 0.00294 0.02 0.00652 0.00373
3L:205420-205570:minus -30.6633 1.93578 1.11009 5.16514 -18.4184 9.0102 -12.211 1.70833 4.3945
0.00976 0.00514 0.00796 0.00424 0.0101 0.0000436 0.00483 0.00955 0.00375
3L:205420-205570:plus -22.8469 1.2844 -1.0367 7.62385 -26.898 -17.4898 -8.23853 6.76389 5.0367
0.00424 0.00653 0.0161 0.000759 0.0203 0.0059 0.00221 0.00114 0.00306
3L:205840-206110:minus -22.6531 3.97959 -2.56881 3.61468 -8.67347 -18.1531 -16.6422 -0.111111
-5.51376 0.00374 0.000669 0.0255 0.0068 0.00154 0.00997 0.0121 0.0173 0.0807
3L:205840-206110:plus -42.1122 -0.623853 14.0183 7.29358 -6.93878 -19.3469 -14.8257 0.625 0.798165
0.0698 0.0127 7.05e-06 0.00104 0.000373 0.0194 0.00866 0.0137 0.0151
3L:20723460-20723610:minus -23.1122 0.146789 5.58716 3.04587 -27 -8.57143 -12.6055 3.25 1.52294

0.0053 0.0098 0.00139 0.00883 0.0233 0.00149 0.00528 0.00542 0.0117
3L:20723460-20723610:plus -31.4388 -1.55963 5.13761 10.7615 -18.1939 -18.0816 -21.7706 3.375 3.36697
0.0122 0.017 0.00168 0.0000826 0.0086 0.00934 0.0283 0.00516 0.00607
3L:20724480-20724630:minus -33.0714 -3.52294 6.09174 11.0183 -17.8163 -9.42857 -20.789 1.52778
-0.0275229 0.0267 0.0297 0.00111 0.000052 0.00682 0.0027 0.0237 0.0102 0.0186
3L:20724480-20724630:plus -31.398 0.834862 2.11927 3.38532 -16.3776 0.826531 -23.6239 1.05556 0.0458716
0.0117 0.00769 0.00553 0.00761 0.003 0.000153 0.0388 0.0119 0.0182
3L:20727340-20727490:minus -23.0306 2.98165 11.578 7.55963 -26.0408 -19.0102 -11.2752 0.652778
5.22936 0.00505 0.0035 0.0000565 0.000807 0.0171 0.0159 0.00395 0.0136 0.00288
3L:20727340-20727490:plus -24.6327 4.66972 10.1376 5.16514 -26.9694 -19.3776 -6.42202 0.0277778
3.88991 0.00824 0.00186 0.000131 0.00424 0.023 0.0195 0.00146 0.0166 0.00467
3L:20762540-20762690:minus -33 6.91743 14.1651 7.29358 -17.7143 -9.57143 -26.8165 2.23611 -1.68807
0.0258 0.000678 6.03e-06 0.00104 0.00627 0.00326 0.0591 0.00792 0.0308
3L:20762540-20762690:plus -31.551 -0.688073 12.5963 6.48624 -18.449 -26.051 -17.844 -1.63889
1.38532 0.0134 0.013 0.0000282 0.00177 0.0107 0.0216 0.0146 0.027 0.0123
3L:20764560-20764710:minus -21.7347 -3.48624 12.2752 7.29358 -27.7959 -18.8571 -16.7615 -4.41667
-1.52294 0.00235 0.0294 0.0000353 0.00104 0.0386 0.0151 0.0123 0.0542 0.0295
3L:20764560-20764710:plus -32.4694 -2.93578 -1.02752 3.6422 -18.3776 -27.3367 -10.2936 -0.708333
8.11009 0.0196 0.0253 0.0161 0.00669 0.00926 0.0347 0.00325 0.0207 0.000597
3L:20764980-20765130:minus -32.4694 0.284404 1.0367 3.84404 -27.1531 -16.2755 -12.8532 7.31944
-1.55046 0.0196 0.00935 0.00816 0.00624 0.0249 0.00413 0.00559 0.00085 0.0297
3L:20764980-20765130:plus -24.2959 -1.16514 3.10092 1.29358 -18.1224 -9.23469 -21.6055 7.375 5.38532
0.00781 0.0151 0.00382 0.019 0.00782 0.00238 0.0275 0.000825 0.00267
3L:20771320-20771470:minus -23 1.12844 4.04587 -0.0183486 -18.7143 -26.7857 -17.9174 4.22222
3.20183 0.00498 0.00691 0.00263 0.027 0.0136 0.0266 0.0148 0.00367 0.0065
3L:20771320-20771470:plus -23.6224 3.97248 1.11927 1.84404 -17.6735 -18.5 -12.0734 2.45833 3.46789
0.00658 0.00243 0.00793 0.0154 0.00572 0.0131 0.00469 0.00731 0.00582
3L:20771840-20771990:minus -24.7449 -3.36697 -1.56881 4.22936 -18.9796 -18.1531 -14.0734 8.80556
3.6055 0.00827 0.0285 0.019 0.00525 0.0155 0.00997 0.00737 0.000362 0.00532
3L:20771840-20771990:plus -23.5612 5.06422 0.752294 2.19266 11.4286 -8.65306 -7.90826 -0.916667
7.63303 0.00653 0.00159 0.009 0.0131 1.97e-06 0.00156 0.00206 0.022 0.000928
3L:20777760-20777910:minus -32.2857 -2.34862 1.55046 2.11927 -27.602 -18.1224 -5.13761 -1 4.30275
0.0185 0.0215 0.0068 0.0135 0.0364 0.00988 0.00103 0.0226 0.00388
3L:20777760-20777910:plus -32.8163 4.01835 5.3945 5.92661 -27.3061 -18.898 -20.4495 -4.02778 10.3486
0.024 0.00239 0.00151 0.00266 0.0315 0.0152 0.0223 0.0496 0.0000988
3L:20778500-20778650:minus -32 0.926606 2.97248 3.50459 -27.5306 -8.38776 -3.06422 1.75 1.99083
0.0171 0.00743 0.00401 0.00713 0.0351 0.00101 0.000578 0.00941 0.00946
3L:20778500-20778650:plus -31.1429 3.20183 3.97248 -0.0642202 -18.9388 -26.5306 -18.4312 7.79167
-3.83486 0.0108 0.00323 0.00271 0.0274 0.0151 0.023 0.016 0.000656 0.0533
3L:20778740-20778890:minus -13.449 -1.75229 4.85321 7.47706 -18.4898 -9.45918 -8.3945 9.47222
1.53211 0.000914 0.0181 0.00189 0.00085 0.0116 0.00279 0.00228 0.000236 0.0116
3L:20778740-20778890:plus -31.1429 3.20183 3.97248 -0.0642202 -18.9388 -26.5306 -18.4312 7.79167
-3.83486 0.0108 0.00323 0.00271 0.0274 0.0151 0.023 0.016 0.000656 0.0533
3L:20779220-20779370:minus -31.4082 13.3878 -0.0366972 4.04587 -18.4184 -36.0408 -9.49541 -2.76389
8.05505 0.0119 0.0000869 0.0117 0.00572 0.0101 0.0837 0.0028 0.0364 0.00064
3L:20779220-20779370:plus -31.4082 7.69725 3.26606 7 -27.1939 -8.72449 -18.422 3.66667 -2.3578
0.0119 0.00045 0.00358 0.00132 0.0267 0.00169 0.0159 0.0046 0.0369
3L:20781100-20781250:minus -32.0714 9.62385 3.73394 1.91743 -7.96939 -25.898 -10.633 0.638889
3.46789 0.0176 0.000161 0.00299 0.015 0.000785 0.0214 0.00347 0.0137 0.00582
3L:20781100-20781250:plus -23.0714 9.38532 3.22936 4.48624 -8.96939 -9.65306 -14.5413 -4.94444 -0.192661
0.0052 0.000183 0.00363 0.00487 0.00226 0.0034 0.00816 0.0609 0.0198
3L:20791620-20791770:minus -43.8571 -3.06422 -3.58716 -0.623853 -27.0816 -17.5 -19.4771 3.75
0.798165 0.094 0.0262 0.0339 0.0326 0.0243 0.00599 0.0189 0.00445 0.0151
3L:20791620-20791770:plus -32.7041 -1.69725 3 3.80734 11.6939 -9.23469 -8.38532 0.0416667 1.70642

0.0222 0.0178 0.00397 0.00627 1.17e-06 0.00238 0.00227 0.0165 0.0108
3L:20802400-20802550:minus -22.0306 -2.82569 -0.807339 3.6055 1.17347 -9.27551 -14.422 3.72222
3.45872 0.00293 0.0246 0.015 0.00686 0.000101 0.00244 0.00796 0.0045 0.00591
3L:20802400-20802550:plus -23.1837 15.3303 0.0550459 1.78899 -26.898 -9.02041 -4.97248 6 -2.98165
0.00553 1.39e-06 0.0114 0.0158 0.0203 0.00213 0.000985 0.00166 0.0445
3L:20802740-20802890:minus -22.3367 15.4037 1.82569 1.78899 -18.5306 -18.051 -12.5229 -1.56944
-0.275229 0.00339 1.16e-06 0.00616 0.0158 0.0118 0.00886 0.00518 0.0265 0.0206
3L:20802740-20802890:plus -12.8878 1.08257 2.89908 2.26606 2.17347 -18.8571 -16.211 2.48611 1.69725
0.000523 0.00703 0.00413 0.0126 0.0000296 0.0151 0.0113 0.00723 0.0108
3L:20809200-20809350:minus -24.2551 13.7798 3.9633 4.0367 -18.8265 -17.9796 -17.1468 5.88889
2.85321 0.00768 0.0000109 0.00272 0.00578 0.0149 0.00764 0.0131 0.00175 0.00733
3L:20809200-20809350:plus -23.9592 15.3119 0.440367 6.86239 -26.9286 -27.898 -11.5413 2.83333 -3.48624
0.00708 1.47e-06 0.01 0.00136 0.0214 0.0537 0.00418 0.00635 0.0494
3L:20834680-20834830:minus -31.5102 13.3878 4.25688 6.56881 -27.7041 -9.72449 -12.2294 1.25 1.85321
0.013 0.0000869 0.00242 0.0017 0.038 0.00352 0.00485 0.0112 0.01
3L:20834680-20834830:plus -41.4796 3.66972 7.16514 7.45872 -8.70408 -18.898 -26.211 1.91667 0.0275229
0.0577 0.00272 0.000671 0.000894 0.00155 0.0152 0.0549 0.00888 0.0183
3L:20942640-20942790:minus -23.1837 3.11009 8.76147 10.5963 -17.3776 -17.0102 -13.8807 -4.09722
8.11009 0.00553 0.00334 0.000288 0.0001 0.00419 0.00453 0.00706 0.0504 0.000597
3L:20942640-20942790:plus -21.8878 2.88991 2.24771 4.46789 -17.0408 -17.8265 -20.0826 -4.86111 0.963303
0.00258 0.00362 0.00527 0.0049 0.00329 0.00699 0.0209 0.0598 0.0145
3L:20943140-20943290:minus -31.8469 -1.29358 1.89908 1.08257 -8.63265 -19.5714 -12.9725 7 6.17431
0.0162 0.0157 0.006 0.0201 0.0013 0.0202 0.00574 0.00101 0.00171
3L:20943140-20943290:plus -23.4082 0.715596 0.165138 5.43119 -19.2755 -9.53061 -8.97248 3.30556 3.95413
0.00623 0.00802 0.011 0.00371 0.0164 0.0031 0.00254 0.0053 0.00448
3L:21002060-21002210:minus -13 3.3578 -2.38532 7.6422 -17.7143 -28.1224 -13.8165 0.708333 9.55046
0.000563 0.00305 0.0242 0.000721 0.00627 0.0583 0.00696 0.0134 0.000321
3L:21002060-21002210:plus -13.602 -2.33028 2.94495 2.66972 -17.3776 -17.9388 -12.5596 -0.166667
11.8532 0.000995 0.0214 0.00405 0.0106 0.00419 0.00738 0.00523 0.0176 0.0000428
3L:21002660-21002810:minus -22.2245 -4.6055 4.22018 4.9633 -18.9796 -0.173469 -24.5413 -0.125
-6.49541 0.00325 0.0398 0.00245 0.0044 0.0155 0.000456 0.0443 0.0174 0.101
3L:21002660-21002810:plus -23 7.84404 14.6514 -0.642202 -26.5714 -8.57143 -17.9266 1.51389 -2
0.00498 0.000415 3.2e-06 0.0328 0.0185 0.00149 0.0148 0.0102 0.0332
3L:21006680-21006830:minus -34.0714 -1.50459 7.22936 2.23853 -8.93878 -9.57143 -21.6514 -1.20833
-0.550459 0.031 0.0168 0.000649 0.0128 0.00216 0.00326 0.0277 0.0239 0.0225
3L:21006680-21006830:plus -22.1224 -0.972477 -0.119266 6.00917 -27.1224 -0.173469 -29.844
-1.66667 -4.45872 0.00304 0.0142 0.0121 0.00255 0.0245 0.000456 0.0889 0.0272 0.0633
3L:21007780-21007930:minus -23.449 -1.12844 -2.87156 3.05505 -18.7959 -18.7143 -10.4312 -3.38889
7.18349 0.00631 0.0149 0.0278 0.00878 0.0148 0.014 0.00334 0.0426 0.00121
3L:21007780-21007930:plus -22.8571 -1.66055 -3.48624 5.86239 -8.56122 -8.42857 -21.0275 -5.09722 10.3394
0.00427 0.0176 0.033 0.00282 0.00112 0.00104 0.0247 0.063 0.000112
3L:21018240-21018390:minus -32.551 1.44954 4.0367 1.41284 -19.051 -27.7449 -10.9908 7.43056
7.22018 0.0207 0.00615 0.00264 0.0183 0.0162 0.0448 0.00373 0.000801 0.00119
3L:21018240-21018390:plus -23.0408 10.3945 -0.522936 2.3578 -7.40816 0.0918367 -15.3853 -0.375
10.3394 0.00512 0.000109 0.0137 0.0121 0.000401 0.000323 0.00968 0.0188 0.000112
3L:21018800-21018950:minus -22.8469 -2.09174 2.09174 2.43119 -17.9796 -17.5714 -12.0275 1.125
5.53211 0.00424 0.0199 0.00558 0.0117 0.00726 0.00616 0.00464 0.0117 0.00245
3L:21018800-21018950:plus -23.0408 4.17431 0.385321 4.3211 -19.051 -9.23469 -12.5596 2.29167 0.192661
0.00512 0.00225 0.0102 0.00514 0.0162 0.00238 0.00523 0.00776 0.0175
3L:21019420-21019570:minus -12 2.11927 -0.981651 7.80734 -17.3776 -8.57143 -15.2385 10.3056
2.84404 0.000305 0.00481 0.0159 0.00063 0.00419 0.00149 0.00941 0.000131 0.00736
3L:21019420-21019570:plus -14.1122 6.95413 -3.06422 1.99083 -18.2245 -17.9388 -6.48624 2.47222 11.8532
0.00119 0.000665 0.0294 0.0144 0.00877 0.00738 0.00149 0.00727 0.0000428
3L:21020000-21020150:minus -32.0408 13.5688 10.7431 1.91743 -27.1939 0.27551 -27.3394 -0.0833333

7.63303	0.0174	0.0000135	0.0000926	0.015	0.0267	0.000257	0.0631	0.0172	0.000928
3L:21020000-21020150:plus	-21.7755	0.229358	0.412844	5.92661	-8.67347	-28.1224	-15.4679	5.26389	3.17431
0.00241	0.00953	0.0101	0.00266	0.00154	0.0583	0.00983	0.00233	0.00653
3L:21020460-21020610:minus	-13.3776	-3.57798	0.93578	2.12844	0.836735	9.30612	-26.3394	3.45833
-8.55046	0.000831	0.0302	0.00845	0.0134	0.000215	0.0000398	0.0558	0.005	0.152
3L:21020460-21020610:plus	-32.8163	1.07339	1.63303	-2.3211	-18.7245	-18.5714	-10.9725	6.09722	-0.330275
0.024	0.00705	0.00661	0.0569	0.0136	0.0136	0.00371	0.00158	0.0208
3L:21020940-21021090:minus	-32.1837	3.64286	-2.63303	6.12844	-18.4082	-18.5	-16.0826	4.68056
1.90826	0.0181	0.00159	0.026	0.00231	0.00937	0.0131	0.011	0.00302	0.00974
3L:21020940-21021090:plus	-32.4694	4.33028	1.3578	3.41284	-8.96939	-28.5612	-3	-2.08333	3.30275
0.0196	0.00212	0.00728	0.00756	0.00226	0.0636	0.000568	0.0305	0.00619
3L:21024820-21024970:minus	-32.0408	-2.08257	0.0366972	4.14679	-19.051	-9.82653	-19.0183	10.1667
-2.34862	0.0174	0.0199	0.0115	0.00542	0.0162	0.00381	0.0175	0.000145	0.0368
3L:21024820-21024970:plus	-24.5102	2.66055	15.7339	7.29358	20.2041	-17.0816	-14.0092	4.72222	1.6422
0.00807	0.00394	2.45e-07	0.00104	3.64e-07	0.00487	0.00727	0.00296	0.0111
3L:21027540-21027690:minus	-41.2143	-0.146789	-3.6422	2.26606	-18.6837	-9.5	-25.3853	2.26389
-7.09174	0.0494	0.0108	0.0345	0.0126	0.0126	0.00294	0.0496	0.00784	0.115
3L:21027540-21027690:plus	-22.4388	-2.2844	0.440367	5.48624	20.2041	-17.2041	-13.6422	-3.20833	2.11009
0.00348	0.0211	0.01	0.00357	3.64e-07	0.00507	0.00669	0.0407	0.00898
3L:21033560-21033710:minus	-22.6224	4	-4.05505	2.43119	-17.8878	-27.9694	-18.6422	3	1.74312
0.00371	0.0024	0.0385	0.0117	0.00704	0.0547	0.0165	0.00597	0.0105
3L:21033560-21033710:plus	-21.6735	-1.05505	8.00917	3.56881	-26.6633	-18.7143	-21.6697	-2.11111	-0.211009
0.00224	0.0146	0.000435	0.00699	0.0189	0.014	0.0278	0.0307	0.02
3L:21128700-21128850:minus	-31.5102	3.66972	3.02752	5.51376	-36.5204	-28.3367	-0.431193	-1.84722
8.10092	0.013	0.00272	0.00393	0.00348	0.0818	0.0606	0.000313	0.0286	0.000623
3L:21128700-21128850:plus	-4.47959	4.33028	-2.6422	2.14679	-18.0816	-28.6735	-13.0642	4.73611	3.45872
0.000154	0.00212	0.0261	0.0133	0.00759	0.0667	0.00587	0.00295	0.00591
3L:21129100-21129250:minus	-14.1429	13.4592	0.770642	3.56881	-27.2653	-19.0816	-9.07339	-1.55556
0.146789	0.0012	0.000064	0.00895	0.00699	0.0302	0.017	0.00259	0.0264	0.0178
3L:21129100-21129250:plus	-31.6633	6.17431	9.63303	3.42202	-17.3776	-18.2755	-11.3394	2.375	-1.22018
0.0141	0.000977	0.000177	0.00751	0.00419	0.0112	0.004	0.00753	0.0278
3L:211380-211630:minus	-38.9592	-5.6789	-1.77982	6.12844	10.398	1.27551	-19.3486	4.43056	7.73394
0.0324	0.0526	0.0202	0.00231	0.0000153	0.0000717	0.0185	0.00336	0.000784
3L:211380-211630:plus	-33.7041	2.80734	-5.63303	-1.17431	-27.4184	-0.316327	-24.3761	9.94444	-2.49541
0.0293	0.00373	0.0579	0.0388	0.0323	0.000524	0.0433	0.000171	0.0387
3L:21165500-21165650:minus	-13.8571	5.73394	3.55046	9.44954	-27.0408	-17.7143	-16.7064	0.902778
2.16514	0.0011	0.00119	0.00321	0.000326	0.0242	0.00634	0.0122	0.0125	0.00879
3L:21165500-21165650:plus	-32.7755	3.3578	13.1284	9.59633	-18.449	-18.4184	-15.367	1.23611	3.76147
0.0234	0.00305	0.0000181	0.000275	0.0107	0.0127	0.00965	0.0112	0.00494
3L:21168660-21168850:minus	-33.6327	0.0642202	6.85321	6.08257	-18.6429	0.612245	-12.0734	1.63889
-2.21101	0.029	0.0101	0.000779	0.00236	0.0121	0.000187	0.00469	0.00978	0.0354
3L:21168660-21168850:plus	-13.2551	1.6789	-0.880734	2.88991	20.4694	0.979592	-7.29358	-0.291667
-0.642202	0.000751	0.00565	0.0154	0.00942	1.65e-07	0.000136	0.00181	0.0183	0.0234
3L:21169060-21169210:minus	-22.5102	-3.68807	4.20183	9.41284	-17.0408	-9.5	-11.9083	-5.56944	-1.31193
0.00354	0.0311	0.00247	0.000354	0.00329	0.00294	0.00452	0.0696	0.0284
3L:21169060-21169210:plus	-31.4694	0.623853	1.80734	3.53211	-27.602	0.27551	-12.7431	3.41667	3.33945
0.0124	0.00829	0.00621	0.00704	0.0364	0.000257	0.00545	0.00508	0.00611
3L:21169740-21169890:minus	-30.8571	-4.54128	-0.66055	0.458716	-17.8571	-9.65306	-28.2018	6.33333
-1.56881	0.0103	0.0391	0.0143	0.024	0.00698	0.0034	0.0707	0.00141	0.0298
3L:21169740-21169890:plus	-22.4184	-0.908257	9.88073	6.86239	-19.0102	-8.5	-6.37615	0.375	-5.33028
0.00345	0.0139	0.000153	0.00136	0.0157	0.00126	0.00145	0.0149	0.0779
3L:21170340-21170490:minus	-21.2245	3.90816	-1.22018	2.19266	-27.0102	-17.2755	-20.8807	8.41667
7.11927	0.0019	0.00102	0.0171	0.0131	0.0237	0.00544	0.0241	0.000458	0.00125
3L:21170340-21170490:plus	-21.4388	-5.30275	5.30275	1.43119	-27	-27.8265	-17.5505	-2.97222	1.48624	0.002

0.0478 0.00157 0.0181 0.0233 0.0498 0.014 0.0384 0.0118
3L:21189760-21189910:minus -14.4796 3.97248 13.4312 7.29358 0.836735 -17.2755 -14.9358 2.45833
-3.56881 0.00142 0.00243 0.0000138 0.00104 0.000215 0.00544 0.00886 0.00731 0.0505
3L:21189760-21189910:plus -22.2959 0.238532 11 3.43119 -8.37755 -26.9694 -11.3119 9.26389 7.16514
0.00335 0.0095 0.0000802 0.00749 0.00106 0.0304 0.00398 0.00027 0.00122
3L:21194380-21194530:minus -31.4796 2.07339 6.76147 4.08257 -27.1939 -8.23469 -29.2018 -1.09722
-6.14679 0.0128 0.00488 0.000811 0.00558 0.0267 0.000875 0.0812 0.0232 0.0921
3L:21194380-21194530:plus -24.1531 0.899083 2.54128 5.21101 -27.3061 -7.42857 -26.9725 -2.06944 -2.44037
0.00744 0.00751 0.00473 0.0042 0.0315 0.000567 0.0603 0.0304 0.0381
3L:21195320-21195470:minus -41.5204 -0.770642 -2.3945 5.85321 -18.7143 -17.2041 -24.7431 7.56944
3.46789 0.0592 0.0133 0.0242 0.00287 0.0136 0.00507 0.0456 0.000743 0.00582
3L:21195320-21195470:plus -14.8163 7.02752 4.55963 6.29358 -27.2347 -27.5612 -5.55046 7.875 5.66055
0.00159 0.000639 0.00214 0.00202 0.0283 0.0393 0.00116 0.000626 0.00227
3L:21195980-21196130:minus -31.5918 2.38532 1.47706 0.752294 -17.3776 -9.65306 -9.58716 -3.41667
-3.51376 0.0136 0.00436 0.00698 0.0221 0.00419 0.0034 0.00285 0.0428 0.0498
3L:21195980-21196130:plus -33.1837 -3.74312 -1.88073 1.69725 -17.4082 -16.2755 -24.1835 1.93056 -10.6789
0.0274 0.0316 0.0209 0.0163 0.00421 0.00413 0.0422 0.00883 0.22
3L:21198380-21198530:minus -32.8469 0.944954 -2.42202 0.623853 -26.9694 -8.57143 -18.5505 9.09722
-1.38532 0.0243 0.00739 0.0244 0.0229 0.023 0.00149 0.0163 0.000301 0.0288
3L:21198380-21198530:plus -3.22449 -0.110092 -0.559633 7.43119 -17.1122 -17.7857 -4.11927 -0.444444
2.93578 0.0000495 0.0107 0.0139 0.000939 0.00341 0.0068 0.000772 0.0192 0.00717
3L:21209900-21210050:minus -23.6633 10.9908 3.95413 3.14679 -18.3878 -16.2755 -16.4128 4.06944
1.68807 0.00665 0.0000808 0.00273 0.00847 0.00931 0.00413 0.0116 0.00391 0.0109
3L:21209900-21210050:plus -3.43878 -1.72477 8.85321 7.21101 1.14286 -18.051 -16.5505 6.02778 -0.146789
0.0000694 0.0179 0.000275 0.0011 0.000113 0.00886 0.0119 0.00164 0.0194
3L:21233220-21233370:minus -22.1327 1.33028 1.09174 7.47706 -27.602 -18.4184 -13.2202 -3.09722
3.66972 0.00304 0.00642 0.00801 0.00085 0.0364 0.0127 0.00608 0.0396 0.00514
3L:21233220-21233370:plus -3.21429 1.63303 6.70642 10.8624 -17.4184 -17.7551 -4.53211 1.72222 6.95413
0.0000482 0.00575 0.000832 0.0000687 0.00457 0.00643 0.000868 0.0095 0.00136
3L:21249720-21249870:minus -22.9592 2.88991 1.37615 2.66972 -26.5918 -18.3469 -18.0459 6.19444
3.6422 0.00475 0.00362 0.00724 0.0106 0.0186 0.0124 0.0151 0.00151 0.00516
3L:21249720-21249870:plus -32.6633 -5.87156 3.86239 2.6055 -8.89796 -28.7041 -10.789 2.09722 8.62385
0.0216 0.0553 0.00283 0.0109 0.00189 0.067 0.00358 0.00832 0.00048
3L:21261020-21261170:minus -21.9286 2.93578 1.3578 2.82569 -7.96939 -18.2041 -16.2477 -0.0277778
1.41284 0.0027 0.00356 0.00728 0.00976 0.000785 0.0103 0.0113 0.0169 0.0121
3L:21261020-21261170:plus -24.4796 3.68367 -0.0275229 2.88073 -27.1939 -17.5 -11.6055 1.40278
-2.83486 0.00804 0.00144 0.0117 0.00945 0.0267 0.00599 0.00423 0.0106 0.0427
3L:212680-212830:minus -31.4796 -0.110092 3.25688 6.01835 2.14286 -18.1224 -19.6789 0.166667
1.34862 0.0128 0.0107 0.0036 0.00248 0.0000316 0.00988 0.0195 0.0159 0.0125
3L:212680-212830:plus -22.9184 0.834862 -1.89908 5.31193 1.17347 -18.0102 -4.31193 8.41667 3.94495
0.00447 0.00769 0.021 0.00393 0.000101 0.00812 0.000815 0.000458 0.00452
3L:21269940-21270090:minus -31.8878 -0.330275 0.972477 2.55046 -18.6837 -18.2755 -23.6422 1.91667
8.20183 0.0164 0.0115 0.00835 0.0112 0.0126 0.0112 0.0389 0.00888 0.000539
3L:21269940-21270090:plus -13.2245 -2.14679 1.66972 6.86239 -17.4898 -18.2755 -6.94495 -3.27778 8.62385
0.000738 0.0203 0.00652 0.00136 0.0053 0.0112 0.00167 0.0414 0.00048
3L:21270280-21270430:minus -22.1837 -0.0275229 1.88991 1.50459 -8.67347 -28.9694 -14.2294 4.91667
7.22018 0.00315 0.0104 0.00602 0.0176 0.00154 0.0721 0.00763 0.00272 0.00119
3L:21270280-21270430:plus -24.1837 8.33945 7.87156 5.57798 -17.1531 0.0918367 -20.3303 -0.111111
-4.84404 0.00751 0.000317 0.000469 0.0034 0.00364 0.000323 0.0218 0.0173 0.0701
3L:21273140-21273290:minus -3.29592 0.522936 0.981651 7.17431 -18.7551 -27.4082 -10.1101 1.56944
5.13761 0.0000549 0.00859 0.00832 0.00115 0.0144 0.0356 0.00314 0.01 0.00298
3L:21273140-21273290:plus -23.0306 3.89908 -0.165138 2.44037 -17.4184 -16.2755 -14.4037 -1.75
4.10092 0.00505 0.0025 0.0122 0.0116 0.00457 0.00413 0.00792 0.0279 0.00413
3L:21274580-21274730:minus -23.1837 14.2661 6.91743 1.44037 -17.7143 -8.57143 -13.1101 1.34722

-1.77982 0.00553 6.1e-06 0.000755 0.018 0.00627 0.00149 0.00593 0.0108 0.0314
3L:21274580-21274730:plus -32.7347 14.1743 5.91743 1.78899 -8.82653 -19.4184 -22.2385 4.66667 3.6789
0.0225 6.8e-06 0.0012 0.0158 0.00163 0.0198 0.0308 0.00304 0.00506
3L:21279040-21279190:minus -31.2551 1.74312 2.48624 5.07339 0.836735 -18.3469 -19.4954 -4.01389
6.07339 0.0113 0.00552 0.00483 0.0043 0.000215 0.0124 0.0189 0.0494 0.0018
3L:21279040-21279190:plus -39.9898 -0.183486 -0.605505 3.46789 -8.93878 -9.86735 -0.614679
2.61111 2.88991 0.0351 0.011 0.0141 0.00726 0.00216 0.00391 0.000326 0.00691 0.00725
3L:21297640-21297790:minus -22.8878 -0.0550459 11.055 4.0367 -18.1122 -7.72449 -17.1468 2.43056
-3.82569 0.00441 0.0105 0.0000772 0.00578 0.00771 0.00068 0.0131 0.00738 0.0531
3L:21297640-21297790:plus -13.9592 0.697248 3.44954 4.81651 -27.5306 -18.0102 -8.33028 5.02778 -0.302752
0.00113 0.00807 0.00334 0.00451 0.0351 0.00812 0.00225 0.00259 0.0208
3L:21311040-21311190:minus -13.3673 13.5306 3.9633 3.77064 -18.7143 -26.6735 -12.5505 0.444444
-0.706422 0.000813 0.0000206 0.00272 0.00637 0.0136 0.0256 0.00522 0.0145 0.024
3L:21311040-21311190:plus -22.2245 -0.908257 -0.0825688 -0.119266 -17.1122 -17.2041 -24.4128
-3.34722 -2.00917 0.00325 0.0139 0.0119 0.0278 0.00341 0.00507 0.0436 0.0421 0.0333
3L:21328220-21328370:minus -30.6327 0.183486 2.08257 6.12844 -26.5306 -9.65306 -18.3119 3.95833
-6.19266 0.00968 0.00968 0.0056 0.00231 0.0185 0.0034 0.0157 0.00409 0.0932
3L:21328220-21328370:plus -33.7755 9.41284 1.23853 2.86239 -27.1224 -18.9286 -14.367 -0.152778
6.17431 0.0297 0.000181 0.0076 0.00954 0.0245 0.0153 0.00786 0.0175 0.00171
3L:21335820-21335970:minus -24.0714 -1.70642 7.11009 6.00917 -27.898 -9.7551 -8.17431 2.51389
3.88991 0.00731 0.0178 0.000691 0.00255 0.04 0.00358 0.00218 0.00716 0.00467
3L:21335820-21335970:plus -12.6633 -0.59633 -0.0550459 4.38532 -9.0102 -19.3469 -12.8165 0.541667
6.73394 0.000458 0.0126 0.0118 0.00501 0.00236 0.0194 0.00554 0.0141 0.00143
3L:21336480-21336630:minus -22.1531 2.46789 0.614679 0.0642202 -27.9388 -17.2755 -13.6147 2.58333
4.78899 0.0031 0.00422 0.00943 0.0264 0.0415 0.00544 0.00665 0.00698 0.00336
3L:21336480-21336630:plus -22.8878 2.16514 5.75229 2.76147 -27.2347 -17.2755 -9.19266 1.41667 4.3945
0.00441 0.00473 0.00129 0.01 0.0283 0.00544 0.00265 0.0106 0.00375
3L:21339880-21340030:minus -33.0408 3.93578 1.68807 3.88991 -27.4592 -28.602 -12.3394 0.263889
8.05505 0.0264 0.00246 0.00648 0.00608 0.0329 0.0648 0.00497 0.0154 0.00064
3L:21339880-21340030:plus -15.0102 0.761468 3.74312 2.61468 -26.5918 -26.8265 -14.9633 -0.555556
8.14679 0.00162 0.00789 0.00297 0.0109 0.0186 0.0285 0.00891 0.0198 0.000562
3L:21340640-21340790:minus -33 -2.04587 2 6.14679 -27.5306 -26.4898 -19.5413 1.63889 -5.66972
0.0258 0.0197 0.00578 0.00224 0.0351 0.0225 0.0191 0.00978 0.0829
3L:21340640-21340790:plus -31.8878 5.19266 1.10092 2.22018 -8.64286 -18.2755 -22.2477 1.84722 -5.68807
0.0164 0.0015 0.00798 0.0129 0.00139 0.0112 0.0308 0.0091 0.0831
3L:21348200-21348350:minus -40.4796 3.98165 -1.93578 1.2844 -27.1939 -17.7857 -25.9725 1.875
7.22018 0.0407 0.00242 0.0212 0.019 0.0267 0.0068 0.0533 0.00901 0.00119
3L:21348200-21348350:plus -14.4796 1.57798 7.84404 3.69725 -26.898 -36.0816 -13.6881 -6.375 4.3945
0.00142 0.00586 0.000476 0.00665 0.0203 0.0857 0.00676 0.082 0.00375
3L:21354400-21354550:minus -30.2245 -1.65138 8.82569 3.94495 -18.449 -18.051 -24.8991 6.05556
2.41284 0.00881 0.0175 0.000279 0.00595 0.0107 0.00886 0.0465 0.00161 0.0082
3L:21354400-21354550:plus -23.2551 -0.66055 -0.816514 5.22936 -8.67347 -19.1531 -15.2477 -2.41667
1.68807 0.00583 0.0129 0.0151 0.0041 0.00154 0.0177 0.00943 0.0333 0.0109
3L:21374460-21374610:minus -4.81633 3.68367 2.41284 5.92661 -8.89796 -18.3469 -25.7339 -0.541667
-2.68807 0.000197 0.00144 0.00496 0.00266 0.00189 0.0124 0.0518 0.0197 0.041
3L:21374460-21374610:plus -14.0102 0.422018 -1.70642 9.59633 -27.3776 0.316327 -18.5596 7.59722 7.72477
0.00114 0.0089 0.0198 0.000275 0.032 0.000243 0.0163 0.000731 0.000798
3L:21375460-21375610:minus -40.0714 0.321101 -2.15596 -1.07339 -17.4898 -18.5612 -18.2202 5.45833
1.90826 0.0359 0.00922 0.0226 0.0377 0.0053 0.0132 0.0155 0.00214 0.00974
3L:21375460-21375610:plus -23.1122 2.08257 9.86239 7.15596 -17.3776 -9.23469 -9.73394 -6.625 3.87156
0.0053 0.00487 0.000155 0.00117 0.00419 0.00238 0.00293 0.0861 0.00477
3L:21426740-21426890:minus -21.7755 3.97959 -1.53211 6.3578 -17.602 0.387755 -14.3945 0 1.6789
0.00241 0.000669 0.0188 0.00195 0.00547 0.000217 0.00791 0.0167 0.011
3L:21426740-21426890:plus -23.8878 -0.165138 -2.08257 -0.458716 -26.9694 -9.5 -12.2477 -3.51389

9.30275 0.00692 0.0109 0.0222 0.0308 0.023 0.00294 0.00487 0.0439 0.00038
3L:21434460-21434610:minus -33.3367 6.66055 4.74312 3.12844 11.6939 1.02041 -26.8532 7.20833
-3.53211 0.0281 0.000771 0.00198 0.00857 1.17e-06 0.000118 0.0594 0.000902 0.0501
3L:21434460-21434610:plus -33.0306 13.3878 2.58716 0.642202 -9.0102 -27.2653 -17 2.44444 1.69725 0.026
0.0000869 0.00465 0.0227 0.00236 0.0333 0.0128 0.00734 0.0108
3L:21438880-21439030:minus -33.3673 5.47706 2.10092 3.05505 -27.7551 -8.72449 -17.9633 1.48611
-2.65138 0.0282 0.00133 0.00556 0.00878 0.0385 0.00169 0.0149 0.0103 0.0406
3L:21438880-21439030:plus -31.7755 1.72477 2.48624 9.59633 -18.4082 -8.5 -16.8349 3.30556 -0.770642
0.0157 0.00555 0.00483 0.000275 0.00937 0.00126 0.0125 0.0053 0.0245
3L:21490460-21490610:minus -31.7041 -1.76147 3.97248 2.88991 20.4694 -19.1939 -2.89908 1.81944
5.90826 0.0146 0.0181 0.00271 0.00942 1.65e-07 0.0178 0.000553 0.00919 0.00198
3L:21490460-21490610:plus -13.3673 -2.74312 5.07339 3.04587 -17.1531 -17.2755 -7.66055 7.34722 1.16514
0.000813 0.024 0.00172 0.00883 0.00364 0.00544 0.00196 0.000838 0.0135
3L:21500640-21500790:minus -23.2245 15.789 3.47706 4.6055 -18.7551 0.540816 -19.8257 0.652778
1.70642 0.00576 3.73e-07 0.0033 0.00476 0.0144 0.0002 0.02 0.0136 0.0108
3L:21500640-21500790:plus -30.4388 12.844 -1.33028 4.43119 -18.4184 -9.79592 -18.7156 4.65278 5.38532
0.00923 0.0000255 0.0177 0.00495 0.0101 0.00377 0.0167 0.00305 0.00267
3L:2150440-2150590:minus -22.7041 5.55963 1.50459 7.02752 -18.1531 -18.3469 -5.6422 -1.18056 3.46789
0.00395 0.00129 0.00692 0.00122 0.00829 0.0124 0.00119 0.0238 0.00582
3L:2150440-2150590:plus -22.2245 5.21101 -0.752294 5.37615 -18.4184 1.2449 -8.66055 -3.76389
1.00917 0.00325 0.00149 0.0148 0.00384 0.0101 0.0000797 0.0024 0.0466 0.0143
3L:21509740-21509890:minus -32.8163 5.18349 -1.3211 2.11009 -7.93878 0.0204082 -18.7523 2.81944
-5.97248 0.024 0.00151 0.0176 0.0136 0.000767 0.000377 0.0168 0.00639 0.0883
3L:21509740-21509890:plus -40.7041 3.68367 5.88991 6.02752 -36.7041 -17.2755 -21.8532 1.43056 -6.81651
0.0428 0.00144 0.00121 0.00246 0.0863 0.00544 0.0287 0.0105 0.109
3L:21510180-21510470:minus -24 -4.21101 -0.66055 4.78899 20.2041 -17.7551 -21.5688 2.08333 4.66055
0.00719 0.0358 0.0143 0.00456 3.64e-07 0.00643 0.0273 0.00837 0.0035
3L:21510180-21510470:plus -32.4388 0.165138 0.119266 6.27523 11.6939 -9.79592 -30.4037 4.66667 -5.04587
0.0195 0.00974 0.0111 0.00206 1.17e-06 0.00377 0.0962 0.00304 0.0735
3L:21516200-21516350:minus -33.2653 -2.25688 0.770642 -1.05505 -17.3469 -9.09184 -31.0826 5.19444
-4.86239 0.0277 0.0209 0.00895 0.0374 0.00394 0.00219 0.105 0.00241 0.0705
3L:21516200-21516350:plus -31.8571 -0.486239 3.76147 9.44037 1.87755 -7.57143 -17.8716 9.18056
-2.18349 0.0163 0.0121 0.00295 0.000339 0.0000492 0.000644 0.0147 0.000285 0.0351
3L:21516860-21517010:minus -21.8163 2.31193 3.44037 4.49541 1.14286 -8.65306 -6.00917 4.90278
-0.0825688 0.00244 0.00448 0.00335 0.00487 0.000113 0.00156 0.00131 0.00274 0.0189
3L:21516860-21517010:plus -24.2245 -1.6789 3.26606 5.46789 -25.9694 -26.602 -17.3761 0.694444 -1.04587
0.00763 0.0177 0.00358 0.00362 0.017 0.0248 0.0136 0.0134 0.0264
3L:2151740-2151890:minus 7.5102 -1.02752 4.49541 10.0275 -17.6429 0.316327 -2.63303 -1.86111 7.58716
1.45e-06 0.0145 0.00219 0.000156 0.00562 0.000243 0.000517 0.0287 0.000954
3L:2151740-2151890:plus -21.8469 3.90816 5.73394 6.02752 -18.4898 -19.0102 -11.3028 6.73611 9.45872
0.00247 0.00102 0.0013 0.00246 0.0116 0.0159 0.00397 0.00115 0.000337
3L:21522360-21522510:minus -22.4898 -1.31193 14.1651 7.29358 0.540816 -8.23469 -12.1376 4.68056
-0.990826 0.00353 0.0158 6.03e-06 0.00104 0.000321 0.000875 0.00475 0.00302 0.026
3L:21522360-21522510:plus -31.7347 3.90816 -1.66972 3.54128 -19.0102 -8.93878 -13.6514 5.98611 -0.110092
0.0149 0.00102 0.0196 0.00702 0.0157 0.00201 0.00671 0.00167 0.0191
3L:21530080-21530230:minus -23.1837 1.77982 7.69725 6.73394 -17.4184 -17.3469 -9.62385 3.83333
-3.78899 0.00553 0.00544 0.000513 0.00152 0.00457 0.0058 0.00287 0.0043 0.0526
3L:21530080-21530230:plus -31.7755 2.87156 3.91743 4.08257 -8.64286 -27.9694 -18.9083 0.958333 0.513761
0.0157 0.00365 0.00277 0.00558 0.00139 0.0547 0.0172 0.0123 0.0162
3L:21532480-21532630:minus -24.2959 6.43119 0.761468 2.3578 -27.602 -19.051 -14.8807 0.361111
3.09174 0.00781 0.000862 0.00897 0.0121 0.0364 0.0165 0.00876 0.0149 0.00678
3L:21532480-21532630:plus -32.8163 6.10092 -1.06422 3.01835 -18.7143 -18.2755 -3.13761 1.91667 0.963303
0.024 0.00101 0.0163 0.0089 0.0136 0.0112 0.000589 0.00888 0.0145
3L:21532860-21533010:minus -32.6633 1.56881 -4.16514 -2.53211 -17.6735 -17.5 -25.1284 6.58333

-0.642202 0.0216 0.00588 0.0397 0.0608 0.00572 0.00599 0.048 0.00124 0.0234
3L:21532860-21533010:plus -21.3673 7.91743 3.99083 1.42202 10.7245 -19.2755 -15.2844 0.652778 -0.229358
0.00195 0.000399 0.00269 0.0182 5.2e-06 0.0185 0.00949 0.0136 0.0202
3L:21533160-21533310:minus -31.3673 -3.66972 3.06422 6.84404 -7.60204 -19.3776 -23.1009 -2.38889
-3.77982 0.0116 0.031 0.00387 0.0014 0.000466 0.0195 0.0357 0.0331 0.0525
3L:21533160-21533310:plus -30.4082 -4.87156 1.92661 3.50459 -17.4898 -7.79592 -15.2569 1.86111 -2.92661
0.00908 0.0427 0.00594 0.00713 0.0053 0.000723 0.00944 0.00905 0.0437
3L:21547640-21547790:minus -24.0306 -0.899083 3.26606 2.84404 -27 -24.8265 -5.75229 6.93056
4.56881 0.00723 0.0139 0.00358 0.00961 0.0233 0.0204 0.00123 0.00104 0.00356
3L:21547640-21547790:plus -21.3673 -2.01835 0.899083 7 -17.6837 -17.051 -19.4954 1.36111 4.33028
0.00195 0.0195 0.00856 0.00132 0.0058 0.00479 0.0189 0.0108 0.00385
3L:21548040-21548190:minus -31.4388 14.9908 6.33945 6.89908 -19.3163 -9.79592 -12.9908 -3.16667
3.98165 0.0122 2.1e-06 0.000991 0.00134 0.0165 0.00377 0.00577 0.0403 0.00439
3L:21548040-21548190:plus -32.8469 -0.844037 5.2844 1.37615 -17.3776 -18.4184 -16.1468 -2.66667
3.61468 0.0243 0.0136 0.00158 0.0185 0.00419 0.0127 0.0111 0.0355 0.00526
3L:21562000-21562150:minus -3.81633 13.5306 -0.642202 2.53211 -18.4592 -18.5714 -23.1651 -0.958333
3.81651 0.000106 0.0000206 0.0143 0.0113 0.0109 0.0136 0.036 0.0223 0.00482
3L:21562000-21562150:plus -34.602 6 -0.706422 4.91743 -17.9796 -7.7551 -11.8165 2.77778 -0.293578
0.032 0.00106 0.0146 0.00447 0.00726 0.000682 0.00443 0.00649 0.0207
3L:21567540-21567690:minus -32.8469 0.348624 1.66972 3.23853 -18.1939 -19.3061 -10.6422 4.70833
1.36697 0.0243 0.00914 0.00652 0.00815 0.0086 0.0187 0.00348 0.00298 0.0124
3L:21567540-21567690:plus -31.5918 -1.69725 1.6789 7.02752 10.6939 19.1531 -9.75229 1.23611 1.59633
0.0136 0.0178 0.0065 0.00122 7.35e-06 7.95e-07 0.00294 0.0112 0.0113
3L:21610560-21610710:minus -23.7347 -0.513761 14.8899 2.25688 -8.89796 1.05102 -21.6055 4.43056
-3.40367 0.00673 0.0123 2.06e-06 0.0127 0.00189 0.000107 0.0275 0.00336 0.0486
3L:21610560-21610710:plus -21.3265 -2.17431 -0.412844 3.53211 20.2041 -18.0816 -24.3211 1.48611
-5.47706 0.00193 0.0204 0.0133 0.00704 3.64e-07 0.00934 0.043 0.0103 0.0801
3L:21619320-21619470:minus -31.6633 6.55046 2.86239 5.59633 -18.0102 -18.0816 -28.0826 7.91667
-0.642202 0.0141 0.000814 0.00418 0.00331 0.00732 0.00934 0.0696 0.000611 0.0234
3L:21619320-21619470:plus -31.8469 2.83486 -0.752294 5.73394 -18.9796 -9.53061 -4.57798 -0.722222
-0.0642202 0.0162 0.0037 0.0148 0.00307 0.0155 0.0031 0.00088 0.0208 0.0188
3L:21627480-21627630:minus -32.4388 -0.174312 0.642202 5.94495 -18.1531 0.0510204 -20.5872
2.26389 -0.504587 0.0195 0.0109 0.00935 0.00261 0.00829 0.000349 0.0228 0.00784 0.0221
3L:21627480-21627630:plus -31.7041 5.33945 3.48624 6.56881 -18.6429 -9.45918 -14.4037 9.22222 0.587156
0.0146 0.00142 0.00329 0.0017 0.0121 0.00279 0.00792 0.000278 0.016
3L:21628340-21628490:minus -32.4796 3.50459 4.30275 3.12844 -27.1939 -18.2755 -8.36697 0.777778
10.3486 0.02 0.00289 0.00237 0.00857 0.0267 0.0112 0.00227 0.0131 0.0000988
3L:21628340-21628490:plus -32.0714 3.22936 -2.3945 2.78899 10.6531 19.0816 -15.4954 0.583333 5.90826
0.0176 0.0032 0.0242 0.00992 0.0000102 1.12e-06 0.00989 0.0139 0.00198
3L:21633960-21634110:minus -32.398 -0.302752 6.09174 7.17431 -28.5306 -8.53061 -15.2661 2.56944
5.6055 0.019 0.0114 0.00111 0.00115 0.061 0.00136 0.00946 0.00701 0.00232
3L:21633960-21634110:plus -24.1939 -1.59633 -3.15596 6.17431 10.9898 0.755102 -15.8257 8.94444 3.51376
0.00753 0.0172 0.0301 0.0022 3.28e-06 0.000175 0.0105 0.000332 0.00566
3L:21673000-21673150:minus -32.4796 5.84404 -2.46789 4.94495 19.9082 -18.6429 -16.1651 0.722222
3.46789 0.02 0.00113 0.0248 0.00444 8.13e-07 0.0139 0.0112 0.0133 0.00582
3L:21673000-21673150:plus -31.7041 3.04587 15.5596 7.29358 0.571429 0.826531 -17.844 3.56944 -6.88991
0.0146 0.00342 6.69e-07 0.00104 0.0003 0.000153 0.0146 0.00478 0.11
3L:2169120-2169270:minus -30.8571 -0.66055 14.3486 7.29358 -26.8571 -17.9388 -14.7798 -0.180556
6.11927 0.0103 0.0129 4.67e-06 0.00104 0.0198 0.00738 0.00858 0.0177 0.00178
3L:2169120-2169270:plus -23.4898 -0.238532 6.56881 1.88073 -17.9796 -0.244898 -16.0642 6.98611
11.3853 0.00639 0.0112 0.000888 0.0152 0.00726 0.000496 0.011 0.00101 0.0000815
3L:2171060-2171210:minus -23.4898 -2.72477 -0.917431 6.21101 -18.449 -18.051 -8.48624 0.611111
3.46789 0.00639 0.0239 0.0156 0.00213 0.0107 0.00886 0.00232 0.0138 0.00582
3L:2171060-2171210:plus -23.2959 1.88991 3.66972 1.63303 -18.7143 -8.79592 -17.0367 10.2639 -2.26606

0.00605 0.00523 0.00306 0.0167 0.0136 0.0019 0.0129 0.000135 0.0359
3L:21721760-21722030:minus -31.4388 6.14679 5.01835 1.86239 -17.4184 -26.8571 -19.6697 1.26389
6.07339 0.0122 0.00099 0.00176 0.0153 0.00457 0.0288 0.0195 0.0111 0.0018
3L:21721760-21722030:plus -31.4388 -0.724771 0.00917431 3.6422 -7.93878 -9.82653 -14.3394 1.13889
-0.293578 0.0122 0.0131 0.0116 0.00669 0.000767 0.00381 0.00782 0.0116 0.0207
3L:21763640-21763790:minus -3.56122 -0.678899 -1.51376 0.963303 -17.0816 -9.5 -20.844 6.72222
5.49541 0.0000803 0.0129 0.0187 0.0208 0.00334 0.00294 0.0239 0.00116 0.0025
3L:21763640-21763790:plus -31.8163 -1.92661 5.83486 3.07339 -25.8571 -17.5 -12.9266 8.94444 4.30275
0.016 0.019 0.00124 0.00873 0.0166 0.00599 0.00568 0.000332 0.00388
3L:21814940-21815090:minus -32.1939 1.86239 -1.47706 6.17431 -27.5 -8.86735 -22.3945 4.77778
-1.43119 0.0181 0.00528 0.0185 0.0022 0.0337 0.00198 0.0316 0.00289 0.029
3L:21814940-21815090:plus -30.4388 6.20183 9.86239 4.22018 0.806122 -17.8571 -19.4128 10.1667 4.19266
0.00923 0.000962 0.000155 0.00531 0.000229 0.00719 0.0187 0.000145 0.00401
3L:21816340-21816490:minus -33.5612 -1.15596 -0.853211 1.12844 -18.0102 -0.469388 -10.1193
4.81944 -0.0733945 0.0288 0.0151 0.0152 0.0198 0.00732 0.000533 0.00315 0.00284 0.0188
3L:21816340-21816490:plus -30.2857 -0.192661 2.10092 9.41284 10.9184 -8.79592 -7.50459 -0.416667
-2.15596 0.00887 0.011 0.00556 0.000354 4.19e-06 0.0019 0.00189 0.019 0.0348
3L:21818000-21818150:minus -22.3265 13.4592 -7.52294 2.3578 -18.4898 -19.1939 5.16514 1.79167
-0.59633 0.00337 0.000064 0.0901 0.0121 0.0116 0.0178 0.0000544 0.00928 0.0229
3L:21818000-21818150:plus -22.6633 -0.816514 5.30275 4.17431 -27.3061 -26.4898 -15.0183
3.01389 -0.0733945 0.00384 0.0135 0.00157 0.00537 0.0315 0.0225 0.00901 0.00593 0.0188
3L:21825340-21825490:minus -32.7041 -0.944954 1.56881 7.27523 10.3878 -16.2755 -23.7706
-3.08333 6.7156 0.0222 0.0141 0.00676 0.00109 0.0000174 0.00413 0.0397 0.0395 0.00144
3L:21825340-21825490:plus -40.551 3.0367 0.0642202 1.66972 -27.5306 -0.244898 -27.3853
7.58333 -4.61468 0.0418 0.00343 0.0113 0.0165 0.0351 0.000496 0.0635 0.000737 0.0658
3L:21825740-21825890:minus -31.6633 4.9633 2.81651 7.00917 -17.2245 -8.27551 -13.5872
5.52778 3.49541 0.0141 0.00165 0.00426 0.00126 0.00384 0.000919 0.00661 0.00207 0.0057
3L:21825740-21825890:plus -24 -0.201835 1.21101 4.33028 1.10204 -17.051 -23.3211 2.98611 1.89908
0.00719 0.011 0.00768 0.00511 0.000124 0.00479 0.037 0.006 0.0098
3L:21833160-21833310:minus -20.9286 14.2385 6.44954 9.24771 -18.4184 -19.1224 -16.367 1.63889
7.6789 0.00183 6.29e-06 0.000941 0.000409 0.0101 0.0175 0.0116 0.00978 0.000866
3L:21833160-21833310:plus -11.9592 0.605505 4.93578 2.12844 -17.449 0.683673 -8.44037
0.902778 9.66055 0.000301 0.00834 0.00183 0.0134 0.00484 0.000184 0.0023 0.0125 0.000268
3L:21833820-21833970:minus -22.8469 -3.12844 2.77982 8.80734 -27.1224 -18.0816 -18.2018
4.33333 2.75229 0.00424 0.0267 0.00432 0.000518 0.0245 0.00934 0.0154 0.0035 0.00748
3L:21833820-21833970:plus -23.8878 5.57798 2.11009 -2.41284 -18.1939 -18.2755 2.81651
6.83333 9.66055 0.00692 0.00128 0.00554 0.0585 0.0086 0.0112 0.000126 0.0011 0.000268
3L:21872580-21872730:minus -31.3673 -0.0366972 2.34862 2.78899 -18.1531 -27.5612 -11.6881
-0.722222 8.10092 0.0116 0.0104 0.00508 0.00992 0.00829 0.0393 0.00431 0.0208 0.000623
3L:21872580-21872730:plus -3.53061 -2.89908 3.6789 7.17431 -26.8571 -8.53061 -16.4862
2.43056 -3.36697 0.0000763 0.0251 0.00305 0.00115 0.0198 0.00136 0.0118 0.00738 0.0482
3L:21873340-21873490:minus -30.7755 -1.86239 1.54128 3.48624 -18.7551 -8.93878 -19.1835
-0.472222 8.10092 0.0102 0.0187 0.00683 0.0072 0.0144 0.00201 0.018 0.0193 0.000623
3L:21873340-21873490:plus -30.3673 3.64286 -0.559633 3.37615 -18.6837 9.86735 -24.3028
-5.06944 -8.45872 0.00897 0.00159 0.0139 0.00762 0.0126 0.0000132 0.0429 0.0626 0.149
3L:21873660-21873810:minus -13.1429 1.07339 1.79817 1.91743 -17.4898 -8.45918 -5.45872
0.805556 11.3853 0.00065 0.00705 0.00623 0.015 0.0053 0.00111 0.00113 0.013 0.0000815
3L:21873660-21873810:plus -12.4796 -2.10092 0.724771 2.83486 -17.9388 -18.5 5.41284 0.5
3.17431 0.000433 0.02 0.00909 0.0097 0.00715 0.0131 5e-05 0.0143 0.00653
3L:21879460-21879610:minus -31 5.68807 -0.770642 3.29358 -8.86735 -0.244898 -20.5872
4.02778 -4.15596 0.0105 0.00122 0.0149 0.00791 0.00173 0.000496 0.0228 0.00397 0.0578
3L:21879460-21879610:plus -31.4796 1.66055 0.0183486 7.27523 -27.0408 -17.2755 -17.0367
2.43056 2.58716 0.0128 0.00569 0.0115 0.00109 0.0242 0.00544 0.0129 0.00738 0.00779
3L:21880240-21880390:minus -14.0306 2.91743 0.651376 1.63303 -8.71429 -18.0816 -2.29358

2.40278 7.84404 0.00115 0.00358 0.00932 0.0167 0.00159 0.00934 0.000476 0.00746 0.000701
3L:21880240-21880390:plus -31.3673 1.89908 10.6789 7.77982 -8.37755 0.540816 -14.1651
6.38889 2.09174 0.0116 0.00521 0.0000957 0.000652 0.00106 0.0002 0.00752 0.00137 0.00904
3L:2192240-2192390:minus -21.8878 -1.3578 5.55046 10.7615 -17.1531 -0.173469 -16.9358
7.83333 -0.440367 0.00258 0.016 0.00141 0.0000826 0.00364 0.000456 0.0127 0.000641 0.0216
3L:2192240-2192390:plus -14.3061 7.97248 3.20183 9.3211 -18.4184 -17.2755 -25.9358 2.63889
10.1284 0.00129 0.000387 0.00367 0.000391 0.0101 0.00544 0.0531 0.00684 0.000129
3L:21935180-21935330:minus -32.7449 9.86239 0.944954 2.27523 -9.45918 1.7551 -11.8073
0.75 3.01835 0.0228 0.000143 0.00842 0.0125 0.00287 0.0000519 0.00442 0.0132 0.00697
3L:21935180-21935330:plus -32.8469 -0.119266 2.06422 3.18349 19.9082 -8.72449 -16.5138
4.73611 1.22018 0.0243 0.0107 0.00564 0.00837 8.13e-07 0.00169 0.0118 0.00295 0.0133
3L:22058840-22058990:minus -30.4796 4.62385 7.2844 4.50459 -18.2653 -8.72449 -20.0734
-1.97222 -2.93578 0.00941 0.00189 0.000632 0.00484 0.00882 0.00169 0.0209 0.0296 0.0439
3L:22058840-22058990:plus -13.8571 -2.22936 5.08257 7.17431 -8.89796 -7.93878 -10.1468
-0.666667 0.917431 0.0011 0.0208 0.00172 0.00115 0.00189 0.000736 0.00316 0.0205 0.0146
3L:22064900-22065050:minus -31.398 11.5229 -0.0825688 1.78899 -27 -28.2653 -17.5046 1.76389
1.63303 0.0117 0.0000606 0.0119 0.0158 0.0233 0.0595 0.0139 0.00937 0.0112
3L:22064900-22065050:plus -20.9286 0.284404 7.10092 2.05505 -18.1939 -16.7857 -15.9174
3.125 1.73394 0.00183 0.00935 0.000693 0.0139 0.0086 0.00426 0.0107 0.00569 0.0106
3L:22065440-22065590:minus -23.5204 9.6789 -0.614679 4.20183 -9.19388 -16.3469 -23.0092
1.75 -3.93578 0.00645 0.000157 0.0141 0.00533 0.00253 0.00416 0.0351 0.00941 0.0547
3L:22065440-22065590:plus -42.6327 12.1927 -1.30275 0.00917431 -17.5306 -7.53061
-6.6055 5.58333 -4.22936 0.0829 0.000041 0.0175 0.0269 0.0054 0.000625 0.00153 0.00202 0.0588
3L:22067540-22067690:minus -32.9592 -2.18349 5.66055 6.69725 -18.6429 -0.173469 -22.7982
-1.93056 3.66972 0.0252 0.0205 0.00134 0.00156 0.0121 0.000456 0.0339 0.0293 0.00514
3L:22067540-22067690:plus -34.3673 10.6881 3.55046 6.56881 -18.8265 -8.72449 -29.156 7.19444
-0.513761 0.0318 0.0000941 0.00321 0.0017 0.0149 0.00169 0.0807 0.000909 0.0221
3L:22068920-22069070:minus -33.0408 13.5306 -0.348624 3.77064 -18.9388 -8.23469 -12.2477
5.65278 -0.513761 0.0264 0.0000206 0.013 0.00637 0.0151 0.000875 0.00487 0.00195 0.0221
3L:22068920-22069070:plus -12.1837 3.37615 -2.69725 5.98165 20.4694 -8.23469 4.41284 -0.319444
5.38532 0.000365 0.00303 0.0265 0.00258 1.65e-07 0.000875 0.000071 0.0184 0.00267
3L:22255980-22256130:minus -32.0714 1.62385 13.0183 5.33028 -17.7143 -8.45918 -14.4587
1.11111 -0.0275229 0.0176 0.00577 0.0000203 0.00389 0.00627 0.00111 0.00802 0.0117 0.0186
3L:22255980-22256130:plus -31.7347 7.78899 15.0367 7.29358 -17.5306 -18.8571 -11.7523
2.43056 1.22018 0.0149 0.000429 1.36e-06 0.00104 0.0054 0.0151 0.00437 0.00738 0.0133
3L:22256380-22256530:minus -32.6633 3.83673 1.26606 1.38532 -18.9796 -9.02041 -17.3211
5.38889 0.779817 0.0216 0.00115 0.00753 0.0185 0.0155 0.00213 0.0135 0.0022 0.0151
3L:22256380-22256530:plus -32.1429 4.94495 3.30275 5.85321 -27.4898 -19.0102 -8.40367
4.80556 5.79817 0.0179 0.00166 0.00353 0.00287 0.0336 0.0159 0.00228 0.00286 0.00208
3L:22259760-22259910:minus -31.8163 2.88073 11.156 5.2844 -17.2245 -27.9694 -9.44037
5.47222 3.98165 0.016 0.00363 0.0000725 0.004 0.00384 0.0547 0.00278 0.00212 0.00439
3L:22259760-22259910:plus -21.6224 6.11009 -1.29358 2.62385 1.10204 -9.5 -12.3761 2.70833
2.20183 0.00213 0.00101 0.0175 0.0108 0.000124 0.00294 0.00501 0.00666 0.00869
3L:22260500-22260650:minus -31.551 5.77982 8.26606 0.412844 -7.37755 -27.6327 -10.9725
4.54167 2.20183 0.0134 0.00117 0.00038 0.0242 0.000398 0.043 0.00371 0.0032 0.00869
3L:22260500-22260650:plus -31.7755 4.11009 3.00917 9.7156 -28.3061 -17.7857 -12.3211
2.34722 3.21101 0.0157 0.00231 0.00396 0.000242 0.0553 0.0068 0.00495 0.00761 0.00644
3L:22281220-22281370:minus -24.2245 14.0917 -0.981651 6.57798 -17.7143 -18.3776 -7.51376
0.833333 5.79817 0.00763 7.62e-06 0.0159 0.00167 0.00627 0.0125 0.0019 0.0128 0.00208
3L:22281220-22281370:plus -23.5204 15 3.63303 0.00917431 -18.7143 -18.3061 -19.4862
6.54167 1.01835 0.00645 2.04e-06 0.00311 0.0269 0.0136 0.0114 0.0189 0.00127 0.0142
3L:22281680-22281830:minus -31 0.165138 1.22936 3.61468 -19.051 -9.7551 -18.6606 0.625 3.20183
0.0105 0.00974 0.00763 0.0068 0.0162 0.00358 0.0165 0.0137 0.0065
3L:22281680-22281830:plus -22.2653 13.4592 -1.18349 0.522936 -8.37755 -26.5612

-7.87156 3.41667 11.3853 0.00331 0.000064 0.0169 0.0234 0.00106 0.0237 0.00205 0.00508 0.0000815
3L:22358900-22359050:minus -22.7347 2.37615 3.22018 2.24771 -17.7143 -0.0918367 -5.86239
6.65278 4.56881 0.00403 0.00437 0.00365 0.0127 0.00627 0.000442 0.00126 0.0012 0.00356
3L:22358900-22359050:plus -33.0102 -1.00917 3.7156 5.22018 -17.6429 -7.5 -17.5505
0.805556 6.25688 0.0259 0.0144 0.00301 0.00418 0.00562 0.000614 0.014 0.013 0.00163
3L:22359080-22359230:minus -22.7347 2.37615 3.22018 2.24771 -17.7143 -0.0918367 -5.86239
6.65278 4.56881 0.00403 0.00437 0.00365 0.0127 0.00627 0.000442 0.00126 0.0012 0.00356
3L:22359080-22359230:plus -32.4082 1.20183 2.69725 7.10092 -8.89796 -9.53061 -24.5138
0.152778 1.2844 0.0191 0.00673 0.00446 0.00121 0.00189 0.0031 0.0442 0.016 0.0129
3L:22360320-22360470:minus -23.2653 -0.53211 8.6055 1.84404 -16.7143 -18.6429 -11 2.25
4.25688 0.00593 0.0123 0.000314 0.0154 0.00319 0.0139 0.00373 0.00788 0.00391
3L:22360320-22360470:plus -22.7041 6.93578 3.53211 6.00917 -17.7551 -18.3469 -20.6789
3.02778 5.65138 0.00395 0.000671 0.00323 0.00255 0.00658 0.0124 0.0232 0.0059 0.00228
3L:2237140-2237290:minus -22.1837 4.19266 6.88073 4.3211 -17.7551 -16.5 -16.4495 0.222222
-2.6055 0.00315 0.00224 0.000768 0.00514 0.00658 0.00416 0.0117 0.0156 0.04
3L:2237140-2237290:plus -41.398 1.82569 -2.77064 3.59633 -27.8265 0.0510204 -15.1651 1.86111
1.01835 0.0554 0.00535 0.027 0.00693 0.0388 0.000349 0.00927 0.00905 0.0142
3L:22394700-22394850:minus -33.7755 0.449541 0.761468 3.46789 -18.4898 -27.7041
-6.98165 11.2917 5.34862 0.0297 0.00882 0.00897 0.00726 0.0116 0.0445 0.00168 0.0000594 0.00271
3L:22394700-22394850:plus -23.0408 9.41284 11.5963 5.22018 0.612245 -8.53061 -15.6789
5.36111 3.46789 0.00512 0.000181 0.0000561 0.00418 0.000269 0.00136 0.0102 0.00223 0.00582
3L:22400960-22401110:minus -20.102 -2.15596 -3.73394 7.17431 -17.4184 -9.0102 -9.45872
-1.27778 -3.3945 0.00164 0.0203 0.0353 0.00115 0.00457 0.00208 0.00278 0.0244 0.0484
3L:22400960-22401110:plus -34.3265 5.06422 -2.45872 1.87156 -26.2347 -8.5 -1.76147
1.26389 1.95413 0.0317 0.00159 0.0247 0.0152 0.0177 0.00126 0.000421 0.0111 0.00964
3L:22401340-22401490:minus -31.9592 6.53211 2.14679 -1.54128 -18.7551 -17.4184 -18.1284
9.45833 1.16514 0.0167 0.000822 0.00547 0.0439 0.0144 0.00585 0.0152 0.000238 0.0135
3L:22401340-22401490:plus -22.8469 2.29358 4.20183 5.45872 -19.051 -17.7857 -11.1193 -0.680556
2.16514 0.00424 0.00451 0.00247 0.00363 0.0162 0.0068 0.00382 0.0206 0.00879
3L:224040-224190:minus -23.8878 -3.10092 3.37615 6.11009 -18.4898 -18.2755 -21.156 0.708333
0.431193 0.00692 0.0265 0.00343 0.00233 0.0116 0.0112 0.0253 0.0134 0.0164
3L:224040-224190:plus -23.1122 0.954128 3.42202 6.49541 -27.2653 -18.2347 -16.5688
-1.80556 3.62385 0.0053 0.00736 0.00337 0.00176 0.0302 0.0104 0.012 0.0283 0.00523
3L:224720-224870:minus -31.9694 1.83486 4.72477 -0.807339 -8.86735 -8.72449 -18.7523
-4.18056 -6.70642 0.0168 0.00533 0.00199 0.0345 0.00173 0.00169 0.0168 0.0513 0.106
3L:224720-224870:plus -21.5102 3.48624 8.47706 7.55963 -27.2347 -19.2755 -4.6789 9.18056 2.11009
0.00202 0.00291 0.000338 0.000807 0.0283 0.0185 0.000906 0.000285 0.00898
3L:2257040-2257190:minus -41.1837 -2.73394 -0.201835 3.04587 -27.5306 -8.42857
-7.37615 -2.65278 -5.80734 0.0485 0.0239 0.0124 0.00883 0.0351 0.00104 0.00184 0.0354 0.0853
3L:2257040-2257190:plus -15.0816 0.137615 3.11009 0.651376 -28 -26.602 -14.4312 5.875
11.3853 0.00163 0.00984 0.00381 0.0226 0.0444 0.0248 0.00797 0.00176 0.0000815
3L:2257440-2257590:minus -32.1122 3.00917 0.348624 2.19266 -17.3061 -17.9388 -10.0826
-0.236111 2.94495 0.0178 0.00347 0.0103 0.0131 0.00389 0.00738 0.00313 0.018 0.00714
3L:2257440-2257590:plus -24.1837 -4.33945 -2.07339 2.75229 -8.37755 -28.602 -14.5963
-5.72222 -1 0.00751 0.037 0.0221 0.0101 0.00106 0.0648 0.00826 0.0719 0.0261
3L:2262700-2262850:minus -29.949 3.55046 0.513761 4.22936 20.4694 -9.53061 -15.8807 4.91667
7.45872 0.00854 0.00284 0.00977 0.00525 1.65e-07 0.0031 0.0106 0.00272 0.00103
3L:2262700-2262850:plus -24.2959 -0.247706 13.8991 11.2936 -18.7857 -19.0816 -14.4771
3.38889 -0.201835 0.00781 0.0112 8.13e-06 0.0000139 0.0148 0.017 0.00805 0.00513 0.0199
3L:2265760-2265910:minus -23.1837 8.57798 -1.6422 1.77982 -18.1122 -18.7857 -15.2385
5.01389 4.88073 0.00553 0.00028 0.0194 0.0158 0.00771 0.0146 0.00941 0.00261 0.00324
3L:2265760-2265910:plus -13.5918 1.94495 0.40367 9.3211 -17.4184 0.316327 -7.52294 4.91667
7.63303 0.00099 0.00512 0.0101 0.000391 0.00457 0.000243 0.0019 0.00272 0.000928
3L:22710320-22710470:minus -21.9286 -2 1.77982 3.47706 -18.7143 -27.5612 -6.29358

3.56944 5.38532 0.0027 0.0194 0.00627 0.00724 0.0136 0.0393 0.00142 0.00478 0.00267
3L:22710320-22710470:plus -40.449 5.66055 -3.02752 4.86239 -27.4184 -8.72449 -24.5505 3.625
-0.59633 0.0402 0.00123 0.0291 0.00449 0.0323 0.00169 0.0444 0.00468 0.0229
3L:22710920-22711070:minus -23.2245 7.70642 -0.844037 3.00917 -17.7143 -0.316327 -16.7431
2.94444 -2.04587 0.00576 0.000447 0.0152 0.00894 0.00627 0.000524 0.0123 0.00609 0.0336
3L:22710920-22711070:plus -31.5102 1.02752 -0.889908 0.770642 -8.7449 -18.6327 -8.33945 1.51389
3.41284 0.013 0.00717 0.0154 0.0219 0.00161 0.0137 0.00225 0.0102 0.006
3L:22717280-22717430:minus -13.6633 13.5306 -1.92661 5.86239 -27.4592 -17.3469 -13.6972 0.611111 -2
0.00101 0.0000206 0.0212 0.00282 0.0329 0.0058 0.00678 0.0138 0.0332
3L:22717280-22717430:plus -41.2857 5.88073 2 3.69725 -27.2653 -18.0102 -11.1468 -0.361111 -0.889908
0.0521 0.00112 0.00578 0.00665 0.0302 0.00812 0.00385 0.0187 0.0253
3L:22721120-22721270:minus -13.1837 -1.07339 -2.18349 1.92661 -9.23469 9.30612 -20.2385 -4.22222
11.3853 0.000705 0.0147 0.0228 0.0148 0.00277 0.0000398 0.0215 0.0518 0.0000815
3L:22721120-22721270:plus -32.1837 4.06422 -1.22018 7.63303 -17.3367 -26.602 -12.3945 -9.26389 3.08257
0.0181 0.00235 0.0171 0.000745 0.00391 0.0248 0.00504 0.137 0.00681
3L:22734740-22734890:minus -24.4082 0.788991 13.7431 9.44954 -18.9796 -8.5 -12.5229 3.55556 -0.46789
0.00797 0.00781 0.0000103 0.000326 0.0155 0.00126 0.00518 0.00481 0.0218
3L:22734740-22734890:plus -11.7755 0.0550459 -5.15596 9.87156 -8.67347 0.0204082 -4.36697 2.30556
2.73394 0.000266 0.0101 0.0514 0.000195 0.00154 0.000377 0.000828 0.00772 0.00756
3L:22735220-22735370:minus -22.4388 -5.30275 2 3.21101 0.622449 -27.5612 -19.156 -3.08333 -0.981651
0.00348 0.0478 0.00578 0.00822 0.000242 0.0393 0.0179 0.0395 0.026
3L:22735220-22735370:plus -13.2551 3.45872 6.94495 2.6422 -18.3469 -18.0816 -5.88991 2.33333 9.56881
0.000751 0.00294 0.000746 0.0107 0.00894 0.00934 0.00127 0.00765 0.000303
3L:22766480-22766630:minus -31.2653 3.68367 9.22018 4.17431 -26.2347 -9.72449 -20.9633 -1.83333
-3.72477 0.0113 0.00144 0.000223 0.00537 0.0177 0.00352 0.0244 0.0285 0.0519
3L:22766480-22766630:plus -30.4388 -0.614679 2.87156 9.92661 -17.3776 0.0918367 -4.86239 -0.847222
-1.27523 0.00923 0.0127 0.00417 0.000169 0.00419 0.000323 0.000955 0.0216 0.0282
3L:22767540-22767690:minus -33.2245 0.174312 -2.33028 3.14679 -18.1531 -18.3061 -22.5413 1.625
-4.55046 0.0276 0.00971 0.0238 0.00847 0.00829 0.0114 0.0324 0.00983 0.0646
3L:22767540-22767690:plus -21.6633 1.05505 1.3211 1.06422 -18.6837 -9.72449 -8.3945 3.06944 9.97248
0.00223 0.00709 0.00738 0.0203 0.0126 0.00352 0.00228 0.00581 0.00016
3L:22781180-22781330:minus -29.7347 -2.50459 2.38532 5.6789 -17.449 -18.5714 -20.422 2.09722
-4.44037 0.00849 0.0225 0.00501 0.00315 0.00484 0.0136 0.0222 0.00832 0.063
3L:22781180-22781330:plus -21.9694 -0.642202 3.61468 -2.70642 -28.8673 -19.3469 -24.4679 1.27778
8.11009 0.00282 0.0128 0.00313 0.0641 0.0652 0.0194 0.0439 0.0111 0.000597
3L:22783760-22783910:minus -33.1122 4.6055 3.79817 -0.137615 -28.1939 -18.0816 -14.9083 3.16667
1.74312 0.0271 0.00191 0.00291 0.0279 0.0482 0.00934 0.00881 0.0056 0.0105
3L:22783760-22783910:plus -32.8571 -0.477064 3.33028 2.33945 -9.19388 -9.38776 -25.3578 2.47222
-6.62385 0.0244 0.0121 0.00349 0.0122 0.00253 0.00266 0.0494 0.00727 0.104
3L:22815680-22815830:minus -31.4082 2.70642 2.00917 5.47706 -19.051 -17.9796 -23.633 1.52778
-2.54128 0.0119 0.00387 0.00576 0.0036 0.0162 0.00764 0.0388 0.0102 0.0393
3L:22815680-22815830:plus -22.8163 0.633028 -2.3578 -1.80734 -26.7041 -27.2653 -23.0642 -3.18056 12.2752
0.00418 0.00826 0.024 0.0479 0.0193 0.0333 0.0355 0.0404 0.0000141
3L:22816400-22816550:minus -21.7041 -3.55046 0.137615 5.06422 -18.1939 -7.94898 -26.6789 -0.0138889
4.19266 0.00231 0.03 0.0111 0.00433 0.0086 0.00074 0.0581 0.0168 0.00401
3L:22816400-22816550:plus -40.4898 -3.58716 0.0458716 6.12844 -26.6735 -17.9796 -26.844 -1.55556
-3.15596 0.0407 0.0303 0.0114 0.00231 0.0191 0.00764 0.0593 0.0264 0.0464
3L:22816980-22817130:minus -40.1429 1.63303 2.3211 2.83486 -18.4898 -18.5714 -20.7156 3.47222
5.58716 0.0363 0.00575 0.00513 0.0097 0.0116 0.0136 0.0234 0.00497 0.00237
3L:22816980-22817130:plus -32.2857 0.440367 5.33028 0.229358 -19.0102 -28.4082 -18.5046 8.91667 6.63303
0.0185 0.00885 0.00155 0.0252 0.0157 0.0615 0.0161 0.000338 0.00149
3L:22817940-22818090:minus -4.4898 4.23853 -1.43119 7.12844 -8.96939 -17.2755 -19.8624 -7.80556
5.58716 0.000154 0.0022 0.0182 0.00118 0.00226 0.00544 0.0201 0.107 0.00237
3L:22817940-22818090:plus -30.949 -3.14679 0.733945 7.80734 -17.449 -18.3469 -11.4037 -5.84722 1.63303

0.0104 0.0268 0.00906 0.00063 0.00484 0.0124 0.00406 0.0738 0.0112
3L:22828560-22828710:minus -21.3571 -2.3945 3.65138 9.87156 -26.6327 1.09184 -19.1009 -2.375
-0.220183 0.00194 0.0218 0.00308 0.000195 0.0188 0.000091 0.0177 0.0329 0.0201
3L:22828560-22828710:plus -32.1837 5.27523 4.34862 1.36697 -17.7857 -9.5 -11.4954 4.18056 4.88073
0.0181 0.00145 0.00233 0.0186 0.00676 0.00294 0.00413 0.00373 0.00324
3L:22831040-22831190:minus -31.7041 3.76147 5.49541 3.14679 -18.7551 -9.23469 -11.4404 -5.125
2.14679 0.0146 0.00263 0.00144 0.00847 0.0144 0.00238 0.00409 0.0634 0.00883
3L:22831040-22831190:plus -39.4796 2.51376 -0.458716 5.43119 -7.56122 -27.3367 -9.69725 -2.55556
-6.20183 0.0334 0.00415 0.0135 0.00371 0.000419 0.0347 0.00291 0.0345 0.0934
3L:22831540-22831690:minus -31.7041 3.42202 -0.422018 4.06422 -17.3776 -19.3061 -15.0734 2.65278
4.11927 0.0146 0.00298 0.0133 0.00564 0.00419 0.0187 0.00911 0.0068 0.00411
3L:22831540-22831690:plus -22 -3.44954 0.880734 2.33028 -25.9694 -36.9286 -10.6422 5.08333 7.63303
0.00291 0.0292 0.00861 0.0123 0.017 0.123 0.00348 0.00253 0.000928
3L:22856840-22856990:minus -14.1837 5.36697 0.458716 5.22936 -27.5306 -17.7857 -20.4312 6.97222 3
0.00124 0.0014 0.00995 0.0041 0.0351 0.0068 0.0222 0.00102 0.00702
3L:22856840-22856990:plus -20.9184 -2.86239 5.14679 7.33028 -26.9286 -16.9388 -6.80734 0.0833333
3.88073 0.00181 0.0248 0.00167 0.00103 0.0214 0.00433 0.00161 0.0163 0.00475
3L:22857820-22857970:minus -23.6224 -0.330275 14.4404 4.17431 -8.33673 -7.5 -18.3119 0.361111
-2.10092 0.00658 0.0115 4.12e-06 0.00537 0.00101 0.000614 0.0157 0.0149 0.0342
3L:22857820-22857970:plus -21.4388 -2.07339 1.24771 3.33028 -8.56122 -18.7143 -15.3303 -2.08333 3.21101
0.002 0.0198 0.00758 0.00778 0.00112 0.014 0.00958 0.0305 0.00644
3L:22858100-22858250:minus -39.7653 -2.20183 2.3211 0.0550459 -27.3367 -18.2755 -15.9541 6.05556
6.44954 0.0339 0.0206 0.00513 0.0265 0.0317 0.0112 0.0108 0.00161 0.00154
3L:22858100-22858250:plus -32.8878 -1.82569 -2.15596 1.57798 -8.97959 -9.82653 -12.0183 5.59722 1.69725
0.0247 0.0185 0.0226 0.0171 0.0023 0.00381 0.00463 0.002 0.0108
3L:22861320-22861470:minus -20.9592 2.18349 5.31193 5.30275 -9.23469 -8.45918 -22 -0.805556
-2.44037 0.00184 0.00469 0.00156 0.00396 0.00277 0.00111 0.0295 0.0213 0.0381
3L:22861320-22861470:plus -32.449 3.64286 2.91743 0.53211 -27.2347 -9.45918 -11.8624 8.41667 1.90826
0.0195 0.00159 0.0041 0.0234 0.0283 0.00279 0.00447 0.000458 0.00974
3L:22863460-22863610:minus -31.6633 -3.05505 1.19266 2.06422 -27.2653 -26.4898 -11.7706 -0.763889
5.22936 0.0141 0.0262 0.00773 0.0139 0.0302 0.0225 0.00439 0.0211 0.00288
3L:22863460-22863610:plus -11.6224 13.5306 6.44954 2.50459 -18.3776 -9.72449 -10.7523 -2.77778 -2.90826
0.000259 0.0000206 0.000941 0.0114 0.00926 0.00352 0.00355 0.0365 0.0435
3L:22864180-22864330:minus -31.8061 6.33028 10.2661 7.2844 -18.6735 -17.0816 -9.49541 -7.81944
1.33028 0.0158 0.000904 0.000122 0.00105 0.0123 0.00487 0.0028 0.108 0.0127
3L:22864180-22864330:plus -30.398 -1.9633 2.50459 5.34862 -17.7143 -18.3469 -6.13761 -3.79167 2.99083
0.00901 0.0192 0.0048 0.00387 0.00627 0.0124 0.00136 0.0469 0.00706
3L:22864640-22864790:minus -14.2245 13.4954 -0.825688 5.92661 -27.4184 -8.42857 -21.7156 -0.388889
0.614679 0.00126 0.0000144 0.0151 0.00266 0.0323 0.00104 0.028 0.0188 0.0158
3L:22864640-22864790:plus -21.3265 -1.3945 2.02752 -0.0366972 -7.96939 -18.7857 -8.77982 -0.597222
1.23853 0.00193 0.0162 0.00572 0.0271 0.000785 0.0146 0.00245 0.0201 0.0132
3L:22865160-22865310:minus -22.9184 2.86239 0.642202 6.21101 -17.4184 -8.20408 -18.9908 0.763889
-0.0733945 0.00447 0.00366 0.00935 0.00213 0.00457 0.000796 0.0174 0.0131 0.0188
3L:22865160-22865310:plus -23.449 13.4592 3.16514 3.18349 -8.89796 -18.0816 -15.3028 2.73611 -4.3945
0.00631 0.000064 0.00373 0.00837 0.00189 0.00934 0.00953 0.00659 0.062
3L:22871220-22871370:minus -30.4388 1.44037 5.25688 -0.0183486 -18.0816 -17.0102 -7.91743 0.0555556
-5.11927 0.00923 0.00617 0.0016 0.027 0.00759 0.00453 0.00207 0.0164 0.0746
3L:22871220-22871370:plus -29.3673 1.9633 1.3945 4.33028 -8.33673 -17.4184 -12.6972 4.25 5.12844
0.00838 0.00509 0.00719 0.00511 0.00101 0.00585 0.00539 0.00362 0.00301
3L:22871740-22871890:minus -39.9184 2.51376 6.69725 8.80734 -19.051 -9.30612 -20.4404 2.40278
0.642202 0.0344 0.00415 0.000836 0.000518 0.0162 0.00257 0.0222 0.00746 0.0157
3L:22871740-22871890:plus -33.2653 0.449541 7.16514 4.3211 -8.16327 -27.3367 -20.6606 -6.27778 5.18349
0.0277 0.00882 0.000671 0.00514 0.000873 0.0347 0.0231 0.0805 0.00295
3L:22872200-22872350:minus -31.7755 -1.85321 -3 1.14679 -8.37755 -27.051 -16.945 3.79167 5.75229

0.0157 0.0186 0.0288 0.0198 0.00106 0.0315 0.0127 0.00437 0.00213
3L:22872200-22872350:plus -31.5918 3.10092 -2.88073 2.09174 0.877551 -10.0204 -28.0275 6.06944 -3.62385
0.0136 0.00335 0.0279 0.0137 0.00019 0.00399 0.069 0.0016 0.051
3L:22902300-22902450:minus -22.3265 3.68367 2.65138 5.15596 -17.7551 -27.1224 -20.367 1.43056
1.33028 0.00337 0.00144 0.00453 0.00426 0.00658 0.0325 0.022 0.0105 0.0127
3L:22902300-22902450:plus -40.102 -0.0366972 4.66972 2.19266 -27.3776 -18.3469 -11.3853 1.98611
5.70642 0.036 0.0104 0.00204 0.0131 0.032 0.0124 0.00404 0.00866 0.00222
3L:22902640-22902790:minus -14.3367 -0.944954 7.40367 3.61468 -8.64286 -7.57143 -13.0092 -2.88889
-0.706422 0.00131 0.0141 0.000595 0.0068 0.00139 0.000644 0.00579 0.0376 0.024
3L:22902640-22902790:plus -21.8878 -0.486239 1.74312 3.59633 -17.449 -0.204082 -22.1009 5.09722
3.05505 0.00258 0.0121 0.00635 0.00693 0.00484 0.000463 0.03 0.00251 0.00692
3L:22903440-22903590:minus -14.3673 -1.3211 4.00917 2.09174 -28 -8.72449 -14.4587 4.94444 10.0367
0.00132 0.0159 0.00267 0.0137 0.0444 0.00169 0.00802 0.00269 0.000146
3L:22903440-22903590:plus -13.7857 3.05505 2.56881 2.04587 -17.9796 -17.8571 -16.9174 -0.166667
-0.761468 0.00108 0.00341 0.00468 0.014 0.00726 0.00719 0.0126 0.0176 0.0244
3L:22933380-22933530:minus -31.3265 0.477064 3.72477 5.6789 -26.9694 -9.7551 -24.2202 11.2778 9
0.0114 0.00873 0.003 0.00315 0.023 0.00358 0.0424 0.0000601 0.000437
3L:22933380-22933530:plus -32.7347 3.59633 2.51376 1.76147 -27.5612 -9.53061 -14.1743 1 -0.669725
0.0225 0.00279 0.00478 0.0159 0.0354 0.0031 0.00754 0.0121 0.0236
3L:22936680-22936830:minus -22.9592 8.22018 2.51376 5.36697 -18.3776 -27.1122 -6.49541 5.44444
7.22018 0.00475 0.000338 0.00478 0.00386 0.00926 0.0316 0.00149 0.00215 0.00119
3L:22936680-22936830:plus -23 5.43119 1.00917 5.2844 -17.8163 1.68367 -14.2294 3.01389 -2.22018
0.00498 0.00136 0.00824 0.004 0.00682 0.0000524 0.00763 0.00593 0.0355
3L:22938320-22938470:minus -31.2143 3.30275 -1.58716 2.44037 -17.1837 -36.1122 -10.0826 0.638889
5.66055 0.0111 0.00311 0.0191 0.0116 0.00366 0.0878 0.00313 0.0137 0.00227
3L:22938320-22938470:plus -22.898 3.15596 0.229358 1.77982 -18.4898 -17.9388 -13.0734 4.56944 2.75229
0.00442 0.00328 0.0107 0.0158 0.0116 0.00738 0.00588 0.00317 0.00748
3L:22938980-22939130:minus -13.0408 1.85321 5.0367 0.770642 -8.71429 -27.5306 -14.7615 -4.70833
7.94495 0.000576 0.0053 0.00175 0.0219 0.00159 0.0374 0.00855 0.0579 0.000667
3L:22938980-22939130:plus 4.62245 -3.21101 -0.119266 6.47706 -7.89796 -10.0204 -8.11009 3.22222
-3.08257 0.0000219 0.0273 0.0121 0.00179 0.000678 0.00399 0.00215 0.00548 0.0455
3L:22940260-22940410:minus -33.2041 2.3578 4.77982 2.31193 -26.2653 -17.051 -6.57798 -4.72222
1.90826 0.0274 0.0044 0.00195 0.0123 0.018 0.00479 0.00152 0.058 0.00974
3L:22940260-22940410:plus -33.0816 -3.3578 3.76147 9.23853 -18.1122 -17.2755 -26.0734 3.40278 5.90826
0.0268 0.0284 0.00295 0.000424 0.00771 0.00544 0.054 0.00511 0.00198
3L:22940560-22940710:minus -23.4898 1.91743 3.63303 0.834862 -18.7143 -8.02041 -7.69725 2.95833
1.26606 0.00639 0.00518 0.00311 0.0215 0.0136 0.000761 0.00197 0.00606 0.013
3L:22940560-22940710:plus -32.7755 1.6789 2.91743 3.23853 -9.27551 -0.0510204 -14.9358 3.44444
-4.07339 0.0234 0.00565 0.0041 0.00815 0.00284 0.000429 0.00886 0.00502 0.0566
3L:22941220-22941370:minus -32.0102 3.81651 0.889908 5.85321 -16.4898 -8.42857 -18.1927 -2.20833
-0.522936 0.0172 0.00258 0.00859 0.00287 0.00312 0.00104 0.0154 0.0315 0.0222
3L:22941220-22941370:plus -32.5102 0.458716 0.376147 2.29358 -18.449 -27.898 -13.9541 -0.277778
1.36697 0.0202 0.00879 0.0102 0.0124 0.0107 0.0537 0.00718 0.0182 0.0124
3L:23088580-23088730:minus -21.9694 4.05102 -1.12844 6.09174 -27.9388 -16.9796 -12.4587 -3.34722
3.52294 0.00282 0.000335 0.0166 0.00235 0.0415 0.0044 0.00511 0.0421 0.00558
3L:23088580-23088730:plus -21.6224 5.21101 0.770642 1.79817 -27.1939 -17.2755 -13.945 3.95833 -0.422018
0.00213 0.00149 0.00895 0.0157 0.0267 0.00544 0.00717 0.00409 0.0213
3L:23092660-23092810:minus -42.0816 -2.47706 4.25688 2.24771 -8.89796 -8.72449 -20.6055 1.88889
-1.22936 0.0694 0.0223 0.00242 0.0127 0.00189 0.00169 0.0229 0.00896 0.0279
3L:23092660-23092810:plus -22.2653 -1.0367 -0.651376 3.7156 -8.93878 -8.42857 -27.8532 1.40278
1.89908 0.00331 0.0145 0.0143 0.00655 0.00216 0.00104 0.0675 0.0106 0.0098
3L:23092920-23093070:minus -39.2959 -3.36697 -3.11927 1.41284 -18.4592 -8.94898 -18.1743 3.54167
1.70642 0.0331 0.0285 0.0298 0.0183 0.0109 0.00203 0.0154 0.00483 0.0108
3L:23092920-23093070:plus -23.1837 -1.89908 -0.963303 1.87156 -17.1939 -0.173469 -13.4862 7.66667

-0.761468 0.00553 0.0189 0.0158 0.0152 0.00377 0.000456 0.00646 0.000704 0.0244
3L:23119040-23119190:minus -21.551 7.06422 3.02752 3.76147 -18.1224 0.72449 -24.9725 1.25 -0.807339
0.00205 0.000628 0.00393 0.00645 0.00782 0.000175 0.047 0.0112 0.0247
3L:23119040-23119190:plus -31.551 1.65138 2.93578 6.83486 10.3571 -8.93878 -19.7248 0.916667 1.95413
0.0134 0.00571 0.00407 0.00142 0.0000208 0.00201 0.0196 0.0125 0.00964
3L:23143500-23143650:minus -31.7347 -0.311927 4.19266 5.57798 -16.449 -18.2347 -20.7064 5.40278
-6.11927 0.0149 0.0115 0.00248 0.0034 0.00307 0.0104 0.0233 0.00219 0.0914
3L:23143500-23143650:plus -33.7347 0.522936 0.211009 2.09174 -9.16327 -18.051 -17.7798 4.86111 -0.59633
0.0294 0.00859 0.0108 0.0137 0.00249 0.00886 0.0145 0.00279 0.0229
3L:23177220-23177370:minus -24.602 15.1376 -0.174312 4.11009 -18.1939 -16.7857 -3.57798 4.98611
5.3945 0.00822 1.81e-06 0.0123 0.00553 0.0086 0.00426 0.000663 0.00264 0.00263
3L:23177220-23177370:plus -24.1837 7.20183 3.70642 5.46789 -17.9286 -7.5 -6.23853 -1.83333 -1.22018
0.00751 0.000584 0.00302 0.00362 0.0071 0.000614 0.0014 0.0285 0.0278
3L:23177740-23177890:minus -31.9592 0.513761 7.6789 2.92661 -16.8163 -9.86735 -19.6881 -4.15278
-2.59633 0.0167 0.00862 0.000518 0.00931 0.00323 0.00391 0.0195 0.051 0.0398
3L:23177740-23177890:plus -13.1122 -4.48624 7.43119 3.48624 -18.8265 -27.6735 -6.70642 -4.125 9.66055
0.000632 0.0385 0.000587 0.0072 0.0149 0.0443 0.00157 0.0507 0.000268
3L:23242260-23242410:minus -22.2347 -0.0550459 3.62385 8.88073 -19.051 -9.60204 -34.211 7.01389
0.715596 0.00325 0.0105 0.00312 0.000479 0.0162 0.00334 0.149 0.000998 0.0154
3L:23242260-23242410:plus -23.1837 4.84404 -1.40367 3.59633 -18.1939 -17.7857 -9.90826 2.75 -0.284404
0.00553 0.00174 0.0181 0.00693 0.0086 0.0068 0.00303 0.00656 0.0207
3L:23331140-23331290:minus -30.4082 8.92661 2.01835 5.56881 -18.8265 -18.051 -21.9358 5.31944
1.53211 0.00908 0.000233 0.00574 0.00342 0.0149 0.00886 0.0291 0.00227 0.0116
3L:23331140-23331290:plus -33.9694 -1.40367 -4.06422 5.73394 -26.1939 0.826531 -22.6514 5.23611 -2.36697
0.0305 0.0163 0.0386 0.00307 0.0175 0.000153 0.0331 0.00236 0.0371
3L:23332320-23332470:minus -22.9592 2.83486 6.66055 3.47706 -18.051 -9.53061 -14.7248 4.76389
-0.504587 0.00475 0.0037 0.000851 0.00724 0.00746 0.0031 0.00848 0.00291 0.0221
3L:23332320-23332470:plus -24.1837 10.1651 0.201835 2.14679 -18.4898 1.05102 -20.2936 4.01389 -1.88991
0.00751 0.000123 0.0108 0.0133 0.0116 0.000107 0.0217 0.004 0.0322
3L:23666920-23667070:minus -24.1837 13.3878 -2.89908 3.7156 -27.5306 -26.898 -15.6239 2.15278
5.70642 0.00751 0.0000869 0.028 0.00655 0.0351 0.0302 0.0101 0.00816 0.00222
3L:23666920-23667070:plus -32.2245 2.10092 0.706422 2.23853 -27.4184 -8.5 -17.578 -1.36111 -6.57798
0.0183 0.00484 0.00914 0.0128 0.0323 0.00126 0.014 0.025 0.103
3L:23679460-23679610:minus -13.8163 4.08257 1.33945 1.41284 -27.5306 -8.57143 -9.24771 0.236111
1.95413 0.00109 0.00233 0.00733 0.0183 0.0351 0.00149 0.00268 0.0155 0.00964
3L:23679460-23679610:plus -32.0408 13.3878 -0.201835 0.486239 -18.4184 -8.53061 -10.1101 -1.06944
4.55963 0.0174 0.0000869 0.0124 0.0237 0.0101 0.00136 0.00314 0.023 0.00357
3L:23688220-23688370:minus -33.1122 -0.174312 3.51376 1.69725 -18.9796 -19.3469 -24.8073 4.20833
-2.06422 0.0271 0.0109 0.00325 0.0163 0.0155 0.0194 0.046 0.00369 0.0339
3L:23688220-23688370:plus -30.9694 2.36697 0.431193 9.59633 -17.7857 -18.3776 -13.0734 2.98611 -1.3578
0.0104 0.00439 0.01 0.000275 0.00676 0.0125 0.00588 0.006 0.0286
3L:23689080-23689230:minus -32.7449 -1.86239 0.981651 2.34862 -19.0918 0.908163 -29.4495 4.94444
-3.22018 0.0228 0.0187 0.00832 0.0121 0.0163 0.000143 0.0841 0.00269 0.0469
3L:23689080-23689230:plus -21.8571 -4.34862 -1.29358 3.13761 -27.2347 -18.3469 -20.9725 2.13889 -1.97248
0.00249 0.0371 0.0175 0.00854 0.0283 0.0124 0.0245 0.0082 0.033
3L:23689640-23689790:minus -39.8776 0.229358 3.27523 -0.522936 11.6939 0.530612 -11.9725 8.70833
7.04587 0.0341 0.00953 0.00357 0.0313 1.17e-06 0.000203 0.00458 0.000384 0.00132
3L:23689640-23689790:plus -32.5102 -0.623853 4.52294 3.27523 -18.7449 -28.7041 -8.93578 4.5 -0.862385
0.0202 0.0127 0.00217 0.00796 0.0138 0.067 0.00253 0.00326 0.0251
3L:23695320-23695470:minus -30.7041 -0.623853 5.18349 0.486239 -8.67347 -9.02041 -1.51376 3.66667
-0.862385 0.00993 0.0127 0.00165 0.0237 0.00154 0.00213 0.000398 0.0046 0.0251
3L:23695320-23695470:plus -31.7041 -0.733945 -0.844037 5.06422 -27.4184 -0.397959 -21.6789
6.80556 -2.43119 0.0146 0.0132 0.0152 0.00433 0.0323 0.000525 0.0278 0.00111 0.038
3L:23708420-23708630:minus -21.9694 1.31193 2.43119 3.87156 -8.89796 -17.7551 -15.2477 7.01389

-0.844037 0.00282 0.00646 0.00493 0.00618 0.00189 0.00643 0.00943 0.000998 0.025
3L:23708420-23708630:plus -23.5918 1.37615 3.34862 6.29358 -8.90816 -8.57143 -1.21101 12.4722 -2.48624
0.00656 0.00631 0.00347 0.00202 0.00192 0.00149 0.000372 0.0000191 0.0387
3L:23710020-23710170:minus -24.551 -0.541284 2.3578 2.55963 -18.7143 -7.5 -12.5963 7.20833
3.41284 0.00814 0.0124 0.00506 0.0111 0.0136 0.000614 0.00527 0.000902 0.006
3L:23710020-23710170:plus -13.3776 0.614679 7.54128 1.61468 -8.56122 -17.2755 -21.0183 1.93056 -9.97248
0.000831 0.00832 0.000555 0.0169 0.00112 0.00544 0.0247 0.00883 0.193
3L:2371980-2372130:minus -32.898 -0.944954 1.46789 3.53211 -8.93878 -17.2041 -21.7706 -2.47222
-0.440367 0.0247 0.0141 0.007 0.00704 0.00216 0.00507 0.0283 0.0338 0.0216
3L:2371980-2372130:plus -40.2143 5.23853 1.66972 7.44037 -17.4082 0.0204082 -16.8349 -4.90278
-4.14679 0.0372 0.00148 0.00652 0.000912 0.00421 0.000377 0.0125 0.0604 0.0576
3L:23731240-23731390:minus -32.0714 4.81651 3.33028 0.550459 -17.7143 -8.5 -15.0367 6.66667 -5.22018
0.0176 0.00175 0.00349 0.0233 0.00627 0.00126 0.00904 0.00119 0.0762
3L:23731240-23731390:plus -30.398 -0.155963 1.94495 -1.24771 -19.051 -18.051 -19.3211 8.20833
-0.541284 0.00901 0.0109 0.0059 0.0398 0.0162 0.00886 0.0184 0.000518 0.0223
3L:2373640-2373790:minus -32.2143 -1.37615 -0.853211 2.31193 -27.6327 0.826531 -10.8257 1.41667
-1.22936 0.0182 0.0161 0.0152 0.0123 0.0369 0.000153 0.00361 0.0106 0.0279
3L:2373640-2373790:plus -31.7449 -4.94495 15.6239 6.90826 -8.30612 -27.1224 -15.3028 6.80556 1.27523
0.0152 0.0435 5.44e-07 0.00133 0.00097 0.0325 0.00953 0.00111 0.013
3L:2377520-2377670:minus -33.2245 0.183486 4.36697 3.19266 -18.4898 -19.0102 -4.3945 -3.79167 3.83486
0.0276 0.00968 0.00231 0.00829 0.0116 0.0159 0.000835 0.0469 0.00481
3L:2377520-2377670:plus -33.2551 0.807339 3.59633 6.54128 -18.1837 -26.3061 -14.7523 0.527778 -1.54128
0.0276 0.00776 0.00315 0.00173 0.00835 0.0219 0.00853 0.0142 0.0296
3L:2387880-2388030:minus -31.9286 3.97959 -0.155963 0.211009 -18.7143 -8.57143 -21.5413 0.402778
-2.87156 0.0166 0.000669 0.0122 0.0254 0.0136 0.00149 0.0271 0.0147 0.043
3L:2387880-2388030:plus -31.3265 -0.155963 5.74312 9.7156 -8.93878 -19.3061 -1.70642 -5.625
1.22018 0.0114 0.0109 0.00129 0.000242 0.00216 0.0187 0.000415 0.0705 0.0133
3L:2392960-2393110:minus -23.7449 1.61468 1.02752 4.37615 -26.6735 -17.7857 -20.578 -2.58333 3.58716
0.00674 0.00578 0.00819 0.00504 0.0191 0.0068 0.0228 0.0348 0.00534
3L:2392960-2393110:plus -30.6327 10.5688 2.02752 1.75229 0.877551 -18.2755 -21.367 0.0277778
-0.0733945 0.00968 0.000101 0.00572 0.0159 0.00019 0.0112 0.0263 0.0166 0.0188
3L:2394460-2394610:minus -31.2143 -3.30275 0.954128 2.55963 -17.1837 -9.53061 -17.7615 4.06944 1.54128
0.0111 0.028 0.0084 0.0111 0.00366 0.0031 0.0144 0.00391 0.0115
3L:2394460-2394610:plus -31.7041 5.47706 2.36697 2.88991 -17.4184 -17.2755 -17.6239 5.65278 0.238532
0.0146 0.00133 0.00505 0.00942 0.00457 0.00544 0.0141 0.00195 0.0173
3L:2400400-2400550:minus -33 3.64286 -0.275229 5.49541 -18.4898 -16.7857 -23.0642 -1.30556 1.44037
0.0258 0.00159 0.0127 0.00355 0.0116 0.00426 0.0355 0.0246 0.012
3L:2400400-2400550:plus -14.4388 5.6055 -0.779817 9.76147 -18.9388 -17.7857 0.366972 3 7.16514
0.00137 0.00126 0.0149 0.000212 0.0151 0.0068 0.000259 0.00597 0.00122
3L:24021500-24021650:minus -40.8878 -0.990826 -1.80734 2.89908 -27.2347 -18.1224 -11.4954 7.79167
0.66055 0.0439 0.0143 0.0204 0.00938 0.0283 0.00988 0.00413 0.000656 0.0156
3L:24021500-24021650:plus -33.0408 3.11009 -5.83486 4.14679 -18.7143 0.72449 -24.633 2.94444 -6.41284
0.0264 0.00334 0.0608 0.00542 0.0136 0.000175 0.0449 0.00609 0.0983
3L:24022240-24022390:minus -43.1122 -3.95413 -3.76147 -2.58716 -27.4898 -18.5714 -30.5596 4.5 3.46789
0.0897 0.0334 0.0356 0.0617 0.0336 0.0136 0.0983 0.00326 0.00582
3L:24022240-24022390:plus -14.602 -1.72477 4.10092 -2.68807 -18.9388 0.0510204 -31.3303 4.26389
-6.01835 0.00149 0.0179 0.00257 0.0637 0.0151 0.000349 0.109 0.0036 0.0892
3L:24022760-24022910:minus -32.8878 -0.926606 1.01835 6.02752 -17.7857 -9.57143 -12.8349 8.29167
-0.550459 0.0247 0.014 0.00821 0.00246 0.00676 0.00326 0.00557 0.000493 0.0225
3L:24022760-24022910:plus -23.7755 4.53211 3.09174 0.59633 -17.4898 -8.72449 -15.2202 2.73611 8.14679
0.00678 0.00196 0.00383 0.023 0.0053 0.00169 0.00937 0.00659 0.000562
3L:2417840-2417990:minus -22.8061 -5.22018 3.18349 2.51376 -17.5612 -18.8265 -16.156 1.56944 3.44954
0.00414 0.0467 0.0037 0.0113 0.00544 0.0148 0.0111 0.01 0.00594
3L:2417840-2417990:plus -29.1429 3.68807 4.81651 10.0275 -18.3469 0.0918367 -10.2202 0.638889

-0.669725 0.00834 0.0027 0.00192 0.000156 0.00894 0.000323 0.00321 0.0137 0.0236
3L:2418540-2418690:minus -32.8163 -0.366972 0.633028 6.44037 -7.89796 -8.79592 -21.9908 0.25 6.42202
0.024 0.0117 0.00938 0.00185 0.000678 0.0019 0.0294 0.0155 0.00156
3L:2418540-2418690:plus -14.5612 -1.88991 -2.43119 -1.04587 -17.7143 -16.9796 -13.3303 5.97222 2.10092
0.00147 0.0188 0.0245 0.0373 0.00627 0.0044 0.00624 0.00168 0.00901
3L:2428720-2428870:minus -32.5102 1.01835 -2.41284 -0.284404 -18.2245 -35.8878 -10.6239 -1.61111
1.73394 0.0202 0.00719 0.0244 0.0291 0.00877 0.0822 0.00347 0.0268 0.0106
3L:2428720-2428870:plus -22.8878 3.97959 -0.972477 11.1927 -17.9286 -17.2755 -8.63303 -5.51389
1.73394 0.00441 0.000669 0.0158 0.0000254 0.0071 0.00544 0.00239 0.0688 0.0106
3L:24530500-24530650:minus -22.9898 -1.7156 -3.85321 3.44954 -8.89796 -27.5612 -17.1009 6.40278
1.54128 0.00482 0.0179 0.0365 0.00738 0.00189 0.0393 0.013 0.00136 0.0115
3L:24530500-24530650:plus -33.1122 -0.587156 -2.2844 7.51376 -26.2653 -18.6429 -23.1835 5.56944
-7.40367 0.0271 0.0126 0.0235 0.000836 0.018 0.0139 0.0362 0.00203 0.122
3L:2457920-2458070:minus -14.6735 -3.70642 1.6789 2.06422 -16.8571 -28.1837 -8.3578 2.27778 7.6422
0.00152 0.0313 0.0065 0.0139 0.00324 0.0584 0.00226 0.0078 0.000893
3L:2457920-2458070:plus -23.0714 -1.77982 5.31193 6.89908 -17.8878 -9.23469 -8.54128 -3.86111 5.70642
0.0052 0.0182 0.00156 0.00134 0.00704 0.00238 0.00234 0.0477 0.00222
3L:2462640-2462790:minus -34.0714 1.44037 5.22018 1.20183 -18.5204 -26.602 -7.75229 -0.555556 -1
0.031 0.00617 0.00162 0.0195 0.0117 0.0248 0.002 0.0198 0.0261
3L:2462640-2462790:plus -31.4796 -0.376147 3.70642 4.65138 -27.7041 -18.2755 -14.7064 6.22222
3.66972 0.0128 0.0117 0.00302 0.00471 0.038 0.0112 0.00845 0.00149 0.00514
3L:2466720-2466870:minus -23.1939 6.13761 3.54128 2.51376 -19.0204 -17.8265 -12.6789 2.08333 5.23853
0.00558 0.000993 0.00322 0.0113 0.0158 0.00699 0.00537 0.00837 0.00285
3L:2466720-2466870:plus -20.4388 -3.41284 4.30275 2.18349 10.7245 -18.0102 17.6972 12.4583 1.25688
0.00167 0.0289 0.00237 0.0131 5.2e-06 0.00812 5.41e-07 0.0000193 0.0131
3L:246860-247010:minus -22.4796 -1.30275 0.12844 -0.0183486 -18.3776 -17.2755 -19.0734 -0.805556
2.0367 0.00352 0.0158 0.0111 0.027 0.00926 0.00544 0.0177 0.0213 0.00923
3L:246860-247010:plus -13.449 4.65138 -2.66055 4.66972 -8.93878 -9.5 -17.4128 6.81944 2.84404 0.000914
0.00187 0.0262 0.00466 0.00216 0.00294 0.0137 0.0011 0.00736
3L:2478520-2478670:minus -20 2.42202 10.9541 3.50459 -8.64286 -27.6327 -12.945 8.61111 1.36697
0.00164 0.0043 0.0000822 0.00713 0.00139 0.043 0.00571 0.000408 0.0124
3L:2478520-2478670:plus -21.2551 2.77982 2.70642 2.00917 -9.20408 -17.7143 -11.3578 -0.930556
4.80734 0.00191 0.00377 0.00444 0.0142 0.00262 0.00634 0.00402 0.0221 0.00334
3L:2488040-2488190:minus -31.1735 0.53211 7.7156 7.37615 -25.9694 -9.79592 -15.8165 8 5.57798
0.0109 0.00856 0.000509 0.000951 0.017 0.00377 0.0105 0.000583 0.00238
3L:2488040-2488190:plus -40.6735 2.41284 3.68807 2.11927 -18.9796 1.05102 -25.1835 -0.111111
-2.6789 0.0426 0.00431 0.00304 0.0135 0.0155 0.000107 0.0483 0.0173 0.0408
3L:2488680-2488830:minus -40.551 -5.68807 2.9633 -1.46789 -18.1939 -26.8265 -15.1743 1.06944 4.88073
0.0418 0.0527 0.00403 0.0428 0.0086 0.0285 0.00929 0.0119 0.00324
3L:2488680-2488830:plus 5.03061 -1.51376 -2.3211 7.70642 -16.3776 1.2449 -2.72477 1.54167 7.84404
0.0000166 0.0168 0.0237 0.000708 0.003 0.0000797 0.000529 0.0101 0.000701
3L:2489260-2489410:minus -21.8571 -1.25688 2.55046 6.57798 -8.37755 -9.02041 -20.7156 -0.75 2
0.00249 0.0155 0.00471 0.00167 0.00106 0.00213 0.0234 0.021 0.00941
3L:2489260-2489410:plus -33.2245 1.50459 3.31193 1.84404 -17.7857 -17.1224 -17.0367 6.27778 5.38532
0.0276 0.00602 0.00352 0.0154 0.00676 0.00499 0.0129 0.00145 0.00267
3L:2490060-2490210:minus -13.2245 -1.66972 -0.302752 0.899083 -18.3469 -9.42857 -19.2018 2.83333
7.73394 0.000738 0.0176 0.0128 0.0212 0.00894 0.0027 0.018 0.00635 0.000784
3L:2490060-2490210:plus -22.7755 5.59633 4.9633 4.23853 -26.9286 -36.0408 -15.4404 -0.569444
10.3486 0.00412 0.00126 0.00181 0.00522 0.0214 0.0837 0.00978 0.0199 0.0000988
3L:2532340-2532490:minus -14.5612 0.0825688 2.19266 3.01835 -7.89796 -19.3061 -15.0459 9.52778
3.27523 0.00147 0.01 0.00538 0.0089 0.000678 0.0187 0.00906 0.000227 0.00625
3L:2532340-2532490:plus -23.3367 1.24771 5.75229 10.5963 -27.2347 -18.051 -13.1009 5.13889 -4.29358
0.00614 0.00661 0.00129 0.0001 0.0283 0.00886 0.00592 0.00247 0.06
3L:2533400-2533550:minus -31.6224 6.2844 4.06422 9.61468 -25.9286 -18.051 -13.2202 -4.86111 -2.12844

0.0137	0.000926	0.00261	0.000255	0.0168	0.00886	0.00608	0.0598	0.0345
3L:2533400-2533550:plus	-32.4796	-3.62385	-1.51376	2.6422	-27	-28.4082	-12.1376	3.18056	8.0367	0.02
0.0306	0.0187	0.0107	0.0233	0.0615	0.00475	0.00557	0.000654
3L:2534480-2534630:minus	-24.3265	1.47706	4.77064	5.50459	-26.9694	-19.2347	-8.34862	4.01389	5.07339
0.00784	0.00608	0.00196	0.00349	0.023	0.018	0.00226	0.004	0.00305
3L:2534480-2534630:plus	-23.8878	-4.75229	1.3578	2.66972	0.836735	-27.602	-20.5596	-7.88889	-3.76147
0.00692	0.0413	0.00728	0.0106	0.000215	0.0416	0.0227	0.109	0.0523
3L:2554500-2554650:minus	-31.7755	6.20183	0.990826	3.33945	-26.6327	-27.6735	12.9633	5.33333	0.293578
0.0157	0.000962	0.00829	0.00774	0.0188	0.0443	3.53e-06	0.00226	0.017
3L:2554500-2554650:plus	-33.3367	0.504587	0.53211	2.27523	-18.7551	-9.42857	-8.26606	-1.29167	0.917431
0.0281	0.00865	0.00971	0.0125	0.0144	0.0027	0.00222	0.0245	0.0146
3L:2570380-2570530:minus	-22.1122	-1.72477	0.486239	1.94495	-27.6327	-8.5	-22.6972	1.33333	-3.57798
0.00304	0.0179	0.00986	0.0147	0.0369	0.00126	0.0333	0.0109	0.0506
3L:2570380-2570530:plus	-24.2551	3.3945	-1.75229	2.55963	-26.9388	-27.6735	-19.6881	3.13889	3.9633
0.00768	0.00301	0.0201	0.0111	0.0215	0.0443	0.0195	0.00566	0.00446
3L:2570820-2570970:minus	-23.2347	3.0367	0.587156	2.68807	-27.3469	0.0510204	-17.8073	-3.04167
3.23853	0.00577	0.00343	0.00952	0.0104	0.0318	0.000349	0.0145	0.0391	0.00639
3L:2570820-2570970:plus	-32.7449	-5.12844	5.09174	-0.0642202	-19.051	-26.8571	-10.156	7.66667
9.97248	0.0228	0.0456	0.00171	0.0274	0.0162	0.0288	0.00317	0.000704	0.00016
3L:2573140-2573290:minus	-31.7449	5.13761	4.87156	4.20183	-17.7959	-9.37755	-20.7156	-1.125	2.21101
0.0152	0.00154	0.00188	0.00533	0.00678	0.00265	0.0234	0.0234	0.00862
3L:2573140-2573290:plus	-23.8163	0.963303	3.12844	3.51376	-26.1939	-9.08163	-25.1835	11.4306	-0.229358
0.00682	0.00734	0.00378	0.00709	0.0175	0.00216	0.0483	0.0000525	0.0202
3L:2573560-2573710:minus	-32.0816	4.14679	-2.38532	-0.321101	-8.70408	-0.316327	-16.4771	10.8333
6.63303	0.0176	0.00228	0.0242	0.0294	0.00155	0.000524	0.0118	0.0000874	0.00149
3L:2573560-2573710:plus	-22.2245	1.87156	4.44954	8.80734	-17.1531	-26.3367	-25.4037	0.847222	1.9633
0.00325	0.00526	0.00224	0.000518	0.00364	0.0221	0.0497	0.0128	0.00951
3L:2576820-2576970:minus	-30.1429	-4.3945	6.23853	3.86239	0.836735	-26.5306	-6.9633	1.54167	1.61468
0.00866	0.0376	0.00104	0.00622	0.000215	0.023	0.00167	0.0101	0.0112
3L:2576820-2576970:plus	-33.8878	3.49541	0.522936	1.16514	-17.7143	-27.8265	-18.211	3.13889	2.11009
0.0302	0.0029	0.00974	0.0197	0.00627	0.0498	0.0154	0.00566	0.00898
3L:2577340-2577490:minus	-14.2653	-0.376147	-0.522936	6.02752	-17.1122	9.82653	-3.77064	4.02778
0.908257	0.00128	0.0117	0.0137	0.00246	0.00341	0.0000148	0.000699	0.00397	0.0147
3L:2577340-2577490:plus	-32.8878	-4.46789	8.43119	4.65138	-17.449	-18.8571	-11.1651	1.72222	2.78899
0.0247	0.0383	0.000347	0.00471	0.00484	0.0151	0.00386	0.0095	0.00745
3L:2581320-2581470:minus	-21.9286	0.440367	1.0367	5.85321	-8.33673	-18.3469	-20.055	-4.95833	-3.84404
0.0027	0.00885	0.00816	0.00287	0.00101	0.0124	0.0208	0.0611	0.0533
3L:2581320-2581470:plus	-31.7041	2.6789	-1.3578	1.23853	-17.1122	-28.0408	-17	-0.166667	9.3945
0.0146	0.00391	0.0178	0.0193	0.00341	0.0551	0.0128	0.0176	0.000358
3L:258160-258310:minus	-31.7755	5.88991	4.04587	3.14679	-19.051	-9.53061	-7.81651	2.18056	4.0367
0.0157	0.00111	0.00263	0.00847	0.0162	0.0031	0.00203	0.00808	0.00424
3L:258160-258310:plus	-13.4796	14	2.08257	3.23853	-18.7551	0.979592	7.54128	-2.83333	1.68807	0.000935
8.52e-06	0.0056	0.00815	0.0144	0.000136	0.0000269	0.0371	0.0109
3L:2581620-2581770:minus	-4.59184	-2.79817	6.77982	3.89908	-27.2347	-18.8265	-2.82569	-0.986111
11.3853	0.000166	0.0244	0.000804	0.00604	0.0283	0.0148	0.000543	0.0225	0.0000815
3L:2581620-2581770:plus	-12.551	0.0183486	2.80734	7.80734	-27.3061	-18.6327	-13.6789	-2.51389
2.94495	0.000444	0.0102	0.00427	0.00063	0.0315	0.0137	0.00675	0.0341	0.00714
3L:2582900-2583050:minus	-20.6633	7.17431	4.36697	9.59633	-17.8571	0.0918367	-21.9083	0.472222
1.89908	0.00173	0.000593	0.00231	0.000275	0.00698	0.000323	0.029	0.0144	0.0098
3L:2582900-2583050:plus	-24.3367	-2.20183	3.27523	6.57798	-17.6429	-18.1531	-3.61468	3.79167	1.74312
0.00789	0.0206	0.00357	0.00167	0.00562	0.00997	0.00067	0.00437	0.0105
3L:2583260-2583410:minus	-40.8163	1.68807	2.56881	6.83486	-18.7143	-8.53061	-7.45872	2.36111	3.66972
0.0436	0.00563	0.00468	0.00142	0.0136	0.00136	0.00187	0.00757	0.00514
3L:2583260-2583410:plus	-13.551	6.22018	-1	5.45872	-18.2245	-27.5306	-6.62385	-2.09722	8.10092

0.000974 0.000954 0.016 0.00363 0.00877 0.0374 0.00154 0.0306 0.000623
3L:2583920-2584070:minus -12.1122 4.00917 3.05505 2.22936 -18.7959 -7.72449 -10.211 -0.0555556
1.66055 0.000336 0.0024 0.00389 0.0128 0.0148 0.00068 0.0032 0.017 0.011
3L:2583920-2584070:plus -32.3673 2.73394 0.376147 6.00917 -8.53061 -18.2041 -25.9541 1.75 -6.53211
0.0189 0.00383 0.0102 0.00255 0.0011 0.0103 0.0532 0.00941 0.102
3L:2585980-2586130:minus -31.7755 -0.917431 1.69725 4.78899 -26.3673 -9.5 -18.6697 6.26389 1.76147
0.0157 0.014 0.00646 0.00456 0.0182 0.00294 0.0166 0.00146 0.0104
3L:2585980-2586130:plus -30.2551 1.19266 -0.889908 0.93578 -18.4898 -18.5714 -11.5413 4.77778
6.44954 0.00885 0.00675 0.0154 0.0209 0.0116 0.0136 0.00418 0.00289 0.00154
3L:2586200-2586350:minus -32.8469 2.77982 5 3.04587 -18.1224 -27.3367 -9.90826 3.16667 5.13761
0.0243 0.00377 0.00178 0.00883 0.00782 0.0347 0.00303 0.0056 0.00298
3L:2586200-2586350:plus -14.1531 1.80734 0.366972 0.0733945 -26.5918 -16.9796 -9.89908 -1.34722
3.83486 0.00121 0.00539 0.0103 0.0263 0.0186 0.0044 0.00302 0.0249 0.00481
3L:2586500-2586650:minus -24.3776 2.65138 4.80734 9.7156 20.2041 -8.93878 -13.9725 0.277778 0.587156
0.00793 0.00395 0.00193 0.000242 3.64e-07 0.00201 0.00721 0.0153 0.016
3L:2586500-2586650:plus -32.551 5.37615 1.74312 3.44954 -19.0102 -8.30612 -24.1743 3.20833 1.23853
0.0207 0.00139 0.00635 0.00738 0.0157 0.000973 0.0421 0.00551 0.0132
3L:2587380-2587530:minus -22.9286 -1.27523 8.94495 -1 -17.3776 -18.051 -25.5596 -2.41667 8.05505
0.0046 0.0156 0.000262 0.0367 0.00419 0.00886 0.0507 0.0333 0.00064
3L:2587380-2587530:plus -30.4796 -6.18349 -4.43119 2.34862 -8.90816 -8.5 -11.2018 -7.04167 -3.78899
0.00941 0.0598 0.0426 0.0121 0.00192 0.00126 0.00389 0.0933 0.0526
3L:2589320-2589470:minus -34.5306 1.44037 -1.65138 5.48624 -18.051 -8.79592 -20.4037 2.44444 -6.43119
0.0319 0.00617 0.0195 0.00357 0.00746 0.0019 0.0221 0.00734 0.0988
3L:2589320-2589470:plus -22.7755 5.12844 -3.34862 -0.458716 -28.2347 -26.9694 -14.4037 3.36111
2.27523 0.00412 0.00154 0.0318 0.0308 0.051 0.0304 0.00792 0.00519 0.00846
3L:2589760-2589910:minus -13.5204 10.4037 0.504587 7.43119 -26.898 0.316327 -14.422 0.666667 -1.83486
0.00097 0.000109 0.0098 0.000939 0.0203 0.000243 0.00796 0.0135 0.0318
3L:2589760-2589910:plus -30.5204 -3.79817 -2.15596 2.7156 -18.1531 -7.20408 -31.7798 -2.22222 -5.87156
0.00951 0.032 0.0226 0.0102 0.00829 0.000548 0.115 0.0316 0.0865
3L:2590300-2590450:minus -21.6939 -0.697248 9.16514 2.66972 -26.9286 -8.64286 1.75229 1.375
-0.761468 0.00226 0.013 0.000231 0.0106 0.0214 0.00154 0.000178 0.0107 0.0244
3L:2590300-2590450:plus -33.0816 2.15596 -1.90826 -0.348624 -19.3163 0.530612 -29.0826 2.43056
1.2844 0.0268 0.00474 0.021 0.0296 0.0165 0.000203 0.0799 0.00738 0.0129
3L:2590640-2590790:minus -31.7755 -1.29358 2.58716 3.23853 10.3571 -8.68367 -21.4312 7.25 6.76147
0.0157 0.0157 0.00465 0.00815 0.0000208 0.00157 0.0266 0.000882 0.00141
3L:2590640-2590790:plus -13.6735 3.57143 4.2844 6.84404 -18.7551 -26.602 -19.8349 1.18056 1.97248
0.00103 0.00175 0.00239 0.0014 0.0144 0.0248 0.02 0.0114 0.00948
3L:2591460-2591610:minus -13.1939 2.04587 7.36697 6.14679 -9.23469 -26.9694 -20.2661 1.26389 5.3211
0.000709 0.00494 0.000605 0.00224 0.00277 0.0304 0.0216 0.0111 0.00275
3L:2591460-2591610:plus -13.1837 4.93578 -1.95413 7 -17.7143 -17.051 -1.83486 6.625 2.51376
0.000705 0.00167 0.0213 0.00132 0.00627 0.00479 0.000428 0.00122 0.00792
3L:2591940-2592090:minus -24.4898 1.7156 -3.30275 -0.211009 -7.93878 -17.3469 -30.9174 3.25 2.15596
0.00807 0.00557 0.0314 0.0286 0.000767 0.0058 0.103 0.00542 0.00881
3L:2591940-2592090:plus -22.9592 0.110092 0.0275229 6.06422 -17.3776 -18.7857 -13.2936 -0.402778
6.49541 0.00475 0.00993 0.0115 0.00239 0.00419 0.0146 0.00618 0.0189 0.00154
3L:2599520-2599670:minus -21.8878 13.5306 13.9541 7.2844 -17.9796 -9.23469 -13.2936 9.01389 5.9633
0.00258 0.0000206 7.63e-06 0.00105 0.00726 0.00238 0.00618 0.000318 0.00188
3L:2599520-2599670:plus -31.4796 0.550459 0.201835 6.12844 -8.60204 -18.4184 -13.2202 2.29167 3.58716
0.0128 0.00851 0.0108 0.00231 0.00125 0.0127 0.00608 0.00776 0.00534
3L:2600180-2600330:minus -23.2245 1.09174 2.94495 5.73394 -8.86735 -9.53061 -18.2936 6.22222 1.29358
0.00576 0.007 0.00405 0.00307 0.00173 0.0031 0.0156 0.00149 0.0128
3L:2600180-2600330:plus -31.7755 13.5306 15.0092 5.43119 -18.4592 -8.94898 -20.1376 -4.77778 -4.0367
0.0157 0.0000206 1.55e-06 0.00371 0.0109 0.00203 0.0211 0.0587 0.0562
3L:262840-262990:minus -21.8469 13.3878 -3.29358 2.19266 -18.2653 -9.23469 -1.69725 -0.402778

-4.66972 0.00247 0.0000869 0.0313 0.0131 0.00882 0.00238 0.000414 0.0189 0.0669
3L:262840-262990:plus -40.4796 0.119266 6.27523 -0.440367 -26.9694 -0.173469 -17.6881 8.83333
3.09174 0.0407 0.0099 0.00102 0.0306 0.023 0.000456 0.0143 0.000356 0.00678
3L:2629440-2629590:minus -11.7755 0.40367 4.48624 7 -26.9694 -27.2653 -16.4495 -5.875 2.17431
0.000266 0.00896 0.0022 0.00132 0.023 0.0333 0.0117 0.0742 0.00872
3L:2629440-2629590:plus -22.6633 6.56881 6.00917 3.89908 -8.60204 -36.4082 -20.6606 1.58333 7.68807
0.00384 0.000806 0.00115 0.00604 0.00125 0.105 0.0231 0.00997 0.000841
3L:2631480-2631630:minus -22.2245 2.76147 2.34862 3.14679 -17.1939 -18.051 -15.8257 0.0277778
3.72477 0.00325 0.0038 0.00508 0.00847 0.00377 0.00886 0.0105 0.0166 0.00497
3L:2631480-2631630:plus -12.8776 9.05505 5.6789 6.00917 -8.89796 -17.4184 -16.7706 4.91667 -0.541284
0.000507 0.000217 0.00133 0.00255 0.00189 0.00585 0.0124 0.00272 0.0223
3L:2632220-2632370:minus -22.1837 1.54128 1.09174 2.78899 -17.1837 -27.4082 -10.7339 6 1.74312
0.00315 0.00594 0.00801 0.00992 0.00366 0.0356 0.00354 0.00166 0.0105
3L:2632220-2632370:plus -21.6327 2.30275 11.1376 5.94495 -27.9694 -7.72449 -6.91743 4.15278 1.89908
0.00215 0.00449 0.0000731 0.00261 0.0437 0.00068 0.00165 0.00377 0.0098
3L:2632620-2632770:minus -23.8061 -5.3211 4.86239 2.54128 -26.9694 -8.23469 -15 0.722222 -1.11009
0.0068 0.048 0.00188 0.0112 0.023 0.000875 0.00897 0.0133 0.0269
3L:2632620-2632770:plus -22.0714 -1.69725 -0.0825688 -0.201835 -18.2653 -37.2551 -11.6972 2.59722
8.11009 0.00299 0.0178 0.0119 0.0285 0.00882 0.142 0.00432 0.00694 0.000597
3L:2633900-2634050:minus -24.7143 2.29358 0.0275229 2.44037 -18.7551 -9.53061 -24.367 0.75 3.88073
0.00826 0.00451 0.0115 0.0116 0.0144 0.0031 0.0433 0.0132 0.00475
3L:2633900-2634050:plus -23.2245 2.43119 0.853211 4.19266 -19.3163 -9.94898 -19.0734 -0.0416667
-3.29358 0.00576 0.00428 0.0087 0.00536 0.0165 0.00393 0.0177 0.017 0.0474
3L:2634280-2634430:minus -32.6633 -0.577982 -2.3945 -0.146789 -16.7143 -17.3469 -27.2752 4.65278
-3.68807 0.0216 0.0125 0.0242 0.028 0.00319 0.0058 0.0626 0.00305 0.0516
3L:2634280-2634430:plus -41.4898 0.862385 2.31193 7.27523 -26.5306 -8.87755 -27.211 -0.666667
-9.19266 0.0577 0.00761 0.00515 0.00109 0.0185 0.00198 0.0621 0.0205 0.171
3L:2635060-2635210:minus -30.4388 -1.86239 -0.981651 2.09174 -18.3878 0.0510204 -22.8532 1.05556
-3.22018 0.00923 0.0187 0.0159 0.0137 0.00931 0.000349 0.0342 0.0119 0.0469
3L:2635060-2635210:plus -22.5204 1.70642 -2.69725 3.53211 -18.7959 -17.051 -16.6697 2.72222 9.75229
0.00356 0.00559 0.0265 0.00704 0.0148 0.00479 0.0122 0.00663 0.000241
3L:263860-264010:minus -31.398 2.98165 0.926606 1.47706 -8.60204 -27.8265 -7.3945 -1.81944 2.57798
0.0117 0.0035 0.00848 0.0178 0.00125 0.0498 0.00185 0.0284 0.0078
3L:263860-264010:plus -31.551 -0.504587 2.97248 1.16514 -17.4184 -18.1224 -13.0734 -4.5 2.73394
0.0134 0.0122 0.00401 0.0197 0.00457 0.00988 0.00588 0.0552 0.00756
3L:2651900-2652050:minus -34.3061 2.12844 -0.394495 8.98165 20.2041 10.7551 -26.0826 1.875
-7.85321 0.0317 0.00479 0.0132 0.000461 3.64e-07 3.17e-06 0.054 0.00901 0.132
3L:2651900-2652050:plus -23.9592 6.3211 -1.30275 4.02752 20.4694 -7.72449 -18.7339 -0.166667
-1.41284 0.00708 0.000909 0.0175 0.00581 1.65e-07 0.00068 0.0167 0.0176 0.0289
3L:266120-266270:minus -31.1735 0.458716 11.211 10.5963 -16.8571 -17.3469 -13.9083 -2 5.38532
0.0109 0.00879 0.0000695 0.0001 0.00324 0.0058 0.00711 0.0298 0.00267
3L:266120-266270:plus -22.9592 1.95413 8.73394 4.37615 -18.4592 -8.23469 -1.87156 7.02778 -0.137615
0.00475 0.0051 0.000293 0.00504 0.0109 0.000875 0.000431 0.000991 0.0194
3L:271560-271710:minus -32.7041 -1.06422 3.14679 4.86239 -26.2245 -8.79592 -20.0826 -0.152778
-1.57798 0.0222 0.0146 0.00375 0.00449 0.0175 0.0019 0.0209 0.0175 0.0299
3L:271560-271710:plus -13.0714 1.50459 3.46789 9.49541 -17.8878 -36.1122 6.72477 -5.52778 1.88073
0.000594 0.00602 0.00331 0.000308 0.00704 0.0878 0.0000335 0.069 0.00987
3L:2766260-2766410:minus -22.5102 -2.6422 -0.458716 -0.330275 -18.4796 9.37755 -8.45872 -1.54167
-0.0733945 0.00354 0.0233 0.0135 0.0295 0.011 0.0000363 0.00231 0.0263 0.0188
3L:2766260-2766410:plus -23 0.706422 -0.669725 7.34862 -25.9694 -26.8265 -8.79817 0.611111 7.73394
0.00498 0.00805 0.0144 0.000998 0.017 0.0285 0.00246 0.0138 0.000784
3L:2766460-2766610:minus -13.6327 14.4312 8.04587 9.08257 0.877551 -18.0102 -12.8165 -0.0277778
-4.63303 0.00101 5.39e-06 0.000428 0.000444 0.00019 0.00812 0.00554 0.0169 0.0661
3L:2766460-2766610:plus -33 13.4037 -1.55046 2.62385 -17.3776 -18.3469 -18.6789 -0.444444 1.22936

0.0258 0.0000154 0.0189 0.0108 0.00419 0.0124 0.0166 0.0192 0.0132
3L:2766900-2767050:minus -40.2857 3.64286 -0.550459 6.21101 -8.33673 -0.540816 -30.1376 1.375
-2.81651 0.0385 0.00159 0.0139 0.00213 0.00101 0.00054 0.0927 0.0107 0.0424
3L:2766900-2767050:plus -24.3673 5.52294 -1.21101 -0.321101 -18.7143 -27.5612 -7.57798 3.94444
1.3211 0.00791 0.00131 0.017 0.0294 0.0136 0.0393 0.00192 0.00411 0.0127
3L:2772240-2772390:minus -23.1837 -0.495413 1.22936 6.37615 -25.8571 9.60204 -13.1193 -3.91667
1.23853 0.00553 0.0122 0.00763 0.00192 0.0166 0.0000248 0.00594 0.0483 0.0132
3L:2772240-2772390:plus -39.9592 0.284404 -1.08257 1.09174 -7.60204 -9.5 -19.945 0.152778 3.09174
0.0348 0.00935 0.0164 0.02 0.000466 0.00294 0.0204 0.016 0.00678
3L:2774260-2774410:minus -21.7041 -2.7156 0.889908 6.16514 -18.7143 1.2449 -13.4404 1.59722 2.58716
0.00231 0.0238 0.00859 0.00223 0.0136 0.0000797 0.00639 0.00993 0.00779
3L:2774260-2774410:plus -33.3673 -3.27523 5.20183 2.57798 10.6531 -27.3061 -19.4679 6.38889 3.46789
0.0282 0.0278 0.00163 0.011 0.0000102 0.0336 0.0188 0.00137 0.00582
3L:2780780-2780930:minus -33.1122 -1.37615 2.98165 2 -27.6429 -9.02041 -26.0367 4.68056 -3.13761
0.0271 0.0161 0.004 0.0143 0.037 0.00213 0.0537 0.00302 0.0461
3L:2780780-2780930:plus -21.4388 4.73394 15.6239 -0.633028 -27.9694 -18.8265 -15.8532 8.55556
-0.247706 0.002 0.00181 5.44e-07 0.0327 0.0437 0.0148 0.0106 0.000422 0.0204
3L:2782120-2782270:minus -22.0408 0.376147 2.78899 9.33028 -25.9694 -17.2347 -13.6697 -2.09722 -0.513761
0.00296 0.00905 0.0043 0.000374 0.017 0.00511 0.00674 0.0306 0.0221
3L:2782120-2782270:plus -23 -0.513761 2.12844 1.20183 -28.2347 -18.2041 -16.9908 3.375 4.10092
0.00498 0.0123 0.00551 0.0195 0.051 0.0103 0.0128 0.00516 0.00413
3L:2787300-2787450:minus -23.102 -1.49541 -0.40367 1.0367 -26 0.530612 -7.34862 8.48611 7.63303
0.00522 0.0167 0.0132 0.0204 0.0171 0.000203 0.00183 0.00044 0.000928
3L:2787300-2787450:plus -22.1122 6.05505 7.80734 4.0367 -8.96939 -18.0102 7.00917 4.47222 2.11009
0.00304 0.00103 0.000485 0.00578 0.00226 0.00812 0.000031 0.0033 0.00898
3L:2809900-2810050:minus -13.1837 1.24771 -1.45872 5.58716 -17.4898 -26.898 -5.51376 3.30556
-0.0733945 0.000705 0.00661 0.0184 0.00334 0.0053 0.0302 0.00115 0.0053 0.0188
3L:2809900-2810050:plus -30.7041 5.87156 -0.706422 2.20183 -8.67347 -16.8265 -20.9817 4.18056
2.25688 0.00993 0.00112 0.0146 0.013 0.00154 0.0043 0.0245 0.00373 0.00853
3L:2818060-2818210:minus -14.5204 5.27523 -1.21101 -0.825688 -17.7857 -16.3469 -18.633 10.1528
1.55963 0.00145 0.00145 0.017 0.0347 0.00676 0.00416 0.0165 0.000147 0.0114
3L:2818060-2818210:plus -31.7041 -3.6055 4.73394 1.87156 -18.4184 -19.2755 -9.86239 1.83333 9.92661
0.0146 0.0304 0.00199 0.0152 0.0101 0.0185 0.003 0.00914 0.000193
3L:2820140-2820290:minus -39.4388 3.68367 2.55963 3.05505 -18.449 -8.86735 -25.4679 0.555556 -2.50459
0.0333 0.00144 0.0047 0.00878 0.0107 0.00198 0.0501 0.014 0.0388
3L:2820140-2820290:plus -22.8878 2.6055 1.33028 2.25688 -18.0816 -0.102041 -29.2385 1.19444
-10.7064 0.00441 0.00402 0.00736 0.0127 0.00759 0.000443 0.0816 0.0114 0.221
3L:2820600-2820750:minus -31.8469 1.87156 0.990826 5.85321 -27.0408 -18.4184 -14.4587 2.94444 5.65138
0.0162 0.00526 0.00829 0.00287 0.0242 0.0127 0.00802 0.00609 0.00228
3L:2820600-2820750:plus -41.2143 5.2844 6.06422 6.02752 -8.70408 -7.72449 -11.7064 -1 -2.49541
0.0494 0.00145 0.00112 0.00246 0.00155 0.00068 0.00433 0.0226 0.0387
3L:2821080-2821230:minus -24.2959 -2.6055 -0.93578 6.00917 -8.16327 -6.45918 -22.3028 -1.41667 1.00917
0.00781 0.0231 0.0156 0.00255 0.000873 0.000541 0.0311 0.0254 0.0143
3L:2821080-2821230:plus -20.6735 -0.477064 -0.990826 4.23853 -18.4898 -17.051 -19.2385 2.625
3.88991 0.00173 0.0121 0.0159 0.00522 0.0116 0.00479 0.0181 0.00687 0.00467
3L:2823780-2823930:minus -13.9898 -0.87156 0.889908 0.633028 -18.9796 -10.0918 -9.22018 -0.583333
8.62385 0.00114 0.0138 0.00859 0.0229 0.0155 0.00404 0.00266 0.02 0.00048
3L:2823780-2823930:plus -30.3673 -0.211009 0.0458716 5.49541 -27.9286 -8.65306 -26.0092 0.888889
-4.62385 0.00897 0.0111 0.0114 0.00355 0.0412 0.00156 0.0535 0.0126 0.066
3L:2851880-2852030:minus -14.3367 1.91743 0.247706 6.56881 -8.64286 -17.2347 -5.75229 1.625 3.15596
0.00131 0.00518 0.0107 0.0017 0.00139 0.00511 0.00123 0.00983 0.00659
3L:2851880-2852030:plus -14.9388 9.68807 2.53211 2.57798 -17.4898 -8.5 -15.8991 2.5 -2.6055 0.00161
0.000156 0.00475 0.011 0.0053 0.00126 0.0107 0.0072 0.04
3L:2867720-2867870:minus -13.8878 -0.733945 6.47706 11.1927 -17.2245 -26.7551 -15.3303 5.95833

5.69725 0.00111 0.0132 0.000928 0.0000254 0.00384 0.0263 0.00958 0.00169 0.00225
3L:2867720-2867870:plus -31.6327 8.37615 -4.61468 2.3578 -18.9796 -0.469388 -32.945 9.38889
-14.3119 0.0138 0.00031 0.0447 0.0121 0.0155 0.000533 0.131 0.000249 0.366
3L:292300-292450:minus -42.449 5.61468 6.68807 9.08257 -18.051 -9.53061 -16.7798 0.152778 -2.94495
0.0767 0.00126 0.000839 0.000444 0.00746 0.0031 0.0124 0.016 0.0441
3L:292300-292450:plus -31.2449 2.85321 2.3945 0.238532 10.6224 -27.7041 -11.7615 1.80556 5.00917
0.0111 0.00367 0.00499 0.0251 0.0000113 0.0445 0.00438 0.00923 0.0031
3L:293600-293750:minus -21.7041 3.68367 1.77982 4.12844 -18.1224 -18.0816 -12.5138 5.68056 -2.36697
0.00231 0.00144 0.00627 0.00549 0.00782 0.00934 0.00517 0.00193 0.0371
3L:293600-293750:plus -32.1735 6.34862 0.0366972 7.16514 -9.5 -18.3469 -13.3119 1.11111 2.86239 0.018
0.000897 0.0115 0.00116 0.00293 0.0124 0.00621 0.0117 0.00728
3L:3038260-3038410:minus -21.3571 0.724771 4.59633 4.36697 -18.1531 -27.6735 -9.06422 5.75 2.3211
0.00194 0.008 0.0021 0.00505 0.00829 0.0443 0.00259 0.00187 0.00839
3L:3038260-3038410:plus -21.7347 -4.66972 3.86239 7.7156 -8.93878 -37.2245 4.86239 1.86111 4.80734
0.00235 0.0404 0.00283 0.000691 0.00216 0.142 0.0000605 0.00905 0.00334
3L:3042340-3042490:minus -33 -1.54128 -1.49541 5.69725 -26.9286 -9.79592 -6.26606 2.56944 -6.37615
0.0258 0.0169 0.0186 0.00314 0.0214 0.00377 0.00141 0.00701 0.0975
3L:3042340-3042490:plus -30.7347 3.97959 -1.20183 5.57798 -7.67347 9.30612 -10.2569 5.65278 1.45872
0.01 0.000669 0.017 0.0034 0.00055 0.0000398 0.00323 0.00195 0.0119
3L:3042560-3042710:minus -24.2959 -1.54128 0.651376 4.31193 -18.4592 -18.2041 -6.26606 2.30556 -6.37615
0.00781 0.0169 0.00932 0.00516 0.0109 0.0103 0.00141 0.00772 0.0975
3L:3042560-3042710:plus -30.7347 3.97959 -1.20183 5.57798 -7.67347 9.30612 -10.2569 5.65278 1.45872
0.01 0.000669 0.017 0.0034 0.00055 0.0000398 0.00323 0.00195 0.0119
3L:3046760-3046910:minus -31.4694 0.458716 2.77064 2.78899 10.9898 -0.173469 -17.9083 1.56944
-0.816514 0.0124 0.00879 0.00433 0.00992 3.28e-06 0.000456 0.0147 0.01 0.0249
3L:3046760-3046910:plus -32.4694 0.688073 6.6789 6.72477 10.6531 9.7551 -19.0734 1.88889 -2.61468
0.0196 0.0081 0.000844 0.00154 0.0000102 0.0000198 0.0177 0.00896 0.0401
3L:3050280-3050430:minus -42.7347 -0.46789 3.81651 0.486239 -26.5306 -0.244898 -16.1284 3.81944
4.14679 0.0844 0.0121 0.00289 0.0237 0.0185 0.000496 0.0111 0.00433 0.00406
3L:3050280-3050430:plus -31.7041 -1.02752 8.7156 7.99083 20.4694 9.30612 -11.5321 -0.944444
3.46789 0.0146 0.0145 0.000296 0.00059 1.65e-07 0.0000398 0.00417 0.0222 0.00582
3L:3052380-3052530:minus -23.551 -0.944954 1.13761 2.78899 -26.2653 -0.244898 -21.8349 4.16667
-0.972477 0.00648 0.0141 0.00788 0.00992 0.018 0.000496 0.0286 0.00375 0.0259
3L:3052380-3052530:plus -23.2959 1.22018 5.90826 6.73394 -18.1531 -18.6327 -10.6606 2.13889 1.73394
0.00605 0.00668 0.0012 0.00152 0.00829 0.0137 0.00349 0.0082 0.0106
3L:3059160-3059310:minus -22.7347 3.59633 2.15596 2.23853 -27.1939 0.122449 -14.4954 1.65278 0.642202
0.00403 0.00279 0.00545 0.0128 0.0267 0.000312 0.00808 0.00974 0.0157
3L:3059160-3059310:plus -22.7755 8.30275 0.807339 6.29358 -18.0816 -9.53061 -12.5688 3.76389 2.95413
0.00412 0.000324 0.00884 0.00202 0.00759 0.0031 0.00524 0.00442 0.0071
3L:3059460-3059610:minus -40.449 0.201835 3.9633 6.6422 -17.7143 -17.2755 -24.0642 5.30556 5.29358
0.0402 0.00962 0.00272 0.00161 0.00627 0.00544 0.0414 0.00229 0.00278
3L:3059460-3059610:plus -23.551 -2.41284 3.22018 6.56881 -27.898 -6.72449 -12.2569 -1.94444 2.26606
0.00648 0.0219 0.00365 0.0017 0.04 0.000544 0.00488 0.0294 0.00847
3L:3060260-3060410:minus -23.2551 0.422018 15.5596 7.29358 -18.1122 -26.6735 -6.41284 0.430556 1.54128
0.00583 0.0089 6.69e-07 0.00104 0.00771 0.0256 0.00146 0.0146 0.0115
3L:3060260-3060410:plus -32.7041 1.15596 0.504587 1.7156 -27.5306 -8.79592 -11.8073
0.791667 -0.87156 0.0222 0.00684 0.0098 0.0162 0.0351 0.0019 0.00442 0.013 0.0252
3L:3069900-3070050:minus -24.1122 1.12844 13.0367 0.211009 -18.7551 -18.2755 -14.7248
-0.430556 9.87156 0.00738 0.00691 2e-05 0.0254 0.0144 0.0112 0.00848 0.0191 0.000217
3L:3069900-3070050:plus -31.4388 -5.75229 9.44954 2.08257 -17.1837 -18.1224 -8.83486
0.263889 12.2752 0.0122 0.0536 0.000196 0.0137 0.00366 0.00988 0.00248 0.0154 0.0000141
3L:3070700-3070850:minus -21.4796 -3.45872 2.14679 0.238532 10.6531 -19.1531 -7.97248
1.81944 5.22018 0.00202 0.0292 0.00547 0.0251 0.0000102 0.0177 0.00209 0.00919 0.00289
3L:3070700-3070850:plus -32.9694 3.26606 -0.862385 2.98165 -18.5204 -8.16327 -20.2202

0.652778	-0.807339	0.0254	0.00315	0.0153	0.00905	0.0117	0.000783	0.0214	0.0136	0.0247
3L:3071060-3071210:minus	-24.2245	12.844	-2.89908	2.3578	-8.57143	-26.5612	-18.1376
3	3.6055	0.00763	0.0000255	0.028	0.0121	0.00113	0.0237	0.0153	0.00597	0.00532
3L:3071060-3071210:plus	-30.2143	-3.20183	1.04587	4.07339	20.4694	-18.5714	-4.83486	5.13889
-0.926606	0.0088	0.0273	0.00813	0.00562	1.65e-07	0.0136	0.000947	0.00247	0.0256
3L:3071280-3071430:minus	-23.5612	3.64286	2.22936	3.51376	-18.7143	-17.898	-15.4954	9.77778
1.78899	0.00653	0.00159	0.00531	0.00709	0.0136	0.00726	0.00989	0.000192	0.0103
3L:3071280-3071430:plus	-32.1429	-0.0733945	1.50459	6.11009	-18.7551	-18.8571	-19.422	4.51389
9.45872	0.0179	0.0106	0.00692	0.00233	0.0144	0.0151	0.0187	0.00324	0.000337
3L:3077760-3077910:minus	-31.6224	-1.23853	0	0.587156	-27.3061	-27.7041	-3.14679
3.91667	5.6055	0.0137	0.0154	0.0116	0.0231	0.0315	0.0445	0.00059	0.00416	0.00232
3L:3077760-3077910:plus	-12.6633	0.614679	3.55046	4.40367	-7.82653	1.16327	-9.0367	4.05556
3.72477	0.000458	0.00832	0.00321	0.00497	0.00059	0.0000831	0.00258	0.00393	0.00497
3L:3090960-3091110:minus	-33	0.550459	-2.37615	6.06422	-17.4184	-9.02041	-26.156	3.625
4.18349	0.0258	0.00851	0.0241	0.00239	0.00457	0.00213	0.0545	0.00468	0.00404
3L:3090960-3091110:plus	-32.3367	2.63303	2.70642	5.43119	-18.9796	1.05102	-11.0917	0.194444
6.89908	0.0187	0.00398	0.00444	0.00371	0.0155	0.000107	0.0038	0.0157	0.00137
3L:311620-311770:minus	-21.7755	3.97959	11.9725	11.0183	-26.9286	-9.23469	-9.21101	0.125
0.825688	0.00241	0.000669	0.0000439	0.000052	0.0214	0.00238	0.00266	0.0161	0.0149
3L:311620-311770:plus	-22.7755	1.20183	0.0550459	2.25688	-17.4898	-26.8571	-12.9817
-2.52778	7.6422	0.00412	0.00673	0.0114	0.0127	0.0053	0.0288	0.00576	0.0343	0.000893
3L:3125440-3125590:minus	-20.6224	4.2844	5.6055	4.02752	-26.1939	-27.4898	-10.9266
0.361111	-0.0366972	0.0017	0.00216	0.00137	0.00581	0.0175	0.0363	0.00368	0.0149	0.0187
3L:3125440-3125590:plus	-33.8878	3.58716	3.22936	6.06422	-18.449	0.0204082	-2.7156	8.41667	2.54128
0.0302	0.0028	0.00363	0.00239	0.0107	0.000377	0.000528	0.000458	0.00787
3L:3125800-3125950:minus	-12.2959	4.11927	5.26606	4.36697	-18.7143	-9.30612	-20.4495	-1
-0.788991	0.000384	0.0023	0.00159	0.00505	0.0136	0.00257	0.0223	0.0226	0.0245
3L:3125800-3125950:plus	-34.1837	-0.266055	-4.11009	2.3945	-28.4898	0.755102	-29.1376
3.63889	-7.33028	0.0314	0.0113	0.0391	0.0119	0.0581	0.000175	0.0805	0.00465	0.121
3L:3126220-3126370:minus	-30.7755	-0.587156	3.79817	2.70642	-27.5306	-17.5714	-11.9817
4.79167	3.14679	0.0102	0.0126	0.00291	0.0103	0.0351	0.00616	0.00459	0.00288	0.00666
3L:3126220-3126370:plus	-21.9286	-1.3211	3.6422	4.22018	-8.63265	-25.5306	-11.4771	12.75
-0.220183	0.0027	0.0159	0.00309	0.00531	0.0013	0.0205	0.00412	0.0000138	0.0201
3L:3127680-3127830:minus	-24.2245	4.77064	-2.48624	4.46789	0.877551	-18.3469	-17.7248
10.4028	3.23853	0.00763	0.00179	0.0249	0.0049	0.00019	0.0124	0.0143	0.000122	0.00639
3L:3127680-3127830:plus	-41.3265	-0.33945	-4.52294	0.0733945	-28.0408	-26.5306
-14.2844	10.125	0.853211	0.0541	0.0116	0.0436	0.0263	0.0455	0.023	0.00772	0.00015	0.0148
3L:3134140-3134290:minus	-13.2245	4.58716	4.38532	5.6422	-18.2245	-9.16327	2.74312	0.0833333
2.06422	0.000738	0.00192	0.0023	0.00325	0.00877	0.00223	0.000129	0.0163	0.00912
3L:3134140-3134290:plus	-30.6939	3.11009	1.3211	6.37615	-17.8571	-19.0102	-0.220183	-10.375
9.61468	0.00979	0.00334	0.00738	0.00192	0.00698	0.0159	0.000298	0.163	0.00029
3L:3142720-3142870:minus	-3.60204	3.90816	0.12844	7.43119	-18.7449	-9.53061	-8.68807
-3.05556	-0.706422	0.0000856	0.00102	0.0111	0.000939	0.0138	0.0031	0.00241	0.0392	0.024
3L:3142720-3142870:plus	-13.1224	0.770642	-3.44037	1.98165	-26.7449	-7.42857	-20.0367
0.638889	8.19266	0.000633	0.00786	0.0326	0.0145	0.0195	0.000567	0.0207	0.0137	0.000548
3L:3143340-3143490:minus	-21.6224	1.25688	5.09174	3.50459	-18.449	0.122449	-13.2385
0.0138889	3.88073	0.00213	0.00659	0.00171	0.00713	0.0107	0.000312	0.00611	0.0167	0.00475
3L:3143340-3143490:plus	-11.8061	-2.16514	3.7156	2.21101	-18.4184	-18.0816	-13.6055
4.04167	7.04587	0.000268	0.0204	0.00301	0.0129	0.0101	0.00934	0.00664	0.00395	0.00132
3L:3143780-3143930:minus	-32.1122	0.0275229	-2.11009	4.02752	-27.1939	-0.0204082
-18.5321	9.09722	-4.43119	0.0178	0.0102	0.0223	0.00581	0.0267	0.000411	0.0162	0.000301
0.0627
3L:3143780-3143930:plus	-40.2551	-0.577982	11.6239	7.29358	11.6939	-9.86735	-21.5046	5.93056
-0.440367	0.0382	0.0125	0.0000551	0.00104	1.17e-06	0.00391	0.027	0.00171	0.0216

3L:3145260-3145410:minus -41.7755 2.3211 4.7156 6.57798 -9.16327 -19.3776 -18.8349
0.791667 -3.66972 0.0652 0.00446 0.002 0.00167 0.00249 0.0195 0.017 0.013 0.0514
3L:3145260-3145410:plus -30.6633 7.44037 4.90826 0.825688 -8.64286 -17.2755 -19.9541 0.875
0.816514 0.00976 0.000515 0.00185 0.0216 0.00139 0.00544 0.0204 0.0127 0.015
3L:3148500-3148650:minus -24 5.61468 2.9633 5.86239 -9.23469 -17.9796 -21.3578 0.263889
0.926606 0.00719 0.00126 0.00403 0.00282 0.00277 0.00764 0.0262 0.0154 0.0146
3L:3148500-3148650:plus -24.1429 -1.57798 1.13761 2.6789 -9.23469 -8.57143 -20.578 6.63889
1.53211 0.0074 0.0171 0.00788 0.0105 0.00277 0.00149 0.0228 0.00121 0.0116
3L:3149100-3149250:minus -14.6327 13.4592 0.733945 11.2936 -27.1939 -8.45918 -14.1835
2.81944 -2.57798 0.0015 0.000064 0.00906 0.0000139 0.0267 0.00111 0.00755 0.00639 0.0397
3L:3149100-3149250:plus -31.0306 -2.2844 -0.568807 0.422018 -18.5612 -19.1531 -13.6239
8.22222 9.30275 0.0105 0.0211 0.0139 0.0241 0.0119 0.0177 0.00667 0.000514 0.00038
3L:3159500-3159650:minus -22 0.844037 -0.0733945 0.633028 -8.60204 -18.2755
-13.6881 3.01389 0.587156 0.00291 0.00766 0.0119 0.0229 0.00125 0.0112 0.00676 0.00593 0.016
3L:3159500-3159650:plus -23.8878 1.81651 -3.55046 4.49541 -8.67347 9.86735 -19.8899 4.18056
-0.0733945 0.00692 0.00537 0.0336 0.00487 0.00154 0.0000132 0.0202 0.00373 0.0188
3L:3159980-3160130:minus -33.0408 -2.04587 6.23853 -0.321101 -28.1224 19.3776 -18.7064
1.33333 -1.33945 0.0264 0.0197 0.00104 0.0294 0.0457 3.24e-07 0.0167 0.0109 0.0285
3L:3159980-3160130:plus -3.85714 3.05505 2.7156 3.88991 -18.1531 -18.051 -21.7339 -3.90278
0.110092 0.00011 0.00341 0.00442 0.00608 0.00829 0.00886 0.0281 0.0481 0.018
3L:3162040-3162190:minus -33.0714 14.4954 -3.88991 1.91743 -27.5306 1.09184 -9.49541
3.27778 3.27523 0.0267 5.09e-06 0.0369 0.015 0.0351 0.000091 0.0028 0.00536 0.00625
3L:3162040-3162190:plus -14.5918 3.53211 -3.93578 2.95413 -27.1531 -9.38776 -15.3394
-1.30556 -0.706422 0.00148 0.00286 0.0373 0.00917 0.0249 0.00266 0.0096 0.0246 0.024
3L:3165080-3165230:minus -13.5612 13.5306 -3.79817 3.77064 -26.9286 -9.38776 -7.62385
-0.708333 1.21101 0.000984 0.0000206 0.036 0.00637 0.0214 0.00266 0.00194 0.0207 0.0134
3L:3165080-3165230:plus -23.449 5.40367 -1.40367 1.3211 -17.3776 -8.64286 -18.945 3.76389 3.88073
0.00631 0.00138 0.0181 0.0189 0.00419 0.00154 0.0173 0.00442 0.00475
3L:3165600-3165750:minus -21.2245 2.11009 -5.22936 -1.05505 -17.7857 -8.79592
-24.5046 5.98611 -3.57798 0.0019 0.00482 0.0523 0.0374 0.00676 0.0019 0.0441 0.00167 0.0506
3L:3165600-3165750:plus -14.4082 -0.761468 7.7156 7.78899 -18.7551 1.05102 -13.0092 1.36111
1.61468 0.00136 0.0133 0.000509 0.000642 0.0144 0.000107 0.00579 0.0108 0.0112
3L:3166120-3166270:minus -4.47959 3.04587 9.77064 7.55963 -18.1837 -8.86735 0.348624
8.38889 3.05505 0.000154 0.00342 0.000162 0.000807 0.00835 0.00198 0.000261 0.000466
0.00692
3L:3166120-3166270:plus -31.9286 6.70642 1.11009 6.00917 -17.1224 -17.9388 -16.8624 3.73611
1.54128 0.0166 0.000754 0.00796 0.00255 0.00343 0.00738 0.0125 0.00447 0.0115
3L:3166480-3166630:minus -22.2551 4.0367 2.94495 5.6422 -18.4184 -9.23469 -8.59633
-0.111111 -1.55046 0.00327 0.00237 0.00405 0.00325 0.0101 0.00238 0.00237 0.0173 0.0297
3L:3166480-3166630:plus -3.67347 2.66972 2.44954 4.23853 -17.1531 -26.2653 3.41284 -1 5.18349
0.0000972 0.00393 0.00489 0.00522 0.00364 0.0218 0.000102 0.0226 0.00295
3L:3167340-3167490:minus -33.1429 -0.12844 -2.66972 5.59633 -18.0102 -9.02041
-25.6697 6.58333 4.0367 0.0272 0.0108 0.0263 0.00331 0.00732 0.00213 0.0514 0.00124 0.00424
3L:3167340-3167490:plus -21.8878 -3.05505 0.504587 9.61468 -18.1224 -8.45918 -21.9725
-2.90278 -9.33028 0.00258 0.0262 0.0098 0.000255 0.00782 0.00111 0.0293 0.0377 0.175
3L:3169200-3169350:minus -41.5102 1.00917 1.02752 8.98165 -8.20408 0.755102 -12.2569
3.11111 -4.16514 0.0582 0.00722 0.00819 0.000461 0.000894 0.000175 0.00488 0.00572 0.0579
3L:3169200-3169350:plus -22.1224 -2.37615 5.59633 3.97248 20.2041 -0.316327 -26.6972 1.52778
-10.5596 0.00304 0.0216 0.00138 0.00587 3.64e-07 0.000524 0.0583 0.0102 0.215
3L:3178560-3178710:minus -32.4796 -0.183486 9.26606 3.18349 -18.4898 -27.3367 -1.92661
1.18056 9.92661 0.02 0.011 0.000217 0.00837 0.0116 0.0347 0.000437 0.0114 0.000193
3L:3178560-3178710:plus -13.0714 8.31193 -0.348624 3.78899 -18.4184 -8.72449 -14.5046
0.375 -1.21101 0.000594 0.000323 0.013 0.00632 0.0101 0.00169 0.0081 0.0149 0.0277
3L:3179040-3179190:minus -41.2551 -2.19266 3.11009 1.91743 -9.16327 -18.5612 -12.633

2.98611 2.41284 0.0513 0.0205 0.00381 0.015 0.00249 0.0132 0.00532 0.006 0.0082
3L:3179040-3179190:plus -20.6633 -0.513761 12.1101 4.07339 -17.4082 -28.5306 -14.6972
-3.72222 2.86239 0.00173 0.0123 0.0000401 0.00562 0.00421 0.0625 0.00843 0.0461 0.00728
3L:3179320-3179470:minus -22.9286 -0.348624 10.7982 3.94495 -18.1531 -18.051 -15.0183 4
4.77064 0.0046 0.0116 0.0000898 0.00595 0.00829 0.00886 0.00901 0.00402 0.0034
3L:3179320-3179470:plus -30.2449 3.02752 -1.3945 3.95413 -17.4184 -18.0102 -6.19266 0.569444
1.58716 0.00881 0.00344 0.018 0.00593 0.00457 0.00812 0.00138 0.014 0.0113
3L:3192600-3192750:minus -22.9184 12.0917 11.422 3.43119 -7.89796 -8.02041 -20.4037
5.52778 0.229358 0.00447 0.0000437 0.0000616 0.00749 0.000678 0.000761 0.0221 0.00207
0.0173
3L:3192600-3192750:plus -33.3367 -3.34862 3.52294 1.84404 -28.5612 -17.7143 -28.1927
-0.291667 2.20183 0.0281 0.0284 0.00324 0.0154 0.0617 0.00634 0.0706 0.0183 0.00869
3L:3193060-3193210:minus -31.8061 0.963303 -0.816514 0.0642202 -9.16327 -9.94898
-15.2569 0.861111 -1.43119 0.0158 0.00734 0.0151 0.0264 0.00249 0.00393 0.00944 0.0127 0.029
3L:3193060-3193210:plus -21.7041 0.440367 2.17431 3.47706 -19.051 -8.93878 -10.3486 13.7222
1.27523 0.00231 0.00885 0.00542 0.00724 0.0162 0.00201 0.00329 3.99e-06 0.013
3L:3193860-3194010:minus -24.3673 -5.41284 -2.77982 2.62385 -28.3061 -18.1224
-14.5138 5.23611 4.92661 0.00791 0.0491 0.0271 0.0108 0.0553 0.00988 0.00811 0.00236 0.00318
3L:3193860-3194010:plus -40.8878 -0.110092 4.86239 4.13761 -8.63265 -8.09184 -22.7339
1.68056 0.779817 0.0439 0.0107 0.00188 0.00546 0.0013 0.000762 0.0335 0.00964 0.0151
3L:319400-319550:minus -41.551 0.440367 4.18349 7.27523 -17.5306 -9.5 -19.6055 4.625 5.18349
0.0607 0.00885 0.00249 0.00109 0.0054 0.00294 0.0193 0.00309 0.00295
3L:319400-319550:plus -22.4082 1.00917 4.94495 4.02752 -17.449 -15.9796 -9.0367 -3.41667 0.330275
0.00345 0.00722 0.00182 0.00581 0.00484 0.00405 0.00258 0.0428 0.0168
3L:3197620-3197770:minus -24.1429 -3.24771 14.1651 7.29358 -27.5612 -17.3061 -15.2477
6.75 -0.440367 0.0074 0.0276 6.03e-06 0.00104 0.0354 0.00548 0.00943 0.00114 0.0216
3L:3197620-3197770:plus -23.2245 0.844037 -1.84404 4.06422 -16.3776 -26.7857 -13.7615
2.75 1.38532 0.00576 0.00766 0.0206 0.00564 0.003 0.0266 0.00688 0.00656 0.0123
3L:319840-319990:minus -30.4796 5.85321 -1.77064 7 -17.449 -17.7143 -13.0092 -0.402778
-0.220183 0.00941 0.00113 0.0202 0.00132 0.00484 0.00634 0.00579 0.0189 0.0201
3L:319840-319990:plus -22.7041 3.90816 -1.55046 4.11009 -9.0102 -9.45918 -11.5963 -2.06944
5.3211 0.00395 0.00102 0.0189 0.00553 0.00236 0.00279 0.00422 0.0304 0.00275
3L:3198780-3198930:minus -22.9592 0.678899 -2.22936 2.30275 -17.7551 -18.0102
-13.0367 -0.111111 3.7156 0.00475 0.00813 0.0231 0.0124 0.00658 0.00812 0.00583 0.0173 0.00502
3L:3198780-3198930:plus -31.8163 3.51376 -1.06422 0.954128 -17.4898 -18.5714 -21.9725
11.625 -1.04587 0.016 0.00288 0.0163 0.0208 0.0053 0.0136 0.0293 0.0000441 0.0264
3L:3199060-3199210:minus -22.5816 4.62385 -1.3945 7.17431 -27.1224 9.37755 -8.18349
-0.0555556 -3.04587 0.00361 0.00189 0.018 0.00115 0.0245 0.0000363 0.00218 0.017 0.0451
3L:3199060-3199210:plus -29.4796 -0.0733945 -2.34862 4.0367 10.9898 -27.5612 -21.0275
-3.44444 5.90826 0.00844 0.0106 0.0239 0.00578 3.28e-06 0.0393 0.0247 0.0431 0.00198
3L:3218940-3219090:minus -33.0408 0.247706 5.45872 7.61468 10.4286 9.60204 -17.633 1.54167
-4.05505 0.0264 0.00947 0.00147 0.000779 0.0000139 0.0000248 0.0141 0.0101 0.0564
3L:3218940-3219090:plus -22.9286 -0.788991 -0.0458716 3.16514 20.4694 -0.540816 -20.1651
3.04167 -1.3578 0.0046 0.0134 0.0118 0.00843 1.65e-07 0.00054 0.0212 0.00587 0.0286
3L:3222620-3222770:minus -21.7653 6.47706 6.83486 3.88073 -18.6429 -9.02041 -14.6239
3.29167 3.45872 0.00237 0.000843 0.000785 0.0061 0.0121 0.00213 0.0083 0.00533 0.00591
3L:3222620-3222770:plus -41.4796 4.02752 0.477064 3.18349 -27.1939 -28.6735 -13.5872
1.08333 5.81651 0.0577 0.00238 0.00989 0.00837 0.0267 0.0667 0.00661 0.0118 0.00204
3L:3223020-3223170:minus -21.5918 -1.72477 -0.284404 2.72477 -18.4592 -8.27551
-11.2661 2.15278 3.20183 0.00209 0.0179 0.0127 0.0102 0.0109 0.000919 0.00394 0.00816 0.0065
3L:3223020-3223170:plus -30.1429 13.5306 0.330275 5.07339 -18.449 -8.38776 -13.8899 1.73611
0.321101 0.00866 0.0000206 0.0104 0.0043 0.0107 0.00101 0.00708 0.00946 0.0169
3L:3223300-3223450:minus -22.9592 13.4592 1.73394 4.7156 -19.051 0.826531 -20.6697
-0.0416667 1.66055 0.00475 0.000064 0.00638 0.00464 0.0162 0.000153 0.0232 0.017 0.011

3L:3223300-3223450:plus -33.0306 4.04587 -0.963303 6.83486 -17.449 -9.79592 -19.4862 -0.263889 -4.44037 0.026 0.00236 0.0158 0.00142 0.00484 0.00377 0.0189 0.0181 0.063
3L:3224000-3224150:minus -30.4388 -2.74312 3.55963 1.56881 -16.7143 -18.051 -13.8349 -6.18056 7.15596 0.00923 0.024 0.0032 0.0172 0.00319 0.00886 0.00699 0.0789 0.00124
3L:3224000-3224150:plus -23.6327 6.37615 5.70642 3.23853 -27.3367 -19.1531 -13.4862 6.18056 5.70642 0.00661 0.000885 0.00131 0.00815 0.0317 0.0177 0.00646 0.00152 0.00222
3L:3224880-3225030:minus -32.2857 0.917431 -3.70642 5.06422 -17.449 -0.469388 -19.6881 3.43056 4.09174 0.0185 0.00746 0.0351 0.00433 0.00484 0.000533 0.0195 0.00505 0.00416
3L:3224880-3225030:plus -20.7041 -2.15596 2.09174 6.12844 10.398 0.979592 -12.2844 3.18056 9.30275 0.00175 0.0203 0.00558 0.00231 0.0000153 0.000136 0.00491 0.00557 0.00038
3L:3225360-3225510:minus -40.9898 1.84404 7.3578 2.98165 -18.1531 -27.9286 -10.7798 0.25 3.6789 0.0455 0.00532 0.000608 0.00905 0.00829 0.054 0.00357 0.0155 0.00506
3L:3225360-3225510:plus -22.1531 8.19266 -1.91743 4.3211 -17.8265 -0.173469 -13.4312 -5.61111 -0.0275229 0.0031 0.000343 0.0211 0.00514 0.00691 0.000456 0.00638 0.0703 0.0186
3L:3226240-3226390:minus -31.7755 1.30275 0.477064 0.201835 -27.1939 -0.0204082 -13.7706 -2.93056 9.34862 0.0157 0.00649 0.00989 0.0255 0.0267 0.000411 0.00689 0.038 0.000371
3L:3226240-3226390:plus -14.2245 -0.706422 7.27523 -2.21101 -27.0102 -17.051 -25.6606 -3.69444 9.45872 0.00126 0.0131 0.000635 0.055 0.0237 0.00479 0.0513 0.0458 0.000337
3L:3235840-3235990:minus -22.6327 2.02752 2.89908 9.33028 -9.20408 0.122449 -26.8532 3.98611 -4.44954 0.00373 0.00497 0.00413 0.000374 0.00262 0.000312 0.0594 0.00404 0.0632
3L:3235840-3235990:plus -24.2959 3.68367 -0.770642 5.74312 -28.4592 -17.4184 -26.6881 5.79167 -0.0917431 0.00781 0.00144 0.0149 0.00302 0.0568 0.00585 0.0582 0.00183 0.019
3L:3238100-3238250:minus -12.8776 -4.30275 0.165138 0.633028 -18.1939 -17.9796 -9.25688 -0.361111 0.788991 0.000507 0.0367 0.011 0.0229 0.0086 0.00764 0.00268 0.0187 0.0151
3L:3238100-3238250:plus -33.1429 2.22936 -1.09174 0.46789 -18.7143 -7.72449 -18.7339 6.125 1.95413 0.0272 0.00461 0.0164 0.0239 0.0136 0.00068 0.0167 0.00156 0.00964
3L:3240020-3240170:minus -31.2551 0.33945 0.541284 5.37615 -17.3776 -28.4082 -14.6881 7.33333 4.92661 0.0113 0.00917 0.00968 0.00384 0.00419 0.0615 0.00842 0.000844 0.00318
3L:3240020-3240170:plus -40.4796 -1.88073 -1 5.65138 -18.0816 -17.3061 -13.0826 3.23611 -4.18349 0.0407 0.0188 0.016 0.00322 0.00759 0.00548 0.00589 0.00545 0.0581
3L:3243180-3243330:minus -23 1.05505 -1.88991 4.78899 -17.1531 -8.72449 -21.7431 0.0833333 0.743119 0.00498 0.00709 0.0209 0.00456 0.00364 0.00169 0.0282 0.0163 0.0153
3L:3243180-3243330:plus -14.0306 6.69725 0.394495 4.20183 -8.82653 -26.898 3.86239 1.93056 7.84404 0.00115 0.000757 0.0102 0.00533 0.00163 0.0302 0.0000868 0.00883 0.000701
3L:3245660-3245810:minus -32.4388 1.63303 1.72477 2.92661 -8.64286 -17.7143 -13.8257 0.222222 1.86239 0.0195 0.00575 0.0064 0.00931 0.00139 0.00634 0.00698 0.0156 0.00994
3L:3245660-3245810:plus -31.4388 -1.69725 9.55046 2.66972 -27.3061 -9.72449 -21.2661 3.04167 3.05505 0.0122 0.0178 0.000185 0.0106 0.0315 0.00352 0.0258 0.00587 0.00692
3L:3246420-3246570:minus -31.6327 3.42202 4.59633 7.61468 -26.898 1.02041 -30.7064 -3.98611 -0.715596 0.0138 0.00298 0.0021 0.000779 0.0203 0.000118 0.1 0.0491 0.0241
3L:3246420-3246570:plus -21.4388 3.33028 3.90826 2.07339 -18.1531 -26.9694 3.30275 -0.333333 1.16514 0.002 0.00308 0.00278 0.0138 0.00829 0.0304 0.000106 0.0185 0.0135
3L:324660-324810:minus -23.7857 15.5963 -0.0733945 5.73394 -28.5714 -9.27551 -22.3578 0.305556 -2.95413 0.00679 8.91e-07 0.0119 0.00307 0.0623 0.00244 0.0314 0.0152 0.0441
3L:324660-324810:plus -32.7755 1.06422 1.90826 6.84404 -27.2653 -19.3061 -27.5229 0.875 -7.0367 0.0234 0.00707 0.00598 0.0014 0.0302 0.0187 0.0646 0.0127 0.114
3L:3249280-3249430:minus -22 -4.26606 3.72477 5.94495 -7.63265 -8.02041 -19.7706 6.18056 0.394495 0.00291 0.0363 0.003 0.00261 0.000479 0.000761 0.0198 0.00152 0.0166
3L:3249280-3249430:plus -13.3776 0.550459 2.93578 5.57798 -19.0918 -17.7143 -20.3578 10.0556 4.49541 0.000831 0.00851 0.00407 0.0034 0.0163 0.00634 0.0219 0.000158 0.00363
3L:3249880-3250030:minus -12.0408 -1.86239 -1.0367 1.91743 -17.602 -8.5 -19.6881 1.41667 1.70642 0.000311 0.0187 0.0161 0.015 0.00547 0.00126 0.0195 0.0106 0.0108
3L:3249880-3250030:plus -33.9592 -0.0917431 3.99083 3.31193 -18.4592 -27.6735 -13.4771 -2.13889 10.0367 0.0304 0.0106 0.00269 0.00786 0.0109 0.0443 0.00645 0.0309 0.000146

3L:3267280-3267430:minus -31.7041 6.68807 3.91743 4.3211 -27.1531 -18.3469 -13.7248 -1.90278 0.495413 0.0146 0.00076 0.00277 0.00514 0.0249 0.0124 0.00682 0.029 0.0162
3L:3267280-3267430:plus -32.6224 0.229358 6.15596 4.04587 -26.7449 -18.0816 -16.2661 2.31944 0.770642 0.0211 0.00953 0.00108 0.00572 0.0195 0.00934 0.0114 0.00769 0.0151
3L:327920-328070:minus -32.7755 -1.82569 0.678899 1.9633 10.6531 -17.5 -20.6972 5.06944 -1.55046 0.0234 0.0185 0.00923 0.0146 0.0000102 0.00599 0.0233 0.00255 0.0297
3L:327920-328070:plus -30.8163 8.56881 -2.31193 2.87156 11.6939 -18.3469 -19.0275 3.43056 -0.642202 0.0103 0.000281 0.0237 0.00948 1.17e-06 0.0124 0.0175 0.00505 0.0234
3L:329940-330090:minus -30.9184 -0.174312 -2.74312 7.63303 -17.1122 -9.86735 -23.6147 8.31944 7.48624 0.0103 0.0109 0.0268 0.000745 0.00341 0.00391 0.0387 0.000485 0.00101
3L:329940-330090:plus -24.0816 -2.36697 7.59633 9.61468 -8.86735 -26.5612 -22.844 5.33333 9 0.00732 0.0216 0.000541 0.000255 0.00173 0.0237 0.0342 0.00226 0.000437
3L:3303400-3303550:minus -23.1837 2.49541 7.66972 -1.50459 -17.3776 -8.20408 -8.42202 3.875 -0.247706 0.00553 0.00418 0.000521 0.0434 0.00419 0.000796 0.00229 0.00423 0.0204
3L:3303400-3303550:plus -22.7449 7.6789 3.76147 6.12844 10.6939 1.05102 -7.25688 2.91667 -1.78899 0.00404 0.000454 0.00295 0.00231 7.35e-06 0.000107 0.00179 0.00616 0.0314
3L:3303640-3303790:minus -40.7449 3.68367 -2.89908 4.02752 -17.6429 -9.30612 -23.0734 3.65278 -1.75229 0.0431 0.00144 0.028 0.00581 0.00562 0.00257 0.0355 0.00462 0.0312
3L:3303640-3303790:plus -22.398 7.6789 5.29358 6.12844 10.6939 -17.5714 -18.9817 2.91667 -1.78899 0.00344 0.000454 0.00157 0.00231 7.35e-06 0.00616 0.0174 0.00616 0.0314
3L:3319300-3319450:minus -40.551 8.78899 1.59633 1.77982 -18.4184 -17.5714 -8.6789 0.791667 -0.440367 0.0418 0.00025 0.00669 0.0158 0.0101 0.00616 0.00241 0.013 0.0216
3L:3319300-3319450:plus -24.1531 14.3394 5.93578 0.889908 -18.449 -18.0102 -18.9266 4 -2.83486 0.00744 5.96e-06 0.00119 0.0213 0.0107 0.00812 0.0172 0.00402 0.0427
3L:3325920-3326070:minus -12.2551 -0.100917 2.81651 2.7156 -8.40816 9.60204 1.47706 -4.79167 2.25688 0.000379 0.0107 0.00426 0.0102 0.00108 0.0000248 0.000193 0.0589 0.00853
3L:3325920-3326070:plus -32 -3.29358 4.22018 1.85321 -8.64286 -19.1224 -20.578 -3.06944 0.293578 0.0171 0.028 0.00245 0.0153 0.00139 0.0175 0.0228 0.0393 0.017
3L:3326240-3326390:minus -24.1531 -2.75229 4.6055 -0.798165 19.9082 -17.5714 -11.5872 -4.19444 3.20183 0.00744 0.0241 0.0021 0.0344 8.13e-07 0.00616 0.00422 0.0515 0.0065
3L:3326240-3326390:plus -21.8163 2.21101 -2.00917 9.59633 -17.4184 0.0918367 -22.2018 9.29167 2.16514 0.00244 0.00464 0.0217 0.000275 0.00457 0.000323 0.0306 0.000266 0.00879
3L:3326960-3327110:minus -40.6224 -7.62385 2.81651 3.86239 -17.449 -28.4082 -20.2294 1.33333 -2.99083 0.0424 0.0844 0.00426 0.00622 0.00484 0.0615 0.0214 0.0109 0.0445
3L:3326960-3327110:plus -33.0714 0.302752 0.192661 -0.284404 -27.1939 -17.2755 -25.1927 2.55556 1.01835 0.0267 0.00929 0.0109 0.0291 0.0267 0.00544 0.0484 0.00705 0.0142
3L:3334780-3334930:minus -31.8163 3.74312 -0.348624 3.94495 -17.7959 -9.72449 -12.3945 6.81944 6 0.016 0.00265 0.013 0.00595 0.00678 0.00352 0.00504 0.0011 0.00184
3L:3334780-3334930:plus -24.5612 -2.37615 2.13761 9.08257 -18.4796 0.0510204 -7.06422 5.11111 -0.229358 0.00819 0.0216 0.00549 0.000444 0.011 0.000349 0.00171 0.0025 0.0202
3L:3336780-3336930:minus -32.7755 11.4128 -2.88073 5.69725 11.4286 -9.53061 -16.8624 2.33333 -4.54128 0.0234 0.0000647 0.0279 0.00314 1.97e-06 0.0031 0.0125 0.00765 0.0645
3L:3336780-3336930:plus -22.7347 -0.651376 3.13761 5.45872 -18.1531 -9.23469 -12.3303 1.44444 -0.495413 0.00403 0.0128 0.00377 0.00363 0.00829 0.00238 0.00496 0.0105 0.022
3L:3341940-3342090:minus -14.3061 -2.0367 4.69725 2.6789 -17.4592 -27.0408 -10.1009 1.91667 3.20183 0.00129 0.0196 0.00202 0.0105 0.00491 0.0307 0.00314 0.00888 0.0065
3L:3341940-3342090:plus -23 0.0550459 -5.3211 0.752294 -18.6429 -17.4286 -17.0642 0 2.36697 0.00498 0.0101 0.0535 0.0221 0.0121 0.00586 0.0129 0.0167 0.00828
3L:3346440-3346590:minus -21.9286 13.5306 -2.95413 9.59633 -18.4184 0.540816 -17.1101 -3.84722 -1.79817 0.0027 0.0000206 0.0285 0.000275 0.0101 0.0002 0.013 0.0475 0.0315
3L:3346440-3346590:plus -32.6327 1.05505 4.70642 4.65138 -18.051 -17.5 -16.8073 1.15278 2.21101 0.0212 0.00709 0.00201 0.00471 0.00746 0.00599 0.0124 0.0115 0.00862
3L:3350080-3350230:minus -30.4082 2.45872 10.6147 6.42202 -8.93878 -8.72449 -5.78899 -2.18056 1.74312 0.00908 0.00424 0.0000988 0.00188 0.00216 0.00169 0.00124 0.0313 0.0105

3L:3350080-3350230:plus -13.449 5.50459 -2.30275 3.56881 -27.1939 -7.5 -9.73394 6.20833 -0.348624
0.000914 0.00132 0.0236 0.00699 0.0267 0.000614 0.00293 0.0015 0.0209
3L:3374160-3374310:minus -32.5102 2.86239 2.97248 1.99083 -17.3776 -9.53061 -15.6147 0.777778 -4.66055
0.0202 0.00366 0.00401 0.0144 0.00419 0.0031 0.0101 0.0131 0.0667
3L:3374160-3374310:plus -11.4082 5.22018 1.81651 5.57798 -18.7551 -17.7143 -11.4037 3.83333 7.84404
0.000251 0.00149 0.00619 0.0034 0.0144 0.00634 0.00406 0.0043 0.000701
3L:3376720-3376870:minus -21.8878 13.5306 14.4404 8.81651 -18.4184 -8.53061 -22.4771 0.75 -1.14679
0.00258 0.0000206 4.12e-06 0.000501 0.0101 0.00136 0.0321 0.0132 0.0272
3L:3376720-3376870:plus -31.8061 2.3211 13.4128 7.2844 -18.5612 -18.3469 -13.5046 5.52778 4.13761
0.0158 0.00446 0.0000141 0.00105 0.0119 0.0124 0.00649 0.00207 0.00408
3L:3377320-3377470:minus -24.2959 4.08257 2.10092 7.37615 -18.4898 -17.3469 -16.1376 3.25 4.10092
0.00781 0.00233 0.00556 0.000951 0.0116 0.0058 0.0111 0.00542 0.00413
3L:3377320-3377470:plus -30.7347 -2.26606 -2.94495 6.2844 -26.8571 -8.79592 -13.8349 -0.0972222
-0.651376 0.01 0.021 0.0284 0.00205 0.0198 0.0019 0.00699 0.0172 0.0235
3L:3377940-3378090:minus -31.6224 -2.46789 1.83486 5.06422 -27.4082 -9.94898 -14.4954 3.59722 8.11009
0.0137 0.0222 0.00614 0.00433 0.0321 0.00393 0.00808 0.00473 0.000597
3L:3377940-3378090:plus -31.1429 -1.65138 5.31193 5.16514 -8.71429 -8.0102 -20.1468 3.30556 0.311927
0.0108 0.0175 0.00156 0.00424 0.00159 0.000754 0.0211 0.0053 0.0169
3L:3380140-3380290:minus -31.3265 0.0458716 5.69725 5.49541 -8.53061 -19.5 -6.16514 1.77778
4.82569 0.0114 0.0101 0.00132 0.00355 0.0011 0.0199 0.00137 0.00932 0.00331
3L:3380140-3380290:plus -13.0408 -1.01835 -3.34862 5.49541 -17.0816 -7.72449 -14.633 -0.930556
1.52294 0.000576 0.0144 0.0318 0.00355 0.00334 0.00068 0.00832 0.0221 0.0117
3L:3384500-3384650:minus -31.4388 3.95413 3.49541 0.669725 -27.3061 -25.9796 -7.23853 1.63889 8.97248
0.0122 0.00244 0.00327 0.0225 0.0315 0.0214 0.00178 0.00978 0.000449
3L:3384500-3384650:plus -13.3367 2.77982 1.73394 -0.0642202 -18.9796 -0.540816 -21.156 1.5
0.816514 0.0008 0.00377 0.00638 0.0274 0.0155 0.00054 0.0253 0.0103 0.015
3L:3386540-3386690:minus -32.6633 0.100917 6.88073 2.70642 -27.0408 -17.2041 -16.6972 2.93056 0.183486
0.0216 0.00996 0.000768 0.0103 0.0242 0.00507 0.0122 0.00612 0.0176
3L:3386540-3386690:plus -31.6224 -1.92661 5.6422 7.73394 -18.7143 -8.79592 -14.0367 1.5 3.6789
0.0137 0.019 0.00135 0.000669 0.0136 0.0019 0.00731 0.0103 0.00506
3L:3386880-3387030:minus -32.1735 0.522936 0.53211 7.44037 -26.9286 -26.898 -8.79817 -5.29167 3.25688
0.018 0.00859 0.00971 0.000912 0.0214 0.0302 0.00246 0.0657 0.00632
3L:3386880-3387030:plus -22.3673 3.64286 -4.44037 1.20183 -27.7041 -8.94898 -15.578 2.40278 -0.678899
0.00341 0.00159 0.0427 0.0195 0.038 0.00203 0.01 0.00746 0.0237
3L:3389580-3389730:minus -21.9592 0.238532 -0.33945 1.92661 -27 -17.7143 -15.4771 0.402778 9.66055
0.00279 0.0095 0.0129 0.0148 0.0233 0.00634 0.00985 0.0147 0.000268
3L:3389580-3389730:plus -24.5204 6.07339 2.22018 7.33028 -17.3776 -9.5 -16.9633 2.875 -0.706422
0.00813 0.00102 0.00532 0.00103 0.00419 0.00294 0.0127 0.00626 0.024
3L:3398520-3398670:minus -40.2959 -0.33945 5.92661 3.0367 -8.56122 -17.4184 -17.0183 -4.36111 -0.220183
0.0389 0.0116 0.00119 0.00886 0.00112 0.00585 0.0129 0.0535 0.0201
3L:3398520-3398670:plus -22.7347 -0.862385 1.11009 6.21101 -18.1531 -18.0102 -17.2569 -7.86111
2.41284 0.00403 0.0137 0.00796 0.00213 0.00829 0.00812 0.0133 0.108 0.0082
3L:3399760-3399910:minus -13.2959 0.247706 -0.100917 3.81651 -26.7041 -18.2755 -9.38532 1.86111
8.62385 0.000775 0.00947 0.012 0.00626 0.0193 0.0112 0.00275 0.00905 0.00048
3L:3399760-3399910:plus -30.5918 0.110092 -0.761468 11.0183 -18.1939 -19.2755 -9.25688 -0.444444
-4.34862 0.00963 0.00993 0.0148 0.000052 0.0086 0.0185 0.00268 0.0192 0.0612
3L:3400880-3401030:minus -31.4286 -2.19266 2.55046 0.743119 0.877551 -36.0816 -14.3394 -8.16667 -0.550459
0.0119 0.0205 0.00471 0.0221 0.00019 0.0857 0.00782 0.114 0.0225
3L:3400880-3401030:plus -30.4796 -0.293578 4.3945 11.0275 -17.4898 -17.7143 -15 -0.833333
-0.834862 0.00941 0.0114 0.00229 0.0000401 0.0053 0.00634 0.00897 0.0215 0.0249
3L:3408920-3409070:minus -22.8163 4.11927 1.07339 9.59633 -27.1939 -9.45918 -16.7798 -3.19444 -1.21101
0.00418 0.0023 0.00806 0.000275 0.0267 0.00279 0.0124 0.0406 0.0277
3L:3408920-3409070:plus -31.4796 -2.33945 5.85321 5.51376 -18.1837 -9.79592 -11.211 -1.06944 1.77982
0.0128 0.0214 0.00123 0.00348 0.00835 0.00377 0.0039 0.023 0.0103

3L:3417240-3417390:minus -30.3673 1.09174 1.3578 2.06422 -19.0918 -18.7143 -0.899083 11.0278
-0.302752 0.00897 0.007 0.00728 0.0139 0.0163 0.014 0.000347 0.0000745 0.0208
3L:3417240-3417390:plus -21.7041 -2.21101 -2 -0.40367 -26.7041 -17.0102 -19.9174 0.722222 7.04587
0.00231 0.0206 0.0216 0.0302 0.0193 0.00453 0.0203 0.0133 0.00132
3L:3417980-3418130:minus 6.43878 0.605505 0.100917 5.59633 -7.89796 -18.7857 7.73394 2.58333 5.90826
3.32e-06 0.00834 0.0112 0.00331 0.000678 0.0146 0.0000255 0.00698 0.00198
3L:3417980-3418130:plus -22.5204 1.65138 4.42202 6.00917 -17.3776 -8.87755 -16.2385 1.05556 10.1284
0.00356 0.00571 0.00226 0.00255 0.00419 0.00198 0.0113 0.0119 0.000129
3L:3418560-3418710:minus -12.1531 -1.62385 4.14679 3.76147 -18.449 -9.20408 -17.844 -0.430556
10.1284 0.000352 0.0174 0.00252 0.00645 0.0107 0.00225 0.0146 0.0191 0.000129
3L:3418560-3418710:plus -12.2551 -2.04587 3.98165 5.49541 -26.9694 -8.23469 -0.394495 5.97222
3.6789 0.000379 0.0197 0.0027 0.00355 0.023 0.000875 0.00031 0.00168 0.00506
3L:3420320-3420470:minus -32.9184 -0.247706 -2.50459 1.61468 -19.051 -8.72449 -24.578 -0.305556
1.74312 0.0248 0.0112 0.025 0.0169 0.0162 0.00169 0.0446 0.0184 0.0105
3L:3420320-3420470:plus -32.6224 -0.220183 -2.04587 2.45872 -17.8163 -7.65306 -12.8899 1.76389
3.02752 0.0211 0.0111 0.0219 0.0116 0.00682 0.000647 0.00564 0.00937 0.00695
3L:342940-343090:minus -13 -1.80734 2.10092 11.2936 -18.1531 -19.051 -10.844 2.22222 1.63303
0.000563 0.0184 0.00556 0.0000139 0.00829 0.0165 0.00362 0.00796 0.0112
3L:342940-343090:plus -23.0408 0.990826 5.6789 1.7156 -18.4898 -16.3469 -10.0826 -2.26389 2.78899
0.00512 0.00726 0.00133 0.0162 0.0116 0.00416 0.00313 0.032 0.00745
3L:3431920-3432070:minus -22.8061 3.33028 2.6422 4.33945 -27.5306 -18.0102 -4.54128 -1.84722 5.38532
0.00414 0.00308 0.00455 0.00509 0.0351 0.00812 0.000871 0.0286 0.00267
3L:3431920-3432070:plus -13.8776 3.68367 1.87156 4.91743 -18.7551 -27.602 -11.2661 1.77778 -3.16514
0.0011 0.00144 0.00606 0.00447 0.0144 0.0416 0.00394 0.00932 0.0464
3L:3433080-3433230:minus -13.9184 0.12844 3.72477 1.40367 -17.7551 -8.65306 -8.65138 -1.83333 3.15596
0.00112 0.00987 0.003 0.0184 0.00658 0.00156 0.00239 0.0285 0.00659
3L:3433080-3433230:plus -31.6327 3.0367 -2.87156 5.49541 -18.7449 -8.5 -16.0459 -1.19444 -8.77982
0.0138 0.00343 0.0278 0.00355 0.0138 0.00126 0.0109 0.0239 0.159
3L:3434200-3434350:minus -14.5612 8.21101 2.82569 2.77064 -17.3061 -9.65306 -10.3486 5.08333 1.07339
0.00147 0.00034 0.00424 0.01 0.00389 0.0034 0.00329 0.00253 0.014
3L:3434200-3434350:plus -12.3265 0.137615 0.752294 3.21101 -17.449 -17.5 -18.9908 9.76389 3
0.000385 0.00984 0.009 0.00822 0.00484 0.00599 0.0174 0.000194 0.00702
3L:3437520-3437670:minus -21.7449 0.321101 1.63303 2.70642 -27.2347 -16.8265 -18.211 5.34722 2.25688
0.00236 0.00922 0.00661 0.0103 0.0283 0.0043 0.0154 0.00225 0.00853
3L:3437520-3437670:plus -31.7041 6.44037 -0.247706 2.7156 -26.7041 -18.0102 -13.789 5.56944
-0.504587 0.0146 0.000858 0.0126 0.0102 0.0193 0.00812 0.00692 0.00203 0.0221
3L:3438100-3438250:minus -31.4388 -1.76147 3.29358 0.137615 -17.2653 -18.7857 -12.5046 8.75 3.14679
0.0122 0.0181 0.00354 0.0259 0.00387 0.0146 0.00516 0.000374 0.00666
3L:3438100-3438250:plus -22.9184 -2.22018 1.3945 5.49541 -18.7449 -19.0102 -17.0092 3.23611 7.68807
0.00447 0.0207 0.00719 0.00355 0.0138 0.0159 0.0128 0.00545 0.000841
3L:3438440-3438590:minus -31.7041 0.422018 1.52294 4.55046 -18.6837 -27.6735 -21.1743 3.65278 6.12844
0.0146 0.0089 0.00687 0.00482 0.0126 0.0443 0.0254 0.00462 0.00174
3L:3438440-3438590:plus -14.6327 -5.66055 1.88073 7.63303 -18.9388 -9.7551 -13.6606 -0.888889
0.559633 0.0015 0.0524 0.00604 0.000745 0.0151 0.00358 0.00672 0.0218 0.016
3L:3438780-3438930:minus -21.7347 -3.10092 5.86239 6.69725 -18.4898 -9.45918 -4.55963 -4.93056 6.11927
0.00235 0.0265 0.00122 0.00156 0.0116 0.00279 0.000875 0.0608 0.00178
3L:3438780-3438930:plus -33.9286 -0.917431 8.04587 4.17431 -18.7551 -17.0102 -21.5596 -2.25
-0.642202 0.0303 0.014 0.000428 0.00537 0.0144 0.00453 0.0272 0.0319 0.0234
3L:3439560-3439710:minus -24.0714 3.64286 1.11927 2.99083 -18.7143 -17.7143 -6.41284 4.48611 5.90826
0.00731 0.00159 0.00793 0.00899 0.0136 0.00634 0.00146 0.00328 0.00198
3L:3439560-3439710:plus -23.3367 0.504587 0.513761 3.3578 -17.4898 -27.6327 -16.9174 6 4.88073
0.00614 0.00865 0.00977 0.00767 0.0053 0.043 0.0126 0.00166 0.00324
3L:3440400-3440550:minus -31.4796 1.66972 1.44954 9.23853 -18.449 -17.7857 -9.98165 0.333333 -1.11927
0.0128 0.00567 0.00705 0.000424 0.0107 0.0068 0.00307 0.0151 0.027

3L:3440400-3440550:plus -29.1837 -1.78899 2.20183 4.3945 -18.4898 -26.602 -14.6697 -1.09722 5.12844
0.00835 0.0183 0.00536 0.00499 0.0116 0.0248 0.00838 0.0232 0.00301
3L:3441960-3442110:minus -30.4388 3.27523 6.80734 9.7156 -17.8163 -17.051 -10.4037 -4.72222 5.74312
0.00923 0.00314 0.000794 0.000242 0.00682 0.00479 0.00332 0.058 0.00215
3L:3441960-3442110:plus -32.2857 -1.24771 0.229358 2.3578 -27.3061 -18.0816 -11.7248 1.125 1.54128
0.0185 0.0155 0.0107 0.0121 0.0315 0.00934 0.00434 0.0117 0.0115
3L:3446440-3446590:minus -30.4796 -2.11009 -1.65138 3.70642 -8.60204 -25.8265 -15.1284 -4 1.89908
0.00941 0.0201 0.0195 0.0066 0.00125 0.0212 0.00921 0.0492 0.0098
3L:3446440-3446590:plus -32.9694 -1.58716 2.29358 4.55046 -27.4592 -17.3469 -15.3394 0.125 3.66972
0.0254 0.0172 0.00518 0.00482 0.0329 0.0058 0.0096 0.0161 0.00514
3L:3447580-3447730:minus -30.4082 0.990826 -1.90826 1.58716 -17.9796 -17.8265 -19.4954 -0.277778
-1.52294 0.00908 0.00726 0.021 0.017 0.00726 0.00699 0.0189 0.0182 0.0295
3L:3447580-3447730:plus -21.8469 -2.3578 -3.91743 2.26606 -18.7143 -26.898 -22.8807 -3.83333 -0.431193
0.00247 0.0215 0.0371 0.0126 0.0136 0.0302 0.0344 0.0473 0.0214
3L:3448220-3448370:minus -22.398 4.52294 4.77064 6.85321 -8.26531 0.0510204 -6.20183 -2.56944
-6.81651 0.00344 0.00197 0.00196 0.00137 0.000934 0.000349 0.00138 0.0346 0.109
3L:3448220-3448370:plus -21.7041 3.22936 11.789 4.07339 -27.0816 -18.5 -15.4495 2.47222 5.58716
0.00231 0.0032 0.0000494 0.00562 0.0243 0.0131 0.0098 0.00727 0.00237
3L:3450320-3450470:minus -31.2551 5.88073 -0.944954 -1.14679 -8.86735 -25.8265 2.51376 -0.513889
5.90826 0.0113 0.00112 0.0157 0.0384 0.00173 0.0212 0.00014 0.0196 0.00198
3L:3450320-3450470:plus -23.3776 1.06422 -1.7156 5.49541 -17.4184 -0.132653 -24.4312 0.0416667
-9.13761 0.00619 0.00707 0.0198 0.00355 0.00457 0.000444 0.0437 0.0165 0.17
3L:3450820-3450970:minus -32.7449 -5.0367 -1.77982 1.69725 -18.9796 -16.9796 -24.8165 -3.125 2.58716
0.0228 0.0446 0.0202 0.0163 0.0155 0.0044 0.046 0.0399 0.00779
3L:3450820-3450970:plus -23.2551 -1.33028 -2.02752 1.3578 -17.449 -28.9286 -11.3394 2.68056 -0.605505
0.00583 0.0159 0.0218 0.0186 0.00484 0.0717 0.004 0.00673 0.023
3L:3453440-3453590:minus -32.7755 0.926606 1.34862 5.49541 -27.1939 -19.0816 -18.6697 4.125 -1.44037
0.0234 0.00743 0.00731 0.00355 0.0267 0.017 0.0166 0.00382 0.029
3L:3453440-3453590:plus -32.8469 -1.44954 5.77064 3.04587 -18.3469 -7.72449 -8.88991 4.19444 -1.22936
0.0243 0.0165 0.00128 0.00883 0.00894 0.00068 0.00251 0.00371 0.0279
3L:3455920-3456070:minus -24.2245 6.54128 -2.29358 5.74312 -26.2245 -9.86735 -11.5046 0.25 1.43119
0.00763 0.000818 0.0236 0.00302 0.0175 0.00391 0.00414 0.0155 0.0121
3L:3455920-3456070:plus -32.8878 6.38532 -1.94495 6.44037 -17.2653 9.79592 -17.7431 12.125 5.6055
0.0247 0.000882 0.0213 0.00185 0.00387 0.0000183 0.0144 0.0000274 0.00232
3L:3461220-3461370:minus -23.8163 0.614679 0.715596 1.56881 -18.051 0.0510204 -14.7339 5.20833
-0.137615 0.00682 0.00832 0.00912 0.0172 0.00746 0.000349 0.0085 0.00239 0.0194
3L:3461220-3461370:plus -23.8878 -2.10092 -4.37615 2.41284 11.6939 -17.4184 -22.6514 3.84722 -4.3578
0.00692 0.02 0.042 0.0118 1.17e-06 0.00585 0.0331 0.00428 0.0614
3L:346860-347010:minus -24.4592 -4.41284 8.0367 3.94495 -7.89796 -18.3469 -17.8991 4.54167 12.2752
0.00802 0.0378 0.00043 0.00595 0.000678 0.0124 0.0147 0.0032 0.0000141
3L:346860-347010:plus -21.6224 -0.46789 -1.42202 2.55046 -18.7551 -17.0102 -8.69725 0.513889 3.46789
0.00213 0.0121 0.0182 0.0112 0.0144 0.00453 0.00242 0.0142 0.00582
3L:3471320-3471470:minus -24.0612 0.40367 8.24771 2.66972 -18.6837 -18.0816 -2.76147 5.08333 1.90826
0.00726 0.00896 0.000384 0.0106 0.0126 0.00934 0.000534 0.00253 0.00974
3L:3471320-3471470:plus -32.3367 0.605505 -0.0275229 5.57798 -19.2755 -9.02041 -17 3.09722 -3.01835
0.0187 0.00834 0.0117 0.0034 0.0164 0.00213 0.0128 0.00575 0.0448
3L:3472080-3472230:minus -20.6224 -5.90816 5.84404 3.79817 10.9898 -16.0102 -4.34862 0.694444 -2.25688
0.0017 0.0134 0.00124 0.00629 3.28e-06 0.00406 0.000824 0.0134 0.0357
3L:3472080-3472230:plus -32.7755 3.23853 -0.926606 4.37615 19.9082 19.3469 -2.55963 9.16667
-2.63303 0.0234 0.00318 0.0156 0.00504 8.13e-07 5.04e-07 0.000508 0.000288 0.0404
3L:347360-347510:minus -31.551 13.5306 2.9633 2.66055 -26.2653 -18.8571 -12.2385 4.01389 -2.05505
0.0134 0.0000206 0.00403 0.0106 0.018 0.0151 0.00486 0.004 0.0338
3L:347360-347510:plus -23.8776 13.4592 1.17431 9.59633 -27.3469 9.57143 -15.1927 1.20833 -0.0275229
0.00687 0.000064 0.00778 0.000275 0.0318 0.0000274 0.00932 0.0113 0.0186

3L:347620-347770:minus -22.0306 -3.30275 -1.77064 2.08257 -7.67347 -28.3367 -11.8991 4.81944 9.14679 0.00293 0.028 0.0202 0.0137 0.00055 0.0606 0.00451 0.00284 0.000414
3L:347620-347770:plus -24.449 -2.86239 0.688073 4.49541 -18.1224 -8.45918 -15.9541 10.875 2.88073 0.00802 0.0248 0.0092 0.00487 0.00782 0.00111 0.0108 0.0000846 0.00727
3L:3485040-3485190:minus -23.449 6.22936 -0.357798 9.88073 -19.0918 -8.45918 -16.3211 5.47222 5.70642 0.00631 0.000949 0.013 0.000183 0.0163 0.00111 0.0115 0.00212 0.00222
3L:3485040-3485190:plus -30.4082 0.458716 9.29358 10.5963 -8.89796 -18.0816 -17.9817 1.95833 0.0183486 0.00908 0.00879 0.000214 0.0001 0.00189 0.00934 0.0149 0.00875 0.0183
3L:3485500-3485650:minus -31.7347 0.247706 5.3945 1.53211 -27.1939 -26.602 -20.2661 7.29167 1.88073 0.0149 0.00947 0.00151 0.0174 0.0267 0.0248 0.0216 0.000863 0.00987
3L:3485500-3485650:plus -28.1429 0.715596 0.587156 7.90826 -16.7143 0.0918367 -19.9541 -6.52778 1.11009 0.00831 0.00802 0.00952 0.000611 0.00319 0.000323 0.0204 0.0845 0.0138
3L:3515940-3516090:minus -23.2245 3.81651 10.5229 3.86239 -18.7551 9.86735 -14.3761 -1.26389 3.05505 0.00576 0.00258 0.000104 0.00622 0.0144 0.0000132 0.00788 0.0243 0.00692
3L:3515940-3516090:plus -40.7245 -1.88991 0.311927 -1.30275 -17.1837 -37.449 -17.6881 5.69444 -2.33945 0.0428 0.0188 0.0104 0.0404 0.00366 0.164 0.0143 0.00191 0.0367
3L:3542660-3542810:minus -21.8163 2.10092 -0.798165 9.08257 -17.602 -9.5 -17.1835 8.70833 9.92661 0.00244 0.00484 0.015 0.000444 0.00547 0.00294 0.0132 0.000384 0.000193
3L:3542660-3542810:plus -13.2551 0.926606 -0.788991 2.3211 -17.2245 -9.68367 -18.0275 -8.48611 -0.522936 0.000751 0.00743 0.0149 0.0123 0.00384 0.00342 0.015 0.121 0.0222
3L:3543600-3543750:minus -20.4388 5.3211 1.83486 7.7156 -8.7449 -36.0816 -11.4954 -0.944444 -0.0733945 0.00167 0.00142 0.00614 0.000691 0.00161 0.0857 0.00413 0.0222 0.0188
3L:3543600-3543750:plus -21.3265 -2.45872 6.63303 2.27523 1.87755 -17.7857 -12.945 -2.55556 -4.57798 0.00193 0.0222 0.000861 0.0125 0.0000492 0.0068 0.00571 0.0345 0.0651
3L:3544560-3544710:minus -32.0714 0.66055 2.65138 6.29358 -8.53061 1.05102 -27.1009 -2.66667 -3.56881 0.0176 0.00818 0.00453 0.00202 0.0011 0.000107 0.0613 0.0355 0.0505
3L:3544560-3544710:plus -13.4796 -0.357798 2.10092 6.06422 -8.89796 -19.3061 -8.23853 -0.0277778 6.37615 0.000935 0.0116 0.00556 0.00239 0.00189 0.0187 0.00221 0.0169 0.00159
3L:3545140-3545290:minus -22.3265 -1.10092 -0.880734 3.17431 -9.27551 -17.7551 -11.5046 1.72222 6.18349 0.00337 0.0148 0.0154 0.00842 0.00284 0.00643 0.00414 0.0095 0.00167
3L:3545140-3545290:plus -2.7449 0 0.192661 7.80734 -18.8265 -27.2653 -9.56881 -0.972222 2.88073 0.0000376 0.0103 0.0109 0.00063 0.0149 0.0333 0.00284 0.0224 0.00727
3L:3545740-3545890:minus -33.3367 -4.06422 2.88991 7.21101 -17.6735 -18.4184 -21.8073 8.48611 5.66055 0.0281 0.0344 0.00414 0.0011 0.00572 0.0127 0.0285 0.00044 0.00227
3L:3545740-3545890:plus -24.5918 -0.559633 -1.27523 1.92661 -18.4184 -17.5714 -22.2936 5.79167 1.72477 0.00821 0.0124 0.0174 0.0148 0.0101 0.00616 0.0311 0.00183 0.0106
3L:3556240-3556390:minus -13.0408 1.04587 5.00917 4.3211 -26.9694 -27.7551 -9.51376 7.97222 7.42202 0.000576 0.00712 0.00177 0.00514 0.023 0.0458 0.00281 0.000593 0.00106
3L:3556240-3556390:plus -31.5102 -0.761468 1.11927 4.61468 -18.1224 -17.8571 -15.0826 3.29167 -0.899083 0.013 0.0133 0.00793 0.00475 0.00782 0.00719 0.00912 0.00533 0.0254
3L:3556760-3556910:minus -33.2959 -2.44954 2.37615 9.24771 -17.3776 -18.8571 -10.7523 8.18056 -1.77982 0.0279 0.0221 0.00503 0.000409 0.00419 0.0151 0.00355 0.000526 0.0314
3L:3556760-3556910:plus -30.8878 5.22936 3.80734 4.31193 -17.3061 -9.53061 -8.66055 0.263889 7.6422 0.0103 0.00148 0.0029 0.00516 0.00389 0.0031 0.0024 0.0154 0.000893
3L:3593720-3593870:minus -32.1939 -0.944954 -6.37615 4.78899 -26.9694 -17.6429 0.779817 5.94444 -1.01835 0.0181 0.0141 0.0693 0.00456 0.023 0.00619 0.000234 0.0017 0.0263
3L:3593720-3593870:plus -30.2143 -2.87156 1.88991 2.86239 -7.60204 -8.94898 -22.6514 -2.5 -2.2844 0.0088 0.0249 0.00602 0.00954 0.000466 0.00203 0.0331 0.034 0.0361
3L:361300-361450:minus -3.36735 8.77982 -1.00917 0.733945 -26.898 -27.4898 -20.0734 3.33333 9.45872 0.000063 0.000252 0.016 0.0222 0.0203 0.0363 0.0209 0.00525 0.000337
3L:361300-361450:plus -22.1122 0.366972 16.2202 8.81651 1.14286 -8.45918 -13.9174 1.26389 -4.11009 0.00304 0.00908 1.24e-07 0.000501 0.000113 0.00111 0.00712 0.0111 0.0571
3L:3765820-3765970:minus -13.898 4.7156 -2.31193 9.7156 -18.051 -8.5 -14.4404 2.98611 -3.23853 0.00111 0.00183 0.0237 0.000242 0.00746 0.00126 0.00799 0.006 0.047

3L:3765820-3765970:plus -31.4388 1.05505 -0.633028 2.83486 -28.5306 -19.5714 -18.7615 7.29167
11.3853 0.0122 0.00709 0.0142 0.0097 0.061 0.0202 0.0168 0.000863 0.0000815
3L:3787640-3787790:minus -31.4796 -2.95413 10.2477 9.59633 -28.7959 -8.45918 -21.9358 2.06944 3.42202
0.0128 0.0255 0.000123 0.000275 0.0641 0.00111 0.0291 0.00841 0.00597
3L:3787640-3787790:plus -13.0714 -2.86239 -0.541284 3.27523 -8.96939 -37.3367 -11.4404 1.95833
11.3853 0.000594 0.0248 0.0138 0.00796 0.00226 0.149 0.00409 0.00875 0.0000815
3L:3788000-3788150:minus -32.1429 2.12844 10.5596 3.90826 10.6531 0.0204082 -11.156 6.61111
7.42202 0.0179 0.00479 0.000102 0.00603 0.0000102 0.000377 0.00385 0.00123 0.00106
3L:3788000-3788150:plus -4.56122 3.97959 2.65138 3.18349 11.6939 -17.7857 -10.2385 1.375 11.3853
0.000162 0.000669 0.00453 0.00837 1.17e-06 0.0068 0.00322 0.0107 0.0000815
3L:3806480-3806630:minus -22.9184 13.3878 12.1743 5.22018 -18.7857 -9.30612 -3.11009 4.48611 1.74312
0.00447 0.0000869 0.0000382 0.00418 0.0148 0.00257 0.000585 0.00328 0.0105
3L:3806480-3806630:plus -32.7041 -0.100917 -2.21101 1.43119 -9.5 -8.42857 -19.7156 2.06944 -0.440367
0.0222 0.0107 0.023 0.0181 0.00293 0.00104 0.0196 0.00841 0.0216
3L:3810080-3810230:minus -4.95918 -0.889908 1.49541 0.954128 -27 -26.8265 -5.73394 -0.722222
-0.87156 0.000213 0.0138 0.00694 0.0208 0.0233 0.0285 0.00122 0.0208 0.0252
3L:3810080-3810230:plus -22.5204 13.3878 4.6422 5.22018 -28.2653 -8.72449 -12.1651 0.833333 1.93578
0.00356 0.0000869 0.00206 0.00418 0.0537 0.00169 0.00478 0.0128 0.00969
3L:3811180-3811330:minus -23.6327 3.11009 1.66972 6.00917 -17.4184 -8.87755 -23.367 5.375 -5.11927
0.00661 0.00334 0.00652 0.00255 0.00457 0.00198 0.0372 0.00222 0.0746
3L:3811180-3811330:plus -21.8571 -0.587156 2.05505 6.3578 20.2041 -8.45918 -19.8716 -2.80556
2.05505 0.00249 0.0126 0.00566 0.00195 3.64e-07 0.00111 0.0201 0.0368 0.00914
3L:3814080-3814230:minus -31.7347 13.4592 15.4495 1.99083 -17.7143 -17.7857 -12.7615 3 -0.87156
0.0149 0.000064 9.17e-07 0.0144 0.00627 0.0068 0.00547 0.00597 0.0252
3L:3814080-3814230:plus -23.9286 13.4592 0.422018 7.90826 -8.56122 -9.23469 -7.77982 -4.375 -6.37615
0.00701 0.000064 0.0101 0.000611 0.00112 0.00238 0.00201 0.0537 0.0975
3L:3817060-3817210:minus -21.5918 -0.0366972 4.61468 7.20183 -17.2245 -35.1531 -11.0092 -2.38889
5.95413 0.00209 0.0104 0.00209 0.00112 0.00384 0.0753 0.00374 0.0331 0.00192
3L:3817060-3817210:plus -41.8163 1.58716 -1.14679 6.00917 -17.449 -17.5 -34.2844 7.72222 -0.394495
0.0662 0.00584 0.0167 0.00255 0.00484 0.00599 0.15 0.000682 0.0212
3L:3818620-3818770:minus -13.1531 11.1468 -2.53211 3.04587 -8.89796 -9.02041 -9.55963 2.26389 -1.56881
0.00067 0.000075 0.0252 0.00883 0.00189 0.00213 0.00284 0.00784 0.0298
3L:3818620-3818770:plus -14.2959 5.68807 -0.0550459 2.27523 -27.0408 -17.9388 -19.0183 4.29167
0.669725 0.00129 0.00122 0.0118 0.0125 0.0242 0.00738 0.0175 0.00356 0.0155
3L:3892560-3892710:minus -4.0102 2.44037 3.33028 4.22018 -26.4898 -8.72449 -11.8349 -1.22222 0.53211
0.000121 0.00427 0.00349 0.00531 0.0183 0.00169 0.00445 0.024 0.0161
3L:3892560-3892710:plus -34.0714 5.88991 4.65138 3.26606 -18.449 -9.60204 -21.211 2.38889 1.70642
0.031 0.00111 0.00206 0.00802 0.0107 0.00334 0.0256 0.00749 0.0108
3L:3893160-3893310:minus -13.9592 -0.495413 -1.9633 0.119266 -18.1531 -36.1122 -17.5596 -5.45833
6.91743 0.00113 0.0122 0.0214 0.026 0.00829 0.0878 0.014 0.068 0.00136
3L:3893160-3893310:plus -22.6224 -2.48624 -0.110092 4.22018 -17.1122 -18.6429 -17.8807 4.22222
2.27523 0.00371 0.0223 0.012 0.00531 0.00341 0.0139 0.0147 0.00367 0.00846
3L:3902020-3902170:minus -13.2245 1.66055 0.788991 2.09174 -18.9796 -17.9388 -16.6147 -2.55556 4
0.000738 0.00569 0.00889 0.0137 0.0155 0.00738 0.012 0.0345 0.0043
3L:3902020-3902170:plus -41.4082 0.93578 -2.7156 7.12844 -18.7449 -16.9796 -22.6881 3.51389 -4.33945
0.0557 0.00741 0.0266 0.00118 0.0138 0.0044 0.0333 0.00489 0.0609
3L:3902540-3902690:minus -11.8878 1.87156 3.77064 3.14679 -17.0816 -17.898 -6.7156 12.3472 4.7156
0.00029 0.00526 0.00294 0.00847 0.00334 0.00726 0.00158 0.0000218 0.00345
3L:3902540-3902690:plus -14.1122 0.119266 2.24771 2.77064 -8.16327 -17.7143 -8.54128 7.61111 1.73394
0.00119 0.0099 0.00527 0.01 0.000873 0.00634 0.00234 0.000726 0.0106
3L:3903320-3903470:minus -23.1837 -0.0550459 1.34862 7.6422 -26.9286 -18.3469 -21.3578 8.61111
0.816514 0.00553 0.0105 0.00731 0.000721 0.0214 0.0124 0.0262 0.000408 0.015
3L:3903320-3903470:plus -32.5102 7.99083 -0.724771 2.77064 20.1735 -9.16327 4.88073 -4.26389
3.41284 0.0202 0.000384 0.0146 0.01 5.68e-07 0.00223 0.0000601 0.0523 0.006

3L:3909540-3909690:minus -22.6633 13.4592 0.972477 5.74312 -25.9694 -26.5306 -20.0826 5.47222 4.78899
0.00384 0.000064 0.00835 0.00302 0.017 0.023 0.0209 0.00212 0.00336
3L:3909540-3909690:plus -13.4796 13.4592 0.0733945 3.41284 -9.27551 -18.3469 -20.2569 4.93056
-0.229358 0.000935 0.000064 0.0113 0.00756 0.00284 0.0124 0.0215 0.00271 0.0202
3L:3937940-3938090:minus -31.5816 4.10092 -1.65138 -1 -8.7449 -0.173469 -15.6055 2.65278 -2.94495
0.0135 0.00231 0.0195 0.0367 0.00161 0.000456 0.0101 0.0068 0.0441
3L:3937940-3938090:plus -33.0408 14.4128 -1.7156 0.743119 -17.7449 -8.45918 -22.789 2.58333 3.05505
0.0264 5.49e-06 0.0198 0.0221 0.0063 0.00111 0.0339 0.00698 0.00692
3L:3940540-3940690:minus -31.6633 -0.93578 5.46789 6.57798 -17.449 -9.5 -25.7339 -0.638889 -5.97248
0.0141 0.014 0.00146 0.00167 0.00484 0.00294 0.0518 0.0203 0.0883
3L:3940540-3940690:plus -32.3776 2 3.86239 5.57798 -19.0102 -7.72449 -25.5321 8.06944 5.33028
0.0189 0.00502 0.00283 0.0034 0.0157 0.00068 0.0505 0.000561 0.00273
3L:3942440-3942590:minus -23.0408 5.33945 9.56881 9.7156 -7.7449 -19.1531 -16.7064 -2.20833 9.75229
0.00512 0.00142 0.000183 0.000242 0.000575 0.0177 0.0122 0.0315 0.000241
3L:3942440-3942590:plus -22.9184 6.78899 11.1193 2.83486 -18.1531 -27.6327 -16.7798 -4.04167 -0.394495
0.00447 0.000723 0.0000737 0.0097 0.00829 0.043 0.0124 0.0497 0.0212
3L:3942740-3942890:minus -31.6633 4.52294 7.63303 2.15596 -17.7857 -10.0918 -17.0275 8.36111 -2.42202
0.0141 0.00197 0.000531 0.0133 0.00676 0.00404 0.0129 0.000474 0.0378
3L:3942740-3942890:plus -20.7755 1.82569 9.65138 1.85321 -8.63265 -17.8265 -17.8349 1.47222 5.38532
0.00176 0.00535 0.000175 0.0153 0.0013 0.00699 0.0146 0.0104 0.00267
3L:4003340-4003490:minus -39.5204 -0.761468 0.678899 1.76147 -26.5306 -18.3061 -20.789 2.54167
-0.706422 0.0334 0.0133 0.00923 0.0159 0.0185 0.0114 0.0237 0.00709 0.024
3L:4003340-4003490:plus -22.8571 2.98165 13.9174 5.86239 10.3571 -8.72449 -12.8807 -8.06944 -0.53211
0.00427 0.0035 8.08e-06 0.00282 0.0000208 0.00169 0.00563 0.112 0.0222
3L:4021340-4021490:minus -33.2551 2.17431 -3.14679 1.3211 -27.1531 -7.72449 -24.1743 4.41667 4.19266
0.0276 0.00471 0.03 0.0189 0.0249 0.00068 0.0421 0.00338 0.00401
3L:4021340-4021490:plus -22.4388 0.66055 6.51376 1.62385 -8.67347 9.30612 -12.8257 7.11111 3.49541
0.00348 0.00818 0.000912 0.0168 0.00154 0.0000398 0.00555 0.000949 0.0057
3L:4040320-4040470:minus -31.1735 3.05505 5.74312 3.78899 -8.93878 -18.0102 -13.7064 -1.625 -1.01835
0.0109 0.00341 0.00129 0.00632 0.00216 0.00812 0.00679 0.0269 0.0263
3L:4040320-4040470:plus -30.1429 1.25688 6.89908 2.88991 20.4694 -9.53061 -14.1743 -0.319444
-5.55046 0.00866 0.00659 0.000762 0.00942 1.65e-07 0.0031 0.00754 0.0184 0.0811
3L:4073980-4074130:minus -23.5204 -1.86239 9.06422 10.7615 -7.60204 -18.0102 -10.844 12.6667 1.95413
0.00645 0.0187 0.000245 0.0000826 0.000466 0.00812 0.00362 0.0000153 0.00964
3L:4073980-4074130:plus -32.8163 -2.3945 -6.37615 -1.05505 -18.7551 -8.94898 -16.8807 2.27778 9.45872
0.024 0.0218 0.0693 0.0374 0.0144 0.00203 0.0126 0.0078 0.000337
3L:4102680-4102830:minus -30.8061 1.70642 2.6422 7 -26.6633 -26.5612 7.23853 2.06944 5.38532
0.0102 0.00559 0.00455 0.00132 0.0189 0.0237 0.0000291 0.00841 0.00267
3L:4102680-4102830:plus -40.7449 3.75229 1.79817 1.77982 -19.2755 -17.051 -24.3853 2.76389 -0.908257
0.0431 0.00264 0.00623 0.0158 0.0164 0.00479 0.0434 0.00652 0.0255
3L:4103940-4104090:minus -23.7653 0.183486 9.74312 2.55963 -9.27551 -27.6327 2.7156 9.52778 1.90826
0.00675 0.00968 0.000166 0.0111 0.00284 0.043 0.00013 0.000227 0.00974
3L:4103940-4104090:plus -22.398 -0.33945 2.11927 5.59633 -18.8265 -8.23469 0.440367 7.41667 6.18349
0.00344 0.0116 0.00553 0.00331 0.0149 0.000875 0.000255 0.000807 0.00167
3L:4108300-4108450:minus -22.8469 6.00917 -0.155963 3.78899 -26.9388 -17.051 -9.37615 1.19444
1.44037 0.00424 0.00105 0.0122 0.00632 0.0215 0.00479 0.00274 0.0114 0.012
3L:4108300-4108450:plus -14.5204 3.30275 -1.49541 0.568807 -27.4082 -26.602 -2.84404 -0.486111
9.04587 0.00145 0.00311 0.0186 0.0232 0.0321 0.0248 0.000546 0.0194 0.000423
3L:4108600-4108750:minus -21.7041 1.3945 13.1743 3.90826 -8.16327 -17.7551 -18.1009 -1.20833 -0.449541
0.00231 0.00627 0.0000172 0.00603 0.000873 0.00643 0.0152 0.0239 0.0216
3L:4108600-4108750:plus -32.0306 7.09174 1.11927 2.82569 -28 -17.5 -10.0183 0.305556 -2.53211
0.0172 0.000619 0.00793 0.00976 0.0444 0.00599 0.00309 0.0152 0.0392
3L:4109820-4109970:minus -31.551 0.844037 -2.31193 3.17431 -18.449 -19.5714 -15.2202 3.63889 4.33028
0.0134 0.00766 0.0237 0.00842 0.0107 0.0202 0.00937 0.00465 0.00385

3L:4109820-4109970:plus -41.3265 -0.944954 6.09174 2.16514 -28.2347 -17.9796 -17.5046 4.18056
-0.137615 0.0541 0.0141 0.00111 0.0132 0.051 0.00764 0.0139 0.00373 0.0194
3L:4118000-4118150:minus -30.3673 4.49541 -4.2844 5.91743 -18.5204 -8.23469 -12.8532 11.7083 -1.25688
0.00897 0.00199 0.041 0.00267 0.0117 0.000875 0.00559 0.0000409 0.028
3L:4118000-4118150:plus -13.4082 0.899083 -3.13761 2.24771 -27.4592 -7.68367 -16.8073 8.625 5.22936
0.000867 0.00751 0.03 0.0127 0.0329 0.000649 0.0124 0.000404 0.00288
3L:4119220-4119370:minus -14.4796 3.64286 4.11927 9.76147 -18.6429 -17.9388 -18.4587 -3.54167 2.15596
0.00142 0.00159 0.00255 0.000212 0.0121 0.00738 0.016 0.0442 0.00881
3L:4119220-4119370:plus -33.0408 2.51376 -2.54128 1.43119 -18.7245 -7.42857 -28.3119 2.83333 5.74312
0.0264 0.00415 0.0253 0.0181 0.0136 0.000567 0.0717 0.00635 0.00215
3L:4120680-4120830:minus -21.8571 -1.00917 2.77064 4.22018 -25.9286 -8.65306 -14.3394 -3.15278 6.04587
0.00249 0.0144 0.00433 0.00531 0.0168 0.00156 0.00782 0.0402 0.00181
3L:4120680-4120830:plus 5.29592 0.40367 10.0459 3.17431 -28.0102 -36.8163 -1.6422 -2.29167 4.78899
0.0000116 0.00896 0.000138 0.00842 0.0449 0.12 0.000409 0.0322 0.00336
3L:4121820-4121970:minus -20.6633 4.0367 2.52294 2.66055 -27.2755 -8.42857 -16.3394 -0.763889
2.94495 0.00173 0.00237 0.00476 0.0106 0.0302 0.00104 0.0115 0.0211 0.00714
3L:4121820-4121970:plus -31.4388 1.25688 -1.84404 5.85321 -25.9694 -8.72449 -19.7798 4.625 -0.513761
0.0122 0.00659 0.0206 0.00287 0.017 0.00169 0.0198 0.00309 0.0221
3L:4122440-4122590:minus -11.6633 1.46789 3.83486 2.92661 -26.6327 -17.3469 0.87156 -3.02778 1
0.000261 0.0061 0.00287 0.00931 0.0188 0.0058 0.000228 0.0389 0.0143
3L:4122440-4122590:plus -22.449 4.6789 11.8073 7.20183 -17.4898 -9.23469 -24.9633 0.902778 -2.68807
0.0035 0.00185 0.0000487 0.00112 0.0053 0.00238 0.0469 0.0125 0.041
3L:4122840-4122990:minus -32.4388 9.68807 -4.58716 1.41284 -27.0816 -17.051 -14.9908 1.88889 4.11927
0.0195 0.000156 0.0444 0.0183 0.0243 0.00479 0.00896 0.00896 0.00411
3L:4122840-4122990:plus -14.3776 0.321101 1.90826 3.3578 -17.9796 -9.53061 -7.48624 -0.958333
0.201835 0.00134 0.00922 0.00598 0.00767 0.00726 0.0031 0.00189 0.0223 0.0175
3L:4123640-4123790:minus -22.6633 6.22018 -1.08257 2.16514 -17.7449 -26.898 -10.7339 5.97222 5.74312
0.00384 0.000954 0.0164 0.0132 0.0063 0.0302 0.00354 0.00168 0.00215
3L:4123640-4123790:plus -41.6224 -1.33028 -3.95413 4.79817 -27.2653 -16.3469 -12.156 7.06944 5.76147
0.0628 0.0159 0.0375 0.00453 0.0302 0.00416 0.00477 0.00097 0.0021
3L:4134000-4134150:minus -32.551 4.49541 4.41284 3.61468 -17.6429 -18.2041 0.798165 -3.09722 1.48624
0.0207 0.00199 0.00227 0.0068 0.00562 0.0103 0.000233 0.0396 0.0118
3L:4134000-4134150:plus -32.1837 -1.02752 -1.98165 2.33945 -18.0816 -8.38776 -20.7156 -3.22222 -2.26606
0.0181 0.0145 0.0215 0.0122 0.00759 0.00101 0.0234 0.0408 0.0359
3L:4134520-4134670:minus -32.2143 0.311927 4.46789 0.944954 10.6531 -26.898 -18.633 -1.13889 0.66055
0.0182 0.00925 0.00222 0.0209 0.0000102 0.0302 0.0165 0.0235 0.0156
3L:4134520-4134670:plus -30.7041 -0.844037 -0.0366972 -2.22018 -18.1531 -8.42857 -13.7798 2.58333
-4.6055 0.00993 0.0136 0.0117 0.0551 0.00829 0.00104 0.00691 0.00698 0.0656
3L:4135980-4136130:minus -22.449 -1.55963 3.38532 6.00917 10.6224 -8.68367 -4.56881 -2.91667 -0.917431
0.0035 0.017 0.00342 0.00255 0.0000113 0.00157 0.000877 0.0378 0.0256
3L:4135980-4136130:plus -14.7041 -1.44037 -1.22018 5.55046 -18.7857 -18.0816 -19.8349 8.41667 3.88991
0.00154 0.0164 0.0171 0.00345 0.0148 0.00934 0.02 0.000458 0.00467
3L:4138880-4139030:minus -32.7755 13.6514 1.25688 4.05505 -19.0102 -19.1531 -9.73394 -0.0555556
-0.550459 0.0234 0.0000126 0.00755 0.00566 0.0157 0.0177 0.00293 0.017 0.0225
3L:4138880-4139030:plus -30.551 1.08257 4.52294 8.98165 10.9898 -17.051 -14.2569 -6.13889 2.21101
0.00959 0.00703 0.00217 0.000461 3.28e-06 0.00479 0.00768 0.0783 0.00862
3L:4170320-4170470:minus -33.6327 1.09174 3.33028 4.65138 -18.9796 -17.8571 -18.2202 7.98611 -2.22018
0.029 0.007 0.00349 0.00471 0.0155 0.00719 0.0155 0.000588 0.0355
3L:4170320-4170470:plus -11.8878 -2.44954 1.30275 2.88991 10.6224 9.60204 -7.30275 -1.44444 -0.743119
0.00029 0.0221 0.00743 0.00942 0.0000113 0.0000248 0.00181 0.0256 0.0243
3L:4186720-4186870:minus -21.4082 0.816514 10.3119 1.47706 -17.7551 9.45918 -18.2477 2.22222 3.46789
0.00198 0.00773 0.000118 0.0178 0.00658 0.0000356 0.0155 0.00796 0.00582
3L:4186720-4186870:plus -31.8776 -0.330275 7.98165 5.66055 -8.63265 -28.1122 -9.3578 -2.09722 -4
0.0163 0.0115 0.000442 0.00317 0.0013 0.0565 0.00273 0.0306 0.0558

3L:4193940-4194090:minus -23.4184 1.83486 -2.49541 6.12844 -17.6837 -0.173469 -32.7706 4.51389
-3.76147 0.00624 0.00533 0.025 0.00231 0.0058 0.000456 0.129 0.00324 0.0523
3L:4193940-4194090:plus -14.5204 -4.25688 14.422 7.29358 -18.3776 -17.1224 -20.1743 -3.33333 2.54128
0.00145 0.0362 4.18e-06 0.00104 0.00926 0.00499 0.0212 0.042 0.00787
3L:4221500-4221650:minus -32.2245 3.90826 2.73394 2.06422 -17.2653 -8.79592 -11.2202 6.01389 6.04587
0.0183 0.00249 0.00439 0.0139 0.00387 0.0019 0.0039 0.00165 0.00181
3L:4221500-4221650:plus -32.8367 -6.19266 6.59633 5.55046 -7.93878 -17.2755 -14.6147 0.333333 -0.706422
0.024 0.0599 0.000876 0.00345 0.000767 0.00544 0.00829 0.0151 0.024
3L:4221880-4222030:minus -14.1837 1.22936 -3.33945 4.7156 -18.4898 -28.7041 -4.88073 -1.40278 5.7156
0.00124 0.00666 0.0317 0.00464 0.0116 0.067 0.00096 0.0253 0.00217
3L:4221880-4222030:plus -31.7041 -0.137615 -0.357798 2.52294 -17.6429 -9.72449 -16.211 3.91667
5.49541 0.0146 0.0108 0.013 0.0113 0.00562 0.00352 0.0113 0.00416 0.0025
3L:4225960-4226110:minus -21.7755 0.192661 1.95413 0.724771 -27.4082 -0.0204082 -10.8165 -1.09722
5.70642 0.00241 0.00965 0.00588 0.0223 0.0321 0.000411 0.0036 0.0232 0.00222
3L:4225960-4226110:plus -29.4388 0.752294 1.0367 7.99083 0.836735 -27.3367 -13.8073 -3.23611 0.0733945
0.00842 0.00791 0.00816 0.00059 0.000215 0.0347 0.00695 0.041 0.0181
3L:4227220-4227370:minus -22.9286 -0.917431 9.91743 9.7156 -17.7143 -9.45918 -19.578 10.3611
5.49541 0.0046 0.014 0.00015 0.000242 0.00627 0.00279 0.0192 0.000125 0.0025
3L:4227220-4227370:plus -31.2551 -1.92661 -0.853211 3.18349 -17.0408 -18.0102 -12.9266 6.31944
-0.706422 0.0113 0.019 0.0152 0.00837 0.00329 0.00812 0.00568 0.00142 0.024
3L:4231640-4231790:minus -20.7041 -1.36697 -0.211009 6.90826 -17.4184 -9.23469 0.302752 -1.94444
7.48624 0.00175 0.0161 0.0124 0.00133 0.00457 0.00238 0.000263 0.0294 0.00101
3L:4231640-4231790:plus -23.1224 1.6789 1.51376 6.47706 -17.4082 -17.3061 -11 4.25 2.94495 0.00531
0.00565 0.00689 0.00179 0.00421 0.00548 0.00373 0.00362 0.00714
3L:4232440-4232590:minus -22.2959 4.33945 0.330275 0.844037 -27 -8.16327 -2.70642 6.08333 9.88073
0.00335 0.00211 0.0104 0.0215 0.0233 0.000783 0.000527 0.00159 0.000206
3L:4232440-4232590:plus -23.2959 3.6422 2.46789 3.58716 1.80612 -9.08163 -15.6972 5.18056 7.22018
0.00605 0.00275 0.00486 0.00694 0.0000557 0.00216 0.0103 0.00242 0.00119
3L:4235080-4235230:minus -22.7041 -0.256881 3.56881 3.98165 -8.93878 -27.7449 -10.0734 -0.527778
1.90826 0.00395 0.0113 0.00318 0.00582 0.00216 0.0448 0.00312 0.0196 0.00974
3L:4235080-4235230:plus -23.5204 2.88073 5.82569 5.69725 -27.2653 -9.79592 -17.2752 11.7917 2.49541
0.00645 0.00363 0.00125 0.00314 0.0302 0.00377 0.0134 0.0000378 0.00794
3L:4236240-4236390:minus -13.8776 -2.51376 5.2844 6.33945 -8.40816 -27.6327 9.47706 10.7083 2.51376
0.0011 0.0225 0.00158 0.00196 0.00108 0.043 0.0000155 0.0000964 0.00792
3L:4236240-4236390:plus -31.3265 -0.908257 1.7156 6.45872 -17.3776 -16.2755 -18.6881 2.59722
-2.77982 0.0114 0.0139 0.00642 0.00182 0.00419 0.00413 0.0166 0.00694 0.042
3L:4240240-4240390:minus -23.5612 -0.422018 0.137615 2.12844 -8.37755 9.60204 -13.4679 6.45833
-1.69725 0.00653 0.0119 0.0111 0.0134 0.00106 0.0000248 0.00644 0.00133 0.0308
3L:4240240-4240390:plus -28.4388 4.77982 2.38532 7.70642 -26.9694 -8.16327 -23.0826 0.138889 -2.48624
0.00831 0.00178 0.00501 0.000708 0.023 0.000783 0.0356 0.016 0.0387
3L:4242720-4242870:minus -32.0816 -0.449541 -3.18349 -0.981651 -18.7245 -17.3469 -16.6239 4.97222
-5.14679 0.0176 0.012 0.0304 0.0365 0.0136 0.0058 0.0121 0.00266 0.0751
3L:4242720-4242870:plus -13.2245 6.54128 -6.42202 6.27523 10.6939 -17.0816 -11.156 1.88889 9.92661
0.000738 0.000818 0.07 0.00206 7.35e-06 0.00487 0.00385 0.00896 0.000193
3L:4252260-4252410:minus -22.1735 0.743119 -1.85321 0.477064 -19.051 -18.2041 -11.7798 -4.65278 2.75229
0.0031 0.00794 0.0207 0.0238 0.0162 0.0103 0.00439 0.0571 0.00748
3L:4252260-4252410:plus -32.9592 -2.20183 4.78899 4.33945 -18.1531 -37.2959 -19.1927 0.986111 1.0367
0.0252 0.0206 0.00194 0.00509 0.00829 0.143 0.018 0.0122 0.0141
3L:4252880-4253210:minus -13.3776 -2.87156 2.70642 6.31193 -18.1531 -7.5 -27.3394 1.36111 -0.743119
0.000831 0.0249 0.00444 0.00199 0.00829 0.000614 0.0631 0.0108 0.0243
3L:4252880-4253210:plus -31.6633 -5.22018 7.78899 4.07339 -17.1837 -27.051 -16.6055 -3.02778 10.3394
0.0141 0.0467 0.00049 0.00562 0.00366 0.0315 0.012 0.0389 0.000112
3L:4253780-4253930:minus -22.9388 3.98165 2.48624 4.3211 -8.97959 -18.1224 2.18349 3.22222 1.78899
0.0046 0.00242 0.00483 0.00514 0.0023 0.00988 0.000156 0.00548 0.0103

3L:4253780-4253930:plus -21.6224 -3.26606 -1.44037 4.33945 -8.67347 -27.6735 -9.68807 -2.34722 3.22018
0.00213 0.0277 0.0183 0.00509 0.00154 0.0443 0.0029 0.0327 0.0064
3L:4254680-4254830:minus -42.8571 2.06422 -0.00917431 2.27523 -28.5306 -9.79592 -22.3486 5.65278
3.46789 0.0873 0.0049 0.0116 0.0125 0.061 0.00377 0.0314 0.00195 0.00582
3L:4254680-4254830:plus -23.9286 3.55963 1.37615 4.61468 10.0918 -17.7857 -4.55046 1.98611 -0.247706
0.00701 0.00283 0.00724 0.00475 0.0000251 0.0068 0.000873 0.00866 0.0204
3L:4259280-4259430:minus -32.8061 0.146789 8.01835 6.89908 -27.2347 -26.602 -22.1651 5.125 5.12844
0.0237 0.0098 0.000433 0.00134 0.0283 0.0248 0.0304 0.00248 0.00301
3L:4259280-4259430:plus -32.9592 5.45872 11.0459 6.57798 -17.1531 -19.3061 -19.6422 2.45833
3.52294 0.0252 0.00134 0.0000777 0.00167 0.00364 0.0187 0.0194 0.00731 0.00558
3L:4277900-4278050:minus -31.7347 0.651376 -2.41284 3.44037 -17.9388 -9.60204
-21.2752 6.79167 2.56881 0.0149 0.00821 0.0244 0.00746 0.00715 0.00334 0.0259 0.00112 0.00783
3L:4277900-4278050:plus -31.6327 -1.68807 5.51376 7.34862 1.87755 -9.42857 -20.1009 1.47222
-6.05505 0.0138 0.0177 0.00143 0.000998 0.0000492 0.0027 0.021 0.0104 0.09
3L:4282780-4282930:minus -22.8878 -0.174312 4.14679 7.27523 -8.56122 -9.68367 -18.9174
2.54167 -0.688073 0.00441 0.0109 0.00252 0.00109 0.00112 0.00342 0.0172 0.00709 0.0238
3L:4282780-4282930:plus -33.2245 4.0367 -2.48624 -0.155963 -18.9796 1.31633 -17.3028
4.08333 -4.85321 0.0276 0.00237 0.0249 0.0282 0.0155 0.000066 0.0134 0.00388 0.0703
3L:4283720-4283870:minus -20.8878 5.69725 10.2936 3.89908 -26.5714 -8.5 -22.1284 7.20833
5.33028 0.0018 0.00121 0.00012 0.00604 0.0185 0.00126 0.0302 0.000902 0.00273
3L:4283720-4283870:plus -5.15306 8.27523 3.9633 9.44954 -26.2653 -17.8571 -13.9725 1.20833
7.42202 0.000226 0.000329 0.00272 0.000326 0.018 0.00719 0.00721 0.0113 0.00106
3L:4286940-4287090:minus -22.1531 6.68807 2.27523 1.55963 -8.82653 -8.72449 -24.9541
2.61111 6.07339 0.0031 0.00076 0.00522 0.0173 0.00163 0.00169 0.0469 0.00691 0.0018
3L:4286940-4287090:plus -23.1531 0.990826 11.8991 3.3578 -8.37755 -8.79592 -19.5229
-3.20833 -1.08257 0.00542 0.00726 0.0000462 0.00767 0.00106 0.0019 0.019 0.0407 0.0267
3L:4292280-4292430:minus -33.1429 -2.92661 2.08257 1.9633 -18.5204 -26.9694 -20.7431
1.59722 3.77064 0.0272 0.0253 0.0056 0.0146 0.0117 0.0304 0.0235 0.00993 0.00491
3L:4292280-4292430:plus -40.4388 3.64286 -1.12844 2.93578 2.14286 -8.79592 -17.633 4.11111
-2.43119 0.0401 0.00159 0.0166 0.00927 0.0000316 0.0019 0.0141 0.00384 0.038
3L:4313460-4313610:minus -42.9184 -0.192661 -4.3945 0.697248 -28.7551 -0.469388
-25.1651 8.22222 -4.84404 0.0881 0.011 0.0422 0.0224 0.0637 0.000533 0.0482 0.000514 0.0701
3L:4313460-4313610:plus -13.4184 2.55963 14.9633 7.29358 -26.8571 -16.2755 -20.9358 0.888889
-1.22018 0.000873 0.00409 1.77e-06 0.00104 0.0198 0.00413 0.0243 0.0126 0.0278
3L:4318620-4318770:minus -32.6735 -1.83486 1.22018 0.880734 -27.9694 -16.3469
-18.3486 9.73611 -0.642202 0.0216 0.0185 0.00765 0.0213 0.0437 0.00416 0.0158 0.000197 0.0234
3L:4318620-4318770:plus -24.2653 0.0275229 -2.55963 4.79817 10.3878 -17.3469 -18.2477
2.70833 -1.79817 0.00773 0.0102 0.0254 0.00453 0.0000174 0.0058 0.0155 0.00666 0.0315
3L:4356160-4356310:minus -12.3673 7.48624 0.642202 4.91743 -27.5306 -8.79592 -21.5505
3.625 -0.40367 0.000396 0.000502 0.00935 0.00447 0.0351 0.0019 0.0272 0.00468 0.0213
3L:4356160-4356310:plus -30.398 4.19266 1.83486 1.75229 -17.602 -27.051 -22.1927 4.29167 0.266055
0.00901 0.00224 0.00614 0.0159 0.00547 0.0315 0.0305 0.00356 0.0171
3L:4356760-4356910:minus -32.3673 -0.449541 -2.05505 3.93578 -18.7551 -8.5 -19.8349
-3.52778 -4.29358 0.0189 0.012 0.022 0.00597 0.0144 0.00126 0.02 0.044 0.06
3L:4356760-4356910:plus -22.8163 3.11927 7.94495 6.01835 -27.1939 -18.7857 -19.3853 5.73611
-5.25688 0.00418 0.00333 0.000451 0.00248 0.0267 0.0146 0.0186 0.00188 0.0768
3L:4357920-4358070:minus -13.4388 3.04587 10.2018 4.17431 -18.1531 -17.3469 -1.14679
1.73611 -0.12844 0.000879 0.00342 0.000126 0.00537 0.00829 0.0058 0.000367 0.00946 0.0193
3L:4357920-4358070:plus -30.4388 4.98165 13.1835 9.23853 -17.1531 -26.2653 -7.29358 1.43056
0.908257 0.00923 0.00164 0.0000171 0.000424 0.00364 0.0218 0.00181 0.0105 0.0147
3L:4364000-4364150:minus -11.9286 -2.26606 6.31193 -1.97248 -18.6837 -17.7551 -12.422
2.72222 5.22018 0.000296 0.021 0.001 0.0508 0.0126 0.00643 0.00507 0.00663 0.00289
3L:4364000-4364150:plus -31.4796 2.26606 0.504587 0.220183 -17.449 -26.7143 -18.844 2.875
3.15596 0.0128 0.00455 0.0098 0.0253 0.00484 0.0257 0.017 0.00626 0.00659

3L:4364440-4364590:minus -32 2.34862 -1.0367 1 -8.0102 -9.09184 -23.7982 7.56944 0.862385
0.0171 0.00441 0.0161 0.0206 0.000826 0.00219 0.0398 0.000743 0.0148
3L:4364440-4364590:plus -24.2551 -0.119266 14.2018 7.29358 0.316327 -0.244898 -24.5872
-3.70833 -6.3211 0.00768 0.0107 5.71e-06 0.00104 0.000341 0.000496 0.0446 0.046 0.096
3L:4367460-4367610:minus -32.4694 0.40367 0.752294 1.6789 -18.4184 -17.0816 -16.8073
6.26389 -3.13761 0.0196 0.00896 0.009 0.0165 0.0101 0.00487 0.0124 0.00146 0.0461
3L:4367460-4367610:plus -23.6531 1.79817 0.40367 3.43119 -18.7857 -10.0918 -11.7431 6.68056 3.16514
0.00661 0.00541 0.0101 0.00749 0.0148 0.00404 0.00436 0.00119 0.00656
3L:4384420-4384570:minus -29.8367 4.24771 0.825688 7.90826 -18.4184 -27.1122 1.27523 -1.30556 1.16514
0.00851 0.00219 0.00878 0.000611 0.0101 0.0316 0.000204 0.0246 0.0135
3L:4384420-4384570:plus 6.84694 4.68807 2.3945 9.7156 -27.6327 -17.7857 -2.94495 -2.01389 -2.22936
1.9e-06 0.00184 0.00499 0.000242 0.0369 0.0068 0.00056 0.0299 0.0356
3L:4387640-4387790:minus -24.3367 5.33028 5.11927 2.98165 -18.4898 -8.72449 -12.8349 -2.02778 6.80734
0.00789 0.00142 0.00169 0.00905 0.0116 0.00169 0.00557 0.03 0.0014
3L:4387640-4387790:plus -23.5204 0.825688 1.53211 4.6422 -27.2245 -18.7857 -12.4954 -1.97222 1.02752
0.00645 0.00771 0.00685 0.00473 0.0272 0.0146 0.00515 0.0296 0.0141
3L:4388100-4388250:minus -32.8061 3.46789 4.15596 1.40367 -27 -27.602 -15.8716 3.48611 -1.79817
0.0237 0.00293 0.00251 0.0184 0.0233 0.0416 0.0106 0.00494 0.0315
3L:4388100-4388250:plus -32.449 2.55046 3.07339 6.69725 -8.82653 -8.57143 -25.8991 6.83333 -2.77982
0.0195 0.0041 0.00386 0.00156 0.00163 0.00149 0.0528 0.0011 0.042
3L:4403420-4403570:minus -13.6327 6.18349 -1.48624 9.44954 -7.89796 -9.72449 -10.2569 -0.458333
3.72477 0.00101 0.000972 0.0185 0.000326 0.000678 0.00352 0.00323 0.0192 0.00497
3L:4403420-4403570:plus -31.7755 3.57798 0.0733945 7.34862 -27.1939 -8.60204 -13.156 0.861111
-0.229358 0.0157 0.00281 0.0113 0.000998 0.0267 0.00151 0.00599 0.0127 0.0202
3L:4406000-4406150:minus -22.2959 5.22018 1.70642 5.93578 -26.9694 -9.86735 -8.53211 2.04167 0.697248
0.00335 0.00149 0.00644 0.00264 0.023 0.00391 0.00234 0.00849 0.0154
3L:4406000-4406150:plus -31.398 14.2936 2.18349 2.77064 -18.4898 0.163265 -24 1.70833 3.46789
0.0117 6.04e-06 0.0054 0.01 0.0116 0.000307 0.041 0.00955 0.00582
3L:440960-441110:minus -23.2653 0.541284 -1.10092 -0.651376 -17.7551 -9.53061 -14.3853 3.44444
0.963303 0.00593 0.00854 0.0165 0.0329 0.00658 0.0031 0.00789 0.00502 0.0145
3L:440960-441110:plus -32.0714 6.42202 3.98165 -0.0917431 -17.7449 -17.8265 -17.8165 4.02778 5.6055
0.0176 0.000867 0.0027 0.0276 0.0063 0.00699 0.0145 0.00397 0.00232
3L:4413000-4413150:minus -23.4082 3.97959 1.55963 9.44954 -16.7143 -19.051 -23.5963 -1.02778 -7.38532
0.00623 0.000669 0.00678 0.000326 0.00319 0.0165 0.0386 0.0227 0.122
3L:4413000-4413150:plus -39.1429 -2.74312 1.20183 7.99083 -18.1531 -9.72449 -23.0367 1.06944 4.13761
0.0327 0.024 0.0077 0.00059 0.00829 0.00352 0.0353 0.0119 0.00408
3L:4413960-4414110:minus -23.0306 4.82569 6.21101 1.84404 -18.7857 -17.2755 -9.90826 -5.38889 -1.58716
0.00505 0.00175 0.00105 0.0154 0.0148 0.00544 0.00303 0.067 0.03
3L:4413960-4414110:plus -30.6327 -1.34862 -2.02752 1.34862 -26.8571 -9.20408 -13.4954 3.34722 -4.77064
0.00968 0.016 0.0218 0.0187 0.0198 0.00225 0.00648 0.00522 0.0687
3L:4414500-4414650:minus -31.6735 -1.20183 -0.440367 -0.311927 -18.6429 -8.42857 -10 2.01389 1
0.0141 0.0153 0.0134 0.0294 0.0121 0.00104 0.00308 0.00858 0.0143
3L:4414500-4414650:plus -30.3265 5.44954 4.16514 10.8624 -26.8571 -18.0102 -16.4679 2.52778 -0.495413
0.0089 0.00135 0.00251 0.0000687 0.0198 0.00812 0.0118 0.00712 0.022
3L:4415240-4415390:minus -31.7347 -5.93578 11.5229 0.550459 -18.4898 -9.86735 -9.97248 -2.55556 3.66972
0.0149 0.0562 0.0000581 0.0233 0.0116 0.00391 0.00306 0.0345 0.00514
3L:4415240-4415390:plus -30.3673 5.80734 7.20183 3.50459 -17.449 -18.1224 -17.8073 4.29167 -4.3211
0.00897 0.00116 0.000658 0.00713 0.00484 0.00988 0.0145 0.00356 0.0605
3L:4415620-4415770:minus -40.0714 -0.697248 -1.86239 -0.119266 -7.93878 -10.0918 -18.7156 5.93056
1.68807 0.0359 0.013 0.0208 0.0278 0.000767 0.00404 0.0167 0.00171 0.0109
3L:4415620-4415770:plus -30.449 1.78899 1.56881 6.6422 -17.4184 -8.68367 -20.2018 -0.444444
1.19266 0.00925 0.00542 0.00676 0.00161 0.00457 0.00157 0.0213 0.0192 0.0134
3L:4540840-4540990:minus -33.9286 2.3211 -0.284404 5.85321 -17.1122 1.09184 -22.1009 -1.91667
3.51376 0.0303 0.00446 0.0127 0.00287 0.00341 0.000091 0.03 0.0291 0.00566

3L:4540840-4540990:plus -23.5204 5.50459 6.83486 5.57798 -28.1939 0.795918 -15.4587 2.52778 3.62385
0.00645 0.00132 0.000785 0.0034 0.0482 0.000155 0.00982 0.00712 0.00523
3L:4541720-4541870:minus -22.449 13.5306 0.899083 11.1927 -18.7551 -17.5714 -7.99083 4.36111 -0.541284
0.0035 0.0000206 0.00856 0.0000254 0.0144 0.00616 0.0021 0.00346 0.0223
3L:4541720-4541870:plus -14.2245 3.70642 -1.63303 9.08257 -26.5306 -17.9796 -20.2294 7.13889 -7.24771
0.00126 0.00268 0.0194 0.000444 0.0185 0.00764 0.0214 0.000935 0.119
3L:4543840-4543990:minus -13.5204 -1.31193 3.44954 2.00917 -26.9286 -18.1224 -15.7615 2.625 11.8532
0.00097 0.0158 0.00334 0.0142 0.0214 0.00988 0.0104 0.00687 0.0000428
3L:4543840-4543990:plus -30.6327 -0.293578 11.8716 7.29358 -18.4184 -16.7143 -18.789 -2.80556
-3.68807 0.00968 0.0114 0.0000468 0.00104 0.0101 0.00419 0.0169 0.0368 0.0516
3L:4544560-4544710:minus -32.551 -2.06422 3.66055 0.633028 -17.7857 -8.82653 -10.8899 2.44444 3.80734
0.0207 0.0198 0.00307 0.0229 0.00676 0.00191 0.00365 0.00734 0.00484
3L:4544560-4544710:plus -32.7347 3.57143 -1.92661 1.22018 -17.7551 0.0510204 -18.0092 8.20833
3.63303 0.0225 0.00175 0.0212 0.0194 0.00658 0.000349 0.015 0.000518 0.00518
3L:4546880-4547030:minus -14.7449 4.55963 -2.21101 5.74312 -7.63265 -19.051 -2.88073 -1.125 1.22018
0.00157 0.00194 0.023 0.00302 0.000479 0.0165 0.000551 0.0234 0.0133
3L:4546880-4547030:plus -31.0306 -0.211009 6.48624 4.33945 -27.2755 -17.2755 -17.6881 -0.527778
2.25688 0.0105 0.0111 0.000925 0.00509 0.0302 0.00544 0.0143 0.0196 0.00853
3L:4613000-4613150:minus -32.0714 -3.3211 12.055 9.23853 -9.23469 -7.72449 -14.9541 4.65278 3.41284
0.0176 0.0282 0.0000412 0.000424 0.00277 0.00068 0.00889 0.00305 0.006
3L:4613000-4613150:plus -51.0714 0.422018 0.110092 4.91743 -27.8673 -10.0204 -21.2294 3.40278 2.37615
0.158 0.0089 0.0112 0.00447 0.0391 0.00399 0.0256 0.00511 0.00825
3L:4614280-4614430:minus -32.7449 -2.29358 0.348624 9.59633 -17.7143 0.0918367 -14.6147 0.513889
-1.13761 0.0228 0.0211 0.0103 0.000275 0.00627 0.000323 0.00829 0.0142 0.0271
3L:4614280-4614430:plus -22.3367 -0.770642 7.06422 6.11009 -18.5612 -19.1224 -21.0734 9.875
8.41284 0.00339 0.0133 0.000706 0.00233 0.0119 0.0175 0.0249 0.000179 0.000519
3L:4626200-4626350:minus -31.1837 -2.12844 2.3578 6.11009 -9.12245 -18.7857 -2.89908 -1.26389 1.46789
0.0109 0.0202 0.00506 0.00233 0.00238 0.0146 0.000553 0.0243 0.0119
3L:4626200-4626350:plus -32.2245 13.5306 0.623853 1.99083 -27.2347 1.16327 -23.789 1.625 -4.87156
0.0183 0.0000206 0.00941 0.0144 0.0283 0.0000831 0.0398 0.00983 0.0708
3L:4627080-4627230:minus -23.2551 0.752294 0.605505 1.20183 0.612245 -17.7551 -21.6514 4.59722 -4.12844
0.00583 0.00791 0.00946 0.0195 0.000269 0.00643 0.0277 0.00313 0.0573
3L:4627080-4627230:plus -22.6633 -1.55963 2.77982 4.66972 -17.8571 -7.38776 -7.79817 -1.48611 4.6789
0.00384 0.017 0.00432 0.00466 0.00698 0.000563 0.00202 0.0259 0.00347
3L:4687220-4687370:minus -3.07143 -0.192661 5.94495 11.0275 -18.1122 -17.7857 13.1193 0.25 2.09174
0.0000441 0.011 0.00118 0.0000401 0.00771 0.0068 3.27e-06 0.0155 0.00904
3L:4687220-4687370:plus -13.1837 0.724771 6.66055 4.36697 -17.8571 -35.8163 4.65138 -1.58333 2.84404
0.000705 0.008 0.000851 0.00505 0.00698 0.0815 0.0000651 0.0266 0.00736
3L:4825700-4825850:minus -22 8.87156 6.06422 3.97248 -17.7143 -17.2755 -16.6055 -0.402778 6.49541
0.00291 0.000239 0.00112 0.00587 0.00627 0.00544 0.012 0.0189 0.00154
3L:4825700-4825850:plus -14.1939 3.37615 16.2202 4.0367 -18.4898 -17.5 -14.2477 -4.48611 4.13761
0.00124 0.00303 1.24e-07 0.00578 0.0116 0.00599 0.00766 0.055 0.00408
3L:490780-490930:minus -3.07143 -2.0367 -0.256881 7 -26.9286 -18.0816 -2.70642 -2.77778 7.62385
0.0000441 0.0196 0.0126 0.00132 0.0214 0.00934 0.000527 0.0365 0.000941
3L:490780-490930:plus -42.9286 0.165138 3.86239 6.00917 -8.19388 -10.0204 -18.789 3.33333 -4.29358
0.0884 0.00974 0.00283 0.00255 0.000878 0.00399 0.0169 0.00525 0.06
3L:505820-505970:minus -30.7347 1.04587 5.68807 4.33945 -18.1939 -26.051 -11.8807 -2.72222 -0.623853
0.01 0.00712 0.00133 0.00509 0.0086 0.0216 0.00449 0.036 0.0232
3L:505820-505970:plus -14.3673 -0.477064 4.70642 6.13761 -18.7143 -19.3061 -13.6147 1.86111 -2.43119
0.00132 0.0121 0.00201 0.00226 0.0136 0.0187 0.00665 0.00905 0.038
3L:5127220-5127370:minus -42.551 2.15596 -2.99083 1.7156 -27.2653 -8.79592 -18.3211 1.13889 5.34862
0.0803 0.00474 0.0288 0.0162 0.0302 0.0019 0.0157 0.0116 0.00271
3L:5127220-5127370:plus -32.7347 13.4592 3.42202 2.05505 -18.4184 -17.6429 -5.09174 6.5 1.22936
0.0225 0.000064 0.00337 0.0139 0.0101 0.00619 0.00102 0.0013 0.0132

3L:5129340-5129490:minus -22.9184 7.09174 -0.0917431 5.37615 -17.6735 -19.0102 -16.8165 1 -0.59633
0.00447 0.000619 0.0119 0.00384 0.00572 0.0159 0.0124 0.0121 0.0229
3L:5129340-5129490:plus -29.398 -2.3945 15.0367 7.29358 -8.44898 -8.72449 -14.5413 3.48611 -3.81651
0.00839 0.0218 1.36e-06 0.00104 0.00109 0.00169 0.00816 0.00494 0.0529
3L:5132600-5132750:minus -21.6735 -2.50459 -0.688073 2.22936 -18.4184 -16.7143 -7.00917 6.20833
-2.05505 0.00224 0.0225 0.0145 0.0128 0.0101 0.00419 0.00169 0.0015 0.0338
3L:5132600-5132750:plus -24 4.21101 3.73394 4.44037 -17.449 -17.5612 -13.7156 3.69444 0.33945
0.00719 0.00222 0.00299 0.00493 0.00484 0.00604 0.00681 0.00455 0.0168
3L:5134600-5134750:minus -31.0714 4.7156 -1.04587 2.68807 -8.20408 -27.6735 -10.367 -1.65278 1.86239
0.0106 0.00183 0.0162 0.0104 0.000894 0.0443 0.0033 0.0271 0.00994
3L:5134600-5134750:plus -34.3367 4.18349 5.00917 2.70642 -8.89796 -8.68367 -16.5413 0.763889 -4.04587
0.0318 0.00224 0.00177 0.0103 0.00189 0.00157 0.0119 0.0131 0.0563
3L:5139080-5139230:minus -22.9184 -5.6422 9.99083 5.37615 0.877551 -8.94898 -14.7523 6.52778 0.954128
0.00447 0.0521 0.000143 0.00384 0.00019 0.00203 0.00853 0.00128 0.0145
3L:5139080-5139230:plus -22 -1.49541 15.5596 7.29358 -27.2347 -8.79592 -11.4495 3 -2.46789 0.00291
0.0167 6.69e-07 0.00104 0.0283 0.0019 0.00409 0.00597 0.0384
3L:5145680-5145830:minus -30.4082 5.26606 2.76147 0.385321 -17.1939 -18.2041 -18.1651 0.444444 2.38532
0.00908 0.00146 0.00435 0.0243 0.00377 0.0103 0.0153 0.0145 0.00822
3L:5145680-5145830:plus -22.0306 -1.72477 8.04587 6.37615 -27.2347 -18.8571 -6.04587 -3.13889 1.95413
0.00293 0.0179 0.000428 0.00192 0.0283 0.0151 0.00133 0.04 0.00964
3L:5165460-5165610:minus -33.4082 -0.449541 8.90826 0.53211 -17.7551 -8.02041 -4.37615 7.18056
5.01835 0.0283 0.012 0.000267 0.0234 0.00658 0.000761 0.00083 0.000915 0.00309
3L:5165460-5165610:plus -23.0816 1.83486 1.38532 6.00917 -17.5306 -8.65306 -20.7339 -2.94444 2.16514
0.00522 0.00533 0.00721 0.00255 0.0054 0.00156 0.0234 0.0381 0.00879
3L:5169360-5169510:minus -22.9184 4.54128 3.58716 2.11009 -26.6633 1.02041 -13.3761 5.13889 -4.0367
0.00447 0.00195 0.00316 0.0136 0.0189 0.000118 0.0063 0.00247 0.0562
3L:5169360-5169510:plus -31.5102 -2.04587 -0.119266 5.85321 -26.2347 -9.30612 -9.84404 11.0833
4.78899 0.013 0.0197 0.0121 0.00287 0.0177 0.00257 0.00299 0.0000712 0.00336
3L:5174140-5174290:minus -33.7041 6.44954 1.77064 6.56881 -27.1939 -18.7857 -25.578 3.34722 0.0275229
0.0293 0.000854 0.00629 0.0017 0.0267 0.0146 0.0508 0.00522 0.0183
3L:5174140-5174290:plus -23.2245 -1.09174 2.46789 6.33945 -8.37755 -26.7857 -19.9358 5.375 -1.56881
0.00576 0.0148 0.00486 0.00196 0.00106 0.0266 0.0204 0.00222 0.0298
3L:5176180-5176330:minus -4.90816 3.22936 -0.917431 1.91743 -17.3469 9.60204 -19.7339 7.23611
9.55046 0.000208 0.0032 0.0156 0.015 0.00394 0.0000248 0.0197 0.000889 0.000321
3L:5176180-5176330:plus -23.7755 2.38532 6.74312 11.0275 -27.0408 -9.37755 -22 -0.791667 11.8991
0.00678 0.00436 0.000818 0.0000401 0.0242 0.00265 0.0295 0.0212 0.0000255
3L:5177360-5177510:minus -31.6735 -7.0367 1.93578 1.06422 -18.7449 -7.42857 -13.1927 2.04167 6.12844
0.0141 0.0736 0.00592 0.0203 0.0138 0.000567 0.00604 0.00849 0.00174
3L:5177360-5177510:plus -22.6633 -1.46789 1.2844 2.06422 -17.1837 -16.9796 -16.1927 -2.47222 1.23853
0.00384 0.0166 0.00748 0.0139 0.00366 0.0044 0.0112 0.0338 0.0132
3L:5177840-5177990:minus -21.6633 -0.0917431 0.440367 6.41284 -26.1939 -8.5 -22.4312 1.43056 -4.34862
0.00223 0.0106 0.01 0.00189 0.0175 0.00126 0.0318 0.0105 0.0612
3L:5177840-5177990:plus -39.7653 -2.17431 -0.954128 1.91743 -17.3367 -19.1531 -7.72477 8.59722
9.88073 0.0339 0.0204 0.0157 0.015 0.00391 0.0177 0.00199 0.000411 0.000206
3L:5181860-5182010:minus -23.1122 0.559633 -0.165138 2.80734 -17.0408 -9.68367 -16.2477 -0.916667
-8.05505 0.0053 0.00848 0.0122 0.00985 0.00329 0.00342 0.0113 0.022 0.137
3L:5181860-5182010:plus -32.7041 4.05505 3.47706 7.00917 -16.449 -18.051 -21.9817 3.16667 -0.495413
0.0222 0.00235 0.0033 0.00126 0.00307 0.00886 0.0294 0.0056 0.022
3L:5239980-5240130:minus -30.9184 -1.11009 -1.30275 -1.14679 -18.1224 -9.45918 -21.8807 -1.66667 8.19266
0.0103 0.0148 0.0175 0.0384 0.00782 0.00279 0.0289 0.0272 0.000548
3L:5239980-5240130:plus -22.8571 -4.19266 1.92661 2.17431 -9.16327 -18.7551 -27.3486 1.65278 2.6789
0.00427 0.0356 0.00594 0.0132 0.00249 0.0141 0.0632 0.00974 0.00763
3L:5240860-5241010:minus -41.8163 5.19266 -2.3945 0.211009 -9.20408 -8.38776 -20.0275 2.26389 -1.22018
0.0662 0.0015 0.0242 0.0254 0.00262 0.00101 0.0207 0.00784 0.0278

3L:5240860-5241010:plus -30.6122 -1.43119 4.27523 3.88991 -18.4592 -17.7551 -18.8807 -0.388889
5.84404 0.00963 0.0164 0.0024 0.00608 0.0109 0.00643 0.0171 0.0188 0.00202
3L:5241440-5241590:minus -31.4388 -0.651376 -0.33945 9.59633 -27.4592 -18.7857 -9.45872 -4.19444
0.963303 0.0122 0.0128 0.0129 0.000275 0.0329 0.0146 0.00278 0.0515 0.0145
3L:5241440-5241590:plus -12.8163 -3.72477 0.678899 3.29358 -26.9694 -26.7551 -13.8073 -2.25 0.513761
0.000489 0.0314 0.00923 0.00791 0.023 0.0263 0.00695 0.0319 0.0162
3L:5270380-5270530:minus -32.8163 0.880734 -0.229358 -0.137615 -17.3061 -8.68367 -16.578 4.02778
-4.86239 0.024 0.00756 0.0125 0.0279 0.00389 0.00157 0.012 0.00397 0.0705
3L:5270380-5270530:plus -22.8571 0.688073 7.37615 6.89908 -17.3061 0.826531 -25.0642 -2.52778 -13.8807
0.00427 0.0081 0.000603 0.00134 0.00389 0.000153 0.0476 0.0343 0.345
3L:5271060-5271210:minus -14.2551 0.825688 -0.0275229 4.3211 -17.7857 -7.65306 -20.367 1.91667
10.3486 0.00127 0.00771 0.0117 0.00514 0.00676 0.000647 0.022 0.00888 0.0000988
3L:5271060-5271210:plus -48.8469 -4.95413 -0.0733945 0.779817 -17.7551 -10.0204 -25.7706 2.77778
3.46789 0.099 0.0436 0.0119 0.0219 0.00658 0.00399 0.052 0.00649 0.00582
3L:531080-531230:minus -13.3776 1.3578 3.41284 2.13761 -17.1837 -7.5 -24.6697 0.888889 -2.83486
0.000831 0.00635 0.00338 0.0134 0.00366 0.000614 0.0451 0.0126 0.0427
3L:531080-531230:plus -14.4796 2.20183 -0.651376 4.36697 -8.86735 -17.051 -13.4037 0.472222 10.3486
0.00142 0.00466 0.0143 0.00505 0.00173 0.00479 0.00634 0.0144 0.0000988
3L:5346860-5347010:minus -29.1837 0.449541 3.20183 4.91743 -17.4898 -18.3061 -12.1743 2.52778 1.79817
0.00835 0.00882 0.00367 0.00447 0.0053 0.0114 0.00479 0.00712 0.0102
3L:5346860-5347010:plus -31.4388 6.45872 -1.37615 5.22018 -18.1939 -27.3367 -13.0367 5.77778 5.80734
0.0122 0.000851 0.0179 0.00418 0.0086 0.0347 0.00583 0.00184 0.00205
3L:5348360-5348510:minus -4.47959 4.66055 2.82569 9.76147 -17.7857 -18.0102 -7.53211 -0.625 2.11009
0.000154 0.00187 0.00424 0.000212 0.00676 0.00812 0.0019 0.0202 0.00898
3L:5348360-5348510:plus -15.0408 6.06422 0.59633 -1.83486 -27.5306 -19.5714 -5.07339 3.72222 -4.06422
0.00162 0.00103 0.00949 0.0484 0.0351 0.0202 0.00101 0.0045 0.0565
3L:5352380-5352530:minus -32.7755 14.4862 13.5321 1.63303 -17.6735 -18.3469 -11.6881 2.33333 0.110092
0.0234 5.19e-06 0.0000127 0.0167 0.00572 0.0124 0.00431 0.00765 0.018
3L:5352380-5352530:plus -31.7449 14.1009 13.156 7.2844 -27.2347 -18.7857 -1.0367 2.18056 4.98165
0.0152 7.56e-06 0.0000178 0.00105 0.0283 0.0146 0.000358 0.00808 0.00313
3L:5353080-5353290:minus -40.551 0.422018 -2.3211 -1.15596 -8.93878 9.37755 -25.4037 0.0972222
-6.7156 0.0418 0.0089 0.0237 0.0386 0.00216 0.0000363 0.0497 0.0162 0.106
3L:5353080-5353290:plus -21.9694 4.25688 -1.63303 4.0367 -18.4184 -27.4082 -14.7798 -4.08333 10.3394
0.00282 0.00218 0.0194 0.00578 0.0101 0.0356 0.00858 0.0502 0.000112
3L:5353460-5353610:minus -40.5918 -3.69725 -1.66055 1.84404 1.07143 0.316327 -20.7615 -1.38889 1.38532
0.0422 0.0312 0.0195 0.0154 0.000125 0.000243 0.0236 0.0252 0.0123
3L:5353460-5353610:plus -23.5204 -2.11927 8.9633 3.89908 -18.7449 -9.38776 -15.1376 -2.25 0.816514
0.00645 0.0201 0.000259 0.00604 0.0138 0.00266 0.00922 0.0319 0.015
3L:5353840-5353990:minus -30.4796 -4.40367 3.6422 3.59633 -8.86735 -17.898 -12.2202 3.18056 2.21101
0.00941 0.0377 0.00309 0.00693 0.00173 0.00726 0.00484 0.00557 0.00862
3L:5353840-5353990:plus -31.4388 -3.3578 4.66972 6.49541 -17.0816 -35.9286 -10.2385 -0.125 1.01835
0.0122 0.0284 0.00204 0.00176 0.00334 0.0825 0.00322 0.0174 0.0142
3L:535480-535630:minus -22.3367 2.68807 4.53211 -2.58716 -27.9694 -9.42857 -14.6514 7 5.95413
0.00339 0.0039 0.00216 0.0617 0.0437 0.0027 0.00835 0.00101 0.00192
3L:535480-535630:plus -13.6735 0.990826 1.72477 6.47706 -18.6837 1.05102 -16.9633 -3.30556 -2.17431
0.00103 0.00726 0.0064 0.00179 0.0126 0.000107 0.0127 0.0417 0.035
3L:5358920-5359070:minus -22.398 1.11009 3.51376 2.62385 -17.1531 -18.2755 -8.00917 7.625 5.18349
0.00344 0.00695 0.00325 0.0108 0.00364 0.0112 0.00211 0.00072 0.00295
3L:5358920-5359070:plus -20.8878 -0.963303 0.266055 3.52294 -8.89796 -19.2041 -16.1835 2.08333
1.34862 0.0018 0.0142 0.0106 0.00706 0.00189 0.0179 0.0112 0.00837 0.0125
3L:5359280-5359430:minus -32.398 -2.72477 -4.72477 -0.568807 0.836735 0.316327 -20.4495 1.11111
3.15596 0.019 0.0239 0.046 0.032 0.000215 0.000243 0.0223 0.0117 0.00659
3L:5359280-5359430:plus -22.2245 -0.862385 10.2569 6.01835 0.540816 0.316327 -20.2477 -1.36111
-1.33945 0.00325 0.0137 0.000122 0.00248 0.000321 0.000243 0.0215 0.025 0.0285

3L:5359720-5359870:minus -22.1939 5.57798 1.22936 7.3578 -17.4184 -7.42857 -20.7982 4.90278 -3.78899
0.00317 0.00128 0.00763 0.000965 0.00457 0.000567 0.0237 0.00274 0.0526
3L:5359720-5359870:plus -20.7347 5.38532 3.93578 7.27523 -26.9694 -18.3469 -5.92661 -3.40278 3.14679
0.00176 0.00139 0.00275 0.00109 0.023 0.0124 0.00129 0.0427 0.00666
3L:5361180-5361330:minus -32.1122 0.266055 10.6881 1.6789 -9.23469 -18.1224 -20.0734 -0.444444
1.44037 0.0178 0.0094 0.0000951 0.0165 0.00277 0.00988 0.0209 0.0192 0.012
3L:5361180-5361330:plus -12.1531 1.58716 0.40367 4.43119 -27.1224 -0.0204082 -7.44954 2.30556
9.61468 0.000352 0.00584 0.0101 0.00495 0.0245 0.000411 0.00187 0.00772 0.00029
3L:540180-540330:minus -32.8571 -0.0366972 1.52294 -0.275229 -18.4184 -26.6735 -9.6789 5.125
1.6422 0.0244 0.0104 0.00687 0.029 0.0101 0.0256 0.0029 0.00248 0.0111
3L:540180-540330:plus -22.8878 6.08257 0.614679 2.88991 -27.1939 0.0510204 -17.3211 -1.77778 -6.76147
0.00441 0.00102 0.00943 0.00942 0.0267 0.000349 0.0135 0.0281 0.107
3L:540540-540690:minus -12.6633 13.2202 1.11009 3.09174 -26.7449 -17.3469 -10.3303 5.05556 2.11927
0.000458 0.0000184 0.00796 0.00867 0.0195 0.0058 0.00328 0.00256 0.00889
3L:540540-540690:plus -23 12.5138 -0.119266 1.95413 -18.3776 -18.3469 -19.3945 -2.94444 1.48624
0.00498 0.0000321 0.0121 0.0147 0.00926 0.0124 0.0186 0.0381 0.0118
3L:543340-543490:minus -33.1531 2.3211 0.284404 3.08257 -17.4082 -17.5714 -21.6972 -2.75 2.25688
0.0273 0.00446 0.0105 0.0087 0.00421 0.00616 0.0279 0.0363 0.00853
3L:543340-543490:plus -19.6327 4.50459 -0.385321 2.69725 -18.6837 10.602 -15.6147 1.23611 5.24771
0.00163 0.00198 0.0131 0.0104 0.0126 4.1e-06 0.0101 0.0112 0.00282
3L:545980-546130:minus -23.1837 -4.15596 -1.43119 2.95413 -16.8878 -17.2755 -9.42202 2.13889 11.3853
0.00553 0.0353 0.0182 0.00917 0.00326 0.00544 0.00277 0.0082 0.0000815
3L:545980-546130:plus -2.84694 3.91743 3.55963 6.02752 -18.1531 -26.7551 -17.4312 -0.736111 5.22018
0.0000385 0.00248 0.0032 0.00246 0.00829 0.0263 0.0137 0.0209 0.00289
3L:548620-548770:minus -30.2551 0.366972 3.77982 3.3578 -18.9796 -18.3469 -17.4771 3.43056 3.78899
0.00885 0.00908 0.00293 0.00767 0.0155 0.0124 0.0138 0.00505 0.00487
3L:548620-548770:plus -31.898 0.357798 -1.88991 7.62385 -17.4184 10.8265 -34.0826 -1.18056 -12.4771
0.0164 0.00911 0.0209 0.000759 0.00457 2.5e-06 0.147 0.0238 0.287
3L:5492000-5492150:minus -32.551 -2.89908 -0.66055 5.73394 -8.60204 -8.16327 -12.7248 2.94444 5.22936
0.0207 0.0251 0.0143 0.00307 0.00125 0.000783 0.00543 0.00609 0.00288
3L:5492000-5492150:plus -22.0714 13.3878 -1.85321 0.00917431 -18.449 -9.64286 -10.3119 6.34722
5.76147 0.00299 0.0000869 0.0207 0.0269 0.0107 0.00339 0.00326 0.0014 0.0021
3L:550840-550990:minus -32.6224 -0.33945 -4.27523 -1.05505 -8.70408 -18.5 0.706422 3.125 5.79817
0.0211 0.0116 0.0409 0.0374 0.00155 0.0131 0.000238 0.00569 0.00208
3L:550840-550990:plus -23.1429 -2.30275 4.41284 9.88073 -17.4184 -17.0102 -8.12844 -2.65278 8.10092
0.00533 0.0212 0.00227 0.000183 0.00457 0.00453 0.00216 0.0354 0.000623
3L:5553280-5553430:minus -34.1429 -1.82569 3.18349 9.44954 -26.9286 -16.7857 -6.76147 2.23611 6.17431
0.0313 0.0185 0.0037 0.000326 0.0214 0.00426 0.00159 0.00792 0.00171
3L:5553280-5553430:plus -33.1429 3.90816 5.09174 6.72477 -27.6429 -18.051 -17.0917 8.27778 0.963303
0.0272 0.00102 0.00171 0.00154 0.037 0.00886 0.013 0.000497 0.0145
3L:5554720-5554870:minus -31.9898 -4.10092 3.07339 6.76147 10.9184 -18.051 -3.00917 -3.08333 1.44037
0.0169 0.0348 0.00386 0.00148 4.19e-06 0.00886 0.000569 0.0395 0.012
3L:5554720-5554870:plus -31.3571 3.97959 0.477064 7.6055 -18.6429 -18.7857 -7.46789 6.40278 1.59633
0.0114 0.000669 0.00989 0.000794 0.0121 0.0146 0.00188 0.00136 0.0113
3L:5556020-5556170:minus -22.9592 3.72477 9.65138 3.04587 -18.051 -10.0204 -5.11927 -0.430556
9.34862 0.00475 0.00266 0.000175 0.00883 0.00746 0.00399 0.00103 0.0191 0.000371
3L:5556020-5556170:plus -22.551 3.61224 -0.908257 2.77982 -18.7959 -18.5 -22.4771 3.18056
-0.220183 0.00359 0.00167 0.0155 0.00997 0.0148 0.0131 0.0321 0.00557 0.0201
3L:5556780-5556930:minus -31.6735 2.84404 -1.19266 7.62385 10.3878 19.0816 -22.9633 0.541667 1.00917
0.0141 0.00368 0.0169 0.000759 0.0000174 1.12e-06 0.0349 0.0141 0.0143
3L:5556780-5556930:plus -23.2959 2.75229 13.1651 7.29358 0.908163 -7.72449 -11.4404 -1.66667 -0.513761
0.00605 0.00381 0.0000173 0.00104 0.000153 0.00068 0.00409 0.0272 0.0221
3L:5558240-5558390:minus -34.1122 -0.486239 6.57798 -0.431193 -18.4184 -8.57143 -20.211 3.59722
-4.0367 0.0312 0.0121 0.000884 0.0306 0.0101 0.00149 0.0214 0.00473 0.0562

3L:5558240-5558390:plus -22.9592 14.4312 10.7339 5.88073 20.2041 -0.0204082 -20.6239 -0.888889
3.55963 0.00475 5.39e-06 0.0000931 0.00275 3.64e-07 0.000411 0.023 0.0218 0.00545
3L:5570840-5570990:minus -31.5816 0.633028 3.11927 7.17431 -18.6735 -27.051 -7.27523 0.680556 0.963303
0.0135 0.00826 0.00379 0.00115 0.0123 0.0315 0.0018 0.0135 0.0145
3L:5570840-5570990:plus -4.96939 0.0550459 3.02752 4.3211 -8.60204 -17.7143 -18.5321 2.69444
-0.697248 0.000218 0.0101 0.00393 0.00514 0.00125 0.00634 0.0162 0.00669 0.0239
3L:5571540-5571690:minus -23.6633 -0.0917431 3.18349 2.34862 -17.4184 -7.79592 -5.78899 2.52778
-1.41284 0.00665 0.0106 0.0037 0.0121 0.00457 0.000723 0.00124 0.00712 0.0289
3L:5571540-5571690:plus -23.1837 7.66055 4.3945 5.81651 -17.449 -18.0102 -17.7523 -2.73611 -2.11009
0.00553 0.000457 0.00229 0.00292 0.00484 0.00812 0.0144 0.0362 0.0343
3L:5576580-5576730:minus -34.2653 -2.69725 0.981651 1.9633 -27.5306 -10.0918 -31.367 3.98611 3.46789
0.0316 0.0237 0.00832 0.0146 0.0351 0.00404 0.109 0.00404 0.00582
3L:5576580-5576730:plus -22.1122 3.82569 -0.412844 6.06422 -17.8571 -8.79592 -15.1743 -1.19444
1.33028 0.00304 0.00257 0.0133 0.00239 0.00698 0.0019 0.00929 0.0239 0.0127
3L:5596780-5596930:minus -32.5102 2.22018 -2.15596 4.24771 -17.4592 -18.8265 -22.4037 1.90278 4.87156
0.0202 0.00463 0.0226 0.00519 0.00491 0.0148 0.0317 0.00892 0.00325
3L:5596780-5596930:plus -23.6327 0.550459 10.1193 5.31193 -26.898 -26.7551 -5.01835 1.08333 1.00917
0.00661 0.00851 0.000133 0.00393 0.0203 0.0263 0.000998 0.0118 0.0143
3L:5597280-5597430:minus -22.1837 10.6606 -3.89908 5.55046 -17.3776 -8.38776 -9.07339 1.31944
-0.0275229 0.00315 0.0000954 0.037 0.00345 0.00419 0.00101 0.00259 0.0109 0.0186
3L:5597280-5597430:plus -20.551 3.80734 0.155963 7.45872 -26.7041 -18.5612 -9.37615 4.76389 -0.495413
0.00168 0.00259 0.011 0.000894 0.0193 0.0132 0.00274 0.00291 0.022
3L:5601340-5601490:minus -24.8571 13.4592 -2.97248 3.76147 10.6531 -8.79592 -19.5505 5.33333 -6.27523
0.00831 0.000064 0.0286 0.00645 0.0000102 0.0019 0.0191 0.00226 0.0952
3L:5601340-5601490:plus -32 13.4592 1.01835 6.09174 -9.16327 -8.23469 -30.3394 2.86111 -3.42202
0.0171 0.000064 0.00821 0.00235 0.00249 0.000875 0.0953 0.00629 0.0488
3L:5647540-5647690:minus -32.4388 -5.16514 3.42202 0.211009 -18.051 -0.0510204 -11.6881 6.06944
1.69725 0.0195 0.0461 0.00337 0.0254 0.00746 0.000429 0.00431 0.0016 0.0108
3L:5647540-5647690:plus -21.9592 -2.48624 3.30275 3.26606 -17.7143 -18.5 1.00917 1.93056 2.11927
0.00279 0.0223 0.00353 0.00802 0.00627 0.0131 0.00022 0.00883 0.00889
3L:5648920-5649070:minus -22.7755 3.68367 4.80734 2.73394 -18.6837 -7.7551 -18.0275 7.06944 2.21101
0.00412 0.00144 0.00193 0.0101 0.0126 0.000682 0.015 0.00097 0.00862
3L:5648920-5649070:plus -20.7755 -2.12844 2.89908 9.08257 -27.1939 1.02041 -13.0459 4.16667 -1.94495
0.00176 0.0202 0.00413 0.000444 0.0267 0.000118 0.00584 0.00375 0.0327
3L:5655320-5655470:minus -32.0408 14.6789 -2.41284 2.7156 -26.8878 -18.0102 -11.3394 -2.11111 7.73394
0.0174 3.91e-06 0.0244 0.0102 0.0199 0.00812 0.004 0.0307 0.000784
3L:5655320-5655470:plus -31.8163 -3.55046 -2.29358 5.47706 -17.9796 0.0510204 -18.156 -0.527778
3.42202 0.016 0.03 0.0236 0.0036 0.00726 0.000349 0.0153 0.0196 0.00597
3L:5744920-5745070:minus -23.2245 2.58716 8.59633 7.20183 -18.449 -17.9388 -19.2844 -0.611111
-5.97248 0.00576 0.00404 0.000315 0.00112 0.0107 0.00738 0.0183 0.0201 0.0883
3L:5744920-5745070:plus -23.3367 7.58716 5.37615 5.57798 -26.9286 -18.1224 -9.08257 5.45833 -2.22936
0.00614 0.000476 0.00152 0.0034 0.0214 0.00988 0.0026 0.00214 0.0356
3L:5745180-5745330:minus -24.5306 -3.58716 -1.53211 3.49541 -18.449 -16.9796 -16.1101 10.3194 8.11009
0.00814 0.0303 0.0188 0.00716 0.0107 0.0044 0.0111 0.000129 0.000597
3L:5745180-5745330:plus -29.7755 8.2844 -0.706422 1.10092 -7.60204 -8.02041 -11.8532 5.56944
-4.08257 0.0085 0.000327 0.0146 0.02 0.000466 0.000761 0.00447 0.00203 0.0567
3L:5745680-5745830:minus -32.6633 2.11927 -2.93578 3.18349 -7.56122 -18.0102 -19.789 4.02778 -2.05505
0.0216 0.00481 0.0283 0.00837 0.000419 0.00812 0.0199 0.00397 0.0338
3L:5745680-5745830:plus -34.5612 2.77982 2.54128 5.15596 -7.97959 -9.72449 -18.9174 6.56944 -2.06422
0.032 0.00377 0.00473 0.00426 0.000799 0.00352 0.0172 0.00125 0.0339
3L:5746020-5746170:minus -33.2653 3.7551 2.02752 0.633028 -18.7551 -0.540816 -16.1651 4.20833
-2.26606 0.0277 0.00122 0.00572 0.0229 0.0144 0.00054 0.0112 0.00369 0.0359
3L:5746020-5746170:plus -22.2245 -4.41284 7.01835 5.85321 1.14286 9.30612 -1.72477 -0.0972222
5.55046 0.00325 0.0378 0.000721 0.00287 0.000113 0.0000398 0.000417 0.0172 0.00241

3L:5758740-5758890:minus -40.8878 1.22018 -4.33028 0.93578 -18.051 -18.3469 -17.789 2.94444 3.98165
0.0439 0.00668 0.0415 0.0209 0.00746 0.0124 0.0145 0.00609 0.00439
3L:5758740-5758890:plus -21 13.4592 0.486239 4.37615 -8.89796 19.4184 -16.4862 -0.527778 -4.75229
0.00186 0.000064 0.00986 0.00504 0.00189 2.36e-07 0.0118 0.0196 0.0683
3L:5762040-5762190:minus -22.9694 14.2294 -0.0733945 4.16514 -8.93878 1.31633 -9.57798 2.63889
1.38532 0.0048 6.48e-06 0.0119 0.00538 0.00216 0.000066 0.00285 0.00684 0.0123
3L:5762040-5762190:plus -32.8163 5.16514 2.40367 7.80734 -27.3367 -9.53061 -12.2385 -3.38889 7.68807
0.024 0.00152 0.00498 0.00063 0.0317 0.0031 0.00486 0.0426 0.000841
3L:5763640-5763830:minus -23.0714 -3.85321 6.54128 4.44954 -17.3776 -16.9796 -17.8073 -2.06944 5.80734
0.0052 0.0325 0.000901 0.00492 0.00419 0.0044 0.0145 0.0304 0.00205
3L:5763640-5763830:plus -31.551 -5.05505 3.95413 4.33028 -8.71429 -36.0408 -14.6972 6.56944 7.22018
0.0134 0.0448 0.00273 0.00511 0.00159 0.0837 0.00843 0.00125 0.00119
3L:5763980-5764130:minus -41.2551 -4.30275 3.98165 -1.82569 -18.4184 -8.94898 -6.33945 0.111111 3.46789
0.0513 0.0367 0.0027 0.0482 0.0101 0.00203 0.00143 0.0162 0.00582
3L:5763980-5764130:plus -22.9592 3.82569 -1.24771 3.93578 -17.7143 -8.57143 -7.44037 3.56944 -4.06422
0.00475 0.00257 0.0172 0.00597 0.00627 0.00149 0.00187 0.00478 0.0565
3L:5770380-5770530:minus -4.7449 2.29358 4.89908 2.45872 10.3878 -17.3469 -10.3945 1.48611 3.20183
0.000191 0.00451 0.00185 0.0116 0.0000174 0.0058 0.00332 0.0103 0.0065
3L:5770380-5770530:plus -13.2551 0.798165 0.944954 -0.651376 -27.5306 -17.2755 -5.91743 2.76389
1.38532 0.000751 0.00779 0.00842 0.0329 0.0351 0.00544 0.00128 0.00652 0.0123
3L:5772680-5772830:minus -24.2959 -0.688073 5.11927 0.59633 11.4286 -18.8571 -6.08257 0.791667
3.66972 0.00781 0.013 0.00169 0.023 1.97e-06 0.0151 0.00134 0.013 0.00514
3L:5772680-5772830:plus -40.9184 6.44954 1.78899 5.49541 -19.051 -8.64286 -28.0275 5.41667 3.56881
0.0443 0.000854 0.00625 0.00355 0.0162 0.00154 0.069 0.00218 0.00543
3L:5773020-5773170:minus -41.2041 2.33945 0.422018 7.61468 -18.9388 9.57143 -22.0642 2.81944 -5.9633
0.0485 0.00443 0.0101 0.000779 0.0151 0.0000274 0.0298 0.00639 0.0882
3L:5773020-5773170:plus -40.1837 -0.972477 5.66972 4.11927 1.43878 0.979592 -14.3394 2.08333
-2.66972 0.0367 0.0142 0.00134 0.00551 0.0000769 0.000136 0.00782 0.00837 0.0407
3L:5787220-5787370:minus -22.9286 7.58716 3.44954 3.78899 -27.0102 -9.45918 -13.5688 4.5 3.42202
0.0046 0.000476 0.00334 0.00632 0.0237 0.00279 0.00658 0.00326 0.00597
3L:5787220-5787370:plus -31.5918 13.4592 -0.972477 3.3578 -8.27551 -17.6429 -12.9817 2.59722
3.94495 0.0136 0.000064 0.0158 0.00767 0.000951 0.00619 0.00576 0.00694 0.00452
3L:5801780-5801930:minus -21.6224 1.9633 2 2.80734 -18.7857 0.0510204 -13.9908 0.944444 3.45872
0.00213 0.00509 0.00578 0.00985 0.0148 0.000349 0.00724 0.0124 0.00591
3L:5801780-5801930:plus -32.7347 3.23853 0.192661 7.27523 -27.1939 -9.86735 -23.9725 -1.05556 1.33028
0.0225 0.00318 0.0109 0.00109 0.0267 0.00391 0.0409 0.0229 0.0127
3L:5802780-5802930:minus -14.5918 3.7156 -1.95413 0.816514 -26.7449 -27.5306 7.82569 2.375 5.33028
0.00148 0.00267 0.0213 0.0216 0.0195 0.0374 0.0000249 0.00753 0.00273
3L:5802780-5802930:plus -21.6327 6.05505 1.12844 7.44037 -27.3061 -26.5306 -15.2936 1.72222 2.29358
0.00215 0.00103 0.00791 0.000912 0.0315 0.023 0.00951 0.0095 0.0084
3L:5803900-5804050:minus -22.1531 1.23853 3.27523 5.42202 1.87755 -8.23469 -14.5046 -4.04167 -2.88073
0.0031 0.00664 0.00357 0.00374 0.0000492 0.000875 0.0081 0.0497 0.0432
3L:5803900-5804050:plus -21.7347 -1.90826 -3.40367 -1.79817 -17.5306 -19.2347 0.834862 0.166667 2.73394
0.00235 0.0189 0.0323 0.0477 0.0054 0.018 0.00023 0.0159 0.00756
3L:5806120-5806270:minus -24.2959 7.3578 1.83486 2.34862 -27.898 -9.86735 -7.06422 2.13889 3.05505
0.00781 0.000538 0.00614 0.0121 0.04 0.00391 0.00171 0.0082 0.00692
3L:5806120-5806270:plus -22.9286 13.5963 -4.08257 7.61468 -18.3776 19.051 -14.8532 8.31944 -5.25688
0.0046 0.0000131 0.0388 0.000779 0.00926 1.23e-06 0.00871 0.000485 0.0768
3L:5838740-5838890:minus -22.4694 2.81651 1.22018 8.81651 -17.8878 -17.0102 4.66972 1.30556 5.17431
0.0035 0.00372 0.00765 0.000501 0.00704 0.00453 0.0000647 0.011 0.00296
3L:5838740-5838890:plus -22.6531 1.68807 -0.33945 3.44037 -18.449 -19.2755 -0.100917 0.888889
3.66972 0.00374 0.00563 0.0129 0.00746 0.0107 0.0185 0.00029 0.0126 0.00514
3L:5864300-5864450:minus -33.1837 -1.73394 2.66055 4.33028 -18.4898 -26.7551 -17.4954 0.347222 9.92661
0.0274 0.018 0.00452 0.00511 0.0116 0.0263 0.0138 0.015 0.000193

3L:5864300-5864450:plus -23.3367 14.4954 8.69725 10.5872 -17.7551 -8.65306 -20.3119 6.15278 0.247706
0.00614 5.09e-06 0.000299 0.000115 0.00658 0.00156 0.0217 0.00154 0.0172
3L:5866520-5866670:minus -33.3265 0.899083 7.10092 6.19266 -8.30612 -17.7143 -13.6147 0.458333 -7.34862
0.0279 0.00751 0.000693 0.00216 0.00097 0.00634 0.00665 0.0145 0.121
3L:5866520-5866670:plus -42.1122 13.5306 11.2569 1.99083 -18.4898 -8.72449 -18.1376 0.680556 0.816514
0.0698 0.0000206 0.0000682 0.0144 0.0116 0.00169 0.0153 0.0135 0.015
3L:5888500-5888650:minus -11.9286 -3.00917 1.75229 9.23853 -18.1531 -18.7857 -10.5138 -0.416667
1.15596 0.000296 0.0258 0.00633 0.000424 0.00829 0.0146 0.00339 0.019 0.0135
3L:5888500-5888650:plus -14.2245 2.44954 1.38532 0.12844 -7.64286 -18.3469 -14.6881 3.91667 3.88073
0.00126 0.00425 0.00721 0.0259 0.000499 0.0124 0.00842 0.00416 0.00475
3L:5890520-5890670:minus -31.8163 3.56881 0.119266 -1.6055 -18.4592 -26.602 -15.945 3.70833 7.16514
0.016 0.00282 0.0111 0.0448 0.0109 0.0248 0.0107 0.00452 0.00122
3L:5890520-5890670:plus -24.5612 7.62385 4.63303 3.86239 -27.3061 -9.5 -20.3578 1.19444 1.2844
0.00819 0.000468 0.00207 0.00622 0.0315 0.00294 0.0219 0.0114 0.0129
3L:589180-589330:minus -22.4388 -0.862385 2.56881 5.21101 -18.6429 -17.0102 -12.3211 0.916667
5.6055 0.00348 0.0137 0.00468 0.0042 0.0121 0.00453 0.00495 0.0125 0.00232
3L:589180-589330:plus -31.8163 8.16514 -0.899083 3.26606 -7.60204 -18.9796 -8.69725 1.81944 0.66055
0.016 0.000349 0.0155 0.00802 0.000466 0.0155 0.00242 0.00919 0.0156
3L:5892520-5892670:minus -32.551 -2.08257 -2.3211 -0.724771 -26.7041 -27.602 -16.8899 7.61111
2.78899 0.0207 0.0199 0.0237 0.0338 0.0193 0.0416 0.0126 0.000726 0.00745
3L:5892520-5892670:plus -22.8878 0.770642 9.31193 4.15596 1.61224 18.8571 -21.2844 1.81944 -6.05505
0.00441 0.00786 0.000211 0.00539 0.0000631 1.47e-06 0.0259 0.00919 0.09
3L:5895560-5895710:minus 5.91837 7.85321 -1.91743 6.21101 -26.8571 -18.8571 -9.12844 -0.0694444
1.27523 7.25e-06 0.000413 0.0211 0.00213 0.0198 0.0151 0.00262 0.0171 0.013
3L:5895560-5895710:plus -33.8163 2.19266 0.477064 2.25688 -26.2653 -18.0102 -12.3028 2.51389 8.41284
0.0299 0.00468 0.00989 0.0127 0.018 0.00812 0.00493 0.00716 0.000519
3L:590160-590310:minus -22.8469 2.00917 0.477064 4.04587 -18.4898 -26.4898 -15.3394 -3.625 7.84404
0.00424 0.005 0.00989 0.00572 0.0116 0.0225 0.0096 0.045 0.000701
3L:590160-590310:plus -13 1 6.05505 5.93578 -17.602 -17.7551 -17.578 -11.0139 -0.770642 0.000563
0.00724 0.00113 0.00264 0.00547 0.00643 0.014 0.178 0.0245
3L:5913980-5914130:minus -32.4388 3.79817 0.165138 5.31193 -26.8163 -9.02041 -16.2477 2.58333 1.27523
0.0195 0.00259 0.011 0.00393 0.0195 0.00213 0.0113 0.00698 0.013
3L:5913980-5914130:plus -33.0714 -0.201835 11.3303 3.54128 -18.449 -8.23469 -12.422 -1.625
3.88073 0.0267 0.011 0.0000653 0.00702 0.0107 0.000875 0.00507 0.0269 0.00475
3L:5915320-5915470:minus -32.4388 4.76147 0.834862 1.57798 -18.3469 -18.051 -23.2752 4.98611 -2.70642
0.0195 0.00179 0.00875 0.0171 0.00894 0.00886 0.0367 0.00264 0.0412
3L:5915320-5915470:plus -22 4.29358 0.908257 11.0183 -18.449 -18.8571 -4.63303 -0.861111 5.12844
0.00291 0.00215 0.00853 0.000052 0.0107 0.0151 0.000894 0.0217 0.00301
3L:5919780-5919930:minus -30.3673 1.6422 9.88073 6.02752 1.91837 -26.5612 -23.2752 0.916667 -4.29358
0.00897 0.00573 0.000153 0.00246 0.0000382 0.0237 0.0367 0.0125 0.06
3L:5919780-5919930:plus -3.40816 0.981651 7.44954 8.88073 -9.23469 0.0204082 -12.0275 2.88889
9.77064 0.0000685 0.00729 0.000581 0.000479 0.00277 0.000377 0.00464 0.00622 0.000228
3L:5920720-5920870:minus -31.4082 -1.54128 3.93578 7.34862 -18.6429 1.05102 -18.5046 7.69444 1.00917
0.0119 0.0169 0.00275 0.000998 0.0121 0.000107 0.0161 0.000693 0.0143
3L:5920720-5920870:plus -21.9286 0.807339 12.9083 3.42202 -27.3367 -17.2755 -6.22936 0.486111 7.22018
0.0027 0.00776 0.0000224 0.00751 0.0317 0.00544 0.00139 0.0144 0.00119
3L:5925560-5925710:minus -22.8163 0.266055 -1.05505 3.45872 -7.64286 0.122449 -21.5138 -6.73611 -2.61468
0.00418 0.0094 0.0162 0.00731 0.000499 0.000312 0.027 0.088 0.0401
3L:5925560-5925710:plus -23.0306 -0.688073 9.51376 3.89908 -17.5306 -28.5612 -3.33945 2.09722
4.73394 0.00505 0.013 0.000189 0.00604 0.0054 0.0636 0.000621 0.00832 0.00342
3L:603900-604050:minus -13.5204 3.84404 3.09174 8.6055 -17.3776 -17.051 13.5229 3.33333 5.66055
0.00097 0.00255 0.00383 0.000567 0.00419 0.00479 2.68e-06 0.00525 0.00227
3L:603900-604050:plus -32.7755 0.697248 2.19266 7.27523 -16.449 -17.1224 -23.9266 2.01389 -2.40367
0.0234 0.00807 0.00538 0.00109 0.00307 0.00499 0.0406 0.00858 0.0376

3L:6048060-6048210:minus -23.2653 1.45872 10.7431 3.88991 -17.4898 -9.27551 -8.18349 2.51389 3.51376
0.00593 0.00613 0.0000926 0.00608 0.0053 0.00244 0.00218 0.00716 0.00566
3L:6048060-6048210:plus -22.3061 13.8807 6.88073 6.09174 -18.7449 -17.0102 -13.9725 -1.125 3.46789
0.00336 9.55e-06 0.000768 0.00235 0.0138 0.00453 0.00721 0.0234 0.00582
3L:605860-606010:minus -14.1837 2.46789 -2.3578 -0.0642202 -7.96939 -27.6327 -16.367 4.41667
-1.09174 0.00124 0.00422 0.024 0.0274 0.000785 0.043 0.0116 0.00338 0.0268
3L:605860-606010:plus -31.7041 0.550459 3.65138 5.16514 -17.9796 -9.79592 -26.4495 0.458333 7.88991
0.0146 0.00851 0.00308 0.00424 0.00726 0.00377 0.0565 0.0145 0.000688
3L:6068340-6068490:minus -31.5918 4.33945 2.34862 3.42202 -8.67347 -17.3469 -11.4587 4.41667 -2.6055
0.0136 0.00211 0.00508 0.00751 0.00154 0.0058 0.0041 0.00338 0.04
3L:6068340-6068490:plus -32.7449 1.68807 2.77064 5.45872 -7.89796 -8.79592 -26.3761 -1.47222 -4.49541
0.0228 0.00563 0.00433 0.00363 0.000678 0.0019 0.056 0.0258 0.0638
3L:6087780-6087930:minus -31.7347 -1.01835 13.7431 1.89908 -17.4898 -26.898 -20.6422 4.75 -0.623853
0.0149 0.0144 0.0000103 0.0151 0.0053 0.0302 0.0231 0.00293 0.0232
3L:6087780-6087930:plus -22.0714 -0.00917431 3.55046 7.34862 -18.1224 -8.93878 -10.1743 4.95833
-2.53211 0.00299 0.0103 0.00321 0.000998 0.00782 0.00201 0.00318 0.00267 0.0392
3L:6088180-6088330:minus -41.1735 -0.669725 2.11009 1.41284 -18.4184 -17.3469 -24.6147 7.66667
1.23853 0.0479 0.0129 0.00554 0.0183 0.0101 0.0058 0.0448 0.000704 0.0132
3L:6088180-6088330:plus -22.2959 3.43119 0.284404 4.65138 -18.0816 -8.42857 -14.0275 9.22222 6.37615
0.00335 0.00297 0.0105 0.00471 0.00759 0.00104 0.0073 0.000278 0.00159
3L:613360-613510:minus -23.7041 -5.7156 4.30275 3.62385 -25.9286 -18.5612 -8.80734 -1.61111 1.55963
0.00669 0.0531 0.00237 0.00673 0.0168 0.0132 0.00247 0.0268 0.0114
3L:613360-613510:plus -22.9286 1.66055 3.22936 5.31193 -17.7143 -18.0816 -20.6147 1.26389 1.13761
0.0046 0.00569 0.00363 0.00393 0.00627 0.00934 0.0229 0.0111 0.0136
3L:614840-614990:minus -21.7653 4.54128 1.89908 3.84404 -26.9286 -26.6735 -1.80734 5.54167 5.59633
0.00237 0.00195 0.006 0.00624 0.0214 0.0256 0.000425 0.00205 0.00234
3L:614840-614990:plus -31.9694 3.77064 -1.49541 2.24771 -18.449 -8.42857 -12.8991 0.347222 -6.29358
0.0168 0.00262 0.0186 0.0127 0.0107 0.00104 0.00565 0.015 0.0955
3L:6167040-6167190:minus -22.6633 5.13761 -1.31193 4.48624 -7.89796 -17.8571 -21.2477 6.31944 -0.220183
0.00384 0.00154 0.0176 0.00487 0.000678 0.00719 0.0257 0.00142 0.0201
3L:6167040-6167190:plus -41.4082 6.11927 6.11927 6.56881 -18.4898 -8.53061 -16.1743 1.86111 -4.37615
0.0557 0.001 0.00109 0.0017 0.0116 0.00136 0.0112 0.00905 0.0617
3L:616760-616910:minus -32.1122 6.0367 8.3945 6.73394 -17.449 -19.051 -14.4862 2.98611 10.3394
0.0178 0.00104 0.000354 0.00152 0.00484 0.0165 0.00807 0.006 0.000112
3L:616760-616910:plus -13.6735 5.11009 3.11009 7.00917 -18.4592 -17.7857 -23.0275 5.66667 10.3394
0.00103 0.00156 0.00381 0.00126 0.0109 0.0068 0.0352 0.00194 0.000112
3L:6175000-6175150:minus -21.9286 -3.13761 4.40367 2.95413 -8.64286 9.60204 -21.4679 4.95833 -7.6055
0.0027 0.0268 0.00228 0.00917 0.00139 0.0000248 0.0268 0.00267 0.126
3L:6175000-6175150:plus -41.5816 -2.55046 3.79817 4.14679 -28.1939 -9.7551 -22.1651 2.66667 4.6789
0.061 0.0227 0.00291 0.00542 0.0482 0.00358 0.0304 0.00677 0.00347
3L:6178060-6178210:minus -32.8469 13.3878 5.6055 0.00917431 -17.449 -8.42857 -22.7523 0.513889
-0.12844 0.0243 0.0000869 0.00137 0.0269 0.00484 0.00104 0.0336 0.0142 0.0193
3L:6178060-6178210:plus -21.7755 0.366972 1.99083 3.26606 -7.67347 -17.051 -5.04587 4.77778 2.57798
0.00241 0.00908 0.0058 0.00802 0.00055 0.00479 0.00101 0.00289 0.0078
3L:6182160-6182310:minus -20.6224 0.412844 -0.743119 7.15596 -18.7143 0.0204082 -28.1468 -2.80556
-0.743119 0.0017 0.00893 0.0147 0.00117 0.0136 0.000377 0.0702 0.0368 0.0243
3L:6182160-6182310:plus -30.5204 4.19266 0.165138 0.633028 -18.4898 -8.68367 -15.3119 -1.94444 2.11009
0.00951 0.00224 0.011 0.0229 0.0116 0.00157 0.00954 0.0294 0.00898
3L:6182860-6183010:minus -32.4898 -4.6055 2.54128 -1.80734 -28.1939 -7.94898 -24.1101 3.69444 1.44037
0.02 0.0398 0.00473 0.0479 0.0482 0.00074 0.0417 0.00455 0.012
3L:6182860-6183010:plus -33.2653 5.9633 0.33945 4.0367 10.6939 -18.3469 -15.2661 -3.18056 4.18349
0.0277 0.00107 0.0104 0.00578 7.35e-06 0.0124 0.00946 0.0404 0.00404
3L:6186280-6186430:minus -31.8878 4.75229 -3.53211 6.00917 -7.63265 -9.68367 -13.2569 3.54167 -0.541284
0.0164 0.0018 0.0334 0.00255 0.000479 0.00342 0.00613 0.00483 0.0223

3L:6186280-6186430:plus -23.7347 4.47706 5.33028 2.6422 -17.4184 -18.3469 -18.9541 3.48611 7.51376
0.00673 0.002 0.00155 0.0107 0.00457 0.0124 0.0173 0.00494 0.000984
3L:6209160-6209310:minus -23.4898 1.66972 -4.2844 0.366972 -9.20408 -9.7551 -22.6055 5.77778 -2.27523
0.00639 0.00567 0.041 0.0244 0.00262 0.00358 0.0328 0.00184 0.036
3L:6209160-6209310:plus -24.0714 0.577982 -0.119266 7.27523 -26 -17.2755 -7.56881 2.20833 5.44037
0.00731 0.00843 0.0121 0.00109 0.0171 0.00544 0.00192 0.008 0.00259
3L:6209700-6209850:minus -23.0306 -0.908257 4.29358 9.49541 -17.602 -17.0102 -2.85321 10.8056
7.84404 0.00505 0.0139 0.00238 0.000308 0.00547 0.00453 0.000547 0.0000893 0.000701
3L:6209700-6209850:plus -30.3673 7.12844 -2.89908 6.08257 -27.1633 -17.4898 4.13761 3.97222 -2.21101
0.00897 0.000607 0.028 0.00236 0.025 0.0059 0.0000785 0.00407 0.0354
3L:6210340-6210490:minus -4.70408 7.9633 -1.87156 3.70642 -8.33673 -8.38776 -15.5596 -0.430556
9.66055 0.000186 0.00039 0.0208 0.0066 0.00101 0.00101 0.01 0.0191 0.000268
3L:6210340-6210490:plus -40.4796 0.376147 5.6422 10.1468 -27.602 -7.79592 -13.5229 8.56944 3.46789
0.0407 0.00905 0.00135 0.000128 0.0364 0.000723 0.00652 0.000418 0.00582
3L:6211120-6211270:minus 5.21429 0.0366972 9.3578 10.5963 -18.4898 -27.2653 -10.2752 -2.875
5.7156 0.0000129 0.0102 0.000206 0.0001 0.0116 0.0333 0.00324 0.0375 0.00217
3L:6211120-6211270:plus -41.8878 4.31193 -3.66055 2.93578 -27.1939 -9.60204 -19.3303 6.69444 -0.761468
0.0672 0.00213 0.0346 0.00927 0.0267 0.00334 0.0184 0.00118 0.0244
3L:6211740-6211890:minus -24.2551 0.0366972 8.62385 10.5963 -17.4898 -19.0816 -22.6239 -3.72222
-0.550459 0.00768 0.0102 0.000311 0.0001 0.0053 0.017 0.0329 0.0461 0.0225
3L:6211740-6211890:plus -21.9286 -2.33945 -2.79817 7.17431 -26.3061 -26.9796 -5.54128 0.0972222
2.36697 0.0027 0.0214 0.0272 0.00115 0.0181 0.0306 0.00116 0.0162 0.00828
3L:6255680-6255830:minus -22.2551 -2.57798 4.08257 6.49541 -18.4898 -27.6735 -1.77064 7.45833 3.14679
0.00327 0.0229 0.00259 0.00176 0.0116 0.0443 0.000421 0.000789 0.00666
3L:6255680-6255830:plus -32.4388 -2.3578 4.78899 4.50459 -9.12245 -9.5 -2.89908 -0.208333 3.29358
0.0195 0.0215 0.00194 0.00484 0.00238 0.00294 0.000553 0.0178 0.00623
3L:6256960-6257110:minus -32.8878 -0.348624 0.917431 -1.48624 -17.1531 -19.051 -9.7156 0.638889
-0.587156 0.0247 0.0116 0.00851 0.0431 0.00364 0.0165 0.00292 0.0137 0.0227
3L:6256960-6257110:plus -20.8878 5.83486 -0.880734 5.48624 -18.7551 -26.7857 -20.9725 -3.625
7.04587 0.0018 0.00114 0.0154 0.00357 0.0144 0.0266 0.0245 0.045 0.00132
3L:6257700-6257850:minus -41.0306 -0.917431 0.238532 5.43119 -18.4898 -9.37755 -19.5872 6.11111
1.27523 0.0464 0.014 0.0107 0.00371 0.0116 0.00265 0.0192 0.00157 0.013
3L:6257700-6257850:plus -41.1837 -2.43119 4.72477 6.72477 -18.0102 -17.0816 -22.4037 3.63889 -0.605505
0.0485 0.022 0.00199 0.00154 0.00732 0.00487 0.0317 0.00465 0.023
3L:626020-626170:minus -23.1939 -1.41284 2.86239 4.22018 -27.9388 -8.79592 -13.6147 2.76389 5.90826
0.00558 0.0163 0.00418 0.00531 0.0415 0.0019 0.00665 0.00652 0.00198
3L:626020-626170:plus -22.9592 -4.17431 0.0275229 3.54128 -17.1939 -26.898 -15.7982 -3.69444 6.7156
0.00475 0.0354 0.0115 0.00702 0.00377 0.0302 0.0105 0.0458 0.00144
3L:628980-629130:minus -22.6633 -1.29358 3.50459 10.8624 -35.7449 -8.72449 -15.5872 1.18056 5.90826
0.00384 0.0157 0.00326 0.0000687 0.0689 0.00169 0.0101 0.0114 0.00198
3L:628980-629130:plus -32.2143 4.23853 6.92661 3.21101 -8.93878 -8.53061 -15.4404 7.15278 2.75229
0.0182 0.0022 0.000753 0.00822 0.00216 0.00136 0.00978 0.000928 0.00748
3L:629620-629770:minus -41.6735 4.15596 -0.431193 3.76147 -27.2347 0.0510204 -21.3486 8.38889
0.908257 0.0637 0.00227 0.0133 0.00645 0.0283 0.000349 0.0262 0.000466 0.0147
3L:629620-629770:plus -23.1122 -4.69725 2.31193 6.36697 0.918367 -17.051 -15.5229 7.69444 -1.53211
0.0053 0.0407 0.00515 0.00193 0.000142 0.00479 0.00994 0.000693 0.0296
3L:630040-630190:minus -32.8878 0.357798 -1.54128 4.14679 -18.7551 -18.4898 -8.3945 1.06944 -2.89908
0.0247 0.00911 0.0188 0.00542 0.0144 0.0129 0.00228 0.0119 0.0434
3L:630040-630190:plus -32.4388 3.61224 3.99083 4.78899 -18.1531 -9.94898 -17.1927 3.69444 3.6055
0.0195 0.00167 0.00269 0.00456 0.00829 0.00393 0.0132 0.00455 0.00532
3L:630780-630930:minus -13.0714 1.91743 3.48624 3.12844 -18.7857 -16.0102 -5.41284 2.98611 3.6789
0.000594 0.00518 0.00329 0.00857 0.0148 0.00406 0.00112 0.006 0.00506
3L:630780-630930:plus -31.4796 2.50459 2.06422 5.75229 -26.898 -9.23469 -13.9908 -8.34722 1.13761
0.0128 0.00417 0.00564 0.00296 0.0203 0.00238 0.00724 0.118 0.0136

3L:634000-634150:minus -14.2245 3.61224 -2.89908 -1.41284 -18.7959 -19.3061 -8.6055 11.5833 8.19266
0.00126 0.00167 0.028 0.0421 0.0148 0.0187 0.00237 0.0000457 0.000548
3L:634000-634150:plus -14.6327 6.72477 -2.93578 9.23853 -18.3776 -16.0102 -11.6789 -5.31944 0.752294
0.0015 0.000747 0.0283 0.000424 0.00926 0.00406 0.0043 0.0661 0.0152
3L:634580-634730:minus -32.8163 1.70642 8.87156 5.49541 -8.67347 -9.23469 -11.6514 1.73611 -1.0367
0.024 0.00559 0.000273 0.00355 0.00154 0.00238 0.00427 0.00946 0.0264
3L:634580-634730:plus -31.449 0.651376 5.45872 5.31193 -28.4184 -8.72449 -16.3945 7.48611 -0.688073
0.0123 0.00821 0.00147 0.00393 0.056 0.00169 0.0116 0.000777 0.0238
3L:639620-639770:minus -21.4082 6.56881 2.14679 4.04587 -35.6735 -25.5306 -17.0642 0.875 2.89908
0.00198 0.000806 0.00547 0.00572 0.0668 0.0205 0.0129 0.0127 0.00722
3L:639620-639770:plus -31.1429 -2.08257 -0.46789 7.10092 -26.5918 -0.0510204 -9.79817 0.319444 3.88991
0.0108 0.0199 0.0135 0.00121 0.0186 0.000429 0.00296 0.0151 0.00467
3L:640400-640550:minus -22.4082 -3.29358 4.83486 2.20183 -8.86735 -18.1224 -12.5413 1.25 3.24771
0.00345 0.028 0.00191 0.013 0.00173 0.00988 0.00521 0.0112 0.00636
3L:640400-640550:plus -4.67347 -2.78899 -0.550459 4.33028 -8.44898 -37.2551 -18.9541 4.91667 5.80734
0.000182 0.0243 0.0139 0.00511 0.00109 0.142 0.0173 0.00272 0.00205
3L:641300-641450:minus -21.5102 0.66055 4.92661 5.57798 -18.7143 0.0510204 -21.1284 4.80556
2.27523 0.00202 0.00818 0.00183 0.0034 0.0136 0.000349 0.0252 0.00286 0.00846
3L:641300-641450:plus -21.7041 -1.2844 4.68807 6.02752 -18.1939 -28.6327 -16.5596 -10.9861 1.83486
0.00231 0.0157 0.00203 0.00246 0.0086 0.066 0.0119 0.178 0.0101
3L:647840-647990:minus -20.8163 1.56881 2.3211 6.47706 -27.1939 -18.051 -10.2661 4.18056 -1.72477
0.00177 0.00588 0.00513 0.00179 0.0267 0.00886 0.00324 0.00373 0.031
3L:647840-647990:plus -32.7041 -2.0367 9.33945 3.09174 -17.4898 -17.2755 -20.8807 4.65278 -0.174312
0.0222 0.0196 0.000208 0.00867 0.0053 0.00544 0.0241 0.00305 0.0196
3L:6481640-6481790:minus -22.9184 5.46789 15 7.29358 -17.5612 1.31633 -20.789 3.31944 3.45872
0.00447 0.00134 1.62e-06 0.00104 0.00544 0.000066 0.0237 0.00528 0.00591
3L:6481640-6481790:plus -23.2653 -2.50459 4.22018 6.21101 -18.1122 -0.173469 -16.4037 10.8333
-1.17431 0.00593 0.0225 0.00245 0.00213 0.00771 0.000456 0.0116 0.0000874 0.0275
3L:648600-648750:minus -24.1531 0.577982 6.21101 9.33028 -8.89796 -9.16327 -12.0642 0.0138889
-1.17431 0.00744 0.00843 0.00105 0.000374 0.00189 0.00223 0.00468 0.0167 0.0275
3L:648600-648750:plus -13.3367 -0.93578 4.78899 10.8624 -18.2245 -27.6327 -8.38532 4.18056 7.6422
0.0008 0.014 0.00194 0.0000687 0.00877 0.043 0.00227 0.00373 0.000893
3L:6488320-6488470:minus -23.8571 12.4495 8.6422 6.48624 -18.7857 -18.7857 -9.58716 1.59722 2.20183
0.00687 0.0000341 0.000308 0.00177 0.0148 0.0146 0.00285 0.00993 0.00869
3L:6488320-6488470:plus -13.602 12.0734 10.5046 7.62385 -18.8265 -16.7143 -24.6881 6.72222 2.42202
0.000995 0.0000443 0.000105 0.000759 0.0149 0.00419 0.0452 0.00116 0.00816
3L:6488700-6488850:minus -42.2143 -1.47706 -0.477064 3.34862 -27.7551 -10.0204 -21.8716 5.40278
-1.9633 0.0712 0.0166 0.0135 0.0077 0.0385 0.00399 0.0288 0.00219 0.0329
3L:6488700-6488850:plus 24.6224 6.9633 7.63303 6.47706 -27.2653 -18.2041 -9.70642 -0.430556
1.25688 1.64e-08 0.000662 0.000531 0.00179 0.0302 0.0103 0.00291 0.0191 0.0131
3L:6489160-6489310:minus -30.6633 2.92661 -0.266055 1.9633 -26.1939 -0.244898 -19.6514 2.73611
-8.97248 0.00976 0.00357 0.0126 0.0146 0.0175 0.000496 0.0194 0.00659 0.165
3L:6489160-6489310:plus -23.8469 5.51376 5.41284 2.61468 -27.602 -27.602 -13.6972 0.263889 4.77982
0.00685 0.00131 0.00149 0.0109 0.0364 0.0416 0.00678 0.0154 0.00339
3L:6489780-6489930:minus -13.4898 4.05505 -1.84404 0.981651 -19.0918 -9.57143 -10.6606 1.83333 1.73394
0.000946 0.00235 0.0206 0.0207 0.0163 0.00326 0.00349 0.00914 0.0106
3L:6489780-6489930:plus -12.0408 5.11927 -3.24771 7.15596 -8.56122 -17.7551 -12.7706 -6.30556 -0.724771
0.000311 0.00155 0.0309 0.00117 0.00112 0.00643 0.00549 0.0809 0.0241
3L:649520-649670:minus -31.9694 -2.17431 8.45872 7 -17.7143 0.204082 -21.0826 -0.305556 3.25688
0.0168 0.0204 0.000341 0.00132 0.00627 0.000296 0.025 0.0184 0.00632
3L:649520-649670:plus -22.8163 -0.486239 3.90826 3.78899 -16.4184 -27.3061 -17.5138 2.56944 1.74312
0.00418 0.0121 0.00278 0.00632 0.00304 0.0336 0.0139 0.00701 0.0105
3L:651620-651770:minus -22.7347 -1.41284 -2.3945 1.53211 -19.0102 -26.8265 -8.05505 2.29167 3.66972
0.00403 0.0163 0.0242 0.0174 0.0157 0.0285 0.00213 0.00776 0.00514

3L:651620-651770:plus -12.3367 5.65138 4.77982 2.25688 -27.9694 -9.57143 -17.9633 0.25 7.73394 0.000393
0.00124 0.00195 0.0127 0.0437 0.00326 0.0149 0.0155 0.000784
3L:654120-654270:minus -23.4796 9.58716 3.95413 6.56881 -18.0102 0.826531 -28.9174 -2.79167 -4.25688
0.00634 0.000165 0.00273 0.0017 0.00732 0.000153 0.078 0.0367 0.0593
3L:654120-654270:plus -24.0714 1.72477 11.9725 7.2844 -27.2653 -26.602 -13.0275 -1.25 9.92661
0.00731 0.00555 0.0000439 0.00105 0.0302 0.0248 0.00582 0.0242 0.000193
3L:6543740-6543890:minus -21.8878 -0.247706 -0.899083 3.19266 -17.7857 -10.0204 -7.04587 3.16667
2.88073 0.00258 0.0112 0.0155 0.00829 0.00676 0.00399 0.00171 0.0056 0.00727
3L:6543740-6543890:plus -13.898 13.5306 1.37615 7.34862 -28.3061 -0.0204082 -8.92661 -6.45833
-1.44037 0.00111 0.0000206 0.00724 0.000998 0.0553 0.000411 0.00252 0.0834 0.029
3L:6545120-6545270:minus -21.6633 2.48624 13.0459 -0.633028 -17.9388 -7.5 -8.15596 2.73611 9.30275
0.00223 0.0042 0.0000199 0.0327 0.00715 0.000614 0.00217 0.00659 0.00038
3L:6545120-6545270:plus -23.2653 0.311927 3.59633 9.3211 -19.051 -9.23469 -14.5505 4.72222 2.99083
0.00593 0.00925 0.00315 0.000391 0.0162 0.00238 0.00818 0.00296 0.00706
3L:6546280-6546430:minus -24.3265 3.90816 -0.688073 5.43119 -17.7857 -17.7857 -10.3303 4.41667
1.74312 0.00784 0.00102 0.0145 0.00371 0.00676 0.0068 0.00328 0.00338 0.0105
3L:6546280-6546430:plus -32.7449 -1.77982 2.87156 5.90826 -18.449 -9.02041 -20.2294 0.291667 6.91743
0.0228 0.0182 0.00417 0.00271 0.0107 0.00213 0.0214 0.0153 0.00136
3L:6599120-6599270:minus -12.8163 0.834862 2.52294 4.13761 -27.5 -9.57143 -16.8716 0.444444 7.63303
0.000489 0.00769 0.00476 0.00546 0.0337 0.00326 0.0126 0.0145 0.000928
3L:6599120-6599270:plus -33.3367 0.174312 6.27523 2.86239 10.0918 -19.1224 -18.6055 -1.38889 -3.63303
0.0281 0.00971 0.00102 0.00954 0.0000251 0.0175 0.0164 0.0252 0.0511
3L:6687500-6687650:minus -32.551 -0.220183 0.302752 2.77982 -27.1939 -8.72449 -25.6514 -3.56944
-0.275229 0.0207 0.0111 0.0105 0.00997 0.0267 0.00169 0.0512 0.0445 0.0206
3L:6687500-6687650:plus -13.4082 0.715596 1.46789 9.59633 -17.1837 -27.5612 -10.9358 3.47222 0.963303
0.000867 0.00802 0.007 0.000275 0.00366 0.0393 0.00369 0.00497 0.0145
3L:6692980-6693130:minus -31.6327 -2.70642 7.11009 1.69725 -18.1531 0.316327 -21.4587 -0.277778
9.14679 0.0138 0.0238 0.000691 0.0163 0.00829 0.000243 0.0267 0.0182 0.000414
3L:6692980-6693130:plus -22.6224 -2.01835 7.3945 4.0367 -17.449 0.979592 -14.0092 5.41667 2.53211
0.00371 0.0195 0.000597 0.00578 0.00484 0.000136 0.00727 0.00218 0.00791
3L:6693520-6693670:minus -13.1122 0.366972 6.82569 4.33945 -26.8878 -9.23469 -15.5688 9.55556 4.91743
0.000632 0.00908 0.000788 0.00509 0.0199 0.00238 0.01 0.000223 0.00321
3L:6693520-6693670:plus -31.4796 5.24771 8.77064 2.56881 -17.4184 -9.37755 -19.7064 1.09722 -1.68807
0.0128 0.00147 0.000287 0.0111 0.00457 0.00265 0.0196 0.0118 0.0308
3L:6696540-6696690:minus -12.9286 0.477064 2.91743 2.49541 -17.8878 -0.173469 -12 -3.29167 0.59633
0.000539 0.00873 0.0041 0.0114 0.00704 0.000456 0.00461 0.0416 0.016
3L:6696540-6696690:plus -12.4796 0.862385 12.9817 3.42202 -17.1531 -28.3367 -12.3945 1.22222 5.34862
0.000433 0.00761 0.0000211 0.00751 0.00364 0.0606 0.00504 0.0113 0.00271
3L:6736160-6736310:minus -12.7755 11.9725 -0.844037 5.98165 -27.2653 -19.051 -5.6055 -0.708333
5.70642 0.000477 0.0000466 0.0152 0.00258 0.0302 0.0165 0.00118 0.0207 0.00222
3L:6736160-6736310:plus -31.7755 12.0917 -3.31193 2.07339 -17.5306 9.23469 -12.3394 -4.5 -1 0.0157
0.0000437 0.0315 0.0138 0.0054 0.0000427 0.00497 0.0552 0.0261
3L:6736700-6736850:minus -33.1429 -4.05505 0.385321 0.0642202 -28.3061 -8.64286 -28.5413 3.125
3.42202 0.0272 0.0343 0.0102 0.0264 0.0553 0.00154 0.074 0.00569 0.00597
3L:6736700-6736850:plus -22.7041 -5.50459 -0.110092 4.37615 -26.2245 -16.9796 -17.8899 1.76389
1.11009 0.00395 0.0503 0.012 0.00504 0.0175 0.0044 0.0147 0.00937 0.0138
3L:6740280-6740430:minus -13.5918 4.6055 -0.0825688 5.90826 -18.1531 -18.0102 -5.58716 0.347222
5.18349 0.00099 0.00191 0.0119 0.00271 0.00829 0.00812 0.00117 0.015 0.00295
3L:6740280-6740430:plus -41.9592 3.64286 -2.3211 0.357798 -17.9388 -9.57143 -24.1193 5.375 -2.40367
0.0679 0.00159 0.0237 0.0245 0.00715 0.00326 0.0418 0.00222 0.0376
3L:6744800-6744950:minus -41.5918 -1.37615 -1.56881 1.14679 -18.051 -8.53061 -18.8716 0.833333 3.46789
0.0623 0.0161 0.019 0.0198 0.00746 0.00136 0.0171 0.0128 0.00582
3L:6744800-6744950:plus -41.6224 -5.40367 0.293578 7.51376 -8.20408 -8.72449 -35.2477 0.944444 -4.27523
0.0628 0.049 0.0105 0.000836 0.000894 0.00169 0.164 0.0124 0.0596

3L:6745440-6745590:minus -22.6327 2.06422 4.33945 2.84404 -28.4592 -18.0102 -16.3303 2.93056 5.3945
0.00373 0.0049 0.00234 0.00961 0.0568 0.00812 0.0115 0.00612 0.00263
3L:6745440-6745590:plus -30.5918 -4.84404 0.743119 5.45872 -7.30612 -16.9388 -17.5872 8.04167 2.05505
0.00963 0.0423 0.00903 0.00363 0.000378 0.00433 0.014 0.00057 0.00914
3L:6745800-6745950:minus -30.2857 -2.83486 -4.45872 2.16514 -17.3776 -9.42857 -19.2661 -6.93056 8.88073
0.00887 0.0246 0.0429 0.0132 0.00419 0.0027 0.0182 0.0913 0.000468
3L:6745800-6745950:plus -24.2959 7.25688 -0.12844 1.95413 -17.7551 -17.8571 -19.3394 3.54167 2.55046
0.00781 0.000568 0.0121 0.0147 0.00658 0.00719 0.0184 0.00483 0.00785
3L:6747960-6748110:minus -23.1837 -0.211009 -0.431193 5.65138 -18.449 -19.0102 -24.1743 -1.76389
-7.27523 0.00553 0.0111 0.0133 0.00322 0.0107 0.0159 0.0421 0.028 0.119
3L:6747960-6748110:plus -40.551 2.21101 3.34862 3.6055 -19.0918 -7.65306 -13.7706 0.833333 7.6422
0.0418 0.00464 0.00347 0.00686 0.0163 0.000647 0.00689 0.0128 0.000893
3L:6749440-6749590:minus -24.1531 0.256881 2.9633 7.6055 -26.1939 -26.5612 -7.7156 1.79167 3.45872
0.00744 0.00944 0.00403 0.000794 0.0175 0.0237 0.00198 0.00928 0.00591
3L:6749440-6749590:plus -31.7755 -2.21101 0.348624 6.27523 -18.1224 -18.0102 -24.1927 -2.91667 -2.98165
0.0157 0.0206 0.0103 0.00206 0.00782 0.00812 0.0422 0.0378 0.0445
3L:680320-680470:minus -14.4184 2.33028 -0.422018 0.53211 -17.3776 -18.5714 -6.3211 3.80556
-0.0275229 0.00137 0.00445 0.0133 0.0234 0.00419 0.0136 0.00143 0.00435 0.0186
3L:680320-680470:plus -32.2551 2.80734 2.88991 1.13761 -26.5306 -19.1224 -18.3028 1.09722 9.42202
0.0184 0.00373 0.00414 0.0198 0.0185 0.0175 0.0157 0.0118 0.000349
3L:681900-682050:minus -23.7755 0.862385 15.6239 -0.0550459 -8.64286 -8.79592 -19.055 -0.111111
0.394495 0.00678 0.00761 5.44e-07 0.0272 0.00139 0.0019 0.0176 0.0173 0.0166
3L:681900-682050:plus -32.4082 -1.53211 0.119266 0.798165 -8.82653 -18.2755 -19.6972 1.44444 -5.3211
0.0191 0.0169 0.0111 0.0218 0.00163 0.0112 0.0196 0.0105 0.0777
3L:682400-682550:minus -24.2245 -0.284404 0.862385 1.84404 -26.4898 -19.4184 -20.8532 1.77778
-0.0275229 0.00763 0.0114 0.00867 0.0154 0.0183 0.0198 0.0239 0.00932 0.0186
3L:682400-682550:plus -32.4082 1.6789 -0.669725 1.68807 -26.0408 -25.5306 -15.7431 1.875 3.46789
0.0191 0.00565 0.0144 0.0164 0.0171 0.0205 0.0104 0.00901 0.00582
3L:685600-685750:minus -23.5918 0.678899 4.26606 2.41284 -17.1531 -8.5 -11.3119 2.65278 4.13761
0.00656 0.00813 0.00241 0.0118 0.00364 0.00126 0.00398 0.0068 0.00408
3L:685600-685750:plus -40.6735 3.64286 0.0458716 9.59633 -18.7143 -7.5 -18.3028 1.56944 -0.174312
0.0426 0.00159 0.0114 0.000275 0.0136 0.000614 0.0157 0.01 0.0196
3L:690380-690530:minus -14.7449 6.26606 0.853211 2.26606 -19.0204 -18.3469 -23.2752 4.76389 5.33028
0.00157 0.000934 0.0087 0.0126 0.0158 0.0124 0.0367 0.00291 0.00273
3L:690380-690530:plus -14.6735 0.376147 15.0092 3.11009 0.94898 -8.65306 -8.88991 5.34722 3.46789
0.00152 0.00905 1.55e-06 0.00862 0.000134 0.00156 0.00251 0.00225 0.00582
3L:692820-692970:minus -33.0408 6.25688 -1.29358 2.87156 2.14286 -9.53061 -25.0826 6.22222 -5.14679
0.0264 0.000938 0.0175 0.00948 0.0000316 0.0031 0.0477 0.00149 0.0751
3L:692820-692970:plus -40.1429 -0.146789 -1.12844 2.94495 10.7245 -17.5714 -27.3853 3.47222 -2.25688
0.0363 0.0108 0.0166 0.0092 5.2e-06 0.00616 0.0635 0.00497 0.0357
3L:6935380-6935530:minus -34.0714 -2.84404 10.844 3.23853 -8.67347 -17.9796 -12.0183 -0.25 -0.220183
0.031 0.0247 0.000088 0.00815 0.00154 0.00764 0.00463 0.0181 0.0201
3L:6935380-6935530:plus -32.7755 13.4592 -3.82569 4.22936 -18.7857 -27.4082 -18.9908 0.222222 3.46789
0.0234 0.000064 0.0362 0.00525 0.0148 0.0356 0.0174 0.0156 0.00582
3L:6935880-6936030:minus -23.5204 1.04587 0.155963 2.7156 -18.4898 -27.3367 -11.7798 -4.75 1.08257
0.00645 0.00712 0.011 0.0102 0.0116 0.0347 0.00439 0.0584 0.0139
3L:6935880-6936030:plus -40.1429 -0.311927 -2.51376 3.11009 -9.23469 -9.30612 -6.89908 4.70833
7.31193 0.0363 0.0115 0.0251 0.00862 0.00277 0.00257 0.00165 0.00298 0.00112
3L:6951220-6951450:minus -4.26531 3.90816 14.9633 7.29358 -27.1939 -8.42857 -19.7248 0.75 -3.13761
0.000132 0.00102 1.77e-06 0.00104 0.0267 0.00104 0.0196 0.0132 0.0461
3L:6951220-6951450:plus -31.7041 2.19266 1.73394 7 0.877551 -18.3469 -12.9817 0.25 -0.862385
0.0146 0.00468 0.00638 0.00132 0.00019 0.0124 0.00576 0.0155 0.0251
3L:6955360-6955510:minus -31.7449 -5.17431 1.69725 0.275229 -18.7551 -26.5306 -9.53211 -0.277778
5.49541 0.0152 0.0462 0.00646 0.025 0.0144 0.023 0.00282 0.0182 0.0025

3L:6955360-6955510:plus -3.81633 -1.36697 -2.17431 9.59633 -17.449 -18.7857 -10.8257 -2.81944 -5.20183
0.000106 0.0161 0.0227 0.000275 0.00484 0.0146 0.00361 0.0369 0.0759
3L:6956420-6956570:minus -40.5102 -2.62385 -2.83486 7.51376 -18.0816 0.94898 -10.9725 1.18056 3
0.0409 0.0232 0.0275 0.000836 0.00759 0.00014 0.00371 0.0114 0.00702
3L:6956420-6956570:plus -31.3673 -0.293578 0.844037 3.6055 -26.7449 -27.5306 -15.9083 -1.90278
5.95413 0.0116 0.0114 0.00872 0.00686 0.0195 0.0374 0.0107 0.029 0.00192
3L:6957360-6957510:minus -22.7041 -1.31193 1.66972 2.62385 -17.3776 -7.65306 -15.7798 -5.58333 11.8532
0.00395 0.0158 0.00652 0.0108 0.00419 0.000647 0.0104 0.0698 0.0000428
3L:6957360-6957510:plus -21.6939 -2.52294 2.16514 2.89908 -18.1939 -27.2653 -3.6422 0.458333
7.58716 0.00226 0.0226 0.00544 0.00938 0.0086 0.0333 0.000675 0.0145 0.000954
3L:6957720-6957870:minus -13.449 -0.293578 3.0367 5.15596 -17.8163 -17.051 -12.8165 -0.527778
-0.559633 0.000914 0.0114 0.00392 0.00426 0.00682 0.00479 0.00554 0.0196 0.0226
3L:6957720-6957870:plus -31.2857 1.72477 -3.90826 1.16514 -18.4796 -27.4082 -17.4862
2.36111 5.6055 0.0113 0.00555 0.037 0.0197 0.011 0.0356 0.0138 0.00757 0.00232
3L:6962100-6962250:minus -24.1531 -0.605505 2.83486 5.73394 10.3571 -8.42857 -28.4404
1.02778 -1.21101 0.00744 0.0126 0.00423 0.00307 0.0000208 0.00104 0.073 0.012 0.0277
3L:6962100-6962250:plus -22.9184 -0.559633 -1.75229 -0.40367 -17.4898 -18.3061 -11.4404
11.625 -3.47706 0.00447 0.0124 0.0201 0.0302 0.0053 0.0114 0.00409 0.0000441 0.0493
3L:6962640-6962790:minus -40.6633 -0.678899 1.80734 -0.321101 -28.2347 -9.72449
-31.8624 1.55556 -2.36697 0.0426 0.0129 0.00621 0.0294 0.051 0.00352 0.116 0.0101 0.0371
3L:6962640-6962790:plus -31.8061 7.25688 4.27523 1.26606 -18.4184 -9.72449 -18.5229 1.86111
5.55963 0.0158 0.000568 0.0024 0.0191 0.0101 0.00352 0.0162 0.00905 0.0024
3L:6969740-6969890:minus -23 9.31193 0.59633 3.7156 0.94898 -17.3469 2.16514 -0.777778 -6.3211
0.00498 0.00019 0.00949 0.00655 0.000134 0.0058 0.000157 0.0211 0.096
3L:6969740-6969890:plus -24.1429 7.78899 4.11927 8.98165 -8.11224 -27.3776 -9.56881 5.13889
5.00917 0.0074 0.000429 0.00255 0.000461 0.000838 0.0351 0.00284 0.00247 0.0031
3L:6970100-6970250:minus -32.4082 1.47706 2.13761 5.76147 -8.89796 -28.1224 -16.9266
7.58333 -0.220183 0.0191 0.00608 0.00549 0.00295 0.00189 0.0583 0.0127 0.000737 0.0201
3L:6970100-6970250:plus -13.7143 0.0183486 -0.119266 6.3578 -19.0102 0.795918 -24.7248
-1.76389 -2.66972 0.00105 0.0102 0.0121 0.00195 0.0157 0.000155 0.0455 0.028 0.0407
3L:6973080-6973230:minus -22.2959 3.66972 -1.58716 2.43119 0.846939 -26.5612 -6.55046
-1.76389 1.2844 0.00335 0.00272 0.0191 0.0117 0.000197 0.0237 0.00151 0.028 0.0129
3L:6973080-6973230:plus -23.5918 4.00917 6.37615 2.09174 -26.1939 0.469388 -19.2477 -0.805556
-3.53211 0.00656 0.0024 0.000974 0.0137 0.0175 0.000212 0.0182 0.0213 0.0501
3L:707760-707910:minus -24.7857 0.87156 -0.816514 2.88073 -28 -8.79592 -17.578 5.18056 9.97248
0.00829 0.00758 0.0151 0.00945 0.0444 0.0019 0.014 0.00242 0.00016
3L:707760-707910:plus -32.2245 -4.82569 0.440367 11.1927 -19.3163 -17.5612 -7.11927
4.26389 3.7156 0.0183 0.0421 0.01 0.0000254 0.0165 0.00604 0.00174 0.0036 0.00502
3L:708120-708270:minus -23.4082 0.431193 -1.77982 2.33945 -7.71429 -9.45918 -16.6606
2.98611 8.19266 0.00623 0.00887 0.0202 0.0122 0.000572 0.00279 0.0121 0.006 0.000548
3L:708120-708270:plus -22.8061 -2.92661 6.94495 5.94495 -7.33673 -9.53061 -8.18349
5.06944 9.34862 0.00414 0.0253 0.000746 0.00261 0.000388 0.0031 0.00218 0.00255 0.000371
3L:710660-710810:minus -32.8163 1.74312 -1.05505 0.486239 -18.5306 -18.0102 -15.7431
0.805556 -1.9633 0.024 0.00552 0.0162 0.0237 0.0118 0.00812 0.0104 0.013 0.0329
3L:710660-710810:plus -41.7041 -0.568807 -5.30275 -3.11927 -28.4898 -8.86735 -28.5229
7.25 -2.44954 0.0641 0.0125 0.0533 0.0707 0.0581 0.00198 0.0739 0.000882 0.0381
3L:7118560-7118710:minus 5.40816 6.6422 7.07339 7.21101 -28 -9.53061 -6.58716 7.02778 -2.44037
0.0000106 0.000778 0.000703 0.0011 0.0444 0.0031 0.00153 0.000991 0.0381
3L:7118560-7118710:plus -22.6224 14.4862 3.62385 3.62385 -17.3367 -18.3469 -12.8716 -2.31944
-3.82569 0.00371 5.19e-06 0.00312 0.00673 0.00391 0.0124 0.00561 0.0325 0.0531
3L:7150240-7150390:minus -10.8469 3.93578 3.26606 5.84404 -17.4184 -7.45918 -11.3853
12.3056 11.8073 0.000237 0.00246 0.00358 0.00289 0.00457 0.000582 0.00404 0.0000228 0.0000538
3L:7150240-7150390:plus -23.1531 6.52294 7.31193 2.19266 -27.3061 -0.173469 -25.844 2.95833
-2.36697 0.00542 0.000826 0.000623 0.0131 0.0315 0.000456 0.0525 0.00606 0.0371

3L:7151020-7151170:minus -32.0408 0.256881 2.38532 2.00917 -27.1939 -8.72449 -13.5229
0.375 -1.68807 0.0174 0.00944 0.00501 0.0142 0.0267 0.00169 0.00652 0.0149 0.0308
3L:7151020-7151170:plus -30.5102 2.92661 3.9633 7.6422 -17.7143 -18.8571 3.52294 11.1944
-0.183486 0.00947 0.00357 0.00272 0.000721 0.00627 0.0151 0.0000981 0.0000647 0.0197
3L:7231500-7231650:minus -41.3673 2.77064 -3.26606 -1.0367 -27.602 0.755102 -28.3945
8.16667 -3.59633 0.0551 0.00378 0.0311 0.0371 0.0364 0.000175 0.0726 0.00053 0.0507
3L:7231500-7231650:plus -40.3265 -1.12844 6.69725 2.30275 10.4286 -0.244898 -16.3578 0.0972222
-5.77064 0.0394 0.0149 0.000836 0.0124 0.0000139 0.000496 0.0115 0.0162 0.0846
3L:7234660-7234810:minus -21.9286 -0.302752 1.22936 2.82569 0.846939 -8.72449 -18.7248
1.20833 -0.284404 0.0027 0.0114 0.00763 0.00976 0.000197 0.00169 0.0167 0.0113 0.0207
3L:7234660-7234810:plus -33.4898 2.10092 0.559633 4.59633 -27.3061 -8.5 -25.9358 3.79167
1.26606 0.0286 0.00484 0.00961 0.00478 0.0315 0.00126 0.0531 0.00437 0.013
3L:7237020-7237170:minus -22.2959 3.70642 -2.22018 7.61468 -7.93878 0.683673 -12.1009
1.125 1.02752 0.00335 0.00268 0.0231 0.000779 0.000767 0.000184 0.00472 0.0117 0.0141
3L:7237020-7237170:plus -32.2143 -1.77064 2.77982 3.70642 1.17347 -18.3469 -24.2936 5.33333
-4.29358 0.0182 0.0182 0.00432 0.0066 0.000101 0.0124 0.0428 0.00226 0.06
3L:723720-723870:minus -31.7755 -2.37615 1.79817 2.74312 -26.8571 -19.3469 -10.4862
2.48611 7.22018 0.0157 0.0216 0.00623 0.0101 0.0198 0.0194 0.00337 0.00723 0.00119
3L:723720-723870:plus -40.1429 1.88073 1.33945 4.21101 -16.6735 -9.02041 -17.3394 5.31944
0.137615 0.0363 0.00524 0.00733 0.00532 0.00313 0.00213 0.0135 0.00227 0.0178
3L:7242440-7242590:minus -13.8163 13.4592 0.256881 4.3211 -17.0816 1.05102 -2.22936
8.56944 1.11927 0.00109 0.000064 0.0106 0.00514 0.00334 0.000107 0.000469 0.000418 0.0137
3L:7242440-7242590:plus -30.7041 1.09174 -5.6789 0.752294 -27.602 -8.79592 -17.9358 2.63889 2.98165
0.00993 0.007 0.0585 0.0221 0.0364 0.0019 0.0148 0.00684 0.00707
3L:7243540-7243690:minus -40.2551 6.49541 -0.477064 4.05505 1.10204 9.79592 -19.9725 4.44444
1.95413 0.0382 0.000837 0.0135 0.00566 0.000124 0.0000183 0.0205 0.00334 0.00964
3L:7243540-7243690:plus -33.1837 -3.6422 -0.559633 1.83486 -9.23469 -18.051 -14.5413 0.861111
5.95413 0.0274 0.0307 0.0139 0.0155 0.00277 0.00886 0.00816 0.0127 0.00192
3L:724420-724570:minus -21.8571 0.752294 11.7982 8.05505 -17.4898 -7.93878 -23.5596 5.56944 2.74312
0.00249 0.00791 0.0000491 0.000578 0.0053 0.000736 0.0384 0.00203 0.00751
3L:724420-724570:plus -29.9592 0.266055 10.4037 5.94495 -17.0816 -16.051 -15.1193 5.91667 2.89908
0.00855 0.0094 0.000112 0.00261 0.00334 0.00408 0.00919 0.00172 0.00722
3L:7316520-7316670:minus -23.0408 0.357798 6.7156 2.06422 10.6531 -8.16327 -10.4679 1.30556 3.30275
0.00512 0.00911 0.000828 0.0139 0.0000102 0.000783 0.00336 0.011 0.00619
3L:7316520-7316670:plus -31.7755 -1.33028 1.6055 -1.61468 20.2041 -7.94898 -27.0917 2.65278 -4.12844
0.0157 0.0159 0.00667 0.0449 3.64e-07 0.00074 0.0612 0.0068 0.0573
3L:7318900-7319050:minus -23.5612 10.8532 8.87156 4.07339 -18.0816 -26.7551 -21.0917 -0.569444
-3.72477 0.00653 0.0000864 0.000273 0.00562 0.00759 0.0263 0.025 0.0199 0.0519
3L:7318900-7319050:plus -31.8163 4.7156 6.25688 5.69725 -17.5204 -18.2755 -16.7156 4.73611 -2.6789
0.016 0.00183 0.00103 0.00314 0.00532 0.0112 0.0122 0.00295 0.0408
3L:7321800-7321950:minus -4.93878 2.06422 -3.3211 4.23853 -17.1531 -0.173469 -15.2569 5.11111
5.81651 0.000213 0.0049 0.0315 0.00522 0.00364 0.000456 0.00944 0.0025 0.00204
3L:7321800-7321950:plus -40.6633 7.31193 -2.55963 -0.266055 -17.449 -10.0204 -18.6789 1.56944
-2.94495 0.0426 0.000551 0.0254 0.029 0.00484 0.00399 0.0166 0.01 0.0441
3L:7328260-7328410:minus -13.6735 10.2752 -1.78899 4.20183 -8.63265 -17.9796 -19.5413 1.88889 1.01835
0.00103 0.000117 0.0203 0.00533 0.0013 0.00764 0.0191 0.00896 0.0142
3L:7328260-7328410:plus -22.2959 10.844 -1.91743 4.20183 -17.6429 -7.79592 -11.4679 -3.05556 -1.27523
0.00335 0.0000872 0.0211 0.00533 0.00562 0.000723 0.00411 0.0392 0.0282
3L:7332480-7332630:minus -24.2245 4.21101 -5.25688 -0.256881 -19.3163 -10.0918 -16.2018 3.11111
-2.68807 0.00763 0.00222 0.0527 0.0289 0.0165 0.00404 0.0112 0.00572 0.041
3L:7332480-7332630:plus -33.0306 -5.93578 -3.3211 9.41284 1.91837 -9.5 -17.4771 4.76389 -1.47706 0.026
0.0562 0.0315 0.000354 0.0000382 0.00294 0.0138 0.00291 0.0292
3L:733300-733450:minus -12.3776 0.678899 -2.43119 2.94495 -28.1224 -27.051 -11 4.33333 8.19266
0.000403 0.00813 0.0245 0.0092 0.0457 0.0315 0.00373 0.0035 0.000548

3L:733300-733450:plus -23.2959 -2.90826 3.58716 7.37615 -28.1531 -17.051 -0.220183 -0.194444
7.6789 0.00605 0.0251 0.00316 0.000951 0.0459 0.00479 0.000298 0.0178 0.000866
3L:7338300-7338450:minus -3.22449 -0.917431 0.220183 10.1468 -18.0816 -9.30612 -10.3303 -3.19444
-1.90826 0.0000495 0.014 0.0108 0.000128 0.00759 0.00257 0.00328 0.0406 0.0324
3L:7338300-7338450:plus -23.2245 1.24771 3.42202 1.66972 -18.7857 -18.051 -10.7615 -0.638889
0.238532 0.00576 0.00661 0.00337 0.0165 0.0148 0.00886 0.00356 0.0203 0.0173
3L:7341300-7341450:minus -31.0612 13.5306 7.07339 7.27523 -18.1531 -18.0816 -20.211 4.41667 1.07339
0.0106 0.0000206 0.000703 0.00109 0.00829 0.00934 0.0214 0.00338 0.014
3L:7341300-7341450:plus -31.5204 3.64286 -0.0825688 5.48624 -18.1224 -9.42857 -14.156 -0.333333
1.02752 0.0131 0.00159 0.0119 0.00357 0.00782 0.0027 0.00751 0.0185 0.0141
3L:7342740-7342890:minus -14.2959 -2.0367 5.48624 2.58716 -17.9388 -8.94898 -16.9174 -1.11111 2.88073
0.00129 0.0196 0.00145 0.011 0.00715 0.00203 0.0126 0.0233 0.00727
3L:7342740-7342890:plus -31.3673 3.68367 2.9633 3.31193 -17.3367 -9.5 -15.6422 1.875 3.97248
0.0116 0.00144 0.00403 0.00786 0.00391 0.00294 0.0102 0.00901 0.00444
3L:7349760-7349910:minus -13.1837 13.4592 12.6514 3.89908 -18.4592 -8.23469 -12.4771 4.09722 -1.05505
0.000705 0.000064 0.0000271 0.00604 0.0109 0.000875 0.00513 0.00386 0.0264
3L:7349760-7349910:plus -31.0408 16.4954 2.83486 7.46789 -19.051 -7.72449 -27.2477 8.48611 0.183486
0.0106 7.12e-08 0.00423 0.000868 0.0162 0.00068 0.0624 0.00044 0.0176
3L:7350380-7350530:minus -41.4796 1.92661 4.44954 1.48624 -18.5204 -9.57143 -16.4037 6.84722 -0.908257
0.0577 0.00516 0.00224 0.0177 0.0117 0.00326 0.0116 0.00109 0.0255
3L:7350380-7350530:plus -23.1837 -0.366972 -2.31193 3.30275 -18.7143 -10.0918 -22.2202 0.611111
2.25688 0.00553 0.0117 0.0237 0.00789 0.0136 0.00404 0.0307 0.0138 0.00853
3L:7353180-7353330:minus -23.2959 -0.376147 8.22936 1 -18.449 -28.3061 -12.5596 5.22222 1.69725
0.00605 0.0117 0.000388 0.0206 0.0107 0.0597 0.00523 0.00238 0.0108
3L:7353180-7353330:plus -22.9592 -2.3578 3.48624 4.65138 -17.7143 -7.72449 -8.6055 2.70833 1.36697
0.00475 0.0215 0.00329 0.00471 0.00627 0.00068 0.00237 0.00666 0.0124
3L:7353540-7353690:minus -13.2143 -2.30275 5.63303 4.07339 -17.1939 -16.9388 -20.0642 -0.444444
2.94495 0.000714 0.0212 0.00136 0.00562 0.00377 0.00433 0.0208 0.0192 0.00714
3L:7353540-7353690:plus -22.1837 15.3303 -2.20183 9.75229 -18.3776 -17.7857 -3.51376 -8.15278 -0.697248
0.00315 1.39e-06 0.0229 0.000226 0.00926 0.0068 0.000652 0.114 0.0239
3L:7354200-7354350:minus -32.7755 -0.568807 0.0733945 1.57798 -8.96939 -16.2755 -23.1376 0.833333
4.09174 0.0234 0.0125 0.0113 0.0171 0.00226 0.00413 0.0359 0.0128 0.00416
3L:7354200-7354350:plus -22.8163 -0.284404 4.45872 3.41284 -26.5918 -27.2653 -0.0275229 -6.33333
6.02752 0.00418 0.0114 0.00223 0.00756 0.0186 0.0333 0.000285 0.0814 0.00183
3L:7361660-7361810:minus -22.8469 -0.568807 0.495413 7.27523 -8.93878 -8.16327 -7.77064 1.16667
-2.83486 0.00424 0.0125 0.00983 0.00109 0.00216 0.000783 0.00201 0.0115 0.0427
3L:7361660-7361810:plus -13.1429 -0.954128 1.65138 2.09174 -17.6429 -8.72449 -1.31193 0.902778
1.2844 0.00065 0.0141 0.00656 0.0137 0.00562 0.00169 0.00038 0.0125 0.0129
3L:7362320-7362470:minus -31.8469 2.18349 2.06422 6.12844 1.14286 -17.5612 -20.0826 -0.458333
-0.908257 0.0162 0.00469 0.00564 0.00231 0.000113 0.00604 0.0209 0.0192 0.0255
3L:7362320-7362470:plus -41.551 -1.87156 -0.449541 5.57798 -27.1939 0.530612 -19.8807 1.72222
-2.26606 0.0607 0.0187 0.0134 0.0034 0.0267 0.000203 0.0202 0.0095 0.0359
3L:7362840-7362990:minus -29.1735 1.45872 -1.09174 1.33028 -18.7551 -18.1939 -5.90826 2.76389 7.18349
0.00835 0.00613 0.0164 0.0188 0.0144 0.0101 0.00128 0.00652 0.00121
3L:7362840-7362990:plus -23.8878 -0.779817 -2.38532 4.40367 10.9898 -17.3469 -11.3761 -2.86111
7.84404 0.00692 0.0134 0.0242 0.00497 3.28e-06 0.0058 0.00403 0.0373 0.000701
3L:7363360-7363510:minus -30.4388 1.45872 8.95413 6.02752 -8.64286 -8.27551 -12.9266 6.48611 0.917431
0.00923 0.00613 0.000261 0.00246 0.00139 0.000919 0.00568 0.00131 0.0146
3L:7363360-7363510:plus -21.2245 2.16514 3.52294 3.6055 -18.3469 -15.9796 -20.2018 -1.75 3.7156
0.0019 0.00473 0.00324 0.00686 0.00894 0.00405 0.0213 0.0279 0.00502
3L:7374620-7374770:minus -31.8163 -0.366972 9.41284 3.40367 -18.1531 -17.7857 -14.5321 1.81944
0.440367 0.016 0.0117 0.000199 0.00759 0.00829 0.0068 0.00814 0.00919 0.0164
3L:7374620-7374770:plus -13.4388 13.4128 1.18349 6.78899 0.877551 -8.45918 -14.156 -1.44444 -2.30275
0.000879 0.0000152 0.00775 0.00146 0.00019 0.00111 0.00751 0.0256 0.0363

3L:7426780-7426930:minus -22.8163 -1.91743 -1.36697 1.88991 -17.2653 -25.898 -17.5963 5.66667 6.54128
0.00418 0.019 0.0179 0.0151 0.00387 0.0214 0.0141 0.00194 0.00151
3L:7426780-7426930:plus -31.4082 -2.84404 0.559633 1.53211 -16.1531 -8.65306 -8.79817 0.25 3.78899
0.0119 0.0247 0.00961 0.0174 0.00297 0.00156 0.00246 0.0155 0.00487
3L:7427980-7428130:minus -31.5816 -0.477064 2.84404 2.08257 -17.8265 -27.9694 -10.8899 7 3.42202
0.0135 0.0121 0.00421 0.0137 0.00691 0.0547 0.00365 0.00101 0.00597
3L:7427980-7428130:plus -21.8571 4.58716 3.24771 8.05505 -18.0816 10.0204 -12.7431 -0.680556
3.20183 0.00249 0.00192 0.00361 0.000578 0.00759 0.0000106 0.00545 0.0206 0.0065
3L:7428360-7428510:minus -13.2245 -0.733945 8.26606 7.70642 -26.9694 -9.30612 -11.422 0.263889
0.981651 0.000738 0.0132 0.00038 0.000708 0.023 0.00257 0.00407 0.0154 0.0144
3L:7428360-7428510:plus -22.8061 1.9633 0.623853 4.37615 -27.1633 0.122449 -14.4587 -2.25 9.34862
0.00414 0.00509 0.00941 0.00504 0.025 0.000312 0.00802 0.0319 0.000371
3L:749200-749350:minus -21.8571 -0.440367 -2.48624 5.43119 -17.9388 -18.9796 -17.6789 3.30556
0.238532 0.00249 0.012 0.0249 0.00371 0.00715 0.0155 0.0142 0.0053 0.0173
3L:749200-749350:plus -22.0714 5.99083 -0.807339 7.17431 -8.04082 -27.2653 -14.2752 15.625 7.62385
0.00299 0.00106 0.015 0.00115 0.000831 0.0333 0.00771 1.02e-07 0.000941
3L:7503660-7503810:minus -30.8469 0.266055 0.669725 1.43119 -27.1939 -8.72449 -6.69725 1.76389 -2.18349
0.0103 0.0094 0.00926 0.0181 0.0267 0.00169 0.00157 0.00937 0.0351
3L:7503660-7503810:plus -41.8878 -2.69725 3.69725 2.93578 -19.3163 -0.540816 -15.4037 1.34722
-2.20183 0.0672 0.0237 0.00303 0.00927 0.0165 0.00054 0.00972 0.0108 0.0353
3L:7504120-7504270:minus -32.0102 4.14679 -2.11927 4.22018 -17.6837 -18.051 -27.1193 4.38889 -4.17431
0.0172 0.00228 0.0224 0.00531 0.0058 0.00886 0.0614 0.00342 0.058
3L:7504120-7504270:plus -14.0408 -3.84404 9.0367 3.23853 1.14286 -9.53061 -15.7339 -2.68056 -3.55046
0.00115 0.0324 0.000249 0.00815 0.000113 0.0031 0.0103 0.0356 0.0503
3L:7521720-7521870:minus -41.8163 2.01835 4.53211 0.284404 -18.7551 -9.02041 -28.5963 6.15278 -3.29358
0.0662 0.00499 0.00216 0.0249 0.0144 0.00213 0.0746 0.00154 0.0474
3L:7521720-7521870:plus -23.1429 13.3878 1.33028 5.86239 -26.898 -8.53061 -14.0275 0.0416667
5.3945 0.00533 0.0000869 0.00736 0.00282 0.0203 0.00136 0.0073 0.0165 0.00263
3L:7632780-7632930:minus -32.1939 7.06422 -5.23853 1.82569 -27.1939 -7.79592 -23.0275 1.90278 -6.6789
0.0181 0.000628 0.0524 0.0156 0.0267 0.000723 0.0352 0.00892 0.105
3L:7632780-7632930:plus -22.9286 4.29358 1.40367 -1.48624 -26.5306 -27.1224 -9.25688 9.22222 3.05505
0.0046 0.00215 0.00717 0.0431 0.0185 0.0325 0.00268 0.000278 0.00692
3L:7633520-7633670:minus -40.9898 0.706422 5.89908 2.78899 2.14286 -0.244898 -18.7523 1.72222
-1.84404 0.0455 0.00805 0.00121 0.00992 0.0000316 0.000496 0.0168 0.0095 0.0319
3L:7633520-7633670:plus -50.0306 -0.238532 1.31193 2.19266 -28.3061 -18.5714 -23.7431 0.625
-4.68807 0.119 0.0112 0.00741 0.0131 0.0553 0.0136 0.0395 0.0137 0.0672
3L:7634720-7634870:minus -23.1837 -2.87156 15.6606 0.559633 2.10204 -8.45918 -16.4771 3.30556 -2.52294
0.00553 0.0249 3.94e-07 0.0232 0.0000336 0.00111 0.0118 0.0053 0.0391
3L:7634720-7634870:plus -33.1122 3.83673 0.678899 -0.137615 -8.71429 -19.3776 -17.3211 0.125
1.74312 0.0271 0.00115 0.00923 0.0279 0.00159 0.0195 0.0135 0.0161 0.0105
3L:7644260-7644410:minus -41.2959 4.2844 -0.0458716 1.06422 -26.2347 -17.051 -21.1101 8.38889
6.04587 0.0529 0.00216 0.0118 0.0203 0.0177 0.00479 0.0251 0.000466 0.00181
3L:7644260-7644410:plus -13.3673 -0.66055 8.24771 10.5872 -18.1837 -26.2653 -7.80734 3.68056 0.183486
0.000813 0.0129 0.000384 0.000115 0.00835 0.0218 0.00202 0.00457 0.0176
3L:7645180-7645330:minus -30.2551 -0.412844 1.70642 1.86239 -26.9694 -26.7857 -13.7706 5.43056
8.95413 0.00885 0.0119 0.00644 0.0153 0.023 0.0266 0.00689 0.00216 0.000458
3L:7645180-7645330:plus -31.7653 -0.981651 1.88991 3.21101 -27.4592 -27.9694 -6.63303 1.69444
3.31193 0.0152 0.0143 0.00602 0.00822 0.0329 0.0547 0.00154 0.0096 0.00618
3L:7647740-7647890:minus -22.5918 0.862385 -1.61468 5.30275 20.4694 -9.45918 -21.1927 -0.638889
5.81651 0.00365 0.00761 0.0193 0.00396 1.65e-07 0.00279 0.0255 0.0203 0.00204
3L:7647740-7647890:plus -30.5102 0.357798 -0.431193 2.81651 -27.2347 -27.3367 -14.156 5.40278
0.0275229 0.00947 0.00911 0.0133 0.00982 0.0283 0.0347 0.00751 0.00219 0.0183
3L:767860-768010:minus -22.9184 -0.449541 1.21101 9.33028 -18.6837 -9.79592 -10.2936 -0.444444
7.84404 0.00447 0.012 0.00768 0.000374 0.0126 0.00377 0.00325 0.0192 0.000701

3L:767860-768010:plus -31.398 2.55963 6.88073 10.8624 -17.6429 -6.79592 -28.0367 0.819444 -6.26606
0.0117 0.00409 0.000768 0.0000687 0.00562 0.000546 0.0691 0.0129 0.0948
3L:769080-769230:minus -33.6224 1.85321 -2.90826 3.05505 -17.9796 -8.79592 -3.0367 3.625 5.65138
0.0289 0.0053 0.0281 0.00878 0.00726 0.0019 0.000573 0.00468 0.00228
3L:769080-769230:plus -31.7041 -1.31193 1.88991 6.11009 -27.4898 -17.2755 -15.5046 3.26389 11.3853
0.0146 0.0158 0.00602 0.00233 0.0336 0.00544 0.0099 0.00539 0.0000815
3L:769320-769470:minus -32.5102 -2.3211 2.77982 -1.18349 -26.4898 -27.1224 -18.211 5.68056 6.17431
0.0202 0.0213 0.00432 0.0389 0.0183 0.0325 0.0154 0.00193 0.00171
3L:769320-769470:plus -20.6633 -0.688073 3.14679 7.34862 -27.2653 -8.53061 -17.578 0.305556 -2.45872
0.00173 0.013 0.00375 0.000998 0.0302 0.00136 0.014 0.0152 0.0383
3L:7712280-7712430:minus -32.4388 -2.62385 -2.05505 7.63303 -18.3469 -27.5306 -11.2844 -5.54167 1.37615
0.0195 0.0232 0.022 0.000745 0.00894 0.0374 0.00396 0.0692 0.0123
3L:7712280-7712430:plus -4.59184 6.05505 -1.00917 5.43119 -26.5306 -9.5 -8.17431 6.06944 0.40367
0.000166 0.00103 0.016 0.00371 0.0185 0.00294 0.00218 0.0016 0.0165
3L:7719500-7719650:minus -32.0408 7.77064 -1.97248 1.78899 -18.449 -8.5 -0.926606 1.45833 -1.27523
0.0174 0.000432 0.0214 0.0158 0.0107 0.00126 0.000349 0.0104 0.0282
3L:7719500-7719650:plus -4.63265 13.4592 -0.477064 1.99083 -27.7551 -18.4184 -14.1193 -2.27778
3.07339 0.000175 0.000064 0.0135 0.0144 0.0385 0.0127 0.00745 0.0321 0.00683
3L:774760-774910:minus -22.4388 -0.201835 7.01835 3.31193 -27.1939 -19.5714 -1.43119 5.97222
-0.146789 0.00348 0.011 0.000721 0.00786 0.0267 0.0202 0.00039 0.00168 0.0194
3L:774760-774910:plus -22.1531 1.40367 7.38532 7.99083 -17.449 -8.65306 -11.1193 0.416667 3.07339
0.0031 0.00625 0.0006 0.00059 0.00484 0.00156 0.00382 0.0147 0.00683
3L:775320-775470:minus -32.6327 1.11009 4.72477 4.08257 -18.1224 -28.6327 -23.3303 5.33333 3.24771
0.0212 0.00695 0.00199 0.00558 0.00782 0.066 0.037 0.00226 0.00636
3L:775320-775470:plus -32.8061 5.40367 6.54128 6.21101 -26.9286 -18.0102 -4.65138 3.68056 1.74312
0.0237 0.00138 0.000901 0.00213 0.0214 0.00812 0.000899 0.00457 0.0105
3L:776720-776870:minus -23.1531 4.21101 8.38532 3.91743 -26.2347 -27.602 -5.14679 -2.58333 -2.33945
0.00542 0.00222 0.000356 0.00601 0.0177 0.0416 0.00104 0.0348 0.0367
3L:776720-776870:plus -34.0408 -1.42202 3.6422 6.69725 -27.0102 -17.2755 -15.1927 -2.51389 3.08257
0.0309 0.0164 0.00309 0.00156 0.0237 0.00544 0.00932 0.0341 0.00681
3L:7774920-7775070:minus -4.15306 -4.94495 -0.495413 5.91743 -8.97959 -18.7551 -11.6422 2.63889
6.25688 0.000127 0.0435 0.0136 0.00267 0.0023 0.0141 0.00427 0.00684 0.00163
3L:7774920-7775070:plus -12.6633 -0.256881 9.11927 4.07339 -27.8265 -0.469388 -10.9174 10.1806
2.73394 0.000458 0.0113 0.000237 0.00562 0.0388 0.000533 0.00367 0.000144 0.00756
3L:7794640-7794790:minus -23.7755 5.93578 7.81651 3.69725 -18.0816 -19.1531 -19.8624 2.51389 10.3394
0.00678 0.00109 0.000481 0.00665 0.00759 0.0177 0.0201 0.00716 0.000112
3L:7794640-7794790:plus -31.8163 5.90826 7.18349 4.15596 -18.449 -28.1939 -19.4587 3.90278 6.95413
0.016 0.0011 0.000665 0.00539 0.0107 0.0591 0.0188 0.00418 0.00136
3L:7797920-7798070:minus -32.8878 -0.889908 6.33945 9.24771 -26.898 -0.397959 -17 6.59722
1.90826 0.0247 0.0138 0.000991 0.000409 0.0203 0.000525 0.0128 0.00124 0.00974
3L:7797920-7798070:plus -41.6224 3.7551 0.0917431 -0.201835 -26.6735 -8.53061 -13.5872
-0.0694444 7.73394 0.0628 0.00122 0.0112 0.0285 0.0191 0.00136 0.00661 0.0171 0.000784
3L:7798500-7798650:minus -22.7755 2.3945 -0.798165 9.44037 -17.3776 -9.45918 -15.9083 9.69444
-2.17431 0.00412 0.00434 0.015 0.000339 0.00419 0.00279 0.0107 0.000203 0.035
3L:7798500-7798650:plus -21.4388 1.38532 -0.477064 0.926606 -8.67347 -19.2755 -10.7339 3.08333
-2.55046 0.002 0.00629 0.0135 0.0209 0.00154 0.0185 0.00354 0.00578 0.0394
3L:7802040-7802190:minus -24.2551 -3.47706 1.7156 4.65138 -17.5306 -0.0204082 -7.94495 3.08333
6.33028 0.00768 0.0294 0.00642 0.00471 0.0054 0.000411 0.00208 0.00578 0.0016
3L:7802040-7802190:plus -3.89796 -0.706422 0.146789 -0.550459 -8.89796 -27.5306 -13.1376 1.94444
11.3853 0.000113 0.0131 0.011 0.0317 0.00189 0.0374 0.00597 0.00879 0.0000815
3L:7802980-7803130:minus -22.0306 0.733945 7.00917 7 -17.4898 -26.8265 -9.97248 -1.56944 9 0.00293
0.00797 0.000725 0.00132 0.0053 0.0285 0.00306 0.0265 0.000437
3L:7802980-7803130:plus -32.7041 5.73394 2.74312 4.78899 -17.1531 -18.7857 -13.4037 8.58333 -0.183486
0.0222 0.00119 0.00438 0.00456 0.00364 0.0146 0.00634 0.000415 0.0197

3L:7808920-7809070:minus -32.4388 -1.36697 1.41284 4.6055 -18.1531 -7.72449 4.74312 3.25 5.12844
0.0195 0.0161 0.00714 0.00476 0.00829 0.00068 0.0000631 0.00542 0.00301
3L:7808920-7809070:plus -11.9286 -1.6789 -3.51376 -2.66972 -27.7551 -16.7551 -6.50459 9.47222 4.80734
0.000296 0.0177 0.0333 0.0635 0.0385 0.0042 0.00149 0.000236 0.00334
3L:7819980-7820130:minus -31.1429 -2.68807 2.78899 6.47706 -26 -19.051 -8.22936 -0.513889 -2.92661
0.0108 0.0236 0.0043 0.00179 0.0171 0.0165 0.0022 0.0196 0.0437
3L:7819980-7820130:plus -31.6633 5.68807 1.87156 1.91743 -28.1224 10.3776 -26.633 0.75 -5.87156
0.0141 0.00122 0.00606 0.015 0.0457 6.32e-06 0.0578 0.0132 0.0865
3L:7821360-7821510:minus -21.1531 3.13761 0.119266 6.3578 -26.9694 -19.0102 -10.844 2.30556 11.8532
0.00188 0.00331 0.0111 0.00195 0.023 0.0159 0.00362 0.00772 0.0000428
3L:7821360-7821510:plus -31.551 -2.14679 -0.174312 4.22018 -18.051 0.826531 -19.7064 -0.291667
3.88991 0.0134 0.0203 0.0123 0.00531 0.00746 0.000153 0.0196 0.0183 0.00467
3L:784760-784910:minus -23.4082 -0.0275229 1.77064 0.788991 -18.1531 -28.7041 -11.7339 -2.20833
2.79817 0.00623 0.0104 0.00629 0.0218 0.00829 0.067 0.00435 0.0315 0.00743
3L:784760-784910:plus -23.1837 -2.37615 1.16514 2.88073 -27.2653 -0.244898 -25.5046 4.01389 1.34862
0.00553 0.0216 0.0078 0.00945 0.0302 0.000496 0.0503 0.004 0.0125
3L:7847960-7848110:minus -12.8163 -2.92661 3.79817 3.76147 -17.4082 -7.72449 0.605505 2.26389 1.44037
0.000489 0.0253 0.00291 0.00645 0.00421 0.00068 0.000244 0.00784 0.012
3L:7847960-7848110:plus -22.4796 2.29358 -2.23853 -2.11009 -26.9694 -27.8265 -10.4771 3.31944 7.63303
0.00352 0.00451 0.0232 0.053 0.023 0.0498 0.00337 0.00528 0.000928
3L:7848640-7848790:minus -4.47959 -2.22936 4.73394 5.37615 -7.96939 -18.3469 1.89908 5.94444 7.36697
0.000154 0.0208 0.00199 0.00384 0.000785 0.0124 0.00017 0.0017 0.00108
3L:7848640-7848790:plus -24.7449 -1.13761 -2.81651 2.25688 -26.8571 0.602041 -10.3945 5.83333 1.25688
0.00827 0.015 0.0274 0.0127 0.0198 0.000188 0.00332 0.00179 0.0131
3L:7849460-7849610:minus -23.2959 -0.880734 2.63303 1.45872 -18.6837 0.316327 -14.0734 1.75 3.22018
0.00605 0.0138 0.00457 0.0179 0.0126 0.000243 0.00737 0.00941 0.0064
3L:7849460-7849610:plus -23.0408 0.816514 -3.11009 7.62385 -26.8163 1.05102 -22.1284 -0.875 -4.33028
0.00512 0.00773 0.0297 0.000759 0.0195 0.000107 0.0302 0.0218 0.0607
3L:7851880-7852030:minus -22.9592 -5.30275 3.00917 5.15596 -17.7143 -18.3469 -13.0826 -0.0972222
1.74312 0.00475 0.0478 0.00396 0.00426 0.00627 0.0124 0.00589 0.0172 0.0105
3L:7851880-7852030:plus -31.7041 3.10092 0.642202 -0.862385 -19.051 -17.3469 -15.2569 4.375
1.83486 0.0146 0.00335 0.00935 0.0351 0.0162 0.0058 0.00944 0.00344 0.0101
3L:7856660-7856810:minus -31.4694 4.69725 -1.47706 5.74312 -28 -8.30612 -7.88073 7.15278 1.53211
0.0124 0.00184 0.0185 0.00302 0.0444 0.000973 0.00205 0.000928 0.0116
3L:7856660-7856810:plus -33.1939 -1.31193 -1.48624 0.844037 -27.2347 -8.20408 -24.2661 4.33333 -4.52294
0.0274 0.0158 0.0185 0.0215 0.0283 0.000796 0.0427 0.0035 0.0642
3L:7857220-7857370:minus -31.0714 -1.82569 -0.275229 3.18349 -28.0102 -27.6735 -18.5229 4.125
3.93578 0.0106 0.0185 0.0127 0.00837 0.0449 0.0443 0.0162 0.00382 0.00456
3L:7857220-7857370:plus -22.7041 4.22018 4.24771 2.22018 -27.7959 -17.5714 -20.3303 2.80556 4.45872
0.00395 0.00221 0.00243 0.0129 0.0386 0.00616 0.0218 0.00642 0.00366
3L:7858060-7858210:minus -12.6735 7.6789 -1.63303 7.70642 -26.8571 -18.7143 -6.47706 -0.472222
5.23853 0.000461 0.000454 0.0194 0.000708 0.0198 0.014 0.00148 0.0193 0.00285
3L:7858060-7858210:plus -23.1531 -1.68807 1.08257 5.33028 -27.2653 -7.72449 -17.7982 1.47222 -1.43119
0.00542 0.0177 0.00803 0.00389 0.0302 0.00068 0.0145 0.0104 0.029
3L:7858340-7858490:minus -20.6224 -3.05505 1.76147 2.01835 -8.56122 -18.2755 3.19266 0.666667 0.238532
0.0017 0.0262 0.00631 0.0141 0.00112 0.0112 0.00011 0.0135 0.0173
3L:7858340-7858490:plus -32.7347 4.05102 2.45872 3.3211 -18.1531 -26.5612 -18.5138 4.30556 5.75229
0.0225 0.000335 0.00488 0.00782 0.00829 0.0237 0.0162 0.00354 0.00213
3L:7859160-7859310:minus -21.5918 4.50459 9.16514 7.00917 -17.9388 -18.7857 -16.0826 -2.05556 2.94495
0.00209 0.00198 0.000231 0.00126 0.00715 0.0146 0.011 0.0303 0.00714
3L:7859160-7859310:plus -32.1429 1.9633 5.99083 1.43119 -18.1837 -18.0102 -21.6789 0.638889 -1.44954
0.0179 0.00509 0.00116 0.0181 0.00835 0.00812 0.0278 0.0137 0.0291
3L:786020-786170:minus -31.4796 -1.57798 0.504587 3.18349 -18.4184 -26.5612 -11.844 3.19444 3.88073
0.0128 0.0171 0.0098 0.00837 0.0101 0.0237 0.00446 0.00554 0.00475

3L:786020-786170:plus -21.7041 -4.87156 8.95413 4.17431 -8.64286 -9.20408 -20.1376 -3.41667 -1.76147
0.00231 0.0427 0.000261 0.00537 0.00139 0.00225 0.0211 0.0428 0.0313
3L:7860580-7860730:minus -31.1735 6.83486 -1.90826 2.14679 -17.4184 -9.42857 -18.2018 2.16667 -2.18349
0.0109 0.000707 0.021 0.0133 0.00457 0.0027 0.0154 0.00812 0.0351
3L:7860580-7860730:plus -20.9694 0.385321 -0.954128 1.47706 -17.449 -17.0102 -7.34862 0.513889
-0.550459 0.00184 0.00902 0.0157 0.0178 0.00484 0.00453 0.00183 0.0142 0.0225
3L:7904200-7904350:minus -3.92857 1.41284 3.56881 9.44954 -18.6837 -9.45918 -12.0183 3.29167 5.01835
0.000116 0.00623 0.00318 0.000326 0.0126 0.00279 0.00463 0.00533 0.00309
3L:7904200-7904350:plus -30.4082 0.834862 -0.87156 5.57798 -27.2347 -0.204082 -11.9083 0.736111
1.02752 0.00908 0.00769 0.0153 0.0034 0.0283 0.000463 0.00452 0.0132 0.0141
3L:7904980-7905130:minus -23.7041 1.33028 0.944954 4.22018 10.6531 -17.051 -12.6697 0.958333 1.21101
0.00669 0.00642 0.00842 0.00531 0.0000102 0.00479 0.00536 0.0123 0.0134
3L:7904980-7905130:plus -21.9694 1.45872 -1.95413 7.44037 11.4286 -27.1939 -24.0092 6.18056 -1.92661
0.00282 0.00613 0.0213 0.000912 1.97e-06 0.0327 0.0411 0.00152 0.0325
3L:7905480-7905630:minus -13.9592 13.4592 3.69725 6.00917 -18.1939 -8.53061 -12.7706 4.81944 2.00917
0.00113 0.000064 0.00303 0.00255 0.0086 0.00136 0.00549 0.00284 0.00934
3L:7905480-7905630:plus -30.6633 -0.33945 1.10092 4.0367 -8.60204 -26.8265 -13.1835 -2.13889 1.22018
0.00976 0.0116 0.00798 0.00578 0.00125 0.0285 0.00603 0.0309 0.0133
3L:7908240-7908510:minus -14.4082 0.183486 14.422 7.29358 20.4694 -18.5612 -13.1193 6.41667 -2.73394
0.00136 0.00968 4.18e-06 0.00104 1.65e-07 0.0132 0.00594 0.00135 0.0416
3L:7908240-7908510:plus -24.1122 -1.77982 -3.38532 9.51376 -18.1531 -19.0816 12.8165 -0.708333
0.908257 0.00738 0.0182 0.0321 0.000292 0.00829 0.017 3.79e-06 0.0207 0.0147
3L:7909060-7909210:minus -32.0714 9.55963 6.89908 4.46789 -26.8878 -28.1939 -11.3761 -1.02778 -0.229358
0.0176 0.000167 0.000762 0.0049 0.0199 0.0591 0.00403 0.0227 0.0202
3L:7909060-7909210:plus -24.1122 -1.61468 -0.449541 1.44037 -17.1531 -17.5612 -21.367 4.47222
3.83486 0.00738 0.0173 0.0134 0.018 0.00364 0.00604 0.0263 0.0033 0.00481
3L:7928240-7928390:minus -30.4388 0.385321 -1.42202 2.84404 -18.6837 -16.3469 -13.2202 0.625 -3.75229
0.00923 0.00902 0.0182 0.00961 0.0126 0.00416 0.00608 0.0137 0.0521
3L:7928240-7928390:plus -31.551 -1.70642 -2.49541 2.66972 -8.64286 -16.7857 -17.578 6.97222 6.76147
0.0134 0.0178 0.025 0.0106 0.00139 0.00426 0.014 0.00102 0.00141
3L:7929300-7929450:minus -12.5204 3.29358 0.477064 4.11009 -7.63265 1.31633 -7.34862 2.18056 -4.36697
0.000441 0.00312 0.00989 0.00553 0.000479 0.000066 0.00183 0.00808 0.0615
3L:7929300-7929450:plus -20.8878 9.24771 3.6789 7 -17.1531 19.1531 -17.1835 -1.16667 -4.45872
0.0018 0.000197 0.00305 0.00132 0.00364 7.95e-07 0.0132 0.0237 0.0633
3L:7929800-7929950:minus -23.5204 -1.88073 -3.02752 5.30275 -18.7551 -8.16327 -13.2752 -1.31944 4.04587
0.00645 0.0188 0.0291 0.00396 0.0144 0.000783 0.00616 0.0247 0.00421
3L:7929800-7929950:plus -24.3673 5.87156 1.30275 4.34862 -17.2653 -27.7041 -7.41284 3.84722 4.80734
0.00791 0.00112 0.00743 0.00507 0.00387 0.0445 0.00186 0.00428 0.00334
3L:7963660-7963810:minus -32.4082 5.48624 3.80734 0.816514 -17.7857 -7.79592 -13.156 1.22222 -0.440367
0.0191 0.00133 0.0029 0.0216 0.00676 0.000723 0.00599 0.0113 0.0216
3L:7963660-7963810:plus -22.0306 13.4592 3.22936 5.59633 -18.4898 -18.0816 -6.18349 7.34722 -0.550459
0.00293 0.000064 0.00363 0.00331 0.0116 0.00934 0.00138 0.000838 0.0225
3L:7968160-7968310:minus -32.5918 13.4592 12.9266 7.2844 -27.2653 -26.8571 -19.0275 8.22222
-0.0183486 0.021 0.000064 0.0000219 0.00105 0.0302 0.0288 0.0175 0.000514 0.0185
3L:7968160-7968310:plus -33.1531 3.97959 -1.80734 0 -18.7551 -0.540816 -36.9541 4.55556 3.0367
0.0273 0.000669 0.0204 0.0269 0.0144 0.00054 0.195 0.00318 0.00694
3L:7969320-7969470:minus -23.3061 5.88991 0.0917431 -0.53211 -28.1939 -9.5 -25.4862 -1 3.9633
0.00606 0.00111 0.0112 0.0314 0.0482 0.00294 0.0502 0.0226 0.00446
3L:7969320-7969470:plus -30.4388 16.4954 -2.3578 1.78899 -17.4184 -8.72449 -12.7339 9.08333 0.513761
0.00923 7.12e-08 0.024 0.0158 0.00457 0.00169 0.00544 0.000304 0.0162
3L:7971940-7972090:minus -23.449 14.6055 -0.0917431 2.3945 -27.9694 -8.53061 -11.6972 5.01389
3.93578 0.00631 4.26e-06 0.0119 0.0119 0.0437 0.00136 0.00432 0.00261 0.00456
3L:7971940-7972090:plus -14.0306 6.66055 6.58716 2.6055 -18.1837 -25.8265 23.211 -1.94444 7.15596
0.00115 0.000771 0.00088 0.0109 0.00835 0.0212 1.16e-08 0.0294 0.00124

3L:7972360-7972510:minus -33.6327 -5.3578 8.37615 1.21101 -9.23469 -18.0102 -18.8991 3.02778 1.74312
0.029 0.0484 0.000358 0.0194 0.00277 0.00812 0.0172 0.0059 0.0105
3L:7972360-7972510:plus -31.3673 -4.31193 -5.52294 2.01835 -8.63265 -18.2347 -17.0734 4.19444 3.26606
0.0116 0.0368 0.0563 0.0141 0.0013 0.0104 0.013 0.00371 0.00627
3L:799180-799330:minus -14.4388 -0.266055 11.1284 7.55963 -8.86735 -18.0816 -14.3945 1.54167
1.27523 0.00137 0.0113 0.0000734 0.000807 0.00173 0.00934 0.00791 0.0101 0.013
3L:799180-799330:plus -32.8163 -1.51376 -1.51376 4.07339 -27.5306 -8.79592 -6.88991 5.93056 -2.24771 0.024
0.0168 0.0187 0.00562 0.0351 0.0019 0.00164 0.00171 0.0357
3L:799700-799850:minus -3.59184 4.48624 4.3211 3.05505 -17.9286 -9.27551 1.63303 -1.19444 3.56881
0.0000835 0.00199 0.00235 0.00878 0.0071 0.00244 0.000184 0.0239 0.00543
3L:799700-799850:plus -30.4388 1 3.3578 -1.02752 -26.6633 -27.602 -19.5872 8.90278 1.57798 0.00923
0.00724 0.00346 0.037 0.0189 0.0416 0.0192 0.000341 0.0114
3L:8056920-8057070:minus -33.9592 1.3211 -3.22936 7.73394 -18.9796 -9.79592 -21.4679 4.29167 -0.59633
0.0304 0.00644 0.0307 0.000669 0.0155 0.00377 0.0268 0.00356 0.0229
3L:8056920-8057070:plus -22.551 -3.05505 1.6055 6.12844 -17.7143 -18.3469 -17.3028 -1.94444 1.56881
0.00359 0.0262 0.00667 0.00231 0.00627 0.0124 0.0134 0.0294 0.0114
3L:8057300-8057450:minus -23.1837 1.86239 0.275229 3.48624 -27.4898 -17.051 -16.7064 2.90278 -6.73394
0.00553 0.00528 0.0106 0.0072 0.0336 0.00479 0.0122 0.00619 0.107
3L:8057300-8057450:plus -23.6735 4.24771 1.87156 1.72477 -18.5612 -27.0408 -13.211 10.2222 5.6055
0.00665 0.00219 0.00606 0.0161 0.0119 0.0307 0.00607 0.000139 0.00232
3L:8070380-8070530:minus -23.3367 -1.86239 4.49541 9.51376 -18.4898 -26.8265 -15.8532 1.41667 7.68807
0.00614 0.0187 0.00219 0.000292 0.0116 0.0285 0.0106 0.0106 0.000841
3L:8070380-8070530:plus -22.5918 3.00917 -4.13761 9.92661 -8.96939 -18.0102 -10.4679 2.56944 7.15596
0.00365 0.00347 0.0394 0.000169 0.00226 0.00812 0.00336 0.00701 0.00124
3L:8075020-8075170:minus -23.0408 4.87156 6.27523 2.33945 -18.7143 -8.65306 -13.7706 3.5 5.12844
0.00512 0.00172 0.00102 0.0122 0.0136 0.00156 0.00689 0.00491 0.00301
3L:8075020-8075170:plus -22.3265 -5.88073 8.05505 10.7615 -18.1939 -8.65306 -19.4037 -2.41667 -0.899083
0.00337 0.0554 0.000425 0.0000826 0.0086 0.00156 0.0186 0.0333 0.0254
3L:8084920-8085070:minus -31.7755 -1.49541 8.68807 7.29358 -18.7143 0.826531 -17.3853 2.11111 -2.19266
0.0157 0.0167 0.0003 0.00104 0.0136 0.000153 0.0136 0.00828 0.0352
3L:8084920-8085070:plus -23.2245 2.66055 0.743119 0.146789 -26.898 -8.27551 -15.0092 -1.61111 4.9633
0.00576 0.00394 0.00903 0.0258 0.0203 0.000919 0.00899 0.0268 0.00316
3L:8085960-8086110:minus -31.6939 -2.05505 5.77064 2.55963 -27.2653 -19.0816 -17.2477 2.52778 -3.33945
0.0142 0.0197 0.00128 0.0111 0.0302 0.017 0.0133 0.00712 0.0479
3L:8085960-8086110:plus -22.3673 -1.33028 8.33028 5.3945 -17.602 -8.38776 -21.1101 -0.430556
0.275229 0.00341 0.0159 0.000367 0.00379 0.00547 0.00101 0.0251 0.0191 0.0171
3L:8087460-8087610:minus -22.9184 -0.743119 3.65138 4.9633 -26.8571 -0.316327 -20.1284 4.625
6.95413 0.00447 0.0132 0.00308 0.0044 0.0198 0.000524 0.0211 0.00309 0.00136
3L:8087460-8087610:plus -22.9286 -1.87156 5.37615 -0.376147 -18.7449 -18.7857 -13.5963 0.847222
1.79817 0.0046 0.0187 0.00152 0.0299 0.0138 0.0146 0.00663 0.0128 0.0102
3L:8088100-8088250:minus -40.5918 -2.9633 0.678899 9.41284 -17.449 -18.6327 -20.8165 0.5 0.651376
0.0422 0.0255 0.00923 0.000354 0.00484 0.0137 0.0238 0.0143 0.0156
3L:8088100-8088250:plus -22.2245 1.65138 -0.504587 0.990826 -17.6837 -16.9796 -21.4404 -2.55556
0.110092 0.00325 0.00571 0.0137 0.0206 0.0058 0.0044 0.0266 0.0345 0.018
3L:8089080-8089230:minus -4.4898 2.06422 1.88073 2.88991 -27.1939 -18.3469 -18.8807 3.61111 -2.21101
0.000154 0.0049 0.00604 0.00942 0.0267 0.0124 0.0171 0.0047 0.0354
3L:8089080-8089230:plus -23.5204 -4.86239 8.75229 10.7615 -26.898 -18.5714 -17.9725 0.569444 -2.68807
0.00645 0.0426 0.000289 0.0000826 0.0203 0.0136 0.0149 0.014 0.041
3L:8089540-8089690:minus -32.2653 1.83486 5.40367 3.77064 -19.051 -26.5306 -25.6881 8.09722 3.52294
0.0184 0.00533 0.0015 0.00637 0.0162 0.023 0.0515 0.000552 0.00558
3L:8089540-8089690:plus -22.7653 1.15596 2.92661 5.47706 -26.9286 -10.0204 1.0367 2.125 1.45872
0.00405 0.00684 0.00408 0.0036 0.0214 0.00399 0.000218 0.00824 0.0119
3L:8103340-8103490:minus -41.4388 1.74312 -1.16514 1.92661 -36.3061 -8.94898 -28.4954 0.819444 3
0.0562 0.00552 0.0168 0.0148 0.0749 0.00203 0.0736 0.0129 0.00702

3L:8103340-8103490:plus -22.5102 -0.348624 6.75229 4.6055 -8.37755 -28.3367 -9.82569 -1.81944
7.37615 0.00354 0.0116 0.000815 0.00476 0.00106 0.0606 0.00298 0.0284 0.00107
3L:8103640-8103790:minus -41.4082 -2.70642 1.88991 4.31193 -17.0816 -18.2347 -24.9908 3.36111 -3.04587
0.0557 0.0238 0.00602 0.00516 0.00334 0.0104 0.0471 0.00519 0.0451
3L:8103640-8103790:plus -23.4082 -4.6055 1.23853 5.82569 -8.63265 -26.5306 -18.156 0.388889 5.38532
0.00623 0.0398 0.0076 0.00291 0.0013 0.023 0.0153 0.0148 0.00267
3L:8109020-8109270:minus -23.2551 13.4592 10.6972 3.89908 -18.449 -26.3061 -20.0642 -1.36111 7.6422
0.00583 0.000064 0.0000948 0.00604 0.0107 0.0219 0.0208 0.025 0.000893
3L:8109020-8109270:plus -31.3673 -2.54128 0.201835 4.0367 -18.449 -18.4286 -18.6239 1.75 -3.15596
0.0116 0.0227 0.0108 0.00578 0.0107 0.0128 0.0165 0.00941 0.0464
3L:8109560-8109710:minus -21.3265 1.75229 8.31193 6.72477 -17.8571 -17.2755 -12.9908 -0.0694444
4.56881 0.00193 0.0055 0.00037 0.00154 0.00698 0.00544 0.00577 0.0171 0.00356
3L:8109560-8109710:plus -14.4082 2.76147 8.30275 3.94495 -18.7143 -26.602 -14.7523 2.04167 4.10092
0.00136 0.0038 0.000372 0.00595 0.0136 0.0248 0.00853 0.00849 0.00413
3L:8114800-8114950:minus -15 6.20183 -0.990826 5.85321 -26.2653 0.602041 -6.95413 0.180556 -0.697248
0.00162 0.000962 0.0159 0.00287 0.018 0.000188 0.00167 0.0158 0.0239
3L:8114800-8114950:plus -31.898 9.6789 2.0367 3.49541 0.653061 -18.0102 -19.4404 1.58333 4.66055
0.0164 0.000157 0.0057 0.00716 0.000239 0.00812 0.0187 0.00997 0.0035
3L:8118200-8118350:minus -13.5204 6.51376 7.02752 6.55963 -26.9694 -18.3061 -16.0826 1.48611 1.90826
0.00097 0.000829 0.000718 0.00172 0.023 0.0114 0.011 0.0103 0.00974
3L:8118200-8118350:plus -21.9694 -1.02752 5.14679 7.37615 -27.5306 -8.72449 -13.7798 2.98611 5.70642
0.00282 0.0145 0.00167 0.000951 0.0351 0.00169 0.00691 0.006 0.00222
3L:8121000-8121150:minus -33.4694 1.59633 3.07339 5.94495 -17.1531 -16.0102 -26.1651 3.06944 2.13761
0.0285 0.00582 0.00386 0.00261 0.00364 0.00406 0.0546 0.00581 0.00885
3L:8121000-8121150:plus -31.1735 0.87156 3.55963 3.88991 10.6531 -26.898 -5.94495 3.05556 3.52294
0.0109 0.00758 0.0032 0.00608 0.0000102 0.0302 0.00129 0.00584 0.00558
3L:8121840-8121990:minus -13.7857 1.33028 -2.08257 3.17431 -9.20408 -27.3776 -19.0642 2.01389 7.25688
0.00108 0.00642 0.0222 0.00842 0.00262 0.0351 0.0176 0.00858 0.00115
3L:8121840-8121990:plus -30.398 4.3578 0.541284 4.19266 -18.1939 -18.1224 -7.21101 9.625 1.11009
0.00901 0.0021 0.00968 0.00536 0.0086 0.00988 0.00177 0.000213 0.0138
3L:8130760-8130910:minus -23.2959 -0.146789 3.40367 1.82569 -26.1939 -9.5 -20.0275 3.52778 1.69725
0.00605 0.0108 0.0034 0.0156 0.0175 0.00294 0.0207 0.00486 0.0108
3L:8130760-8130910:plus -30.6327 -6.43119 1.65138 5.57798 1.58163 -9.7551 -5.3578 -0.347222
-2.88991 0.00968 0.0636 0.00656 0.0034 0.0000662 0.00358 0.0011 0.0186 0.0433
3L:8152040-8152190:minus -22.8469 3.80734 11.3119 7.29358 -8.89796 -18.5714 -19.1193 2.625 3.46789
0.00424 0.00259 0.0000659 0.00104 0.00189 0.0136 0.0178 0.00687 0.00582
3L:8152040-8152190:plus -22.6327 -2.6789 12.9083 4.07339 -18.4184 -8.86735 -19.1193 2.31944 2.57798
0.00373 0.0236 0.0000224 0.00562 0.0101 0.00198 0.0178 0.00769 0.0078
3L:8152840-8152990:minus -4.89796 5.31193 4.80734 9.61468 -18.4898 -8.5 -12.633 2.16667 -2.22018
0.000208 0.00143 0.00193 0.000255 0.0116 0.00126 0.00532 0.00812 0.0355
3L:8152840-8152990:plus -33.3061 -0.614679 1.7156 1.26606 -19.2755 -9.02041 -5.37615 1.34722
5.9633 0.0279 0.0127 0.00642 0.0191 0.0164 0.00213 0.0011 0.0108 0.00188
3L:8168940-8169090:minus -22.0714 2.58716 9.01835 0.486239 -8.67347 0.27551 -13.5688 1.48611 6.95413
0.00299 0.00404 0.000251 0.0237 0.00154 0.000257 0.00658 0.0103 0.00136
3L:8168940-8169090:plus -34.6327 0.422018 1.65138 5.22018 -18.4898 -17.2755 -23.5688 9.72222 7.22018
0.032 0.0089 0.00656 0.00418 0.0116 0.00544 0.0384 0.000199 0.00119
3L:8171480-8171630:minus -24.5918 12.844 12.3119 7.29358 -18.4184 -8.5 -13.7248 8.41667 -4.36697
0.00821 0.0000255 0.0000343 0.00104 0.0101 0.00126 0.00682 0.000458 0.0615
3L:8171480-8171630:plus -24.0408 3.83673 2.94495 5.93578 -18.3469 -18.051 -14.8716 4.26389 4.06422
0.00726 0.00115 0.00405 0.00264 0.00894 0.00886 0.00874 0.0036 0.00418
3L:8173920-8174070:minus -33.4898 3.84404 -0.715596 2.33945 -27.5 -8.72449 -25.4312 3.27778
0.458716 0.0286 0.00255 0.0146 0.0122 0.0337 0.00169 0.0498 0.00536 0.0163
3L:8173920-8174070:plus -33.0714 3.68367 4.70642 3.78899 1.17347 -8.79592 -18.7156 4 -5.07339
0.0267 0.00144 0.00201 0.00632 0.000101 0.0019 0.0167 0.00402 0.0739

3L:8179960-8180110:minus -23.7755 -0.899083 -0.40367 2.81651 -27.4592 -9.45918 -7.85321 -2.11111
5.66055 0.00678 0.0139 0.0132 0.00982 0.0329 0.00279 0.00204 0.0307 0.00227
3L:8179960-8180110:plus -30.4082 4.05102 2.17431 2.86239 -7.56122 -17.7551 -17.5963 -1.98611 4.14679
0.00908 0.000335 0.00542 0.00954 0.000419 0.00643 0.0141 0.0297 0.00406
3L:8180280-8180430:minus -31.7347 3.97959 6.44954 1.55963 -26.9592 -25.898 -4.02752 5.09722 1.27523
0.0149 0.000669 0.000941 0.0173 0.0215 0.0214 0.000752 0.00251 0.013
3L:8180280-8180430:plus -24.5612 -3.85321 -0.614679 3.13761 -9.19388 -0.244898 -13.4771 3.15278
-1.18349 0.00819 0.0325 0.0141 0.00854 0.00253 0.000496 0.00645 0.00563 0.0276
3L:8190920-8191070:minus -23 11.7064 -3.04587 9.44954 -27.3061 -18.051 -11.945 2.06944 5.44037
0.00498 0.0000549 0.0292 0.000326 0.0315 0.00886 0.00456 0.00841 0.00259
3L:8190920-8191070:plus -31.449 10.211 2.02752 2.19266 -17.4184 -18.0102 -16.5229 0.916667 3.08257
0.0123 0.000121 0.00572 0.0131 0.00457 0.00812 0.0119 0.0125 0.00681
3L:820280-820430:minus -22.551 3.25688 8.3211 7.20183 -18.4898 -19.1224 -17.844 1.04167 7.37615
0.00359 0.00316 0.000368 0.00112 0.0116 0.0175 0.0146 0.012 0.00107
3L:820280-820430:plus -23.8571 -3.66055 6.16514 7.37615 -18.1531 -18.7551 -4.87156 2.90278 -2.11009
0.00687 0.0309 0.00107 0.000951 0.00829 0.0141 0.000957 0.00619 0.0343
3L:820560-820710:minus -30.2551 -2.02752 6.38532 7.77982 -8.86735 -18.7857 -6.07339 2.98611 3.29358
0.00885 0.0196 0.00097 0.000652 0.00173 0.0146 0.00134 0.006 0.00623
3L:820560-820710:plus -22.5918 -2.33945 4.22018 4.08257 -18.449 -35.449 -19.211 4.72222 -0.743119
0.00365 0.0214 0.00245 0.00558 0.0107 0.0789 0.018 0.00296 0.0243
3L:821280-821430:minus -32.1122 -1.77064 8.40367 7.90826 -17.7551 -18.5714 -12.0642 -6.31944 1.68807
0.0178 0.0182 0.000352 0.000611 0.00658 0.0136 0.00468 0.0811 0.0109
3L:821280-821430:plus -31.551 1.29358 2.21101 2.70642 -18.2653 -18.2755 -19.2844 3.40278 2.27523
0.0134 0.00651 0.00534 0.0103 0.00882 0.0112 0.0183 0.00511 0.00846
3L:821760-821910:minus -31.7857 0.963303 6.19266 1.46789 -18.3469 -16.2755 -8.15596 -3.22222 1.78899
0.0157 0.00734 0.00106 0.0179 0.00894 0.00413 0.00217 0.0408 0.0103
3L:821760-821910:plus -23.0306 1.98165 6.27523 5.65138 -17.7143 -26.8571 -16.844 -4.16667 5.12844
0.00505 0.00505 0.00102 0.00322 0.00627 0.0288 0.0125 0.0512 0.00301
3L:822160-822310:minus -31.7041 3 1.05505 9.24771 -7.63265 -18.051 -15.0459 -9.25 -6.29358
0.0146 0.00348 0.00811 0.000409 0.000479 0.00886 0.00906 0.137 0.0955
3L:822160-822310:plus -31.5918 -1.23853 4.66055 -3.33945 -8.67347 -27.6735 -16.4312 1.29167 3.40367
0.0136 0.0154 0.00205 0.0737 0.00154 0.0443 0.0117 0.011 0.00603
3L:822960-823110:minus -24.0714 2 2.6055 4.79817 -17.4898 -17.5714 -19.578 5.63889 1.27523
0.00731 0.00502 0.00462 0.00453 0.0053 0.00616 0.0192 0.00196 0.013
3L:822960-823110:plus -14.1122 1.25688 -0.981651 3.70642 -17.8163 -17.3061 -2.88991 -5.59722 0.752294
0.00119 0.00659 0.0159 0.0066 0.00682 0.00548 0.000552 0.0701 0.0152
3L:823860-824010:minus -38.6531 -1.3578 3.77064 5.77982 -26.9388 -8.42857 -4.09174 -2.25 -1.11927
0.0321 0.016 0.00294 0.00294 0.0215 0.00104 0.000766 0.0319 0.027
3L:823860-824010:plus -12.449 3.25688 3.61468 2.27523 -19.051 -27.602 -14.0367 7.63889 1.6422
0.000428 0.00316 0.00313 0.0125 0.0162 0.0416 0.00731 0.000715 0.0111
3L:8282960-8283110:minus -31.4796 8.05505 6.27523 7.77982 -9.23469 -9.23469 -16.4404 0.833333 1.63303
0.0128 0.000371 0.00102 0.000652 0.00277 0.00238 0.0117 0.0128 0.0112
3L:8282960-8283110:plus -31.7347 12.2385 2.85321 4.44037 -9.16327 -18.0102 -11.9817 1.22222 -2.04587
0.0149 0.0000393 0.0042 0.00493 0.00249 0.00812 0.00459 0.0113 0.0336
3L:8290100-8290250:minus -41.2959 5.80734 -0.550459 3.59633 -18.7143 -0.204082 -22.4037 3.56944
-3.36697 0.0529 0.00116 0.0139 0.00693 0.0136 0.000463 0.0317 0.00478 0.0482
3L:8290100-8290250:plus -34 6.23853 14.055 3.24771 -18.6837 -18.2041 -16.5046 2.81944 5.76147
0.0307 0.000946 6.79e-06 0.00808 0.0126 0.0103 0.0118 0.00639 0.0021
3L:8292480-8292630:minus -22.6633 4.12844 7.34862 4.04587 -27.1939 -19.1531 -20.0275 2.90278 0.348624
0.00384 0.00229 0.000611 0.00572 0.0267 0.0177 0.0207 0.00619 0.0167
3L:8292480-8292630:plus -23.7041 2.86239 4.69725 4.05505 -26.9694 -9.65306 -17.0826 -0.888889
3.88073 0.00669 0.00366 0.00202 0.00566 0.023 0.0034 0.013 0.0218 0.00475
3L:8292940-8293090:minus -13.3265 15.5963 0.146789 5.59633 0.877551 -17.7143 -11.789 1.66667 1.22936
0.000779 8.91e-07 0.011 0.00331 0.00019 0.00634 0.0044 0.00969 0.0132

3L:8292940-8293090:plus -12.8163 -0.743119 10.3119 3.9633 -8.64286 -18.2041 -17.3028 -5.05556
-4.42202 0.000489 0.0132 0.000118 0.0059 0.00139 0.0103 0.0134 0.0624 0.0626
3L:8297120-8297270:minus -31.551 -1.43119 1.15596 7.17431 -17.1531 -27.2653 -15.4495 2.23611 1.25688
0.0134 0.0164 0.00783 0.00115 0.00364 0.0333 0.0098 0.00792 0.0131
3L:8297120-8297270:plus -34.1429 5.36697 -1.48624 0.93578 -18.449 -18.3061 -25.2936 5.38889 5.23853
0.0313 0.0014 0.0185 0.0209 0.0107 0.0114 0.049 0.0022 0.00285
3L:8301900-8302050:minus -32.8061 -1.46789 8.3945 -0.458716 -18.7551 -27.8265 -8.6422 1.59722
5.3945 0.0237 0.0166 0.000354 0.0308 0.0144 0.0498 0.00239 0.00993 0.00263
3L:8301900-8302050:plus -21.8878 0.880734 9.06422 6.02752 -28.1224 -8.42857 -19.4862 -0.444444
-7.83486 0.00258 0.00756 0.000245 0.00246 0.0457 0.00104 0.0189 0.0192 0.131
3L:8308280-8308430:minus -22.7041 -3.98165 0.862385 -3.54128 -18.5306 -26.602 -12.8073 0.152778 6.49541
0.00395 0.0337 0.00867 0.0767 0.0118 0.0248 0.00553 0.016 0.00154
3L:8308280-8308430:plus -20.551 -3.85321 1.88073 1.66972 -7.56122 -8.45918 -12.1927 -0.986111
9.04587 0.00168 0.0325 0.00604 0.0165 0.000419 0.00111 0.00481 0.0225 0.000423
3L:8308580-8308730:minus -40.2857 -1.02752 1.57798 6.29358 10.6939 -17.5714 -14.1835 2.18056 -0.357798
0.0385 0.0145 0.00674 0.00202 7.35e-06 0.00616 0.00755 0.00808 0.021
3L:8308580-8308730:plus -32.7041 -4.21101 0.981651 0.990826 -18.4184 9.79592 -26.1927 2.45833 -3.50459
0.0222 0.0358 0.00832 0.0206 0.0101 0.0000183 0.0548 0.00731 0.0497
3L:833160-833310:minus -14.8163 4.88991 1.37615 3.86239 -26.6735 -18.1531 0.311927 5.76389 -2.22018
0.00159 0.0017 0.00724 0.00622 0.0191 0.00997 0.000263 0.00185 0.0355
3L:833160-833310:plus -40.4796 -0.431193 6.42202 5.57798 -18.4184 -17.2755 -24.8349 8.86111 1.74312
0.0407 0.0119 0.000953 0.0034 0.0101 0.00544 0.0461 0.000349 0.0105
3L:8332100-8332250:minus -23.2653 1.7156 -0.761468 5.49541 -27.1939 -18.1224 -18.6055 0.402778
2.07339 0.00593 0.00557 0.0148 0.00355 0.0267 0.00988 0.0164 0.0147 0.00906
3L:8332100-8332250:plus -41.5102 5.76147 3.55046 5.50459 -17.8265 -18.3061 -3.08257 7.18056 7.25688
0.0582 0.00118 0.00321 0.00349 0.00691 0.0114 0.00058 0.000915 0.00115
3L:8333540-8333690:minus -23.6327 -0.513761 15.5229 -0.633028 -8.64286 -8.79592 -10.945 3
5.07339 0.00661 0.0123 7.23e-07 0.0327 0.00139 0.0019 0.00369 0.00597 0.00305
3L:8333540-8333690:plus -23.4796 -2.65138 12.0734 7.29358 -18.4898 -8.65306 -20.945 -3.61111 -4.98165
0.00634 0.0234 0.0000408 0.00104 0.0116 0.00156 0.0243 0.0449 0.0725
3L:8339440-8339590:minus -24.1122 9.15596 1.3578 2.77982 -27.2653 -8.72449 -22.8991 5.29167 -0.981651
0.00738 0.000207 0.00728 0.00997 0.0302 0.00169 0.0345 0.0023 0.026
3L:8339440-8339590:plus -32.0714 10.7798 13.6606 3.90826 -18.4184 -18.6327 -7.63303 2.51389 1.3211
0.0176 0.0000896 0.0000112 0.00603 0.0101 0.0137 0.00195 0.00716 0.0127
3L:8339920-8340070:minus -29.9592 4.55963 5.79817 1.7156 -18.6429 -9.42857 -17.9633 -1.79167 4.33028
0.00855 0.00194 0.00126 0.0162 0.0121 0.0027 0.0149 0.0282 0.00385
3L:8339920-8340070:plus -21.9898 9.34862 -6.79817 2.50459 -27.3367 -27.5612 -7.77064 -1.08333 -1.43119
0.00282 0.000187 0.0765 0.0114 0.0317 0.0393 0.00201 0.0231 0.029
3L:834000-834150:minus -33.2653 -0.522936 -0.513761 2.88073 -27.4898 -0.397959 -29.211
4.20833 -0.908257 0.0277 0.0123 0.0137 0.00945 0.0336 0.000525 0.0813 0.00369 0.0255
3L:834000-834150:plus -31 2.68807 13.6422 -0.0183486 -27.4592 0.0918367 -20.1743 5.41667 -0.651376
0.0105 0.0039 0.0000116 0.027 0.0329 0.000323 0.0212 0.00218 0.0235
3L:8352380-8352530:minus -31.4388 4.70642 11.3761 7 -18.4184 -26.602 -4.66972 -0.166667 11.4771
0.0122 0.00183 0.0000633 0.00132 0.0101 0.0248 0.000903 0.0176 0.0000679
3L:8352380-8352530:plus -22.1939 1.89908 2.6789 9.41284 -17.0816 -18.0102 -26.5229 0.916667 0.816514
0.00317 0.00521 0.00449 0.000354 0.00334 0.00812 0.057 0.0125 0.015
3L:8353460-8353610:minus -31.102 3.33945 3.77982 3.31193 -27.4898 -18.1224 -16.1193 6.36111 12.2752
0.0106 0.00307 0.00293 0.00786 0.0336 0.00988 0.0111 0.00139 0.0000141
3L:8353460-8353610:plus -23.4082 -2.76147 1.31193 1.68807 -26.7041 -17.898 -11.9725 5.52778 3.6789
0.00623 0.0241 0.00741 0.0164 0.0193 0.00726 0.00458 0.00207 0.00506
3L:8353960-8354110:minus -13.4898 -0.220183 0.100917 9.33028 -27.1939 -8.45918 -19.7615 -3.27778
0.385321 0.000946 0.0111 0.0112 0.000374 0.0267 0.00111 0.0198 0.0414 0.0166
3L:8353960-8354110:plus -22.1837 -0.53211 13.2569 7.29358 -18.4082 -18.0816 -12.6697 5.36111 7.22018
0.00315 0.0123 0.0000159 0.00104 0.00937 0.00934 0.00536 0.00223 0.00119

3L:835560-835710:minus	-31.7449	6.34862	5.04587	5.94495	-27.5612	-10.0204	-7.74312	6.66667	1.66972
0.0152	0.000897	0.00174	0.00261	0.0354	0.00399	0.00199	0.00119	0.011
3L:835560-835710:plus	-33.449	-0.844037	3.11009	-0.614679	-17.1837	-27.602	-16.1284	4.36111
4.34862	0.0284	0.0136	0.00381	0.0324	0.00366	0.0416	0.0111	0.00346	0.00384
3L:837640-837790:minus	-23.1837	7.34862	9.06422	3.89908	-17.7143	-18.051	-22.3486	-3.34722	-2.77982
0.00553	0.000541	0.000245	0.00604	0.00627	0.00886	0.0314	0.0421	0.042
3L:837640-837790:plus	-21.4388	-7.19266	1.20183	3.18349	-18.1531	-16.3469	-15.9817	4.04167	4.14679	0.002
0.0764	0.0077	0.00837	0.00829	0.00416	0.0108	0.00395	0.00406
3L:8392480-8392630:minus	-32.8469	1	1.66972	-0.137615	-7.89796	-19.0816	-19.3853	2.19444	5.44037
0.0243	0.00724	0.00652	0.0279	0.000678	0.017	0.0186	0.00804	0.00259
3L:8392480-8392630:plus	-13.4898	1.77064	14.9633	7.29358	-28.4592	-9.94898	-11.8991	4.58333	4.07339
0.000946	0.00546	1.77e-06	0.00104	0.0568	0.00393	0.00451	0.00315	0.00417
3L:839700-839850:minus	-32.4388	-2.97248	-3.98165	3.88991	-27.2347	-16.9796	4.10092	7.18056	1.38532
0.0195	0.0256	0.0378	0.00608	0.0283	0.0044	0.0000795	0.000915	0.0123
3L:839700-839850:plus	-23	5.34862	-2.52294	-0.688073	-18.051	-17.5714	-16.6514	2.27778	5.75229
0.00498	0.00141	0.0252	0.0333	0.00746	0.00616	0.0121	0.0078	0.00213
3L:8398360-8398510:minus	-31.6939	12.844	-3.04587	2.00917	-18.9796	-18.0102	-15.2018	3.34722	0.917431
0.0142	0.0000255	0.0292	0.0142	0.0155	0.00812	0.00934	0.00522	0.0146
3L:8398360-8398510:plus	-31.898	1.27523	0.990826	6.83486	-18.7551	0.94898	-14.8532	2.36111	-3.44037
0.0164	0.00655	0.00829	0.00142	0.0144	0.00014	0.00871	0.00757	0.049
3L:8404860-8405010:minus	-21.9388	-2.98165	2.75229	2.34862	-8.67347	-18.2041	-12.7523	-0.805556
-3.47706	0.0027	0.0257	0.00436	0.0121	0.00154	0.0103	0.00546	0.0213	0.0493
3L:8404860-8405010:plus	-31.7347	1.44954	-0.275229	0.376147	-18.1939	-18.3469	-16.6606	9.25	1.2844
0.0149	0.00615	0.0127	0.0244	0.0086	0.0124	0.0121	0.000273	0.0129
3L:841300-841450:minus	-34.1531	6.29358	3.61468	4.07339	-8.64286	-19.5714	-11.8532	-1.41667	-1.01835
0.0314	0.000922	0.00313	0.00562	0.00139	0.0202	0.00447	0.0254	0.0263
3L:841300-841450:plus	-24.898	3.61468	4.34862	7.73394	-27.898	-0.316327	-23.7339	7.66667	-5.6055
0.00831	0.00277	0.00233	0.000669	0.04	0.000524	0.0394	0.000704	0.0821
3L:8418240-8418390:minus	-31.1837	0.770642	5.20183	6.31193	-18.3469	-9.20408	-27.8165	-9.66667	-6.78899
0.0109	0.00786	0.00163	0.00199	0.00894	0.00225	0.0671	0.146	0.108
3L:8418240-8418390:plus	-21.398	-2.53211	5.53211	7.80734	-27.4082	-36.6633	-3.75229	-1.06944	9.61468
0.00197	0.0226	0.00142	0.00063	0.0321	0.115	0.000696	0.023	0.00029
3L:8434420-8434570:minus	-41.398	-5.19266	-3.37615	0.733945	-27.3367	-36.8163	-14.0917	6.68056	4.80734
0.0554	0.0464	0.032	0.0222	0.0317	0.12	0.0074	0.00119	0.00334
3L:8434420-8434570:plus	-23.0612	-0.211009	-0.211009	0.40367	-27.4592	0.122449	-5.11927	0.986111
2.85321	0.00512	0.0111	0.0124	0.0242	0.0329	0.000312	0.00103	0.0122	0.00733
3L:8435000-8435150:minus	-22.1837	13.3878	-0.688073	2.98165	-8.93878	-9.23469	-24.4771	7.16667
-2.83486	0.00315	0.0000869	0.0145	0.00905	0.00216	0.00238	0.0439	0.000922	0.0427
3L:8435000-8435150:plus	-41.551	2.14679	-1.54128	4.3211	-8.63265	-28.1224	-18.0183	-1.125	-2.51376
0.0607	0.00476	0.0188	0.00514	0.0013	0.0583	0.015	0.0234	0.039
3L:8442460-8442610:minus	-22.8571	0.66055	2.29358	2.06422	-18.1837	-8.65306	-20.8899	5.52778	0.642202
0.00427	0.00818	0.00518	0.0139	0.00835	0.00156	0.0241	0.00207	0.0157
3L:8442460-8442610:plus	-31.9286	3.98165	5.57798	10.5963	-18.4184	-7.57143	-13.3028	2.25	1.63303
0.0166	0.00242	0.00139	0.0001	0.0101	0.000644	0.0062	0.00788	0.0112
3L:8443300-8443450:minus	-12.2245	2.84404	-0.495413	9.08257	-18.6429	0.0918367	-27.2844	1.01389
-8.16514	0.000376	0.00368	0.0136	0.000444	0.0121	0.000323	0.0627	0.0121	0.14
3L:8443300-8443450:plus	-30.1837	-3.54128	-4.56881	2.54128	-18.4184	-8.23469	-18.5963	1.875	8.11009
0.00873	0.0299	0.0442	0.0112	0.0101	0.000875	0.0164	0.00901	0.000597
3L:8448360-8448510:minus	-22.9184	-0.201835	7.2844	2.77982	-19.051	-17.7143	-17.0642	-1.47222
-4.93578	0.00447	0.011	0.000632	0.00997	0.0162	0.00634	0.0129	0.0258	0.0718
3L:8448360-8448510:plus	-21.8878	-0.46789	-3.12844	10.0275	-26.9694	-9.23469	-18.0642	-3.125	3.66972
0.00258	0.0121	0.0299	0.000156	0.023	0.00238	0.0151	0.0399	0.00514
3L:8502880-8503030:minus	-33.8163	-3.50459	-1.66055	1.58716	1.87755	19.1531	-14.4862	2.18056	5.81651
0.0299	0.0296	0.0195	0.017	0.0000492	7.95e-07	0.00807	0.00808	0.00204

3L:8502880-8503030:plus -14.7041 -2.16514 5.23853 6.84404 1.14286 -18.8571 -24.1927 -2.29167 1.22018
0.00154 0.0204 0.00161 0.0014 0.000113 0.0151 0.0422 0.0322 0.0133
3L:8507860-8508010:minus -31.7755 2.98165 1.63303 6.84404 1.87755 -7.72449 -22.6514 -1.04167 -4.62385
0.0157 0.0035 0.00661 0.0014 0.0000492 0.00068 0.0331 0.0228 0.066
3L:8507860-8508010:plus -32.551 5.47706 3.33028 1.40367 -17.9286 -8.72449 -20.3945 2.09722 3.16514
0.0207 0.00133 0.00349 0.0184 0.0071 0.00169 0.0221 0.00832 0.00656
3L:8511740-8511890:minus -20.8469 6.62385 0.33945 5.43119 -28.2245 -0.540816 -18.3578 4.54167
6.18349 0.00178 0.000785 0.0104 0.00371 0.049 0.00054 0.0158 0.0032 0.00167
3L:8511740-8511890:plus -31.5918 1.79817 1.85321 9.61468 -26.1939 -18.051 -16.8624 9.73611 8.11009
0.0136 0.00541 0.0061 0.000255 0.0175 0.00886 0.0125 0.000197 0.000597
3L:851180-851330:minus -23.3367 7.2844 3.3945 -0.550459 8.86735 -8.72449 -25.2018 -4.375
-2.20183 0.00614 0.000559 0.00341 0.0317 0.00173 0.00169 0.0484 0.0537 0.0353
3L:851180-851330:plus -31.7755 0.302752 4.58716 3.19266 -9.19388 -17.9796 -8.68807 0.333333 -3.3211
0.0157 0.00929 0.00211 0.00829 0.00253 0.00764 0.00241 0.0151 0.0478
3L:8516560-8516710:minus -31.5102 2.3578 2.08257 11.0183 -18.449 -17.2347 -5.94495 8.08333 -1.18349
0.013 0.0044 0.0056 0.000052 0.0107 0.00511 0.00129 0.000556 0.0276
3L:8516560-8516710:plus -24.7143 8.19266 -2.07339 7.80734 -7.86735 -9.09184 -20.1284 7 7.24771
0.00826 0.000343 0.0221 0.00063 0.000617 0.00219 0.0211 0.00101 0.00116
3L:8518240-8518390:minus -41.5918 4.22936 1.10092 0.412844 -28.2347 -9.79592 -27.8899 5.93056 -3.33028
0.0623 0.0022 0.00798 0.0242 0.051 0.00377 0.0678 0.00171 0.0478
3L:8518240-8518390:plus -31.0306 14.6789 1.80734 2.53211 -28.0102 -17.2755 -14.2661 -0.0138889
9.92661 0.0105 3.91e-06 0.00621 0.0113 0.0449 0.00544 0.00769 0.0168 0.000193
3L:8519440-8519590:minus -41.6633 -2.88073 6.48624 0.66055 -19.3163 -17.3469 -22.2661 6.58333 -0.926606
0.0636 0.0249 0.000925 0.0225 0.0165 0.0058 0.0309 0.00124 0.0256
3L:8519440-8519590:plus -14.5204 6.49541 -3.48624 1.83486 -18.3878 -18.5 -10.5963 -0.347222
4.51376 0.00145 0.000837 0.033 0.0155 0.00931 0.0131 0.00345 0.0186 0.0036
3L:8520660-8520810:minus -33.8878 0.651376 -2.82569 2.23853 -8.89796 -18.5714 -31.0183 5.19444 -4.08257
0.0302 0.00821 0.0275 0.0128 0.00189 0.0136 0.105 0.00241 0.0567
3L:8520660-8520810:plus -21.6327 3.7551 0.0183486 6.00917 -27.2653 -0.173469 -12.4312 0.416667
0.963303 0.00215 0.00122 0.0115 0.00255 0.0302 0.000456 0.00508 0.0147 0.0145
3L:8521800-8521950:minus -29.6633 0.880734 4.51376 0.642202 -17.7857 -36.1122 -0.247706 -0.791667
5.81651 0.00847 0.00756 0.00218 0.0227 0.00676 0.0878 0.0003 0.0212 0.00204
3L:8521800-8521950:plus -30.7449 1.66055 1.97248 7.3578 -27.5 -0.173469 -21.3211 0.152778
3.42202 0.0101 0.00569 0.00584 0.000965 0.0337 0.000456 0.0261 0.016 0.00597
3L:8538020-8538170:minus -32.7347 4.65138 0.220183 6.6422 -7.67347 -19.0816 -19.4312 5.875 2.99083
0.0225 0.00187 0.0108 0.00161 0.00055 0.017 0.0187 0.00176 0.00706
3L:8538020-8538170:plus -23.1837 15.1376 4.58716 1.78899 20.4694 0.244898 -12.6606 -2.79167 -0.697248
0.00553 1.81e-06 0.00211 0.0158 1.65e-07 0.000285 0.00535 0.0367 0.0239
3L:8538680-8538830:minus -31.7041 3.78899 9.04587 7.63303 -8.96939 -18.6429 -11.6606 2.52778 -0.440367
0.0146 0.0026 0.000248 0.000745 0.00226 0.0139 0.00428 0.00712 0.0216
3L:8538680-8538830:plus -22.2245 0.137615 8.69725 3.90826 -18.7857 -17.3469 -23.2661 4.70833 2.01835
0.00325 0.00984 0.000299 0.00603 0.0148 0.0058 0.0366 0.00298 0.00928
3L:8539580-8539730:minus -14.5612 -0.0550459 -0.110092 1.08257 -8.93878 -26.5306 -6.44037 -1.45833
9.23853 0.00147 0.0105 0.012 0.0201 0.00216 0.023 0.00147 0.0257 0.000397
3L:8539580-8539730:plus -3.85714 3.50459 -2.70642 7.17431 -17.4184 -19.0102 5.55963 -4.88889 5.59633
0.00011 0.00289 0.0265 0.00115 0.00457 0.0159 0.0000477 0.0602 0.00234
3L:8540440-8540590:minus -33.1429 5.63303 0.798165 1.65138 -18.9388 -18.051 -21.2752 4.06944 5.66055
0.0272 0.00125 0.00886 0.0166 0.0151 0.00886 0.0259 0.00391 0.00227
3L:8540440-8540590:plus -32.6633 7.53211 -1.13761 0.174312 -17.9796 -9.38776 -31.8991 6.41667 -11.9817
0.0216 0.000489 0.0167 0.0257 0.00726 0.00266 0.117 0.00135 0.264
3L:8540720-8540870:minus -31.8061 -2.08257 0.550459 1.84404 -18.5204 -28.1122 -1.3945 5.08333 5.6055
0.0158 0.0199 0.00965 0.0154 0.0117 0.0565 0.000387 0.00253 0.00232
3L:8540720-8540870:plus -11.4082 1.92661 5.88991 10.0275 -17.0408 0.0510204 -14.9633 3.11111
-1.76147 0.000251 0.00516 0.00121 0.000156 0.00329 0.000349 0.00891 0.00572 0.0313

3L:8541400-8541550:minus -31.7755 2.58716 -0.100917 -0.201835 -27.0408 -17.5714 -17.7064 9.80556
1.01835 0.0157 0.00404 0.012 0.0285 0.0242 0.00616 0.0143 0.000188 0.0142
3L:8541400-8541550:plus -21.9286 -1.82569 5.61468 6.17431 0.836735 -27.4082 -10.8624 -0.680556
5.22936 0.0027 0.0185 0.00137 0.0022 0.000215 0.0356 0.00363 0.0206 0.00288
3L:8542960-8543110:minus -12.3673 3.90816 -2.48624 7.78899 -27.1939 -17.7143 -11.7339 -5.90278 1.65138
0.000396 0.00102 0.0249 0.000642 0.0267 0.00634 0.00435 0.0746 0.011
3L:8542960-8543110:plus -22.1122 1 2.69725 7.27523 -8.86735 -8.53061 -14.1743 4.84722 -2.26606
0.00304 0.00724 0.00446 0.00109 0.00173 0.00136 0.00754 0.00281 0.0359
3L:8569260-8569410:minus -23.0408 0.238532 15.4495 5.3945 -17.6429 -19.2755 -14.633 -1.43056 1.87156
0.00512 0.0095 9.17e-07 0.00379 0.00562 0.0185 0.00832 0.0255 0.00989
3L:8569260-8569410:plus -43.0714 6.6055 -0.220183 5.92661 -27.8265 -8.7551 -28.0734 4.30556
-2.15596 0.0893 0.000793 0.0125 0.00266 0.0388 0.00172 0.0695 0.00354 0.0348
3L:8593980-8594130:minus -22.5204 10.9266 10.5688 3.70642 10.398 0.0204082 -13.367 2.04167
-3.99083 0.00356 0.0000837 0.000101 0.0066 0.0000153 0.000377 0.00629 0.00849 0.0556
3L:8593980-8594130:plus -32.5102 4.93578 3.84404 1.98165 -9.23469 -17.1633 -8.3211 -2.79167 -2.04587
0.0202 0.00167 0.00286 0.0145 0.00277 0.005 0.00225 0.0367 0.0336
3L:8597180-8597330:minus -24.3265 3.90816 15.4862 2.76147 -18.7551 -25.4898 -13.4771 0.555556 1.01835
0.00784 0.00102 7.92e-07 0.01 0.0144 0.0205 0.00645 0.014 0.0142
3L:8597180-8597330:plus -14.551 3.97959 15.0459 2.66972 -7.89796 -18.051 -7.93578 0.194444 5.38532
0.00146 0.000669 1.26e-06 0.0106 0.000678 0.00886 0.00208 0.0157 0.00267
3L:8616880-8617030:minus -30.7347 13.4592 5.09174 2.04587 -26.9286 -8.65306 -12.4679 6.45833 0.568807
0.01 0.000064 0.00171 0.014 0.0214 0.00156 0.00512 0.00133 0.016
3L:8616880-8617030:plus -31.4796 13.5306 7.33945 2.55963 -17.7551 -9.08163 -9.87156 0.763889 -0.33945
0.0128 0.0000206 0.000614 0.0111 0.00658 0.00216 0.00301 0.0131 0.0209
3L:8627080-8627230:minus -22.7347 -3.07339 7.42202 5.90826 -26.2653 -9.5 -13.9633 -1.05556 -2.46789
0.00403 0.0263 0.000589 0.00271 0.018 0.00294 0.0072 0.0229 0.0384
3L:8627080-8627230:plus -22.4082 -1.33945 9.77982 6.49541 -9.54082 -17.7857 -15.6422 5.68056 3.21101
0.00345 0.0159 0.000162 0.00176 0.00294 0.0068 0.0102 0.00193 0.00644
3L:8653480-8653630:minus -21.6224 -2.23853 0.0825688 6.02752 -18.1531 -36.1122 -8.73394 -4.13889
-1.6055 0.00213 0.0208 0.0113 0.00246 0.00829 0.0878 0.00243 0.0508 0.0302
3L:8653480-8653630:plus -21.7755 -2.89908 -1.15596 5.74312 -18.0816 -8.72449 -10.0092 -2.02778 3.6789
0.00241 0.0251 0.0168 0.00302 0.00759 0.00169 0.00308 0.03 0.00506
3L:8655380-8655530:minus -34 -1.23853 -1.80734 0.137615 -17.4898 -18.1224 -12.8991 1.5 3.88073 0.0307
0.0154 0.0204 0.0259 0.0053 0.00988 0.00565 0.0103 0.00475
3L:8655380-8655530:plus -13.2245 3.57143 0.59633 11.0183 -17.6837 -19.051 -16.3303 4.72222 0.981651
0.000738 0.00175 0.00949 0.000052 0.0058 0.0165 0.0115 0.00296 0.0144
3L:8668980-8669130:minus -31.551 4.89908 6.69725 4.20183 -18.4592 -27.5612 -6.04587 6.20833 1.56881
0.0134 0.0017 0.000836 0.00533 0.0109 0.0393 0.00133 0.0015 0.0114
3L:8668980-8669130:plus 24.8061 2.56881 6.70642 7.17431 -8.82653 0.387755 -0.963303 4.81944
-3.41284 7.81e-09 0.00407 0.000832 0.00115 0.00163 0.000217 0.000352 0.00284 0.0487
3L:8676680-8676830:minus -31.551 1.27523 2.72477 2.63303 -18.7551 0.755102 -11.4954 1.04167 2.85321
0.0134 0.00655 0.00441 0.0107 0.0144 0.000175 0.00413 0.012 0.00733
3L:8676680-8676830:plus -42.4082 3.51376 2.88073 2.77982 -17.7143 -9.5 -26.7523 0.472222 -4.83486
0.0761 0.00288 0.00415 0.00997 0.00627 0.00294 0.0587 0.0144 0.0699
3L:8677440-8677590:minus -40.9898 -3.41284 8.34862 5.31193 -27.602 -8.5 -17.0092 -1.79167 1.34862
0.0455 0.0289 0.000363 0.00393 0.0364 0.00126 0.0128 0.0282 0.0125
3L:8677440-8677590:plus -32.9694 1.14679 -2.94495 1.24771 -18.4592 -8.72449 -26.3119 5.5 0.0642202
0.0254 0.00686 0.0284 0.0192 0.0109 0.00169 0.0556 0.00209 0.0181
3L:8679620-8679770:minus -33.7755 -0.568807 3.50459 4.59633 -28.2347 -18.3469 -20.8073 3.875
1.83486 0.0297 0.0125 0.00326 0.00478 0.051 0.0124 0.0237 0.00423 0.0101
3L:8679620-8679770:plus -31.3673 -3.69725 5.45872 -1.08257 -8.93878 -7.79592 -21.5321 4.08333 4.02752
0.0116 0.0312 0.00147 0.0378 0.00216 0.000723 0.0271 0.00388 0.00428
3L:8684900-8685050:minus -31.4388 -4.18349 -0.192661 4.04587 -27.2653 -17.0816 -20.2844 -4.19444
3.07339 0.0122 0.0355 0.0123 0.00572 0.0302 0.00487 0.0216 0.0515 0.00683

3L:8684900-8685050:plus -22.1531 1.12844 4.17431 3.33028 -17.5612 -28.3776 -13 3.34722 3.20183 0.0031 0.00691 0.0025 0.00778 0.00544 0.0609 0.00578 0.00522 0.0065
3L:8687160-8687310:minus -2.77551 -6.3578 7.24771 10.7615 -17.7551 -18.0102 -18.8807 -1.875 0.550459 0.0000378 0.0625 0.000644 0.0000826 0.00658 0.00812 0.0171 0.0288 0.0161
3L:8687160-8687310:plus -23.0714 5.33028 2.93578 3.56881 -16.4898 -7.79592 -15.4587 7.41667 -2.2844 0.0052 0.00142 0.00407 0.00699 0.00312 0.000723 0.00982 0.000807 0.0361
3L:8691700-8691850:minus -21.6633 -0.733945 3.98165 7 -18.3776 -18.3469 -12.156 -0.444444 2.94495 0.00223 0.0132 0.0027 0.00132 0.00926 0.0124 0.00477 0.0192 0.00714
3L:8691700-8691850:plus -3.85714 -0.669725 -0.972477 3.05505 -17.3469 -18.051 -19.3028 -6.02778 0.0917431 0.00011 0.0129 0.0158 0.00878 0.00394 0.00886 0.0183 0.0765 0.018
3L:8693480-8693630:minus -30.449 -0.66055 0.825688 6.22936 -35.9694 -18.1224 -22.6147 -1.54167 -1.68807 0.00925 0.0129 0.00878 0.00209 0.0709 0.00988 0.0329 0.0263 0.0308
3L:8693480-8693630:plus -21.398 3.33945 6.44037 6.72477 -8.37755 -17.9796 -18.6514 -2.875 -1.9633 0.00197 0.00307 0.000945 0.00154 0.00106 0.00764 0.0165 0.0375 0.0329
3L:8696980-8697130:minus -42.4796 0.853211 -2.85321 4.86239 -18.9388 -18.051 -14 5.05556 1.27523 0.0777 0.00763 0.0277 0.00449 0.0151 0.00886 0.00725 0.00256 0.013
3L:8696980-8697130:plus -31.4796 -0.155963 6.99083 6.57798 -18.5306 -18.2755 -12.1651 4.45833 4.6055 0.0128 0.0109 0.000731 0.00167 0.0118 0.0112 0.00478 0.00332 0.00352
3L:8698140-8698290:minus -21.4796 -0.357798 6.05505 1.79817 -18.5204 -18.051 -2.11927 5.44444 3.87156 0.00202 0.0116 0.00113 0.0157 0.0117 0.00886 0.000457 0.00215 0.00477
3L:8698140-8698290:plus -33.7347 3.89908 7 6.00917 -18.1224 -8.23469 -16.633 14.3611 1.07339 0.0294 0.0025 0.000727 0.00255 0.00782 0.000875 0.0121 1.51e-06 0.014
3L:8714460-8714610:minus -32.7755 -2.18349 5.49541 1.99083 -26.5918 -8.57143 -13.7339 3.84722 -0.642202 0.0234 0.0205 0.00144 0.0144 0.0186 0.00149 0.00684 0.00428 0.0234
3L:8714460-8714610:plus -22 -1.45872 12.2844 7.29358 -18.5612 -18.051 -17.1927 1.31944 -0.981651 0.00291 0.0165 0.0000351 0.00104 0.0119 0.00886 0.0132 0.0109 0.026
3L:871860-872010:minus -24.1837 0.825688 2.66055 3.48624 -27.1224 -8.42857 -26.3303 3.51389 2.63303 0.00751 0.00771 0.00452 0.0072 0.0245 0.00104 0.0557 0.00489 0.00771
3L:871860-872010:plus -21.8878 13.4128 -5.11927 4.75229 1.17347 -26.3776 -14.9541 -3.63889 4.56881 0.00258 0.0000152 0.0509 0.00461 0.000101 0.0222 0.00889 0.0452 0.00356
3L:872380-872530:minus -32.6633 -2.15596 -0.715596 0.302752 -8.67347 -36.2245 -5.93578 -0.763889 7.73394 0.0216 0.0203 0.0146 0.0248 0.00154 0.0923 0.00129 0.0211 0.000784
3L:872380-872530:plus -13.9184 2.3211 -4.29358 2.33945 -17.6429 -18.7857 -17.7798 1.19444 0.348624 0.00112 0.00446 0.0411 0.0122 0.00562 0.0146 0.0145 0.0114 0.0167
3L:872600-872750:minus -22.5204 4.08257 -1.02752 6.00917 -17.1531 -26.8265 -13.5872 2.13889 0.926606 0.00356 0.00233 0.0161 0.00255 0.00364 0.0285 0.00661 0.0082 0.0146
3L:872600-872750:plus -30.9184 0.788991 13.0367 3.61468 -18.449 -19.2041 -24.8257 -3.18056 -0.0275229 0.0103 0.00781 2e-05 0.0068 0.0107 0.0179 0.0461 0.0404 0.0186
3L:872820-872970:minus -22.5204 4.08257 -1.02752 6.00917 -17.1531 -26.8265 -13.5872 2.13889 0.926606 0.00356 0.00233 0.0161 0.00255 0.00364 0.0285 0.00661 0.0082 0.0146
3L:872820-872970:plus -30.9184 0.788991 13.0367 3.61468 -18.449 -19.2041 -24.8257 -3.18056 -0.0275229 0.0103 0.00781 2e-05 0.0068 0.0107 0.0179 0.0461 0.0404 0.0186
3L:8734340-8734490:minus -22.2551 2.11927 1.3211 -1.18349 -18.1122 -9.79592 -9.83486 2.73611 5.00917 0.00327 0.00481 0.00738 0.0389 0.00771 0.00377 0.00298 0.00659 0.0031
3L:8734340-8734490:plus -32.2449 8.58716 5.70642 2.24771 -17.7143 -17.9388 -10.4495 -4.18056 3.93578 0.0183 0.000278 0.00131 0.0127 0.00627 0.00738 0.00335 0.0513 0.00456
3L:875520-875670:minus -24.2551 4.3211 1.04587 2.26606 -17.5204 -18.3469 -23.5138 1.27778 -0.110092 0.00768 0.00213 0.00813 0.0126 0.00532 0.0124 0.0381 0.0111 0.0191
3L:875520-875670:plus -22.1837 6.18349 -0.0917431 5.31193 -17.4184 -9.72449 -2.66055 7.84722 3.87156 0.00315 0.000972 0.0119 0.00393 0.00457 0.00352 0.000521 0.000636 0.00477
3L:8756020-8756170:minus -31.3673 -0.0550459 8.92661 3.89908 -27.7041 -27.8265 -15.055 -2.79167 0.963303 0.0116 0.0105 0.000264 0.00604 0.038 0.0498 0.00907 0.0367 0.0145
3L:8756020-8756170:plus -13.3776 2.22936 2.95413 5.47706 -18.7245 -9.5 -17.0917 0.722222 -1.21101 0.000831 0.00461 0.00404 0.0036 0.0136 0.00294 0.013 0.0133 0.0277

3L:8756840-8756990:minus -23.7347 -1.58716 2.18349 4.22018 -8.63265 -26.3367 -15.3119
-0.541667 1.33028 0.00673 0.0172 0.0054 0.00531 0.0013 0.0221 0.00954 0.0197 0.0127
3L:8756840-8756990:plus -21.9286 -3.49541 1.88991 7.6422 -8.04082 0.0510204 -2.88991
-1.91667 2.89908 0.0027 0.0295 0.00602 0.000721 0.000831 0.000349 0.000552 0.0291
0.00722
3L:8757100-8757250:minus -33.2245 1.20183 2.09174 3.95413 -18.7143 -9.02041 -29.5046
5.15278 -0.293578 0.0276 0.00673 0.00558 0.00593 0.0136 0.00213 0.0848 0.00245 0.0207
3L:8757100-8757250:plus -31.1429 -5.06422 -0.0275229 11.2936 -8.96939 -18.6429 -26.0459
0.333333 -2.00917 0.0108 0.0449 0.0117 0.0000139 0.00226 0.0139 0.0538 0.0151 0.0333
3L:8757920-8758070:minus -32.0714 1.0367 0.733945 5.57798 -27.1531 9.37755 -24.7431
-2.125 -6.01835 0.0176 0.00714 0.00906 0.0034 0.0249 0.0000363 0.0456 0.0308 0.0892
3L:8757920-8758070:plus -13.7143 -0.412844 -0.412844 2.7156 -9.20408 -0.469388 -28.367
10.4444 9.55046 0.00105 0.0119 0.0133 0.0102 0.00262 0.000533 0.0723 0.000118 0.000321
3L:8759440-8759590:minus -23.9286 -5.29358 -2.11927 0.963303 -17.4898 -27.6735
-20.1101 9.69444 11.8532 0.00701 0.0476 0.0224 0.0208 0.0053 0.0443 0.021 0.000203 0.0000428
3L:8759440-8759590:plus -30.1429 4.05505 4.15596 11.2936 -17.6429 -9.23469 -19.9633 -2.79167
3.14679 0.00866 0.00235 0.00251 0.0000139 0.00562 0.00238 0.0205 0.0367 0.00666
3L:876060-876210:minus -13.8878 -1.3578 9.76147 4.08257 -18.4592 -25.8265 -16.6697 -1.93056
4.9633 0.00111 0.016 0.000163 0.00558 0.0109 0.0212 0.0122 0.0293 0.00316
3L:876060-876210:plus -20.6633 0.954128 2.6422 3.74312 -27.1531 -8.72449 -15.2569
2.13889 -1.73394 0.00173 0.00736 0.00455 0.00651 0.0249 0.00169 0.00944 0.0082 0.0311
3L:876600-876750:minus -14.7041 0.53211 7.58716 10.5963 -27.3061 -27.6735 -8.15596 4.19444
2.47706 0.00154 0.00856 0.000543 0.0001 0.0315 0.0443 0.00217 0.00371 0.00801
3L:876600-876750:plus -23.1837 4.37615 2.62385 6.57798 -18.9796 -18.7143 -23.1101 -0.652778
2.0367 0.00553 0.00208 0.00458 0.00167 0.0155 0.014 0.0357 0.0204 0.00923
3L:881040-881190:minus -20.6939 -1.43119 -0.376147 3.3578 -17.0816 -19.051 -5.08257
-1.73611 9.45872 0.00174 0.0164 0.0131 0.00767 0.00334 0.0165 0.00102 0.0277 0.000337
3L:881040-881190:plus -23 9 5.76147 7 -18.1224 -8.72449 -24.0917 11.1111 1.53211 0.00498
0.000224 0.00128 0.00132 0.00782 0.00169 0.0416 0.0000695 0.0116
3L:8819020-8819170:minus -22.551 -0.183486 -4.76147 1.77982 -17.1939 1.16327 -7.01835
-0.541667 9.66055 0.00359 0.011 0.0464 0.0158 0.00377 0.0000831 0.00169 0.0197 0.000268
3L:8819020-8819170:plus -30.2857 3.77064 -3.05505 1.83486 -17.7449 -17.7551 -6.77982
1.65278 2.93578 0.00887 0.00262 0.0293 0.0155 0.0063 0.00643 0.0016 0.00974 0.00717
3L:881980-882130:minus -20.1735 2.70642 6.81651 6.06422 -26.8571 -9.23469 -8.58716 5.52778
2.3211 0.00165 0.00387 0.000791 0.00239 0.0198 0.00238 0.00236 0.00207 0.00839
3L:881980-882130:plus -21.2143 -4.44037 15.6239 -0.513761 -17.4898 -36.0408 -8.06422
3.95833 9.87156 0.00189 0.0381 5.44e-07 0.0313 0.0053 0.0837 0.00213 0.00409 0.000217
3L:8820060-8820210:minus -33.2653 3.37615 4.3945 5.29358 -18.5204 -9.23469 -24.2294
4.61111 1.08257 0.0277 0.00303 0.00229 0.00398 0.0117 0.00238 0.0424 0.00311 0.0139
3L:8820060-8820210:plus -13.7857 -1.26606 -2.59633 2.23853 -27.8265 -9.5 -25.8257 1.48611
-0.93578 0.00108 0.0156 0.0257 0.0128 0.0388 0.00294 0.0524 0.0103 0.0257
3L:8820440-8820590:minus -33.1531 -1.33945 1.27523 5.90826 -8.93878 -18.3061 -21.6239 2.69444 4.23853
0.0273 0.0159 0.0075 0.00271 0.00216 0.0114 0.0275 0.00669 0.00394
3L:8820440-8820590:plus -11.8163 8.05505 2.21101 6.16514 -17.4184 -17.9796 -13.1927 -4.45833 3.88073
0.000272 0.000371 0.00534 0.00223 0.00457 0.00764 0.00604 0.0547 0.00475
3L:885060-885210:minus -23.6327 -3.20183 0.266055 1.56881 -17.7143 -26.9694 -13.9908 -1.875 2.25688
0.00661 0.0273 0.0106 0.0172 0.00627 0.0304 0.00724 0.0288 0.00853
3L:885060-885210:plus -12.0714 3.7551 2.29358 9.44037 -18.6837 -18.7857 -8.65138 1.84722 3.17431
0.000318 0.00122 0.00518 0.000339 0.0126 0.0146 0.00239 0.0091 0.00653
3L:887780-887930:minus -21.7755 11.7706 0.642202 3.77064 -16.7143 -27.6327 -9.54128 -0.208333
5.3211 0.00241 0.0000525 0.00935 0.00637 0.00319 0.043 0.00283 0.0178 0.00275
3L:887780-887930:plus -33.0102 1.20183 7.52294 6.14679 -18.7857 -0.244898 -16.8807 0.486111 -4.70642
0.0259 0.00673 0.00056 0.00224 0.0148 0.000496 0.0126 0.0144 0.0676
3L:8884360-8884510:minus -31.7041 1.29358 5.18349 4.23853 -26.9286 -18.0816 -24.0092 3.48611 -0.119266

0.0146 0.00651 0.00165 0.00522 0.0214 0.00934 0.0411 0.00494 0.0192
3L:8884360-8884510:plus -24.1429 3.61224 -3.78899 6.33945 -17.7143 -10.0204 -11.0275 -2.40278 4.73394
0.0074 0.00167 0.0359 0.00196 0.00627 0.00399 0.00375 0.0332 0.00342
3L:8884600-8884750:minus -33.0408 -1.07339 1.07339 3.33028 -8.33673 -8.82653 -21.5321 6.59722 -3.08257
0.0264 0.0147 0.00806 0.00778 0.00101 0.00191 0.0271 0.00124 0.0455
3L:8884600-8884750:plus -24.1429 3.61224 -3.78899 6.33945 -17.7143 -10.0204 -11.0275 -2.40278 4.73394
0.0074 0.00167 0.0359 0.00196 0.00627 0.00399 0.00375 0.0332 0.00342
3L:8905540-8905690:minus -23.6327 -2.36697 3.68807 -1.97248 -28.3776 0.979592 -15.5321 10.3333 1.74312
0.00661 0.0216 0.00304 0.0508 0.0558 0.000136 0.00995 0.000128 0.0105
3L:8905540-8905690:plus -14.6327 2.77982 2.0367 9.7156 -7.33673 -27.6735 -1.58716 1.04167 3.41284
0.0015 0.00377 0.0057 0.000242 0.000388 0.0443 0.000404 0.012 0.006
3L:8941160-8941310:minus -22.1837 6.58716 -1.38532 7.70642 -18.6837 -17.7551 -8.69725 0.291667 0.963303
0.00315 0.000799 0.018 0.000708 0.0126 0.00643 0.00242 0.0153 0.0145
3L:8941160-8941310:plus -21.551 2.17431 4.79817 0.412844 -17.449 -9.57143 -12.0642 1.66667 -1.11009
0.00205 0.00471 0.00193 0.0242 0.00484 0.00326 0.00468 0.00969 0.0269
3L:8941360-8941510:minus -3.40816 -3.3211 -1.20183 5.43119 -17.6429 -18.2041 -5.77064 0.902778 -1.14679
0.0000685 0.0282 0.017 0.00371 0.00562 0.0103 0.00123 0.0125 0.0272
3L:8941360-8941510:plus -20.6939 4.34862 1.11009 7 -18.4184 -9.20408 -10.8899 7.59722 -0.715596
0.00174 0.0021 0.00796 0.00132 0.0101 0.00225 0.00365 0.000731 0.0241
3L:8944740-8944890:minus -22.551 3.76147 -0.614679 5.63303 -26.3061 -9.23469 -21.367 0.125
-2.0367 0.00359 0.00263 0.0141 0.00326 0.0181 0.00238 0.0263 0.0161 0.0336
3L:8944740-8944890:plus -14.3673 -5.76147 5.97248 6.19266 -26.9694 -9.53061 -8.04587 6.5 9.45872
0.00132 0.0538 0.00117 0.00216 0.023 0.0031 0.00212 0.0013 0.000337
3L:8947280-8947430:minus -13.1224 -0.954128 1.14679 0.220183 -17.7857 -18.7857 -15.4862 -0.486111
7.63303 0.000633 0.0141 0.00785 0.0253 0.00676 0.0146 0.00987 0.0194 0.000928
3L:8947280-8947430:plus -23.2857 5.50459 -0.0917431 3.44954 -18.2245 -18.0816 -9.70642 0.0277778
3.05505 0.00593 0.00132 0.0119 0.00738 0.00877 0.00934 0.00291 0.0166 0.00692
3L:8947540-8947690:minus -20.5918 13.4592 -5.22936 2.0367 -18.4184 -8.94898 -21.6972 1.65278 1.78899
0.00169 0.000064 0.0523 0.0141 0.0101 0.00203 0.0279 0.00974 0.0103
3L:8947540-8947690:plus -23.2653 1.34862 2.17431 1.74312 -17.3061 -18.1224 -19.0459 -0.319444
0.385321 0.00593 0.00637 0.00542 0.016 0.00389 0.00988 0.0176 0.0184 0.0166
3L:8949840-8949990:minus -14.6735 3.30275 3.87156 2.11009 -18.4592 -0.27551 -9.07339 9.47222 -1.13761
0.00152 0.00311 0.00282 0.0136 0.0109 0.000502 0.00259 0.000236 0.0271
3L:8949840-8949990:plus -14.4082 13.4592 6.30275 3.45872 -9.23469 -26.7551 -14.8991 0.125 -2.01835
0.00136 0.000064 0.00101 0.00731 0.00277 0.0263 0.00879 0.0161 0.0334
3L:8957680-8957830:minus -34.2959 13.5306 13.5872 7.45872 -26.5306 -9.02041 -25.0734 3.54167 -4.76147
0.0316 0.0000206 0.0000122 0.000894 0.0185 0.00213 0.0476 0.00483 0.0686
3L:8957680-8957830:plus -33.1122 13.3878 12.4679 5.90826 -19.0204 -18.898 -8.53211 13.5139 1.72477
0.0271 0.0000869 0.0000307 0.00271 0.0158 0.0152 0.00234 5.44e-06 0.0106
3L:8959780-8959930:minus -24.2551 -1.3945 -2.92661 1.14679 -18.1531 -18.3061 -18.3486 7.04167 -0.651376
0.00768 0.0162 0.0283 0.0198 0.00829 0.0114 0.0158 0.000984 0.0235
3L:8959780-8959930:plus -30.2143 8.18349 -2.78899 2.83486 -18.4184 -36.5204 -12.8532 -2.625 1.69725
0.0088 0.000345 0.0272 0.0097 0.0101 0.109 0.00559 0.0351 0.0108
3L:8961580-8961730:minus -40.4796 -1.40367 -1.37615 5.57798 -27.8265 -9.09184 -18.4037 3.25 3.46789
0.0407 0.0163 0.0179 0.0034 0.0388 0.00219 0.0159 0.00542 0.00582
3L:8961580-8961730:plus -21.9592 5.0367 2.81651 7.77982 10.9184 -36.0408 -5.9633 1.73611 5.3945
0.00279 0.0016 0.00426 0.000652 4.19e-06 0.0837 0.0013 0.00946 0.00263
3L:8967840-8967990:minus -22 -2.01835 -0.669725 2.12844 -18.0816 -7.38776 -18.2936 -2.77778 7.94495
0.00291 0.0195 0.0144 0.0134 0.00759 0.000563 0.0156 0.0365 0.000667
3L:8967840-8967990:plus -32.5102 13.5306 -1.75229 2.54128 -18.3776 9.37755 -11.7339 7.5 3.77982
0.0202 0.0000206 0.0201 0.0112 0.00926 0.0000363 0.00435 0.000771 0.00489
3L:8971920-8972070:minus -23.6327 -1.44954 14.7248 7.2844 -8.33673 -17.0102 -8.45872 1.02778 -6.06422
0.00661 0.0165 2.82e-06 0.00105 0.00101 0.00453 0.00231 0.012 0.0902
3L:8971920-8972070:plus -22.7041 3.61224 -0.568807 -0.293578 -17.4184 -9.86735 -17.7431 -4.65278

-1.22018 0.00395 0.00167 0.0139 0.0292 0.00457 0.00391 0.0144 0.0571 0.0278
3L:8978640-8978790:minus -31.8469 -1.92661 -2.44037 6.16514 0.836735 -8.72449 -12.8165 0.277778 -0.53211
0.0162 0.019 0.0246 0.00223 0.000215 0.00169 0.00554 0.0153 0.0222
3L:8978640-8978790:plus -23.2959 3.80734 14.8257 1.83486 -27.1531 0.755102 -17.0275 1.98611 -1.36697
0.00605 0.00259 2.55e-06 0.0155 0.0249 0.000175 0.0129 0.00866 0.0286
3L:8979140-8979290:minus -21.9286 -0.0917431 8.15596 10.7615 0.877551 -26.5612 1.74312 -3.01389
5.17431 0.0027 0.0106 0.000403 0.0000826 0.00019 0.0237 0.000178 0.0388 0.00296
3L:8979140-8979290:plus -30.5102 8.52294 4.42202 8.61468 -17.4184 -9.57143 -23.156 5.97222 1.73394
0.00947 0.000288 0.00226 0.000552 0.00457 0.00326 0.036 0.00168 0.0106
3L:8979660-8979810:minus -42.2857 0.697248 7.3211 6.02752 -17.449 -26.898 -15.0092 3.54167 -0.449541
0.0731 0.00807 0.00062 0.00246 0.00484 0.0302 0.00899 0.00483 0.0216
3L:8979660-8979810:plus -21.8163 5.66972 9.40367 8.80734 20.4694 -8.79592 -20.1468 -3.625 -4.10092
0.00244 0.00123 0.0002 0.000518 1.65e-07 0.0019 0.0211 0.045 0.057
3L:8990120-8990270:minus -31.102 2.57798 4.41284 7.72477 -18.449 -19.3061 -18.4771 -3.16667 -2.69725
0.0106 0.00406 0.00227 0.000681 0.0107 0.0187 0.0161 0.0403 0.0411
3L:8990120-8990270:plus -30.0306 4.12844 -1.26606 1.33028 -18.3878 -18.7857 -2.21101 -0.833333
9.30275 0.00856 0.00229 0.0173 0.0188 0.00931 0.0146 0.000467 0.0215 0.00038
3L:8990280-8990430:minus -31.102 2.57798 4.41284 7.72477 -18.449 -19.3061 -18.4771 -3.16667 -2.69725
0.0106 0.00406 0.00227 0.000681 0.0107 0.0187 0.0161 0.0403 0.0411
3L:8990280-8990430:plus -22.7041 -3.78899 -0.926606 2.06422 -7.67347 -17.051 -8.76147 1.33333
3.99083 0.00395 0.032 0.0156 0.0139 0.00055 0.00479 0.00245 0.0109 0.00433
3L:8991080-8991230:minus -11.8163 10.1376 0.211009 2.50459 -18.4184 -17.9388 -10.7156 -2.06944 1.14679
0.000272 0.000125 0.0108 0.0114 0.0101 0.00738 0.00353 0.0304 0.0136
3L:8991080-8991230:plus -39.6531 -0.990826 0.137615 3.14679 10.6531 0.0510204 -16.4679 0.777778
3.66972 0.0336 0.0143 0.0111 0.00847 0.0000102 0.000349 0.0118 0.0131 0.00514
3L:9040360-9040510:minus -32.2959 2.46789 -0.155963 5.31193 -18.7143 -8.72449 -27.3394 4.16667
-2.08257 0.0186 0.00422 0.0122 0.00393 0.0136 0.00169 0.0631 0.00375 0.034
3L:9040360-9040510:plus -30.9286 -0.0733945 2.30275 6.44954 -7.64286 -0.540816 -14.5413 -0.305556
-0.651376 0.0104 0.0106 0.00517 0.00183 0.000499 0.00054 0.00816 0.0184 0.0235
3L:9065400-9065550:minus -30.6633 -0.357798 -4.05505 2.11009 -19.3163 -7.16327 -21.2844 5.34722
5.58716 0.00976 0.0116 0.0385 0.0136 0.0165 0.000547 0.0259 0.00225 0.00237
3L:9065400-9065550:plus -33.0408 -0.559633 10.3119 2.77064 -18.4184 -18.9388 -25.7982 8.33333
0.66055 0.0264 0.0124 0.000118 0.01 0.0101 0.0154 0.0522 0.000481 0.0156
3L:9066060-9066210:minus -42.8571 -1.85321 1.87156 3.11009 -27.5714 -17.2041 -23.6606 3.16667 -2.26606
0.0873 0.0186 0.00606 0.00862 0.0357 0.00507 0.039 0.0056 0.0359
3L:9066060-9066210:plus -21.6939 -2.88073 14.8257 7.29358 10.9184 -27.602 -22.6514 -1.06944 -6.27523
0.00226 0.0249 2.55e-06 0.00104 4.19e-06 0.0416 0.0331 0.023 0.0952
3L:9067580-9067730:minus -39.9184 -4.83486 3.05505 7.80734 -17.3776 -26.898 -17.7064 4.25 -3.68807
0.0344 0.0422 0.00389 0.00063 0.00419 0.0302 0.0143 0.00362 0.0516
3L:9067580-9067730:plus -31.8469 -1.15596 -0.422018 11.0183 -17.4898 -28.4082 -11.3119 5.83333
6.95413 0.0162 0.0151 0.0133 0.000052 0.0053 0.0615 0.00398 0.00179 0.00136
3L:9085500-9085650:minus -22.8571 4.16514 -0.963303 3.87156 -18.051 -9.16327 -18.8716 0.694444
-0.440367 0.00427 0.00226 0.0158 0.00618 0.00746 0.00223 0.0171 0.0134 0.0216
3L:9085500-9085650:plus -22.8469 -1.07339 3.51376 7.17431 -17.1939 -19.0102 -8.52294 0.805556 1.25688
0.00424 0.0147 0.00325 0.00115 0.00377 0.0159 0.00234 0.013 0.0131
3L:9086040-9086190:minus -31.3673 0.614679 2.29358 2.85321 -18.4184 -9.82653 -15.3486 3.94444 -3.88073
0.0116 0.00832 0.00518 0.00955 0.0101 0.00381 0.00961 0.00411 0.0539
3L:9086040-9086190:plus -22.8878 -2.22936 6.47706 4.08257 -8.26531 -8.57143 -8.70642 -6.375 1.89908
0.00441 0.0208 0.000928 0.00558 0.000934 0.00149 0.00242 0.082 0.0098
3L:9089020-9089170:minus -23.2959 -0.40367 4.33028 9.33028 -8.40816 -9.02041 -8.14679 0.430556 -2.77982
0.00605 0.0118 0.00235 0.000374 0.00108 0.00213 0.00217 0.0146 0.042
3L:9089020-9089170:plus -21.8878 -4.05505 13.2936 7.29358 0.877551 -8.57143 -13.1468 2.36111 2.88991
0.00258 0.0343 0.0000155 0.00104 0.00019 0.00149 0.00598 0.00757 0.00725
3L:9089860-9090010:minus -31.7449 -4.41284 7.36697 4.16514 -17.6837 -8.53061 -25.4404 1.90278 3.81651

0.0152 0.0378 0.000605 0.00538 0.0058 0.00136 0.0499 0.00892 0.00482
3L:9089860-9090010:plus -13.9898 6.19266 -0.440367 4.86239 -17.5612 -18.0816 -1.3578 10.1111
7.36697 0.00114 0.000967 0.0134 0.00449 0.00544 0.00934 0.000384 0.000151 0.00108
3L:9118380-9118530:minus -31.6327 7.07339 5.12844 7.37615 -17.7143 -18.4184 -12.9908 -3.15278 0.642202
0.0138 0.000624 0.00169 0.000951 0.00627 0.0127 0.00577 0.0402 0.0157
3L:9118380-9118530:plus -31.551 0 4.44954 7.80734 -18.4898 -27.1939 -10.8624 1.625 1.37615
0.0134 0.0103 0.00224 0.00063 0.0116 0.0327 0.00363 0.00983 0.0123
3L:9127580-9127730:minus -21.6224 -3.34862 14.844 -0.46789 -17.0408 -8.87755 -5.86239 0.680556 5.75229
0.00213 0.0284 2.33e-06 0.0309 0.00329 0.00198 0.00126 0.0135 0.00213
3L:9127580-9127730:plus -30.6327 5.26606 2.90826 2 -9.20408 -7.45918 -25.4495 -0.986111 -0.366972
0.00968 0.00146 0.00411 0.0143 0.00262 0.000582 0.05 0.0225 0.021
3L:9130260-9130410:minus -32.9592 8.68807 10.8624 3.43119 -19.051 -8.5 -21.7523 1.26389 -0.816514
0.0252 0.000264 0.0000868 0.00749 0.0162 0.00126 0.0282 0.0111 0.0249
3L:9130260-9130410:plus -3.89796 15.1376 11.7798 5.30275 -9.20408 -17.3469 -14.8716 -0.263889
1.69725 0.000113 1.81e-06 0.0000501 0.00396 0.00262 0.0058 0.00874 0.0181 0.0108
3L:9328800-9328950:minus -13.1837 0.0642202 4.51376 0.587156 -17.449 -18.1224 -4.44037 -2.26389
-0.12844 0.000705 0.0101 0.00218 0.0231 0.00484 0.00988 0.000846 0.032 0.0193
3L:9328800-9328950:plus -4.33673 7.66972 10.2294 10.8624 -27.1224 -9.45918 -19.789 -0.125 -2.66972
0.00014 0.000456 0.000125 0.0000687 0.0245 0.00279 0.0199 0.0174 0.0407
3L:9333400-9333550:minus -13.0408 1.27523 4.86239 4.40367 -26.898 0.612245 -11.9083 3.05556 12.2752
0.000576 0.00655 0.00188 0.00497 0.0203 0.000187 0.00452 0.00584 0.0000141
3L:9333400-9333550:plus -21.6633 1.59633 8.95413 10.7615 -17.449 -27.6327 -2.62385 -0.319444
3.14679 0.00223 0.00582 0.000261 0.0000826 0.00484 0.043 0.000516 0.0184 0.00666
3L:9342580-9342730:minus -14.2245 0.522936 0.623853 6.49541 -27.6327 -26.898 -11.6881 0.166667 7.63303
0.00126 0.00859 0.00941 0.00176 0.0369 0.0302 0.00431 0.0159 0.000928
3L:9342580-9342730:plus -12.5918 4.07339 4.12844 10.8624 -17.1122 -27.3776 -10.8349 -0.791667
2.56881 0.000449 0.00234 0.00254 0.0000687 0.00341 0.0351 0.00361 0.0212 0.00783
3L:9351200-9351350:minus -23.2245 -0.394495 6.51376 5.31193 -17.1837 -8.68367 -18.9633 -1.65278
-0.220183 0.00576 0.0118 0.000912 0.00393 0.00366 0.00157 0.0173 0.0271 0.0201
3L:9351200-9351350:plus -31.551 3.79817 1.77064 3.62385 -17.449 -26.8265 -7.34862 1.54167 10.0367
0.0134 0.00259 0.00629 0.00673 0.00484 0.0285 0.00183 0.0101 0.000146
3L:9353380-9353530:minus -33.5918 4.58716 -4.33028 3.76147 -29.0918 0.72449 -24.3119 7.59722 -0.220183
0.0289 0.00192 0.0415 0.00645 0.0653 0.000175 0.0429 0.000731 0.0201
3L:9353380-9353530:plus -23.3776 -0.0458716 -3.2844 1.10092 -17.2245 -19.5714 -22.2752 8.55556
5.84404 0.00619 0.0105 0.0312 0.02 0.00384 0.0202 0.031 0.000422 0.00202
3L:9360040-9360190:minus -23.4796 2.49541 0.146789 2.18349 -18.1531 -27.1939 -6.77982 6.43056 4.7156
0.00634 0.00418 0.011 0.0131 0.00829 0.0327 0.0016 0.00134 0.00345
3L:9360040-9360190:plus -15.0102 3.23853 1.11927 3.9633 -17.0816 -7.27551 -21.8716 0.208333 -0.366972
0.00162 0.00318 0.00793 0.0059 0.00334 0.000561 0.0288 0.0157 0.021
3L:9360420-9360570:minus -42.1531 -0.678899 -0.275229 1.57798 -19.2755 -8.94898 -22.5688 3.27778
-3.08257 0.0701 0.0129 0.0127 0.0171 0.0164 0.00203 0.0326 0.00536 0.0455
3L:9360420-9360570:plus -30.4388 8.05505 2.88073 3.11927 -8.60204 -17.2755 -13.2018 6.45833 3.46789
0.00923 0.000371 0.00415 0.00858 0.00125 0.00544 0.00605 0.00133 0.00582
3L:9360700-9360850:minus -21.6327 -3.79817 -0.321101 5.90826 -18.3776 -8.93878 -21.6972 2.20833
1.06422 0.00215 0.032 0.0129 0.00271 0.00926 0.00201 0.0279 0.008 0.014
3L:9360700-9360850:plus -31.7449 -2.61468 -1.55046 2.55963 -28.2653 -18.9286 -14.7523 4.69444 3.98165
0.0152 0.0232 0.0189 0.0111 0.0537 0.0153 0.00853 0.003 0.00439
3L:9364140-9364290:minus -32.4694 -3.69725 6.88073 6.33028 -17.4184 -8.72449 -18.6881 1 -6.37615
0.0196 0.0312 0.000768 0.00198 0.00457 0.00169 0.0166 0.0121 0.0975
3L:9364140-9364290:plus -33.2653 -2.41284 2.08257 1 -27.5714 1.05102 -34.7615 5.84722 -4.84404
0.0277 0.0219 0.0056 0.0206 0.0357 0.000107 0.157 0.00178 0.0701
3L:9365840-9365990:minus -31.7347 -2.14679 4.04587 5.47706 -26.2653 -19.2347 -15.055 2.29167 -2.27523
0.0149 0.0203 0.00263 0.0036 0.018 0.018 0.00907 0.00776 0.036
3L:9365840-9365990:plus -34.4082 -1.55046 9.43119 10.5872 -27.2347 -9.23469 -6.21101 5.19444 -4.99083

0.0319 0.017 0.000198 0.000115 0.0283 0.00238 0.00139 0.00241 0.0726
3L:9366700-9366850:minus -23.7857 2.72477 -1.02752 5.06422 -37.3061 1.16327 -36.9725 2.43056 -2.7156
0.00679 0.00385 0.0161 0.00433 0.128 0.0000831 0.195 0.00738 0.0412
3L:9366700-9366850:plus -30.7041 -0.651376 -2.62385 0.853211 -8.37755 -18.3469 -19.1193 3.38889
7.22018 0.00993 0.0128 0.0259 0.0214 0.00106 0.0124 0.0178 0.00513 0.00119
3L:9370720-9370870:minus -31.0816 1.59633 0.513761 6.00917 -28.4898 0.0510204 -16.1468 3.70833
-0.247706 0.0106 0.00582 0.00977 0.00255 0.0581 0.000349 0.0111 0.00452 0.0204
3L:9370720-9370870:plus -22.5204 -2.47706 4.79817 9.3211 -27.1633 9.86735 -13.6514 -0.263889
-0.486239 0.00356 0.0223 0.00193 0.000391 0.025 0.0000132 0.00671 0.0181 0.0219
3L:9373160-9373310:minus -33.0408 3.75229 -0.651376 2.09174 -18.449 -7.79592 -23.1743 8.51389
1.66055 0.0264 0.00264 0.0143 0.0137 0.0107 0.000723 0.0361 0.000433 0.011
3L:9373160-9373310:plus -30.551 -5.78899 13.5046 -0.522936 -26.6735 -28.3776 -18.6972 5.91667
1.11927 0.00959 0.0541 0.000013 0.0313 0.0191 0.0609 0.0166 0.00172 0.0137
3L:9374720-9374870:minus -24.4082 7.74312 -2.99083 2.78899 -27.1939 -26.6735 -12.2202 4.38889 4.13761
0.00797 0.000438 0.0288 0.00992 0.0267 0.0256 0.00484 0.00342 0.00408
3L:9374720-9374870:plus 14.5816 5.23853 5.50459 10.5872 -26.9286 -9.30612 -14.0642 13.0694 5.18349
1.17e-06 0.00148 0.00144 0.000115 0.0214 0.00257 0.00736 9.55e-06 0.00295
3L:9376780-9376930:minus -24.7449 3.73394 -0.0917431 4.34862 -18.7551 -7.57143 -17.0092 9.94444
-4.69725 0.00827 0.00266 0.0119 0.00507 0.0144 0.000644 0.0128 0.000171 0.0673
3L:9376780-9376930:plus -32.9286 -2.20183 -0.541284 7.33945 -18.3878 -9.37755 -2.69725 15.8611
-0.651376 0.025 0.0206 0.0138 0.00102 0.00931 0.00265 0.000526 5.15e-08 0.0235
3L:9392240-9392390:minus -23.2245 1.08257 2.37615 7.27523 20.4694 -18.1224 -14.945 -0.958333
0.137615 0.00576 0.00703 0.00503 0.00109 1.65e-07 0.00988 0.00887 0.0223 0.0178
3L:9392240-9392390:plus -31.6327 0.541284 -2.74312 0.752294 0.877551 0.0510204 -20.6147 1.77778
-4.59633 0.0138 0.00854 0.0268 0.0221 0.00019 0.000349 0.0229 0.00932 0.0653
3L:9394300-9394450:minus -31.7755 -2.7156 4.33028 3.27523 -17.1122 -38.4796 -3.95413 4.41667 5.90826
0.0157 0.0238 0.00235 0.00796 0.00341 0.195 0.000736 0.00338 0.00198
3L:9394300-9394450:plus -13.6735 3.05505 1.77064 1.69725 -27.7959 -8.42857 -7.76147 -3.18056 11.3853
0.00103 0.00341 0.00629 0.0163 0.0386 0.00104 0.002 0.0404 0.0000815
3L:9395020-9395170:minus -12.1122 -1.62385 2.30275 7.16514 -27.2653 -17.5 -20.945 5.375 5.70642
0.000336 0.0174 0.00517 0.00116 0.0302 0.00599 0.0243 0.00222 0.00222
3L:9395020-9395170:plus -23.3367 -1.9633 -2.48624 -0.275229 -8.67347 -18.051 -21.4587 -1.77778
11.8532 0.00614 0.0192 0.0249 0.029 0.00154 0.00886 0.0267 0.0281 0.0000428
3L:9395460-9395610:minus -21.6327 14.1101 2.63303 8.05505 10.9184 -8.53061 -19.9817 -3.86111 -4.45872
0.00215 7.46e-06 0.00457 0.000578 4.19e-06 0.00136 0.0205 0.0477 0.0633
3L:9395460-9395610:plus -13.449 14.8532 2.50459 3.77064 -17.1939 -27.9694 -5.75229 -2.13889 4.19266
0.000914 2.86e-06 0.0048 0.00637 0.00377 0.0547 0.00123 0.0309 0.00401
3L:9403920-9404070:minus -23 0.926606 4.33945 4.05505 10.6531 -8.72449 -9.77982 1.22222 -3.07339
0.00498 0.00743 0.00234 0.00566 0.0000102 0.00169 0.00295 0.0113 0.0454
3L:9403920-9404070:plus -24.1837 3.68367 0.853211 5.86239 -27.2347 -0.173469 -5.27523 -3.26389
-2.48624 0.00751 0.00144 0.0087 0.00282 0.0283 0.000456 0.00107 0.0413 0.0387
3L:9413280-9413430:minus -23.9184 2.02752 -0.0550459 7 -8.56122 -26.4898 -12.0459 1.375 -3.87156
0.00695 0.00497 0.0118 0.00132 0.00112 0.0225 0.00466 0.0107 0.0537
3L:9413280-9413430:plus -24.2245 2.22936 2.62385 6.57798 -18.3776 -16.5 -7.22936 4.15278 -2.01835
0.00763 0.00461 0.00458 0.00167 0.00926 0.00416 0.00178 0.00377 0.0334
3L:9416300-9416450:minus -33 5.99083 4.82569 1.69725 -9.16327 -18.5714 -15.7248 3.73611 -1.22018
0.0258 0.00106 0.00191 0.0163 0.00249 0.0136 0.0103 0.00447 0.0278
3L:9416300-9416450:plus 15 4.63303 4.09174 9.24771 -18.7143 -17.7551 -14.945 5.30556 7.24771
5.44e-07 0.00189 0.00258 0.000409 0.0136 0.00643 0.00887 0.00229 0.00116
3L:9419940-9420090:minus -32.0714 13.4592 -0.0275229 3.77064 -27.8571 -35.3367 -12.2661 -1.65278
-0.642202 0.0176 0.000064 0.0117 0.00637 0.039 0.0764 0.00489 0.0271 0.0234
3L:9419940-9420090:plus -31.4796 -1.84404 7.33945 3.33945 2.14286 -18.3469 -16.9633 0.305556 3.09174
0.0128 0.0186 0.000614 0.00774 0.0000316 0.0124 0.0127 0.0152 0.00678
3L:9422840-9422990:minus -31.1429 0.816514 0.155963 2.45872 10.6531 10.3061 -21.7798 0.611111 -0.917431

0.0108 0.00773 0.011 0.0116 0.0000102 7.25e-06 0.0283 0.0138 0.0256
3L:9422840-9422990:plus -22.7449 2.44037 -4.40367 -0.146789 -27.5612 -19.6429 -17.3853 1.36111
1.2844 0.00404 0.00427 0.0423 0.028 0.0354 0.0204 0.0136 0.0108 0.0129
3L:9423380-9423530:minus -22 -1.36697 1.15596 1.16514 -27.4592 -17.6429 -14.5321 4.125 1.17431
0.00291 0.0161 0.00783 0.0197 0.0329 0.00619 0.00814 0.00382 0.0134
3L:9423380-9423530:plus -31.1837 6.00917 0.0366972 6.21101 20.2041 -8.53061 -22.7798 0.0138889
1.59633 0.0109 0.00105 0.0115 0.00213 3.64e-07 0.00136 0.0338 0.0167 0.0113
3L:9428460-9428610:minus -22.6327 2.45872 0.155963 4.02752 -17.7449 -18.3469 5.01835 1.08333 4.92661
0.00373 0.00424 0.011 0.00581 0.0063 0.0124 0.0000572 0.0118 0.00318
3L:9428460-9428610:plus -22.2653 1.75229 14.0917 3.80734 -8.93878 -8.42857 -22.4312 0.347222 -1.79817
0.00331 0.0055 6.39e-06 0.00627 0.00216 0.00104 0.0318 0.015 0.0315
3L:9435300-9435450:minus -22.3571 -0.110092 -1.3578 4.31193 -9.23469 -8.65306 -13.4312 1.81944
1.90826 0.00339 0.0107 0.0178 0.00516 0.00277 0.00156 0.00638 0.00919 0.00974
3L:9435300-9435450:plus -32.5918 3.90826 4.94495 5.94495 -17.7143 -0.469388 -24.9083 3.51389
-1.95413 0.021 0.00249 0.00182 0.00261 0.00627 0.000533 0.0466 0.00489 0.0328
3L:9435940-9436090:minus -24.3367 1.91743 -0.238532 1.46789 -18.4898 -8.7551 -20.3028 -2.30556
4.78899 0.00789 0.00518 0.0125 0.0179 0.0116 0.00172 0.0217 0.0323 0.00336
3L:9435940-9436090:plus -33.0408 1.44037 2.0367 4.08257 -18.7857 -8.79592 -21.6514 -0.194444
6.89908 0.0264 0.00617 0.0057 0.00558 0.0148 0.0019 0.0277 0.0178 0.00137
3L:9436180-9436330:minus -33.4898 -2.30275 2.89908 1.41284 0.27551 0.755102 -18.2936 3.15278 0.816514
0.0286 0.0212 0.00413 0.0183 0.00035 0.000175 0.0156 0.00563 0.015
3L:9436180-9436330:plus -33.2245 -0.486239 -2.13761 10.0275 -18.3776 -10.0918 -30.1651 8.11111
-4.66055 0.0276 0.0121 0.0225 0.000156 0.00926 0.00404 0.0931 0.000547 0.0667
3L:9439840-9439990:minus -22.7347 1.97248 1.74312 8.61468 -18.5204 -7.23469 -12.4771 7.375 -0.944954
0.00403 0.00507 0.00635 0.000552 0.0117 0.000557 0.00513 0.000825 0.0257
3L:9439840-9439990:plus -31.6633 2.19266 4.45872 2.31193 10.7245 10.8265 -17.4954 0.486111 -2.05505
0.0141 0.00468 0.00223 0.0123 5.2e-06 2.5e-06 0.0138 0.0144 0.0338
3L:9440320-9440470:minus -31.3367 -2.82569 2.9633 -0.165138 -18.1531 -18.8265 -17.5321 1.01389
9.04587 0.0114 0.0246 0.00403 0.0282 0.00829 0.0148 0.0139 0.0121 0.000423
3L:9440320-9440470:plus -12.4796 6.04587 4.07339 6.74312 -17.4184 -26.2653 -18.3486 4.47222 1.95413
0.000433 0.00104 0.0026 0.0015 0.00457 0.0218 0.0158 0.0033 0.00964
3L:9440700-9440850:minus -31.1429 3.86239 0.651376 3.24771 -18.7449 -18.1224 -5.54128 8.5 9.45872
0.0108 0.00253 0.00932 0.00808 0.0138 0.00988 0.00116 0.000436 0.000337
3L:9440700-9440850:plus -21.1939 -1.79817 -0.33945 4.23853 -16.7143 0.163265 -19.8991 4 -3.3945
0.00189 0.0183 0.0129 0.00522 0.00319 0.000307 0.0202 0.00402 0.0484
3L:9444840-9444990:minus -22.3367 7.38532 3.33028 1.0367 -27.3061 -17.7857 -12.4679 1.04167 0.752294
0.00339 0.000529 0.00349 0.0204 0.0315 0.0068 0.00512 0.012 0.0152
3L:9444840-9444990:plus -22.1837 14.7523 -1.57798 4.74312 -17.4184 -19.1939 -18.3486 3.86111 3.88073
0.00315 3.48e-06 0.019 0.00462 0.00457 0.0178 0.0158 0.00425 0.00475
3L:9448660-9448810:minus -22.3673 -3.08257 0.53211 4.33945 -8.40816 -27.6327 -12.0092 -5.29167 6.33028
0.00341 0.0264 0.00971 0.00509 0.00108 0.043 0.00462 0.0657 0.0016
3L:9448660-9448810:plus -31.1837 -5.41284 -3.90826 1.83486 -17.0816 -8.5 -8.33945 4.34722 7.36697
0.0109 0.0491 0.037 0.0155 0.00334 0.00126 0.00225 0.00348 0.00108
3L:9449240-9449390:minus -13.449 0.46789 -2.11009 -0.743119 -17.4592 -27.602 -0.93578 -4.02778
8.10092 0.000914 0.00876 0.0223 0.0339 0.00491 0.0416 0.00035 0.0496 0.000623
3L:9449240-9449390:plus -32 -0.394495 3.59633 0.0275229 -18.449 -0.469388 -22.4679 2.625
-2.15596 0.0171 0.0118 0.00315 0.0267 0.0107 0.000533 0.032 0.00687 0.0348
3L:9451020-9451170:minus -21.7041 -4.50459 14.6147 2.01835 -18.1531 -27.3367 -16.0092 1.58333 8.0367
0.00231 0.0387 3.32e-06 0.0141 0.00829 0.0347 0.0109 0.00997 0.000654
3L:9451020-9451170:plus -30.4082 2.57798 4.78899 -0.440367 -18.3469 -18.8265 -21.5872 1.69444
7.31193 0.00908 0.00406 0.00194 0.0306 0.00894 0.0148 0.0274 0.0096 0.00112
3L:9453840-9453990:minus -13.4898 9.33945 1.43119 3.54128 -18.449 0.0510204 -19.1284 7.30556
1.27523 0.000946 0.000188 0.0071 0.00702 0.0107 0.000349 0.0178 0.000857 0.013
3L:9453840-9453990:plus -23.1429 -0.192661 -0.963303 1.75229 -17.4184 -26.6735 -14.2752 -0.638889

3.09174 0.00533 0.011 0.0158 0.0159 0.00457 0.0256 0.00771 0.0203 0.00678
3L:9454100-9454250:minus -13.4898 9.33945 1.43119 3.54128 -18.449 -18.1224 -19.1284 7.30556 1.27523
0.000946 0.000188 0.0071 0.00702 0.0107 0.00988 0.0178 0.000857 0.013
3L:9454100-9454250:plus -13.3367 3.84404 9.09174 10.7615 -27.1224 -8.53061 -14.9633 -3.40278 -2.48624
0.0008 0.00255 0.000241 0.0000826 0.0245 0.00136 0.00891 0.0427 0.0387
3L:9455300-9455450:minus -24.2245 2.34862 -0.284404 -0.412844 -17.6429 -8.20408 -13.7706 8.23611
-3.26606 0.00763 0.00441 0.0127 0.0304 0.00562 0.000796 0.00689 0.00051 0.0472
3L:9455300-9455450:plus -14.6327 0.715596 7.79817 -0.981651 -8.92857 -26.4898 -7.84404 1.36111
10.3394 0.0015 0.00802 0.000487 0.0365 0.00194 0.0225 0.00204 0.0108 0.000112
3L:9470060-9470210:minus -21.9286 -1.80734 5.42202 6.74312 -7.60204 -9.37755 -21.4037 0.916667 2.11009
0.0027 0.0184 0.00149 0.0015 0.000466 0.00265 0.0265 0.0125 0.00898
3L:9470060-9470210:plus -32.5408 -2.3945 1.86239 1.06422 -18.4898 -36.449 -20.2294 0.375 3.72477
0.0204 0.0218 0.00608 0.0203 0.0116 0.109 0.0214 0.0149 0.00497
3L:9470840-9470990:minus -31.0612 0.568807 2.55963 2.26606 -25.9286 -26.8571 -15.6239 -5.83333 -6.01835
0.0106 0.00845 0.0047 0.0126 0.0168 0.0288 0.0101 0.0736 0.0892
3L:9470840-9470990:plus -13.0816 2.55963 3.33945 6.25688 -7.71429 -9.42857 -5.24771 4.18056 5.86239
0.000599 0.00409 0.00348 0.00208 0.000572 0.0027 0.00107 0.00373 0.00201
3L:9481000-9481150:minus -31.7347 2.12844 5.45872 4.31193 -18.7143 -9.53061 -14.5596 4.08333 -0.697248
0.0149 0.00479 0.00147 0.00516 0.0136 0.0031 0.00819 0.00388 0.0239
3L:9481000-9481150:plus -32.7449 2.69725 0.165138 3.94495 -18.051 -8.20408 -21.2018 5.98611 3.88991
0.0228 0.00389 0.011 0.00595 0.00746 0.000796 0.0255 0.00167 0.00467
3L:9490800-9490950:minus -32.8061 1.91743 3.53211 9.23853 1.21429 -16.2041 -9.40367 -1.36111 0.752294
0.0237 0.00518 0.00323 0.000424 0.0000864 0.00408 0.00276 0.025 0.0152
3L:9490800-9490950:plus -31 5.01835 0.834862 6.72477 -18.4898 -17.0102 -4.44037 2.69444 1.48624
0.0105 0.00162 0.00875 0.00154 0.0116 0.00453 0.000846 0.00669 0.0118
3L:9495520-9495670:minus -4.67347 3.90816 2.99083 4.3211 -18.4082 -18.898 -5.84404 5.63889 1.90826
0.000182 0.00102 0.00399 0.00514 0.00937 0.0152 0.00126 0.00196 0.00974
3L:9495520-9495670:plus -31.0102 5.04587 5.08257 3.79817 -8.86735 -9.53061 -9.84404 0.208333 1.33028
0.0105 0.0016 0.00172 0.00629 0.00173 0.0031 0.00299 0.0157 0.0127
3L:9498300-9498450:minus -31.7041 -2.78899 1.94495 7.51376 -17.8163 1.02041 -8.55046 7.68056 0.917431
0.0146 0.0243 0.0059 0.000836 0.00682 0.000118 0.00235 0.000698 0.0146
3L:9498300-9498450:plus -31.2857 0 1.30275 2.11927 -27.0408 -10.0204 -4.76147 8.83333 11.3853
0.0113 0.0103 0.00743 0.0135 0.0242 0.00399 0.000927 0.000356 0.0000815
3L:9502320-9502470:minus -20.6735 2.37615 2.81651 2.97248 -26.9286 -9.42857 -17.6789 6.43056 9.92661
0.00173 0.00437 0.00426 0.00909 0.0214 0.0027 0.0142 0.00134 0.000193
3L:9502320-9502470:plus -22.1122 -1.3578 3.23853 9.61468 -17.4898 -17.5 -14.211 -1.23611 8.11009
0.00304 0.016 0.00362 0.000255 0.0053 0.00599 0.0076 0.0241 0.000597
3L:9502620-9502770:minus -4 -1.07339 1.84404 2.58716 -18.4082 -18.9286 1.24771 1.68056 2.54128
0.000121 0.0147 0.00612 0.011 0.00937 0.0153 0.000206 0.00964 0.00787
3L:9502620-9502770:plus -22.3776 13.4592 1.73394 3.02752 10.6531 -8.94898 -23.9541 6.26389 1.46789
0.00342 0.000064 0.00638 0.00887 0.0000102 0.00203 0.0408 0.00146 0.0119
3L:9504620-9504770:minus -33.0306 -2.22018 -1.93578 1.49541 19.9082 -17.1633 -8.12844 3.26389 -0.229358
0.026 0.0207 0.0212 0.0176 8.13e-07 0.005 0.00216 0.00539 0.0202
3L:9504620-9504770:plus -31.0714 8.02752 0.724771 1.74312 10.6939 -18.4184 -24.5413 0.291667 -4.87156
0.0106 0.000376 0.00909 0.016 7.35e-06 0.0127 0.0443 0.0153 0.0708
3L:9540200-9540350:minus -22.1122 -1.20183 2.90826 1.78899 -17.8163 -18.9796 -22.3945 -0.375 4.04587
0.00304 0.0153 0.00411 0.0158 0.00682 0.0155 0.0316 0.0188 0.00421
3L:9540200-9540350:plus -31.7041 -2.27523 7.87156 7.37615 -7.93878 -9.16327 -4.14679 -3.72222 -0.761468
0.0146 0.021 0.000469 0.000951 0.000767 0.00223 0.000778 0.0461 0.0244
3L:9545220-9545370:minus -30.3265 3.95413 0.247706 3.76147 -27.1939 9.60204 -26.789 7.22222 -2.6789
0.0089 0.00244 0.0107 0.00645 0.0267 0.0000248 0.0589 0.000895 0.0408
3L:9545220-9545370:plus -34.3776 0.880734 5.40367 8.61468 -8.23469 -17.0816 -16.6697 4.56944 5.76147
0.0319 0.00756 0.0015 0.000552 0.000927 0.00487 0.0122 0.00317 0.0021
3L:9545740-9545890:minus -22.8163 -0.917431 1.6422 2.19266 -8.67347 -27.2653 -12.3945 1.31944

5.34862 0.00418 0.014 0.00659 0.0131 0.00154 0.0333 0.00504 0.0109 0.00271
3L:9545740-9545890:plus -32.6939 -0.183486 4.97248 6.00917 -8.67347 -27.898 -6.50459 4.375
1.27523 0.0217 0.011 0.0018 0.00255 0.00154 0.0537 0.00149 0.00344 0.013
3L:9608920-9609070:minus -23.6224 -0.825688 0.522936 5.55046 -8.90816 -18.1224 -18.5046 10.7639
0.238532 0.00658 0.0136 0.00974 0.00345 0.00192 0.00988 0.0161 0.0000924 0.0173
3L:9608920-9609070:plus -20.6224 -0.165138 -1.73394 7.34862 -18.4592 -8.53061 -11.0275 3.41667
1.77982 0.0017 0.0109 0.02 0.000998 0.0109 0.00136 0.00375 0.00508 0.0103
3L:9609740-9609890:minus -32.9694 -1.57798 -2.36697 2.18349 -17.449 0.755102 -12.6055 6.54167 -2.36697
0.0254 0.0171 0.0241 0.0131 0.00484 0.000175 0.00528 0.00127 0.0371
3L:9609740-9609890:plus -21.6633 0.440367 2.18349 4.07339 -26.1939 -17.051 -3.53211 6.98611 -1.24771
0.00223 0.00885 0.0054 0.00562 0.0175 0.00479 0.000655 0.00101 0.028
3L:9617280-9617430:minus -22.551 -1.88073 -4.0367 5.75229 -27.1939 -7.7551 -17.7064 5.97222 3.29358
0.00359 0.0188 0.0384 0.00296 0.0267 0.000682 0.0143 0.00168 0.00623
3L:9617280-9617430:plus -30.9286 5.69725 2.76147 6.00917 -18.4184 -8.68367 -14.0092 1.31944 -3.77982
0.0104 0.00121 0.00435 0.00255 0.0101 0.00157 0.00727 0.0109 0.0525
3L:9623040-9623190:minus -33.4796 1.22018 2.98165 1.92661 -17.9796 -8.57143 -27.6514 7.16667 -5.27523
0.0285 0.00668 0.004 0.0148 0.00726 0.00149 0.0657 0.000922 0.0771
3L:9623040-9623190:plus -40.2245 2.20183 3.6055 3.88991 10.6939 -17.7551 -20.5505 -3.76389 -4.41284
0.0374 0.00466 0.00314 0.00608 7.35e-06 0.00643 0.0227 0.0466 0.0624
3L:9654680-9654830:minus -23.0306 8.44954 -0.275229 4.05505 -18.4592 -8.7551 -12.4771 6.27778
-0.192661 0.00505 0.000299 0.0127 0.00566 0.0109 0.00172 0.00513 0.00145 0.0198
3L:9654680-9654830:plus -31.1429 -3.3945 5.95413 2.55963 -7.67347 -27.9694 -5.80734 2.45833 3.29358
0.0108 0.0287 0.00118 0.0111 0.00055 0.0547 0.00124 0.00731 0.00623
3L:9659980-9660130:minus -33 3.81651 4.11009 9.3211 -18.7143 1.02041 -20.789 4.06944 -0.192661
0.0258 0.00258 0.00256 0.000391 0.0136 0.000118 0.0237 0.00391 0.0198
3L:9659980-9660130:plus -32.4796 -2.45872 0.963303 5.77982 -18.1531 -9.86735 -7.62385 3.15278 -4.25688
0.02 0.0222 0.00837 0.00294 0.00829 0.00391 0.00194 0.00563 0.0593
3L:9665480-9665630:minus -13.449 5.11009 7.06422 5.7156 -17.7551 -18.3469 -14.9541 1.58333 -0.697248
0.000914 0.00156 0.000706 0.00309 0.00658 0.0124 0.00889 0.00997 0.0239
3L:9665480-9665630:plus -32.7347 3.92661 3.45872 1.43119 -18.4184 -9.57143 -15.1193 -1.59722 -3.87156
0.0225 0.00247 0.00332 0.0181 0.0101 0.00326 0.00919 0.0267 0.0537
3L:9666480-9666630:minus -13.0816 0.201835 1.79817 3.6055 -18.8265 -26.7551 -10.2294 7.27778 4.44037
0.000599 0.00962 0.00623 0.00686 0.0149 0.0263 0.00321 0.000869 0.00367
3L:9666480-9666630:plus -31.4388 0.449541 5.92661 2.99083 -8.56122 -28.1224 -8.6055 4.27778 -5.08257
0.0122 0.00882 0.00119 0.00899 0.00112 0.0583 0.00237 0.00358 0.0741
3L:9668740-9668890:minus -3.22449 1.48624 -1.51376 7.80734 -27.1224 1.38776 -24.7615 -0.388889
-5.58716 0.0000495 0.00606 0.0187 0.00063 0.0245 0.0000559 0.0457 0.0188 0.0817
3L:9668740-9668890:plus -21.5102 -0.59633 16.3945 7.29358 -27.6735 -17.2755 3.61468 -2.54167 12.2752
0.00202 0.0126 6.75e-08 0.00104 0.0375 0.00544 0.0000949 0.0344 0.0000141
3L:9671440-9671590:minus -21.2959 -3.22018 1.27523 0.119266 -18.1531 -8.5 -6.33945 0.777778 -0.550459
0.00192 0.0274 0.0075 0.026 0.00829 0.00126 0.00143 0.0131 0.0225
3L:9671440-9671590:plus -31.3265 3.69725 4.6789 1.33028 -17.4592 -28.7041 -11.1651 1.48611 10.0367
0.0114 0.00269 0.00203 0.0188 0.00491 0.067 0.00386 0.0103 0.000146
3L:9672200-9672350:minus -30.6633 -1.16514 12.4862 7.29358 -9.27551 -10.0204 -7.6055 2.38889 -5.31193
0.00976 0.0151 0.0000304 0.00104 0.00284 0.00399 0.00194 0.00749 0.0776
3L:9672200-9672350:plus -38.9592 0.825688 13.5872 7.45872 -17.449 -8.5 -18.6972 -3.06944 -7.44037
0.0324 0.00771 0.0000122 0.000894 0.00484 0.00126 0.0166 0.0393 0.123
3L:9684600-9684750:minus -24.2245 2.07339 -2.07339 6.00917 -28.3061 -8.02041 -24.2752 3.15278 -2.85321
0.00763 0.00488 0.0221 0.00255 0.0553 0.000761 0.0427 0.00563 0.0429
3L:9684600-9684750:plus -23.1531 1.43119 -1.0367 4.08257 -7.89796 -17.7551 -10.6972 0.513889 8.62385
0.00542 0.00619 0.0161 0.00558 0.000678 0.00643 0.00352 0.0142 0.00048
3L:9686420-9686570:minus -21.5102 -1.00917 -1.38532 -0.119266 -18.0918 -27.6735 5.92661 3.80556
3.66972 0.00202 0.0144 0.018 0.0278 0.00761 0.0443 0.0000424 0.00435 0.00514
3L:9686420-9686570:plus -31.5204 -0.440367 2.51376 -1.48624 -17.7857 -0.173469 -19.7523 0.111111

5.75229 0.0131 0.012 0.00478 0.0431 0.00676 0.000456 0.0197 0.0162 0.00213
3L:9687580-9687730:minus -39.2143 4.51376 -0.192661 5.57798 -17.0816 -19.1224 -23.055 2.59722
-0.12844 0.0329 0.00198 0.0123 0.0034 0.00334 0.0175 0.0354 0.00694 0.0193
3L:9687580-9687730:plus -14.0408 14.1284 1.41284 2.88991 -27 -18.2755 -12.8716 3.22222 -0.33945
0.00115 7.2e-06 0.00714 0.00942 0.0233 0.0112 0.00561 0.00548 0.0209
3L:9688540-9688690:minus -20.8878 -0.431193 4.14679 9.61468 -18.1122 -16.051 -13.5046 -2.16667
-0.440367 0.0018 0.0119 0.00252 0.000255 0.00771 0.00408 0.00649 0.0312 0.0216
3L:9688540-9688690:plus -14.4796 3.97959 14.9633 7.29358 -16.1531 -17.4286 -16.2202 -3.73611
-0.00917431 0.00142 0.000669 1.77e-06 0.00104 0.00297 0.00586 0.0113 0.0463 0.0184
3L:9690000-9690150:minus -31.6633 -0.779817 0.211009 6.12844 -18.4082 -18.0102 -10.211 2.45833
3.72477 0.0141 0.0134 0.0108 0.00231 0.00937 0.00812 0.0032 0.00731 0.00497
3L:9690000-9690150:plus -24.0714 -0.880734 6.76147 1.77982 -27.5714 10.5306 -19.9358 1.19444
0.568807 0.00731 0.0138 0.000811 0.0158 0.0357 5.67e-06 0.0204 0.0114 0.016
3L:9691320-9691470:minus -33.1122 0.614679 3.97248 3.11009 -27.0102 -9.86735 -16.1468 4.33333 9.45872
0.0271 0.00832 0.00271 0.00862 0.0237 0.00391 0.0111 0.0035 0.000337
3L:9691320-9691470:plus -31.4796 2.75229 0.587156 3.44954 20.1735 -9.53061 -11.4495 0.722222 3.46789
0.0128 0.00381 0.00952 0.00738 5.68e-07 0.0031 0.00409 0.0133 0.00582
3L:9697360-9697510:minus -42.8571 -0.0917431 -2 -2.11927 -27.602 -16.2041 -17.5413 5.91667 3.46789
0.0873 0.0106 0.0216 0.0532 0.0364 0.00408 0.0139 0.00172 0.00582
3L:9697360-9697510:plus -39.2245 3.10092 -0.155963 5.42202 -18.9796 -9.68367 -20.7431 1.79167
3.41284 0.0329 0.00335 0.0122 0.00374 0.0155 0.00342 0.0235 0.00928 0.006
3L:9706880-9707030:minus -33.0306 -0.550459 2.22018 3.44954 1.87755 -8.5 -17.8991 2.19444 3.45872
0.026 0.0124 0.00532 0.00738 0.0000492 0.00126 0.0147 0.00804 0.00591
3L:9706880-9707030:plus -41.8469 0.412844 -4.0367 2.94495 -9.23469 -8.57143 -24.6972 7.80556 -6.56881
0.0665 0.00893 0.0384 0.0092 0.00277 0.00149 0.0453 0.000651 0.103
3L:9707700-9707850:minus -23.0408 -2.0367 -1.55046 6.12844 -17.449 -17.7143 -18.4587 0.833333 -0.12844
0.00512 0.0196 0.0189 0.00231 0.00484 0.00634 0.016 0.0128 0.0193
3L:9707700-9707850:plus -40.9592 4.25688 -1.22936 5.86239 -26.9694 -17.5714 -28.3853 5.41667 1.33028
0.0449 0.00218 0.0171 0.00282 0.023 0.00616 0.0725 0.00218 0.0127
3L:9709780-9709930:minus -31.7041 5.55963 3.24771 6.00917 -9.16327 -26.4898 -28.7982 8.61111 0.146789
0.0146 0.00129 0.00361 0.00255 0.00249 0.0225 0.0767 0.000408 0.0178
3L:9709780-9709930:plus -31.3265 2.2844 0.486239 3.44037 -17.7551 -26.602 -6.57798 1.72222 7.72477
0.0114 0.00452 0.00986 0.00746 0.00658 0.0248 0.00152 0.0095 0.000798
3L:9712260-9712410:minus -32.7041 2.11009 1.85321 3.21101 10.6531 -0.173469 -22.3119 -2.66667
3.88991 0.0222 0.00482 0.0061 0.00822 0.0000102 0.000456 0.0312 0.0355 0.00467
3L:9712260-9712410:plus -32.8469 -1.72477 4.88073 2.3945 -19.0102 -0.244898 -10.5596 9.65278
-2.42202 0.0243 0.0179 0.00187 0.0119 0.0157 0.000496 0.00342 0.000209 0.0378
3L:9715200-9715350:minus -32.1429 -0.926606 2.6055 7 -27.9388 -26.898 -14.7706 -2.58333 -1.23853
0.0179 0.014 0.00462 0.00132 0.0415 0.0302 0.00856 0.0348 0.0279
3L:9715200-9715350:plus -20.898 -1.85321 -1.65138 2.30275 -18.1531 -19.0816 -17.8807 3.15278 1.24771
0.0018 0.0186 0.0195 0.0124 0.00829 0.017 0.0147 0.00563 0.0131
3L:9716160-9716310:minus -23.1531 -0.798165 0.137615 0.633028 11.6531 -8.45918 -20.2018 -0.777778
-2.43119 0.00542 0.0134 0.0111 0.0229 1.55e-06 0.00111 0.0213 0.0211 0.038
3L:9716160-9716310:plus -31.7347 7.09174 3.88073 3.54128 -26.898 -17.9388 -13.633 -0.0277778
0.614679 0.0149 0.000619 0.00281 0.00702 0.0203 0.00738 0.00668 0.0169 0.0158
3L:9716460-9716610:minus -23.1531 -0.798165 0.137615 2.0367 11.6531 -8.45918 -21.8991 -0.0416667
0.743119 0.00542 0.0134 0.0111 0.0141 1.55e-06 0.00111 0.029 0.017 0.0153
3L:9716460-9716610:plus -31.7347 7.09174 3.88073 4.6055 -26.898 -7.93878 -13.633 -0.0277778
0.614679 0.0149 0.000619 0.00281 0.00476 0.0203 0.000736 0.00668 0.0169 0.0158
3L:9719900-9720050:minus -24.4898 13.4592 6.59633 2.05505 -17.1531 -9.02041 -11.4037 3.81944 7.22018
0.00807 0.000064 0.000876 0.0139 0.00364 0.00213 0.00406 0.00433 0.00119
3L:9719900-9720050:plus -20.3673 13.4592 6.83486 3.40367 10.6939 -7.5 -6.59633 5.875 2.11009
0.00166 0.000064 0.000785 0.00759 7.35e-06 0.000614 0.00153 0.00176 0.00898
3L:9723020-9723170:minus -42.8878 0.733945 -0.513761 1.44037 -18.4898 -9.34694 -24.945 9.26389

-0.486239 0.088 0.00797 0.0137 0.018 0.0116 0.00261 0.0468 0.00027 0.0219
3L:9723020-9723170:plus -22.6633 2.62385 5.65138 0.550459 -17.3776 -9.53061 -19.8716 8.18056 5.70642
0.00384 0.00399 0.00135 0.0233 0.00419 0.0031 0.0201 0.000526 0.00222
3L:9753540-9753690:minus -13.1122 2.01835 0.779817 11.0183 -26.8571 -8.23469 -17.2477 3.98611 1.27523
0.000632 0.00499 0.00892 0.000052 0.0198 0.000875 0.0133 0.00404 0.013
3L:9753540-9753690:plus -14.3367 -0.449541 4.48624 7.17431 -17.6735 -9.57143 -17.1376 -2.20833
-4.98165 0.00131 0.012 0.0022 0.00115 0.00572 0.00326 0.0131 0.0315 0.0725
3L:9756600-9756750:minus -40.4388 13.4592 5.54128 2.62385 -17.3469 -10.0918 -16.0917 4.76389 10.3486
0.0401 0.000064 0.00141 0.0108 0.00394 0.00404 0.011 0.00291 0.0000988
3L:9756600-9756750:plus -14.2245 0.678899 3.68807 5.84404 -17.9286 -26.8571 -5.24771 5.44444 0.917431
0.00126 0.00813 0.00304 0.00289 0.0071 0.0288 0.00107 0.00215 0.0146
3L:9759000-9759150:minus -4.33673 1.15596 -0.256881 2.08257 -17.7551 -17.2041 -14.7706 0.361111
0.0458716 0.00014 0.00684 0.0126 0.0137 0.00658 0.00507 0.00856 0.0149 0.0182
3L:9759000-9759150:plus -29.2143 1.87156 6.02752 2.69725 -8.90816 -8.60204 -7.92661 2.81944 1.21101
0.00836 0.00526 0.00114 0.0104 0.00192 0.00151 0.00207 0.00639 0.0134
3L:9815920-9816070:minus -40.2857 -2.17431 8.04587 7.17431 -18.7551 -9.5 -15.3303 0.291667 -0.0366972
0.0385 0.0204 0.000428 0.00115 0.0144 0.00294 0.00958 0.0153 0.0187
3L:9815920-9816070:plus -14.602 -0.192661 0.59633 2.23853 -27.1939 -16.9388 -15.4771 0.208333
3.20183 0.00149 0.011 0.00949 0.0128 0.0267 0.00433 0.00985 0.0157 0.0065
3L:9827860-9828010:minus -23.1939 0.449541 4.74312 4.22018 -17.4898 -9.0102 -8.50459 0.569444 2.09174
0.00558 0.00882 0.00198 0.00531 0.0053 0.00208 0.00233 0.014 0.00904
3L:9827860-9828010:plus -32.1939 2.78899 4.37615 3.6055 -18.1122 -0.244898 -14.422 1.31944
-2.11009 0.0181 0.00376 0.0023 0.00686 0.00771 0.000496 0.00796 0.0109 0.0343
3L:9829780-9829930:minus -32.4796 13.5306 1.53211 2.70642 -19.3163 9.45918 -21.9725 2.31944 -2.94495
0.02 0.0000206 0.00685 0.0103 0.0165 0.0000356 0.0293 0.00769 0.0441
3L:9829780-9829930:plus -33 1.25688 -2.72477 5.57798 -27.4898 -17.5714 -10.9266 3.56944 7.51376
0.0258 0.00659 0.0267 0.0034 0.0336 0.00616 0.00368 0.00478 0.000984
3L:9831660-9831810:minus -24 8.49541 14.6147 5.06422 -27.2347 -17.5612 -26.0275 -4.11111 4.04587
0.00719 0.000292 3.32e-06 0.00433 0.0283 0.00604 0.0537 0.0505 0.00421
3L:9831660-9831810:plus -23.4796 5.95413 -1.65138 -1.30275 -8.67347 -17.2041 -23.1376 1.95833 0.311927
0.00634 0.00108 0.0195 0.0404 0.00154 0.00507 0.0359 0.00875 0.0169
3L:9839140-9839290:minus -40.5204 -1.88073 12.5688 -0.633028 -9.5 -17.8265 -12.7523 1.83333 5.95413
0.0413 0.0188 0.0000286 0.0327 0.00293 0.00699 0.00546 0.00914 0.00192
3L:9839140-9839290:plus -13.449 2.37615 2.86239 -0.651376 -27.9694 -0.244898 -4.87156 6.5
-0.238532 0.000914 0.00437 0.00418 0.0329 0.0437 0.000496 0.000957 0.0013 0.0203
3L:9840160-9840310:minus -21.6224 -4.11927 10.8073 6.19266 -26.7449 -8.20408 -13.7431 0.763889 4.6055
0.00213 0.0349 0.0000896 0.00216 0.0195 0.000796 0.00685 0.0131 0.00352
3L:9840160-9840310:plus -23.3265 6.49541 -0.93578 4.05505 -26.9286 -8.72449 -12.8073 5.08333 5.80734
0.00607 0.000837 0.0156 0.00566 0.0214 0.00169 0.00553 0.00253 0.00205
3L:9840740-9840890:minus -24.5204 3.90816 -3.01835 4.08257 -27.0408 -27.1939 -12.4954 9.34722 1.86239
0.00813 0.00102 0.029 0.00558 0.0242 0.0327 0.00515 0.000256 0.00994
3L:9840740-9840890:plus -32.0306 -0.706422 -0.522936 1.68807 -18.2245 -9.02041 -9.82569 2.75
1.53211 0.0172 0.0131 0.0137 0.0164 0.00877 0.00213 0.00298 0.00656 0.0116
3L:9841160-9841310:minus -30.7143 -1.12844 2.75229 2.72477 -18.4184 -9.57143 -13.9266 10.8333 -2.72477
0.00993 0.0149 0.00436 0.0102 0.0101 0.00326 0.00714 0.0000874 0.0414
3L:9841160-9841310:plus -31.8163 -0.844037 6.30275 3.70642 -17.449 -18.0816 -6.2844 5.76389
2.51376 0.016 0.0136 0.00101 0.0066 0.00484 0.00934 0.00141 0.00185 0.00792
3L:9842120-9842270:minus -30.398 -2.16514 4.7156 6.84404 -27.4592 0.795918 -25.5229 1.13889 -1.06422
0.00901 0.0204 0.002 0.0014 0.0329 0.000155 0.0504 0.0116 0.0266
3L:9842120-9842270:plus -22.4796 -1.50459 3.81651 2.63303 -18.0816 -16.7143 -3.17431 4.13889 6.6422
0.00352 0.0168 0.00289 0.0107 0.00759 0.00419 0.000595 0.0038 0.00148
3L:9846400-9846550:minus -30.8878 5.47706 0.12844 2.25688 -27.5714 -19.1224 -20.5413 4.58333 2.88991
0.0103 0.00133 0.0111 0.0127 0.0357 0.0175 0.0226 0.00315 0.00725
3L:9846400-9846550:plus -23.6327 -1.78899 4.55963 7.21101 -27.0408 -27.602 -13.055 1.63889 9.97248

0.00661 0.0183 0.00214 0.0011 0.0242 0.0416 0.00585 0.00978 0.00016
3L:9847160-9847310:minus -22.2245 3.6422 -0.458716 4.04587 -18.4898 1.34694 -18.8073 1.41667 -2
0.00325 0.00275 0.0135 0.00572 0.0116 0.0000568 0.0169 0.0106 0.0332
3L:9847160-9847310:plus -31.1735 3.17431 6.63303 6.72477 -17.4184 -19.3061 -15.3578 0.0972222 -1
0.0109 0.00326 0.000861 0.00154 0.00457 0.0187 0.00963 0.0162 0.0261
3L:9847820-9847970:minus -22.2857 -3.18349 -1.6789 1.84404 -26.8571 -19.0816 -3.07339 3.04167 7.6422
0.00331 0.0271 0.0196 0.0154 0.0198 0.017 0.000579 0.00587 0.000893
3L:9847820-9847970:plus -31.4796 1.55046 7.80734 5.57798 -26.9286 -18.051 -21.3578 3.41667 -2.92661
0.0128 0.00592 0.000485 0.0034 0.0214 0.00886 0.0262 0.00508 0.0437
3L:9848340-9848490:minus -20.7041 0.706422 -0.40367 1.74312 10.6939 -9.53061 -9.11927 -2.125 -2.18349
0.00175 0.00805 0.0132 0.016 7.35e-06 0.0031 0.00262 0.0308 0.0351
3L:9848340-9848490:plus -24.449 9.23853 0.0917431 5.58716 -27.1939 -8.72449 -27.6881 5.61111
-6.49541 0.00802 0.000198 0.0112 0.00334 0.0267 0.00169 0.066 0.00199 0.101
3L:9848760-9848910:minus -22.9184 -3.22936 -1.74312 9.7156 -17.7143 -8.5 -18.633 0.569444 -1.02752
0.00447 0.0275 0.02 0.000242 0.00627 0.00126 0.0165 0.014 0.0263
3L:9848760-9848910:plus -40.7245 0.330275 3.75229 -0.981651 -18.2245 -28.3367 -16.5963 -1.31944
4.13761 0.0428 0.00919 0.00296 0.0365 0.00877 0.0606 0.012 0.0247 0.00408
3L:9852820-9852970:minus -31.5102 -1.49541 0.0183486 -0.614679 1.80612 -36.6633 -13.4404 3.09722
3.88991 0.013 0.0167 0.0115 0.0324 0.0000557 0.115 0.00639 0.00575 0.00467
3L:9852820-9852970:plus -13.6735 14.8532 2.38532 3.77064 -8.96939 -18.0816 -25.5046 3.55556 1.88073
0.00103 2.86e-06 0.00501 0.00637 0.00226 0.00934 0.0503 0.00481 0.00987
3L:9856840-9856990:minus -31.1735 3.08257 5.56881 7.72477 -18.1531 -19.2347 -20.8532 2.22222 7.73394
0.0109 0.00338 0.0014 0.000681 0.00829 0.018 0.0239 0.00796 0.000784
3L:9856840-9856990:plus -12.2245 1.27523 7.51376 6.72477 -8.64286 -17.7551 -13.9083 1.06944 7.62385
0.000376 0.00655 0.000563 0.00154 0.00139 0.00643 0.00711 0.0119 0.000941
3L:9857560-9857710:minus -24.449 8.04587 -1.47706 4.79817 -28.0408 -18.5 -16.9817 2.38889 -3.42202
0.00802 0.000373 0.0185 0.00453 0.0455 0.0131 0.0128 0.00749 0.0488
3L:9857560-9857710:plus -29.551 0.733945 4.46789 1.58716 11.6939 -18.2347 -11.7156 1.15278 5.59633
0.00845 0.00797 0.00222 0.017 1.17e-06 0.0104 0.00433 0.0115 0.00234
3L:9881700-9881850:minus -33.1122 3.10092 1.09174 5.29358 -27.3061 19.1531 -13.8624 -0.513889
0.614679 0.0271 0.00335 0.00801 0.00398 0.0315 7.95e-07 0.00704 0.0196 0.0158
3L:9881700-9881850:plus -23.2653 1.09174 -0.513761 4.02752 -9.45918 -10.0204 -20.422 1.29167
-3.26606 0.00593 0.007 0.0137 0.00581 0.00287 0.00399 0.0222 0.011 0.0472
3L:9882100-9882250:minus -33.3673 1.02752 -1.63303 1.26606 -28.2755 -0.540816 -23.9633 6.16667
-0.00917431 0.0282 0.00717 0.0194 0.0191 0.0539 0.00054 0.0408 0.00153 0.0184
3L:9882100-9882250:plus -3.92857 0.192661 8.69725 5.94495 -27.4184 0.244898 -14 5.875 3.87156
0.000116 0.00965 0.000299 0.00261 0.0323 0.000285 0.00725 0.00176 0.00477
3L:9885620-9885770:minus -31.551 6.77982 -2.6422 2.20183 -8.97959 -8.57143 -23.1743 1.375 0.66055
0.0134 0.000727 0.0261 0.013 0.0023 0.00149 0.0361 0.0107 0.0156
3L:9885620-9885770:plus -32.2143 7.3211 -2.65138 1.48624 -27.0408 -18.5612 -9.2844 2.16667 3.77982
0.0182 0.000548 0.0261 0.0177 0.0242 0.0132 0.0027 0.00812 0.00489
3L:9890360-9890510:minus -31.8061 2.63303 2.45872 6.41284 -8.90816 -26.7551 -12.4404 -3.95833 4.25688
0.0158 0.00398 0.00488 0.00189 0.00192 0.0263 0.00509 0.0488 0.00391
3L:9890360-9890510:plus -22.8776 6.10092 13.9725 -0.633028 -8.64286 -35.1122 -21.3578 0.194444
0.623853 0.00428 0.00101 7.38e-06 0.0327 0.00139 0.0748 0.0262 0.0157 0.0158
3L:9891620-9891770:minus -22.6224 2.54128 3.05505 3.44037 -18.5306 -9.53061 -8.7156 -3 1.31193
0.00371 0.00411 0.00389 0.00746 0.0118 0.0031 0.00242 0.0386 0.0127
3L:9891620-9891770:plus -31.4796 1.46789 2.3945 5.57798 -27.4592 -17.5 -11.5872 1.125 1.46789
0.0128 0.0061 0.00499 0.0034 0.0329 0.00599 0.00422 0.0117 0.0119
3L:9892480-9892630:minus -30.7041 4.43119 11.1009 3.18349 -27.2347 -8.16327 -13.7156 3.20833 -2.79817
0.00993 0.00204 0.000075 0.00837 0.0283 0.000783 0.00681 0.00551 0.0423
3L:9892480-9892630:plus -4.89796 2.05505 9.37615 3.90826 -17.7857 -8.5 -25.4495 -2.76389 -5.22018
0.000208 0.00492 0.000204 0.00603 0.00676 0.00126 0.05 0.0364 0.0762
3L:9961420-9961570:minus -40.5102 -1.00917 0.678899 6.44954 -7.93878 -28.4082 -11.3303 1.98611 4.88073

0.0409	0.0144	0.00923	0.00183	0.000767	0.0615	0.00399	0.00866	0.00324
3L:9961420-9961570:plus	-13.1837	-1.88073	-1.42202	5.06422	-8.70408	0.346939	-3.10092	-2.15278	0.550459
0.000705	0.0188	0.0182	0.00433	0.00155	0.000221	0.000583	0.0311	0.0161
3L:9962620-9962770:minus	-33.0306	-1.01835	1.10092	1.22936	-26.9286	-28.602	-9.78899	-0.277778
7.68807	0.026	0.0144	0.00798	0.0193	0.0214	0.0648	0.00296	0.0182	0.000841
3L:9962620-9962770:plus	-22.7755	0.137615	3.33945	2.34862	-17.4184	-16.7551	-17.2385	0.527778	4.56881
0.00412	0.00984	0.00348	0.0121	0.00457	0.0042	0.0133	0.0142	0.00356
3L:9965200-9965350:minus	-33.102	-1.13761	14.8532	2.70642	-8.64286	-26.3367	2.04587	5	-2.10092
0.0268	0.015	2.28e-06	0.0103	0.00139	0.0221	0.000163	0.00263	0.0342
3L:9965200-9965350:plus	-23.7041	-4.29358	-0.0458716	7.17431	10.3878	-18.051	-24.1651	1.875
0.816514	0.00669	0.0366	0.0118	0.00115	0.0000174	0.00886	0.042	0.00901	0.015
3LHet:1396000-1396150:minus	-21.6939	-1.10092	4.79817	1.06422	-18.4898	-0.540816	-18.1101	7.33333
-5.97248	0.00226	0.0148	0.00193	0.0203	0.0116	0.00054	0.0152	0.000844	0.0883
3LHet:1396000-1396150:plus	-32.7755	8.08257	0.238532	-3.01835	-17.7551	0.755102	-11.6697	3.41667
4.14679	0.0234	0.000365	0.0107	0.069	0.00658	0.000175	0.00429	0.00508	0.00406
3LHet:1396360-1396510:minus	-24.5306	6.21101	1.98165	-3.26606	-17.4184	0.0510204	-30.0092	2.54167
-4.3211	0.00814	0.000958	0.00582	0.0727	0.00457	0.000349	0.091	0.00709	0.0605
3LHet:1396360-1396510:plus	-32.8061	13.4592	8.38532	5.86239	-9.23469	-7.5	-13.8257	0.180556
-0.0733945	0.0237	0.000064	0.000356	0.00282	0.00277	0.000614	0.00698	0.0158	0.0188
3LHet:1396860-1397010:minus	-29.4082	3.86239	1.62385	2.43119	10.4286	-18.1224	-27.5872	7.86111
-1.22018	0.0084	0.00253	0.00663	0.0117	0.0000139	0.00988	0.0652	0.000631	0.0278
3LHet:1396860-1397010:plus	-31.4388	4.75229	-1.06422	2.22018	-18.2653	-18.2755	-13.3853	3.75	-0.440367
0.0122	0.0018	0.0163	0.0129	0.00882	0.0112	0.00631	0.00445	0.0216
3LHet:2189820-2189970:minus	-23.2653	7.79817	4.37615	5.73394	-28.0816	-8.72449	-18.6055	9.27778
1.27523	0.00593	0.000426	0.0023	0.00307	0.0455	0.00169	0.0164	0.000268	0.013
3LHet:2189820-2189970:plus	-24.3776	4.24771	5.88991	4.78899	-17.7143	-8.5	-22.4128	4.63889	-0.440367
0.00793	0.00219	0.00121	0.00456	0.00627	0.00126	0.0317	0.00307	0.0216
3LHet:2190640-2190790:minus	-29.9184	3.57798	1.72477	1.92661	-8.67347	0.316327	-15.1193	3.63889
-0.165138	0.00854	0.00281	0.0064	0.0148	0.00154	0.000243	0.00919	0.00465	0.0195
3LHet:2190640-2190790:plus	-22.449	-3.17431	3.26606	6.63303	-16.8163	-18.051	-12.6514	8.04167
-2.84404	0.0035	0.027	0.00358	0.00161	0.00323	0.00886	0.00534	0.00057	0.0428
3LHet:2243500-2243650:minus	-31.8878	5.07339	2.17431	6.00917	-8.93878	-9.79592	-22.2661	4.5	-0.348624
0.0164	0.00158	0.00542	0.00255	0.00216	0.00377	0.0309	0.00326	0.0209
3LHet:2243500-2243650:plus	-22.5918	13.844	2.22936	1.91743	-27.2755	-18.0102	-16.4679	4.20833	3
0.00365	9.98e-06	0.00531	0.015	0.0302	0.00812	0.0118	0.00369	0.00702
3LHet:2397660-2397810:minus	-13.2959	5.94495	-2.56881	3.78899	-27.4184	-9.79592	-27.7064	0.847222
-3.12844	0.000775	0.00108	0.0255	0.00632	0.0323	0.00377	0.0662	0.0128	0.046
3LHet:2397660-2397810:plus	-30.3673	-2.91743	3.59633	3.88991	-17.6429	9.53061	-7.27523	-0.930556
-2.48624	0.00897	0.0252	0.00315	0.00608	0.00562	0.0000316	0.0018	0.0221	0.0387
3LHet:2397980-2398130:minus	-32.7347	6.72477	1.16514	6.6422	-8.96939	-0.204082	-22.2202	2.19444
-0.440367	0.0225	0.000747	0.0078	0.00161	0.00226	0.000463	0.0307	0.00804	0.0216
3LHet:2397980-2398130:plus	-13.5204	6.40367	0.651376	4.22936	-18.7143	-17.8571	-15.5872	4.76389
5.90826	0.00097	0.000874	0.00932	0.00525	0.0136	0.00719	0.0101	0.00291	0.00198
3LHet:2398500-2398650:minus	-23.9592	-0.963303	3.78899	5.34862	-27.1939	-0.0918367	-10.7523
1.91667	3.81651	0.00708	0.0142	0.00292	0.00387	0.0267	0.000442	0.00355	0.00888	0.00482
3LHet:2398500-2398650:plus	-32.4796	-2.49541	0.87156	0.366972	-27.2347	-18.1224	-12.4587	-4.86111
-1.33945	0.02	0.0224	0.00864	0.0244	0.0283	0.00988	0.00511	0.0598	0.0285
3LHet:2398940-2399090:minus	-21.551	6.6055	0.807339	2.07339	-17.6429	-9.7551	-16.1009	4.375
-5.80734	0.00205	0.000793	0.00884	0.0138	0.00562	0.00358	0.011	0.00344	0.0853
3LHet:2398940-2399090:plus	-24.8571	5.49541	-0.697248	5.57798	-18.1837	-17.6429	-14.1193	4.34722
-0.0825688	0.00831	0.00132	0.0145	0.0034	0.00835	0.00619	0.00745	0.00348	0.0189
3LHet:2399520-2399670:minus	-31.7347	-2.14679	3.08257	3.78899	-7.82653	-17.0102	-1.23853	-0.0833333
2.99083	0.0149	0.0203	0.00385	0.00632	0.00059	0.00453	0.000374	0.0172	0.00706
3LHet:2399520-2399670:plus	-21.8469	3.68367	-1.57798	6.47706	-7.89796	-9.7551	-13.7706	1.58333

-4.44037 0.00247 0.00144 0.019 0.00179 0.000678 0.00358 0.00689 0.00997 0.063
3LHet:2409760-2409910:minus -31.8163 -1.80734 -0.587156 0.53211 -18.9796 -8.23469 -23.6239 3.70833
1.82569 0.016 0.0184 0.014 0.0234 0.0155 0.000875 0.0388 0.00452 0.0101
3LHet:2409760-2409910:plus -31.8163 -1.77982 8.34862 6.73394 10.7245 -9.02041 -22.8991 8.59722
3.88991 0.016 0.0182 0.000363 0.00152 5.2e-06 0.00213 0.0345 0.000411 0.00467
3LHet:2410000-2410150:minus -3.56122 3.97959 -2.87156 6.09174 -27.5306 -9.57143 -24.4679 4.625
-2.44037 0.0000803 0.000669 0.0278 0.00235 0.0351 0.00326 0.0439 0.00309 0.0381
3LHet:2410000-2410150:plus -32.2143 -2.02752 -0.669725 2.3211 -17.449 -17.2755 -20.4037 1.36111
7.73394 0.0182 0.0196 0.0144 0.0123 0.00484 0.00544 0.0221 0.0108 0.000784
3LHet:459480-459630:minus -31 1.20183 -1.76147 9.51376 -19.0102 -18.2347 -10.5229 3.98611 -4.74312
0.0105 0.00673 0.0201 0.000292 0.0157 0.0104 0.0034 0.00404 0.0682
3LHet:459480-459630:plus -33.3776 1.04587 -1.31193 1.44037 -27.2245 1.12245 -20.4037 7.72222 5.70642
0.0282 0.00712 0.0176 0.018 0.0272 0.000085 0.0221 0.000682 0.00222
3LHet:594360-594510:minus -40.2653 13.4592 1.6789 5.86239 -8.23469 -8.57143 -29.7615 0.555556 1.17431
0.0383 0.000064 0.0065 0.00282 0.000927 0.00149 0.0879 0.014 0.0134
3LHet:594360-594510:plus -32 8.01835 -4.94495 4.02752 -28.4592 0.530612 -14.6972 2.90278 1.48624
0.0171 0.000378 0.0487 0.00581 0.0568 0.000203 0.00843 0.00619 0.0118
3LHet:594640-594790:minus -41.6633 0.944954 3.25688 5.22018 -27.1939 -19.051 -10.4771 8.84722 -4.41284
0.0636 0.00739 0.0036 0.00418 0.0267 0.0165 0.00337 0.000353 0.0624
3LHet:594640-594790:plus -31.7347 -2.26606 8.85321 2.83486 -17.449 -18.3469 -7.80734 3.23611 -2
0.0149 0.021 0.000275 0.0097 0.00484 0.0124 0.00202 0.00545 0.0332
3LHet:601280-601430:minus -31.7347 13.3878 0.963303 7.44037 -8.93878 -9.5 -14.1009 9.06944 -4.47706
0.0149 0.0000869 0.00837 0.000912 0.00216 0.00294 0.00742 0.000307 0.0636
3LHet:601280-601430:plus -23.3367 5.02752 7.98165 7.21101 -17.0408 -18.2041 -14.9633 4.34722 2.07339
0.00614 0.00161 0.000442 0.0011 0.00329 0.0103 0.00891 0.00348 0.00906
3LHet:606180-606430:minus -39.8061 -2.33028 0.93578 4.6422 -17.8265 -0.204082 -31.7523 2.04167
0.266055 0.034 0.0214 0.00845 0.00473 0.00691 0.000463 0.115 0.00849 0.0171
3LHet:606180-606430:plus -23.2245 -2.16514 4.09174 3.13761 -18.1939 -16.9388 -21.3945 5.75 -2.6422
0.00576 0.0204 0.00258 0.00854 0.0086 0.00433 0.0264 0.00187 0.0405
3LHet:607120-607270:minus -23.1122 8.80734 2.6422 8.6055 -26.9694 -8.72449 -12.9083 0.930556 -1.22018
0.0053 0.000248 0.00455 0.000567 0.023 0.00169 0.00566 0.0124 0.0278
3LHet:607120-607270:plus -12.4082 8.52294 3.31193 5.45872 -17.6837 0.316327 -6.9633 6.41667 -2.46789
0.000414 0.000288 0.00352 0.00363 0.0058 0.000243 0.00167 0.00135 0.0384
3LHet:715580-715730:minus -14.7449 7.6789 -1.0367 2.14679 -18.3469 -18.051 -13.4587 -3.88889 12.2752
0.00157 0.000454 0.0161 0.0133 0.00894 0.00886 0.00642 0.048 0.0000141
3LHet:715580-715730:plus -14.4082 -0.926606 -0.348624 2.19266 -27 -17.898 2.42202 2.81944
-0.761468 0.00136 0.014 0.013 0.0131 0.0233 0.00726 0.000144 0.00639 0.0244
3R:10108540-10108690:minus -21.8878 2.6789 -0.816514 5.45872 -18.4898 -17.9796 -24.7248 -3.94444
-0.440367 0.00258 0.00391 0.0151 0.00363 0.0116 0.00764 0.0455 0.0486 0.0216
3R:10108540-10108690:plus -23.102 4.52294 -3.11927 5.22936 -19.0918 -10.0204 1.55046 3.375 3.20183
0.00522 0.00197 0.0298 0.0041 0.0163 0.00399 0.000189 0.00516 0.0065
3R:10109160-10109310:minus -31.3673 1.05505 0.761468 5.73394 -18.5204 -19.0102 -18.3303 5.13889
4.55963 0.0116 0.00709 0.00897 0.00307 0.0117 0.0159 0.0157 0.00247 0.00357
3R:10109160-10109310:plus -24.1837 5.63303 0.440367 2.31193 -8.71429 -37.9592 -14.2569 4.26389 12.2752
0.00751 0.00125 0.01 0.0123 0.00159 0.18 0.00768 0.0036 0.0000141
3R:10109640-10109790:minus -23.5612 3.55046 -1.68807 2.45872 -17.0816 -17.2755 -31.7615 5.22222
-0.440367 0.00653 0.00284 0.0197 0.0116 0.00334 0.00544 0.115 0.00238 0.0216
3R:10109640-10109790:plus -23.1531 -2.3945 0.100917 2.72477 -17.449 -7.5 -17.211 0.0833333 -0.706422
0.00542 0.0218 0.0112 0.0102 0.00484 0.000614 0.0132 0.0163 0.024
3R:10110980-10111130:minus -23.9286 13.4592 2.12844 5.47706 -17.9796 -8.42857 -13.3394
3.90278 -0.779817 0.00701 0.000064 0.00551 0.0036 0.00726 0.00104 0.00625 0.00418 0.0245
3R:10110980-10111130:plus -23 1.44037 1.12844 1.84404 1.87755 -8.72449 -20.4128 3.88889 1.48624
0.00498 0.00617 0.00791 0.0154 0.0000492 0.00169 0.0221 0.00421 0.0118
3R:10112160-10112310:minus -21.9286 1.24771 -2.43119 2.95413 -26.8571 -10.0204 -20.2569

5.27778 1.89908 0.0027 0.00661 0.0245 0.00917 0.0198 0.00399 0.0215 0.00232 0.0098
3R:10112160-10112310:plus -30.2245 7.58716 2.3578 2.66972 -19.0102 -7.23469 -28.2477
3.22222 -0.614679 0.00881 0.000476 0.00506 0.0106 0.0157 0.000557 0.0711 0.00548 0.0231
3R:10114800-10114950:minus -32.7041 1.62385 0.807339 1.62385 -8.86735 -9.27551 -18.2018
-3.88889 -2.33945 0.0222 0.00577 0.00884 0.0168 0.00173 0.00244 0.0154 0.048 0.0367
3R:10114800-10114950:plus -31.1429 -0.266055 4.37615 5.86239 -17.0816 -9.45918 -11.2294
-0.152778 1.2844 0.0108 0.0113 0.0023 0.00282 0.00334 0.00279 0.00391 0.0175 0.0129
3R:10116000-10116150:minus -22.7449 -6.11009 -1.52294 2.11927 -17.4184 -26.3061
-18.4679 -2.31944 1.99083 0.00404 0.0587 0.0187 0.0135 0.00457 0.0219 0.0161 0.0325 0.00946
3R:10116000-10116150:plus -23.602 -0.155963 -0.0275229 2.58716 -18.7857 -27.602 -18.0367
3.625 7.31193 0.00657 0.0109 0.0117 0.011 0.0148 0.0416 0.015 0.00468 0.00112
3R:10119620-10119770:minus -31.6327 1.37615 -0.100917 5.74312 -26.898 -8.7551 -23.0734 8.93056
2.49541 0.0138 0.00631 0.012 0.00302 0.0203 0.00172 0.0355 0.000335 0.00794
3R:10119620-10119770:plus -23.4898 2.19266 -0.0642202 6.00917 -8.86735 0.122449 -20.2844
4.13889 -5.19266 0.00639 0.00468 0.0118 0.00255 0.00173 0.000312 0.0216 0.0038 0.0758
3R:10121620-10121770:minus -30.3673 1.78899 1.01835 4.81651 -18.6837 -8.5 -9.27523 3.375
-0.908257 0.00897 0.00542 0.00821 0.00451 0.0126 0.00126 0.00269 0.00516 0.0255
3R:10121620-10121770:plus -22.0408 4.97248 3.49541 5.74312 -17.4184 -9.23469 -21.6697
-0.0555556 1.61468 0.00296 0.00165 0.00327 0.00302 0.00457 0.00238 0.0278 0.017 0.0112
3R:10123900-10124050:minus -22.7755 -0.614679 2.87156 3.7156 -7.93878 -19.1224 -4.81651
-3.01389 -1.44954 0.00412 0.0127 0.00417 0.00655 0.000767 0.0175 0.000942 0.0388 0.0291
3R:10123900-10124050:plus -13.4082 -0.981651 4.89908 2.47706 0.571429 -18.051 -18.5413
-2.625 7.42202 0.000867 0.0143 0.00185 0.0115 0.0003 0.00886 0.0162 0.0351 0.00106
3R:10133860-10134010:minus -14.7449 -4.84404 7.87156 7.10092 0.908163 -9.60204 -12.844
-0.777778 -0.880734 0.00157 0.0423 0.000469 0.00121 0.000153 0.00334 0.00558 0.0211 0.0252
3R:10133860-10134010:plus -21.8878 5.91743 -0.651376 4.20183 -17.7857 -18.9388 -12.844
-0.277778 7.62385 0.00258 0.0011 0.0143 0.00533 0.00676 0.0154 0.00558 0.0182 0.000941
3R:10134240-10134390:minus -31.4082 -1.48624 -2.7156 1.33028 20.4694 -8.5 -8.31193 -1.91667
-6.70642 0.0119 0.0167 0.0266 0.0188 1.65e-07 0.00126 0.00224 0.0291 0.106
3R:10134240-10134390:plus -34.3673 -6 1.6789 2.14679 -27.7959 -8.57143 -27.0183 1.75
-0.504587 0.0318 0.0571 0.0065 0.0133 0.0386 0.00149 0.0606 0.00941 0.0221
3R:10141660-10141810:minus -24.8265 3.68367 4.69725 7.61468 -27.3061 19.051 -26.3578 4.44444
-2.22018 0.0083 0.00144 0.00202 0.000779 0.0315 1.23e-06 0.0559 0.00334 0.0355
3R:10141660-10141810:plus -31.551 -1.87156 5.22936 3.04587 10.0918 -27.602 -24.2752 0.541667
5.3945 0.0134 0.0187 0.00162 0.00883 0.0000251 0.0416 0.0427 0.0141 0.00263
3R:10152260-10152410:minus -12.0714 1.89908 0 7.34862 10.6939 -17.3469 -16.3211 -1.80556
3.76147 0.000318 0.00521 0.0116 0.000998 7.35e-06 0.0058 0.0115 0.0283 0.00494
3R:10152260-10152410:plus -29.7755 4.02752 -0.715596 6.12844 -18.4898 -18.4286 -16.2661
-0.611111 -2.33028 0.0085 0.00238 0.0146 0.00231 0.0116 0.0128 0.0114 0.0201 0.0366
3R:10152640-10152790:minus -41.8163 -2.01835 -1.27523 3.33945 -27.2653 -19.1224
-16.9174 6.91667 1.74312 0.0662 0.0195 0.0174 0.00774 0.0302 0.0175 0.0126 0.00105 0.0105
3R:10152640-10152790:plus -13.0714 16.3761 14.6147 9.3211 -7.97959 9.60204 -14.5963 -0.833333
-0.697248 0.000594 1.59e-07 3.32e-06 0.000391 0.000799 0.0000248 0.00826 0.0215
0.0239
3R:10153660-10153810:minus -41.4694 0.93578 3.27523 6.45872 -19.0102 -8.09184 -10.3028
6.18056 1 0.0565 0.00741 0.00357 0.00182 0.0157 0.000762 0.00326 0.00152 0.0143
3R:10153660-10153810:plus -21.7041 0.201835 14.8532 6.12844 -17.9388 -18.6429 -6.20183
0.194444 -4.41284 0.00231 0.00962 2.28e-06 0.00231 0.00715 0.0139 0.00138 0.0157 0.0624
3R:10172760-10172910:minus -31.4796 1.77064 2.55046 5.31193 -18.0816 -18.3061 -11.6239
5.33333 7.15596 0.0128 0.00546 0.00471 0.00393 0.00759 0.0114 0.00425 0.00226 0.00124
3R:10172760-10172910:plus -13.2653 3.87156 -1.9633 2.94495 -27.0102 -27.3776 -12.1743
6.31944 7.22018 0.000759 0.00252 0.0214 0.0092 0.0237 0.0351 0.00479 0.00142 0.00119
3R:10187440-10187590:minus -32.6735 -3.87156 14.7156 5.74312 -8.37755 -17.2755 -15.7706
-4.31944 -2.72477 0.0216 0.0327 2.9e-06 0.00302 0.00106 0.00544 0.0104 0.053 0.0414

3R:10187440-10187590:plus -13 3.09174 5.20183 4.07339 -28.1939 -8.16327 -20.211 5.94444 7.22018
0.000563 0.00337 0.00163 0.00562 0.0482 0.000783 0.0214 0.0017 0.00119
3R:10188760-10188910:minus -32.0408 2.34862 3.95413 2.30275 -8.67347 -19.0816 12.7431 3.75
9.66055 0.0174 0.00441 0.00273 0.0124 0.00154 0.017 3.94e-06 0.00445 0.000268
3R:10188760-10188910:plus -32.9286 4.40367 2.6055 2.88991 -17.6429 -17.2347 -16.9174
-0.916667 -1.48624 0.025 0.00206 0.00462 0.00942 0.00562 0.00511 0.0126 0.022 0.0293
3R:10191760-10191910:minus -24.4796 5.05505 -1.92661 7.27523 -17.7143 -18.1939 -13.2018
9.97222 5.34862 0.00804 0.00159 0.0212 0.00109 0.00627 0.0101 0.00605 0.000167 0.00271
3R:10191760-10191910:plus -13.4082 0.770642 1.07339 3.18349 -8.67347 -19.4184 -11.6606
4.11111 3.23853 0.000867 0.00786 0.00806 0.00837 0.00154 0.0198 0.00428 0.00384 0.00639
3R:10195180-10195330:minus -14.0816 -1.92661 -0.0642202 2.04587 -7.89796 -7.65306
-9.57798 -2.41667 5.70642 0.00117 0.019 0.0118 0.014 0.000678 0.000647 0.00285 0.0333
0.00222
3R:10195180-10195330:plus -21.9694 1.70642 -0.779817 9.7156 -18.4184 -17.6429 -11.9541
0.111111 6.04587 0.00282 0.00559 0.0149 0.000242 0.0101 0.00619 0.00456 0.0162 0.00181
3R:10195940-10196090:minus -12.8878 1.57798 -1.95413 11.2936 -7.96939 -18.8571 -20.5413
2.5 1.48624 0.000523 0.00586 0.0213 0.0000139 0.000785 0.0151 0.0226 0.0072 0.0118
3R:10195940-10196090:plus -30.5102 7.50459 7.69725 2.66972 -18.7857 -17.3469 -5.30275
4.31944 1.72477 0.00947 0.000498 0.000513 0.0106 0.0148 0.0058 0.00108 0.00352 0.0106
3R:10198580-10198730:minus -21.7347 -2.01835 4.9633 7.37615 -17.6735 -26.5612 -5.31193
-0.0833333 3.21101 0.00235 0.0195 0.00181 0.000951 0.00572 0.0237 0.00108 0.0172 0.00644
3R:10198580-10198730:plus -22.9286 0.844037 -0.46789 1.10092 -17.1939 -18.6429
-21.4495 7.69444 2.90826 0.0046 0.00766 0.0135 0.02 0.00377 0.0139 0.0267 0.000693 0.00718
3R:10199320-10199470:minus -30.8163 -1.27523 2.14679 6.48624 -27.4592 -8.5 -6.90826 3.5
11.8532 0.0103 0.0156 0.00547 0.00177 0.0329 0.00126 0.00165 0.00491 0.0000428
3R:10199320-10199470:plus -21.8878 1.58716 -0.0642202 5.49541 -16.7143 -16.9388 -20.2385
6.18056 9.66055 0.00258 0.00584 0.0118 0.00355 0.00319 0.00433 0.0215 0.00152 0.000268
3R:10201940-10202090:minus -23.8061 -0.183486 5.48624 11.1927 -17.0408 -18.7143 -19.3945
-0.472222 -1.17431 0.0068 0.011 0.00145 0.0000254 0.00329 0.014 0.0186 0.0193 0.0275
3R:10201940-10202090:plus -34.1429 -3.88073 -0.614679 5.43119 -18.2245 -18.6327
-10.1743 4.98611 1.78899 0.0313 0.0328 0.0141 0.00371 0.00877 0.0137 0.00318 0.00264 0.0103
3R:10208180-10208330:minus -32.449 3.83673 7.58716 6.12844 -18.8265 -26.5612 -24.3211 -6.68056
-2.94495 0.0195 0.00115 0.000543 0.00231 0.0149 0.0237 0.043 0.0871 0.0441
3R:10208180-10208330:plus -30.6633 3.47706 1.62385 1.44037 -9.16327 -9.5 -14.422 -1.66667
-6.57798 0.00976 0.00292 0.00663 0.018 0.00249 0.00294 0.00796 0.0272 0.103
3R:10286080-10286230:minus -30.8163 0.917431 -0.798165 3.88991 -18.7857 -19.3776
-12.367 2.11111 10.0367 0.0103 0.00746 0.015 0.00608 0.0148 0.0195 0.005 0.00828 0.000146
3R:10286080-10286230:plus -34.449 1.53211 -2.74312 7.15596 -8.86735 -16.2755 -14.422 5.33333
-0.220183 0.0319 0.00596 0.0268 0.00117 0.00173 0.00413 0.00796 0.00226 0.0201
3R:10300720-10300870:minus -22.7755 7.37615 5.21101 -0.880734 -27.5306 -18.6429 2.6055
7.06944 7.63303 0.00412 0.000532 0.00163 0.0353 0.0351 0.0139 0.000135 0.00097 0.000928
3R:10300720-10300870:plus -22.1837 2.44037 4.45872 10.8624 -17.3776 -19.0816 -11.7982
2.59722 1.22936 0.00315 0.00427 0.00223 0.0000687 0.00419 0.017 0.00441 0.00694 0.0132
3R:10305720-10305870:minus -23.2245 7.3578 3.89908 1.10092 -17.4898 -9.38776 -4.01835
2.77778 1.21101 0.00576 0.000538 0.00279 0.02 0.0053 0.00266 0.00075 0.00649 0.0134
3R:10305720-10305870:plus -33.0714 7.36697 -1.6422 0.779817 -18.4898 -26.898 -8.22936
3.26389 6.33028 0.0267 0.000535 0.0194 0.0219 0.0116 0.0302 0.0022 0.00539 0.0016
3R:10306640-10306790:minus -22.2245 8.04587 0.990826 2.25688 -27.1939 -0.540816 -17.7982
2.13889 3.20183 0.00325 0.000373 0.00829 0.0127 0.0267 0.00054 0.0145 0.0082 0.0065
3R:10306640-10306790:plus -22.1122 6.11927 -3.07339 -0.715596 -18.1939 -36.0408
-8.93578 3.79167 9.92661 0.00304 0.001 0.0294 0.0336 0.0086 0.0837 0.00253 0.00437 0.000193
3R:10307420-10307570:minus -22.9286 -3.82569 8.38532 2.66972 -17.4898 -18.1224 -12.8991
3.83333 7.04587 0.0046 0.0323 0.000356 0.0106 0.0053 0.00988 0.00565 0.0043 0.00132
3R:10307420-10307570:plus 4.62245 0.559633 -0.146789 1.7156 -8.93878 -27.4898 -0.247706

-0.541667 9.34862 0.0000219 0.00848 0.0122 0.0162 0.00216 0.0363 0.0003 0.0197 0.000371
3R:10337240-10337390:minus -30.2143 9.45872 11.2752 7.29358 -26.6735 -18.7857 -19.0183 -0.986111
1.49541 0.0088 0.000176 0.0000673 0.00104 0.0191 0.0146 0.0175 0.0225 0.0118
3R:10337240-10337390:plus -23.2551 2.88073 14.422 7.29358 -8.89796 -18.9796 -16.7982 -1.625 1.85321
0.00583 0.00363 4.18e-06 0.00104 0.00189 0.0155 0.0124 0.0269 0.01
3R:10339320-10339470:minus -22.1531 7.79817 13.9908 7.2844 -17.4082 -18.7857 12.0092 6.43056
-3.10092 0.0031 0.000426 7.33e-06 0.00105 0.00421 0.0146 5.61e-06 0.00134 0.0458
3R:10339320-10339470:plus -40.551 -0.816514 -0.908257 2.27523 -8.23469 -17.2755 -12.2018 0.791667
-2.20183 0.0418 0.0135 0.0155 0.0125 0.000927 0.00544 0.00482 0.013 0.0353
3R:10383820-10383970:minus -23.3265 -5.69725 0.348624 3.04587 -8.97959 -27.6327 -10.2569 -6.08333
-0.40367 0.00607 0.0529 0.0103 0.00883 0.0023 0.043 0.00323 0.0774 0.0213
3R:10383820-10383970:plus -28.3673 -0.926606 9.89908 3.90826 -26.8163 -9.94898 -6.61468 4.59722
-0.59633 0.00831 0.014 0.000151 0.00603 0.0195 0.00393 0.00154 0.00313 0.0229
3R:10394540-10394690:minus -31.6939 1.61468 -0.697248 5.43119 -18.4184 0.122449 -13.3303 -3.95833
4.56881 0.0142 0.00578 0.0145 0.00371 0.0101 0.000312 0.00624 0.0488 0.00356
3R:10394540-10394690:plus -22 8.08257 -3.23853 -1.68807 -26.9694 -8.79592 -4.37615 7.33333 -0.972477
0.00291 0.000365 0.0308 0.0461 0.023 0.0019 0.00083 0.000844 0.0259
3R:10400720-10400870:minus -31.4388 5.58716 1.72477 3.48624 -17.0408 0.387755 -10.0459 0.680556
-2.9633 0.0122 0.00127 0.0064 0.0072 0.00329 0.000217 0.0031 0.0135 0.0442
3R:10400720-10400870:plus -23.3673 -0.40367 0.605505 1.89908 -17.9796 1.82653 2.74312 6.375 3.46789
0.00617 0.0118 0.00946 0.0151 0.00726 0.0000498 0.000129 0.00138 0.00582
3R:10401060-10401210:minus -31.2959 -0.972477 2.89908 3.9633 -27.5306 -0.27551 -13.2202 -3.86111
7.84404 0.0113 0.0142 0.00413 0.0059 0.0351 0.000502 0.00608 0.0477 0.000701
3R:10401060-10401210:plus -32.7041 -0.678899 2.01835 2.11927 0.846939 -17.2755 -6.10092 -5.20833
1.6422 0.0222 0.0129 0.00574 0.0135 0.000197 0.00544 0.00135 0.0645 0.0111
3R:10401560-10401710:minus -32.551 -2.21101 0.394495 4.19266 -26.6327 -18.3469 -19.9083 -0.0277778
4.75229 0.0207 0.0206 0.0102 0.00536 0.0188 0.0124 0.0203 0.0169 0.00341
3R:10401560-10401710:plus -30.8776 -2.00917 2.65138 3.07339 -8.82653 -27.8265 -21.3303 3.18056 0.385321
0.0103 0.0195 0.00453 0.00873 0.00163 0.0498 0.0261 0.00557 0.0166
3R:1043000-1043150:minus -11.7755 2.6789 0.495413 4.33945 -27.1224 -27.4898 -16.9358 0.680556 4.10092
0.000266 0.00391 0.00983 0.00509 0.0245 0.0363 0.0127 0.0135 0.00413
3R:1043000-1043150:plus -12.9184 2.9633 -0.266055 -0.0366972 -18.1939 0.0918367 -5.36697
-2.27778 3.15596 0.000532 0.00353 0.0126 0.0271 0.0086 0.000323 0.0011 0.0321 0.00659
3R:10450900-10451050:minus -21.7347 2.01835 1.40367 7.33028 -17.4184 -9.23469 -14.1284 3.98611
-1.75229 0.00235 0.00499 0.00717 0.00103 0.00457 0.00238 0.00746 0.00404 0.0312
3R:10450900-10451050:plus -14.4796 3.37615 1.24771 1.89908 -17.7143 -18.051 1.18349 2.45833 -2.40367
0.00142 0.00303 0.00758 0.0151 0.00627 0.00886 0.00021 0.00731 0.0376
3R:10451500-10451650:minus -14.102 7.46789 0.192661 8.80734 0.653061 -28.3367 -7.89908 0.777778
1.95413 0.00117 0.000506 0.0109 0.000518 0.000239 0.0606 0.00206 0.0131 0.00964
3R:10451500-10451650:plus -31.4388 -0.183486 0.844037 3.94495 1.94898 -8.5 -20.5413 0.708333 2.58716
0.0122 0.011 0.00872 0.00595 0.0000358 0.00126 0.0226 0.0134 0.00779
3R:10475280-10475430:minus -14.3367 -1.57798 -1.70642 1.10092 -27.3061 -18.051 -12.0459 4.68056
11.3853 0.00131 0.0171 0.0198 0.02 0.0315 0.00886 0.00466 0.00302 0.0000815
3R:10475280-10475430:plus -14.4082 0.302752 2.20183 5.58716 -28.2347 -8.42857 3.3211 3.27778 -1.58716
0.00136 0.00929 0.00536 0.00334 0.051 0.00104 0.000106 0.00536 0.03
3R:10475800-10475950:minus -31.4796 -3.48624 5.59633 6.76147 -18.7551 -8.79592 -18.2844 1.31944
-0.513761 0.0128 0.0294 0.00138 0.00148 0.0144 0.0019 0.0156 0.0109 0.0221
3R:10475800-10475950:plus -24.2245 -1.7156 -3.12844 1.63303 -27.0102 -9.20408 -11.8073 5.625 6.80734
0.00763 0.0179 0.0299 0.0167 0.0237 0.00225 0.00442 0.00198 0.0014
3R:10476820-10477050:minus -22.8163 -2.66972 0.87156 3.40367 -18.6735 -17.1633 -15.3303 -0.611111
-0.889908 0.00418 0.0235 0.00864 0.00759 0.0123 0.005 0.00958 0.0201 0.0253
3R:10476820-10477050:plus -22.9592 -0.174312 -2.75229 -0.229358 -7.86735 -19.051 -15.5688 2.61111
7.84404 0.00475 0.0109 0.0269 0.0287 0.000617 0.0165 0.01 0.00691 0.000701
3R:10480740-10480890:minus -31.7857 1.97248 -1.89908 0.706422 -28.2347 -18.1224 -9.91743 3.91667

7.68807 0.0157 0.00507 0.021 0.0223 0.051 0.00988 0.00303 0.00416 0.000841
3R:10480740-10480890:plus -22.8061 -1.14679 2.88991 3.54128 -17.7551 -9.38776 -18.6422 -1.27778 9.87156
0.00414 0.015 0.00414 0.00702 0.00658 0.00266 0.0165 0.0244 0.000217
3R:10481380-10481530:minus -28.9184 2.50459 -3.41284 0.522936 -17.7143 -8.53061 -12.7156 3.90278
-0.623853 0.00832 0.00417 0.0324 0.0234 0.00627 0.00136 0.00542 0.00418 0.0232
3R:10481380-10481530:plus -33.5612 13.5229 0.642202 4.02752 -19.0102 1.09184 -24.8991 3.55556 -0.220183
0.0288 0.000014 0.00935 0.00581 0.0157 0.000091 0.0465 0.00481 0.0201
3R:10485220-10485370:minus -22.8163 2.18349 2.2844 3.09174 -18.3776 -8.79592 -9.2844 1.27778
5.81651 0.00418 0.00469 0.0052 0.00867 0.00926 0.0019 0.0027 0.0111 0.00204
3R:10485220-10485370:plus -22.7041 3.3578 -2.98165 -0.0550459 -8.30612 -27.0408 -16.7156 -5.97222
8.19266 0.00395 0.00305 0.0287 0.0272 0.00097 0.0307 0.0122 0.0757 0.000548
3R:10488820-10488970:minus -30.2551 13.5306 -0.422018 1.99083 10.3878 -18.0102 -10.6789 4.25
7.73394 0.00885 0.0000206 0.0133 0.0144 0.0000174 0.00812 0.0035 0.00362 0.000784
3R:10488820-10488970:plus -33.0714 4.56881 2.84404 1.3211 -27.1224 -18.0102 -15.9358 4.70833 -2.68807
0.0267 0.00193 0.00421 0.0189 0.0245 0.00812 0.0107 0.00298 0.041
3R:10513160-10513310:minus -23.6633 -0.201835 3.18349 1.99083 -8.67347 -16.5714 -13.7064 4.97222
3.14679 0.00665 0.011 0.0037 0.0144 0.00154 0.00417 0.00679 0.00266 0.00666
3R:10513160-10513310:plus -22.398 5.75229 4.20183 3.50459 -8.89796 -26.6735 -18.6697 -2.51389 1.2844
0.00344 0.00118 0.00247 0.00713 0.00189 0.0256 0.0166 0.0341 0.0129
3R:10515880-10516030:minus -12.9184 -3.36697 12.1101 3.43119 -18.7857 -19.051 -2.45872 -0.125
6.16514 0.000532 0.0285 0.0000401 0.00749 0.0148 0.0165 0.000496 0.0174 0.00172
3R:10515880-10516030:plus -22.6327 3.87156 5.29358 6.11009 -9.16327 1.20408 -20.5138 -0.847222
-0.651376 0.00373 0.00252 0.00157 0.00233 0.00249 0.0000826 0.0225 0.0216 0.0235
3R:10522780-10522930:minus -22.7755 -1.69725 7.53211 1.92661 -28.3061 -36.2245 -17.5229 3.45833
11.8532 0.00412 0.0178 0.000558 0.0148 0.0553 0.0923 0.0139 0.005 0.0000428
3R:10522780-10522930:plus -31.7347 1.7156 1.36697 6.00917 -17.4184 -8.72449 -12.2202 5.23611 -0.908257
0.0149 0.00557 0.00726 0.00255 0.00457 0.00169 0.00484 0.00236 0.0255
3R:10529240-10529390:minus -32.9184 4.94495 0.0275229 3.18349 -18.4184 -26.5612 -1.30275 0.763889
6.6422 0.0248 0.00166 0.0115 0.00837 0.0101 0.0237 0.000379 0.0131 0.00148
3R:10529240-10529390:plus -14.6735 4.30275 4.56881 5.90826 -27.3776 -18.0816 -20.9266 1.375
-0.0733945 0.00152 0.00214 0.00213 0.00271 0.032 0.00934 0.0243 0.0107 0.0188
3R:10531140-10531290:minus -40.6327 -0.53211 3.02752 2.57798 -17.3776 -10.0204 -15.4037 -2.22222
-0.12844 0.0424 0.0123 0.00393 0.011 0.00419 0.00399 0.00972 0.0316 0.0193
3R:10531140-10531290:plus -31.7755 -0.366972 4.10092 5.88991 -18.1939 -18.051 -9.16514 4.76389
-2.2844 0.0157 0.0117 0.00257 0.00273 0.0086 0.00886 0.00264 0.00291 0.0361
3R:10535560-10535710:minus -30.2551 0.513761 1.44037 2.92661 -18.1939 -17.7857 -15.6147 -3.45833
2.20183 0.00885 0.00862 0.00707 0.00931 0.0086 0.0068 0.0101 0.0433 0.00869
3R:10535560-10535710:plus -31.8469 -0.752294 -2.68807 -1.99083 -18.1531 -18.3469 -14.5321 6.15278
9.45872 0.0162 0.0132 0.0264 0.0512 0.00829 0.0124 0.00814 0.00154 0.000337
3R:10537580-10537830:minus -23 -0.651376 -0.174312 6.41284 -18.1939 -18.4184 -13.1193 4.56944
2.74312 0.00498 0.0128 0.0123 0.00189 0.0086 0.0127 0.00594 0.00317 0.00751
3R:10537580-10537830:plus -21.8469 -0.0366972 -3.9633 6.12844 -28.0408 -19.3061 -10.789 -5.68056
0.275229 0.00247 0.0104 0.0376 0.00231 0.0455 0.0187 0.00358 0.0713 0.0171
3R:10540060-10540210:minus -31.8061 -2.42202 2.78899 2.6422 -26.8163 -9.42857 -19.7798 2.36111
0.816514 0.0158 0.0219 0.0043 0.0107 0.0195 0.0027 0.0198 0.00757 0.015
3R:10540060-10540210:plus -22.7755 -0.577982 3.34862 1.08257 -18.5204 -27.602 -11.7982 1.63889
10.3394 0.00412 0.0125 0.00347 0.0201 0.0117 0.0416 0.00441 0.00978 0.000112
3R:10541940-10542090:minus -23.1122 -1.95413 -0.908257 7.27523 -8.93878 -8.53061 -18.0367 -0.847222
0.743119 0.0053 0.0192 0.0155 0.00109 0.00216 0.00136 0.015 0.0216 0.0153
3R:10541940-10542090:plus -41.5816 2.53211 6.3945 4.86239 -27.0408 -27.6327 -20.1651 6.66667 1.86239
0.061 0.00413 0.000966 0.00449 0.0242 0.043 0.0212 0.00119 0.00994
3R:10542860-10543010:minus -33 -2.01835 2.20183 8.88073 -17.3776 0.826531 -17.7339 0.819444 1.89908
0.0258 0.0195 0.00536 0.000479 0.00419 0.000153 0.0144 0.0129 0.0098
3R:10542860-10543010:plus -13.2143 -0.889908 14.8899 2.75229 -8.67347 -18.3469 -3.43119 4.01389

1.21101 0.000714 0.0138 2.06e-06 0.0101 0.00154 0.0124 0.000637 0.004 0.0134
3R:10547500-10547650:minus -21.8163 2.66055 3.52294 3.11009 -9.0102 -18.4184 -10.5413 9.29167
-0.12844 0.00244 0.00394 0.00324 0.00862 0.00236 0.0127 0.00341 0.000266 0.0193
3R:10547500-10547650:plus -13.8571 0.926606 -1.95413 0.211009 -17.2245 -17.9796 -7.78899 -1.06944 5.44037
0.0011 0.00743 0.0213 0.0254 0.00384 0.00764 0.00201 0.023 0.00259
3R:10548120-10548270:minus -22.7755 3.15596 -2.31193 2.0367 -18.449 -17.3469 -14.4037 -1.125
11.8073 0.00412 0.00328 0.0237 0.0141 0.0107 0.0058 0.00792 0.0234 0.0000538
3R:10548120-10548270:plus -30.5918 3.85321 -2.97248 9.7156 -17.3776 0.0510204 -28.6697 0.722222
-9.6789 0.00963 0.00254 0.0286 0.000242 0.00419 0.000349 0.0754 0.0133 0.184
3R:10549520-10549670:minus -30.1837 2.21101 -3.56881 6.51376 -8.60204 -17.4898 -1.07339 -2.59722
1.95413 0.00873 0.00464 0.0338 0.00175 0.00125 0.0059 0.000361 0.0349 0.00964
3R:10549520-10549670:plus -22.8878 0.541284 -1.42202 5.3945 -16.8163 -9.94898 -6.47706 -0.652778
12.2752 0.00441 0.00854 0.0182 0.00379 0.00323 0.00393 0.00148 0.0204 0.0000141
3R:10549860-10550010:minus -13.2959 -2.72477 -1.11927 -1.00917 -8.63265 -7.72449 -4.17431 -1.29167
3.07339 0.000775 0.0239 0.0166 0.0368 0.0013 0.00068 0.000784 0.0245 0.00683
3R:10549860-10550010:plus -13.3367 -2.16514 -3.58716 6.47706 -27.1939 0.316327 -7.10092 1.13889 6.7156
0.0008 0.0204 0.0339 0.00179 0.0267 0.000243 0.00173 0.0116 0.00144
3R:10554880-10555030:minus -21.9592 4.78899 1.10092 2.88991 0.836735 -17.7551 5.52294 0.222222
-1.27523 0.00279 0.00177 0.00798 0.00942 0.000215 0.00643 0.0000482 0.0156 0.0282
3R:10554880-10555030:plus -30.9592 3.13761 3.55963 9.33028 -18.449 -8.45918 -15.6514 5.5 6.17431
0.0104 0.00331 0.0032 0.000374 0.0107 0.00111 0.0102 0.00209 0.00171
3R:10555680-10555830:minus -39.8776 -0.174312 1.04587 -1.76147 -17.7143 -16.8265 -16.2844 4.95833
-2.33028 0.0341 0.0109 0.00813 0.0471 0.00627 0.0043 0.0114 0.00267 0.0366
3R:10555680-10555830:plus -3.33673 3.97959 6.6789 5.57798 -17.4898 -9.20408 -12.1193 5.56944 -5.27523
0.0000599 0.000669 0.000844 0.0034 0.0053 0.00225 0.00473 0.00203 0.0771
3R:10556500-10556650:minus -22.0306 2.36697 6.77982 3.10092 -8.40816 -7.79592 -9.12844 1.69444
1.47706 0.00293 0.00439 0.000804 0.00865 0.00108 0.000723 0.00262 0.0096 0.0119
3R:10556500-10556650:plus -23.9592 13.4592 -1.99083 2.75229 -17.602 -17.9796 -24.9817 1.58333 -0.550459
0.00708 0.000064 0.0216 0.0101 0.00547 0.00764 0.047 0.00997 0.0225
3R:10557220-10557370:minus -3.15306 0.155963 6.24771 10.8624 -26.2245 0.0510204 -19.055 -0.347222
2.34862 0.000047 0.00977 0.00103 0.0000687 0.0175 0.000349 0.0176 0.0186 0.0083
3R:10557220-10557370:plus -22.9286 3.64286 0.192661 3.19266 -17.7143 -9.60204 -14.7339 3.19444 -4.43119
0.0046 0.00159 0.0109 0.00829 0.00627 0.00334 0.0085 0.00554 0.0627
3R:10563860-10564010:minus -32.5204 -1.9633 -2.72477 5.90826 -26.8163 -8.61224 -25.5413 4.54167
-2.37615 0.0204 0.0192 0.0267 0.00271 0.0195 0.00152 0.0505 0.0032 0.0373
3R:10563860-10564010:plus -33.7041 -3.37615 3.16514 3.98165 -26.9694 -36.5204 -14.9083 8.45833 7.07339
0.0293 0.0286 0.00373 0.00582 0.023 0.109 0.00881 0.000447 0.0013
3R:10564980-10565130:minus -14.3673 -3.24771 0.944954 5.93578 -7.26531 -27.8265 -6.66055 -3.34722
11.3853 0.00132 0.0276 0.00842 0.00264 0.000375 0.0498 0.00155 0.0421 0.0000815
3R:10564980-10565130:plus -32.8571 -1.74312 -3.75229 3.54128 -17.7551 -18.5 -13.4954 5.41667 -2.22018
0.0244 0.018 0.0355 0.00702 0.00658 0.0131 0.00648 0.00218 0.0355
3R:10565440-10565590:minus -31.2449 -4.3578 1.37615 6.62385 1.14286 -37.1837 -6.44954 4.13889
4.14679 0.0111 0.0372 0.00724 0.00163 0.000113 0.14 0.00147 0.0038 0.00406
3R:10565440-10565590:plus -20.9286 -6.7156 1.98165 -1.51376 -17.5306 -17.051 -13.8624 2.09722 3.23853
0.00183 0.0682 0.00582 0.0436 0.0054 0.00479 0.00704 0.00832 0.00639
3R:10565940-10566090:minus -23.7755 -1.82569 5.82569 1.91743 -27.3469 -27.3776 -14.7982 0.291667
8.0367 0.00678 0.0185 0.00125 0.015 0.0318 0.0351 0.00861 0.0153 0.000654
3R:10565940-10566090:plus -22.6735 -1.19266 0.284404 2.14679 -28.4592 -15.9796 -33.6055 3.44444 -6.27523
0.00385 0.0152 0.0105 0.0133 0.0568 0.00405 0.14 0.00502 0.0952
3R:10567000-10567150:minus -31.4388 -1.18349 3.2844 10.8624 -27.2653 -8.0102 -17.4128 4.43056
2.27523 0.0122 0.0152 0.00356 0.0000687 0.0302 0.000754 0.0137 0.00336 0.00846
3R:10567000-10567150:plus -4.78571 11.8716 -0.46789 3.45872 -18.6735 -18.051 -9.63303 1.95833 -4.63303
0.000194 0.0000496 0.0135 0.00731 0.0123 0.00886 0.00288 0.00875 0.0661
3R:10567280-10567430:minus -32 6.05505 3.23853 5.93578 0.316327 -9.16327 -23.211 4.88889 5.6055

0.0171 0.00103 0.00362 0.00264 0.000341 0.00223 0.0363 0.00276 0.00232
3R:10567280-10567430:plus -4.78571 11.8716 -0.46789 3.45872 -18.6735 -18.051 -9.63303 1.95833 -4.63303
0.000194 0.0000496 0.0135 0.00731 0.0123 0.00886 0.00288 0.00875 0.0661
3R:10575120-10575270:minus -24.5204 3.92661 0.642202 4.91743 -28.5306 -8.5 -28.9817 3.20833
-0.0825688 0.00813 0.00247 0.00935 0.00447 0.061 0.00126 0.0787 0.00551 0.0189
3R:10575120-10575270:plus -32.8061 2.72477 12.367 4.07339 -17.3776 -9.72449 -11.2844 0.388889 1.74312
0.0237 0.00385 0.0000334 0.00562 0.00419 0.00352 0.00396 0.0148 0.0105
3R:1057960-1058110:minus -31.4694 13.4592 7.3578 5.94495 -27.7041 -8.53061 -11.0826 1.86111 1.73394
0.0124 0.000064 0.000608 0.00261 0.038 0.00136 0.0038 0.00905 0.0106
3R:1057960-1058110:plus -41.4388 7.23853 3.93578 5.89908 -8.96939 -19.3469 -28.7156 4.84722 -3.25688
0.0562 0.000573 0.00275 0.00272 0.00226 0.0194 0.0758 0.00281 0.0472
3R:10627280-10627430:minus -20.9592 -2.57798 -3.45872 5.3945 -17.449 -10.0204 -11.4128 2.91667
4.88073 0.00184 0.0229 0.0328 0.00379 0.00484 0.00399 0.00406 0.00616 0.00324
3R:10627280-10627430:plus -22.6939 1.08257 -5.6789 1.69725 -18.9796 -9.72449 -10.7431 -3.80556 7.63303
0.00388 0.00703 0.0585 0.0163 0.0155 0.00352 0.00355 0.047 0.000928
3R:10705240-10705390:minus -32.8469 0.862385 -1.66055 6.00917 -27.2653 -9.45918 -17.5229 6.45833
-4.30275 0.0243 0.00761 0.0195 0.00255 0.0302 0.00279 0.0139 0.00133 0.0602
3R:10705240-10705390:plus -13.1837 -2.44037 3.02752 3.40367 -26.898 -18.7143 -9.70642 5.75 7.73394
0.000705 0.0221 0.00393 0.00759 0.0203 0.014 0.00291 0.00187 0.000784
3R:10711780-10711930:minus -23.2959 -1.88073 4.46789 1.50459 -17.7143 -9.0102 -12.9725 -0.888889
8.11009 0.00605 0.0188 0.00222 0.0176 0.00627 0.00208 0.00574 0.0218 0.000597
3R:10711780-10711930:plus -31.7347 13.3878 4.56881 4.22018 -9.16327 -8.27551 -5.66972 4.69444 -0.862385
0.0149 0.0000869 0.00213 0.00531 0.00249 0.000919 0.0012 0.003 0.0251
3R:10715940-10716090:minus -24.4388 1.34862 14.5505 7.2844 0.877551 -18.2041 -23.7523 -1.79167
-0.917431 0.00797 0.00637 3.58e-06 0.00105 0.00019 0.0103 0.0396 0.0282 0.0256
3R:10715940-10716090:plus -31.4694 -4.11927 2.15596 4.24771 -17.2245 -7.5 -11.7248 -2.30556 1.16514
0.0124 0.0349 0.00545 0.00519 0.00384 0.000614 0.00434 0.0323 0.0135
3R:10716280-10716430:minus -40.449 -3.15596 4.74312 1.40367 -9.27551 -9.30612 -14.6789 -1.90278
-3.08257 0.0402 0.0269 0.00198 0.0184 0.00284 0.00257 0.0084 0.029 0.0455
3R:10716280-10716430:plus -31.1429 3.53211 2.84404 2.57798 -8.89796 -28.3776 -17.3945 -2.38889 5.12844
0.0108 0.00286 0.00421 0.011 0.00189 0.0609 0.0136 0.0331 0.00301
3R:10719640-10719790:minus -31.4082 2.42202 -0.229358 6.16514 -27.1939 -0.173469 -15.3028
-0.125 -8.93578 0.0119 0.0043 0.0125 0.00223 0.0267 0.000456 0.00953 0.0174 0.164
3R:10719640-10719790:plus -30.8163 -1.11009 0.862385 0.486239 -18.5306 -8.79592 -2.49541 5.69444 3.05505
0.0103 0.0148 0.00867 0.0237 0.0118 0.0019 0.0005 0.00191 0.00692
3R:10720180-10720330:minus -22.2959 -1.38532 0.495413 4.12844 1.17347 0.826531 -24.3486 0.0833333
3.66972 0.00335 0.0162 0.00983 0.00549 0.000101 0.000153 0.0432 0.0163 0.00514
3R:10720180-10720330:plus -32.7347 -1.70642 5.33945 6.49541 -19.0102 -27.051 -17.7982 6.05556 -6.50459
0.0225 0.0178 0.00154 0.00176 0.0157 0.0315 0.0145 0.00161 0.101
3R:10724020-10724170:minus -31.4388 13.3878 8.6055 2.83486 20.2041 -17.5 -15.7982 0.444444
0.357798 0.0122 0.0000869 0.000314 0.0097 3.64e-07 0.00599 0.0105 0.0145 0.0166
3R:10724020-10724170:plus -23.8571 2.06422 1.94495 9.59633 -27.7551 9.64286 -25.844 6.34722 -6.30275
0.00687 0.0049 0.0059 0.000275 0.0385 0.0000222 0.0525 0.0014 0.0956
3R:10729760-10729910:minus -23.3367 7.82569 6.90826 5.86239 -18.3776 -17.2755 6.7156 -3.72222
3.72477 0.00614 0.00042 0.000758 0.00282 0.00926 0.00544 0.0000336 0.0461 0.00497
3R:10729760-10729910:plus -23.1837 -0.944954 1.3945 2.66055 -8.96939 -17.2755 -3.7156 -0.0972222
-2.55963 0.00553 0.0141 0.00719 0.0106 0.00226 0.00544 0.000689 0.0172 0.0395
3R:1077060-1077210:minus -20.8878 -0.59633 -3.6789 -0.284404 -18.7143 -28.5612 -18.9083 -0.930556
6.7156 0.0018 0.0126 0.0348 0.0291 0.0136 0.0636 0.0172 0.0221 0.00144
3R:1077060-1077210:plus -10.7755 5.12844 2.34862 3.95413 -17.7143 -18.7143 -3.3578 4.05556 -4.44037
0.000235 0.00154 0.00508 0.00593 0.00627 0.014 0.000624 0.00393 0.063
3R:1077400-1077550:minus -14.4082 9.12844 0.816514 6.6422 -17.8163 0.540816 -21.8349 -0.263889
3.46789 0.00136 0.000209 0.0088 0.00161 0.00682 0.0002 0.0286 0.0181 0.00582
3R:1077400-1077550:plus -31.5102 -1.42202 -1.3945 2 -18.8265 0.530612 -17.5321 2.25 4.49541 0.013

0.0164 0.018 0.0143 0.0149 0.000203 0.0139 0.00788 0.00363
3R:1082420-1082570:minus -23 7.04587 9.66055 3.70642 -9.12245 -27.6327 -6.66055 -4.52778 3.46789
0.00498 0.000633 0.000174 0.0066 0.00238 0.043 0.00155 0.0556 0.00582
3R:1082420-1082570:plus -22.9184 14.3394 2.58716 7.43119 -8.97959 -18.5612 -10.9541 -2.09722 -0.770642
0.00447 5.96e-06 0.00465 0.000939 0.0023 0.0132 0.0037 0.0306 0.0245
3R:1089740-1089890:minus -22.551 -0.0917431 -0.541284 -2.00917 -17.7857 -9.02041 -12.055 3.68056
0.605505 0.00359 0.0106 0.0138 0.0514 0.00676 0.00213 0.00467 0.00457 0.0159
3R:1089740-1089890:plus -30.9694 -1.51376 1.06422 3.88991 -17.9796 -19.2755 -5.62385 -0.847222
6.37615 0.0104 0.0168 0.00808 0.00608 0.00726 0.0185 0.00118 0.0216 0.00159
3R:1090600-1090750:minus -21.8163 -1.98165 9.86239 5.84404 -16.0816 -18.5714 -8.02752 -0.555556
-0.174312 0.00244 0.0193 0.000155 0.00289 0.00294 0.0136 0.00212 0.0198 0.0196
3R:1090600-1090750:plus 15.2551 -3.50459 9.13761 7.55963 -18.1224 -18.7857 -8.49541 -1.58333 2.11009
3.48e-07 0.0296 0.000235 0.000807 0.00782 0.0146 0.00232 0.0266 0.00898
3R:10919780-10919930:minus -22.3367 3.33945 1.38532 2.63303 -17.2245 -27.3367 -7.22936 -2.65278
1.6789 0.00339 0.00307 0.00721 0.0107 0.00384 0.0347 0.00178 0.0354 0.011
3R:10919780-10919930:plus -29.4388 10.1376 2.13761 1.57798 -18.6837 -9.57143 -5.88991 7.06944 1.19266
0.00842 0.000125 0.00549 0.0171 0.0126 0.00326 0.00127 0.00097 0.0134
3R:1092000-1092150:minus -14.5918 0.449541 5.23853 0.669725 -18.7143 -18.0816 -3.19266 2.63889 5.34862
0.00148 0.00882 0.00161 0.0225 0.0136 0.00934 0.000598 0.00684 0.00271
3R:1092000-1092150:plus -21.8878 2.7156 6.6055 6.72477 -26.7449 -18.7857 -22.9358 -6.29167 -3.45872
0.00258 0.00386 0.000872 0.00154 0.0195 0.0146 0.0347 0.0807 0.0492
3R:10920320-10920470:minus -21.9286 1.50459 1.21101 7 -18.449 -27.2653 -0.412844 0.541667
-1.11009 0.0027 0.00602 0.00768 0.00132 0.0107 0.0333 0.000311 0.0141 0.0269
3R:10920320-10920470:plus -31.2551 -2.50459 10.211 3.90826 -9.19388 -18.3469 -16.3945 1.98611 3.3211
0.0113 0.0225 0.000126 0.00603 0.00253 0.0124 0.0116 0.00866 0.00616
3R:10920700-10920850:minus -31.3265 3.64286 -0.559633 7.37615 -27.1939 -9.5 -16.2477 3.94444
3.87156 0.0114 0.00159 0.0139 0.000951 0.0267 0.00294 0.0113 0.00411 0.00477
3R:10920700-10920850:plus -23.1837 1.2844 1.74312 5.43119 -27.3061 -17.9388 -15.9266 -3.34722 11.4771
0.00553 0.00653 0.00635 0.00371 0.0315 0.00738 0.0107 0.0421 0.0000679
3R:10921420-10921570:minus -2.29592 7.26606 3.13761 3.61468 -18.449 -18.5612 -18.8165 4.04167
3.6789 0.000028 0.000565 0.00377 0.0068 0.0107 0.0132 0.017 0.00395 0.00506
3R:10921420-10921570:plus -31.5102 -1.34862 1.31193 7.34862 -28.2347 -18.051 -10.4679 -0.0833333
-2.05505 0.013 0.016 0.00741 0.000998 0.051 0.00886 0.00336 0.0172 0.0338
3R:10954300-10954450:minus -22.7041 2.76147 -0.0275229 5.65138 -18.7143 -9.53061 -18.1927 -4.11111
-6.58716 0.00395 0.0038 0.0117 0.00322 0.0136 0.0031 0.0154 0.0505 0.103
3R:10954300-10954450:plus -11.6327 -4.87156 6.00917 6.19266 -8.93878 -9.65306 -20.945 -0.708333
5.84404 0.000259 0.0427 0.00115 0.00216 0.00216 0.0034 0.0243 0.0207 0.00202
3R:10956340-10956490:minus -42.4388 2.69725 0.642202 1.49541 -19.0918 -17.1224 -19.8257 4.02778
3.08257 0.0766 0.00389 0.00935 0.0176 0.0163 0.00499 0.02 0.00397 0.00681
3R:10956340-10956490:plus -41.4082 0.623853 0.238532 6.00917 -17.602 9.30612 -34.9725 2.38889 -18.2294
0.0557 0.00829 0.0107 0.00255 0.00547 0.0000398 0.16 0.00749 0.551
3R:10956840-10956990:minus -24.0408 0.541284 -2.44954 0.0458716 -26.8571 -8.72449 -18.1284 -1.80556
5.75229 0.00726 0.00854 0.0246 0.0266 0.0198 0.00169 0.0152 0.0283 0.00213
3R:10956840-10956990:plus -32.0714 -4.37615 5.63303 -0.440367 -18.6735 -18.9796 -17.5138 4.76389
11.8532 0.0176 0.0374 0.00136 0.0306 0.0123 0.0155 0.0139 0.00291 0.0000428
3R:10957560-10957710:minus 5.67347 4.90816 11 7.55963 -7.89796 -16.9796 15.4312 -0.319444
7.45872 8.49e-06 0.00023 0.0000802 0.000807 0.000678 0.0044 1.18e-06 0.0184 0.00103
3R:10957560-10957710:plus -22.9592 0.623853 0.623853 2.41284 -26.898 -17.7143 -13.4587 6.47222 3.24771
0.00475 0.00829 0.00941 0.0118 0.0203 0.00634 0.00642 0.00132 0.00636
3R:10960420-10960570:minus -23.4082 -0.311927 1.82569 4.06422 -17.7857 -17.9796 -14.3119 -0.5
-2.89908 0.00623 0.0115 0.00616 0.00564 0.00676 0.00764 0.00777 0.0195 0.0434
3R:10960420-10960570:plus -22.1531 1.6789 3 5.29358 -18.7143 0.826531 -22.0367 0.305556 -0.119266
0.0031 0.00565 0.00397 0.00398 0.0136 0.000153 0.0297 0.0152 0.0192
3R:10962680-10962830:minus -22.102 4.12844 -0.394495 2.25688 -26.8571 -26.8265 -7.70642 -2.43056

1.93578 0.003 0.00229 0.0132 0.0127 0.0198 0.0285 0.00198 0.0334 0.00969
3R:10962680-10962830:plus -13.1122 5.16514 3.46789 9.44954 -18.1939 -26.3061 -9.44037 -4.5 2.21101
0.000632 0.00152 0.00331 0.000326 0.0086 0.0219 0.00278 0.0552 0.00862
3R:10972580-10972730:minus -30.6327 -2.47706 4.95413 -0.422018 -18.4796 -18.9796 -10.0734 -0.763889
-3.99083 0.00968 0.0223 0.00181 0.0305 0.011 0.0155 0.00312 0.0211 0.0556
3R:10972580-10972730:plus -21.4082 0.715596 3.58716 3.80734 -17.6429 -9.7551 -22.4495 4.38889 9.87156
0.00198 0.00802 0.00316 0.00627 0.00562 0.00358 0.0319 0.00342 0.000217
3R:10973260-10973410:minus -12.8878 2.91743 1.68807 9.08257 -18.1531 -8.38776 -20.3303 -2.06944
2.16514 0.000523 0.00358 0.00648 0.000444 0.00829 0.00101 0.0218 0.0304 0.00879
3R:10973260-10973410:plus -31.6633 -3.31193 0.715596 2.12844 10.6531 -7.94898 -20.2385 1.41667 9.61468
0.0141 0.0281 0.00912 0.0134 0.0000102 0.00074 0.0215 0.0106 0.00029
3R:11022200-11022350:minus -33.7755 7.3578 2.6789 6.78899 -19.051 -8.79592 -22.5413 4.65278
0.183486 0.0297 0.000538 0.00449 0.00146 0.0162 0.0019 0.0324 0.00305 0.0176
3R:11022200-11022350:plus -31.6633 8.7156 -2.48624 4.34862 -18.7143 -18.3061 -0.724771 5.51389
1.38532 0.0141 0.00026 0.0249 0.00507 0.0136 0.0114 0.000334 0.00208 0.0123
3R:11022900-11023050:minus -41.2959 -1.59633 1.01835 5.06422 -27.1633 -16.051 -25.8807 3.76389
-5.95413 0.0529 0.0172 0.00821 0.00433 0.025 0.00408 0.0527 0.00442 0.088
3R:11022900-11023050:plus -30.6633 -1.63303 -4.09174 3.94495 -27.4592 -7.94898 -20.8165 -0.486111
-2.59633 0.00976 0.0174 0.0389 0.00595 0.0329 0.00074 0.0238 0.0194 0.0398
3R:11024080-11024230:minus -23.1531 1.81651 -1.3945 6.22936 -26.1939 -8.45918 -17.7706 1.95833
2.37615 0.00542 0.00537 0.018 0.00209 0.0175 0.00111 0.0144 0.00875 0.00825
3R:11024080-11024230:plus -23.2245 3.15596 8.13761 11.1927 -18.4898 -17.898 -16.945 2.01389 3.92661
0.00576 0.00328 0.000407 0.0000254 0.0116 0.00726 0.0127 0.00858 0.00461
3R:11034820-11034970:minus -43.0714 2.90826 -1.70642 3.7156 -19.0204 -8.57143 -24.6514 4.88889
-0.422018 0.0893 0.0036 0.0198 0.00655 0.0158 0.00149 0.045 0.00276 0.0213
3R:11034820-11034970:plus -31.7755 0.587156 -0.247706 2.55046 -8.93878 -27.5612 -23.0092 -1.06944
-3.13761 0.0157 0.0084 0.0126 0.0112 0.00216 0.0393 0.0351 0.023 0.0461
3R:11044920-11045070:minus -34.2245 0.146789 3.06422 0.798165 -27.5306 9.30612 -19.6239 8.90278
3.81651 0.0315 0.0098 0.00387 0.0218 0.0351 0.0000398 0.0193 0.000341 0.00482
3R:11044920-11045070:plus -21.9592 13.3878 14.8257 9.49541 -26.9286 -8.45918 -18.8532 2.36111 -3.07339
0.00279 0.0000869 2.55e-06 0.000308 0.0214 0.00111 0.017 0.00757 0.0454
3R:11048800-11048950:minus -32.2143 -3.38532 3.33028 1.69725 11.4286 1.16327 -20.7248 -0.236111
-1 0.0182 0.0287 0.00349 0.0163 1.97e-06 0.0000831 0.0234 0.018 0.0261
3R:11048800-11048950:plus -23.5612 7.19266 0.201835 -0.798165 -18.5204 -7.79592 8.04587 0.680556
-1.97248 0.00653 0.000587 0.0108 0.0344 0.0117 0.000723 0.0000235 0.0135 0.033
3R:11051120-11051270:minus -20.9592 0.46789 1.82569 5.46789 10.6224 -18.0102 -7.74312 0.583333
-0.541284 0.00184 0.00876 0.00616 0.00362 0.0000113 0.00812 0.00199 0.0139 0.0223
3R:11051120-11051270:plus -31.2143 5.06422 -0.229358 7.63303 -27.1939 -19.3469 -13.8716 4.25 0.963303
0.0111 0.00159 0.0125 0.000745 0.0267 0.0194 0.00705 0.00362 0.0145
3R:11055040-11055190:minus -32.4388 -0.926606 0.229358 2.53211 -18.0816 0.27551 -12.3211 4.34722
-7.11009 0.0195 0.014 0.0107 0.0113 0.00759 0.000257 0.00495 0.00348 0.116
3R:11055040-11055190:plus -40.9184 4.51376 0.0550459 7.61468 -9.19388 9.57143 -19.7156 2.97222
1.58716 0.0443 0.00198 0.0114 0.000779 0.00253 0.0000274 0.0196 0.00603 0.0113
3R:11065920-11066070:minus -32.9592 -0.431193 1.3578 5.82569 -17.4898 -17.5 -11.3853 3.31944
0.908257 0.0252 0.0119 0.00728 0.00291 0.0053 0.00599 0.00404 0.00528 0.0147
3R:11065920-11066070:plus -22.4388 2.93578 4.49541 4.04587 -27.2347 -0.244898 -15.1468 -3 0.825688
0.00348 0.00356 0.00219 0.00572 0.0283 0.000496 0.00924 0.0386 0.0149
3R:11069280-11069430:minus -21.6327 -4.50459 0.330275 3.48624 -17.3776 -17.2041 -20.4312 3.95833
-1.11927 0.00215 0.0387 0.0104 0.0072 0.00419 0.00507 0.0222 0.00409 0.027
3R:11069280-11069430:plus -30.6633 -1.0367 10.6972 7.95413 -17.0816 -9.23469 -11.1927 4.01389 -0.697248
0.00976 0.0145 0.0000948 0.000599 0.00334 0.00238 0.00388 0.004 0.0239
3R:11072640-11072790:minus -31.4796 -2.05505 -1.77064 0.12844 -18.2245 -18.2755 -17.8532 1.91667
2.16514 0.0128 0.0197 0.0202 0.0259 0.00877 0.0112 0.0146 0.00888 0.00879
3R:11072640-11072790:plus -23.449 -1.11927 5.22936 6.00917 -27.2347 -9.7551 -25.6147 0.611111 -2.79817

0.00631 0.0149 0.00162 0.00255 0.0283 0.00358 0.051 0.0138 0.0423
3R:1108700-1108850:minus -24.5204 -4.25688 4.78899 0.155963 -8.60204 -26.8265 -19.5872 -0.402778
1.78899 0.00813 0.0362 0.00194 0.0258 0.00125 0.0285 0.0192 0.0189 0.0103
3R:1108700-1108850:plus -32.4694 -0.816514 8.27523 3.21101 -17.449 -9.42857 -23.3394 -3.43056
-4.65138 0.0196 0.0135 0.000378 0.00822 0.00484 0.0027 0.0371 0.043 0.0664
3R:11089120-11089270:minus -24.2653 14.8716 1.81651 3.84404 -26.9694 -26.602 -5.29358 -1.98611
-0.477064 0.00773 2.8e-06 0.00619 0.00624 0.023 0.0248 0.00108 0.0297 0.0218
3R:11089120-11089270:plus -14.449 13.4592 3.78899 7.99083 -17.7143 -18.0816 -3.44037 5.48611 3.93578
0.0014 0.000064 0.00292 0.00059 0.00627 0.00934 0.000639 0.00211 0.00456
3R:11091440-11091590:minus -22.2245 5.51376 1.21101 7.45872 -18.9388 -19.0102 -14.6972 4.875
1.79817 0.00325 0.00131 0.00768 0.000894 0.0151 0.0159 0.00843 0.00277 0.0102
3R:11091440-11091590:plus -22.6633 -2.7156 4.20183 6.78899 -17.1224 -17.7857 -13.2661 -7.06944 1.74312
0.00384 0.0238 0.00247 0.00146 0.00343 0.0068 0.00614 0.0938 0.0105
3R:11094320-11094470:minus -31.4796 -4.07339 5.07339 5.57798 -6.89796 -8.16327 -22.5046 2.47222
3.19266 0.0128 0.0345 0.00172 0.0034 0.000364 0.000783 0.0322 0.00727 0.00652
3R:11094320-11094470:plus -30.6633 -4.33945 8.57798 3.90826 -7.89796 0.0510204 -6.48624 5.91667
3.41284 0.00976 0.037 0.000318 0.00603 0.000678 0.000349 0.00149 0.00172 0.006
3R:11103840-11103990:minus -20.3265 4.88991 -1.40367 1.74312 10.6939 -9.72449 -13.3028 5.51389
5.0367 0.00165 0.0017 0.0181 0.016 7.35e-06 0.00352 0.0062 0.00208 0.00306
3R:11103840-11103990:plus -21.6327 -1.14679 -0.541284 5.75229 -17.6429 -26.7857 -11.8073 -6.77778
3.52294 0.00215 0.015 0.0138 0.00296 0.00562 0.0266 0.00442 0.0887 0.00558
3R:11104300-11104450:minus -32.5918 -2.77982 1.12844 2.77064 11.4286 -18.5 -15.7339 3.55556
-2.78899 0.021 0.0243 0.00791 0.01 1.97e-06 0.0131 0.0103 0.00481 0.0422
3R:11104300-11104450:plus -32.6224 3.72477 0.146789 4.95413 -28.2653 -18.8571 -12.2661 3.40278 2.88991
0.0211 0.00266 0.011 0.00442 0.0537 0.0151 0.00489 0.00511 0.00725
3R:11112440-11112590:minus -31 6.94495 -0.440367 0.981651 -17.4184 -9.5 -9.13761 2.20833 -2.48624
0.0105 0.000669 0.0134 0.0207 0.00457 0.00294 0.00262 0.008 0.0387
3R:11112440-11112590:plus -22.8469 -0.155963 -1.69725 3.94495 -27.9694 -18.051 -12.5596 -1.26389
11.3853 0.00424 0.0109 0.0197 0.00595 0.0437 0.00886 0.00523 0.0243 0.0000815
3R:11113140-11113290:minus -20.6327 0.385321 0.302752 1.62385 -18.7857 -8.94898 -11.789 2.31944
-2.36697 0.00171 0.00902 0.0105 0.0168 0.0148 0.00203 0.0044 0.00769 0.0371
3R:11113140-11113290:plus -24.0306 1.05505 6.54128 5.69725 -27.2653 -28.898 -11.578 3.65278 2.16514
0.00723 0.00709 0.000901 0.00314 0.0302 0.0713 0.00421 0.00462 0.00879
3R:11113640-11113790:minus -12.1531 4.54128 1.23853 6.82569 -17.6429 -9.37755 -19.5229 1.83333
-2.6789 0.000352 0.00195 0.0076 0.00144 0.00562 0.00265 0.019 0.00914 0.0408
3R:11113640-11113790:plus -33.7347 0.00917431 13.9908 3.23853 20.2041 -18.051 -22.6697 1.34722
-4.86239 0.0294 0.0103 7.33e-06 0.00815 3.64e-07 0.00886 0.0332 0.0108 0.0705
3R:11116540-11116690:minus -23.9592 -5.25688 7.92661 6.16514 -17.4184 -18.0102 -4.26606 -1.73611
7.45872 0.00708 0.0472 0.000455 0.00223 0.00457 0.00812 0.000805 0.0277 0.00103
3R:11116540-11116690:plus -12.7347 4.6422 -2.83486 7.80734 -17.6429 -16.9388 -20.5229 -7.84722 -1.6055
0.000468 0.00188 0.0275 0.00063 0.00562 0.00433 0.0226 0.108 0.0302
3R:11117360-11117510:minus -14.5918 0.0917431 1.2844 7.80734 -7.37755 -9.57143 10.3119 1.375
2.11927 0.00148 0.00999 0.00748 0.00063 0.000398 0.00326 0.0000116 0.0107 0.00889
3R:11117360-11117510:plus -30.4388 -3.52294 5.26606 6.16514 -18.0816 -7.72449 -15.5596 -5.11111 1.16514
0.00923 0.0297 0.00159 0.00223 0.00759 0.00068 0.01 0.0632 0.0135
3R:11118460-11118610:minus -30.5102 -1.05505 1.75229 2.40367 0.612245 -27.5612 -11.2385 -0.875
3.87156 0.00947 0.0146 0.00633 0.0119 0.000269 0.0393 0.00392 0.0218 0.00477
3R:11118460-11118610:plus -21.6224 -2.87156 -2.61468 3.17431 -9.16327 -17.5 -14.7064 2.20833 -3.93578
0.00213 0.0249 0.0259 0.00842 0.00249 0.00599 0.00845 0.008 0.0547
3R:11119740-11119890:minus -32.398 -0.183486 5.01835 2.80734 -26.7449 -38.0306 -12.844 3.61111
6.7156 0.019 0.011 0.00176 0.00985 0.0195 0.18 0.00558 0.0047 0.00144
3R:11119740-11119890:plus -13.6327 0.862385 8.17431 10.8624 -9.19388 -18.7857 -8.16514 2.54167 -4.45872
0.00101 0.00761 0.000399 0.0000687 0.00253 0.0146 0.00218 0.00709 0.0633
3R:11145560-11145710:minus -14.2143 -2.25688 9.34862 5.57798 -17.9286 -27.3367 -4.04587 -1.91667

-0.990826	0.00124	0.0209	0.000207	0.0034	0.0071	0.0347	0.000756	0.0291	0.026
3R:11145560-11145710:plus	-32.6633	-2.24771	2.0367	2.00917	-18.7143	-9.5	-22.578	7.61111	-2.54128
0.0216	0.0209	0.0057	0.0142	0.0136	0.00294	0.0327	0.000726	0.0393
3R:11145900-11146050:minus	-21.8163	-1.09174	1.6055	5.58716	-17.7857	-17.6429	-20.7706	4.22222
1.74312	0.00244	0.0148	0.00667	0.00334	0.00676	0.00619	0.0236	0.00367	0.0105
3R:11145900-11146050:plus	-33.0714	1.95413	-0.972477	-0.614679	-27.5612	0.0510204	-9.12844	4.375
5.23853	0.0267	0.0051	0.0158	0.0324	0.0354	0.000349	0.00262	0.00344	0.00285
3R:11153700-11153850:minus	-32.5204	0.0183486	5.43119	0.926606	-8.57143	-17.051	-19.5963	3.34722
9.3945	0.0204	0.0102	0.00148	0.0209	0.00113	0.00479	0.0192	0.00522	0.000358
3R:11153700-11153850:plus	-31.8469	-4.24771	5.26606	3.05505	-17.7551	-18.1224	-11.3211	3.08333	4.7156
0.0162	0.0362	0.00159	0.00878	0.00658	0.00988	0.00399	0.00578	0.00345
3R:11158880-11159030:minus	-14.1429	13.3878	-1.21101	5.90826	-26.9286	-16.9388	-21.1009	10.4306
5.9633	0.0012	0.0000869	0.017	0.00271	0.0214	0.00433	0.025	0.000119	0.00188
3R:11158880-11159030:plus	-29.551	5.47706	-0.706422	3.76147	-18.0204	-8.72449	-9.24771	2.65278
0.816514	0.00845	0.00133	0.0146	0.00645	0.00733	0.00169	0.00268	0.0068	0.015
3R:11166260-11166410:minus	-30.3673	1.86239	4.21101	11.2936	-18.3469	-8.93878	-22.4679	3.08333
-1.50459	0.00897	0.00528	0.00246	0.0000139	0.00894	0.00201	0.032	0.00578	0.0294
3R:11166260-11166410:plus	-31.6735	8.15596	-0.211009	6.57798	-27.4898	-10.0204	-21.8899	4.58333
0.183486	0.0141	0.000351	0.0124	0.00167	0.0336	0.00399	0.0289	0.00315	0.0176
3R:11168620-11168770:minus	5.11224	-3.59633	2.69725	3.3578	-17.4898	-8.64286	0.889908	1.88889
2.58716	0.0000151	0.0303	0.00446	0.00767	0.0053	0.00154	0.000227	0.00896	0.00779
3R:11168620-11168770:plus	-22.4898	0.963303	3.73394	1.6055	-26.7449	9.37755	-16.7523	4.47222	-2.44037
0.00353	0.00734	0.00299	0.0169	0.0195	0.0000363	0.0123	0.0033	0.0381
3R:11169180-11169330:minus	-32.8469	0.908257	12.367	2.04587	-18.1939	-7.65306	-18.3303	0.944444
9.55046	0.0243	0.00748	0.0000334	0.014	0.0086	0.000647	0.0157	0.0124	0.000321
3R:11169180-11169330:plus	-32.8163	-1.14679	12.0459	7.2844	-18.6837	-18.1633	-22.6055	3.875	1.48624
0.024	0.015	0.0000414	0.00105	0.0126	0.01	0.0328	0.00423	0.0118
3R:11171860-11172010:minus	-24.2959	15.1376	-3.90826	1.36697	-17.4898	-7.42857	-5.76147	2.95833
-0.605505	0.00781	1.81e-06	0.037	0.0186	0.0053	0.000567	0.00123	0.00606	0.023
3R:11171860-11172010:plus	-23	5.6422	2.6422	5.47706	-18.4898	-19.2041	-14.3303	-0.958333	5.34862
0.00498	0.00124	0.00455	0.0036	0.0116	0.0179	0.0078	0.0223	0.00271
3R:11173700-11173850:minus	-32.7755	1.07339	4.83486	2.69725	-8.7449	-19.051	-16.3303	-2.65278
-0.174312	0.0234	0.00705	0.00191	0.0104	0.00161	0.0165	0.0115	0.0354	0.0196
3R:11173700-11173850:plus	-30.5918	-2.36697	0.155963	2.62385	-27.4592	-17.7857	-6.42202	1.59722	5.3945
0.00963	0.0216	0.011	0.0108	0.0329	0.0068	0.00146	0.00993	0.00263
3R:11176800-11176950:minus	-33.5102	0	15.6606	-0.284404	-17.3776	-27.6735	-17.2752	4.95833
-0.504587	0.0286	0.0103	3.94e-07	0.0291	0.00419	0.0443	0.0134	0.00267	0.0221
3R:11176800-11176950:plus	-31.6327	6.66055	1.3211	2.30275	-8.93878	-26.3061	-20.9266	4.11111	6.04587
0.0138	0.000771	0.00738	0.0124	0.00216	0.0219	0.0243	0.00384	0.00181
3R:11182580-11182730:minus	-31.898	-2.86239	1.83486	6.6422	0.836735	-18.051	-17.4312	1.44444
1.79817	0.0164	0.0248	0.00614	0.00161	0.000215	0.00886	0.0137	0.0105	0.0102
3R:11182580-11182730:plus	-30.5204	3.90816	0.678899	3.40367	-17.8571	-17.3469	-18.5596	0.0138889
-0.743119	0.00951	0.00102	0.00923	0.00759	0.00698	0.0058	0.0163	0.0167	0.0243
3R:11187180-11187330:minus	-22.9286	-4.18349	-1.77064	2.98165	-8.71429	-18.1224	-7.01835	2	-1.9633
0.0046	0.0355	0.0202	0.00905	0.00159	0.00988	0.00169	0.00862	0.0329
3R:11187180-11187330:plus	-22.7347	15.6514	5.75229	5.3945	-9.5	-9.82653	-18.367	1.51389	-4.75229
0.00403	8.05e-07	0.00129	0.00379	0.00293	0.00381	0.0158	0.0102	0.0683
3R:11189520-11189670:minus	-31.6735	-0.293578	-0.963303	2.09174	-19.2755	-17.3469	-22.4495
4.97222	-4.6422	0.0141	0.0114	0.0158	0.0137	0.0164	0.0058	0.0319	0.00266	0.0662
3R:11189520-11189670:plus	-40.7449	4.86239	-2.11009	1.66972	-28.2653	-8.79592	-11.4771	3.41667	-2.52294
0.0431	0.00172	0.0223	0.0165	0.0537	0.0019	0.00412	0.00508	0.0391
3R:11193280-11193430:minus	-13.449	1.16514	0.733945	0.357798	-17.4082	-8.65306	-24.4587	7.73611
-2.83486	0.000914	0.00682	0.00906	0.0245	0.00421	0.00156	0.0438	0.000677	0.0427
3R:11193280-11193430:plus	-32.5102	13.8532	-0.348624	0.00917431	-18.9796	-17.051	-9.47706	3.05556

4.30275 0.0202 9.88e-06 0.013 0.0269 0.0155 0.00479 0.00279 0.00584 0.00388
3R:11197600-11197750:minus -24.3265 13.5306 14.1927 7.29358 -18.4184 1.05102 -2 3.33333 -1.36697
0.00784 0.0000206 5.79e-06 0.00104 0.0101 0.000107 0.000444 0.00525 0.0286
3R:11197600-11197750:plus -24.449 10.7706 1.74312 4.95413 -27.2653 -18.3469 -7.78899 8.70833 -2.88991
0.00802 0.0000902 0.00635 0.00442 0.0302 0.0124 0.00201 0.000384 0.0433
3R:1123200-1123350:minus -32.0408 -1.0367 6.62385 2.88991 20.4694 -8.57143 -16.7064 10.6528 0.12844
0.0174 0.0145 0.000865 0.00942 1.65e-07 0.00149 0.0122 0.000101 0.0179
3R:1123200-1123350:plus -31.7755 -1.49541 4.72477 -1.50459 -17.4898 -18.1224 -25.7248 3.06944 0.816514
0.0157 0.0167 0.00199 0.0434 0.0053 0.00988 0.0517 0.00581 0.015
3R:11234100-11234250:minus -13.1837 4.80734 0.954128 7.70642 -26.2653 -18.4184 5.21101 11.625
0.816514 0.000705 0.00176 0.0084 0.000708 0.018 0.0127 0.0000536 0.0000441 0.015
3R:11234100-11234250:plus -23.7041 7.20183 -1.88991 5.43119 -8.93878 -35.8163 -10.5229 -0.722222
2.11009 0.00669 0.000584 0.0209 0.00371 0.00216 0.0815 0.0034 0.0208 0.00898
3R:11235080-11235230:minus -13.1224 -3.33028 -1.46789 2.7156 -27.2653 -26.5306 -11.6697 -1.27778 3
0.000633 0.0282 0.0184 0.0102 0.0302 0.023 0.00429 0.0244 0.00702
3R:11235080-11235230:plus -13.7041 2.30275 0.66055 3.76147 -18.0816 -28.3367 -12.6881 2.56944 5.65138
0.00104 0.00449 0.00929 0.00645 0.00759 0.0606 0.00538 0.00701 0.00228
3R:11235480-11235710:minus -31.5612 1.73394 2.82569 7.27523 -17.7143 -18.1224 -10.9908 4.5 6.11927
0.0134 0.00553 0.00424 0.00109 0.00627 0.00988 0.00373 0.00326 0.00178
3R:11235480-11235710:plus -24.2551 1.85321 -1.08257 3.78899 1.10204 -35.449 -8.12844 4.5 1.16514
0.00768 0.0053 0.0164 0.00632 0.000124 0.0789 0.00216 0.00326 0.0135
3R:11236140-11236290:minus -32.9286 4.16514 -1.27523 3.87156 -17.9388 -16.2755 -19.6697 -0.208333
-5.94495 0.025 0.00226 0.0174 0.00618 0.00715 0.00413 0.0195 0.0178 0.0878
3R:11236140-11236290:plus -20.6633 -1.37615 -2.99083 5.65138 -17.7143 -9.7551 -16.6697 6.63889 -2.10092
0.00173 0.0161 0.0288 0.00322 0.00627 0.00358 0.0122 0.00121 0.0342
3R:1124780-1124930:minus -31.5918 2.65138 3.38532 6.00917 -26.5306 -18.051 -16.2385 3.40278 -0.100917
0.0136 0.00395 0.00342 0.00255 0.0185 0.00886 0.0113 0.00511 0.019
3R:1124780-1124930:plus -23.449 -0.321101 -4.55046 3.79817 -18.5306 -7.79592 -15.3028 5.31944
2.16514 0.00631 0.0115 0.044 0.00629 0.0118 0.000723 0.00953 0.00227 0.00879
3R:1126620-1126770:minus -21.9592 -0.486239 -6.30275 6.16514 -17.4184 -9.0102 -17.9725 3.875
3.0367 0.00279 0.0121 0.0681 0.00223 0.00457 0.00208 0.0149 0.00423 0.00694
3R:1126620-1126770:plus -32.7041 0.137615 2.34862 7.27523 -18.4184 -18.3469 -16.8073 3.54167 -4.51376
0.0222 0.00984 0.00508 0.00109 0.0101 0.0124 0.0124 0.00483 0.064
3R:1128840-1128990:minus -31.5102 4.2844 2.77064 7.27523 -8.86735 -17.3469 -14.789 -2.06944 3.77982
0.013 0.00216 0.00433 0.00109 0.00173 0.0058 0.00859 0.0304 0.00489
3R:1128840-1128990:plus -24.3673 -0.440367 8.20183 5.22936 -17.449 -18.3469 -8.69725 0.930556
-2.0367 0.00791 0.012 0.000393 0.0041 0.00484 0.0124 0.00242 0.0124 0.0336
3R:11299680-11299830:minus -23.9286 10.156 -1.86239 3.46789 -27.3367 -17.051 -12.6972 2.94444
3.45872 0.00701 0.000124 0.0208 0.00726 0.0317 0.00479 0.00539 0.00609 0.00591
3R:11299680-11299830:plus -4.71429 1.36697 2.70642 10.0275 -18.1531 1.09184 1.0367 -1.48611 1.12844
0.000187 0.00633 0.00444 0.000156 0.00829 0.000091 0.000218 0.0259 0.0137
3R:11301900-11302050:minus -21.6224 3.11009 11.6239 10.1468 -18.2245 -27.6735 -1.79817 -7.23611
5.90826 0.00213 0.00334 0.0000551 0.000128 0.00877 0.0443 0.000424 0.0967 0.00198
3R:11301900-11302050:plus -31.9286 6.42202 -2.91743 7.46789 -26.5306 -17.8571 -21.8165 2.06944 3.92661
0.0166 0.000867 0.0282 0.000868 0.0185 0.00719 0.0285 0.00841 0.00461
3R:11302140-11302290:minus -21.6224 2.26606 11.6239 5.33028 -26.9286 -18.0816 -17.7798 5.86111
8.10092 0.00213 0.00455 0.0000551 0.00389 0.0214 0.00934 0.0145 0.00177 0.000623
3R:11302140-11302290:plus -21.7755 -1.13761 1.23853 3.92661 -18.7143 -28.1224 -6.31193 6.16667 11.3853
0.00241 0.015 0.0076 0.00601 0.0136 0.0583 0.00142 0.00153 0.0000815
3R:11311680-11311830:minus -24 0.431193 1.14679 2.62385 20.4694 -9.57143 -25.6422 4.375 3.51376
0.00719 0.00887 0.00785 0.0108 1.65e-07 0.00326 0.0512 0.00344 0.00566
3R:11311680-11311830:plus -33.8061 3.7551 -2.2844 2.55046 -8.60204 -19.051 -15.4587 3.54167 0.816514
0.0298 0.00122 0.0235 0.0112 0.00125 0.0165 0.00982 0.00483 0.015
3R:11334580-11334730:minus -2.10204 3.27523 8.37615 2.83486 -27.2347 -17.7551 2.40367 -2.38889

1.12844	0.0000252	0.00314	0.000358	0.0097	0.0283	0.00643	0.000145	0.0331	0.0137
3R:11334580-11334730:plus	-31.7041	3.68367	-3.02752	-1.33028	-9.20408	-8.57143	-30.2294	3.68056	-4.34862	0.0146	0.00144	0.0291	0.0409	0.00262	0.00149	0.0939	0.00457	0.0612
3R:1133860-1134010:minus	-13.1837	-2.06422	3.80734	10.5872	-18.449	-26.2653	-9.98165	1.98611	7.84404	0.000705	0.0198	0.0029	0.000115	0.0107	0.0218	0.00307	0.00866	0.000701
3R:1133860-1134010:plus	-13.8776	0.275229	3.62385	3.01835	-17.449	-18.4898	6.06422	8.51389	5.13761	0.0011	0.00938	0.00312	0.0089	0.00484	0.0129	0.0000406	0.000433	0.00298
3R:11363080-11363230:minus	-12.7755	-0.752294	0.00917431	11.1927	-17.3776	-18.3776	-3.37615	11.5694	1.68807	0.000477	0.0132	0.0116	0.0000254	0.00419	0.0125	0.000628	0.0000463	0.0109
3R:11363080-11363230:plus	-24.2959	2.40367	-0.321101	4.31193	-7.89796	-18.3061	-14.6789	2.20833	-7.73394	0.00781	0.00433	0.0129	0.00516	0.000678	0.0114	0.0084	0.008	0.129
3R:11373420-11373570:minus	-11.6939	-2.91743	-1.77064	11.0183	-17.449	-26.5612	-7.88991	-0.277778	2.16514	0.000261	0.0252	0.0202	0.000052	0.00484	0.0237	0.00206	0.0182	0.00879
3R:11373420-11373570:plus	-33.7755	1.69725	5.3578	3.0367	-18.7143	-17.8571	-15.7615	1.70833	5.84404	0.0297	0.00561	0.00153	0.00886	0.0136	0.00719	0.0104	0.00955	0.00202
3R:11373960-11374110:minus	-32.398	-1.55046	-1.07339	2.22018	-26.1531	0.612245	-8.95413	3.375	1.27523	0.019	0.017	0.0163	0.0129	0.0172	0.000187	0.00254	0.00516	0.013
3R:11373960-11374110:plus	-32.8163	1.13761	-3.45872	5.31193	-27.602	-18.9796	-10.2018	2	-0.440367	0.024	0.00689	0.0328	0.00393	0.0364	0.0155	0.0032	0.00862	0.0216
3R:1143500-1143650:minus	-22.6327	0.266055	0.431193	3.56881	-17.7143	-8.5	-28.0367	3.75	-5.93578	0.00373	0.0094	0.01	0.00699	0.00627	0.00126	0.0691	0.00445	0.0877
3R:1143500-1143650:plus	-24.2551	1.84404	1.22936	1.66972	-27.3469	-19.0816	-10.1927	1.81944	9.04587	0.00768	0.00532	0.00763	0.0165	0.0318	0.017	0.00319	0.00919	0.000423
3R:11451840-11451990:minus	-21.7449	-3.07339	1.91743	-0.678899	-18.1939	-25.898	-16.6972	10.625	11.8532	0.00236	0.0263	0.00596	0.0332	0.0086	0.0214	0.0122	0.000103	0.0000428
3R:11451840-11451990:plus	-32.7041	0.954128	4.6422	7.17431	-17.6429	-9.34694	-2.14679	6.63889	0.394495	0.0222	0.00736	0.00206	0.00115	0.00562	0.00261	0.00046	0.00121	0.0166
3R:11477400-11477550:minus	-31.3265	2.61468	-1.17431	6.00917	1.65306	-19.051	-10.7982	5.41667	0.394495	0.0114	0.004	0.0169	0.00255	0.0000586	0.0165	0.00359	0.00218	0.0166
3R:11477400-11477550:plus	-23.0408	1.13761	-0.440367	2.9633	-17.4184	-16.9388	-20.844	-4.31944	-4.54128	0.00512	0.00689	0.0134	0.00914	0.00457	0.00433	0.0239	0.053	0.0645
3R:11491840-11491990:minus	-31.1429	1.74312	3.68807	7.47706	-18.0816	-8.23469	-18.6514	-1.44444	-3.00917	0.0108	0.00552	0.00304	0.00085	0.00759	0.000875	0.0165	0.0256	0.0447
3R:11491840-11491990:plus	-23.6633	-2.22936	1.73394	11.0183	-18.1122	-9.45918	-1.26606	-1.29167	1.38532	0.00665	0.0208	0.00638	0.000052	0.00771	0.00279	0.000376	0.0245	0.0123
3R:1149600-1149750:minus	-12.4082	6.3211	5.07339	9.33028	-17.449	-17.9388	17.6881	-2.04167	0.908257	0.000414	0.000909	0.00172	0.000374	0.00484	0.00738	5.43e-07	0.0301	0.0147
3R:1149600-1149750:plus	-22.7347	5.63303	5.78899	3.05505	-27.0102	-26.051	-7.63303	2.54167	7.63303	0.00403	0.00125	0.00127	0.00878	0.0237	0.0216	0.00195	0.00709	0.000928
3R:11503420-11503570:minus	-22.449	0.0642202	7.0367	3.3578	-17.6429	-27.5306	7.52294	2.58333	5.75229	0.0035	0.0101	0.000715	0.00767	0.00562	0.0374	0.000027	0.00698	0.00213
3R:11503420-11503570:plus	-21.398	3.97959	0.577982	2.33028	-17.3776	-8.45918	-18.6972	1.43056	-1.59633	0.00197	0.000669	0.00955	0.0123	0.00419	0.00111	0.0166	0.0105	0.0301
3R:11618340-11618490:minus	-23.9184	-1.45872	1.42202	2.29358	-16.1531	-18.1224	-0.376147	-2.11111	-0.431193	0.00695	0.0165	0.00712	0.0124	0.00297	0.00988	0.000309	0.0307	0.0214
3R:11618340-11618490:plus	-30.7755	-0.697248	1.40367	9.59633	-8.53061	-9.45918	-29.8624	-1.51389	-1.22936	0.0102	0.013	0.00717	0.000275	0.0011	0.00279	0.0892	0.0261	0.0279
3R:11619340-11619490:minus	-23.2959	13.5306	5.15596	4.05505	-18.9796	-8.30612	-11.4771	4.48611	2.66972	0.00605	0.0000206	0.00167	0.00566	0.0155	0.000973	0.00412	0.00328	0.00764
3R:11619340-11619490:plus	-41.2143	4.12844	1.09174	6.37615	-27.2653	-36.1122	-14.0092	4.13889	5.6055	0.0494	0.00229	0.00801	0.00192	0.0302	0.0878	0.00727	0.0038	0.00232
3R:11620140-11620290:minus	-34.1122	-2.93578	2.33945	0.0275229	-8.96939	-18.8571	-26.2844	6.58333	0.183486	0.0312	0.0253	0.0051	0.0267	0.00226	0.0151	0.0554	0.00124	0.0176
3R:11620140-11620290:plus	-32.8469	-1.63303	0.385321	2	-28.602	-9.86735	-14	6.34722	4.61468	0.0243

0.0174 0.0102 0.0143 0.0631 0.00391 0.00725 0.0014 0.00351
3R:11629380-11629530:minus -14.602 9.85321 6.72477 7.27523 -17.4184 -16.7857 -14.8807 2.375
-4.84404 0.00149 0.000144 0.000825 0.00109 0.00457 0.00426 0.00876 0.00753 0.0701
3R:11629380-11629530:plus -32.8469 8.04587 5.91743 -0.412844 -17.4184 -8.38776 -20.2752 1.20833
1.3211 0.0243 0.000373 0.0012 0.0304 0.00457 0.00101 0.0216 0.0113 0.0127
3R:11629820-11629970:minus -30.6327 0.12844 -0.192661 6.21101 1.10204 -8.57143 -9.26606 3
5.33028 0.00968 0.00987 0.0123 0.00213 0.000124 0.00149 0.00269 0.00597 0.00273
3R:11629820-11629970:plus 14.398 7.11009 3.22018 7.62385 -26.7041 0.244898 4.01835 1.47222 2.36697
1.3e-06 0.000613 0.00365 0.000759 0.0193 0.000285 0.000082 0.0104 0.00828
3R:11660320-11660470:minus -31.2143 9.33945 -1.33945 2.6789 20.2041 -18.3469 3.70642 3.26389
-0.183486 0.0111 0.000188 0.0177 0.0105 3.64e-07 0.0124 0.0000918 0.00539 0.0197
3R:11660320-11660470:plus -23.9286 8.18349 2.10092 8.05505 -18.7551 -9.30612 -17.4954 4.69444 -1.88991
0.00701 0.000345 0.00556 0.000578 0.0144 0.00257 0.0138 0.003 0.0322
3R:11662840-11662990:minus -40.4796 7.56881 3.6055 5.29358 -17.4898 0.0510204 -6.49541 2.23611
3.88073 0.0407 0.000481 0.00314 0.00398 0.0053 0.000349 0.00149 0.00792 0.00475
3R:11662840-11662990:plus -34.2245 14.1468 -2.29358 1.66972 -17.6735 -17.2755 -27.8899 8.61111 -4.61468
0.0315 7.12e-06 0.0236 0.0165 0.00572 0.00544 0.0678 0.000408 0.0658
3R:11677880-11678030:minus -31.5102 0.477064 12.7706 0.0366972 -17.0408 -27.7551 -15.5413 -2.98611
-3.83486 0.013 0.00873 0.0000251 0.0266 0.00329 0.0458 0.00997 0.0385 0.0533
3R:11677880-11678030:plus -22.1531 -2.22018 6.76147 9.59633 -17.1939 0.0204082 -21.4587 1.23611
3.98165 0.0031 0.0207 0.000811 0.000275 0.00377 0.000377 0.0267 0.0112 0.00439
3R:11679220-11679370:minus -14.0714 -3.17431 2.49541 9.49541 -18.4184 -27.3367 15.2752 -4.68056
6.73394 0.00117 0.027 0.00481 0.000308 0.0101 0.0347 1.25e-06 0.0575 0.00143
3R:11679220-11679370:plus -31.1429 3.64286 8.88991 3.89908 -17.0816 -17.6429 -15.1835 8.86111 -4.22936
0.0108 0.00159 0.00027 0.00604 0.00334 0.00619 0.00931 0.000349 0.0588
3R:11683240-11683390:minus -13.7041 -2.22936 -1.33028 6.72477 -8.44898 -9.53061 -1.48624 1.56944
-0.917431 0.00104 0.0208 0.0177 0.00154 0.00109 0.0031 0.000395 0.01 0.0256
3R:11683240-11683390:plus -24.2245 7.91743 5.68807 5.06422 -18.449 -18.6429 -18.5963 4.55556 -0.174312
0.00763 0.000399 0.00133 0.00433 0.0107 0.0139 0.0164 0.00318 0.0196
3R:11727080-11727230:minus 14.6224 -5.12844 2.04587 9.49541 -17.2245 -18.7143 2.23853 12.25
0.697248 1.14e-06 0.0456 0.00568 0.000308 0.00384 0.014 0.000153 0.0000241 0.0154
3R:11727080-11727230:plus -20.8163 -5.29358 3.26606 1.75229 -8.71429 -17.7551 -11.1468 3.69444 7.53211
0.00177 0.0476 0.00358 0.0159 0.00159 0.00643 0.00385 0.00455 0.000968
3R:11759480-11759630:minus -21.6224 -0.926606 0.733945 6.86239 -17.9796 -9.23469 -10.8624 3.36111
6.95413 0.00213 0.014 0.00906 0.00136 0.00726 0.00238 0.00363 0.00519 0.00136
3R:11759480-11759630:plus -31.102 14.2477 15.0367 7.29358 -26.9286 -0.0918367 -22.0367 6.95833
1.27523 0.0106 6.21e-06 1.36e-06 0.00104 0.0214 0.000442 0.0297 0.00103 0.013
3R:11760720-11760870:minus -22.2653 -1.94495 14.8532 1.21101 -26.8571 0.755102 -9.70642 1.125
-1.57798 0.00331 0.0191 2.28e-06 0.0194 0.0198 0.000175 0.00291 0.0117 0.0299
3R:11760720-11760870:plus -23 2.99083 14.5505 7.37615 -17.1939 -18.9796 -11.2385 -2.55556 1.85321
0.00498 0.00349 3.58e-06 0.000951 0.00377 0.0155 0.00392 0.0345 0.01
3R:11789480-11789630:minus -32.0408 5.3945 13.9174 2.27523 1.94898 -18.051 -1.42202 2.52778
-5.76147 0.0174 0.00138 8.08e-06 0.0125 0.0000358 0.00886 0.00039 0.00712 0.0844
3R:11789480-11789630:plus -32 9.79817 -0.266055 4.08257 -9.23469 -9.53061 -23.7615 1.55556 -2.3945
0.0171 0.000148 0.0126 0.00558 0.00277 0.0031 0.0396 0.0101 0.0375
3R:11791040-11791190:minus -22.3367 14.4312 4.79817 1.78899 -17.6429 -9.09184 -15.7982 2.61111
-0.908257 0.00339 5.39e-06 0.00193 0.0158 0.00562 0.00219 0.0105 0.00691 0.0255
3R:11791040-11791190:plus -34 10.156 -2.75229 7.00917 -8.93878 -17.2755 -14.9633 3.34722 1.48624
0.0307 0.000124 0.0269 0.00126 0.00216 0.00544 0.00891 0.00522 0.0118
3R:11798640-11798830:minus -22.9286 8.97248 -1.76147 2.50459 -28.3061 -17.2347 -5.20183 6.43056
-0.0733945 0.0046 0.000227 0.0201 0.0114 0.0553 0.00511 0.00105 0.00134 0.0188
3R:11798640-11798830:plus -22.2857 0.587156 5.88991 3.51376 10.3878 -19.051 -20.2661 -1.70833 -2.99083
0.00331 0.0084 0.00121 0.00709 0.0000174 0.0165 0.0216 0.0275 0.0445
3R:11798900-11799050:minus -22.5204 14.5688 0.770642 4.08257 -27.2653 -8.79592 -8.34862 4.22222

3.9633 0.00356 4.39e-06 0.00895 0.00558 0.0302 0.0019 0.00226 0.00367 0.00446
3R:11798900-11799050:plus -22.2857 2.72477 3.70642 2.02752 -17.8163 -27.4082 -15.5138 10.9722 -0.174312
0.00331 0.00385 0.00302 0.0141 0.00682 0.0356 0.00992 0.0000781 0.0196
3R:11799460-11799610:minus -22.9184 0.119266 6.69725 6.01835 -17.4184 -9.30612 -12.5596 3.11111
2.2844 0.00447 0.0099 0.000836 0.00248 0.00457 0.00257 0.00523 0.00572 0.00842
3R:11799460-11799610:plus -23.8878 0.0183486 -2.89908 -1.02752 -26.4898 -27.602 -13.9083 3.70833
11.8532 0.00692 0.0102 0.028 0.037 0.0183 0.0416 0.00711 0.00452 0.0000428
3R:11800520-11800670:minus -33.3776 6.24771 1.73394 0.651376 -18.6429 -16.2755 -21.8991 5.31944
0.93578 0.0282 0.000942 0.00638 0.0226 0.0121 0.00413 0.029 0.00227 0.0145
3R:11800520-11800670:plus -40.1429 0.724771 -1.04587 2.55963 -18.4898 -18.6429 -22.6697 6.59722 2.15596
0.0363 0.008 0.0162 0.0111 0.0116 0.0139 0.0332 0.00124 0.00881
3R:11801000-11801150:minus -40.5204 -1.11009 2.58716 1.14679 1.65306 -8.27551 -26.7248 7.44444
0.724771 0.0413 0.0148 0.00465 0.0198 0.0000586 0.000919 0.0585 0.000795 0.0153
3R:11801000-11801150:plus -24.1837 3.90816 -2.87156 4.22936 -19.0204 -8.23469 -15.8165 0.361111 5.70642
0.00751 0.00102 0.0278 0.00525 0.0158 0.000875 0.0105 0.0149 0.00222
3R:1180600-1180750:minus -33.5306 0.697248 -2.65138 4.49541 10.3571 -7.72449 -25.1376 -0.0694444
-6.15596 0.0287 0.00807 0.0261 0.00487 0.0000208 0.00068 0.048 0.0171 0.0923
3R:1180600-1180750:plus -31.4796 0.651376 0.412844 1.74312 10.9898 9.82653 -20.7706 -3.26389 3.45872
0.0128 0.00821 0.0101 0.016 3.28e-06 0.0000148 0.0236 0.0413 0.00591
3R:11809940-11810090:minus -3.59184 0.0733945 1.33028 -0.0275229 -18.3776 -9.02041 -11.8165
-1.65278 1.05505 0.0000835 0.0101 0.00736 0.027 0.00926 0.00213 0.00443 0.0271 0.014
3R:11809940-11810090:plus -13.1122 -1.59633 1.55046 4.08257 -17.7143 -26.2653 -5.55963 0.25 0.0458716
0.000632 0.0172 0.0068 0.00558 0.00627 0.0218 0.00116 0.0155 0.0182
3R:11810560-11810710:minus -3.40816 -4.76147 0.385321 4.74312 -8.71429 -36.5918 -11.9266
4.61111 9.66055 0.0000685 0.0414 0.0102 0.00462 0.00159 0.111 0.00454 0.00311 0.000268
3R:11810560-11810710:plus -30.7449 4.44954 8.48624 6.19266 -18.9796 -0.173469 -31.3578
-3.72222 -14.055 0.0101 0.00202 0.000336 0.00216 0.0155 0.000456 0.109 0.0461 0.353
3R:11812340-11812490:minus -30.1735 6.44037 0.220183 6.3578 -7.37755 -17.7143 -10.1743
0.333333 6.04587 0.00869 0.000858 0.0108 0.00195 0.000398 0.00634 0.00318 0.0151 0.00181
3R:11812340-11812490:plus -20.8469 4.73394 0.366972 7.70642 -8.93878 -27.7857 11.0275
0.555556 4.91743 0.00178 0.00181 0.0103 0.000708 0.00216 0.0466 8.71e-06 0.014 0.00321
3R:11813100-11813250:minus -14.1837 4.40367 1.22018 2.07339 -18.1939 -18.7143 -4.33945
3.08333 1.23853 0.00124 0.00206 0.00765 0.0138 0.0086 0.014 0.000822 0.00578 0.0132
3R:11813100-11813250:plus -34.2245 1.00917 2.33945 5.93578 -18.4898 -18.7857 -25.4862
6.01389 -3.76147 0.0315 0.00722 0.0051 0.00264 0.0116 0.0146 0.0502 0.00165 0.0523
3R:11817520-11817670:minus -32.551 1.88991 2.53211 -0.541284 -18.1224 -17.9796 -17.1101
-3.69444 2.21101 0.0207 0.00523 0.00475 0.0316 0.00782 0.00764 0.013 0.0458 0.00862
3R:11817520-11817670:plus -14.5612 4.02752 2.80734 2.88991 -27.2245 -18.7143 1.42202 -0.555556
5.6055 0.00147 0.00238 0.00427 0.00942 0.0272 0.014 0.000196 0.0198 0.00232
3R:11819000-11819150:minus -22.2347 3.40367 4.77982 3.93578 -17.3469 -8.42857 -17.3578
-5.45833 2.37615 0.00325 0.003 0.00195 0.00597 0.00394 0.00104 0.0136 0.068 0.00825
3R:11819000-11819150:plus -42.551 4.55963 2.41284 0.366972 -28.2653 0.0204082 -16.7523 6
-0.504587 0.0803 0.00194 0.00496 0.0244 0.0537 0.000377 0.0123 0.00166 0.0221
3R:11819660-11819810:minus -22.1224 0.513761 4.19266 2.93578 -8.96939 -18.3469 -10.1101
-5.44444 3.20183 0.00304 0.00862 0.00248 0.00927 0.00226 0.0124 0.00314 0.0678 0.0065
3R:11819660-11819810:plus -22.4082 1.3211 2.27523 9.33028 -18.7857 -8.45918 -9.83486
0.597222 0.550459 0.00345 0.00644 0.00522 0.000374 0.0148 0.00111 0.00298 0.0139 0.0161
3R:11820220-11820370:minus -21.8878 3.61468 0.825688 7.80734 -17.8571 -18.0102 -13.2569
7.09722 -3.51376 0.00258 0.00277 0.00878 0.00063 0.00698 0.00812 0.00613 0.000956 0.0498
3R:11820220-11820370:plus -2.70408 2.30275 6.6055 10.8624 -16.3776 -27.8571 6.11009 -0.152778
1.44037 0.0000372 0.00449 0.000872 0.0000687 0.003 0.0508 0.0000401 0.0175 0.012
3R:11830780-11830930:minus -32.4796 -1.66972 -3.25688 -1.84404 -26.8878 -9.72449
-27.0642 -0.458333 1.76147 0.02 0.0176 0.031 0.0485 0.0199 0.00352 0.061 0.0192 0.0104
3R:11830780-11830930:plus -22.7041 1.91743 5.41284 5.42202 -18.5306 -8.72449 -17.4679

-1.56944 -0.0733945 0.00395 0.00518 0.00149 0.00374 0.0118 0.00169 0.0138 0.0265 0.0188
3R:11832000-11832150:minus -30.9286 8.17431 -0.211009 3.23853 -27.2653 9.23469 -14.6514
8.80556 -5.02752 0.0104 0.000347 0.0124 0.00815 0.0302 0.0000427 0.00835 0.000362 0.0732
3R:11832000-11832150:plus -29.8776 1.15596 1.18349 2.85321 -17.3776 -8.23469 -17.2936
0.680556 -8.7156 0.00852 0.00684 0.00775 0.00955 0.00419 0.000875 0.0134 0.0135 0.157
3R:1183840-1183990:minus -38.8776 2.93578 -0.495413 5.73394 10.4286 -9.02041 -17.8165
1.94444 1.23853 0.0322 0.00356 0.0136 0.00307 0.0000139 0.00213 0.0145 0.00879 0.0132
3R:1183840-1183990:plus -40.8163 -2.2844 0.119266 0.917431 10.6531 -10.0204 -28.1193
0.916667 -10.2477 0.0436 0.0211 0.0111 0.021 0.0000102 0.00399 0.0699 0.0125 0.203
3R:11847820-11847970:minus -21.3265 3.68367 -2.49541 2.59633 -27.7041 -35.1122 -7.50459
-5.58333 2.46789 0.00193 0.00144 0.025 0.0109 0.038 0.0748 0.00189 0.0698 0.00805
3R:11847820-11847970:plus -32.3265 -2.3578 -0.743119 3.6422 -18.0816 -18.0102 -17.5413
-1.98611 0.238532 0.0187 0.0215 0.0147 0.00669 0.00759 0.00812 0.0139 0.0297 0.0173
3R:11850000-11850150:minus -22.398 -5.16514 3.45872 3.04587 -26.1939 -27.6327 -10.1009
1.125 3.24771 0.00344 0.0461 0.00332 0.00883 0.0175 0.043 0.00314 0.0117 0.00636
3R:11850000-11850150:plus -30.7755 -1.34862 -1.82569 2.05505 -18.449 -17.1224 -19.3028
3.79167 -1.47706 0.0102 0.016 0.0205 0.0139 0.0107 0.00499 0.0183 0.00437 0.0292
3R:11851620-11851770:minus -31.9592 2.24771 -2.65138 6.11009 -26.9694 -18.0102 -9.66055
3.52778 1.36697 0.0167 0.00458 0.0261 0.00233 0.023 0.00812 0.00289 0.00486 0.0124
3R:11851620-11851770:plus -14.6735 -0.623853 -0.394495 11.0183 -17.0408 -17.7857
2.04587 -0.638889 3.44954 0.00152 0.0127 0.0132 0.000052 0.00329 0.0068 0.000163 0.0203 0.00594
3R:11855920-11856070:minus -34.1837 0.201835 3.9633 6.6422 -19.2755 0.0204082 -18.0642
4.13889 1.48624 0.0314 0.00962 0.00272 0.00161 0.0164 0.000377 0.0151 0.0038 0.0118
3R:11855920-11856070:plus -23 6.98165 -5.44954 3.84404 -18.2653 -18.7857 -9.3945 2.05556
4.70642 0.00498 0.000656 0.0553 0.00624 0.00882 0.0146 0.00275 0.00845 0.00346
3R:11857080-11857230:minus -14.3367 5.25688 -0.174312 9.75229 -17.7143 -8.72449 -16.8991
5.26389 5.87156 0.00131 0.00146 0.0123 0.000226 0.00627 0.00169 0.0126 0.00233 0.00199
3R:11857080-11857230:plus -12.0408 -3.68807 3.58716 4.33945 -7.56122 -8.57143 -17.5229
1.06944 -3.9633 0.000311 0.0311 0.00316 0.00509 0.000419 0.00149 0.0139 0.0119 0.0551
3R:11863040-11863190:minus -32.9694 3.79817 5.34862 7.47706 -26.9694 -9.5 -27.4128 4.90278
-1.11927 0.0254 0.00259 0.00154 0.00085 0.023 0.00294 0.0637 0.00274 0.027
3R:11863040-11863190:plus -14.3469 8.24771 2.6789 3.88991 -27.1224 0.122449 -16.0826
3.95833 1.73394 0.00131 0.000333 0.00449 0.00608 0.0245 0.000312 0.011 0.00409 0.0106
3R:11874740-11874890:minus -30.4082 1.27523 1.76147 4.3211 -18.4184 -17.9796 -19.1651
0.347222 3.72477 0.00908 0.00655 0.00631 0.00514 0.0101 0.00764 0.0179 0.015 0.00497
3R:11874740-11874890:plus -24.2347 -0.458716 2.74312 -1.76147 -29.0918 -9.09184
-21.5321 3.16667 3.46789 0.00763 0.012 0.00438 0.0471 0.0653 0.00219 0.0271 0.0056 0.00582
3R:11875100-11875250:minus -32.0714 6.88073 0.0275229 5.43119 -8.86735 -9.42857 -16.789
4.25 0.724771 0.0176 0.000691 0.0115 0.00371 0.00173 0.0027 0.0124 0.00362 0.0153
3R:11875100-11875250:plus -40.4796 13.5306 -0.477064 6.27523 -7.60204 -9.53061 -14.211
-0.652778 -1.57798 0.0407 0.0000206 0.0135 0.00206 0.000466 0.0031 0.0076 0.0204 0.0299
3R:11877140-11877290:minus -13 6.9633 8.09174 5.74312 -18.4592 -17.7857 -12.4495 -1.34722
-0.669725 0.000563 0.000662 0.000416 0.00302 0.0109 0.0068 0.0051 0.0249 0.0236
3R:11877140-11877290:plus -22.6327 -1.88073 1.44954 11.2936 -26.1939 -18.2755 -20.8899
6.34722 0.385321 0.00373 0.0188 0.00705 0.0000139 0.0175 0.0112 0.0241 0.0014 0.0166
3R:1190880-1191030:minus -32.9286 1 5.62385 3.86239 -26.5306 -17.9388 -9.77982
-0.416667 1.74312 0.025 0.00724 0.00136 0.00622 0.0185 0.00738 0.00295 0.019 0.0105
3R:1190880-1191030:plus -31.8469 3.9633 -0.669725 -0.568807 -8.67347 -9.86735 -24.1927
1.29167 1.9633 0.0162 0.00244 0.0144 0.032 0.00154 0.00391 0.0422 0.011 0.00951
3R:11924480-11924630:minus -32.398 -0.348624 1.97248 5.33028 -7.60204 -18.0102 -14.8991
-0.138889 4.66055 0.019 0.0116 0.00584 0.00389 0.000466 0.00812 0.00879 0.0175 0.0035
3R:11924480-11924630:plus -41.7347 0.633028 2.23853 1.57798 1.61224 -18.0816 -23.2661 0.5
7.68807 0.0643 0.00826 0.00529 0.0171 0.0000631 0.00934 0.0366 0.0143 0.000841
3R:11924860-11925010:minus -31.4694 0.66055 4.77982 1.69725 -18.6429 -17.9388 -10.6697

4.79167 11.8532 0.0124 0.00818 0.00195 0.0163 0.0121 0.00738 0.0035 0.00288 0.0000428
3R:11924860-11925010:plus -33.1429 9.88991 5.41284 8.81651 -18.1939 -8.57143 0.706422
0.513889 1.12844 0.0272 0.000141 0.00149 0.000501 0.0086 0.00149 0.000238 0.0142 0.0137
3R:11925140-11925290:minus -32.7143 4.54128 0.880734 2.49541 -18.1122 -17.9796 -1.55046
0.166667 3.31193 0.0222 0.00195 0.00861 0.0114 0.00771 0.00764 0.000401 0.0159 0.00618
3R:11925140-11925290:plus -32.4388 -0.770642 1.3578 1.92661 -17.3776 -35.8163 -16.0459
3.25 -0.504587 0.0195 0.0133 0.00728 0.0148 0.00419 0.0815 0.0109 0.00542 0.0221
3R:11925680-11925830:minus -21.7041 -0.119266 0.266055 -1.56881 -27.2755 -25.5306
-19.633 -4.98611 3.17431 0.00231 0.0107 0.0106 0.0442 0.0302 0.0205 0.0193 0.0615 0.00653
3R:11925680-11925830:plus -22.3265 -3.00917 4.53211 7.47706 -18.6837 -18.2755 -18.7706
-0.625 2.11009 0.00337 0.0258 0.00216 0.00085 0.0126 0.0112 0.0168 0.0202 0.00898
3R:11926160-11926310:minus -23.6633 5.15596 11.3394 5.30275 -18.4184 -18.7857 -17.1927
-0.680556 7.25688 0.00665 0.00153 0.000065 0.00396 0.0101 0.0146 0.0132 0.0206 0.00115
3R:11926160-11926310:plus -31.551 3.43119 8.15596 8.81651 -17.7551 -9.86735 -11.7706 1.25
-3.61468 0.0134 0.00297 0.000403 0.000501 0.00658 0.00391 0.00439 0.0112 0.0509
3R:1193400-1193550:minus -31.4388 0.623853 0.275229 -0.422018 -8.33673 0.316327
1.23853 5.83333 3.66972 0.0122 0.00829 0.0106 0.0305 0.00101 0.000243 0.000207 0.00179 0.00514
3R:1193400-1193550:plus -30.5918 2.16514 -0.917431 4.33945 -27.1939 -17.8265 -19.4771
0.0277778 -1.81651 0.00963 0.00473 0.0156 0.00509 0.0267 0.00699 0.0189 0.0166 0.0317
3R:1193960-1194110:minus -14.5612 4.05505 5.51376 5.92661 20.4694 -9.5 -12.1101 3.59722 5.22018
0.00147 0.00235 0.00143 0.00266 1.65e-07 0.00294 0.00472 0.00473 0.00289
3R:1193960-1194110:plus -39.949 -1.77064 -2.73394 2.72477 -18.6735 -18.2755 -17.6239
2.41667 -5.45872 0.0345 0.0182 0.0268 0.0102 0.0123 0.0112 0.0141 0.00742 0.0799
3R:11972440-11972590:minus -13.1837 -3.66972 12.0459 11.2936 -18.1531 -26.898 -10.6514
-2.625 -4.04587 0.000705 0.031 0.0000414 0.0000139 0.00829 0.0302 0.00348 0.0351 0.0563
3R:11972440-11972590:plus -31.9286 0.614679 -1.6789 5.75229 -7.86735 -17.9796 -20.8532
-1.29167 -0.119266 0.0166 0.00832 0.0196 0.00296 0.000617 0.00764 0.0239 0.0245 0.0192
3R:1197380-1197530:minus -22.449 2.41284 1.90826 2.84404 -26.2653 9.30612 -4.15596 5.18056
-1.13761 0.0035 0.00431 0.00598 0.00961 0.018 0.0000398 0.00078 0.00242 0.0271
3R:1197380-1197530:plus -13.5816 -5.36697 3.87156 2.19266 -18.3776 -27.602 -14.6514 0.277778
3.66972 0.000984 0.0486 0.00282 0.0131 0.00926 0.0416 0.00835 0.0153 0.00514
3R:11983740-11983890:minus -21.6224 -0.366972 4.21101 3.2844 -18.1939 -9.42857 -12.6881
6.34722 3.6055 0.00213 0.0117 0.00246 0.00793 0.0086 0.0027 0.00538 0.0014 0.00532
3R:11983740-11983890:plus -20.9286 -0.577982 7.21101 11.0183 -17.4184 -9.23469 4.30275
6.59722 7.18349 0.00183 0.0125 0.000655 0.000052 0.00457 0.00238 0.0000739 0.00124 0.00121
3R:1200300-1200450:minus -24.2551 8.08257 -2.05505 7.44037 -26.2245 -9.53061 -7.91743
-0.930556 3.05505 0.00768 0.000365 0.022 0.000912 0.0175 0.0031 0.00207 0.0221 0.00692
3R:1200300-1200450:plus -23.3776 4.00917 -1.33028 0.201835 -17.7551 -9.65306 -14.8257
3.59722 0.963303 0.00619 0.0024 0.0177 0.0255 0.00658 0.0034 0.00866 0.00473 0.0145
3R:1200640-1200790:minus -32.3367 13.4592 0.119266 6.84404 -18.1939 -18.2755 -25.8073
3.18056 -0.963303 0.0187 0.000064 0.0111 0.0014 0.0086 0.0112 0.0522 0.00557 0.0258
3R:1200640-1200790:plus -24.1837 13.4592 6.82569 3.47706 1.61224 -17.7143 -22.8073 0.305556
-5.14679 0.00751 0.000064 0.000788 0.00724 0.0000631 0.00634 0.034 0.0152 0.0751
3R:12012660-12012810:minus -30.6327 9.88991 -0.990826 3.88991 11.6939 -18.3469 -15.1101
2.40278 7.68807 0.00968 0.000141 0.0159 0.00608 1.17e-06 0.0124 0.00917 0.00746 0.000841
3R:12012660-12012810:plus -2.15306 8.6422 0.926606 6.31193 -27.1939 -18.9388 -8.65138
-0.152778 1.79817 0.0000259 0.00027 0.00848 0.00199 0.0267 0.0154 0.00239 0.0175 0.0102
3R:12028700-12028850:minus -21.6939 0.0642202 6.11927 9.49541 -18.3061 -9.08163 -10.7706
4.26389 3.45872 0.00226 0.0101 0.00109 0.000308 0.00884 0.00216 0.00357 0.0036 0.00591
3R:12028700-12028850:plus -23.2245 3.88991 3.44037 -1.10092 -17.1531 -27.6735 -9.47706
0.666667 10.3486 0.00576 0.00251 0.00335 0.038 0.00364 0.0443 0.00279 0.0135 0.0000988
3R:12030620-12030770:minus -21.7041 -2.09174 12.3028 4.07339 -17.4184 -18.3469 -10.8716
6.70833 2.85321 0.00231 0.0199 0.0000345 0.00562 0.00457 0.0124 0.00364 0.00117 0.00733
3R:12030620-12030770:plus -32.2857 -2.10092 12.1927 6.00917 -27.4184 -8.5 -21.5872 0.25

2.85321 0.0185 0.02 0.0000376 0.00255 0.0323 0.00126 0.0274 0.0155 0.00733
3R:12031100-12031250:minus -29.551 11.1468 -4.3945 0.642202 -27.2959 -8.45918 -10.4587
4.16667 1.86239 0.00845 0.000075 0.0422 0.0227 0.0303 0.00111 0.00336 0.00375 0.00994
3R:12031100-12031250:plus -31.8469 1.15596 9.77982 2.62385 -9.23469 -17.3469 -9.38532
0.444444 -1.57798 0.0162 0.00684 0.000162 0.0108 0.00277 0.0058 0.00275 0.0145 0.0299
3R:12032220-12032370:minus -20.398 1.66972 2.77064 5.42202 -26.9694 -18.0816 -2.97248
-0.138889 5.86239 0.00166 0.00567 0.00433 0.00374 0.023 0.00934 0.000564 0.0175 0.00201
3R:12032220-12032370:plus -22.9184 0.93578 3.88991 3.00917 -17.449 -19.1224 -15.3394 4.5
-1.15596 0.00447 0.00741 0.0028 0.00894 0.00484 0.0175 0.0096 0.00326 0.0273
3R:12032840-12032990:minus -14.8265 6.57798 4.97248 6.3578 -18.4184 -17.7143 -0.12844 5.16667
2.78899 0.00159 0.000803 0.0018 0.00195 0.0101 0.00634 0.000292 0.00244 0.00745
3R:12032840-12032990:plus -32.0408 3.68367 -2.6055 0.798165 -16.1531 -8.42857 -19.4587 2.63889 4.21101
0.0174 0.00144 0.0258 0.0218 0.00297 0.00104 0.0188 0.00684 0.00396
3R:12033880-12034030:minus -13.1122 3.97959 2.27523 6.33028 -8.67347 -9.79592 -11.0183 3.05556
-1.97248 0.000632 0.000669 0.00522 0.00198 0.00154 0.00377 0.00375 0.00584 0.033
3R:12033880-12034030:plus -23.3673 1.77982 0.917431 1.95413 -17.7143 -8.79592 6.88073 1.18056 4.92661
0.00617 0.00544 0.00851 0.0147 0.00627 0.0019 0.0000321 0.0114 0.00318
3R:12035100-12035250:minus -30.4082 2.97248 -0.678899 3.6055 -18.3776 -16.8265 -21.8716 -3.16667
-6.76147 0.00908 0.00351 0.0144 0.00686 0.00926 0.0043 0.0288 0.0403 0.107
3R:12035100-12035250:plus -31.1735 4.77982 0.385321 -0.0917431 -17.1939 -27.2653 -14.3211 -2.13889
3.88073 0.0109 0.00178 0.0102 0.0276 0.00377 0.0333 0.00778 0.0309 0.00475
3R:12035980-12036130:minus -30.3265 5.26606 5.84404 6.02752 -17.6735 -18.0816 -10.4312 -5.27778
-4.11927 0.0089 0.00146 0.00124 0.00246 0.00572 0.00934 0.00334 0.0655 0.0572
3R:12035980-12036130:plus -31.1327 -6.40367 1.33945 2.08257 -18.7857 -19.1531 -5.66972 3.48611 0.12844
0.0107 0.0632 0.00733 0.0137 0.0148 0.0177 0.0012 0.00494 0.0179
3R:12037080-12037230:minus -13.1122 -1.08257 1.49541 6.11009 -18.1531 -26.602 -17.844 -4.90278
0.440367 0.000632 0.0147 0.00694 0.00233 0.00829 0.0248 0.0146 0.0604 0.0164
3R:12037080-12037230:plus -14.0306 5.24771 5.87156 3.18349 -8.70408 -28.4082 -4.88991 1.51389 4.25688
0.00115 0.00147 0.00122 0.00837 0.00155 0.0615 0.000962 0.0102 0.00391
3R:12037980-12038130:minus -31.9694 -0.440367 6.53211 7.37615 -18.7551 -18.051 -5.95413 0.277778
-1.52294 0.0168 0.012 0.000904 0.000951 0.0144 0.00886 0.00129 0.0153 0.0295
3R:12037980-12038130:plus -13.2245 -1.30275 0.12844 5.82569 -18.4184 -27.5306 -1.68807 -3.44444 -2.15596
0.000738 0.0158 0.0111 0.00291 0.0101 0.0374 0.000414 0.0431 0.0348
3R:12049080-12049230:minus -3.07143 -0.155963 4.3578 6.72477 -26.1939 -27.0408 -12.4587 0.486111
3.20183 0.0000441 0.0109 0.00232 0.00154 0.0175 0.0307 0.00511 0.0144 0.0065
3R:12049080-12049230:plus 4.58163 1.11009 4.87156 6.72477 -18.4184 -9.42857 -15.6606 -9.11111 -5.99083
0.0000222 0.00695 0.00188 0.00154 0.0101 0.0027 0.0102 0.134 0.0886
3R:12050440-12050590:minus -33.4082 3.57143 1.62385 0.972477 -25.9286 -18.1224 -23.3394 2.72222
0.357798 0.0283 0.00175 0.00663 0.0207 0.0168 0.00988 0.0371 0.00663 0.0166
3R:12050440-12050590:plus -20.8878 -3.51376 -0.385321 9.24771 -18.1224 -18.7857 -14.6514 -1.40278
9.92661 0.0018 0.0297 0.0131 0.000409 0.00782 0.0146 0.00835 0.0253 0.000193
3R:12058620-12058770:minus -2.29592 3.97959 5.23853 6.21101 -27.1531 -8.79592 -8.81651 2.45833
-3.80734 0.000028 0.000669 0.00161 0.00213 0.0249 0.0019 0.00247 0.00731 0.0528
3R:12058620-12058770:plus -21.4796 4.43119 -3.17431 4.56881 -27.1531 -26.8265 8.88991 2.25 -0.798165
0.00202 0.00204 0.0303 0.0048 0.0249 0.0285 0.0000185 0.00788 0.0246
3R:12060180-12060330:minus -4.55102 2.0367 0.633028 2.59633 -8.37755 -27.898 -12.633 -6.94444
3.10092 0.000159 0.00495 0.00938 0.0109 0.00106 0.0537 0.00532 0.0916 0.00674
3R:12060180-12060330:plus -22.0306 -3.93578 14.5138 -0.229358 -17.0816 -18.7143 -7.20183 -0.152778
7.42202 0.00293 0.0333 3.77e-06 0.0287 0.00334 0.014 0.00177 0.0175 0.00106
3R:12061040-12061190:minus -14.0816 -2.11927 2.21101 5.48624 -27.0102 -18.3469 -20.0826 -1.27778
8.95413 0.00117 0.0201 0.00534 0.00357 0.0237 0.0124 0.0209 0.0244 0.000458
3R:12061040-12061190:plus -21.9898 -2.07339 3.63303 5.69725 -27 -18.3061 -3.00917 -2.30556 2.42202
0.00282 0.0198 0.00311 0.00314 0.0233 0.0114 0.000569 0.0323 0.00816
3R:12062260-12062410:minus -23.2653 0.651376 -1.62385 -2.87156 -18.5204 -9.23469 -24.2844 6.88889

-1.68807 0.00593 0.00821 0.0193 0.0668 0.0117 0.00238 0.0428 0.00107 0.0308
3R:12062260-12062410:plus -23 2.89908 1.76147 0.651376 -18.4184 -27.8265 -9.43119 -4.83333 10.0367
0.00498 0.00361 0.00631 0.0226 0.0101 0.0498 0.00277 0.0595 0.000146
3R:12062880-12063030:minus -32.8061 0.944954 4.9633 1.36697 -17.9796 -10.0204 -16.7156 -0.291667
-1.48624 0.0237 0.00739 0.00181 0.0186 0.00726 0.00399 0.0122 0.0183 0.0293
3R:12062880-12063030:plus -23.1531 -1.22018 3.77064 6.84404 -17.6837 -18.0102 -9.73394 -3.95833 1.38532
0.00542 0.0154 0.00294 0.0014 0.0058 0.00812 0.00293 0.0488 0.0123
3R:12066340-12066490:minus -14.1837 7.6422 9.14679 3.69725 -18.2245 -8.5 -11.3945 3.45833 4.18349
0.00124 0.000462 0.000233 0.00665 0.00877 0.00126 0.00405 0.005 0.00404
3R:12066340-12066490:plus -22.4388 0.440367 5.07339 9.24771 -18.4898 -18.7857 -10.5413 -4.48611 -2.25688
0.00348 0.00885 0.00172 0.000409 0.0116 0.0146 0.00341 0.055 0.0357
3R:12067040-12067190:minus -11.5102 -1.6789 9.80734 7.63303 -8.89796 0.826531 -0.688073 1.80556
0.550459 0.000253 0.0177 0.000159 0.000745 0.00189 0.000153 0.000331 0.00923 0.0161
3R:12067040-12067190:plus -2.36735 -0.642202 -0.0366972 11.2936 -18.0816 -8.45918 -4.90826 -8.68056
3.6055 0.0000302 0.0128 0.0117 0.0000139 0.00759 0.00111 0.000967 0.125 0.00532
3R:12068220-12068370:minus -22.6735 -0.752294 8.14679 6.72477 -8.70408 -19.0816 -15.7982 0.0972222
9.87156 0.00385 0.0132 0.000404 0.00154 0.00155 0.017 0.0105 0.0162 0.000217
3R:12068220-12068370:plus -21.8571 -3.34862 5.42202 -0.0733945 -27.4082 -9.72449 -11.3761 -1.72222
2.99083 0.00249 0.0284 0.00149 0.0274 0.0321 0.00352 0.00403 0.0276 0.00706
3R:12070480-12070630:minus -14.8571 -2.17431 3.55046 7.2844 -26.6735 -26.602 -12.156 5.52778
11.3853 0.0016 0.0204 0.00321 0.00105 0.0191 0.0248 0.00477 0.00207 0.0000815
3R:12070480-12070630:plus -12.3367 5.63303 2.55963 2.76147 -17.7143 -9.79592 -12.8073 1.06944 1.25688
0.000393 0.00125 0.0047 0.01 0.00627 0.00377 0.00553 0.0119 0.0131
3R:12071260-12071410:minus -31.5816 -3.31193 -2.51376 5.3945 -27.3367 -18.3469 -0.256881 0.263889
3.31193 0.0135 0.0281 0.0251 0.00379 0.0317 0.0124 0.000301 0.0154 0.00618
3R:12071260-12071410:plus -24.5306 -0.568807 6.57798 3.90826 -28.3061 -27.602 -12.9174 -2.55556
3.22018 0.00814 0.0125 0.000884 0.00603 0.0553 0.0416 0.00567 0.0345 0.0064
3R:12076060-12076210:minus -31.2857 -1.33945 -1.2844 3.36697 -8.33673 -27.1224 3.07339 7.02778
3.66972 0.0113 0.0159 0.0174 0.00764 0.00101 0.0325 0.000115 0.000991 0.00514
3R:12076060-12076210:plus -21.9286 -0.678899 5.6789 2.62385 -18.449 -18.051 -9.80734 -5.29167
1.36697 0.0027 0.0129 0.00133 0.0108 0.0107 0.00886 0.00297 0.0657 0.0124
3R:12077680-12077830:minus -22.8061 -0.853211 13.6422 -0.0733945 -17.602 -8.42857 -12.2844
-5.52778 1.6422 0.00414 0.0137 0.0000116 0.0274 0.00547 0.00104 0.00491 0.069 0.0111
3R:12077680-12077830:plus -24 1.55963 1.04587 11.0183 -18.5612 -17.2041 -18.0367 2.43056 1.06422
0.00719 0.0059 0.00813 0.000052 0.0119 0.00507 0.015 0.00738 0.014
3R:12080840-12080990:minus -22.7755 0.0642202 1.00917 4.17431 -18.3776 -26.3367 -18.1009 -1.45833
-6.89908 0.00412 0.0101 0.00824 0.00537 0.00926 0.0221 0.0152 0.0257 0.111
3R:12080840-12080990:plus -31.6633 -3.01835 2.84404 3.81651 -8.67347 -18.2755 -12.2202 1.84722 4.40367
0.0141 0.0259 0.00421 0.00626 0.00154 0.0112 0.00484 0.0091 0.00373
3R:12081840-12081990:minus -22.8163 2.38532 4.09174 5.65138 -19.2755 0.0204082 -17.789 -0.652778
-0.651376 0.00418 0.00436 0.00258 0.00322 0.0164 0.000377 0.0145 0.0204 0.0235
3R:12081840-12081990:plus -13.4796 2.62385 0.715596 11.0275 -17.4592 -8.86735 -9.48624 -0.180556
5.38532 0.000935 0.00399 0.00912 0.0000401 0.00491 0.00198 0.0028 0.0177 0.00267
3R:12089840-12089990:minus -31.7347 1.25688 7.77064 8.77982 -18.1224 -18.5 -24.1101 -2.31944
3.45872 0.0149 0.00659 0.000494 0.000533 0.00782 0.0131 0.0417 0.0325 0.00591
3R:12089840-12089990:plus -4.63265 2.24771 16.2202 2.34862 -18.7245 -9.72449 -3.22018 -2.16667 -2.10092
0.000175 0.00458 1.24e-07 0.0121 0.0136 0.00352 0.000602 0.0312 0.0342
3R:12098060-12098210:minus -13.1429 1.75229 -0.119266 0.0825688 -17.7551 -26.7551 -18.0183
5.40278 -0.0275229 0.00065 0.0055 0.0121 0.0262 0.00658 0.0263 0.015 0.00219 0.0186
3R:12098060-12098210:plus -41.2959 -1.68807 -0.201835 7.72477 10.4286 -8.86735 -11.3303 -0.694444
-2.52294 0.0529 0.0177 0.0124 0.000681 0.0000139 0.00198 0.00399 0.0206 0.0391
3R:12098680-12098830:minus -41.6327 -2.69725 0.93578 -2.08257 -28.5306 -8.45918 -22.2202 1.51389
3.83486 0.0631 0.0237 0.00845 0.0526 0.061 0.00111 0.0307 0.0102 0.00481
3R:12098680-12098830:plus -23.0408 -1.06422 -6.22936 2.23853 -7.60204 -15.9796 -15.4128 3.48611 2.84404

0.00512 0.0146 0.0669 0.0128 0.000466 0.00405 0.00973 0.00494 0.00736
3R:12099520-12099670:minus -13.449 1.14679 3.11009 2.07339 19.9082 1.05102 -15.8073 2.22222
9.66055 0.000914 0.00686 0.00381 0.0138 8.13e-07 0.000107 0.0105 0.00796 0.000268
3R:12099520-12099670:plus -41.8878 5.43119 1.34862 6.11009 -18.8265 9.08163 -18.4587 6.77778 -2.36697
0.0672 0.00136 0.00731 0.00233 0.0149 0.0000431 0.016 0.00113 0.0371
3R:12106060-12106210:minus -23.1531 4.72477 4.24771 5.48624 -9.23469 -28.3776 6.80734 1.01389
3.47706 0.00542 0.00182 0.00243 0.00357 0.00277 0.0609 0.0000327 0.0121 0.00575
3R:12106060-12106210:plus -23.5204 -3.66055 4.01835 7.17431 -17.449 -18.0102 -13.7431 1.75 -0.669725
0.00645 0.0309 0.00266 0.00115 0.00484 0.00812 0.00685 0.00941 0.0236
3R:12106700-12106850:minus -30.4796 0.0366972 2.77982 1.94495 -17.449 -17.4184 -7.79817 4.79167
10.3394 0.00941 0.0102 0.00432 0.0147 0.00484 0.00585 0.00202 0.00288 0.000112
3R:12106700-12106850:plus -32.9694 5.02752 0.0642202 5.73394 -18.4184 -9.60204 -17.8991 3.90278
0.0275229 0.0254 0.00161 0.0113 0.00307 0.0101 0.00334 0.0147 0.00418 0.0183
3R:12107980-12108130:minus -31.9286 2.6055 3.01835 2.63303 -27.7959 0.0204082 -30.6881 0.805556
1.33028 0.0166 0.00402 0.00394 0.0107 0.0386 0.000377 0.1 0.013 0.0127
3R:12107980-12108130:plus -32.8061 -1.78899 13.1835 1.66972 -17.9286 -26.7143 -19.6697 -2.80556 -0.284404
0.0237 0.0183 0.0000171 0.0165 0.0071 0.0257 0.0195 0.0368 0.0207
3R:12113600-12113750:minus -31.3571 2.15596 4.56881 2.55963 -18.449 -27.3367 -19.0734 6.04167
0.183486 0.0114 0.00474 0.00213 0.0111 0.0107 0.0347 0.0177 0.00162 0.0176
3R:12113600-12113750:plus -32.6735 2.74312 5.02752 6.57798 -8.93878 -0.173469 -18.9817 -0.847222
-4.44954 0.0216 0.00382 0.00176 0.00167 0.00216 0.000456 0.0174 0.0216 0.0632
3R:12117700-12117850:minus -22.898 -1.76147 2.16514 3.95413 -18.5306 -9.42857 -18.578 -0.652778
10.1284 0.00442 0.0181 0.00544 0.00593 0.0118 0.0027 0.0163 0.0204 0.000129
3R:12117700-12117850:plus -29.4388 -0.330275 4.62385 6.19266 -8.60204 -9.5 -11.6972 0.361111 4.91743
0.00842 0.0115 0.00208 0.00216 0.00125 0.00294 0.00432 0.0149 0.00321
3R:12146060-12146210:minus -22.2959 0 2.91743 2.98165 -27.1939 -7.65306 -20.5505 -0.652778
-6.22018 0.00335 0.0103 0.0041 0.00905 0.0267 0.000647 0.0227 0.0204 0.0938
3R:12146060-12146210:plus -31.398 -3.95413 6.50459 6.19266 -17.4184 -28.3367 -5.6422 -0.569444
-0.926606 0.0117 0.0334 0.000917 0.00216 0.00457 0.0606 0.00119 0.0199 0.0256
3R:12164240-12164390:minus -21.9184 -2.13761 14.5138 3.88991 -18.5204 -9.20408 -2.91743 11.1389
0.293578 0.00261 0.0202 3.77e-06 0.00608 0.0117 0.00225 0.000556 0.0000678 0.017
3R:12164240-12164390:plus -30.8163 5.25688 -1.58716 1.95413 -17.4184 0.0510204 -5.81651 4.70833
5.3211 0.0103 0.00146 0.0191 0.0147 0.00457 0.000349 0.00125 0.00298 0.00275
3R:12167660-12167810:minus -22.7347 -1.99083 5.41284 2.70642 10.6531 -18.4898 -12.2569 -1.66667
1.79817 0.00403 0.0194 0.00149 0.0103 0.0000102 0.0129 0.00488 0.0272 0.0102
3R:12167660-12167810:plus -31.4082 8.45872 -1.44037 11.2936 -18.1122 -10.0918 -8.59633 0.458333 1.9633
0.0119 0.000297 0.0183 0.0000139 0.00771 0.00404 0.00237 0.0145 0.00951
3R:12173500-12173650:minus -41.4796 -0.348624 0.963303 5.49541 -7.85714 -9.72449 -19.2294 5.34722
-6.04587 0.0577 0.0116 0.00837 0.00355 0.000594 0.00352 0.0181 0.00225 0.0898
3R:12173500-12173650:plus -24.0306 2.24771 -2.3945 3.94495 -27.2347 1.05102 -17.3486 2.94444 2.93578
0.00723 0.00458 0.0242 0.00595 0.0283 0.000107 0.0135 0.00609 0.00717
3R:12174100-12174250:minus -31.5204 -3.86239 4.29358 6.72477 -26.6633 -16.2755 -3.13761 1.66667
-0.0825688 0.0131 0.0326 0.00238 0.00154 0.0189 0.00413 0.000589 0.00969 0.0189
3R:12174100-12174250:plus -34.1122 -0.963303 2.46789 2.88073 -27.1939 -8.57143 -22.0459 5.98611
-1.08257 0.0312 0.0142 0.00486 0.00945 0.0267 0.00149 0.0297 0.00167 0.0267
3R:12175000-12175150:minus -21.8061 -0.541284 11.0092 9.49541 -26.6327 -18.051 -3.15596 -4.05556
1.83486 0.00241 0.0124 0.0000797 0.000308 0.0188 0.00886 0.000592 0.0499 0.0101
3R:12175000-12175150:plus -14.7041 7.48624 -3.33945 7.73394 -17.7143 -36.8878 -10.633 3.13889
-0.0825688 0.00154 0.000502 0.0317 0.000669 0.00627 0.123 0.00347 0.00566 0.0189
3R:12176920-12177070:minus -13 3.77064 0.238532 4.56881 -19.051 -19.0102 -7.49541 -3.86111 7.36697
0.000563 0.00262 0.0107 0.0048 0.0162 0.0159 0.00189 0.0477 0.00108
3R:12176920-12177070:plus -23.3367 3.80734 1.2844 5.30275 -16.7551 -9.53061 -19.4037 -2.09722 4.88073
0.00614 0.00259 0.00748 0.00396 0.00322 0.0031 0.0186 0.0306 0.00324
3R:12184740-12184890:minus -42.0306 -0.0917431 -0.825688 1.44037 -9.20408 -16.3469 -21.5229

5.20833 1.78899 0.0689 0.0106 0.0151 0.018 0.00262 0.00416 0.027 0.00239 0.0103
3R:12184740-12184890:plus -34.2959 3.0367 -2.27523 5.30275 -27.1939 -18.2755 -27.633 3.72222 -2.18349
0.0316 0.00343 0.0234 0.00396 0.0267 0.0112 0.0656 0.0045 0.0351
3R:1219520-1219670:minus -34.3367 8.21101 6.58716 2.84404 -28.4184 -0.244898 -27.3394 4.27778
-4.84404 0.0318 0.00034 0.00088 0.00961 0.056 0.000496 0.0631 0.00358 0.0701
3R:1219520-1219670:plus -23.1939 0.825688 -1.22936 2.88991 20.4694 -9.45918 -16.5596 7.5 11.8991
0.00558 0.00771 0.0171 0.00942 1.65e-07 0.00279 0.0119 0.000771 0.0000255
3R:12257500-12257650:minus -33.449 6.43119 0.00917431 2.55046 -19.2755 -9.09184 -13.0092 2.27778
-4.07339 0.0284 0.000862 0.0116 0.0112 0.0164 0.00219 0.00579 0.0078 0.0566
3R:12257500-12257650:plus -30.1429 -2.33028 3.63303 4.06422 -18.7857 -26.602 -15.3119 2.88889 3.49541
0.00866 0.0214 0.00311 0.00564 0.0148 0.0248 0.00954 0.00622 0.0057
3R:12258680-12258830:minus -33.2959 4.88991 3.81651 2.70642 -8.93878 -8.45918 -22.1468 4.26389
-4.29358 0.0279 0.0017 0.00289 0.0103 0.00216 0.00111 0.0303 0.0036 0.06
3R:12258680-12258830:plus -23.7449 1.84404 2.86239 3.14679 -17.7449 -17.8571 -8.3945 7.95833 3
0.00674 0.00532 0.00418 0.00847 0.0063 0.00719 0.00228 0.000597 0.00702
3R:12260560-12260710:minus -13.6735 -0.0825688 0.40367 2.19266 -17.8265 -19.6429 -21.0642 7.40278
0.862385 0.00103 0.0106 0.0101 0.0131 0.00691 0.0204 0.0249 0.000813 0.0148
3R:12260560-12260710:plus -21.7755 -1.88073 4.43119 2.80734 -17.7551 -18.051 -18.1468 1.27778 -1.68807
0.00241 0.0188 0.00225 0.00985 0.00658 0.00886 0.0153 0.0111 0.0308
3R:12262220-12262370:minus -23.8571 4.17431 13.2385 7.29358 -19.051 -9.16327 -8.56881 -1.25
-2.78899 0.00687 0.00225 0.000016 0.00104 0.0162 0.00223 0.00236 0.0242 0.0422
3R:12262220-12262370:plus -23.1939 4.37615 13.844 7.47706 -8.92857 -0.316327 -7.88073 2.5 1.74312
0.00558 0.00208 8.84e-06 0.00085 0.00194 0.000524 0.00205 0.0072 0.0105
3R:12267400-12267550:minus -23.5612 2.49541 14.4404 3.13761 -7.89796 -18.898 -20.5596 10.2639
7.6422 0.00653 0.00418 4.12e-06 0.00854 0.000678 0.0152 0.0227 0.000135 0.000893
3R:12267400-12267550:plus -21 8.29358 2.86239 4.11009 -27.2653 -8.5 -22.844 2.80556 2.42202 0.00186
0.000326 0.00418 0.00553 0.0302 0.00126 0.0342 0.00642 0.00816
3R:12268680-12268830:minus -23.2959 14.0275 0.412844 2.66972 -8.93878 -17.7143 -20.4862 -0.222222
-0.899083 0.00605 8.23e-06 0.0101 0.0106 0.00216 0.00634 0.0224 0.0179 0.0254
3R:12268680-12268830:plus -13.4388 5.15596 6.22018 -1.63303 -18.4184 -27.9694 -3.80734 2.33333 2.25688
0.000879 0.00153 0.00105 0.0452 0.0101 0.0547 0.000707 0.00765 0.00853
3R:12268840-12268990:minus -23.2959 14.0275 0.412844 2.66972 -8.93878 -17.7143 -20.4862 -0.222222
-0.899083 0.00605 8.23e-06 0.0101 0.0106 0.00216 0.00634 0.0224 0.0179 0.0254
3R:12268840-12268990:plus -13.4388 5.15596 6.22018 -1.63303 -18.4184 -27.9694 -3.80734 2.33333 2.25688
0.000879 0.00153 0.00105 0.0452 0.0101 0.0547 0.000707 0.00765 0.00853
3R:12272740-12272890:minus -21.6224 9.3945 1.47706 6.06422 -19.051 -18.3469 -5.56881 -3.38889
6.54128 0.00213 0.000182 0.00698 0.00239 0.0162 0.0124 0.00117 0.0426 0.00151
3R:12272740-12272890:plus -30.9694 2.06422 0.293578 6.41284 -17.2653 -17.5 -18.0734 -3.125 7.6422
0.0104 0.0049 0.0105 0.00189 0.00387 0.00599 0.0151 0.0399 0.000893
3R:12273580-12273730:minus -13.2551 0.486239 2.79817 4.3945 -8.67347 -18.2755 -0.889908 3.72222
4.3945 0.000751 0.00871 0.00429 0.00499 0.00154 0.0112 0.000346 0.0045 0.00375
3R:12273580-12273730:plus -14.3367 -1.04587 9.04587 10.5872 -8.93878 -9.23469 -18.4404 0.916667 9.55046
0.00131 0.0145 0.000248 0.000115 0.00216 0.00238 0.016 0.0125 0.000321
3R:12274040-12274190:minus -31.5102 -0.651376 1.69725 3.19266 -17.4082 -8.79592 -14.0642 -3.43056
-3.51376 0.013 0.0128 0.00646 0.00829 0.00421 0.0019 0.00736 0.043 0.0498
[truncated: 1,233,964 more chars]
